# Molecular Analysis of SARS-CoV-2 Circulating in Bangladesh during 2020 Revealed Lineage Diversity and Potential Mutations

**DOI:** 10.3390/microorganisms9051035

**Published:** 2021-05-12

**Authors:** Rokshana Parvin, Sultana Zahura Afrin, Jahan Ara Begum, Salma Ahmed, Mohammed Nooruzzaman, Emdadul Haque Chowdhury, Anne Pohlmann, Shyamal Kumar Paul

**Affiliations:** 1 Department of Pathology, Faculty of Veterinary Science, Bangladesh Agricultural University, Mymensingh 2202, Bangladesh; jahan.begum@bau.edu.bd (J.A.B.); mohammed.nooruzzaman@bau.edu.bd (M.N.); emdad@bau.edu.bd (E.H.C.); 2 Department of Microbiology, Mymensingh Medical College, Mymensingh 2200, Bangladesh; szafrin2@gmail.com (S.Z.A.); ahmed.salma51@yahoo.com (S.A.); 3 Institute of Diagnostic Virology, Friedrich-Loeffler-Institut, 17493 Greifswald-Insel Riems, Germany; anne.pohlmann@fli.de

**Keywords:** SARS-CoV-2, Bangladesh, clade, lineage B.1.1.7, evolution, mutation

## Abstract

Virus evolution and mutation analyses are crucial for tracing virus transmission, the potential variants, and other pathogenic determinants. Despite continuing circulation of the SARS-CoV-2, very limited studies have been conducted on genetic evolutionary analysis of the virus in Bangladesh. In this study, a total of 791 complete genome sequences of SARS-CoV-2 from Bangladesh deposited in the GISAID database during March 2020 to January 2021 were analyzed. Phylogenetic analysis revealed circulation of seven GISAID clades G, GH, GR, GRY, L, O, and S or five Nextstrain clades 20A, 20B, 20C, 19A, and 19B in the country during the study period. The GISAID clade GR or the Nextstrain clade 20B or lineage B.1.1.25 is predominant in Bangladesh and closely related to the sequences from India, USA, Canada, UK, and Italy. The GR clade or B.1.1.25 lineage is likely to be responsible for the widespread community transmission of SARS-CoV-2 in the country during the first wave of infection. Significant amino acid diversity was observed among Bangladeshi SARS-CoV-2 isolates, where a total of 1023 mutations were detected. In particular, the D614G mutation in the spike protein (S_D614G) was found in 97% of the sequences. However, the introduction of lineage B.1.1.7 (UK variant/S_N501Y) and S_E484K mutation in lineage B.1.1.25 in a few sequences reported in late December 2020 is of particular concern. The wide genomic diversity indicated multiple introductions of SARS-CoV-2 into Bangladesh through various routes. Therefore, a continuous and extensive genome sequence analysis would be necessary to understand the genomic epidemiology of SARS-CoV-2 in Bangladesh.

## 1. Introduction

During the year 2020, the corona virus disease-19 (COVID-19) pandemic spread quickly across the world, and it is still circulating in waves. The disease is caused by the newly discovered coronavirus-2, which causes severe acute respiratory syndrome (SARS-CoV-2) [1,2]. Despite the fact that many countermeasures against SARS-CoV-2 transmission have been implemented around the world, there are no indications that the pandemic will be over anytime soon.

SARS-CoV-2 is an enveloped, positive-sense, single-stranded RNA virus that belongs to the family Coronaviridae. There are four genera of coronaviruses (CoVs), namely, Alphacoronavirus (αCoV), Betacoronavirus (βCoV), Deltacoronavirus (δCoV), and Gammacoronavirus (γCoV) [3]. CoVs have repeatedly crossed species barriers, and a few have emerged as important human pathogens such as severe acute respiratory syndrome virus (SARS-CoV), Middle East respiratory syndrome coronavirus (MERS-CoV), and the recent pandemic SARS-CoV-2 [1,4,5]. The SARS-CoV-2 viral genome is approximately 27–30 kb nucleotides in length. Excluding the 5’-cap structure and 3’-poly-A tail, the genome of SARS-CoV-2 consists of 10 open reading frames (ORFs), of which about two-third encompass the viral ORF1ab gene and encode polyproteins (PP) 1a and PP1b [6]. The two polyproteins are further cleaved into 16 non-structural proteins (nsp1 to nsp16). The remaining ORFs encode for four structural proteins, namely spike glycoprotein (S), matrix protein (M), envelope protein (E), and nucleocapsid protein (N), and at least five accessory proteins, ORF 3a/NS3, ORF 6/NS6, ORF 7a/NS7a, ORF 7b/NS7b, and ORF 8/NS8 [6,7]. The four main structural proteins of the virus are encoded by ORFs near the 3’ terminus of the genome, among which the spike (S) protein induces the neutralizing antibody in the host and the receptor-binding domain (RBD) determines the pathogenic potential of the virus [6,7,8].

SARS-CoV-2 undergoes rapid mutation and recombination like other RNA viruses, leading to the formation of diverse evolutionary groups. Consequently, within a year of emergence, the SARS-CoV-2 has evolved into nine clades, G, GH, GR, GRY, GV, L, O, S, and V (GISAID nomenclature; https://www.epicov.org/epi3/frontend#lightbox-2062061000 (accessed on 16 February 2021); [9]), and two lineages (A and B lineages) with many sub-lineages [10]. Another nomenclature system has been used by Nextstrain [11] to designate clades of SARS-CoV-2, and it has also been followed among researchers. In addition, within these different clades and lineages, extremely infectious new variants have been reported from different countries [12,13]. SARS-CoV-2 has been the subject of many molecular epidemiological studies, the majority of which focus on mutations in the viral spike protein, which is responsible for binding to the host angiotensin-converting enzyme 2 (ACE2) and initiating the viral entry process [14,15,16]. Current studies have mainly been focused on three viral lineages, B.1.1.7, B.1.351, and P.1, because of their significant impact on viral transmissibility and pathogenicity.

In Bangladesh, the first case of COVID-19 was identified on 8 March 2020 by the Institute of Epidemiology, Disease Control and Research (IEDCR). Following this, the infection rate skyrocketed, hitting 25% in August 2020, before steadily decreasing with a small rise in December 2020 (15%). Bangladesh had the lowest number of cases in January–February 2021, but it unfortunately started to grow again in March (IEDCR; Coronavirus COVID-19 dashboard, 2020; http://103.247.238.92/webportal/pages/covid19.php (accessed on 17 February 2021)). However, the second wave started in March 2021 and the number of new cases increased manyfold. Until March 2021, nearly 574,000 confirmed cases were reported, with a total of 8720 fatalities in Bangladesh (WHO; https://experience.arcgis.com/experience/56d2642cb379485ebf78371e744b8c6a (accessed on 17 February 2021)). Bangladesh started the vaccination program on 7 February 2021, and by 20 March, a total of 4.76 million doses of vaccine were administered, putting the country ahead of many global powerhouses (https://github.com/owid/covid-19-data/tree/master/public/data/vaccinations (accessed on 17 February 2021).

Whole-genome sequencing and phylogenetic analyses of the viral genomes are crucial to understand the virus evolution and outbreak dynamics. The availability of genomic data in a public repository such as GISAID [9] allows researchers from all over the world to access the resources and address relevant hypotheses. Similarly, this provides us with a unique opportunity to learn about the origin, evolution, and spread of the SARS-CoV-2 in Bangladesh and to compare it with many global SARS-CoV-2 isolates. There is very little genetic evidence about the SARS-CoV-2 outbreak in Bangladesh. As a result, the current research will provide some basic information about the virus genotypes and phenotypes that are currently circulating in the country.

In this paper, we analyzed the 791 complete genome sequences of SARS-CoV-2 strains available in the GISAID database predominantly sampled from different administrative divisions of Bangladesh during March 2020 to January 2021. Furthermore, we systematically analyzed the phylogenetic clusters of genomes and assessed the mutation dynamics that have a potential role in viral pathogenesis.

## 2. Materials and Methods

### 2.1. Genome Sequence Metadata Retrieval and Data Curation

The sequence of Bangladeshi SARS-CoV-2 genomes deposited in the GISAID database (https://www.gisaid.org/ (accessed on 20 February 2021) during the period 1 March 2020–31 January 2021 were selected and their corresponding metadata were retrieved for the analysis. A total of 856 sequences were available during the selected time period from which 791 complete genome sequences were considered for the analysis in this study. The sequences were obtained mainly from nasopharyngeal swabs of covid patients of both sexes aged between 8 days and 85 years (Supplementary Figure S1). We considered the full-genome sequences for nucleotide substitution rates, phylogenetic clustering, and amino acid substitution analysis. The remining 65 sequences were excluded from the analysis due to their partial length and sequence ambiguities as the accuracy of sequences would be case sensitive. Reference strain (Wuhan-Hu-1, NC_045512) was downloaded from the NCBI database to compare the phylogenetic origin and substitution analysis. Furthermore, high-quality genome sequences from each of the nine clades and selected closest sequences of non-Bangladesh origin were retrieved from GISAID and used in the molecular analysis.

### 2.2. Phylogenetic Analysis

The selected genomic sequences of SARS-CoV-2 were initially analyzed through “One Click Workflows” (https://ngphylogeny.fr/workflows/oneclick/ (accessed on 22 February 2021), which is a web service dedicated to phylogenetic analysis [17]. The program provides a complete set of phylogenetic tools and workflows adapted to various contexts and various levels of user expertise. It is built around the main steps of most phylogenetic analyses where input data were in FASTA format, multiple alignment was carried out in multiple alignment with fast Fourier transformation (MAFFT) [18], alignment curation was performed in Block Mapping and Gathering with Entropy (BMGE) [19], Fast Distance-Based Phylogeny Inference Program (FastME) [20] tree inference was shown, bootstrapping for big data was incorporated within the package [21] and finally tree rendering was shown in Newick display [22] and visualized in iTOL [23].

The collected genomic sequences of Bangladeshi SARS-CoV-2 along with reference Wuhan strain (NC_045512) were aligned independently using multiple alignment with fast Fourier transformation (MAFFT v7, © 2013 Kazutaka Katoh) online platform (https://mafft.cbrc.jp/alignment/software/ (accessed on 23 February 2021) [24]. Nextclade beta (https://clades.nextstrain.org/results (accessed on 23 February 2021) was used to check the sequence quality and to generate the clade designated tree. A Randomized Axelerated Maximum Likelihood (RAxML) tree was built based on the general time-reversible (GTR) model using RAxML v1.0.0 [25] with 1000 bootstrap replicates. The dataset generated in “One Click Workflows” was used to analyze the independent RAxML tree. Time-scaled trees of the alignments were calculated with BEAST (1.10.4) software package [26] using strict clock and coalescent constant population tree models. Summary maximum clade credibility trees (MCC) for the post-burn-in posterior time-scaled trees were created using TreeAnnotator. The tree was furnished using FigTree v1.4.2 (http://tree.bio.ed.ac.uk/software/figtree (accessed on 23 February 2021) and iTOL [23].

### 2.3. Evaluation of Deduced Amino Acid Substitutions

Following the GISAID EpiCoV database workflow (https://www.epicov.org/epi3/frontend#31c5b2 (accessed on 24 February 2021), a multi-FASTA file of SARS-CoV-2 viral amino acid (AA) of 791 Bangladeshi strains was aligned and compared with the reference strain (hCoV-19/Wuhan/WIV04/2019) using BioEdit software. The CoVsurver algorithm, which is available on the GISAID platform, was extensively used to check AA substitutions and crosscheck any functional implications on specific regions, especially at the spike (S) surface glycoprotein. Replacements in the large polyprotein 1ab (nsp1-nsp16), four structural proteins (S, E, M, and N), and other accessory proteins (NS3, NS6, NS7a, NS7b, and NS8) by mutation, deletion, insertion, or existence of stop codon were analyzed to understand their biological signature.

## 3. Results

### 3.1. Phylogenetic Clusters and Evolutionary Relationship Analysis

**Figure 1:**
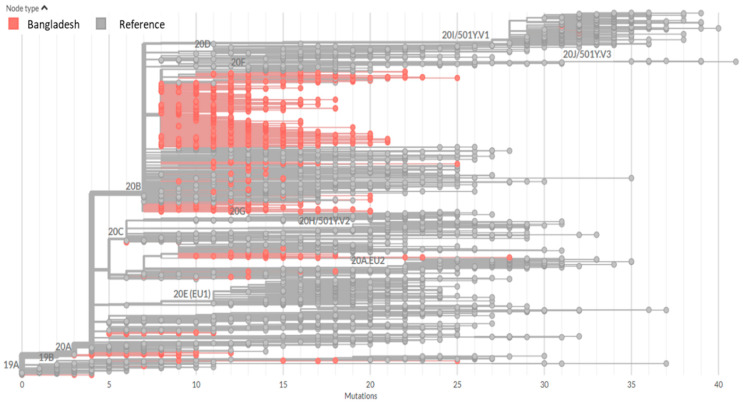
Nextstrain phylogeny tree showing evolutionary relationships of SARS-CoV-2 viruses. The tree was built using 791 Bangladeshi strains and 1902 global reference strains (Africa = 256, Asia = 431, Europe = 719, North America = 210, Oceania = 131, South America = 155). The red node suggests Bangladeshi strains.

**Figure 2:**
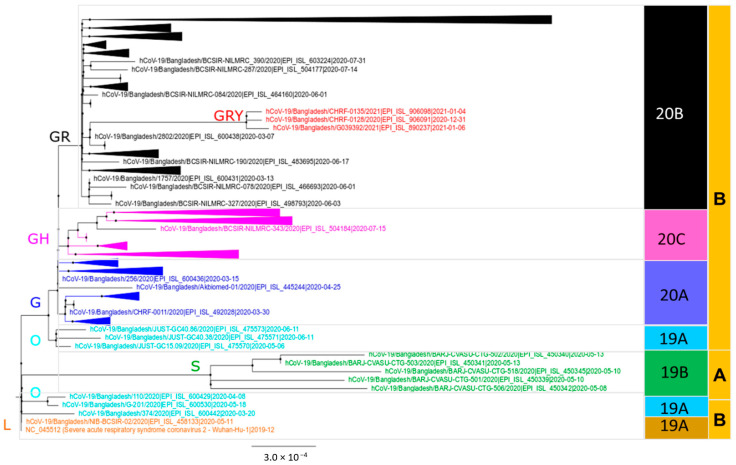
RAxML phylogenetic tree of 791 complete sequences of Bangladeshi SARS-CoV-2 showing the branch separating clades and lineages. The tree is rooted on Wuhan-Hu-1 (NC045512) reference strain. Seven different clades (GISAID) are highlighted as G = blue, GH = pink, GR = black, GRY = red, O = cyan, S = green, and L = orange. Five Nextstrain clades (20A, 20B, 20C, 19A, and 19B), as well as lineages A and B, are outlined in the left two-color columns. Compressed sub-tree indicates taxon names from similar clade branch/clusters. The full tree is available as Supplementary Figure S1.

The evolutionary fitness of SARS-CoV-2 was investigated by comparing the distance matrix (Supplementary Figure S2) among Bangladeshi strains of different clades or lineages and with the reference strain (Wuhan-Hu-1/NC_045512). Among SARS-CoV-2 sequences reported from Bangladesh, 241C > T, 3037C > T, 14408C > T, 23403A > G, 28881G > A, and 28883G > C mutations were the six most abundant nucleotide mutations that occurred in 98% sequences. Beside these, several sporadic and unique mutations were also found in the sequences analyzed. The Nextstrain clade assignment revealed that Bangladeshi SARS-CoV-2 produced at least five distinct clades: 20A, 20B, 20C, 19A, and 19B (Figure 1). Similarly, the RAxML tree produced from this dataset revealed the existence of seven clades (G, GH, GR, GRY, L, O, and S), with the majority of the sequences belonging to clade GR, which formed a broad cluster in the phylogenetic tree and was divided into several sub-branches (Figure 2; Supplementary Figure S1). In the lineage system, on the other hand, the existence of both lineages A (2%) and B (98%) was confirmed, where lineage B was the dominant lineage, which was further divided into 28 sub-lineages (Table 1). The lineage B.1.1.25 (*n* = 565) belonged to the GISAID clade GR and the Nextstrain clade 20B was the most prevalent lineage (71.4%) among the SARS-CoV-2 sequences reported from Bangladesh. The comparison with global B.1.1.25 sequences revealed that the Bangladeshi strains are closely related to strains from India, USA, Canada, UK, and Italy. The highly transmissible UK variant of lineage B.1.1.7 (Clade GRY) has been found in three sequences from Bangladesh so far.

**Figure 3:**
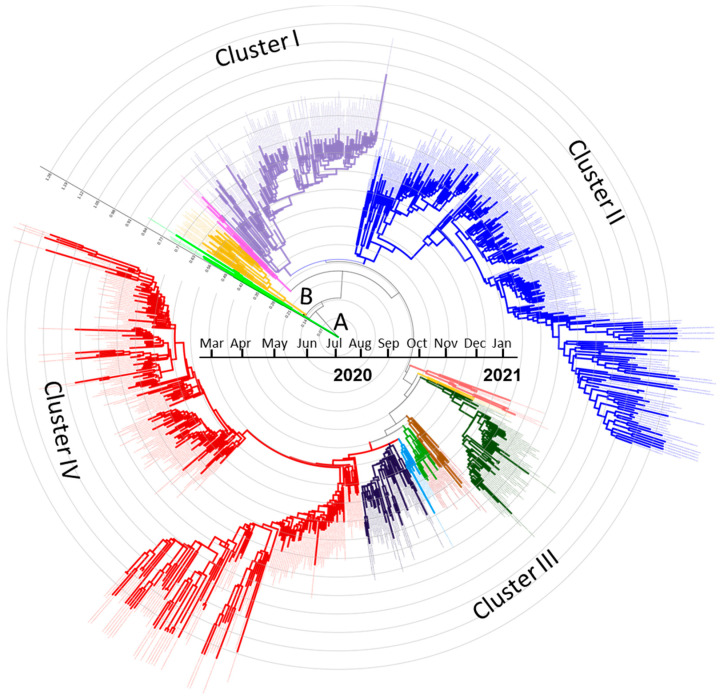
A time-scaled MCC tree indicating multiple clusters with many subdivisions (color separated). Viruses from March–September 2020 were incorporated in Cluster I, Cluster II, and Cluster III, whereas the viruses from October–December 2020 and January 2021 were assimilated into Cluster IV. All generated clusters belong to lineage B. Branch lengths were noted at the root of the tree. A and B indicate the origin of lineages A and B. The tree is rooted on Wuhan-Hu-1 (NC045512) reference strain. The full tree is available as Supplementary Figure S2.

The time-scaled MCC tree yielded from the sequence dataset (Figure 3) is broadly consistent with the generated ML tree (Figure 2). Four separate clusters emerged from the lineage B. Sequences sampled from March to September 2020, the first phase of the pandemic, were integrated into clusters I–III, with several subdivisions. Furthermore, Cluster IV emerged during the fourth quarter of 2020 to January 2021 (Figure 3; Supplementary Figure S2) and included some viruses from the first wave of the pandemic. Lineage A viruses were introduced in the country during March–May 2020 and have not been identified afterwards. By contrast, lineage B was introduced at the same time but quickly spread across the country, indicating that community transmission occurred from the strains of lineage B.

### 3.2. Clades and Lineage Distribution of Bangladeshi SARS-CoV-2 Strains

**Figure 4:**
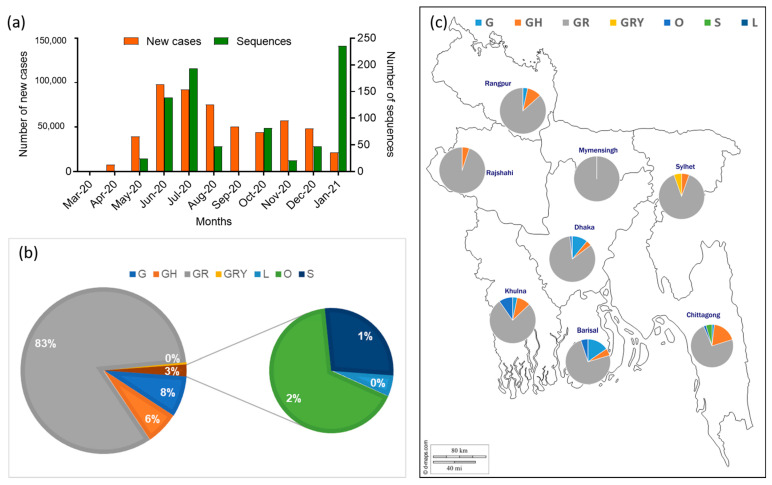
Distribution of SARS-CoV-2 genomes in Bangladesh. (**a**) The graph represents the number of sequences submitted in the GISAID database and the monthly confirmed new cases in Bangladesh. (**b**) The proportionate prevalence of the seven clades of SARS-CoV-2 detected in Bangladesh. (**c**) The proportionate distribution of seven clades of SARS-CoV-2 in eight administrative divisions of Bangladesh.

The analysis included 791 full genome sequences of SARS-CoV-2 strains submitted in the GISAID until 31 January 2021 from eight divisions of Bangladesh. Although the number of sequences submitted to databases were insufficient in comparison with the number of new cases reported from Bangladesh (Figure 4a), the sequence information would provide molecular insight into the SARS-CoV-2 circulating in the region. The highest number of full-genome sequences (*n* = 339) was submitted in the database from the capital city Dhaka, followed by Chattogram (*n* = 109), the second-largest metropolitan city, while a significant number of sequences (*n* = 149) were not listed under any division in the database.

Seven out of nine established clades classified for SARS-CoV-2 (GISAID) were identified in the country: G (*n* = 63), GH (*n* = 51), GR (*n* = 656), GRY (*n* = 3), L (*n* = 1), O (*n* = 12), S (*n* = 5), of which GR (83%) was the predominant clade (Figure 4b). When individual sequences from each division were analyzed, predominance of the GR clade was also observed in all eight divisions (Figure 4c; Supplementary Table S1).

The three clades G, GH, and O were detected at the beginning of the pandemic in March 2020, which continued till November 2020. A single isolate sampled during May 2020 clustered into clade L and soon disappeared. The most frequently detected clade GR (*n* = 656) was introduced in April 2020 and is now widely circulating in the country. The GRY clade was introduced in late December 2020, and only three strains have been reported so far. Considering the lineage classification system [10], both lineages A (*n* = 9) and B (*n* = 782) were detected in the country, where lineage B was the principal lineage that was further subdivided into several sub-lineages (Table 1).

### 3.3. Mutational Dimension and Variant Determination

**Figure 5:**
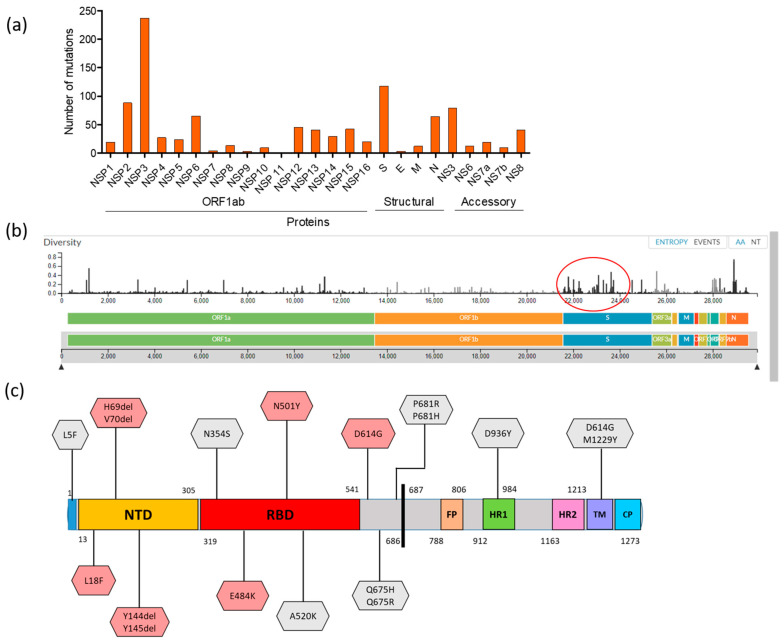
Mutational profile of Bangladeshi SARS-CoV-2. (**a**) Graph showing the total number of mutations detected by each encoded protein. (**b**) Nextstrain generated amino acid (AA) diversity graph of respective proteins; the spike protein had the most diversity (red circled). Diversity scale ranges from 0.0 to 0.8 are shown on the left side of the graph. (**c**) Illustration of selected amino acid substitutions in the S protein that are responsible for increased infectivity. The reddish substitutions are globally established, naturally occurring variants that increase infectivity, whereas the gray substitutions are found to be infective in experimental infection [8].

Several distinct amino acid (AA) substitutions were observed among Bangladeshi sequences. In 791 SARS-CoV-2 genome sequences, a total of 1023 AA mutations were identified, where 667 mutations were detected in polyprotein 1ab (nsp1-nsp16), 118 in spike (S) protein, and 3, 12, and 63 in E, M, and N protein, respectively. In addition, 159 mutations were detected in five accessory proteins (NS3 = 79, NS6 = 12, NS7a = 19, NS7b = 09, and NS8 = 40) (Figure 5a). Details of the mutations are listed in Supplementary Table S1. Within the polyprotein 1ab, the nsp3 encoded the highest number of mutations (*n* = 237). The rate of mutation in Bangladeshi SARS-CoV-2 was an estimated 22.213 substitutions per year. We further analyzed the AA diversity using the Nextclade web tools. The average diversity was highest in the S protein (0.0263) and lowest in NS7b (0.0078), measured as base substitution per site. The diversity calculated by the other protein can be found in Supplementary Table S2, and the diversity graph is shown in Figure 5b.

The identified 118 mutations in the S protein were critically analyzed to assess their biological significance and are listed in Supplementary Table S2. Several mutations, located at the S1 subunit, functionally linked to host transition, antigenic drift, host surface receptor-binding or antibody recognition sites, ligand binding and viral oligomerization interfaces, could all have a significant effect on pathogenesis and epidemiological signatures. In Bangladesh, the globally recognized mutation D614G (the most common form of SARS-CoV2) in the S protein was found in 767 out of 791 sequences (96%) belonging to lineage B (mainly B1.1.25). The other significant S protein mutations observed at the N-terminal domain (NTD) were L18F (*n* = 1), His69del (*n* = 5), V70del (*n* = 5), and Y144del/Y145 del (*n* = 7), depicted in Figure 4c. Notably, two potential mutations, E484K (B.1.1.25) and N501Y (B.1.1.7-UK Variant/GRY clade), at the receptor-binding domain (RBD) were determined in this analysis: E484K occurred in two strains sampled on 19 December 2020 from the capital city Dhaka (EPI_ISL_890188 and EPI_ISL_774976), whereas N501Y was found in three strains collected on 31 December 2020 (EPI_ISL_906091), 4 January 2021 (EPI_ISL_906098), and 6 January 2021 (EPI_ISL_890237), respectively, from another big city, Sylhet. In summary, the spike E484K mutation and the UK variant strain (B.1.1.7) were introduced in Bangladesh in late December 2020 and already emerged as the next dominant SARS-CoV-2 variant in the country. Furthermore, other potential mutations (L5F, N354S, A520K, Q675H/R, P681H/R, D936Y, and M1229Y) were detected in the current study (Figure 5c) and have been described for increased infectivity of the virus in vivo [8].

## 4. Discussion

In this study, we comprehensively analyzed 791 SARS-CoV-2 complete genome sequences from Bangladesh. Limited molecular analysis of Bangladeshi SARS-CoV-2 was reported previously [27,28,29]. We used different bioinformatics approaches to systemically identify the evolutionary matrix, major clades, and lineage designation among the circulated viruses. Further deduced AA mutations were detected in the virus population. The findings could provide a genetic background for the SARS-CoV-2 molecular epidemiological trends in Bangladesh.

The novel coronavirus SARS-CoV-2, which began the current pandemic, is expected to evolve further by accumulating mutations in its genome over time in global populations, much like other RNA viruses. Whole-genome sequence analysis is an unquestionable requirement for researchers and vaccine and therapeutic developers to keep track of the circulating virus strains. Furthermore, emergence of new variants is of particular concern for the ongoing vaccine effectiveness. SARS-CoV-2 was detected in Bangladesh in March 2020, showed many ups and downs over the year, and is still circulating. The phylogenetic analysis revealed genetic diversification among Bangladeshi strains. Phylogenetic analysis using the GISAID [9]/Nextstrain [11]/Lineage [10] nomenclature system identified several clades and lineages among Bangladeshi virus populations.

The time-scale MCC tree revealed four clusters of Bangladeshi strains without assigning any particular time period; however, strains from the last quarter of 2020 and January 2021 formed a distinct cluster, hereafter named Cluster IV in the current study. Single-nucleotide polymorphisms (SNPs) and nucleotide distance matrix (Supplementary Figure S2) are the reason behind the formation of multiple clusters. However, since these studies did not define a particular time frame or regional cluster, it is obvious that multiple strains are circulating throughout the nation, with the risk of rapid evolution. The GISAID or Nextstrain clades are differentiated based on their mutations in amino acid sequences. More than 97% of the Bangladeshi strains belonged to the G clade: its two major branches GH and GR clades and the newly introduced GRY clade. The common distinctive feature of these three clades is S_D614G mutation. The mutation S_D614G has become globally prevalent, which may increase the infectivity of SARS-CoV-2 [30] that has been observed in 95.12% of global sequences. The S glycoprotein plays pivotal roles in the viral infection and pathogenesis of SARS-CoV-2. The S protein is composed of two functional subunits, S1 and S2. The S1 subunit consists of an N-terminal domain (NTD) and a receptor-binding domain (RBD). The S1 subunit binds to the human angiotensin-converting enzyme-2 (ACE-2) receptors, which in turn mediate the fusion of the virus, the key to the infection process, whereas the S2 subunit contains fusion peptide (FP), heptad repeat 1 (HR1), central helix, connector domain, heptad repeat 2 (HR2), transmembrane domain (TM), and cytoplasmic domain (CD). The function of S2 subunit is to fuse the membranes of viruses with host cells [31,32]. The GR clade, in which the N_G204R mutation in the nucleocapsid protein is combined with the widespread S_D614G mutation, accounted for around 83% of the Bangladeshi sequences. Similarly, Nextstrain clade 20B and lineage B are also quite common in the country. B.1.1.25 is the most common lineage found in the current analysis, with 565 sequences closely related to global sequences of the same lineage from India, the United States, Canada, and the United Kingdom, as well as in low frequencies from Germany, Switzerland, Hong Kong, and Cyprus. However, the introduction of lineage B.1.1.7/GRY (UK variant/S_N501Y mutation) in late December 2020 in Bangladesh was of particular concern. Three variants of SARS-CoV-2, B.1.1.7 (UK variant), B.1.351 (South African variant), and P.1 (Brazilian variant), have exhibited distinct features that are extremely infectious and highly transmissible [13,33]. Another significant S_E484K mutation at the RBD, called an escape mutant [33,34], has already been found in the South African variant (B.1.351), Brazilian variant (P.1), and UK variant (B.1.1.7) and was also observed in the current study in two Bangladeshi strains under lineage B.1.1.25. This mutation helps the virus slip past the body’s immune response but does not abolish the neutralizing activity of convalescent or post-vaccinated sera [33,34,35]. Bangladeshi strains exhibited other S protein mutations or variants, as well as combined variants, such as L5F, L18F, N354S, A520K, Q675H/R, P681H/R, L5F + D614G, and D614G + M1229Y, which have been previously linked with increased infectivity under experimental setups [8] and may have significant biological properties in response to natural infection. In addition, some Bangladeshi strains had H69del + V70del, and Y144del or Y145del, which has shown a twofold higher infectivity than wild type [36], and has a decreased sensitivity to convalescent sera [8] respectively. Furthermore, mutations observed at Y789N, S803L, and N1074H in Bangladeshi strains that creating or deleting potential glycosylation sites, may also affect antigenic and other properties of the circulating strains [37]. Very recently, Bangladesh reported the presence of B.1.351 (South African variant) in the popular medium through IEDCR raised the possibility of many other new variants’ introduction anytime soon; however, that may require further extensive sequence analysis from currently circulating viruses.

Many other missense and synonymous mutations found in respective proteins of Bangladeshi virus populations such as ORF1ab/nsp12_P323L, NS3_Q57H, NS8_R52I, N_S194L, N_R203K, and N_G204R, which are also common worldwide [38]. The mutation NSP12_P323L plays a prominent role in protein folding and aggregation [39]. There were several other low-frequency mutations, and some novel mutations occurred in the Bangladeshi SARS-CoV-2 (Supplementary Table S1). These mutations seem to be rare, and they may be the result of the virus adapting to the host’s genetic history, environmental conditions, or other unknown causes that need to be investigated further.

Molecular genomic dynamics, using GISAID, Nextstrain, or other bioinformatics platforms applied to SARS-CoV-2 characterization in Bangladesh, revealed many aspects of the circulating viruses already known to the authorities, especially the introduction of SARS-CoV-2 strains into Bangladesh from many other foreign countries at the beginning of the pandemic. Our study further stated the new introduction of the highly transmissible S_E484K mutant and presence of the variant B.1.1.7 strain in Bangladesh. Due to high diversity and the continuously evolving nature of the virus, clade and lineage designation is being updated from time to time.

## 5. Conclusions

In conclusion, this study reports the genetic diversity that contributes to the identification and circulation pattern of SARS-CoV-2 clades and lineages in Bangladesh during the outbreak period in 2020. Of these 7 clades and 28 lineages, the GR clade and B.1.1.25 lineage were widely circulated and are likely to be the key reason for community transmission in the region. It cannot be ruled out that the transmissibility potential of the virus was accelerated by the widespread circulation of multiple lineages or clades and simultaneous distribution of SARS-CoV-2 strains, resulting in a true viral storm in such a densely populated area. Although infectivity and case fatality rates are lower in Bangladesh compared to many other countries, it is still uncertain if the differences in fatality rates or transmission speed observed in different countries are the result of clade virulence differences or other unknown factors. As a result, it is likely that the comparative genomic research would help in better understanding the virus pathogenesis and virulence.

## Figures and Tables

**Table 1:** Clade and lineage distribution of circulating strains of SARS-CoV-2 from Bangladesh.

Clades	Lineages	Number of Strains	Percentage	Time of Introduction	Present Status
G	A	63	7.9%	07-03-2020	14-07-2020
	B				
	B.1				
GH	B.1	51	6.4%	21-03-2020	23-11-2020
	B.1.159				
	B.1.260				
	B.1.36				
	B.1.36.16				
GR	B.1.1.10	656	82.9%	18-04-2020	31-01-2021 Circulating
	B.1.1.103				
	B.1.1.107				
	B.1.1.12				
	B.1.1.127				
	B.1.1.128				
	B.1.1.145				
	B.1.1.175				
	B.1.1.220				
	B.1.1.25				
	B.1.1.250				
	B.1.1.256				
	B.1.1.269				
	B.1.1.279				
	B.1.1.296				
	B.1.1.304				
	B.1.1.316				
	B.1.1.59				
	B.1.1.74				
	B.1.1.80				
GRY	B.1.1.7	3	0.3%	31-12-2020	31-01-2021 Circulating
L	B	1	0.1%	11-05-2020	Not seen to date
O	B	12	1.5%	20-03-2020	15-07-2020
	B.1				
	B.1.1.25				
	B.1.1.316				
	B.1.1.74				
	B.1.36.16				
	B.40				
S	A	5	0.6%	03-05-2020	13-05-2020
7 clades	28	791			

## Data Availability

The sequences analyzed in this study were downloaded from the GISAID (https://www.gisaid.org (accessed on 15 February 2021) database. The sequence metadata and other related documents generated for bioinformatics are available as Supplementary Files.

## Supplementary Materials

The following are available online at https://www.mdpi.com/article/10.3390/microorganisms9051035/s1: Figure S1: RAxML phylogenetic tree of 791 complete sequences of Bangladeshi SARS-CoV-2 showing branch-separating clades and lineages. The tree is rooted on Wuhan-Hu-1 (NC045512) reference strain. Seven different clades (GISAID) are highlighted as G = blue, GH = pink, GR = black, GRY = red, O = cyan, S = green, and L= orange. Figure S2: A time-scaled MCC tree indicating the multiple clusters with many subdivisions (color separated). The tree is rooted on Wuhan-Hu-1 (NC045512) reference strain. Table S1: Distribution of SARS-CoV-2 clades in eight divisions of Bangladesh. Table S2: Amino acid substitutions of spike (S) protein among Bangladeshi SARS-CoV-2 strains (sequence available at GISAID until 31 January 2021) compared to Spike_hCoV-19/Wuhan/WIV04/2019.
