# Supplementary material for: Molecular Analysis of SARS-CoV-2 Circulating in Bangladesh during 2020 Revealed Lineage Diversity and Potential Mutations
Source: Microorganisms. 2021 May 12;9(5):1035. doi: 10.3390/microorganisms9051035 (PMC8150345; doi:10.3390/microorganisms9051035)
Supplement: Supplementary file 1 [file microorganisms-09-01035-s001.zip › Supplemental Table S1.pdf]

Suppl. Table S1. Distribution of SARS-CoV-2 clades in eight divisions of Bangladesh

Division/ Total number of sequences (n)	Clades						
	G	GH	GR	GRY	0	S	L
Dhaka (n=339)	37 (10%)	13 (3%)	283 (83%)	0	5 (1%)	0	1 (0%)
Chattagram ((n=109)	2 (1%)	20 (18%)	80 (73%)	0	2 (1%)	5 (4%)	0
Rajshahi (n=40)	0	2 (5%)	38 (95%)	0	0	0	0
Khulna (n=31)	1 (3%)	3 (9%)	24 (77%)	0	3 (9%)	0	0
Sylhet (n=37)	0	2 (5%)	33 (89%)	2 (5%)	0	0	0
Barishal (n=39)	6 (15%)	2 (5%)	29 (74%)	0	2(5%)	0	0
Rangpur (n=30)	1 (3%)	3 (10%)	26 (86%)	0	0	0	0
Mymensingh (n=17)	0	0	17 (100%)	0	0	0	0
*Anonymous (n=149)	16 (10%)	6 (4%)	126 (85%)	1 (0%)	0	0	0
Total (n=791)	63	51	656	3	12	5	1

* The sequences are from Bangladesh but the division name is unknown.
