# Supplementary material for: Molecular Analysis of SARS-CoV-2 Circulating in Bangladesh during 2020 Revealed Lineage Diversity and Potential Mutations
Source: Microorganisms. 2021 May 12;9(5):1035. doi: 10.3390/microorganisms9051035 (PMC8150345; doi:10.3390/microorganisms9051035)
Supplement: Supplementary file 1 [file microorganisms-09-01035-s001.zip › Supplemental Table S2.pdf]

Suppl. Table S2. Amino acid substitutions of spike (S) protein among Bangladeshi SARS CoV 2 strains (sequence available at GISAID until 31st January 2021) compared to Spike_hCoV-19/Wuhan/WIV04/2019

Mutations	Biological signature/Functions (GISAID*)	Speciality
L5F	Antigenic Drift	Infectious variant in an in vivo experimental [8]
S12F	N/A	N/A
S13I	N/A	N/A
Q14H	Antigenic Drift	N/A
Q14R	Antigenic Drift	N/A
C15F	N/A	N/A
L18F	Antigenic Drift	N/A
P26L	N/A	N/A
A27S	N/A	N/A
T29I	N/A	N/A
F32L	N/A	N/A
F43L	Antibody Recognition Sites<br>Viral Oligomerization Interfaces	N/A
H49Y	Antibody Recognition Sites<br>Viral Oligomerization Interfaces	N/A
A67S	N/A	N/A
I68T	N/A	N/A
H69del	Antigenic Drift	H69del+V70del have 2-fold higher infectivity compared to wild type [36]
V70del	Antigenic Drift	
G75V	N/A	N/A
T76I	N/A	N/A
T95I	N/A	N/A
E96A		N/A
S98F	N/A	N/A
R102I	N/A	N/A
S112L	Antibody Recognition Sites<br>Viral Oligomerization Interfaces	N/A
V127F	N/A	N/A
D138Y	N/A	N/A
D138H	N/A	N/A
G142V	N/A	N/A
V143F	Antibody Recognition Sites	N/A
Y144del	Antigenic Drift<br>Antibody Recognition Sites	Decreased sensitivity to convalescent sera [8]
Y145del	Antibody Recognition Sites	Y145del is similar to Y144del in SARS-CoV-2 [8]
H146Y	N/A	N/A
H146Q	Antibody Recognition Sites<br>Ligand Binding	N/A
W152L	Antibody Recognition Sites	N/A
E154G	N/A	N/A
S155I	N/A	N/A
E156D	N/A	N/A
F157L	N/A	N/A
G172C	N/A	N/A

L176F	N/A	N/A
M177I	N/A	N/A
G184D	N/A	N/A
F186L	N/A	N/A
N211Y	N/A	N/A
V213L	N/A	N/A
D215Y	N/A	N/A
S221L	N/A	N/A
T236S	Viral Oligomerization Interfaces	N/A
Y248H	N/A	N/A
G261R	N/A	N/A
N354S	Host Change and Antigenic Drift<br>Antibody Recognition Sites<br>Viral Oligomerization Interfaces	N/A
S359T	Host Change.<br>Ligand Binding<br>Viral Oligomerization Interfaces	N/A
V382L	Antigenic Drift<br>Antibody Recognition Sites<br>Viral Oligomerization Interfaces	N/A
E484K	Host Change and Antigenic Drift<br>Host Cell Receptor Binding<br>Antibody Recognition Sites	In a deep mutational scanning experiment that expresses Spike RBD in a yeast-display platform, E484K mildly increases the binding to ACE2 (apparent dissociation constant delta-log10 value: 0.06) [33]
N501Y	Antigenic Drift and Host Change<br>Host Surface Receptor Binding<br>Antibody Recognition Sites<br>Viral Oligomerization Interfaces	In a deep mutational scanning experiment that expresses Spike RBD in a yeast-display platform, N501Y mildly increases the binding to ACE2 (apparent dissociation constant delta-log10 value: 0.24) [33]
E516Q	Antibody Recognition Sites<br>Viral Oligomerization Interfaces	N/A
L518I	Only in Bangladesh	N/A
A520S	Host Change<br>Viral Oligomerization Interfaces	N/A
T547I	Viral Oligomerization Interfaces	N/A
K558N	Ligand Binding<br>Viral Oligomerization Interfaces	N/A
I569S	Viral Oligomerization Interfaces	N/A
A570D	Viral Oligomerization Interfaces	N/A
T573I	Viral Oligomerization Interfaces	N/A
D574Y	N/A	N/A
G594S	N/A	N/A
I569S	Viral Oligomerization Interfaces	Bangladesh only
D614G	Ligand Binding<br>Viral Oligomerization Interfaces	Most common in Bangladesh<br>D614G mutant increases the infectivity SARS-CoV-2 [3*0*]
V622F	N/A	N/A
S640Y	N/A	N/A

A647S	Viral Oligomerization Interfaces	N/A
A653V	N/A	N/A
E654Q	N/A	N/A
N658D	N/A	N/A
Y660F	N/A	N/A
Q675H	Antigenic Drift	N/A
Q675R	Antigenic Drift	N/A
Q677H	N/A	N/A
N679K	N/A	N/A
S698L	Viral Oligomerization Interfaces	N/A
P681R	Furin Cleavage Site	N/A
P681H	Furin Cleavage Site	N/A
T716I	N/A	N/A
G769V	Viral Oligomerization Interfaces	N/A
A771S	N/A	N/A
A783S	N/A	N/A
Q787L	Viral Oligomerization Interfaces	N/A
Y789N	Viral Oligomerization Interfaces	Creates a new potential N-glycosylation site at position 789 which may also affect antigenic and other properties of this strain. In detail, the motif at positions 789-791 changed from YKT (no glyco) to NKT (glyco) [37].
T791I	N/A	N/A
F797C	Ligand Binding<br>Viral Oligomerization Interfaces	N/A
G799S	Viral Oligomerization Interfaces	N/A
S803L		Removes a potential N-glycosylation site at position 801 which may also affect antigenic and other properties of this strain. In detail, the motif at positions 801-803 changed from NFS (glyco) to NFL (no glyco) [37].
I834V	Ligand Binding<br>Viral Oligomerization Interfaces	N/A
D843B	Viral Oligomerization Interfaces	N/A
K854N	Viral Oligomerization Interfaces	N/A
A871V	Antigenic Drift<br>Viral Oligomerization Interfaces	N/A
D936Y	N/A	N/A
S939F	N/A	N/A
T941A	N/A	N/A
A942V	Viral Oligomerization Interfaces	N/A
S982A	Viral Oligomerization Interfaces	N/A
V1068F	Viral Oligomerization Interfaces	N/A
N1074H	Antigenic drift<br>Ligand Binding<br>Viral Oligomerization Interfaces	Mutation Spike N1074H removes a potential N-glycosylation site at position 1074 which may also affect antigenic and other properties of this strain. In detail, the motif at positions 1074-1076 changed from

		NFT (glyco) to HFT (no glyco) [37].
D1084Y	N/A	N/A
D1084H	N/A	N/A
R1091H	N/A	N/A
V1104L	N/A	N/A
F1109L	N/A	N/A
T1117I	Viral Oligomerization Interfaces	N/A
D1118Y	Viral Oligomerization Interfaces	N/A
D1118H	Viral Oligomerization Interfaces	N/A
V1122L	Viral Oligomerization Interfaces	N/A
G1167V	Viral Oligomerization Interfaces	N/A
R1185H	N/A	N/A
K1191N	Viral Oligomerization Interfaces	N/A
M1229I	N/A	N/A
M1233I	N/A	N/A
C1247F	N/A	N/A
C1250F	N/A	N/A
**118**		

*Functions are mentioned in GISAID CovSurver
