# Supplementary figures and images for: Molecular Analysis of SARS-CoV-2 Circulating in Bangladesh during 2020 Revealed Lineage Diversity and Potential Mutations
Source: Microorganisms. 2021 May 12;9(5):1035. doi: 10.3390/microorganisms9051035 (PMC8150345; doi:10.3390/microorganisms9051035)

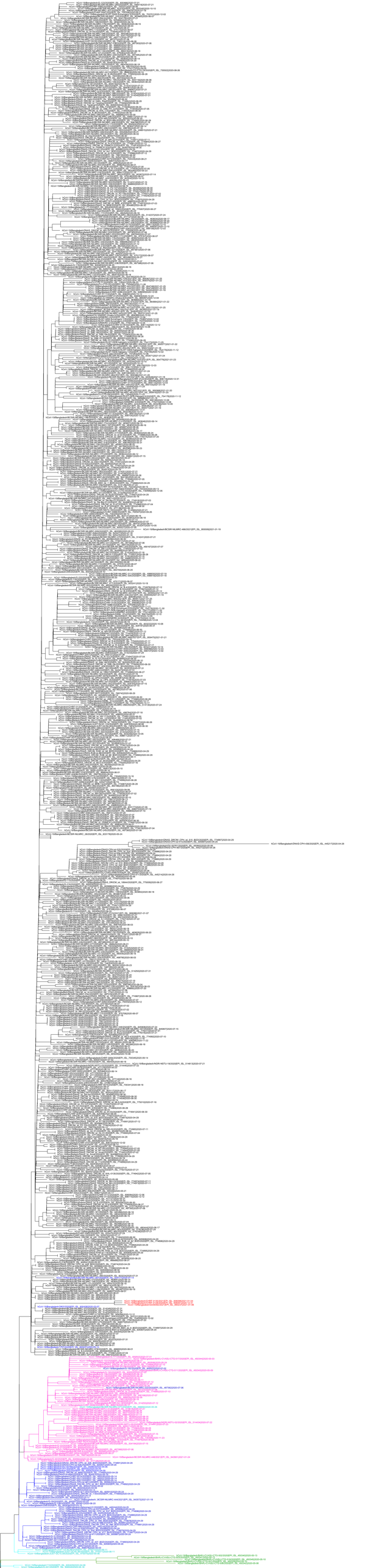

Supplement: Supplementary file 1 [file microorganisms-09-01035-s001.zip › Supplementa Fig. S1.pdf]

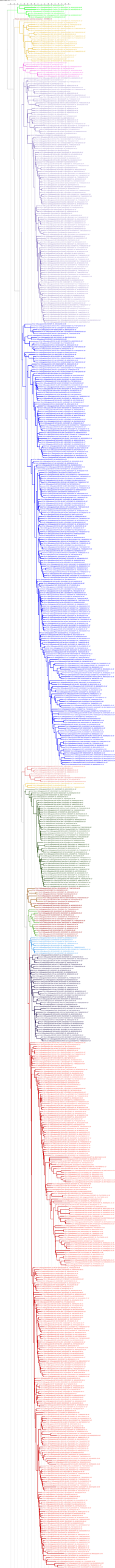

Supplement: Supplementary file 1 [file microorganisms-09-01035-s001.zip › Supplemental Fig. S2.pdf]
