# Supplementary material for: Molecular Analysis of SARS-CoV-2 Circulating in Bangladesh during 2020 Revealed Lineage Diversity and Potential Mutations
Source: Microorganisms. 2021 May 12;9(5):1035. doi: 10.3390/microorganisms9051035 (PMC8150345; doi:10.3390/microorganisms9051035)
Supplement: Supplementary file 1 [file Supplemental file 2_FastME_Distance_matrix.pdf › Supplemental file 2_FastME_Distance_matrix.pdf]

853

S001	0.000000	0.000000	0.012337	0.012101	0.012101	0.012169	

0.012011	0.011943	0.012169	0.011597	0.011664	0.011632	0.011597
0.011631	0.011799	0.011597	0.011631	0.011698	0.011664	0.011631
0.011597	0.011631	0.011732	0.011598	0.011631	0.011631	0.011977
0.011866	0.011631	0.011530	0.011765	0.011698	0.011665	0.011698
0.011664	0.011765	0.011664	0.011664	0.011698	0.011664	0.011832
0.011597	0.011664	0.011698	0.011631	0.011732	0.011765	0.011698
0.011664	0.011833	0.011732	0.012000	0.011664	0.011933	0.011664
0.011631	0.011732	0.011799	0.011933	0.012034	0.011732	0.011832
0.011698	0.011765	0.011664	0.011900	0.012135	0.011664	0.011732
0.011631	0.011732	0.011698	0.011765	0.011698	0.011698	0.011698
0.011597	0.011631	0.011597	0.011698	0.011664	0.011700	0.011765
0.011698	0.011664	0.011732	0.011631	0.011765	0.011597	0.011664
0.011832	0.011765	0.012000	0.011832	0.011664	0.011900	0.011664
0.011616	0.011845	0.011664	0.011373	0.011620	0.011686	0.011572
0.012452	0.011620	0.011348	0.011899	0.011900	0.011698	0.011799
0.011732	0.011631	0.011564	0.011664	0.011765	0.011698	0.011564
0.011597	0.011765	0.011900	0.011832	0.011732	0.011698	0.011631
0.011597	0.011597	0.011665	0.011631	0.011833	0.011799	0.011732
0.011833	0.011900	0.011631	0.011631	0.011665	0.011665	0.011668
0.011737	0.011665	0.011665	0.011564	0.011639	0.011586	0.011665
0.011670	0.011610	0.011688	0.011647	0.011570	0.011608	0.011634
0.011924	0.011681	0.011715	0.011598	0.011613	0.011733	0.011699
0.011767	0.011701	0.011699	0.011664	0.011463	0.012101	0.011900
0.011933	0.011799	0.011664	0.011766	0.011564	0.011799	0.011598
0.011664	0.011664	0.011631	0.011597	0.011631	0.011597	0.011698
0.011665	0.011766	0.011698	0.011597	0.011701	0.011799	0.011804
0.011799	0.011699	0.011597	0.011532	0.011665	0.011598	0.011531
0.011638	0.011799	0.011598	0.011799	0.011564	0.011665	0.011631
0.011732	0.011631	0.011665	0.011631	0.011665	0.011530	0.011665
0.011698	0.011631	0.011698	0.011631	0.011530	0.011698	0.011934
0.012034	0.011967	0.012035	0.011802	0.012035	0.011530	0.011631
0.011668	0.012031	0.011792	0.011575	0.012045	0.011538	0.011651
0.012045	0.011945	0.011799	0.011967	0.011538	0.011900	0.011866
0.011799	0.011967	0.011664	0.011596	0.011689	0.011698	0.011703
0.011866	0.011634	0.011872	0.011974	0.011765	0.011900	0.011941
0.011669	0.011943	0.011670	0.011838	0.012036	0.011564	0.011768
0.011732	0.011664	0.011564	0.011564	0.011631	0.011631	0.011698
0.011631	0.011698	0.011597	0.011698	0.011631	0.011631	0.011698
0.011631	0.012068	0.011967	0.012001	0.011530	0.011631	0.011664
0.011664	0.011631	0.011698	0.011732	0.011597	0.011597	0.011597
0.012135	0.011765	0.011765	0.011564	0.011664	0.011833	0.011597
0.011597	0.011664	0.011631	0.011799	0.011597	0.011732	0.011866
0.011799	0.011631	0.011631	0.011564	0.011564	0.011732	0.011664
0.011564	0.011664	0.011530	0.011564	0.011698	0.011833	0.011597
0.011564	0.011698	0.011632	0.011530	0.011866	0.011530	0.011698
0.011642	0.011597	0.011900	0.011732	0.011765	0.011631	0.011732
0.011597	0.011933	0.011530	0.011799	0.011597	0.011597	0.011530
0.011765	0.011967	0.011664	0.011732	0.011564	0.011967	0.011597
0.011597	0.011597	0.011631	0.011732	0.011631	0.011732	0.011799
0.011799	0.011765	0.011664	0.011765	0.011732	0.011597	0.011564
0.011597	0.011597	0.011664	0.011597	0.011564	0.011732	0.011597
0.011631	0.011631	0.011765	0.011799	0.011698	0.011631	0.011631
0.011698	0.011631	0.011732	0.011597	0.011732	0.011832	0.012135
0.011631	0.012034	0.011647	0.018202	0.011387	0.011611	0.012002
0.011960	0.012010	0.011532	0.011466	0.011939	0.012027	0.011546
0.011629	0.011732	0.011333	0.011437	0.011685	0.011505	0.011447
0.011623	0.011843	0.011839	0.011722	0.011765	0.011262	0.011737
0.011644	0.011765	0.011765	0.011732	0.011698	0.011698	0.011665

0.011698	0.011631	0.011665	0.011698	0.011665	0.011598	0.011497
0.011497	0.011698	0.011597	0.011698	0.011732	0.011765	0.011530
0.011698	0.011665	0.012001	0.012001	0.011732	0.011766	0.011799
0.011564	0.011564	0.011597	0.011698	0.011564	0.011799	0.011799
0.011833	0.011799	0.011765	0.011866	0.012035	0.011869	0.011698
0.011698	0.011766	0.011799	0.011631	0.011698	0.011665	0.011698
0.011766	0.011766	0.011799	0.011766	0.011666	0.011866	0.011833
0.011704	0.011598	0.011698	0.011799	0.011698	0.011833	0.011866
0.011967	0.011538	0.011900	0.011866	0.011799	0.011967	0.011598
0.011768	0.011665	0.011497	0.011497	0.011698	0.011597	0.011698
0.011732	0.011765	0.011530	0.011698	0.011665	0.012001	0.012001
0.011732	0.011766	0.011564	0.011497	0.012373	0.011775	0.011588
0.011799	0.011754	0.011763	0.011718	0.011830	0.011716	0.011784
0.012262	0.011510	0.011643	0.011614	0.011921	0.011843	0.011945
0.012284	0.011776	0.011940	0.012001	0.011866	0.011866	0.011905
0.011564	0.011732	0.011866	0.011832	0.011838	0.011832	0.011801
0.011664	0.011597	0.011631	0.011664	0.011564	0.011631	0.011530
0.011664	0.011698	0.011597	0.011564	0.011664	0.011597	0.011631
0.011631	0.011564	0.011631	0.011664	0.011564	0.011765	0.011799
0.011698	0.011765	0.011698	0.011698	0.011631	0.012034	0.011664
0.011687	0.011631	0.011867	0.011875	0.011732	0.011732	0.011732
0.011732	0.011664	0.011631	0.011664	0.011765	0.011765	0.011698
0.011698	0.011832	0.012000	0.011732	0.011933	0.011933	0.011799
0.011631	0.011698	0.011732	0.012135	0.011699	0.011732	0.011698
0.011732	0.011664	0.011664	0.011967	0.011900	0.011933	0.011664
0.011765	0.011832	0.011698	0.011765	0.011732	0.011597	0.011933
0.011698	0.011664	0.011664	0.011832	0.011664	0.011765	0.012001
0.011698	0.012079	0.012068	0.011670	0.011399	0.011774	0.011370
0.011500	0.011917	0.011622	0.011641	0.011402	0.011518	0.011538
0.011672	0.011389	0.011506	0.011390	0.011384	0.011596	0.011506
0.011550	0.011454	0.011765	0.011694	0.011701	0.011833	0.011900
0.011900	0.011766	0.011872	0.011874	0.012010	0.012050	0.011698
0.011933	0.011799	0.011664	0.011766	0.011564	0.011799	0.011598
0.011664	0.011664	0.011631	0.011597	0.011631	0.011597	0.011698
0.011689	0.011690	0.011677	0.011711	0.011847	0.011812	0.011812
0.011810	0.011733	0.011540	0.011564	0.011670	0.011840	0.011841
0.011632	0.011704	0.011936	0.011905	0.011834	0.011868	0.011530
0.011732	0.011732	0.011732	0.011698	0.012236	0.011866	0.011933
0.011967	0.012057	0.011674	0.011753	0.011631	0.011698	0.011262
0.011834	0.011732	0.011866	0.011866	0.012004	0.011866	0.011650
0.011533	0.011510	0.011605	0.011604	0.011709	0.011545	0.011876
0.011678	0.011806	0.011584	0.011510	0.011666	0.011745	0.011631
0.011597	0.011597	0.011665	0.011631	0.011833	0.011799	0.011732
0.011833	0.011900	0.011631	0.011631	0.011665	0.011665	0.011668
0.011737	0.011665	0.011665	0.011701	0.011833	0.011900	0.011900
0.011766	0.011872	0.011874	0.012010	0.012050	0.011698	0.011866
0.011262	0.011262	0.011635	0.011826	0.011755	0.011775	0.011656
0.011818	0.012063	0.011907	0.011706	0.011598	0.011799	0.011665
0.011766	0.011698	0.011597	0.011701	0.011799	0.011804	0.011699
0.011597	0.011532	0.011665	0.011598	0.011531	0.011638	0.011799
0.011698	0.011631	0.011665	0.011698	0.011665	0.011598	0.011799
0.011664	0.011698	0.011698	0.011631	0.011698	0.011933	0.011597
0.011631	0.012034	0.011631	0.011664	0.011631	0.012000	0.011403
0.011903	0.011358	0.011411	0.011866	0.011671	0.011850	0.011353
0.011375	0.011799	0.011900	0.012011	0.011868	0.012008	0.012137
0.012036	0.012075	0.012034	0.012222	0.012001	0.012741	0.012038
0.012777	0.012410	0.012544	0.012951	0.012000	0.011698	0.012001
0.012407	0.012376	0.011967	0.011866	0.011935	0.011935	0.011906
0.011799	0.011807	0.011866	0.011834	0.012204	0.012270	0.012272
0.012171	0.012238	0.012376	0.012143	0.012408	0.012415	0.012475

0.012475	0.012509	0.012460	0.012476	0.012477	0.011463	0.012002
0.012103	0.012607	0.012078	0.012337	0.012812	0.011876	0.011766
0.011732	0.011732	0.012203	0.012213	0.012363	0.012068	0.011734
S002	0.000000	0.000000	0.012337	0.012101	0.012101	0.012169
0.012011	0.011943	0.012169	0.011597	0.011664	0.011632	0.011597
0.011631	0.011799	0.011597	0.011631	0.011698	0.011664	0.011631
0.011597	0.011631	0.011732	0.011598	0.011631	0.011631	0.011977
0.011866	0.011631	0.011530	0.011765	0.011698	0.011665	0.011698
0.011664	0.011765	0.011664	0.011664	0.011698	0.011664	0.011832
0.011597	0.011664	0.011698	0.011631	0.011732	0.011765	0.011698
0.011664	0.011833	0.011732	0.012000	0.011664	0.011933	0.011664
0.011631	0.011732	0.011799	0.011933	0.012034	0.011732	0.011832
0.011698	0.011765	0.011664	0.011900	0.012135	0.011664	0.011732
0.011631	0.011732	0.011698	0.011765	0.011698	0.011698	0.011698
0.011597	0.011631	0.011597	0.011698	0.011664	0.011700	0.011765
0.011698	0.011664	0.011732	0.011631	0.011765	0.011597	0.011664
0.011832	0.011765	0.012000	0.011832	0.011664	0.011900	0.011664
0.011616	0.011845	0.011664	0.011373	0.011620	0.011686	0.011572
0.012452	0.011620	0.011348	0.011899	0.011900	0.011698	0.011799
0.011732	0.011631	0.011564	0.011664	0.011765	0.011698	0.011564
0.011597	0.011765	0.011900	0.011832	0.011732	0.011698	0.011631
0.011597	0.011597	0.011665	0.011631	0.011833	0.011799	0.011732
0.011833	0.011900	0.011631	0.011631	0.011665	0.011665	0.011668
0.011737	0.011665	0.011665	0.011564	0.011639	0.011586	0.011665
0.011670	0.011610	0.011688	0.011647	0.011570	0.011608	0.011634
0.011924	0.011681	0.011715	0.011598	0.011613	0.011733	0.011699
0.011767	0.011701	0.011699	0.011664	0.011463	0.012101	0.011900
0.011933	0.011799	0.011664	0.011766	0.011564	0.011799	0.011598
0.011664	0.011664	0.011631	0.011597	0.011631	0.011597	0.011698
0.011665	0.011766	0.011698	0.011597	0.011701	0.011799	0.011804
0.011799	0.011699	0.011597	0.011532	0.011665	0.011598	0.011531
0.011638	0.011799	0.011598	0.011799	0.011564	0.011665	0.011631
0.011732	0.011631	0.011665	0.011631	0.011665	0.011530	0.011665
0.011698	0.011631	0.011698	0.011631	0.011530	0.011698	0.011934
0.012034	0.011967	0.012035	0.011802	0.012035	0.011530	0.011631
0.011668	0.012031	0.011792	0.011575	0.012045	0.011538	0.011651
0.012045	0.011945	0.011799	0.011967	0.011538	0.011900	0.011866
0.011799	0.011967	0.011664	0.011596	0.011689	0.011698	0.011703
0.011866	0.011634	0.011872	0.011974	0.011765	0.011900	0.011941
0.011669	0.011943	0.011670	0.011838	0.012036	0.011564	0.011768
0.011732	0.011664	0.011564	0.011564	0.011631	0.011631	0.011698
0.011631	0.011698	0.011597	0.011698	0.011631	0.011631	0.011698
0.011631	0.012068	0.011967	0.012001	0.011530	0.011631	0.011664
0.011664	0.011631	0.011698	0.011732	0.011597	0.011597	0.011597
0.012135	0.011765	0.011765	0.011564	0.011664	0.011833	0.011597
0.011597	0.011664	0.011631	0.011799	0.011597	0.011732	0.011866
0.011799	0.011631	0.011631	0.011564	0.011564	0.011732	0.011664
0.011564	0.011664	0.011530	0.011564	0.011698	0.011833	0.011597
0.011564	0.011698	0.011632	0.011530	0.011866	0.011530	0.011698
0.011642	0.011597	0.011900	0.011732	0.011765	0.011631	0.011732
0.011597	0.011933	0.011530	0.011799	0.011597	0.011597	0.011530
0.011765	0.011967	0.011664	0.011732	0.011564	0.011967	0.011597
0.011597	0.011597	0.011631	0.011732	0.011631	0.011732	0.011799
0.011799	0.011765	0.011664	0.011765	0.011732	0.011597	0.011564
0.011597	0.011597	0.011664	0.011597	0.011564	0.011732	0.011597
0.011631	0.011631	0.011765	0.011799	0.011698	0.011631	0.011631
0.011698	0.011631	0.011732	0.011597	0.011732	0.011832	0.012135
0.011631	0.012034	0.011647	0.018202	0.011387	0.011611	0.012002
0.011960	0.012010	0.011532	0.011466	0.011939	0.012027	0.011546
0.011629	0.011732	0.011333	0.011437	0.011685	0.011505	0.011447

0.011623	0.011843	0.011839	0.011722	0.011765	0.011262	0.011737
0.011644	0.011765	0.011765	0.011732	0.011698	0.011698	0.011665
0.011698	0.011631	0.011665	0.011698	0.011665	0.011598	0.011497
0.011497	0.011698	0.011597	0.011698	0.011732	0.011765	0.011530
0.011698	0.011665	0.012001	0.012001	0.011732	0.011766	0.011799
0.011564	0.011564	0.011597	0.011698	0.011564	0.011799	0.011799
0.011833	0.011799	0.011765	0.011866	0.012035	0.011869	0.011698
0.011698	0.011766	0.011799	0.011631	0.011698	0.011665	0.011698
0.011766	0.011766	0.011799	0.011766	0.011666	0.011866	0.011833
0.011704	0.011598	0.011698	0.011799	0.011698	0.011833	0.011866
0.011967	0.011538	0.011900	0.011866	0.011799	0.011967	0.011598
0.011768	0.011665	0.011497	0.011497	0.011698	0.011597	0.011698
0.011732	0.011765	0.011530	0.011698	0.011665	0.012001	0.012001
0.011732	0.011766	0.011564	0.011497	0.012373	0.011775	0.011588
0.011799	0.011754	0.011763	0.011718	0.011830	0.011716	0.011784
0.012262	0.011510	0.011643	0.011614	0.011921	0.011843	0.011945
0.012284	0.011776	0.011940	0.012001	0.011866	0.011866	0.011905
0.011564	0.011732	0.011866	0.011832	0.011838	0.011832	0.011801
0.011664	0.011597	0.011631	0.011664	0.011564	0.011631	0.011530
0.011664	0.011698	0.011597	0.011564	0.011664	0.011597	0.011631
0.011631	0.011564	0.011631	0.011664	0.011564	0.011765	0.011799
0.011698	0.011765	0.011698	0.011698	0.011631	0.012034	0.011664
0.011687	0.011631	0.011867	0.011875	0.011732	0.011732	0.011732
0.011732	0.011664	0.011631	0.011664	0.011765	0.011765	0.011698
0.011698	0.011832	0.012000	0.011732	0.011933	0.011933	0.011799
0.011631	0.011698	0.011732	0.012135	0.011699	0.011732	0.011698
0.011732	0.011664	0.011664	0.011967	0.011900	0.011933	0.011664
0.011765	0.011832	0.011698	0.011765	0.011732	0.011597	0.011933
0.011698	0.011664	0.011664	0.011832	0.011664	0.011765	0.012001
0.011698	0.012079	0.012068	0.011670	0.011399	0.011774	0.011370
0.011500	0.011917	0.011622	0.011641	0.011402	0.011518	0.011538
0.011672	0.011389	0.011506	0.011390	0.011384	0.011596	0.011506
0.011550	0.011454	0.011765	0.011694	0.011701	0.011833	0.011900
0.011900	0.011766	0.011872	0.011874	0.012010	0.012050	0.011698
0.011933	0.011799	0.011664	0.011766	0.011564	0.011799	0.011598
0.011664	0.011664	0.011631	0.011597	0.011631	0.011597	0.011698
0.011689	0.011690	0.011677	0.011711	0.011847	0.011812	0.011812
0.011810	0.011733	0.011540	0.011564	0.011670	0.011840	0.011841
0.011632	0.011704	0.011936	0.011905	0.011834	0.011868	0.011530
0.011732	0.011732	0.011732	0.011698	0.012236	0.011866	0.011933
0.011967	0.012057	0.011674	0.011753	0.011631	0.011698	0.011262
0.011834	0.011732	0.011866	0.011866	0.012004	0.011866	0.011650
0.011533	0.011510	0.011605	0.011604	0.011709	0.011545	0.011876
0.011678	0.011806	0.011584	0.011510	0.011666	0.011745	0.011631
0.011597	0.011597	0.011665	0.011631	0.011833	0.011799	0.011732
0.011833	0.011900	0.011631	0.011631	0.011665	0.011665	0.011668
0.011737	0.011665	0.011665	0.011701	0.011833	0.011900	0.011900
0.011766	0.011872	0.011874	0.012010	0.012050	0.011698	0.011866
0.011262	0.011262	0.011635	0.011826	0.011755	0.011775	0.011656
0.011818	0.012063	0.011907	0.011706	0.011598	0.011799	0.011665
0.011766	0.011698	0.011597	0.011701	0.011799	0.011804	0.011699
0.011597	0.011532	0.011665	0.011598	0.011531	0.011638	0.011799
0.011698	0.011631	0.011665	0.011698	0.011665	0.011598	0.011799
0.011664	0.011698	0.011698	0.011631	0.011698	0.011933	0.011597
0.011631	0.012034	0.011631	0.011664	0.011631	0.012000	0.011403
0.011903	0.011358	0.011411	0.011866	0.011671	0.011850	0.011353
0.011375	0.011799	0.011900	0.012011	0.011868	0.012008	0.012137
0.012036	0.012075	0.012034	0.012222	0.012001	0.012741	0.012038
0.012777	0.012410	0.012544	0.012951	0.012000	0.011698	0.012001
0.012407	0.012376	0.011967	0.011866	0.011935	0.011935	0.011906

0.011799	0.011807	0.011866	0.011834	0.012204	0.012270	0.012272
0.012171	0.012238	0.012376	0.012143	0.012408	0.012415	0.012475
0.012475	0.012509	0.012460	0.012476	0.012477	0.011463	0.012002
0.012103	0.012607	0.012078	0.012337	0.012812	0.011876	0.011766
0.011732	0.011732	0.012203	0.012213	0.012363	0.012068	0.011734
S003	0.012337	0.012337	0.000000	0.011429	0.011429	0.011496
0.012046	0.011978	0.011631	0.011765	0.012001	0.011767	0.011933
0.011900	0.012135	0.011866	0.011967	0.012034	0.012001	0.011967
0.011933	0.011967	0.011732	0.011901	0.011934	0.011967	0.012011
0.011933	0.011967	0.011866	0.011765	0.012034	0.011833	0.012034
0.012001	0.012101	0.012001	0.012001	0.012034	0.012000	0.012169
0.011866	0.012000	0.012034	0.011967	0.012001	0.012101	0.012034
0.012001	0.012169	0.012069	0.012068	0.012001	0.012270	0.012001
0.011967	0.012001	0.012102	0.012270	0.012102	0.012001	0.012101
0.012034	0.012102	0.011933	0.012236	0.012203	0.012001	0.012068
0.011967	0.012068	0.011967	0.012101	0.012034	0.012034	0.012034
0.011933	0.011967	0.011933	0.011967	0.012000	0.012037	0.012101
0.012034	0.012000	0.012068	0.011967	0.012034	0.011933	0.012001
0.012169	0.012101	0.012135	0.012169	0.012001	0.012236	0.012001
0.011988	0.011882	0.012004	0.011685	0.011695	0.011797	0.011533
0.012158	0.011671	0.011761	0.012236	0.012236	0.011967	0.012068
0.012068	0.012034	0.012101	0.012001	0.012068	0.012034	0.012034
0.012001	0.012034	0.012303	0.012169	0.012068	0.012035	0.011967
0.011934	0.011866	0.012001	0.011967	0.011833	0.011799	0.011732
0.011833	0.011900	0.011967	0.011967	0.012001	0.012001	0.012007
0.012075	0.012001	0.012001	0.011672	0.011711	0.011656	0.011800
0.011772	0.011713	0.011761	0.011750	0.011706	0.011711	0.011770
0.011744	0.011784	0.011818	0.011733	0.011717	0.011868	0.011767
0.011834	0.011836	0.011834	0.012135	0.012068	0.012438	0.012169
0.012001	0.012068	0.012001	0.012035	0.011900	0.011866	0.011934
0.012001	0.012001	0.011967	0.011933	0.011967	0.011934	0.012035
0.012001	0.012102	0.012035	0.011934	0.012038	0.012136	0.012143
0.012136	0.012035	0.011934	0.011870	0.012001	0.011934	0.011867
0.011842	0.012136	0.011934	0.012136	0.011900	0.012001	0.011967
0.012068	0.011967	0.012001	0.011967	0.012001	0.011867	0.012001
0.012035	0.011967	0.012035	0.011967	0.012001	0.012035	0.012270
0.012102	0.012034	0.012102	0.012106	0.012102	0.012001	0.011967
0.011871	0.011990	0.011929	0.011884	0.012113	0.011811	0.011729
0.012080	0.011809	0.012169	0.012371	0.012017	0.012236	0.012203
0.012135	0.012303	0.012056	0.011926	0.011815	0.011934	0.011974
0.012068	0.011904	0.012245	0.012110	0.012135	0.012169	0.012247
0.012042	0.012045	0.012042	0.012075	0.012238	0.011900	0.011835
0.012068	0.012001	0.011866	0.011900	0.011967	0.011967	0.012034
0.011967	0.012034	0.011933	0.011967	0.011967	0.011967	0.012034
0.011967	0.012135	0.012034	0.012068	0.011866	0.011967	0.012001
0.012001	0.011967	0.012034	0.012001	0.011933	0.011933	0.011933
0.012135	0.012034	0.012101	0.011900	0.012000	0.012169	0.011933
0.011933	0.012000	0.011967	0.012135	0.011933	0.011799	0.011933
0.011866	0.011967	0.011967	0.011900	0.011900	0.012068	0.012001
0.011900	0.012000	0.011765	0.011799	0.011933	0.011800	0.011833
0.011800	0.011933	0.011867	0.011765	0.011933	0.011765	0.011933
0.011882	0.011933	0.011866	0.011967	0.012034	0.011967	0.012068
0.011933	0.012270	0.011866	0.012068	0.011866	0.011866	0.011866
0.012101	0.012001	0.011933	0.012068	0.011900	0.012034	0.011866
0.011933	0.011933	0.011967	0.012068	0.011967	0.012068	0.012135
0.012135	0.012101	0.012001	0.012102	0.012068	0.011933	0.011900
0.011933	0.011933	0.011933	0.011866	0.011900	0.012068	0.011933
0.011900	0.011967	0.012101	0.012068	0.012034	0.011967	0.011967
0.011967	0.011967	0.012068	0.011933	0.012068	0.012169	0.012135
0.011967	0.012102	0.011751	0.018314	0.011649	0.011855	0.011828

0.011787	0.011835	0.011899	0.011641	0.011885	0.011855	0.011972
0.011440	0.011610	0.011685	0.011772	0.011685	0.011752	0.011749
0.011624	0.011619	0.011617	0.011523	0.012102	0.012135	0.012143
0.011747	0.012102	0.012102	0.012001	0.012034	0.012035	0.012002
0.012034	0.011967	0.012001	0.012035	0.012001	0.011934	0.011967
0.011967	0.012035	0.011934	0.012035	0.012068	0.012102	0.012001
0.011967	0.011934	0.012068	0.012068	0.012068	0.012103	0.012136
0.011900	0.011900	0.011934	0.012035	0.011900	0.012136	0.012136
0.012169	0.012169	0.012102	0.012203	0.012102	0.012206	0.012034
0.012035	0.012102	0.012136	0.011900	0.012035	0.012001	0.012034
0.012103	0.012102	0.012136	0.012102	0.012004	0.012203	0.012169
0.012043	0.011934	0.012035	0.012136	0.012035	0.012102	0.012202
0.012371	0.012017	0.012236	0.012203	0.012135	0.012303	0.011766
0.012105	0.011935	0.011967	0.011967	0.012035	0.011934	0.012035
0.012068	0.012102	0.012001	0.011967	0.011934	0.012068	0.012068
0.012068	0.012103	0.012169	0.012102	0.012474	0.011953	0.011975
0.011939	0.012101	0.011974	0.012100	0.012006	0.012061	0.011888
0.012469	0.011787	0.012123	0.011856	0.012094	0.012149	0.012047
0.012421	0.011852	0.012279	0.012337	0.012202	0.011866	0.012244
0.011900	0.012068	0.012202	0.012169	0.012176	0.011900	0.011599
0.012001	0.011933	0.011967	0.012001	0.011900	0.011967	0.011866
0.011933	0.011967	0.011933	0.011900	0.012001	0.011933	0.011967
0.011967	0.011900	0.011967	0.012001	0.011900	0.012101	0.012135
0.012034	0.012034	0.012034	0.012034	0.011967	0.012102	0.012000
0.011582	0.011900	0.011732	0.011705	0.012068	0.012068	0.012068
0.012068	0.012001	0.011967	0.012001	0.012101	0.012101	0.012034
0.012034	0.012101	0.012068	0.012068	0.012270	0.012270	0.012135
0.011967	0.012034	0.012068	0.012203	0.012035	0.012068	0.012034
0.012068	0.011933	0.011933	0.012236	0.012236	0.012202	0.012001
0.012102	0.012169	0.012034	0.012102	0.012068	0.011933	0.012001
0.012034	0.012001	0.011933	0.012169	0.012001	0.012101	0.012135
0.011933	0.012114	0.012135	0.011711	0.011568	0.011842	0.011473
0.011610	0.011605	0.011728	0.011820	0.011793	0.011824	0.011698
0.011672	0.011653	0.011728	0.011523	0.011653	0.011596	0.011953
0.011927	0.011847	0.011800	0.011800	0.011971	0.011900	0.011900
0.011900	0.011968	0.012143	0.011874	0.012214	0.012227	0.012034
0.012001	0.012068	0.012001	0.012035	0.011900	0.011866	0.011934
0.012001	0.012001	0.011967	0.011933	0.011967	0.011934	0.012035
0.011794	0.011795	0.011780	0.011814	0.011950	0.011915	0.011914
0.011912	0.011867	0.011651	0.011672	0.011772	0.011942	0.011943
0.011767	0.011806	0.012071	0.012041	0.011969	0.012003	0.012135
0.012068	0.012135	0.012135	0.012034	0.012338	0.012202	0.012270
0.012304	0.012129	0.011948	0.011892	0.011799	0.012135	0.012135
0.012171	0.012169	0.012202	0.012202	0.012201	0.012337	0.011719
0.011703	0.011718	0.011741	0.011706	0.011812	0.011657	0.011706
0.011746	0.011630	0.011680	0.011697	0.011800	0.011848	0.011967
0.011934	0.011866	0.012001	0.011967	0.011833	0.011799	0.011732
0.011833	0.011900	0.011967	0.011967	0.012001	0.012001	0.012007
0.012075	0.012001	0.012001	0.011971	0.011900	0.011900	0.011900
0.011968	0.012143	0.011874	0.012214	0.012227	0.012034	0.012203
0.012135	0.012135	0.011974	0.011896	0.012069	0.012117	0.011656
0.012094	0.012063	0.011907	0.011978	0.011934	0.012136	0.012001
0.012102	0.012035	0.011934	0.012038	0.012136	0.012143	0.012035
0.011934	0.011870	0.012001	0.011934	0.011867	0.011842	0.012136
0.012034	0.011967	0.012001	0.012035	0.012001	0.011934	0.011732
0.012001	0.012034	0.012034	0.011967	0.012034	0.012270	0.011866
0.011967	0.012102	0.011967	0.012001	0.011967	0.012068	0.011746
0.011566	0.011613	0.011512	0.011664	0.011753	0.011671	0.011604
0.011480	0.011597	0.011698	0.011603	0.011498	0.011602	0.011935
0.011632	0.011703	0.011832	0.011709	0.011463	0.012404	0.011498

0.012103	0.011869	0.012071	0.012511	0.012068	0.011665	0.011799
0.012373	0.012140	0.011698	0.011665	0.011531	0.011531	0.011771
0.011597	0.011569	0.011664	0.011430	0.012103	0.012270	0.012137
0.012070	0.012204	0.012173	0.011974	0.012543	0.012551	0.012610
0.012577	0.012610	0.012563	0.012577	0.012613	0.012068	0.012170
0.012171	0.012843	0.012214	0.012405	0.012879	0.011740	0.011632
0.011799	0.011799	0.012137	0.012048	0.012187	0.012135	0.011633
S004	0.012101	0.012101	0.011429	0.000000	0.000000	0.011262
0.011807	0.011739	0.011396	0.011698	0.011765	0.011733	0.011698
0.011664	0.011900	0.011631	0.011731	0.011799	0.011765	0.011731
0.011698	0.011732	0.011496	0.011665	0.011699	0.011732	0.011773
0.011698	0.011732	0.011631	0.011530	0.011799	0.011698	0.011799
0.011765	0.011866	0.011765	0.011765	0.011799	0.011765	0.011933
0.011631	0.011765	0.011799	0.011732	0.011765	0.011866	0.011799
0.011765	0.011933	0.011833	0.011832	0.011765	0.012034	0.011765
0.011731	0.011765	0.011866	0.012034	0.011866	0.011765	0.011866
0.011799	0.011866	0.011765	0.012000	0.011967	0.011765	0.011832
0.011732	0.011832	0.011799	0.011866	0.011799	0.011799	0.011799
0.011698	0.011732	0.011698	0.011732	0.011765	0.011801	0.011866
0.011799	0.011765	0.011832	0.011731	0.011799	0.011698	0.011765
0.011933	0.011866	0.011900	0.011933	0.011765	0.012000	0.011765
0.011950	0.011845	0.011966	0.011646	0.011620	0.011760	0.011454
0.012085	0.011620	0.011678	0.012000	0.012000	0.011732	0.011832
0.011832	0.011799	0.011866	0.011765	0.011832	0.011799	0.011799
0.011765	0.011799	0.012068	0.011933	0.011832	0.011799	0.011732
0.011698	0.011631	0.011765	0.011732	0.011597	0.011564	0.011497
0.011597	0.011665	0.011732	0.011732	0.011766	0.011765	0.011770
0.011838	0.011765	0.011765	0.011636	0.011675	0.011621	0.011766
0.011738	0.011679	0.011724	0.011716	0.011672	0.011677	0.011736
0.011708	0.011750	0.011784	0.011699	0.011683	0.011834	0.011733
0.011800	0.011802	0.011800	0.011899	0.011832	0.012202	0.011934
0.011765	0.011833	0.011765	0.011799	0.011665	0.011631	0.011698
0.011765	0.011765	0.011732	0.011698	0.011731	0.011698	0.011799
0.011765	0.011866	0.011799	0.011698	0.011802	0.011900	0.011905
0.011900	0.011799	0.011698	0.011633	0.011765	0.011698	0.011631
0.011604	0.011900	0.011698	0.011900	0.011664	0.011766	0.011732
0.011833	0.011732	0.011765	0.011732	0.011765	0.011631	0.011765
0.011799	0.011732	0.011799	0.011732	0.011765	0.011799	0.012035
0.011866	0.011799	0.011867	0.011869	0.011867	0.011765	0.011732
0.011702	0.011950	0.011838	0.011643	0.011875	0.011640	0.011690
0.011841	0.011774	0.011933	0.012135	0.011846	0.012000	0.011967
0.011899	0.012068	0.011842	0.011678	0.011731	0.011799	0.011838
0.011967	0.011870	0.012007	0.012008	0.012001	0.012034	0.012111
0.011906	0.012011	0.012008	0.011940	0.012204	0.011664	0.011802
0.011832	0.011765	0.011631	0.011664	0.011732	0.011732	0.011799
0.011732	0.011799	0.011698	0.011732	0.011732	0.011731	0.011799
0.011732	0.011900	0.011799	0.011833	0.011631	0.011732	0.011765
0.011765	0.011731	0.011799	0.011765	0.011698	0.011698	0.011698
0.011900	0.011799	0.011866	0.011664	0.011765	0.011933	0.011698
0.011698	0.011765	0.011731	0.011900	0.011698	0.011564	0.011698
0.011631	0.011732	0.011731	0.011664	0.011664	0.011832	0.011765
0.011664	0.011765	0.011631	0.011664	0.011799	0.011665	0.011698
0.011665	0.011799	0.011732	0.011631	0.011698	0.011631	0.011799
0.011813	0.011698	0.011732	0.011833	0.011799	0.011732	0.011832
0.011698	0.012034	0.011631	0.011832	0.011631	0.011631	0.011631
0.011866	0.011766	0.011765	0.011832	0.011664	0.011799	0.011631
0.011698	0.011698	0.011731	0.011832	0.011731	0.011832	0.011900
0.011900	0.011866	0.011765	0.011866	0.011832	0.011698	0.011664
0.011698	0.011698	0.011765	0.011631	0.011664	0.011832	0.011698
0.011664	0.011732	0.011866	0.011832	0.011799	0.011731	0.011732

0.011799	0.011732	0.011832	0.011698	0.011832	0.011933	0.011967
0.011732	0.011866	0.011716	0.018239	0.011518	0.011917	0.011793
0.011753	0.011799	0.011794	0.011524	0.011885	0.011820	0.011850
0.011392	0.011549	0.011646	0.011730	0.011646	0.011751	0.011749
0.011577	0.011544	0.011542	0.011391	0.011866	0.011900	0.011906
0.011713	0.011866	0.011866	0.011765	0.011799	0.011799	0.011766
0.011799	0.011732	0.011765	0.011799	0.011766	0.011698	0.011732
0.011732	0.011799	0.011698	0.011799	0.011833	0.011866	0.011765
0.011732	0.011698	0.011833	0.011833	0.011833	0.011867	0.011900
0.011665	0.011665	0.011698	0.011799	0.011665	0.011900	0.011900
0.011933	0.011933	0.011866	0.011967	0.011867	0.011970	0.011799
0.011799	0.011866	0.011900	0.011665	0.011799	0.011765	0.011799
0.011867	0.011866	0.011900	0.011867	0.011767	0.011967	0.011934
0.011805	0.011698	0.011799	0.011900	0.011799	0.011866	0.011967
0.012135	0.011846	0.012000	0.011967	0.011899	0.012068	0.011699
0.011869	0.011766	0.011732	0.011732	0.011799	0.011698	0.011799
0.011833	0.011866	0.011765	0.011732	0.011698	0.011833	0.011833
0.011833	0.011867	0.011933	0.011866	0.012440	0.011918	0.011764
0.011904	0.011858	0.011904	0.011822	0.011970	0.011888	0.011853
0.012365	0.011753	0.011882	0.011821	0.012059	0.011979	0.012013
0.012386	0.011776	0.012041	0.012101	0.011967	0.011631	0.012007
0.011664	0.011832	0.011967	0.011933	0.011939	0.011664	0.011363
0.011765	0.011698	0.011732	0.011765	0.011664	0.011732	0.011631
0.011698	0.011732	0.011698	0.011664	0.011765	0.011698	0.011732
0.011731	0.011664	0.011732	0.011765	0.011664	0.011866	0.011900
0.011799	0.011799	0.011799	0.011799	0.011731	0.011866	0.011765
0.011373	0.011732	0.011699	0.011671	0.011832	0.011832	0.011832
0.011832	0.011765	0.011731	0.011765	0.011866	0.011866	0.011799
0.011799	0.011933	0.011832	0.011832	0.012034	0.012034	0.011899
0.011731	0.011799	0.011832	0.011967	0.011799	0.011832	0.011799
0.011832	0.011698	0.011698	0.012001	0.012001	0.011967	0.011765
0.011866	0.011933	0.011799	0.011866	0.011832	0.011698	0.011765
0.011799	0.011765	0.011698	0.011933	0.011765	0.011866	0.011900
0.011799	0.011875	0.011900	0.011629	0.011455	0.011808	0.011421
0.011719	0.011527	0.011692	0.011730	0.011695	0.011722	0.011591
0.011570	0.011587	0.011617	0.011456	0.011545	0.011663	0.011912
0.011889	0.011769	0.011765	0.011730	0.011734	0.011665	0.011665
0.011665	0.011733	0.011906	0.011636	0.011976	0.011979	0.011799
0.011765	0.011833	0.011765	0.011799	0.011665	0.011631	0.011698
0.011765	0.011765	0.011732	0.011698	0.011731	0.011698	0.011799
0.011759	0.011760	0.011745	0.011780	0.011916	0.011880	0.011880
0.011878	0.011834	0.011614	0.011636	0.011738	0.011908	0.011909
0.011733	0.011772	0.012037	0.012007	0.011935	0.011969	0.011900
0.011832	0.011900	0.011899	0.011799	0.012102	0.011967	0.012034
0.012068	0.011879	0.011777	0.011857	0.011732	0.011900	0.011900
0.011935	0.011967	0.011967	0.011967	0.012161	0.012101	0.011685
0.011601	0.011580	0.011707	0.011672	0.011641	0.011620	0.011672
0.011712	0.011543	0.011584	0.011622	0.011766	0.011813	0.011732
0.011698	0.011631	0.011765	0.011732	0.011597	0.011564	0.011497
0.011597	0.011665	0.011732	0.011732	0.011766	0.011765	0.011770
0.011838	0.011765	0.011765	0.011734	0.011665	0.011665	0.011665
0.011733	0.011906	0.011636	0.011976	0.011979	0.011799	0.011967
0.011900	0.011900	0.011737	0.011861	0.011825	0.011878	0.011621
0.011921	0.011972	0.011823	0.011808	0.011698	0.011900	0.011765
0.011866	0.011799	0.011698	0.011802	0.011900	0.011905	0.011799
0.011698	0.011633	0.011765	0.011698	0.011631	0.011604	0.011900
0.011799	0.011732	0.011765	0.011799	0.011766	0.011698	0.011631
0.011765	0.011799	0.011799	0.011731	0.011799	0.012034	0.011631
0.011732	0.011866	0.011731	0.011765	0.011731	0.011832	0.011660
0.011454	0.011528	0.011461	0.011584	0.011671	0.011635	0.011520

0.011428	0.011362	0.011463	0.011569	0.011430	0.011534	0.011699
0.011598	0.011669	0.011597	0.011675	0.011228	0.012169	0.011464
0.012001	0.011835	0.011970	0.012443	0.011832	0.011564	0.011564
0.012339	0.011903	0.011530	0.011430	0.011497	0.011497	0.011534
0.011362	0.011331	0.011429	0.011396	0.012036	0.012102	0.012103
0.012002	0.012103	0.012207	0.011940	0.012475	0.012483	0.012542
0.012543	0.012577	0.012494	0.012543	0.012545	0.011832	0.012069
0.012137	0.012606	0.012146	0.012169	0.012643	0.011706	0.011598
0.011564	0.011564	0.012035	0.011965	0.012151	0.011900	0.011566
S005 0.012101	0.012101	0.011429	0.000000	0.000000	0.011262	
0.011807	0.011739	0.011396	0.011698	0.011765	0.011733	0.011698
0.011664	0.011900	0.011631	0.011731	0.011799	0.011765	0.011731
0.011698	0.011732	0.011496	0.011665	0.011699	0.011732	0.011773
0.011698	0.011732	0.011631	0.011530	0.011799	0.011698	0.011799
0.011765	0.011866	0.011765	0.011765	0.011799	0.011765	0.011933
0.011631	0.011765	0.011799	0.011732	0.011765	0.011866	0.011799
0.011765	0.011933	0.011833	0.011832	0.011765	0.012034	0.011765
0.011731	0.011765	0.011866	0.012034	0.011866	0.011765	0.011866
0.011799	0.011866	0.011765	0.012000	0.011967	0.011765	0.011832
0.011732	0.011832	0.011799	0.011866	0.011799	0.011799	0.011799
0.011698	0.011732	0.011698	0.011732	0.011765	0.011801	0.011866
0.011799	0.011765	0.011832	0.011731	0.011799	0.011698	0.011765
0.011933	0.011866	0.011900	0.011933	0.011765	0.012000	0.011765
0.011950	0.011845	0.011966	0.011646	0.011620	0.011760	0.011454
0.012085	0.011620	0.011678	0.012000	0.012000	0.011732	0.011832
0.011832	0.011799	0.011866	0.011765	0.011832	0.011799	0.011799
0.011765	0.011799	0.012068	0.011933	0.011832	0.011799	0.011732
0.011698	0.011631	0.011765	0.011732	0.011597	0.011564	0.011497
0.011597	0.011665	0.011732	0.011732	0.011766	0.011765	0.011770
0.011838	0.011765	0.011765	0.011636	0.011675	0.011621	0.011766
0.011738	0.011679	0.011724	0.011716	0.011672	0.011677	0.011736
0.011708	0.011750	0.011784	0.011699	0.011683	0.011834	0.011733
0.011800	0.011802	0.011800	0.011899	0.011832	0.012202	0.011934
0.011765	0.011833	0.011765	0.011799	0.011665	0.011631	0.011698
0.011765	0.011765	0.011732	0.011698	0.011731	0.011698	0.011799
0.011765	0.011866	0.011799	0.011698	0.011802	0.011900	0.011905
0.011900	0.011799	0.011698	0.011633	0.011765	0.011698	0.011631
0.011604	0.011900	0.011698	0.011900	0.011664	0.011766	0.011732
0.011833	0.011732	0.011765	0.011732	0.011765	0.011631	0.011765
0.011799	0.011732	0.011799	0.011732	0.011765	0.011799	0.012035
0.011866	0.011799	0.011867	0.011869	0.011867	0.011765	0.011732
0.011702	0.011950	0.011838	0.011643	0.011875	0.011640	0.011690
0.011841	0.011774	0.011933	0.012135	0.011846	0.012000	0.011967
0.011899	0.012068	0.011842	0.011678	0.011731	0.011799	0.011838
0.011967	0.011870	0.012007	0.012008	0.012001	0.012034	0.012111
0.011906	0.012011	0.012008	0.011940	0.012204	0.011664	0.011802
0.011832	0.011765	0.011631	0.011664	0.011732	0.011732	0.011799
0.011732	0.011799	0.011698	0.011732	0.011732	0.011731	0.011799
0.011732	0.011900	0.011799	0.011833	0.011631	0.011732	0.011765
0.011765	0.011731	0.011799	0.011765	0.011698	0.011698	0.011698
0.011900	0.011799	0.011866	0.011664	0.011765	0.011933	0.011698
0.011698	0.011765	0.011731	0.011900	0.011698	0.011564	0.011698
0.011631	0.011732	0.011731	0.011664	0.011664	0.011832	0.011765
0.011664	0.011765	0.011631	0.011664	0.011799	0.011665	0.011698
0.011665	0.011799	0.011732	0.011631	0.011698	0.011631	0.011799
0.011813	0.011698	0.011732	0.011833	0.011799	0.011732	0.011832
0.011698	0.012034	0.011631	0.011832	0.011631	0.011631	0.011631
0.011866	0.011766	0.011765	0.011832	0.011664	0.011799	0.011631
0.011698	0.011698	0.011731	0.011832	0.011731	0.011832	0.011900
0.011900	0.011866	0.011765	0.011866	0.011832	0.011698	0.011664

0.011698	0.011698	0.011765	0.011631	0.011664	0.011832	0.011698
0.011664	0.011732	0.011866	0.011832	0.011799	0.011731	0.011732
0.011799	0.011732	0.011832	0.011698	0.011832	0.011933	0.011967
0.011732	0.011866	0.011716	0.018239	0.011518	0.011917	0.011793
0.011753	0.011799	0.011794	0.011524	0.011885	0.011820	0.011850
0.011392	0.011549	0.011646	0.011730	0.011646	0.011751	0.011749
0.011577	0.011544	0.011542	0.011391	0.011866	0.011900	0.011906
0.011713	0.011866	0.011866	0.011765	0.011799	0.011799	0.011766
0.011799	0.011732	0.011765	0.011799	0.011766	0.011698	0.011732
0.011732	0.011799	0.011698	0.011799	0.011833	0.011866	0.011765
0.011732	0.011698	0.011833	0.011833	0.011833	0.011867	0.011900
0.011665	0.011665	0.011698	0.011799	0.011665	0.011900	0.011900
0.011933	0.011933	0.011866	0.011967	0.011867	0.011970	0.011799
0.011799	0.011866	0.011900	0.011665	0.011799	0.011765	0.011799
0.011867	0.011866	0.011900	0.011867	0.011767	0.011967	0.011934
0.011805	0.011698	0.011799	0.011900	0.011799	0.011866	0.011967
0.012135	0.011846	0.012000	0.011967	0.011899	0.012068	0.011699
0.011869	0.011766	0.011732	0.011732	0.011799	0.011698	0.011799
0.011833	0.011866	0.011765	0.011732	0.011698	0.011833	0.011833
0.011833	0.011867	0.011933	0.011866	0.012440	0.011918	0.011764
0.011904	0.011858	0.011904	0.011822	0.011970	0.011888	0.011853
0.012365	0.011753	0.011882	0.011821	0.012059	0.011979	0.012013
0.012386	0.011776	0.012041	0.012101	0.011967	0.011631	0.012007
0.011664	0.011832	0.011967	0.011933	0.011939	0.011664	0.011363
0.011765	0.011698	0.011732	0.011765	0.011664	0.011732	0.011631
0.011698	0.011732	0.011698	0.011664	0.011765	0.011698	0.011732
0.011731	0.011664	0.011732	0.011765	0.011664	0.011866	0.011900
0.011799	0.011799	0.011799	0.011799	0.011731	0.011866	0.011765
0.011373	0.011732	0.011699	0.011671	0.011832	0.011832	0.011832
0.011832	0.011765	0.011731	0.011765	0.011866	0.011866	0.011799
0.011799	0.011933	0.011832	0.011832	0.012034	0.012034	0.011899
0.011731	0.011799	0.011832	0.011967	0.011799	0.011832	0.011799
0.011832	0.011698	0.011698	0.012001	0.012001	0.011967	0.011765
0.011866	0.011933	0.011799	0.011866	0.011832	0.011698	0.011765
0.011799	0.011765	0.011698	0.011933	0.011765	0.011866	0.011900
0.011799	0.011875	0.011900	0.011629	0.011455	0.011808	0.011421
0.011719	0.011527	0.011692	0.011730	0.011695	0.011722	0.011591
0.011570	0.011587	0.011617	0.011456	0.011545	0.011663	0.011912
0.011889	0.011769	0.011765	0.011730	0.011734	0.011665	0.011665
0.011665	0.011733	0.011906	0.011636	0.011976	0.011979	0.011799
0.011765	0.011833	0.011765	0.011799	0.011665	0.011631	0.011698
0.011765	0.011765	0.011732	0.011698	0.011731	0.011698	0.011799
0.011759	0.011760	0.011745	0.011780	0.011916	0.011880	0.011880
0.011878	0.011834	0.011614	0.011636	0.011738	0.011908	0.011909
0.011733	0.011772	0.012037	0.012007	0.011935	0.011969	0.011900
0.011832	0.011900	0.011899	0.011799	0.012102	0.011967	0.012034
0.012068	0.011879	0.011777	0.011857	0.011732	0.011900	0.011900
0.011935	0.011967	0.011967	0.011967	0.012161	0.012101	0.011685
0.011601	0.011580	0.011707	0.011672	0.011641	0.011620	0.011672
0.011712	0.011543	0.011584	0.011622	0.011766	0.011813	0.011732
0.011698	0.011631	0.011765	0.011732	0.011597	0.011564	0.011497
0.011597	0.011665	0.011732	0.011732	0.011766	0.011765	0.011770
0.011838	0.011765	0.011765	0.011734	0.011665	0.011665	0.011665
0.011733	0.011906	0.011636	0.011976	0.011979	0.011799	0.011967
0.011900	0.011900	0.011737	0.011861	0.011825	0.011878	0.011621
0.011921	0.011972	0.011823	0.011808	0.011698	0.011900	0.011765
0.011866	0.011799	0.011698	0.011802	0.011900	0.011905	0.011799
0.011698	0.011633	0.011765	0.011698	0.011631	0.011604	0.011900
0.011799	0.011732	0.011765	0.011799	0.011766	0.011698	0.011631
0.011765	0.011799	0.011799	0.011731	0.011799	0.012034	0.011631

0.011732	0.011866	0.011731	0.011765	0.011731	0.011832	0.011660
0.011454	0.011528	0.011461	0.011584	0.011671	0.011635	0.011520
0.011428	0.011362	0.011463	0.011569	0.011430	0.011534	0.011699
0.011598	0.011669	0.011597	0.011675	0.011228	0.012169	0.011464
0.012001	0.011835	0.011970	0.012443	0.011832	0.011564	0.011564
0.012339	0.011903	0.011530	0.011430	0.011497	0.011497	0.011534
0.011362	0.011331	0.011429	0.011396	0.012036	0.012102	0.012103
0.012002	0.012103	0.012207	0.011940	0.012475	0.012483	0.012542
0.012543	0.012577	0.012494	0.012543	0.012545	0.011832	0.012069
0.012137	0.012606	0.012146	0.012169	0.012643	0.011706	0.011598
0.011564	0.011564	0.012035	0.011965	0.012151	0.011900	0.011566
S006	0.012169	0.012169	0.011496	0.011262	0.011262	0.000000
0.011875	0.011807	0.011463	0.011765	0.011832	0.011801	0.011765
0.011732	0.011967	0.011698	0.011799	0.011866	0.011832	0.011799
0.011765	0.011799	0.011564	0.011732	0.011766	0.011799	0.011841
0.011765	0.011799	0.011698	0.011597	0.011866	0.011833	0.011866
0.011832	0.011933	0.011832	0.011832	0.011866	0.011832	0.012000
0.011698	0.011832	0.011866	0.011799	0.011832	0.011933	0.011866
0.011832	0.012001	0.011900	0.011900	0.011832	0.012101	0.011832
0.011799	0.011833	0.011933	0.012101	0.011933	0.011832	0.011933
0.011866	0.011933	0.011832	0.012068	0.012034	0.011832	0.011900
0.011799	0.011900	0.011866	0.011933	0.011866	0.011866	0.011866
0.011765	0.011799	0.011765	0.011799	0.011832	0.011868	0.011933
0.011866	0.011832	0.011900	0.011799	0.011866	0.011765	0.011832
0.012000	0.011933	0.011967	0.012000	0.011832	0.012068	0.011832
0.011988	0.011882	0.012004	0.011685	0.011695	0.011797	0.011533
0.012158	0.011671	0.011761	0.012067	0.012068	0.011799	0.011900
0.011900	0.011866	0.011933	0.011832	0.011900	0.011866	0.011866
0.011832	0.011866	0.012135	0.012000	0.011900	0.011866	0.011799
0.011765	0.011698	0.011833	0.011799	0.011665	0.011631	0.011564
0.011665	0.011732	0.011799	0.011799	0.011833	0.011833	0.011837
0.011906	0.011833	0.011833	0.011672	0.011747	0.011691	0.011834
0.011806	0.011748	0.011761	0.011750	0.011740	0.011746	0.011804
0.011744	0.011819	0.011853	0.011766	0.011717	0.011902	0.011800
0.011868	0.011869	0.011867	0.011967	0.011900	0.012269	0.012001
0.011832	0.011900	0.011832	0.011866	0.011732	0.011698	0.011766
0.011832	0.011832	0.011799	0.011765	0.011799	0.011765	0.011866
0.011833	0.011934	0.011866	0.011765	0.011869	0.011967	0.011973
0.011967	0.011867	0.011765	0.011701	0.011833	0.011766	0.011699
0.011672	0.011967	0.011766	0.011967	0.011732	0.011833	0.011799
0.011900	0.011799	0.011833	0.011799	0.011833	0.011698	0.011833
0.011866	0.011799	0.011866	0.011799	0.011833	0.011866	0.012102
0.011934	0.011866	0.011934	0.011937	0.011934	0.011833	0.011799
0.011770	0.012031	0.011838	0.011712	0.011943	0.011708	0.011729
0.011909	0.011843	0.012000	0.012202	0.011914	0.012068	0.012034
0.011967	0.012135	0.011913	0.011719	0.011731	0.011866	0.011906
0.012034	0.011937	0.012075	0.012076	0.012068	0.012102	0.012145
0.011974	0.012045	0.012076	0.012007	0.012272	0.011731	0.011869
0.011900	0.011832	0.011698	0.011731	0.011799	0.011799	0.011866
0.011799	0.011866	0.011765	0.011799	0.011799	0.011799	0.011866
0.011799	0.011967	0.011866	0.011900	0.011698	0.011799	0.011832
0.011832	0.011799	0.011866	0.011832	0.011765	0.011765	0.011765
0.011967	0.011866	0.011933	0.011732	0.011832	0.012001	0.011765
0.011765	0.011832	0.011799	0.011967	0.011765	0.011631	0.011765
0.011698	0.011799	0.011799	0.011732	0.011732	0.011900	0.011832
0.011732	0.011832	0.011698	0.011732	0.011866	0.011732	0.011765
0.011732	0.011866	0.011800	0.011698	0.011765	0.011698	0.011866
0.011882	0.011765	0.011799	0.011900	0.011866	0.011799	0.011900
0.011765	0.012101	0.011698	0.011900	0.011698	0.011698	0.011698
0.011933	0.011833	0.011832	0.011900	0.011732	0.011866	0.011698

0.011765	0.011765	0.011799	0.011900	0.011799	0.011900	0.011967
0.011967	0.011933	0.011832	0.011933	0.011900	0.011765	0.011732
0.011765	0.011765	0.011832	0.011698	0.011732	0.011900	0.011765
0.011732	0.011799	0.011933	0.011900	0.011799	0.011799	0.011799
0.011866	0.011799	0.011900	0.011765	0.011900	0.012000	0.012034
0.011799	0.011933	0.011751	0.018314	0.011650	0.011917	0.011828
0.011787	0.011835	0.011899	0.011699	0.011885	0.011855	0.011972
0.011487	0.011610	0.011685	0.011772	0.011685	0.011752	0.011749
0.011670	0.011619	0.011617	0.011457	0.011933	0.011967	0.011974
0.011747	0.011933	0.011933	0.011833	0.011866	0.011867	0.011833
0.011866	0.011799	0.011832	0.011866	0.011833	0.011765	0.011799
0.011799	0.011866	0.011765	0.011866	0.011900	0.011933	0.011832
0.011799	0.011766	0.011900	0.011900	0.011900	0.011935	0.011967
0.011732	0.011732	0.011765	0.011866	0.011732	0.011967	0.011967
0.012001	0.012000	0.011933	0.012034	0.011934	0.012037	0.011866
0.011866	0.011934	0.011967	0.011732	0.011866	0.011833	0.011866
0.011934	0.011934	0.011967	0.011934	0.011835	0.012034	0.012001
0.011873	0.011765	0.011866	0.011967	0.011866	0.011934	0.012034
0.012202	0.011914	0.012068	0.012034	0.011967	0.012135	0.011766
0.011936	0.011834	0.011799	0.011799	0.011866	0.011765	0.011866
0.011900	0.011933	0.011832	0.011799	0.011766	0.011900	0.011900
0.011900	0.011935	0.012000	0.011933	0.012507	0.011918	0.011799
0.011904	0.011892	0.011939	0.011857	0.012006	0.011923	0.011922
0.012400	0.011787	0.011917	0.011856	0.012093	0.012047	0.012047
0.012420	0.011814	0.012109	0.012169	0.012034	0.011698	0.012074
0.011731	0.011900	0.012034	0.012000	0.012007	0.011732	0.011431
0.011832	0.011765	0.011799	0.011832	0.011732	0.011799	0.011698
0.011765	0.011799	0.011765	0.011732	0.011832	0.011765	0.011799
0.011799	0.011731	0.011799	0.011832	0.011732	0.011933	0.011967
0.011866	0.011866	0.011866	0.011866	0.011799	0.011933	0.011832
0.011408	0.011799	0.011766	0.011705	0.011900	0.011900	0.011900
0.011900	0.011832	0.011799	0.011832	0.011933	0.011933	0.011866
0.011866	0.012000	0.011900	0.011900	0.012101	0.012101	0.011967
0.011799	0.011866	0.011900	0.012034	0.011867	0.011900	0.011866
0.011900	0.011765	0.011765	0.012068	0.012068	0.012034	0.011832
0.011933	0.012000	0.011866	0.011933	0.011899	0.011765	0.011832
0.011866	0.011832	0.011765	0.012000	0.011832	0.011933	0.011967
0.011866	0.011943	0.011967	0.011711	0.011512	0.011842	0.011473
0.011720	0.011605	0.011728	0.011820	0.011744	0.011824	0.011698
0.011621	0.011653	0.011728	0.011523	0.011599	0.011730	0.011953
0.011927	0.011847	0.011800	0.011800	0.011802	0.011732	0.011732
0.011732	0.011800	0.011973	0.011704	0.012044	0.012050	0.011866
0.011832	0.011900	0.011832	0.011866	0.011732	0.011698	0.011766
0.011832	0.011832	0.011799	0.011765	0.011799	0.011765	0.011866
0.011829	0.011830	0.011780	0.011848	0.011984	0.011949	0.011948
0.011946	0.011901	0.011614	0.011672	0.011806	0.011976	0.011977
0.011800	0.011839	0.012104	0.012075	0.012002	0.012037	0.011967
0.011900	0.011967	0.011967	0.011866	0.012169	0.012034	0.012102
0.012136	0.011951	0.011845	0.011927	0.011799	0.011967	0.011967
0.012002	0.012034	0.012034	0.012034	0.012240	0.012169	0.011719
0.011635	0.011614	0.011741	0.011706	0.011675	0.011620	0.011706
0.011746	0.011586	0.011632	0.011660	0.011834	0.011882	0.011799
0.011765	0.011698	0.011833	0.011799	0.011665	0.011631	0.011564
0.011665	0.011732	0.011799	0.011799	0.011833	0.011833	0.011837
0.011906	0.011833	0.011833	0.011802	0.011732	0.011732	0.011732
0.011800	0.011973	0.011704	0.012044	0.012050	0.011866	0.012034
0.011967	0.011967	0.011804	0.011931	0.011860	0.011946	0.011691
0.011990	0.012063	0.011907	0.011876	0.011766	0.011967	0.011833
0.011934	0.011866	0.011765	0.011869	0.011967	0.011973	0.011867
0.011765	0.011701	0.011833	0.011766	0.011699	0.011672	0.011967

0.011866	0.011799	0.011832	0.011866	0.011833	0.011765	0.011698
0.011832	0.011866	0.011866	0.011799	0.011866	0.012101	0.011698
0.011799	0.011933	0.011799	0.011832	0.011799	0.011900	0.011746
0.011510	0.011613	0.011512	0.011664	0.011753	0.011671	0.011604
0.011533	0.011429	0.011530	0.011603	0.011498	0.011601	0.011766
0.011666	0.011737	0.011664	0.011743	0.011295	0.012236	0.011532
0.012069	0.011903	0.012037	0.012511	0.011900	0.011631	0.011631
0.012407	0.011971	0.011597	0.011497	0.011565	0.011565	0.011601
0.011429	0.011399	0.011496	0.011464	0.012103	0.012169	0.012171
0.012070	0.012170	0.012275	0.012008	0.012543	0.012551	0.012610
0.012610	0.012644	0.012528	0.012611	0.012612	0.011900	0.012136
0.012204	0.012674	0.012214	0.012237	0.012710	0.011774	0.011665
0.011631	0.011631	0.012103	0.012007	0.012187	0.011967	0.011633
S007	0.012011	0.012011	0.012046	0.011807	0.011807	0.011875
0.000000	0.011196	0.011841	0.011604	0.011671	0.011639	0.011569
0.011535	0.011807	0.011535	0.011637	0.011671	0.011637	0.011637
0.011603	0.011637	0.011468	0.011604	0.011638	0.011637	0.011196
0.011604	0.011637	0.011535	0.011502	0.011705	0.011672	0.011705
0.011671	0.011773	0.011671	0.011671	0.011705	0.011671	0.011841
0.011535	0.011671	0.011705	0.011637	0.011671	0.011773	0.011705
0.011671	0.011841	0.011706	0.011332	0.011671	0.011909	0.011671
0.011637	0.011672	0.011807	0.011909	0.011434	0.011671	0.011773
0.011705	0.011773	0.011671	0.011909	0.011468	0.011671	0.011739
0.011637	0.011739	0.011705	0.011773	0.011637	0.011705	0.011705
0.011569	0.011637	0.011603	0.011637	0.011671	0.011673	0.011739
0.011705	0.011637	0.011739	0.011603	0.011502	0.011603	0.011671
0.011807	0.011773	0.011535	0.011841	0.011637	0.011875	0.011671
0.011806	0.011286	0.011819	0.011532	0.011589	0.011694	0.011417
0.012135	0.011419	0.011558	0.011909	0.011909	0.011637	0.011501
0.011705	0.011705	0.011773	0.011671	0.011773	0.011705	0.011637
0.011603	0.011671	0.011977	0.011841	0.011705	0.011706	0.011638
0.011604	0.011536	0.011672	0.011604	0.011570	0.011536	0.011468
0.011570	0.011638	0.011638	0.011638	0.011672	0.011672	0.011675
0.011744	0.011638	0.011672	0.011533	0.011609	0.011557	0.011638
0.011643	0.011582	0.011658	0.011619	0.011576	0.011580	0.011641
0.011534	0.011583	0.011688	0.011604	0.011550	0.011707	0.011639
0.011673	0.011708	0.011707	0.011773	0.011739	0.012045	0.011808
0.011671	0.011740	0.011671	0.011706	0.011570	0.011536	0.011604
0.011671	0.011671	0.011638	0.011603	0.011637	0.011604	0.011706
0.011672	0.011774	0.011706	0.011604	0.011708	0.011808	0.011813
0.011808	0.011672	0.011604	0.011538	0.011672	0.011604	0.011536
0.011541	0.011808	0.011604	0.011808	0.011570	0.011672	0.011638
0.011706	0.011604	0.011638	0.011638	0.011672	0.011536	0.011672
0.011706	0.011638	0.011672	0.011604	0.011638	0.011706	0.011944
0.011366	0.011298	0.011366	0.011811	0.011434	0.011672	0.011638
0.011607	0.011960	0.011663	0.011581	0.011369	0.011543	0.011738
0.011366	0.011611	0.011841	0.012045	0.011751	0.011909	0.011876
0.011807	0.011977	0.011852	0.011771	0.011741	0.011706	0.011779
0.011876	0.011743	0.011916	0.011985	0.011875	0.011944	0.011986
0.011779	0.011988	0.011848	0.011847	0.012116	0.011569	0.011776
0.011705	0.011671	0.011569	0.011569	0.011637	0.011637	0.011705
0.011637	0.011705	0.011603	0.011637	0.011637	0.011603	0.011705
0.011637	0.011400	0.011298	0.011332	0.011535	0.011637	0.011637
0.011671	0.011569	0.011705	0.011671	0.011603	0.011603	0.011603
0.011400	0.011705	0.011773	0.011569	0.011671	0.011841	0.011603
0.011603	0.011637	0.011637	0.011807	0.011603	0.011536	0.011671
0.011604	0.011637	0.011637	0.011569	0.011569	0.011739	0.011671
0.011569	0.011671	0.011536	0.011569	0.011705	0.011502	0.011604
0.011570	0.011705	0.011604	0.011536	0.011264	0.011536	0.011705
0.011788	0.011603	0.011230	0.011740	0.011705	0.011603	0.011739

0.011603	0.011909	0.011535	0.011536	0.011535	0.011535	0.011535
0.011773	0.011638	0.011671	0.011739	0.011569	0.011298	0.011535
0.011603	0.011603	0.011637	0.011739	0.011637	0.011739	0.011807
0.011807	0.011773	0.011671	0.011773	0.011739	0.011603	0.011569
0.011603	0.011603	0.011671	0.011535	0.011569	0.011739	0.011603
0.011569	0.011637	0.011773	0.011739	0.011705	0.011637	0.011638
0.011671	0.011637	0.011705	0.011603	0.011739	0.011841	0.011468
0.011637	0.011434	0.011654	0.018217	0.011325	0.011554	0.011272
0.011235	0.011274	0.011800	0.011587	0.011337	0.011305	0.011612
0.011634	0.011368	0.011492	0.011608	0.011535	0.011458	0.011623
0.011344	0.011513	0.011361	0.011598	0.011773	0.011808	0.011813
0.011686	0.011774	0.011773	0.011672	0.011671	0.011672	0.011672
0.011672	0.011603	0.011671	0.011706	0.011672	0.011604	0.011638
0.011638	0.011706	0.011604	0.011706	0.011740	0.011774	0.011671
0.011603	0.011604	0.011400	0.011400	0.011740	0.011775	0.011808
0.011570	0.011570	0.011604	0.011706	0.011570	0.011808	0.011808
0.011842	0.011841	0.011773	0.011875	0.011434	0.011878	0.011706
0.011706	0.011774	0.011808	0.011638	0.011706	0.011672	0.011706
0.011774	0.011774	0.011808	0.011774	0.011673	0.011876	0.011808
0.011711	0.011604	0.011706	0.011808	0.011706	0.011774	0.011875
0.012045	0.011751	0.011909	0.011876	0.011807	0.011977	0.011604
0.011776	0.011672	0.011638	0.011638	0.011706	0.011604	0.011706
0.011740	0.011774	0.011671	0.011603	0.011604	0.011400	0.011400
0.011740	0.011775	0.011841	0.011773	0.012251	0.011856	0.011772
0.011843	0.011797	0.011914	0.011796	0.011911	0.011828	0.011723
0.012207	0.011691	0.011823	0.011760	0.011966	0.011886	0.011990
0.012230	0.011824	0.011950	0.012011	0.011875	0.011604	0.011915
0.011569	0.011739	0.011875	0.011841	0.011847	0.011569	0.011537
0.011671	0.011603	0.011637	0.011671	0.011569	0.011637	0.011535
0.011603	0.011637	0.011603	0.011569	0.011671	0.011603	0.011637
0.011637	0.011569	0.011637	0.011671	0.011569	0.011773	0.011807
0.011705	0.011705	0.011705	0.011705	0.011637	0.011434	0.011671
0.011448	0.011638	0.011604	0.011609	0.011671	0.011671	0.011739
0.011739	0.011671	0.011637	0.011671	0.011773	0.011773	0.011671
0.011705	0.011841	0.011332	0.011739	0.011943	0.011943	0.011773
0.011637	0.011705	0.011739	0.011536	0.011672	0.011671	0.011705
0.011739	0.011603	0.011603	0.011910	0.011910	0.011875	0.011671
0.011739	0.011773	0.011705	0.011739	0.011705	0.011603	0.011230
0.011705	0.011637	0.011603	0.011841	0.011671	0.011773	0.011502
0.011705	0.011332	0.011400	0.011510	0.011402	0.011714	0.011424
0.011393	0.011765	0.011593	0.011600	0.011601	0.011368	0.011328
0.011523	0.011260	0.011453	0.011327	0.011332	0.011534	0.011754
0.011742	0.011654	0.011666	0.011702	0.011708	0.011570	0.011638
0.011638	0.011707	0.011813	0.011609	0.011952	0.011990	0.011705
0.011671	0.011740	0.011671	0.011706	0.011570	0.011536	0.011604
0.011671	0.011671	0.011638	0.011603	0.011637	0.011604	0.011706
0.011661	0.011662	0.011649	0.011649	0.011787	0.011752	0.011751
0.011750	0.011707	0.011546	0.011533	0.011643	0.011746	0.011816
0.011639	0.011677	0.011912	0.011915	0.011843	0.011877	0.011807
0.011739	0.011807	0.011807	0.011705	0.011671	0.011875	0.011909
0.011944	0.011348	0.011612	0.011761	0.011637	0.011807	0.011807
0.011775	0.011875	0.011875	0.011875	0.011897	0.012011	0.011657
0.011539	0.011551	0.011646	0.011610	0.011648	0.011589	0.011198
0.011685	0.011461	0.011690	0.011661	0.011673	0.011718	0.011638
0.011604	0.011536	0.011672	0.011604	0.011570	0.011536	0.011468
0.011570	0.011638	0.011638	0.011638	0.011672	0.011672	0.011675
0.011744	0.011638	0.011672	0.011708	0.011570	0.011638	0.011638
0.011707	0.011813	0.011609	0.011952	0.011990	0.011705	0.011875
0.011807	0.011807	0.011642	0.011835	0.011764	0.011749	0.011663
0.011827	0.012033	0.011834	0.011713	0.011604	0.011808	0.011672

0.011774	0.011706	0.011604	0.011708	0.011808	0.011813	0.011672
0.011604	0.011538	0.011672	0.011604	0.011536	0.011541	0.011808
0.011672	0.011603	0.011671	0.011706	0.011672	0.011604	0.011536
0.011671	0.011705	0.011671	0.011637	0.011705	0.011943	0.011535
0.011637	0.011434	0.011637	0.011671	0.011637	0.011332	0.011578
0.011854	0.011402	0.011414	0.011509	0.011552	0.011569	0.011355
0.011378	0.011536	0.011638	0.011747	0.011605	0.011745	0.011877
0.011775	0.011779	0.011773	0.011959	0.011705	0.012454	0.011742
0.012490	0.012186	0.012322	0.012665	0.011637	0.011638	0.011740
0.012286	0.012050	0.011706	0.011570	0.011673	0.011673	0.011539
0.011536	0.011541	0.011603	0.011571	0.011945	0.011978	0.012013
0.011877	0.011979	0.012118	0.011848	0.012287	0.012294	0.012355
0.012355	0.012355	0.012303	0.012321	0.012323	0.011739	0.011910
0.012047	0.012522	0.011918	0.011978	0.012456	0.011541	0.011434
0.011332	0.011400	0.011945	0.011936	0.012058	0.011739	0.011469
S008 0.011943	0.011943	0.011978	0.011739	0.011739	0.011807	
0.011196	0.000000	0.011773	0.011536	0.011603	0.011537	0.011502
0.011468	0.011739	0.011468	0.011569	0.011603	0.011569	0.011569
0.011535	0.011569	0.011400	0.011536	0.011570	0.011569	0.011128
0.011536	0.011569	0.011468	0.011434	0.011637	0.011604	0.011637
0.011603	0.011705	0.011603	0.011603	0.011637	0.011603	0.011773
0.011468	0.011603	0.011637	0.011569	0.011603	0.011705	0.011637
0.011603	0.011773	0.011638	0.011264	0.011603	0.011841	0.011603
0.011569	0.011604	0.011739	0.011841	0.011366	0.011603	0.011705
0.011637	0.011705	0.011603	0.011841	0.011400	0.011603	0.011671
0.011569	0.011671	0.011637	0.011705	0.011569	0.011637	0.011637
0.011501	0.011569	0.011535	0.011570	0.011603	0.011571	0.011671
0.011637	0.011569	0.011671	0.011535	0.011434	0.011535	0.011603
0.011739	0.011705	0.011468	0.011773	0.011569	0.011807	0.011603
0.011732	0.011210	0.011743	0.011453	0.011476	0.011619	0.011338
0.012024	0.011317	0.011475	0.011841	0.011841	0.011569	0.011434
0.011637	0.011637	0.011705	0.011603	0.011705	0.011637	0.011569
0.011535	0.011603	0.011909	0.011773	0.011637	0.011638	0.011570
0.011536	0.011468	0.011604	0.011536	0.011502	0.011468	0.011400
0.011502	0.011570	0.011570	0.011570	0.011604	0.011604	0.011607
0.011676	0.011570	0.011604	0.011424	0.011499	0.011450	0.011536
0.011540	0.011477	0.011546	0.011514	0.011473	0.011476	0.011539
0.011425	0.011549	0.011583	0.011502	0.011445	0.011605	0.011536
0.011571	0.011606	0.011604	0.011705	0.011671	0.011977	0.011740
0.011603	0.011672	0.011603	0.011638	0.011502	0.011468	0.011536
0.011603	0.011603	0.011570	0.011536	0.011569	0.011536	0.011638
0.011604	0.011706	0.011638	0.011536	0.011640	0.011740	0.011744
0.011740	0.011570	0.011536	0.011470	0.011604	0.011536	0.011468
0.011473	0.011740	0.011536	0.011740	0.011502	0.011604	0.011570
0.011638	0.011536	0.011570	0.011570	0.011604	0.011468	0.011604
0.011638	0.011570	0.011604	0.011536	0.011570	0.011638	0.011876
0.011298	0.011230	0.011298	0.011708	0.011366	0.011604	0.011570
0.011504	0.011837	0.011571	0.011477	0.011300	0.011440	0.011619
0.011298	0.011508	0.011773	0.011977	0.011682	0.011841	0.011807
0.011739	0.011909	0.011780	0.011645	0.011656	0.011638	0.011711
0.011808	0.011674	0.011847	0.011916	0.011807	0.011876	0.011917
0.011711	0.011919	0.011779	0.011779	0.012047	0.011501	0.011708
0.011637	0.011603	0.011501	0.011501	0.011569	0.011569	0.011637
0.011569	0.011637	0.011535	0.011569	0.011569	0.011535	0.011637
0.011569	0.011332	0.011230	0.011264	0.011468	0.011569	0.011569
0.011603	0.011502	0.011637	0.011603	0.011535	0.011535	0.011535
0.011332	0.011637	0.011705	0.011502	0.011603	0.011773	0.011535
0.011535	0.011569	0.011569	0.011739	0.011535	0.011468	0.011603
0.011536	0.011569	0.011569	0.011502	0.011501	0.011671	0.011603
0.011502	0.011603	0.011468	0.011502	0.011637	0.011434	0.011536

0.011502	0.011637	0.011536	0.011468	0.011196	0.011468	0.011637
0.011718	0.011536	0.011162	0.011672	0.011637	0.011535	0.011671
0.011535	0.011841	0.011468	0.011468	0.011468	0.011468	0.011468
0.011705	0.011570	0.011603	0.011671	0.011501	0.011230	0.011468
0.011535	0.011535	0.011569	0.011671	0.011569	0.011671	0.011739
0.011739	0.011705	0.011603	0.011705	0.011671	0.011535	0.011502
0.011535	0.011535	0.011603	0.011468	0.011502	0.011671	0.011535
0.011502	0.011569	0.011705	0.011671	0.011637	0.011569	0.011570
0.011603	0.011569	0.011637	0.011535	0.011671	0.011773	0.011468
0.011569	0.011366	0.011549	0.018141	0.011259	0.011554	0.011201
0.011165	0.011203	0.011694	0.011470	0.011337	0.011235	0.011612
0.011538	0.011368	0.011413	0.011524	0.011456	0.011458	0.011537
0.011249	0.011400	0.011248	0.011396	0.011705	0.011740	0.011745
0.011616	0.011706	0.011705	0.011604	0.011604	0.011604	0.011604
0.011604	0.011536	0.011604	0.011638	0.011604	0.011536	0.011570
0.011570	0.011638	0.011536	0.011638	0.011672	0.011706	0.011603
0.011536	0.011536	0.011332	0.011332	0.011672	0.011707	0.011740
0.011502	0.011502	0.011536	0.011638	0.011502	0.011740	0.011740
0.011774	0.011773	0.011705	0.011807	0.011366	0.011810	0.011638
0.011638	0.011706	0.011740	0.011570	0.011638	0.011604	0.011638
0.011672	0.011672	0.011740	0.011672	0.011571	0.011808	0.011740
0.011643	0.011536	0.011638	0.011740	0.011638	0.011706	0.011807
0.011977	0.011682	0.011841	0.011807	0.011739	0.011909	0.011536
0.011674	0.011570	0.011570	0.011570	0.011638	0.011536	0.011638
0.011672	0.011706	0.011603	0.011536	0.011536	0.011332	0.011332
0.011672	0.011707	0.011773	0.011705	0.012183	0.011783	0.011701
0.011772	0.011727	0.011843	0.011726	0.011840	0.011758	0.011653
0.012137	0.011621	0.011753	0.011690	0.011896	0.011817	0.011921
0.012161	0.011709	0.011881	0.011943	0.011807	0.011536	0.011847
0.011501	0.011671	0.011807	0.011773	0.011778	0.011502	0.011435
0.011603	0.011535	0.011569	0.011603	0.011502	0.011569	0.011468
0.011535	0.011569	0.011535	0.011502	0.011603	0.011535	0.011569
0.011569	0.011501	0.011569	0.011603	0.011501	0.011705	0.011739
0.011637	0.011637	0.011637	0.011637	0.011569	0.011366	0.011603
0.011377	0.011570	0.011536	0.011541	0.011603	0.011603	0.011671
0.011671	0.011603	0.011569	0.011603	0.011705	0.011705	0.011603
0.011637	0.011773	0.011264	0.011671	0.011875	0.011875	0.011705
0.011569	0.011637	0.011671	0.011468	0.011638	0.011603	0.011637
0.011671	0.011535	0.011535	0.011842	0.011841	0.011807	0.011603
0.011671	0.011705	0.011637	0.011671	0.011637	0.011535	0.011162
0.011637	0.011569	0.011535	0.011773	0.011603	0.011705	0.011434
0.011637	0.011264	0.011332	0.011428	0.011288	0.011610	0.011320
0.011393	0.011648	0.011487	0.011555	0.011503	0.011317	0.011274
0.011420	0.011193	0.011397	0.011260	0.011278	0.011466	0.011672
0.011666	0.011575	0.011595	0.011595	0.011640	0.011502	0.011570
0.011570	0.011604	0.011744	0.011540	0.011884	0.011919	0.011637
0.011603	0.011672	0.011603	0.011638	0.011502	0.011468	0.011536
0.011603	0.011603	0.011570	0.011536	0.011569	0.011536	0.011638
0.011555	0.011556	0.011545	0.011545	0.011683	0.011648	0.011647
0.011646	0.011604	0.011434	0.011424	0.011540	0.011643	0.011712
0.011536	0.011574	0.011810	0.011813	0.011741	0.011775	0.011739
0.011671	0.011739	0.011739	0.011637	0.011603	0.011807	0.011841
0.011876	0.011276	0.011543	0.011656	0.011570	0.011740	0.011739
0.011707	0.011807	0.011807	0.011807	0.011857	0.011943	0.011587
0.011471	0.011481	0.011577	0.011541	0.011579	0.011514	0.011129
0.011615	0.011327	0.011544	0.011548	0.011570	0.011614	0.011570
0.011536	0.011468	0.011604	0.011536	0.011502	0.011468	0.011400
0.011502	0.011570	0.011570	0.011570	0.011604	0.011604	0.011607
0.011676	0.011570	0.011604	0.011640	0.011502	0.011570	0.011570
0.011604	0.011744	0.011540	0.011884	0.011919	0.011637	0.011807

0.011739	0.011739	0.011539	0.011765	0.011658	0.011680	0.011557
0.011722	0.011940	0.011748	0.011645	0.011536	0.011740	0.011604
0.011706	0.011638	0.011536	0.011640	0.011740	0.011744	0.011570
0.011536	0.011470	0.011604	0.011536	0.011468	0.011473	0.011740
0.011604	0.011536	0.011604	0.011638	0.011604	0.011536	0.011468
0.011603	0.011637	0.011603	0.011569	0.011637	0.011875	0.011468
0.011569	0.011366	0.011569	0.011603	0.011569	0.011264	0.011492
0.011741	0.011317	0.011313	0.011428	0.011470	0.011460	0.011271
0.011272	0.011468	0.011570	0.011644	0.011503	0.011642	0.011775
0.011707	0.011710	0.011705	0.011856	0.011637	0.012386	0.011640
0.012421	0.012084	0.012254	0.012563	0.011569	0.011570	0.011638
0.012184	0.011948	0.011638	0.011502	0.011571	0.011571	0.011505
0.011468	0.011438	0.011536	0.011468	0.011843	0.011910	0.011911
0.011809	0.011877	0.012016	0.011745	0.012184	0.012191	0.012252
0.012253	0.012253	0.012200	0.012219	0.012220	0.011671	0.011842
0.011945	0.012454	0.011849	0.011910	0.012354	0.011438	0.011332
0.011264	0.011332	0.011876	0.011853	0.011951	0.011671	0.011367
S009 0.012169	0.012169	0.011631	0.011396	0.011396	0.011463	
0.011841	0.011773	0.000000	0.011765	0.011832	0.011733	0.011765
0.011731	0.011899	0.011698	0.011799	0.011866	0.011832	0.011799
0.011765	0.011799	0.011631	0.011732	0.011766	0.011799	0.011807
0.011765	0.011799	0.011698	0.011664	0.011866	0.011832	0.011866
0.011832	0.011933	0.011832	0.011832	0.011866	0.011832	0.012000
0.011698	0.011832	0.011866	0.011799	0.011832	0.011933	0.011866
0.011832	0.012000	0.011900	0.011900	0.011832	0.012101	0.011832
0.011799	0.011833	0.011933	0.012101	0.011933	0.011832	0.011933
0.011866	0.011933	0.011832	0.012067	0.012034	0.011765	0.011900
0.011799	0.011899	0.011866	0.011933	0.011866	0.011866	0.011866
0.011765	0.011799	0.011765	0.011799	0.011832	0.011801	0.011933
0.011866	0.011832	0.011899	0.011799	0.011866	0.011765	0.011832
0.012000	0.011933	0.011967	0.012000	0.011832	0.012068	0.011765
0.011913	0.011807	0.011928	0.011607	0.011657	0.011760	0.011533
0.012195	0.011620	0.011678	0.012067	0.012068	0.011799	0.011900
0.011899	0.011866	0.011933	0.011832	0.011900	0.011866	0.011866
0.011832	0.011866	0.012135	0.011933	0.011899	0.011866	0.011732
0.011698	0.011631	0.011765	0.011732	0.011665	0.011631	0.011564
0.011665	0.011732	0.011732	0.011732	0.011766	0.011765	0.011770
0.011838	0.011765	0.011765	0.011636	0.011675	0.011621	0.011766
0.011738	0.011679	0.011724	0.011716	0.011672	0.011677	0.011736
0.011708	0.011750	0.011784	0.011699	0.011683	0.011834	0.011733
0.011800	0.011802	0.011800	0.011967	0.011899	0.012269	0.011934
0.011832	0.011833	0.011799	0.011799	0.011665	0.011631	0.011698
0.011832	0.011832	0.011732	0.011765	0.011799	0.011698	0.011799
0.011765	0.011866	0.011799	0.011698	0.011734	0.011900	0.011905
0.011900	0.011799	0.011698	0.011633	0.011765	0.011698	0.011631
0.011604	0.011900	0.011698	0.011900	0.011732	0.011766	0.011732
0.011833	0.011732	0.011765	0.011732	0.011765	0.011631	0.011765
0.011799	0.011732	0.011799	0.011732	0.011765	0.011799	0.012035
0.011866	0.011799	0.011867	0.011869	0.011867	0.011765	0.011732
0.011702	0.011950	0.011838	0.011643	0.011807	0.011640	0.011729
0.011807	0.011774	0.012000	0.012202	0.011846	0.012068	0.012034
0.011967	0.012068	0.011949	0.011719	0.011857	0.011866	0.011906
0.012034	0.011937	0.012075	0.012076	0.012068	0.012102	0.012179
0.011974	0.012079	0.012076	0.012007	0.012272	0.011731	0.011869
0.011899	0.011832	0.011698	0.011731	0.011799	0.011731	0.011866
0.011799	0.011866	0.011765	0.011799	0.011799	0.011799	0.011866
0.011799	0.011967	0.011866	0.011900	0.011698	0.011799	0.011832
0.011832	0.011799	0.011866	0.011832	0.011765	0.011765	0.011765
0.011967	0.011866	0.011933	0.011731	0.011832	0.012000	0.011765
0.011765	0.011832	0.011799	0.011967	0.011765	0.011631	0.011765

0.011698	0.011799	0.011799	0.011732	0.011731	0.011900	0.011832
0.011732	0.011832	0.011698	0.011732	0.011866	0.011732	0.011732
0.011732	0.011866	0.011800	0.011698	0.011765	0.011698	0.011799
0.011916	0.011765	0.011799	0.011900	0.011866	0.011799	0.011899
0.011765	0.012101	0.011698	0.011900	0.011698	0.011698	0.011698
0.011933	0.011833	0.011832	0.011900	0.011731	0.011866	0.011698
0.011765	0.011765	0.011799	0.011899	0.011799	0.011899	0.011967
0.011967	0.011933	0.011832	0.011933	0.011899	0.011765	0.011731
0.011765	0.011765	0.011832	0.011698	0.011731	0.011900	0.011765
0.011731	0.011799	0.011933	0.011900	0.011866	0.011799	0.011799
0.011866	0.011799	0.011899	0.011765	0.011899	0.011933	0.012034
0.011799	0.011933	0.011716	0.018276	0.011518	0.011855	0.011793
0.011753	0.011799	0.011899	0.011640	0.011775	0.011820	0.011850
0.011392	0.011610	0.011606	0.011730	0.011646	0.011653	0.011706
0.011576	0.011582	0.011579	0.011457	0.011933	0.011933	0.011906
0.011782	0.011866	0.011866	0.011765	0.011866	0.011866	0.011766
0.011799	0.011732	0.011765	0.011799	0.011766	0.011698	0.011732
0.011732	0.011799	0.011698	0.011799	0.011833	0.011866	0.011799
0.011799	0.011698	0.011833	0.011833	0.011900	0.011800	0.011900
0.011665	0.011665	0.011698	0.011799	0.011665	0.011900	0.011900
0.011933	0.012000	0.011933	0.011967	0.011867	0.011970	0.011799
0.011799	0.011866	0.011900	0.011665	0.011799	0.011765	0.011799
0.011867	0.011866	0.011900	0.011867	0.011767	0.011967	0.011934
0.011873	0.011698	0.011799	0.011900	0.011799	0.011866	0.012034
0.012202	0.011846	0.012068	0.012034	0.011967	0.012068	0.011699
0.011869	0.011699	0.011732	0.011732	0.011799	0.011698	0.011799
0.011833	0.011866	0.011799	0.011799	0.011698	0.011833	0.011833
0.011900	0.011800	0.012000	0.011933	0.012507	0.011989	0.011869
0.011974	0.011927	0.012009	0.011926	0.012041	0.011957	0.011922
0.012434	0.011822	0.011951	0.011890	0.012128	0.012047	0.012081
0.012454	0.011814	0.012075	0.012101	0.011967	0.011698	0.012041
0.011731	0.011832	0.012034	0.012000	0.012007	0.011731	0.011363
0.011832	0.011765	0.011799	0.011832	0.011731	0.011799	0.011698
0.011765	0.011799	0.011765	0.011731	0.011832	0.011765	0.011799
0.011799	0.011731	0.011799	0.011832	0.011731	0.011933	0.011967
0.011866	0.011866	0.011866	0.011866	0.011799	0.011933	0.011832
0.011547	0.011799	0.011766	0.011739	0.011900	0.011899	0.011899
0.011900	0.011832	0.011799	0.011832	0.011866	0.011933	0.011866
0.011866	0.012000	0.011900	0.011899	0.012101	0.012101	0.011967
0.011799	0.011866	0.011900	0.012034	0.011867	0.011900	0.011866
0.011899	0.011765	0.011765	0.012068	0.012068	0.012034	0.011832
0.011933	0.012000	0.011866	0.011933	0.011899	0.011765	0.011832
0.011866	0.011832	0.011765	0.012000	0.011832	0.011933	0.011967
0.011866	0.011909	0.011967	0.011629	0.011512	0.011808	0.011421
0.011610	0.011527	0.011692	0.011686	0.011695	0.011671	0.011538
0.011570	0.011521	0.011561	0.011456	0.011491	0.011663	0.011872
0.011852	0.011769	0.011765	0.011765	0.011734	0.011665	0.011665
0.011732	0.011733	0.011906	0.011704	0.011976	0.011979	0.011866
0.011832	0.011833	0.011799	0.011799	0.011665	0.011631	0.011698
0.011832	0.011832	0.011732	0.011765	0.011799	0.011698	0.011799
0.011724	0.011760	0.011745	0.011780	0.011916	0.011880	0.011880
0.011878	0.011834	0.011577	0.011600	0.011670	0.011908	0.011909
0.011733	0.011772	0.012037	0.012007	0.011935	0.011902	0.011967
0.011899	0.011967	0.011967	0.011799	0.012169	0.012034	0.012101
0.012068	0.011951	0.011845	0.011857	0.011799	0.011900	0.011967
0.012002	0.012034	0.012034	0.012034	0.012122	0.012169	0.011754
0.011669	0.011649	0.011775	0.011740	0.011709	0.011695	0.011740
0.011780	0.011586	0.011680	0.011622	0.011766	0.011813	0.011732
0.011698	0.011631	0.011765	0.011732	0.011665	0.011631	0.011564
0.011665	0.011732	0.011732	0.011732	0.011766	0.011765	0.011770

0.011838	0.011765	0.011765	0.011734	0.011665	0.011665	0.011732
0.011733	0.011906	0.011704	0.011976	0.011979	0.011866	0.011967
0.011967	0.011967	0.011737	0.011861	0.011755	0.011878	0.011621
0.011921	0.011972	0.011865	0.011808	0.011698	0.011900	0.011765
0.011866	0.011799	0.011698	0.011734	0.011900	0.011905	0.011799
0.011698	0.011633	0.011765	0.011698	0.011631	0.011604	0.011900
0.011799	0.011732	0.011765	0.011799	0.011766	0.011698	0.011631
0.011832	0.011866	0.011866	0.011799	0.011866	0.012101	0.011698
0.011799	0.011933	0.011799	0.011832	0.011799	0.011900	0.011660
0.011510	0.011528	0.011461	0.011624	0.011671	0.011635	0.011520
0.011428	0.011429	0.011530	0.011569	0.011430	0.011534	0.011699
0.011598	0.011669	0.011664	0.011675	0.011295	0.012202	0.011464
0.012001	0.011835	0.011970	0.012443	0.011900	0.011564	0.011564
0.012339	0.011903	0.011597	0.011497	0.011497	0.011497	0.011601
0.011429	0.011331	0.011496	0.011396	0.012036	0.012169	0.012103
0.012070	0.012103	0.012207	0.011940	0.012475	0.012483	0.012542
0.012543	0.012577	0.012494	0.012543	0.012545	0.011899	0.012069
0.012137	0.012674	0.012146	0.012236	0.012643	0.011706	0.011598
0.011631	0.011631	0.012035	0.012007	0.012151	0.011967	0.011566
S010	0.011597	0.011597	0.011765	0.011698	0.011698	0.011765
0.011604	0.011536	0.011765	0.000000	0.011262	0.011228	0.011195
0.011228	0.011396	0.011195	0.011228	0.011295	0.011262	0.011228
0.011195	0.011228	0.011329	0.011195	0.011228	0.011228	0.011570
0.011463	0.011228	0.011127	0.011362	0.011295	0.011262	0.011295
0.011262	0.011362	0.011262	0.011262	0.011295	0.011262	0.011430
0.011195	0.011262	0.011295	0.011228	0.011329	0.011362	0.011295
0.011262	0.011430	0.011329	0.011597	0.011262	0.011530	0.011262
0.011228	0.011329	0.011396	0.011530	0.011631	0.011329	0.011429
0.011295	0.011362	0.011262	0.011497	0.011732	0.011262	0.011329
0.011228	0.011329	0.011295	0.011362	0.011295	0.011295	0.011295
0.011195	0.011228	0.011195	0.011295	0.011262	0.011296	0.011362
0.011295	0.011262	0.011329	0.011228	0.011362	0.011195	0.011262
0.011429	0.011362	0.011597	0.011430	0.011262	0.011497	0.011262
0.011431	0.011658	0.011475	0.011177	0.011172	0.011244	0.011375
0.012011	0.011467	0.011184	0.011497	0.011497	0.011295	0.011396
0.011329	0.011295	0.011362	0.011262	0.011362	0.011295	0.011295
0.011262	0.011362	0.011564	0.011429	0.011329	0.011295	0.011228
0.011195	0.011195	0.011262	0.011228	0.011430	0.011396	0.011329
0.011430	0.011497	0.011228	0.011228	0.011262	0.011262	0.011263
0.011331	0.011262	0.011262	0.011096	0.011169	0.011130	0.011262
0.011263	0.011198	0.011245	0.011233	0.011162	0.011197	0.011229
0.011456	0.011267	0.011301	0.011195	0.011199	0.011329	0.011296
0.011363	0.011296	0.011296	0.011396	0.011329	0.011698	0.011497
0.011530	0.011396	0.011262	0.011363	0.011161	0.011396	0.011195
0.011262	0.011262	0.011228	0.011195	0.011228	0.011195	0.011295
0.011262	0.011362	0.011295	0.011195	0.011296	0.011396	0.011398
0.011396	0.011295	0.011195	0.011128	0.011262	0.011195	0.011128
0.011230	0.011396	0.011195	0.011396	0.011161	0.011262	0.011228
0.011329	0.011228	0.011262	0.011228	0.011262	0.011127	0.011262
0.011295	0.011228	0.011295	0.011228	0.011262	0.011295	0.011530
0.011631	0.011564	0.011631	0.011397	0.011631	0.011262	0.011228
0.011263	0.011505	0.011335	0.011163	0.011637	0.011129	0.011218
0.011638	0.011536	0.011429	0.011631	0.011333	0.011497	0.011463
0.011396	0.011564	0.011416	0.011225	0.011270	0.011295	0.011365
0.011463	0.011364	0.011500	0.011568	0.011497	0.011564	0.011670
0.011399	0.011569	0.011500	0.011432	0.011699	0.011161	0.011363
0.011329	0.011262	0.011161	0.011161	0.011228	0.011228	0.011295
0.011228	0.011295	0.011195	0.011295	0.011228	0.011228	0.011295
0.011228	0.011665	0.011564	0.011597	0.011127	0.011228	0.011262
0.011262	0.011228	0.011295	0.011329	0.011195	0.011195	0.011195

0.011732	0.011362	0.011362	0.011161	0.011262	0.011430	0.011195
0.011195	0.011262	0.011228	0.011396	0.011195	0.011329	0.011463
0.011396	0.011228	0.011228	0.011161	0.011161	0.011329	0.011262
0.011161	0.011262	0.011127	0.011161	0.011295	0.011430	0.011195
0.011161	0.011295	0.011228	0.011127	0.011463	0.011127	0.011295
0.011368	0.011195	0.011497	0.011329	0.011362	0.011228	0.011329
0.011195	0.011530	0.011127	0.011396	0.011195	0.011195	0.011127
0.011362	0.011564	0.011262	0.011329	0.011161	0.011564	0.011195
0.011195	0.011195	0.011228	0.011329	0.011228	0.011329	0.011396
0.011396	0.011362	0.011262	0.011362	0.011329	0.011195	0.011161
0.011195	0.011195	0.011262	0.011195	0.011161	0.011329	0.011195
0.011228	0.011228	0.011362	0.011396	0.011295	0.011228	0.011228
0.011295	0.011228	0.011329	0.011195	0.011329	0.011429	0.011665
0.011228	0.011631	0.011233	0.017747	0.011126	0.011366	0.011583
0.011545	0.011588	0.011322	0.011292	0.011664	0.011612	0.011242
0.011487	0.011487	0.011138	0.011228	0.011216	0.011258	0.011232
0.011482	0.011395	0.011394	0.011193	0.011362	0.011396	0.011398
0.011266	0.011362	0.011362	0.011329	0.011295	0.011295	0.011262
0.011295	0.011228	0.011262	0.011295	0.011262	0.011195	0.011228
0.011228	0.011295	0.011195	0.011295	0.011329	0.011362	0.011262
0.011295	0.011262	0.011598	0.011598	0.011329	0.011363	0.011396
0.011161	0.011161	0.011195	0.011295	0.011161	0.011396	0.011396
0.011430	0.011429	0.011362	0.011463	0.011631	0.011464	0.011295
0.011295	0.011362	0.011396	0.011228	0.011295	0.011262	0.011295
0.011363	0.011363	0.011396	0.011363	0.011262	0.011463	0.011430
0.011297	0.011195	0.011295	0.011396	0.011295	0.011430	0.011463
0.011631	0.011333	0.011497	0.011463	0.011396	0.011564	0.011195
0.011363	0.011262	0.011228	0.011228	0.011295	0.011195	0.011295
0.011329	0.011362	0.011262	0.011295	0.011262	0.011598	0.011598
0.011329	0.011363	0.011429	0.011362	0.012002	0.011417	0.011342
0.011412	0.011372	0.011482	0.011372	0.011480	0.011405	0.011370
0.011953	0.011268	0.011403	0.011337	0.011611	0.011468	0.011570
0.011978	0.011437	0.011534	0.011597	0.011463	0.011463	0.011500
0.011161	0.011329	0.011463	0.011429	0.011432	0.011429	0.011397
0.011262	0.011195	0.011228	0.011262	0.011161	0.011228	0.011127
0.011262	0.011295	0.011195	0.011161	0.011262	0.011195	0.011228
0.011228	0.011161	0.011228	0.011262	0.011161	0.011362	0.011396
0.011295	0.011362	0.011295	0.011295	0.011228	0.011631	0.011262
0.011304	0.011228	0.011463	0.011467	0.011329	0.011329	0.011329
0.011329	0.011262	0.011228	0.011262	0.011362	0.011362	0.011295
0.011295	0.011430	0.011597	0.011329	0.011530	0.011530	0.011396
0.011228	0.011295	0.011329	0.011732	0.011295	0.011329	0.011295
0.011329	0.011262	0.011262	0.011564	0.011497	0.011530	0.011262
0.011362	0.011430	0.011295	0.011362	0.011329	0.011195	0.011530
0.011295	0.011262	0.011262	0.011430	0.011262	0.011362	0.011597
0.011228	0.011672	0.011665	0.011467	0.011173	0.011366	0.011267
0.011170	0.011683	0.011201	0.011417	0.011207	0.011366	0.011273
0.011468	0.000000	0.011227	0.000000	0.011168	0.011261	0.011344
0.011362	0.011257	0.011342	0.011272	0.011296	0.011430	0.011497
0.011497	0.011363	0.011466	0.011467	0.011603	0.011626	0.011295
0.011530	0.011396	0.011262	0.011363	0.011161	0.011396	0.011195
0.011262	0.011262	0.011228	0.011195	0.011228	0.011195	0.011295
0.011270	0.011270	0.011266	0.011300	0.011437	0.011402	0.011402
0.011401	0.011329	0.011097	0.011132	0.011263	0.011433	0.011433
0.011228	0.011297	0.011531	0.011500	0.011430	0.011464	0.011396
0.011329	0.011396	0.011396	0.011295	0.011866	0.011463	0.011530
0.011564	0.011630	0.011265	0.011337	0.011228	0.011430	0.011396
0.011430	0.011463	0.011463	0.011463	0.011610	0.011597	0.011234
0.011128	0.011130	0.011230	0.011196	0.011299	0.011135	0.011468
0.011266	0.011367	0.011108	0.011248	0.011262	0.011334	0.011228

0.011195	0.011195	0.011262	0.011228	0.011430	0.011396	0.011329
0.011430	0.011497	0.011228	0.011228	0.011262	0.011262	0.011263
0.011331	0.011262	0.011262	0.011296	0.011430	0.011497	0.011497
0.011363	0.011466	0.011467	0.011603	0.011626	0.011295	0.011463
0.011396	0.011396	0.011229	0.011408	0.011338	0.011367	0.011235
0.011405	0.011561	0.011357	0.011298	0.011195	0.011396	0.011262
0.011362	0.011295	0.011195	0.011296	0.011396	0.011398	0.011295
0.011195	0.011128	0.011262	0.011195	0.011128	0.011230	0.011396
0.011295	0.011228	0.011262	0.011295	0.011262	0.011195	0.011396
0.011262	0.011295	0.011295	0.011228	0.011295	0.011530	0.011195
0.011228	0.011631	0.011228	0.011262	0.011228	0.011597	0.011189
0.011622	0.011145	0.011261	0.011422	0.011508	0.011419	0.011144
0.011218	0.011396	0.011497	0.011603	0.011464	0.011601	0.011733
0.011632	0.011669	0.011631	0.011880	0.011597	0.012472	0.011633
0.012372	0.012139	0.012274	0.012545	0.011597	0.011295	0.011598
0.012036	0.011971	0.011564	0.011463	0.011531	0.011531	0.011500
0.011396	0.011399	0.011463	0.011430	0.011800	0.011866	0.011867
0.011767	0.011867	0.011970	0.011737	0.012036	0.012042	0.012104
0.012104	0.012138	0.012084	0.012104	0.012106	0.011329	0.011632
0.011699	0.012203	0.011704	0.011934	0.012407	0.011468	0.011363
0.011329	0.011329	0.011800	0.011800	0.011940	0.011665	0.011330
S011 0.011664	0.011664	0.012001	0.011765	0.011765	0.011832	
0.011671	0.011603	0.011832	0.011262	0.000000	0.011296	0.011262
0.011295	0.011463	0.011262	0.011295	0.011362	0.011329	0.011295
0.011262	0.011295	0.011396	0.011262	0.011295	0.011295	0.011637
0.011530	0.011295	0.011194	0.011429	0.011362	0.011329	0.011362
0.011329	0.011429	0.011329	0.011329	0.011362	0.011329	0.011496
0.011262	0.011329	0.011362	0.011295	0.011396	0.011429	0.011362
0.011329	0.011496	0.011396	0.011664	0.011329	0.011597	0.011329
0.011295	0.011396	0.011463	0.011597	0.011698	0.011396	0.011496
0.011362	0.011429	0.011329	0.011564	0.011799	0.011329	0.011396
0.011295	0.011362	0.011362	0.011429	0.011362	0.011362	0.011362
0.011262	0.011295	0.011262	0.011362	0.011329	0.011363	0.011429
0.011362	0.011329	0.011396	0.011295	0.011429	0.011262	0.011329
0.011496	0.011429	0.011664	0.011496	0.011329	0.011564	0.011329
0.011468	0.011695	0.011513	0.011217	0.011210	0.011318	0.011415
0.012048	0.011467	0.011225	0.011563	0.011564	0.011362	0.011463
0.011396	0.011362	0.011429	0.011329	0.011429	0.011362	0.011362
0.011329	0.011429	0.011631	0.011496	0.011396	0.011362	0.011295
0.011262	0.011262	0.011329	0.011295	0.011497	0.011463	0.011396
0.011497	0.011564	0.011295	0.011295	0.011329	0.011329	0.011330
0.011398	0.011329	0.011329	0.011204	0.011277	0.011236	0.011329
0.011331	0.011266	0.011282	0.011302	0.011230	0.011266	0.011297
0.011564	0.011336	0.011370	0.011262	0.011268	0.011397	0.011363
0.011430	0.011363	0.011363	0.011463	0.011396	0.011765	0.011564
0.011597	0.011463	0.011329	0.011429	0.011228	0.011463	0.011262
0.011329	0.011329	0.011295	0.011262	0.011295	0.011262	0.011362
0.011329	0.011429	0.011362	0.011262	0.011363	0.011463	0.011466
0.011463	0.011362	0.011262	0.011195	0.011329	0.011262	0.011195
0.011298	0.011463	0.011262	0.011463	0.011228	0.011329	0.011295
0.011396	0.011295	0.011329	0.011295	0.011329	0.011195	0.011329
0.011362	0.011295	0.011362	0.011295	0.011329	0.011362	0.011597
0.011698	0.011631	0.011698	0.011431	0.011698	0.011329	0.011295
0.011330	0.011586	0.011472	0.011197	0.011705	0.011197	0.011296
0.011705	0.011604	0.011496	0.011698	0.011402	0.011564	0.011530
0.011463	0.011631	0.011486	0.011349	0.011354	0.011362	0.011432
0.011530	0.011431	0.011567	0.011635	0.011564	0.011631	0.011738
0.011466	0.011637	0.011568	0.011500	0.011766	0.011228	0.011431
0.011396	0.011329	0.011228	0.011228	0.011295	0.011295	0.011362
0.011295	0.011362	0.011262	0.011362	0.011295	0.011295	0.011362

0.011295	0.011731	0.011631	0.011664	0.011194	0.011295	0.011329
0.011329	0.011295	0.011362	0.011396	0.011262	0.011262	0.011262
0.011799	0.011429	0.011429	0.011228	0.011329	0.011496	0.011262
0.011262	0.011329	0.011295	0.011463	0.011262	0.011396	0.011530
0.011463	0.011295	0.011295	0.011228	0.011228	0.011396	0.011329
0.011228	0.011329	0.011195	0.011228	0.011362	0.011497	0.011262
0.011228	0.011362	0.011295	0.011195	0.011530	0.011195	0.011362
0.011437	0.011262	0.011564	0.011396	0.011429	0.011295	0.011396
0.011262	0.011597	0.011194	0.011463	0.011262	0.011262	0.011194
0.011429	0.011631	0.011329	0.011396	0.011228	0.011631	0.011262
0.011262	0.011262	0.011295	0.011396	0.011295	0.011396	0.011463
0.011463	0.011429	0.011329	0.011429	0.011396	0.011262	0.011228
0.011262	0.011262	0.011329	0.011262	0.011228	0.011329	0.011262
0.011295	0.011295	0.011429	0.011463	0.011362	0.011295	0.011295
0.011362	0.011295	0.011396	0.011262	0.011396	0.011496	0.011799
0.011295	0.011698	0.011267	0.017785	0.011257	0.011366	0.011618
0.011579	0.011623	0.011322	0.011234	0.011719	0.011647	0.011364
0.011487	0.011549	0.011177	0.011186	0.011216	0.011307	0.011232
0.011482	0.011433	0.011431	0.011259	0.011429	0.011463	0.011466
0.011335	0.011429	0.011429	0.011396	0.011362	0.011362	0.011329
0.011362	0.011295	0.011329	0.011362	0.011329	0.011262	0.011295
0.011295	0.011362	0.011262	0.011362	0.011396	0.011429	0.011329
0.011362	0.011329	0.011665	0.011665	0.011396	0.011430	0.011463
0.011228	0.011228	0.011262	0.011362	0.011228	0.011463	0.011463
0.011497	0.011496	0.011429	0.011530	0.011698	0.011531	0.011362
0.011362	0.011429	0.011463	0.011295	0.011362	0.011329	0.011362
0.011430	0.011430	0.011463	0.011430	0.011330	0.011530	0.011497
0.011365	0.011262	0.011362	0.011463	0.011362	0.011497	0.011530
0.011698	0.011402	0.011564	0.011530	0.011463	0.011631	0.011262
0.011431	0.011329	0.011295	0.011295	0.011362	0.011262	0.011362
0.011396	0.011429	0.011329	0.011362	0.011329	0.011665	0.011665
0.011396	0.011430	0.011496	0.011429	0.012069	0.011489	0.011412
0.011482	0.011441	0.011552	0.011441	0.011550	0.011474	0.011439
0.012021	0.011337	0.011471	0.011406	0.011680	0.011536	0.011638
0.012046	0.011512	0.011601	0.011664	0.011530	0.011530	0.011567
0.011228	0.011396	0.011530	0.011496	0.011499	0.011496	0.011464
0.011127	0.011262	0.011295	0.011329	0.011228	0.011295	0.011194
0.011329	0.011362	0.011262	0.011228	0.011329	0.011262	0.011295
0.011295	0.011228	0.011295	0.011329	0.011228	0.011429	0.011463
0.011362	0.011429	0.011362	0.011362	0.011295	0.011698	0.011329
0.011373	0.011295	0.011530	0.011535	0.011396	0.011396	0.011396
0.011396	0.011329	0.011295	0.011329	0.011429	0.011429	0.011362
0.011362	0.011429	0.011664	0.011396	0.011597	0.011597	0.011463
0.011295	0.011362	0.011396	0.011799	0.011362	0.011396	0.011362
0.011396	0.011329	0.011329	0.011631	0.011564	0.011597	0.011329
0.011429	0.011496	0.011362	0.011429	0.011396	0.011262	0.011597
0.011362	0.011329	0.011329	0.011496	0.011329	0.011429	0.011664
0.011362	0.011739	0.011731	0.011507	0.011286	0.011434	0.011318
0.011225	0.011722	0.011236	0.011462	0.011304	0.011416	0.011379
0.011468	0.011126	0.011339	0.011192	0.011276	0.011395	0.011385
0.011399	0.011296	0.011377	0.011307	0.011363	0.011497	0.011564
0.011564	0.011430	0.011533	0.011535	0.011670	0.011696	0.011362
0.011597	0.011463	0.011329	0.011429	0.011228	0.011463	0.011262
0.011329	0.011329	0.011295	0.011262	0.011295	0.011262	0.011362
0.011340	0.011340	0.011334	0.011368	0.011505	0.011470	0.011470
0.011469	0.011396	0.011171	0.011168	0.011331	0.011501	0.011501
0.011296	0.011365	0.011599	0.011567	0.011497	0.011531	0.011463
0.011396	0.011463	0.011463	0.011362	0.011933	0.011530	0.011597
0.011631	0.011701	0.011333	0.011407	0.011295	0.011497	0.011463
0.011497	0.011530	0.011530	0.011530	0.011689	0.011664	0.011303

0.011195	0.011199	0.011298	0.011264	0.011368	0.011210	0.011536
0.011335	0.011455	0.011203	0.011360	0.011329	0.011402	0.011295
0.011262	0.011262	0.011329	0.011295	0.011497	0.011463	0.011396
0.011497	0.011564	0.011295	0.011295	0.011329	0.011329	0.011330
0.011398	0.011329	0.011329	0.011363	0.011497	0.011564	0.011564
0.011430	0.011533	0.011535	0.011670	0.011696	0.011362	0.011530
0.011463	0.011463	0.011297	0.011478	0.011408	0.011435	0.011235
0.011473	0.011607	0.011357	0.011366	0.011262	0.011463	0.011329
0.011429	0.011362	0.011262	0.011363	0.011463	0.011466	0.011362
0.011262	0.011195	0.011329	0.011262	0.011195	0.011298	0.011463
0.011362	0.011295	0.011329	0.011362	0.011329	0.011262	0.011463
0.011329	0.011362	0.011362	0.011295	0.011362	0.011597	0.011262
0.011295	0.011698	0.011295	0.011329	0.011295	0.011664	0.011189
0.011678	0.011230	0.011261	0.011463	0.011549	0.011455	0.011144
0.011218	0.011463	0.011564	0.011671	0.011531	0.011635	0.011800
0.011699	0.011737	0.011631	0.011914	0.011664	0.012539	0.011700
0.012439	0.012207	0.012341	0.012612	0.011664	0.011362	0.011665
0.012103	0.012004	0.011631	0.011530	0.011598	0.011598	0.011567
0.011463	0.011433	0.011530	0.011497	0.011867	0.011933	0.011867
0.011834	0.011934	0.012038	0.011770	0.012104	0.012110	0.012171
0.012171	0.012205	0.012118	0.012171	0.012173	0.011396	0.011699
0.011766	0.012270	0.011772	0.012001	0.012474	0.011536	0.011430
0.011396	0.011396	0.011867	0.011924	0.012010	0.011732	0.011397
S012	0.011632	0.011632	0.011767	0.011733	0.011733	0.011801
0.011639	0.011537	0.011733	0.011228	0.011296	0.000000	0.011228
0.011262	0.011430	0.011228	0.011262	0.011262	0.011296	0.011262
0.011228	0.011262	0.011363	0.011229	0.011262	0.011262	0.011605
0.011498	0.011262	0.011161	0.011397	0.011329	0.011296	0.011329
0.011296	0.011397	0.011296	0.011296	0.011329	0.011296	0.011464
0.011228	0.011296	0.011329	0.011262	0.011363	0.011397	0.011329
0.011296	0.011464	0.011363	0.011632	0.011296	0.011565	0.011296
0.011262	0.011363	0.011430	0.011565	0.011666	0.011363	0.011464
0.011329	0.011397	0.011296	0.011531	0.011767	0.011296	0.011363
0.011262	0.011363	0.011329	0.011397	0.011329	0.011329	0.011329
0.011228	0.011262	0.011228	0.011329	0.011296	0.011330	0.011397
0.011329	0.011296	0.011363	0.011262	0.011397	0.011229	0.011296
0.011464	0.011397	0.011632	0.011464	0.011296	0.011531	0.011296
0.011431	0.011658	0.011475	0.011177	0.011172	0.011318	0.011375
0.012011	0.011467	0.011184	0.011531	0.011531	0.011329	0.011430
0.011363	0.011329	0.011397	0.011296	0.011397	0.011329	0.011329
0.011296	0.011397	0.011599	0.011464	0.011363	0.011330	0.011262
0.011228	0.011228	0.011296	0.011262	0.011464	0.011430	0.011363
0.011464	0.011531	0.011262	0.011262	0.011296	0.011296	0.011297
0.011365	0.011296	0.011296	0.011168	0.011241	0.011201	0.011296
0.011298	0.011232	0.011282	0.011267	0.011196	0.011232	0.011196
0.011529	0.011302	0.011336	0.011229	0.011233	0.011363	0.011330
0.011397	0.011330	0.011329	0.011430	0.011363	0.011733	0.011498
0.011565	0.011430	0.011296	0.011397	0.011195	0.011430	0.011229
0.011296	0.011296	0.011262	0.011228	0.011262	0.011228	0.011329
0.011296	0.011397	0.011329	0.011228	0.011330	0.011430	0.011433
0.011430	0.011329	0.011228	0.011162	0.011296	0.011229	0.011161
0.011264	0.011431	0.011229	0.011431	0.011195	0.011296	0.011262
0.011363	0.011262	0.011296	0.011262	0.011296	0.011161	0.011296
0.011329	0.011262	0.011329	0.011262	0.011296	0.011329	0.011531
0.011666	0.011599	0.011666	0.011431	0.011666	0.011296	0.011262
0.011297	0.011586	0.011381	0.011198	0.011672	0.011163	0.011296
0.011673	0.011571	0.011464	0.011666	0.011368	0.011531	0.011498
0.011430	0.011599	0.011452	0.011308	0.011354	0.011329	0.011399
0.011498	0.011398	0.011535	0.011603	0.011531	0.011599	0.011705
0.011433	0.011604	0.011535	0.011467	0.011734	0.011195	0.011398

0.011363	0.011296	0.011195	0.011195	0.011262	0.011262	0.011329
0.011262	0.011329	0.011228	0.011329	0.011262	0.011262	0.011329
0.011262	0.011700	0.011599	0.011632	0.011161	0.011262	0.011296
0.011296	0.011262	0.011329	0.011363	0.011228	0.011228	0.011228
0.011767	0.011363	0.011397	0.011195	0.011296	0.011464	0.011229
0.011228	0.011296	0.011262	0.011430	0.011228	0.011363	0.011498
0.011430	0.011262	0.011262	0.011195	0.011195	0.011363	0.011296
0.011195	0.011296	0.011161	0.011195	0.011329	0.011464	0.011229
0.011195	0.011329	0.011262	0.011161	0.011498	0.011161	0.011329
0.011403	0.011161	0.011531	0.011363	0.011397	0.011262	0.011363
0.011228	0.011565	0.011161	0.011430	0.011228	0.011228	0.011161
0.011397	0.011599	0.011296	0.011363	0.011195	0.011599	0.011228
0.011229	0.011228	0.011262	0.011363	0.011262	0.011363	0.011430
0.011430	0.011397	0.011296	0.011397	0.011363	0.011228	0.011195
0.011228	0.011228	0.011296	0.011228	0.011195	0.011363	0.011228
0.011262	0.011262	0.011397	0.011430	0.011329	0.011262	0.011262
0.011329	0.011262	0.011363	0.011228	0.011363	0.011464	0.011700
0.011262	0.011666	0.011267	0.017748	0.011191	0.011366	0.011618
0.011579	0.011623	0.011322	0.011292	0.011609	0.011647	0.011303
0.011487	0.011426	0.011138	0.011270	0.011216	0.011208	0.011232
0.011482	0.011395	0.011394	0.011259	0.011397	0.011430	0.011433
0.011301	0.011397	0.011397	0.011363	0.011296	0.011296	0.011296
0.011329	0.011262	0.011296	0.011329	0.011296	0.011229	0.011262
0.011262	0.011329	0.011228	0.011330	0.011363	0.011397	0.011296
0.011329	0.011296	0.011632	0.011632	0.011363	0.011397	0.011430
0.011195	0.011195	0.011228	0.011329	0.011195	0.011430	0.011430
0.011464	0.011464	0.011397	0.011498	0.011666	0.011499	0.011329
0.011329	0.011397	0.011430	0.011262	0.011329	0.011296	0.011329
0.011397	0.011397	0.011430	0.011397	0.011296	0.011498	0.011464
0.011332	0.011229	0.011329	0.011430	0.011330	0.011464	0.011498
0.011666	0.011368	0.011531	0.011498	0.011430	0.011599	0.011229
0.011398	0.011296	0.011262	0.011262	0.011329	0.011228	0.011330
0.011363	0.011397	0.011296	0.011329	0.011296	0.011632	0.011632
0.011363	0.011397	0.011464	0.011397	0.012037	0.011454	0.011378
0.011448	0.011408	0.011518	0.011407	0.011516	0.011440	0.011406
0.011990	0.011303	0.011438	0.011372	0.011647	0.011503	0.011606
0.012014	0.011475	0.011331	0.011329	0.011329	0.011363	0.011297
0.011195	0.011229	0.011363	0.011329	0.011331	0.011464	0.011431
0.011296	0.011228	0.011262	0.011296	0.011195	0.011262	0.011161
0.011296	0.011329	0.011228	0.011195	0.011296	0.011228	0.011262
0.011262	0.011195	0.011262	0.011296	0.011195	0.011397	0.011430
0.011329	0.011397	0.011329	0.011329	0.011262	0.011666	0.011296
0.011339	0.011262	0.011498	0.011502	0.011363	0.011363	0.011363
0.011363	0.011296	0.011262	0.011296	0.011397	0.011397	0.011329
0.011329	0.011464	0.011632	0.011363	0.011565	0.011565	0.011430
0.011262	0.011329	0.011363	0.011767	0.011330	0.011363	0.011329
0.011363	0.011296	0.011296	0.011531	0.011532	0.011565	0.011296
0.011397	0.011464	0.011329	0.011397	0.011363	0.011229	0.011565
0.011329	0.011296	0.011296	0.011464	0.011296	0.011397	0.011632
0.011262	0.011707	0.011700	0.011467	0.011229	0.011400	0.011267
0.011170	0.011683	0.011236	0.011417	0.011255	0.011264	0.011379
0.011468	0.011126	0.011339	0.011192	0.011222	0.011395	0.011344
0.011362	0.011257	0.011377	0.011307	0.011330	0.011464	0.011531
0.011498	0.011397	0.011501	0.011502	0.011638	0.011662	0.011296
0.011565	0.011430	0.011296	0.011397	0.011195	0.011430	0.011229
0.011296	0.011296	0.011262	0.011228	0.011262	0.011228	0.011329
0.011305	0.011305	0.011300	0.011334	0.011471	0.011436	0.011436
0.011435	0.011363	0.011171	0.011168	0.011298	0.011467	0.011468
0.011262	0.011331	0.011566	0.011534	0.011464	0.011498	0.011430
0.011363	0.011430	0.011430	0.011329	0.011902	0.011498	0.011565

0.011599	0.011667	0.011300	0.011372	0.011262	0.011464	0.011430
0.011465	0.011464	0.011498	0.011498	0.011611	0.011632	0.011269
0.011162	0.011164	0.011265	0.011162	0.011334	0.011172	0.011503
0.011301	0.011455	0.011203	0.011323	0.011296	0.011368	0.011262
0.011228	0.011228	0.011296	0.011262	0.011464	0.011430	0.011363
0.011464	0.011531	0.011262	0.011262	0.011296	0.011296	0.011297
0.011365	0.011296	0.011296	0.011330	0.011464	0.011531	0.011498
0.011397	0.011501	0.011502	0.011638	0.011662	0.011296	0.011498
0.011430	0.011430	0.011264	0.011444	0.011373	0.011401	0.011270
0.011439	0.011607	0.011442	0.011333	0.011229	0.011431	0.011296
0.011397	0.011329	0.011228	0.011330	0.011430	0.011433	0.011329
0.011228	0.011162	0.011296	0.011229	0.011161	0.011264	0.011431
0.011329	0.011262	0.011296	0.011329	0.011296	0.011229	0.011430
0.011296	0.011329	0.011329	0.011262	0.011329	0.011565	0.011228
0.011262	0.011666	0.011262	0.011296	0.011262	0.011632	0.011189
0.011679	0.011188	0.011261	0.011422	0.011508	0.011419	0.011144
0.011218	0.011430	0.011531	0.011638	0.011498	0.011636	0.011767
0.011667	0.011704	0.011666	0.011915	0.011632	0.012509	0.011667
0.012374	0.012173	0.012310	0.012579	0.011632	0.011329	0.011632
0.012071	0.011837	0.011599	0.011498	0.011565	0.011565	0.011535
0.011430	0.011434	0.011498	0.011464	0.011835	0.011902	0.011902
0.011802	0.011902	0.012004	0.011771	0.012071	0.012077	0.012139
0.012139	0.012173	0.012119	0.012139	0.012140	0.011363	0.011666
0.011734	0.012205	0.011739	0.011970	0.012138	0.011502	0.011397
0.011363	0.011363	0.011835	0.011884	0.011976	0.011700	0.011364
S013	0.011597	0.011597	0.011933	0.011698	0.011698	0.011765
0.011569	0.011502	0.011765	0.011195	0.011262	0.011228	0.000000
0.011228	0.011396	0.011194	0.011228	0.011295	0.011262	0.011228
0.011194	0.011228	0.011329	0.011195	0.011228	0.011228	0.011535
0.011463	0.011228	0.011127	0.011362	0.011228	0.011262	0.011295
0.011262	0.011362	0.011262	0.011262	0.011295	0.011262	0.011429
0.011194	0.011262	0.011295	0.011228	0.011329	0.011362	0.011295
0.011262	0.011429	0.011329	0.011597	0.011262	0.011530	0.011262
0.011228	0.011329	0.011396	0.011530	0.011631	0.011329	0.011429
0.011295	0.011362	0.011262	0.011496	0.011732	0.011262	0.011329
0.011228	0.011329	0.011295	0.011362	0.011295	0.011295	0.011295
0.011194	0.011228	0.011194	0.011295	0.011262	0.011296	0.011362
0.011295	0.011262	0.011329	0.011228	0.011362	0.011194	0.011262
0.011429	0.011362	0.011597	0.011429	0.011262	0.011463	0.011262
0.011394	0.011620	0.011437	0.011138	0.011172	0.011281	0.011336
0.012011	0.011416	0.011143	0.011496	0.011496	0.011295	0.011396
0.011329	0.011295	0.011362	0.011262	0.011362	0.011295	0.011295
0.011262	0.011362	0.011564	0.011429	0.011329	0.011295	0.011228
0.011195	0.011195	0.011262	0.011228	0.011429	0.011396	0.011329
0.011429	0.011497	0.011228	0.011228	0.011262	0.011262	0.011263
0.011331	0.011262	0.011262	0.011132	0.011205	0.011165	0.011262
0.011263	0.011198	0.011245	0.011233	0.011162	0.011197	0.011229
0.011492	0.011267	0.011301	0.011195	0.011129	0.011329	0.011296
0.011363	0.011296	0.011296	0.011396	0.011329	0.011698	0.011497
0.011530	0.011396	0.011262	0.011362	0.011161	0.011396	0.011195
0.011262	0.011262	0.011228	0.011194	0.011228	0.011195	0.011295
0.011262	0.011362	0.011295	0.011195	0.011296	0.011396	0.011398
0.011396	0.011295	0.011195	0.011128	0.011262	0.011195	0.011127
0.011230	0.011396	0.011195	0.011396	0.011161	0.011262	0.011228
0.011329	0.011228	0.011262	0.011228	0.011262	0.011127	0.011262
0.011295	0.011228	0.011295	0.011228	0.011262	0.011295	0.011530
0.011631	0.011564	0.011631	0.011397	0.011631	0.011262	0.011228
0.011263	0.011545	0.011380	0.011163	0.011637	0.011129	0.011257
0.011603	0.011536	0.011429	0.011631	0.011333	0.011496	0.011463
0.011396	0.011564	0.011415	0.011225	0.011270	0.011295	0.011365

0.011463	0.011364	0.011500	0.011568	0.011497	0.011564	0.011670
0.011399	0.011569	0.011500	0.011432	0.011699	0.011161	0.011363
0.011329	0.011262	0.011161	0.011161	0.011228	0.011228	0.011295
0.011228	0.011295	0.011194	0.011295	0.011228	0.011228	0.011295
0.011228	0.011664	0.011564	0.011597	0.011127	0.011228	0.011262
0.011262	0.011228	0.011295	0.011329	0.011194	0.011194	0.011194
0.011731	0.011362	0.011362	0.011161	0.011262	0.011429	0.011194
0.011194	0.011262	0.011228	0.011396	0.011194	0.011329	0.011463
0.011396	0.011228	0.011228	0.011161	0.011161	0.011329	0.011262
0.011161	0.011262	0.011127	0.011161	0.011295	0.011430	0.011195
0.011161	0.011295	0.011228	0.011127	0.011463	0.011127	0.011295
0.011368	0.011195	0.011496	0.011329	0.011362	0.011228	0.011329
0.011194	0.011530	0.011127	0.011396	0.011194	0.011194	0.011127
0.011362	0.011564	0.011262	0.011329	0.011161	0.011564	0.011194
0.011194	0.011194	0.011228	0.011329	0.011228	0.011329	0.011396
0.011396	0.011362	0.011262	0.011362	0.011329	0.011194	0.011161
0.011194	0.011194	0.011262	0.011194	0.011161	0.011329	0.011194
0.011228	0.011228	0.011362	0.011396	0.011295	0.011228	0.011228
0.011295	0.011228	0.011329	0.011194	0.011329	0.011429	0.011732
0.011228	0.011631	0.011233	0.017747	0.011126	0.011305	0.011583
0.011545	0.011588	0.011270	0.011234	0.011609	0.011612	0.011242
0.011440	0.011426	0.011099	0.011186	0.011216	0.011208	0.011146
0.011436	0.011395	0.011394	0.011259	0.011362	0.011396	0.011398
0.011266	0.011362	0.011362	0.011329	0.011295	0.011295	0.011262
0.011295	0.011228	0.011262	0.011295	0.011262	0.011195	0.011228
0.011228	0.011295	0.011195	0.011295	0.011329	0.011362	0.011262
0.011295	0.011262	0.011597	0.011597	0.011329	0.011363	0.011396
0.011161	0.011161	0.011195	0.011295	0.011161	0.011396	0.011396
0.011429	0.011429	0.011362	0.011463	0.011631	0.011464	0.011295
0.011295	0.011362	0.011396	0.011228	0.011295	0.011262	0.011295
0.011363	0.011362	0.011396	0.011363	0.011262	0.011463	0.011429
0.011297	0.011195	0.011295	0.011396	0.011295	0.011430	0.011463
0.011631	0.011333	0.011496	0.011463	0.011396	0.011564	0.011195
0.011363	0.011262	0.011228	0.011228	0.011295	0.011195	0.011295
0.011329	0.011362	0.011262	0.011295	0.011262	0.011597	0.011597
0.011329	0.011363	0.011429	0.011362	0.012002	0.011417	0.011341
0.011412	0.011372	0.011482	0.011372	0.011480	0.011405	0.011370
0.011953	0.011268	0.011403	0.011337	0.011611	0.011468	0.011570
0.011978	0.011437	0.011533	0.011597	0.011463	0.011463	0.011499
0.011161	0.011329	0.011463	0.011429	0.011432	0.011429	0.011397
0.011262	0.011194	0.011228	0.011262	0.011161	0.011228	0.011127
0.011262	0.011295	0.011194	0.011161	0.011262	0.011194	0.011228
0.011228	0.011161	0.011228	0.011262	0.011161	0.011362	0.011396
0.011295	0.011362	0.011295	0.011295	0.011228	0.011631	0.011262
0.011304	0.011228	0.011463	0.011467	0.011329	0.011329	0.011329
0.011329	0.011262	0.011228	0.011262	0.011362	0.011362	0.011228
0.011295	0.011429	0.011597	0.011329	0.011530	0.011530	0.011396
0.011228	0.011295	0.011329	0.011732	0.011295	0.011329	0.011295
0.011329	0.011262	0.011262	0.011564	0.011497	0.011530	0.011262
0.011362	0.011429	0.011295	0.011362	0.011329	0.011194	0.011530
0.011295	0.011262	0.011262	0.011429	0.011262	0.011362	0.011597
0.011295	0.011637	0.011664	0.011426	0.011173	0.011366	0.011215
0.011115	0.011644	0.011201	0.011373	0.011207	0.011315	0.011326
0.011417	0.000000	0.011283	0.000000	0.011168	0.011261	0.011303
0.011324	0.011218	0.011342	0.011272	0.011296	0.011429	0.011497
0.011497	0.011363	0.011466	0.011467	0.011602	0.011626	0.011295
0.011530	0.011396	0.011262	0.011362	0.011161	0.011396	0.011195
0.011262	0.011262	0.011228	0.011194	0.011228	0.011195	0.011295
0.011270	0.011270	0.011266	0.011300	0.011437	0.011402	0.011402
0.011401	0.011329	0.011134	0.011132	0.011263	0.011433	0.011433

0.011228	0.011297	0.011531	0.011500	0.011430	0.011464	0.011396
0.011329	0.011396	0.011396	0.011295	0.011866	0.011463	0.011530
0.011564	0.011630	0.011265	0.011337	0.011228	0.011429	0.011396
0.011430	0.011463	0.011463	0.011463	0.011610	0.011597	0.011234
0.011128	0.011130	0.011230	0.011196	0.011299	0.011135	0.011468
0.011266	0.011367	0.011108	0.011248	0.011262	0.011334	0.011228
0.011195	0.011195	0.011262	0.011228	0.011429	0.011396	0.011329
0.011429	0.011497	0.011228	0.011228	0.011262	0.011262	0.011263
0.011331	0.011262	0.011262	0.011296	0.011429	0.011497	0.011497
0.011363	0.011466	0.011467	0.011602	0.011626	0.011295	0.011463
0.011396	0.011396	0.011229	0.011408	0.011338	0.011367	0.011235
0.011405	0.011607	0.011399	0.011298	0.011195	0.011396	0.011262
0.011362	0.011295	0.011195	0.011296	0.011396	0.011398	0.011295
0.011195	0.011128	0.011262	0.011195	0.011127	0.011230	0.011396
0.011295	0.011228	0.011262	0.011295	0.011262	0.011195	0.011396
0.011262	0.011295	0.011295	0.011228	0.011295	0.011530	0.011194
0.011228	0.011631	0.011228	0.011262	0.011228	0.011597	0.011146
0.011622	0.011145	0.011211	0.011422	0.011467	0.011419	0.011102
0.011165	0.011396	0.011496	0.011603	0.011464	0.011601	0.011733
0.011632	0.011669	0.011631	0.011880	0.011597	0.012472	0.011633
0.012372	0.012139	0.012273	0.012545	0.011597	0.011295	0.011598
0.012036	0.011971	0.011564	0.011463	0.011531	0.011531	0.011500
0.011396	0.011399	0.011463	0.011430	0.011800	0.011866	0.011867
0.011766	0.011867	0.011970	0.011737	0.012036	0.012042	0.012104
0.012104	0.012138	0.012084	0.012104	0.012105	0.011329	0.011631
0.011699	0.012202	0.011704	0.011933	0.012406	0.011468	0.011363
0.011329	0.011329	0.011799	0.011842	0.011940	0.011664	0.011330
S014	0.011631	0.011631	0.011900	0.011664	0.011664	0.011732
0.011535	0.011468	0.011731	0.011228	0.011295	0.011262	0.011228
0.000000	0.011429	0.011161	0.011262	0.011329	0.011295	0.011262
0.011228	0.011262	0.011295	0.011228	0.011262	0.011262	0.011501
0.011429	0.011262	0.011161	0.011329	0.011329	0.011295	0.011329
0.011295	0.011396	0.011295	0.011295	0.011329	0.011295	0.011463
0.011161	0.011295	0.011329	0.011262	0.011295	0.011396	0.011329
0.011295	0.011463	0.011362	0.011564	0.011295	0.011564	0.011295
0.011262	0.011295	0.011429	0.011564	0.011597	0.011295	0.011396
0.011329	0.011396	0.011295	0.011530	0.011698	0.011295	0.011362
0.011262	0.011362	0.011329	0.011396	0.011329	0.011329	0.011329
0.011228	0.011262	0.011228	0.011262	0.011295	0.011329	0.011396
0.011329	0.011295	0.011362	0.011262	0.011329	0.011228	0.011295
0.011463	0.011396	0.011631	0.011463	0.011295	0.011530	0.011295
0.011431	0.011583	0.011475	0.011177	0.011209	0.011318	0.011296
0.011975	0.011365	0.011184	0.011530	0.011530	0.011262	0.011362
0.011362	0.011329	0.011396	0.011295	0.011396	0.011329	0.011329
0.011295	0.011329	0.011597	0.011463	0.011362	0.011329	0.011262
0.011228	0.011161	0.011295	0.011262	0.011396	0.011362	0.011295
0.011396	0.011463	0.011262	0.011262	0.011295	0.011295	0.011297
0.011364	0.011295	0.011295	0.011168	0.011241	0.011200	0.011296
0.011297	0.011232	0.011282	0.011267	0.011196	0.011231	0.011263
0.011456	0.011301	0.011336	0.011228	0.011233	0.011363	0.011262
0.011329	0.011330	0.011329	0.011429	0.011362	0.011732	0.011463
0.011496	0.011362	0.011295	0.011329	0.011195	0.011362	0.011228
0.011295	0.011295	0.011262	0.011228	0.011262	0.011228	0.011329
0.011295	0.011396	0.011329	0.011228	0.011330	0.011430	0.011432
0.011430	0.011329	0.011228	0.011161	0.011295	0.011228	0.011161
0.011264	0.011430	0.011228	0.011430	0.011195	0.011295	0.011262
0.011362	0.011262	0.011295	0.011262	0.011295	0.011161	0.011295
0.011329	0.011262	0.011329	0.011262	0.011295	0.011329	0.011564
0.011597	0.011530	0.011597	0.011431	0.011598	0.011295	0.011262
0.011229	0.011505	0.011380	0.011197	0.011603	0.011163	0.011296

0.011569	0.011502	0.011463	0.011664	0.011367	0.011530	0.011497
0.011429	0.011597	0.011451	0.011307	0.011354	0.011329	0.011398
0.011497	0.011397	0.011533	0.011601	0.011530	0.011597	0.011704
0.011432	0.011603	0.011534	0.011466	0.011733	0.011194	0.011397
0.011362	0.011295	0.011194	0.011194	0.011262	0.011262	0.011329
0.011262	0.011329	0.011228	0.011262	0.011262	0.011262	0.011329
0.011262	0.011631	0.011530	0.011564	0.011161	0.011262	0.011295
0.011295	0.011262	0.011329	0.011295	0.011228	0.011228	0.011228
0.011698	0.011329	0.011396	0.011194	0.011295	0.011463	0.011228
0.011228	0.011295	0.011262	0.011429	0.011228	0.011362	0.011496
0.011429	0.011262	0.011262	0.011194	0.011194	0.011362	0.011295
0.011194	0.011295	0.011161	0.011194	0.011329	0.011396	0.011228
0.011195	0.011329	0.011262	0.011161	0.011429	0.011161	0.011329
0.011402	0.011228	0.011463	0.011362	0.011329	0.011262	0.011362
0.011228	0.011564	0.011161	0.011362	0.011161	0.011161	0.011161
0.011396	0.011530	0.011295	0.011362	0.011194	0.011530	0.011161
0.011228	0.011228	0.011262	0.011362	0.011262	0.011362	0.011429
0.011429	0.011396	0.011295	0.011396	0.011362	0.011228	0.011194
0.011228	0.011228	0.011295	0.011161	0.011194	0.011362	0.011228
0.011194	0.011262	0.011396	0.011362	0.011329	0.011262	0.011262
0.011329	0.011262	0.011362	0.011228	0.011362	0.011463	0.011698
0.011262	0.011597	0.011267	0.017785	0.011257	0.011366	0.011548
0.011510	0.011553	0.011322	0.011292	0.011664	0.011578	0.011364
0.011392	0.011487	0.011138	0.011228	0.011177	0.011258	0.011232
0.011389	0.011358	0.011357	0.011126	0.011396	0.011429	0.011432
0.011301	0.011396	0.011396	0.011295	0.011329	0.011329	0.011295
0.011329	0.011262	0.011295	0.011329	0.011295	0.011228	0.011262
0.011262	0.011329	0.011228	0.011329	0.011362	0.011396	0.011295
0.011262	0.011228	0.011564	0.011564	0.011362	0.011396	0.011430
0.011195	0.011195	0.011228	0.011329	0.011195	0.011429	0.011429
0.011463	0.011463	0.011396	0.011497	0.011597	0.011498	0.011329
0.011329	0.011396	0.011429	0.011262	0.011329	0.011295	0.011329
0.011396	0.011396	0.011429	0.011396	0.011296	0.011497	0.011463
0.011331	0.011228	0.011329	0.011430	0.011329	0.011396	0.011496
0.011664	0.011367	0.011530	0.011497	0.011429	0.011597	0.011228
0.011397	0.011295	0.011262	0.011262	0.011329	0.011228	0.011329
0.011362	0.011396	0.011295	0.011262	0.011228	0.011564	0.011564
0.011362	0.011396	0.011463	0.011396	0.011968	0.011453	0.011377
0.011447	0.011407	0.011517	0.011406	0.011515	0.011439	0.011405
0.011918	0.011302	0.011437	0.011371	0.011645	0.011502	0.011604
0.011944	0.011399	0.011567	0.011631	0.011496	0.011429	0.011533
0.011194	0.011362	0.011496	0.011463	0.011466	0.011396	0.011363
0.011295	0.011228	0.011262	0.011295	0.011194	0.011262	0.011161
0.011228	0.011262	0.011228	0.011194	0.011295	0.011228	0.011262
0.011262	0.011194	0.011262	0.011295	0.011194	0.011396	0.011429
0.011329	0.011329	0.011329	0.011329	0.011262	0.011597	0.011295
0.011269	0.011262	0.011430	0.011433	0.011362	0.011362	0.011362
0.011362	0.011295	0.011262	0.011295	0.011396	0.011396	0.011329
0.011329	0.011463	0.011564	0.011362	0.011564	0.011564	0.011429
0.011262	0.011329	0.011362	0.011698	0.011329	0.011362	0.011329
0.011362	0.011228	0.011228	0.011530	0.011530	0.011497	0.011295
0.011396	0.011463	0.011329	0.011396	0.011362	0.011228	0.011496
0.011329	0.011295	0.011228	0.011463	0.011295	0.011396	0.011631
0.011329	0.011603	0.011631	0.011385	0.011117	0.011332	0.011163
0.011170	0.011605	0.011236	0.011328	0.011158	0.011315	0.011273
0.011366	0.011192	0.011339	0.011192	0.011222	0.011395	0.011344
0.011362	0.011257	0.011307	0.011307	0.011330	0.011396	0.011463
0.011463	0.011329	0.011432	0.011433	0.011569	0.011590	0.011329
0.011496	0.011362	0.011295	0.011329	0.011195	0.011362	0.011228
0.011295	0.011295	0.011262	0.011228	0.011262	0.011228	0.011329

0.011305	0.011305	0.011300	0.011334	0.011471	0.011436	0.011436
0.011435	0.011363	0.011134	0.011132	0.011297	0.011467	0.011467
0.011262	0.011331	0.011565	0.011533	0.011464	0.011498	0.011429
0.011362	0.011429	0.011429	0.011329	0.011832	0.011496	0.011564
0.011597	0.011594	0.011299	0.011372	0.011262	0.011463	0.011429
0.011464	0.011496	0.011496	0.011496	0.011650	0.011631	0.011268
0.011162	0.011164	0.011264	0.011230	0.011265	0.011172	0.011434
0.011300	0.011367	0.011203	0.011285	0.011296	0.011368	0.011262
0.011228	0.011161	0.011295	0.011262	0.011396	0.011362	0.011295
0.011396	0.011463	0.011262	0.011262	0.011295	0.011295	0.011297
0.011364	0.011295	0.011295	0.011330	0.011396	0.011463	0.011463
0.011329	0.011432	0.011433	0.011569	0.011590	0.011329	0.011496
0.011429	0.011429	0.011263	0.011443	0.011373	0.011401	0.011270
0.011439	0.011607	0.011399	0.011332	0.011228	0.011430	0.011295
0.011396	0.011329	0.011228	0.011330	0.011430	0.011432	0.011329
0.011228	0.011161	0.011295	0.011228	0.011161	0.011264	0.011430
0.011329	0.011262	0.011295	0.011329	0.011295	0.011228	0.011362
0.011295	0.011329	0.011329	0.011262	0.011329	0.011564	0.011161
0.011262	0.011597	0.011262	0.011295	0.011262	0.011564	0.011189
0.011566	0.011188	0.011161	0.011382	0.011427	0.011383	0.011144
0.011113	0.011362	0.011463	0.011569	0.011430	0.011568	0.011699
0.011598	0.011635	0.011597	0.011846	0.011564	0.012438	0.011599
0.012338	0.012105	0.012240	0.012511	0.011564	0.011262	0.011564
0.012002	0.011937	0.011530	0.011430	0.011497	0.011497	0.011466
0.011362	0.011365	0.011429	0.011396	0.011766	0.011832	0.011834
0.011733	0.011833	0.011937	0.011703	0.012003	0.012008	0.012070
0.012070	0.012104	0.012050	0.012071	0.012072	0.011362	0.011598
0.011666	0.012169	0.011670	0.011900	0.012373	0.011434	0.011329
0.011295	0.011295	0.011766	0.011800	0.011904	0.011631	0.011296
S015	0.011799	0.011799	0.012135	0.011900	0.011900	0.011967
0.011807	0.011739	0.011899	0.011396	0.011463	0.011430	0.011396
0.011429	0.000000	0.011396	0.011429	0.011496	0.011463	0.011429
0.011396	0.011429	0.011530	0.011396	0.011430	0.011429	0.011773
0.011664	0.011429	0.011329	0.011564	0.011496	0.011463	0.011497
0.011463	0.011564	0.011463	0.011463	0.011496	0.011463	0.011631
0.011396	0.011463	0.011496	0.011429	0.011530	0.011564	0.011496
0.011463	0.011631	0.011530	0.011799	0.011463	0.011732	0.011463
0.011429	0.011530	0.011597	0.011732	0.011832	0.011530	0.011631
0.011496	0.011564	0.011463	0.011698	0.011933	0.011396	0.011530
0.011429	0.011530	0.011497	0.011564	0.011496	0.011496	0.011496
0.011396	0.011429	0.011396	0.011497	0.011463	0.011498	0.011564
0.011497	0.011463	0.011530	0.011429	0.011564	0.011396	0.011463
0.011631	0.011564	0.011799	0.011631	0.011463	0.011698	0.011396
0.011653	0.011882	0.011702	0.011412	0.011359	0.011502	0.011572
0.012195	0.011670	0.011390	0.011698	0.011698	0.011496	0.011597
0.011530	0.011496	0.011564	0.011463	0.011564	0.011496	0.011496
0.011463	0.011564	0.011765	0.011564	0.011530	0.011497	0.011430
0.011396	0.011396	0.011463	0.011429	0.011631	0.011597	0.011530
0.011631	0.011698	0.011429	0.011429	0.011463	0.011463	0.011465
0.011533	0.011463	0.011463	0.011348	0.011422	0.011376	0.011464
0.011467	0.011404	0.011466	0.011440	0.011366	0.011403	0.011432
0.011708	0.011474	0.011508	0.011396	0.011406	0.011531	0.011497
0.011565	0.011498	0.011497	0.011597	0.011530	0.011900	0.011698
0.011731	0.011597	0.011463	0.011564	0.011362	0.011597	0.011396
0.011463	0.011463	0.011429	0.011396	0.011429	0.011396	0.011497
0.011463	0.011564	0.011497	0.011396	0.011431	0.011598	0.011601
0.011598	0.011430	0.011396	0.011330	0.011463	0.011396	0.011329
0.011434	0.011598	0.011396	0.011598	0.011362	0.011463	0.011430
0.011530	0.011429	0.011463	0.011430	0.011463	0.011329	0.011463
0.011497	0.011429	0.011497	0.011429	0.011463	0.011497	0.011732

0.011833	0.011765	0.011833	0.011599	0.011833	0.011463	0.011429
0.011465	0.011747	0.011609	0.011369	0.011773	0.011333	0.011454
0.011841	0.011740	0.011631	0.011832	0.011538	0.011698	0.011665
0.011597	0.011765	0.011629	0.011472	0.011521	0.011497	0.011568
0.011665	0.011566	0.011703	0.011771	0.011698	0.011765	0.011873
0.011602	0.011773	0.011703	0.011635	0.011901	0.011362	0.011566
0.011496	0.011463	0.011362	0.011362	0.011429	0.011362	0.011496
0.011429	0.011497	0.011396	0.011496	0.011429	0.011429	0.011496
0.011429	0.011866	0.011765	0.011799	0.011329	0.011429	0.011463
0.011463	0.011429	0.011497	0.011530	0.011396	0.011396	0.011396
0.011933	0.011564	0.011564	0.011362	0.011463	0.011631	0.011396
0.011396	0.011463	0.011429	0.011597	0.011396	0.011530	0.011664
0.011597	0.011429	0.011429	0.011362	0.011362	0.011530	0.011463
0.011362	0.011463	0.011329	0.011362	0.011497	0.011631	0.011362
0.011363	0.011496	0.011430	0.011329	0.011664	0.011329	0.011429
0.011574	0.011396	0.011698	0.011530	0.011564	0.011429	0.011530
0.011396	0.011732	0.011329	0.011597	0.011396	0.011396	0.011329
0.011564	0.011766	0.011463	0.011530	0.011362	0.011765	0.011396
0.011396	0.011396	0.011429	0.011530	0.011429	0.011530	0.011597
0.011597	0.011564	0.011463	0.011564	0.011530	0.011396	0.011362
0.011396	0.011396	0.011463	0.011396	0.011362	0.011530	0.011396
0.011429	0.011429	0.011564	0.011597	0.011496	0.011429	0.011429
0.011496	0.011429	0.011530	0.011396	0.011530	0.011564	0.011933
0.011429	0.011832	0.011440	0.017899	0.011191	0.011549	0.011793
0.011752	0.011799	0.011479	0.011466	0.011829	0.011820	0.011425
0.011630	0.011671	0.011372	0.011437	0.011411	0.011406	0.011447
0.011717	0.011582	0.011579	0.011457	0.011564	0.011597	0.011601
0.011472	0.011564	0.011564	0.011530	0.011429	0.011430	0.011463
0.011497	0.011429	0.011463	0.011497	0.011463	0.011396	0.011429
0.011429	0.011497	0.011396	0.011497	0.011530	0.011564	0.011463
0.011496	0.011463	0.011799	0.011799	0.011530	0.011497	0.011598
0.011362	0.011362	0.011396	0.011497	0.011362	0.011597	0.011597
0.011631	0.011631	0.011564	0.011664	0.011833	0.011666	0.011497
0.011497	0.011564	0.011597	0.011430	0.011497	0.011463	0.011497
0.011564	0.011564	0.011597	0.011564	0.011464	0.011665	0.011631
0.011500	0.011396	0.011497	0.011598	0.011497	0.011631	0.011664
0.011832	0.011538	0.011698	0.011665	0.011597	0.011765	0.011396
0.011566	0.011396	0.011429	0.011429	0.011497	0.011396	0.011497
0.011530	0.011564	0.011463	0.011496	0.011463	0.011799	0.011799
0.011530	0.011497	0.011631	0.011564	0.012204	0.011596	0.011517
0.011587	0.011580	0.011693	0.011545	0.011690	0.011612	0.011577
0.012159	0.011475	0.011608	0.011544	0.011818	0.011672	0.011775
0.012182	0.011626	0.011736	0.011799	0.011664	0.011664	0.011702
0.011362	0.011530	0.011664	0.011631	0.011634	0.011597	0.011599
0.011463	0.011396	0.011429	0.011463	0.011362	0.011429	0.011329
0.011463	0.011496	0.011396	0.011362	0.011463	0.011396	0.011429
0.011429	0.011362	0.011429	0.011463	0.011362	0.011564	0.011597
0.011497	0.011564	0.011496	0.011497	0.011429	0.011832	0.011463
0.011478	0.011429	0.011665	0.011671	0.011530	0.011530	0.011530
0.011530	0.011463	0.011429	0.011463	0.011497	0.011564	0.011496
0.011496	0.011631	0.011799	0.011530	0.011732	0.011732	0.011597
0.011429	0.011496	0.011530	0.011933	0.011497	0.011530	0.011497
0.011530	0.011463	0.011463	0.011765	0.011698	0.011665	0.011463
0.011564	0.011631	0.011496	0.011564	0.011530	0.011396	0.011731
0.011496	0.011463	0.011463	0.011631	0.011463	0.011564	0.011799
0.011496	0.011875	0.011866	0.011670	0.011342	0.011570	0.011421
0.011335	0.011878	0.011411	0.011596	0.011451	0.011417	0.011485
0.011621	0.011126	0.011450	0.011258	0.011330	0.011462	0.011587
0.011588	0.011454	0.011553	0.011448	0.011498	0.011597	0.011698
0.011698	0.011565	0.011669	0.011670	0.011806	0.011838	0.011497

0.011731	0.011597	0.011463	0.011564	0.011362	0.011597	0.011396
0.011463	0.011463	0.011429	0.011396	0.011429	0.011396	0.011497
0.011479	0.011480	0.011471	0.011505	0.011642	0.011607	0.011606
0.011605	0.011531	0.011356	0.011348	0.011399	0.011636	0.011637
0.011430	0.011501	0.011734	0.011702	0.011632	0.011599	0.011597
0.011530	0.011597	0.011597	0.011497	0.012068	0.011664	0.011732
0.011766	0.011843	0.011469	0.011545	0.011429	0.011631	0.011597
0.011632	0.011664	0.011664	0.011664	0.011847	0.011799	0.011442
0.011297	0.011337	0.011435	0.011400	0.011470	0.011321	0.011672
0.011472	0.011630	0.011394	0.011472	0.011464	0.011539	0.011430
0.011396	0.011396	0.011463	0.011429	0.011631	0.011597	0.011530
0.011631	0.011698	0.011429	0.011429	0.011463	0.011463	0.011465
0.011533	0.011463	0.011463	0.011498	0.011597	0.011698	0.011698
0.011565	0.011669	0.011670	0.011806	0.011838	0.011497	0.011664
0.011597	0.011597	0.011432	0.011617	0.011442	0.011537	0.011446
0.011611	0.011881	0.011568	0.011502	0.011396	0.011598	0.011463
0.011564	0.011497	0.011396	0.011431	0.011598	0.011601	0.011430
0.011396	0.011330	0.011463	0.011396	0.011329	0.011434	0.011598
0.011497	0.011429	0.011463	0.011497	0.011463	0.011396	0.011597
0.011463	0.011497	0.011496	0.011429	0.011496	0.011732	0.011396
0.011429	0.011832	0.011429	0.011463	0.011429	0.011799	0.011403
0.011791	0.011358	0.011411	0.011624	0.011712	0.011634	0.011353
0.011375	0.011597	0.011698	0.011773	0.011666	0.011805	0.011935
0.011834	0.011872	0.011832	0.012086	0.011799	0.012674	0.011835
0.012574	0.012342	0.012476	0.012748	0.011799	0.011497	0.011799
0.012238	0.012173	0.011765	0.011665	0.011733	0.011733	0.011703
0.011597	0.011569	0.011664	0.011632	0.012002	0.012068	0.012069
0.011969	0.012069	0.012173	0.011940	0.012239	0.012246	0.012306
0.012307	0.012340	0.012289	0.012307	0.012308	0.011530	0.011833
0.011901	0.012404	0.011908	0.012135	0.012609	0.011671	0.011564
0.011530	0.011530	0.012001	0.012048	0.012151	0.011866	0.011532
S016	0.011597	0.011597	0.011866	0.011631	0.011631	0.011698
0.011535	0.011468	0.011698	0.011195	0.011262	0.011228	0.011194
0.011161	0.011396	0.000000	0.011228	0.011295	0.011262	0.011228
0.011194	0.011228	0.011262	0.011195	0.011228	0.011228	0.011501
0.011396	0.011228	0.011127	0.011295	0.011295	0.011262	0.011295
0.011262	0.011362	0.011262	0.011262	0.011295	0.011262	0.011429
0.011127	0.011262	0.011295	0.011228	0.011262	0.011362	0.011295
0.011262	0.011429	0.011329	0.011530	0.011262	0.011530	0.011262
0.011228	0.011262	0.011396	0.011530	0.011564	0.011262	0.011362
0.011295	0.011362	0.011262	0.011496	0.011664	0.011262	0.011329
0.011228	0.011329	0.011295	0.011362	0.011295	0.011295	0.011295
0.011194	0.011228	0.011194	0.011228	0.011262	0.011296	0.011362
0.011295	0.011262	0.011329	0.011228	0.011295	0.011194	0.011262
0.011429	0.011362	0.011597	0.011429	0.011262	0.011496	0.011262
0.011431	0.011583	0.011475	0.011177	0.011172	0.011281	0.011296
0.011938	0.011365	0.011184	0.011496	0.011496	0.011161	0.011329
0.011329	0.011295	0.011362	0.011262	0.011362	0.011295	0.011295
0.011262	0.011295	0.011564	0.011362	0.011329	0.011295	0.011228
0.011195	0.011127	0.011262	0.011228	0.011362	0.011329	0.011262
0.011362	0.011429	0.011228	0.011228	0.011262	0.011262	0.011263
0.011331	0.011262	0.011262	0.011132	0.011205	0.011165	0.011262
0.011263	0.011198	0.011245	0.011233	0.011162	0.011197	0.011229
0.011420	0.011267	0.011301	0.011195	0.011199	0.011329	0.011228
0.011296	0.011296	0.011296	0.011396	0.011329	0.011698	0.011430
0.011463	0.011329	0.011262	0.011295	0.011161	0.011329	0.011195
0.011262	0.011262	0.011228	0.011194	0.011228	0.011195	0.011295
0.011262	0.011362	0.011295	0.011195	0.011296	0.011396	0.011398
0.011396	0.011295	0.011195	0.011128	0.011262	0.011195	0.011127
0.011230	0.011396	0.011195	0.011396	0.011161	0.011262	0.011228

0.011329	0.011228	0.011262	0.011228	0.011262	0.011127	0.011262
0.011295	0.011228	0.011295	0.011228	0.011262	0.011295	0.011530
0.011564	0.011496	0.011564	0.011397	0.011564	0.011262	0.011228
0.011128	0.011464	0.011335	0.011163	0.011569	0.011129	0.011257
0.011569	0.011468	0.011429	0.011631	0.011333	0.011496	0.011463
0.011396	0.011564	0.011415	0.011266	0.011312	0.011295	0.011365
0.011463	0.011364	0.011500	0.011568	0.011496	0.011564	0.011670
0.011398	0.011569	0.011500	0.011432	0.011699	0.011161	0.011363
0.011329	0.011262	0.011161	0.011161	0.011228	0.011228	0.011295
0.011228	0.011295	0.011194	0.011228	0.011228	0.011228	0.011295
0.011228	0.011597	0.011496	0.011530	0.011127	0.011228	0.011262
0.011262	0.011228	0.011295	0.011262	0.011194	0.011194	0.011194
0.011664	0.011295	0.011362	0.011161	0.011262	0.011429	0.011194
0.011194	0.011262	0.011228	0.011396	0.011194	0.011329	0.011463
0.011396	0.011228	0.011228	0.011161	0.011161	0.011329	0.011262
0.011161	0.011262	0.011127	0.011161	0.011295	0.011362	0.011195
0.011161	0.011295	0.011228	0.011127	0.011396	0.011127	0.011295
0.011368	0.011194	0.011429	0.011329	0.011295	0.011228	0.011329
0.011194	0.011530	0.011127	0.011329	0.000000	0.000000	0.011127
0.011362	0.011497	0.011262	0.011329	0.011161	0.011496	0.000000
0.011194	0.011194	0.011228	0.011329	0.011228	0.011329	0.011396
0.011396	0.011362	0.011262	0.011362	0.011329	0.011194	0.011161
0.011194	0.011194	0.011262	0.011127	0.011161	0.011329	0.011194
0.011094	0.011228	0.011362	0.011329	0.011295	0.011228	0.011228
0.011295	0.011228	0.011329	0.011194	0.011329	0.011429	0.011664
0.011228	0.011564	0.011233	0.017747	0.011191	0.011366	0.011514
0.011475	0.011517	0.011322	0.011292	0.011609	0.011543	0.011303
0.011392	0.011487	0.011138	0.011228	0.011138	0.011258	0.011232
0.011389	0.011321	0.011320	0.000000	0.011362	0.011396	0.011398
0.011266	0.011362	0.011362	0.011262	0.011295	0.011295	0.011262
0.011295	0.011228	0.011262	0.011295	0.011262	0.011195	0.011228
0.011228	0.011295	0.011195	0.011295	0.011329	0.011362	0.011262
0.011228	0.011195	0.011530	0.011530	0.011329	0.011363	0.011396
0.011161	0.011161	0.011195	0.011295	0.011161	0.011396	0.011396
0.011429	0.011429	0.011362	0.011463	0.011564	0.011464	0.011295
0.011295	0.011362	0.011396	0.011228	0.011295	0.011262	0.011295
0.011363	0.011362	0.011396	0.011362	0.011262	0.011463	0.011429
0.011297	0.011195	0.011295	0.011396	0.011295	0.011362	0.011463
0.011631	0.011333	0.011496	0.011463	0.011396	0.011564	0.011195
0.011363	0.011262	0.011228	0.011228	0.011295	0.011195	0.011295
0.011329	0.011362	0.011262	0.011228	0.011195	0.011530	0.011530
0.011329	0.011363	0.011429	0.011362	0.011934	0.011417	0.011341
0.011412	0.011372	0.011482	0.011372	0.011480	0.011405	0.011370
0.011884	0.011268	0.011403	0.011337	0.011611	0.011468	0.011570
0.011910	0.011362	0.011533	0.011597	0.011463	0.011396	0.011499
0.011161	0.011329	0.011463	0.011429	0.011432	0.011362	0.011330
0.011262	0.011194	0.011228	0.011262	0.011161	0.011228	0.011127
0.011194	0.011228	0.011194	0.011161	0.011262	0.011194	0.011228
0.011228	0.011161	0.011228	0.011262	0.011161	0.011362	0.011396
0.011295	0.011295	0.011295	0.011295	0.011228	0.011564	0.011262
0.011234	0.011228	0.011396	0.011399	0.011329	0.011329	0.011329
0.011329	0.011262	0.011228	0.011262	0.011362	0.011362	0.011295
0.011295	0.011429	0.011530	0.011329	0.011530	0.011530	0.011396
0.011228	0.011295	0.011329	0.011664	0.011295	0.011329	0.011295
0.011329	0.011194	0.011194	0.011497	0.011497	0.011463	0.011262
0.011362	0.011429	0.011295	0.011362	0.011329	0.011194	0.011463
0.011295	0.011262	0.011194	0.011429	0.011262	0.011362	0.011597
0.011295	0.011603	0.011597	0.011385	0.000000	0.011298	0.011163
0.011170	0.011605	0.011201	0.011283	0.011109	0.011264	0.011220
0.011315	0.011126	0.011283	0.011126	0.011168	0.011328	0.011344

0.011362	0.011257	0.011201	0.011272	0.011296	0.011362	0.011429
0.011429	0.011296	0.011398	0.011365	0.011467	0.011555	0.011295
0.011463	0.011329	0.011262	0.011295	0.011161	0.011329	0.011195
0.011262	0.011262	0.011228	0.011194	0.011228	0.011195	0.011295
0.011270	0.011270	0.011266	0.011300	0.011436	0.011402	0.011402
0.011401	0.011329	0.011134	0.011132	0.011263	0.011433	0.011433
0.011228	0.011297	0.011531	0.011499	0.011430	0.011464	0.011396
0.011329	0.011396	0.011396	0.011295	0.011799	0.011463	0.011530
0.011564	0.011558	0.011265	0.011337	0.011228	0.011429	0.011396
0.011430	0.011463	0.011463	0.011463	0.011571	0.011597	0.011234
0.011128	0.011130	0.011230	0.011196	0.011231	0.011135	0.011400
0.011266	0.011323	0.011156	0.011285	0.011262	0.011334	0.011228
0.011195	0.011127	0.011262	0.011228	0.011362	0.011329	0.011262
0.011362	0.011429	0.011228	0.011228	0.011262	0.011262	0.011263
0.011331	0.011262	0.011262	0.011296	0.011362	0.011429	0.011429
0.011296	0.011398	0.011365	0.011467	0.011555	0.011295	0.011463
0.011396	0.011396	0.011229	0.011408	0.011338	0.011367	0.011235
0.011404	0.011561	0.011357	0.011298	0.011195	0.011396	0.011262
0.011362	0.011295	0.011195	0.011296	0.011396	0.011398	0.011295
0.011195	0.011128	0.011262	0.011195	0.011127	0.011230	0.011396
0.011295	0.011228	0.011262	0.011295	0.011262	0.011195	0.011329
0.011262	0.011295	0.011295	0.011228	0.011295	0.011530	0.011127
0.011228	0.011564	0.011228	0.011262	0.011228	0.011530	0.011189
0.011510	0.011188	0.011161	0.011342	0.011426	0.011347	0.011144
0.011113	0.011329	0.011429	0.011535	0.011397	0.011534	0.011665
0.011565	0.011601	0.011564	0.011812	0.011530	0.012404	0.011565
0.012304	0.012072	0.012206	0.012477	0.011530	0.011228	0.011530
0.011969	0.011903	0.011496	0.011396	0.011464	0.011464	0.011432
0.011329	0.011331	0.011396	0.011363	0.011733	0.011799	0.011800
0.011699	0.011800	0.011903	0.011669	0.011969	0.011974	0.012036
0.012037	0.012070	0.012015	0.012037	0.012038	0.011329	0.011564
0.011632	0.012135	0.011636	0.011866	0.012339	0.011400	0.011295
0.011262	0.011262	0.011732	0.011759	0.011869	0.011597	0.011262
S017 0.011631	0.011631	0.011967	0.011731	0.011731	0.011799	
0.011637	0.011569	0.011799	0.011228	0.011295	0.011262	0.011228
0.011262	0.011429	0.011228	0.000000	0.011329	0.011295	0.011262
0.011228	0.011262	0.011362	0.011228	0.011262	0.011262	0.011603
0.011496	0.011262	0.011161	0.011396	0.011329	0.011295	0.011329
0.011295	0.011396	0.011295	0.011295	0.011262	0.011295	0.011463
0.011228	0.011295	0.011262	0.011262	0.011362	0.011329	0.011329
0.011295	0.011396	0.011362	0.011631	0.011228	0.011564	0.011228
0.011262	0.011362	0.011429	0.011564	0.011664	0.011362	0.011463
0.011329	0.011396	0.011295	0.011530	0.011765	0.011295	0.011362
0.011262	0.011362	0.011329	0.011396	0.011329	0.011329	0.011329
0.011228	0.011262	0.011228	0.011329	0.011295	0.011329	0.011396
0.011329	0.011295	0.011362	0.011262	0.011396	0.011228	0.011295
0.011463	0.011396	0.011631	0.011463	0.011295	0.011530	0.011295
0.011468	0.011695	0.011475	0.011177	0.011172	0.011281	0.011414
0.012048	0.011416	0.011184	0.011463	0.011530	0.011329	0.011429
0.011362	0.011329	0.011396	0.011295	0.011396	0.011329	0.011329
0.011295	0.011396	0.011597	0.011463	0.011362	0.011329	0.011262
0.011228	0.011228	0.011295	0.011262	0.011463	0.011429	0.011362
0.011463	0.011530	0.011262	0.011262	0.011295	0.011295	0.011297
0.011364	0.011295	0.011295	0.011168	0.011241	0.011200	0.011296
0.011297	0.011232	0.011245	0.011267	0.011196	0.011231	0.011263
0.011528	0.011301	0.011336	0.011228	0.011233	0.011363	0.011329
0.011396	0.011330	0.011329	0.011429	0.011362	0.011664	0.011530
0.011564	0.011430	0.011295	0.011396	0.011195	0.011429	0.011228
0.011295	0.011295	0.011262	0.011228	0.011262	0.011228	0.011329
0.011295	0.011396	0.011329	0.011228	0.011330	0.011430	0.011432

0.011430	0.011329	0.011228	0.011161	0.011295	0.011228	0.011161
0.011264	0.011430	0.011228	0.011430	0.011195	0.011295	0.011262
0.011362	0.011262	0.011295	0.011262	0.011295	0.011161	0.011295
0.011329	0.011262	0.011329	0.011262	0.011295	0.011329	0.011564
0.011664	0.011597	0.011665	0.011431	0.011665	0.011295	0.011262
0.011297	0.011586	0.011380	0.011197	0.011671	0.011163	0.011218
0.011671	0.011570	0.011463	0.011664	0.011367	0.011530	0.011497
0.011362	0.011597	0.011380	0.011266	0.011312	0.011329	0.011331
0.011497	0.011397	0.011533	0.011601	0.011530	0.011597	0.011704
0.011432	0.011603	0.011466	0.011466	0.011699	0.011194	0.011397
0.011362	0.011295	0.011194	0.011194	0.011262	0.011262	0.011329
0.011262	0.011329	0.011228	0.011329	0.011262	0.011262	0.011262
0.011262	0.011698	0.011597	0.011631	0.011161	0.011262	0.011295
0.011295	0.011262	0.011329	0.011362	0.011228	0.011228	0.011228
0.011765	0.011396	0.011396	0.011194	0.011295	0.011396	0.011228
0.011228	0.011295	0.011262	0.011429	0.011228	0.011362	0.011496
0.011429	0.011262	0.011262	0.011194	0.011194	0.011362	0.011295
0.011194	0.011295	0.011161	0.011194	0.011329	0.011463	0.011228
0.011195	0.011329	0.011262	0.011161	0.011496	0.011161	0.011329
0.011402	0.011228	0.011530	0.011362	0.011396	0.011262	0.011295
0.011228	0.011564	0.011161	0.011429	0.011228	0.011228	0.011161
0.011396	0.011597	0.011295	0.011362	0.011194	0.011597	0.011228
0.011228	0.011228	0.011262	0.011362	0.011262	0.011362	0.011429
0.011429	0.011396	0.011295	0.011396	0.011362	0.011228	0.011194
0.011228	0.011228	0.011295	0.011228	0.011194	0.011362	0.011228
0.011262	0.011262	0.011396	0.011429	0.011329	0.011262	0.011262
0.011329	0.011262	0.011362	0.011228	0.011362	0.011463	0.011765
0.011262	0.011664	0.011267	0.017785	0.011191	0.011366	0.011618
0.011579	0.011623	0.011270	0.011234	0.011664	0.011647	0.011303
0.011392	0.011487	0.011177	0.011186	0.011255	0.011258	0.011189
0.011436	0.011433	0.011394	0.011259	0.011396	0.011429	0.011432
0.011301	0.011396	0.011396	0.011362	0.011329	0.011329	0.011295
0.011329	0.011262	0.011295	0.011329	0.011295	0.011228	0.011262
0.011262	0.011329	0.011228	0.011329	0.011362	0.011396	0.011295
0.011329	0.011295	0.011631	0.011631	0.011362	0.011329	0.011430
0.011195	0.011195	0.011228	0.011329	0.011195	0.011429	0.011429
0.011463	0.011463	0.011396	0.011497	0.011665	0.011498	0.011329
0.011329	0.011396	0.011429	0.011262	0.011329	0.011295	0.011262
0.011396	0.011396	0.011429	0.011396	0.011296	0.011497	0.011463
0.011331	0.011228	0.011329	0.011430	0.011329	0.011463	0.011496
0.011664	0.011367	0.011530	0.011497	0.011362	0.011597	0.011228
0.011296	0.011295	0.011262	0.011262	0.011329	0.011228	0.011329
0.011362	0.011396	0.011295	0.011329	0.011295	0.011631	0.011631
0.011362	0.011329	0.011463	0.011396	0.012035	0.011453	0.011377
0.011447	0.011407	0.011517	0.011406	0.011515	0.011439	0.011405
0.011987	0.011302	0.011437	0.011371	0.011645	0.011502	0.011604
0.012012	0.011475	0.011567	0.011631	0.011496	0.011496	0.011533
0.011194	0.011362	0.011496	0.011463	0.011466	0.011463	0.011431
0.011295	0.011228	0.011262	0.011295	0.011194	0.011262	0.011161
0.011295	0.011329	0.011228	0.011194	0.011295	0.011228	0.011262
0.011194	0.011194	0.011262	0.011295	0.011194	0.011396	0.011429
0.011329	0.011396	0.011329	0.011329	0.011262	0.011664	0.011295
0.011338	0.011262	0.011497	0.011501	0.011362	0.011362	0.011362
0.011362	0.011295	0.011262	0.011295	0.011396	0.011396	0.011329
0.011329	0.011463	0.011631	0.011362	0.011564	0.011564	0.011429
0.011262	0.011329	0.011362	0.011765	0.011329	0.011362	0.011329
0.011362	0.011295	0.011295	0.011597	0.011530	0.011564	0.011295
0.011396	0.011463	0.011329	0.011396	0.011362	0.011228	0.011564
0.011329	0.011295	0.011295	0.011463	0.011295	0.011396	0.011631
0.011329	0.011705	0.011698	0.011507	0.011229	0.011400	0.011267

0.011170	0.011683	0.011236	0.011417	0.011255	0.011315	0.011326
0.011417	0.011126	0.011283	0.011126	0.011222	0.011328	0.011385
0.011399	0.011296	0.011377	0.011307	0.011330	0.011463	0.011530
0.011530	0.011396	0.011500	0.011501	0.011636	0.011625	0.011329
0.011564	0.011430	0.011295	0.011396	0.011195	0.011429	0.011228
0.011295	0.011295	0.011262	0.011228	0.011262	0.011228	0.011329
0.011305	0.011305	0.011231	0.011334	0.011471	0.011436	0.011367
0.011435	0.011363	0.011134	0.011132	0.011297	0.011467	0.011467
0.011262	0.011331	0.011565	0.011533	0.011464	0.011498	0.011429
0.011362	0.011429	0.011429	0.011329	0.011900	0.011496	0.011564
0.011597	0.011665	0.011299	0.011372	0.011262	0.011463	0.011429
0.011464	0.011496	0.011496	0.011496	0.011571	0.011631	0.011234
0.011162	0.011164	0.011264	0.011230	0.011333	0.011135	0.011502
0.011300	0.011411	0.011108	0.011322	0.011296	0.011368	0.011262
0.011228	0.011228	0.011295	0.011262	0.011463	0.011429	0.011362
0.011463	0.011530	0.011262	0.011262	0.011295	0.011295	0.011297
0.011364	0.011295	0.011295	0.011330	0.011463	0.011530	0.011530
0.011396	0.011500	0.011501	0.011636	0.011625	0.011329	0.011496
0.011429	0.011429	0.011229	0.011443	0.011373	0.011401	0.011270
0.011439	0.011607	0.011399	0.011332	0.011228	0.011430	0.011295
0.011396	0.011329	0.011228	0.011330	0.011430	0.011432	0.011329
0.011228	0.011161	0.011295	0.011228	0.011161	0.011264	0.011430
0.011329	0.011262	0.011295	0.011329	0.011295	0.011228	0.011430
0.011295	0.011329	0.011329	0.011262	0.011329	0.011564	0.011228
0.011262	0.011664	0.011262	0.011295	0.011262	0.011631	0.011146
0.011566	0.011230	0.011211	0.011463	0.011549	0.011455	0.011102
0.011165	0.011429	0.011463	0.011569	0.011430	0.011568	0.011699
0.011598	0.011635	0.011597	0.011914	0.011631	0.012505	0.011666
0.012405	0.012173	0.012307	0.012578	0.011631	0.011329	0.011564
0.012070	0.011937	0.011597	0.011497	0.011564	0.011564	0.011534
0.011429	0.011433	0.011496	0.011464	0.011833	0.011900	0.011901
0.011800	0.011901	0.012004	0.011770	0.012070	0.012076	0.012137
0.012138	0.012172	0.012118	0.012138	0.012139	0.011362	0.011665
0.011733	0.012236	0.011738	0.011967	0.012440	0.011502	0.011396
0.011362	0.011362	0.011833	0.011883	0.011975	0.011698	0.011363
S018	0.011698	0.011698	0.012034	0.011799	0.011799	0.011866
0.011671	0.011603	0.011866	0.011295	0.011362	0.011262	0.011295
0.011329	0.011496	0.011295	0.011329	0.000000	0.011362	0.011329
0.011295	0.011329	0.011429	0.011295	0.011329	0.011329	0.011637
0.011564	0.011329	0.011228	0.011463	0.011396	0.011362	0.011396
0.011362	0.011463	0.011362	0.011362	0.011396	0.011362	0.011530
0.011295	0.011362	0.011396	0.011329	0.011429	0.011463	0.011396
0.011362	0.011530	0.011430	0.011698	0.011362	0.011631	0.011362
0.011329	0.011429	0.011496	0.011631	0.011732	0.011429	0.011530
0.011396	0.011463	0.011362	0.011597	0.011832	0.011362	0.011429
0.011329	0.011429	0.011396	0.011463	0.011396	0.011396	0.011396
0.011295	0.011329	0.011295	0.011396	0.011362	0.011397	0.011463
0.011396	0.011362	0.011429	0.011329	0.011463	0.011295	0.011362
0.011530	0.011463	0.011698	0.011530	0.011362	0.011597	0.011362
0.011468	0.011695	0.011513	0.011216	0.011247	0.011391	0.011414
0.012085	0.011467	0.011184	0.011597	0.011597	0.011396	0.011496
0.011429	0.011396	0.011463	0.011362	0.011463	0.011396	0.011396
0.011362	0.011463	0.011664	0.011530	0.011429	0.011396	0.011329
0.011295	0.011295	0.011362	0.011329	0.011530	0.011497	0.011429
0.011530	0.011597	0.011329	0.011329	0.011362	0.011362	0.011364
0.011432	0.011362	0.011362	0.011240	0.011313	0.011271	0.011363
0.011365	0.011301	0.011355	0.011336	0.011264	0.011300	0.011330
0.011600	0.011370	0.011405	0.011296	0.011302	0.011430	0.011396
0.011464	0.011397	0.011396	0.011496	0.011429	0.011799	0.011598
0.011631	0.011497	0.011362	0.011463	0.011262	0.011496	0.011295

0.011362	0.011362	0.011329	0.011295	0.011329	0.011295	0.011396
0.011362	0.011463	0.011396	0.011295	0.011363	0.011497	0.011499
0.011497	0.011396	0.011295	0.011229	0.011362	0.011295	0.011228
0.011332	0.011497	0.011295	0.011497	0.011262	0.011362	0.011329
0.011430	0.011329	0.011362	0.011329	0.011362	0.011228	0.011362
0.011396	0.011329	0.011396	0.011329	0.011362	0.011396	0.011631
0.011732	0.011664	0.011732	0.011498	0.011732	0.011362	0.011329
0.011364	0.011667	0.011517	0.011266	0.011705	0.011231	0.011375
0.011705	0.011638	0.011530	0.011732	0.011436	0.011597	0.011564
0.011496	0.011664	0.011486	0.011349	0.011395	0.011396	0.011466
0.011564	0.011465	0.011601	0.011669	0.011597	0.011665	0.011772
0.011466	0.011671	0.011602	0.011534	0.011800	0.011262	0.011464
0.011429	0.011362	0.011262	0.011262	0.011329	0.011329	0.011396
0.011329	0.011396	0.011295	0.011396	0.011329	0.011329	0.011396
0.011329	0.011765	0.011664	0.011698	0.011228	0.011329	0.011362
0.011362	0.011329	0.011396	0.011429	0.011295	0.011295	0.011295
0.011832	0.011463	0.011463	0.011262	0.011362	0.011530	0.011295
0.011295	0.011362	0.011329	0.011497	0.011295	0.011429	0.011564
0.011496	0.011329	0.011329	0.011262	0.011262	0.011429	0.011362
0.011262	0.011362	0.011228	0.011262	0.011396	0.011530	0.011295
0.011262	0.011396	0.011329	0.011228	0.011564	0.011228	0.011329
0.011471	0.011295	0.011597	0.011429	0.011463	0.011329	0.011429
0.011295	0.011631	0.011228	0.011496	0.011295	0.011295	0.011228
0.011463	0.011665	0.011362	0.011429	0.011262	0.011664	0.011295
0.011295	0.011295	0.011329	0.011429	0.011329	0.011429	0.011496
0.011496	0.011463	0.011362	0.011463	0.011429	0.011295	0.011262
0.011295	0.011295	0.011362	0.011295	0.011262	0.011429	0.011295
0.011329	0.011329	0.011463	0.011496	0.011396	0.011329	0.011329
0.011396	0.011329	0.011429	0.011295	0.011429	0.011530	0.011832
0.011329	0.011732	0.011336	0.017785	0.011126	0.011488	0.011688
0.011648	0.011693	0.011322	0.011292	0.011719	0.011716	0.011364
0.011487	0.011548	0.011177	0.011270	0.011255	0.011307	0.011275
0.011482	0.011470	0.011431	0.011259	0.011463	0.011497	0.011500
0.011369	0.011463	0.011463	0.011429	0.011396	0.011396	0.011362
0.011396	0.011329	0.011362	0.011396	0.011362	0.011295	0.011329
0.011329	0.011396	0.011295	0.011396	0.011430	0.011396	0.011362
0.011396	0.011362	0.011698	0.011698	0.011430	0.011464	0.011497
0.011262	0.011262	0.011295	0.011396	0.011262	0.011497	0.011497
0.011530	0.011530	0.011463	0.011564	0.011732	0.011565	0.011396
0.011396	0.011463	0.011497	0.011329	0.011396	0.011362	0.011396
0.011463	0.011463	0.011497	0.011463	0.011363	0.011564	0.011530
0.011331	0.011295	0.011329	0.011497	0.011396	0.011530	0.011496
0.011732	0.011436	0.011597	0.011564	0.011496	0.011664	0.011295
0.011464	0.011363	0.011329	0.011329	0.011396	0.011295	0.011396
0.011430	0.011396	0.011362	0.011396	0.011362	0.011698	0.011698
0.011430	0.011464	0.011530	0.011463	0.012103	0.011524	0.011412
0.011517	0.011476	0.011482	0.011476	0.011585	0.011508	0.011474
0.012021	0.011372	0.011505	0.011441	0.011714	0.011570	0.011673
0.012080	0.011437	0.011635	0.011698	0.011564	0.011564	0.011601
0.011262	0.011429	0.011564	0.011530	0.011533	0.011530	0.011498
0.011362	0.011295	0.011329	0.011362	0.011262	0.011329	0.011228
0.011362	0.011396	0.011295	0.011262	0.011362	0.011295	0.011329
0.011329	0.011262	0.011329	0.011362	0.011262	0.011463	0.011496
0.011396	0.011463	0.011396	0.011396	0.011329	0.011732	0.011362
0.011408	0.011329	0.011564	0.011569	0.011429	0.011429	0.011429
0.011429	0.011362	0.011329	0.011362	0.011463	0.011463	0.011396
0.011329	0.011530	0.011698	0.011429	0.011631	0.011631	0.011496
0.011329	0.011396	0.011429	0.011832	0.011396	0.011429	0.011396
0.011429	0.011362	0.011362	0.011665	0.011597	0.011631	0.011362
0.011463	0.011530	0.011396	0.011463	0.011429	0.011295	0.011631

0.011396	0.011362	0.011362	0.011530	0.011362	0.011463	0.011698
0.011396	0.011739	0.011765	0.011507	0.011173	0.011468	0.011267
0.011280	0.011722	0.011306	0.011417	0.011255	0.011366	0.011379
0.011468	0.000000	0.011338	0.011192	0.011222	0.011328	0.011385
0.011399	0.011296	0.011448	0.011377	0.011397	0.011530	0.011597
0.011597	0.011464	0.011567	0.011569	0.011704	0.011732	0.011396
0.011631	0.011497	0.011362	0.011463	0.011262	0.011496	0.011295
0.011362	0.011362	0.011329	0.011295	0.011329	0.011295	0.011396
0.011375	0.011375	0.011368	0.011403	0.011539	0.011504	0.011504
0.011503	0.011430	0.011208	0.011204	0.011365	0.011535	0.011535
0.011329	0.011331	0.011632	0.011601	0.011531	0.011565	0.011496
0.011429	0.011496	0.011496	0.011396	0.011967	0.011564	0.011631
0.011665	0.011701	0.011367	0.011441	0.011329	0.011530	0.011496
0.011531	0.011564	0.011564	0.011564	0.011650	0.011698	0.011303
0.011229	0.011233	0.011332	0.011298	0.011402	0.011172	0.011570
0.011335	0.011455	0.011203	0.011322	0.011363	0.011437	0.011329
0.011295	0.011295	0.011362	0.011329	0.011530	0.011497	0.011429
0.011530	0.011597	0.011329	0.011329	0.011362	0.011362	0.011364
0.011432	0.011362	0.011362	0.011397	0.011530	0.011597	0.011597
0.011464	0.011567	0.011569	0.011704	0.011732	0.011396	0.011496
0.011496	0.011496	0.011331	0.011478	0.011442	0.011469	0.011340
0.011508	0.011744	0.011526	0.011400	0.011295	0.011497	0.011362
0.011463	0.011396	0.011295	0.011363	0.011497	0.011499	0.011396
0.011295	0.011229	0.011362	0.011295	0.011228	0.011332	0.011497
0.011396	0.011329	0.011362	0.011396	0.011362	0.011295	0.011497
0.011362	0.011396	0.011396	0.011329	0.011396	0.011631	0.011295
0.011329	0.011732	0.011329	0.011362	0.011329	0.011698	0.011189
0.011622	0.011230	0.011261	0.011503	0.011549	0.011491	0.011186
0.011218	0.011496	0.011597	0.011705	0.011565	0.011703	0.011834
0.011733	0.011770	0.011731	0.011983	0.011698	0.012573	0.011734
0.012473	0.012241	0.012375	0.012646	0.011698	0.011396	0.011698
0.012137	0.012072	0.011664	0.011564	0.011632	0.011632	0.011601
0.011496	0.011501	0.011564	0.011531	0.011901	0.011900	0.011968
0.011867	0.011968	0.012072	0.011838	0.012138	0.012144	0.012205
0.012205	0.012239	0.012186	0.012205	0.012207	0.011429	0.011732
0.011800	0.012303	0.011806	0.012034	0.012508	0.011570	0.011464
0.011429	0.011429	0.011900	0.011924	0.012045	0.011765	0.011431
S019	0.011664	0.011664	0.012001	0.011765	0.011765	0.011832
0.011637	0.011569	0.011832	0.011262	0.011329	0.011296	0.011262
0.011295	0.011463	0.011262	0.011295	0.011362	0.000000	0.011295
0.011262	0.011094	0.011396	0.011262	0.011295	0.011295	0.011603
0.011530	0.011295	0.011194	0.011429	0.011362	0.011329	0.011362
0.011329	0.011429	0.011329	0.011329	0.011362	0.011329	0.011497
0.011262	0.011329	0.011362	0.011295	0.011396	0.011429	0.011362
0.011329	0.011497	0.011396	0.011664	0.011329	0.011597	0.011329
0.011295	0.011396	0.011463	0.011597	0.011698	0.011396	0.011496
0.011362	0.011429	0.011329	0.011564	0.011799	0.011329	0.011396
0.011295	0.011396	0.011362	0.011429	0.011362	0.011362	0.011362
0.011262	0.011295	0.011262	0.011362	0.011329	0.011363	0.011429
0.011362	0.011329	0.011396	0.011295	0.011429	0.011262	0.011329
0.011496	0.011429	0.011664	0.011497	0.011329	0.011564	0.011329
0.011468	0.011695	0.011513	0.011216	0.011247	0.011355	0.011415
0.012085	0.011467	0.011225	0.011564	0.011564	0.011362	0.011463
0.011396	0.011362	0.011429	0.011329	0.011429	0.011362	0.011362
0.011329	0.011429	0.011631	0.011496	0.011396	0.011362	0.011295
0.011262	0.011262	0.011329	0.011295	0.011497	0.011463	0.011396
0.011497	0.011564	0.011295	0.011295	0.011329	0.011329	0.011330
0.011398	0.011329	0.011329	0.011204	0.011277	0.011236	0.011329
0.011331	0.011266	0.011318	0.011302	0.011230	0.011266	0.011297
0.011564	0.011336	0.011370	0.011262	0.011268	0.011397	0.011363

0.011430	0.011364	0.011363	0.011463	0.011396	0.011765	0.011564
0.011597	0.011463	0.011329	0.011430	0.011228	0.011463	0.011262
0.011329	0.011329	0.011295	0.011262	0.011295	0.011262	0.011362
0.011329	0.011430	0.011362	0.011262	0.011363	0.011463	0.011466
0.011463	0.011362	0.011262	0.011195	0.011329	0.011262	0.011195
0.011298	0.011463	0.011262	0.011463	0.011228	0.011329	0.011295
0.011396	0.011295	0.011329	0.011295	0.011329	0.011195	0.011329
0.011362	0.011295	0.011362	0.011295	0.011329	0.011362	0.011597
0.011698	0.011631	0.011698	0.011465	0.011698	0.011329	0.011295
0.011330	0.011586	0.011335	0.011232	0.011705	0.011197	0.011296
0.011671	0.011604	0.011496	0.011698	0.011402	0.011564	0.011530
0.011463	0.011631	0.011486	0.011349	0.011395	0.011362	0.011432
0.011530	0.011431	0.011567	0.011635	0.011564	0.011631	0.011738
0.011466	0.011637	0.011568	0.011500	0.011767	0.011228	0.011431
0.011396	0.011329	0.011228	0.011228	0.011295	0.011295	0.011362
0.011295	0.011362	0.011262	0.011362	0.011295	0.011295	0.011362
0.011094	0.011731	0.011631	0.011664	0.011194	0.011295	0.011329
0.011329	0.011295	0.011362	0.011396	0.011262	0.011262	0.011262
0.011799	0.011429	0.011429	0.011228	0.011329	0.011497	0.011262
0.011262	0.011329	0.011295	0.011463	0.011262	0.011396	0.011530
0.011463	0.011295	0.011295	0.011228	0.011228	0.011396	0.011329
0.011228	0.011329	0.011195	0.011228	0.011362	0.011497	0.011262
0.011228	0.011362	0.011295	0.011195	0.011530	0.011195	0.011362
0.011437	0.011262	0.011564	0.011396	0.011429	0.011295	0.011396
0.011262	0.011597	0.011194	0.011463	0.011262	0.011262	0.011194
0.011429	0.011631	0.011329	0.011396	0.011228	0.011631	0.011262
0.011262	0.011262	0.011295	0.011396	0.011295	0.011396	0.011463
0.011463	0.011429	0.011329	0.011429	0.011396	0.011262	0.011228
0.011262	0.011262	0.011329	0.011262	0.011228	0.011396	0.011262
0.011295	0.011295	0.011429	0.011463	0.011362	0.011295	0.011295
0.011362	0.011295	0.011396	0.011262	0.011396	0.011496	0.011799
0.011295	0.011698	0.011302	0.017823	0.011191	0.011488	0.011653
0.011614	0.011658	0.011374	0.011350	0.011774	0.011682	0.011425
0.011534	0.011610	0.011177	0.011270	0.011294	0.011356	0.011275
0.011482	0.011470	0.011468	0.011193	0.011429	0.011463	0.011466
0.011335	0.011429	0.011429	0.011396	0.011362	0.011362	0.011329
0.011362	0.011295	0.011329	0.011362	0.011329	0.011262	0.011295
0.011295	0.011362	0.011262	0.011362	0.011396	0.011429	0.011329
0.011362	0.011329	0.011665	0.011665	0.011396	0.011430	0.011463
0.011228	0.011228	0.011262	0.011362	0.011228	0.011463	0.011463
0.011497	0.011496	0.011429	0.011530	0.011698	0.011532	0.011362
0.011362	0.011430	0.011463	0.011295	0.011362	0.011329	0.011362
0.011430	0.011430	0.011463	0.011430	0.011330	0.011530	0.011497
0.011365	0.011262	0.011362	0.011463	0.011362	0.011497	0.011530
0.011698	0.011402	0.011564	0.011530	0.011463	0.011631	0.011262
0.011431	0.011329	0.011295	0.011295	0.011362	0.011262	0.011362
0.011396	0.011429	0.011329	0.011362	0.011329	0.011665	0.011665
0.011396	0.011430	0.011496	0.011429	0.012069	0.011489	0.011412
0.011482	0.011442	0.011552	0.011441	0.011550	0.011474	0.011439
0.012022	0.011337	0.011471	0.011406	0.011680	0.011536	0.011639
0.012046	0.011512	0.011601	0.011664	0.011530	0.011530	0.011567
0.011228	0.011396	0.011530	0.011496	0.011499	0.011496	0.011464
0.011329	0.011262	0.011295	0.011329	0.011228	0.011295	0.011194
0.011329	0.011362	0.011262	0.011228	0.011329	0.011262	0.011295
0.011295	0.011228	0.011295	0.011329	0.011228	0.011429	0.011463
0.011362	0.011429	0.011362	0.011362	0.011295	0.011698	0.011329
0.011373	0.011295	0.011530	0.011535	0.011396	0.011396	0.011396
0.011396	0.011329	0.011295	0.011329	0.011429	0.011429	0.011362
0.011362	0.011497	0.011664	0.011396	0.011597	0.011597	0.011463
0.011295	0.011362	0.011396	0.011799	0.011362	0.011396	0.011362

0.011396	0.011329	0.011329	0.011631	0.011564	0.011597	0.011329
0.011429	0.011497	0.011362	0.011429	0.011396	0.011262	0.011597
0.011362	0.011329	0.011329	0.011497	0.011329	0.011429	0.011664
0.011362	0.011705	0.011732	0.011507	0.011229	0.011434	0.011318
0.011280	0.011722	0.011271	0.011462	0.011304	0.011417	0.011432
0.011468	0.011192	0.011394	0.011192	0.011276	0.011395	0.011385
0.011399	0.011296	0.011412	0.011342	0.011364	0.011497	0.011564
0.011564	0.011430	0.011533	0.011535	0.011670	0.011696	0.011362
0.011597	0.011463	0.011329	0.011430	0.011228	0.011463	0.011262
0.011329	0.011329	0.011295	0.011262	0.011295	0.011262	0.011362
0.011340	0.011340	0.011334	0.011368	0.011505	0.011470	0.011470
0.011469	0.011396	0.011208	0.011204	0.011331	0.011501	0.011501
0.011296	0.011365	0.011599	0.011567	0.011497	0.011531	0.011463
0.011396	0.011463	0.011463	0.011362	0.011933	0.011530	0.011597
0.011631	0.011701	0.011333	0.011372	0.011295	0.011497	0.011463
0.011497	0.011530	0.011530	0.011530	0.011689	0.011664	0.011303
0.011195	0.011199	0.011298	0.011264	0.011368	0.011209	0.011536
0.011335	0.011455	0.011203	0.011322	0.011329	0.011402	0.011295
0.011262	0.011262	0.011329	0.011295	0.011497	0.011463	0.011396
0.011497	0.011564	0.011295	0.011295	0.011329	0.011329	0.011330
0.011398	0.011329	0.011329	0.011364	0.011497	0.011564	0.011564
0.011430	0.011533	0.011535	0.011670	0.011696	0.011362	0.011530
0.011463	0.011463	0.011297	0.011478	0.011408	0.011435	0.011305
0.011473	0.011698	0.011399	0.011366	0.011262	0.011463	0.011329
0.011430	0.011362	0.011262	0.011363	0.011463	0.011466	0.011362
0.011262	0.011195	0.011329	0.011262	0.011195	0.011298	0.011463
0.011362	0.011295	0.011329	0.011362	0.011329	0.011262	0.011463
0.011329	0.011362	0.011362	0.011295	0.011362	0.011597	0.011262
0.011295	0.011698	0.011295	0.011329	0.011295	0.011664	0.011232
0.011679	0.011230	0.011261	0.011503	0.011549	0.011491	0.011186
0.011218	0.011463	0.011564	0.011671	0.011531	0.011669	0.011800
0.011699	0.011737	0.011698	0.011949	0.011664	0.012539	0.011700
0.012439	0.012207	0.012341	0.012613	0.011664	0.011362	0.011665
0.012103	0.012038	0.011631	0.011530	0.011598	0.011598	0.011567
0.011463	0.011467	0.011530	0.011497	0.011867	0.011866	0.011935
0.011766	0.011867	0.012038	0.011804	0.012104	0.012110	0.012171
0.012172	0.012205	0.012152	0.012172	0.012173	0.011396	0.011699
0.011767	0.012270	0.011772	0.012001	0.012474	0.011536	0.011430
0.011396	0.011396	0.011867	0.011883	0.012010	0.011732	0.011397
S020 0.011631	0.011631	0.011967	0.011731	0.011731	0.011799	
0.011637	0.011569	0.011799	0.011228	0.011295	0.011262	0.011228
0.011262	0.011429	0.011228	0.011262	0.011329	0.011295	0.000000
0.011228	0.011262	0.011362	0.011228	0.011262	0.011262	0.011603
0.011496	0.011262	0.011161	0.011396	0.011329	0.011295	0.011329
0.011295	0.011396	0.011295	0.011295	0.011329	0.011295	0.011463
0.011228	0.011295	0.011329	0.011262	0.011362	0.011396	0.011329
0.011295	0.011463	0.011362	0.011631	0.011295	0.011564	0.011295
0.011262	0.011362	0.011429	0.011564	0.011664	0.011362	0.011463
0.011329	0.011396	0.011295	0.011530	0.011765	0.011295	0.011362
0.011262	0.011362	0.011329	0.011396	0.011329	0.011329	0.011329
0.011228	0.011262	0.011228	0.011329	0.011295	0.011329	0.011396
0.011329	0.011295	0.011362	0.011262	0.011396	0.011228	0.011295
0.011463	0.011396	0.011631	0.011463	0.011295	0.011530	0.011295
0.011431	0.011658	0.011475	0.011177	0.011172	0.011318	0.011375
0.012011	0.011467	0.011102	0.011530	0.011530	0.011329	0.011429
0.011362	0.011329	0.011396	0.011295	0.011396	0.011329	0.011329
0.011295	0.011396	0.011597	0.011463	0.011362	0.011329	0.011262
0.011228	0.011228	0.011295	0.011262	0.011463	0.011429	0.011362
0.011463	0.011530	0.011262	0.011262	0.011295	0.011295	0.011297
0.011364	0.011295	0.011295	0.011168	0.011241	0.011200	0.011296

0.011297	0.011232	0.011245	0.011267	0.011196	0.011231	0.011263
0.011528	0.011301	0.011336	0.011161	0.011233	0.011363	0.011329
0.011396	0.011330	0.011329	0.011429	0.011362	0.011732	0.011530
0.011564	0.011430	0.011295	0.011396	0.011195	0.011429	0.011228
0.011295	0.011295	0.011262	0.011228	0.011262	0.011228	0.011329
0.011295	0.011396	0.011329	0.011228	0.011330	0.011430	0.011432
0.011430	0.011329	0.011228	0.011161	0.011295	0.011228	0.011161
0.011196	0.011430	0.011228	0.011430	0.011195	0.011295	0.011262
0.011362	0.011262	0.011295	0.011262	0.011295	0.011161	0.011295
0.011329	0.011195	0.011329	0.011262	0.011295	0.011329	0.011564
0.011664	0.011597	0.011665	0.011431	0.011665	0.011295	0.011195
0.011297	0.011586	0.011426	0.011197	0.011671	0.011163	0.011257
0.011671	0.011570	0.011463	0.011664	0.011367	0.011530	0.011497
0.011429	0.011597	0.011451	0.011307	0.011270	0.011262	0.011398
0.011497	0.011397	0.011533	0.011601	0.011530	0.011597	0.011704
0.011432	0.011603	0.011534	0.011466	0.011733	0.011194	0.011397
0.011362	0.011295	0.011194	0.011194	0.011262	0.011262	0.011329
0.011262	0.011329	0.011228	0.011329	0.011262	0.011194	0.011329
0.011262	0.011698	0.011597	0.011631	0.011161	0.011262	0.011295
0.011295	0.011262	0.011329	0.011362	0.011228	0.011228	0.011228
0.011765	0.011396	0.011396	0.011194	0.011295	0.011463	0.011228
0.011228	0.011295	0.011262	0.011429	0.011228	0.011362	0.011496
0.011429	0.011262	0.011194	0.011194	0.011194	0.011362	0.011295
0.011194	0.011295	0.011161	0.011194	0.011329	0.011463	0.011228
0.011195	0.011329	0.011262	0.011161	0.011496	0.011161	0.011329
0.011402	0.011228	0.011530	0.011362	0.011396	0.011262	0.011362
0.011228	0.011564	0.011161	0.011429	0.011228	0.011228	0.011161
0.011396	0.011597	0.011295	0.011362	0.011194	0.011597	0.011228
0.011228	0.011161	0.011262	0.011362	0.011262	0.011362	0.011429
0.011429	0.011396	0.011295	0.011396	0.011362	0.011228	0.011194
0.011228	0.011228	0.011295	0.011228	0.011194	0.011362	0.011228
0.011262	0.011262	0.011396	0.011429	0.011329	0.011262	0.011262
0.011329	0.011262	0.011362	0.011228	0.011362	0.011463	0.011765
0.011262	0.011664	0.011267	0.017747	0.011257	0.011488	0.011618
0.011579	0.011623	0.011322	0.011292	0.011719	0.011647	0.011364
0.011487	0.011548	0.011138	0.011270	0.011216	0.011307	0.011232
0.011482	0.011395	0.011394	0.011126	0.011396	0.011429	0.011432
0.011301	0.011396	0.011396	0.011362	0.011329	0.011329	0.011295
0.011329	0.011262	0.011295	0.011329	0.011295	0.011228	0.011262
0.011262	0.011329	0.011228	0.011329	0.011362	0.011396	0.011295
0.011329	0.011295	0.011631	0.011631	0.011362	0.011396	0.011430
0.011195	0.011195	0.011228	0.011329	0.011195	0.011429	0.011429
0.011463	0.011463	0.011396	0.011497	0.011665	0.011498	0.011329
0.011329	0.011396	0.011429	0.011262	0.011329	0.011295	0.011329
0.011396	0.011396	0.011429	0.011396	0.011296	0.011497	0.011463
0.011331	0.011228	0.011329	0.011430	0.011329	0.011463	0.011496
0.011664	0.011367	0.011530	0.011497	0.011429	0.011597	0.011228
0.011397	0.011295	0.011262	0.011262	0.011329	0.011228	0.011329
0.011362	0.011396	0.011295	0.011329	0.011295	0.011631	0.011631
0.011362	0.011396	0.011463	0.011396	0.012035	0.011453	0.011377
0.011447	0.011407	0.011517	0.011406	0.011515	0.011439	0.011405
0.011987	0.011302	0.011437	0.011371	0.011645	0.011502	0.011604
0.012012	0.011437	0.011567	0.011631	0.011496	0.011496	0.011533
0.011194	0.011362	0.011496	0.011463	0.011466	0.011463	0.011431
0.011295	0.011161	0.011262	0.011295	0.011194	0.011262	0.011161
0.011295	0.011329	0.011228	0.011194	0.011295	0.011228	0.011262
0.011262	0.011194	0.011262	0.011295	0.011194	0.011396	0.011429
0.011329	0.011396	0.011329	0.011329	0.011262	0.011664	0.011295
0.011338	0.011262	0.011497	0.011501	0.011362	0.011362	0.011362
0.011362	0.011295	0.011262	0.011295	0.011396	0.011396	0.011329

0.011329	0.011463	0.011631	0.011362	0.011564	0.011564	0.011429
0.011262	0.011329	0.011362	0.011765	0.011329	0.011362	0.011329
0.011362	0.011295	0.011295	0.011597	0.011530	0.011564	0.011295
0.011396	0.011463	0.011329	0.011396	0.011362	0.011228	0.011564
0.011329	0.011295	0.011295	0.011463	0.011295	0.011396	0.011631
0.011329	0.011705	0.011698	0.011467	0.011229	0.011400	0.011267
0.011280	0.011683	0.011236	0.011417	0.011255	0.011366	0.011379
0.011468	0.011192	0.011339	0.011192	0.011222	0.011395	0.011344
0.011362	0.011257	0.011377	0.011307	0.011330	0.011463	0.011530
0.011530	0.011396	0.011500	0.011501	0.011569	0.011661	0.011329
0.011564	0.011430	0.011295	0.011396	0.011195	0.011429	0.011228
0.011295	0.011295	0.011262	0.011228	0.011262	0.011228	0.011329
0.011305	0.011305	0.011231	0.011334	0.011471	0.011436	0.011436
0.011435	0.011363	0.011171	0.011168	0.011297	0.011467	0.011467
0.011262	0.011331	0.011565	0.011533	0.011464	0.011498	0.011429
0.011362	0.011429	0.011429	0.011329	0.011900	0.011496	0.011564
0.011597	0.011665	0.011265	0.011372	0.011262	0.011463	0.011429
0.011464	0.011496	0.011496	0.011496	0.011571	0.011631	0.011268
0.011162	0.011164	0.011264	0.011230	0.011333	0.011098	0.011502
0.011232	0.011367	0.011108	0.011248	0.011296	0.011368	0.011262
0.011228	0.011228	0.011295	0.011262	0.011463	0.011429	0.011362
0.011463	0.011530	0.011262	0.011262	0.011295	0.011295	0.011297
0.011364	0.011295	0.011295	0.011330	0.011463	0.011530	0.011530
0.011396	0.011500	0.011501	0.011569	0.011661	0.011329	0.011496
0.011429	0.011429	0.011263	0.011443	0.011373	0.011401	0.011270
0.011439	0.011652	0.011441	0.011332	0.011228	0.011430	0.011295
0.011396	0.011329	0.011228	0.011330	0.011430	0.011432	0.011329
0.011228	0.011161	0.011295	0.011228	0.011161	0.011196	0.011430
0.011329	0.011262	0.011295	0.011329	0.011295	0.011228	0.011430
0.011295	0.011329	0.011329	0.011262	0.011329	0.011564	0.011228
0.011262	0.011664	0.011262	0.011295	0.011262	0.011631	0.011189
0.011678	0.011188	0.011261	0.011422	0.011508	0.011419	0.011144
0.011218	0.011429	0.011530	0.011637	0.011498	0.011635	0.011766
0.011666	0.011703	0.011664	0.011914	0.011631	0.012505	0.011666
0.012405	0.012173	0.012307	0.012578	0.011631	0.011329	0.011631
0.012070	0.012004	0.011597	0.011497	0.011564	0.011564	0.011534
0.011429	0.011433	0.011496	0.011464	0.011833	0.011900	0.011901
0.011800	0.011901	0.012004	0.011770	0.012070	0.012076	0.012137
0.012138	0.012172	0.012118	0.012138	0.012139	0.011362	0.011665
0.011733	0.012236	0.011738	0.011967	0.012440	0.011502	0.011396
0.011362	0.011362	0.011833	0.011800	0.011975	0.011698	0.011363
S021	0.011597	0.011597	0.011933	0.011698	0.011698	0.011765
0.011603	0.011535	0.011765	0.011195	0.011262	0.011228	0.011194
0.011228	0.011396	0.011194	0.011228	0.011295	0.011262	0.011228
0.000000	0.011228	0.011329	0.011195	0.011228	0.011228	0.011569
0.011463	0.011228	0.011127	0.011362	0.011295	0.011262	0.011295
0.011262	0.011362	0.011262	0.011262	0.011295	0.011262	0.011429
0.011194	0.011262	0.011295	0.011228	0.011329	0.011362	0.011295
0.011262	0.011429	0.011329	0.011597	0.011262	0.011530	0.011262
0.011228	0.011329	0.011396	0.011530	0.011631	0.011329	0.011429
0.011295	0.011362	0.011262	0.011496	0.011732	0.011262	0.011329
0.011228	0.011329	0.011295	0.011362	0.011295	0.011295	0.011295
0.011194	0.011228	0.011194	0.011295	0.011262	0.011296	0.011362
0.011295	0.011262	0.011329	0.011228	0.011362	0.011194	0.011262
0.011429	0.011362	0.011597	0.011429	0.011262	0.011496	0.011262
0.011431	0.011658	0.011475	0.011177	0.011172	0.011281	0.011375
0.012011	0.011467	0.011184	0.011429	0.011496	0.011295	0.011396
0.011329	0.011295	0.011362	0.011262	0.011362	0.011295	0.011295
0.011262	0.011362	0.011564	0.011429	0.011329	0.011295	0.011228
0.011195	0.011195	0.011262	0.011228	0.011429	0.011396	0.011329

0.011429	0.011497	0.011228	0.011228	0.011262	0.011262	0.011263
0.011331	0.011262	0.011262	0.011132	0.011205	0.011165	0.011262
0.011263	0.011198	0.011245	0.011233	0.011162	0.011197	0.011229
0.011492	0.011267	0.011301	0.011195	0.011199	0.011329	0.011296
0.011363	0.011296	0.011296	0.011396	0.011329	0.011698	0.011497
0.011530	0.011396	0.011262	0.011362	0.011161	0.011396	0.011195
0.011262	0.011262	0.011228	0.011194	0.011228	0.011195	0.011295
0.011262	0.011362	0.011295	0.011195	0.011296	0.011396	0.011398
0.011396	0.011295	0.011195	0.011128	0.011262	0.011195	0.011127
0.011230	0.011396	0.011195	0.011396	0.011161	0.011262	0.011228
0.011329	0.011228	0.011262	0.011228	0.011262	0.011127	0.011262
0.011295	0.011228	0.011295	0.011228	0.011262	0.011295	0.011530
0.011631	0.011564	0.011631	0.011397	0.011631	0.011262	0.011228
0.011263	0.011545	0.011289	0.011163	0.011637	0.011129	0.011257
0.011637	0.011536	0.011429	0.011631	0.011333	0.011496	0.011463
0.011396	0.011564	0.011415	0.011266	0.011312	0.011295	0.011365
0.011463	0.011364	0.011500	0.011568	0.011497	0.011564	0.011670
0.011399	0.011569	0.011500	0.011432	0.011699	0.011161	0.011363
0.011329	0.011262	0.011161	0.011161	0.011228	0.011228	0.011295
0.011228	0.011295	0.011194	0.011295	0.011228	0.011228	0.011295
0.011228	0.011664	0.011564	0.011597	0.011127	0.011228	0.011262
0.011262	0.011228	0.011295	0.011329	0.011194	0.011194	0.011194
0.011731	0.011362	0.011362	0.011161	0.011262	0.011429	0.011194
0.011194	0.011262	0.011228	0.011396	0.011194	0.011329	0.011463
0.011396	0.011228	0.011228	0.011161	0.011161	0.011329	0.011262
0.011161	0.011262	0.011127	0.011161	0.011295	0.011430	0.011195
0.011161	0.011295	0.011228	0.011127	0.011463	0.011127	0.011295
0.011368	0.011195	0.011496	0.011329	0.011362	0.011228	0.011329
0.011194	0.011530	0.011127	0.011396	0.011194	0.011194	0.011127
0.011362	0.011564	0.011262	0.011329	0.011161	0.011564	0.011194
0.011194	0.011194	0.011228	0.011329	0.011228	0.011329	0.011396
0.011396	0.011362	0.011262	0.011362	0.011329	0.011194	0.011161
0.011194	0.011194	0.011262	0.011194	0.011161	0.011329	0.011194
0.011228	0.011228	0.011362	0.011396	0.011295	0.011228	0.011228
0.011295	0.011228	0.011329	0.011194	0.011329	0.011429	0.011732
0.011228	0.011631	0.011233	0.017747	0.011191	0.011305	0.011583
0.011545	0.011588	0.011270	0.011234	0.011609	0.011612	0.011242
0.011487	0.011426	0.011138	0.011186	0.011216	0.011208	0.011232
0.011482	0.011395	0.011394	0.011259	0.011362	0.011396	0.011398
0.011266	0.011362	0.011362	0.011329	0.011295	0.011295	0.011262
0.011295	0.011228	0.011262	0.011295	0.011262	0.011195	0.011228
0.011228	0.011295	0.011195	0.011295	0.011329	0.011362	0.011262
0.011295	0.011262	0.011597	0.011597	0.011329	0.011363	0.011396
0.011161	0.011161	0.011195	0.011295	0.011161	0.011396	0.011396
0.011429	0.011429	0.011362	0.011463	0.011631	0.011464	0.011295
0.011295	0.011362	0.011396	0.011228	0.011295	0.011262	0.011295
0.011295	0.011295	0.011329	0.011363	0.011262	0.011463	0.011429
0.011297	0.011195	0.011295	0.011396	0.011295	0.011430	0.011463
0.011631	0.011333	0.011496	0.011463	0.011396	0.011564	0.011195
0.011363	0.011262	0.011228	0.011228	0.011295	0.011195	0.011295
0.011329	0.011362	0.011262	0.011295	0.011262	0.011597	0.011597
0.011329	0.011363	0.011429	0.011362	0.012002	0.011417	0.011341
0.011412	0.011372	0.011482	0.011372	0.011480	0.011405	0.011370
0.011953	0.011268	0.011403	0.011337	0.011611	0.011468	0.011570
0.011978	0.011437	0.011533	0.011597	0.011463	0.011463	0.011499
0.011161	0.011329	0.011463	0.011429	0.011432	0.011429	0.011397
0.011262	0.011194	0.011228	0.011262	0.011161	0.011228	0.011127
0.011262	0.011295	0.011194	0.011161	0.011262	0.011194	0.011228
0.011228	0.011161	0.011228	0.011262	0.011161	0.011362	0.011396
0.011295	0.011362	0.011295	0.011295	0.011228	0.011631	0.011262

0.011304	0.011228	0.011463	0.011467	0.011329	0.011329	0.011329
0.011329	0.011262	0.011228	0.011262	0.011362	0.011362	0.011295
0.011295	0.011429	0.011597	0.011329	0.011530	0.011530	0.011396
0.011228	0.011295	0.011329	0.011732	0.011295	0.011329	0.011295
0.011329	0.011262	0.011262	0.011564	0.011497	0.011530	0.011262
0.011362	0.011429	0.011295	0.011362	0.011329	0.011194	0.011530
0.011295	0.011262	0.011262	0.011429	0.011262	0.011362	0.011597
0.011295	0.011671	0.011664	0.011467	0.011229	0.011366	0.011215
0.011115	0.011683	0.011201	0.011417	0.011255	0.011315	0.011379
0.011468	0.011126	0.011339	0.011192	0.011222	0.011328	0.011344
0.011362	0.011257	0.011342	0.011272	0.011296	0.011429	0.011497
0.011497	0.011363	0.011466	0.011467	0.011602	0.011590	0.011295
0.011530	0.011396	0.011262	0.011362	0.011161	0.011396	0.011195
0.011262	0.011262	0.011228	0.011194	0.011228	0.011195	0.011295
0.011270	0.011270	0.011266	0.011300	0.011437	0.011402	0.011402
0.011401	0.011329	0.011134	0.011132	0.011263	0.011433	0.011433
0.011228	0.011297	0.011531	0.011500	0.011430	0.011464	0.011396
0.011329	0.011396	0.011396	0.011295	0.011866	0.011463	0.011530
0.011564	0.011630	0.011265	0.011337	0.011228	0.011429	0.011396
0.011430	0.011463	0.011463	0.011463	0.011610	0.011597	0.011234
0.011128	0.011130	0.011230	0.011196	0.011299	0.011135	0.011468
0.011266	0.011367	0.011156	0.011285	0.011262	0.011334	0.011228
0.011195	0.011195	0.011262	0.011228	0.011429	0.011396	0.011329
0.011429	0.011497	0.011228	0.011228	0.011262	0.011262	0.011263
0.011331	0.011262	0.011262	0.011296	0.011429	0.011497	0.011497
0.011363	0.011466	0.011467	0.011602	0.011590	0.011295	0.011463
0.011396	0.011396	0.011229	0.011408	0.011303	0.011367	0.011200
0.011405	0.011561	0.011399	0.011298	0.011195	0.011396	0.011262
0.011362	0.011295	0.011195	0.011296	0.011396	0.011398	0.011295
0.011195	0.011128	0.011262	0.011195	0.011127	0.011230	0.011396
0.011295	0.011228	0.011262	0.011295	0.011262	0.011195	0.011396
0.011262	0.011295	0.011295	0.011228	0.011295	0.011530	0.011194
0.011228	0.011631	0.011228	0.011262	0.011228	0.011597	0.011189
0.011678	0.011188	0.011261	0.011422	0.011508	0.011419	0.011144
0.011218	0.011396	0.011496	0.011603	0.011464	0.011601	0.011733
0.011632	0.011669	0.011631	0.011880	0.011597	0.012472	0.011633
0.012372	0.012139	0.012273	0.012545	0.011597	0.011295	0.011598
0.012036	0.011971	0.011564	0.011463	0.011531	0.011531	0.011500
0.011396	0.011399	0.011463	0.011430	0.011800	0.011866	0.011867
0.011766	0.011867	0.011970	0.011737	0.012036	0.012042	0.012104
0.012104	0.012138	0.012084	0.012104	0.012105	0.011329	0.011631
0.011699	0.012202	0.011704	0.011933	0.012406	0.011468	0.011363
0.011329	0.011329	0.011799	0.011842	0.011940	0.011664	0.011330
S022	0.011631	0.011631	0.011967	0.011732	0.011732	0.011799
0.011637	0.011569	0.011799	0.011228	0.011295	0.011262	0.011228
0.011262	0.011429	0.011228	0.011262	0.011329	0.011094	0.011262
0.011228	0.000000	0.011362	0.011228	0.011262	0.011262	0.011603
0.011496	0.011262	0.011161	0.011396	0.011329	0.011295	0.011329
0.011295	0.011396	0.011295	0.011295	0.011329	0.011295	0.011463
0.011228	0.011295	0.011329	0.011262	0.011362	0.011396	0.011329
0.011295	0.011463	0.011362	0.011631	0.011295	0.011564	0.011295
0.011262	0.011362	0.011429	0.011564	0.011664	0.011362	0.011463
0.011329	0.011396	0.011295	0.011530	0.011765	0.011295	0.011362
0.011262	0.011362	0.011329	0.011396	0.011329	0.011329	0.011329
0.011228	0.011262	0.011228	0.011329	0.011295	0.011329	0.011396
0.011329	0.011295	0.011362	0.011262	0.011396	0.011228	0.011295
0.011463	0.011396	0.011631	0.011463	0.011295	0.011530	0.011295
0.011468	0.011695	0.011513	0.011216	0.011210	0.011318	0.011415
0.012048	0.011467	0.011225	0.011530	0.011530	0.011329	0.011429
0.011362	0.011329	0.011396	0.011295	0.011396	0.011329	0.011329

0.011295	0.011396	0.011597	0.011463	0.011362	0.011329	0.011262
0.011228	0.011228	0.011295	0.011262	0.011463	0.011430	0.011362
0.011463	0.011530	0.011262	0.011262	0.011295	0.011295	0.011297
0.011364	0.011295	0.011295	0.011168	0.011241	0.011200	0.011296
0.011297	0.011232	0.011282	0.011267	0.011196	0.011232	0.011263
0.011528	0.011302	0.011336	0.011228	0.011233	0.011363	0.011329
0.011397	0.011330	0.011329	0.011429	0.011362	0.011732	0.011530
0.011564	0.011430	0.011295	0.011396	0.011195	0.011429	0.011228
0.011295	0.011295	0.011262	0.011228	0.011262	0.011228	0.011329
0.011295	0.011396	0.011329	0.011228	0.011330	0.011430	0.011432
0.011430	0.011329	0.011228	0.011161	0.011295	0.011228	0.011161
0.011264	0.011430	0.011228	0.011430	0.011195	0.011295	0.011262
0.011362	0.011262	0.011295	0.011262	0.011295	0.011161	0.011295
0.011329	0.011262	0.011329	0.011262	0.011295	0.011329	0.011564
0.011664	0.011597	0.011665	0.011431	0.011665	0.011295	0.011262
0.011297	0.011545	0.011335	0.011197	0.011671	0.011163	0.011257
0.011671	0.011570	0.011463	0.011664	0.011368	0.011530	0.011497
0.011429	0.011597	0.011451	0.011307	0.011354	0.011329	0.011398
0.011497	0.011397	0.011533	0.011601	0.011530	0.011597	0.011704
0.011432	0.011603	0.011534	0.011466	0.011733	0.011194	0.011397
0.011362	0.011295	0.011194	0.011194	0.011262	0.011262	0.011329
0.011262	0.011329	0.011228	0.011329	0.011262	0.011262	0.011329
0.000000	0.011698	0.011597	0.011631	0.011161	0.011262	0.011295
0.011295	0.011262	0.011329	0.011362	0.011228	0.011228	0.011228
0.011765	0.011396	0.011396	0.011194	0.011295	0.011463	0.011228
0.011228	0.011295	0.011262	0.011429	0.011228	0.011362	0.011496
0.011429	0.011262	0.011262	0.011194	0.011194	0.011362	0.011295
0.011194	0.011295	0.011161	0.011195	0.011329	0.011463	0.011228
0.011195	0.011329	0.011262	0.011161	0.011496	0.011161	0.011329
0.011403	0.011228	0.011530	0.011362	0.011396	0.011262	0.011362
0.011228	0.011564	0.011161	0.011429	0.011228	0.011228	0.011161
0.011396	0.011598	0.011295	0.011362	0.011194	0.011597	0.011228
0.011228	0.011228	0.011262	0.011362	0.011262	0.011362	0.011429
0.011429	0.011396	0.011295	0.011396	0.011362	0.011228	0.011194
0.011228	0.011228	0.011295	0.011228	0.011194	0.011362	0.011228
0.011262	0.011262	0.011396	0.011429	0.011329	0.011262	0.011262
0.011329	0.011262	0.011362	0.011228	0.011362	0.011463	0.011765
0.011262	0.011664	0.011267	0.017785	0.011191	0.011488	0.011618
0.011579	0.011623	0.011374	0.011350	0.011774	0.011647	0.011425
0.011534	0.011610	0.011177	0.011270	0.011255	0.011356	0.011275
0.011482	0.011433	0.011431	0.011126	0.011396	0.011429	0.011432
0.011301	0.011396	0.011396	0.011362	0.011329	0.011329	0.011295
0.011329	0.011262	0.011295	0.011329	0.011295	0.011228	0.011262
0.011262	0.011329	0.011228	0.011329	0.011362	0.011396	0.011295
0.011329	0.011295	0.011631	0.011631	0.011362	0.011396	0.011430
0.011195	0.011195	0.011228	0.011329	0.011195	0.011430	0.011430
0.011463	0.011463	0.011396	0.011497	0.011665	0.011498	0.011329
0.011329	0.011396	0.011430	0.011262	0.011329	0.011295	0.011329
0.011396	0.011396	0.011429	0.011396	0.011296	0.011497	0.011463
0.011331	0.011228	0.011329	0.011430	0.011329	0.011463	0.011496
0.011664	0.011368	0.011530	0.011497	0.011429	0.011597	0.011228
0.011397	0.011296	0.011262	0.011262	0.011329	0.011228	0.011329
0.011362	0.011396	0.011295	0.011329	0.011295	0.011631	0.011631
0.011362	0.011396	0.011463	0.011396	0.012035	0.011453	0.011377
0.011447	0.011407	0.011517	0.011406	0.011515	0.011439	0.011405
0.011987	0.011302	0.011437	0.011371	0.011645	0.011502	0.011605
0.012012	0.011475	0.011567	0.011631	0.011496	0.011497	0.011533
0.011194	0.011362	0.011496	0.011463	0.011466	0.011463	0.011431
0.011295	0.011228	0.011262	0.011295	0.011194	0.011262	0.011161
0.011295	0.011329	0.011228	0.011194	0.011295	0.011228	0.011262

0.011262	0.011194	0.011262	0.011295	0.011194	0.011396	0.011429
0.011329	0.011396	0.011329	0.011329	0.011262	0.011664	0.011295
0.011338	0.011262	0.011497	0.011501	0.011362	0.011362	0.011362
0.011362	0.011295	0.011262	0.011295	0.011396	0.011396	0.011329
0.011329	0.011463	0.011631	0.011362	0.011564	0.011564	0.011429
0.011262	0.011329	0.011362	0.011765	0.011329	0.011362	0.011329
0.011362	0.011295	0.011295	0.011597	0.011530	0.011564	0.011295
0.011396	0.011463	0.011329	0.011396	0.011362	0.011228	0.011564
0.011329	0.011295	0.011295	0.011463	0.011295	0.011396	0.011631
0.011329	0.011705	0.011698	0.011507	0.011229	0.011400	0.011318
0.011280	0.011722	0.011236	0.011462	0.011304	0.011417	0.011432
0.011468	0.011192	0.011394	0.011192	0.011276	0.011395	0.011385
0.011399	0.011296	0.011377	0.011307	0.011330	0.011463	0.011530
0.011530	0.011396	0.011500	0.011501	0.011636	0.011661	0.011329
0.011564	0.011430	0.011295	0.011396	0.011195	0.011429	0.011228
0.011295	0.011295	0.011262	0.011228	0.011262	0.011228	0.011329
0.011305	0.011305	0.011300	0.011334	0.011471	0.011436	0.011436
0.011435	0.011363	0.011171	0.011168	0.011297	0.011467	0.011467
0.011262	0.011331	0.011565	0.011533	0.011464	0.011498	0.011429
0.011362	0.011429	0.011429	0.011329	0.011900	0.011497	0.011564
0.011597	0.011665	0.011299	0.011337	0.011262	0.011463	0.011429
0.011464	0.011496	0.011496	0.011496	0.011650	0.011631	0.011268
0.011162	0.011164	0.011264	0.011230	0.011333	0.011172	0.011502
0.011300	0.011411	0.011156	0.011322	0.011296	0.011368	0.011262
0.011228	0.011228	0.011295	0.011262	0.011463	0.011430	0.011362
0.011463	0.011530	0.011262	0.011262	0.011295	0.011295	0.011297
0.011364	0.011295	0.011295	0.011330	0.011463	0.011530	0.011530
0.011396	0.011500	0.011501	0.011636	0.011661	0.011329	0.011496
0.011429	0.011429	0.011263	0.011443	0.011373	0.011401	0.011270
0.011439	0.011653	0.011357	0.011332	0.011228	0.011430	0.011295
0.011396	0.011329	0.011228	0.011330	0.011430	0.011432	0.011329
0.011228	0.011161	0.011295	0.011228	0.011161	0.011264	0.011430
0.011329	0.011262	0.011295	0.011329	0.011295	0.011228	0.011430
0.011295	0.011329	0.011329	0.011262	0.011329	0.011564	0.011228
0.011262	0.011664	0.011262	0.011295	0.011262	0.011631	0.011232
0.011679	0.011230	0.011261	0.011463	0.011549	0.011455	0.011186
0.011218	0.011429	0.011530	0.011637	0.011498	0.011635	0.011766
0.011666	0.011703	0.011664	0.011914	0.011631	0.012505	0.011667
0.012406	0.012173	0.012307	0.012579	0.011631	0.011329	0.011631
0.012070	0.012005	0.011597	0.011497	0.011565	0.011565	0.011534
0.011429	0.011433	0.011496	0.011464	0.011834	0.011900	0.011901
0.011800	0.011901	0.012004	0.011770	0.012070	0.012076	0.012137
0.012138	0.012172	0.012118	0.012138	0.012139	0.011362	0.011665
0.011733	0.012236	0.011738	0.011967	0.012440	0.011502	0.011396
0.011362	0.011362	0.011833	0.011842	0.011975	0.011698	0.011363
S023	0.011732	0.011732	0.011732	0.011496	0.011496	0.011564
0.011468	0.011400	0.011631	0.011329	0.011396	0.011363	0.011329
0.011295	0.011530	0.011262	0.011362	0.011429	0.011396	0.011362
0.011329	0.011362	0.000000	0.011329	0.011362	0.011362	0.011434
0.011329	0.011362	0.011262	0.011094	0.011429	0.011396	0.011429
0.011396	0.011496	0.011396	0.011396	0.011429	0.011396	0.011564
0.011262	0.011396	0.011429	0.011362	0.011396	0.011496	0.011429
0.011396	0.011564	0.011463	0.011463	0.011396	0.011664	0.011396
0.011362	0.011396	0.011530	0.011664	0.011497	0.011396	0.011496
0.011429	0.011496	0.011396	0.011631	0.011597	0.011396	0.011463
0.011362	0.011463	0.011429	0.011496	0.011429	0.011429	0.011429
0.011329	0.011362	0.011329	0.011362	0.011396	0.011430	0.011496
0.011429	0.011396	0.011463	0.011362	0.011429	0.011329	0.011396
0.011564	0.011496	0.011530	0.011564	0.011396	0.011631	0.011396
0.011579	0.011508	0.011626	0.011334	0.011321	0.011391	0.011139

0.011792	0.011213	0.011348	0.011631	0.011631	0.011362	0.011463
0.011463	0.011429	0.011496	0.011396	0.011496	0.011429	0.011429
0.011396	0.011429	0.011698	0.011564	0.011463	0.011429	0.011362
0.011329	0.011262	0.011396	0.011362	0.011228	0.011195	0.011127
0.011228	0.011295	0.011362	0.011362	0.011396	0.011396	0.011398
0.011466	0.011396	0.011396	0.011276	0.011349	0.011306	0.011396
0.011399	0.011335	0.011392	0.011371	0.011298	0.011334	0.011364
0.011348	0.011405	0.011439	0.011329	0.011337	0.011464	0.011363
0.011430	0.011431	0.011430	0.011530	0.011463	0.011832	0.011564
0.011396	0.011463	0.011396	0.011430	0.011295	0.011262	0.011329
0.011396	0.011396	0.011362	0.011329	0.011362	0.011329	0.011429
0.011396	0.011497	0.011429	0.011329	0.011397	0.011530	0.011533
0.011530	0.011362	0.011329	0.011263	0.011396	0.011329	0.011262
0.011264	0.011530	0.011329	0.011530	0.011295	0.011396	0.011362
0.011463	0.011362	0.011396	0.011362	0.011396	0.011262	0.011396
0.011429	0.011362	0.011429	0.011362	0.011396	0.011429	0.011665
0.011497	0.011429	0.011497	0.011532	0.011497	0.011396	0.011362
0.011330	0.011586	0.011472	0.011300	0.011501	0.011265	0.011375
0.011502	0.011400	0.011564	0.011765	0.011470	0.011631	0.011597
0.011530	0.011698	0.011557	0.011431	0.011479	0.011429	0.011500
0.011597	0.011499	0.011635	0.011703	0.011631	0.011698	0.011805
0.011534	0.011705	0.011636	0.011567	0.011834	0.011295	0.011498
0.011463	0.011396	0.011295	0.011295	0.011362	0.011362	0.011429
0.011362	0.011429	0.011329	0.011362	0.011362	0.011362	0.011429
0.011362	0.011530	0.011429	0.011463	0.011262	0.011362	0.011396
0.011396	0.011362	0.011429	0.011396	0.011329	0.011329	0.011329
0.011597	0.011429	0.011496	0.011295	0.011396	0.011564	0.011329
0.011329	0.011396	0.011362	0.011530	0.011329	0.011262	0.011396
0.011329	0.011362	0.011362	0.011295	0.011295	0.011463	0.011396
0.011295	0.011396	0.011262	0.011295	0.011429	0.011295	0.011329
0.011295	0.011429	0.011363	0.011262	0.011329	0.011262	0.011429
0.011505	0.011329	0.011362	0.011463	0.011429	0.011362	0.011463
0.011329	0.011664	0.011262	0.011463	0.011262	0.011262	0.011262
0.011496	0.011430	0.011396	0.011463	0.011295	0.011429	0.011262
0.011329	0.011329	0.011362	0.011463	0.011362	0.011463	0.011530
0.011530	0.011496	0.011396	0.011496	0.011463	0.011329	0.011295
0.011329	0.011329	0.011396	0.011262	0.011295	0.011463	0.011329
0.011295	0.011362	0.011496	0.011463	0.011429	0.011362	0.011362
0.011429	0.011362	0.011463	0.011329	0.011463	0.011564	0.011530
0.011362	0.011497	0.011371	0.017899	0.011322	0.011611	0.011444
0.011406	0.011447	0.011427	0.011234	0.011555	0.011475	0.011607
0.011392	0.011243	0.011294	0.011395	0.011255	0.011455	0.011404
0.011248	0.011246	0.011246	0.000000	0.011496	0.011530	0.011534
0.011404	0.011497	0.011497	0.011396	0.011429	0.011430	0.011396
0.011429	0.011362	0.011396	0.011429	0.011396	0.011329	0.011362
0.011362	0.011429	0.011329	0.011430	0.011463	0.011497	0.011396
0.011362	0.011329	0.011463	0.011463	0.011463	0.011497	0.011530
0.011295	0.011295	0.011329	0.011429	0.011295	0.011530	0.011530
0.011564	0.011564	0.011496	0.011597	0.011497	0.011599	0.011429
0.011429	0.011497	0.011530	0.011362	0.011429	0.011396	0.011429
0.011497	0.011497	0.011530	0.011497	0.011397	0.011597	0.011564
0.011433	0.011329	0.011429	0.011530	0.011430	0.011497	0.011597
0.011765	0.011470	0.011631	0.011597	0.011530	0.011698	0.011329
0.011498	0.011396	0.011362	0.011362	0.011429	0.011329	0.011430
0.011463	0.011497	0.011396	0.011362	0.011329	0.011463	0.011463
0.011463	0.011497	0.011564	0.011496	0.012069	0.011560	0.011482
0.011552	0.011511	0.011622	0.011510	0.011620	0.011543	0.011508
0.012021	0.011406	0.011540	0.011475	0.011749	0.011604	0.011707
0.012046	0.011512	0.011669	0.011731	0.011597	0.011262	0.011635
0.011295	0.011463	0.011597	0.011564	0.011567	0.011295	0.011262

0.011396	0.011329	0.011362	0.011396	0.011295	0.011362	0.011262
0.011329	0.011362	0.011329	0.011295	0.011396	0.011329	0.011362
0.011362	0.011295	0.011362	0.011396	0.011295	0.011496	0.011530
0.011429	0.011429	0.011429	0.011429	0.011362	0.011497	0.011396
0.011095	0.011362	0.011329	0.011332	0.011463	0.011463	0.011463
0.011463	0.011396	0.011362	0.011396	0.011496	0.011496	0.011429
0.011429	0.011564	0.011463	0.011463	0.011664	0.011664	0.011530
0.011362	0.011429	0.011463	0.011597	0.011430	0.011463	0.011429
0.011463	0.011329	0.011329	0.011631	0.011631	0.011597	0.011396
0.011496	0.011564	0.011429	0.011496	0.011463	0.011329	0.011396
0.011429	0.011396	0.011329	0.011564	0.011396	0.011496	0.011530
0.011429	0.011535	0.011530	0.011304	0.011117	0.011434	0.011163
0.011390	0.011527	0.011341	0.011507	0.011353	0.011366	0.011326
0.011213	0.011258	0.011394	0.011192	0.011276	0.011395	0.011506
0.011512	0.011414	0.011412	0.011412	0.011431	0.011295	0.011362
0.011228	0.011430	0.011533	0.011264	0.011670	0.011696	0.011429
0.011396	0.011463	0.011396	0.011430	0.011295	0.011262	0.011329
0.011396	0.011396	0.011362	0.011329	0.011362	0.011329	0.011429
0.011409	0.011410	0.011403	0.011437	0.011573	0.011538	0.011538
0.011537	0.011464	0.011282	0.011276	0.011399	0.011568	0.011569
0.011363	0.011433	0.011666	0.011635	0.011565	0.011599	0.011530
0.011463	0.011530	0.011530	0.011429	0.011732	0.011597	0.011664
0.011698	0.011487	0.011401	0.011476	0.011362	0.011564	0.011530
0.011565	0.011597	0.011597	0.011597	0.011768	0.011731	0.011372
0.011263	0.011268	0.011366	0.011332	0.011368	0.011284	0.011332
0.011403	0.011236	0.011298	0.011435	0.011396	0.011471	0.011362
0.011329	0.011262	0.011396	0.011362	0.011228	0.011195	0.011127
0.011228	0.011295	0.011362	0.011362	0.011396	0.011396	0.011398
0.011466	0.011396	0.011396	0.011431	0.011295	0.011362	0.011228
0.011430	0.011533	0.011264	0.011670	0.011696	0.011429	0.011597
0.011530	0.011530	0.011365	0.011547	0.011477	0.011503	0.011375
0.011508	0.011698	0.011484	0.011434	0.011329	0.011530	0.011396
0.011497	0.011429	0.011329	0.011397	0.011530	0.011533	0.011362
0.011329	0.011263	0.011396	0.011329	0.011262	0.011264	0.011530
0.011429	0.011362	0.011396	0.011429	0.011396	0.011329	0.011262
0.011396	0.011429	0.011429	0.011362	0.011429	0.011664	0.011262
0.011362	0.011497	0.011362	0.011396	0.011362	0.011463	0.011360
0.011510	0.011230	0.011161	0.011262	0.011345	0.011276	0.011186
0.011113	0.011262	0.011362	0.011468	0.011330	0.011466	0.011598
0.011498	0.011534	0.011496	0.011744	0.011463	0.012337	0.011498
0.012237	0.012004	0.012139	0.012410	0.011463	0.011362	0.011463
0.012103	0.011836	0.011429	0.011329	0.011396	0.011396	0.011365
0.011262	0.011264	0.011329	0.011296	0.011665	0.011732	0.011733
0.011632	0.011733	0.011836	0.011601	0.012104	0.012110	0.012171
0.012171	0.012205	0.012152	0.012172	0.012173	0.011463	0.011699
0.011766	0.012270	0.011772	0.011799	0.012272	0.011332	0.011228
0.011194	0.011194	0.011665	0.011677	0.011799	0.011530	0.011195
S024	0.011598	0.011598	0.011901	0.011665	0.011665	0.011732
0.011604	0.011536	0.011732	0.011195	0.011262	0.011229	0.011195
0.011228	0.011396	0.011195	0.011228	0.011295	0.011262	0.011228
0.011195	0.011228	0.011329	0.000000	0.011228	0.011228	0.011570
0.011463	0.011228	0.011127	0.011363	0.011295	0.011262	0.011295
0.011262	0.011363	0.011262	0.011262	0.011295	0.011262	0.011430
0.011195	0.011262	0.011295	0.011228	0.011329	0.011363	0.011295
0.011262	0.011430	0.011329	0.011598	0.011262	0.011531	0.011262
0.011228	0.011329	0.011396	0.011531	0.011631	0.011329	0.011430
0.011295	0.011363	0.011262	0.011497	0.011732	0.011262	0.011329
0.011228	0.011329	0.011295	0.011363	0.011295	0.011295	0.011295
0.011195	0.011228	0.011195	0.011295	0.011262	0.011296	0.011363
0.011295	0.011262	0.011329	0.011228	0.011363	0.011195	0.011262

0.011430	0.011363	0.011598	0.011430	0.011262	0.011497	0.011262
0.011394	0.011621	0.011438	0.011139	0.011172	0.011244	0.011336
0.012013	0.011417	0.011143	0.011497	0.011497	0.011295	0.011396
0.011329	0.011295	0.011363	0.011262	0.011363	0.011295	0.011295
0.011262	0.011363	0.011564	0.011430	0.011329	0.011295	0.011228
0.011195	0.011195	0.011262	0.011228	0.011430	0.011396	0.011329
0.011430	0.011497	0.011228	0.011228	0.011262	0.011262	0.011263
0.011331	0.011262	0.011262	0.011132	0.011205	0.011166	0.011262
0.011264	0.011198	0.011245	0.011233	0.011162	0.011197	0.011229
0.011493	0.011267	0.011302	0.011195	0.011199	0.011330	0.011296
0.011363	0.011296	0.011296	0.011396	0.011329	0.011699	0.011497
0.011530	0.011396	0.011262	0.011363	0.011161	0.011396	0.011195
0.011262	0.011262	0.011228	0.011195	0.011228	0.011195	0.011295
0.011262	0.011363	0.011296	0.011195	0.011296	0.011396	0.011365
0.011396	0.011296	0.011195	0.011128	0.011262	0.011195	0.011128
0.011230	0.011396	0.011195	0.011396	0.011161	0.011262	0.011228
0.011329	0.011228	0.011262	0.011228	0.011262	0.011128	0.011262
0.011296	0.011228	0.011296	0.011228	0.011262	0.011295	0.011531
0.011632	0.011564	0.011632	0.011398	0.011632	0.011262	0.011228
0.011263	0.011505	0.011381	0.011163	0.011637	0.011129	0.011257
0.011638	0.011503	0.011430	0.011631	0.011334	0.011497	0.011463
0.011329	0.011564	0.011416	0.011266	0.011270	0.011295	0.011365
0.011463	0.011364	0.011500	0.011568	0.011497	0.011564	0.011671
0.011399	0.011570	0.011501	0.011432	0.011700	0.011161	0.011364
0.011329	0.011262	0.011161	0.011161	0.011228	0.011228	0.011295
0.011228	0.011295	0.011195	0.011295	0.011228	0.011228	0.011295
0.011228	0.011665	0.011564	0.011598	0.011127	0.011228	0.011262
0.011262	0.011228	0.011295	0.011329	0.011195	0.011195	0.011195
0.011699	0.011363	0.011363	0.011161	0.011262	0.011430	0.011195
0.011195	0.011262	0.011228	0.011396	0.011195	0.011329	0.011463
0.011396	0.011228	0.011228	0.011161	0.011161	0.011329	0.011262
0.011161	0.011262	0.011128	0.011161	0.011295	0.011430	0.011195
0.011161	0.011295	0.011228	0.011128	0.011463	0.011128	0.011295
0.011368	0.011195	0.011497	0.011329	0.011363	0.011228	0.011329
0.011195	0.011531	0.011127	0.011396	0.011195	0.011195	0.011127
0.011363	0.011564	0.011262	0.011329	0.011161	0.011564	0.011195
0.011195	0.011195	0.011228	0.011329	0.011228	0.011329	0.011396
0.011396	0.011363	0.011262	0.011363	0.011329	0.011195	0.011161
0.011195	0.011195	0.011262	0.011195	0.011161	0.011329	0.011195
0.011228	0.011228	0.011363	0.011396	0.011295	0.011228	0.011228
0.011295	0.011228	0.011329	0.011195	0.011329	0.011430	0.011732
0.011228	0.011631	0.011233	0.017718	0.011191	0.011305	0.011549
0.011510	0.011553	0.011217	0.011176	0.011610	0.011579	0.011243
0.011393	0.011427	0.011099	0.011186	0.011178	0.011209	0.011189
0.011436	0.011396	0.011394	0.011259	0.011363	0.011396	0.011399
0.011267	0.011363	0.011363	0.011329	0.011295	0.011296	0.011262
0.011295	0.011228	0.011262	0.011296	0.011262	0.011195	0.011228
0.011228	0.011296	0.011195	0.011296	0.011329	0.011363	0.011262
0.011295	0.011262	0.011598	0.011598	0.011329	0.011363	0.011396
0.011161	0.011161	0.011195	0.011296	0.011161	0.011396	0.011396
0.011430	0.011430	0.011363	0.011463	0.011632	0.011397	0.011295
0.011296	0.011363	0.011396	0.011228	0.011295	0.011262	0.011295
0.011363	0.011363	0.011396	0.011363	0.011262	0.011464	0.011430
0.011298	0.011195	0.011295	0.011396	0.011296	0.011430	0.011463
0.011631	0.011334	0.011497	0.011463	0.011329	0.011564	0.011195
0.011364	0.011262	0.011228	0.011228	0.011296	0.011195	0.011296
0.011329	0.011363	0.011262	0.011295	0.011262	0.011598	0.011598
0.011329	0.011363	0.011430	0.011363	0.012003	0.011418	0.011342
0.011412	0.011373	0.011482	0.011372	0.011481	0.011405	0.011371
0.011953	0.011268	0.011403	0.011337	0.011577	0.011469	0.011571

0.011979	0.011437	0.011534	0.011598	0.011463	0.011463	0.011500
0.011161	0.011329	0.011463	0.011430	0.011432	0.011430	0.011364
0.011262	0.011195	0.011228	0.011262	0.011161	0.011228	0.011127
0.011262	0.011295	0.011195	0.011161	0.011262	0.011195	0.011228
0.011228	0.011161	0.011228	0.011262	0.011161	0.011363	0.011396
0.011295	0.011363	0.011295	0.011295	0.011228	0.011631	0.011262
0.011304	0.011228	0.011464	0.011468	0.011329	0.011329	0.011329
0.011329	0.011262	0.011228	0.011262	0.011363	0.011363	0.011295
0.011295	0.011430	0.011598	0.011329	0.011531	0.011531	0.011396
0.011228	0.011295	0.011329	0.011732	0.011296	0.011329	0.011295
0.011329	0.011262	0.011262	0.011564	0.011497	0.011531	0.011262
0.011363	0.011396	0.011295	0.011363	0.011329	0.011195	0.011531
0.011295	0.011262	0.011262	0.011430	0.011262	0.011363	0.011598
0.011295	0.011672	0.011665	0.011426	0.011173	0.011366	0.011215
0.011115	0.011645	0.011201	0.011373	0.011207	0.011315	0.011326
0.011418	0.000000	0.011283	0.000000	0.011168	0.011329	0.011304
0.011324	0.011218	0.011307	0.011272	0.011296	0.011430	0.011497
0.011497	0.011363	0.011466	0.011467	0.011603	0.011626	0.011295
0.011530	0.011396	0.011262	0.011363	0.011161	0.011396	0.011195
0.011262	0.011262	0.011228	0.011195	0.011228	0.011195	0.011295
0.011270	0.011270	0.011266	0.011300	0.011437	0.011402	0.011402
0.011401	0.011329	0.011134	0.011132	0.011264	0.011433	0.011434
0.011229	0.011298	0.011532	0.011500	0.011430	0.011397	0.011396
0.011329	0.011396	0.011396	0.011295	0.011867	0.011463	0.011531
0.011564	0.011630	0.011231	0.011338	0.011228	0.011430	0.011396
0.011431	0.011463	0.011463	0.011463	0.011572	0.011598	0.011234
0.011128	0.011130	0.011231	0.011196	0.011300	0.011135	0.011468
0.011266	0.011411	0.011156	0.011285	0.011262	0.011334	0.011228
0.011195	0.011195	0.011262	0.011228	0.011430	0.011396	0.011329
0.011430	0.011497	0.011228	0.011228	0.011262	0.011262	0.011263
0.011331	0.011262	0.011262	0.011296	0.011430	0.011497	0.011497
0.011363	0.011466	0.011467	0.011603	0.011626	0.011295	0.011463
0.011396	0.011396	0.011229	0.011409	0.011338	0.011367	0.011236
0.011405	0.011608	0.011399	0.011298	0.011195	0.011396	0.011262
0.011363	0.011296	0.011195	0.011296	0.011396	0.011365	0.011296
0.011195	0.011128	0.011262	0.011195	0.011128	0.011230	0.011396
0.011295	0.011228	0.011262	0.011296	0.011262	0.011195	0.011396
0.011262	0.011295	0.011295	0.011228	0.011295	0.011531	0.011195
0.011228	0.011631	0.011228	0.011262	0.011228	0.011598	0.011146
0.011567	0.011145	0.011211	0.011383	0.011468	0.011419	0.011102
0.011165	0.011363	0.011463	0.011570	0.011431	0.011568	0.011700
0.011599	0.011636	0.011598	0.011847	0.011564	0.012439	0.011600
0.012306	0.012107	0.012241	0.012546	0.011598	0.011296	0.011565
0.012037	0.011938	0.011531	0.011430	0.011498	0.011498	0.011466
0.011363	0.011366	0.011430	0.011397	0.011801	0.011867	0.011868
0.011767	0.011868	0.011971	0.011737	0.012038	0.012043	0.012105
0.012105	0.012139	0.012085	0.012105	0.012107	0.011329	0.011632
0.011700	0.012204	0.011705	0.011934	0.012408	0.011468	0.011363
0.011329	0.011329	0.011800	0.011801	0.011905	0.011665	0.011330
S025 0.011631	0.011631	0.011934	0.011699	0.011699	0.011766	
0.011638	0.011570	0.011766	0.011228	0.011295	0.011262	0.011228
0.011262	0.011430	0.011228	0.011262	0.011329	0.011295	0.011262
0.011228	0.011262	0.011362	0.011228	0.000000	0.011262	0.011604
0.011497	0.011262	0.011161	0.011396	0.011262	0.011295	0.011262
0.011295	0.011329	0.011295	0.011295	0.011329	0.011295	0.011463
0.011228	0.011295	0.011329	0.011262	0.011363	0.011396	0.011329
0.011295	0.011463	0.011363	0.011631	0.011295	0.011497	0.011295
0.011262	0.011363	0.011430	0.011497	0.011665	0.011363	0.011463
0.011262	0.011329	0.011295	0.011463	0.011766	0.011295	0.011363
0.011262	0.011363	0.011329	0.011396	0.011329	0.011329	0.011329

0.011228	0.011262	0.011228	0.011329	0.011295	0.011330	0.011396
0.011329	0.011295	0.011295	0.011262	0.011396	0.011161	0.011295
0.011463	0.011396	0.011631	0.011463	0.011295	0.011463	0.011295
0.011468	0.011696	0.011513	0.011217	0.011210	0.011318	0.011415
0.012049	0.011467	0.011225	0.011530	0.011531	0.011329	0.011430
0.011363	0.011329	0.011396	0.011295	0.011396	0.011262	0.011329
0.011295	0.011396	0.011598	0.011463	0.011363	0.011329	0.011195
0.011228	0.011228	0.011228	0.011262	0.011463	0.011430	0.011363
0.011463	0.011531	0.011262	0.011262	0.011295	0.011295	0.011297
0.011365	0.011295	0.011296	0.011132	0.011241	0.011201	0.011296
0.011298	0.011232	0.011282	0.011267	0.011196	0.011232	0.011263
0.011529	0.011302	0.011267	0.011229	0.011233	0.011363	0.011330
0.011397	0.011330	0.011329	0.011430	0.011362	0.011665	0.011531
0.011564	0.011430	0.011295	0.011396	0.011195	0.011430	0.011228
0.011295	0.011295	0.011262	0.011228	0.011262	0.011228	0.011329
0.011228	0.011329	0.011329	0.011228	0.011330	0.011396	0.011432
0.011363	0.011329	0.011228	0.011162	0.011295	0.011228	0.011161
0.011264	0.011363	0.011161	0.011363	0.011195	0.011296	0.011262
0.011295	0.011262	0.011228	0.011262	0.011296	0.011161	0.011228
0.011262	0.011262	0.011329	0.011262	0.011295	0.011329	0.011564
0.011665	0.011598	0.011665	0.011431	0.011665	0.011295	0.011262
0.011297	0.011546	0.011289	0.011198	0.011671	0.011163	0.011297
0.011672	0.011571	0.011463	0.011665	0.011368	0.011531	0.011430
0.011430	0.011598	0.011451	0.011308	0.011354	0.011329	0.011399
0.011497	0.011398	0.011534	0.011534	0.011531	0.011598	0.011704
0.011433	0.011536	0.011535	0.011466	0.011734	0.011195	0.011397
0.011295	0.011295	0.011195	0.011195	0.011195	0.011262	0.011329
0.011195	0.011262	0.011161	0.011329	0.011195	0.011262	0.011329
0.011262	0.011698	0.011598	0.011631	0.011161	0.011262	0.011295
0.011295	0.011262	0.011329	0.011363	0.011228	0.011228	0.011228
0.011732	0.011396	0.011396	0.011195	0.011295	0.011396	0.011228
0.011228	0.011295	0.011262	0.011363	0.011228	0.011363	0.011430
0.011430	0.011228	0.011262	0.011195	0.011195	0.011363	0.011295
0.011195	0.011262	0.011161	0.011195	0.011329	0.011464	0.011228
0.011195	0.011329	0.011262	0.011161	0.011497	0.011161	0.011329
0.011334	0.011228	0.011531	0.011363	0.011396	0.011262	0.011363
0.011228	0.011497	0.011161	0.011430	0.011228	0.011228	0.011161
0.011329	0.011598	0.011295	0.011363	0.011195	0.011598	0.011228
0.011228	0.011228	0.011262	0.011295	0.011262	0.011329	0.011430
0.011430	0.011396	0.011295	0.011396	0.011295	0.011228	0.011195
0.011228	0.011228	0.011295	0.011228	0.011127	0.011363	0.011228
0.011262	0.011262	0.011396	0.011430	0.011329	0.011195	0.011262
0.011329	0.011262	0.011363	0.011228	0.011363	0.011463	0.011766
0.011262	0.011665	0.011267	0.017755	0.011191	0.011489	0.011619
0.011580	0.011624	0.011322	0.011293	0.011665	0.011648	0.011304
0.011440	0.011610	0.011177	0.011228	0.011217	0.011357	0.011276
0.011483	0.011433	0.011394	0.011193	0.011396	0.011430	0.011433
0.011232	0.011396	0.011396	0.011363	0.011329	0.011329	0.011296
0.011329	0.011262	0.011295	0.011329	0.011296	0.011228	0.011262
0.011262	0.011329	0.011228	0.011329	0.011363	0.011396	0.011295
0.011329	0.011296	0.011598	0.011598	0.011363	0.011397	0.011430
0.011195	0.011195	0.011228	0.011262	0.011195	0.011430	0.011430
0.011463	0.011463	0.011396	0.011497	0.011665	0.011498	0.011262
0.011329	0.011396	0.011363	0.011262	0.011329	0.011295	0.011329
0.011397	0.011396	0.011430	0.011329	0.011229	0.011497	0.011396
0.011331	0.011228	0.011329	0.011430	0.011329	0.011464	0.011497
0.011665	0.011368	0.011531	0.011430	0.011430	0.011598	0.011228
0.011397	0.011296	0.011262	0.011262	0.011329	0.011228	0.011329
0.011363	0.011396	0.011295	0.011329	0.011296	0.011598	0.011598
0.011363	0.011397	0.011463	0.011396	0.012037	0.011454	0.011377

0.011447	0.011407	0.011517	0.011407	0.011516	0.011440	0.011405
0.011988	0.011303	0.011437	0.011372	0.011646	0.011503	0.011537
0.012013	0.011475	0.011568	0.011631	0.011497	0.011497	0.011534
0.011195	0.011295	0.011497	0.011463	0.011466	0.011463	0.011397
0.011295	0.011161	0.011195	0.011295	0.011127	0.011262	0.011161
0.011295	0.011329	0.011228	0.011195	0.011295	0.011228	0.011262
0.011262	0.011195	0.011262	0.011295	0.011195	0.011329	0.011430
0.011329	0.011396	0.011329	0.011329	0.011262	0.011665	0.011295
0.011339	0.011262	0.011497	0.011502	0.011295	0.011363	0.011363
0.011363	0.011228	0.011262	0.011228	0.011396	0.011396	0.011329
0.011329	0.011463	0.011631	0.011362	0.011564	0.011564	0.011430
0.011262	0.011329	0.011363	0.011766	0.011329	0.011295	0.011329
0.011363	0.011295	0.011295	0.011598	0.011463	0.011564	0.011295
0.011396	0.011430	0.011329	0.011396	0.011363	0.011228	0.011564
0.011329	0.011295	0.011295	0.011463	0.011295	0.011396	0.011631
0.011329	0.011706	0.011699	0.011508	0.011230	0.011400	0.011215
0.011280	0.011684	0.011236	0.011418	0.011207	0.011366	0.011326
0.011469	0.011126	0.011283	0.011126	0.011168	0.011262	0.011304
0.011362	0.011178	0.011378	0.011307	0.011330	0.011463	0.011531
0.011531	0.011397	0.011500	0.011501	0.011637	0.011662	0.011329
0.011564	0.011430	0.011295	0.011396	0.011195	0.011430	0.011228
0.011295	0.011295	0.011262	0.011228	0.011262	0.011228	0.011329
0.011305	0.011305	0.011300	0.011334	0.011471	0.011300	0.011436
0.011435	0.011363	0.011134	0.011168	0.011298	0.011467	0.011434
0.011262	0.011331	0.011566	0.011534	0.011464	0.011498	0.011430
0.011363	0.011430	0.011430	0.011329	0.011901	0.011497	0.011564
0.011598	0.011666	0.011299	0.011372	0.011262	0.011463	0.011430
0.011464	0.011497	0.011497	0.011497	0.011651	0.011631	0.011269
0.011162	0.011164	0.011265	0.011230	0.011334	0.011172	0.011502
0.011301	0.011411	0.011156	0.011323	0.011296	0.011368	0.011195
0.011228	0.011228	0.011228	0.011262	0.011463	0.011430	0.011363
0.011463	0.011531	0.011262	0.011262	0.011295	0.011295	0.011297
0.011365	0.011295	0.011296	0.011330	0.011463	0.011531	0.011531
0.011397	0.011500	0.011501	0.011637	0.011662	0.011329	0.011497
0.011430	0.011430	0.011263	0.011443	0.011373	0.011401	0.011271
0.011370	0.011607	0.011399	0.011332	0.011161	0.011363	0.011228
0.011329	0.011329	0.011228	0.011330	0.011396	0.011432	0.011329
0.011228	0.011162	0.011295	0.011228	0.011161	0.011264	0.011363
0.011329	0.011262	0.011295	0.011329	0.011296	0.011228	0.011430
0.011295	0.011329	0.011329	0.011262	0.011329	0.011564	0.011228
0.011262	0.011665	0.011262	0.011228	0.011262	0.011631	0.011232
0.011567	0.011231	0.011261	0.011463	0.011549	0.011455	0.011186
0.011218	0.011396	0.011497	0.011604	0.011465	0.011602	0.011733
0.011633	0.011670	0.011631	0.011881	0.011598	0.012473	0.011634
0.012373	0.012140	0.012275	0.012580	0.011631	0.011329	0.011598
0.012071	0.011972	0.011564	0.011464	0.011464	0.011464	0.011500
0.011396	0.011400	0.011463	0.011430	0.011834	0.011867	0.011902
0.011801	0.011902	0.012005	0.011771	0.012071	0.012077	0.012138
0.012139	0.012173	0.012119	0.012139	0.012140	0.011362	0.011666
0.011734	0.012237	0.011739	0.011968	0.012442	0.011502	0.011397
0.011362	0.011362	0.011834	0.011883	0.011976	0.011699	0.011364
S026 0.011631	0.011631	0.011967	0.011732	0.011732	0.011799	
0.011637	0.011569	0.011799	0.011228	0.011295	0.011262	0.011228
0.011262	0.011429	0.011228	0.011262	0.011329	0.011295	0.011262
0.011228	0.011262	0.011362	0.011228	0.011262	0.000000	0.011603
0.011496	0.011262	0.011161	0.011396	0.011329	0.011295	0.011329
0.011295	0.011396	0.011295	0.011295	0.011329	0.011295	0.011463
0.011228	0.011295	0.011329	0.011262	0.011362	0.011396	0.011329
0.011295	0.011463	0.011362	0.011631	0.011295	0.011564	0.011295
0.011262	0.011362	0.011429	0.011564	0.011664	0.011362	0.011463

0.011329	0.011396	0.011295	0.011530	0.011765	0.011295	0.011362
0.011262	0.011362	0.011329	0.011396	0.011329	0.011329	0.011329
0.011228	0.011262	0.011228	0.011329	0.011295	0.011329	0.011396
0.011329	0.011295	0.011362	0.011262	0.011396	0.011228	0.011295
0.011463	0.011396	0.011631	0.011463	0.011295	0.011530	0.011295
0.011431	0.011658	0.011437	0.011138	0.011135	0.011281	0.011336
0.012011	0.011416	0.011143	0.011530	0.011463	0.011329	0.011429
0.011362	0.011329	0.011396	0.011295	0.011396	0.011329	0.011329
0.011295	0.011396	0.011597	0.011463	0.011362	0.011329	0.011262
0.011228	0.011228	0.011295	0.011262	0.011463	0.011429	0.011362
0.011463	0.011530	0.011262	0.011262	0.011295	0.011295	0.011297
0.011364	0.011295	0.011295	0.011168	0.011205	0.011200	0.011296
0.011297	0.011232	0.011208	0.011267	0.011196	0.011232	0.011263
0.011528	0.011301	0.011336	0.011228	0.011233	0.011363	0.011329
0.011396	0.011330	0.011329	0.011429	0.011362	0.011732	0.011530
0.011564	0.011430	0.011295	0.011396	0.011195	0.011429	0.011228
0.011295	0.011295	0.011262	0.011228	0.011262	0.011228	0.011329
0.011295	0.011396	0.011329	0.011228	0.011330	0.011430	0.011432
0.011430	0.011329	0.011228	0.011161	0.011295	0.011228	0.011161
0.011264	0.011430	0.011228	0.011430	0.011161	0.011295	0.011262
0.011362	0.011262	0.011295	0.011262	0.011295	0.011161	0.011295
0.011329	0.011262	0.011329	0.011262	0.011295	0.011329	0.011564
0.011664	0.011597	0.011665	0.011397	0.011665	0.011295	0.011262
0.011297	0.011545	0.011426	0.011163	0.011671	0.011163	0.011257
0.011671	0.011570	0.011396	0.011664	0.011368	0.011530	0.011497
0.011429	0.011597	0.011451	0.011307	0.011354	0.011329	0.011398
0.011497	0.011397	0.011533	0.011601	0.011530	0.011597	0.011704
0.011432	0.011603	0.011534	0.011466	0.011733	0.011194	0.011397
0.011362	0.011295	0.011194	0.011194	0.011262	0.011262	0.011329
0.011262	0.011329	0.011228	0.011329	0.011262	0.011262	0.011329
0.011262	0.011698	0.011597	0.011631	0.011161	0.011262	0.011295
0.011295	0.011262	0.011329	0.011362	0.011228	0.011228	0.011228
0.011765	0.011396	0.011396	0.011194	0.011295	0.011463	0.011228
0.011228	0.011295	0.011262	0.011429	0.011228	0.011362	0.011496
0.011429	0.011262	0.011262	0.011194	0.011194	0.011362	0.011295
0.011194	0.011295	0.011161	0.011195	0.011329	0.011463	0.011228
0.011195	0.011329	0.011262	0.011161	0.011496	0.011161	0.011329
0.011402	0.011228	0.011530	0.011362	0.011396	0.011262	0.011362
0.011228	0.011564	0.011161	0.011429	0.011228	0.011228	0.011161
0.011396	0.011564	0.011295	0.011362	0.011194	0.011597	0.011228
0.011228	0.011228	0.011262	0.011362	0.011262	0.011362	0.011429
0.011429	0.011396	0.011295	0.011396	0.011362	0.011228	0.011194
0.011228	0.011228	0.011295	0.011228	0.011194	0.011362	0.011228
0.011262	0.011262	0.011396	0.011429	0.011329	0.011262	0.011262
0.011329	0.011262	0.011362	0.011228	0.011362	0.011463	0.011765
0.011262	0.011664	0.011233	0.017747	0.011126	0.011427	0.011583
0.011545	0.011588	0.011217	0.011176	0.011664	0.011612	0.011303
0.011392	0.011487	0.011099	0.011228	0.011138	0.011258	0.011232
0.011436	0.011358	0.011394	0.011126	0.011396	0.011429	0.011432
0.011301	0.011396	0.011396	0.011362	0.011329	0.011329	0.011295
0.011329	0.011262	0.011295	0.011329	0.011295	0.011228	0.011262
0.011262	0.011329	0.011228	0.011329	0.011362	0.011396	0.011295
0.011329	0.011295	0.011631	0.011631	0.011362	0.011396	0.011430
0.011195	0.011195	0.011228	0.011329	0.011195	0.011429	0.011429
0.011463	0.011396	0.011329	0.011497	0.011665	0.011498	0.011329
0.011329	0.011396	0.011429	0.011262	0.011329	0.011295	0.011329
0.011396	0.011396	0.011429	0.011396	0.011296	0.011497	0.011463
0.011331	0.011228	0.011329	0.011430	0.011329	0.011463	0.011496
0.011664	0.011368	0.011530	0.011497	0.011429	0.011597	0.011228
0.011397	0.011295	0.011262	0.011262	0.011329	0.011228	0.011329

0.011362	0.011396	0.011295	0.011329	0.011295	0.011631	0.011631
0.011362	0.011396	0.011463	0.011396	0.012035	0.011453	0.011377
0.011447	0.011407	0.011517	0.011406	0.011515	0.011439	0.011405
0.011987	0.011302	0.011437	0.011371	0.011645	0.011502	0.011604
0.012012	0.011475	0.011567	0.011631	0.011496	0.011497	0.011533
0.011194	0.011362	0.011496	0.011463	0.011466	0.011463	0.011431
0.011295	0.011228	0.011262	0.011295	0.011194	0.011262	0.011161
0.011295	0.011329	0.011228	0.011194	0.011295	0.011228	0.011262
0.011262	0.011194	0.011262	0.011295	0.011194	0.011396	0.011429
0.011329	0.011396	0.011329	0.011329	0.011262	0.011664	0.011295
0.011338	0.011262	0.011497	0.011501	0.011362	0.011362	0.011362
0.011362	0.011295	0.011262	0.011295	0.011396	0.011396	0.011329
0.011329	0.011463	0.011631	0.011362	0.011564	0.011564	0.011429
0.011262	0.011329	0.011362	0.011765	0.011329	0.011362	0.011329
0.011362	0.011295	0.011295	0.011597	0.011530	0.011564	0.011295
0.011396	0.011463	0.011329	0.011396	0.011362	0.011228	0.011564
0.011329	0.011295	0.011295	0.011463	0.011295	0.011396	0.011631
0.011329	0.011705	0.011698	0.011426	0.011117	0.011400	0.011163
0.011225	0.011644	0.011201	0.011373	0.011207	0.011264	0.011273
0.011366	0.000000	0.011227	0.000000	0.011168	0.011261	0.011304
0.011362	0.011218	0.011342	0.011272	0.011330	0.011463	0.011530
0.011530	0.011396	0.011500	0.011501	0.011636	0.011661	0.011329
0.011564	0.011430	0.011295	0.011396	0.011195	0.011429	0.011228
0.011295	0.011295	0.011262	0.011228	0.011262	0.011228	0.011329
0.011305	0.011305	0.011300	0.011334	0.011471	0.011436	0.011436
0.011435	0.011363	0.011171	0.011168	0.011297	0.011467	0.011467
0.011262	0.011331	0.011565	0.011533	0.011464	0.011498	0.011429
0.011362	0.011429	0.011429	0.011329	0.011900	0.011496	0.011564
0.011597	0.011630	0.011299	0.011372	0.011262	0.011463	0.011429
0.011464	0.011496	0.011496	0.011496	0.011650	0.011631	0.011268
0.011162	0.011164	0.011264	0.011230	0.011333	0.011172	0.011502
0.011300	0.011455	0.011203	0.011322	0.011296	0.011368	0.011262
0.011228	0.011228	0.011295	0.011262	0.011463	0.011429	0.011362
0.011463	0.011530	0.011262	0.011262	0.011295	0.011295	0.011297
0.011364	0.011295	0.011295	0.011330	0.011463	0.011530	0.011530
0.011396	0.011500	0.011501	0.011636	0.011661	0.011329	0.011496
0.011429	0.011429	0.011263	0.011443	0.011373	0.011401	0.011235
0.011439	0.011607	0.011441	0.011332	0.011228	0.011430	0.011295
0.011396	0.011329	0.011228	0.011330	0.011430	0.011432	0.011329
0.011228	0.011161	0.011295	0.011228	0.011161	0.011264	0.011430
0.011329	0.011262	0.011295	0.011329	0.011295	0.011228	0.011430
0.011295	0.011329	0.011329	0.011262	0.011329	0.011564	0.011228
0.011262	0.011664	0.011262	0.011295	0.011262	0.011631	0.011146
0.011566	0.011145	0.011211	0.011382	0.011467	0.011419	0.011102
0.011113	0.011429	0.011530	0.011637	0.011498	0.011601	0.011766
0.011666	0.011703	0.011664	0.011880	0.011631	0.012505	0.011667
0.012405	0.012173	0.012307	0.012578	0.011631	0.011329	0.011631
0.012070	0.011971	0.011597	0.011497	0.011565	0.011565	0.011534
0.011429	0.011399	0.011496	0.011464	0.011833	0.011900	0.011901
0.011800	0.011901	0.012004	0.011737	0.012070	0.012076	0.012137
0.012138	0.012172	0.012084	0.012138	0.012139	0.011362	0.011665
0.011733	0.012236	0.011738	0.011967	0.012440	0.011502	0.011396
0.011362	0.011362	0.011833	0.011883	0.011975	0.011698	0.011363
S027 0.011977	0.011977	0.012011	0.011773	0.011773	0.011841	
0.011196	0.011128	0.011807	0.011570	0.011637	0.011605	0.011535
0.011501	0.011773	0.011501	0.011603	0.011637	0.011603	0.011603
0.011569	0.011603	0.011434	0.011570	0.011604	0.011603	0.000000
0.011569	0.011603	0.011501	0.011468	0.011671	0.011637	0.011671
0.011637	0.011739	0.011637	0.011637	0.011671	0.011637	0.011807
0.011501	0.011637	0.011671	0.011603	0.011637	0.011739	0.011671

0.011637	0.011807	0.011672	0.011298	0.011637	0.011875	0.011637
0.011603	0.011638	0.011773	0.011875	0.011400	0.011637	0.011739
0.011671	0.011739	0.011637	0.011875	0.011434	0.011637	0.011705
0.011603	0.011705	0.011671	0.011739	0.011603	0.011671	0.011671
0.011535	0.011603	0.011569	0.011603	0.011637	0.011639	0.011705
0.011671	0.011603	0.011705	0.011569	0.011467	0.011569	0.011637
0.011773	0.011739	0.011501	0.011807	0.011603	0.011841	0.011637
0.011806	0.011286	0.011819	0.011532	0.011551	0.011694	0.011417
0.012098	0.011419	0.011557	0.011875	0.011875	0.011603	0.011467
0.011671	0.011671	0.011739	0.011637	0.011739	0.011671	0.011603
0.011569	0.011637	0.011943	0.011807	0.011671	0.011672	0.011604
0.011570	0.011502	0.011638	0.011570	0.011536	0.011502	0.011434
0.011536	0.011604	0.011604	0.011604	0.011638	0.011638	0.011641
0.011710	0.011604	0.011638	0.011497	0.011573	0.011521	0.011604
0.011608	0.011547	0.011621	0.011584	0.011541	0.011545	0.011607
0.011498	0.011618	0.011653	0.011570	0.011515	0.011673	0.011604
0.011639	0.011674	0.011672	0.011739	0.011705	0.012011	0.011774
0.011637	0.011706	0.011637	0.011672	0.011536	0.011502	0.011570
0.011637	0.011637	0.011604	0.011569	0.011603	0.011570	0.011672
0.011638	0.011740	0.011672	0.011570	0.011674	0.011774	0.011778
0.011774	0.011638	0.011570	0.011504	0.011638	0.011570	0.011502
0.011507	0.011774	0.011570	0.011774	0.011535	0.011638	0.011604
0.011672	0.011570	0.011604	0.011604	0.011638	0.011502	0.011638
0.011672	0.011604	0.011638	0.011570	0.011604	0.011672	0.011910
0.011332	0.011264	0.011332	0.011776	0.011400	0.011638	0.011604
0.011573	0.011919	0.011663	0.011546	0.011335	0.011509	0.011698
0.011298	0.011576	0.011807	0.012011	0.011717	0.011875	0.011841
0.011773	0.011943	0.011779	0.011729	0.011698	0.011672	0.011745
0.011841	0.011708	0.011881	0.011950	0.011841	0.011909	0.011951
0.011745	0.011953	0.011814	0.011813	0.012081	0.011535	0.011742
0.011671	0.011637	0.011535	0.011535	0.011603	0.011603	0.011671
0.011603	0.011671	0.011569	0.011603	0.011603	0.011569	0.011671
0.011603	0.011366	0.011264	0.011298	0.011501	0.011603	0.011603
0.011637	0.011535	0.011671	0.011637	0.011569	0.011569	0.011569
0.011366	0.011671	0.011739	0.011535	0.011637	0.011807	0.011569
0.011569	0.011603	0.011603	0.011773	0.011569	0.011502	0.011637
0.011570	0.011603	0.011603	0.011535	0.011535	0.011705	0.011637
0.011535	0.011637	0.011502	0.011535	0.011671	0.011468	0.011570
0.011536	0.011671	0.011570	0.011502	0.011230	0.011502	0.011671
0.011753	0.011569	0.011196	0.011706	0.011671	0.011569	0.011705
0.011569	0.011875	0.011501	0.011502	0.011501	0.011501	0.011501
0.011739	0.011604	0.011637	0.011705	0.011535	0.011264	0.011501
0.011569	0.011569	0.011603	0.011705	0.011603	0.011705	0.011773
0.011773	0.011739	0.011637	0.011739	0.011705	0.011569	0.011535
0.011569	0.011569	0.011637	0.011501	0.011535	0.011705	0.011569
0.011535	0.011603	0.011739	0.011705	0.011671	0.011603	0.011604
0.011637	0.011603	0.011671	0.011569	0.011705	0.011807	0.011501
0.011603	0.011400	0.011619	0.018216	0.011391	0.011616	0.011272
0.011235	0.011274	0.011799	0.011528	0.011448	0.011305	0.011735
0.011634	0.011491	0.011492	0.011608	0.011535	0.011558	0.011580
0.011344	0.011475	0.011323	0.011463	0.011739	0.011773	0.011779
0.011651	0.011740	0.011739	0.011638	0.011637	0.011638	0.011638
0.011637	0.011569	0.011637	0.011672	0.011638	0.011570	0.011604
0.011604	0.011672	0.011570	0.011672	0.011706	0.011740	0.011637
0.011569	0.011570	0.011366	0.011366	0.011706	0.011741	0.011774
0.011536	0.011536	0.011570	0.011672	0.011536	0.011774	0.011774
0.011807	0.011807	0.011739	0.011841	0.011400	0.011844	0.011671
0.011672	0.011740	0.011774	0.011604	0.011672	0.011638	0.011671
0.011740	0.011740	0.011774	0.011740	0.011639	0.011841	0.011774
0.011677	0.011570	0.011672	0.011774	0.011672	0.011740	0.011841

0.012011	0.011717	0.011875	0.011841	0.011773	0.011943	0.011570
0.011742	0.011638	0.011604	0.011604	0.011672	0.011570	0.011672
0.011706	0.011740	0.011637	0.011569	0.011570	0.011366	0.011366
0.011706	0.011741	0.011807	0.011739	0.012217	0.011820	0.011736
0.011807	0.011762	0.011878	0.011761	0.011875	0.011793	0.011688
0.012172	0.011656	0.011788	0.011725	0.011931	0.011852	0.011955
0.012195	0.011786	0.011915	0.011977	0.011841	0.011570	0.011881
0.011535	0.011705	0.011841	0.011807	0.011812	0.011535	0.011503
0.011637	0.011569	0.011603	0.011637	0.011535	0.011603	0.011501
0.011569	0.011603	0.011569	0.011535	0.011637	0.011569	0.011603
0.011603	0.011535	0.011603	0.011637	0.011535	0.011739	0.011773
0.011671	0.011671	0.011671	0.011671	0.011603	0.011400	0.011637
0.011412	0.011603	0.011570	0.011575	0.011637	0.011637	0.011705
0.011705	0.011637	0.011603	0.011637	0.011739	0.011739	0.011637
0.011671	0.011807	0.011298	0.011705	0.011909	0.011909	0.011671
0.011603	0.011671	0.011705	0.011501	0.011672	0.011637	0.011671
0.011705	0.011569	0.011569	0.011875	0.011875	0.011841	0.011637
0.011705	0.011739	0.011671	0.011705	0.011671	0.011569	0.011196
0.011671	0.011603	0.011569	0.011807	0.011637	0.011739	0.011468
0.011671	0.011264	0.011366	0.011510	0.011402	0.011679	0.011424
0.011448	0.011726	0.011558	0.011645	0.011601	0.011419	0.011381
0.011523	0.011326	0.011509	0.011394	0.011386	0.011602	0.011754
0.011742	0.011654	0.011666	0.011666	0.011674	0.011536	0.011604
0.011604	0.011672	0.011779	0.011575	0.011918	0.011954	0.011671
0.011637	0.011706	0.011637	0.011672	0.011536	0.011502	0.011570
0.011637	0.011637	0.011604	0.011569	0.011603	0.011570	0.011672
0.011626	0.011627	0.011545	0.011615	0.011752	0.011717	0.011716
0.011715	0.011672	0.011508	0.011497	0.011608	0.011711	0.011781
0.011604	0.011643	0.011878	0.011881	0.011809	0.011843	0.011773
0.011705	0.011773	0.011773	0.011671	0.011637	0.011841	0.011875
0.011910	0.011312	0.011578	0.011726	0.011603	0.011773	0.011773
0.011741	0.011841	0.011841	0.011841	0.011937	0.011977	0.011622
0.011505	0.011516	0.011611	0.011576	0.011613	0.011551	0.011163
0.011650	0.011372	0.011544	0.011585	0.011638	0.011683	0.011604
0.011570	0.011502	0.011638	0.011570	0.011536	0.011502	0.011434
0.011536	0.011604	0.011604	0.011604	0.011638	0.011638	0.011641
0.011710	0.011604	0.011638	0.011674	0.011536	0.011604	0.011604
0.011672	0.011779	0.011575	0.011918	0.011954	0.011671	0.011841
0.011773	0.011773	0.011608	0.011800	0.011693	0.011715	0.011628
0.011792	0.011986	0.011833	0.011679	0.011570	0.011774	0.011638
0.011740	0.011672	0.011570	0.011674	0.011774	0.011778	0.011638
0.011570	0.011504	0.011638	0.011570	0.011502	0.011507	0.011774
0.011637	0.011569	0.011637	0.011672	0.011638	0.011570	0.011502
0.011637	0.011671	0.011637	0.011603	0.011671	0.011909	0.011501
0.011603	0.011400	0.011603	0.011637	0.011603	0.011298	0.011578
0.011854	0.011402	0.011414	0.011509	0.011552	0.011533	0.011355
0.011378	0.011502	0.011603	0.011713	0.011571	0.011711	0.011843
0.011741	0.011745	0.011739	0.011925	0.011671	0.012420	0.011708
0.012455	0.012152	0.012288	0.012631	0.011603	0.011604	0.011706
0.012252	0.012016	0.011672	0.011536	0.011639	0.011639	0.011539
0.011502	0.011506	0.011569	0.011536	0.011911	0.011944	0.011979
0.011843	0.011945	0.012084	0.011813	0.012252	0.012259	0.012320
0.012321	0.012321	0.012269	0.012287	0.012289	0.011705	0.011876
0.012013	0.012488	0.011918	0.011944	0.012422	0.011507	0.011400
0.011298	0.011366	0.011910	0.011894	0.012022	0.011705	0.011435
S028 0.011866	0.011866	0.011933	0.011698	0.011698	0.011765	
0.011604	0.011536	0.011765	0.011463	0.011530	0.011498	0.011463
0.011429	0.011664	0.011396	0.011496	0.011564	0.011530	0.011496
0.011463	0.011496	0.011329	0.011463	0.011497	0.011496	0.011569
0.000000	0.011496	0.011396	0.011362	0.011564	0.011530	0.011564

0.011530	0.011631	0.011530	0.011530	0.011564	0.011530	0.011698
0.011396	0.011530	0.011564	0.011496	0.011530	0.011631	0.011564
0.011530	0.011698	0.011598	0.011597	0.011530	0.011799	0.011530
0.011496	0.011530	0.011597	0.011799	0.011631	0.011530	0.011631
0.011564	0.011631	0.011530	0.011765	0.011732	0.011530	0.011597
0.011496	0.011597	0.011564	0.011631	0.011564	0.011564	0.011564
0.011463	0.011496	0.011463	0.011496	0.011530	0.011565	0.011631
0.011564	0.011530	0.011597	0.011496	0.011564	0.011463	0.011530
0.011698	0.011631	0.011664	0.011698	0.011530	0.011731	0.011530
0.011728	0.011658	0.011739	0.011490	0.011433	0.011576	0.011336
0.011975	0.011416	0.011472	0.011765	0.011765	0.011496	0.011597
0.011597	0.011564	0.011631	0.011530	0.011631	0.011564	0.011564
0.011530	0.011564	0.011832	0.011698	0.011597	0.011564	0.011497
0.011463	0.011396	0.011530	0.011497	0.011430	0.011396	0.011329
0.011430	0.011497	0.011497	0.011497	0.011530	0.011530	0.011533
0.011601	0.011530	0.011530	0.011420	0.011458	0.011446	0.011531
0.011534	0.011473	0.011503	0.011509	0.011434	0.011471	0.011499
0.011492	0.011543	0.011542	0.011464	0.011475	0.011598	0.011497
0.011565	0.011566	0.011564	0.011664	0.011597	0.011900	0.011698
0.011429	0.011597	0.011530	0.011564	0.011429	0.011161	0.011463
0.011530	0.011530	0.011497	0.011463	0.011496	0.011463	0.011564
0.011530	0.011631	0.011564	0.011463	0.011566	0.011665	0.011669
0.011665	0.011564	0.011463	0.011397	0.011530	0.011463	0.011396
0.011400	0.011665	0.011463	0.011665	0.011429	0.011530	0.011497
0.011597	0.011497	0.011530	0.011497	0.011530	0.011396	0.011530
0.011564	0.011497	0.011564	0.011497	0.011530	0.011564	0.011799
0.011631	0.011564	0.011631	0.011667	0.011631	0.011530	0.011497
0.011466	0.011748	0.011609	0.011437	0.011637	0.011401	0.011493
0.011637	0.011536	0.011698	0.011900	0.011607	0.011765	0.011732
0.011664	0.011832	0.011664	0.011555	0.011605	0.011564	0.011635
0.011732	0.011633	0.011770	0.011838	0.011765	0.011833	0.011941
0.011669	0.011841	0.011771	0.011703	0.011969	0.011429	0.011633
0.011597	0.011530	0.011429	0.011429	0.011496	0.011496	0.011564
0.011496	0.011564	0.011463	0.011496	0.011463	0.011496	0.011564
0.011496	0.011664	0.011564	0.011597	0.011396	0.011496	0.011530
0.011530	0.011496	0.011564	0.011530	0.011463	0.011463	0.011463
0.011732	0.011564	0.011631	0.011429	0.011530	0.011698	0.011463
0.011463	0.011530	0.011496	0.011664	0.011463	0.011396	0.011530
0.011463	0.011496	0.011496	0.011429	0.011429	0.011597	0.011530
0.011429	0.011530	0.011396	0.011429	0.011564	0.011430	0.011463
0.011430	0.011564	0.011497	0.011396	0.011463	0.011396	0.011564
0.011608	0.011463	0.011497	0.011597	0.011564	0.011496	0.011597
0.011463	0.011799	0.011396	0.011597	0.011396	0.011396	0.011396
0.011631	0.011564	0.011530	0.011597	0.011429	0.011564	0.011396
0.011463	0.011463	0.011496	0.011597	0.011496	0.011597	0.011664
0.011664	0.011631	0.011530	0.011631	0.011597	0.011463	0.011429
0.011463	0.011463	0.011530	0.011396	0.011429	0.011597	0.011463
0.011429	0.011496	0.011631	0.011597	0.011564	0.011496	0.011497
0.011564	0.011496	0.011597	0.011463	0.011597	0.011698	0.011732
0.011496	0.011631	0.011509	0.018012	0.011453	0.011733	0.011583
0.011545	0.011588	0.011689	0.011466	0.011720	0.011613	0.011728
0.011582	0.011427	0.011450	0.011521	0.011450	0.011653	0.011576
0.011436	0.011246	0.011394	0.011193	0.011631	0.011665	0.011669
0.011541	0.011631	0.011631	0.011530	0.011564	0.011564	0.011530
0.011564	0.011496	0.011530	0.011564	0.011530	0.011463	0.011497
0.011497	0.011564	0.011463	0.011564	0.011597	0.011631	0.011530
0.011496	0.011463	0.011598	0.011598	0.011597	0.011632	0.011665
0.011429	0.011429	0.011463	0.011564	0.011429	0.011665	0.011665
0.011698	0.011698	0.011631	0.011732	0.011631	0.011734	0.011564
0.011564	0.011631	0.011665	0.011497	0.011564	0.011530	0.011564

0.011631	0.011631	0.011665	0.011631	0.011532	0.011732	0.011698
0.011568	0.011463	0.011564	0.011665	0.011564	0.011631	0.011731
0.011900	0.011607	0.011765	0.011732	0.011664	0.011832	0.011463
0.011633	0.011531	0.011497	0.011497	0.011564	0.011463	0.011564
0.011597	0.011631	0.011530	0.011496	0.011463	0.011598	0.011598
0.011597	0.011632	0.011698	0.011631	0.012204	0.011703	0.011588
0.011693	0.011650	0.011763	0.011614	0.011760	0.011681	0.011646
0.012159	0.011545	0.011677	0.011613	0.011887	0.011740	0.011843
0.012148	0.011625	0.011804	0.011866	0.011732	0.011463	0.011770
0.011429	0.011597	0.011731	0.011698	0.011702	0.011194	0.011397
0.011530	0.011463	0.011496	0.011530	0.011429	0.011496	0.011396
0.011463	0.011496	0.011463	0.011429	0.011530	0.011463	0.011496
0.011496	0.011429	0.011496	0.011530	0.011429	0.011631	0.011664
0.011564	0.011564	0.011564	0.011564	0.011496	0.011631	0.011530
0.011304	0.011497	0.000000	0.000000	0.011597	0.011597	0.011597
0.011597	0.011530	0.011496	0.011530	0.011631	0.011631	0.011564
0.011564	0.011698	0.011597	0.011597	0.011799	0.011799	0.011664
0.011496	0.011564	0.011597	0.011732	0.011564	0.011597	0.011564
0.011597	0.011463	0.011463	0.011765	0.011765	0.011732	0.011530
0.011631	0.011698	0.011564	0.011631	0.011597	0.011463	0.011530
0.011564	0.011530	0.011463	0.011698	0.011530	0.011631	0.011665
0.011564	0.011671	0.011664	0.011223	0.011342	0.011570	0.011267
0.011555	0.011644	0.011446	0.011641	0.011499	0.011518	0.011485
0.011264	0.011455	0.011561	0.011456	0.011438	0.011663	0.011628
0.011663	0.011532	0.011553	0.011553	0.011566	0.011195	0.011262
0.011497	0.011564	0.011669	0.011467	0.011806	0.011838	0.011564
0.011429	0.011597	0.011530	0.011564	0.011429	0.011161	0.011463
0.011530	0.011530	0.011497	0.011463	0.011496	0.011463	0.011564
0.011549	0.011550	0.011540	0.011574	0.011710	0.011675	0.011675
0.011673	0.011598	0.011429	0.011420	0.011534	0.011704	0.011705
0.011497	0.011568	0.011801	0.011770	0.011699	0.011733	0.011664
0.011597	0.011664	0.011664	0.011564	0.011866	0.011732	0.011799
0.011833	0.011630	0.011538	0.011580	0.011497	0.011698	0.011664
0.011699	0.011731	0.011731	0.011731	0.011886	0.011866	0.011511
0.011398	0.011406	0.011503	0.011468	0.011504	0.011433	0.011468
0.011540	0.011367	0.011441	0.011547	0.011531	0.011608	0.011497
0.011463	0.011396	0.011530	0.011497	0.011430	0.011396	0.011329
0.011430	0.011497	0.011497	0.011497	0.011530	0.011530	0.011533
0.011601	0.011530	0.011530	0.011566	0.011195	0.011262	0.011497
0.011564	0.011669	0.011467	0.011806	0.011838	0.011564	0.011732
0.011664	0.011664	0.011500	0.011687	0.011616	0.011639	0.011516
0.011680	0.011789	0.011695	0.011570	0.011463	0.011665	0.011530
0.011631	0.011564	0.011463	0.011566	0.011665	0.011669	0.011564
0.011463	0.011397	0.011530	0.011463	0.011396	0.011400	0.011665
0.011564	0.011496	0.011530	0.011564	0.011530	0.011463	0.011396
0.011530	0.011564	0.011564	0.011496	0.011564	0.011799	0.011396
0.011496	0.011631	0.011496	0.011530	0.011496	0.011597	0.011489
0.011735	0.011358	0.011361	0.011181	0.011264	0.011204	0.011311
0.011323	0.011396	0.011497	0.011603	0.011464	0.011602	0.011733
0.011632	0.011669	0.011631	0.011880	0.011597	0.012472	0.011633
0.012372	0.012139	0.012274	0.012545	0.011597	0.011497	0.011598
0.012238	0.011971	0.011564	0.011463	0.011531	0.011531	0.011500
0.011396	0.011399	0.011463	0.011430	0.011800	0.011866	0.011867
0.011767	0.011867	0.011971	0.011737	0.012239	0.012246	0.012306
0.012307	0.012340	0.012289	0.012307	0.012308	0.011597	0.011833
0.011901	0.012404	0.011908	0.011934	0.012407	0.011468	0.011363
0.011329	0.011329	0.011800	0.011801	0.011940	0.011664	0.011330
S029	0.011631	0.011631	0.011967	0.011732	0.011732	0.011799
0.011637	0.011569	0.011799	0.011228	0.011295	0.011262	0.011228
0.011262	0.011429	0.011228	0.011262	0.011329	0.011295	0.011262

0.011228	0.011262	0.011362	0.011228	0.011262	0.011262	0.011603
0.011496	0.000000	0.011161	0.011396	0.011329	0.011295	0.011329
0.011295	0.011396	0.011295	0.011295	0.011329	0.011295	0.011463
0.011228	0.011295	0.011329	0.011262	0.011362	0.011396	0.011329
0.011295	0.011463	0.011362	0.011631	0.011295	0.011564	0.011295
0.011262	0.011362	0.011429	0.011564	0.011664	0.011362	0.011463
0.011329	0.011396	0.011295	0.011530	0.011765	0.011295	0.011362
0.011262	0.011362	0.011329	0.011396	0.011329	0.011329	0.011329
0.011228	0.011262	0.011228	0.011329	0.011295	0.011329	0.011396
0.011329	0.011295	0.011362	0.011262	0.011396	0.011228	0.011295
0.011463	0.011396	0.011631	0.011463	0.011295	0.011530	0.011295
0.011468	0.011695	0.011513	0.011216	0.011210	0.011281	0.011415
0.012048	0.011518	0.011225	0.011530	0.011530	0.011329	0.011429
0.011362	0.011329	0.011396	0.011295	0.011396	0.011329	0.011329
0.011295	0.011396	0.011597	0.011463	0.011362	0.011329	0.011262
0.011228	0.011228	0.011295	0.011262	0.011463	0.011430	0.011362
0.011463	0.011530	0.011262	0.011262	0.011295	0.011295	0.011297
0.011364	0.011295	0.011295	0.011168	0.011205	0.000000	0.011296
0.011297	0.011232	0.000000	0.000000	0.011196	0.011232	0.011263
0.011528	0.011302	0.011336	0.011228	0.011233	0.011363	0.011329
0.011397	0.011330	0.011329	0.011429	0.011362	0.011732	0.011530
0.011564	0.011430	0.011295	0.011396	0.011195	0.011429	0.011228
0.011295	0.011295	0.011262	0.011228	0.011262	0.011228	0.011329
0.011295	0.011396	0.011329	0.011228	0.011330	0.011430	0.011432
0.011396	0.011329	0.011228	0.011161	0.011295	0.011228	0.011161
0.011264	0.011396	0.011228	0.011396	0.011195	0.011295	0.011262
0.011362	0.011262	0.011295	0.011262	0.011295	0.011161	0.011295
0.011329	0.011262	0.011329	0.011262	0.011295	0.011329	0.011564
0.011665	0.011597	0.011665	0.011431	0.011665	0.011295	0.011262
0.011297	0.011586	0.011380	0.011197	0.011671	0.011163	0.011296
0.011671	0.011570	0.011463	0.011664	0.011368	0.011530	0.011497
0.011429	0.011597	0.011415	0.011225	0.011312	0.011329	0.011365
0.011497	0.011397	0.011533	0.011602	0.011530	0.011597	0.011704
0.011432	0.011603	0.011534	0.011466	0.011733	0.011194	0.011397
0.011362	0.011295	0.011194	0.011194	0.011262	0.011262	0.011329
0.011262	0.011329	0.011228	0.011329	0.011262	0.011262	0.011329
0.011262	0.011698	0.011597	0.011631	0.011161	0.011262	0.011295
0.011295	0.011262	0.011329	0.011362	0.011228	0.011228	0.011228
0.011765	0.011396	0.011396	0.011195	0.011295	0.011463	0.011228
0.011228	0.011295	0.011262	0.011429	0.011228	0.011362	0.011496
0.011429	0.011262	0.011262	0.011194	0.011194	0.011362	0.011295
0.011194	0.011295	0.011161	0.011195	0.011329	0.011463	0.011228
0.011195	0.011329	0.011262	0.011161	0.011496	0.011161	0.011329
0.011403	0.011228	0.011530	0.011362	0.011396	0.011262	0.011362
0.011228	0.011564	0.011161	0.011429	0.011228	0.011228	0.011161
0.011396	0.011598	0.011295	0.011362	0.011194	0.011597	0.011228
0.011228	0.011228	0.011262	0.011362	0.011262	0.011362	0.011429
0.011429	0.011396	0.011295	0.011396	0.011362	0.011228	0.011195
0.011228	0.011228	0.011295	0.011228	0.011195	0.011362	0.011228
0.011262	0.011262	0.011396	0.011429	0.011329	0.011262	0.011262
0.011329	0.011262	0.011362	0.011228	0.011362	0.011463	0.011765
0.011262	0.011664	0.011267	0.017786	0.011257	0.011427	0.011618
0.011579	0.011623	0.011322	0.011292	0.011774	0.011647	0.011425
0.011487	0.011610	0.011177	0.011270	0.011255	0.011356	0.011275
0.011483	0.011433	0.011431	0.011193	0.011396	0.011429	0.011432
0.011266	0.011362	0.011396	0.011362	0.011329	0.011329	0.011295
0.011329	0.011262	0.011295	0.011329	0.011295	0.011228	0.011262
0.011262	0.011329	0.011228	0.011329	0.011362	0.011396	0.011295
0.011329	0.011295	0.011631	0.011631	0.011362	0.011396	0.011430
0.011195	0.011195	0.011228	0.011329	0.011195	0.011430	0.011430

0.011463	0.011463	0.011396	0.011497	0.011665	0.011498	0.011329
0.011329	0.011396	0.011430	0.011262	0.011329	0.011295	0.011329
0.011396	0.011396	0.011429	0.011396	0.011296	0.011497	0.011463
0.011331	0.011228	0.011329	0.011430	0.011329	0.011463	0.011496
0.011664	0.011368	0.011530	0.011497	0.011429	0.011597	0.011228
0.011397	0.011296	0.011262	0.011262	0.011329	0.011228	0.011329
0.011362	0.011396	0.011295	0.011329	0.011295	0.011631	0.011631
0.011362	0.011396	0.011463	0.011396	0.012036	0.011453	0.011342
0.011447	0.011407	0.011482	0.011406	0.011515	0.011439	0.011405
0.011987	0.011302	0.011437	0.011371	0.011645	0.011502	0.011605
0.012012	0.011437	0.011567	0.011631	0.011496	0.011497	0.011533
0.011194	0.011362	0.011496	0.011463	0.011466	0.011463	0.011431
0.011295	0.011228	0.011262	0.011295	0.011195	0.011262	0.011161
0.011295	0.011329	0.011228	0.011195	0.011295	0.011228	0.011262
0.011262	0.011194	0.011262	0.011295	0.011194	0.011396	0.011429
0.011329	0.011396	0.011329	0.011329	0.011262	0.011664	0.011295
0.011304	0.011262	0.011497	0.011501	0.011362	0.011362	0.011362
0.011362	0.011295	0.011262	0.011295	0.011396	0.011396	0.011329
0.011329	0.011463	0.011631	0.011362	0.011564	0.011564	0.011429
0.011262	0.011329	0.011362	0.011765	0.011329	0.011362	0.011329
0.011362	0.011295	0.011295	0.011597	0.011530	0.011564	0.011295
0.011396	0.011463	0.011329	0.011396	0.011362	0.011228	0.011564
0.011329	0.011295	0.011295	0.011463	0.011295	0.011396	0.011631
0.011329	0.011705	0.011698	0.011507	0.011229	0.011400	0.011267
0.011225	0.011722	0.011236	0.011462	0.011304	0.011417	0.011432
0.011468	0.011258	0.011339	0.011192	0.011276	0.011395	0.011385
0.011362	0.011296	0.011377	0.011307	0.011330	0.011463	0.011530
0.011530	0.011396	0.011500	0.011501	0.011636	0.011661	0.011329
0.011564	0.011430	0.011295	0.011396	0.011195	0.011429	0.011228
0.011295	0.011295	0.011262	0.011228	0.011262	0.011228	0.011329
0.011305	0.011305	0.011300	0.011334	0.011471	0.011436	0.011436
0.011435	0.011363	0.011171	0.011168	0.011297	0.011467	0.011467
0.011262	0.011331	0.011565	0.011533	0.011464	0.011498	0.011429
0.011362	0.011429	0.011429	0.011329	0.011900	0.011497	0.011564
0.011598	0.011665	0.011299	0.011372	0.011262	0.011463	0.011429
0.011464	0.011496	0.011497	0.011497	0.011611	0.011631	0.011234
0.011162	0.011130	0.000000	0.011230	0.011299	0.011135	0.011502
0.011300	0.011411	0.011108	0.011285	0.011296	0.011368	0.011262
0.011228	0.011228	0.011295	0.011262	0.011463	0.011430	0.011362
0.011463	0.011530	0.011262	0.011262	0.011295	0.011295	0.011297
0.011364	0.011295	0.011295	0.011330	0.011463	0.011530	0.011530
0.011396	0.011500	0.011501	0.011636	0.011661	0.011329	0.011496
0.011429	0.011429	0.011263	0.011443	0.011338	0.011401	0.011270
0.011439	0.011561	0.011441	0.011332	0.011228	0.011396	0.011295
0.011396	0.011329	0.011228	0.011330	0.011430	0.011432	0.011329
0.011228	0.011161	0.011295	0.011228	0.011161	0.011264	0.011396
0.011329	0.011262	0.011295	0.011329	0.011295	0.011228	0.011430
0.011295	0.011329	0.011329	0.011262	0.011329	0.011564	0.011228
0.011262	0.011664	0.011262	0.011295	0.011262	0.011631	0.011189
0.011679	0.011230	0.011261	0.011463	0.011549	0.011455	0.011186
0.011218	0.011429	0.011530	0.011637	0.011498	0.011635	0.011766
0.011666	0.011703	0.011664	0.011914	0.011631	0.012505	0.011667
0.012406	0.012173	0.012307	0.012579	0.011631	0.011329	0.011631
0.012002	0.012005	0.011597	0.011497	0.011565	0.011565	0.011534
0.011429	0.011433	0.011496	0.011464	0.011834	0.011900	0.011901
0.011800	0.011901	0.012004	0.011771	0.012070	0.012076	0.012137
0.012138	0.012172	0.012118	0.012138	0.012072	0.011362	0.011665
0.011733	0.012236	0.011738	0.011967	0.012440	0.011502	0.011396
0.011362	0.011362	0.011833	0.011842	0.011975	0.011698	0.011363

S030	0.011530	0.011530	0.011866	0.011631	0.011631	0.011698
0.011535	0.011468	0.011698	0.011127	0.011194	0.011161	0.011127
0.011161	0.011329	0.011127	0.011161	0.011228	0.011194	0.011161
0.011127	0.011161	0.011262	0.011127	0.011161	0.011161	0.011501
0.011396	0.011161	0.000000	0.011295	0.011228	0.011195	0.011228
0.011194	0.011295	0.011194	0.011194	0.011228	0.011194	0.011362
0.011127	0.011194	0.011228	0.011161	0.011262	0.011295	0.011228
0.011194	0.011362	0.011262	0.011530	0.011194	0.011463	0.011194
0.011161	0.011262	0.011329	0.011463	0.011564	0.011262	0.011362
0.011228	0.011295	0.011195	0.011429	0.011664	0.011194	0.011262
0.011161	0.011262	0.011228	0.011295	0.011228	0.011228	0.011228
0.011127	0.011161	0.011127	0.011228	0.011194	0.011228	0.011295
0.011228	0.011194	0.011262	0.011161	0.011295	0.011127	0.011194
0.011362	0.011295	0.011530	0.011362	0.011194	0.011429	0.011194
0.011357	0.011583	0.011400	0.011099	0.011098	0.011207	0.011296
0.011938	0.011365	0.011102	0.011429	0.011429	0.011228	0.011329
0.011262	0.011228	0.011295	0.011194	0.011295	0.011228	0.011228
0.011194	0.011295	0.011496	0.011362	0.011262	0.011228	0.011161
0.011127	0.011127	0.011195	0.011161	0.011362	0.011329	0.011262
0.011362	0.011429	0.011161	0.011161	0.011195	0.011195	0.011195
0.011263	0.011195	0.011195	0.000000	0.011133	0.011095	0.011195
0.011196	0.011129	0.011171	0.011164	0.011094	0.011129	0.011162
0.011420	0.011198	0.011232	0.011128	0.011129	0.011262	0.011228
0.011296	0.011229	0.011228	0.011329	0.011262	0.011631	0.011430
0.011463	0.011329	0.011195	0.011295	0.011094	0.011329	0.011127
0.011194	0.011194	0.011161	0.011127	0.011161	0.011127	0.011228
0.011195	0.011295	0.011228	0.011127	0.011229	0.011329	0.011331
0.011329	0.011228	0.011127	0.000000	0.011195	0.011127	0.000000
0.011162	0.011329	0.011127	0.011329	0.011094	0.011195	0.011161
0.011262	0.011161	0.011195	0.011161	0.011195	0.000000	0.011195
0.011228	0.011161	0.011228	0.011161	0.011195	0.011228	0.011463
0.011564	0.011496	0.011564	0.011330	0.011564	0.011195	0.011161
0.011195	0.011464	0.011289	0.011095	0.011569	0.000000	0.011178
0.011569	0.011468	0.011362	0.011564	0.011265	0.011429	0.011396
0.011329	0.011496	0.011344	0.011184	0.011228	0.011228	0.011297
0.011396	0.011296	0.011432	0.011500	0.011429	0.011497	0.011602
0.011331	0.011501	0.011432	0.011364	0.011632	0.011094	0.011296
0.011262	0.011194	0.011094	0.011094	0.011161	0.011161	0.011228
0.011161	0.011228	0.011127	0.011228	0.011161	0.011161	0.011228
0.011161	0.011597	0.011496	0.011530	0.000000	0.011161	0.011195
0.011194	0.011161	0.011228	0.011262	0.011127	0.011127	0.011127
0.011664	0.011295	0.011295	0.011094	0.011194	0.011362	0.011127
0.011127	0.011194	0.011161	0.011329	0.011127	0.011262	0.011396
0.011329	0.011161	0.011161	0.011094	0.011094	0.011262	0.011194
0.011094	0.011194	0.000000	0.011094	0.011228	0.011362	0.011127
0.011094	0.011228	0.011161	0.000000	0.011396	0.000000	0.011228
0.011300	0.011127	0.011429	0.011262	0.011295	0.011161	0.011262
0.011127	0.011463	0.000000	0.011329	0.011127	0.011127	0.000000
0.011295	0.011497	0.011195	0.011262	0.011094	0.011496	0.011127
0.011127	0.011127	0.011161	0.011262	0.011161	0.011262	0.011329
0.011329	0.011295	0.011194	0.011295	0.011262	0.011127	0.011094
0.011127	0.011127	0.011195	0.011127	0.011094	0.011262	0.011127
0.011161	0.011161	0.011295	0.011329	0.011228	0.011161	0.011161
0.011228	0.011161	0.011262	0.011127	0.011262	0.011362	0.011664
0.011161	0.011564	0.011164	0.017672	0.011126	0.011305	0.011514
0.011475	0.011517	0.011217	0.011176	0.011609	0.011543	0.011242
0.011392	0.011426	0.000000	0.011144	0.011138	0.011208	0.011146
0.011389	0.011321	0.011320	0.011126	0.011295	0.011329	0.011331
0.011198	0.011295	0.011295	0.011262	0.011228	0.011228	0.011195
0.011228	0.011161	0.011195	0.011228	0.011195	0.011127	0.011161

0.011161	0.011228	0.011127	0.011228	0.011262	0.011295	0.011195
0.011228	0.011195	0.011530	0.011530	0.011262	0.011296	0.011329
0.011094	0.011094	0.011127	0.011228	0.011094	0.011329	0.011329
0.011362	0.011362	0.011295	0.011396	0.011564	0.011397	0.011228
0.011228	0.011295	0.011329	0.011161	0.011228	0.011195	0.011228
0.011295	0.011295	0.011329	0.011295	0.011195	0.011396	0.011362
0.011230	0.011127	0.011228	0.011329	0.011228	0.011362	0.011396
0.011564	0.011265	0.011429	0.011396	0.011329	0.011496	0.011128
0.011296	0.011195	0.011161	0.011161	0.011228	0.011127	0.011228
0.011262	0.011295	0.011195	0.011228	0.011195	0.011530	0.011530
0.011262	0.011296	0.011362	0.011295	0.011934	0.011346	0.011271
0.011341	0.011303	0.011411	0.011303	0.011410	0.011336	0.011302
0.011884	0.011199	0.011334	0.011268	0.011542	0.011400	0.011502
0.011910	0.011362	0.011466	0.011530	0.011396	0.011396	0.011432
0.011094	0.011262	0.011396	0.011362	0.011364	0.011362	0.011330
0.011194	0.011127	0.011161	0.011194	0.011094	0.011161	0.000000
0.011194	0.011228	0.011127	0.011094	0.011194	0.011127	0.011161
0.011161	0.011094	0.011161	0.011195	0.011094	0.011295	0.011329
0.011228	0.011295	0.011228	0.011228	0.011161	0.011564	0.011194
0.011234	0.011161	0.011396	0.011399	0.011262	0.011262	0.011262
0.011262	0.011195	0.011161	0.011195	0.011295	0.011295	0.011228
0.011228	0.011362	0.011530	0.011262	0.011463	0.011463	0.011329
0.011161	0.011228	0.011262	0.011664	0.011228	0.011262	0.011228
0.011262	0.011194	0.011194	0.011497	0.011429	0.011463	0.011194
0.011295	0.011362	0.011228	0.011295	0.011262	0.011127	0.011463
0.011228	0.011194	0.011194	0.011362	0.011194	0.011295	0.011530
0.011228	0.011603	0.011597	0.011385	0.011117	0.011298	0.011163
0.011115	0.011605	0.011131	0.011328	0.011158	0.011264	0.011273
0.011366	0.000000	0.011227	0.000000	0.011114	0.011261	0.011263
0.011286	0.011178	0.011272	0.011201	0.011229	0.011362	0.011429
0.011429	0.011296	0.011398	0.011399	0.011535	0.011555	0.011228
0.011463	0.011329	0.011195	0.011295	0.011094	0.011329	0.011127
0.011194	0.011194	0.011161	0.011127	0.011161	0.011127	0.011228
0.011200	0.011200	0.011197	0.011231	0.011368	0.011333	0.011333
0.011333	0.011262	0.000000	0.000000	0.011196	0.011365	0.011366
0.011161	0.011230	0.011464	0.011432	0.011363	0.011397	0.011329
0.011262	0.011329	0.011329	0.011228	0.011799	0.011396	0.011463
0.011497	0.011558	0.011197	0.011268	0.011161	0.011362	0.011329
0.011363	0.011396	0.011396	0.011396	0.011532	0.011530	0.011164
0.000000	0.000000	0.011162	0.011128	0.011231	0.000000	0.011400
0.011197	0.011323	0.000000	0.011210	0.011195	0.011265	0.011161
0.011127	0.011127	0.011195	0.011161	0.011362	0.011329	0.011262
0.011362	0.011429	0.011161	0.011161	0.011195	0.011195	0.011195
0.011263	0.011195	0.011195	0.011229	0.011362	0.011429	0.011429
0.011296	0.011398	0.011399	0.011535	0.011555	0.011228	0.011396
0.011329	0.011329	0.011162	0.011339	0.011269	0.011299	0.011165
0.011336	0.011516	0.011314	0.011230	0.011127	0.011329	0.011195
0.011295	0.011228	0.011127	0.011229	0.011329	0.011331	0.011228
0.011127	0.000000	0.011195	0.011127	0.000000	0.011162	0.011329
0.011228	0.011161	0.011195	0.011228	0.011195	0.011127	0.011329
0.011194	0.011228	0.011228	0.011161	0.011228	0.011463	0.011127
0.011161	0.011564	0.011161	0.011194	0.011161	0.011530	0.011103
0.011566	0.011103	0.011161	0.011342	0.011426	0.011347	0.000000
0.011113	0.011329	0.011429	0.011535	0.011397	0.011534	0.011665
0.011565	0.011601	0.011564	0.011812	0.011530	0.012404	0.011565
0.012304	0.012072	0.012206	0.012477	0.011530	0.011228	0.011530
0.011969	0.011903	0.011496	0.011396	0.011464	0.011464	0.011432
0.011329	0.011331	0.011396	0.011363	0.011733	0.011799	0.011800
0.011699	0.011800	0.011903	0.011669	0.011969	0.011974	0.012036
0.012037	0.012070	0.012015	0.012037	0.012038	0.011262	0.011564

0.011632	0.012135	0.011636	0.011866	0.012339	0.011400	0.011295
0.011262	0.011262	0.011732	0.011759	0.011869	0.011597	0.011262
S031	0.011765	0.011765	0.011765	0.011530	0.011530	0.011597
0.011502	0.011434	0.011664	0.011362	0.011429	0.011397	0.011362
0.011329	0.011564	0.011295	0.011396	0.011463	0.011429	0.011396
0.011362	0.011396	0.011094	0.011363	0.011396	0.011396	0.011468
0.011362	0.011396	0.011295	0.000000	0.011463	0.011429	0.011463
0.011429	0.011530	0.011429	0.011429	0.011463	0.011429	0.011597
0.011295	0.011429	0.011463	0.011396	0.011429	0.011530	0.011463
0.011429	0.011597	0.011497	0.011497	0.011429	0.011698	0.011429
0.011396	0.011429	0.011564	0.011698	0.011530	0.011429	0.011530
0.011463	0.011530	0.011429	0.011664	0.011631	0.011429	0.011496
0.011396	0.011496	0.011463	0.011530	0.011463	0.011463	0.011463
0.011362	0.011396	0.011362	0.011396	0.011429	0.011464	0.011530
0.011463	0.011429	0.011496	0.011396	0.011463	0.011362	0.011429
0.011597	0.011530	0.011564	0.011597	0.011429	0.011664	0.011429
0.011616	0.011508	0.011664	0.011334	0.011359	0.011428	0.011139
0.011828	0.011213	0.011390	0.011664	0.011664	0.011396	0.011496
0.011496	0.011463	0.011530	0.011429	0.011530	0.011463	0.011463
0.011429	0.011463	0.011731	0.011597	0.011496	0.011463	0.011396
0.011362	0.011295	0.011429	0.011396	0.011262	0.011228	0.011161
0.011262	0.011329	0.011396	0.011396	0.011430	0.011429	0.011432
0.011500	0.011429	0.011429	0.011312	0.011386	0.011341	0.011430
0.011433	0.011369	0.011429	0.011405	0.011332	0.011368	0.011398
0.011384	0.011439	0.011473	0.011363	0.011371	0.011497	0.011396
0.011464	0.011465	0.011464	0.011564	0.011496	0.011866	0.011597
0.011429	0.011497	0.011429	0.011463	0.011329	0.011295	0.011362
0.011429	0.011429	0.011396	0.011362	0.011396	0.011362	0.011463
0.011429	0.011530	0.011463	0.011362	0.011431	0.011564	0.011567
0.011564	0.011396	0.011362	0.011296	0.011429	0.011362	0.011295
0.011298	0.011564	0.011362	0.011564	0.011329	0.011430	0.011396
0.011497	0.011396	0.011429	0.011396	0.011429	0.011295	0.011429
0.011463	0.011396	0.011463	0.011396	0.011429	0.011463	0.011698
0.011530	0.011463	0.011530	0.011566	0.011531	0.011429	0.011396
0.011364	0.011626	0.011472	0.011335	0.011535	0.011299	0.011414
0.011536	0.011434	0.011597	0.011799	0.011504	0.011664	0.011631
0.011564	0.011732	0.011593	0.011472	0.011521	0.011463	0.011534
0.011631	0.011532	0.011669	0.011737	0.011664	0.011732	0.011839
0.011568	0.011739	0.011669	0.011601	0.011867	0.011329	0.011532
0.011496	0.011429	0.011329	0.011329	0.011396	0.011396	0.011463
0.011396	0.011463	0.011362	0.011396	0.011396	0.011396	0.011463
0.011396	0.011564	0.011463	0.011497	0.011295	0.011396	0.011429
0.011429	0.011396	0.011463	0.011429	0.011362	0.011362	0.011362
0.011631	0.011463	0.011530	0.011329	0.011429	0.011597	0.011362
0.011362	0.011429	0.011396	0.011564	0.011362	0.011295	0.011429
0.011362	0.011396	0.011396	0.011329	0.011329	0.011496	0.011429
0.011329	0.011429	0.011295	0.011329	0.011463	0.011329	0.011362
0.011329	0.011463	0.011396	0.011295	0.011362	0.011295	0.011463
0.011539	0.011362	0.011396	0.011497	0.011463	0.011396	0.011496
0.011362	0.011698	0.011295	0.011496	0.011295	0.011295	0.011295
0.011530	0.011463	0.011429	0.011496	0.011329	0.011463	0.011295
0.011362	0.011362	0.011396	0.011496	0.011396	0.011496	0.011564
0.011564	0.011530	0.011429	0.011530	0.011496	0.011362	0.011329
0.011362	0.011362	0.011429	0.011295	0.011329	0.011496	0.011362
0.011329	0.011396	0.011530	0.011496	0.011463	0.011396	0.011396
0.011463	0.011396	0.011496	0.011362	0.011496	0.011597	0.011564
0.011396	0.011530	0.011405	0.017936	0.011322	0.011611	0.011479
0.011441	0.011482	0.011427	0.011234	0.011555	0.011509	0.011607
0.011440	0.011243	0.011294	0.011437	0.011294	0.011455	0.011404
0.011248	0.011284	0.011283	0.011126	0.011530	0.011564	0.011567

0.011438	0.011530	0.011530	0.011429	0.011463	0.011463	0.011430
0.011463	0.011396	0.011429	0.011463	0.011430	0.011362	0.011396
0.011396	0.011463	0.011362	0.011463	0.011497	0.011530	0.011429
0.011396	0.011362	0.011497	0.011497	0.011497	0.011531	0.011564
0.011329	0.011329	0.011362	0.011463	0.011329	0.011564	0.011564
0.011597	0.011597	0.011530	0.011631	0.011530	0.011633	0.011463
0.011463	0.011530	0.011564	0.011396	0.011463	0.011429	0.011463
0.011531	0.011530	0.011564	0.011530	0.011431	0.011631	0.011597
0.011466	0.011362	0.011463	0.011564	0.011463	0.011530	0.011631
0.011799	0.011504	0.011664	0.011631	0.011564	0.011732	0.011363
0.011532	0.011430	0.011396	0.011396	0.011463	0.011362	0.011463
0.011497	0.011530	0.011429	0.011396	0.011362	0.011497	0.011497
0.011497	0.011531	0.011597	0.011530	0.012103	0.011596	0.011517
0.011587	0.011545	0.011657	0.011545	0.011655	0.011577	0.011543
0.012056	0.011441	0.011574	0.011510	0.011783	0.011638	0.011741
0.012080	0.011550	0.011703	0.011765	0.011631	0.011295	0.011668
0.011329	0.011497	0.011631	0.011597	0.011601	0.011329	0.011296
0.011429	0.011362	0.011396	0.011429	0.011329	0.011396	0.011295
0.011362	0.011396	0.011362	0.011329	0.011429	0.011362	0.011396
0.011396	0.011329	0.011396	0.011429	0.011329	0.011530	0.011564
0.011463	0.011463	0.011463	0.011463	0.011396	0.011530	0.011429
0.011130	0.011396	0.011363	0.011365	0.011497	0.011496	0.011496
0.011496	0.011429	0.011396	0.011429	0.011530	0.011530	0.011463
0.011463	0.011597	0.011497	0.011496	0.011698	0.011698	0.011564
0.011396	0.011463	0.011496	0.011631	0.011463	0.011497	0.011463
0.011496	0.011362	0.011362	0.011664	0.011664	0.011631	0.011429
0.011530	0.011597	0.011463	0.011530	0.011496	0.011362	0.011429
0.011463	0.011429	0.011362	0.011597	0.011429	0.011530	0.011564
0.011463	0.011569	0.011564	0.011304	0.011117	0.011468	0.011215
0.011390	0.011527	0.011376	0.011507	0.011353	0.011366	0.011326
0.011213	0.011258	0.011394	0.011192	0.011276	0.011395	0.011547
0.011550	0.011454	0.011448	0.011448	0.011465	0.011329	0.011396
0.011262	0.011464	0.011567	0.011297	0.011704	0.011732	0.011463
0.011429	0.011497	0.011429	0.011463	0.011329	0.011295	0.011362
0.011429	0.011429	0.011396	0.011362	0.011396	0.011362	0.011463
0.011444	0.011445	0.011437	0.011471	0.011608	0.011573	0.011572
0.011571	0.011497	0.011319	0.011312	0.011433	0.011602	0.011603
0.011396	0.011467	0.011700	0.011668	0.011598	0.011632	0.011564
0.011496	0.011564	0.011563	0.011463	0.011765	0.011631	0.011698
0.011732	0.011523	0.011435	0.011511	0.011396	0.011597	0.011564
0.011598	0.011631	0.011631	0.011631	0.011807	0.011765	0.011407
0.011297	0.011303	0.011401	0.011366	0.011402	0.011321	0.011366
0.011437	0.011279	0.011346	0.011472	0.011430	0.011505	0.011396
0.011362	0.011295	0.011429	0.011396	0.011262	0.011228	0.011161
0.011262	0.011329	0.011396	0.011396	0.011430	0.011429	0.011432
0.011500	0.011429	0.011429	0.011465	0.011329	0.011396	0.011262
0.011464	0.011567	0.011297	0.011704	0.011732	0.011463	0.011631
0.011564	0.011564	0.011398	0.011582	0.011477	0.011537	0.011411
0.011542	0.011698	0.011484	0.011468	0.011362	0.011564	0.011429
0.011530	0.011463	0.011362	0.011431	0.011564	0.011567	0.011396
0.011362	0.011296	0.011429	0.011362	0.011295	0.011298	0.011564
0.011463	0.011396	0.011429	0.011463	0.011430	0.011362	0.011295
0.011429	0.011463	0.011463	0.011396	0.011463	0.011698	0.011295
0.011396	0.011530	0.011396	0.011429	0.011396	0.011497	0.011403
0.011510	0.011273	0.011161	0.011302	0.011386	0.011311	0.011227
0.011113	0.011295	0.011396	0.011502	0.011363	0.011500	0.011632
0.011531	0.011567	0.011530	0.011778	0.011497	0.012371	0.011532
0.012271	0.012038	0.012172	0.012444	0.011496	0.011396	0.011497
0.012137	0.011870	0.011463	0.011362	0.011430	0.011430	0.011398
0.011295	0.011298	0.011362	0.011329	0.011699	0.011765	0.011766

0.011598	0.011766	0.011869	0.011635	0.012138	0.012144	0.012205
0.012205	0.012239	0.012186	0.012205	0.012207	0.011496	0.011732
0.011800	0.012303	0.011806	0.011833	0.012305	0.011366	0.011262
0.011228	0.011228	0.011699	0.011718	0.011834	0.011564	0.011229
S032	0.011698	0.011698	0.012034	0.011799	0.011799	0.011866
0.011705	0.011637	0.011866	0.011295	0.011362	0.011329	0.011228
0.011329	0.011496	0.011295	0.011329	0.011396	0.011362	0.011329
0.011295	0.011329	0.011429	0.011295	0.011262	0.011329	0.011671
0.011564	0.011329	0.011228	0.011463	0.000000	0.011362	0.011329
0.011362	0.011396	0.011362	0.011362	0.011396	0.011362	0.011530
0.011295	0.011362	0.011396	0.011329	0.011429	0.011463	0.011396
0.011362	0.011530	0.011430	0.011698	0.011362	0.011564	0.011362
0.011329	0.011429	0.011497	0.011564	0.011732	0.011429	0.011530
0.011329	0.011396	0.011362	0.011530	0.011832	0.011362	0.011429
0.011329	0.011429	0.011396	0.011463	0.011396	0.011396	0.011396
0.011295	0.011329	0.011295	0.011396	0.011362	0.011363	0.011463
0.011396	0.011362	0.011362	0.011329	0.011463	0.011228	0.011362
0.011530	0.011463	0.011698	0.011530	0.011362	0.011530	0.011362
0.011505	0.011733	0.011551	0.011216	0.011247	0.011355	0.011454
0.012085	0.011467	0.011266	0.011597	0.011597	0.011362	0.011497
0.011429	0.011396	0.011463	0.011362	0.011463	0.011295	0.011396
0.011362	0.011463	0.011664	0.011530	0.011429	0.011396	0.011262
0.011295	0.011295	0.011295	0.011329	0.011530	0.011497	0.011429
0.011530	0.011597	0.011329	0.011329	0.011362	0.011362	0.011364
0.011432	0.011362	0.011362	0.011168	0.011277	0.011236	0.011363
0.011365	0.011301	0.011318	0.011336	0.011230	0.011266	0.011297
0.011564	0.011336	0.011301	0.011296	0.011302	0.011430	0.011396
0.011464	0.011397	0.011396	0.011496	0.011429	0.011732	0.011598
0.011631	0.011497	0.011362	0.011463	0.011262	0.011496	0.011295
0.011362	0.011362	0.011329	0.011295	0.011329	0.011295	0.011396
0.011295	0.011396	0.011396	0.011295	0.011397	0.011497	0.011499
0.011430	0.011396	0.011295	0.011229	0.011362	0.011295	0.011228
0.011332	0.011430	0.011228	0.011430	0.011262	0.011362	0.011329
0.011362	0.011329	0.011295	0.011329	0.011362	0.011228	0.011295
0.011329	0.011329	0.011396	0.011329	0.011362	0.011396	0.011631
0.011665	0.011664	0.011732	0.011465	0.011732	0.011362	0.011329
0.011330	0.011545	0.011472	0.011232	0.011739	0.011231	0.011336
0.011739	0.011638	0.011530	0.011732	0.011436	0.011597	0.011497
0.011496	0.011664	0.011486	0.011266	0.011354	0.011396	0.011466
0.011564	0.011465	0.011601	0.011601	0.011597	0.011665	0.011772
0.011500	0.011603	0.011568	0.011500	0.011800	0.011262	0.011465
0.011362	0.011362	0.011262	0.011262	0.011262	0.011329	0.011396
0.011262	0.011329	0.011228	0.011396	0.011262	0.011329	0.011396
0.011329	0.011765	0.011664	0.011698	0.011228	0.011329	0.011362
0.011362	0.011329	0.011396	0.011429	0.011295	0.011295	0.011295
0.011832	0.011463	0.011463	0.011262	0.011362	0.011463	0.011295
0.011295	0.011362	0.011329	0.011429	0.011295	0.011429	0.011496
0.011496	0.011329	0.011329	0.011262	0.011262	0.011429	0.011362
0.011262	0.011362	0.011228	0.011262	0.011396	0.011497	0.011295
0.011262	0.011396	0.011329	0.011228	0.011564	0.011228	0.011396
0.011402	0.011295	0.011597	0.011430	0.011463	0.011329	0.011429
0.011295	0.011564	0.011228	0.011497	0.011295	0.011295	0.011228
0.011396	0.011665	0.011362	0.011429	0.011262	0.011664	0.011295
0.011295	0.011295	0.011329	0.011362	0.011329	0.011429	0.011496
0.011497	0.011463	0.011362	0.011463	0.011362	0.011295	0.011262
0.011295	0.011295	0.011362	0.011295	0.011194	0.011429	0.011295
0.011329	0.011329	0.011463	0.011496	0.011396	0.011262	0.011329
0.011396	0.011329	0.011429	0.011295	0.011429	0.011530	0.011832
0.011329	0.011732	0.011302	0.017823	0.011191	0.011366	0.011653
0.011614	0.011658	0.011374	0.011350	0.011664	0.011682	0.011303

0.011487	0.011487	0.011216	0.011270	0.011255	0.011307	0.011189
0.011482	0.011470	0.011431	0.011325	0.011463	0.011497	0.011466
0.011301	0.011463	0.011463	0.011429	0.011396	0.011396	0.011362
0.011396	0.011329	0.011362	0.011396	0.011362	0.011295	0.011329
0.011329	0.011396	0.011295	0.011396	0.011430	0.011463	0.011362
0.011396	0.011362	0.011698	0.011698	0.011430	0.011464	0.011497
0.011262	0.011262	0.011295	0.011329	0.011262	0.011497	0.011497
0.011530	0.011530	0.011463	0.011564	0.011732	0.011565	0.011329
0.011396	0.011463	0.011429	0.011329	0.011396	0.011362	0.011396
0.011463	0.011463	0.011497	0.011396	0.011296	0.011564	0.011463
0.011399	0.011295	0.011396	0.011497	0.011396	0.011530	0.011564
0.011732	0.011436	0.011597	0.011497	0.011496	0.011664	0.011295
0.011464	0.011363	0.011329	0.011329	0.011396	0.011295	0.011396
0.011430	0.011463	0.011362	0.011396	0.011362	0.011698	0.011698
0.011430	0.011464	0.011530	0.011463	0.012103	0.011453	0.011447
0.011482	0.011407	0.011552	0.011476	0.011550	0.011508	0.011439
0.012022	0.011337	0.011471	0.011406	0.011680	0.011536	0.011604
0.012080	0.011512	0.011635	0.011698	0.011564	0.011564	0.011601
0.011262	0.011362	0.011564	0.011530	0.011533	0.011530	0.011464
0.011362	0.011228	0.011262	0.011362	0.011194	0.011329	0.011228
0.011362	0.011396	0.011295	0.011262	0.011362	0.011295	0.011329
0.011329	0.011262	0.011329	0.011362	0.011262	0.011396	0.011496
0.011396	0.011463	0.011396	0.011396	0.011329	0.011732	0.011362
0.011373	0.011329	0.011564	0.011569	0.011362	0.011429	0.011429
0.011362	0.011295	0.011329	0.011295	0.011463	0.011463	0.011396
0.011396	0.011530	0.011698	0.011429	0.011631	0.011631	0.011496
0.011329	0.011396	0.011362	0.011832	0.011396	0.011362	0.011396
0.011429	0.011362	0.011362	0.011665	0.011530	0.011631	0.011362
0.011463	0.011530	0.011396	0.011463	0.011429	0.011295	0.011631
0.011396	0.011362	0.011362	0.011530	0.011362	0.011463	0.011698
0.011396	0.011773	0.011698	0.011548	0.011173	0.011468	0.011267
0.011170	0.011761	0.011271	0.011507	0.011255	0.011417	0.011379
0.011519	0.011126	0.011339	0.011126	0.011222	0.011328	0.011385
0.011399	0.011257	0.011412	0.011342	0.011397	0.011530	0.011597
0.011597	0.011464	0.011567	0.011569	0.011704	0.011732	0.011396
0.011631	0.011497	0.011362	0.011463	0.011262	0.011496	0.011295
0.011362	0.011362	0.011329	0.011295	0.011329	0.011295	0.011396
0.011340	0.011340	0.011368	0.011368	0.011505	0.011436	0.011504
0.011469	0.011430	0.011208	0.011204	0.011365	0.011535	0.011535
0.011329	0.011399	0.011632	0.011601	0.011497	0.011565	0.011496
0.011429	0.011496	0.011496	0.011396	0.011967	0.011530	0.011631
0.011665	0.011737	0.011367	0.011441	0.011329	0.011530	0.011496
0.011531	0.011564	0.011564	0.011564	0.011729	0.011698	0.011338
0.011229	0.011233	0.011333	0.011298	0.011368	0.011210	0.011570
0.011369	0.011411	0.011156	0.011360	0.011363	0.011402	0.011262
0.011295	0.011295	0.011295	0.011329	0.011530	0.011497	0.011429
0.011530	0.011597	0.011329	0.011329	0.011362	0.011362	0.011364
0.011432	0.011362	0.011362	0.011397	0.011530	0.011597	0.011597
0.011464	0.011567	0.011569	0.011704	0.011732	0.011396	0.011564
0.011496	0.011496	0.011331	0.011513	0.011442	0.011469	0.011305
0.011404	0.011652	0.011483	0.011400	0.011228	0.011430	0.011295
0.011396	0.011396	0.011295	0.011397	0.011497	0.011499	0.011396
0.011295	0.011229	0.011362	0.011295	0.011228	0.011332	0.011430
0.011396	0.011329	0.011362	0.011396	0.011362	0.011295	0.011497
0.011362	0.011396	0.011396	0.011329	0.011396	0.011631	0.011295
0.011329	0.011732	0.011329	0.011295	0.011329	0.011698	0.011274
0.011678	0.011230	0.011261	0.011463	0.011589	0.011491	0.011186
0.011218	0.011496	0.011597	0.011705	0.011531	0.011669	0.011834
0.011733	0.011770	0.011731	0.011949	0.011698	0.012573	0.011734
0.012473	0.012207	0.012375	0.012612	0.011698	0.011396	0.011699

0.012137	0.012038	0.011664	0.011564	0.011632	0.011632	0.011601
0.011496	0.011467	0.011564	0.011531	0.011901	0.011967	0.011968
0.011868	0.011968	0.012038	0.011737	0.012138	0.012144	0.012205
0.012205	0.012239	0.012152	0.012206	0.012173	0.011429	0.011732
0.011800	0.012303	0.011772	0.012034	0.012508	0.011536	0.011464
0.011429	0.011429	0.011900	0.011924	0.012045	0.011765	0.011397
S033	0.011665	0.011665	0.011833	0.011698	0.011698	0.011833
0.011672	0.011604	0.011832	0.011262	0.011329	0.011296	0.011262
0.011295	0.011463	0.011262	0.011295	0.011362	0.011329	0.011295
0.011262	0.011295	0.011396	0.011262	0.011295	0.011295	0.011637
0.011530	0.011295	0.011195	0.011429	0.011362	0.000000	0.011362
0.011329	0.011429	0.011329	0.011329	0.011362	0.011329	0.011497
0.011262	0.011329	0.011362	0.011295	0.011396	0.011429	0.011362
0.011329	0.011497	0.011396	0.011664	0.011329	0.011597	0.011329
0.011295	0.011396	0.011463	0.011597	0.011698	0.011396	0.011497
0.011362	0.011430	0.011329	0.011564	0.011799	0.011329	0.011396
0.011295	0.011396	0.011362	0.011429	0.011362	0.011362	0.011362
0.011262	0.011295	0.011262	0.011362	0.011329	0.011363	0.011429
0.011362	0.011329	0.011396	0.011295	0.011429	0.011262	0.011329
0.011497	0.011429	0.011664	0.011497	0.011329	0.011564	0.011329
0.011505	0.011733	0.011551	0.011255	0.011210	0.011355	0.011415
0.012011	0.011467	0.011184	0.011564	0.011564	0.011362	0.011463
0.011396	0.011362	0.011429	0.011329	0.011430	0.011362	0.011362
0.011329	0.011429	0.011631	0.011497	0.011396	0.011362	0.011295
0.011262	0.011262	0.011329	0.011295	0.011497	0.011463	0.011396
0.011497	0.011564	0.011295	0.011295	0.011329	0.011329	0.011330
0.011398	0.011329	0.011329	0.011204	0.011277	0.011236	0.011329
0.011331	0.011266	0.011318	0.011302	0.011230	0.011266	0.011297
0.011564	0.011336	0.011370	0.011262	0.011268	0.011397	0.011363
0.011430	0.011363	0.011363	0.011463	0.011396	0.011765	0.011564
0.011597	0.011463	0.011329	0.011430	0.011228	0.011463	0.011262
0.011329	0.011329	0.011295	0.011262	0.011295	0.011262	0.011362
0.011329	0.011430	0.011363	0.011262	0.011364	0.011463	0.011466
0.011463	0.011363	0.011262	0.011195	0.011329	0.011262	0.011195
0.011298	0.011463	0.011262	0.011463	0.011228	0.011329	0.011295
0.011396	0.011295	0.011329	0.011295	0.011329	0.011195	0.011329
0.011363	0.011295	0.011362	0.011295	0.011329	0.011362	0.011598
0.011698	0.011631	0.011698	0.011465	0.011699	0.011329	0.011295
0.011330	0.011626	0.011426	0.011232	0.011705	0.011197	0.011296
0.011705	0.011604	0.011497	0.011698	0.011402	0.011564	0.011530
0.011463	0.011631	0.011451	0.011349	0.011354	0.011362	0.011432
0.011530	0.011431	0.011567	0.011635	0.011564	0.011631	0.011738
0.011466	0.011637	0.011568	0.011500	0.011767	0.011228	0.011431
0.011396	0.011329	0.011228	0.011228	0.011295	0.011295	0.011362
0.011295	0.011362	0.011262	0.011362	0.011295	0.011295	0.011362
0.011295	0.011732	0.011631	0.011665	0.011195	0.011295	0.011329
0.011329	0.011295	0.011362	0.011396	0.011262	0.011262	0.011262
0.011799	0.011429	0.011429	0.011228	0.011329	0.011497	0.011262
0.011262	0.011329	0.011295	0.011463	0.011262	0.011396	0.011530
0.011463	0.011295	0.011295	0.011228	0.011228	0.011396	0.011329
0.011228	0.011329	0.011195	0.011228	0.011362	0.011497	0.011262
0.011228	0.011362	0.011295	0.011195	0.011530	0.011195	0.011362
0.011437	0.011262	0.011564	0.011396	0.011429	0.011295	0.011396
0.011262	0.011597	0.011195	0.011463	0.011262	0.011262	0.011195
0.011429	0.011631	0.011329	0.011396	0.011228	0.011631	0.011262
0.011262	0.011262	0.011295	0.011396	0.011295	0.011396	0.011463
0.011463	0.011429	0.011329	0.011430	0.011396	0.011262	0.011228
0.011262	0.011262	0.011329	0.011262	0.011228	0.011396	0.011262
0.011295	0.011295	0.011429	0.011463	0.011362	0.011295	0.011295
0.011362	0.011295	0.011396	0.011262	0.011396	0.011497	0.011732

0.011295	0.011698	0.011302	0.017785	0.011257	0.011427	0.011653
0.011614	0.011658	0.011374	0.011292	0.011829	0.011682	0.011364
0.011487	0.011671	0.011216	0.011311	0.011255	0.011406	0.011318
0.011482	0.011433	0.011431	0.011259	0.011429	0.011463	0.011466
0.011335	0.011430	0.011430	0.011396	0.011362	0.011363	0.011329
0.011362	0.011295	0.011329	0.011362	0.011329	0.011262	0.011295
0.011295	0.011362	0.011262	0.011363	0.011396	0.011430	0.011329
0.011362	0.011329	0.011665	0.011665	0.011396	0.011430	0.011463
0.011228	0.011228	0.011262	0.011363	0.011228	0.011463	0.011463
0.011497	0.011497	0.011429	0.011530	0.011698	0.011532	0.011362
0.011362	0.011430	0.011463	0.011295	0.011362	0.011329	0.011362
0.011430	0.011430	0.011463	0.011430	0.011330	0.011530	0.011497
0.011365	0.011262	0.011362	0.011463	0.011363	0.011497	0.011530
0.011698	0.011402	0.011564	0.011530	0.011463	0.011631	0.011262
0.011431	0.011329	0.011295	0.011295	0.011362	0.011262	0.011363
0.011396	0.011430	0.011329	0.011362	0.011329	0.011665	0.011665
0.011396	0.011430	0.011497	0.011429	0.012069	0.011489	0.011412
0.011482	0.011442	0.011552	0.011441	0.011550	0.011474	0.011439
0.012022	0.011337	0.011471	0.011406	0.011680	0.011536	0.011639
0.012046	0.011512	0.011601	0.011665	0.011530	0.011530	0.011567
0.011228	0.011396	0.011530	0.011497	0.011499	0.011497	0.011464
0.011329	0.011262	0.011295	0.011329	0.011228	0.011295	0.011195
0.011329	0.011362	0.011262	0.011228	0.011329	0.011262	0.011295
0.011295	0.011228	0.011295	0.011329	0.011228	0.011429	0.011463
0.011362	0.011429	0.011362	0.011362	0.011295	0.011698	0.011329
0.011373	0.011295	0.011530	0.011535	0.011396	0.011396	0.011396
0.011396	0.011329	0.011295	0.011329	0.011429	0.011429	0.011362
0.011362	0.011497	0.011664	0.011396	0.011597	0.011597	0.011463
0.011295	0.011362	0.011396	0.011799	0.011363	0.011396	0.011362
0.011396	0.011329	0.011329	0.011631	0.011564	0.011597	0.011329
0.011430	0.011497	0.011362	0.011430	0.011396	0.011262	0.011597
0.011362	0.011329	0.011329	0.011497	0.011329	0.011429	0.011665
0.011295	0.011739	0.011732	0.011507	0.011229	0.011434	0.011267
0.011335	0.011683	0.011271	0.011417	0.011255	0.011366	0.011379
0.011468	0.011192	0.011339	0.011192	0.011222	0.011395	0.011425
0.011437	0.011296	0.011412	0.011236	0.011364	0.011497	0.011564
0.011564	0.011430	0.011534	0.011535	0.011671	0.011696	0.011362
0.011597	0.011463	0.011329	0.011430	0.011228	0.011463	0.011262
0.011329	0.011329	0.011295	0.011262	0.011295	0.011262	0.011362
0.011340	0.011340	0.011334	0.011368	0.011505	0.011470	0.011470
0.011469	0.011396	0.011208	0.011204	0.011331	0.011501	0.011501
0.011296	0.011365	0.011599	0.011567	0.011497	0.011531	0.011463
0.011396	0.011463	0.011463	0.011362	0.011934	0.011530	0.011598
0.011631	0.011701	0.011333	0.011407	0.011295	0.011497	0.011463
0.011498	0.011530	0.011530	0.011530	0.011689	0.011665	0.011303
0.011195	0.011199	0.011298	0.011264	0.011368	0.011209	0.011536
0.011335	0.011455	0.011203	0.011360	0.011329	0.011402	0.011295
0.011262	0.011262	0.011329	0.011295	0.011497	0.011463	0.011396
0.011497	0.011564	0.011295	0.011295	0.011329	0.011329	0.011330
0.011398	0.011329	0.011329	0.011364	0.011497	0.011564	0.011564
0.011430	0.011534	0.011535	0.011671	0.011696	0.011362	0.011530
0.011463	0.011463	0.011297	0.011478	0.011408	0.011435	0.011270
0.011473	0.011698	0.011399	0.011366	0.011262	0.011463	0.011329
0.011430	0.011363	0.011262	0.011364	0.011463	0.011466	0.011363
0.011262	0.011195	0.011329	0.011262	0.011195	0.011298	0.011463
0.011362	0.011295	0.011329	0.011362	0.011329	0.011262	0.011463
0.011329	0.011362	0.011362	0.011295	0.011362	0.011597	0.011262
0.011295	0.011698	0.011295	0.011329	0.011295	0.011664	0.011189
0.011678	0.011188	0.011261	0.011422	0.011508	0.011491	0.011144
0.011218	0.011463	0.011564	0.011671	0.011531	0.011669	0.011800

0.011699	0.011737	0.011698	0.011949	0.011664	0.012539	0.011700
0.012439	0.012207	0.012341	0.012612	0.011664	0.011362	0.011665
0.012103	0.012039	0.011631	0.011530	0.011598	0.011598	0.011568
0.011463	0.011467	0.011530	0.011497	0.011867	0.011933	0.011935
0.011834	0.011934	0.012038	0.011804	0.012104	0.012110	0.012171
0.012172	0.012205	0.012152	0.012172	0.012173	0.011396	0.011699
0.011766	0.012270	0.011772	0.012001	0.012474	0.011536	0.011430
0.011396	0.011396	0.011867	0.011924	0.012010	0.011732	0.011397
S034	0.011698	0.011698	0.012034	0.011799	0.011799	0.011866
0.011705	0.011637	0.011866	0.011295	0.011362	0.011329	0.011295
0.011329	0.011497	0.011295	0.011329	0.011396	0.011362	0.011329
0.011295	0.011329	0.011429	0.011295	0.011262	0.011329	0.011671
0.011564	0.011329	0.011228	0.011463	0.011329	0.011362	0.000000
0.011362	0.011396	0.011362	0.011362	0.011396	0.011362	0.011530
0.011295	0.011362	0.011396	0.011329	0.011429	0.011463	0.011396
0.011362	0.011530	0.011430	0.011698	0.011362	0.011564	0.011362
0.011329	0.011430	0.011497	0.011564	0.011732	0.011429	0.011530
0.011329	0.011396	0.011362	0.011530	0.011833	0.011362	0.011429
0.011329	0.011429	0.011396	0.011463	0.011396	0.011396	0.011396
0.011295	0.011329	0.011295	0.011396	0.011362	0.011397	0.011463
0.011396	0.011362	0.011362	0.011329	0.011463	0.011228	0.011362
0.011530	0.011463	0.011698	0.011530	0.011362	0.011530	0.011362
0.011468	0.011695	0.011437	0.011177	0.011135	0.011318	0.011336
0.012011	0.011365	0.011143	0.011597	0.011597	0.011396	0.011497
0.011429	0.011396	0.011463	0.011362	0.011463	0.011329	0.011396
0.011362	0.011463	0.011664	0.011530	0.011429	0.011396	0.011262
0.011295	0.011295	0.011295	0.011329	0.011530	0.011497	0.011430
0.011530	0.011597	0.011329	0.011329	0.011362	0.011362	0.011364
0.011432	0.011362	0.011362	0.011096	0.011241	0.011271	0.011363
0.011365	0.011301	0.011208	0.011267	0.011264	0.011300	0.011330
0.011456	0.011370	0.011301	0.011296	0.011233	0.011430	0.011397
0.011464	0.011397	0.011396	0.011496	0.011429	0.011732	0.011598
0.011631	0.011497	0.011362	0.011463	0.011262	0.011497	0.011295
0.011362	0.011362	0.011329	0.011295	0.011329	0.011295	0.011396
0.011295	0.011396	0.011396	0.011295	0.011397	0.011497	0.011500
0.011430	0.011396	0.011295	0.011229	0.011362	0.011295	0.011228
0.011332	0.011430	0.011228	0.011430	0.011262	0.011363	0.011329
0.011362	0.011329	0.011295	0.011329	0.011362	0.011228	0.011295
0.011329	0.011329	0.011396	0.011329	0.011362	0.011396	0.011631
0.011732	0.011664	0.011732	0.011498	0.011732	0.011362	0.011329
0.011364	0.011626	0.011426	0.011266	0.011739	0.011231	0.011336
0.011739	0.011570	0.011530	0.011732	0.011436	0.011597	0.011497
0.011497	0.011664	0.011522	0.011390	0.011437	0.011396	0.011466
0.011564	0.011465	0.011601	0.011602	0.011597	0.011665	0.011772
0.011500	0.011603	0.011602	0.011534	0.011800	0.011262	0.011465
0.011362	0.011362	0.011262	0.011262	0.011262	0.011329	0.011396
0.011262	0.011329	0.011228	0.011396	0.011262	0.011329	0.011396
0.011329	0.011765	0.011664	0.011698	0.011228	0.011329	0.011362
0.011362	0.011329	0.011396	0.011429	0.011295	0.011295	0.011295
0.011832	0.011463	0.011463	0.011262	0.011362	0.011463	0.011295
0.011295	0.011362	0.011329	0.011429	0.011295	0.011429	0.011496
0.011497	0.011329	0.011329	0.011262	0.011262	0.011430	0.011362
0.011262	0.011362	0.011228	0.011262	0.011396	0.011530	0.011295
0.011262	0.011396	0.011329	0.011228	0.011564	0.011228	0.011396
0.011403	0.011295	0.011597	0.011430	0.011463	0.011329	0.011429
0.011295	0.011564	0.011228	0.011497	0.011295	0.011295	0.011228
0.011396	0.011597	0.011362	0.011429	0.011262	0.011664	0.011295
0.011295	0.011295	0.011329	0.011362	0.011329	0.011429	0.011497
0.011497	0.011463	0.011362	0.011463	0.011362	0.011295	0.011262
0.011295	0.011295	0.011362	0.011295	0.011194	0.011429	0.011295

0.011329	0.011329	0.011463	0.011497	0.011396	0.011262	0.011329
0.011396	0.011329	0.011429	0.011295	0.011429	0.011530	0.011832
0.011329	0.011732	0.011336	0.017748	0.011126	0.011488	0.011688
0.011648	0.011623	0.011270	0.011176	0.011609	0.011716	0.011303
0.011392	0.011487	0.011138	0.011228	0.011177	0.011307	0.011275
0.011389	0.011358	0.011357	0.011126	0.011463	0.011497	0.011500
0.011301	0.011463	0.011463	0.011430	0.011396	0.011396	0.011363
0.011396	0.011329	0.011362	0.011396	0.011363	0.011295	0.011329
0.011329	0.011396	0.011295	0.011396	0.011430	0.011463	0.011362
0.011396	0.011362	0.011698	0.011698	0.011430	0.011464	0.011497
0.011262	0.011262	0.011295	0.011329	0.011262	0.011497	0.011497
0.011530	0.011530	0.011463	0.011564	0.011732	0.011565	0.011329
0.011396	0.011463	0.011430	0.011329	0.011396	0.011362	0.011396
0.011463	0.011463	0.011497	0.011396	0.011296	0.011564	0.011463
0.011399	0.011295	0.011396	0.011497	0.011396	0.011530	0.011564
0.011732	0.011436	0.011597	0.011497	0.011497	0.011664	0.011295
0.011464	0.011363	0.011329	0.011329	0.011396	0.011295	0.011396
0.011430	0.011463	0.011362	0.011396	0.011362	0.011698	0.011698
0.011430	0.011464	0.011530	0.011463	0.012103	0.011525	0.011447
0.011517	0.011476	0.011587	0.011476	0.011585	0.011508	0.011474
0.012056	0.011372	0.011505	0.011441	0.011714	0.011570	0.011605
0.012080	0.011550	0.011635	0.011698	0.011564	0.011564	0.011601
0.011262	0.011362	0.011564	0.011530	0.011533	0.011530	0.011498
0.011362	0.011228	0.011262	0.011362	0.011194	0.011329	0.011228
0.011362	0.011396	0.011295	0.011262	0.011362	0.011295	0.011329
0.011329	0.011262	0.011329	0.011362	0.011262	0.011396	0.011497
0.011396	0.011463	0.011396	0.011396	0.011329	0.011732	0.011362
0.011408	0.011329	0.011564	0.011569	0.011362	0.011429	0.011429
0.011429	0.011295	0.011329	0.011295	0.011463	0.011463	0.011396
0.011396	0.011530	0.011698	0.011429	0.011631	0.011631	0.011496
0.011329	0.011396	0.011429	0.011833	0.011396	0.011362	0.011396
0.011429	0.011362	0.011362	0.011665	0.011530	0.011631	0.011362
0.011463	0.011530	0.011396	0.011463	0.011429	0.011295	0.011631
0.011396	0.011362	0.011362	0.011530	0.011362	0.011463	0.011698
0.011396	0.011773	0.011765	0.011426	0.011117	0.011468	0.011163
0.011280	0.011644	0.011201	0.011373	0.011158	0.011315	0.011273
0.011366	0.000000	0.011227	0.000000	0.011114	0.011261	0.011263
0.011362	0.011139	0.011448	0.011377	0.011397	0.011530	0.011598
0.011597	0.011464	0.011567	0.011569	0.011704	0.011696	0.011396
0.011631	0.011497	0.011362	0.011463	0.011262	0.011497	0.011295
0.011362	0.011362	0.011329	0.011295	0.011329	0.011295	0.011396
0.011305	0.011340	0.011368	0.011403	0.011539	0.011436	0.011504
0.011503	0.011430	0.011171	0.011132	0.011365	0.011535	0.011535
0.011329	0.011399	0.011633	0.011601	0.011531	0.011565	0.011496
0.011429	0.011497	0.011496	0.011396	0.011967	0.011564	0.011631
0.011665	0.011737	0.011367	0.011441	0.011329	0.011530	0.011497
0.011531	0.011564	0.011564	0.011564	0.011689	0.011698	0.011338
0.011229	0.011233	0.011333	0.011298	0.011402	0.011247	0.011570
0.011369	0.011499	0.011251	0.011397	0.011363	0.011437	0.011262
0.011295	0.011295	0.011295	0.011329	0.011530	0.011497	0.011430
0.011530	0.011597	0.011329	0.011329	0.011362	0.011362	0.011364
0.011432	0.011362	0.011362	0.011397	0.011530	0.011598	0.011597
0.011464	0.011567	0.011569	0.011704	0.011696	0.011396	0.011564
0.011497	0.011497	0.011331	0.011513	0.011442	0.011469	0.011305
0.011232	0.011653	0.011483	0.011400	0.011228	0.011430	0.011295
0.011396	0.011396	0.011295	0.011397	0.011497	0.011500	0.011396
0.011295	0.011229	0.011362	0.011295	0.011228	0.011332	0.011430
0.011396	0.011329	0.011362	0.011396	0.011363	0.011295	0.011497
0.011362	0.011396	0.011396	0.011329	0.011396	0.011631	0.011295
0.011329	0.011732	0.011329	0.011295	0.011329	0.011698	0.011146

0.011566	0.011145	0.011161	0.011382	0.011467	0.011419	0.011102
0.011113	0.011496	0.011597	0.011705	0.011565	0.011703	0.011834
0.011733	0.011770	0.011732	0.011983	0.011698	0.012573	0.011734
0.012473	0.012241	0.012375	0.012646	0.011698	0.011396	0.011699
0.012137	0.012072	0.011664	0.011564	0.011632	0.011632	0.011567
0.011496	0.011501	0.011564	0.011531	0.011901	0.011967	0.011968
0.011868	0.011968	0.012072	0.011838	0.012138	0.012144	0.012205
0.012206	0.012239	0.012186	0.012206	0.012207	0.011429	0.011732
0.011800	0.012303	0.011806	0.012034	0.012508	0.011570	0.011464
0.011429	0.011429	0.011900	0.011965	0.012045	0.011765	0.011431
S035	0.011664	0.011664	0.012001	0.011765	0.011765	0.011832
0.011671	0.011603	0.011832	0.011262	0.011329	0.011296	0.011262
0.011295	0.011463	0.011262	0.011295	0.011362	0.011329	0.011295
0.011262	0.011295	0.011396	0.011262	0.011295	0.011295	0.011637
0.011530	0.011295	0.011194	0.011429	0.011362	0.011329	0.011362
0.000000	0.011429	0.011329	0.011329	0.011362	0.011329	0.011497
0.011262	0.011329	0.011362	0.011295	0.011396	0.011429	0.011161
0.011329	0.011497	0.011396	0.011664	0.011329	0.011597	0.011329
0.011295	0.011396	0.011463	0.011597	0.011698	0.011396	0.011496
0.011362	0.011429	0.011329	0.011564	0.011799	0.011329	0.011329
0.011295	0.011396	0.011362	0.011429	0.011362	0.011362	0.011362
0.011262	0.011295	0.011262	0.011362	0.011329	0.011363	0.011429
0.011362	0.011329	0.011396	0.011295	0.011429	0.011262	0.011329
0.011496	0.011429	0.011664	0.011497	0.011329	0.011564	0.011329
0.011468	0.011695	0.011513	0.011216	0.011247	0.011355	0.011415
0.012085	0.011518	0.011225	0.011564	0.011564	0.011362	0.011463
0.011396	0.011362	0.011429	0.011329	0.011429	0.011362	0.011362
0.011329	0.011429	0.011631	0.011496	0.011396	0.011362	0.011295
0.011262	0.011262	0.011329	0.011295	0.011497	0.011463	0.011396
0.011497	0.011564	0.011295	0.011295	0.011329	0.011329	0.011330
0.011398	0.011329	0.011329	0.011168	0.011241	0.011201	0.011329
0.011331	0.011266	0.011318	0.011302	0.011196	0.011232	0.011297
0.011528	0.011302	0.011370	0.011262	0.011268	0.011397	0.011363
0.011430	0.011364	0.011363	0.011463	0.011396	0.011765	0.011564
0.011597	0.011463	0.011329	0.011430	0.011228	0.011463	0.011262
0.011329	0.011329	0.011295	0.011262	0.011295	0.011262	0.011362
0.011329	0.011430	0.011362	0.011262	0.011363	0.011463	0.011466
0.011463	0.011362	0.011262	0.011195	0.011329	0.011262	0.011195
0.011298	0.011463	0.011262	0.011463	0.011228	0.011329	0.011295
0.011396	0.011295	0.011329	0.011295	0.011329	0.011195	0.011329
0.011362	0.011295	0.011362	0.011295	0.011329	0.011362	0.011597
0.011698	0.011631	0.011698	0.011465	0.011698	0.011329	0.011295
0.011330	0.011626	0.011426	0.011232	0.011705	0.011197	0.011336
0.011705	0.011604	0.011496	0.011698	0.011402	0.011564	0.011530
0.011463	0.011631	0.011451	0.011307	0.011354	0.011362	0.011432
0.011530	0.011431	0.011567	0.011635	0.011564	0.011631	0.011738
0.011466	0.011637	0.011534	0.011466	0.011767	0.011228	0.011431
0.011396	0.011329	0.011228	0.011228	0.011295	0.011295	0.011362
0.011295	0.011362	0.011262	0.011362	0.011295	0.011295	0.011362
0.011295	0.011731	0.011631	0.011664	0.011194	0.011295	0.011329
0.011329	0.011295	0.011362	0.011396	0.011262	0.011262	0.011262
0.011799	0.011429	0.011429	0.011228	0.011329	0.011497	0.011262
0.011262	0.011329	0.011295	0.011463	0.011262	0.011396	0.011530
0.011463	0.011295	0.011295	0.011228	0.011228	0.011396	0.011329
0.011228	0.011329	0.011195	0.011228	0.011362	0.011463	0.011262
0.011228	0.011362	0.011295	0.011195	0.011530	0.011195	0.011362
0.011437	0.011262	0.011564	0.011396	0.011429	0.011295	0.011396
0.011262	0.011597	0.011194	0.011463	0.011262	0.011262	0.011194
0.011429	0.011631	0.011329	0.011396	0.011228	0.011631	0.011262
0.011262	0.011262	0.011295	0.011396	0.011295	0.011396	0.011463

0.011463	0.011429	0.011329	0.011429	0.011396	0.011262	0.011228
0.011262	0.011262	0.011329	0.011262	0.011228	0.011396	0.011262
0.011295	0.011295	0.011429	0.011463	0.011362	0.011295	0.011295
0.011362	0.011295	0.011396	0.011262	0.011396	0.011496	0.011799
0.011295	0.011698	0.011302	0.017823	0.011322	0.011366	0.011653
0.011614	0.011658	0.011374	0.011350	0.011774	0.011682	0.011303
0.011534	0.011487	0.011177	0.011270	0.011294	0.011356	0.011232
0.011529	0.011470	0.011468	0.011391	0.011429	0.011463	0.011466
0.011301	0.011429	0.011429	0.011396	0.011362	0.011362	0.011329
0.011362	0.011295	0.011329	0.011362	0.011329	0.011262	0.011295
0.011295	0.011362	0.011262	0.011362	0.011396	0.011429	0.011329
0.011362	0.011329	0.011665	0.011665	0.011396	0.011430	0.011463
0.011228	0.011228	0.011262	0.011362	0.011228	0.011463	0.011463
0.011497	0.011496	0.011429	0.011530	0.011698	0.011532	0.011362
0.011362	0.011430	0.011463	0.011295	0.011362	0.011329	0.011362
0.011430	0.011430	0.011463	0.011430	0.011330	0.011530	0.011497
0.011365	0.011262	0.011362	0.011463	0.011362	0.011497	0.011530
0.011698	0.011402	0.011564	0.011530	0.011463	0.011631	0.011262
0.011431	0.011329	0.011295	0.011295	0.011362	0.011262	0.011362
0.011396	0.011429	0.011329	0.011362	0.011329	0.011665	0.011665
0.011396	0.011430	0.011496	0.011429	0.012069	0.011453	0.011412
0.011447	0.011407	0.011517	0.011441	0.011515	0.011439	0.011405
0.012022	0.011302	0.011437	0.011371	0.011646	0.011502	0.011639
0.012046	0.011475	0.011601	0.011664	0.011530	0.011530	0.011567
0.011228	0.011396	0.011530	0.011496	0.011499	0.011496	0.011464
0.011329	0.011262	0.011295	0.011329	0.011228	0.011295	0.011194
0.011329	0.011362	0.011262	0.011228	0.011329	0.011262	0.011295
0.011295	0.011228	0.011295	0.011329	0.011228	0.011429	0.011463
0.011362	0.011429	0.011362	0.011295	0.011295	0.011698	0.011329
0.011338	0.011295	0.011530	0.011535	0.011396	0.011396	0.011396
0.011396	0.011329	0.011295	0.011329	0.011429	0.011429	0.011362
0.011362	0.011497	0.011664	0.011396	0.011597	0.011597	0.011463
0.011295	0.011362	0.011396	0.011799	0.011362	0.011396	0.011362
0.011396	0.011329	0.011329	0.011631	0.011564	0.011597	0.011329
0.011429	0.011497	0.011362	0.011429	0.011396	0.011262	0.011597
0.011362	0.011329	0.011329	0.011497	0.011329	0.011429	0.011664
0.011362	0.011739	0.011732	0.011507	0.011229	0.011434	0.011318
0.011170	0.011722	0.011271	0.011462	0.011304	0.011417	0.011432
0.011519	0.011258	0.011394	0.011258	0.011276	0.011462	0.011385
0.011399	0.011296	0.011412	0.011342	0.011364	0.011497	0.011564
0.011564	0.011430	0.011533	0.011535	0.011670	0.011696	0.011362
0.011597	0.011463	0.011329	0.011430	0.011228	0.011463	0.011262
0.011329	0.011329	0.011295	0.011262	0.011295	0.011262	0.011362
0.011305	0.011305	0.011334	0.011334	0.011471	0.011470	0.011470
0.011469	0.011396	0.011171	0.011168	0.011331	0.011501	0.011501
0.011296	0.011365	0.011599	0.011567	0.011497	0.011531	0.011463
0.011396	0.011463	0.011463	0.011362	0.011933	0.011530	0.011597
0.011631	0.011701	0.011333	0.011407	0.011295	0.011497	0.011463
0.011497	0.011530	0.011530	0.011530	0.011689	0.011664	0.011303
0.011195	0.011199	0.011298	0.011264	0.011333	0.011172	0.011536
0.011335	0.011455	0.011156	0.011322	0.011329	0.011368	0.011295
0.011262	0.011262	0.011329	0.011295	0.011497	0.011463	0.011396
0.011497	0.011564	0.011295	0.011295	0.011329	0.011329	0.011330
0.011398	0.011329	0.011329	0.011364	0.011497	0.011564	0.011564
0.011430	0.011533	0.011535	0.011670	0.011696	0.011362	0.011530
0.011463	0.011463	0.011297	0.011478	0.011408	0.011435	0.011305
0.011473	0.011698	0.011441	0.011366	0.011262	0.011463	0.011329
0.011430	0.011362	0.011262	0.011363	0.011463	0.011466	0.011362
0.011262	0.011195	0.011329	0.011262	0.011195	0.011298	0.011463
0.011362	0.011295	0.011329	0.011362	0.011329	0.011262	0.011463

0.011329	0.011362	0.011362	0.011295	0.011362	0.011597	0.011262
0.011295	0.011698	0.011295	0.011329	0.011295	0.011664	0.011232
0.011679	0.011188	0.011311	0.011463	0.011549	0.011491	0.011186
0.011270	0.011463	0.011564	0.011671	0.011531	0.011669	0.011800
0.011699	0.011737	0.011698	0.011949	0.011664	0.012539	0.011700
0.012439	0.012207	0.012341	0.012545	0.011664	0.011362	0.011665
0.012103	0.012038	0.011631	0.011530	0.011598	0.011598	0.011567
0.011463	0.011467	0.011530	0.011497	0.011867	0.011933	0.011935
0.011834	0.011934	0.012038	0.011804	0.012104	0.012076	0.012171
0.012172	0.012205	0.012152	0.012172	0.012173	0.011396	0.011699
0.011767	0.012270	0.011772	0.012001	0.012474	0.011536	0.011430
0.011396	0.011396	0.011867	0.011883	0.012010	0.011732	0.011397
S036	0.011765	0.011765	0.012101	0.011866	0.011866	0.011933
0.011773	0.011705	0.011933	0.011362	0.011429	0.011397	0.011362
0.011396	0.011564	0.011362	0.011396	0.011463	0.011429	0.011396
0.011362	0.011396	0.011496	0.011363	0.011329	0.011396	0.011739
0.011631	0.011396	0.011295	0.011530	0.011396	0.011429	0.011396
0.011429	0.000000	0.011429	0.011429	0.011463	0.011429	0.011597
0.011362	0.011429	0.011463	0.011396	0.011496	0.011530	0.011463
0.011429	0.011597	0.011497	0.011765	0.011429	0.011631	0.011429
0.011396	0.011497	0.011564	0.011631	0.011799	0.011497	0.011597
0.011396	0.011329	0.011429	0.011597	0.011900	0.011429	0.011497
0.011396	0.011496	0.011463	0.011530	0.011463	0.011463	0.011463
0.011362	0.011396	0.011362	0.011463	0.011429	0.011464	0.011530
0.011463	0.011429	0.011429	0.011396	0.011530	0.011295	0.011429
0.011597	0.011530	0.011765	0.011530	0.011429	0.011597	0.011429
0.011616	0.011845	0.011664	0.011334	0.011321	0.011465	0.011572
0.012195	0.011569	0.011390	0.011664	0.011664	0.011463	0.011564
0.011497	0.011463	0.011530	0.011429	0.011530	0.011396	0.011463
0.011429	0.011530	0.011732	0.011597	0.011497	0.011463	0.011329
0.011362	0.011362	0.011362	0.011396	0.011597	0.011564	0.011497
0.011597	0.011665	0.011396	0.011396	0.011430	0.011429	0.011432
0.011500	0.011430	0.011430	0.011276	0.011386	0.011341	0.011430
0.011433	0.011370	0.011429	0.011405	0.011332	0.011369	0.011398
0.011636	0.011439	0.011405	0.011363	0.011371	0.011498	0.011464
0.011531	0.011465	0.011464	0.011564	0.011496	0.011799	0.011665
0.011698	0.011564	0.011429	0.011530	0.011329	0.011564	0.011362
0.011429	0.011429	0.011396	0.011362	0.011396	0.011362	0.011463
0.011362	0.011463	0.011463	0.011362	0.011465	0.011564	0.011533
0.011497	0.011463	0.011362	0.011296	0.011429	0.011362	0.011295
0.011400	0.011497	0.011295	0.011497	0.011329	0.011430	0.011396
0.011430	0.011396	0.011362	0.011396	0.011430	0.011295	0.011362
0.011396	0.011396	0.011463	0.011396	0.011429	0.011463	0.011698
0.011799	0.011732	0.011799	0.011566	0.011799	0.011429	0.011396
0.011432	0.011667	0.011472	0.011335	0.011807	0.011299	0.011414
0.011807	0.011706	0.011597	0.011799	0.011504	0.011664	0.011564
0.011564	0.011732	0.011593	0.011390	0.011479	0.011463	0.011534
0.011631	0.011532	0.011669	0.011669	0.011664	0.011732	0.011839
0.011568	0.011671	0.011670	0.011601	0.011868	0.011329	0.011532
0.011429	0.011429	0.011329	0.011329	0.011329	0.011396	0.011463
0.011329	0.011396	0.011295	0.011463	0.011329	0.011396	0.011463
0.011396	0.011832	0.011732	0.011765	0.011295	0.011396	0.011429
0.011429	0.011396	0.011463	0.011497	0.011362	0.011362	0.011362
0.011832	0.011530	0.011530	0.011329	0.011429	0.011396	0.011362
0.011362	0.011429	0.011396	0.011497	0.011362	0.011496	0.011564
0.011564	0.011329	0.011396	0.011329	0.011329	0.011497	0.011429
0.011329	0.011429	0.011295	0.011329	0.011463	0.011598	0.011362
0.011329	0.011463	0.011396	0.011295	0.011631	0.011295	0.011463
0.011471	0.011362	0.011664	0.011497	0.011530	0.011396	0.011496
0.011362	0.011631	0.011295	0.011564	0.011362	0.011362	0.011295

0.011463	0.011732	0.011429	0.011497	0.011329	0.011732	0.011362
0.011362	0.011362	0.011329	0.011295	0.011396	0.011496	0.011564
0.011496	0.011530	0.011429	0.011530	0.011429	0.011362	0.011329
0.011362	0.011362	0.011429	0.011362	0.011262	0.011497	0.011362
0.011396	0.011396	0.011530	0.011564	0.011463	0.011329	0.011396
0.011463	0.011396	0.011496	0.011362	0.011496	0.011597	0.011900
0.011396	0.011799	0.011405	0.017936	0.011387	0.011427	0.011758
0.011718	0.011764	0.011479	0.011408	0.011774	0.011785	0.011364
0.011582	0.011610	0.011333	0.011395	0.011372	0.011406	0.011361
0.011576	0.011582	0.011542	0.011523	0.011530	0.011564	0.011568
0.011369	0.011530	0.011530	0.011497	0.011463	0.011463	0.011430
0.011463	0.011396	0.011429	0.011463	0.011430	0.011362	0.011396
0.011396	0.011463	0.011362	0.011463	0.011497	0.011530	0.011429
0.011463	0.011430	0.011765	0.011765	0.011497	0.011531	0.011564
0.011329	0.011329	0.011362	0.011396	0.011329	0.011564	0.011564
0.011597	0.011597	0.011530	0.011631	0.011799	0.011633	0.011396
0.011463	0.011530	0.011497	0.011396	0.011463	0.011430	0.011463
0.011531	0.011530	0.011564	0.011463	0.011363	0.011631	0.011530
0.011467	0.011362	0.011463	0.011564	0.011463	0.011598	0.011631
0.011799	0.011504	0.011664	0.011564	0.011564	0.011732	0.011363
0.011532	0.011430	0.011396	0.011396	0.011463	0.011362	0.011463
0.011497	0.011530	0.011429	0.011463	0.011430	0.011765	0.011765
0.011497	0.011531	0.011597	0.011530	0.012170	0.011596	0.011517
0.011587	0.011546	0.011658	0.011545	0.011655	0.011577	0.011543
0.012125	0.011441	0.011574	0.011510	0.011783	0.011638	0.011673
0.012148	0.011626	0.011703	0.011765	0.011631	0.011631	0.011668
0.011329	0.011429	0.011631	0.011597	0.011601	0.011597	0.011565
0.011429	0.011295	0.011329	0.011429	0.011262	0.011396	0.011295
0.011429	0.011463	0.011362	0.011329	0.011429	0.011362	0.011396
0.011396	0.011329	0.011396	0.011429	0.011329	0.000000	0.011564
0.011463	0.011530	0.011463	0.011463	0.011396	0.011799	0.011429
0.011478	0.011396	0.011631	0.011637	0.011429	0.011496	0.011496
0.011497	0.011362	0.011396	0.011362	0.011530	0.011530	0.011463
0.011463	0.011597	0.011765	0.011496	0.011698	0.011698	0.011530
0.011396	0.011463	0.011497	0.011900	0.011463	0.011429	0.011463
0.011496	0.011429	0.011429	0.011732	0.011597	0.011698	0.011429
0.011530	0.011597	0.011463	0.011530	0.011496	0.011362	0.011698
0.011463	0.011429	0.011429	0.011597	0.011429	0.011530	0.011765
0.011463	0.011841	0.011832	0.011629	0.011399	0.011536	0.011370
0.011335	0.011878	0.011376	0.011596	0.011402	0.011569	0.011485
0.011570	0.011324	0.011450	0.011324	0.011330	0.011462	0.011506
0.011512	0.011375	0.011518	0.011448	0.011465	0.011597	0.011665
0.011665	0.011531	0.011635	0.011636	0.011772	0.011767	0.011463
0.011698	0.011564	0.011429	0.011530	0.011329	0.011564	0.011362
0.011429	0.011429	0.011396	0.011362	0.011396	0.011362	0.011463
0.011444	0.011445	0.011437	0.011471	0.011608	0.011504	0.011572
0.011571	0.011497	0.011282	0.011276	0.011433	0.011602	0.011603
0.011396	0.011467	0.011700	0.011669	0.011598	0.011632	0.011564
0.011496	0.011564	0.011564	0.011463	0.012034	0.011597	0.011698
0.011732	0.011808	0.011435	0.011476	0.011396	0.011597	0.011564
0.011598	0.011631	0.011631	0.011631	0.011807	0.011765	0.011407
0.011297	0.011303	0.011401	0.011366	0.011470	0.011321	0.011638
0.011437	0.011543	0.011298	0.011472	0.011430	0.011505	0.011329
0.011362	0.011362	0.011362	0.011396	0.011597	0.011564	0.011497
0.011597	0.011665	0.011396	0.011396	0.011430	0.011429	0.011432
0.011500	0.011430	0.011430	0.011465	0.011597	0.011665	0.011665
0.011531	0.011635	0.011636	0.011772	0.011767	0.011463	0.011631
0.011564	0.011564	0.011398	0.011582	0.011512	0.011537	0.011411
0.011508	0.011698	0.011526	0.011468	0.011295	0.011497	0.011362
0.011463	0.011463	0.011362	0.011465	0.011564	0.011533	0.011463

0.011362	0.011296	0.011429	0.011362	0.011295	0.011400	0.011497
0.011463	0.011396	0.011429	0.011463	0.011430	0.011362	0.011564
0.011429	0.011463	0.011463	0.011396	0.011463	0.011698	0.011362
0.011396	0.011799	0.011396	0.011362	0.011396	0.011765	0.011360
0.011791	0.011358	0.011361	0.011624	0.011671	0.011599	0.011311
0.011323	0.011564	0.011664	0.011773	0.011632	0.011771	0.011901
0.011733	0.011838	0.011799	0.012051	0.011765	0.012640	0.011801
0.012540	0.012308	0.012442	0.012714	0.011765	0.011463	0.011766
0.012205	0.012140	0.011732	0.011631	0.011699	0.011699	0.011669
0.011564	0.011569	0.011631	0.011598	0.011968	0.012034	0.012036
0.011935	0.012035	0.012139	0.011906	0.012205	0.012212	0.012272
0.012273	0.012307	0.012255	0.012273	0.012274	0.011496	0.011800
0.011868	0.012371	0.011874	0.012102	0.012575	0.011638	0.011531
0.011496	0.011496	0.011968	0.012048	0.012116	0.011832	0.011498
S037	0.011664	0.011664	0.012001	0.011765	0.011765	0.011832
0.011671	0.011603	0.011832	0.011262	0.011329	0.011296	0.011262
0.011295	0.011463	0.011262	0.011295	0.011362	0.011329	0.011295
0.011262	0.011295	0.011396	0.011262	0.011295	0.011295	0.011637
0.011530	0.011295	0.011194	0.011429	0.011362	0.011329	0.011362
0.011329	0.011429	0.000000	0.011329	0.011295	0.011329	0.011496
0.011262	0.011329	0.011295	0.011228	0.011396	0.011429	0.011362
0.011329	0.011429	0.011396	0.011664	0.011262	0.011597	0.011262
0.011295	0.011396	0.011396	0.011597	0.011698	0.011396	0.011496
0.011362	0.011429	0.011329	0.011564	0.011799	0.011329	0.011396
0.011295	0.011396	0.011362	0.011429	0.011362	0.011295	0.011295
0.011262	0.011295	0.011262	0.011362	0.011329	0.011363	0.011429
0.011362	0.011329	0.011396	0.011295	0.011429	0.011262	0.011329
0.011496	0.011429	0.011664	0.011497	0.011329	0.011564	0.011262
0.011505	0.011733	0.011551	0.011256	0.011247	0.011355	0.011454
0.012085	0.011518	0.011266	0.011564	0.011564	0.011362	0.011463
0.011396	0.011362	0.011429	0.011329	0.011429	0.011362	0.011362
0.011329	0.011429	0.011631	0.011496	0.011396	0.011362	0.011295
0.011262	0.011262	0.011329	0.011295	0.011497	0.011463	0.011396
0.011497	0.011564	0.011295	0.011295	0.011329	0.011329	0.011330
0.011398	0.011329	0.011329	0.011204	0.011277	0.011236	0.011329
0.011331	0.011266	0.011318	0.011302	0.011230	0.011266	0.011297
0.011564	0.011336	0.011370	0.011262	0.011268	0.011397	0.011363
0.011430	0.011363	0.011363	0.011463	0.011396	0.011765	0.011564
0.011597	0.011463	0.011329	0.011430	0.011228	0.011463	0.011262
0.011329	0.011329	0.011295	0.011262	0.011295	0.011262	0.011362
0.011329	0.011430	0.011362	0.011262	0.011363	0.011463	0.011466
0.011463	0.011362	0.011262	0.011195	0.011329	0.011262	0.011195
0.011298	0.011463	0.011262	0.011463	0.011228	0.011329	0.011295
0.011396	0.011295	0.011329	0.011295	0.011329	0.011195	0.011329
0.011362	0.011295	0.011362	0.011295	0.011329	0.011362	0.011597
0.011698	0.011631	0.011698	0.011397	0.011698	0.011329	0.011295
0.011330	0.011626	0.011426	0.011232	0.011705	0.011197	0.011336
0.011705	0.011604	0.011496	0.011698	0.011402	0.011564	0.011530
0.011463	0.011631	0.011486	0.011349	0.011395	0.011362	0.011432
0.011530	0.011431	0.011567	0.011635	0.011564	0.011631	0.011738
0.011466	0.011637	0.011568	0.011500	0.011767	0.011228	0.011431
0.011396	0.011329	0.011228	0.011228	0.011295	0.011295	0.011295
0.011295	0.011362	0.011262	0.011362	0.011295	0.011295	0.011362
0.011295	0.011731	0.011631	0.011664	0.011194	0.011295	0.011329
0.011329	0.011295	0.011362	0.011396	0.011262	0.011262	0.011262
0.011799	0.011429	0.011429	0.011228	0.011329	0.011497	0.011262
0.011262	0.011329	0.011295	0.011463	0.011262	0.011396	0.011530
0.011463	0.011295	0.011295	0.011228	0.011228	0.011396	0.011329
0.011228	0.011329	0.011195	0.011228	0.011362	0.011497	0.011262
0.011228	0.011362	0.011295	0.011195	0.011530	0.011195	0.011362

0.011437	0.011262	0.011564	0.011396	0.011429	0.011295	0.011329
0.011262	0.011597	0.011194	0.011463	0.011262	0.011262	0.011194
0.011429	0.011631	0.011329	0.011396	0.011228	0.011631	0.011262
0.011262	0.011262	0.011295	0.011396	0.011295	0.011396	0.011463
0.011463	0.011429	0.011262	0.011429	0.011396	0.011194	0.011228
0.011262	0.011194	0.011329	0.011262	0.011228	0.011396	0.011262
0.011295	0.011295	0.011429	0.011463	0.011362	0.011295	0.011295
0.011362	0.011295	0.011396	0.011262	0.011396	0.011496	0.011799
0.011295	0.011698	0.011302	0.017823	0.011387	0.011427	0.011653
0.011614	0.011658	0.011427	0.011292	0.011774	0.011682	0.011425
0.011582	0.011610	0.011216	0.011311	0.011294	0.011356	0.011275
0.011576	0.011470	0.011468	0.011259	0.011429	0.011463	0.011466
0.011335	0.011429	0.011429	0.011396	0.011362	0.011362	0.011329
0.011362	0.011295	0.011329	0.011362	0.011329	0.011262	0.011295
0.011295	0.011362	0.011262	0.011362	0.011396	0.011429	0.011329
0.011362	0.011329	0.011665	0.011665	0.011396	0.011430	0.011463
0.011228	0.011228	0.011262	0.011362	0.011228	0.011463	0.011463
0.011497	0.011496	0.011429	0.011530	0.011698	0.011498	0.011362
0.011362	0.011429	0.011463	0.011295	0.011362	0.011329	0.011362
0.011430	0.011430	0.011463	0.011430	0.011330	0.011530	0.011497
0.011331	0.011262	0.011362	0.011396	0.011362	0.011497	0.011530
0.011698	0.011402	0.011564	0.011530	0.011463	0.011631	0.011262
0.011431	0.011329	0.011295	0.011295	0.011362	0.011262	0.011362
0.011396	0.011429	0.011329	0.011362	0.011329	0.011665	0.011665
0.011396	0.011430	0.011496	0.011429	0.012069	0.011489	0.011412
0.011482	0.011442	0.011552	0.011441	0.011550	0.011474	0.011439
0.012021	0.011337	0.011471	0.011406	0.011680	0.011536	0.011639
0.012046	0.011512	0.011601	0.011664	0.011530	0.011530	0.011567
0.011228	0.011396	0.011530	0.011496	0.011499	0.011496	0.011464
0.011329	0.011262	0.011295	0.011329	0.011228	0.011295	0.011194
0.011329	0.011362	0.011262	0.011228	0.011329	0.011262	0.011295
0.011295	0.011228	0.011295	0.011329	0.011228	0.011429	0.011463
0.011362	0.011429	0.011362	0.011362	0.011295	0.011698	0.011329
0.011373	0.011295	0.011530	0.011535	0.011396	0.011329	0.011396
0.011396	0.011329	0.011295	0.011329	0.011429	0.011429	0.011362
0.011362	0.011496	0.011664	0.011396	0.011597	0.011597	0.011463
0.011295	0.011362	0.011396	0.011799	0.011362	0.011396	0.011362
0.011396	0.011329	0.011329	0.011631	0.011564	0.011597	0.011329
0.011429	0.011496	0.011362	0.011429	0.011396	0.011262	0.011597
0.011362	0.011329	0.011329	0.011496	0.011329	0.011429	0.011664
0.011362	0.011739	0.011732	0.011548	0.011342	0.011434	0.011267
0.011225	0.011761	0.011271	0.011507	0.011353	0.011366	0.011485
0.011570	0.011258	0.011394	0.011258	0.011330	0.011529	0.011425
0.011437	0.011336	0.011412	0.011342	0.011364	0.011497	0.011564
0.011564	0.011430	0.011533	0.011535	0.011670	0.011696	0.011362
0.011597	0.011463	0.011329	0.011430	0.011228	0.011463	0.011262
0.011329	0.011329	0.011295	0.011262	0.011295	0.011262	0.011362
0.011340	0.011340	0.011334	0.011368	0.011505	0.011470	0.011470
0.011469	0.011396	0.011208	0.011204	0.011331	0.011501	0.011501
0.011296	0.011365	0.011531	0.011567	0.011497	0.011531	0.011463
0.011396	0.011463	0.011463	0.011362	0.011933	0.011530	0.011597
0.011631	0.011701	0.011333	0.011407	0.011295	0.011497	0.011463
0.011497	0.011530	0.011530	0.011530	0.011610	0.011664	0.011303
0.011195	0.011199	0.011298	0.011264	0.011368	0.011209	0.011536
0.011335	0.011499	0.011251	0.011360	0.011329	0.011402	0.011295
0.011262	0.011262	0.011329	0.011295	0.011497	0.011463	0.011396
0.011497	0.011564	0.011295	0.011295	0.011329	0.011329	0.011330
0.011398	0.011329	0.011329	0.011364	0.011497	0.011564	0.011564
0.011430	0.011533	0.011535	0.011670	0.011696	0.011362	0.011530
0.011463	0.011463	0.011229	0.011478	0.011408	0.011435	0.011305

0.011473	0.011698	0.011484	0.011366	0.011262	0.011463	0.011329
0.011430	0.011362	0.011262	0.011363	0.011463	0.011466	0.011362
0.011262	0.011195	0.011329	0.011262	0.011195	0.011298	0.011463
0.011362	0.011295	0.011329	0.011362	0.011329	0.011262	0.011463
0.011329	0.011362	0.011362	0.011295	0.011362	0.011597	0.011262
0.011295	0.011698	0.011295	0.011329	0.011295	0.011664	0.011274
0.011791	0.011273	0.011361	0.011503	0.011589	0.011491	0.011227
0.011323	0.011463	0.011564	0.011671	0.011531	0.011669	0.011800
0.011699	0.011737	0.011698	0.011949	0.011664	0.012539	0.011700
0.012439	0.012207	0.012341	0.012612	0.011664	0.011362	0.011665
0.012103	0.012038	0.011631	0.011530	0.011598	0.011598	0.011567
0.011463	0.011467	0.011530	0.011497	0.011867	0.011933	0.011935
0.011834	0.011934	0.012038	0.011804	0.012104	0.012110	0.012171
0.012172	0.012205	0.012152	0.012172	0.012173	0.011396	0.011699
0.011766	0.012270	0.011772	0.012001	0.012474	0.011536	0.011430
0.011396	0.011396	0.011867	0.011883	0.012010	0.011732	0.011397
S038	0.011664	0.011664	0.012001	0.011765	0.011765	0.011832
0.011671	0.011603	0.011832	0.011262	0.011329	0.011296	0.011262
0.011295	0.011463	0.011262	0.011295	0.011362	0.011329	0.011295
0.011262	0.011295	0.011396	0.011262	0.011295	0.011295	0.011637
0.011530	0.011295	0.011194	0.011429	0.011362	0.011329	0.011362
0.011329	0.011429	0.011329	0.000000	0.011362	0.011329	0.011497
0.011262	0.011329	0.011362	0.011295	0.011396	0.011429	0.011362
0.011329	0.011497	0.011396	0.011664	0.011329	0.011597	0.011329
0.011295	0.011396	0.011463	0.011597	0.011698	0.011396	0.011496
0.011362	0.011429	0.011329	0.011564	0.011799	0.011329	0.011396
0.011295	0.011396	0.011362	0.011429	0.011362	0.011362	0.011362
0.011262	0.011295	0.011262	0.011362	0.011329	0.011363	0.011429
0.011362	0.011329	0.011396	0.011295	0.011429	0.011262	0.011329
0.011496	0.011429	0.011664	0.011497	0.011329	0.011564	0.011329
0.011505	0.011733	0.011551	0.011255	0.011247	0.011355	0.011454
0.012085	0.011569	0.011266	0.011564	0.011564	0.011362	0.011463
0.011396	0.011362	0.011429	0.011329	0.011429	0.011362	0.011362
0.011329	0.011429	0.011631	0.011496	0.011396	0.011362	0.011295
0.011262	0.011262	0.011329	0.011295	0.011497	0.011463	0.011396
0.011497	0.011564	0.011295	0.011295	0.011329	0.011329	0.011330
0.011398	0.011329	0.011329	0.011204	0.011277	0.011236	0.011329
0.011331	0.011266	0.011318	0.011302	0.011230	0.011266	0.011297
0.011564	0.011336	0.011370	0.011262	0.011268	0.011397	0.011363
0.011430	0.011364	0.011363	0.011463	0.011396	0.011765	0.011564
0.011597	0.011463	0.011329	0.011430	0.011228	0.011463	0.011262
0.011329	0.011329	0.011295	0.011262	0.011295	0.011262	0.011362
0.011329	0.011430	0.011362	0.011262	0.011363	0.011463	0.011466
0.011463	0.011362	0.011262	0.011195	0.011329	0.011262	0.011195
0.011298	0.011463	0.011262	0.011463	0.011228	0.011329	0.011295
0.011396	0.011295	0.011329	0.011295	0.011329	0.011195	0.011329
0.011362	0.011295	0.011362	0.011295	0.011329	0.011362	0.011597
0.011698	0.011631	0.011698	0.011465	0.011698	0.011329	0.011295
0.011330	0.011626	0.011380	0.011232	0.011705	0.011197	0.011336
0.011705	0.011604	0.011496	0.011698	0.011402	0.011564	0.011463
0.011463	0.011631	0.011486	0.011349	0.011395	0.011362	0.011432
0.011530	0.011431	0.011567	0.011635	0.011564	0.011631	0.011738
0.011466	0.011637	0.011568	0.011500	0.011767	0.011228	0.011431
0.011396	0.011329	0.011228	0.011228	0.011295	0.011295	0.011362
0.011295	0.011362	0.011262	0.011362	0.011295	0.011295	0.011362
0.011295	0.011731	0.011631	0.011664	0.011194	0.011295	0.011329
0.011329	0.011295	0.011362	0.011329	0.011262	0.011262	0.011262
0.011799	0.011429	0.011429	0.011228	0.011329	0.011497	0.011262
0.011262	0.011329	0.011295	0.011463	0.011262	0.011396	0.011530
0.011463	0.011295	0.011295	0.011228	0.011228	0.011396	0.011329

0.011228	0.011329	0.011195	0.011228	0.011362	0.011497	0.011262
0.011228	0.011362	0.011295	0.011195	0.011530	0.011195	0.011362
0.011437	0.011262	0.011564	0.011396	0.011429	0.011295	0.011396
0.011262	0.011597	0.011194	0.011463	0.011262	0.011262	0.011194
0.011429	0.011631	0.011329	0.011396	0.011228	0.011631	0.011262
0.011262	0.011194	0.011295	0.011396	0.011295	0.011396	0.011463
0.011463	0.011429	0.011329	0.011430	0.011396	0.011262	0.011228
0.011262	0.011262	0.011329	0.011262	0.011228	0.011396	0.011262
0.011295	0.011295	0.011429	0.011463	0.011362	0.011295	0.011295
0.011362	0.011295	0.011396	0.011262	0.011396	0.011496	0.011799
0.011295	0.011698	0.011302	0.017823	0.011322	0.011427	0.011653
0.011614	0.011658	0.011427	0.011408	0.011719	0.011682	0.011364
0.011582	0.011549	0.011216	0.011312	0.011294	0.011307	0.011318
0.011576	0.011470	0.011468	0.011391	0.011429	0.011463	0.011466
0.011335	0.011430	0.011429	0.011396	0.011362	0.011362	0.011329
0.011362	0.011295	0.011329	0.011362	0.011329	0.011262	0.011295
0.011295	0.011362	0.011262	0.011363	0.011396	0.011430	0.011329
0.011362	0.011329	0.011665	0.011665	0.011396	0.011430	0.011463
0.011228	0.011228	0.011262	0.011362	0.011228	0.011396	0.011396
0.011497	0.011496	0.011429	0.011530	0.011698	0.011532	0.011362
0.011362	0.011430	0.011463	0.011295	0.011362	0.011329	0.011362
0.011430	0.011430	0.011463	0.011430	0.011330	0.011530	0.011497
0.011365	0.011262	0.011362	0.011463	0.011363	0.011497	0.011530
0.011698	0.011402	0.011564	0.011463	0.011463	0.011631	0.011262
0.011431	0.011329	0.011295	0.011295	0.011362	0.011262	0.011363
0.011396	0.011430	0.011329	0.011362	0.011329	0.011665	0.011665
0.011396	0.011430	0.011496	0.011429	0.012069	0.011489	0.011412
0.011482	0.011442	0.011552	0.011441	0.011550	0.011474	0.011439
0.012022	0.011337	0.011471	0.011406	0.011680	0.011536	0.011639
0.012046	0.011512	0.011601	0.011664	0.011530	0.011530	0.011567
0.011228	0.011396	0.011530	0.011496	0.011499	0.011496	0.011464
0.011329	0.011262	0.011295	0.011329	0.011228	0.011295	0.011194
0.011329	0.011362	0.011262	0.011228	0.011329	0.011262	0.011295
0.011295	0.011228	0.011295	0.011329	0.011228	0.011429	0.011463
0.011362	0.011429	0.011362	0.011362	0.011295	0.011698	0.011329
0.011373	0.011295	0.011530	0.011535	0.011396	0.011396	0.011396
0.011396	0.011329	0.011295	0.011329	0.011429	0.011429	0.011362
0.011362	0.011497	0.011664	0.011396	0.011597	0.011597	0.011463
0.011295	0.011362	0.011396	0.011799	0.011362	0.011396	0.011362
0.011396	0.011329	0.011329	0.011631	0.011564	0.011597	0.011329
0.011429	0.011497	0.011362	0.011429	0.011396	0.011262	0.011597
0.011295	0.011329	0.011329	0.011497	0.011329	0.011429	0.011664
0.011362	0.011739	0.011732	0.011548	0.011342	0.011434	0.011370
0.011225	0.011761	0.011271	0.011462	0.011353	0.011417	0.011432
0.011570	0.011258	0.011394	0.011192	0.011276	0.011395	0.011425
0.011437	0.011336	0.011412	0.011342	0.011364	0.011497	0.011564
0.011564	0.011430	0.011534	0.011535	0.011670	0.011696	0.011362
0.011597	0.011463	0.011329	0.011430	0.011228	0.011463	0.011262
0.011329	0.011329	0.011295	0.011262	0.011295	0.011262	0.011362
0.011340	0.011340	0.011334	0.011368	0.011505	0.011470	0.011470
0.011469	0.011396	0.011208	0.011204	0.011331	0.011501	0.011501
0.011296	0.011365	0.011599	0.011567	0.011497	0.011531	0.011463
0.011396	0.011463	0.011463	0.011362	0.011933	0.011530	0.011597
0.011631	0.011701	0.011333	0.011407	0.011295	0.011497	0.011463
0.011497	0.011530	0.011530	0.011530	0.011611	0.011664	0.011303
0.011195	0.011199	0.011299	0.011264	0.011368	0.011210	0.011536
0.011335	0.011499	0.011251	0.011360	0.011329	0.011402	0.011295
0.011262	0.011262	0.011329	0.011295	0.011497	0.011463	0.011396
0.011497	0.011564	0.011295	0.011295	0.011329	0.011329	0.011330
0.011398	0.011329	0.011329	0.011364	0.011497	0.011564	0.011564

0.011430	0.011534	0.011535	0.011670	0.011696	0.011362	0.011530
0.011463	0.011463	0.011297	0.011478	0.011408	0.011435	0.011270
0.011473	0.011698	0.011483	0.011366	0.011262	0.011463	0.011329
0.011430	0.011362	0.011262	0.011363	0.011463	0.011466	0.011362
0.011262	0.011195	0.011329	0.011262	0.011195	0.011298	0.011463
0.011362	0.011295	0.011329	0.011362	0.011329	0.011262	0.011463
0.011329	0.011362	0.011362	0.011295	0.011362	0.011597	0.011262
0.011295	0.011698	0.011295	0.011329	0.011295	0.011664	0.011275
0.011791	0.011273	0.011361	0.011503	0.011589	0.011491	0.011227
0.011323	0.011463	0.011564	0.011671	0.011531	0.011669	0.011800
0.011699	0.011737	0.011698	0.011949	0.011664	0.012539	0.011700
0.012439	0.012207	0.012341	0.012613	0.011664	0.011363	0.011665
0.012103	0.012038	0.011631	0.011530	0.011598	0.011598	0.011567
0.011463	0.011467	0.011530	0.011497	0.011867	0.011933	0.011935
0.011834	0.011934	0.012038	0.011804	0.012104	0.012110	0.012171
0.012172	0.012205	0.012152	0.012172	0.012173	0.011396	0.011699
0.011767	0.012270	0.011772	0.012001	0.012474	0.011536	0.011430
0.011396	0.011396	0.011799	0.011924	0.012010	0.011732	0.011397
S039	0.011698	0.011698	0.012034	0.011799	0.011799	0.011866
0.011705	0.011637	0.011866	0.011295	0.011362	0.011329	0.011295
0.011329	0.011496	0.011295	0.011262	0.011396	0.011362	0.011329
0.011295	0.011329	0.011429	0.011295	0.011329	0.011329	0.011671
0.011564	0.011329	0.011228	0.011463	0.011396	0.011362	0.011396
0.011362	0.011463	0.011295	0.011362	0.000000	0.011362	0.011530
0.011295	0.011362	0.011127	0.011262	0.011429	0.011396	0.011396
0.011362	0.011262	0.011430	0.011698	0.011094	0.011631	0.011094
0.011329	0.011429	0.011362	0.011631	0.011731	0.011429	0.011530
0.011396	0.011463	0.011362	0.011597	0.011832	0.011362	0.011429
0.011329	0.011429	0.011396	0.011463	0.011396	0.011329	0.011329
0.011295	0.011329	0.011295	0.011396	0.011362	0.011397	0.011463
0.011396	0.011362	0.011429	0.011329	0.011463	0.011295	0.011362
0.011530	0.011463	0.011698	0.011530	0.011362	0.011597	0.011295
0.011505	0.011732	0.011551	0.011295	0.011284	0.011355	0.011454
0.012121	0.011569	0.011266	0.011530	0.011597	0.011396	0.011496
0.011429	0.011396	0.011463	0.011362	0.011463	0.011396	0.011396
0.011362	0.011463	0.011664	0.011530	0.011429	0.011396	0.011329
0.011295	0.011295	0.011362	0.011329	0.011530	0.011497	0.011429
0.011530	0.011597	0.011329	0.011329	0.011362	0.011362	0.011364
0.011432	0.011362	0.011362	0.011240	0.011313	0.011271	0.011363
0.011365	0.011301	0.011355	0.011336	0.011264	0.011300	0.011330
0.011600	0.011370	0.011405	0.011296	0.011302	0.011430	0.011396
0.011464	0.011397	0.011396	0.011496	0.011429	0.011732	0.011597
0.011631	0.011497	0.011362	0.011463	0.011262	0.011496	0.011295
0.011362	0.011362	0.011329	0.011295	0.011329	0.011295	0.011396
0.011362	0.011463	0.011396	0.011295	0.011397	0.011497	0.011499
0.011497	0.011396	0.011295	0.011229	0.011362	0.011295	0.011228
0.011332	0.011497	0.011295	0.011497	0.011262	0.011362	0.011329
0.011430	0.011329	0.011362	0.011329	0.011362	0.011228	0.011362
0.011396	0.011329	0.011396	0.011329	0.011362	0.011396	0.011631
0.011732	0.011664	0.011732	0.011363	0.011732	0.011362	0.011329
0.011364	0.011667	0.011472	0.011266	0.011739	0.011231	0.011296
0.011739	0.011638	0.011530	0.011732	0.011436	0.011597	0.011564
0.011429	0.011664	0.011486	0.011307	0.011354	0.011396	0.011466
0.011564	0.011431	0.011601	0.011635	0.011597	0.011665	0.011738
0.011500	0.011637	0.011534	0.011533	0.011767	0.011262	0.011464
0.011429	0.011362	0.011262	0.011262	0.011329	0.011329	0.011329
0.011329	0.011396	0.011295	0.011396	0.011329	0.011329	0.011329
0.011329	0.011765	0.011664	0.011698	0.011228	0.011329	0.011362
0.011362	0.011329	0.011396	0.011429	0.011295	0.011295	0.011295
0.011832	0.011463	0.011463	0.011262	0.011362	0.011463	0.011295

0.011295	0.011362	0.011329	0.011497	0.011295	0.011429	0.011564
0.011496	0.011329	0.011329	0.011262	0.011262	0.011429	0.011362
0.011262	0.011362	0.011228	0.011262	0.011396	0.011530	0.011295
0.011262	0.011396	0.011329	0.011228	0.011564	0.011228	0.011396
0.011437	0.011295	0.011597	0.011429	0.011463	0.011329	0.011094
0.011295	0.011631	0.011228	0.011496	0.011295	0.011295	0.011228
0.011463	0.011665	0.011362	0.011429	0.011262	0.011664	0.011295
0.011295	0.011295	0.011329	0.011429	0.011329	0.011429	0.011496
0.011496	0.011463	0.011295	0.011463	0.011429	0.011228	0.011262
0.011295	0.011228	0.011362	0.011295	0.011262	0.011429	0.011295
0.011329	0.011329	0.011463	0.011496	0.011396	0.011329	0.011329
0.011396	0.011329	0.011429	0.011295	0.011429	0.011530	0.011832
0.011329	0.011732	0.011336	0.017861	0.011387	0.011611	0.011688
0.011648	0.011693	0.011427	0.011350	0.011829	0.011716	0.011485
0.011534	0.011671	0.011216	0.011353	0.011333	0.011406	0.011361
0.011576	0.011507	0.011505	0.011126	0.011463	0.011497	0.011500
0.011335	0.011463	0.011463	0.011429	0.011396	0.011396	0.011362
0.011396	0.011329	0.011362	0.011396	0.011362	0.011295	0.011329
0.011329	0.011396	0.011295	0.011396	0.011429	0.011463	0.011362
0.011396	0.011362	0.011698	0.011698	0.011430	0.011396	0.011497
0.011262	0.011262	0.011295	0.011396	0.011262	0.011497	0.011497
0.011530	0.011530	0.011463	0.011564	0.011732	0.011565	0.011396
0.011396	0.011396	0.011497	0.011329	0.011396	0.011362	0.011329
0.011463	0.011463	0.011497	0.011463	0.011363	0.011564	0.011530
0.011399	0.011295	0.011396	0.011430	0.011396	0.011530	0.011564
0.011732	0.011436	0.011597	0.011564	0.011429	0.011664	0.011295
0.011397	0.011363	0.011329	0.011329	0.011396	0.011295	0.011396
0.011429	0.011463	0.011362	0.011396	0.011362	0.011698	0.011698
0.011430	0.011396	0.011530	0.011463	0.012103	0.011453	0.011377
0.011447	0.011407	0.011552	0.011441	0.011515	0.011439	0.011474
0.012021	0.011302	0.011437	0.011371	0.011645	0.011570	0.011639
0.012046	0.011475	0.011635	0.011698	0.011564	0.011564	0.011601
0.011262	0.011429	0.011564	0.011530	0.011533	0.011530	0.011498
0.011362	0.011295	0.011329	0.011362	0.011262	0.011329	0.011228
0.011362	0.011396	0.011295	0.011262	0.011362	0.011295	0.011329
0.011262	0.011262	0.011329	0.011362	0.011262	0.011463	0.011496
0.011396	0.011463	0.011396	0.011396	0.011329	0.011731	0.011362
0.011373	0.011329	0.011564	0.011535	0.011429	0.011362	0.011429
0.011429	0.011362	0.011329	0.011362	0.011463	0.011463	0.011396
0.011396	0.011530	0.011698	0.011429	0.011631	0.011631	0.011496
0.011329	0.011396	0.011429	0.011832	0.011396	0.011429	0.011396
0.011429	0.011362	0.011362	0.011665	0.011597	0.011631	0.011362
0.011463	0.011530	0.011396	0.011463	0.011429	0.011295	0.011631
0.011396	0.011362	0.011362	0.011530	0.011362	0.011463	0.011698
0.011396	0.011773	0.011765	0.011548	0.011342	0.011468	0.011267
0.011390	0.011761	0.011306	0.011507	0.011353	0.011213	0.011485
0.011570	0.011258	0.011450	0.011324	0.011330	0.011529	0.011425
0.011437	0.011336	0.011448	0.011377	0.011397	0.011530	0.011597
0.011597	0.011464	0.011567	0.011569	0.011704	0.011732	0.011396
0.011631	0.011497	0.011362	0.011463	0.011262	0.011496	0.011295
0.011362	0.011362	0.011329	0.011295	0.011329	0.011295	0.011396
0.011375	0.011375	0.011300	0.011403	0.011539	0.011504	0.011436
0.011503	0.011430	0.011208	0.011204	0.011365	0.011535	0.011535
0.011329	0.011399	0.011632	0.011601	0.011531	0.011565	0.011496
0.011429	0.011496	0.011496	0.011396	0.011967	0.011564	0.011631
0.011665	0.011737	0.011367	0.011441	0.011329	0.011530	0.011496
0.011531	0.011564	0.011564	0.011564	0.011650	0.011698	0.011303
0.011195	0.011199	0.011298	0.011264	0.011368	0.011209	0.011536
0.011335	0.011455	0.011203	0.011322	0.011363	0.011437	0.011329
0.011295	0.011295	0.011362	0.011329	0.011530	0.011497	0.011429

0.011530	0.011597	0.011329	0.011329	0.011362	0.011362	0.011364
0.011432	0.011362	0.011362	0.011397	0.011530	0.011597	0.011597
0.011464	0.011567	0.011569	0.011704	0.011732	0.011396	0.011564
0.011496	0.011496	0.011263	0.011513	0.011442	0.011469	0.011340
0.011508	0.011698	0.011484	0.011400	0.011295	0.011497	0.011362
0.011463	0.011396	0.011295	0.011397	0.011497	0.011499	0.011396
0.011295	0.011229	0.011362	0.011295	0.011228	0.011332	0.011497
0.011396	0.011329	0.011362	0.011396	0.011362	0.011295	0.011463
0.011362	0.011396	0.011396	0.011329	0.011396	0.011631	0.011295
0.011329	0.011731	0.011329	0.011362	0.011329	0.011698	0.011317
0.011735	0.011273	0.011361	0.011503	0.011589	0.011527	0.011269
0.011323	0.011496	0.011530	0.011637	0.011498	0.011635	0.011766
0.011666	0.011669	0.011664	0.011983	0.011698	0.012573	0.011734
0.012473	0.012241	0.012375	0.012646	0.011698	0.011396	0.011631
0.012137	0.012005	0.011664	0.011564	0.011632	0.011632	0.011601
0.011496	0.011501	0.011564	0.011531	0.011901	0.011967	0.011968
0.011867	0.011968	0.012072	0.011838	0.012138	0.012144	0.012205
0.012205	0.012239	0.012186	0.012205	0.012207	0.011429	0.011732
0.011800	0.012303	0.011806	0.012034	0.012508	0.011570	0.011464
0.011429	0.011429	0.011900	0.011883	0.012045	0.011765	0.011431
S040	0.011664	0.011664	0.012000	0.011765	0.011765	0.011832
0.011671	0.011603	0.011832	0.011262	0.011329	0.011296	0.011262
0.011295	0.011463	0.011262	0.011295	0.011362	0.011329	0.011295
0.011262	0.011295	0.011396	0.011262	0.011295	0.011295	0.011637
0.011530	0.011295	0.011194	0.011429	0.011362	0.011329	0.011362
0.011329	0.011429	0.011329	0.011329	0.011362	0.000000	0.011496
0.011262	0.011329	0.011362	0.011295	0.011396	0.011429	0.011362
0.011329	0.011497	0.011396	0.011664	0.011329	0.011597	0.011329
0.011295	0.011396	0.011463	0.011597	0.011698	0.011396	0.011496
0.011362	0.011429	0.011329	0.011496	0.011799	0.011329	0.011396
0.011295	0.011396	0.011362	0.011429	0.011362	0.011362	0.011362
0.011262	0.011295	0.011262	0.011362	0.011329	0.011363	0.011429
0.011362	0.011329	0.011396	0.011295	0.011429	0.011262	0.011329
0.011496	0.011429	0.011664	0.011295	0.011329	0.011564	0.011329
0.011357	0.011732	0.011551	0.011255	0.011247	0.011207	0.011454
0.012085	0.011518	0.011266	0.011564	0.011564	0.011362	0.011463
0.011396	0.011362	0.011429	0.011329	0.011429	0.011362	0.011362
0.011329	0.011429	0.011631	0.011496	0.011396	0.011362	0.011295
0.011262	0.011262	0.011329	0.011295	0.011497	0.011463	0.011396
0.011497	0.011564	0.011295	0.011295	0.011329	0.011329	0.011330
0.011398	0.011329	0.011329	0.011204	0.011277	0.011235	0.011329
0.011128	0.011266	0.011318	0.011302	0.011230	0.011266	0.011297
0.011564	0.011336	0.011370	0.011262	0.011268	0.011397	0.011363
0.011430	0.011363	0.011363	0.011463	0.011396	0.011765	0.011564
0.011597	0.011463	0.011329	0.011430	0.011228	0.011463	0.011262
0.011329	0.011329	0.011295	0.011262	0.011295	0.011262	0.011362
0.011329	0.011429	0.011362	0.011262	0.011363	0.011463	0.011466
0.011463	0.011362	0.011262	0.011195	0.011329	0.011262	0.011195
0.011298	0.011463	0.011262	0.011463	0.011228	0.011329	0.011295
0.011396	0.011295	0.011329	0.011295	0.011329	0.011195	0.011329
0.011362	0.011295	0.011362	0.011295	0.011329	0.011362	0.011597
0.011698	0.011631	0.011698	0.011465	0.011698	0.011329	0.011295
0.011330	0.011626	0.011380	0.011232	0.011705	0.011197	0.011296
0.011705	0.011604	0.011496	0.011698	0.011402	0.011564	0.011530
0.011463	0.011631	0.011486	0.011349	0.011395	0.011362	0.011432
0.011530	0.011431	0.011567	0.011635	0.011564	0.011631	0.011738
0.011466	0.011637	0.011568	0.011297	0.011766	0.011228	0.011431
0.011396	0.011329	0.011228	0.011228	0.011295	0.011295	0.011362
0.011295	0.011362	0.011262	0.011362	0.011295	0.011295	0.011362
0.011295	0.011731	0.011631	0.011664	0.011194	0.011295	0.011329

0.011329	0.011295	0.011362	0.011396	0.011262	0.011262	0.011262
0.011799	0.011429	0.011429	0.011228	0.011329	0.011497	0.011262
0.011262	0.011329	0.011295	0.011463	0.011262	0.011396	0.011530
0.011463	0.011295	0.011295	0.011228	0.011228	0.011396	0.011329
0.011228	0.011329	0.011194	0.011228	0.011161	0.011497	0.011262
0.011228	0.011161	0.011295	0.011194	0.011530	0.011195	0.011362
0.011437	0.011262	0.011564	0.011396	0.011295	0.011295	0.011396
0.011262	0.011597	0.011194	0.011463	0.011262	0.011262	0.011194
0.011429	0.011631	0.011329	0.011396	0.011228	0.011631	0.011262
0.011262	0.011262	0.011295	0.011396	0.011295	0.011396	0.011463
0.011262	0.011429	0.011329	0.011429	0.011396	0.011262	0.011228
0.011262	0.011262	0.011329	0.011262	0.011228	0.011396	0.011262
0.011295	0.011295	0.011429	0.011463	0.011362	0.011295	0.011295
0.011362	0.011295	0.011396	0.011262	0.011396	0.011496	0.011799
0.011295	0.011698	0.011302	0.017823	0.011191	0.011488	0.011653
0.011614	0.011658	0.011270	0.011292	0.011774	0.011682	0.011425
0.011534	0.011610	0.011216	0.011311	0.011255	0.011356	0.011318
0.011529	0.011470	0.011468	0.011193	0.011429	0.011463	0.011466
0.011335	0.011429	0.011429	0.011396	0.011362	0.011362	0.011329
0.011362	0.011295	0.011329	0.011362	0.011329	0.011262	0.011295
0.011295	0.011362	0.011262	0.011362	0.011396	0.011228	0.011329
0.011362	0.011329	0.011665	0.011665	0.011396	0.011430	0.011463
0.011228	0.011228	0.011262	0.011362	0.011228	0.011463	0.011463
0.011497	0.011496	0.011429	0.011530	0.011698	0.011532	0.011362
0.011362	0.011429	0.011463	0.011295	0.011362	0.011329	0.011362
0.011430	0.011430	0.011463	0.011430	0.011330	0.011530	0.011497
0.011365	0.011262	0.011362	0.011463	0.011362	0.011497	0.011530
0.011698	0.011402	0.011564	0.011530	0.011463	0.011631	0.011262
0.011431	0.011329	0.011295	0.011295	0.011362	0.011262	0.011362
0.011396	0.011228	0.011329	0.011362	0.011329	0.011665	0.011665
0.011396	0.011430	0.011496	0.011429	0.012069	0.011489	0.011412
0.011271	0.011441	0.011552	0.011441	0.011550	0.011474	0.011439
0.012021	0.011337	0.011471	0.011406	0.011680	0.011536	0.011639
0.012046	0.011512	0.011601	0.011664	0.011530	0.011530	0.011567
0.011228	0.011396	0.011530	0.011496	0.011499	0.011496	0.011464
0.011329	0.011262	0.011295	0.011329	0.011228	0.011295	0.011194
0.011262	0.011295	0.011262	0.011228	0.011329	0.011262	0.011295
0.011295	0.011228	0.011295	0.011329	0.011228	0.011429	0.011329
0.011228	0.011295	0.011161	0.011295	0.011295	0.011698	0.011329
0.011373	0.011295	0.011530	0.011535	0.011396	0.011396	0.011396
0.011396	0.011329	0.011295	0.011329	0.011429	0.011429	0.011362
0.011362	0.011496	0.011664	0.011396	0.011597	0.011597	0.011463
0.011295	0.011362	0.011396	0.011799	0.011362	0.011396	0.011362
0.011396	0.011329	0.011329	0.011631	0.011564	0.011597	0.011329
0.011429	0.011496	0.011362	0.011429	0.011396	0.011262	0.011597
0.011362	0.011329	0.011329	0.011496	0.011329	0.011429	0.011664
0.011362	0.011739	0.011731	0.011548	0.011229	0.011434	0.011267
0.011280	0.011761	0.011271	0.011462	0.011353	0.011366	0.011379
0.011468	0.011192	0.011339	0.011126	0.011276	0.011328	0.011425
0.011437	0.011336	0.011412	0.011342	0.011364	0.011497	0.011564
0.011564	0.011430	0.011533	0.011535	0.011670	0.011696	0.011362
0.011597	0.011463	0.011329	0.011430	0.011228	0.011463	0.011262
0.011329	0.011329	0.011295	0.011262	0.011295	0.011262	0.011362
0.011340	0.011340	0.011334	0.011368	0.011505	0.011470	0.011470
0.011469	0.011396	0.011208	0.011204	0.011331	0.011501	0.011501
0.011296	0.011365	0.011599	0.011567	0.011497	0.011531	0.011463
0.011396	0.011463	0.011463	0.011362	0.011933	0.011530	0.011597
0.011631	0.011665	0.011333	0.011407	0.011295	0.011497	0.011463
0.011497	0.011530	0.011530	0.011530	0.011650	0.011664	0.011303
0.011195	0.011199	0.011298	0.011264	0.011368	0.011209	0.011536

0.011335	0.011455	0.011203	0.011360	0.011329	0.011402	0.011295	
0.011262	0.011262	0.011329	0.011295	0.011497	0.011463	0.011396	
0.011497	0.011564	0.011295	0.011295	0.011329	0.011329	0.011330	
0.011398	0.011329	0.011329	0.011364	0.011497	0.011564	0.011564	
0.011430	0.011533	0.011535	0.011670	0.011696	0.011362	0.011530	
0.011463	0.011463	0.011297	0.011478	0.011408	0.011435	0.011305	
0.011473	0.011652	0.011441	0.011366	0.011262	0.011463	0.011329	
0.011429	0.011362	0.011262	0.011363	0.011463	0.011466	0.011362	
0.011262	0.011195	0.011329	0.011262	0.011195	0.011298	0.011463	
0.011362	0.011295	0.011329	0.011362	0.011329	0.011262	0.011463	
0.011329	0.011362	0.011362	0.011295	0.011161	0.011530	0.011262	
0.011295	0.011698	0.011295	0.011329	0.011295	0.011664	0.011274	
0.011678	0.011273	0.011311	0.011503	0.011589	0.011491	0.011227	
0.011218	0.011463	0.011564	0.011671	0.011531	0.011669	0.011800	
0.011699	0.011737	0.011698	0.011949	0.011664	0.012539	0.011700	
0.012439	0.012207	0.012341	0.012612	0.011664	0.011362	0.011665	
0.012103	0.012038	0.011631	0.011530	0.011598	0.011598	0.011567	
0.011463	0.011467	0.011530	0.011497	0.011867	0.011933	0.011935	
0.011834	0.011934	0.012038	0.011804	0.012104	0.012110	0.012171	
0.012171	0.012205	0.012152	0.012172	0.012173	0.011396	0.011699	
0.011766	0.012270	0.011772	0.012001	0.012474	0.011536	0.011430	
0.011396	0.011396	0.011867	0.011924	0.012010	0.011731	0.011397	
S041	0.011832	0.011832	0.012169	0.011933	0.011933	0.012000	
0.011841	0.011773	0.012000	0.011430	0.011496	0.011464	0.011429	
0.011463	0.011631	0.011429	0.011463	0.011530	0.011497	0.011463	
0.011429	0.011463	0.011564	0.011430	0.011463	0.011463	0.011807	
0.011698	0.011463	0.011362	0.011597	0.011530	0.011497	0.011530	
0.011497	0.011597	0.011496	0.011497	0.011530	0.011496	0.000000	
0.011429	0.011496	0.011530	0.011463	0.011564	0.011597	0.011530	
0.011497	0.011665	0.011564	0.011832	0.011496	0.011765	0.011496	
0.011463	0.011564	0.011631	0.011765	0.011866	0.011564	0.011664	
0.011530	0.011597	0.011497	0.011731	0.011967	0.011497	0.011564	
0.011463	0.011564	0.011530	0.011597	0.011530	0.011530	0.011530	
0.011429	0.011463	0.011429	0.011530	0.011496	0.011531	0.011597	
0.011530	0.011496	0.011496	0.011463	0.011597	0.011429	0.011496	
0.011664	0.011597	0.011832	0.011665	0.011497	0.011732	0.011496	
0.011691	0.011920	0.011739	0.011451	0.011433	0.011539	0.011651	
0.012268	0.011772	0.011472	0.011731	0.011732	0.011530	0.011631	
0.011564	0.011530	0.011597	0.011496	0.011597	0.011530	0.011530	
0.011496	0.011597	0.011799	0.011664	0.011564	0.011530	0.011463	
0.011430	0.011429	0.011497	0.011463	0.011665	0.011631	0.011564	
0.011665	0.011732	0.011463	0.011463	0.011497	0.011497	0.011499	
0.011567	0.011497	0.011497	0.011384	0.011422	0.011376	0.011497	
0.011501	0.011438	0.011503	0.011474	0.011400	0.011437	0.011466	
0.011744	0.011508	0.011473	0.011430	0.011440	0.011565	0.011531	
0.011598	0.011532	0.011531	0.011631	0.011564	0.011933	0.011665	
0.011765	0.011631	0.011497	0.011598	0.011396	0.011631	0.011430	
0.011497	0.011496	0.011463	0.011429	0.011463	0.011430	0.011530	
0.011497	0.011597	0.011530	0.011430	0.011532	0.011631	0.011635	
0.011631	0.011530	0.011430	0.011364	0.011497	0.011430	0.011363	
0.011468	0.011631	0.011430	0.011631	0.011396	0.011497	0.011463	
0.011564	0.011463	0.011497	0.011463	0.011497	0.011362	0.011497	
0.011530	0.011463	0.011530	0.011463	0.011497	0.011530	0.011765	
0.011866	0.011799	0.011866	0.011633	0.011867	0.011497	0.011463	
0.011499	0.011788	0.011609	0.011403	0.011875	0.011367	0.011532	
0.011875	0.011774	0.011664	0.011866	0.011572	0.011732	0.011698	
0.011631	0.011799	0.011629	0.011472	0.011521	0.011530	0.011568	
0.011698	0.011600	0.011736	0.011737	0.011732	0.011799	0.011907	
0.011636	0.011739	0.011737	0.011669	0.011935	0.011396	0.011599	
0.011564	0.011497	0.011396	0.011396	0.011463	0.011463	0.011530	

0.011463	0.011530	0.011429	0.011530	0.011463	0.011463	0.011530
0.011463	0.011899	0.011799	0.011832	0.011362	0.011463	0.011497
0.011497	0.011463	0.011530	0.011564	0.011429	0.011429	0.011429
0.011967	0.011597	0.011597	0.011396	0.011496	0.011665	0.011429
0.011429	0.011496	0.011463	0.011631	0.011429	0.011564	0.011698
0.011631	0.011463	0.011463	0.011396	0.011396	0.011564	0.011496
0.011396	0.011496	0.011362	0.011396	0.011530	0.011665	0.011429
0.011396	0.011530	0.011463	0.011362	0.011698	0.011362	0.011530
0.011608	0.011429	0.011732	0.011564	0.011597	0.011463	0.011564
0.011429	0.011765	0.011362	0.011631	0.011429	0.011429	0.011362
0.011597	0.011799	0.011497	0.011564	0.011396	0.011799	0.011429
0.011429	0.011429	0.011463	0.011564	0.011463	0.011564	0.011631
0.011631	0.011597	0.011496	0.011597	0.011564	0.011429	0.011396
0.011429	0.011429	0.011497	0.011429	0.011396	0.011564	0.011429
0.011463	0.011463	0.011597	0.011631	0.011530	0.011463	0.011463
0.011530	0.011463	0.011564	0.011429	0.011564	0.011664	0.011967
0.011463	0.011866	0.011474	0.018012	0.011584	0.011611	0.011828
0.011787	0.011834	0.011584	0.011466	0.012050	0.011854	0.011729
0.011724	0.011915	0.011411	0.011479	0.011490	0.011603	0.011447
0.011717	0.011656	0.011653	0.011391	0.011597	0.011631	0.011635
0.011438	0.011597	0.011597	0.011564	0.011530	0.011530	0.011497
0.011530	0.011463	0.011497	0.011530	0.011497	0.011430	0.011463
0.011463	0.011530	0.011430	0.011530	0.011564	0.011597	0.011496
0.011530	0.011497	0.011833	0.011833	0.011564	0.011598	0.011631
0.011396	0.011396	0.011430	0.011530	0.011396	0.011631	0.011631
0.011665	0.011664	0.011597	0.011698	0.011866	0.011700	0.011530
0.011530	0.011597	0.011631	0.011463	0.011530	0.011497	0.011530
0.011598	0.011598	0.011631	0.011598	0.011498	0.011698	0.011665
0.011263	0.011430	0.011530	0.011631	0.011530	0.011665	0.011698
0.011866	0.011572	0.011732	0.011698	0.011631	0.011799	0.011430
0.011599	0.011497	0.011463	0.011463	0.011530	0.011430	0.011530
0.011564	0.011597	0.011496	0.011530	0.011497	0.011833	0.011833
0.011564	0.011598	0.011597	0.011597	0.012204	0.011667	0.011552
0.011658	0.011615	0.011693	0.011614	0.011725	0.011646	0.011612
0.012159	0.011510	0.011642	0.011579	0.011852	0.011706	0.011741
0.012182	0.011663	0.011770	0.011832	0.011698	0.011698	0.011736
0.011396	0.011564	0.011698	0.011664	0.011668	0.011664	0.011633
0.011496	0.011429	0.011463	0.011496	0.011396	0.011463	0.011362
0.011496	0.011530	0.011429	0.011396	0.011497	0.011429	0.011463
0.011463	0.011396	0.011463	0.011497	0.011396	0.011597	0.011631
0.011530	0.011597	0.011530	0.011530	0.011463	0.011866	0.011496
0.011512	0.011463	0.011699	0.011705	0.011564	0.011564	0.011564
0.011564	0.011497	0.011463	0.011497	0.011597	0.011597	0.011530
0.011530	0.011664	0.011832	0.011564	0.011765	0.011765	0.011631
0.011463	0.011530	0.011564	0.011967	0.011530	0.011564	0.011530
0.011564	0.011496	0.011496	0.011799	0.011732	0.011765	0.011497
0.011597	0.011664	0.011530	0.011597	0.011564	0.011429	0.011765
0.011530	0.011496	0.011496	0.011664	0.011497	0.011597	0.011833
0.011530	0.011909	0.011900	0.011751	0.011568	0.011604	0.011525
0.011445	0.011956	0.011447	0.011730	0.011597	0.011722	0.011751
0.011774	0.011587	0.011673	0.011523	0.011599	0.011730	0.011628
0.011625	0.011532	0.011589	0.011518	0.011532	0.011665	0.011732
0.011732	0.011598	0.011703	0.011704	0.011840	0.011873	0.011530
0.011765	0.011631	0.011497	0.011598	0.011396	0.011631	0.011430
0.011497	0.011496	0.011463	0.011429	0.011463	0.011430	0.011530
0.011514	0.011515	0.011505	0.011540	0.011676	0.011641	0.011641
0.011639	0.011565	0.011393	0.011384	0.011501	0.011670	0.011671
0.011464	0.011534	0.011767	0.011736	0.011666	0.011700	0.011631
0.011564	0.011631	0.011631	0.011530	0.012102	0.011698	0.011765
0.011799	0.011879	0.011504	0.011580	0.011463	0.011664	0.011631

0.011666	0.011698	0.011698	0.011698	0.011886	0.011832	0.011442
0.011364	0.011337	0.011469	0.011434	0.011538	0.011359	0.011706
0.011506	0.011586	0.011346	0.011510	0.011497	0.011573	0.011463
0.011430	0.011429	0.011497	0.011463	0.011665	0.011631	0.011564
0.011665	0.011732	0.011463	0.011463	0.011497	0.011497	0.011499
0.011567	0.011497	0.011497	0.011532	0.011665	0.011732	0.011732
0.011598	0.011703	0.011704	0.011840	0.011873	0.011530	0.011698
0.011631	0.011631	0.011466	0.011652	0.011581	0.011605	0.011481
0.011646	0.011881	0.011695	0.011536	0.011430	0.011631	0.011497
0.011597	0.011530	0.011430	0.011532	0.011631	0.011635	0.011530
0.011430	0.011364	0.011497	0.011430	0.011363	0.011468	0.011631
0.011530	0.011463	0.011497	0.011530	0.011497	0.011430	0.011631
0.011496	0.011530	0.011530	0.011463	0.011530	0.011765	0.011429
0.011463	0.011866	0.011463	0.011497	0.011463	0.011832	0.011446
0.012016	0.011485	0.011511	0.011704	0.011793	0.011670	0.011436
0.011480	0.011631	0.011732	0.011841	0.011700	0.011838	0.011968
0.011868	0.011906	0.011866	0.012120	0.011832	0.012708	0.011869
0.012608	0.012376	0.012510	0.012782	0.011832	0.011530	0.011833
0.012239	0.012207	0.011799	0.011698	0.011766	0.011766	0.011737
0.011631	0.011637	0.011698	0.011665	0.012036	0.012102	0.012103
0.012002	0.012103	0.012207	0.011974	0.012239	0.012246	0.012306
0.012307	0.012341	0.012289	0.012307	0.012309	0.011564	0.011833
0.011935	0.012438	0.011908	0.012102	0.012643	0.011705	0.011598
0.011564	0.011564	0.012035	0.012048	0.012186	0.011900	0.011566
S042	0.011597	0.011597	0.011866	0.011631	0.011631	0.011698
0.011535	0.011468	0.011698	0.011195	0.011262	0.011228	0.011194
0.011161	0.011396	0.011127	0.011228	0.011295	0.011262	0.011228
0.011194	0.011228	0.011262	0.011195	0.011228	0.011228	0.011501
0.011396	0.011228	0.011127	0.011295	0.011295	0.011262	0.011295
0.011262	0.011362	0.011262	0.011262	0.011295	0.011262	0.011429
0.000000	0.011262	0.011295	0.011228	0.011262	0.011362	0.011295
0.011262	0.011429	0.011329	0.011530	0.011262	0.011530	0.011262
0.011228	0.011262	0.011396	0.011530	0.011564	0.011262	0.011362
0.011295	0.011362	0.011262	0.011496	0.011664	0.011262	0.011329
0.011228	0.011329	0.011295	0.011362	0.011295	0.011295	0.011295
0.011194	0.011228	0.011194	0.011228	0.011262	0.011296	0.011362
0.011295	0.011262	0.011329	0.011228	0.011295	0.011194	0.011262
0.011429	0.011362	0.011597	0.011429	0.011262	0.011496	0.011262
0.011431	0.011583	0.011437	0.011138	0.011135	0.011281	0.011257
0.011938	0.011314	0.011143	0.011496	0.011496	0.011228	0.011329
0.011329	0.011295	0.011362	0.011262	0.011362	0.011295	0.011295
0.011262	0.011295	0.011564	0.011429	0.011329	0.011295	0.011228
0.011195	0.011127	0.011262	0.011228	0.011362	0.011329	0.011262
0.011362	0.011429	0.011228	0.011228	0.011262	0.011262	0.011263
0.011331	0.011262	0.011262	0.011132	0.011169	0.011165	0.011262
0.011263	0.011198	0.011208	0.011233	0.011162	0.011197	0.011229
0.011420	0.011267	0.011301	0.011195	0.011199	0.011329	0.011228
0.011296	0.011296	0.011296	0.011396	0.011329	0.011698	0.011430
0.011463	0.011329	0.011262	0.011295	0.011161	0.011329	0.011195
0.011262	0.011262	0.011228	0.011194	0.011228	0.011195	0.011295
0.011262	0.011362	0.011295	0.011195	0.011296	0.011396	0.011398
0.011396	0.011295	0.011195	0.011128	0.011262	0.011195	0.011127
0.011230	0.011396	0.011195	0.011396	0.011161	0.011262	0.011228
0.011329	0.011228	0.011262	0.011228	0.011262	0.011127	0.011262
0.011295	0.011228	0.011295	0.011228	0.011262	0.011295	0.011530
0.011564	0.011496	0.011564	0.011397	0.011564	0.011262	0.011228
0.011195	0.011464	0.011335	0.011163	0.011569	0.011129	0.011257
0.011569	0.011468	0.011429	0.011631	0.011333	0.011496	0.011463
0.011396	0.011564	0.011415	0.011266	0.011312	0.011295	0.011365
0.011463	0.011364	0.011500	0.011568	0.011497	0.011564	0.011670

0.011399	0.011569	0.011500	0.011432	0.011699	0.011161	0.011363
0.011329	0.011262	0.011161	0.011161	0.011228	0.011228	0.011295
0.011228	0.011295	0.011194	0.011228	0.011228	0.011228	0.011295
0.011228	0.011597	0.011497	0.011530	0.011127	0.011228	0.011262
0.011262	0.011228	0.011295	0.011262	0.011194	0.011194	0.011194
0.011664	0.011295	0.011362	0.011161	0.011262	0.011429	0.011194
0.011194	0.011262	0.011228	0.011396	0.011194	0.011329	0.011463
0.011396	0.011228	0.011228	0.011161	0.011161	0.011329	0.011262
0.011161	0.011262	0.011127	0.011161	0.011295	0.011362	0.011195
0.011161	0.011295	0.011228	0.011127	0.011396	0.011127	0.011295
0.011368	0.011195	0.011429	0.011329	0.011295	0.011228	0.011329
0.011194	0.011530	0.011127	0.011329	0.011127	0.011127	0.011127
0.011362	0.011497	0.011262	0.011329	0.011161	0.011497	0.011127
0.011194	0.011194	0.011228	0.011329	0.011228	0.011329	0.011396
0.011396	0.011362	0.011262	0.011362	0.011329	0.011194	0.011161
0.011194	0.011194	0.011262	0.000000	0.011161	0.011329	0.011194
0.011161	0.011228	0.011362	0.011329	0.011295	0.011228	0.011228
0.011295	0.011228	0.011329	0.011194	0.011329	0.011429	0.011664
0.011228	0.011564	0.011233	0.017747	0.011191	0.011366	0.011514
0.011475	0.011517	0.011270	0.011234	0.011609	0.011543	0.011303
0.011345	0.011426	0.011099	0.011228	0.011099	0.011208	0.011232
0.011342	0.011284	0.011320	0.000000	0.011362	0.011396	0.011398
0.011266	0.011362	0.011362	0.011262	0.011295	0.011295	0.011262
0.011295	0.011228	0.011262	0.011295	0.011262	0.011195	0.011228
0.011228	0.011295	0.011195	0.011295	0.011329	0.011362	0.011262
0.011228	0.011195	0.011530	0.011530	0.011329	0.011363	0.011396
0.011161	0.011161	0.011195	0.011295	0.011161	0.011396	0.011396
0.011429	0.011429	0.011362	0.011463	0.011564	0.011464	0.011295
0.011295	0.011362	0.011396	0.011228	0.011295	0.011262	0.011295
0.011363	0.011362	0.011396	0.011363	0.011262	0.011463	0.011429
0.011297	0.011195	0.011295	0.011396	0.011295	0.011362	0.011463
0.011631	0.011333	0.011496	0.011463	0.011396	0.011564	0.011195
0.011363	0.011262	0.011228	0.011228	0.011295	0.011195	0.011295
0.011329	0.011362	0.011262	0.011228	0.011195	0.011530	0.011530
0.011329	0.011363	0.011429	0.011362	0.011935	0.011417	0.011341
0.011412	0.011372	0.011482	0.011372	0.011480	0.011405	0.011370
0.011884	0.011268	0.011403	0.011337	0.011611	0.011468	0.011570
0.011910	0.011362	0.011533	0.011597	0.011463	0.011396	0.011499
0.011161	0.011329	0.011463	0.011429	0.011432	0.011362	0.011330
0.011262	0.011194	0.011228	0.011262	0.011161	0.011228	0.011127
0.011194	0.011228	0.011194	0.011161	0.011262	0.011194	0.011228
0.011228	0.011161	0.011228	0.011262	0.011161	0.011362	0.011396
0.011295	0.011295	0.011295	0.011295	0.011228	0.011564	0.011262
0.011234	0.011228	0.011396	0.011399	0.011329	0.011329	0.011329
0.011329	0.011262	0.011228	0.011262	0.011362	0.011362	0.011295
0.011295	0.011429	0.011530	0.011329	0.011530	0.011530	0.011396
0.011228	0.011295	0.011329	0.011664	0.011295	0.011329	0.011295
0.011329	0.011194	0.011194	0.011497	0.011497	0.011463	0.011262
0.011362	0.011429	0.011295	0.011362	0.011329	0.011194	0.011463
0.011295	0.011262	0.011194	0.011429	0.011262	0.011362	0.011597
0.011295	0.011603	0.011597	0.011345	0.000000	0.011298	0.011112
0.011170	0.011566	0.011201	0.011283	0.011109	0.011264	0.011220
0.011315	0.011126	0.011283	0.011126	0.011168	0.011328	0.011303
0.011362	0.011218	0.011272	0.011272	0.011296	0.011362	0.011430
0.011429	0.011296	0.011398	0.011399	0.011535	0.011555	0.011295
0.011463	0.011329	0.011262	0.011295	0.011161	0.011329	0.011195
0.011262	0.011262	0.011228	0.011194	0.011228	0.011195	0.011295
0.011270	0.011270	0.011266	0.011300	0.011437	0.011402	0.011402
0.011401	0.011329	0.011134	0.011132	0.011263	0.011433	0.011433
0.011228	0.011297	0.011531	0.011500	0.011430	0.011464	0.011396

0.011329	0.011396	0.011396	0.011295	0.011799	0.011463	0.011530
0.011564	0.011559	0.011265	0.011337	0.011228	0.011429	0.011396
0.011430	0.011463	0.011463	0.011463	0.011610	0.011597	0.011234
0.011128	0.011130	0.011230	0.011196	0.011231	0.011135	0.011400
0.011266	0.011323	0.011156	0.011285	0.011262	0.011334	0.011228
0.011195	0.011127	0.011262	0.011228	0.011362	0.011329	0.011262
0.011362	0.011429	0.011228	0.011228	0.011262	0.011262	0.011263
0.011331	0.011262	0.011262	0.011296	0.011362	0.011430	0.011429
0.011296	0.011398	0.011399	0.011535	0.011555	0.011295	0.011463
0.011396	0.011396	0.011229	0.011408	0.011338	0.011367	0.011235
0.011405	0.011561	0.011357	0.011298	0.011195	0.011396	0.011262
0.011362	0.011295	0.011195	0.011296	0.011396	0.011398	0.011295
0.011195	0.011128	0.011262	0.011195	0.011127	0.011230	0.011396
0.011295	0.011228	0.011262	0.011295	0.011262	0.011195	0.011329
0.011262	0.011295	0.011295	0.011228	0.011295	0.011530	0.000000
0.011228	0.011564	0.011228	0.011262	0.011228	0.011530	0.011146
0.011510	0.011145	0.011110	0.011302	0.011386	0.011347	0.011102
0.000000	0.011329	0.011429	0.011535	0.011397	0.011534	0.011665
0.011565	0.011601	0.011564	0.011812	0.011530	0.012404	0.011565
0.012304	0.012072	0.012206	0.012477	0.011530	0.011228	0.011530
0.011969	0.011903	0.011497	0.011396	0.011464	0.011464	0.011432
0.011329	0.011331	0.011396	0.011363	0.011733	0.011799	0.011800
0.011699	0.011800	0.011903	0.011669	0.011969	0.011974	0.012036
0.012037	0.012070	0.012016	0.012037	0.012038	0.011329	0.011564
0.011632	0.012135	0.011636	0.011866	0.012339	0.011400	0.011295
0.011262	0.011262	0.011732	0.011759	0.011869	0.011597	0.011262
S043	0.011664	0.011664	0.012000	0.011765	0.011765	0.011832
0.011671	0.011603	0.011832	0.011262	0.011329	0.011296	0.011262
0.011295	0.011463	0.011262	0.011295	0.011362	0.011329	0.011295
0.011262	0.011295	0.011396	0.011262	0.011295	0.011295	0.011637
0.011530	0.011295	0.011194	0.011429	0.011362	0.011329	0.011362
0.011329	0.011429	0.011329	0.011329	0.011362	0.011329	0.011496
0.011262	0.000000	0.011362	0.011295	0.011396	0.011429	0.011362
0.011329	0.011497	0.011396	0.011664	0.011329	0.011597	0.011329
0.011295	0.011396	0.011463	0.011597	0.011698	0.011396	0.011496
0.011362	0.011429	0.011329	0.011563	0.011799	0.011329	0.011396
0.011295	0.011396	0.011362	0.011429	0.011362	0.011362	0.011362
0.011262	0.011295	0.011262	0.011362	0.011329	0.011363	0.011429
0.011362	0.011329	0.011396	0.011295	0.011429	0.011262	0.011329
0.011496	0.011429	0.011664	0.011496	0.011329	0.011564	0.011329
0.011505	0.011732	0.011551	0.011255	0.011247	0.011318	0.011454
0.012085	0.011518	0.011225	0.011564	0.011564	0.011362	0.011463
0.011396	0.011362	0.011429	0.011329	0.011429	0.011362	0.011362
0.011329	0.011429	0.011631	0.011496	0.011396	0.011362	0.011295
0.011262	0.011262	0.011329	0.011295	0.011497	0.011463	0.011396
0.011497	0.011564	0.011295	0.011295	0.011329	0.011329	0.011330
0.011398	0.011329	0.011329	0.011204	0.011277	0.011235	0.011329
0.011331	0.011266	0.011318	0.011302	0.011230	0.011266	0.011297
0.011564	0.011336	0.011370	0.011262	0.011268	0.011397	0.011363
0.011430	0.011363	0.011363	0.011463	0.011396	0.011698	0.011564
0.011597	0.011463	0.011329	0.011430	0.011228	0.011463	0.011262
0.011329	0.011329	0.011295	0.011262	0.011295	0.011262	0.011362
0.011329	0.011429	0.011362	0.011262	0.011363	0.011463	0.011466
0.011463	0.011362	0.011262	0.011195	0.011329	0.011262	0.011195
0.011298	0.011463	0.011262	0.011463	0.011228	0.011329	0.011295
0.011396	0.011295	0.011329	0.011295	0.011329	0.011195	0.011329
0.011362	0.011295	0.011362	0.011295	0.011329	0.011362	0.011597
0.011698	0.011631	0.011698	0.011465	0.011698	0.011329	0.011295
0.011330	0.011626	0.011426	0.011232	0.011705	0.011197	0.011336
0.011705	0.011604	0.011496	0.011698	0.011402	0.011564	0.011530

0.011463	0.011631	0.011486	0.011349	0.011354	0.011362	0.011432
0.011530	0.011431	0.011567	0.011635	0.011564	0.011631	0.011738
0.011466	0.011637	0.011568	0.011500	0.011766	0.011228	0.011431
0.011396	0.011329	0.011228	0.011228	0.011295	0.011295	0.011362
0.011295	0.011362	0.011262	0.011362	0.011295	0.011295	0.011362
0.011295	0.011731	0.011631	0.011664	0.011194	0.011295	0.011329
0.011329	0.011295	0.011362	0.011396	0.011262	0.011262	0.011262
0.011799	0.011429	0.011429	0.011228	0.011329	0.011497	0.011262
0.011262	0.011329	0.011295	0.011463	0.011262	0.011396	0.011530
0.011463	0.011295	0.011295	0.011228	0.011228	0.011396	0.011329
0.011228	0.011329	0.011194	0.011228	0.011362	0.011497	0.011262
0.011228	0.011362	0.011295	0.011194	0.011530	0.011195	0.011362
0.011437	0.011262	0.011564	0.011396	0.011429	0.011295	0.011396
0.011262	0.011597	0.011194	0.011463	0.011262	0.011262	0.011194
0.011429	0.011631	0.011329	0.011396	0.011228	0.011631	0.011262
0.011262	0.011262	0.011295	0.011396	0.011295	0.011396	0.011463
0.011463	0.011429	0.011329	0.011429	0.011396	0.011262	0.011228
0.011262	0.011262	0.011329	0.011262	0.011228	0.011396	0.011262
0.011295	0.011295	0.011429	0.011463	0.011362	0.011295	0.011295
0.011362	0.011295	0.011396	0.011262	0.011396	0.011496	0.011799
0.011295	0.011698	0.011302	0.017785	0.011257	0.011366	0.011653
0.011614	0.011658	0.011374	0.011350	0.011774	0.011682	0.011425
0.011534	0.011610	0.011216	0.011270	0.011255	0.011356	0.011318
0.011529	0.011470	0.011431	0.011193	0.011429	0.011463	0.011466
0.011335	0.011429	0.011429	0.011396	0.011362	0.011362	0.011329
0.011362	0.011295	0.011329	0.011362	0.011329	0.011262	0.011295
0.011295	0.011362	0.011262	0.011362	0.011396	0.011429	0.011329
0.011362	0.011329	0.011665	0.011665	0.011396	0.011430	0.011463
0.011228	0.011228	0.011262	0.011362	0.011228	0.011463	0.011463
0.011497	0.011496	0.011429	0.011530	0.011698	0.011532	0.011362
0.011362	0.011429	0.011463	0.011295	0.011362	0.011329	0.011362
0.011430	0.011430	0.011463	0.011430	0.011330	0.011530	0.011497
0.011365	0.011262	0.011362	0.011463	0.011362	0.011497	0.011530
0.011698	0.011402	0.011564	0.011530	0.011463	0.011631	0.011262
0.011431	0.011329	0.011295	0.011295	0.011362	0.011262	0.011362
0.011396	0.011429	0.011329	0.011362	0.011329	0.011665	0.011665
0.011396	0.011430	0.011496	0.011429	0.012069	0.011489	0.011412
0.011482	0.011441	0.011552	0.011441	0.011550	0.011474	0.011439
0.012021	0.011337	0.011471	0.011406	0.011680	0.011536	0.011639
0.012046	0.011512	0.011601	0.011664	0.011530	0.011530	0.011567
0.011228	0.011396	0.011530	0.011496	0.011499	0.011496	0.011464
0.011329	0.011262	0.011295	0.011329	0.011228	0.011295	0.011194
0.011329	0.011362	0.011262	0.011228	0.011329	0.011262	0.011295
0.011295	0.011228	0.011295	0.011329	0.011228	0.011429	0.011463
0.011362	0.011429	0.011362	0.011362	0.011295	0.011698	0.000000
0.011373	0.011295	0.011530	0.011535	0.011396	0.011396	0.011396
0.011396	0.011329	0.011295	0.011329	0.011429	0.011429	0.011362
0.011362	0.011496	0.011664	0.011396	0.011597	0.011597	0.011463
0.011295	0.011362	0.011396	0.011799	0.011362	0.011396	0.011362
0.011396	0.011329	0.011329	0.011631	0.011564	0.011597	0.011329
0.011429	0.011496	0.011362	0.011429	0.011396	0.011262	0.011597
0.011362	0.011329	0.011329	0.011496	0.011329	0.011429	0.011664
0.011362	0.011739	0.011731	0.011548	0.011286	0.011434	0.011318
0.011280	0.011761	0.011271	0.011417	0.011353	0.011416	0.011379
0.011519	0.011126	0.011338	0.011192	0.011222	0.011395	0.011425
0.011437	0.011336	0.011412	0.011342	0.011364	0.011497	0.011564
0.011564	0.011430	0.011533	0.011535	0.011670	0.011696	0.011362
0.011597	0.011463	0.011329	0.011430	0.011228	0.011463	0.011262
0.011329	0.011329	0.011295	0.011262	0.011295	0.011262	0.011362
0.011340	0.011340	0.011334	0.011368	0.011505	0.011470	0.011470

0.011469	0.011396	0.011208	0.011204	0.011331	0.011501	0.011501
0.011296	0.011365	0.011599	0.011567	0.011497	0.011531	0.011463
0.011396	0.011463	0.011463	0.011362	0.011933	0.011530	0.011597
0.011631	0.011701	0.011333	0.011407	0.011295	0.011497	0.011463
0.011497	0.011530	0.011530	0.011530	0.011650	0.011664	0.011303
0.011195	0.011199	0.011298	0.011264	0.011368	0.011172	0.011536
0.011335	0.011455	0.011203	0.011360	0.011329	0.011402	0.011295
0.011262	0.011262	0.011329	0.011295	0.011497	0.011463	0.011396
0.011497	0.011564	0.011295	0.011295	0.011329	0.011329	0.011330
0.011398	0.011329	0.011329	0.011364	0.011497	0.011564	0.011564
0.011430	0.011533	0.011535	0.011670	0.011696	0.011362	0.011530
0.011463	0.011463	0.011297	0.011478	0.011408	0.011435	0.011305
0.011439	0.011652	0.011483	0.011366	0.011262	0.011463	0.011329
0.011429	0.011362	0.011262	0.011363	0.011463	0.011466	0.011362
0.011262	0.011195	0.011329	0.011262	0.011195	0.011298	0.011463
0.011362	0.011295	0.011329	0.011362	0.011329	0.011262	0.011463
0.011329	0.011362	0.011362	0.011295	0.011362	0.011597	0.011262
0.011295	0.011698	0.011295	0.011329	0.011295	0.011664	0.011232
0.011734	0.011273	0.011311	0.011503	0.011589	0.011491	0.011227
0.011270	0.011463	0.011564	0.011671	0.011531	0.011669	0.011800
0.011699	0.011737	0.011698	0.011949	0.011664	0.012539	0.011700
0.012439	0.012207	0.012341	0.012612	0.011664	0.011362	0.011665
0.012103	0.012038	0.011631	0.011530	0.011598	0.011598	0.011567
0.011463	0.011467	0.011530	0.011497	0.011867	0.011933	0.011935
0.011834	0.011934	0.012038	0.011804	0.012104	0.012110	0.012171
0.012171	0.012205	0.012152	0.012172	0.012173	0.011396	0.011699
0.011766	0.012270	0.011772	0.012001	0.012474	0.011536	0.011430
0.011396	0.011396	0.011867	0.011924	0.012010	0.011731	0.011397
S044 0.011698	0.011698	0.012034	0.011799	0.011799	0.011866	
0.011705	0.011637	0.011866	0.011295	0.011362	0.011329	0.011295
0.011329	0.011496	0.011295	0.011262	0.011396	0.011362	0.011329
0.011295	0.011329	0.011429	0.011295	0.011329	0.011329	0.011671
0.011564	0.011329	0.011228	0.011463	0.011396	0.011362	0.011396
0.011362	0.011463	0.011295	0.011362	0.011127	0.011362	0.011530
0.011295	0.011362	0.000000	0.011262	0.011429	0.011396	0.011396
0.011362	0.011262	0.011430	0.011698	0.011094	0.011631	0.011094
0.011329	0.011429	0.011362	0.011631	0.011732	0.011429	0.011530
0.011396	0.011463	0.011362	0.011597	0.011832	0.011362	0.011429
0.011329	0.011429	0.011396	0.011463	0.011396	0.011329	0.011329
0.011295	0.011329	0.011295	0.011396	0.011362	0.011397	0.011463
0.011396	0.011362	0.011429	0.011329	0.011463	0.011295	0.011362
0.011530	0.011463	0.011698	0.011530	0.011362	0.011597	0.011295
0.011505	0.011733	0.011551	0.011295	0.011284	0.011355	0.011454
0.012121	0.011569	0.011266	0.011530	0.011597	0.011396	0.011497
0.011429	0.011396	0.011463	0.011362	0.011463	0.011396	0.011396
0.011362	0.011463	0.011664	0.011530	0.011429	0.011396	0.011329
0.011295	0.011295	0.011362	0.011329	0.011530	0.011497	0.011429
0.011530	0.011597	0.011329	0.011329	0.011362	0.011362	0.011364
0.011432	0.011362	0.011362	0.011240	0.011313	0.011271	0.011363
0.011365	0.011301	0.011355	0.011336	0.011264	0.011300	0.011330
0.011600	0.011370	0.011405	0.011296	0.011302	0.011430	0.011396
0.011464	0.011397	0.011396	0.011496	0.011429	0.011732	0.011598
0.011631	0.011497	0.011362	0.011463	0.011262	0.011496	0.011295
0.011362	0.011362	0.011329	0.011295	0.011329	0.011295	0.011396
0.011362	0.011463	0.011396	0.011295	0.011397	0.011497	0.011499
0.011497	0.011396	0.011295	0.011229	0.011362	0.011295	0.011228
0.011332	0.011497	0.011295	0.011497	0.011262	0.011362	0.011329
0.011430	0.011329	0.011362	0.011329	0.011362	0.011228	0.011362
0.011396	0.011329	0.011396	0.011329	0.011362	0.011396	0.011631
0.011732	0.011664	0.011732	0.011364	0.011732	0.011362	0.011329

0.011364	0.011667	0.011472	0.011266	0.011739	0.011231	0.011336
0.011739	0.011638	0.011530	0.011732	0.011436	0.011597	0.011564
0.011429	0.011664	0.011522	0.011349	0.011395	0.011396	0.011466
0.011564	0.011465	0.011601	0.011669	0.011597	0.011665	0.011772
0.011500	0.011671	0.011534	0.011534	0.011767	0.011262	0.011465
0.011429	0.011362	0.011262	0.011262	0.011329	0.011329	0.011329
0.011329	0.011396	0.011295	0.011396	0.011329	0.011329	0.011329
0.011329	0.011765	0.011664	0.011698	0.011228	0.011329	0.011362
0.011362	0.011329	0.011396	0.011429	0.011295	0.011295	0.011295
0.011832	0.011463	0.011463	0.011262	0.011362	0.011463	0.011295
0.011295	0.011362	0.011329	0.011497	0.011295	0.011429	0.011564
0.011496	0.011329	0.011329	0.011262	0.011262	0.011429	0.011362
0.011262	0.011362	0.011228	0.011262	0.011396	0.011530	0.011295
0.011262	0.011396	0.011329	0.011228	0.011564	0.011228	0.011396
0.011471	0.011295	0.011597	0.011430	0.011463	0.011329	0.011161
0.011295	0.011631	0.011228	0.011497	0.011295	0.011295	0.011228
0.011463	0.011665	0.011362	0.011429	0.011262	0.011664	0.011295
0.011295	0.011295	0.011329	0.011429	0.011329	0.011429	0.011496
0.011497	0.011463	0.011295	0.011463	0.011429	0.011228	0.011262
0.011295	0.011228	0.011362	0.011295	0.011262	0.011429	0.011295
0.011329	0.011329	0.011463	0.011496	0.011396	0.011329	0.011329
0.011396	0.011329	0.011429	0.011295	0.011429	0.011530	0.011832
0.011329	0.011732	0.011336	0.017861	0.011387	0.011611	0.011688
0.011648	0.011693	0.011427	0.011350	0.011829	0.011716	0.011485
0.011534	0.011671	0.011216	0.011353	0.011333	0.011406	0.011361
0.011576	0.011507	0.011505	0.011193	0.011463	0.011497	0.011500
0.011369	0.011463	0.011463	0.011429	0.011396	0.011396	0.011363
0.011396	0.011329	0.011362	0.011396	0.011362	0.011295	0.011329
0.011329	0.011396	0.011295	0.011396	0.011430	0.011463	0.011362
0.011396	0.011362	0.011698	0.011698	0.011430	0.011396	0.011497
0.011262	0.011262	0.011295	0.011396	0.011262	0.011497	0.011497
0.011530	0.011530	0.011463	0.011564	0.011732	0.011565	0.011396
0.011396	0.011463	0.011497	0.011329	0.011396	0.011362	0.011329
0.011463	0.011463	0.011497	0.011463	0.011363	0.011564	0.011530
0.011365	0.011295	0.011396	0.011430	0.011396	0.011530	0.011564
0.011732	0.011436	0.011597	0.011564	0.011429	0.011664	0.011295
0.011397	0.011363	0.011329	0.011329	0.011396	0.011295	0.011396
0.011430	0.011463	0.011362	0.011396	0.011362	0.011698	0.011698
0.011430	0.011396	0.011530	0.011463	0.012103	0.011489	0.011412
0.011482	0.011442	0.011587	0.011476	0.011550	0.011474	0.011474
0.012056	0.011337	0.011471	0.011406	0.011680	0.011570	0.011673
0.012080	0.011512	0.011635	0.011698	0.011564	0.011564	0.011601
0.011262	0.011429	0.011564	0.011530	0.011533	0.011530	0.011498
0.011362	0.011295	0.011329	0.011362	0.011262	0.011329	0.011228
0.011362	0.011396	0.011295	0.011262	0.011362	0.011295	0.011329
0.011262	0.011262	0.011329	0.011362	0.011262	0.011463	0.011496
0.011396	0.011463	0.011396	0.011396	0.011329	0.011732	0.011362
0.011408	0.011329	0.011564	0.011569	0.011429	0.011362	0.011429
0.011429	0.011362	0.011329	0.011362	0.011463	0.011463	0.011396
0.011396	0.011530	0.011698	0.011429	0.011631	0.011631	0.011496
0.011329	0.011396	0.011429	0.011832	0.011396	0.011430	0.011396
0.011429	0.011362	0.011362	0.011665	0.011597	0.011631	0.011362
0.011463	0.011530	0.011396	0.011463	0.011429	0.011295	0.011631
0.011396	0.011362	0.011362	0.011530	0.011362	0.011463	0.011698
0.011396	0.011773	0.011765	0.011548	0.011342	0.011468	0.011318
0.011390	0.011761	0.011306	0.011507	0.011353	0.011213	0.011485
0.011570	0.011258	0.011450	0.011324	0.011330	0.011529	0.011425
0.011437	0.011336	0.011448	0.011377	0.011397	0.011530	0.011597
0.011597	0.011464	0.011567	0.011569	0.011704	0.011696	0.011396
0.011631	0.011497	0.011362	0.011463	0.011262	0.011496	0.011295

0.011362	0.011362	0.011329	0.011295	0.011329	0.011295	0.011396
0.011375	0.011375	0.011300	0.011403	0.011539	0.011504	0.011436
0.011503	0.011430	0.011245	0.011204	0.011365	0.011535	0.011535
0.011329	0.011399	0.011632	0.011601	0.011531	0.011565	0.011496
0.011429	0.011496	0.011496	0.011396	0.011967	0.011564	0.011631
0.011665	0.011737	0.011367	0.011441	0.011329	0.011530	0.011497
0.011531	0.011564	0.011564	0.011564	0.011611	0.011698	0.011338
0.011229	0.011233	0.011333	0.011298	0.011402	0.011247	0.011570
0.011369	0.011499	0.011251	0.011360	0.011363	0.011437	0.011329
0.011295	0.011295	0.011362	0.011329	0.011530	0.011497	0.011429
0.011530	0.011597	0.011329	0.011329	0.011362	0.011362	0.011364
0.011432	0.011362	0.011362	0.011397	0.011530	0.011597	0.011597
0.011464	0.011567	0.011569	0.011704	0.011696	0.011396	0.011564
0.011497	0.011497	0.011263	0.011513	0.011442	0.011469	0.011340
0.011508	0.011653	0.011526	0.011400	0.011295	0.011497	0.011362
0.011463	0.011396	0.011295	0.011397	0.011497	0.011499	0.011396
0.011295	0.011229	0.011362	0.011295	0.011228	0.011332	0.011497
0.011396	0.011329	0.011362	0.011396	0.011362	0.011295	0.011497
0.011362	0.011396	0.011396	0.011329	0.011396	0.011631	0.011295
0.011329	0.011732	0.011329	0.011362	0.011329	0.011698	0.011317
0.011735	0.011273	0.011361	0.011503	0.011589	0.011527	0.011269
0.011323	0.011496	0.011530	0.011637	0.011498	0.011635	0.011766
0.011666	0.011703	0.011664	0.011983	0.011698	0.012539	0.011734
0.012473	0.012241	0.012375	0.012646	0.011698	0.011396	0.011631
0.012137	0.012005	0.011664	0.011564	0.011632	0.011632	0.011601
0.011496	0.011501	0.011564	0.011531	0.011901	0.011967	0.011968
0.011868	0.011968	0.012072	0.011838	0.012138	0.012110	0.012205
0.012205	0.012239	0.012186	0.012206	0.012207	0.011429	0.011732
0.011800	0.012303	0.011806	0.012034	0.012508	0.011570	0.011464
0.011429	0.011429	0.011900	0.011924	0.012045	0.011765	0.011431
S045	0.011631	0.011631	0.011967	0.011732	0.011732	0.011799
0.011637	0.011569	0.011799	0.011228	0.011295	0.011262	0.011228
0.011262	0.011429	0.011228	0.011262	0.011329	0.011295	0.011262
0.011228	0.011262	0.011362	0.011228	0.011262	0.011262	0.011603
0.011496	0.011262	0.011161	0.011396	0.011329	0.011295	0.011329
0.011295	0.011396	0.011228	0.011295	0.011262	0.011295	0.011463
0.011228	0.011295	0.011262	0.000000	0.011362	0.011396	0.011329
0.011295	0.011396	0.011362	0.011631	0.011228	0.011564	0.011228
0.011262	0.011362	0.011362	0.011564	0.011664	0.011362	0.011463
0.011329	0.011396	0.011295	0.011530	0.011765	0.011295	0.011362
0.011262	0.011362	0.011329	0.011396	0.011329	0.011127	0.011127
0.011228	0.011262	0.011228	0.011329	0.011295	0.011329	0.011396
0.011329	0.011295	0.011362	0.011262	0.011396	0.011228	0.011295
0.011463	0.011396	0.011631	0.011463	0.011295	0.011530	0.011228
0.011468	0.011695	0.011513	0.011216	0.011210	0.011318	0.011415
0.012048	0.011416	0.011225	0.011530	0.011530	0.011329	0.011429
0.011362	0.011329	0.011396	0.011295	0.011396	0.011329	0.011329
0.011295	0.011396	0.011597	0.011463	0.011362	0.011329	0.011262
0.011228	0.011228	0.011295	0.011262	0.011463	0.011430	0.011362
0.011463	0.011530	0.011262	0.011262	0.011295	0.011295	0.011297
0.011364	0.011295	0.011295	0.011168	0.011241	0.011200	0.011296
0.011297	0.011232	0.011282	0.011267	0.011196	0.011231	0.011263
0.011528	0.011301	0.011336	0.011228	0.011233	0.011363	0.011329
0.011396	0.011330	0.011329	0.011429	0.011362	0.011731	0.011530
0.011564	0.011429	0.011295	0.011396	0.011195	0.011429	0.011228
0.011295	0.011295	0.011262	0.011228	0.011262	0.011228	0.011329
0.011295	0.011396	0.011329	0.011228	0.011330	0.011430	0.011432
0.011430	0.011329	0.011228	0.011161	0.011295	0.011228	0.011161
0.011264	0.011430	0.011228	0.011430	0.011195	0.011295	0.011262
0.011362	0.011262	0.011295	0.011262	0.011295	0.011161	0.011295

0.011329	0.011262	0.011329	0.011262	0.011295	0.011329	0.011564
0.011664	0.011597	0.011665	0.011364	0.011665	0.011295	0.011262
0.011263	0.011586	0.011335	0.011197	0.011671	0.011163	0.011296
0.011671	0.011570	0.011463	0.011664	0.011368	0.011530	0.011497
0.011429	0.011597	0.011451	0.011307	0.011354	0.011329	0.011398
0.011497	0.011397	0.011533	0.011601	0.011530	0.011597	0.011704
0.011432	0.011603	0.011534	0.011466	0.011733	0.011194	0.011397
0.011362	0.011295	0.011194	0.011194	0.011262	0.011262	0.011194
0.011262	0.011329	0.011228	0.011329	0.011262	0.011262	0.011329
0.011262	0.011698	0.011597	0.011631	0.011161	0.011262	0.011295
0.011295	0.011262	0.011329	0.011362	0.011228	0.011228	0.011228
0.011765	0.011396	0.011396	0.011194	0.011295	0.011463	0.011228
0.011228	0.011295	0.011262	0.011429	0.011228	0.011362	0.011496
0.011429	0.011262	0.011262	0.011195	0.011194	0.011362	0.011295
0.011195	0.011295	0.011161	0.011195	0.011329	0.011463	0.011228
0.011195	0.011329	0.011262	0.011161	0.011496	0.011161	0.011329
0.011402	0.011228	0.011530	0.011362	0.011396	0.011262	0.011295
0.011228	0.011564	0.011161	0.011429	0.011228	0.011228	0.011161
0.011396	0.011598	0.011295	0.011362	0.011194	0.011597	0.011228
0.011228	0.011228	0.011262	0.011362	0.011262	0.011362	0.011429
0.011429	0.011396	0.011094	0.011396	0.011362	0.011161	0.011194
0.011228	0.011161	0.011295	0.011228	0.011194	0.011362	0.011228
0.011262	0.011262	0.011396	0.011429	0.011329	0.011262	0.011262
0.011329	0.011262	0.011362	0.011228	0.011362	0.011463	0.011765
0.011262	0.011664	0.011267	0.017785	0.011191	0.011488	0.011618
0.011579	0.011623	0.011374	0.011234	0.011774	0.011647	0.011364
0.011487	0.011610	0.011177	0.011270	0.011255	0.011356	0.011275
0.011436	0.011433	0.011431	0.011259	0.011396	0.011429	0.011432
0.011301	0.011396	0.011396	0.011362	0.011329	0.011329	0.011295
0.011329	0.011262	0.011295	0.011329	0.011295	0.011228	0.011262
0.011262	0.011329	0.011228	0.011329	0.011362	0.011396	0.011295
0.011329	0.011295	0.011631	0.011631	0.011362	0.011396	0.011430
0.011195	0.011195	0.011228	0.011329	0.011195	0.011429	0.011429
0.011463	0.011463	0.011396	0.011496	0.011665	0.011498	0.011329
0.011329	0.011396	0.011429	0.011262	0.011329	0.011295	0.011329
0.011396	0.011396	0.011429	0.011396	0.011296	0.011497	0.011463
0.011331	0.011228	0.011329	0.011362	0.011329	0.011463	0.011496
0.011664	0.011368	0.011530	0.011497	0.011429	0.011597	0.011228
0.011397	0.011296	0.011262	0.011262	0.011329	0.011228	0.011329
0.011362	0.011396	0.011295	0.011329	0.011295	0.011631	0.011631
0.011362	0.011396	0.011463	0.011396	0.012035	0.011453	0.011377
0.011447	0.011407	0.011517	0.011406	0.011515	0.011439	0.011405
0.011987	0.011302	0.011437	0.011371	0.011645	0.011502	0.011604
0.012012	0.011475	0.011567	0.011631	0.011496	0.011497	0.011533
0.011194	0.011362	0.011496	0.011463	0.011466	0.011463	0.011431
0.011295	0.011228	0.011262	0.011295	0.011194	0.011262	0.011161
0.011295	0.011329	0.011228	0.011194	0.011295	0.011228	0.011262
0.011262	0.011194	0.011262	0.011295	0.011194	0.011396	0.011429
0.011329	0.011396	0.011329	0.011329	0.011262	0.011664	0.011295
0.011338	0.011262	0.011497	0.011501	0.011362	0.011295	0.011362
0.011362	0.011295	0.011262	0.011295	0.011396	0.011396	0.011329
0.011329	0.011463	0.011631	0.011362	0.011564	0.011564	0.011429
0.011262	0.011329	0.011362	0.011765	0.011329	0.011362	0.011329
0.011362	0.011295	0.011295	0.011597	0.011530	0.011564	0.011295
0.011396	0.011463	0.011329	0.011396	0.011362	0.011228	0.011564
0.011329	0.011295	0.011295	0.011463	0.011295	0.011396	0.011631
0.011329	0.011705	0.011698	0.011507	0.011286	0.011400	0.011215
0.011280	0.011722	0.011236	0.011417	0.011304	0.011315	0.011379
0.011417	0.011126	0.011339	0.011126	0.011222	0.011328	0.011385
0.011399	0.011296	0.011377	0.011307	0.011330	0.011463	0.011530

0.011530	0.011396	0.011500	0.011501	0.011636	0.011661	0.011329
0.011564	0.011429	0.011295	0.011396	0.011195	0.011429	0.011228
0.011295	0.011295	0.011262	0.011228	0.011262	0.011228	0.011329
0.011270	0.011270	0.011300	0.011334	0.011471	0.011436	0.011436
0.011435	0.011363	0.011134	0.011132	0.011297	0.011467	0.011467
0.011262	0.011331	0.011565	0.011533	0.011464	0.011498	0.011429
0.011362	0.011429	0.011429	0.011329	0.011900	0.011496	0.011564
0.011597	0.011630	0.011299	0.011372	0.011262	0.011463	0.011429
0.011464	0.011496	0.011496	0.011496	0.011610	0.011631	0.011268
0.011162	0.011164	0.011264	0.011230	0.011333	0.011172	0.011502
0.011300	0.011455	0.011203	0.011322	0.011296	0.011368	0.011262
0.011228	0.011228	0.011295	0.011262	0.011463	0.011430	0.011362
0.011463	0.011530	0.011262	0.011262	0.011295	0.011295	0.011297
0.011364	0.011295	0.011295	0.011330	0.011463	0.011530	0.011530
0.011396	0.011500	0.011501	0.011636	0.011661	0.011329	0.011496
0.011429	0.011429	0.011196	0.011443	0.011373	0.011401	0.011270
0.011439	0.011607	0.011357	0.011332	0.011228	0.011430	0.011295
0.011396	0.011329	0.011228	0.011330	0.011430	0.011432	0.011329
0.011228	0.011161	0.011295	0.011228	0.011161	0.011264	0.011430
0.011329	0.011262	0.011295	0.011329	0.011295	0.011228	0.011430
0.011295	0.011329	0.011329	0.011262	0.011329	0.011564	0.011228
0.011262	0.011664	0.011262	0.011295	0.011262	0.011631	0.011232
0.011678	0.011230	0.011211	0.011463	0.011549	0.011455	0.011186
0.011165	0.011429	0.011530	0.011637	0.011498	0.011635	0.011766
0.011666	0.011703	0.011664	0.011914	0.011631	0.012505	0.011666
0.012405	0.012173	0.012307	0.012578	0.011631	0.011329	0.011631
0.012070	0.012004	0.011597	0.011497	0.011565	0.011565	0.011534
0.011429	0.011433	0.011496	0.011464	0.011833	0.011900	0.011901
0.011800	0.011901	0.012004	0.011770	0.012070	0.012076	0.012137
0.012138	0.012171	0.012118	0.012138	0.012139	0.011362	0.011665
0.011733	0.012236	0.011738	0.011967	0.012440	0.011502	0.011396
0.011362	0.011362	0.011833	0.011883	0.011975	0.011698	0.011363
S046 0.011732	0.011732	0.012001	0.011765	0.011765	0.011832	
0.011671	0.011603	0.011832	0.011329	0.011396	0.011363	0.011329
0.011295	0.011530	0.011262	0.011362	0.011429	0.011396	0.011362
0.011329	0.011362	0.011396	0.011329	0.011363	0.011362	0.011637
0.011530	0.011362	0.011262	0.011429	0.011429	0.011396	0.011429
0.011396	0.011496	0.011396	0.011396	0.011429	0.011396	0.011564
0.011262	0.011396	0.011429	0.011362	0.000000	0.011496	0.011429
0.011396	0.011564	0.011463	0.011664	0.011396	0.011664	0.011396
0.011362	0.000000	0.011530	0.011664	0.011698	0.011396	0.011496
0.011429	0.011496	0.011396	0.011631	0.011799	0.011396	0.011463
0.011362	0.011463	0.011429	0.011496	0.011429	0.011429	0.011429
0.011329	0.011362	0.011329	0.011362	0.011396	0.011430	0.011496
0.011429	0.011396	0.011463	0.011362	0.011429	0.011329	0.011396
0.011564	0.011496	0.011731	0.011564	0.011396	0.011631	0.011396
0.011579	0.011733	0.011626	0.011334	0.011321	0.011428	0.011454
0.012085	0.011518	0.011348	0.011631	0.011631	0.011362	0.011463
0.011463	0.011429	0.011496	0.011396	0.011496	0.011429	0.011429
0.011396	0.011429	0.011698	0.011563	0.011463	0.011429	0.011362
0.011329	0.011262	0.011396	0.011362	0.011497	0.011463	0.011396
0.011497	0.011564	0.011362	0.011362	0.011396	0.011396	0.011398
0.011466	0.011396	0.011396	0.011276	0.011349	0.011306	0.011396
0.011399	0.011335	0.011392	0.011371	0.011298	0.011334	0.011364
0.011564	0.011405	0.011439	0.011329	0.011337	0.011464	0.011363
0.011430	0.011431	0.011430	0.011530	0.011463	0.011832	0.011564
0.011597	0.011463	0.011396	0.011430	0.011295	0.011463	0.011329
0.011396	0.011396	0.011362	0.011329	0.011362	0.011329	0.011429
0.011396	0.011497	0.011430	0.011329	0.011431	0.011530	0.011533
0.011530	0.011430	0.011329	0.011263	0.011396	0.011329	0.011262

0.011366	0.011530	0.011329	0.011530	0.011295	0.011396	0.011362
0.011463	0.011362	0.011396	0.011362	0.011396	0.011262	0.011396
0.011430	0.011362	0.011430	0.011362	0.011396	0.011429	0.011665
0.011698	0.011631	0.011698	0.011532	0.011698	0.011396	0.011362
0.011330	0.011626	0.011426	0.011300	0.011705	0.011265	0.011414
0.011705	0.011604	0.011564	0.011765	0.011470	0.011631	0.011597
0.011530	0.011698	0.011557	0.011390	0.011479	0.011429	0.011500
0.011597	0.011499	0.011635	0.011703	0.011631	0.011698	0.011805
0.011534	0.011705	0.011636	0.011567	0.011834	0.011295	0.011498
0.011463	0.011396	0.011295	0.011295	0.011362	0.011362	0.011429
0.011362	0.011429	0.011329	0.011362	0.011362	0.011362	0.011429
0.011362	0.011664	0.011631	0.011664	0.011262	0.011362	0.011396
0.011396	0.011362	0.011429	0.011396	0.011329	0.011329	0.011329
0.011799	0.011429	0.011496	0.011295	0.011396	0.011564	0.011329
0.011329	0.011396	0.011362	0.011530	0.011329	0.011463	0.011597
0.011530	0.011362	0.011362	0.011295	0.011295	0.011463	0.011396
0.011295	0.011396	0.011262	0.011295	0.011429	0.011497	0.011329
0.011295	0.011429	0.011363	0.011262	0.011530	0.011262	0.011429
0.011505	0.011329	0.011564	0.011463	0.011429	0.011362	0.011463
0.011329	0.011664	0.011262	0.011463	0.011262	0.011262	0.011262
0.011496	0.011631	0.011396	0.011463	0.011295	0.011631	0.011262
0.011329	0.011329	0.011362	0.011463	0.011362	0.011463	0.011530
0.011530	0.011496	0.011396	0.011497	0.011463	0.011329	0.011295
0.011329	0.011329	0.011396	0.011262	0.011295	0.011463	0.011329
0.011295	0.011362	0.011496	0.011463	0.011429	0.011362	0.011362
0.011429	0.011362	0.011463	0.011329	0.011463	0.011564	0.011799
0.011362	0.011698	0.011371	0.017823	0.011387	0.011427	0.011653
0.011614	0.011658	0.011427	0.011408	0.011719	0.011682	0.011425
0.011534	0.011548	0.011294	0.011353	0.011294	0.011307	0.011361
0.011529	0.011470	0.011468	0.011259	0.011496	0.011530	0.011534
0.011404	0.011497	0.011497	0.011396	0.011429	0.011430	0.011396
0.011429	0.011362	0.011396	0.011430	0.011396	0.011329	0.011362
0.011362	0.011430	0.011329	0.011430	0.011463	0.011497	0.011396
0.011362	0.011329	0.011665	0.011665	0.011463	0.011497	0.011530
0.011295	0.011295	0.011329	0.011430	0.011295	0.011530	0.011530
0.011564	0.011564	0.011496	0.011597	0.011698	0.011599	0.011429
0.011430	0.011497	0.011530	0.011362	0.011429	0.011396	0.011429
0.011497	0.011497	0.011530	0.011430	0.011397	0.011597	0.011564
0.011433	0.011329	0.011429	0.011530	0.011430	0.011497	0.011597
0.011765	0.011470	0.011631	0.011597	0.011530	0.011698	0.011295
0.011498	0.011396	0.011362	0.011362	0.011430	0.011329	0.011430
0.011463	0.011497	0.011396	0.011362	0.011329	0.011665	0.011665
0.011463	0.011497	0.011563	0.011496	0.012069	0.011560	0.011482
0.011552	0.011511	0.011622	0.011510	0.011620	0.011543	0.011508
0.012021	0.011406	0.011540	0.011475	0.011749	0.011604	0.011707
0.012046	0.011512	0.011669	0.011732	0.011597	0.011530	0.011635
0.011295	0.011463	0.011597	0.011563	0.011567	0.011496	0.011464
0.011396	0.011329	0.011362	0.011396	0.011295	0.011362	0.011262
0.011329	0.011362	0.011329	0.011295	0.011396	0.011329	0.011362
0.011362	0.011295	0.011362	0.011396	0.011295	0.011496	0.011530
0.011429	0.011429	0.011429	0.011429	0.011362	0.011698	0.011396
0.011373	0.011362	0.011530	0.011535	0.011463	0.011463	0.011463
0.011463	0.011396	0.011362	0.011396	0.011496	0.011496	0.011429
0.011429	0.011564	0.011664	0.011463	0.011664	0.011664	0.011530
0.011362	0.011429	0.011463	0.011799	0.011430	0.011463	0.011429
0.011463	0.011329	0.011329	0.011631	0.011631	0.011597	0.011396
0.011496	0.011564	0.011429	0.011496	0.011463	0.011329	0.011597
0.011429	0.011396	0.011329	0.011564	0.011396	0.011496	0.011732
0.011429	0.011739	0.011731	0.011548	0.011286	0.011434	0.011370
0.011225	0.011761	0.011341	0.011507	0.011353	0.011518	0.011432

0.011519	0.011323	0.011505	0.011324	0.011437	0.011529	0.011506
0.011512	0.011414	0.011412	0.011412	0.011431	0.011497	0.011564
0.011564	0.011430	0.011533	0.011535	0.011670	0.011696	0.011429
0.011597	0.011463	0.011396	0.011430	0.011295	0.011463	0.011329
0.011396	0.011396	0.011362	0.011329	0.011362	0.011329	0.011429
0.011409	0.011410	0.011403	0.011437	0.011573	0.011538	0.011538
0.011537	0.011464	0.011282	0.011276	0.011399	0.011568	0.011569
0.011363	0.011433	0.011666	0.011635	0.011565	0.011599	0.011530
0.011463	0.011530	0.011530	0.011429	0.011933	0.011597	0.011665
0.011698	0.011701	0.011401	0.011441	0.011362	0.011564	0.011530
0.011565	0.011597	0.011597	0.011597	0.011768	0.011731	0.011372
0.011263	0.011268	0.011367	0.011332	0.011368	0.011284	0.011536
0.011403	0.011499	0.011298	0.011435	0.011396	0.011471	0.011362
0.011329	0.011262	0.011396	0.011362	0.011497	0.011463	0.011396
0.011497	0.011564	0.011362	0.011362	0.011396	0.011396	0.011398
0.011466	0.011396	0.011396	0.011431	0.011497	0.011564	0.011564
0.011430	0.011533	0.011535	0.011670	0.011696	0.011429	0.011597
0.011530	0.011530	0.011364	0.011547	0.011477	0.011503	0.011340
0.011542	0.011698	0.011526	0.011434	0.011329	0.011530	0.011396
0.011497	0.011430	0.011329	0.011431	0.011530	0.011533	0.011430
0.011329	0.011263	0.011396	0.011329	0.011262	0.011366	0.011530
0.011429	0.011362	0.011396	0.011430	0.011396	0.011329	0.011463
0.011396	0.011429	0.011429	0.011362	0.011429	0.011664	0.011262
0.011362	0.011698	0.011362	0.011396	0.011362	0.011664	0.011317
0.011678	0.011358	0.011311	0.011503	0.011589	0.011491	0.011269
0.011270	0.011463	0.011564	0.011671	0.011531	0.011669	0.011800
0.011699	0.011737	0.011698	0.011949	0.011664	0.012539	0.011700
0.012439	0.012207	0.012341	0.012612	0.011664	0.011362	0.011665
0.012103	0.012038	0.011631	0.011530	0.011598	0.011598	0.011567
0.011463	0.011467	0.011530	0.011497	0.011867	0.011933	0.011935
0.011834	0.011934	0.012038	0.011804	0.012036	0.012110	0.012171
0.012172	0.012205	0.012152	0.012172	0.012173	0.011463	0.011699
0.011766	0.012270	0.011772	0.012001	0.012474	0.011536	0.011430
0.011396	0.011396	0.011867	0.011924	0.012010	0.011731	0.011397
S047	0.011765	0.011765	0.012101	0.011866	0.011866	0.011933
0.011773	0.011705	0.011933	0.011362	0.011429	0.011397	0.011362
0.011396	0.011564	0.011362	0.011329	0.011463	0.011429	0.011396
0.011362	0.011396	0.011496	0.011363	0.011396	0.011396	0.011739
0.011631	0.011396	0.011295	0.011530	0.011463	0.011429	0.011463
0.011429	0.011530	0.011429	0.011429	0.011396	0.011429	0.011597
0.011362	0.011429	0.011396	0.011396	0.011496	0.000000	0.011463
0.011429	0.011530	0.011497	0.011765	0.011362	0.011698	0.011362
0.011396	0.011497	0.011564	0.011698	0.011799	0.011496	0.011597
0.011463	0.011530	0.011429	0.011664	0.011900	0.011429	0.011496
0.011396	0.011496	0.011463	0.011530	0.011463	0.011463	0.011463
0.011362	0.011396	0.011362	0.011463	0.011429	0.011464	0.011530
0.011463	0.011429	0.011496	0.011396	0.011530	0.011362	0.011429
0.011597	0.011530	0.011765	0.011597	0.011429	0.011664	0.011429
0.011616	0.011845	0.011664	0.011373	0.011359	0.011428	0.011572
0.012195	0.011721	0.011390	0.011530	0.011597	0.011463	0.011564
0.011496	0.011463	0.011530	0.011429	0.011530	0.011463	0.011463
0.011429	0.011530	0.011732	0.011597	0.011496	0.011463	0.011396
0.011362	0.011362	0.011430	0.011396	0.011597	0.011564	0.011497
0.011597	0.011665	0.011396	0.011396	0.011430	0.011429	0.011432
0.011500	0.011429	0.011430	0.011312	0.011386	0.011341	0.011430
0.011433	0.011369	0.011429	0.011405	0.011332	0.011368	0.011398
0.011672	0.011439	0.011473	0.011363	0.011371	0.011497	0.011464
0.011531	0.011465	0.011464	0.011563	0.011496	0.011799	0.011665
0.011698	0.011564	0.011429	0.011530	0.011329	0.011564	0.011362
0.011429	0.011429	0.011396	0.011362	0.011396	0.011362	0.011463

0.011430	0.011530	0.011463	0.011362	0.011464	0.011564	0.011567
0.011564	0.011463	0.011362	0.011296	0.011429	0.011362	0.011295
0.011400	0.011564	0.011362	0.011564	0.011329	0.011430	0.011396
0.011497	0.011396	0.011430	0.011396	0.011430	0.011295	0.011430
0.011463	0.011396	0.011463	0.011396	0.011429	0.011463	0.011698
0.011799	0.011732	0.011799	0.011566	0.011799	0.011429	0.011396
0.011432	0.011747	0.011563	0.011335	0.011807	0.011299	0.011414
0.011807	0.011706	0.011530	0.011799	0.011504	0.011597	0.011631
0.011496	0.011664	0.011593	0.011431	0.011437	0.011463	0.011534
0.011631	0.011532	0.011635	0.011737	0.011664	0.011732	0.011839
0.011568	0.011739	0.011602	0.011601	0.011834	0.011329	0.011532
0.011496	0.011429	0.011329	0.011329	0.011396	0.011396	0.011463
0.011396	0.011463	0.011362	0.011463	0.011396	0.011396	0.011396
0.011396	0.011832	0.011732	0.011765	0.011295	0.011396	0.011429
0.011429	0.011396	0.011463	0.011496	0.011362	0.011362	0.011362
0.011899	0.011530	0.011530	0.011329	0.011429	0.011530	0.011362
0.011362	0.011429	0.011396	0.011564	0.011362	0.011496	0.011631
0.011564	0.011396	0.011396	0.011329	0.011329	0.011497	0.011429
0.011329	0.011429	0.011295	0.011329	0.011463	0.011598	0.011362
0.011329	0.011463	0.011396	0.011295	0.011631	0.011295	0.011463
0.011539	0.011362	0.011664	0.011497	0.011530	0.011396	0.011429
0.011362	0.011698	0.011295	0.011564	0.011362	0.011362	0.011295
0.011530	0.011732	0.011429	0.011496	0.011329	0.011732	0.011362
0.011362	0.011362	0.011396	0.011496	0.011396	0.011496	0.011564
0.011564	0.011530	0.011429	0.011530	0.011496	0.011362	0.011329
0.011362	0.011362	0.011429	0.011362	0.011329	0.011496	0.011362
0.011396	0.011396	0.011530	0.011564	0.011463	0.011396	0.011396
0.011463	0.011396	0.011496	0.011362	0.011496	0.011530	0.011900
0.011396	0.011799	0.011405	0.017899	0.011322	0.011488	0.011758
0.011718	0.011764	0.011479	0.011466	0.011774	0.011785	0.011425
0.011629	0.011610	0.011294	0.011395	0.011333	0.011356	0.011404
0.011717	0.011582	0.011579	0.011391	0.011530	0.011564	0.011567
0.011438	0.011530	0.011463	0.011497	0.011463	0.011463	0.011430
0.011463	0.011396	0.011362	0.011463	0.011430	0.011362	0.011396
0.011396	0.011463	0.011362	0.011463	0.011497	0.011530	0.011429
0.011463	0.011430	0.011765	0.011765	0.011497	0.011464	0.011564
0.011329	0.011329	0.011362	0.011463	0.011329	0.011564	0.011564
0.011530	0.011530	0.011530	0.011564	0.011799	0.011565	0.011463
0.011396	0.011530	0.011564	0.011396	0.011463	0.011429	0.011396
0.011463	0.011463	0.011497	0.011530	0.011363	0.011631	0.011597
0.011467	0.011362	0.011463	0.011564	0.011463	0.011598	0.011631
0.011799	0.011504	0.011597	0.011631	0.011496	0.011664	0.011363
0.000000	0.011430	0.011396	0.011396	0.011463	0.011362	0.011463
0.011497	0.011530	0.011429	0.011463	0.011430	0.011765	0.011765
0.011497	0.011464	0.011597	0.011530	0.012170	0.011560	0.011517
0.011587	0.011511	0.011657	0.011545	0.011620	0.011577	0.011508
0.012125	0.011441	0.011574	0.011510	0.011783	0.011638	0.011741
0.012148	0.011626	0.011703	0.011765	0.011631	0.011631	0.011668
0.011329	0.011497	0.011631	0.011597	0.011601	0.011597	0.011565
0.011429	0.011362	0.011396	0.011429	0.011329	0.011396	0.011295
0.011429	0.011463	0.011362	0.011329	0.011429	0.011362	0.011396
0.011329	0.011329	0.011396	0.011429	0.011329	0.011530	0.011564
0.011463	0.011530	0.011463	0.011463	0.011396	0.011799	0.011429
0.011478	0.011396	0.011631	0.011637	0.011497	0.011496	0.011496
0.011497	0.011429	0.011396	0.011429	0.011530	0.011530	0.011463
0.011463	0.011597	0.011765	0.011496	0.011698	0.011698	0.011564
0.011396	0.011463	0.011497	0.011900	0.011463	0.011497	0.011463
0.011429	0.011429	0.011429	0.011732	0.011665	0.011698	0.011429
0.011530	0.011597	0.011463	0.011530	0.011496	0.011362	0.011698
0.011463	0.011429	0.011429	0.011597	0.011429	0.011530	0.011765

0.011463	0.011841	0.011832	0.011670	0.011399	0.011536	0.011421
0.011335	0.011839	0.011376	0.011596	0.011451	0.011467	0.011538
0.011672	0.011258	0.011505	0.011258	0.011437	0.011462	0.011506
0.011550	0.011414	0.011518	0.011448	0.011465	0.011597	0.011665
0.011665	0.011531	0.011635	0.011636	0.011772	0.011767	0.011463
0.011698	0.011564	0.011429	0.011530	0.011329	0.011564	0.011362
0.011429	0.011429	0.011396	0.011362	0.011396	0.011362	0.011463
0.011409	0.011445	0.011368	0.011471	0.011608	0.011573	0.011504
0.011571	0.011497	0.011319	0.011276	0.011433	0.011602	0.011603
0.011396	0.011467	0.011700	0.011669	0.011531	0.011632	0.011564
0.011496	0.011564	0.011564	0.011463	0.012034	0.011564	0.011631
0.011732	0.011772	0.011435	0.011511	0.011396	0.011597	0.011564
0.011598	0.011631	0.011564	0.011564	0.011729	0.011765	0.011407
0.011297	0.011303	0.011401	0.011366	0.011470	0.011321	0.011638
0.011437	0.011543	0.011298	0.011472	0.011430	0.011505	0.011396
0.011362	0.011362	0.011430	0.011396	0.011597	0.011564	0.011497
0.011597	0.011665	0.011396	0.011396	0.011430	0.011429	0.011432
0.011500	0.011429	0.011430	0.011465	0.011597	0.011665	0.011665
0.011531	0.011635	0.011636	0.011772	0.011767	0.011463	0.011631
0.011564	0.011564	0.011398	0.011582	0.011512	0.011537	0.011411
0.011577	0.011789	0.011611	0.011468	0.011362	0.011564	0.011430
0.011530	0.011463	0.011362	0.011464	0.011564	0.011567	0.011463
0.011362	0.011296	0.011429	0.011362	0.011295	0.011400	0.011564
0.011463	0.011396	0.011362	0.011463	0.011430	0.011362	0.011564
0.011362	0.011463	0.011463	0.011396	0.011463	0.011698	0.011362
0.011396	0.011799	0.011396	0.011429	0.011396	0.011765	0.011403
0.011791	0.011400	0.011511	0.011624	0.011712	0.011599	0.011353
0.011427	0.011564	0.011597	0.011705	0.011565	0.011703	0.011834
0.011733	0.011770	0.011732	0.012051	0.011765	0.012640	0.011801
0.012540	0.012308	0.012442	0.012714	0.011765	0.011463	0.011699
0.012204	0.012072	0.011732	0.011631	0.011699	0.011699	0.011669
0.011564	0.011569	0.011631	0.011598	0.011968	0.012034	0.012036
0.011935	0.012035	0.012139	0.011906	0.012205	0.012212	0.012272
0.012273	0.012307	0.012255	0.012273	0.012274	0.011496	0.011800
0.011867	0.012371	0.011874	0.012102	0.012575	0.011637	0.011531
0.011463	0.011496	0.011968	0.012048	0.012116	0.011832	0.011498
S048	0.011698	0.011698	0.012034	0.011799	0.011799	0.011866
0.011705	0.011637	0.011866	0.011295	0.011362	0.011329	0.011295
0.011329	0.011496	0.011295	0.011329	0.011396	0.011362	0.011329
0.011295	0.011329	0.011429	0.011295	0.011329	0.011329	0.011671
0.011564	0.011329	0.011228	0.011463	0.011396	0.011362	0.011396
0.011161	0.011463	0.011362	0.011362	0.011396	0.011362	0.011530
0.011295	0.011362	0.011396	0.011329	0.011429	0.011463	0.000000
0.011362	0.011530	0.011430	0.011698	0.011362	0.011631	0.011362
0.011329	0.011429	0.011497	0.011631	0.011732	0.011429	0.011530
0.011396	0.011463	0.011362	0.011597	0.011832	0.011362	0.011362
0.011329	0.011429	0.011396	0.011463	0.011396	0.011396	0.011396
0.011295	0.011329	0.011295	0.011396	0.011362	0.011397	0.011463
0.011396	0.011362	0.011429	0.011329	0.011463	0.011295	0.011362
0.011530	0.011463	0.011698	0.011530	0.011362	0.011597	0.011362
0.011542	0.011770	0.011588	0.011295	0.011284	0.011391	0.011493
0.012121	0.011620	0.011307	0.011597	0.011597	0.011396	0.011497
0.011429	0.011396	0.011463	0.011362	0.011463	0.011396	0.011396
0.011362	0.011463	0.011664	0.011530	0.011429	0.011396	0.011329
0.011295	0.011295	0.011362	0.011329	0.011530	0.011497	0.011429
0.011530	0.011597	0.011329	0.011329	0.011362	0.011362	0.011364
0.011432	0.011362	0.011362	0.011204	0.011277	0.011236	0.011363
0.011365	0.011301	0.011355	0.011336	0.011230	0.011266	0.011330
0.011564	0.011336	0.011405	0.011296	0.011302	0.011430	0.011396
0.011464	0.011397	0.011396	0.011496	0.011429	0.011799	0.011598

0.011631	0.011497	0.011362	0.011463	0.011262	0.011496	0.011295
0.011362	0.011362	0.011329	0.011295	0.011329	0.011295	0.011396
0.011362	0.011463	0.011396	0.011295	0.011397	0.011497	0.011499
0.011497	0.011396	0.011295	0.011229	0.011362	0.011295	0.011228
0.011332	0.011497	0.011295	0.011497	0.011262	0.011362	0.011329
0.011430	0.011329	0.011362	0.011329	0.011362	0.011228	0.011362
0.011396	0.011329	0.011396	0.011329	0.011362	0.011396	0.011631
0.011732	0.011664	0.011732	0.011498	0.011732	0.011362	0.011329
0.011364	0.011667	0.011472	0.011266	0.011739	0.011231	0.011375
0.011739	0.011638	0.011530	0.011732	0.011436	0.011597	0.011564
0.011496	0.011664	0.011486	0.011307	0.011354	0.011396	0.011466
0.011564	0.011465	0.011601	0.011669	0.011597	0.011665	0.011772
0.011500	0.011671	0.011568	0.011500	0.011800	0.011262	0.011465
0.011429	0.011362	0.011262	0.011262	0.011329	0.011329	0.011396
0.011329	0.011396	0.011295	0.011396	0.011329	0.011329	0.011396
0.011329	0.011765	0.011664	0.011698	0.011228	0.011329	0.011362
0.011362	0.011329	0.011396	0.011429	0.011295	0.011295	0.011295
0.011832	0.011463	0.011463	0.011262	0.011362	0.011530	0.011295
0.011295	0.011362	0.011329	0.011497	0.011295	0.011429	0.011564
0.011496	0.011329	0.011329	0.011262	0.011262	0.011429	0.011362
0.011262	0.011362	0.011228	0.011262	0.011396	0.011497	0.011295
0.011262	0.011396	0.011329	0.011228	0.011564	0.011228	0.011396
0.011471	0.011295	0.011597	0.011430	0.011463	0.011329	0.011429
0.011295	0.011631	0.011228	0.011497	0.011295	0.011295	0.011228
0.011463	0.011665	0.011362	0.011429	0.011262	0.011664	0.011295
0.011295	0.011295	0.011329	0.011429	0.011329	0.011429	0.011496
0.011497	0.011463	0.011362	0.011463	0.011429	0.011295	0.011262
0.011295	0.011295	0.011362	0.011295	0.011262	0.011429	0.011295
0.011329	0.011329	0.011463	0.011496	0.011396	0.011329	0.011329
0.011396	0.011329	0.011429	0.011295	0.011429	0.011530	0.011832
0.011329	0.011732	0.011336	0.017823	0.011388	0.011427	0.011688
0.011648	0.011693	0.011427	0.011408	0.011829	0.011716	0.011364
0.011629	0.011549	0.011255	0.011353	0.011333	0.011406	0.011318
0.011623	0.011507	0.011505	0.011391	0.011463	0.011497	0.011500
0.011335	0.011463	0.011463	0.011429	0.011396	0.011396	0.011363
0.011396	0.011329	0.011362	0.011396	0.011362	0.011295	0.011329
0.011329	0.011396	0.011295	0.011396	0.011430	0.011463	0.011362
0.011396	0.011362	0.011698	0.011698	0.011430	0.011464	0.011497
0.011262	0.011262	0.011295	0.011396	0.011262	0.011497	0.011497
0.011530	0.011530	0.011463	0.011564	0.011732	0.011565	0.011396
0.011396	0.011463	0.011497	0.011329	0.011396	0.011362	0.011396
0.011463	0.011463	0.011497	0.011463	0.011363	0.011564	0.011530
0.011399	0.011295	0.011396	0.011497	0.011396	0.011530	0.011564
0.011732	0.011436	0.011597	0.011564	0.011496	0.011664	0.011295
0.011464	0.011363	0.011329	0.011329	0.011396	0.011295	0.011396
0.011430	0.011463	0.011362	0.011396	0.011362	0.011698	0.011698
0.011430	0.011464	0.011530	0.011463	0.012103	0.011489	0.011447
0.011482	0.011442	0.011552	0.011476	0.011550	0.011474	0.011439
0.012056	0.011337	0.011471	0.011406	0.011680	0.011536	0.011673
0.012080	0.011512	0.011635	0.011698	0.011564	0.011564	0.011601
0.011262	0.011429	0.011564	0.011530	0.011533	0.011530	0.011498
0.011362	0.011295	0.011329	0.011362	0.011262	0.011329	0.011228
0.011362	0.011396	0.011295	0.011262	0.011362	0.011295	0.011329
0.011329	0.011262	0.011329	0.011362	0.011262	0.011463	0.011496
0.011396	0.011463	0.011396	0.011329	0.011329	0.011732	0.011362
0.011373	0.011329	0.011564	0.011569	0.011429	0.011429	0.011429
0.011429	0.011362	0.011329	0.011362	0.011463	0.011463	0.011396
0.011396	0.011530	0.011698	0.011429	0.011631	0.011631	0.011496
0.011329	0.011396	0.011429	0.011832	0.011396	0.011430	0.011396
0.011429	0.011362	0.011362	0.011665	0.011597	0.011631	0.011362

0.011463	0.011530	0.011396	0.011463	0.011429	0.011295	0.011631
0.011396	0.011362	0.011362	0.011530	0.011362	0.011463	0.011698
0.011396	0.011773	0.011765	0.011589	0.011286	0.011468	0.011370
0.011225	0.011800	0.011306	0.011551	0.011402	0.011518	0.011538
0.011621	0.011324	0.011506	0.011324	0.011384	0.011529	0.011466
0.011475	0.011375	0.011448	0.011377	0.011397	0.011530	0.011597
0.011597	0.011464	0.011567	0.011569	0.011704	0.011732	0.011396
0.011631	0.011497	0.011362	0.011463	0.011262	0.011496	0.011295
0.011362	0.011362	0.011329	0.011295	0.011329	0.011295	0.011396
0.011340	0.011340	0.011368	0.011368	0.011505	0.011504	0.011504
0.011503	0.011430	0.011208	0.011204	0.011365	0.011535	0.011535
0.011329	0.011399	0.011632	0.011601	0.011531	0.011565	0.011496
0.011429	0.011496	0.011496	0.011396	0.011967	0.011564	0.011631
0.011665	0.011737	0.011367	0.011441	0.011329	0.011530	0.011497
0.011531	0.011564	0.011564	0.011564	0.011728	0.011698	0.011338
0.011229	0.011233	0.011333	0.011298	0.011368	0.011210	0.011570
0.011369	0.011455	0.011156	0.011360	0.011363	0.011402	0.011329
0.011295	0.011295	0.011362	0.011329	0.011530	0.011497	0.011429
0.011530	0.011597	0.011329	0.011329	0.011362	0.011362	0.011364
0.011432	0.011362	0.011362	0.011397	0.011530	0.011597	0.011597
0.011464	0.011567	0.011569	0.011704	0.011732	0.011396	0.011564
0.011497	0.011497	0.011331	0.011513	0.011442	0.011469	0.011340
0.011508	0.011744	0.011484	0.011400	0.011295	0.011497	0.011362
0.011463	0.011396	0.011295	0.011397	0.011497	0.011499	0.011396
0.011295	0.011229	0.011362	0.011295	0.011228	0.011332	0.011497
0.011396	0.011329	0.011362	0.011396	0.011362	0.011295	0.011497
0.011362	0.011396	0.011396	0.011329	0.011396	0.011631	0.011295
0.011329	0.011732	0.011329	0.011362	0.011329	0.011698	0.011317
0.011735	0.011273	0.011411	0.011503	0.011630	0.011527	0.011269
0.011375	0.011496	0.011597	0.011705	0.011565	0.011703	0.011834
0.011733	0.011770	0.011732	0.011983	0.011698	0.012573	0.011734
0.012473	0.012241	0.012375	0.012646	0.011698	0.011396	0.011699
0.012137	0.012072	0.011664	0.011564	0.011632	0.011632	0.011567
0.011496	0.011501	0.011564	0.011531	0.011901	0.011967	0.011968
0.011868	0.011968	0.012072	0.011838	0.012138	0.012110	0.012205
0.012205	0.012239	0.012186	0.012206	0.012207	0.011429	0.011732
0.011800	0.012303	0.011806	0.012034	0.012508	0.011570	0.011464
0.011429	0.011429	0.011900	0.011924	0.012045	0.011765	0.011431
S049	0.011664	0.011664	0.012001	0.011765	0.011765	0.011832
0.011671	0.011603	0.011832	0.011262	0.011329	0.011296	0.011262
0.011295	0.011463	0.011262	0.011295	0.011362	0.011329	0.011295
0.011262	0.011295	0.011396	0.011262	0.011295	0.011295	0.011637
0.011530	0.011295	0.011194	0.011429	0.011362	0.011329	0.011362
0.011329	0.011429	0.011329	0.011329	0.011362	0.011329	0.011497
0.011262	0.011329	0.011362	0.011295	0.011396	0.011429	0.011362
0.000000	0.011497	0.011396	0.011664	0.011329	0.011597	0.011329
0.011295	0.011396	0.011463	0.011597	0.011698	0.011396	0.011496
0.011362	0.011429	0.011329	0.011564	0.011799	0.011329	0.011396
0.011295	0.011396	0.011362	0.011429	0.011362	0.011362	0.011362
0.011262	0.011295	0.011262	0.011362	0.011329	0.011363	0.011429
0.011362	0.011329	0.011396	0.011295	0.011429	0.011262	0.011329
0.011496	0.011429	0.011664	0.011497	0.011329	0.011564	0.011329
0.011505	0.011733	0.011551	0.011256	0.011247	0.011355	0.011454
0.012085	0.011569	0.011266	0.011564	0.011564	0.011362	0.011463
0.011396	0.011362	0.011429	0.011329	0.011429	0.011362	0.011362
0.011329	0.011429	0.011631	0.011496	0.011396	0.011362	0.011295
0.011262	0.011262	0.011329	0.011295	0.011497	0.011463	0.011396
0.011497	0.011564	0.011295	0.011295	0.011329	0.011329	0.011330
0.011398	0.011329	0.011329	0.011204	0.011277	0.011236	0.011329
0.011331	0.011266	0.011318	0.011302	0.011230	0.011266	0.011297

0.011564	0.011336	0.011370	0.011262	0.011268	0.011397	0.011363
0.011430	0.011364	0.011363	0.011463	0.011396	0.011765	0.011564
0.011597	0.011463	0.011329	0.011430	0.011228	0.011463	0.011262
0.011329	0.011329	0.011295	0.011262	0.011295	0.011262	0.011362
0.011329	0.011430	0.011362	0.011262	0.011363	0.011463	0.011466
0.011463	0.011362	0.011262	0.011195	0.011329	0.011262	0.011195
0.011298	0.011463	0.011262	0.011463	0.011228	0.011329	0.011295
0.011396	0.011295	0.011329	0.011295	0.011329	0.011195	0.011329
0.011362	0.011295	0.011362	0.011295	0.011329	0.011362	0.011597
0.011698	0.011631	0.011698	0.011465	0.011698	0.011329	0.011295
0.011330	0.011586	0.011472	0.011232	0.011637	0.011163	0.011296
0.011705	0.011604	0.011496	0.011698	0.011402	0.011564	0.011530
0.011463	0.011631	0.011486	0.011307	0.011354	0.011362	0.011432
0.011530	0.011431	0.011567	0.011635	0.011564	0.011631	0.011738
0.011466	0.011637	0.011568	0.011500	0.011767	0.011228	0.011431
0.011396	0.011329	0.011228	0.011228	0.011295	0.011295	0.011362
0.011295	0.011362	0.011262	0.011362	0.011295	0.011295	0.011362
0.011295	0.011731	0.011631	0.011664	0.011194	0.011295	0.011329
0.011329	0.011295	0.011362	0.011396	0.011262	0.011262	0.011262
0.011799	0.011429	0.011429	0.011228	0.011329	0.011497	0.011262
0.011262	0.011329	0.011295	0.011463	0.011262	0.011396	0.011530
0.011463	0.011295	0.011295	0.011228	0.011228	0.011396	0.011329
0.011228	0.011329	0.011195	0.011228	0.011362	0.011497	0.011262
0.011228	0.011362	0.011295	0.011195	0.011530	0.011195	0.011362
0.011437	0.011262	0.011564	0.011396	0.011429	0.011295	0.011396
0.011262	0.011597	0.011194	0.011463	0.011262	0.011262	0.011194
0.011429	0.011631	0.011329	0.011396	0.011228	0.011631	0.011262
0.011262	0.011262	0.011295	0.011396	0.011295	0.011396	0.011463
0.011463	0.011429	0.011329	0.011429	0.011396	0.011262	0.011228
0.011262	0.011262	0.011329	0.011262	0.011228	0.011396	0.011262
0.011295	0.011295	0.011429	0.011463	0.011362	0.011295	0.011295
0.011362	0.011295	0.011396	0.011262	0.011396	0.011496	0.011799
0.011295	0.011698	0.011302	0.017823	0.011388	0.011366	0.011653
0.011614	0.011658	0.011322	0.011292	0.011774	0.011682	0.011364
0.011582	0.011549	0.011216	0.011270	0.011294	0.011356	0.011275
0.011576	0.011470	0.011468	0.011391	0.011429	0.011463	0.011466
0.011335	0.011429	0.011429	0.011396	0.011362	0.011362	0.011329
0.011362	0.011295	0.011329	0.011362	0.011329	0.011262	0.011295
0.011295	0.011362	0.011262	0.011362	0.011396	0.011429	0.011329
0.011362	0.011329	0.011665	0.011665	0.011396	0.011430	0.011463
0.011228	0.011228	0.011262	0.011362	0.011228	0.011463	0.011463
0.011497	0.011496	0.011429	0.011530	0.011698	0.011532	0.011362
0.011362	0.011430	0.011463	0.011295	0.011362	0.011329	0.011362
0.011430	0.011430	0.011463	0.011430	0.011330	0.011530	0.011497
0.011365	0.011262	0.011362	0.011463	0.011362	0.011497	0.011530
0.011698	0.011402	0.011564	0.011530	0.011463	0.011631	0.011262
0.011431	0.011329	0.011295	0.011295	0.011362	0.011262	0.011362
0.011396	0.011429	0.011329	0.011362	0.011329	0.011665	0.011665
0.011396	0.011430	0.011496	0.011429	0.012069	0.011489	0.011412
0.011482	0.011442	0.011552	0.011441	0.011550	0.011474	0.011439
0.012022	0.011337	0.011471	0.011406	0.011680	0.011536	0.011639
0.012046	0.011475	0.011601	0.011664	0.011530	0.011530	0.011567
0.011228	0.011396	0.011530	0.011496	0.011499	0.011496	0.011464
0.011329	0.011262	0.011295	0.011329	0.011228	0.011295	0.011194
0.011329	0.011362	0.011262	0.011228	0.011329	0.011262	0.011295
0.011295	0.011228	0.011295	0.011329	0.011228	0.011429	0.011463
0.011362	0.011429	0.011362	0.011362	0.011295	0.011698	0.011329
0.011373	0.011295	0.011530	0.011535	0.011396	0.011396	0.011396
0.011396	0.011329	0.011295	0.011329	0.011429	0.011429	0.011362
0.011362	0.011497	0.011664	0.011396	0.011597	0.011597	0.011463

0.011295	0.011362	0.011396	0.011799	0.011362	0.011396	0.011362
0.011396	0.011329	0.011329	0.011631	0.011564	0.011597	0.011329
0.011429	0.011497	0.011362	0.011429	0.011396	0.011262	0.011597
0.011362	0.011329	0.011329	0.011497	0.011329	0.011429	0.011664
0.011362	0.011739	0.011732	0.011548	0.011286	0.011434	0.011370
0.011170	0.011761	0.011271	0.011507	0.011353	0.011417	0.011485
0.011570	0.011192	0.011450	0.011258	0.011330	0.011529	0.011425
0.011437	0.011336	0.011412	0.011342	0.011364	0.011497	0.011564
0.011564	0.011430	0.011533	0.011535	0.011670	0.011661	0.011362
0.011597	0.011463	0.011329	0.011430	0.011228	0.011463	0.011262
0.011329	0.011329	0.011295	0.011262	0.011295	0.011262	0.011362
0.011340	0.011340	0.011334	0.011368	0.011505	0.011470	0.011470
0.011469	0.011396	0.011208	0.011204	0.011331	0.011501	0.011501
0.011296	0.011365	0.011599	0.011567	0.011497	0.011531	0.011463
0.011396	0.011463	0.011463	0.011362	0.011933	0.011530	0.011597
0.011631	0.011701	0.011333	0.011407	0.011295	0.011497	0.011463
0.011497	0.011530	0.011530	0.011530	0.011650	0.011664	0.011303
0.011195	0.011199	0.011298	0.011264	0.011368	0.011209	0.011536
0.011335	0.011499	0.011203	0.011360	0.011329	0.011402	0.011295
0.011262	0.011262	0.011329	0.011295	0.011497	0.011463	0.011396
0.011497	0.011564	0.011295	0.011295	0.011329	0.011329	0.011330
0.011398	0.011329	0.011329	0.011364	0.011497	0.011564	0.011564
0.011430	0.011533	0.011535	0.011670	0.011661	0.011362	0.011530
0.011463	0.011463	0.011263	0.011443	0.011408	0.011435	0.011305
0.011473	0.011607	0.011441	0.011366	0.011262	0.011463	0.011329
0.011430	0.011362	0.011262	0.011363	0.011463	0.011466	0.011362
0.011262	0.011195	0.011329	0.011262	0.011195	0.011298	0.011463
0.011362	0.011295	0.011329	0.011362	0.011329	0.011262	0.011463
0.011329	0.011362	0.011362	0.011295	0.011362	0.011597	0.011262
0.011295	0.011698	0.011295	0.011329	0.011295	0.011664	0.011274
0.011791	0.011273	0.011361	0.011503	0.011589	0.011491	0.011227
0.011323	0.011463	0.011564	0.011671	0.011531	0.011669	0.011800
0.011699	0.011737	0.011698	0.011949	0.011664	0.012539	0.011700
0.012439	0.012207	0.012341	0.012613	0.011664	0.011362	0.011665
0.012103	0.012038	0.011631	0.011530	0.011598	0.011598	0.011567
0.011463	0.011467	0.011530	0.011497	0.011867	0.011933	0.011935
0.011834	0.011934	0.012038	0.011804	0.012104	0.012110	0.012171
0.012172	0.012205	0.012152	0.012172	0.012173	0.011396	0.011699
0.011767	0.012270	0.011772	0.012001	0.012474	0.011536	0.011430
0.011396	0.011396	0.011867	0.011924	0.012010	0.011732	0.011397
S050	0.011833	0.011833	0.012169	0.011933	0.011933	0.012001
0.011841	0.011773	0.012000	0.011430	0.011496	0.011464	0.011429
0.011463	0.011631	0.011429	0.011396	0.011530	0.011497	0.011463
0.011429	0.011463	0.011564	0.011430	0.011463	0.011463	0.011807
0.011698	0.011463	0.011362	0.011597	0.011530	0.011497	0.011530
0.011497	0.011597	0.011429	0.011497	0.011262	0.011497	0.011665
0.011429	0.011497	0.011262	0.011396	0.011564	0.011530	0.011530
0.011497	0.000000	0.011564	0.011832	0.011228	0.011765	0.011228
0.011463	0.011564	0.011497	0.011765	0.011866	0.011564	0.011665
0.011530	0.011597	0.011497	0.011732	0.011967	0.011497	0.011564
0.011463	0.011564	0.011530	0.011597	0.011530	0.011463	0.011463
0.011429	0.011463	0.011429	0.011530	0.011497	0.011531	0.011597
0.011530	0.011497	0.011564	0.011463	0.011597	0.011429	0.011497
0.011664	0.011597	0.011832	0.011665	0.011497	0.011732	0.011429
0.011654	0.011882	0.011664	0.011451	0.011396	0.011502	0.011612
0.012268	0.011670	0.011431	0.011664	0.011732	0.011530	0.011631
0.011564	0.011530	0.011597	0.011497	0.011597	0.011530	0.011530
0.011496	0.011597	0.011799	0.011664	0.011564	0.011530	0.011463
0.011430	0.011430	0.011497	0.011463	0.011665	0.011631	0.011564
0.011665	0.011732	0.011463	0.011463	0.011497	0.011497	0.011499

0.011567	0.011497	0.011497	0.011384	0.011458	0.011411	0.011497
0.011501	0.011438	0.011503	0.011474	0.011400	0.011437	0.011466
0.011744	0.011508	0.011543	0.011430	0.011441	0.011565	0.011531
0.011599	0.011532	0.011531	0.011631	0.011564	0.011866	0.011732
0.011765	0.011631	0.011497	0.011598	0.011396	0.011631	0.011430
0.011497	0.011497	0.011463	0.011429	0.011463	0.011430	0.011530
0.011497	0.011598	0.011531	0.011430	0.011532	0.011631	0.011635
0.011631	0.011531	0.011430	0.011364	0.011497	0.011430	0.011363
0.011468	0.011631	0.011430	0.011631	0.011396	0.011497	0.011463
0.011564	0.011463	0.011497	0.011463	0.011497	0.011362	0.011497
0.011531	0.011463	0.011530	0.011463	0.011497	0.011530	0.011766
0.011866	0.011799	0.011867	0.011498	0.011867	0.011497	0.011463
0.011499	0.011788	0.011609	0.011403	0.011875	0.011367	0.011454
0.011875	0.011774	0.011664	0.011866	0.011573	0.011732	0.011698
0.011564	0.011799	0.011664	0.011514	0.011563	0.011530	0.011602
0.011698	0.011600	0.011737	0.011805	0.011732	0.011799	0.011907
0.011636	0.011807	0.011670	0.011669	0.011902	0.011396	0.011599
0.011564	0.011497	0.011396	0.011396	0.011463	0.011463	0.011463
0.011463	0.011530	0.011429	0.011530	0.011463	0.011463	0.011463
0.011463	0.011900	0.011799	0.011833	0.011362	0.011463	0.011497
0.011497	0.011463	0.011530	0.011564	0.011429	0.011429	0.011429
0.011967	0.011597	0.011530	0.011396	0.011497	0.011598	0.011429
0.011429	0.011496	0.011463	0.011631	0.011429	0.011564	0.011698
0.011631	0.011463	0.011463	0.011396	0.011396	0.011564	0.011497
0.011396	0.011497	0.011362	0.011396	0.011530	0.011665	0.011430
0.011396	0.011530	0.011463	0.011362	0.011698	0.011362	0.011530
0.011608	0.011430	0.011732	0.011564	0.011597	0.011463	0.011295
0.011429	0.011765	0.011362	0.011631	0.011429	0.011429	0.011362
0.011597	0.011799	0.011497	0.011564	0.011396	0.011799	0.011429
0.011429	0.011429	0.011463	0.011564	0.011463	0.011564	0.011631
0.011631	0.011597	0.011429	0.011598	0.011564	0.011362	0.011396
0.011429	0.011362	0.011497	0.011429	0.011396	0.011564	0.011429
0.011463	0.011463	0.011597	0.011631	0.011530	0.011463	0.011463
0.011530	0.011463	0.011564	0.011429	0.011564	0.011664	0.011967
0.011463	0.011866	0.011474	0.018012	0.011519	0.011733	0.011828
0.011787	0.011834	0.011584	0.011525	0.011995	0.011855	0.011668
0.011677	0.011854	0.011372	0.011521	0.011490	0.011554	0.011491
0.011670	0.011656	0.011654	0.011325	0.011597	0.011631	0.011635
0.011507	0.011598	0.011597	0.011564	0.011530	0.011531	0.011497
0.011530	0.011463	0.011497	0.011530	0.011497	0.011430	0.011463
0.011463	0.011530	0.011430	0.011531	0.011564	0.011598	0.011497
0.011530	0.011497	0.011833	0.011833	0.011564	0.011531	0.011631
0.011396	0.011396	0.011430	0.011531	0.011396	0.011631	0.011631
0.011665	0.011664	0.011597	0.011698	0.011867	0.011700	0.011530
0.011530	0.011598	0.011631	0.011463	0.011530	0.011497	0.011463
0.011598	0.011598	0.011631	0.011598	0.011498	0.011698	0.011665
0.011534	0.011430	0.011530	0.011564	0.011531	0.011665	0.011698
0.011866	0.011573	0.011732	0.011698	0.011564	0.011799	0.011430
0.011532	0.011497	0.011463	0.011463	0.011530	0.011430	0.011531
0.011564	0.011598	0.011497	0.011530	0.011497	0.011833	0.011833
0.011564	0.011531	0.011664	0.011597	0.012238	0.011632	0.011553
0.011623	0.011580	0.011728	0.011580	0.011690	0.011612	0.011612
0.012194	0.011476	0.011608	0.011544	0.011818	0.011707	0.011809
0.012216	0.011663	0.011770	0.011833	0.011698	0.011698	0.011736
0.011396	0.011564	0.011698	0.011664	0.011668	0.011664	0.011633
0.011496	0.011429	0.011463	0.011497	0.011396	0.011463	0.011362
0.011497	0.011530	0.011429	0.011396	0.011497	0.011429	0.011463
0.011396	0.011396	0.011463	0.011497	0.011396	0.011597	0.011631
0.011530	0.011597	0.011530	0.011530	0.011463	0.011866	0.011497
0.011547	0.011463	0.011699	0.011705	0.011564	0.011497	0.011564

0.011564	0.011497	0.011463	0.011497	0.011597	0.011597	0.011530
0.011530	0.011665	0.011832	0.011564	0.011766	0.011766	0.011631
0.011463	0.011530	0.011564	0.011967	0.011530	0.011564	0.011530
0.011564	0.011497	0.011497	0.011799	0.011732	0.011765	0.011497
0.011597	0.011665	0.011530	0.011597	0.011564	0.011429	0.011765
0.011530	0.011497	0.011497	0.011665	0.011497	0.011597	0.011833
0.011530	0.011909	0.011900	0.011670	0.011455	0.011604	0.011473
0.011555	0.011917	0.011447	0.011686	0.011499	0.011366	0.011645
0.011672	0.011455	0.011617	0.011457	0.011492	0.011663	0.011587
0.011588	0.011493	0.011589	0.011518	0.011532	0.011665	0.011732
0.011732	0.011598	0.011703	0.011704	0.011840	0.011838	0.011530
0.011765	0.011631	0.011497	0.011598	0.011396	0.011631	0.011430
0.011497	0.011497	0.011463	0.011429	0.011463	0.011430	0.011530
0.011514	0.011515	0.011437	0.011540	0.011676	0.011641	0.011573
0.011640	0.011565	0.011393	0.011348	0.011501	0.011670	0.011671
0.011464	0.011535	0.011767	0.011737	0.011666	0.011700	0.011631
0.011564	0.011631	0.011631	0.011530	0.012102	0.011698	0.011766
0.011799	0.011879	0.011504	0.011580	0.011463	0.011665	0.011631
0.011666	0.011698	0.011698	0.011698	0.011808	0.011833	0.011477
0.011364	0.011372	0.011469	0.011434	0.011539	0.011396	0.011706
0.011506	0.011586	0.011441	0.011510	0.011497	0.011574	0.011463
0.011430	0.011430	0.011497	0.011463	0.011665	0.011631	0.011564
0.011665	0.011732	0.011463	0.011463	0.011497	0.011497	0.011499
0.011567	0.011497	0.011497	0.011532	0.011665	0.011732	0.011732
0.011598	0.011703	0.011704	0.011840	0.011838	0.011530	0.011698
0.011631	0.011631	0.011398	0.011652	0.011582	0.011605	0.011481
0.011611	0.011835	0.011653	0.011536	0.011430	0.011631	0.011497
0.011598	0.011531	0.011430	0.011532	0.011631	0.011635	0.011531
0.011430	0.011364	0.011497	0.011430	0.011363	0.011468	0.011631
0.011530	0.011463	0.011497	0.011530	0.011497	0.011430	0.011631
0.011497	0.011530	0.011530	0.011463	0.011530	0.011765	0.011429
0.011463	0.011866	0.011463	0.011497	0.011463	0.011832	0.011446
0.011847	0.011400	0.011461	0.011664	0.011712	0.011670	0.011395
0.011428	0.011631	0.011665	0.011773	0.011633	0.011771	0.011901
0.011801	0.011838	0.011799	0.012120	0.011832	0.012708	0.011869
0.012608	0.012376	0.012510	0.012782	0.011832	0.011531	0.011766
0.012272	0.012140	0.011799	0.011698	0.011767	0.011767	0.011737
0.011631	0.011637	0.011698	0.011666	0.012036	0.012102	0.012103
0.012003	0.012103	0.012207	0.011974	0.012273	0.012280	0.012340
0.012341	0.012374	0.012323	0.012341	0.012342	0.011564	0.011867
0.011935	0.012438	0.011942	0.012169	0.012643	0.011706	0.011598
0.011564	0.011564	0.012035	0.012089	0.012187	0.011900	0.011566
S051 0.011732	0.011732	0.012069	0.011833	0.011833	0.011900	
0.011706	0.011638	0.011900	0.011329	0.011396	0.011363	0.011329
0.011362	0.011530	0.011329	0.011362	0.011430	0.011396	0.011362
0.011329	0.011362	0.011463	0.011329	0.011363	0.011362	0.011672
0.011598	0.011362	0.011262	0.011497	0.011430	0.011396	0.011430
0.011396	0.011497	0.011396	0.011396	0.011430	0.011396	0.011564
0.011329	0.011396	0.011430	0.011362	0.011463	0.011497	0.011430
0.011396	0.011564	0.000000	0.011732	0.011396	0.011665	0.011396
0.011362	0.011463	0.011530	0.011665	0.011766	0.011463	0.011564
0.011430	0.011497	0.011396	0.011631	0.011867	0.011396	0.011463
0.011362	0.011463	0.011430	0.011497	0.011430	0.011430	0.011430
0.011329	0.011362	0.011329	0.011430	0.011396	0.011431	0.011497
0.011430	0.011396	0.011463	0.011362	0.011497	0.011329	0.011396
0.011564	0.011497	0.011732	0.011564	0.011396	0.011631	0.011396
0.011506	0.011733	0.011513	0.011256	0.011247	0.011392	0.011415
0.012086	0.011467	0.011225	0.011631	0.011631	0.011430	0.011531
0.011463	0.011430	0.011497	0.011396	0.011497	0.011430	0.011430
0.011396	0.011497	0.011699	0.011564	0.011463	0.011430	0.011363

0.011329	0.011329	0.011396	0.011363	0.011564	0.011531	0.011463
0.011564	0.011631	0.011363	0.011363	0.011396	0.011396	0.011398
0.011466	0.011396	0.011396	0.011276	0.011314	0.011306	0.011397
0.011399	0.011335	0.011282	0.011371	0.011298	0.011334	0.011364
0.011637	0.011405	0.011405	0.011329	0.011337	0.011464	0.011430
0.011498	0.011431	0.011430	0.011463	0.011463	0.011833	0.011632
0.011665	0.011531	0.011396	0.011497	0.011295	0.011530	0.011329
0.011396	0.011396	0.011363	0.011329	0.011362	0.011329	0.011430
0.011396	0.011497	0.011430	0.011329	0.011431	0.011531	0.011534
0.011531	0.011430	0.011329	0.011263	0.011396	0.011329	0.011262
0.011366	0.011531	0.011329	0.011531	0.011295	0.011396	0.011363
0.011463	0.011363	0.011396	0.011363	0.011396	0.011262	0.011396
0.011430	0.011363	0.011430	0.011363	0.011396	0.011430	0.011665
0.011766	0.011699	0.011766	0.011532	0.011766	0.011396	0.011363
0.011398	0.011626	0.011518	0.011266	0.011773	0.011265	0.011336
0.011740	0.011673	0.011564	0.011766	0.011470	0.011631	0.011598
0.011530	0.011699	0.011558	0.011431	0.011480	0.011430	0.011500
0.011598	0.011499	0.011635	0.011704	0.011631	0.011699	0.011806
0.011534	0.011706	0.011636	0.011568	0.011835	0.011295	0.011499
0.011463	0.011396	0.011295	0.011295	0.011362	0.011362	0.011430
0.011362	0.011430	0.011329	0.011430	0.011362	0.011362	0.011430
0.011362	0.011799	0.011699	0.011732	0.011262	0.011362	0.011396
0.011396	0.011362	0.011430	0.011463	0.011329	0.011329	0.011329
0.011867	0.011497	0.011497	0.011295	0.011396	0.011564	0.011329
0.011329	0.011396	0.011362	0.011531	0.011329	0.011463	0.011598
0.011530	0.011362	0.011362	0.011295	0.011295	0.011463	0.011396
0.011295	0.011396	0.011262	0.011295	0.011430	0.011564	0.011329
0.011296	0.011430	0.011363	0.011262	0.011598	0.011262	0.011430
0.011506	0.011329	0.011631	0.011463	0.011497	0.011362	0.011463
0.011329	0.011665	0.011262	0.011531	0.011329	0.011329	0.011262
0.011497	0.011699	0.011396	0.011463	0.011295	0.011699	0.011329
0.011329	0.011329	0.011362	0.011463	0.011362	0.011463	0.011530
0.011530	0.011497	0.011396	0.011497	0.011463	0.011329	0.011295
0.011329	0.011329	0.011396	0.011329	0.011295	0.011463	0.011329
0.011362	0.011362	0.011497	0.011530	0.011430	0.011362	0.011363
0.011430	0.011362	0.011463	0.011329	0.011463	0.011564	0.011867
0.011362	0.011766	0.011337	0.017830	0.011322	0.011428	0.011689
0.011649	0.011694	0.011374	0.011234	0.011719	0.011717	0.011425
0.011535	0.011549	0.011216	0.011312	0.011333	0.011406	0.011276
0.011482	0.011470	0.011505	0.011325	0.011497	0.011531	0.011534
0.011404	0.011497	0.011497	0.011463	0.011430	0.011430	0.011396
0.011430	0.011363	0.011396	0.011430	0.011396	0.011329	0.011363
0.011363	0.011430	0.011329	0.011430	0.011463	0.011497	0.011396
0.011430	0.011396	0.011732	0.011732	0.011463	0.011498	0.011531
0.011295	0.011295	0.011329	0.011430	0.011295	0.011531	0.011531
0.011564	0.011564	0.011497	0.011598	0.011766	0.011566	0.011430
0.011430	0.011497	0.011531	0.011363	0.011430	0.011396	0.011430
0.011497	0.011497	0.011531	0.011497	0.011397	0.011598	0.011564
0.011399	0.011329	0.011430	0.011531	0.011430	0.011564	0.011598
0.011766	0.011470	0.011631	0.011598	0.011530	0.011699	0.011329
0.011498	0.011397	0.011363	0.011363	0.011430	0.011329	0.011430
0.011463	0.011497	0.011396	0.011430	0.011396	0.011732	0.011732
0.011463	0.011498	0.011564	0.011497	0.012138	0.011561	0.011483
0.011553	0.011511	0.011623	0.011476	0.011621	0.011543	0.011509
0.012091	0.011407	0.011540	0.011475	0.011749	0.011605	0.011707
0.012115	0.011588	0.011669	0.011732	0.011598	0.011598	0.011635
0.011295	0.011463	0.011598	0.011564	0.011567	0.011564	0.011532
0.011396	0.011329	0.011362	0.011396	0.011295	0.011362	0.011262
0.011396	0.011430	0.011329	0.011295	0.011396	0.011329	0.011362
0.011362	0.011295	0.011362	0.011396	0.011295	0.011497	0.011530

0.011430	0.011497	0.011430	0.011430	0.011362	0.011766	0.011396
0.011443	0.011363	0.011598	0.011603	0.011463	0.011463	0.011463
0.011463	0.011396	0.011362	0.011396	0.011497	0.011497	0.011430
0.011430	0.011564	0.011732	0.011463	0.011665	0.011665	0.011530
0.011362	0.011430	0.011463	0.011867	0.011430	0.011463	0.011430
0.011463	0.011396	0.011396	0.011699	0.011631	0.011665	0.011396
0.011497	0.011564	0.011430	0.011497	0.011463	0.011329	0.011665
0.011430	0.011396	0.011396	0.011564	0.011396	0.011497	0.011732
0.011430	0.011774	0.011799	0.011508	0.011229	0.011502	0.011318
0.011225	0.011723	0.011271	0.011463	0.011304	0.011417	0.011432
0.011519	0.011258	0.011394	0.011192	0.011222	0.011462	0.011385
0.011437	0.011296	0.011448	0.011377	0.011431	0.011564	0.011632
0.011631	0.011498	0.011602	0.011603	0.011739	0.011768	0.011430
0.011665	0.011531	0.011396	0.011497	0.011295	0.011530	0.011329
0.011396	0.011396	0.011363	0.011329	0.011362	0.011329	0.011430
0.011410	0.011410	0.011403	0.011437	0.011574	0.011539	0.011539
0.011538	0.011464	0.011245	0.011240	0.011399	0.011569	0.011570
0.011363	0.011433	0.011667	0.011635	0.011565	0.011599	0.011530
0.011463	0.011530	0.011530	0.011430	0.012001	0.011598	0.011665
0.011699	0.011773	0.011401	0.011476	0.011363	0.011564	0.011530
0.011565	0.011598	0.011598	0.011598	0.011690	0.011732	0.011373
0.011263	0.011268	0.011367	0.011332	0.011436	0.011284	0.011604
0.011403	0.011499	0.011251	0.011398	0.011397	0.011471	0.011363
0.011329	0.011329	0.011396	0.011363	0.011564	0.011531	0.011463
0.011564	0.011631	0.011363	0.011363	0.011396	0.011396	0.011398
0.011466	0.011396	0.011396	0.011431	0.011564	0.011632	0.011631
0.011498	0.011602	0.011603	0.011739	0.011768	0.011430	0.011598
0.011530	0.011530	0.011365	0.011478	0.011478	0.011503	0.011341
0.011508	0.011653	0.011484	0.011434	0.011329	0.011531	0.011396
0.011497	0.011430	0.011329	0.011431	0.011531	0.011534	0.011430
0.011329	0.011263	0.011396	0.011329	0.011262	0.011366	0.011531
0.011430	0.011363	0.011396	0.011430	0.011396	0.011329	0.011531
0.011396	0.011430	0.011430	0.011362	0.011430	0.011665	0.011329
0.011362	0.011766	0.011362	0.011396	0.011362	0.011732	0.011232
0.011735	0.011230	0.011261	0.011503	0.011549	0.011491	0.011186
0.011218	0.011530	0.011631	0.011740	0.011599	0.011704	0.011868
0.011767	0.011805	0.011766	0.011984	0.011732	0.012608	0.011768
0.012508	0.012276	0.012410	0.012682	0.011732	0.011430	0.011733
0.012172	0.012073	0.011699	0.011598	0.011666	0.011666	0.011636
0.011530	0.011501	0.011598	0.011565	0.011935	0.012001	0.012003
0.011902	0.012003	0.012106	0.011839	0.012172	0.012179	0.012239
0.012240	0.012274	0.012187	0.012240	0.012242	0.011463	0.011767
0.011835	0.012338	0.011841	0.012069	0.012543	0.011604	0.011498
0.011463	0.011463	0.011935	0.011966	0.012046	0.011799	0.011465
S052	0.012000	0.012000	0.012068	0.011832	0.011832	0.011900
0.011332	0.011264	0.011900	0.011597	0.011664	0.011632	0.011597
0.011564	0.011799	0.011530	0.011631	0.011698	0.011664	0.011631
0.011597	0.011631	0.011463	0.011598	0.011631	0.011631	0.011298
0.011597	0.011631	0.011530	0.011497	0.011698	0.011664	0.011698
0.011664	0.011765	0.011664	0.011664	0.011698	0.011664	0.011832
0.011530	0.011664	0.011698	0.011631	0.011664	0.011765	0.011698
0.011664	0.011832	0.011732	0.000000	0.011664	0.011933	0.011664
0.011631	0.011665	0.011799	0.011933	0.011429	0.011664	0.011765
0.011698	0.011765	0.011664	0.011899	0.011463	0.011664	0.011732
0.011631	0.011731	0.011698	0.011765	0.011698	0.011698	0.011698
0.011597	0.011631	0.011597	0.011631	0.011664	0.011700	0.011765
0.011698	0.011664	0.011732	0.011631	0.011496	0.011597	0.011664
0.011832	0.011765	0.011530	0.011832	0.011664	0.011900	0.011664
0.011876	0.011359	0.011890	0.011568	0.011583	0.011723	0.011493
0.012121	0.011467	0.011637	0.011899	0.011900	0.011631	0.011530

0.011732	0.011698	0.011765	0.011664	0.011765	0.011698	0.011698
0.011664	0.011698	0.011967	0.011832	0.011732	0.011698	0.011631
0.011597	0.011530	0.011665	0.011631	0.011564	0.011530	0.011463
0.011564	0.011631	0.011631	0.011631	0.011665	0.011665	0.011668
0.011736	0.011665	0.011665	0.011528	0.011603	0.011551	0.011665
0.011636	0.011576	0.011651	0.011612	0.011570	0.011574	0.011634
0.011528	0.011646	0.011680	0.011598	0.011579	0.011733	0.011632
0.011699	0.011701	0.011699	0.011799	0.011731	0.012101	0.011833
0.011664	0.011732	0.011664	0.011698	0.011564	0.011530	0.011598
0.011664	0.011664	0.011631	0.011597	0.011631	0.011597	0.011698
0.011665	0.011765	0.011698	0.011597	0.011700	0.011799	0.011804
0.011799	0.011698	0.011597	0.011532	0.011665	0.011597	0.011530
0.011468	0.011799	0.011597	0.011799	0.011564	0.011665	0.011631
0.011732	0.011631	0.011665	0.011631	0.011665	0.011530	0.011665
0.011698	0.011631	0.011698	0.011631	0.011665	0.011698	0.011933
0.011362	0.011295	0.011362	0.011802	0.011430	0.011665	0.011631
0.011601	0.011909	0.011701	0.011575	0.011365	0.011538	0.011729
0.011366	0.011604	0.011832	0.012034	0.011743	0.011900	0.011866
0.011799	0.011967	0.011842	0.011719	0.011773	0.011698	0.011771
0.011866	0.011769	0.011906	0.011974	0.011900	0.011967	0.012077
0.011805	0.011977	0.011839	0.011838	0.012103	0.011564	0.011768
0.011731	0.011664	0.011564	0.011564	0.011631	0.011631	0.011698
0.011631	0.011698	0.011597	0.011631	0.011631	0.011631	0.011698
0.011631	0.011396	0.011295	0.011329	0.011530	0.011631	0.011664
0.011664	0.011564	0.011698	0.011664	0.011597	0.011597	0.011597
0.011463	0.011698	0.011765	0.011564	0.011664	0.011833	0.011597
0.011597	0.011664	0.011631	0.011799	0.011597	0.011530	0.011664
0.011597	0.011631	0.011631	0.011564	0.011564	0.011732	0.011664
0.011564	0.011664	0.011530	0.011564	0.011698	0.011497	0.011597
0.011564	0.011698	0.011631	0.011530	0.011262	0.011530	0.011698
0.011779	0.011597	0.011228	0.011732	0.011698	0.011631	0.011731
0.011597	0.011933	0.011530	0.011530	0.011530	0.011530	0.011530
0.011765	0.011631	0.011664	0.011732	0.011564	0.011295	0.011530
0.011597	0.011597	0.011631	0.011732	0.011631	0.011731	0.011799
0.011799	0.011765	0.011664	0.011765	0.011731	0.011597	0.011564
0.011597	0.011597	0.011664	0.011530	0.011564	0.011732	0.011597
0.011564	0.011631	0.011765	0.011732	0.011698	0.011631	0.011631
0.011698	0.011631	0.011731	0.011597	0.011732	0.011832	0.011530
0.011631	0.011429	0.011647	0.018163	0.011387	0.011611	0.011304
0.011268	0.011306	0.011899	0.011698	0.011445	0.011336	0.011728
0.011677	0.011426	0.011567	0.011688	0.011529	0.011554	0.011663
0.011389	0.011507	0.011357	0.011589	0.011765	0.011799	0.011804
0.011678	0.011765	0.011765	0.011665	0.011698	0.011698	0.011665
0.011698	0.011631	0.011664	0.011698	0.011665	0.011597	0.011631
0.011631	0.011698	0.011597	0.011698	0.011732	0.011765	0.011664
0.011631	0.011598	0.011396	0.011396	0.011732	0.011766	0.011799
0.011564	0.011564	0.011597	0.011698	0.011564	0.011799	0.011799
0.011833	0.011832	0.011765	0.011866	0.011429	0.011869	0.011698
0.011698	0.011765	0.011799	0.011631	0.011698	0.011665	0.011698
0.011766	0.011766	0.011799	0.011766	0.011633	0.011866	0.011833
0.011704	0.011597	0.011698	0.011799	0.011698	0.011766	0.011866
0.012034	0.011743	0.011900	0.011866	0.011799	0.011967	0.011598
0.011768	0.011665	0.011631	0.011631	0.011698	0.011597	0.011698
0.011732	0.011765	0.011664	0.011631	0.011598	0.011396	0.011396
0.011732	0.011766	0.011832	0.011765	0.012339	0.011846	0.011764
0.011834	0.011788	0.011904	0.011788	0.011900	0.011819	0.011750
0.012297	0.011683	0.011814	0.011752	0.012025	0.011877	0.011979
0.012318	0.011814	0.011939	0.012000	0.011866	0.011597	0.011905
0.011564	0.011732	0.011866	0.011832	0.011837	0.011564	0.011532
0.011664	0.011597	0.011631	0.011664	0.011564	0.011631	0.011530

0.011597	0.011631	0.011597	0.011564	0.011664	0.011597	0.011631
0.011631	0.011564	0.011631	0.011664	0.011564	0.011765	0.011799
0.011698	0.011698	0.011698	0.011698	0.011631	0.011429	0.011664
0.011443	0.011631	0.011598	0.011603	0.011664	0.011664	0.011731
0.011732	0.011664	0.011631	0.011664	0.011765	0.011765	0.011698
0.011698	0.011832	0.011329	0.011731	0.011933	0.011933	0.011799
0.011631	0.011698	0.011732	0.011530	0.011698	0.011664	0.011698
0.011732	0.011597	0.011597	0.011900	0.011900	0.011866	0.011664
0.011765	0.011832	0.011698	0.011765	0.011731	0.011597	0.011262
0.011698	0.011664	0.011597	0.011832	0.011664	0.011765	0.011530
0.011698	0.011400	0.011396	0.011589	0.011455	0.011706	0.011473
0.011445	0.011800	0.011622	0.011730	0.011646	0.011366	0.011432
0.011621	0.011324	0.011506	0.011390	0.011384	0.011596	0.011831
0.011814	0.011729	0.011694	0.011694	0.011701	0.011564	0.011631
0.011631	0.011699	0.011804	0.011603	0.011942	0.011979	0.011698
0.011664	0.011732	0.011664	0.011698	0.011564	0.011530	0.011598
0.011664	0.011664	0.011631	0.011597	0.011631	0.011597	0.011698
0.011654	0.011655	0.011642	0.011677	0.011813	0.011778	0.011777
0.011776	0.011733	0.011540	0.011528	0.011636	0.011806	0.011807
0.011632	0.011670	0.011936	0.011905	0.011834	0.011868	0.011799
0.011731	0.011799	0.011799	0.011698	0.011664	0.011866	0.011933
0.011967	0.011345	0.011606	0.011753	0.011631	0.011832	0.011799
0.011834	0.011866	0.011866	0.011866	0.011965	0.012000	0.011650
0.011533	0.011545	0.011639	0.011604	0.011641	0.011583	0.011196
0.011678	0.011455	0.011680	0.011697	0.011665	0.011710	0.011631
0.011597	0.011530	0.011665	0.011631	0.011564	0.011530	0.011463
0.011564	0.011631	0.011631	0.011631	0.011665	0.011665	0.011668
0.011736	0.011665	0.011665	0.011701	0.011564	0.011631	0.011631
0.011699	0.011804	0.011603	0.011942	0.011979	0.011698	0.011866
0.011799	0.011799	0.011635	0.011826	0.011755	0.011775	0.011656
0.011818	0.011972	0.011865	0.011706	0.011597	0.011799	0.011665
0.011765	0.011698	0.011597	0.011700	0.011799	0.011804	0.011698
0.011597	0.011532	0.011665	0.011597	0.011530	0.011468	0.011799
0.011698	0.011631	0.011664	0.011698	0.011665	0.011597	0.011530
0.011664	0.011698	0.011698	0.011631	0.011698	0.011933	0.011530
0.011631	0.011429	0.011631	0.011664	0.011631	0.000000	0.011660
0.011960	0.011485	0.011461	0.011543	0.011630	0.011563	0.011395
0.011427	0.011530	0.011631	0.011739	0.011599	0.011737	0.011868
0.011767	0.011804	0.011765	0.012017	0.011732	0.012607	0.011768
0.012507	0.012275	0.012409	0.012681	0.011664	0.011631	0.011732
0.012373	0.012106	0.011698	0.011598	0.011666	0.011666	0.011601
0.011530	0.011535	0.011597	0.011565	0.011935	0.012001	0.012002
0.011902	0.012002	0.012106	0.011872	0.012374	0.012381	0.012441
0.012442	0.012475	0.012426	0.012442	0.012443	0.011731	0.011968
0.012036	0.012539	0.012044	0.012001	0.012474	0.011536	0.011430
0.011329	0.011396	0.011934	0.011965	0.012046	0.011732	0.011465
S053	0.011664	0.011664	0.012001	0.011765	0.011765	0.011832
0.011671	0.011603	0.011832	0.011262	0.011329	0.011296	0.011262
0.011295	0.011463	0.011262	0.011228	0.011362	0.011329	0.011295
0.011262	0.011295	0.011396	0.011262	0.011295	0.011295	0.011637
0.011530	0.011295	0.011194	0.011429	0.011362	0.011329	0.011362
0.011329	0.011429	0.011262	0.011329	0.011094	0.011329	0.011496
0.011262	0.011329	0.011094	0.011228	0.011396	0.011362	0.011362
0.011329	0.011228	0.011396	0.011664	0.000000	0.011597	0.000000
0.011295	0.011396	0.011329	0.011597	0.011698	0.011396	0.011496
0.011362	0.011429	0.011329	0.011564	0.011799	0.011329	0.011396
0.011295	0.011396	0.011362	0.011429	0.011362	0.011295	0.011295
0.011262	0.011295	0.011262	0.011362	0.011329	0.011363	0.011429
0.011362	0.011329	0.011396	0.011295	0.011429	0.011262	0.011329
0.011496	0.011429	0.011664	0.011497	0.011329	0.011564	0.011262

0.011468	0.011695	0.011513	0.011255	0.011247	0.011318	0.011414
0.012085	0.011518	0.011225	0.011496	0.011564	0.011362	0.011463
0.011396	0.011362	0.011429	0.011329	0.011429	0.011362	0.011362
0.011329	0.011429	0.011631	0.011496	0.011396	0.011362	0.011295
0.011262	0.011262	0.011329	0.011295	0.011497	0.011463	0.011396
0.011497	0.011564	0.011295	0.011295	0.011329	0.011329	0.011330
0.011398	0.011329	0.011329	0.011204	0.011277	0.011236	0.011329
0.011331	0.011266	0.011318	0.011302	0.011230	0.011266	0.011297
0.011564	0.011336	0.011370	0.011262	0.011268	0.011397	0.011363
0.011430	0.011363	0.011363	0.011463	0.011396	0.011698	0.011564
0.011597	0.011463	0.011329	0.011430	0.011228	0.011463	0.011262
0.011329	0.011329	0.011295	0.011262	0.011295	0.011262	0.011362
0.011329	0.011429	0.011362	0.011262	0.011363	0.011463	0.011466
0.011463	0.011362	0.011262	0.011195	0.011329	0.011262	0.011195
0.011298	0.011463	0.011262	0.011463	0.011228	0.011329	0.011295
0.011396	0.011295	0.011329	0.011295	0.011329	0.011195	0.011329
0.011362	0.011295	0.011362	0.011295	0.011329	0.011362	0.011597
0.011698	0.011631	0.011698	0.011330	0.011698	0.011329	0.011295
0.011330	0.011626	0.011472	0.011232	0.011705	0.011197	0.011296
0.011705	0.011604	0.011496	0.011698	0.011402	0.011564	0.011530
0.011396	0.011631	0.011486	0.011307	0.011354	0.011362	0.011432
0.011530	0.011431	0.011567	0.011635	0.011564	0.011631	0.011738
0.011466	0.011637	0.011500	0.011500	0.011733	0.011228	0.011431
0.011396	0.011329	0.011228	0.011228	0.011295	0.011295	0.011295
0.011295	0.011362	0.011262	0.011362	0.011295	0.011295	0.011295
0.011295	0.011731	0.011631	0.011664	0.011194	0.011295	0.011329
0.011329	0.011295	0.011362	0.011396	0.011262	0.011262	0.011262
0.011799	0.011429	0.011429	0.011228	0.011329	0.011429	0.011262
0.011262	0.011329	0.011295	0.011463	0.011262	0.011396	0.011530
0.011463	0.011295	0.011295	0.011228	0.011228	0.011396	0.011329
0.011228	0.011329	0.011195	0.011228	0.011362	0.011497	0.011262
0.011228	0.011362	0.011295	0.011195	0.011530	0.011195	0.011362
0.011437	0.011262	0.011564	0.011396	0.011429	0.011295	0.011127
0.011262	0.011597	0.011194	0.011463	0.011262	0.011262	0.011194
0.011429	0.011631	0.011329	0.011396	0.011228	0.011631	0.011262
0.011262	0.011262	0.011295	0.011396	0.011295	0.011396	0.011463
0.011463	0.011429	0.011262	0.011429	0.011396	0.011194	0.011228
0.011262	0.011194	0.011329	0.011262	0.011228	0.011396	0.011262
0.011295	0.011295	0.011429	0.011463	0.011362	0.011295	0.011295
0.011362	0.011295	0.011396	0.011262	0.011396	0.011496	0.011799
0.011295	0.011698	0.011302	0.017823	0.011322	0.011549	0.011653
0.011614	0.011658	0.011374	0.011292	0.011774	0.011682	0.011425
0.011487	0.011610	0.011177	0.011311	0.011294	0.011356	0.011318
0.011529	0.011470	0.011468	0.011126	0.011429	0.011463	0.011466
0.011335	0.011429	0.011429	0.011396	0.011362	0.011362	0.011329
0.011362	0.011295	0.011329	0.011362	0.011329	0.011262	0.011295
0.011295	0.011362	0.011262	0.011362	0.011396	0.011429	0.011329
0.011362	0.011329	0.011665	0.011665	0.011396	0.011363	0.011463
0.011228	0.011228	0.011262	0.011362	0.011228	0.011463	0.011463
0.011497	0.011496	0.011429	0.011530	0.011698	0.011532	0.011362
0.011362	0.011429	0.011463	0.011295	0.011362	0.011329	0.011295
0.011430	0.011430	0.011463	0.011430	0.011330	0.011530	0.011497
0.011365	0.011262	0.011362	0.011396	0.011362	0.011497	0.011530
0.011698	0.011402	0.011564	0.011530	0.011396	0.011631	0.011262
0.011363	0.011329	0.011295	0.011295	0.011362	0.011262	0.011362
0.011396	0.011429	0.011329	0.011362	0.011329	0.011665	0.011665
0.011396	0.011363	0.011496	0.011429	0.012069	0.011453	0.011377
0.011447	0.011407	0.011552	0.011441	0.011515	0.011439	0.011439
0.012021	0.011302	0.011437	0.011371	0.011645	0.011536	0.011639
0.012046	0.011475	0.011601	0.011664	0.011530	0.011530	0.011567

0.011228	0.011396	0.011530	0.011496	0.011499	0.011496	0.011464
0.011329	0.011262	0.011295	0.011329	0.011228	0.011295	0.011194
0.011329	0.011362	0.011262	0.011228	0.011329	0.011262	0.011295
0.011228	0.011228	0.011295	0.011329	0.011228	0.011429	0.011463
0.011362	0.011429	0.011362	0.011362	0.011295	0.011698	0.011329
0.011373	0.011295	0.011530	0.011535	0.011396	0.011329	0.011396
0.011396	0.011329	0.011295	0.011329	0.011429	0.011429	0.011362
0.011362	0.011496	0.011664	0.011396	0.011597	0.011597	0.011463
0.011295	0.011362	0.011396	0.011799	0.011362	0.011396	0.011362
0.011396	0.011329	0.011329	0.011631	0.011564	0.011597	0.011329
0.011429	0.011496	0.011362	0.011429	0.011396	0.011262	0.011597
0.011362	0.011329	0.011329	0.011496	0.011329	0.011429	0.011664
0.011362	0.011739	0.011731	0.011507	0.011286	0.011434	0.011267
0.011335	0.011722	0.011271	0.011462	0.011304	0.011162	0.011432
0.011519	0.011192	0.011394	0.011258	0.011276	0.011462	0.011385
0.011399	0.011296	0.011412	0.011342	0.011364	0.011497	0.011564
0.011564	0.011430	0.011533	0.011535	0.011670	0.011696	0.011362
0.011597	0.011463	0.011329	0.011430	0.011228	0.011463	0.011262
0.011329	0.011329	0.011295	0.011262	0.011295	0.011262	0.011362
0.011340	0.011340	0.011266	0.011368	0.011505	0.011470	0.011402
0.011469	0.011396	0.011208	0.011168	0.011331	0.011501	0.011501
0.011296	0.011365	0.011599	0.011567	0.011497	0.011531	0.011463
0.011396	0.011463	0.011463	0.011362	0.011933	0.011530	0.011597
0.011631	0.011701	0.011333	0.011407	0.011295	0.011497	0.011463
0.011497	0.011530	0.011530	0.011530	0.011611	0.011664	0.011303
0.011195	0.011199	0.011298	0.011264	0.011368	0.011209	0.011536
0.011335	0.011455	0.011203	0.011322	0.011329	0.011402	0.011295
0.011262	0.011262	0.011329	0.011295	0.011497	0.011463	0.011396
0.011497	0.011564	0.011295	0.011295	0.011329	0.011329	0.011330
0.011398	0.011329	0.011329	0.011364	0.011497	0.011564	0.011564
0.011430	0.011533	0.011535	0.011670	0.011696	0.011362	0.011530
0.011463	0.011463	0.011229	0.011478	0.011408	0.011435	0.011305
0.011473	0.011653	0.011484	0.011366	0.011262	0.011463	0.011329
0.011429	0.011362	0.011262	0.011363	0.011463	0.011466	0.011362
0.011262	0.011195	0.011329	0.011262	0.011195	0.011298	0.011463
0.011362	0.011295	0.011329	0.011362	0.011329	0.011262	0.011463
0.011329	0.011362	0.011362	0.011295	0.011362	0.011597	0.011262
0.011295	0.011698	0.011295	0.011329	0.011295	0.011664	0.011274
0.011678	0.011230	0.011311	0.011463	0.011549	0.011491	0.011227
0.011270	0.011463	0.011496	0.011603	0.011464	0.011601	0.011733
0.011632	0.011669	0.011631	0.011949	0.011664	0.012539	0.011700
0.012439	0.012207	0.012341	0.012612	0.011664	0.011362	0.011598
0.012103	0.011971	0.011631	0.011530	0.011598	0.011598	0.011567
0.011463	0.011467	0.011530	0.011497	0.011867	0.011933	0.011935
0.011834	0.011934	0.012038	0.011804	0.012104	0.012110	0.012171
0.012172	0.012205	0.012152	0.012172	0.012173	0.011396	0.011699
0.011766	0.012270	0.011772	0.012001	0.012474	0.011536	0.011430
0.011396	0.011396	0.011867	0.011883	0.012010	0.011732	0.011397
S054	0.011933	0.011933	0.012270	0.012034	0.012034	0.012101
0.011909	0.011841	0.012101	0.011530	0.011597	0.011565	0.011530
0.011564	0.011732	0.011530	0.011564	0.011631	0.011597	0.011564
0.011530	0.011564	0.011664	0.011531	0.011497	0.011564	0.011875
0.011799	0.011564	0.011463	0.011698	0.011564	0.011597	0.011564
0.011597	0.011631	0.011597	0.011597	0.011631	0.011597	0.011765
0.011530	0.011597	0.011631	0.011564	0.011664	0.011698	0.011631
0.011597	0.011765	0.011665	0.011933	0.011597	0.000000	0.011597
0.011564	0.011665	0.011732	0.000000	0.011967	0.011664	0.011765
0.011564	0.011631	0.011597	0.011765	0.012068	0.011597	0.011664
0.011564	0.011664	0.011631	0.011698	0.011631	0.011631	0.011631
0.011530	0.011564	0.011530	0.011631	0.011597	0.011632	0.011698

0.011631	0.011597	0.011597	0.011564	0.011698	0.011463	0.011530
0.011765	0.011698	0.011933	0.011765	0.011597	0.011765	0.011597
0.011765	0.011994	0.011777	0.011451	0.011508	0.011649	0.011690
0.012341	0.011670	0.011431	0.011832	0.011832	0.011631	0.011732
0.011664	0.011631	0.011698	0.011597	0.011698	0.011564	0.011631
0.011597	0.011698	0.011900	0.011765	0.011664	0.011631	0.011497
0.011530	0.011530	0.011530	0.011564	0.011765	0.011732	0.011665
0.011765	0.011833	0.011564	0.011564	0.011597	0.011597	0.011601
0.011669	0.011597	0.011597	0.011456	0.011530	0.011516	0.011598
0.011602	0.011541	0.011577	0.011578	0.011502	0.011540	0.011567
0.011852	0.011612	0.011577	0.011531	0.011544	0.011666	0.011632
0.011699	0.011633	0.011632	0.011731	0.011664	0.011967	0.011833
0.011866	0.011732	0.011597	0.011698	0.011497	0.011732	0.011530
0.011597	0.011597	0.011564	0.011530	0.011564	0.011530	0.011631
0.011530	0.011631	0.011631	0.011530	0.011633	0.011732	0.011736
0.011665	0.011631	0.011530	0.011465	0.011597	0.011530	0.011463
0.011570	0.011665	0.011463	0.011665	0.011429	0.011598	0.011497
0.011597	0.011564	0.011530	0.011497	0.011530	0.011463	0.011530
0.011564	0.011564	0.011631	0.011564	0.011597	0.011631	0.011866
0.011967	0.011900	0.011967	0.011734	0.011967	0.011597	0.011564
0.011601	0.011909	0.011609	0.011472	0.011976	0.011469	0.011611
0.011943	0.011876	0.011765	0.011967	0.011675	0.011832	0.011732
0.011732	0.011900	0.011771	0.011637	0.011689	0.011631	0.011703
0.011799	0.011701	0.011838	0.011601	0.011832	0.011900	0.012009
0.011737	0.011603	0.011839	0.011770	0.012036	0.011496	0.011700
0.011597	0.011597	0.011496	0.011496	0.011496	0.011564	0.011631
0.011496	0.011564	0.011463	0.011631	0.011496	0.011564	0.011631
0.011564	0.012000	0.011900	0.011933	0.011463	0.011564	0.011597
0.011597	0.011496	0.011631	0.011664	0.011530	0.011530	0.011530
0.012068	0.011698	0.011698	0.011496	0.011597	0.011698	0.011530
0.011530	0.011597	0.011564	0.011664	0.011530	0.011664	0.011731
0.011731	0.011564	0.011564	0.011496	0.011496	0.011664	0.011597
0.011496	0.011597	0.011463	0.011496	0.011631	0.011766	0.011530
0.011497	0.011631	0.011564	0.011463	0.011799	0.011463	0.011631
0.011642	0.011530	0.011832	0.011665	0.011698	0.011564	0.011664
0.011530	0.000000	0.011463	0.011732	0.011530	0.011530	0.011463
0.011631	0.011900	0.011597	0.011664	0.011496	0.011900	0.011530
0.011530	0.011530	0.011564	0.011597	0.011564	0.011664	0.011731
0.011732	0.011631	0.011597	0.011698	0.011597	0.011530	0.011496
0.011530	0.011530	0.011597	0.011530	0.011429	0.011664	0.011530
0.011564	0.011564	0.011698	0.011732	0.011631	0.011496	0.011564
0.011631	0.011564	0.011664	0.011530	0.011664	0.011765	0.012068
0.011564	0.011967	0.011578	0.018087	0.011518	0.011733	0.011932
0.011891	0.011940	0.011532	0.011466	0.011939	0.011958	0.011485
0.011677	0.011793	0.011450	0.011479	0.011450	0.011554	0.011576
0.011670	0.011731	0.011690	0.011523	0.011698	0.011732	0.011737
0.011541	0.011698	0.011698	0.011665	0.011631	0.011631	0.011598
0.011631	0.011564	0.011530	0.011631	0.011598	0.011530	0.011564
0.011564	0.011631	0.011530	0.011631	0.011665	0.011698	0.011597
0.011631	0.011597	0.011934	0.011934	0.011665	0.011699	0.011732
0.011497	0.011497	0.011530	0.011564	0.011497	0.011732	0.011732
0.011765	0.011765	0.011698	0.011799	0.011967	0.011801	0.011564
0.011631	0.011698	0.011665	0.011564	0.011631	0.011597	0.011631
0.011699	0.011698	0.011732	0.011631	0.011464	0.011799	0.011698
0.011636	0.011530	0.011631	0.011732	0.011631	0.011766	0.011799
0.011967	0.011675	0.011832	0.011732	0.011732	0.011900	0.011531
0.011700	0.011598	0.011564	0.011564	0.011631	0.011530	0.011631
0.011665	0.011698	0.011597	0.011631	0.011597	0.011934	0.011934
0.011665	0.011699	0.011765	0.011698	0.012339	0.011775	0.011693
0.011763	0.011719	0.011833	0.011718	0.011830	0.011750	0.011715

0.012297	0.011614	0.011745	0.011683	0.011956	0.011808	0.011638
0.012318	0.011814	0.011872	0.011933	0.011799	0.011799	0.011837
0.011496	0.011597	0.011731	0.011698	0.011770	0.011765	0.011734
0.011597	0.011463	0.011496	0.011530	0.011429	0.011564	0.011463
0.011597	0.011631	0.011530	0.011496	0.011597	0.011530	0.011564
0.011564	0.011496	0.011564	0.011597	0.011496	0.011631	0.011731
0.011631	0.011698	0.011631	0.011631	0.011564	0.011967	0.011597
0.011652	0.011564	0.011799	0.011807	0.011597	0.011664	0.011664
0.011664	0.011530	0.011564	0.011530	0.011698	0.011698	0.011631
0.011631	0.011765	0.011933	0.011597	0.011866	0.011866	0.011731
0.011564	0.011631	0.011664	0.012068	0.011631	0.011597	0.011631
0.011664	0.011597	0.011597	0.011832	0.011765	0.011799	0.011597
0.011698	0.011765	0.011631	0.011698	0.011664	0.011530	0.011866
0.011631	0.011597	0.011597	0.011765	0.011597	0.011698	0.011933
0.011631	0.011977	0.012000	0.011792	0.011455	0.011706	0.011421
0.011500	0.011956	0.011552	0.011596	0.011499	0.011671	0.011591
0.011723	0.011389	0.011561	0.011324	0.011437	0.011529	0.011628
0.011663	0.011493	0.011694	0.011624	0.011633	0.011765	0.011833
0.011833	0.011699	0.011804	0.011806	0.011942	0.011979	0.011631
0.011866	0.011732	0.011597	0.011698	0.011497	0.011732	0.011530
0.011597	0.011597	0.011564	0.011530	0.011564	0.011530	0.011631
0.011619	0.011620	0.011608	0.011642	0.011779	0.011675	0.011743
0.011742	0.011665	0.011503	0.011492	0.011602	0.011772	0.011773
0.011565	0.011636	0.011868	0.011838	0.011767	0.011801	0.011731
0.011664	0.011732	0.011731	0.011631	0.012202	0.011732	0.011866
0.011900	0.011915	0.011606	0.011684	0.011564	0.011765	0.011732
0.011767	0.011799	0.011799	0.011799	0.011925	0.011933	0.011581
0.011466	0.011476	0.011571	0.011536	0.011641	0.011508	0.011808
0.011609	0.011674	0.011441	0.011622	0.011598	0.011676	0.011497
0.011530	0.011530	0.011530	0.011564	0.011765	0.011732	0.011665
0.011765	0.011833	0.011564	0.011564	0.011597	0.011597	0.011601
0.011669	0.011597	0.011597	0.011633	0.011765	0.011833	0.011833
0.011699	0.011804	0.011806	0.011942	0.011979	0.011631	0.011799
0.011732	0.011732	0.011567	0.011756	0.011651	0.011707	0.011516
0.011646	0.011926	0.011695	0.011638	0.011463	0.011665	0.011530
0.011631	0.011631	0.011530	0.011633	0.011732	0.011736	0.011631
0.011530	0.011465	0.011597	0.011530	0.011463	0.011570	0.011665
0.011631	0.011564	0.011530	0.011631	0.011598	0.011530	0.011732
0.011597	0.011631	0.011631	0.011564	0.011631	0.011866	0.011530
0.011564	0.011967	0.011564	0.011530	0.011564	0.011933	0.011446
0.011960	0.011485	0.011461	0.011745	0.011793	0.011778	0.011395
0.011427	0.011731	0.011832	0.011943	0.011801	0.011872	0.012069
0.011969	0.012007	0.011967	0.012188	0.011933	0.012809	0.011970
0.012709	0.012477	0.012611	0.012883	0.011933	0.011631	0.011934
0.012373	0.012309	0.011900	0.011799	0.011867	0.011867	0.011838
0.011731	0.011739	0.011799	0.011766	0.012137	0.012202	0.012204
0.012103	0.012204	0.012308	0.012075	0.012374	0.012381	0.012441
0.012442	0.012475	0.012426	0.012442	0.012443	0.011664	0.011968
0.012036	0.012539	0.012010	0.012203	0.012676	0.011807	0.011699
0.011664	0.011664	0.012136	0.012254	0.012292	0.012000	0.011667
S055 0.011664	0.011664	0.012001	0.011765	0.011765	0.011832	
0.011671	0.011603	0.011832	0.011262	0.011329	0.011296	0.011262
0.011295	0.011463	0.011262	0.011228	0.011362	0.011329	0.011295
0.011262	0.011295	0.011396	0.011262	0.011295	0.011295	0.011637
0.011530	0.011295	0.011194	0.011429	0.011362	0.011329	0.011362
0.011329	0.011429	0.011262	0.011329	0.011094	0.011329	0.011496
0.011262	0.011329	0.011094	0.011228	0.011396	0.011362	0.011362
0.011329	0.011228	0.011396	0.011664	0.000000	0.011597	0.000000
0.011295	0.011396	0.011329	0.011597	0.011698	0.011396	0.011496
0.011362	0.011429	0.011329	0.011564	0.011799	0.011329	0.011396

0.011295	0.011396	0.011362	0.011429	0.011362	0.011295	0.011295
0.011262	0.011295	0.011262	0.011362	0.011329	0.011363	0.011429
0.011362	0.011329	0.011396	0.011295	0.011429	0.011262	0.011329
0.011496	0.011429	0.011664	0.011497	0.011329	0.011564	0.011262
0.011468	0.011695	0.011513	0.011255	0.011247	0.011318	0.011414
0.012085	0.011518	0.011225	0.011496	0.011564	0.011362	0.011463
0.011396	0.011362	0.011429	0.011329	0.011429	0.011362	0.011362
0.011329	0.011429	0.011631	0.011496	0.011396	0.011362	0.011295
0.011262	0.011262	0.011329	0.011295	0.011497	0.011463	0.011396
0.011497	0.011564	0.011295	0.011295	0.011329	0.011329	0.011330
0.011398	0.011329	0.011329	0.011204	0.011277	0.011236	0.011329
0.011331	0.011266	0.011318	0.011302	0.011230	0.011266	0.011297
0.011564	0.011336	0.011370	0.011262	0.011268	0.011397	0.011363
0.011430	0.011363	0.011363	0.011463	0.011396	0.011698	0.011564
0.011597	0.011463	0.011329	0.011430	0.011228	0.011463	0.011262
0.011329	0.011329	0.011295	0.011262	0.011295	0.011262	0.011362
0.011329	0.011429	0.011362	0.011262	0.011363	0.011463	0.011466
0.011463	0.011362	0.011262	0.011195	0.011329	0.011262	0.011195
0.011298	0.011463	0.011262	0.011463	0.011228	0.011329	0.011295
0.011396	0.011295	0.011329	0.011295	0.011329	0.011195	0.011329
0.011362	0.011295	0.011362	0.011295	0.011329	0.011362	0.011597
0.011698	0.011631	0.011698	0.011330	0.011698	0.011329	0.011295
0.011330	0.011626	0.011472	0.011232	0.011705	0.011197	0.011296
0.011705	0.011604	0.011496	0.011698	0.011402	0.011564	0.011530
0.011396	0.011631	0.011486	0.011307	0.011354	0.011362	0.011432
0.011530	0.011431	0.011567	0.011635	0.011564	0.011631	0.011738
0.011466	0.011637	0.011500	0.011500	0.011733	0.011228	0.011431
0.011396	0.011329	0.011228	0.011228	0.011295	0.011295	0.011295
0.011295	0.011362	0.011262	0.011362	0.011295	0.011295	0.011295
0.011295	0.011731	0.011631	0.011664	0.011194	0.011295	0.011329
0.011329	0.011295	0.011362	0.011396	0.011262	0.011262	0.011262
0.011799	0.011429	0.011429	0.011228	0.011329	0.011429	0.011262
0.011262	0.011329	0.011295	0.011463	0.011262	0.011396	0.011530
0.011463	0.011295	0.011295	0.011228	0.011228	0.011396	0.011329
0.011228	0.011329	0.011195	0.011228	0.011362	0.011497	0.011262
0.011228	0.011362	0.011295	0.011195	0.011530	0.011195	0.011362
0.011437	0.011262	0.011564	0.011396	0.011429	0.011295	0.011127
0.011262	0.011597	0.011194	0.011463	0.011262	0.011262	0.011194
0.011429	0.011631	0.011329	0.011396	0.011228	0.011631	0.011262
0.011262	0.011262	0.011295	0.011396	0.011295	0.011396	0.011463
0.011463	0.011429	0.011262	0.011429	0.011396	0.011194	0.011228
0.011262	0.011194	0.011329	0.011262	0.011228	0.011396	0.011262
0.011295	0.011295	0.011429	0.011463	0.011362	0.011295	0.011295
0.011362	0.011295	0.011396	0.011262	0.011396	0.011496	0.011799
0.011295	0.011698	0.011302	0.017823	0.011322	0.011549	0.011653
0.011614	0.011658	0.011374	0.011292	0.011774	0.011682	0.011425
0.011487	0.011610	0.011177	0.011311	0.011294	0.011356	0.011318
0.011529	0.011470	0.011468	0.011126	0.011429	0.011463	0.011466
0.011335	0.011429	0.011429	0.011396	0.011362	0.011362	0.011329
0.011362	0.011295	0.011329	0.011362	0.011329	0.011262	0.011295
0.011295	0.011362	0.011262	0.011362	0.011396	0.011429	0.011329
0.011362	0.011329	0.011665	0.011665	0.011396	0.011363	0.011463
0.011228	0.011228	0.011262	0.011362	0.011228	0.011463	0.011463
0.011497	0.011496	0.011429	0.011530	0.011698	0.011532	0.011362
0.011362	0.011429	0.011463	0.011295	0.011362	0.011329	0.011295
0.011430	0.011430	0.011463	0.011430	0.011330	0.011530	0.011497
0.011365	0.011262	0.011362	0.011396	0.011362	0.011497	0.011530
0.011698	0.011402	0.011564	0.011530	0.011396	0.011631	0.011262
0.011363	0.011329	0.011295	0.011295	0.011362	0.011262	0.011362
0.011396	0.011429	0.011329	0.011362	0.011329	0.011665	0.011665

0.011396	0.011363	0.011496	0.011429	0.012069	0.011453	0.011377
0.011447	0.011407	0.011552	0.011441	0.011515	0.011439	0.011439
0.012021	0.011302	0.011437	0.011371	0.011645	0.011536	0.011639
0.012046	0.011475	0.011601	0.011664	0.011530	0.011530	0.011567
0.011228	0.011396	0.011530	0.011496	0.011499	0.011496	0.011464
0.011329	0.011262	0.011295	0.011329	0.011228	0.011295	0.011194
0.011329	0.011362	0.011262	0.011228	0.011329	0.011262	0.011295
0.011228	0.011228	0.011295	0.011329	0.011228	0.011429	0.011463
0.011362	0.011429	0.011362	0.011362	0.011295	0.011698	0.011329
0.011373	0.011295	0.011530	0.011535	0.011396	0.011329	0.011396
0.011396	0.011329	0.011295	0.011329	0.011429	0.011429	0.011362
0.011362	0.011496	0.011664	0.011396	0.011597	0.011597	0.011463
0.011295	0.011362	0.011396	0.011799	0.011362	0.011396	0.011362
0.011396	0.011329	0.011329	0.011631	0.011564	0.011597	0.011329
0.011429	0.011496	0.011362	0.011429	0.011396	0.011262	0.011597
0.011362	0.011329	0.011329	0.011496	0.011329	0.011429	0.011664
0.011362	0.011739	0.011731	0.011507	0.011286	0.011434	0.011267
0.011335	0.011722	0.011271	0.011462	0.011304	0.011162	0.011432
0.011519	0.011192	0.011394	0.011258	0.011276	0.011462	0.011385
0.011399	0.011296	0.011412	0.011342	0.011364	0.011497	0.011564
0.011564	0.011430	0.011533	0.011535	0.011670	0.011696	0.011362
0.011597	0.011463	0.011329	0.011430	0.011228	0.011463	0.011262
0.011329	0.011329	0.011295	0.011262	0.011295	0.011262	0.011362
0.011340	0.011340	0.011266	0.011368	0.011505	0.011470	0.011402
0.011469	0.011396	0.011208	0.011168	0.011331	0.011501	0.011501
0.011296	0.011365	0.011599	0.011567	0.011497	0.011531	0.011463
0.011396	0.011463	0.011463	0.011362	0.011933	0.011530	0.011597
0.011631	0.011701	0.011333	0.011407	0.011295	0.011497	0.011463
0.011497	0.011530	0.011530	0.011530	0.011611	0.011664	0.011303
0.011195	0.011199	0.011298	0.011264	0.011368	0.011209	0.011536
0.011335	0.011455	0.011203	0.011322	0.011329	0.011402	0.011295
0.011262	0.011262	0.011329	0.011295	0.011497	0.011463	0.011396
0.011497	0.011564	0.011295	0.011295	0.011329	0.011329	0.011330
0.011398	0.011329	0.011329	0.011364	0.011497	0.011564	0.011564
0.011430	0.011533	0.011535	0.011670	0.011696	0.011362	0.011530
0.011463	0.011463	0.011229	0.011478	0.011408	0.011435	0.011305
0.011473	0.011653	0.011484	0.011366	0.011262	0.011463	0.011329
0.011429	0.011362	0.011262	0.011363	0.011463	0.011466	0.011362
0.011262	0.011195	0.011329	0.011262	0.011195	0.011298	0.011463
0.011362	0.011295	0.011329	0.011362	0.011329	0.011262	0.011463
0.011329	0.011362	0.011362	0.011295	0.011362	0.011597	0.011262
0.011295	0.011698	0.011295	0.011329	0.011295	0.011664	0.011274
0.011678	0.011230	0.011311	0.011463	0.011549	0.011491	0.011227
0.011270	0.011463	0.011496	0.011603	0.011464	0.011601	0.011733
0.011632	0.011669	0.011631	0.011949	0.011664	0.012539	0.011700
0.012439	0.012207	0.012341	0.012612	0.011664	0.011362	0.011598
0.012103	0.011971	0.011631	0.011530	0.011598	0.011598	0.011567
0.011463	0.011467	0.011530	0.011497	0.011867	0.011933	0.011935
0.011834	0.011934	0.012038	0.011804	0.012104	0.012110	0.012171
0.012172	0.012205	0.012152	0.012172	0.012173	0.011396	0.011699
0.011766	0.012270	0.011772	0.012001	0.012474	0.011536	0.011430
0.011396	0.011396	0.011867	0.011883	0.012010	0.011732	0.011397
S056	0.011631	0.011631	0.011967	0.011731	0.011731	0.011799
0.011637	0.011569	0.011799	0.011228	0.011295	0.011262	0.011228
0.011262	0.011429	0.011228	0.011262	0.011329	0.011295	0.011262
0.011228	0.011262	0.011362	0.011228	0.011262	0.011262	0.011603
0.011496	0.011262	0.011161	0.011396	0.011329	0.011295	0.011329
0.011295	0.011396	0.011295	0.011295	0.011329	0.011295	0.011463
0.011228	0.011295	0.011329	0.011262	0.011362	0.011396	0.011329
0.011295	0.011463	0.011362	0.011631	0.011295	0.011564	0.011295

0.000000	0.011362	0.011429	0.011564	0.011664	0.011362	0.011463
0.011329	0.011396	0.011295	0.011530	0.011765	0.011295	0.011362
0.011262	0.011362	0.011329	0.011396	0.011329	0.011329	0.011329
0.011228	0.011262	0.011228	0.011329	0.011295	0.011329	0.011396
0.011329	0.011295	0.011362	0.011262	0.011396	0.011228	0.011295
0.011463	0.011396	0.011631	0.011463	0.011295	0.011530	0.011295
0.011468	0.011695	0.011513	0.011216	0.011210	0.011318	0.011414
0.012048	0.011467	0.011225	0.011530	0.011530	0.011329	0.011429
0.011362	0.011329	0.011396	0.011295	0.011396	0.011329	0.011329
0.011295	0.011396	0.011597	0.011463	0.011362	0.011329	0.011262
0.011228	0.011228	0.011295	0.011262	0.011463	0.011429	0.011362
0.011463	0.011530	0.011262	0.011262	0.011295	0.011295	0.011297
0.011364	0.011295	0.011295	0.011168	0.011241	0.011200	0.011296
0.011297	0.011232	0.011282	0.011267	0.011196	0.011231	0.011263
0.011528	0.011301	0.011336	0.011228	0.011233	0.011363	0.011329
0.011396	0.011330	0.011329	0.011429	0.011362	0.011732	0.011530
0.011564	0.011429	0.011295	0.011396	0.011195	0.011429	0.011228
0.011295	0.011295	0.011262	0.011228	0.011262	0.011228	0.011329
0.011295	0.011396	0.011329	0.011228	0.011330	0.011430	0.011432
0.011430	0.011329	0.011228	0.011161	0.011295	0.011228	0.011161
0.011264	0.011430	0.011228	0.011430	0.011195	0.011295	0.011262
0.011362	0.011262	0.011295	0.011262	0.011295	0.011161	0.011295
0.011329	0.011262	0.011329	0.011262	0.011295	0.011329	0.011564
0.011664	0.011597	0.011665	0.011431	0.011665	0.011295	0.011262
0.011297	0.011586	0.011426	0.011197	0.011671	0.011163	0.011296
0.011671	0.011570	0.011463	0.011664	0.011367	0.011530	0.011497
0.011429	0.011597	0.011451	0.011266	0.011312	0.011329	0.011398
0.011497	0.011397	0.011533	0.011601	0.011530	0.011597	0.011704
0.011432	0.011603	0.011534	0.011466	0.011733	0.011194	0.011397
0.011362	0.011295	0.011194	0.011194	0.011262	0.011262	0.011329
0.011262	0.011329	0.011228	0.011329	0.011262	0.011262	0.011329
0.011262	0.011698	0.011597	0.011631	0.011161	0.011262	0.011295
0.011295	0.011262	0.011329	0.011362	0.011228	0.011228	0.011228
0.011765	0.011396	0.011396	0.011194	0.011295	0.011463	0.011228
0.011228	0.011295	0.011262	0.011429	0.011228	0.011362	0.011496
0.011429	0.011262	0.011262	0.011194	0.011194	0.011362	0.011295
0.011194	0.011295	0.011161	0.011194	0.011329	0.011463	0.011228
0.011195	0.011329	0.011262	0.011161	0.011496	0.011161	0.011329
0.011402	0.011228	0.011530	0.011362	0.011396	0.011262	0.011362
0.011228	0.011564	0.011161	0.011429	0.011228	0.011228	0.011161
0.011396	0.011597	0.011295	0.011362	0.011194	0.011597	0.011228
0.011228	0.011228	0.011262	0.011362	0.011262	0.011362	0.011429
0.011429	0.011396	0.011295	0.011396	0.011362	0.011228	0.011194
0.011228	0.011228	0.011295	0.011228	0.011194	0.011362	0.011228
0.011262	0.011262	0.011396	0.011429	0.011329	0.011262	0.011262
0.011329	0.011262	0.011362	0.011228	0.011362	0.011463	0.011765
0.011262	0.011664	0.011267	0.017785	0.011191	0.011305	0.011618
0.011579	0.011623	0.011270	0.011234	0.011664	0.011647	0.011242
0.011487	0.011426	0.011177	0.011186	0.011255	0.011258	0.011232
0.011482	0.011433	0.011431	0.011325	0.011396	0.011429	0.011432
0.011301	0.011396	0.011396	0.011362	0.011329	0.011329	0.011295
0.011329	0.011262	0.011295	0.011329	0.011295	0.011228	0.011262
0.011262	0.011329	0.011228	0.011329	0.011362	0.011396	0.011295
0.011329	0.011295	0.011631	0.011631	0.011362	0.011396	0.011430
0.011195	0.011195	0.011228	0.011329	0.011195	0.011429	0.011429
0.011463	0.011463	0.011396	0.011496	0.011665	0.011498	0.011329
0.011329	0.011396	0.011429	0.011262	0.011329	0.011295	0.011329
0.011396	0.011396	0.011429	0.011396	0.011296	0.011497	0.011463
0.011331	0.011228	0.011329	0.011430	0.011329	0.011463	0.011496
0.011664	0.011367	0.011530	0.011497	0.011429	0.011597	0.011228

0.011397	0.011295	0.011262	0.011262	0.011329	0.011228	0.011329
0.011362	0.011396	0.011295	0.011329	0.011295	0.011631	0.011631
0.011362	0.011396	0.011463	0.011396	0.012035	0.011453	0.011377
0.011447	0.011407	0.011517	0.011406	0.011515	0.011439	0.011405
0.011987	0.011302	0.011437	0.011371	0.011645	0.011502	0.011604
0.012012	0.011437	0.011567	0.011631	0.011496	0.011497	0.011533
0.011194	0.011362	0.011496	0.011463	0.011466	0.011463	0.011431
0.011295	0.011228	0.011262	0.011295	0.011194	0.011262	0.011161
0.011295	0.011329	0.011228	0.011194	0.011295	0.011228	0.011262
0.011262	0.011194	0.011262	0.011295	0.011194	0.011396	0.011429
0.011329	0.011396	0.011329	0.011329	0.011262	0.011664	0.011295
0.011338	0.011262	0.011497	0.011501	0.011362	0.011362	0.011362
0.011362	0.011295	0.011262	0.011295	0.011396	0.011396	0.011329
0.011329	0.011463	0.011631	0.011362	0.011564	0.011564	0.011429
0.011262	0.011329	0.011362	0.011765	0.011329	0.011362	0.011329
0.011362	0.011295	0.011295	0.011597	0.011530	0.011564	0.011295
0.011396	0.011463	0.011329	0.011396	0.011362	0.011228	0.011564
0.011329	0.011295	0.011295	0.011463	0.011295	0.011396	0.011631
0.011329	0.011705	0.011698	0.011507	0.011229	0.011400	0.011267
0.011115	0.011722	0.011236	0.011462	0.011255	0.011315	0.011379
0.011468	0.011126	0.011339	0.011192	0.011222	0.011395	0.011385
0.011399	0.011296	0.011377	0.011307	0.011330	0.011463	0.011530
0.011530	0.011396	0.011500	0.011501	0.011636	0.011661	0.011329
0.011564	0.011429	0.011295	0.011396	0.011195	0.011429	0.011228
0.011295	0.011295	0.011262	0.011228	0.011262	0.011228	0.011329
0.011305	0.011305	0.011300	0.011334	0.011471	0.011436	0.011436
0.011435	0.011363	0.011171	0.011168	0.011297	0.011467	0.011467
0.011262	0.011331	0.011565	0.011533	0.011464	0.011498	0.011429
0.011362	0.011429	0.011429	0.011329	0.011900	0.011496	0.011564
0.011597	0.011665	0.011299	0.011372	0.011262	0.011463	0.011429
0.011464	0.011496	0.011496	0.011496	0.011610	0.011631	0.011268
0.011162	0.011164	0.011264	0.011230	0.011333	0.011172	0.011502
0.011300	0.011455	0.011203	0.011322	0.011296	0.011368	0.011262
0.011228	0.011228	0.011295	0.011262	0.011463	0.011429	0.011362
0.011463	0.011530	0.011262	0.011262	0.011295	0.011295	0.011297
0.011364	0.011295	0.011295	0.011330	0.011463	0.011530	0.011530
0.011396	0.011500	0.011501	0.011636	0.011661	0.011329	0.011496
0.011429	0.011429	0.011263	0.011443	0.011373	0.011401	0.011270
0.011439	0.011607	0.011441	0.011332	0.011228	0.011430	0.011295
0.011396	0.011329	0.011228	0.011330	0.011430	0.011432	0.011329
0.011228	0.011161	0.011295	0.011228	0.011161	0.011264	0.011430
0.011329	0.011262	0.011295	0.011329	0.011295	0.011228	0.011430
0.011295	0.011329	0.011329	0.000000	0.011329	0.011564	0.011228
0.011262	0.011664	0.000000	0.011295	0.011262	0.011631	0.011189
0.011678	0.011230	0.011261	0.011422	0.011508	0.011455	0.011144
0.011218	0.011429	0.011530	0.011637	0.011498	0.011635	0.011766
0.011666	0.011703	0.011664	0.011914	0.011631	0.012505	0.011666
0.012405	0.012173	0.012307	0.012578	0.011631	0.011329	0.011631
0.012070	0.012004	0.011597	0.011497	0.011565	0.011565	0.011534
0.011429	0.011433	0.011496	0.011464	0.011833	0.011900	0.011901
0.011800	0.011901	0.012004	0.011770	0.012070	0.012076	0.012137
0.012138	0.012171	0.012118	0.012138	0.012139	0.011362	0.011665
0.011733	0.012236	0.011738	0.011967	0.012440	0.011502	0.011396
0.011362	0.011362	0.011833	0.011842	0.011975	0.011698	0.011363
S057	0.011732	0.011732	0.012001	0.011765	0.011765	0.011833
0.011672	0.011604	0.011833	0.011329	0.011396	0.011363	0.011329
0.011295	0.011530	0.011262	0.011362	0.011429	0.011396	0.011362
0.011329	0.011362	0.011396	0.011329	0.011363	0.011362	0.011638
0.011530	0.011362	0.011262	0.011429	0.011429	0.011396	0.011430
0.011396	0.011497	0.011396	0.011396	0.011429	0.011396	0.011564

0.011262	0.011396	0.011429	0.011362	0.000000	0.011497	0.011429
0.011396	0.011564	0.011463	0.011665	0.011396	0.011665	0.011396
0.011362	0.000000	0.011530	0.011665	0.011698	0.011396	0.011497
0.011429	0.011497	0.011396	0.011631	0.011799	0.011396	0.011463
0.011362	0.011463	0.011429	0.011497	0.011429	0.011429	0.011429
0.011329	0.011362	0.011329	0.011362	0.011396	0.011430	0.011497
0.011430	0.011396	0.011463	0.011362	0.011429	0.011329	0.011396
0.011564	0.011497	0.011732	0.011564	0.011396	0.011631	0.011396
0.011580	0.011733	0.011626	0.011334	0.011322	0.011428	0.011454
0.012085	0.011518	0.011349	0.011631	0.011631	0.011362	0.011463
0.011463	0.011429	0.011497	0.011396	0.011497	0.011429	0.011429
0.011396	0.011429	0.011698	0.011564	0.011463	0.011430	0.011363
0.011329	0.011262	0.011396	0.011362	0.011497	0.011463	0.011396
0.011497	0.011564	0.011362	0.011362	0.011396	0.011396	0.011398
0.011466	0.011396	0.011396	0.011276	0.011350	0.011306	0.011396
0.011399	0.011335	0.011392	0.011371	0.011298	0.011334	0.011364
0.011564	0.011405	0.011439	0.011329	0.011337	0.011464	0.011363
0.011430	0.011431	0.011430	0.011530	0.011463	0.011833	0.011564
0.011597	0.011463	0.011396	0.011430	0.011295	0.011463	0.011329
0.011396	0.011396	0.011362	0.011329	0.011362	0.011329	0.011430
0.011396	0.011497	0.011430	0.011329	0.011431	0.011530	0.011533
0.011531	0.011430	0.011329	0.011263	0.011396	0.011329	0.011262
0.011366	0.011531	0.011329	0.011531	0.011295	0.011396	0.011362
0.011463	0.011362	0.011396	0.011363	0.011396	0.011262	0.011396
0.011430	0.011362	0.011430	0.011362	0.011396	0.011430	0.011665
0.011698	0.011631	0.011699	0.011532	0.011699	0.011396	0.011362
0.011331	0.011626	0.011426	0.011300	0.011705	0.011265	0.011414
0.011706	0.011604	0.011564	0.011765	0.011470	0.011631	0.011598
0.011530	0.011698	0.011558	0.011390	0.011479	0.011430	0.011500
0.011598	0.011499	0.011635	0.011703	0.011631	0.011698	0.011806
0.011534	0.011705	0.011636	0.011568	0.011834	0.011295	0.011498
0.011463	0.011396	0.011295	0.011295	0.011362	0.011362	0.011429
0.011362	0.011430	0.011329	0.011362	0.011362	0.011362	0.011429
0.011362	0.011665	0.011631	0.011665	0.011262	0.011362	0.011396
0.011396	0.011362	0.011430	0.011396	0.011329	0.011329	0.011329
0.011799	0.011429	0.011497	0.011295	0.011396	0.011564	0.011329
0.011329	0.011396	0.011362	0.011530	0.011329	0.011463	0.011597
0.011530	0.011362	0.011362	0.011295	0.011295	0.011463	0.011396
0.011295	0.011396	0.011262	0.011295	0.011430	0.011497	0.011329
0.011295	0.011429	0.011363	0.011262	0.011530	0.011262	0.011430
0.011505	0.011329	0.011564	0.011463	0.011430	0.011362	0.011463
0.011329	0.011665	0.011262	0.011463	0.011262	0.011262	0.011262
0.011497	0.011631	0.011396	0.011463	0.011295	0.011631	0.011262
0.011329	0.011329	0.011362	0.011463	0.011362	0.011463	0.011530
0.011530	0.011497	0.011396	0.011497	0.011463	0.011329	0.011295
0.011329	0.011329	0.011396	0.011262	0.011295	0.011463	0.011329
0.011295	0.011362	0.011497	0.011463	0.011429	0.011362	0.011362
0.011429	0.011362	0.011463	0.011329	0.011463	0.011564	0.011799
0.011362	0.011698	0.011371	0.017826	0.011388	0.011427	0.011653
0.011614	0.011658	0.011427	0.011408	0.011720	0.011682	0.011425
0.011535	0.011549	0.011294	0.011353	0.011295	0.011307	0.011361
0.011530	0.011470	0.011468	0.011259	0.011497	0.011530	0.011534
0.011404	0.011497	0.011497	0.011396	0.011430	0.011430	0.011396
0.011430	0.011362	0.011396	0.011430	0.011396	0.011329	0.011362
0.011362	0.011430	0.011329	0.011430	0.011463	0.011497	0.011396
0.011362	0.011329	0.011665	0.011665	0.011463	0.011497	0.011531
0.011295	0.011295	0.011329	0.011430	0.011295	0.011530	0.011530
0.011564	0.011564	0.011497	0.011597	0.011699	0.011599	0.011430
0.011430	0.011497	0.011530	0.011362	0.011430	0.011396	0.011430
0.011497	0.011497	0.011530	0.011430	0.011397	0.011598	0.011564

0.011433	0.011329	0.011430	0.011531	0.011430	0.011497	0.011597
0.011765	0.011470	0.011631	0.011598	0.011530	0.011698	0.011295
0.011498	0.011396	0.011362	0.011362	0.011430	0.011329	0.011430
0.011463	0.011497	0.011396	0.011362	0.011329	0.011665	0.011665
0.011463	0.011497	0.011564	0.011497	0.012070	0.011560	0.011482
0.011552	0.011511	0.011623	0.011511	0.011620	0.011543	0.011509
0.012022	0.011406	0.011540	0.011475	0.011749	0.011604	0.011707
0.012046	0.011512	0.011669	0.011732	0.011597	0.011530	0.011635
0.011295	0.011463	0.011597	0.011564	0.011567	0.011497	0.011464
0.011396	0.011329	0.011362	0.011396	0.011295	0.011362	0.011262
0.011329	0.011362	0.011329	0.011295	0.011396	0.011329	0.011362
0.011362	0.011295	0.011362	0.011396	0.011295	0.011497	0.011530
0.011430	0.011430	0.011429	0.011430	0.011362	0.011698	0.011396
0.011373	0.011362	0.011531	0.011535	0.011463	0.011463	0.011463
0.011463	0.011396	0.011362	0.011396	0.011497	0.011497	0.011429
0.011429	0.011564	0.011665	0.011463	0.011665	0.011665	0.011530
0.011362	0.011429	0.011463	0.011799	0.011430	0.011463	0.011430
0.011463	0.011329	0.011329	0.011631	0.011631	0.011598	0.011396
0.011497	0.011564	0.011429	0.011497	0.011463	0.011329	0.011597
0.011429	0.011396	0.011329	0.011564	0.011396	0.011497	0.011732
0.011429	0.011739	0.011732	0.011548	0.011286	0.011434	0.011370
0.011225	0.011761	0.011341	0.011507	0.011353	0.011518	0.011432
0.011519	0.011323	0.011506	0.011325	0.011438	0.011529	0.011506
0.011512	0.011414	0.011413	0.011413	0.011431	0.011497	0.011564
0.011564	0.011430	0.011534	0.011535	0.011671	0.011697	0.011430
0.011597	0.011463	0.011396	0.011430	0.011295	0.011463	0.011329
0.011396	0.011396	0.011362	0.011329	0.011362	0.011329	0.011430
0.011410	0.011410	0.011403	0.011437	0.011574	0.011539	0.011538
0.011537	0.011464	0.011282	0.011276	0.011399	0.011569	0.011569
0.011363	0.011433	0.011666	0.011635	0.011565	0.011599	0.011530
0.011463	0.011530	0.011530	0.011430	0.011934	0.011597	0.011665
0.011699	0.011701	0.011401	0.011441	0.011362	0.011564	0.011530
0.011565	0.011597	0.011597	0.011597	0.011768	0.011732	0.011372
0.011263	0.011268	0.011367	0.011332	0.011368	0.011284	0.011536
0.011403	0.011499	0.011298	0.011435	0.011397	0.011471	0.011363
0.011329	0.011262	0.011396	0.011362	0.011497	0.011463	0.011396
0.011497	0.011564	0.011362	0.011362	0.011396	0.011396	0.011398
0.011466	0.011396	0.011396	0.011431	0.011497	0.011564	0.011564
0.011430	0.011534	0.011535	0.011671	0.011697	0.011430	0.011597
0.011530	0.011530	0.011365	0.011547	0.011477	0.011503	0.011341
0.011542	0.011698	0.011526	0.011434	0.011329	0.011531	0.011396
0.011497	0.011430	0.011329	0.011431	0.011530	0.011533	0.011430
0.011329	0.011263	0.011396	0.011329	0.011262	0.011366	0.011531
0.011430	0.011362	0.011396	0.011430	0.011396	0.011329	0.011463
0.011396	0.011430	0.011429	0.011362	0.011429	0.011665	0.011262
0.011362	0.011698	0.011362	0.011396	0.011362	0.011665	0.011317
0.011679	0.011358	0.011311	0.011503	0.011590	0.011491	0.011269
0.011270	0.011463	0.011564	0.011672	0.011532	0.011669	0.011800
0.011700	0.011737	0.011698	0.011949	0.011665	0.012540	0.011701
0.012440	0.012207	0.012341	0.012613	0.011665	0.011363	0.011665
0.012104	0.012039	0.011631	0.011530	0.011598	0.011598	0.011568
0.011463	0.011467	0.011530	0.011497	0.011868	0.011934	0.011935
0.011834	0.011935	0.012038	0.011805	0.012037	0.012110	0.012171
0.012172	0.012206	0.012153	0.012172	0.012174	0.011463	0.011699
0.011767	0.012270	0.011772	0.012001	0.012474	0.011536	0.011430
0.011396	0.011396	0.011867	0.011924	0.012010	0.011732	0.011397
S058	0.011799	0.011799	0.012102	0.011866	0.011866	0.011933
0.011807	0.011739	0.011933	0.011396	0.011463	0.011430	0.011396
0.011429	0.011597	0.011396	0.011429	0.011496	0.011463	0.011429
0.011396	0.011429	0.011530	0.011396	0.011430	0.011429	0.011773

0.011597	0.011429	0.011329	0.011564	0.011497	0.011463	0.011497
0.011463	0.011564	0.011396	0.011463	0.011362	0.011463	0.011631
0.011396	0.011463	0.011362	0.011362	0.011530	0.011564	0.011497
0.011463	0.011497	0.011530	0.011799	0.011329	0.011732	0.011329
0.011429	0.011530	0.000000	0.011732	0.011832	0.011530	0.011631
0.011496	0.011564	0.011463	0.011698	0.011933	0.011463	0.011530
0.011429	0.011530	0.011497	0.011564	0.011496	0.011429	0.011429
0.011396	0.011429	0.011396	0.011497	0.011463	0.011498	0.011564
0.011497	0.011463	0.011530	0.011429	0.011564	0.011396	0.011463
0.011631	0.011564	0.011799	0.011631	0.011463	0.011698	0.011396
0.011542	0.011770	0.011626	0.011334	0.011359	0.011465	0.011493
0.012195	0.011569	0.011307	0.011698	0.011698	0.011496	0.011597
0.011530	0.011496	0.011564	0.011463	0.011497	0.011497	0.011496
0.011463	0.011564	0.011765	0.011631	0.011530	0.011497	0.011430
0.011396	0.011396	0.011463	0.011430	0.011631	0.011597	0.011530
0.011631	0.011698	0.011429	0.011429	0.011463	0.011463	0.011465
0.011533	0.011463	0.011463	0.011348	0.011422	0.011376	0.011464
0.011467	0.011404	0.011429	0.011440	0.011366	0.011403	0.011432
0.011708	0.011474	0.011508	0.011396	0.011406	0.011531	0.011497
0.011565	0.011498	0.011497	0.011597	0.011530	0.011900	0.011698
0.011731	0.011598	0.011463	0.011564	0.011362	0.011597	0.011396
0.011463	0.011463	0.011430	0.011396	0.011429	0.011396	0.011497
0.011463	0.011564	0.011497	0.011396	0.011498	0.011598	0.011601
0.011598	0.011497	0.011396	0.011330	0.011463	0.011396	0.011329
0.011434	0.011598	0.011396	0.011598	0.011362	0.011463	0.011430
0.011530	0.011429	0.011463	0.011430	0.011463	0.011329	0.011463
0.011497	0.011430	0.011497	0.011429	0.011463	0.011497	0.011732
0.011833	0.011765	0.011833	0.011431	0.011833	0.011463	0.011430
0.011466	0.011707	0.011609	0.011335	0.011841	0.011333	0.011375
0.011841	0.011740	0.011631	0.011833	0.011538	0.011698	0.011665
0.011597	0.011765	0.011593	0.011390	0.011479	0.011497	0.011568
0.011665	0.011566	0.011703	0.011771	0.011698	0.011765	0.011873
0.011602	0.011739	0.011703	0.011635	0.011901	0.011362	0.011566
0.011530	0.011429	0.011295	0.011362	0.011429	0.011429	0.011429
0.011429	0.011497	0.011396	0.011497	0.011429	0.011429	0.011496
0.011429	0.011866	0.011765	0.011799	0.011329	0.011429	0.011463
0.011463	0.011429	0.011497	0.011530	0.011396	0.011396	0.011396
0.011933	0.011564	0.011564	0.011362	0.011463	0.011631	0.011396
0.011396	0.011463	0.011429	0.011597	0.011396	0.011530	0.011664
0.011597	0.011429	0.011429	0.011362	0.011362	0.011530	0.011463
0.011362	0.011463	0.011329	0.011362	0.011497	0.011631	0.011396
0.011329	0.011497	0.011430	0.011329	0.011664	0.011329	0.011497
0.011540	0.011396	0.011698	0.011530	0.011564	0.011429	0.011396
0.011396	0.011732	0.011329	0.011597	0.011396	0.011396	0.011329
0.011564	0.011732	0.011463	0.011530	0.011362	0.011765	0.011396
0.011396	0.011396	0.011429	0.011530	0.011429	0.011530	0.011597
0.011597	0.011564	0.011396	0.011564	0.011530	0.011329	0.011362
0.011396	0.011329	0.011463	0.011396	0.011362	0.011530	0.011396
0.011429	0.011429	0.011564	0.011597	0.011496	0.011429	0.011430
0.011497	0.011429	0.011530	0.011396	0.011530	0.011631	0.011933
0.011429	0.011832	0.011405	0.017937	0.011387	0.011672	0.011758
0.011718	0.011764	0.011479	0.011408	0.011884	0.011785	0.011546
0.011629	0.011732	0.011255	0.011395	0.011412	0.011455	0.011404
0.011623	0.011582	0.011579	0.011193	0.011564	0.011597	0.011601
0.011438	0.011564	0.011564	0.011530	0.011497	0.011497	0.011463
0.011497	0.011429	0.011463	0.011497	0.011463	0.011396	0.011429
0.011429	0.011497	0.011396	0.011497	0.011530	0.011564	0.011463
0.011497	0.011463	0.011799	0.011799	0.011530	0.011565	0.011598
0.011362	0.011362	0.011396	0.011497	0.011362	0.011597	0.011597
0.011631	0.011631	0.011564	0.011665	0.011833	0.011666	0.011497

0.011497	0.011564	0.011598	0.011430	0.011497	0.011463	0.011497
0.011564	0.011564	0.011597	0.011564	0.011464	0.011665	0.011631
0.011500	0.011396	0.011497	0.011531	0.011497	0.011631	0.011664
0.011833	0.011538	0.011698	0.011665	0.011597	0.011765	0.011396
0.011566	0.011464	0.011429	0.011429	0.011497	0.011396	0.011497
0.011530	0.011564	0.011463	0.011497	0.011463	0.011799	0.011799
0.011530	0.011565	0.011631	0.011564	0.012204	0.011560	0.011482
0.011552	0.011511	0.011658	0.011545	0.011620	0.011543	0.011577
0.012125	0.011406	0.011540	0.011475	0.011749	0.011672	0.011775
0.012182	0.011588	0.011736	0.011799	0.011664	0.011664	0.011702
0.011362	0.011530	0.011664	0.011631	0.011635	0.011631	0.011599
0.011463	0.011396	0.011429	0.011463	0.011362	0.011429	0.011329
0.011463	0.011496	0.011396	0.011362	0.011463	0.011396	0.011429
0.011429	0.011362	0.011429	0.011463	0.011362	0.011564	0.011597
0.011497	0.011564	0.011497	0.011497	0.011429	0.011832	0.011463
0.011478	0.011429	0.011598	0.011569	0.011530	0.011463	0.011530
0.011530	0.011463	0.011429	0.011463	0.011564	0.011564	0.011497
0.011496	0.011631	0.011799	0.011530	0.011732	0.011732	0.011597
0.011429	0.011497	0.011530	0.011933	0.011497	0.011530	0.011497
0.011530	0.011463	0.011463	0.011765	0.011698	0.011732	0.011463
0.011564	0.011631	0.011496	0.011564	0.011530	0.011396	0.011732
0.011496	0.011463	0.011463	0.011631	0.011463	0.011564	0.011799
0.011496	0.011875	0.011866	0.011589	0.011399	0.011570	0.011267
0.011445	0.011878	0.011376	0.011551	0.011353	0.011366	0.011485
0.011621	0.011258	0.011394	0.011258	0.011330	0.011529	0.011425
0.011475	0.011414	0.011518	0.011448	0.011498	0.011631	0.011698
0.011698	0.011565	0.011635	0.011670	0.011806	0.011838	0.011497
0.011731	0.011598	0.011463	0.011564	0.011362	0.011597	0.011396
0.011463	0.011463	0.011430	0.011396	0.011429	0.011396	0.011497
0.011479	0.011480	0.011471	0.011505	0.011642	0.011607	0.011607
0.011605	0.011531	0.011319	0.011348	0.011467	0.011636	0.011637
0.011430	0.011501	0.011734	0.011703	0.011632	0.011666	0.011597
0.011530	0.011597	0.011597	0.011497	0.012068	0.011664	0.011732
0.011766	0.011844	0.011469	0.011545	0.011396	0.011631	0.011597
0.011632	0.011664	0.011664	0.011664	0.011768	0.011799	0.011407
0.011297	0.011303	0.011401	0.011366	0.011470	0.011321	0.011638
0.011437	0.011586	0.011346	0.011435	0.011464	0.011539	0.011430
0.011396	0.011396	0.011463	0.011430	0.011631	0.011597	0.011530
0.011631	0.011698	0.011429	0.011429	0.011463	0.011463	0.011465
0.011533	0.011463	0.011463	0.011498	0.011631	0.011698	0.011698
0.011565	0.011635	0.011670	0.011806	0.011838	0.011497	0.011664
0.011597	0.011597	0.011365	0.011617	0.011547	0.011537	0.011411
0.011611	0.011744	0.011568	0.011502	0.011396	0.011598	0.011463
0.011564	0.011497	0.011396	0.011498	0.011598	0.011601	0.011497
0.011396	0.011330	0.011463	0.011396	0.011329	0.011434	0.011598
0.011497	0.011429	0.011463	0.011497	0.011463	0.011396	0.011597
0.011463	0.011497	0.011496	0.011429	0.011497	0.011732	0.011396
0.011429	0.011832	0.011429	0.011463	0.011429	0.011799	0.011360
0.011847	0.011315	0.011411	0.011543	0.011630	0.011599	0.011353
0.011375	0.011597	0.011698	0.011807	0.011666	0.011771	0.011935
0.011834	0.011838	0.011832	0.012051	0.011765	0.012640	0.011835
0.012540	0.012308	0.012442	0.012748	0.011799	0.011430	0.011799
0.012238	0.012140	0.011765	0.011665	0.011733	0.011733	0.011703
0.011597	0.011569	0.011664	0.011632	0.012002	0.012068	0.012069
0.011969	0.012069	0.012173	0.011906	0.012239	0.012246	0.012306
0.012307	0.012340	0.012255	0.012307	0.012308	0.011530	0.011833
0.011901	0.012404	0.011908	0.012135	0.012609	0.011672	0.011564
0.011530	0.011530	0.012001	0.012006	0.012151	0.011866	0.011532
S059	0.011933	0.011933	0.012270	0.012034	0.012034	0.012101
0.011909	0.011841	0.012101	0.011530	0.011597	0.011565	0.011530

0.011564	0.011732	0.011530	0.011564	0.011631	0.011597	0.011564
0.011530	0.011564	0.011664	0.011531	0.011497	0.011564	0.011875
0.011799	0.011564	0.011463	0.011698	0.011564	0.011597	0.011564
0.011597	0.011631	0.011597	0.011597	0.011631	0.011597	0.011765
0.011530	0.011597	0.011631	0.011564	0.011664	0.011698	0.011631
0.011597	0.011765	0.011665	0.011933	0.011597	0.000000	0.011597
0.011564	0.011665	0.011732	0.000000	0.011967	0.011664	0.011765
0.011564	0.011631	0.011597	0.011765	0.012068	0.011597	0.011664
0.011564	0.011664	0.011631	0.011698	0.011631	0.011631	0.011631
0.011530	0.011564	0.011530	0.011631	0.011597	0.011632	0.011698
0.011631	0.011597	0.011597	0.011564	0.011698	0.011463	0.011530
0.011765	0.011698	0.011933	0.011765	0.011597	0.011765	0.011597
0.011765	0.011994	0.011777	0.011451	0.011508	0.011649	0.011690
0.012341	0.011670	0.011431	0.011832	0.011832	0.011631	0.011732
0.011664	0.011631	0.011698	0.011597	0.011698	0.011564	0.011631
0.011597	0.011698	0.011900	0.011765	0.011664	0.011631	0.011497
0.011530	0.011530	0.011530	0.011564	0.011765	0.011732	0.011665
0.011765	0.011833	0.011564	0.011564	0.011597	0.011597	0.011601
0.011669	0.011597	0.011597	0.011456	0.011530	0.011516	0.011598
0.011602	0.011541	0.011577	0.011578	0.011502	0.011540	0.011567
0.011852	0.011612	0.011577	0.011531	0.011544	0.011666	0.011632
0.011699	0.011633	0.011632	0.011731	0.011664	0.011967	0.011833
0.011866	0.011732	0.011597	0.011698	0.011497	0.011732	0.011530
0.011597	0.011597	0.011564	0.011530	0.011564	0.011530	0.011631
0.011530	0.011631	0.011631	0.011530	0.011633	0.011732	0.011736
0.011665	0.011631	0.011530	0.011465	0.011597	0.011530	0.011463
0.011570	0.011665	0.011463	0.011665	0.011429	0.011598	0.011497
0.011597	0.011564	0.011530	0.011497	0.011530	0.011463	0.011530
0.011564	0.011564	0.011631	0.011564	0.011597	0.011631	0.011866
0.011967	0.011900	0.011967	0.011734	0.011967	0.011597	0.011564
0.011601	0.011909	0.011609	0.011472	0.011976	0.011469	0.011611
0.011943	0.011876	0.011765	0.011967	0.011675	0.011832	0.011732
0.011732	0.011900	0.011771	0.011637	0.011689	0.011631	0.011703
0.011799	0.011701	0.011838	0.011601	0.011832	0.011900	0.012009
0.011737	0.011603	0.011839	0.011770	0.012036	0.011496	0.011700
0.011597	0.011597	0.011496	0.011496	0.011496	0.011564	0.011631
0.011496	0.011564	0.011463	0.011631	0.011496	0.011564	0.011631
0.011564	0.012000	0.011900	0.011933	0.011463	0.011564	0.011597
0.011597	0.011496	0.011631	0.011664	0.011530	0.011530	0.011530
0.012068	0.011698	0.011698	0.011496	0.011597	0.011698	0.011530
0.011530	0.011597	0.011564	0.011664	0.011530	0.011664	0.011731
0.011731	0.011564	0.011564	0.011496	0.011496	0.011664	0.011597
0.011496	0.011597	0.011463	0.011496	0.011631	0.011766	0.011530
0.011497	0.011631	0.011564	0.011463	0.011799	0.011463	0.011631
0.011642	0.011530	0.011832	0.011665	0.011698	0.011564	0.011664
0.011530	0.000000	0.011463	0.011732	0.011530	0.011530	0.011463
0.011631	0.011900	0.011597	0.011664	0.011496	0.011900	0.011530
0.011530	0.011530	0.011564	0.011597	0.011564	0.011664	0.011731
0.011732	0.011631	0.011597	0.011698	0.011597	0.011530	0.011496
0.011530	0.011530	0.011597	0.011530	0.011429	0.011664	0.011530
0.011564	0.011564	0.011698	0.011732	0.011631	0.011496	0.011564
0.011631	0.011564	0.011664	0.011530	0.011664	0.011765	0.012068
0.011564	0.011967	0.011578	0.018087	0.011518	0.011733	0.011932
0.011891	0.011940	0.011532	0.011466	0.011939	0.011958	0.011485
0.011677	0.011793	0.011450	0.011479	0.011450	0.011554	0.011576
0.011670	0.011731	0.011690	0.011523	0.011698	0.011732	0.011737
0.011541	0.011698	0.011698	0.011665	0.011631	0.011631	0.011598
0.011631	0.011564	0.011530	0.011631	0.011598	0.011530	0.011564
0.011564	0.011631	0.011530	0.011631	0.011665	0.011698	0.011597
0.011631	0.011597	0.011934	0.011934	0.011665	0.011699	0.011732

0.011497	0.011497	0.011530	0.011564	0.011497	0.011732	0.011732
0.011765	0.011765	0.011698	0.011799	0.011967	0.011801	0.011564
0.011631	0.011698	0.011665	0.011564	0.011631	0.011597	0.011631
0.011699	0.011698	0.011732	0.011631	0.011464	0.011799	0.011698
0.011636	0.011530	0.011631	0.011732	0.011631	0.011766	0.011799
0.011967	0.011675	0.011832	0.011732	0.011732	0.011900	0.011531
0.011700	0.011598	0.011564	0.011564	0.011631	0.011530	0.011631
0.011665	0.011698	0.011597	0.011631	0.011597	0.011934	0.011934
0.011665	0.011699	0.011765	0.011698	0.012339	0.011775	0.011693
0.011763	0.011719	0.011833	0.011718	0.011830	0.011750	0.011715
0.012297	0.011614	0.011745	0.011683	0.011956	0.011808	0.011638
0.012318	0.011814	0.011872	0.011933	0.011799	0.011799	0.011837
0.011496	0.011597	0.011731	0.011698	0.011770	0.011765	0.011734
0.011597	0.011463	0.011496	0.011530	0.011429	0.011564	0.011463
0.011597	0.011631	0.011530	0.011496	0.011597	0.011530	0.011564
0.011564	0.011496	0.011564	0.011597	0.011496	0.011631	0.011731
0.011631	0.011698	0.011631	0.011631	0.011564	0.011967	0.011597
0.011652	0.011564	0.011799	0.011807	0.011597	0.011664	0.011664
0.011664	0.011530	0.011564	0.011530	0.011698	0.011698	0.011631
0.011631	0.011765	0.011933	0.011597	0.011866	0.011866	0.011731
0.011564	0.011631	0.011664	0.012068	0.011631	0.011597	0.011631
0.011664	0.011597	0.011597	0.011832	0.011765	0.011799	0.011597
0.011698	0.011765	0.011631	0.011698	0.011664	0.011530	0.011866
0.011631	0.011597	0.011597	0.011765	0.011597	0.011698	0.011933
0.011631	0.011977	0.012000	0.011792	0.011455	0.011706	0.011421
0.011500	0.011956	0.011552	0.011596	0.011499	0.011671	0.011591
0.011723	0.011389	0.011561	0.011324	0.011437	0.011529	0.011628
0.011663	0.011493	0.011694	0.011624	0.011633	0.011765	0.011833
0.011833	0.011699	0.011804	0.011806	0.011942	0.011979	0.011631
0.011866	0.011732	0.011597	0.011698	0.011497	0.011732	0.011530
0.011597	0.011597	0.011564	0.011530	0.011564	0.011530	0.011631
0.011619	0.011620	0.011608	0.011642	0.011779	0.011675	0.011743
0.011742	0.011665	0.011503	0.011492	0.011602	0.011772	0.011773
0.011565	0.011636	0.011868	0.011838	0.011767	0.011801	0.011731
0.011664	0.011732	0.011731	0.011631	0.012202	0.011732	0.011866
0.011900	0.011915	0.011606	0.011684	0.011564	0.011765	0.011732
0.011767	0.011799	0.011799	0.011799	0.011925	0.011933	0.011581
0.011466	0.011476	0.011571	0.011536	0.011641	0.011508	0.011808
0.011609	0.011674	0.011441	0.011622	0.011598	0.011676	0.011497
0.011530	0.011530	0.011530	0.011564	0.011765	0.011732	0.011665
0.011765	0.011833	0.011564	0.011564	0.011597	0.011597	0.011601
0.011669	0.011597	0.011597	0.011633	0.011765	0.011833	0.011833
0.011699	0.011804	0.011806	0.011942	0.011979	0.011631	0.011799
0.011732	0.011732	0.011567	0.011756	0.011651	0.011707	0.011516
0.011646	0.011926	0.011695	0.011638	0.011463	0.011665	0.011530
0.011631	0.011631	0.011530	0.011633	0.011732	0.011736	0.011631
0.011530	0.011465	0.011597	0.011530	0.011463	0.011570	0.011665
0.011631	0.011564	0.011530	0.011631	0.011598	0.011530	0.011732
0.011597	0.011631	0.011631	0.011564	0.011631	0.011866	0.011530
0.011564	0.011967	0.011564	0.011530	0.011564	0.011933	0.011446
0.011960	0.011485	0.011461	0.011745	0.011793	0.011778	0.011395
0.011427	0.011731	0.011832	0.011943	0.011801	0.011872	0.012069
0.011969	0.012007	0.011967	0.012188	0.011933	0.012809	0.011970
0.012709	0.012477	0.012611	0.012883	0.011933	0.011631	0.011934
0.012373	0.012309	0.011900	0.011799	0.011867	0.011867	0.011838
0.011731	0.011739	0.011799	0.011766	0.012137	0.012202	0.012204
0.012103	0.012204	0.012308	0.012075	0.012374	0.012381	0.012441
0.012442	0.012475	0.012426	0.012442	0.012443	0.011664	0.011968
0.012036	0.012539	0.012010	0.012203	0.012676	0.011807	0.011699
0.011664	0.011664	0.012136	0.012254	0.012292	0.012000	0.011667

S060	0.012034	0.012034	0.012102	0.011866	0.011866	0.011933
0.011434	0.011366	0.011933	0.011631	0.011698	0.011666	0.011631
0.011597	0.011832	0.011564	0.011664	0.011732	0.011698	0.011664
0.011631	0.011664	0.011497	0.011631	0.011665	0.011664	0.011400
0.011631	0.011664	0.011564	0.011530	0.011732	0.011698	0.011732
0.011698	0.011799	0.011698	0.011698	0.011731	0.011698	0.011866
0.011564	0.011698	0.011732	0.011664	0.011698	0.011799	0.011732
0.011698	0.011866	0.011766	0.011429	0.011698	0.011967	0.011698
0.011664	0.011698	0.011832	0.011967	0.000000	0.011698	0.011799
0.011731	0.011799	0.011698	0.011933	0.011564	0.011698	0.011765
0.011664	0.011765	0.011732	0.011799	0.011731	0.011732	0.011732
0.011631	0.011664	0.011631	0.011664	0.011698	0.011733	0.011799
0.011732	0.011698	0.011765	0.011664	0.011597	0.011631	0.011698
0.011866	0.011799	0.011564	0.011866	0.011698	0.011933	0.011698
0.011913	0.011471	0.011928	0.011646	0.011620	0.011760	0.011533
0.012121	0.011467	0.011637	0.011933	0.011933	0.011664	0.011564
0.011765	0.011731	0.011799	0.011698	0.011799	0.011732	0.011731
0.011698	0.011731	0.012000	0.011866	0.011765	0.011732	0.011665
0.011631	0.011564	0.011698	0.011665	0.011598	0.011564	0.011497
0.011598	0.011665	0.011665	0.011665	0.011698	0.011698	0.011702
0.011770	0.011698	0.011698	0.011528	0.011603	0.011551	0.011699
0.011670	0.011610	0.011687	0.011647	0.011570	0.011574	0.011634
0.011528	0.011646	0.011680	0.011632	0.011613	0.011767	0.011666
0.011733	0.011734	0.011733	0.011832	0.011765	0.012135	0.011867
0.011698	0.011766	0.011698	0.011732	0.011597	0.011564	0.011631
0.011698	0.011698	0.011665	0.011631	0.011664	0.011631	0.011732
0.011698	0.011799	0.011732	0.011631	0.011734	0.011833	0.011838
0.011833	0.011732	0.011631	0.011566	0.011698	0.011631	0.011564
0.011570	0.011833	0.011631	0.011833	0.011597	0.011698	0.011665
0.011766	0.011665	0.011698	0.011665	0.011698	0.011564	0.011698
0.011732	0.011665	0.011732	0.011665	0.011698	0.011732	0.011967
0.011463	0.011396	0.011463	0.011836	0.011396	0.011698	0.011665
0.011634	0.011869	0.011701	0.011609	0.011467	0.011572	0.011769
0.011468	0.011638	0.011866	0.012068	0.011777	0.011933	0.011900
0.011832	0.012000	0.011842	0.011761	0.011689	0.011732	0.011805
0.011900	0.011802	0.011939	0.012008	0.011933	0.012001	0.012111
0.011839	0.012011	0.011839	0.011838	0.012137	0.011597	0.011802
0.011765	0.011698	0.011597	0.011597	0.011664	0.011664	0.011731
0.011664	0.011732	0.011631	0.011664	0.011664	0.011664	0.011731
0.011664	0.011496	0.011396	0.011429	0.011564	0.011664	0.011698
0.011698	0.011597	0.011732	0.011698	0.011631	0.011631	0.011631
0.011564	0.011732	0.011799	0.011597	0.011698	0.011866	0.011631
0.011631	0.011698	0.011664	0.011832	0.011631	0.011564	0.011698
0.011631	0.011664	0.011664	0.011597	0.011597	0.011765	0.011698
0.011597	0.011698	0.011564	0.011597	0.011732	0.011531	0.011631
0.011598	0.011732	0.011665	0.011564	0.011262	0.011564	0.011732
0.011814	0.011631	0.011329	0.011765	0.011732	0.011664	0.011765
0.011631	0.011967	0.011564	0.011631	0.011564	0.011564	0.011564
0.011799	0.011665	0.011698	0.011765	0.011597	0.011396	0.011564
0.011631	0.011631	0.011664	0.011765	0.011664	0.011765	0.011832
0.011832	0.011799	0.011698	0.011799	0.011765	0.011564	0.011597
0.011631	0.011564	0.011698	0.011564	0.011597	0.011765	0.011631
0.011597	0.011664	0.011799	0.011765	0.011732	0.011664	0.011665
0.011732	0.011664	0.011765	0.011631	0.011765	0.011866	0.011362
0.011664	0.011329	0.011681	0.018201	0.011518	0.011855	0.011409
0.011372	0.011412	0.011899	0.011640	0.011610	0.011440	0.011850
0.011677	0.011671	0.011606	0.011688	0.011529	0.011751	0.011663
0.011389	0.011544	0.011357	0.011391	0.011799	0.011833	0.011838
0.011713	0.011799	0.011799	0.011698	0.011732	0.011732	0.011699
0.011732	0.011664	0.011698	0.011732	0.011698	0.011631	0.011665

0.011665	0.011732	0.011631	0.011732	0.011765	0.011799	0.011698
0.011664	0.011564	0.011362	0.011362	0.011766	0.011800	0.011833
0.011597	0.011597	0.011631	0.011732	0.011597	0.011833	0.011833
0.011866	0.011866	0.011799	0.011900	0.011396	0.011902	0.011732
0.011732	0.011799	0.011833	0.011665	0.011732	0.011698	0.011732
0.011800	0.011799	0.011833	0.011799	0.011700	0.011900	0.011866
0.011738	0.011631	0.011732	0.011799	0.011732	0.011799	0.011899
0.012068	0.011777	0.011933	0.011900	0.011832	0.012000	0.011631
0.011802	0.011699	0.011665	0.011665	0.011732	0.011631	0.011732
0.011765	0.011799	0.011698	0.011664	0.011564	0.011362	0.011362
0.011766	0.011800	0.011866	0.011799	0.012373	0.011846	0.011799
0.011834	0.011788	0.011904	0.011822	0.011900	0.011819	0.011750
0.012297	0.011683	0.011814	0.011752	0.012025	0.011877	0.012013
0.012353	0.011777	0.011973	0.012034	0.011900	0.011631	0.011939
0.011597	0.011765	0.011899	0.011866	0.011871	0.011597	0.011566
0.011698	0.011631	0.011664	0.011698	0.011597	0.011664	0.011564
0.011631	0.011664	0.011631	0.011597	0.011698	0.011631	0.011664
0.011664	0.011597	0.011664	0.011698	0.011597	0.011799	0.011832
0.011732	0.011732	0.011732	0.011732	0.011664	0.000000	0.011698
0.011478	0.011665	0.011631	0.011637	0.011698	0.011698	0.011765
0.011698	0.011698	0.011664	0.011698	0.011799	0.011799	0.011732
0.011731	0.011866	0.011429	0.011765	0.011967	0.011967	0.011832
0.011664	0.011732	0.011698	0.011496	0.011732	0.011698	0.011732
0.011765	0.011631	0.011631	0.011934	0.011933	0.011900	0.011698
0.011799	0.011866	0.011732	0.011799	0.011765	0.011631	0.011362
0.011731	0.011698	0.011631	0.011866	0.011698	0.011799	0.011631
0.011731	0.011501	0.011496	0.011629	0.011399	0.011740	0.011473
0.011664	0.011800	0.011657	0.011775	0.011646	0.011569	0.011538
0.011570	0.011455	0.011617	0.011523	0.011491	0.011730	0.011831
0.011814	0.011769	0.011730	0.011730	0.011734	0.011598	0.011665
0.011665	0.011733	0.011838	0.011637	0.011976	0.012015	0.011732
0.011698	0.011766	0.011698	0.011732	0.011597	0.011564	0.011631
0.011698	0.011698	0.011665	0.011631	0.011664	0.011631	0.011732
0.011654	0.011655	0.011677	0.011677	0.011813	0.011812	0.011709
0.011776	0.011766	0.011540	0.011492	0.011670	0.011840	0.011841
0.011666	0.011704	0.011969	0.011939	0.011868	0.011902	0.011832
0.011765	0.011832	0.011832	0.011732	0.011631	0.011900	0.011967
0.012001	0.011416	0.011640	0.011788	0.011664	0.011866	0.011832
0.011868	0.011899	0.011900	0.011900	0.011965	0.012034	0.011685
0.011567	0.011580	0.011673	0.011638	0.011641	0.011545	0.011298
0.011712	0.011411	0.011584	0.011660	0.011699	0.011710	0.011665
0.011631	0.011564	0.011698	0.011665	0.011598	0.011564	0.011497
0.011598	0.011665	0.011665	0.011665	0.011698	0.011698	0.011702
0.011770	0.011698	0.011698	0.011734	0.011598	0.011665	0.011665
0.011733	0.011838	0.011637	0.011976	0.012015	0.011732	0.011900
0.011832	0.011832	0.011669	0.011861	0.011790	0.011809	0.011691
0.011852	0.012017	0.011865	0.011740	0.011631	0.011833	0.011698
0.011799	0.011732	0.011631	0.011734	0.011833	0.011838	0.011732
0.011631	0.011566	0.011698	0.011631	0.011564	0.011570	0.011833
0.011732	0.011664	0.011698	0.011732	0.011698	0.011631	0.011564
0.011698	0.011732	0.011731	0.011664	0.011732	0.011967	0.011564
0.011664	0.000000	0.011664	0.011698	0.011664	0.011429	0.011660
0.011848	0.011443	0.011461	0.011503	0.011630	0.011599	0.011436
0.011428	0.011564	0.011665	0.011774	0.011633	0.011771	0.011901
0.011801	0.011838	0.011799	0.012017	0.011765	0.012641	0.011802
0.012541	0.012309	0.012443	0.012715	0.011698	0.011665	0.011766
0.012407	0.012140	0.011732	0.011631	0.011699	0.011699	0.011669
0.011564	0.011569	0.011631	0.011598	0.011969	0.012035	0.012036
0.011935	0.012036	0.012140	0.011906	0.012408	0.012415	0.012475
0.012475	0.012509	0.012460	0.012476	0.012477	0.011765	0.012002

0.012070	0.012573	0.012078	0.012035	0.012508	0.011570	0.011464
0.011362	0.011429	0.011968	0.011924	0.012081	0.011765	0.011498
S061	0.011732	0.011732	0.012001	0.011765	0.011765	0.011832
0.011671	0.011603	0.011832	0.011329	0.011396	0.011363	0.011329
0.011295	0.011530	0.011262	0.011362	0.011429	0.011396	0.011362
0.011329	0.011362	0.011396	0.011329	0.011363	0.011362	0.011637
0.011530	0.011362	0.011262	0.011429	0.011429	0.011396	0.011429
0.011396	0.011497	0.011396	0.011396	0.011429	0.011396	0.011564
0.011262	0.011396	0.011429	0.011362	0.011396	0.011496	0.011429
0.011396	0.011564	0.011463	0.011664	0.011396	0.011664	0.011396
0.011362	0.011396	0.011530	0.011664	0.011698	0.000000	0.011497
0.011429	0.011497	0.011396	0.011631	0.011799	0.011396	0.011463
0.011362	0.011463	0.011429	0.011497	0.011429	0.011429	0.011429
0.011329	0.011362	0.011329	0.011362	0.011396	0.011430	0.011496
0.011429	0.011396	0.011463	0.011362	0.011429	0.011329	0.011396
0.011564	0.011496	0.011732	0.011564	0.011396	0.011631	0.011396
0.011579	0.011733	0.011626	0.011295	0.011321	0.011428	0.011454
0.012048	0.011365	0.011266	0.011631	0.011631	0.011362	0.011463
0.011463	0.011429	0.011496	0.011396	0.011497	0.011429	0.011429
0.011396	0.011429	0.011698	0.011564	0.011463	0.011430	0.011362
0.011329	0.011262	0.011396	0.011362	0.011497	0.011463	0.011396
0.011497	0.011564	0.011362	0.011362	0.011396	0.011396	0.011398
0.011466	0.011396	0.011396	0.011240	0.011313	0.011271	0.011396
0.011399	0.011335	0.011355	0.011371	0.011298	0.011334	0.011364
0.011528	0.011370	0.011439	0.011329	0.011337	0.011464	0.011363
0.011430	0.011431	0.011430	0.011530	0.011463	0.011832	0.011564
0.011597	0.011463	0.011396	0.011430	0.011295	0.011463	0.011329
0.011329	0.011396	0.011362	0.011329	0.011362	0.011329	0.011430
0.011396	0.011497	0.011430	0.011329	0.011431	0.011530	0.011533
0.011530	0.011430	0.011329	0.011263	0.011396	0.011329	0.011262
0.011366	0.011531	0.011329	0.011530	0.011295	0.011396	0.011362
0.011463	0.011362	0.011396	0.011362	0.011396	0.011262	0.011396
0.011430	0.011362	0.011430	0.011362	0.011396	0.011430	0.011665
0.011698	0.011631	0.011698	0.011532	0.011699	0.011396	0.011362
0.011330	0.011586	0.011426	0.011300	0.011671	0.011265	0.011414
0.011705	0.011604	0.011564	0.011765	0.011470	0.011631	0.011597
0.011530	0.011698	0.011522	0.011349	0.011354	0.011430	0.011500
0.011597	0.011499	0.011635	0.011703	0.011631	0.011698	0.011806
0.011534	0.011705	0.011636	0.011567	0.011834	0.011295	0.011498
0.011463	0.011396	0.011295	0.011295	0.011362	0.011362	0.011429
0.011362	0.011429	0.011329	0.011362	0.011362	0.011362	0.011429
0.011362	0.011732	0.011631	0.011664	0.011262	0.011362	0.011396
0.011396	0.011362	0.011429	0.011396	0.011329	0.011329	0.011329
0.011799	0.011429	0.011497	0.011295	0.011396	0.011564	0.011329
0.011329	0.011396	0.011362	0.011530	0.011329	0.011463	0.011597
0.011530	0.011362	0.011362	0.011295	0.011295	0.011463	0.011396
0.011295	0.011396	0.011262	0.011295	0.011429	0.011497	0.011329
0.011295	0.011429	0.011363	0.011262	0.011530	0.011262	0.011429
0.011505	0.011329	0.011564	0.011463	0.011429	0.011362	0.011463
0.011329	0.011664	0.011262	0.011463	0.011262	0.011262	0.011262
0.011496	0.011631	0.011396	0.011463	0.011295	0.011631	0.011262
0.011329	0.011329	0.011362	0.011463	0.011362	0.011463	0.011530
0.011530	0.011497	0.011396	0.011497	0.011463	0.011329	0.011295
0.011329	0.011329	0.011396	0.011262	0.011295	0.011463	0.011329
0.011295	0.011362	0.011497	0.011463	0.011429	0.011362	0.011362
0.011429	0.011362	0.011463	0.011329	0.011463	0.011564	0.011799
0.011362	0.011698	0.011371	0.017861	0.011191	0.011488	0.011653
0.011614	0.011658	0.011374	0.011292	0.011774	0.011682	0.011425
0.011345	0.011610	0.011294	0.011311	0.011177	0.011357	0.011318
0.011342	0.011470	0.011431	0.011193	0.011497	0.011530	0.011534

0.011404	0.011497	0.011497	0.011396	0.011429	0.011430	0.011396
0.011430	0.011362	0.011396	0.011430	0.011396	0.011329	0.011362
0.011362	0.011430	0.011329	0.011430	0.011463	0.011497	0.011396
0.011362	0.011329	0.011665	0.011665	0.011463	0.011497	0.011530
0.011295	0.011295	0.011329	0.011430	0.011295	0.011530	0.011530
0.011564	0.011564	0.011497	0.011597	0.011698	0.011599	0.011430
0.011430	0.011497	0.011530	0.011362	0.011430	0.011396	0.011430
0.011497	0.011497	0.011530	0.011497	0.011397	0.011597	0.011564
0.011433	0.011329	0.011430	0.011530	0.011430	0.011497	0.011597
0.011765	0.011470	0.011631	0.011597	0.011530	0.011698	0.011329
0.011498	0.011396	0.011362	0.011362	0.011430	0.011329	0.011430
0.011463	0.011497	0.011396	0.011362	0.011329	0.011665	0.011665
0.011463	0.011497	0.011564	0.011496	0.012069	0.011560	0.011482
0.011552	0.011511	0.011622	0.011510	0.011620	0.011543	0.011508
0.012022	0.011406	0.011540	0.011475	0.011749	0.011604	0.011707
0.012046	0.011475	0.011669	0.011732	0.011597	0.011530	0.011635
0.011295	0.011463	0.011597	0.011564	0.011567	0.011496	0.011464
0.011396	0.011329	0.011362	0.011396	0.011295	0.011362	0.011262
0.011329	0.011362	0.011329	0.011295	0.011396	0.011329	0.011362
0.011362	0.011295	0.011362	0.011396	0.011295	0.011497	0.011530
0.011429	0.011429	0.011429	0.011429	0.011362	0.011698	0.011396
0.011373	0.011362	0.011531	0.011535	0.011463	0.011463	0.011463
0.011463	0.011396	0.011362	0.011396	0.011497	0.011497	0.011429
0.011429	0.011564	0.011664	0.011463	0.011665	0.011665	0.011530
0.011362	0.011429	0.011463	0.011799	0.011430	0.011463	0.011429
0.011463	0.011329	0.011329	0.011631	0.011631	0.011597	0.011396
0.011497	0.011564	0.011429	0.011497	0.011463	0.011329	0.011597
0.011429	0.011396	0.011329	0.011564	0.011396	0.011496	0.011732
0.011429	0.011739	0.011732	0.011548	0.011117	0.011434	0.011163
0.011280	0.011722	0.011341	0.011417	0.011255	0.011417	0.011273
0.011366	0.011192	0.011283	0.011126	0.011276	0.011328	0.011506
0.011512	0.011414	0.011413	0.011412	0.011431	0.011497	0.011564
0.011564	0.011430	0.011534	0.011535	0.011670	0.011696	0.011429
0.011597	0.011463	0.011396	0.011430	0.011295	0.011463	0.011329
0.011329	0.011396	0.011362	0.011329	0.011362	0.011329	0.011430
0.011410	0.011410	0.011403	0.011437	0.011573	0.011538	0.011538
0.011537	0.011464	0.011282	0.011276	0.011399	0.011568	0.011569
0.011363	0.011433	0.011666	0.011635	0.011565	0.011599	0.011530
0.011463	0.011530	0.011530	0.011429	0.011934	0.011597	0.011665
0.011698	0.011665	0.011401	0.011476	0.011362	0.011564	0.011530
0.011565	0.011597	0.011597	0.011597	0.011689	0.011732	0.011338
0.011263	0.011268	0.011367	0.011332	0.011368	0.011209	0.011536
0.011403	0.011367	0.011156	0.011435	0.011396	0.011471	0.011362
0.011329	0.011262	0.011396	0.011362	0.011497	0.011463	0.011396
0.011497	0.011564	0.011362	0.011362	0.011396	0.011396	0.011398
0.011466	0.011396	0.011396	0.011431	0.011497	0.011564	0.011564
0.011430	0.011534	0.011535	0.011670	0.011696	0.011429	0.011597
0.011530	0.011530	0.011365	0.011513	0.011442	0.011469	0.011340
0.011542	0.011698	0.011526	0.011434	0.011329	0.011530	0.011396
0.011497	0.011430	0.011329	0.011431	0.011530	0.011533	0.011430
0.011329	0.011263	0.011396	0.011329	0.011262	0.011366	0.011531
0.011430	0.011362	0.011396	0.011430	0.011396	0.011329	0.011463
0.011396	0.011429	0.011429	0.011362	0.011429	0.011664	0.011262
0.011362	0.011698	0.011362	0.011396	0.011362	0.011664	0.011189
0.011566	0.011315	0.011110	0.011463	0.011549	0.011491	0.011186
0.000000	0.011463	0.011564	0.011671	0.011532	0.011669	0.011800
0.011700	0.011737	0.011698	0.011949	0.011664	0.012539	0.011700
0.012439	0.012207	0.012341	0.012613	0.011664	0.011363	0.011665
0.012104	0.012038	0.011631	0.011530	0.011598	0.011598	0.011567
0.011463	0.011467	0.011530	0.011497	0.011867	0.011933	0.011935

0.011834	0.011935	0.012038	0.011804	0.012104	0.012110	0.012171
0.012172	0.012206	0.012152	0.012172	0.012173	0.011463	0.011699
0.011767	0.012270	0.011772	0.012001	0.012474	0.011536	0.011430
0.011396	0.011396	0.011867	0.011883	0.012010	0.011732	0.011397
S062	0.011832	0.011832	0.012101	0.011866	0.011866	0.011933
0.011773	0.011705	0.011933	0.011429	0.011496	0.011464	0.011429
0.011396	0.011631	0.011362	0.011463	0.011530	0.011496	0.011463
0.011429	0.011463	0.011496	0.011430	0.011463	0.011463	0.011739
0.011631	0.011463	0.011362	0.011530	0.011530	0.011497	0.011530
0.011496	0.011597	0.011496	0.011496	0.011530	0.011496	0.011664
0.011362	0.011496	0.011530	0.011463	0.011496	0.011597	0.011530
0.011496	0.011665	0.011564	0.011765	0.011496	0.011765	0.011496
0.011463	0.011497	0.011631	0.011765	0.011799	0.011497	0.000000
0.011530	0.011597	0.011497	0.011731	0.011900	0.011496	0.011564
0.011463	0.011564	0.011530	0.011597	0.011530	0.011530	0.011530
0.011429	0.011463	0.011429	0.011463	0.011496	0.011531	0.011597
0.011530	0.011496	0.011564	0.011463	0.011530	0.011429	0.011496
0.011664	0.011597	0.011832	0.011664	0.011496	0.011732	0.011496
0.011691	0.011807	0.011739	0.011412	0.011433	0.011539	0.011533
0.012195	0.011569	0.011472	0.011731	0.011732	0.011463	0.011564
0.011564	0.011530	0.011597	0.011496	0.011597	0.011530	0.011530
0.011496	0.011530	0.011799	0.011664	0.011564	0.011530	0.011463
0.011430	0.011362	0.011497	0.011463	0.011597	0.011564	0.011497
0.011597	0.011665	0.011463	0.011463	0.011497	0.011497	0.011499
0.011567	0.011497	0.011497	0.011384	0.011458	0.011411	0.011497
0.011500	0.011438	0.011503	0.011474	0.011400	0.011437	0.011466
0.011636	0.011508	0.011542	0.011430	0.011440	0.011565	0.011464
0.011531	0.011532	0.011531	0.011631	0.011564	0.011933	0.011665
0.011698	0.011295	0.011497	0.011262	0.011396	0.011564	0.011430
0.011496	0.011496	0.011463	0.011429	0.011463	0.011429	0.011530
0.011497	0.011597	0.011530	0.011429	0.011532	0.011631	0.011635
0.011631	0.011530	0.011429	0.011364	0.011497	0.011430	0.011363
0.011468	0.011631	0.011430	0.011631	0.011396	0.011497	0.011463
0.011564	0.011463	0.011497	0.011463	0.011497	0.011362	0.011497
0.011530	0.011463	0.011530	0.011463	0.011497	0.011530	0.011765
0.011732	0.011732	0.011799	0.011633	0.011799	0.011497	0.011463
0.011432	0.011667	0.011563	0.011403	0.011773	0.011367	0.011493
0.011807	0.011706	0.011664	0.011866	0.011572	0.011732	0.011698
0.011631	0.011799	0.011664	0.011472	0.011563	0.011530	0.011602
0.011698	0.011600	0.011736	0.011805	0.011732	0.011732	0.011907
0.011635	0.011807	0.011737	0.011669	0.011935	0.011396	0.011599
0.011564	0.011496	0.011396	0.011396	0.011463	0.011463	0.011530
0.011463	0.011530	0.011429	0.011463	0.011463	0.011463	0.011530
0.011463	0.011832	0.011732	0.011765	0.011362	0.011463	0.011497
0.011496	0.011463	0.011530	0.011228	0.011429	0.011429	0.011429
0.011900	0.011530	0.011597	0.011396	0.011496	0.011665	0.011429
0.011429	0.011496	0.011463	0.011631	0.011429	0.011564	0.011698
0.011631	0.011463	0.011463	0.011396	0.011396	0.011564	0.011496
0.011396	0.011496	0.011362	0.011396	0.011530	0.011530	0.011429
0.011396	0.011530	0.011463	0.011362	0.011631	0.011362	0.011530
0.011608	0.011429	0.011664	0.011564	0.011530	0.011463	0.011564
0.011429	0.011765	0.011362	0.011564	0.011362	0.011362	0.011362
0.011597	0.011732	0.011497	0.011564	0.011396	0.011732	0.011362
0.011429	0.011429	0.011463	0.011564	0.011396	0.011564	0.011631
0.011631	0.011597	0.011496	0.011597	0.011564	0.011429	0.011396
0.011429	0.011429	0.011497	0.011362	0.011396	0.011564	0.011429
0.011396	0.011463	0.011597	0.011564	0.011530	0.011463	0.011463
0.011530	0.011463	0.011564	0.011429	0.011564	0.011664	0.011900
0.011463	0.011799	0.011474	0.018012	0.011387	0.011549	0.011758
0.011718	0.011764	0.011584	0.011466	0.011884	0.011785	0.011607

0.011677	0.011671	0.011372	0.011521	0.011294	0.011505	0.011404
0.011623	0.011582	0.011579	0.011391	0.011597	0.011631	0.011635
0.011507	0.011597	0.011597	0.011430	0.011530	0.011530	0.011497
0.011530	0.011463	0.011497	0.011530	0.011497	0.011430	0.011463
0.011463	0.011530	0.011430	0.011530	0.011564	0.011597	0.011496
0.011463	0.011430	0.011765	0.011765	0.011564	0.011598	0.011631
0.011396	0.011396	0.011430	0.011530	0.011396	0.011631	0.011631
0.011665	0.011664	0.011597	0.011698	0.011799	0.011666	0.011530
0.011530	0.011597	0.011631	0.011463	0.011530	0.011497	0.011530
0.011598	0.011598	0.011631	0.011598	0.011498	0.011698	0.011665
0.011500	0.011430	0.011530	0.011631	0.011530	0.011598	0.011698
0.011866	0.011572	0.011732	0.011698	0.011631	0.011799	0.011430
0.011599	0.011497	0.011463	0.011463	0.011530	0.011430	0.011530
0.011564	0.011597	0.011496	0.011463	0.011430	0.011765	0.011765
0.011564	0.011598	0.011664	0.011597	0.012170	0.011667	0.011588
0.011658	0.011615	0.011728	0.011614	0.011725	0.011646	0.011612
0.012125	0.011510	0.011642	0.011579	0.011783	0.011706	0.011809
0.012148	0.011626	0.011770	0.011832	0.011698	0.011631	0.011736
0.011396	0.011564	0.011698	0.011664	0.011668	0.011597	0.011565
0.011496	0.011429	0.011463	0.011496	0.011396	0.011463	0.011362
0.011429	0.011463	0.011429	0.011396	0.011496	0.011429	0.011463
0.011463	0.011396	0.011463	0.011497	0.011396	0.011597	0.011631
0.011530	0.011530	0.011530	0.011530	0.011463	0.011799	0.011496
0.011478	0.011463	0.011631	0.011637	0.011564	0.011564	0.011564
0.011564	0.011497	0.011463	0.011497	0.011597	0.011597	0.011530
0.011530	0.011664	0.011765	0.011564	0.011765	0.011765	0.011631
0.011463	0.011530	0.011564	0.011900	0.011530	0.011564	0.011530
0.011564	0.011429	0.011429	0.011732	0.011732	0.011698	0.011496
0.011597	0.011664	0.011530	0.011597	0.011564	0.011429	0.011698
0.011530	0.011496	0.011429	0.011664	0.011496	0.011597	0.011832
0.011530	0.011841	0.011832	0.011629	0.011286	0.011536	0.011422
0.011390	0.011839	0.011447	0.011596	0.011451	0.011518	0.011538
0.011519	0.011455	0.011561	0.011324	0.011545	0.011462	0.011628
0.011625	0.011532	0.011518	0.011483	0.011532	0.011597	0.011665
0.011665	0.011262	0.011364	0.011636	0.011704	0.011519	0.011530
0.011698	0.011295	0.011497	0.011262	0.011396	0.011564	0.011430
0.011496	0.011496	0.011463	0.011429	0.011463	0.011429	0.011530
0.011514	0.011480	0.011505	0.011540	0.011676	0.011641	0.011641
0.011639	0.011565	0.011393	0.011384	0.011501	0.011670	0.011671
0.011464	0.011534	0.011767	0.011736	0.011666	0.011700	0.011631
0.011564	0.011631	0.011631	0.011530	0.012034	0.011698	0.011765
0.011799	0.011772	0.011503	0.011580	0.011463	0.011664	0.011631
0.011666	0.011698	0.011698	0.011698	0.011768	0.011832	0.011476
0.011364	0.011372	0.011469	0.011434	0.011470	0.011396	0.011638
0.011506	0.011411	0.011298	0.011510	0.011497	0.011573	0.011463
0.011430	0.011362	0.011497	0.011463	0.011597	0.011564	0.011497
0.011597	0.011665	0.011463	0.011463	0.011497	0.011497	0.011499
0.011567	0.011497	0.011497	0.011532	0.011597	0.011665	0.011665
0.011262	0.011364	0.011636	0.011704	0.011519	0.011530	0.011698
0.011631	0.011631	0.011466	0.011652	0.011581	0.011605	0.011481
0.011646	0.011789	0.011568	0.011536	0.011430	0.011631	0.011497
0.011597	0.011530	0.011429	0.011532	0.011631	0.011635	0.011530
0.011429	0.011364	0.011497	0.011430	0.011363	0.011468	0.011631
0.011530	0.011463	0.011497	0.011530	0.011497	0.011430	0.011564
0.011496	0.011530	0.011530	0.011463	0.011530	0.011765	0.011362
0.011463	0.011799	0.011463	0.011496	0.011463	0.011765	0.011489
0.011791	0.011485	0.011411	0.011624	0.011712	0.011599	0.011436
0.011270	0.011564	0.011664	0.011773	0.011632	0.011771	0.011901
0.011800	0.011838	0.011799	0.012051	0.011765	0.012573	0.011801
0.012540	0.012241	0.012375	0.012714	0.011765	0.011463	0.011766

0.012205	0.012140	0.011732	0.011631	0.011699	0.011699	0.011669
0.011564	0.011569	0.011631	0.011598	0.011968	0.011967	0.012036
0.011935	0.012035	0.012139	0.011838	0.012205	0.012212	0.012272
0.012273	0.012307	0.012255	0.012273	0.012274	0.011564	0.011800
0.011868	0.012371	0.011874	0.012102	0.012575	0.011638	0.011531
0.011496	0.011496	0.011968	0.011965	0.012116	0.011832	0.011498
S063	0.011698	0.011698	0.012034	0.011799	0.011799	0.011866
0.011705	0.011637	0.011866	0.011295	0.011362	0.011329	0.011295
0.011329	0.011496	0.011295	0.011329	0.011396	0.011362	0.011329
0.011295	0.011329	0.011429	0.011295	0.011262	0.011329	0.011671
0.011564	0.011329	0.011228	0.011463	0.011329	0.011362	0.011329
0.011362	0.011396	0.011362	0.011362	0.011396	0.011362	0.011530
0.011295	0.011362	0.011396	0.011329	0.011429	0.011463	0.011396
0.011362	0.011530	0.011430	0.011698	0.011362	0.011564	0.011362
0.011329	0.011429	0.011496	0.011564	0.011731	0.011429	0.011530
0.000000	0.011396	0.011362	0.011329	0.011832	0.011362	0.011429
0.011329	0.011429	0.011396	0.011463	0.011396	0.011396	0.011396
0.011295	0.011329	0.011295	0.011396	0.011362	0.011397	0.011463
0.011396	0.011362	0.011362	0.011329	0.011463	0.011228	0.011362
0.011530	0.011463	0.011698	0.011530	0.011362	0.011530	0.011362
0.011542	0.011770	0.011588	0.011295	0.011284	0.011391	0.011493
0.012121	0.011518	0.011307	0.011597	0.011597	0.011396	0.011496
0.011429	0.011396	0.011463	0.011362	0.011463	0.011329	0.011396
0.011362	0.011463	0.011664	0.011530	0.011429	0.011396	0.011262
0.011295	0.011295	0.011295	0.011329	0.011530	0.011497	0.011429
0.011530	0.011597	0.011329	0.011329	0.011362	0.011362	0.011364
0.011432	0.011362	0.011362	0.011204	0.011313	0.011271	0.011363
0.011365	0.011301	0.011355	0.011336	0.011264	0.011266	0.011330
0.011600	0.011370	0.011336	0.011296	0.011302	0.011430	0.011396
0.011464	0.011397	0.011396	0.011496	0.011429	0.011731	0.011597
0.011631	0.011497	0.011362	0.011463	0.011262	0.011496	0.011295
0.011362	0.011362	0.011329	0.011295	0.011329	0.011295	0.011396
0.011295	0.011396	0.011396	0.011295	0.011397	0.011497	0.011499
0.011430	0.011396	0.011295	0.011229	0.011295	0.011295	0.011228
0.011332	0.011430	0.011228	0.011430	0.011262	0.011362	0.011329
0.011362	0.011329	0.011295	0.011329	0.011362	0.011228	0.011295
0.011329	0.011329	0.011396	0.011329	0.011362	0.011396	0.011631
0.011732	0.011664	0.011732	0.011498	0.011732	0.011362	0.011329
0.011364	0.011586	0.011472	0.011266	0.011739	0.011231	0.011375
0.011739	0.011638	0.011530	0.011732	0.011436	0.011597	0.011497
0.011496	0.011664	0.011522	0.011349	0.011395	0.011396	0.011466
0.011564	0.011465	0.011601	0.011601	0.011597	0.011665	0.011772
0.011500	0.011603	0.011602	0.011533	0.011800	0.011262	0.011464
0.011362	0.011362	0.011262	0.011262	0.011262	0.011329	0.011396
0.011262	0.011329	0.011228	0.011396	0.011262	0.011329	0.011396
0.011329	0.011765	0.011664	0.011698	0.011228	0.011329	0.011362
0.011362	0.011329	0.011396	0.011429	0.011295	0.011295	0.011295
0.011832	0.011463	0.011463	0.011262	0.011362	0.011463	0.011295
0.011295	0.011362	0.011329	0.011429	0.011295	0.011429	0.011496
0.011496	0.011329	0.011329	0.011262	0.011262	0.011429	0.011362
0.011262	0.011362	0.011228	0.011262	0.011396	0.011530	0.011295
0.011262	0.011396	0.011329	0.011228	0.011564	0.011195	0.011396
0.011402	0.011295	0.011597	0.011429	0.011463	0.011329	0.011429
0.011295	0.011564	0.011228	0.011496	0.011295	0.011295	0.011228
0.011396	0.011665	0.011362	0.011429	0.011262	0.011664	0.011295
0.011295	0.011295	0.011329	0.011362	0.011329	0.011429	0.011496
0.011496	0.011463	0.011362	0.011463	0.011094	0.011295	0.011262
0.011295	0.011295	0.011362	0.011295	0.011194	0.011429	0.011295
0.011329	0.011329	0.011463	0.011496	0.011396	0.011262	0.011329
0.011396	0.011329	0.011429	0.011295	0.011429	0.011530	0.011832

0.011329	0.011732	0.011336	0.017861	0.011322	0.011427	0.011688
0.011648	0.011693	0.011374	0.011408	0.011719	0.011716	0.011364
0.011582	0.011548	0.011255	0.011270	0.011294	0.011406	0.011318
0.011576	0.011507	0.011468	0.011325	0.011463	0.011497	0.011500
0.011301	0.011463	0.011463	0.011429	0.011396	0.011396	0.011362
0.011396	0.011329	0.011362	0.011396	0.011362	0.011295	0.011329
0.011329	0.011396	0.011295	0.011396	0.011429	0.011463	0.011362
0.011396	0.011362	0.011698	0.011698	0.011430	0.011464	0.011497
0.011262	0.011262	0.011295	0.011329	0.011262	0.011497	0.011497
0.011530	0.011530	0.011463	0.011564	0.011732	0.011565	0.011329
0.011396	0.011463	0.011429	0.011329	0.011396	0.011362	0.011396
0.011463	0.011463	0.011497	0.011396	0.011296	0.011564	0.011463
0.011399	0.011295	0.011396	0.011497	0.011396	0.011530	0.011564
0.011732	0.011436	0.011597	0.011497	0.011496	0.011664	0.011295
0.011464	0.011363	0.011329	0.011329	0.011396	0.011295	0.011396
0.011429	0.011463	0.011362	0.011396	0.011362	0.011698	0.011698
0.011430	0.011464	0.011530	0.011463	0.012103	0.011489	0.011412
0.011482	0.011476	0.011587	0.011441	0.011585	0.011508	0.011474
0.012056	0.011372	0.011505	0.011441	0.011714	0.011570	0.011604
0.012080	0.011512	0.011635	0.011698	0.011564	0.011564	0.011601
0.011262	0.011362	0.011564	0.011530	0.011533	0.011530	0.011498
0.011362	0.011228	0.011262	0.011362	0.011194	0.011329	0.011228
0.011362	0.011396	0.011295	0.011262	0.011362	0.011295	0.011329
0.011329	0.011262	0.011329	0.011362	0.011262	0.011396	0.011496
0.011396	0.011463	0.011396	0.011396	0.011329	0.011731	0.011362
0.011373	0.011329	0.011564	0.011569	0.011362	0.011429	0.011429
0.011429	0.011295	0.011329	0.011295	0.011463	0.011463	0.011396
0.011396	0.011530	0.011698	0.011429	0.011631	0.011631	0.011496
0.011329	0.011396	0.011429	0.011832	0.011396	0.011362	0.011396
0.011429	0.011362	0.011362	0.011665	0.011530	0.011631	0.011362
0.011463	0.011530	0.011396	0.011463	0.011429	0.011295	0.011631
0.011396	0.011362	0.011362	0.011530	0.011362	0.011463	0.011698
0.011396	0.011773	0.011765	0.011588	0.011286	0.011468	0.011370
0.011280	0.011800	0.011306	0.011551	0.011304	0.011518	0.011485
0.011519	0.011258	0.011450	0.011258	0.011276	0.011462	0.011425
0.011437	0.011296	0.011448	0.011377	0.011397	0.011530	0.011597
0.011597	0.011464	0.011567	0.011569	0.011704	0.011732	0.011396
0.011631	0.011497	0.011362	0.011463	0.011262	0.011496	0.011295
0.011362	0.011362	0.011329	0.011295	0.011329	0.011295	0.011396
0.011375	0.011375	0.011368	0.011403	0.011539	0.011436	0.011504
0.011503	0.011430	0.011245	0.011240	0.011365	0.011535	0.011535
0.011329	0.011399	0.011632	0.011601	0.011531	0.011565	0.011496
0.011429	0.011496	0.011496	0.011396	0.011967	0.011564	0.011631
0.011665	0.011737	0.011367	0.011441	0.011329	0.011530	0.011496
0.011531	0.011564	0.011564	0.011564	0.011728	0.011698	0.011338
0.011229	0.011199	0.011332	0.011298	0.011368	0.011209	0.011570
0.011369	0.011411	0.011203	0.011360	0.011363	0.011437	0.011262
0.011295	0.011295	0.011295	0.011329	0.011530	0.011497	0.011429
0.011530	0.011597	0.011329	0.011329	0.011362	0.011362	0.011364
0.011432	0.011362	0.011362	0.011397	0.011530	0.011597	0.011597
0.011464	0.011567	0.011569	0.011704	0.011732	0.011396	0.011564
0.011496	0.011496	0.011331	0.011513	0.011408	0.011469	0.011340
0.011439	0.011653	0.011441	0.011400	0.011228	0.011430	0.011295
0.011396	0.011396	0.011295	0.011397	0.011497	0.011499	0.011396
0.011295	0.011229	0.011295	0.011295	0.011228	0.011332	0.011430
0.011396	0.011329	0.011362	0.011396	0.011362	0.011295	0.011497
0.011362	0.011396	0.011396	0.011329	0.011396	0.011631	0.011295
0.011329	0.011731	0.011329	0.011295	0.011329	0.011698	0.011317
0.011734	0.011315	0.011311	0.011543	0.011630	0.011527	0.011269
0.011323	0.011496	0.011597	0.011671	0.011565	0.011703	0.011834

0.011733	0.011770	0.011731	0.011983	0.011698	0.012573	0.011734
0.012473	0.012241	0.012375	0.012646	0.011698	0.011396	0.011698
0.012137	0.012072	0.011664	0.011564	0.011632	0.011632	0.011601
0.011496	0.011501	0.011564	0.011531	0.011901	0.011967	0.011968
0.011867	0.011968	0.012072	0.011838	0.012138	0.012144	0.012205
0.012205	0.012239	0.012186	0.012205	0.012207	0.011429	0.011732
0.011800	0.012303	0.011806	0.012034	0.012508	0.011570	0.011464
0.011429	0.011429	0.011900	0.011924	0.012045	0.011765	0.011431
S064	0.011765	0.011765	0.012102	0.011866	0.011866	0.011933
0.011773	0.011705	0.011933	0.011362	0.011429	0.011397	0.011362
0.011396	0.011564	0.011362	0.011396	0.011463	0.011429	0.011396
0.011362	0.011396	0.011496	0.011363	0.011329	0.011396	0.011739
0.011631	0.011396	0.011295	0.011530	0.011396	0.011430	0.011396
0.011429	0.011329	0.011429	0.011429	0.011463	0.011429	0.011597
0.011362	0.011429	0.011463	0.011396	0.011496	0.011530	0.011463
0.011429	0.011597	0.011497	0.011765	0.011429	0.011631	0.011429
0.011396	0.011497	0.011564	0.011631	0.011799	0.011497	0.011597
0.011396	0.000000	0.011430	0.011597	0.011900	0.011429	0.011497
0.011396	0.011496	0.011463	0.011530	0.011463	0.011463	0.011463
0.011362	0.011396	0.011362	0.011463	0.011429	0.011464	0.011530
0.011463	0.011429	0.011429	0.011396	0.011530	0.011295	0.011429
0.011597	0.011530	0.011765	0.011530	0.011429	0.011597	0.011429
0.011579	0.011807	0.011664	0.011295	0.011284	0.011465	0.011493
0.012158	0.011467	0.011349	0.011664	0.011664	0.011463	0.011564
0.011497	0.011463	0.011530	0.011429	0.011530	0.011396	0.011463
0.011429	0.011530	0.011732	0.011597	0.011497	0.011463	0.011329
0.011362	0.011362	0.011362	0.011396	0.011597	0.011564	0.011497
0.011597	0.011665	0.011396	0.011396	0.011430	0.011429	0.011432
0.011500	0.011430	0.011430	0.011276	0.011386	0.011341	0.011430
0.011433	0.011370	0.011429	0.011405	0.011332	0.011369	0.011398
0.011672	0.011439	0.011405	0.011363	0.011371	0.011498	0.011464
0.011531	0.011465	0.011464	0.011564	0.011496	0.011799	0.011665
0.011698	0.011564	0.011429	0.011530	0.011329	0.011564	0.011362
0.011429	0.011429	0.011396	0.011362	0.011396	0.011362	0.011463
0.011362	0.011463	0.011463	0.011362	0.011465	0.011564	0.011533
0.011497	0.011463	0.011362	0.011296	0.011430	0.011362	0.011295
0.011400	0.011497	0.011295	0.011497	0.011329	0.011430	0.011396
0.011430	0.011396	0.011362	0.011396	0.011430	0.011295	0.011362
0.011396	0.011396	0.011463	0.011396	0.011430	0.011463	0.011698
0.011799	0.011732	0.011799	0.011566	0.011799	0.011429	0.011396
0.011432	0.011626	0.011380	0.011335	0.011807	0.011299	0.011454
0.011807	0.011706	0.011597	0.011799	0.011504	0.011665	0.011564
0.011564	0.011732	0.011593	0.011390	0.011521	0.011463	0.011534
0.011631	0.011532	0.011669	0.011669	0.011665	0.011732	0.011839
0.011568	0.011671	0.011670	0.011601	0.011868	0.011329	0.011532
0.011429	0.011429	0.011329	0.011329	0.011329	0.011396	0.011463
0.011329	0.011396	0.011295	0.011463	0.011329	0.011396	0.011463
0.011396	0.011832	0.011732	0.011765	0.011295	0.011396	0.011430
0.011429	0.011396	0.011463	0.011497	0.011362	0.011362	0.011362
0.011900	0.011530	0.011530	0.011329	0.011429	0.011262	0.011362
0.011362	0.011429	0.011396	0.011497	0.011362	0.011497	0.011564
0.011564	0.011396	0.011396	0.011329	0.011329	0.011497	0.011429
0.011329	0.011429	0.011295	0.011329	0.011463	0.011598	0.011362
0.011329	0.011463	0.011396	0.011295	0.011631	0.011295	0.011463
0.011471	0.011362	0.011664	0.011497	0.011530	0.011396	0.011497
0.011362	0.011631	0.011295	0.011429	0.011362	0.011362	0.011295
0.011463	0.011732	0.011430	0.011497	0.011329	0.011732	0.011362
0.011362	0.011362	0.011396	0.011094	0.011396	0.011497	0.011564
0.011497	0.011463	0.011429	0.011530	0.011429	0.011362	0.011329
0.011362	0.011362	0.011430	0.011362	0.011262	0.011497	0.011362

0.011396	0.011396	0.011530	0.011564	0.011463	0.011329	0.011396
0.011463	0.011396	0.011496	0.011362	0.011497	0.011597	0.011900
0.011396	0.011799	0.011405	0.017899	0.011257	0.011488	0.011758
0.011718	0.011729	0.011322	0.011234	0.011719	0.011785	0.011303
0.011535	0.011610	0.011294	0.011353	0.011372	0.011356	0.011318
0.011482	0.011544	0.011505	0.011457	0.011530	0.011564	0.011568
0.011369	0.011530	0.011530	0.011497	0.011463	0.011463	0.011430
0.011463	0.011396	0.011429	0.011463	0.011430	0.011362	0.011396
0.011396	0.011463	0.011362	0.011463	0.011497	0.011530	0.011429
0.011463	0.011430	0.011766	0.011766	0.011497	0.011531	0.011564
0.011329	0.011329	0.011362	0.011396	0.011329	0.011564	0.011564
0.011597	0.011597	0.011530	0.011631	0.011799	0.011633	0.011396
0.011463	0.011530	0.011497	0.011396	0.011463	0.011430	0.011463
0.011531	0.011530	0.011564	0.011463	0.011363	0.011631	0.011530
0.011467	0.011362	0.011463	0.011564	0.011463	0.011598	0.011631
0.011799	0.011504	0.011665	0.011564	0.011564	0.011732	0.011363
0.011532	0.011430	0.011396	0.011396	0.011463	0.011362	0.011463
0.011497	0.011530	0.011429	0.011463	0.011430	0.011766	0.011766
0.011497	0.011531	0.011597	0.011530	0.012170	0.011596	0.011517
0.011588	0.011546	0.011658	0.011545	0.011655	0.011577	0.011543
0.012125	0.011441	0.011574	0.011510	0.011783	0.011638	0.011673
0.012148	0.011626	0.011703	0.011765	0.011631	0.011631	0.011668
0.011329	0.011429	0.011631	0.011597	0.011601	0.011597	0.011565
0.011429	0.011295	0.011329	0.011429	0.011262	0.011396	0.011295
0.011429	0.011463	0.011362	0.011329	0.011429	0.011362	0.011396
0.011396	0.011329	0.011396	0.011430	0.011329	0.011329	0.011564
0.011463	0.011530	0.011463	0.011463	0.011396	0.011799	0.011429
0.011478	0.011396	0.011631	0.011637	0.011430	0.011496	0.011497
0.011497	0.011362	0.011396	0.011362	0.011530	0.011530	0.011463
0.011463	0.011597	0.011765	0.011496	0.011698	0.011698	0.011564
0.011396	0.011463	0.011497	0.011900	0.011463	0.011430	0.011463
0.011497	0.011429	0.011429	0.011732	0.011597	0.011698	0.011429
0.011463	0.011530	0.011463	0.011463	0.011496	0.011295	0.011698
0.011463	0.011429	0.011429	0.011597	0.011429	0.011530	0.011765
0.011463	0.011841	0.011832	0.011548	0.011286	0.011502	0.011267
0.011280	0.011761	0.011341	0.011507	0.011304	0.011467	0.011379
0.011468	0.011192	0.011339	0.011192	0.011222	0.011328	0.011506
0.011512	0.011336	0.011518	0.011412	0.011465	0.011597	0.011665
0.011665	0.011531	0.011635	0.011636	0.011772	0.011767	0.011463
0.011698	0.011564	0.011429	0.011530	0.011329	0.011564	0.011362
0.011429	0.011429	0.011396	0.011362	0.011396	0.011362	0.011463
0.011444	0.011445	0.011437	0.011471	0.011608	0.011504	0.011572
0.011571	0.011497	0.011319	0.011312	0.011433	0.011602	0.011603
0.011397	0.011467	0.011700	0.011669	0.011598	0.011632	0.011564
0.011496	0.011564	0.011564	0.011463	0.012034	0.011597	0.011698
0.011732	0.011808	0.011435	0.011476	0.011396	0.011597	0.011564
0.011598	0.011631	0.011631	0.011631	0.011807	0.011765	0.011407
0.011297	0.011303	0.011401	0.011366	0.011470	0.011321	0.011638
0.011437	0.011586	0.011298	0.011472	0.011430	0.011505	0.011329
0.011362	0.011362	0.011362	0.011396	0.011597	0.011564	0.011497
0.011597	0.011665	0.011396	0.011396	0.011430	0.011429	0.011432
0.011500	0.011430	0.011430	0.011465	0.011597	0.011665	0.011665
0.011531	0.011635	0.011636	0.011772	0.011767	0.011463	0.011631
0.011564	0.011564	0.011398	0.011582	0.011477	0.011537	0.011376
0.011508	0.011744	0.011526	0.011468	0.011295	0.011497	0.011362
0.011463	0.011463	0.011362	0.011465	0.011564	0.011533	0.011463
0.011362	0.011296	0.011430	0.011362	0.011295	0.011400	0.011497
0.011463	0.011396	0.011429	0.011463	0.011430	0.011362	0.011564
0.011429	0.011463	0.011463	0.011396	0.011463	0.011698	0.011362
0.011396	0.011799	0.011396	0.011362	0.011396	0.011765	0.011317

0.011735	0.011315	0.011261	0.011584	0.011630	0.011599	0.011269
0.011218	0.011564	0.011664	0.011773	0.011633	0.011737	0.011901
0.011801	0.011838	0.011799	0.012017	0.011765	0.012640	0.011802
0.012540	0.012308	0.012442	0.012714	0.011765	0.011463	0.011766
0.012205	0.012140	0.011732	0.011631	0.011699	0.011699	0.011669
0.011564	0.011535	0.011631	0.011598	0.011968	0.012034	0.012036
0.011935	0.012036	0.012139	0.011906	0.012205	0.012212	0.012272
0.012273	0.012307	0.012255	0.012273	0.012275	0.011496	0.011800
0.011868	0.012371	0.011874	0.012102	0.012575	0.011638	0.011531
0.011496	0.011496	0.011968	0.012048	0.012116	0.011832	0.011498
S065	0.011664	0.011664	0.011933	0.011765	0.011765	0.011832
0.011671	0.011603	0.011832	0.011262	0.011329	0.011296	0.011262
0.011295	0.011463	0.011262	0.011295	0.011362	0.011329	0.011295
0.011262	0.011295	0.011396	0.011262	0.011295	0.011295	0.011637
0.011530	0.011295	0.011195	0.011429	0.011362	0.011329	0.011362
0.011329	0.011429	0.011329	0.011329	0.011362	0.011329	0.011497
0.011262	0.011329	0.011362	0.011295	0.011396	0.011429	0.011362
0.011329	0.011497	0.011396	0.011664	0.011329	0.011597	0.011329
0.011295	0.011396	0.011463	0.011597	0.011698	0.011396	0.011497
0.011362	0.011430	0.000000	0.011564	0.011799	0.011329	0.011396
0.011295	0.011396	0.011295	0.011429	0.011362	0.011362	0.011362
0.011262	0.011295	0.011262	0.011362	0.011329	0.011363	0.011429
0.011362	0.011329	0.011396	0.011295	0.011429	0.011262	0.011329
0.011496	0.011429	0.011664	0.011497	0.011329	0.011564	0.011329
0.011468	0.011695	0.011513	0.011216	0.011210	0.011318	0.011415
0.012048	0.011518	0.011225	0.011564	0.011564	0.011362	0.011463
0.011396	0.011362	0.011429	0.011329	0.011430	0.011362	0.011362
0.011329	0.011429	0.011631	0.011496	0.011396	0.011362	0.011295
0.011262	0.011262	0.011329	0.011295	0.011497	0.011463	0.011396
0.011497	0.011564	0.011295	0.011295	0.011329	0.011329	0.011330
0.011398	0.011329	0.011329	0.011204	0.011277	0.011236	0.011329
0.011331	0.011266	0.011282	0.011302	0.011230	0.011266	0.011297
0.011564	0.011336	0.011370	0.011262	0.011268	0.011397	0.011363
0.011430	0.011364	0.011363	0.011463	0.011396	0.011765	0.011564
0.011597	0.011463	0.011329	0.011430	0.011228	0.011463	0.011262
0.011329	0.011329	0.011295	0.011262	0.011295	0.011262	0.011362
0.011329	0.011430	0.011363	0.011262	0.011363	0.011463	0.011466
0.011463	0.011363	0.011262	0.011195	0.011329	0.011262	0.011195
0.011264	0.011463	0.011262	0.011463	0.011228	0.011329	0.011295
0.011396	0.011295	0.011329	0.011295	0.011329	0.011195	0.011329
0.011363	0.011295	0.011362	0.011295	0.011329	0.011362	0.011597
0.011698	0.011631	0.011698	0.011431	0.011699	0.011329	0.011295
0.011330	0.011586	0.011472	0.011198	0.011705	0.011197	0.011296
0.011705	0.011570	0.011496	0.011698	0.011402	0.011564	0.011530
0.011463	0.011631	0.011486	0.011349	0.011396	0.011362	0.011432
0.011530	0.011364	0.011567	0.011635	0.011564	0.011631	0.011738
0.011466	0.011637	0.011568	0.011500	0.011767	0.011228	0.011431
0.011396	0.011329	0.011228	0.011228	0.011295	0.011295	0.011362
0.011295	0.011362	0.011262	0.011362	0.011295	0.011295	0.011362
0.011295	0.011732	0.011631	0.011664	0.011195	0.011295	0.011262
0.011329	0.011295	0.011362	0.011396	0.011262	0.011262	0.011262
0.011799	0.011429	0.011429	0.011228	0.011329	0.011497	0.011262
0.011262	0.011329	0.011295	0.011463	0.011262	0.011396	0.011530
0.011463	0.011295	0.011295	0.011228	0.011228	0.011396	0.011329
0.011228	0.011329	0.011195	0.011228	0.011362	0.011497	0.011262
0.011228	0.011362	0.011295	0.011195	0.011530	0.011195	0.011362
0.011437	0.011262	0.011564	0.011396	0.011429	0.011295	0.011396
0.011262	0.011597	0.011195	0.011463	0.011262	0.011262	0.011195
0.011429	0.011598	0.000000	0.011396	0.011228	0.011631	0.011262
0.011262	0.011262	0.011295	0.011396	0.011295	0.011396	0.011463

0.011463	0.011429	0.011329	0.011430	0.011396	0.011262	0.011228
0.011262	0.011262	0.000000	0.011262	0.011228	0.011396	0.011262
0.011295	0.011295	0.011429	0.011463	0.011362	0.011295	0.011295
0.011295	0.011295	0.011396	0.011262	0.011396	0.011497	0.011799
0.011295	0.011698	0.011267	0.017785	0.011257	0.011427	0.011618
0.011579	0.011623	0.011322	0.011234	0.011719	0.011647	0.011364
0.011487	0.011549	0.011177	0.011270	0.011255	0.011307	0.011275
0.011529	0.011433	0.011431	0.011259	0.011429	0.011463	0.011466
0.011335	0.011430	0.011429	0.011396	0.011362	0.011363	0.011329
0.011362	0.011295	0.011329	0.011362	0.011329	0.011262	0.011295
0.011295	0.011362	0.011262	0.011161	0.011396	0.011430	0.011329
0.011362	0.011329	0.011665	0.011665	0.011396	0.011430	0.011463
0.011228	0.011228	0.011262	0.011363	0.011228	0.011463	0.011463
0.011497	0.011496	0.011429	0.011530	0.011698	0.011532	0.011362
0.011362	0.011430	0.011463	0.011295	0.011362	0.011329	0.011362
0.011430	0.011430	0.011463	0.011430	0.011330	0.011530	0.011497
0.011365	0.011262	0.011362	0.011463	0.011363	0.011497	0.011530
0.011698	0.011402	0.011564	0.011530	0.011463	0.011631	0.011262
0.011431	0.011329	0.011295	0.011295	0.011362	0.011262	0.011161
0.011396	0.011430	0.011329	0.011362	0.011329	0.011665	0.011665
0.011396	0.011430	0.011496	0.011429	0.012069	0.011489	0.011412
0.011482	0.011442	0.011552	0.011441	0.011550	0.011474	0.011440
0.012022	0.011337	0.011471	0.011406	0.011680	0.011536	0.011639
0.012046	0.011513	0.011601	0.011664	0.011530	0.011530	0.011567
0.011228	0.011396	0.011530	0.011496	0.011499	0.011496	0.011464
0.011329	0.011262	0.011295	0.011329	0.011228	0.011295	0.011195
0.011329	0.011362	0.011262	0.011228	0.011329	0.011262	0.011295
0.011295	0.011228	0.011295	0.011329	0.011228	0.011429	0.011463
0.011362	0.011429	0.011362	0.011362	0.011295	0.011698	0.011329
0.011373	0.011295	0.011531	0.011535	0.011396	0.011396	0.011396
0.011396	0.011329	0.011295	0.011329	0.011429	0.011429	0.011362
0.011362	0.011429	0.011664	0.011396	0.011597	0.011597	0.011463
0.011295	0.011362	0.011396	0.011799	0.011362	0.011396	0.011362
0.011396	0.011329	0.011329	0.011631	0.011564	0.011597	0.011329
0.011430	0.011497	0.011362	0.011430	0.011396	0.011262	0.011597
0.011362	0.011329	0.011329	0.011497	0.011329	0.011429	0.011665
0.011362	0.011739	0.011732	0.011507	0.011229	0.011434	0.011215
0.011225	0.011722	0.011236	0.011462	0.011304	0.011366	0.011379
0.011519	0.011192	0.011339	0.011192	0.011222	0.011194	0.011385
0.011399	0.011296	0.011377	0.011307	0.011364	0.011497	0.011564
0.011564	0.011430	0.011534	0.011535	0.011670	0.011661	0.011362
0.011597	0.011463	0.011329	0.011430	0.011228	0.011463	0.011262
0.011329	0.011329	0.011295	0.011262	0.011295	0.011262	0.011362
0.011340	0.011340	0.011334	0.011368	0.011505	0.011470	0.011470
0.011469	0.011329	0.011208	0.011204	0.011331	0.011501	0.011501
0.011296	0.011365	0.011599	0.011567	0.011497	0.011531	0.011463
0.011396	0.011463	0.011463	0.011362	0.011934	0.011530	0.011598
0.011631	0.011701	0.011333	0.011407	0.011295	0.011497	0.011463
0.011498	0.011530	0.011530	0.011530	0.011650	0.011664	0.011303
0.011196	0.011199	0.011299	0.011264	0.011368	0.011210	0.011536
0.011335	0.011499	0.011251	0.011360	0.011329	0.011402	0.011295
0.011262	0.011262	0.011329	0.011295	0.011497	0.011463	0.011396
0.011497	0.011564	0.011295	0.011295	0.011329	0.011329	0.011330
0.011398	0.011329	0.011329	0.011364	0.011497	0.011564	0.011564
0.011430	0.011534	0.011535	0.011670	0.011661	0.011362	0.011530
0.011463	0.011463	0.011297	0.011478	0.011408	0.011435	0.011270
0.011439	0.011653	0.011484	0.011366	0.011262	0.011463	0.011329
0.011430	0.011363	0.011262	0.011363	0.011463	0.011466	0.011363
0.011262	0.011195	0.011329	0.011262	0.011195	0.011264	0.011463
0.011362	0.011295	0.011329	0.011362	0.011329	0.011262	0.011463

0.011329	0.011362	0.011362	0.011295	0.011362	0.011597	0.011262
0.011295	0.011698	0.011295	0.011329	0.011295	0.011664	0.011232
0.011679	0.011188	0.011261	0.011463	0.011549	0.011455	0.011186
0.011218	0.011463	0.011564	0.011671	0.011532	0.011635	0.011800
0.011700	0.011737	0.011698	0.011915	0.011664	0.012539	0.011700
0.012439	0.012207	0.012341	0.012613	0.011664	0.011363	0.011665
0.012104	0.012005	0.011631	0.011530	0.011598	0.011598	0.011567
0.011463	0.011433	0.011530	0.011497	0.011867	0.011933	0.011935
0.011834	0.011935	0.012038	0.011771	0.012104	0.012110	0.012171
0.012172	0.012206	0.012118	0.012172	0.012173	0.011396	0.011699
0.011767	0.012270	0.011772	0.012001	0.012474	0.011536	0.011430
0.011396	0.011396	0.011867	0.011924	0.012010	0.011664	0.011397
S066	0.011900	0.011900	0.012236	0.012000	0.012000	0.012068
0.011909	0.011841	0.012067	0.011497	0.011564	0.011531	0.011496
0.011530	0.011698	0.011496	0.011530	0.011597	0.011564	0.011530
0.011496	0.011530	0.011631	0.011497	0.011463	0.011530	0.011875
0.011765	0.011530	0.011429	0.011664	0.011530	0.011564	0.011530
0.011564	0.011597	0.011564	0.011564	0.011597	0.011496	0.011731
0.011496	0.011563	0.011597	0.011530	0.011631	0.011664	0.011597
0.011564	0.011732	0.011631	0.011899	0.011564	0.011765	0.011564
0.011530	0.011631	0.011698	0.011765	0.011933	0.011631	0.011731
0.011329	0.011597	0.011564	0.000000	0.012034	0.011564	0.011631
0.011530	0.011631	0.011597	0.011664	0.011597	0.011597	0.011597
0.011496	0.011530	0.011496	0.011597	0.011563	0.011599	0.011664
0.011597	0.011563	0.011563	0.011530	0.011664	0.011429	0.011564
0.011731	0.011664	0.011899	0.011664	0.011564	0.011731	0.011563
0.011653	0.011957	0.011777	0.011490	0.011433	0.011502	0.011690
0.012305	0.011772	0.011513	0.011799	0.011799	0.011597	0.011698
0.011631	0.011597	0.011664	0.011564	0.011664	0.011530	0.011597
0.011563	0.011664	0.011866	0.011731	0.011631	0.011597	0.011463
0.011497	0.011497	0.011497	0.011530	0.011732	0.011698	0.011631
0.011732	0.011799	0.011530	0.011530	0.011564	0.011564	0.011567
0.011635	0.011564	0.011564	0.011384	0.011530	0.011481	0.011564
0.011500	0.011507	0.011540	0.011509	0.011468	0.011471	0.011533
0.011780	0.011577	0.011542	0.011497	0.011475	0.011632	0.011598
0.011665	0.011599	0.011598	0.011698	0.011631	0.011933	0.011799
0.011832	0.011698	0.011564	0.011665	0.011463	0.011698	0.011497
0.011564	0.011564	0.011530	0.011496	0.011530	0.011497	0.011597
0.011497	0.011597	0.011597	0.011497	0.011599	0.011698	0.011702
0.011631	0.011598	0.011497	0.011431	0.011497	0.011497	0.011430
0.011536	0.011631	0.011429	0.011631	0.011463	0.011564	0.011530
0.011564	0.011530	0.011497	0.011530	0.011564	0.011429	0.011497
0.011530	0.011530	0.011597	0.011530	0.011564	0.011597	0.011833
0.011933	0.011866	0.011933	0.011701	0.011934	0.011564	0.011530
0.011567	0.011747	0.011563	0.011472	0.011942	0.011435	0.011572
0.011943	0.011842	0.011731	0.011933	0.011641	0.011731	0.011698
0.011698	0.011866	0.011735	0.011596	0.011563	0.011597	0.011669
0.011765	0.011667	0.011804	0.011804	0.011799	0.011866	0.011975
0.011703	0.011807	0.011805	0.011669	0.012002	0.011463	0.011667
0.011563	0.011564	0.011463	0.011463	0.011463	0.011530	0.011597
0.011463	0.011530	0.011429	0.011597	0.011463	0.011530	0.011597
0.011530	0.011967	0.011866	0.011899	0.011429	0.011530	0.011564
0.011564	0.011530	0.011597	0.011631	0.011496	0.011496	0.011496
0.012034	0.011664	0.011664	0.011463	0.011563	0.011664	0.011496
0.011496	0.011563	0.011530	0.011631	0.011496	0.011631	0.011698
0.011698	0.011530	0.011530	0.011463	0.011463	0.011631	0.011564
0.011463	0.011563	0.011429	0.011463	0.011530	0.011732	0.011497
0.011463	0.011530	0.011531	0.011429	0.011765	0.011396	0.011597
0.011608	0.011496	0.011799	0.011631	0.011597	0.011530	0.011631
0.011496	0.011765	0.011429	0.011698	0.011496	0.011496	0.011429

0.011597	0.011866	0.011564	0.011631	0.011463	0.011866	0.011496
0.011496	0.011496	0.011530	0.011564	0.011530	0.011631	0.011698
0.011631	0.011664	0.011563	0.011664	0.011362	0.011496	0.011463
0.011496	0.011496	0.011564	0.011496	0.011396	0.011631	0.011496
0.011530	0.011530	0.011664	0.011698	0.011597	0.011463	0.011530
0.011597	0.011530	0.011631	0.011496	0.011631	0.011731	0.012034
0.011530	0.011866	0.011509	0.018011	0.011387	0.011549	0.011862
0.011822	0.011869	0.011584	0.011582	0.011884	0.011889	0.011546
0.011724	0.011732	0.011450	0.011521	0.011450	0.011554	0.011533
0.011764	0.011693	0.011653	0.011523	0.011664	0.011698	0.011703
0.011507	0.011665	0.011664	0.011631	0.011597	0.011598	0.011564
0.011597	0.011530	0.011564	0.011597	0.011564	0.011497	0.011530
0.011530	0.011597	0.011497	0.011597	0.011631	0.011597	0.011564
0.011597	0.011564	0.011900	0.011900	0.011631	0.011665	0.011698
0.011463	0.011463	0.011497	0.011530	0.011463	0.011698	0.011698
0.011732	0.011731	0.011664	0.011765	0.011933	0.011767	0.011530
0.011597	0.011665	0.011631	0.011530	0.011597	0.011564	0.011597
0.011665	0.011665	0.011698	0.011598	0.011498	0.011765	0.011665
0.011602	0.011497	0.011597	0.011698	0.011597	0.011732	0.011765
0.011933	0.011641	0.011731	0.011698	0.011698	0.011866	0.011497
0.011667	0.011564	0.011530	0.011530	0.011597	0.011497	0.011597
0.011631	0.011597	0.011564	0.011597	0.011564	0.011900	0.011900
0.011631	0.011665	0.011731	0.011664	0.012305	0.011739	0.011658
0.011658	0.011684	0.011798	0.011684	0.011795	0.011715	0.011681
0.012262	0.011579	0.011711	0.011648	0.011921	0.011774	0.011809
0.012284	0.011776	0.011838	0.011899	0.011765	0.011765	0.011804
0.011463	0.011564	0.011765	0.011731	0.011736	0.011731	0.011700
0.011563	0.011429	0.011463	0.011564	0.011396	0.011530	0.011429
0.011496	0.011530	0.011496	0.011463	0.011564	0.011496	0.011530
0.011530	0.011463	0.011530	0.011564	0.011463	0.011597	0.011631
0.011530	0.011597	0.011530	0.011530	0.011530	0.011933	0.011563
0.011617	0.011530	0.011766	0.011773	0.011564	0.011631	0.011631
0.011631	0.011496	0.011530	0.011496	0.011664	0.011664	0.011597
0.011597	0.011731	0.011899	0.011631	0.011832	0.011832	0.011698
0.011530	0.011597	0.011631	0.012034	0.011598	0.011564	0.011597
0.011631	0.011563	0.011563	0.011866	0.011732	0.011832	0.011564
0.011664	0.011731	0.011597	0.011664	0.011631	0.011496	0.011832
0.011597	0.011564	0.011563	0.011731	0.011564	0.011664	0.011899
0.011597	0.011977	0.011967	0.011751	0.011511	0.011638	0.011525
0.011445	0.011995	0.011482	0.011685	0.011499	0.011569	0.011538
0.011723	0.011323	0.011505	0.011390	0.011383	0.011529	0.011628
0.011625	0.011493	0.011624	0.011553	0.011599	0.011732	0.011799
0.011799	0.011665	0.011770	0.011772	0.011908	0.011944	0.011597
0.011832	0.011698	0.011564	0.011665	0.011463	0.011698	0.011497
0.011564	0.011564	0.011530	0.011496	0.011530	0.011497	0.011597
0.011584	0.011585	0.011540	0.011608	0.011744	0.011641	0.011709
0.011708	0.011632	0.011429	0.011420	0.011568	0.011738	0.011739
0.011531	0.011602	0.011835	0.011804	0.011733	0.011767	0.011698
0.011631	0.011698	0.011698	0.011597	0.012169	0.011765	0.011832
0.011866	0.011915	0.011572	0.011649	0.011530	0.011732	0.011698
0.011733	0.011765	0.011765	0.011765	0.011886	0.011899	0.011546
0.011432	0.011406	0.011537	0.011502	0.011607	0.011471	0.011774
0.011575	0.011630	0.011489	0.011622	0.011564	0.011642	0.011463
0.011497	0.011497	0.011497	0.011530	0.011732	0.011698	0.011631
0.011732	0.011799	0.011530	0.011530	0.011564	0.011564	0.011567
0.011635	0.011564	0.011564	0.011599	0.011732	0.011799	0.011799
0.011665	0.011770	0.011772	0.011908	0.011944	0.011597	0.011765
0.011698	0.011698	0.011534	0.011721	0.011651	0.011673	0.011551
0.011611	0.011926	0.011653	0.011604	0.011429	0.011631	0.011497
0.011597	0.011597	0.011497	0.011599	0.011698	0.011702	0.011598

0.011497	0.011431	0.011497	0.011497	0.011430	0.011536	0.011631
0.011597	0.011530	0.011564	0.011597	0.011564	0.011497	0.011698
0.011564	0.011597	0.011597	0.011530	0.011530	0.011765	0.011496
0.011530	0.011933	0.011530	0.011496	0.011530	0.011899	0.011489
0.011903	0.011485	0.011561	0.011745	0.011793	0.011706	0.011436
0.011480	0.011698	0.011799	0.011909	0.011767	0.011906	0.012036
0.011935	0.011973	0.011933	0.012188	0.011899	0.012775	0.011936
0.012675	0.012443	0.012577	0.012849	0.011899	0.011598	0.011900
0.012339	0.012275	0.011866	0.011765	0.011834	0.011834	0.011804
0.011698	0.011705	0.011765	0.011733	0.012103	0.012169	0.012170
0.012069	0.012170	0.012274	0.012041	0.012340	0.012347	0.012407
0.012408	0.012441	0.012357	0.012408	0.012409	0.011631	0.011934
0.012002	0.012505	0.012010	0.012236	0.012710	0.011773	0.011665
0.011631	0.011631	0.012102	0.012212	0.012221	0.011967	0.011633
S067	0.012135	0.012135	0.012203	0.011967	0.011967	0.012034
0.011468	0.011400	0.012034	0.011732	0.011799	0.011767	0.011732
0.011698	0.011933	0.011664	0.011765	0.011832	0.011799	0.011765
0.011732	0.011765	0.011597	0.011732	0.011766	0.011765	0.011434
0.011732	0.011765	0.011664	0.011631	0.011832	0.011799	0.011833
0.011799	0.011900	0.011799	0.011799	0.011832	0.011799	0.011967
0.011664	0.011799	0.011832	0.011765	0.011799	0.011900	0.011832
0.011799	0.011967	0.011867	0.011463	0.011799	0.012068	0.011799
0.011765	0.011799	0.011933	0.012068	0.011564	0.011799	0.011900
0.011832	0.011900	0.011799	0.012034	0.000000	0.011799	0.011866
0.011765	0.011866	0.011833	0.011900	0.011832	0.011832	0.011832
0.011732	0.011765	0.011732	0.011765	0.011799	0.011835	0.011900
0.011833	0.011799	0.011866	0.011765	0.011631	0.011732	0.011799
0.011967	0.011900	0.011664	0.011967	0.011799	0.012034	0.011799
0.011988	0.011434	0.011966	0.011646	0.011657	0.011797	0.011533
0.012195	0.011416	0.011637	0.012034	0.012034	0.011765	0.011664
0.011866	0.011832	0.011900	0.011799	0.011900	0.011832	0.011832
0.011799	0.011832	0.012102	0.011967	0.011866	0.011833	0.011766
0.011732	0.011665	0.011799	0.011766	0.011699	0.011665	0.011598
0.011699	0.011766	0.011765	0.011765	0.011799	0.011799	0.011803
0.011872	0.011799	0.011799	0.011600	0.011675	0.011691	0.011800
0.011772	0.011713	0.011688	0.011716	0.011706	0.011711	0.011770
0.011600	0.011784	0.011784	0.011733	0.011683	0.011868	0.011767
0.011834	0.011836	0.011834	0.011933	0.011866	0.012236	0.011968
0.011799	0.011867	0.011799	0.011833	0.011698	0.011665	0.011732
0.011799	0.011799	0.011766	0.011732	0.011765	0.011732	0.011833
0.011799	0.011900	0.011833	0.011732	0.011836	0.011934	0.011939
0.011934	0.011833	0.011732	0.011667	0.011799	0.011732	0.011665
0.011672	0.011934	0.011732	0.011934	0.011698	0.011799	0.011766
0.011867	0.011765	0.011799	0.011766	0.011799	0.011665	0.011799
0.011833	0.011766	0.011833	0.011765	0.011799	0.011833	0.012068
0.011497	0.011429	0.011362	0.011937	0.011564	0.011799	0.011766
0.011736	0.012072	0.011792	0.011712	0.011501	0.011674	0.011887
0.011502	0.011707	0.011967	0.012169	0.011880	0.012034	0.012001
0.011933	0.012101	0.011984	0.011843	0.011899	0.011833	0.011906
0.012001	0.011904	0.012041	0.012110	0.012034	0.012102	0.012213
0.011941	0.012113	0.011975	0.011974	0.012238	0.011698	0.011903
0.011866	0.011799	0.011698	0.011698	0.011765	0.011765	0.011832
0.011765	0.011833	0.011732	0.011765	0.011765	0.011765	0.011832
0.011765	0.011530	0.011295	0.011329	0.011664	0.011765	0.011799
0.011799	0.011698	0.011833	0.011799	0.011732	0.011732	0.011732
0.011597	0.011832	0.011900	0.011698	0.011799	0.011967	0.011732
0.011732	0.011799	0.011765	0.011933	0.011732	0.011665	0.011799
0.011732	0.011765	0.011765	0.011698	0.011698	0.011866	0.011799
0.011698	0.011799	0.011665	0.011698	0.011833	0.011632	0.011732
0.011699	0.011832	0.011766	0.011665	0.011396	0.011665	0.011833

0.011917	0.011732	0.011362	0.011866	0.011833	0.011765	0.011866
0.011732	0.012068	0.011664	0.011530	0.011664	0.011664	0.011664
0.011900	0.011766	0.011799	0.011866	0.011698	0.011295	0.011664
0.011732	0.011732	0.011765	0.011866	0.011765	0.011866	0.011933
0.011933	0.011900	0.011799	0.011900	0.011866	0.011732	0.011698
0.011732	0.011732	0.011799	0.011664	0.011698	0.011866	0.011732
0.011698	0.011765	0.011900	0.011866	0.011832	0.011765	0.011766
0.011832	0.011765	0.011866	0.011732	0.011866	0.011967	0.011664
0.011765	0.011564	0.011785	0.018201	0.011388	0.011855	0.011444
0.011406	0.011412	0.011951	0.011641	0.011500	0.011475	0.011850
0.011725	0.011549	0.011607	0.011773	0.011646	0.011702	0.011749
0.011342	0.011545	0.011431	0.011523	0.011900	0.011934	0.011940
0.011816	0.011900	0.011900	0.011799	0.011833	0.011833	0.011800
0.011833	0.011765	0.011799	0.011833	0.011799	0.011732	0.011765
0.011765	0.011833	0.011732	0.011833	0.011866	0.011900	0.011799
0.011765	0.011732	0.011530	0.011530	0.011866	0.011901	0.011934
0.011698	0.011698	0.011732	0.011833	0.011698	0.011934	0.011934
0.011967	0.011967	0.011900	0.012001	0.011564	0.012004	0.011833
0.011833	0.011900	0.011934	0.011766	0.011833	0.011799	0.011833
0.011901	0.011900	0.011934	0.011901	0.011801	0.012001	0.011967
0.011839	0.011732	0.011833	0.011934	0.011833	0.011900	0.012000
0.012169	0.011880	0.012034	0.012001	0.011933	0.012101	0.011732
0.011903	0.011800	0.011765	0.011765	0.011833	0.011732	0.011833
0.011866	0.011900	0.011799	0.011765	0.011732	0.011530	0.011530
0.011866	0.011901	0.011967	0.011900	0.012474	0.011989	0.011905
0.011975	0.011927	0.012045	0.011926	0.012041	0.011957	0.011888
0.012435	0.011822	0.011951	0.011891	0.012163	0.012013	0.012116
0.012455	0.011965	0.012075	0.012135	0.012001	0.011732	0.012041
0.011698	0.011866	0.012000	0.011967	0.011973	0.011698	0.011667
0.011799	0.011732	0.011765	0.011799	0.011698	0.011765	0.011664
0.011732	0.011765	0.011732	0.011698	0.011799	0.011732	0.011765
0.011765	0.011698	0.011765	0.011799	0.011698	0.011900	0.011933
0.011833	0.011833	0.011832	0.011833	0.011765	0.011564	0.011799
0.011582	0.011765	0.011733	0.011739	0.011799	0.011799	0.011866
0.011866	0.011799	0.011765	0.011799	0.011900	0.011900	0.011832
0.011832	0.011967	0.011463	0.011866	0.012068	0.012068	0.011933
0.011765	0.011832	0.011866	0.011665	0.011833	0.011799	0.011833
0.011866	0.011732	0.011732	0.012035	0.012035	0.012001	0.011799
0.011900	0.011967	0.011832	0.011900	0.011866	0.011732	0.011396
0.011832	0.011799	0.011732	0.011967	0.011799	0.011900	0.011665
0.011832	0.011535	0.011530	0.011589	0.011455	0.011842	0.011473
0.011665	0.011800	0.011693	0.011641	0.011695	0.011518	0.011432
0.011519	0.011324	0.011506	0.011391	0.011384	0.011596	0.011912
0.011927	0.011808	0.011836	0.011835	0.011836	0.011698	0.011766
0.011766	0.011834	0.011940	0.011739	0.012078	0.012121	0.011833
0.011799	0.011867	0.011799	0.011833	0.011698	0.011665	0.011732
0.011799	0.011799	0.011766	0.011732	0.011765	0.011732	0.011833
0.011759	0.011795	0.011780	0.011814	0.011950	0.011915	0.011914
0.011912	0.011868	0.011614	0.011600	0.011772	0.011942	0.011943
0.011767	0.011806	0.012071	0.012041	0.011969	0.012003	0.011933
0.011866	0.011933	0.011933	0.011832	0.011799	0.012001	0.012068
0.012102	0.011345	0.011743	0.011892	0.011765	0.011967	0.011933
0.011969	0.012000	0.012001	0.012001	0.012083	0.012135	0.011789
0.011669	0.011684	0.011775	0.011740	0.011778	0.011695	0.011332
0.011815	0.011587	0.011823	0.011847	0.011800	0.011848	0.011766
0.011732	0.011665	0.011799	0.011766	0.011699	0.011665	0.011598
0.011699	0.011766	0.011765	0.011765	0.011799	0.011799	0.011803
0.011872	0.011799	0.011799	0.011836	0.011698	0.011766	0.011766
0.011834	0.011940	0.011739	0.012078	0.012121	0.011833	0.012001
0.011933	0.011933	0.011771	0.011931	0.011895	0.011912	0.011796

0.011922	0.012063	0.011992	0.011842	0.011732	0.011934	0.011799
0.011900	0.011833	0.011732	0.011836	0.011934	0.011939	0.011833
0.011732	0.011667	0.011799	0.011732	0.011665	0.011672	0.011934
0.011833	0.011765	0.011799	0.011833	0.011799	0.011732	0.011665
0.011799	0.011833	0.011832	0.011765	0.011832	0.012068	0.011664
0.011765	0.011564	0.011765	0.011799	0.011765	0.011463	0.011660
0.011904	0.011528	0.011411	0.011584	0.011671	0.011635	0.011478
0.011375	0.011665	0.011766	0.011876	0.011734	0.011873	0.012002
0.011902	0.011940	0.011900	0.012155	0.011866	0.012742	0.011903
0.012642	0.012410	0.012544	0.012817	0.011799	0.011766	0.011867
0.012508	0.012242	0.011833	0.011732	0.011800	0.011800	0.011737
0.011665	0.011671	0.011732	0.011700	0.012070	0.012136	0.012137
0.012037	0.012137	0.012241	0.012008	0.012509	0.012517	0.012576
0.012577	0.012611	0.012563	0.012577	0.012579	0.011866	0.012103
0.012171	0.012674	0.012180	0.012136	0.012609	0.011672	0.011565
0.011463	0.011530	0.012069	0.012130	0.012187	0.011866	0.011600
S068 0.011664	0.011664	0.012001	0.011765	0.011765	0.011832	
0.011671	0.011603	0.011765	0.011262	0.011329	0.011296	0.011262
0.011295	0.011396	0.011262	0.011295	0.011362	0.011329	0.011295
0.011262	0.011295	0.011396	0.011262	0.011295	0.011295	0.011637
0.011530	0.011295	0.011194	0.011429	0.011362	0.011329	0.011362
0.011329	0.011429	0.011329	0.011329	0.011362	0.011329	0.011497
0.011262	0.011329	0.011362	0.011295	0.011396	0.011429	0.011362
0.011329	0.011497	0.011396	0.011664	0.011329	0.011597	0.011329
0.011295	0.011396	0.011463	0.011597	0.011698	0.011396	0.011496
0.011362	0.011429	0.011329	0.011564	0.011799	0.000000	0.011396
0.011295	0.011396	0.011362	0.011429	0.011362	0.011362	0.011362
0.011262	0.011295	0.011262	0.011362	0.011329	0.011363	0.011429
0.011362	0.011329	0.011396	0.011295	0.011429	0.011262	0.011329
0.011496	0.011429	0.011664	0.011497	0.011329	0.011564	0.011262
0.011505	0.011733	0.011551	0.011256	0.011247	0.011355	0.011454
0.012085	0.011569	0.011266	0.011564	0.011564	0.011362	0.011463
0.011396	0.011362	0.011429	0.011329	0.011429	0.011362	0.011362
0.011329	0.011429	0.011631	0.011429	0.011396	0.011362	0.011295
0.011262	0.011262	0.011329	0.011295	0.011497	0.011463	0.011396
0.011497	0.011564	0.011295	0.011295	0.011329	0.011329	0.011330
0.011398	0.011329	0.011329	0.011204	0.011277	0.011236	0.011329
0.011331	0.011266	0.011318	0.011302	0.011230	0.011266	0.011297
0.011564	0.011336	0.011370	0.011262	0.011268	0.011397	0.011363
0.011430	0.011364	0.011363	0.011463	0.011396	0.011765	0.011564
0.011597	0.011463	0.011329	0.011430	0.011228	0.011463	0.011262
0.011329	0.011329	0.011295	0.011262	0.011295	0.011262	0.011362
0.011329	0.011430	0.011362	0.011262	0.011296	0.011463	0.011466
0.011463	0.011362	0.011262	0.011195	0.011329	0.011262	0.011195
0.011298	0.011463	0.011262	0.011463	0.011228	0.011329	0.011295
0.011396	0.011295	0.011329	0.011295	0.011329	0.011195	0.011329
0.011362	0.011295	0.011362	0.011295	0.011329	0.011362	0.011597
0.011698	0.011631	0.011698	0.011465	0.011698	0.011329	0.011295
0.011330	0.011586	0.011380	0.011232	0.011637	0.011197	0.011257
0.011705	0.011604	0.011496	0.011698	0.011402	0.011564	0.011530
0.011463	0.011631	0.011451	0.011307	0.011354	0.011362	0.011432
0.011530	0.011397	0.011567	0.011601	0.011564	0.011631	0.011704
0.011466	0.011603	0.011568	0.011500	0.011767	0.011228	0.011431
0.011396	0.011329	0.011228	0.011228	0.011295	0.011228	0.011362
0.011295	0.011362	0.011262	0.011362	0.011295	0.011295	0.011362
0.011295	0.011731	0.011631	0.011664	0.011194	0.011295	0.011329
0.011329	0.011295	0.011362	0.011396	0.011262	0.011262	0.011262
0.011799	0.011429	0.011429	0.011228	0.011329	0.011497	0.011262
0.011262	0.011329	0.011295	0.011463	0.011262	0.011396	0.011530
0.011463	0.011295	0.011295	0.011228	0.011228	0.011396	0.011329

0.011228	0.011329	0.011195	0.011228	0.011362	0.011497	0.011228
0.011228	0.011362	0.011295	0.011195	0.011530	0.011195	0.011295
0.011403	0.011262	0.011564	0.011396	0.011429	0.011295	0.011396
0.011262	0.011597	0.011194	0.011463	0.011262	0.011262	0.011194
0.011429	0.011631	0.011329	0.011396	0.011228	0.011631	0.011262
0.011262	0.011262	0.011295	0.011396	0.011295	0.011396	0.011463
0.011463	0.011429	0.011329	0.011429	0.011396	0.011262	0.011228
0.011262	0.011262	0.011329	0.011262	0.011228	0.011329	0.011262
0.011295	0.011295	0.011362	0.011463	0.011362	0.011295	0.011295
0.011362	0.011295	0.011329	0.011262	0.011396	0.011362	0.011799
0.011295	0.011698	0.011302	0.017823	0.011388	0.011427	0.011653
0.011614	0.011658	0.011427	0.011350	0.011719	0.011682	0.011364
0.011582	0.011549	0.011216	0.011311	0.011294	0.011307	0.011318
0.011576	0.011470	0.011468	0.011325	0.011429	0.011463	0.011466
0.011301	0.011429	0.011429	0.011396	0.011362	0.011362	0.011329
0.011362	0.011295	0.011329	0.011362	0.011329	0.011262	0.011295
0.011295	0.011362	0.011262	0.011362	0.011396	0.011429	0.011329
0.011362	0.011329	0.011665	0.011665	0.011396	0.011363	0.011463
0.011228	0.011228	0.011262	0.011362	0.011228	0.011463	0.011463
0.011497	0.011496	0.011429	0.011530	0.011698	0.011532	0.011362
0.011362	0.011430	0.011463	0.011295	0.011362	0.011329	0.011362
0.011430	0.011430	0.011463	0.011430	0.011330	0.011530	0.011497
0.011365	0.011262	0.011362	0.011463	0.011362	0.011497	0.011530
0.011698	0.011402	0.011564	0.011530	0.011463	0.011631	0.011262
0.011431	0.011195	0.011295	0.011295	0.011362	0.011262	0.011362
0.011396	0.011429	0.011329	0.011362	0.011329	0.011665	0.011665
0.011396	0.011363	0.011496	0.011429	0.012069	0.011453	0.011377
0.011447	0.011407	0.011517	0.011406	0.011515	0.011439	0.011439
0.011987	0.011302	0.011437	0.011371	0.011645	0.011536	0.011605
0.012012	0.011475	0.011601	0.011664	0.011530	0.011530	0.011567
0.011228	0.011396	0.011530	0.011496	0.011499	0.011496	0.011464
0.011329	0.011262	0.011295	0.011329	0.011228	0.011295	0.011194
0.011329	0.011362	0.011262	0.011228	0.011329	0.011262	0.011295
0.011295	0.011228	0.011295	0.011329	0.011228	0.011429	0.011463
0.011362	0.011429	0.011362	0.011362	0.011295	0.011698	0.011329
0.011338	0.011295	0.011530	0.011501	0.011396	0.011396	0.011396
0.011396	0.011329	0.011295	0.011329	0.011228	0.011429	0.011362
0.011362	0.011497	0.011664	0.011396	0.011597	0.011597	0.011463
0.011295	0.011362	0.011396	0.011799	0.011362	0.011396	0.011362
0.011396	0.011329	0.011329	0.011631	0.011564	0.011597	0.011329
0.011429	0.011497	0.011362	0.011429	0.011396	0.011262	0.011597
0.011362	0.011329	0.011329	0.011497	0.011329	0.011429	0.011664
0.011362	0.011739	0.011732	0.011548	0.011342	0.011434	0.011267
0.011225	0.011761	0.011271	0.011507	0.011353	0.011467	0.011485
0.011570	0.011324	0.011450	0.011324	0.011330	0.011462	0.011425
0.011437	0.011336	0.011412	0.011342	0.011364	0.011497	0.011564
0.011564	0.011430	0.011533	0.011535	0.011670	0.011661	0.011362
0.011597	0.011463	0.011329	0.011430	0.011228	0.011463	0.011262
0.011329	0.011329	0.011295	0.011262	0.011295	0.011262	0.011362
0.011340	0.011340	0.011334	0.011368	0.011505	0.011470	0.011470
0.011469	0.011396	0.011134	0.011204	0.011264	0.011501	0.011501
0.011296	0.011365	0.011599	0.011567	0.011497	0.011464	0.011463
0.011396	0.011463	0.011463	0.011362	0.011933	0.011530	0.011597
0.011631	0.011701	0.011333	0.011407	0.011295	0.011497	0.011463
0.011497	0.011530	0.011530	0.011530	0.011650	0.011664	0.011268
0.011162	0.011164	0.011264	0.011230	0.011333	0.011172	0.011502
0.011300	0.011455	0.011156	0.011322	0.011329	0.011402	0.011295
0.011262	0.011262	0.011329	0.011295	0.011497	0.011463	0.011396
0.011497	0.011564	0.011295	0.011295	0.011329	0.011329	0.011330
0.011398	0.011329	0.011329	0.011364	0.011497	0.011564	0.011564

0.011430	0.011533	0.011535	0.011670	0.011661	0.011362	0.011530
0.011463	0.011463	0.011297	0.011478	0.011269	0.011435	0.011305
0.011473	0.011698	0.011441	0.011366	0.011262	0.011463	0.011329
0.011430	0.011362	0.011262	0.011296	0.011463	0.011466	0.011362
0.011262	0.011195	0.011329	0.011262	0.011195	0.011298	0.011463
0.011362	0.011295	0.011329	0.011362	0.011329	0.011262	0.011463
0.011329	0.011362	0.011362	0.011295	0.011362	0.011597	0.011262
0.011295	0.011698	0.011295	0.011329	0.011295	0.011664	0.011274
0.011791	0.011273	0.011361	0.011503	0.011589	0.011491	0.011227
0.011323	0.011463	0.011564	0.011671	0.011531	0.011669	0.011800
0.011699	0.011703	0.011698	0.011949	0.011664	0.012539	0.011700
0.012439	0.012207	0.012341	0.012613	0.011664	0.011362	0.011665
0.012103	0.012038	0.011631	0.011530	0.011598	0.011598	0.011534
0.011463	0.011467	0.011530	0.011497	0.011867	0.011933	0.011935
0.011834	0.011934	0.012038	0.011804	0.012104	0.012110	0.012171
0.012172	0.012205	0.012152	0.012172	0.012173	0.011396	0.011699
0.011767	0.012270	0.011772	0.012001	0.012474	0.011536	0.011430
0.011396	0.011396	0.011867	0.011883	0.012010	0.011732	0.011397
S069	0.011732	0.011732	0.012068	0.011832	0.011832	0.011900
0.011739	0.011671	0.011900	0.011329	0.011396	0.011363	0.011329
0.011362	0.011530	0.011329	0.011362	0.011429	0.011396	0.011362
0.011329	0.011362	0.011463	0.011329	0.011363	0.011362	0.011705
0.011597	0.011362	0.011262	0.011496	0.011429	0.011396	0.011429
0.011329	0.011497	0.011396	0.011396	0.011429	0.011396	0.011564
0.011329	0.011396	0.011429	0.011362	0.011463	0.011496	0.011362
0.011396	0.011564	0.011463	0.011732	0.011396	0.011664	0.011396
0.011362	0.011463	0.011530	0.011664	0.011765	0.011463	0.011564
0.011429	0.011497	0.011396	0.011631	0.011866	0.011396	0.000000
0.011362	0.011463	0.011429	0.011497	0.011429	0.011429	0.011429
0.011329	0.011362	0.011329	0.011429	0.011396	0.011430	0.011496
0.011429	0.011396	0.011463	0.011362	0.011496	0.011329	0.011396
0.011564	0.011496	0.011732	0.011564	0.011396	0.011631	0.011396
0.011579	0.011770	0.011626	0.011295	0.011321	0.011428	0.011493
0.012158	0.011518	0.011307	0.011631	0.011631	0.011429	0.011530
0.011463	0.011429	0.011496	0.011396	0.011497	0.011429	0.011429
0.011396	0.011496	0.011698	0.011564	0.011463	0.011430	0.011362
0.011329	0.011329	0.011396	0.011362	0.011564	0.011530	0.011463
0.011564	0.011631	0.011362	0.011362	0.011396	0.011396	0.011398
0.011466	0.011396	0.011396	0.011276	0.011349	0.011306	0.011396
0.011399	0.011335	0.011392	0.011371	0.011298	0.011334	0.011364
0.011636	0.011405	0.011439	0.011329	0.011337	0.011464	0.011430
0.011497	0.011431	0.011430	0.011530	0.011463	0.011832	0.011631
0.011664	0.011530	0.011396	0.011497	0.011295	0.011530	0.011329
0.011396	0.011396	0.011362	0.011329	0.011362	0.011329	0.011430
0.011396	0.011497	0.011430	0.011329	0.011431	0.011530	0.011533
0.011530	0.011430	0.011329	0.011263	0.011396	0.011329	0.011262
0.011366	0.011530	0.011329	0.011530	0.011295	0.011396	0.011362
0.011463	0.011362	0.011396	0.011362	0.011396	0.011262	0.011396
0.011430	0.011362	0.011430	0.011362	0.011396	0.011430	0.011665
0.011765	0.011698	0.011766	0.011532	0.011766	0.011396	0.011362
0.011398	0.011707	0.011472	0.011300	0.011773	0.011265	0.011414
0.011773	0.011672	0.011564	0.011765	0.011470	0.011631	0.011597
0.011530	0.011698	0.011558	0.011431	0.011479	0.011430	0.011500
0.011597	0.011499	0.011635	0.011703	0.011631	0.011698	0.011806
0.011534	0.011705	0.011636	0.011567	0.011834	0.011295	0.011498
0.011463	0.011396	0.011295	0.011295	0.011362	0.011362	0.011429
0.011362	0.011429	0.011329	0.011429	0.011362	0.011362	0.011429
0.011362	0.011799	0.011698	0.011732	0.011262	0.011362	0.011396
0.011396	0.011362	0.011429	0.011463	0.011329	0.011329	0.011329
0.011866	0.011496	0.011497	0.011295	0.011396	0.011564	0.011329

0.011329	0.011396	0.011362	0.011530	0.011329	0.011463	0.011597
0.011530	0.011362	0.011362	0.011295	0.011295	0.011463	0.011396
0.011295	0.011396	0.011262	0.011295	0.011429	0.011564	0.011329
0.011295	0.011429	0.011363	0.011262	0.011597	0.011262	0.011429
0.011505	0.011329	0.011631	0.011463	0.011497	0.011362	0.011463
0.011329	0.011664	0.011262	0.011530	0.011329	0.011329	0.011262
0.011496	0.011698	0.011396	0.011463	0.011295	0.011698	0.011329
0.011329	0.011329	0.011362	0.011463	0.011362	0.011463	0.011530
0.011530	0.011497	0.011396	0.011497	0.011463	0.011329	0.011295
0.011329	0.011329	0.011396	0.011329	0.011295	0.011463	0.011329
0.011362	0.011362	0.011496	0.011530	0.011429	0.011362	0.011362
0.011429	0.011362	0.011463	0.011329	0.011463	0.011564	0.011866
0.011362	0.011765	0.011371	0.017899	0.011388	0.011488	0.011723
0.011683	0.011729	0.011427	0.011350	0.011775	0.011751	0.011485
0.011629	0.011671	0.011255	0.011353	0.011372	0.011455	0.011361
0.011576	0.011544	0.011542	0.011391	0.011497	0.011530	0.011534
0.011404	0.011497	0.011497	0.011463	0.011429	0.011430	0.011396
0.011430	0.011362	0.011396	0.011430	0.011396	0.011329	0.011362
0.011362	0.011430	0.011329	0.011430	0.011463	0.011497	0.011396
0.011429	0.011396	0.011732	0.011732	0.011463	0.011497	0.011530
0.011295	0.011295	0.011329	0.011430	0.011295	0.011530	0.011530
0.011564	0.011564	0.011497	0.011597	0.011766	0.011599	0.011430
0.011430	0.011497	0.011530	0.011362	0.011430	0.011396	0.011430
0.011497	0.011497	0.011530	0.011497	0.011397	0.011597	0.011564
0.011433	0.011329	0.011430	0.011530	0.011430	0.011564	0.011597
0.011765	0.011470	0.011631	0.011597	0.011530	0.011698	0.011329
0.011498	0.011396	0.011362	0.011362	0.011430	0.011329	0.011430
0.011463	0.011497	0.011396	0.011429	0.011396	0.011732	0.011732
0.011463	0.011497	0.011564	0.011496	0.012137	0.011560	0.011482
0.011552	0.011511	0.011622	0.011510	0.011620	0.011543	0.011508
0.012090	0.011406	0.011540	0.011475	0.011749	0.011604	0.011707
0.012114	0.011588	0.011669	0.011732	0.011597	0.011597	0.011635
0.011295	0.011463	0.011597	0.011564	0.011567	0.011564	0.011532
0.011396	0.011329	0.011362	0.011396	0.011295	0.011362	0.011262
0.011396	0.011429	0.011329	0.011295	0.011396	0.011329	0.011362
0.011362	0.011295	0.011362	0.011396	0.011295	0.011497	0.011530
0.011429	0.011497	0.011429	0.011362	0.011362	0.011765	0.011396
0.011443	0.011362	0.011598	0.011603	0.011463	0.011463	0.011463
0.011463	0.011396	0.011362	0.011396	0.011497	0.011497	0.011429
0.011429	0.011564	0.011732	0.011463	0.011665	0.011665	0.011530
0.011362	0.011429	0.011463	0.011866	0.011430	0.011463	0.011429
0.011463	0.011396	0.011396	0.011698	0.011631	0.011665	0.011396
0.011497	0.011564	0.011429	0.011497	0.011463	0.011329	0.011664
0.011429	0.011396	0.011396	0.011564	0.011396	0.011496	0.011732
0.011429	0.011807	0.011799	0.011589	0.011342	0.011502	0.011421
0.011335	0.011800	0.011341	0.011552	0.011353	0.011467	0.011485
0.011570	0.011324	0.011450	0.011324	0.011330	0.011529	0.011466
0.011512	0.011414	0.011483	0.011412	0.011431	0.011564	0.011631
0.011631	0.011497	0.011601	0.011603	0.011704	0.011767	0.011429
0.011664	0.011530	0.011396	0.011497	0.011295	0.011530	0.011329
0.011396	0.011396	0.011362	0.011329	0.011362	0.011329	0.011430
0.011410	0.011410	0.011403	0.011437	0.011573	0.011538	0.011538
0.011537	0.011464	0.011282	0.011276	0.011399	0.011568	0.011569
0.011363	0.011433	0.011666	0.011635	0.011565	0.011599	0.011530
0.011463	0.011530	0.011530	0.011429	0.012001	0.011597	0.011665
0.011698	0.011772	0.011401	0.011476	0.011362	0.011564	0.011530
0.011565	0.011597	0.011597	0.011597	0.011729	0.011732	0.011372
0.011263	0.011268	0.011367	0.011332	0.011436	0.011284	0.011604
0.011403	0.011586	0.011346	0.011435	0.011396	0.011471	0.011362
0.011329	0.011329	0.011396	0.011362	0.011564	0.011530	0.011463

0.011564	0.011631	0.011362	0.011362	0.011396	0.011396	0.011398
0.011466	0.011396	0.011396	0.011431	0.011564	0.011631	0.011631
0.011497	0.011601	0.011603	0.011704	0.011767	0.011429	0.011597
0.011530	0.011530	0.011365	0.011547	0.011477	0.011503	0.011376
0.011542	0.011744	0.011483	0.011434	0.011329	0.011530	0.011396
0.011497	0.011430	0.011329	0.011431	0.011530	0.011533	0.011430
0.011329	0.011263	0.011396	0.011329	0.011262	0.011366	0.011530
0.011430	0.011362	0.011396	0.011430	0.011396	0.011329	0.011530
0.011396	0.011429	0.011429	0.011362	0.011429	0.011664	0.011329
0.011362	0.011765	0.011362	0.011396	0.011362	0.011732	0.011317
0.011791	0.011315	0.011311	0.011543	0.011630	0.011563	0.011311
0.011270	0.011530	0.011631	0.011739	0.011599	0.011737	0.011867
0.011767	0.011804	0.011765	0.012017	0.011732	0.012606	0.011768
0.012507	0.012274	0.012409	0.012680	0.011731	0.011430	0.011732
0.012171	0.012106	0.011698	0.011597	0.011665	0.011665	0.011635
0.011530	0.011535	0.011597	0.011565	0.011935	0.012001	0.012002
0.011901	0.012002	0.012106	0.011872	0.012171	0.012144	0.012238
0.012239	0.012273	0.012221	0.012239	0.012241	0.011463	0.011766
0.011834	0.012337	0.011840	0.012068	0.012541	0.011604	0.011497
0.011463	0.011463	0.011934	0.012006	0.012081	0.011799	0.011465
S070	0.011631	0.011631	0.011967	0.011732	0.011732	0.011799
0.011637	0.011569	0.011799	0.011228	0.011295	0.011262	0.011228
0.011262	0.011429	0.011228	0.011262	0.011329	0.011295	0.011262
0.011228	0.011262	0.011362	0.011228	0.011262	0.011262	0.011603
0.011496	0.011262	0.011161	0.011396	0.011329	0.011295	0.011329
0.011295	0.011396	0.011295	0.011295	0.011329	0.011295	0.011463
0.011228	0.011295	0.011329	0.011262	0.011362	0.011396	0.011329
0.011295	0.011463	0.011362	0.011631	0.011295	0.011564	0.011295
0.011262	0.011362	0.011429	0.011564	0.011664	0.011362	0.011463
0.011329	0.011396	0.011295	0.011530	0.011765	0.011295	0.011362
0.000000	0.011362	0.011329	0.011396	0.011329	0.011329	0.011329
0.011228	0.011262	0.011228	0.011329	0.011295	0.011329	0.011396
0.011329	0.011295	0.011362	0.011262	0.011396	0.011161	0.011295
0.011463	0.011396	0.011631	0.011463	0.011295	0.011530	0.011295
0.011468	0.011695	0.011513	0.011216	0.011210	0.011318	0.011415
0.012048	0.011518	0.011225	0.011530	0.011530	0.011329	0.011429
0.011362	0.011329	0.011396	0.011295	0.011396	0.011329	0.011329
0.011295	0.011396	0.011597	0.011463	0.011362	0.011329	0.011262
0.011228	0.011228	0.011295	0.011262	0.011463	0.011430	0.011362
0.011463	0.011530	0.011262	0.011262	0.011295	0.011295	0.011297
0.011364	0.011295	0.011295	0.011168	0.011241	0.011200	0.011296
0.011297	0.011232	0.011282	0.011267	0.011196	0.011232	0.011263
0.011528	0.011302	0.011336	0.011228	0.011233	0.011363	0.011329
0.011397	0.011330	0.011329	0.011429	0.011362	0.011732	0.011530
0.011564	0.011430	0.011295	0.011396	0.011195	0.011429	0.011228
0.011295	0.011295	0.011262	0.011228	0.011262	0.011228	0.011329
0.011295	0.011396	0.011329	0.011228	0.011330	0.011430	0.011432
0.011430	0.011329	0.011228	0.011161	0.011295	0.011228	0.011161
0.011264	0.011430	0.011228	0.011430	0.011195	0.011295	0.011262
0.011362	0.011262	0.011295	0.011262	0.011295	0.011161	0.011295
0.011329	0.011262	0.011329	0.011262	0.011295	0.011329	0.011564
0.011664	0.011597	0.011665	0.011431	0.011665	0.011295	0.011262
0.011297	0.011586	0.011380	0.011197	0.011671	0.011163	0.011296
0.011671	0.011570	0.011463	0.011664	0.011368	0.011530	0.011497
0.011429	0.011597	0.011415	0.011266	0.011312	0.011329	0.011398
0.011497	0.011397	0.011533	0.011601	0.011530	0.011597	0.011704
0.011432	0.011603	0.011534	0.011466	0.011733	0.011194	0.011397
0.011362	0.011295	0.011194	0.011194	0.011262	0.011262	0.011329
0.011262	0.011329	0.011228	0.011329	0.011262	0.011262	0.011329
0.011262	0.011698	0.011597	0.011631	0.011161	0.011262	0.011295

0.011295	0.011262	0.011329	0.011362	0.011228	0.011228	0.011228
0.011765	0.011396	0.011396	0.011194	0.011295	0.011463	0.011228
0.011228	0.011295	0.011262	0.011429	0.011228	0.011362	0.011496
0.011429	0.011262	0.011262	0.011194	0.011194	0.011362	0.011295
0.011194	0.011295	0.011161	0.011195	0.011329	0.011463	0.011228
0.011195	0.011329	0.011262	0.011161	0.011496	0.011161	0.011329
0.011368	0.011228	0.011530	0.011362	0.011329	0.011262	0.011362
0.011228	0.011564	0.011161	0.011429	0.011228	0.011228	0.011161
0.011396	0.011598	0.011295	0.011362	0.011194	0.011597	0.011228
0.011228	0.011228	0.011262	0.011362	0.011262	0.011362	0.011429
0.011429	0.011396	0.011295	0.011396	0.011362	0.011228	0.011194
0.011228	0.011228	0.011295	0.011228	0.011194	0.011362	0.011228
0.011262	0.011262	0.011396	0.011429	0.011329	0.011262	0.011262
0.011329	0.011262	0.011362	0.011228	0.011362	0.011463	0.011765
0.011262	0.011664	0.011267	0.017785	0.011257	0.011366	0.011618
0.011579	0.011623	0.011322	0.011292	0.011719	0.011647	0.011303
0.011534	0.011487	0.011177	0.011228	0.011255	0.011307	0.011275
0.011529	0.011433	0.011431	0.011325	0.011396	0.011429	0.011432
0.011301	0.011396	0.011396	0.011362	0.011329	0.011329	0.011295
0.011329	0.011262	0.011295	0.011329	0.011295	0.011228	0.011262
0.011262	0.011329	0.011228	0.011329	0.011362	0.011396	0.011295
0.011329	0.011295	0.011631	0.011631	0.011362	0.011396	0.011430
0.011195	0.011195	0.011228	0.011329	0.011195	0.011430	0.011430
0.011463	0.011463	0.011396	0.011497	0.011665	0.011498	0.011329
0.011329	0.011396	0.011430	0.011262	0.011329	0.011295	0.011329
0.011396	0.011396	0.011429	0.011396	0.011296	0.011497	0.011463
0.011331	0.011228	0.011329	0.011430	0.011329	0.011463	0.011496
0.011664	0.011368	0.011530	0.011497	0.011429	0.011597	0.011228
0.011397	0.011296	0.011262	0.011262	0.011329	0.011228	0.011329
0.011362	0.011396	0.011295	0.011329	0.011295	0.011631	0.011631
0.011362	0.011396	0.011463	0.011396	0.012035	0.011453	0.011341
0.011447	0.011407	0.011482	0.011372	0.011515	0.011439	0.011405
0.011953	0.011302	0.011437	0.011371	0.011645	0.011502	0.011605
0.012012	0.011437	0.011567	0.011631	0.011496	0.011497	0.011533
0.011194	0.011362	0.011496	0.011463	0.011466	0.011463	0.011431
0.011295	0.011228	0.011262	0.011295	0.011194	0.011262	0.011161
0.011295	0.011329	0.011228	0.011194	0.011295	0.011228	0.011262
0.011262	0.011194	0.011262	0.011295	0.011194	0.011396	0.011429
0.011329	0.011329	0.011329	0.011329	0.011262	0.011664	0.011295
0.011304	0.011262	0.011497	0.011501	0.011362	0.011362	0.011362
0.011362	0.011295	0.011262	0.011295	0.011396	0.011396	0.011329
0.011329	0.011463	0.011631	0.011362	0.011564	0.011564	0.011429
0.011262	0.011329	0.011362	0.011765	0.011329	0.011362	0.011329
0.011362	0.011295	0.011295	0.011597	0.011530	0.011564	0.011295
0.011396	0.011463	0.011329	0.011396	0.011362	0.011228	0.011564
0.011329	0.011295	0.011295	0.011463	0.011295	0.011396	0.011631
0.011329	0.011705	0.011698	0.011507	0.011286	0.011400	0.011318
0.011225	0.011722	0.011236	0.011462	0.011304	0.011366	0.011432
0.011519	0.011258	0.011394	0.011258	0.011276	0.011462	0.011385
0.011399	0.011296	0.011377	0.011307	0.011330	0.011463	0.011530
0.011530	0.011396	0.011500	0.011501	0.011636	0.011661	0.011329
0.011564	0.011430	0.011295	0.011396	0.011195	0.011429	0.011228
0.011295	0.011295	0.011262	0.011228	0.011262	0.011228	0.011329
0.011305	0.011305	0.011300	0.011334	0.011471	0.011436	0.011436
0.011435	0.011363	0.011171	0.011168	0.011297	0.011467	0.011467
0.011262	0.011331	0.011565	0.011533	0.011464	0.011498	0.011429
0.011362	0.011429	0.011429	0.011329	0.011900	0.011497	0.011564
0.011597	0.011665	0.011299	0.011372	0.011262	0.011463	0.011429
0.011464	0.011496	0.011496	0.011496	0.011650	0.011631	0.011268
0.011162	0.011164	0.011264	0.011230	0.011333	0.011172	0.011502

0.011300	0.011411	0.011156	0.011285	0.011296	0.011368	0.011262
0.011228	0.011228	0.011295	0.011262	0.011463	0.011430	0.011362
0.011463	0.011530	0.011262	0.011262	0.011295	0.011295	0.011297
0.011364	0.011295	0.011295	0.011330	0.011463	0.011530	0.011530
0.011396	0.011500	0.011501	0.011636	0.011661	0.011329	0.011496
0.011429	0.011429	0.011263	0.011443	0.011373	0.011401	0.011270
0.011439	0.011653	0.011357	0.011332	0.011228	0.011430	0.011295
0.011396	0.011329	0.011228	0.011330	0.011430	0.011432	0.011329
0.011228	0.011161	0.011295	0.011228	0.011161	0.011264	0.011430
0.011329	0.011262	0.011295	0.011329	0.011295	0.011228	0.011430
0.011295	0.011329	0.011329	0.011262	0.011329	0.011564	0.011228
0.000000	0.011664	0.011262	0.011295	0.011262	0.011631	0.011232
0.011735	0.011230	0.011311	0.011463	0.011549	0.011455	0.011186
0.011270	0.011429	0.011530	0.011637	0.011498	0.011635	0.011766
0.011666	0.011703	0.011664	0.011914	0.011631	0.012505	0.011667
0.012406	0.012173	0.012307	0.012579	0.011631	0.011329	0.011631
0.012070	0.012005	0.011597	0.011497	0.011565	0.011565	0.011534
0.011429	0.011433	0.011496	0.011464	0.011834	0.011900	0.011901
0.011800	0.011901	0.012004	0.011770	0.012070	0.012042	0.012137
0.012138	0.012172	0.012118	0.012138	0.012139	0.011362	0.011665
0.011733	0.012236	0.011738	0.011967	0.012440	0.011502	0.011396
0.011362	0.011362	0.011833	0.011842	0.011975	0.011698	0.011363
S071	0.011732	0.011732	0.012068	0.011832	0.011832	0.011900
0.011739	0.011671	0.011899	0.011329	0.011362	0.011363	0.011329
0.011362	0.011530	0.011329	0.011362	0.011429	0.011396	0.011362
0.011329	0.011362	0.011463	0.011329	0.011363	0.011362	0.011705
0.011597	0.011362	0.011262	0.011496	0.011429	0.011396	0.011429
0.011396	0.011496	0.011396	0.011396	0.011429	0.011396	0.011564
0.011329	0.011396	0.011429	0.011362	0.011463	0.011496	0.011429
0.011396	0.011564	0.011463	0.011731	0.011396	0.011664	0.011396
0.011362	0.011463	0.011530	0.011664	0.011765	0.011463	0.011564
0.011429	0.011496	0.011396	0.011631	0.011866	0.011396	0.011463
0.011362	0.000000	0.011429	0.011496	0.011429	0.011429	0.011429
0.011329	0.011362	0.011329	0.011429	0.011396	0.011430	0.011496
0.011429	0.011396	0.011463	0.011362	0.011496	0.011329	0.011396
0.011564	0.011496	0.011731	0.011564	0.011396	0.011631	0.011396
0.011542	0.011770	0.011588	0.011295	0.011247	0.011391	0.011415
0.012085	0.011518	0.011225	0.011631	0.011631	0.011429	0.011530
0.011463	0.011429	0.011496	0.011396	0.011496	0.011429	0.011429
0.011396	0.011496	0.011698	0.011564	0.011463	0.011429	0.011362
0.011329	0.011329	0.011396	0.011362	0.011564	0.011530	0.011463
0.011564	0.011631	0.011362	0.011362	0.011396	0.011396	0.011398
0.011466	0.011396	0.011396	0.011276	0.011349	0.011306	0.011396
0.011399	0.011335	0.011355	0.011371	0.011298	0.011334	0.011364
0.011636	0.011405	0.011439	0.011329	0.011337	0.011464	0.011430
0.011497	0.011431	0.011430	0.011530	0.011463	0.011832	0.011631
0.011664	0.011530	0.011396	0.011497	0.011295	0.011530	0.011329
0.011396	0.011396	0.011362	0.011329	0.011362	0.011329	0.011429
0.011396	0.011497	0.011430	0.011329	0.011431	0.011530	0.011533
0.011530	0.011430	0.011329	0.011263	0.011396	0.011329	0.011262
0.011366	0.011530	0.011329	0.011530	0.011295	0.011396	0.011362
0.011463	0.011362	0.011396	0.011362	0.011396	0.011262	0.011396
0.011430	0.011362	0.011429	0.011362	0.011396	0.011429	0.011665
0.011765	0.011698	0.011765	0.011498	0.011766	0.011396	0.011362
0.011398	0.011667	0.011472	0.011266	0.011773	0.011265	0.011375
0.011773	0.011672	0.011564	0.011765	0.011470	0.011631	0.011597
0.011530	0.011698	0.011522	0.011390	0.011437	0.011429	0.011500
0.011597	0.011499	0.011635	0.011703	0.011631	0.011698	0.011805
0.011534	0.011705	0.011636	0.011567	0.011834	0.011295	0.011498
0.011463	0.011396	0.011295	0.011295	0.011362	0.011362	0.011429

0.011362	0.011429	0.011329	0.011429	0.011362	0.011362	0.011429
0.011362	0.011799	0.011698	0.011731	0.011262	0.011362	0.011396
0.011396	0.011362	0.011429	0.011463	0.011329	0.011329	0.011329
0.011866	0.011496	0.011496	0.011295	0.011396	0.011496	0.011329
0.011329	0.011396	0.011362	0.011530	0.011329	0.011463	0.011597
0.011530	0.011362	0.011362	0.011295	0.011295	0.011463	0.011396
0.011295	0.011396	0.011262	0.011295	0.011429	0.011564	0.011329
0.011295	0.011429	0.011363	0.011262	0.011597	0.011262	0.011429
0.011505	0.011329	0.011631	0.011463	0.011496	0.011362	0.011463
0.011329	0.011664	0.011262	0.011530	0.011329	0.011329	0.011262
0.011496	0.011698	0.011396	0.011463	0.011295	0.011698	0.011329
0.011329	0.011329	0.011362	0.011463	0.011362	0.011463	0.011530
0.011530	0.011496	0.011396	0.011497	0.011463	0.011329	0.011295
0.011329	0.011329	0.011396	0.011329	0.011295	0.011463	0.011329
0.011362	0.011362	0.011496	0.011530	0.011429	0.011362	0.011362
0.011429	0.011362	0.011463	0.011329	0.011463	0.011564	0.011866
0.011362	0.011765	0.011336	0.017823	0.011322	0.011549	0.011688
0.011648	0.011693	0.011374	0.011408	0.011829	0.011716	0.011425
0.011535	0.011610	0.011216	0.011353	0.011333	0.011406	0.011361
0.011529	0.011470	0.011468	0.011259	0.011496	0.011530	0.011534
0.011404	0.011497	0.011497	0.011463	0.011429	0.011430	0.011396
0.011429	0.011362	0.011396	0.011429	0.011396	0.011329	0.011362
0.011362	0.011429	0.011329	0.011430	0.011463	0.011497	0.011396
0.011429	0.011396	0.011732	0.011732	0.011463	0.011497	0.011530
0.011295	0.011295	0.011329	0.011430	0.011295	0.011530	0.011530
0.011564	0.011564	0.011496	0.011597	0.011765	0.011599	0.011429
0.011429	0.011497	0.011530	0.011362	0.011429	0.011396	0.011429
0.011497	0.011497	0.011530	0.011497	0.011397	0.011597	0.011564
0.011433	0.011329	0.011429	0.011530	0.011430	0.011564	0.011597
0.011765	0.011470	0.011631	0.011597	0.011530	0.011698	0.011329
0.011498	0.011396	0.011362	0.011362	0.011429	0.011329	0.011430
0.011463	0.011497	0.011396	0.011429	0.011396	0.011732	0.011732
0.011463	0.011497	0.011564	0.011496	0.012136	0.011524	0.011482
0.011517	0.011511	0.011622	0.011510	0.011620	0.011543	0.011508
0.012090	0.011406	0.011540	0.011441	0.011749	0.011604	0.011707
0.012114	0.011550	0.011669	0.011731	0.011597	0.011597	0.011635
0.011295	0.011463	0.011597	0.011563	0.011567	0.011564	0.011532
0.011362	0.011329	0.011362	0.011396	0.011295	0.011362	0.011262
0.011396	0.011429	0.011329	0.011295	0.011396	0.011329	0.011362
0.011362	0.011295	0.011362	0.011396	0.011295	0.011496	0.011530
0.011429	0.011496	0.011429	0.011429	0.011362	0.011765	0.011396
0.011443	0.011362	0.011598	0.011603	0.011463	0.011463	0.011463
0.011463	0.011396	0.011362	0.011396	0.011496	0.011496	0.011429
0.011429	0.011530	0.011731	0.011463	0.011664	0.011664	0.011530
0.011362	0.011429	0.011463	0.011866	0.011430	0.011463	0.011429
0.011463	0.011396	0.011396	0.011698	0.011631	0.011664	0.011396
0.011496	0.011564	0.011429	0.011496	0.011463	0.011329	0.011664
0.011429	0.011396	0.011396	0.011564	0.011396	0.011496	0.011732
0.011429	0.011807	0.011799	0.011548	0.011229	0.011502	0.011318
0.011335	0.011761	0.011306	0.011507	0.011304	0.011417	0.011432
0.011468	0.011258	0.011394	0.011258	0.011276	0.011395	0.011425
0.011437	0.011296	0.011448	0.011342	0.011431	0.011564	0.011631
0.011631	0.011497	0.011601	0.011603	0.011738	0.011732	0.011429
0.011664	0.011530	0.011396	0.011497	0.011295	0.011530	0.011329
0.011396	0.011396	0.011362	0.011329	0.011362	0.011329	0.011429
0.011409	0.011410	0.011403	0.011437	0.011573	0.011538	0.011538
0.011537	0.011464	0.011282	0.011276	0.011399	0.011568	0.011569
0.011363	0.011433	0.011666	0.011635	0.011565	0.011599	0.011530
0.011463	0.011530	0.011530	0.011429	0.012000	0.011597	0.011664
0.011698	0.011772	0.011401	0.011441	0.011362	0.011564	0.011530

0.011565	0.011597	0.011597	0.011597	0.011689	0.011732	0.011372
0.011263	0.011268	0.011366	0.011332	0.011436	0.011247	0.011604
0.011403	0.011586	0.011298	0.011397	0.011396	0.011471	0.011362
0.011329	0.011329	0.011396	0.011362	0.011564	0.011530	0.011463
0.011564	0.011631	0.011362	0.011362	0.011396	0.011396	0.011398
0.011466	0.011396	0.011396	0.011431	0.011564	0.011631	0.011631
0.011497	0.011601	0.011603	0.011738	0.011732	0.011429	0.011597
0.011530	0.011530	0.011365	0.011547	0.011477	0.011503	0.011340
0.011542	0.011653	0.011484	0.011434	0.011329	0.011530	0.011396
0.011497	0.011430	0.011329	0.011431	0.011530	0.011533	0.011430
0.011329	0.011263	0.011396	0.011329	0.011262	0.011366	0.011530
0.011429	0.011362	0.011396	0.011429	0.011396	0.011329	0.011530
0.011396	0.011429	0.011429	0.011362	0.011429	0.011664	0.011329
0.011362	0.011765	0.011362	0.011396	0.011362	0.011731	0.011274
0.011622	0.011230	0.011311	0.011463	0.011589	0.011527	0.011227
0.011270	0.011530	0.011631	0.011739	0.011599	0.011703	0.011867
0.011767	0.011804	0.011765	0.011983	0.011731	0.012606	0.011768
0.012507	0.012274	0.012408	0.012680	0.011731	0.011430	0.011732
0.012171	0.012072	0.011698	0.011597	0.011665	0.011665	0.011635
0.011530	0.011501	0.011597	0.011565	0.011934	0.012000	0.012002
0.011901	0.012002	0.012105	0.011838	0.012171	0.012178	0.012238
0.012239	0.012273	0.012186	0.012239	0.012240	0.011463	0.011766
0.011834	0.012337	0.011840	0.012068	0.012541	0.011603	0.011497
0.011463	0.011463	0.011934	0.011965	0.012080	0.011799	0.011465
S072	0.011698	0.011698	0.011967	0.011799	0.011799	0.011866
0.011705	0.011637	0.011866	0.011295	0.011362	0.011329	0.011295
0.011329	0.011497	0.011295	0.011329	0.011396	0.011362	0.011329
0.011295	0.011329	0.011429	0.011295	0.011329	0.011329	0.011671
0.011564	0.011329	0.011228	0.011463	0.011396	0.011362	0.011396
0.011362	0.011463	0.011362	0.011362	0.011396	0.011362	0.011530
0.011295	0.011362	0.011396	0.011329	0.011429	0.011463	0.011396
0.011362	0.011530	0.011430	0.011698	0.011362	0.011631	0.011362
0.011329	0.011429	0.011497	0.011631	0.011732	0.011429	0.011530
0.011396	0.011463	0.011295	0.011597	0.011833	0.011362	0.011429
0.011329	0.011429	0.000000	0.011463	0.011396	0.011396	0.011396
0.011295	0.011329	0.011295	0.011396	0.011362	0.011397	0.011463
0.011396	0.011362	0.011429	0.011329	0.011463	0.011295	0.011362
0.011530	0.011463	0.011698	0.011530	0.011362	0.011597	0.011362
0.011542	0.011770	0.011588	0.011295	0.011284	0.011391	0.011493
0.012121	0.011620	0.011307	0.011597	0.011597	0.011396	0.011497
0.011429	0.011396	0.011463	0.011362	0.011463	0.011396	0.011396
0.011362	0.011463	0.011664	0.011530	0.011429	0.011396	0.011329
0.011295	0.011295	0.011362	0.011329	0.011530	0.011497	0.011430
0.011530	0.011597	0.011329	0.011329	0.011362	0.011362	0.011364
0.011432	0.011362	0.011362	0.011204	0.011241	0.011201	0.011363
0.011365	0.011301	0.011319	0.011336	0.011264	0.011300	0.011330
0.011528	0.011336	0.011405	0.011296	0.011302	0.011430	0.011397
0.011464	0.011397	0.011396	0.011496	0.011429	0.011799	0.011598
0.011631	0.011497	0.011362	0.011463	0.011262	0.011497	0.011295
0.011362	0.011362	0.011329	0.011295	0.011329	0.011295	0.011396
0.011362	0.011463	0.011396	0.011295	0.011330	0.011497	0.011499
0.011497	0.011396	0.011295	0.011229	0.011362	0.011295	0.011228
0.011332	0.011497	0.011295	0.011497	0.011262	0.011362	0.011329
0.011430	0.011329	0.011362	0.011329	0.011362	0.011228	0.011362
0.011396	0.011329	0.011396	0.011329	0.011362	0.011396	0.011631
0.011732	0.011664	0.011732	0.011498	0.011732	0.011362	0.011329
0.011364	0.011667	0.011518	0.011266	0.011739	0.011231	0.011336
0.011739	0.011638	0.011530	0.011732	0.011436	0.011597	0.011564
0.011497	0.011664	0.011486	0.011349	0.011395	0.011396	0.011432
0.011564	0.011465	0.011601	0.011669	0.011597	0.011665	0.011772

0.011500	0.011671	0.011602	0.011534	0.011800	0.011262	0.011465
0.011429	0.011362	0.011262	0.011262	0.011329	0.011329	0.011396
0.011329	0.011396	0.011295	0.011396	0.011329	0.011329	0.011396
0.011329	0.011765	0.011664	0.011698	0.011228	0.011329	0.011362
0.011362	0.011329	0.011396	0.011429	0.011295	0.011295	0.011295
0.011832	0.011463	0.011463	0.011262	0.011362	0.011530	0.011295
0.011295	0.011362	0.011329	0.011497	0.011295	0.011429	0.011564
0.011497	0.011329	0.011329	0.011262	0.011262	0.011429	0.011362
0.011262	0.011362	0.011228	0.011262	0.011396	0.011530	0.011295
0.011262	0.011396	0.011329	0.011228	0.011564	0.011228	0.011396
0.011471	0.011295	0.011597	0.011430	0.011463	0.011329	0.011429
0.011295	0.011631	0.011228	0.011497	0.011295	0.011295	0.011228
0.011463	0.011665	0.011295	0.011429	0.011262	0.011664	0.011295
0.011295	0.011295	0.011329	0.011429	0.011329	0.011429	0.011497
0.011497	0.011463	0.011362	0.011463	0.011429	0.011295	0.011262
0.011295	0.011295	0.011295	0.011295	0.011262	0.011429	0.011295
0.011329	0.011329	0.011463	0.011497	0.011396	0.011329	0.011329
0.011329	0.011329	0.011429	0.011295	0.011429	0.011530	0.011832
0.011329	0.011732	0.011336	0.017861	0.011322	0.011549	0.011688
0.011648	0.011693	0.011427	0.011408	0.011885	0.011716	0.011486
0.011582	0.011732	0.011255	0.011353	0.011333	0.011455	0.011361
0.011576	0.011507	0.011505	0.011325	0.011463	0.011497	0.011500
0.011369	0.011463	0.011463	0.011430	0.011396	0.011396	0.011363
0.011396	0.011329	0.011362	0.011396	0.011362	0.011295	0.011329
0.011329	0.011396	0.011295	0.011396	0.011430	0.011463	0.011362
0.011396	0.011362	0.011698	0.011698	0.011430	0.011396	0.011497
0.011262	0.011262	0.011295	0.011396	0.011262	0.011497	0.011497
0.011530	0.011530	0.011463	0.011564	0.011732	0.011565	0.011396
0.011396	0.011463	0.011497	0.011329	0.011396	0.011362	0.011396
0.011463	0.011463	0.011497	0.011463	0.011363	0.011564	0.011530
0.011365	0.011295	0.011396	0.011497	0.011396	0.011530	0.011564
0.011732	0.011436	0.011597	0.011564	0.011497	0.011664	0.011295
0.011464	0.011363	0.011329	0.011329	0.011396	0.011295	0.011396
0.011430	0.011463	0.011362	0.011396	0.011362	0.011698	0.011698
0.011430	0.011396	0.011530	0.011463	0.012103	0.011525	0.011412
0.011517	0.011476	0.011587	0.011476	0.011585	0.011508	0.011474
0.012056	0.011372	0.011505	0.011441	0.011714	0.011570	0.011673
0.012080	0.011512	0.011635	0.011698	0.011564	0.011564	0.011601
0.011262	0.011429	0.011564	0.011530	0.011533	0.011530	0.011498
0.011362	0.011295	0.011329	0.011362	0.011262	0.011329	0.011228
0.011362	0.011396	0.011295	0.011262	0.011362	0.011295	0.011329
0.011329	0.011262	0.011329	0.011362	0.011262	0.011463	0.011497
0.011396	0.011463	0.011396	0.011396	0.011329	0.011732	0.011362
0.011373	0.011329	0.011564	0.011569	0.011430	0.011429	0.011429
0.011429	0.011362	0.011329	0.011362	0.011463	0.011463	0.011396
0.011396	0.011396	0.011698	0.011429	0.011631	0.011631	0.011496
0.011329	0.011396	0.011429	0.011833	0.011396	0.011430	0.011396
0.011429	0.011362	0.011362	0.011665	0.011597	0.011631	0.011362
0.011463	0.011530	0.011396	0.011463	0.011429	0.011295	0.011631
0.011396	0.011362	0.011362	0.011530	0.011362	0.011463	0.011698
0.011396	0.011773	0.011765	0.011589	0.011286	0.011468	0.011370
0.011335	0.011800	0.011306	0.011552	0.011402	0.011518	0.011485
0.011570	0.011389	0.011394	0.011258	0.011330	0.011328	0.011466
0.011475	0.011375	0.011448	0.011377	0.011397	0.011530	0.011598
0.011597	0.011464	0.011567	0.011569	0.011704	0.011732	0.011396
0.011631	0.011497	0.011362	0.011463	0.011262	0.011497	0.011295
0.011362	0.011362	0.011329	0.011295	0.011329	0.011295	0.011396
0.011375	0.011305	0.011368	0.011403	0.011539	0.011504	0.011504
0.011503	0.011430	0.011171	0.011240	0.011365	0.011535	0.011535
0.011329	0.011399	0.011633	0.011601	0.011531	0.011565	0.011496

0.011429	0.011497	0.011496	0.011396	0.011967	0.011564	0.011631
0.011665	0.011737	0.011367	0.011441	0.011329	0.011530	0.011497
0.011531	0.011564	0.011564	0.011564	0.011729	0.011698	0.011303
0.011229	0.011199	0.011333	0.011298	0.011402	0.011209	0.011570
0.011369	0.011499	0.011203	0.011360	0.011363	0.011437	0.011329
0.011295	0.011295	0.011362	0.011329	0.011530	0.011497	0.011430
0.011530	0.011597	0.011329	0.011329	0.011362	0.011362	0.011364
0.011432	0.011362	0.011362	0.011397	0.011530	0.011598	0.011597
0.011464	0.011567	0.011569	0.011704	0.011732	0.011396	0.011564
0.011497	0.011497	0.011331	0.011513	0.011442	0.011469	0.011341
0.011508	0.011744	0.011526	0.011400	0.011295	0.011497	0.011362
0.011463	0.011396	0.011295	0.011330	0.011497	0.011499	0.011396
0.011295	0.011229	0.011362	0.011295	0.011228	0.011332	0.011497
0.011396	0.011329	0.011362	0.011396	0.011362	0.011295	0.011497
0.011362	0.011396	0.011396	0.011329	0.011396	0.011631	0.011295
0.011329	0.011732	0.011329	0.011362	0.011329	0.011698	0.011274
0.011735	0.011273	0.011361	0.011543	0.011630	0.011527	0.011269
0.011323	0.011496	0.011597	0.011705	0.011565	0.011703	0.011834
0.011733	0.011770	0.011732	0.011983	0.011698	0.012573	0.011734
0.012473	0.012241	0.012375	0.012646	0.011698	0.011396	0.011699
0.012137	0.012072	0.011664	0.011564	0.011632	0.011632	0.011601
0.011496	0.011501	0.011564	0.011531	0.011901	0.011967	0.011968
0.011868	0.011968	0.012072	0.011838	0.012138	0.012144	0.012205
0.012205	0.012239	0.012186	0.012206	0.012207	0.011429	0.011732
0.011800	0.012303	0.011806	0.012034	0.012508	0.011570	0.011464
0.011429	0.011429	0.011900	0.011883	0.012045	0.011765	0.011431
S073	0.011765	0.011765	0.012101	0.011866	0.011866	0.011933
0.011773	0.011705	0.011933	0.011362	0.011429	0.011397	0.011362
0.011396	0.011564	0.011362	0.011396	0.011463	0.011429	0.011396
0.011362	0.011396	0.011496	0.011363	0.011396	0.011396	0.011739
0.011631	0.011396	0.011295	0.011530	0.011463	0.011429	0.011463
0.011429	0.011530	0.011429	0.011429	0.011463	0.011429	0.011597
0.011362	0.011429	0.011463	0.011396	0.011496	0.011530	0.011463
0.011429	0.011597	0.011497	0.011765	0.011429	0.011698	0.011429
0.011396	0.011497	0.011564	0.011698	0.011799	0.011497	0.011597
0.011463	0.011530	0.011429	0.011664	0.011900	0.011429	0.011497
0.011396	0.011496	0.011463	0.000000	0.011463	0.011463	0.011463
0.011362	0.011396	0.011362	0.011463	0.011429	0.011464	0.011530
0.011463	0.011429	0.011496	0.011396	0.011530	0.011362	0.011429
0.011597	0.011530	0.011765	0.011597	0.011429	0.011664	0.011429
0.011579	0.011807	0.011626	0.011334	0.011321	0.011428	0.011533
0.012158	0.011670	0.011349	0.011664	0.011664	0.011463	0.011564
0.011497	0.011463	0.011530	0.011429	0.011530	0.011463	0.011463
0.011429	0.011530	0.011664	0.011597	0.011497	0.011463	0.011396
0.011362	0.011362	0.011430	0.011396	0.011597	0.011564	0.011497
0.011597	0.011665	0.011396	0.011396	0.011430	0.011429	0.011432
0.011500	0.011430	0.011430	0.011312	0.011386	0.011341	0.011430
0.011433	0.011370	0.011392	0.011405	0.011332	0.011368	0.011398
0.011672	0.011439	0.011473	0.011363	0.011371	0.011498	0.011464
0.011531	0.011465	0.011464	0.011564	0.011496	0.011866	0.011665
0.011698	0.011564	0.011429	0.011530	0.011329	0.011564	0.011362
0.011429	0.011429	0.011396	0.011362	0.011396	0.011362	0.011463
0.011430	0.011530	0.011463	0.011362	0.011465	0.011564	0.011567
0.011564	0.011463	0.011362	0.011296	0.011429	0.011362	0.011295
0.011400	0.011564	0.011362	0.011564	0.011329	0.011430	0.011396
0.011497	0.011396	0.011430	0.011396	0.011430	0.011295	0.011430
0.011463	0.011396	0.011463	0.011396	0.011429	0.011463	0.011698
0.011799	0.011732	0.011799	0.011532	0.011799	0.011429	0.011396
0.011432	0.011626	0.011609	0.011300	0.011807	0.011299	0.011414
0.011807	0.011706	0.011597	0.011732	0.011504	0.011664	0.011631

0.011564	0.011664	0.011593	0.011431	0.011479	0.011463	0.011534
0.011631	0.011532	0.011669	0.011737	0.011664	0.011732	0.011839
0.011568	0.011739	0.011670	0.011601	0.011868	0.011329	0.011532
0.011496	0.011429	0.011329	0.011329	0.011396	0.011396	0.011463
0.011396	0.011463	0.011362	0.011463	0.011396	0.011396	0.011463
0.011396	0.011832	0.011732	0.011765	0.011295	0.011396	0.011429
0.011429	0.011396	0.011463	0.011497	0.011362	0.011362	0.011362
0.011899	0.011530	0.011530	0.011329	0.011429	0.011597	0.011362
0.011362	0.011429	0.011396	0.011564	0.011362	0.011496	0.011631
0.011564	0.011396	0.011396	0.011329	0.011329	0.011497	0.011429
0.011329	0.011429	0.011295	0.011329	0.011463	0.011598	0.011362
0.011329	0.011463	0.011396	0.011295	0.011631	0.011295	0.011463
0.011539	0.011362	0.011664	0.011497	0.011530	0.011396	0.011496
0.011362	0.011698	0.011295	0.011564	0.011362	0.011362	0.011295
0.011530	0.011698	0.011429	0.011497	0.011329	0.011732	0.011362
0.011362	0.011362	0.011396	0.011497	0.011396	0.011496	0.011564
0.011564	0.011530	0.011429	0.011530	0.011496	0.011362	0.011329
0.011362	0.011362	0.011429	0.011362	0.011329	0.011497	0.011362
0.011396	0.011396	0.011530	0.011564	0.011463	0.011396	0.011396
0.011463	0.011396	0.011496	0.011362	0.011496	0.011597	0.011900
0.011396	0.011799	0.011371	0.017899	0.011519	0.011549	0.011723
0.011683	0.011729	0.011479	0.011466	0.011939	0.011751	0.011607
0.011677	0.011732	0.011294	0.011395	0.011372	0.011455	0.011361
0.011670	0.011544	0.011542	0.011325	0.011530	0.011564	0.011567
0.011438	0.011530	0.011530	0.011497	0.011463	0.011463	0.011430
0.011463	0.011396	0.011429	0.011463	0.011430	0.011362	0.011396
0.011396	0.011127	0.011362	0.011463	0.011497	0.011530	0.011429
0.011463	0.011430	0.011765	0.011765	0.011497	0.011531	0.011564
0.011329	0.011329	0.011362	0.011463	0.011329	0.011564	0.011564
0.011597	0.011597	0.011530	0.011631	0.011799	0.011633	0.011463
0.011463	0.011530	0.011564	0.011396	0.011463	0.011430	0.011463
0.011531	0.011530	0.011564	0.011531	0.011431	0.011631	0.011597
0.011433	0.011362	0.011463	0.011564	0.011463	0.011598	0.011631
0.011732	0.011504	0.011664	0.011631	0.011564	0.011664	0.011363
0.011532	0.011430	0.011396	0.011396	0.011127	0.011362	0.011463
0.011497	0.011530	0.011429	0.011463	0.011430	0.011765	0.011765
0.011497	0.011531	0.011597	0.011530	0.012170	0.011596	0.011517
0.011587	0.011546	0.011657	0.011545	0.011655	0.011577	0.011543
0.012125	0.011441	0.011574	0.011510	0.011783	0.011638	0.011741
0.012148	0.011626	0.011703	0.011765	0.011631	0.011631	0.011668
0.011329	0.011497	0.011631	0.011597	0.011601	0.011597	0.011565
0.011429	0.011362	0.011396	0.011429	0.011329	0.011396	0.011295
0.011429	0.011463	0.011362	0.011329	0.011429	0.011362	0.011396
0.011396	0.011329	0.011396	0.011429	0.011329	0.011530	0.011564
0.011463	0.011530	0.011463	0.011463	0.011396	0.011799	0.011429
0.011478	0.011396	0.011631	0.011637	0.011497	0.011496	0.011496
0.011497	0.011429	0.011396	0.011429	0.011530	0.011530	0.011463
0.011463	0.011597	0.011765	0.011496	0.011698	0.011698	0.011564
0.011396	0.011463	0.011497	0.011900	0.011463	0.011497	0.011463
0.011496	0.011429	0.011429	0.011732	0.011665	0.011698	0.011429
0.011530	0.011597	0.011463	0.011530	0.011496	0.011362	0.011698
0.011463	0.011429	0.011429	0.011597	0.011429	0.011530	0.011765
0.011463	0.011841	0.011832	0.011629	0.011455	0.011536	0.011473
0.011390	0.011839	0.011341	0.011596	0.011451	0.011569	0.011591
0.011672	0.011389	0.011561	0.011390	0.011438	0.011663	0.011506
0.011512	0.011414	0.011483	0.011412	0.011465	0.011597	0.011665
0.011665	0.011531	0.011635	0.011636	0.011738	0.011767	0.011463
0.011698	0.011564	0.011429	0.011530	0.011329	0.011564	0.011362
0.011429	0.011429	0.011396	0.011362	0.011396	0.011362	0.011463
0.011444	0.011445	0.011437	0.011471	0.011608	0.011573	0.011572

0.011571	0.011497	0.011319	0.011312	0.011433	0.011602	0.011603
0.011396	0.011467	0.011700	0.011669	0.011598	0.011632	0.011564
0.011496	0.011564	0.011564	0.011463	0.012034	0.011631	0.011698
0.011732	0.011808	0.011435	0.011511	0.011396	0.011597	0.011564
0.011598	0.011631	0.011631	0.011631	0.011768	0.011765	0.011407
0.011297	0.011303	0.011401	0.011366	0.011470	0.011284	0.011638
0.011437	0.011586	0.011346	0.011472	0.011430	0.011505	0.011396
0.011362	0.011362	0.011430	0.011396	0.011597	0.011564	0.011497
0.011597	0.011665	0.011396	0.011396	0.011430	0.011429	0.011432
0.011500	0.011430	0.011430	0.011465	0.011597	0.011665	0.011665
0.011531	0.011635	0.011636	0.011738	0.011767	0.011463	0.011631
0.011564	0.011564	0.011398	0.011582	0.011512	0.011537	0.011376
0.011577	0.011744	0.011568	0.011468	0.011362	0.011564	0.011430
0.011530	0.011463	0.011362	0.011465	0.011564	0.011567	0.011463
0.011362	0.011296	0.011429	0.011362	0.011295	0.011400	0.011564
0.011463	0.011396	0.011429	0.011463	0.011430	0.011362	0.011564
0.011429	0.011463	0.011463	0.011396	0.011463	0.011698	0.011362
0.011396	0.011799	0.011396	0.011429	0.011396	0.011765	0.011360
0.011847	0.011358	0.011461	0.011584	0.011671	0.011563	0.011311
0.011428	0.011564	0.011664	0.011773	0.011632	0.011737	0.011901
0.011800	0.011838	0.011799	0.012017	0.011765	0.012640	0.011801
0.012540	0.012308	0.012442	0.012714	0.011765	0.011463	0.011766
0.012205	0.012106	0.011732	0.011631	0.011699	0.011699	0.011669
0.011564	0.011535	0.011631	0.011598	0.011968	0.012034	0.012036
0.011935	0.012035	0.012139	0.011872	0.012205	0.012212	0.012272
0.012273	0.012307	0.012221	0.012273	0.012274	0.011496	0.011800
0.011868	0.012371	0.011874	0.012102	0.012575	0.011638	0.011531
0.011496	0.011496	0.011968	0.012048	0.012116	0.011832	0.011498
S074	0.011698	0.011698	0.012034	0.011799	0.011799	0.011866
0.011637	0.011569	0.011866	0.011295	0.011362	0.011329	0.011295
0.011329	0.011496	0.011295	0.011329	0.011396	0.011362	0.011329
0.011295	0.011329	0.011429	0.011295	0.011329	0.011329	0.011603
0.011564	0.011329	0.011228	0.011463	0.011396	0.011362	0.011396
0.011362	0.011463	0.011362	0.011362	0.011396	0.011362	0.011530
0.011295	0.011362	0.011396	0.011329	0.011429	0.011463	0.011396
0.011362	0.011530	0.011430	0.011698	0.011362	0.011631	0.011362
0.011329	0.011429	0.011496	0.011631	0.011731	0.011429	0.011530
0.011396	0.011463	0.011362	0.011597	0.011832	0.011362	0.011429
0.011329	0.011429	0.011396	0.011463	0.000000	0.011396	0.011396
0.011295	0.011329	0.011295	0.011396	0.011362	0.011397	0.011463
0.011396	0.011362	0.011429	0.011194	0.011463	0.011295	0.011362
0.011530	0.011463	0.011698	0.011530	0.011362	0.011597	0.011362
0.011468	0.011695	0.011513	0.011216	0.011210	0.011355	0.011415
0.012085	0.011467	0.011225	0.011597	0.011597	0.011396	0.011496
0.011429	0.011396	0.011463	0.011362	0.011463	0.011396	0.011396
0.011362	0.011262	0.011664	0.011530	0.011429	0.011396	0.011329
0.011295	0.011295	0.011362	0.011329	0.011530	0.011497	0.011429
0.011530	0.011597	0.011329	0.011329	0.011362	0.011362	0.011364
0.011432	0.011362	0.011362	0.011204	0.011313	0.011271	0.011363
0.011365	0.011301	0.011318	0.011336	0.011264	0.011300	0.011330
0.011600	0.011302	0.011405	0.011296	0.011302	0.011430	0.011396
0.011464	0.011397	0.011396	0.011496	0.011429	0.011799	0.011597
0.011631	0.011497	0.011362	0.011463	0.011262	0.011496	0.011295
0.011362	0.011362	0.011329	0.011295	0.011329	0.011295	0.011396
0.011362	0.011463	0.011396	0.011295	0.011397	0.011497	0.011466
0.011497	0.011396	0.011295	0.011195	0.011362	0.011295	0.011228
0.011298	0.011497	0.011295	0.011497	0.011262	0.011362	0.011329
0.011430	0.011329	0.011362	0.011329	0.011362	0.011228	0.011362
0.011396	0.011329	0.011396	0.011329	0.011362	0.011396	0.011631
0.011732	0.011664	0.011732	0.011465	0.011732	0.011362	0.011329

0.011364	0.011586	0.011426	0.011232	0.011739	0.011231	0.011296
0.011671	0.011638	0.011530	0.011732	0.011436	0.011597	0.011564
0.011496	0.011664	0.011486	0.011349	0.011395	0.011396	0.011466
0.011564	0.011465	0.011601	0.011669	0.011597	0.011665	0.011738
0.011500	0.011637	0.011602	0.011533	0.011800	0.011262	0.011464
0.011429	0.011362	0.011262	0.011262	0.011329	0.011329	0.011396
0.011329	0.011396	0.011295	0.011396	0.011329	0.011329	0.011396
0.011329	0.011765	0.011664	0.011698	0.011228	0.011329	0.011362
0.011362	0.011329	0.011396	0.011429	0.011295	0.011295	0.011295
0.011832	0.011463	0.011463	0.011262	0.011362	0.011530	0.011295
0.011295	0.011362	0.011329	0.011497	0.011295	0.011429	0.011564
0.011496	0.011329	0.011329	0.011262	0.011262	0.011429	0.011362
0.011262	0.011362	0.011228	0.011262	0.011396	0.011530	0.011295
0.011262	0.011396	0.011329	0.011228	0.011564	0.011228	0.011396
0.011437	0.011295	0.011597	0.011429	0.011463	0.011329	0.011429
0.011295	0.011631	0.011228	0.011496	0.011295	0.011295	0.011228
0.011463	0.011665	0.011362	0.011429	0.011262	0.011664	0.011295
0.011295	0.011295	0.011329	0.011429	0.011329	0.011429	0.011496
0.011496	0.011463	0.011362	0.011463	0.011429	0.011295	0.011262
0.011295	0.011295	0.011362	0.011295	0.011262	0.011429	0.011295
0.011329	0.011329	0.011463	0.011496	0.011396	0.011329	0.011329
0.011262	0.011329	0.011429	0.011295	0.011429	0.011530	0.011832
0.011329	0.011732	0.011302	0.017823	0.011257	0.011427	0.011653
0.011614	0.011658	0.011322	0.011292	0.011719	0.011682	0.011364
0.011487	0.011549	0.011177	0.011270	0.011294	0.011307	0.011232
0.011482	0.011470	0.011468	0.011325	0.011463	0.011497	0.011500
0.011335	0.011463	0.011463	0.011429	0.011396	0.011396	0.011329
0.011396	0.011329	0.011362	0.011396	0.011362	0.011295	0.011329
0.011329	0.011396	0.011295	0.011396	0.011429	0.011463	0.011362
0.011396	0.011362	0.011698	0.011698	0.011430	0.011464	0.011497
0.011262	0.011262	0.011295	0.011396	0.011262	0.011497	0.011497
0.011530	0.011530	0.011463	0.011564	0.011732	0.011565	0.011396
0.011396	0.011463	0.011497	0.011329	0.011396	0.011362	0.011396
0.011463	0.011463	0.011497	0.011463	0.011363	0.011564	0.011530
0.011399	0.011295	0.011396	0.011497	0.011396	0.011530	0.011564
0.011732	0.011436	0.011597	0.011564	0.011496	0.011664	0.011295
0.011464	0.011363	0.011329	0.011329	0.011396	0.011295	0.011396
0.011429	0.011463	0.011362	0.011396	0.011362	0.011698	0.011698
0.011430	0.011464	0.011530	0.011463	0.012103	0.011489	0.011412
0.011482	0.011441	0.011552	0.011441	0.011550	0.011474	0.011474
0.012021	0.011337	0.011471	0.011406	0.011680	0.011570	0.011638
0.012046	0.011512	0.011635	0.011698	0.011564	0.011564	0.011601
0.011262	0.011429	0.011564	0.011530	0.011533	0.011530	0.011464
0.011362	0.011295	0.011329	0.011362	0.011262	0.011329	0.011228
0.011362	0.011396	0.011295	0.011262	0.011362	0.011295	0.011329
0.011329	0.011262	0.011329	0.011362	0.011262	0.011463	0.011496
0.011396	0.011463	0.011396	0.011396	0.011329	0.011731	0.011362
0.011373	0.011329	0.011564	0.011535	0.011429	0.011429	0.011429
0.011429	0.011362	0.011329	0.011362	0.011463	0.011463	0.011396
0.011396	0.011530	0.011698	0.011429	0.011631	0.011631	0.011496
0.011329	0.011396	0.011429	0.011832	0.011396	0.011429	0.011396
0.011429	0.011362	0.011362	0.011665	0.011597	0.011631	0.011362
0.011463	0.011530	0.011396	0.011463	0.011429	0.011295	0.011631
0.011396	0.011362	0.011362	0.011530	0.011362	0.011463	0.011698
0.011396	0.011705	0.011765	0.011467	0.011229	0.011468	0.011215
0.011225	0.011722	0.011271	0.011462	0.011255	0.011366	0.011379
0.011468	0.011192	0.011339	0.011192	0.011222	0.011395	0.011385
0.011399	0.011296	0.011412	0.011342	0.011397	0.011530	0.011597
0.011597	0.011464	0.011567	0.011569	0.011704	0.011732	0.011396
0.011631	0.011497	0.011362	0.011463	0.011262	0.011496	0.011295

0.011362	0.011362	0.011329	0.011295	0.011329	0.011295	0.011396
0.011375	0.011375	0.011368	0.011197	0.011539	0.011504	0.011504
0.011503	0.011228	0.011171	0.011204	0.011365	0.011535	0.011535
0.011329	0.011399	0.011632	0.011601	0.011531	0.011565	0.011496
0.011429	0.011496	0.011496	0.011396	0.011967	0.011564	0.011631
0.011665	0.011737	0.011367	0.011441	0.011329	0.011530	0.011496
0.011329	0.011564	0.011564	0.011564	0.011728	0.011698	0.011303
0.011195	0.011199	0.011298	0.011264	0.011368	0.011209	0.011536
0.011335	0.011499	0.011251	0.011285	0.011363	0.011437	0.011329
0.011295	0.011295	0.011362	0.011329	0.011530	0.011497	0.011429
0.011530	0.011597	0.011329	0.011329	0.011362	0.011362	0.011364
0.011432	0.011362	0.011362	0.011397	0.011530	0.011597	0.011597
0.011464	0.011567	0.011569	0.011704	0.011732	0.011396	0.011564
0.011496	0.011496	0.011331	0.011513	0.011442	0.011265	0.011305
0.011473	0.011653	0.011483	0.011400	0.011295	0.011497	0.011362
0.011463	0.011396	0.011295	0.011397	0.011497	0.011466	0.011396
0.011295	0.011195	0.011362	0.011295	0.011228	0.011298	0.011497
0.011396	0.011329	0.011362	0.011396	0.011362	0.011295	0.011497
0.011362	0.011396	0.011396	0.011329	0.011396	0.011631	0.011295
0.011329	0.011731	0.011329	0.011362	0.011329	0.011698	0.011189
0.011679	0.011188	0.011261	0.011503	0.011508	0.011491	0.011144
0.011218	0.011496	0.011597	0.011705	0.011565	0.011669	0.011834
0.011733	0.011770	0.011731	0.011949	0.011698	0.012573	0.011734
0.012473	0.012207	0.012375	0.012646	0.011698	0.011396	0.011698
0.012137	0.012038	0.011664	0.011564	0.011632	0.011632	0.011601
0.011496	0.011467	0.011564	0.011531	0.011901	0.011967	0.011968
0.011867	0.011968	0.012038	0.011804	0.012138	0.012144	0.012205
0.012205	0.012239	0.012152	0.012205	0.012207	0.011429	0.011732
0.011800	0.012303	0.011806	0.012034	0.012508	0.011570	0.011464
0.011429	0.011429	0.011900	0.011924	0.012045	0.011765	0.011431
S075 0.011698	0.011698	0.012034	0.011799	0.011799	0.011866	
0.011705	0.011637	0.011866	0.011295	0.011362	0.011329	0.011295
0.011329	0.011496	0.011295	0.011329	0.011396	0.011362	0.011329
0.011295	0.011329	0.011429	0.011295	0.011329	0.011329	0.011671
0.011564	0.011329	0.011228	0.011463	0.011396	0.011362	0.011396
0.011362	0.011463	0.011295	0.011362	0.011329	0.011362	0.011530
0.011295	0.011362	0.011329	0.011127	0.011429	0.011463	0.011396
0.011362	0.011463	0.011430	0.011698	0.011295	0.011631	0.011295
0.011329	0.011429	0.011429	0.011631	0.011732	0.011429	0.011530
0.011396	0.011463	0.011362	0.011597	0.011832	0.011362	0.011429
0.011329	0.011429	0.011396	0.011463	0.011396	0.000000	0.000000
0.011295	0.011329	0.011295	0.011396	0.011362	0.011397	0.011463
0.011396	0.011362	0.011429	0.011329	0.011463	0.011295	0.011362
0.011530	0.011463	0.011698	0.011530	0.011362	0.011597	0.011295
0.011505	0.011733	0.011551	0.011256	0.011284	0.011355	0.011454
0.012121	0.011467	0.011266	0.011597	0.011597	0.011396	0.011497
0.011429	0.011396	0.011463	0.011362	0.011463	0.011396	0.011396
0.011362	0.011463	0.011664	0.011530	0.011429	0.011396	0.011329
0.011295	0.011295	0.011362	0.011329	0.011530	0.011497	0.011429
0.011530	0.011597	0.011329	0.011329	0.011362	0.011362	0.011364
0.011432	0.011362	0.011362	0.011240	0.011313	0.011271	0.011363
0.011365	0.011301	0.011355	0.011336	0.011264	0.011300	0.011330
0.011600	0.011370	0.011405	0.011296	0.011302	0.011430	0.011396
0.011464	0.011397	0.011396	0.011496	0.011429	0.011799	0.011598
0.011631	0.011497	0.011362	0.011463	0.011262	0.011497	0.011295
0.011362	0.011362	0.011329	0.011295	0.011329	0.011295	0.011396
0.011362	0.011463	0.011396	0.011295	0.011397	0.011497	0.011499
0.011497	0.011396	0.011295	0.011229	0.011362	0.011295	0.011228
0.011332	0.011497	0.011295	0.011497	0.011262	0.011362	0.011329
0.011430	0.011329	0.011362	0.011329	0.011362	0.011228	0.011362

0.011396	0.011329	0.011396	0.011329	0.011362	0.011396	0.011631
0.011732	0.011664	0.011732	0.011431	0.011732	0.011362	0.011329
0.011330	0.011667	0.011426	0.011232	0.011739	0.011231	0.011375
0.011739	0.011638	0.011530	0.011732	0.011436	0.011597	0.011564
0.011496	0.011664	0.011522	0.011390	0.011437	0.011396	0.011466
0.011564	0.011465	0.011601	0.011669	0.011597	0.011665	0.011772
0.011500	0.011671	0.011602	0.011534	0.011800	0.011262	0.011465
0.011429	0.011362	0.011262	0.011262	0.011329	0.011329	0.011262
0.011329	0.011396	0.011295	0.011396	0.011329	0.011329	0.011396
0.011329	0.011765	0.011664	0.011698	0.011228	0.011329	0.011362
0.011362	0.011329	0.011396	0.011429	0.011295	0.011295	0.011295
0.011832	0.011463	0.011463	0.011262	0.011362	0.011530	0.011295
0.011295	0.011362	0.011329	0.011497	0.011295	0.011429	0.011564
0.011496	0.011329	0.011329	0.011262	0.011262	0.011429	0.011362
0.011262	0.011362	0.011228	0.011262	0.011396	0.011530	0.011295
0.011262	0.011396	0.011329	0.011228	0.011564	0.011228	0.011396
0.011471	0.011295	0.011597	0.011430	0.011463	0.011329	0.011362
0.011295	0.011631	0.011228	0.011497	0.011295	0.011295	0.011228
0.011463	0.011665	0.011362	0.011429	0.011262	0.011664	0.011295
0.011295	0.011295	0.011329	0.011429	0.011329	0.011429	0.011496
0.011497	0.011463	0.011161	0.011463	0.011429	0.011228	0.011262
0.011295	0.011228	0.011362	0.011295	0.011262	0.011429	0.011295
0.011329	0.011329	0.011463	0.011496	0.011396	0.011329	0.011329
0.011396	0.011329	0.011429	0.011295	0.011429	0.011530	0.011832
0.011329	0.011732	0.011336	0.017823	0.011257	0.011488	0.011653
0.011614	0.011658	0.011427	0.011292	0.011829	0.011682	0.011364
0.011534	0.011610	0.011216	0.011311	0.011294	0.011406	0.011318
0.011482	0.011507	0.011505	0.011391	0.011463	0.011497	0.011500
0.011369	0.011463	0.011463	0.011429	0.011396	0.011396	0.011363
0.011396	0.011329	0.011362	0.011396	0.011362	0.011295	0.011329
0.011329	0.011396	0.011295	0.011396	0.011430	0.011463	0.011362
0.011396	0.011362	0.011698	0.011698	0.011430	0.011464	0.011497
0.011262	0.011262	0.011295	0.011396	0.011262	0.011497	0.011497
0.011530	0.011530	0.011463	0.011564	0.011732	0.011565	0.011396
0.011396	0.011463	0.011497	0.011329	0.011396	0.011362	0.011396
0.011463	0.011463	0.011497	0.011463	0.011363	0.011564	0.011530
0.011399	0.011295	0.011396	0.011430	0.011396	0.011530	0.011564
0.011732	0.011436	0.011597	0.011564	0.011496	0.011664	0.011295
0.011464	0.011363	0.011329	0.011329	0.011396	0.011295	0.011396
0.011430	0.011463	0.011362	0.011396	0.011362	0.011698	0.011698
0.011430	0.011464	0.011530	0.011463	0.012103	0.011524	0.011447
0.011517	0.011476	0.011587	0.011476	0.011585	0.011508	0.011474
0.012056	0.011372	0.011505	0.011441	0.011714	0.011570	0.011673
0.012080	0.011550	0.011635	0.011698	0.011564	0.011564	0.011601
0.011262	0.011429	0.011564	0.011530	0.011533	0.011530	0.011498
0.011362	0.011295	0.011329	0.011362	0.011262	0.011329	0.011228
0.011362	0.011396	0.011295	0.011262	0.011362	0.011295	0.011329
0.011329	0.011262	0.011329	0.011362	0.011262	0.011463	0.011496
0.011396	0.011463	0.011396	0.011396	0.011329	0.011732	0.011362
0.011408	0.011329	0.011564	0.011569	0.011429	0.011362	0.011429
0.011429	0.011362	0.011329	0.011362	0.011463	0.011463	0.011396
0.011396	0.011530	0.011698	0.011429	0.011631	0.011631	0.011496
0.011329	0.011396	0.011429	0.011832	0.011396	0.011429	0.011396
0.011429	0.011362	0.011362	0.011665	0.011597	0.011631	0.011362
0.011463	0.011530	0.011396	0.011463	0.011429	0.011295	0.011631
0.011396	0.011362	0.011362	0.011530	0.011362	0.011463	0.011698
0.011396	0.011773	0.011765	0.011548	0.011342	0.011468	0.011267
0.011280	0.011800	0.011306	0.011462	0.011353	0.011366	0.011432
0.011468	0.011192	0.011394	0.011192	0.011276	0.011395	0.011425
0.011437	0.011336	0.011412	0.011377	0.011397	0.011530	0.011597

0.011597	0.011464	0.011567	0.011569	0.011704	0.011732	0.011396
0.011631	0.011497	0.011362	0.011463	0.011262	0.011497	0.011295
0.011362	0.011362	0.011329	0.011295	0.011329	0.011295	0.011396
0.011340	0.011340	0.011368	0.011403	0.011539	0.011504	0.011504
0.011503	0.011430	0.011208	0.011204	0.011365	0.011535	0.011535
0.011329	0.011399	0.011632	0.011601	0.011531	0.011565	0.011496
0.011429	0.011496	0.011496	0.011396	0.011967	0.011564	0.011631
0.011665	0.011701	0.011367	0.011441	0.011329	0.011530	0.011497
0.011531	0.011564	0.011564	0.011564	0.011650	0.011698	0.011338
0.011229	0.011233	0.011333	0.011298	0.011402	0.011247	0.011570
0.011369	0.011543	0.011298	0.011397	0.011363	0.011437	0.011329
0.011295	0.011295	0.011362	0.011329	0.011530	0.011497	0.011429
0.011530	0.011597	0.011329	0.011329	0.011362	0.011362	0.011364
0.011432	0.011362	0.011362	0.011397	0.011530	0.011597	0.011597
0.011464	0.011567	0.011569	0.011704	0.011732	0.011396	0.011564
0.011497	0.011497	0.011263	0.011513	0.011442	0.011469	0.011340
0.011508	0.011698	0.011441	0.011400	0.011295	0.011497	0.011362
0.011463	0.011396	0.011295	0.011397	0.011497	0.011499	0.011396
0.011295	0.011229	0.011362	0.011295	0.011228	0.011332	0.011497
0.011396	0.011329	0.011362	0.011396	0.011362	0.011295	0.011497
0.011362	0.011396	0.011396	0.011329	0.011396	0.011631	0.011295
0.011329	0.011732	0.011329	0.011362	0.011329	0.011698	0.011274
0.011735	0.011273	0.011261	0.011503	0.011589	0.011527	0.011227
0.011218	0.011496	0.011597	0.011705	0.011565	0.011703	0.011834
0.011733	0.011770	0.011732	0.011983	0.011698	0.012573	0.011734
0.012473	0.012241	0.012375	0.012646	0.011698	0.011396	0.011699
0.012137	0.012072	0.011664	0.011564	0.011632	0.011632	0.011601
0.011496	0.011501	0.011564	0.011531	0.011901	0.011967	0.011968
0.011868	0.011968	0.012072	0.011838	0.012138	0.012144	0.012137
0.012205	0.012239	0.012186	0.012205	0.012207	0.011429	0.011732
0.011800	0.012303	0.011806	0.012034	0.012508	0.011570	0.011464
0.011429	0.011429	0.011900	0.011924	0.012045	0.011765	0.011431
S076	0.011698	0.011698	0.012034	0.011799	0.011799	0.011866
0.011705	0.011637	0.011866	0.011295	0.011362	0.011329	0.011295
0.011329	0.011496	0.011295	0.011329	0.011396	0.011362	0.011329
0.011295	0.011329	0.011429	0.011295	0.011329	0.011329	0.011671
0.011564	0.011329	0.011228	0.011463	0.011396	0.011362	0.011396
0.011362	0.011463	0.011295	0.011362	0.011329	0.011362	0.011530
0.011295	0.011362	0.011329	0.011127	0.011429	0.011463	0.011396
0.011362	0.011463	0.011430	0.011698	0.011295	0.011631	0.011295
0.011329	0.011429	0.011429	0.011631	0.011732	0.011429	0.011530
0.011396	0.011463	0.011362	0.011597	0.011832	0.011362	0.011429
0.011329	0.011429	0.011396	0.011463	0.011396	0.000000	0.000000
0.011295	0.011329	0.011295	0.011396	0.011362	0.011397	0.011463
0.011396	0.011362	0.011429	0.011329	0.011463	0.011295	0.011362
0.011530	0.011463	0.011698	0.011530	0.011362	0.011597	0.011295
0.011505	0.011733	0.011551	0.011256	0.011284	0.011355	0.011454
0.012121	0.011467	0.011266	0.011597	0.011597	0.011396	0.011497
0.011429	0.011396	0.011463	0.011362	0.011463	0.011396	0.011396
0.011362	0.011463	0.011664	0.011530	0.011429	0.011396	0.011329
0.011295	0.011295	0.011362	0.011329	0.011530	0.011497	0.011429
0.011530	0.011597	0.011329	0.011329	0.011362	0.011362	0.011364
0.011432	0.011362	0.011362	0.011240	0.011313	0.011271	0.011363
0.011365	0.011301	0.011355	0.011336	0.011264	0.011300	0.011330
0.011600	0.011370	0.011405	0.011296	0.011302	0.011430	0.011396
0.011464	0.011397	0.011396	0.011496	0.011429	0.011799	0.011598
0.011631	0.011497	0.011362	0.011463	0.011262	0.011497	0.011295
0.011362	0.011362	0.011329	0.011295	0.011329	0.011295	0.011396
0.011362	0.011463	0.011396	0.011295	0.011397	0.011497	0.011499
0.011497	0.011396	0.011295	0.011229	0.011362	0.011295	0.011228

0.011332	0.011497	0.011295	0.011497	0.011262	0.011362	0.011329
0.011430	0.011329	0.011362	0.011329	0.011362	0.011228	0.011362
0.011396	0.011329	0.011396	0.011329	0.011362	0.011396	0.011631
0.011732	0.011664	0.011732	0.011431	0.011732	0.011362	0.011329
0.011330	0.011667	0.011426	0.011232	0.011739	0.011231	0.011375
0.011739	0.011638	0.011530	0.011732	0.011436	0.011597	0.011564
0.011496	0.011664	0.011522	0.011390	0.011437	0.011396	0.011466
0.011564	0.011465	0.011601	0.011669	0.011597	0.011665	0.011772
0.011500	0.011671	0.011602	0.011534	0.011800	0.011262	0.011465
0.011429	0.011362	0.011262	0.011262	0.011329	0.011329	0.011262
0.011329	0.011396	0.011295	0.011396	0.011329	0.011329	0.011396
0.011329	0.011765	0.011664	0.011698	0.011228	0.011329	0.011362
0.011362	0.011329	0.011396	0.011429	0.011295	0.011295	0.011295
0.011832	0.011463	0.011463	0.011262	0.011362	0.011530	0.011295
0.011295	0.011362	0.011329	0.011497	0.011295	0.011429	0.011564
0.011496	0.011329	0.011329	0.011262	0.011262	0.011429	0.011362
0.011262	0.011362	0.011228	0.011262	0.011396	0.011530	0.011295
0.011262	0.011396	0.011329	0.011228	0.011564	0.011228	0.011396
0.011471	0.011295	0.011597	0.011430	0.011463	0.011329	0.011362
0.011295	0.011631	0.011228	0.011497	0.011295	0.011295	0.011228
0.011463	0.011665	0.011362	0.011429	0.011262	0.011664	0.011295
0.011295	0.011295	0.011329	0.011429	0.011329	0.011429	0.011496
0.011497	0.011463	0.011161	0.011463	0.011429	0.011228	0.011262
0.011295	0.011228	0.011362	0.011295	0.011262	0.011429	0.011295
0.011329	0.011329	0.011463	0.011496	0.011396	0.011329	0.011329
0.011396	0.011329	0.011429	0.011295	0.011429	0.011530	0.011832
0.011329	0.011732	0.011336	0.017823	0.011257	0.011488	0.011653
0.011614	0.011658	0.011427	0.011292	0.011829	0.011682	0.011364
0.011534	0.011610	0.011216	0.011311	0.011294	0.011406	0.011318
0.011482	0.011507	0.011505	0.011391	0.011463	0.011497	0.011500
0.011369	0.011463	0.011463	0.011429	0.011396	0.011396	0.011363
0.011396	0.011329	0.011362	0.011396	0.011362	0.011295	0.011329
0.011329	0.011396	0.011295	0.011396	0.011430	0.011463	0.011362
0.011396	0.011362	0.011698	0.011698	0.011430	0.011464	0.011497
0.011262	0.011262	0.011295	0.011396	0.011262	0.011497	0.011497
0.011530	0.011530	0.011463	0.011564	0.011732	0.011565	0.011396
0.011396	0.011463	0.011497	0.011329	0.011396	0.011362	0.011396
0.011463	0.011463	0.011497	0.011463	0.011363	0.011564	0.011530
0.011399	0.011295	0.011396	0.011430	0.011396	0.011530	0.011564
0.011732	0.011436	0.011597	0.011564	0.011496	0.011664	0.011295
0.011464	0.011363	0.011329	0.011329	0.011396	0.011295	0.011396
0.011430	0.011463	0.011362	0.011396	0.011362	0.011698	0.011698
0.011430	0.011464	0.011530	0.011463	0.012103	0.011524	0.011447
0.011517	0.011476	0.011587	0.011476	0.011585	0.011508	0.011474
0.012056	0.011372	0.011505	0.011441	0.011714	0.011570	0.011673
0.012080	0.011550	0.011635	0.011698	0.011564	0.011564	0.011601
0.011262	0.011429	0.011564	0.011530	0.011533	0.011530	0.011498
0.011362	0.011295	0.011329	0.011362	0.011262	0.011329	0.011228
0.011362	0.011396	0.011295	0.011262	0.011362	0.011295	0.011329
0.011329	0.011262	0.011329	0.011362	0.011262	0.011463	0.011496
0.011396	0.011463	0.011396	0.011396	0.011329	0.011732	0.011362
0.011408	0.011329	0.011564	0.011569	0.011429	0.011362	0.011429
0.011429	0.011362	0.011329	0.011362	0.011463	0.011463	0.011396
0.011396	0.011530	0.011698	0.011429	0.011631	0.011631	0.011496
0.011329	0.011396	0.011429	0.011832	0.011396	0.011429	0.011396
0.011429	0.011362	0.011362	0.011665	0.011597	0.011631	0.011362
0.011463	0.011530	0.011396	0.011463	0.011429	0.011295	0.011631
0.011396	0.011362	0.011362	0.011530	0.011362	0.011463	0.011698
0.011396	0.011773	0.011765	0.011548	0.011342	0.011468	0.011267
0.011280	0.011800	0.011306	0.011462	0.011353	0.011366	0.011432

0.011468	0.011192	0.011394	0.011192	0.011276	0.011395	0.011425
0.011437	0.011336	0.011412	0.011377	0.011397	0.011530	0.011597
0.011597	0.011464	0.011567	0.011569	0.011704	0.011732	0.011396
0.011631	0.011497	0.011362	0.011463	0.011262	0.011497	0.011295
0.011362	0.011362	0.011329	0.011295	0.011329	0.011295	0.011396
0.011340	0.011340	0.011368	0.011403	0.011539	0.011504	0.011504
0.011503	0.011430	0.011208	0.011204	0.011365	0.011535	0.011535
0.011329	0.011399	0.011632	0.011601	0.011531	0.011565	0.011496
0.011429	0.011496	0.011496	0.011396	0.011967	0.011564	0.011631
0.011665	0.011701	0.011367	0.011441	0.011329	0.011530	0.011497
0.011531	0.011564	0.011564	0.011564	0.011650	0.011698	0.011338
0.011229	0.011233	0.011333	0.011298	0.011402	0.011247	0.011570
0.011369	0.011543	0.011298	0.011397	0.011363	0.011437	0.011329
0.011295	0.011295	0.011362	0.011329	0.011530	0.011497	0.011429
0.011530	0.011597	0.011329	0.011329	0.011362	0.011362	0.011364
0.011432	0.011362	0.011362	0.011397	0.011530	0.011597	0.011597
0.011464	0.011567	0.011569	0.011704	0.011732	0.011396	0.011564
0.011497	0.011497	0.011263	0.011513	0.011442	0.011469	0.011340
0.011508	0.011698	0.011441	0.011400	0.011295	0.011497	0.011362
0.011463	0.011396	0.011295	0.011397	0.011497	0.011499	0.011396
0.011295	0.011229	0.011362	0.011295	0.011228	0.011332	0.011497
0.011396	0.011329	0.011362	0.011396	0.011362	0.011295	0.011497
0.011362	0.011396	0.011396	0.011329	0.011396	0.011631	0.011295
0.011329	0.011732	0.011329	0.011362	0.011329	0.011698	0.011274
0.011735	0.011273	0.011261	0.011503	0.011589	0.011527	0.011227
0.011218	0.011496	0.011597	0.011705	0.011565	0.011703	0.011834
0.011733	0.011770	0.011732	0.011983	0.011698	0.012573	0.011734
0.012473	0.012241	0.012375	0.012646	0.011698	0.011396	0.011699
0.012137	0.012072	0.011664	0.011564	0.011632	0.011632	0.011601
0.011496	0.011501	0.011564	0.011531	0.011901	0.011967	0.011968
0.011868	0.011968	0.012072	0.011838	0.012138	0.012144	0.012137
0.012205	0.012239	0.012186	0.012205	0.012207	0.011429	0.011732
0.011800	0.012303	0.011806	0.012034	0.012508	0.011570	0.011464
0.011429	0.011429	0.011900	0.011924	0.012045	0.011765	0.011431
S077	0.011597	0.011597	0.011933	0.011698	0.011698	0.011765
0.011569	0.011501	0.011765	0.011195	0.011262	0.011228	0.011194
0.011228	0.011396	0.011194	0.011228	0.011295	0.011262	0.011228
0.011194	0.011228	0.011329	0.011195	0.011228	0.011228	0.011535
0.011463	0.011228	0.011127	0.011362	0.011295	0.011262	0.011295
0.011262	0.011362	0.011262	0.011262	0.011295	0.011262	0.011429
0.011194	0.011262	0.011295	0.011228	0.011329	0.011362	0.011295
0.011262	0.011429	0.011329	0.011597	0.011262	0.011530	0.011262
0.011228	0.011329	0.011396	0.011530	0.011631	0.011329	0.011429
0.011295	0.011362	0.011262	0.011496	0.011732	0.011262	0.011329
0.011228	0.011329	0.011295	0.011362	0.011295	0.011295	0.011295
0.000000	0.011228	0.011194	0.011295	0.011262	0.011296	0.011362
0.011295	0.011262	0.011329	0.011228	0.011362	0.011194	0.011262
0.011429	0.011362	0.011597	0.011429	0.011262	0.011496	0.011262
0.011431	0.011658	0.011475	0.011177	0.011172	0.011281	0.011375
0.012011	0.011467	0.011184	0.011496	0.011496	0.011295	0.011396
0.011329	0.011295	0.011362	0.011262	0.011362	0.011295	0.011295
0.011262	0.011362	0.011564	0.011429	0.011329	0.011295	0.011228
0.011195	0.011195	0.011262	0.011228	0.011429	0.011396	0.011329
0.011429	0.011497	0.011228	0.011228	0.011262	0.011262	0.011263
0.011331	0.011262	0.011262	0.011132	0.011205	0.011165	0.011262
0.011263	0.011198	0.011245	0.011233	0.011162	0.011197	0.011229
0.011492	0.011267	0.011301	0.011195	0.011199	0.011329	0.011296
0.011363	0.011296	0.011296	0.011396	0.011329	0.011698	0.011497
0.011530	0.011396	0.011262	0.011362	0.011161	0.011396	0.011195
0.011262	0.011262	0.011228	0.011194	0.011228	0.011195	0.011295

0.011262	0.011362	0.011295	0.011195	0.011296	0.011396	0.011398
0.011396	0.011295	0.011195	0.011128	0.011262	0.011195	0.011127
0.011230	0.011396	0.011195	0.011396	0.011161	0.011262	0.011228
0.011329	0.011228	0.011262	0.011228	0.011262	0.011127	0.011262
0.011295	0.011228	0.011295	0.011228	0.011262	0.011295	0.011530
0.011631	0.011564	0.011631	0.011397	0.011631	0.011262	0.011228
0.011263	0.011545	0.011380	0.011163	0.011637	0.011129	0.011257
0.011603	0.011536	0.011429	0.011631	0.011333	0.011496	0.011463
0.011396	0.011564	0.011415	0.011266	0.011312	0.011295	0.011365
0.011463	0.011364	0.011500	0.011568	0.011496	0.011564	0.011670
0.011398	0.011569	0.011500	0.011432	0.011699	0.011161	0.011363
0.011329	0.011262	0.011161	0.011161	0.011228	0.011228	0.011295
0.011228	0.011295	0.011194	0.011295	0.011228	0.011228	0.011295
0.011228	0.011664	0.011564	0.011597	0.011127	0.011228	0.011262
0.011262	0.011228	0.011295	0.011329	0.011194	0.011194	0.011194
0.011731	0.011362	0.011362	0.011161	0.011262	0.011429	0.011194
0.011194	0.011262	0.011228	0.011396	0.011194	0.011329	0.011463
0.011396	0.011228	0.011228	0.011161	0.011161	0.011329	0.011262
0.011161	0.011262	0.011127	0.011161	0.011295	0.011430	0.011195
0.011161	0.011295	0.011228	0.011127	0.011463	0.011127	0.011295
0.011368	0.011194	0.011496	0.011329	0.011362	0.011228	0.011329
0.011194	0.011530	0.011127	0.011396	0.011194	0.011194	0.011127
0.011362	0.011564	0.011262	0.011329	0.011161	0.011564	0.011194
0.011194	0.011194	0.011228	0.011329	0.011228	0.011329	0.011396
0.011396	0.011362	0.011262	0.011362	0.011329	0.011194	0.011161
0.011194	0.011194	0.011262	0.011194	0.011161	0.011329	0.011194
0.011228	0.011228	0.011362	0.011396	0.011295	0.011228	0.011228
0.011295	0.011228	0.011329	0.011194	0.011329	0.011429	0.011731
0.011228	0.011631	0.011233	0.017747	0.011257	0.011366	0.011583
0.011545	0.011588	0.011322	0.011292	0.011719	0.011612	0.011364
0.011487	0.011548	0.011138	0.011228	0.011216	0.011307	0.011232
0.011482	0.011395	0.011394	0.011193	0.011362	0.011396	0.011398
0.011266	0.011362	0.011362	0.011329	0.011295	0.011295	0.011262
0.011295	0.011228	0.011262	0.011295	0.011262	0.011195	0.011228
0.011228	0.011295	0.011195	0.011295	0.011329	0.011362	0.011262
0.011295	0.011262	0.011597	0.011597	0.011329	0.011363	0.011396
0.011161	0.011161	0.011195	0.011295	0.011161	0.011396	0.011396
0.011429	0.011429	0.011362	0.011463	0.011631	0.011464	0.011295
0.011295	0.011362	0.011396	0.011228	0.011295	0.011262	0.011295
0.011363	0.011362	0.011396	0.011362	0.011262	0.011463	0.011429
0.011297	0.011195	0.011295	0.011396	0.011295	0.011430	0.011463
0.011631	0.011333	0.011496	0.011463	0.011396	0.011564	0.011195
0.011363	0.011262	0.011228	0.011228	0.011295	0.011195	0.011295
0.011329	0.011362	0.011262	0.011295	0.011262	0.011597	0.011597
0.011329	0.011363	0.011429	0.011362	0.012002	0.011417	0.011341
0.011412	0.011372	0.011482	0.011372	0.011480	0.011405	0.011370
0.011953	0.011268	0.011403	0.011337	0.011611	0.011468	0.011570
0.011978	0.011437	0.011533	0.011597	0.011463	0.011463	0.011499
0.011161	0.011329	0.011463	0.011429	0.011432	0.011429	0.011397
0.011262	0.011194	0.011228	0.011262	0.011161	0.011228	0.011127
0.011262	0.011295	0.011194	0.011161	0.011262	0.011194	0.011228
0.011228	0.011161	0.011228	0.011262	0.011161	0.011362	0.011396
0.011295	0.011362	0.011295	0.011295	0.011228	0.011631	0.011262
0.011304	0.011228	0.011463	0.011467	0.011329	0.011329	0.011329
0.011329	0.011262	0.011228	0.011262	0.011362	0.011362	0.011295
0.011295	0.011429	0.011597	0.011329	0.011530	0.011530	0.011396
0.011228	0.011295	0.011329	0.011732	0.011295	0.011329	0.011295
0.011329	0.011262	0.011262	0.011564	0.011497	0.011530	0.011262
0.011362	0.011429	0.011295	0.011362	0.011329	0.011194	0.011530
0.011295	0.011262	0.011262	0.011429	0.011262	0.011362	0.011597

0.011295	0.011637	0.011664	0.011467	0.011229	0.011366	0.011267
0.011170	0.011683	0.011201	0.011417	0.011255	0.011366	0.011379
0.011468	0.011192	0.011338	0.011192	0.011222	0.011395	0.011344
0.011362	0.011257	0.011342	0.011272	0.011296	0.011429	0.011497
0.011497	0.011363	0.011466	0.011467	0.011602	0.011590	0.011295
0.011530	0.011396	0.011262	0.011362	0.011161	0.011396	0.011195
0.011262	0.011262	0.011228	0.011194	0.011228	0.011195	0.011295
0.011270	0.011270	0.011266	0.011300	0.011436	0.011402	0.011402
0.011401	0.011329	0.011134	0.011132	0.011263	0.011433	0.011433
0.011228	0.011297	0.011531	0.011499	0.011430	0.011464	0.011396
0.011329	0.011396	0.011396	0.011295	0.011866	0.011463	0.011530
0.011564	0.011630	0.011265	0.011337	0.011228	0.011429	0.011396
0.011430	0.011463	0.011463	0.011463	0.011610	0.011597	0.011234
0.011128	0.011130	0.011230	0.011196	0.011299	0.011135	0.011468
0.011266	0.011411	0.011156	0.011248	0.011262	0.011334	0.011228
0.011195	0.011195	0.011262	0.011228	0.011429	0.011396	0.011329
0.011429	0.011497	0.011228	0.011228	0.011262	0.011262	0.011263
0.011331	0.011262	0.011262	0.011296	0.011429	0.011497	0.011497
0.011363	0.011466	0.011467	0.011602	0.011590	0.011295	0.011463
0.011396	0.011396	0.011229	0.011408	0.011338	0.011367	0.011235
0.011404	0.011607	0.011399	0.011298	0.011195	0.011396	0.011262
0.011362	0.011295	0.011195	0.011296	0.011396	0.011398	0.011295
0.011195	0.011128	0.011262	0.011195	0.011127	0.011230	0.011396
0.011295	0.011228	0.011262	0.011295	0.011262	0.011195	0.011396
0.011262	0.011295	0.011295	0.011228	0.011295	0.011530	0.011194
0.011228	0.011631	0.011228	0.011262	0.011228	0.011597	0.011189
0.011678	0.011188	0.011261	0.011422	0.011508	0.011419	0.011144
0.011218	0.011396	0.011496	0.011603	0.011464	0.011601	0.011733
0.011632	0.011669	0.011631	0.011880	0.011597	0.012471	0.011633
0.012372	0.012139	0.012273	0.012545	0.011597	0.011295	0.011598
0.012036	0.011971	0.011564	0.011463	0.011531	0.011531	0.011500
0.011396	0.011399	0.011463	0.011430	0.011800	0.011866	0.011867
0.011766	0.011867	0.011970	0.011737	0.012036	0.012042	0.012103
0.012104	0.012138	0.012084	0.012104	0.012105	0.011329	0.011631
0.011699	0.012202	0.011704	0.011933	0.012406	0.011468	0.011363
0.011329	0.011329	0.011799	0.011842	0.011939	0.011664	0.011330
S078	0.011631	0.011631	0.011967	0.011732	0.011732	0.011799
0.011637	0.011569	0.011799	0.011228	0.011295	0.011262	0.011228
0.011262	0.011429	0.011228	0.011262	0.011329	0.011295	0.011262
0.011228	0.011262	0.011362	0.011228	0.011262	0.011262	0.011603
0.011496	0.011262	0.011161	0.011396	0.011329	0.011295	0.011329
0.011295	0.011396	0.011295	0.011295	0.011329	0.011295	0.011463
0.011228	0.011295	0.011329	0.011262	0.011362	0.011396	0.011329
0.011295	0.011463	0.011362	0.011631	0.011295	0.011564	0.011295
0.011262	0.011362	0.011429	0.011564	0.011664	0.011362	0.011463
0.011329	0.011396	0.011295	0.011530	0.011765	0.011295	0.011362
0.011262	0.011362	0.011329	0.011396	0.011329	0.011329	0.011329
0.011228	0.000000	0.011228	0.011329	0.011295	0.011329	0.011396
0.011329	0.011295	0.011362	0.011262	0.011396	0.011228	0.011295
0.011463	0.011396	0.011631	0.011463	0.011295	0.011530	0.011295
0.011431	0.011658	0.011475	0.011177	0.011172	0.011281	0.011375
0.012011	0.011467	0.011184	0.011530	0.011530	0.011329	0.011429
0.011362	0.011329	0.011396	0.011295	0.011396	0.011329	0.011329
0.011295	0.011396	0.011597	0.011463	0.011362	0.011329	0.011262
0.011228	0.011228	0.011295	0.011262	0.011463	0.011430	0.011362
0.011463	0.011530	0.011262	0.011262	0.011295	0.011295	0.011297
0.011364	0.011295	0.011295	0.011168	0.011241	0.011200	0.011296
0.011297	0.011232	0.011245	0.011267	0.011196	0.011231	0.011263
0.011528	0.011301	0.011336	0.011228	0.011233	0.011363	0.011329
0.011396	0.011330	0.011329	0.011429	0.011362	0.011731	0.011530

0.011564	0.011429	0.011295	0.011396	0.011195	0.011429	0.011228
0.011295	0.011295	0.011262	0.011228	0.011262	0.011228	0.011329
0.011295	0.011396	0.011329	0.011228	0.011330	0.011430	0.011432
0.011430	0.011329	0.011228	0.011161	0.011295	0.011228	0.011161
0.011264	0.011430	0.011228	0.011430	0.011195	0.011295	0.011262
0.011362	0.011262	0.011295	0.011262	0.011295	0.011161	0.011295
0.011329	0.011262	0.011329	0.011262	0.011295	0.011329	0.011564
0.011664	0.011597	0.011665	0.011431	0.011665	0.011295	0.011262
0.011297	0.011545	0.011426	0.011163	0.011671	0.011163	0.011257
0.011671	0.011570	0.011463	0.011664	0.011368	0.011530	0.011497
0.011429	0.011597	0.011451	0.011307	0.011312	0.011329	0.011398
0.011497	0.011397	0.011533	0.011601	0.011530	0.011597	0.011704
0.011432	0.011603	0.011534	0.011466	0.011733	0.011194	0.011397
0.011362	0.011295	0.011194	0.011194	0.011262	0.011262	0.011329
0.011262	0.011329	0.011228	0.011329	0.011262	0.011262	0.011329
0.011262	0.011698	0.011597	0.011631	0.011161	0.011262	0.011295
0.011295	0.011262	0.011329	0.011362	0.011228	0.011228	0.011228
0.011765	0.011396	0.011396	0.011194	0.011295	0.011463	0.011228
0.011228	0.011295	0.011262	0.011429	0.011228	0.011362	0.011496
0.011429	0.011262	0.011262	0.011195	0.011194	0.011362	0.011295
0.011195	0.011295	0.011161	0.011195	0.011329	0.011463	0.011228
0.011195	0.011329	0.011262	0.011161	0.011496	0.011161	0.011329
0.011402	0.011228	0.011530	0.011362	0.011396	0.011262	0.011362
0.011228	0.011564	0.011161	0.011429	0.011228	0.011228	0.011161
0.011396	0.011598	0.011295	0.011362	0.011194	0.011597	0.011228
0.011228	0.011228	0.011262	0.011362	0.011262	0.011362	0.011429
0.011429	0.011396	0.011295	0.011396	0.011362	0.011228	0.011194
0.011228	0.011228	0.011295	0.011228	0.011194	0.011362	0.011228
0.011262	0.011262	0.011396	0.011429	0.011329	0.011262	0.011262
0.011329	0.011262	0.011362	0.011228	0.011362	0.011463	0.011765
0.011262	0.011664	0.011233	0.017747	0.011257	0.011366	0.011583
0.011545	0.011588	0.011322	0.011234	0.011719	0.011612	0.011364
0.011487	0.011549	0.011138	0.011186	0.011177	0.011307	0.011232
0.011482	0.011395	0.011394	0.011193	0.011396	0.011429	0.011432
0.011301	0.011396	0.011396	0.011362	0.011329	0.011329	0.011295
0.011329	0.011262	0.011295	0.011329	0.011295	0.011228	0.011262
0.011262	0.011329	0.011228	0.011329	0.011362	0.011396	0.011295
0.011329	0.011295	0.011631	0.011631	0.011362	0.011396	0.011430
0.011195	0.011195	0.011228	0.011329	0.011195	0.011429	0.011429
0.011463	0.011463	0.011396	0.011496	0.011665	0.011498	0.011329
0.011329	0.011396	0.011429	0.011262	0.011329	0.011295	0.011329
0.011396	0.011396	0.011429	0.011396	0.011296	0.011497	0.011463
0.011331	0.011228	0.011329	0.011430	0.011329	0.011463	0.011496
0.011664	0.011368	0.011530	0.011497	0.011429	0.011597	0.011228
0.011397	0.011296	0.011262	0.011262	0.011329	0.011228	0.011329
0.011362	0.011396	0.011295	0.011329	0.011295	0.011631	0.011631
0.011362	0.011396	0.011463	0.011396	0.012035	0.011453	0.011377
0.011447	0.011407	0.011517	0.011406	0.011515	0.011439	0.011405
0.011987	0.011302	0.011437	0.011371	0.011645	0.011502	0.011604
0.012012	0.011475	0.011567	0.011631	0.011496	0.011497	0.011533
0.011194	0.011362	0.011496	0.011463	0.011466	0.011463	0.011431
0.011295	0.011228	0.011262	0.011295	0.011194	0.011262	0.011161
0.011295	0.011329	0.011228	0.011194	0.011295	0.011228	0.011262
0.011262	0.011194	0.011262	0.011295	0.011194	0.011396	0.011429
0.011329	0.011396	0.011329	0.011329	0.011262	0.011664	0.011295
0.011338	0.011262	0.011497	0.011501	0.011362	0.011362	0.011362
0.011362	0.011295	0.011262	0.011295	0.011396	0.011396	0.011329
0.011329	0.011463	0.011631	0.011362	0.011564	0.011564	0.011429
0.011262	0.011329	0.011362	0.011765	0.011329	0.011362	0.011329
0.011362	0.011295	0.011295	0.011597	0.011530	0.011564	0.011295

0.011396	0.011463	0.011329	0.011396	0.011362	0.011228	0.011564
0.011329	0.011295	0.011295	0.011463	0.011295	0.011396	0.011631
0.011329	0.011705	0.011698	0.011467	0.011229	0.011400	0.011267
0.011225	0.011683	0.011201	0.011417	0.011255	0.011366	0.011379
0.011468	0.011192	0.011339	0.011192	0.011222	0.011395	0.011344
0.011362	0.011257	0.011342	0.011272	0.011330	0.011463	0.011530
0.011530	0.011396	0.011500	0.011501	0.011636	0.011661	0.011329
0.011564	0.011429	0.011295	0.011396	0.011195	0.011429	0.011228
0.011295	0.011295	0.011262	0.011228	0.011262	0.011228	0.011329
0.011305	0.011305	0.011300	0.011334	0.011471	0.011436	0.011436
0.011435	0.011363	0.011171	0.011168	0.011297	0.011467	0.011467
0.011262	0.011331	0.011565	0.011533	0.011464	0.011498	0.011429
0.011362	0.011429	0.011429	0.011329	0.011900	0.011496	0.011564
0.011597	0.011665	0.011299	0.011372	0.011262	0.011463	0.011429
0.011464	0.011496	0.011496	0.011496	0.011610	0.011631	0.011268
0.011162	0.011164	0.011264	0.011230	0.011333	0.011172	0.011502
0.011300	0.011411	0.011156	0.011322	0.011296	0.011368	0.011262
0.011228	0.011228	0.011295	0.011262	0.011463	0.011430	0.011362
0.011463	0.011530	0.011262	0.011262	0.011295	0.011295	0.011297
0.011364	0.011295	0.011295	0.011330	0.011463	0.011530	0.011530
0.011396	0.011500	0.011501	0.011636	0.011661	0.011329	0.011496
0.011429	0.011429	0.011263	0.011443	0.011373	0.011401	0.011235
0.011439	0.011607	0.011399	0.011332	0.011228	0.011430	0.011295
0.011396	0.011329	0.011228	0.011330	0.011430	0.011432	0.011329
0.011228	0.011161	0.011295	0.011228	0.011161	0.011264	0.011430
0.011329	0.011262	0.011295	0.011329	0.011295	0.011228	0.011430
0.011295	0.011329	0.011329	0.011262	0.011329	0.011564	0.011228
0.011262	0.011664	0.011262	0.011295	0.011262	0.011631	0.011189
0.011678	0.011188	0.011261	0.011422	0.011508	0.011419	0.011144
0.011218	0.011429	0.011530	0.011637	0.011498	0.011601	0.011766
0.011666	0.011703	0.011664	0.011880	0.011631	0.012505	0.011666
0.012405	0.012173	0.012307	0.012578	0.011631	0.011329	0.011631
0.012070	0.011971	0.011597	0.011497	0.011565	0.011565	0.011534
0.011429	0.011399	0.011496	0.011464	0.011833	0.011900	0.011901
0.011800	0.011901	0.012004	0.011737	0.012070	0.012076	0.012137
0.012138	0.012171	0.012084	0.012138	0.012139	0.011362	0.011665
0.011733	0.012236	0.011738	0.011967	0.012440	0.011502	0.011396
0.011362	0.011362	0.011833	0.011883	0.011975	0.011698	0.011363
S079	0.011597	0.011597	0.011933	0.011698	0.011698	0.011765
0.011603	0.011535	0.011765	0.011195	0.011262	0.011228	0.011194
0.011228	0.011396	0.011194	0.011228	0.011295	0.011262	0.011228
0.011194	0.011228	0.011329	0.011195	0.011228	0.011228	0.011569
0.011463	0.011228	0.011127	0.011362	0.011295	0.011262	0.011295
0.011262	0.011362	0.011262	0.011262	0.011295	0.011262	0.011429
0.011194	0.011262	0.011295	0.011228	0.011329	0.011362	0.011295
0.011262	0.011429	0.011329	0.011597	0.011262	0.011530	0.011262
0.011228	0.011329	0.011396	0.011530	0.011631	0.011329	0.011429
0.011295	0.011362	0.011262	0.011496	0.011732	0.011262	0.011329
0.011228	0.011329	0.011295	0.011362	0.011295	0.011295	0.011295
0.011194	0.011228	0.000000	0.011295	0.011262	0.011296	0.011362
0.011295	0.011262	0.011329	0.011228	0.011362	0.011194	0.011262
0.011429	0.011362	0.011597	0.011429	0.011262	0.011496	0.011262
0.011394	0.011620	0.011437	0.011138	0.011135	0.011244	0.011336
0.011975	0.011416	0.011143	0.011496	0.011496	0.011295	0.011396
0.011329	0.011295	0.011362	0.011262	0.011362	0.011295	0.011295
0.011262	0.011362	0.011564	0.011429	0.011329	0.011295	0.011228
0.011195	0.011195	0.011262	0.011228	0.011429	0.011396	0.011329
0.011429	0.011497	0.011228	0.011228	0.011262	0.011262	0.011263
0.011331	0.011262	0.011262	0.011096	0.011205	0.011165	0.011262
0.011263	0.011198	0.011208	0.011198	0.011162	0.011197	0.011229

0.011456	0.011267	0.011301	0.011195	0.011164	0.011329	0.011296
0.011363	0.011296	0.011296	0.011396	0.011329	0.011698	0.011497
0.011530	0.011396	0.011262	0.011362	0.011161	0.011396	0.011195
0.011262	0.011262	0.011228	0.011195	0.011228	0.011195	0.011295
0.011262	0.011362	0.011295	0.011195	0.011296	0.011396	0.011398
0.011396	0.011295	0.011195	0.011128	0.011262	0.011195	0.011127
0.011230	0.011396	0.011195	0.011396	0.011161	0.011262	0.011228
0.011329	0.011228	0.011262	0.011228	0.011262	0.011127	0.011262
0.011295	0.011228	0.011295	0.011228	0.011262	0.011295	0.011530
0.011631	0.011564	0.011631	0.011397	0.011631	0.011262	0.011228
0.011263	0.011545	0.011380	0.011163	0.011637	0.011129	0.011257
0.011637	0.011536	0.011429	0.011631	0.011333	0.011496	0.011463
0.011396	0.011564	0.011415	0.011266	0.011312	0.011295	0.011365
0.011463	0.011364	0.011500	0.011568	0.011497	0.011564	0.011670
0.011399	0.011569	0.011500	0.011432	0.011699	0.011161	0.011363
0.011329	0.011262	0.011161	0.011161	0.011228	0.011228	0.011295
0.011228	0.011295	0.011194	0.011295	0.011228	0.011228	0.011295
0.011228	0.011664	0.011564	0.011597	0.011127	0.011228	0.011262
0.011262	0.011228	0.011295	0.011329	0.011194	0.011194	0.011194
0.011731	0.011362	0.011362	0.011161	0.011262	0.011429	0.011194
0.011194	0.011262	0.011228	0.011396	0.011194	0.011329	0.011463
0.011396	0.011228	0.011228	0.011161	0.011161	0.011329	0.011262
0.011161	0.011262	0.011127	0.011161	0.011295	0.011430	0.011195
0.011161	0.011295	0.011228	0.011127	0.011463	0.011127	0.011295
0.011368	0.011195	0.011496	0.011329	0.011362	0.011228	0.011329
0.011194	0.011530	0.011127	0.011396	0.011194	0.011194	0.011127
0.011362	0.011564	0.011262	0.011329	0.011161	0.011564	0.011194
0.011194	0.011194	0.011228	0.011329	0.011228	0.011329	0.011396
0.011396	0.011362	0.011262	0.011362	0.011329	0.011194	0.011161
0.011194	0.011194	0.011262	0.011194	0.011161	0.011329	0.011194
0.011228	0.011228	0.011362	0.011396	0.011295	0.011228	0.011228
0.011295	0.011228	0.011329	0.011194	0.011329	0.011429	0.011731
0.011228	0.011631	0.011198	0.017710	0.011191	0.011366	0.011548
0.011510	0.011553	0.011270	0.011234	0.011664	0.011578	0.011303
0.011440	0.011487	0.011099	0.011186	0.011177	0.011258	0.011189
0.011436	0.011358	0.011357	0.011126	0.011362	0.011396	0.011398
0.011266	0.011362	0.011362	0.011329	0.011295	0.011295	0.011262
0.011295	0.011228	0.011262	0.011295	0.011262	0.011195	0.011228
0.011228	0.011295	0.011195	0.011295	0.011329	0.011362	0.011262
0.011295	0.011262	0.011597	0.011597	0.011329	0.011363	0.011396
0.011161	0.011161	0.011195	0.011295	0.011161	0.011396	0.011396
0.011429	0.011429	0.011362	0.011463	0.011631	0.011464	0.011295
0.011295	0.011362	0.011396	0.011228	0.011295	0.011262	0.011295
0.011363	0.011362	0.011396	0.011363	0.011262	0.011463	0.011429
0.011297	0.011195	0.011295	0.011396	0.011295	0.011430	0.011463
0.011631	0.011333	0.011496	0.011463	0.011396	0.011564	0.011195
0.011363	0.011262	0.011228	0.011228	0.011295	0.011195	0.011295
0.011329	0.011362	0.011262	0.011295	0.011262	0.011597	0.011597
0.011329	0.011363	0.011429	0.011362	0.012002	0.011417	0.011341
0.011412	0.011372	0.011482	0.011372	0.011480	0.011405	0.011370
0.011953	0.011268	0.011403	0.011337	0.011611	0.011468	0.011570
0.011978	0.011437	0.011533	0.011597	0.011463	0.011463	0.011499
0.011161	0.011329	0.011463	0.011429	0.011432	0.011429	0.011397
0.011262	0.011194	0.011228	0.011262	0.011161	0.011228	0.011127
0.011262	0.011295	0.011194	0.011161	0.011262	0.011194	0.011228
0.011228	0.011161	0.011228	0.011262	0.011161	0.011362	0.011396
0.011295	0.011362	0.011295	0.011295	0.011228	0.011631	0.011262
0.011304	0.011228	0.011463	0.011467	0.011329	0.011329	0.011329
0.011329	0.011262	0.011228	0.011262	0.011362	0.011362	0.011295
0.011295	0.011429	0.011597	0.011329	0.011530	0.011530	0.011396

0.011228	0.011295	0.011329	0.011732	0.011295	0.011329	0.011295
0.011329	0.011262	0.011262	0.011564	0.011497	0.011530	0.011262
0.011362	0.011429	0.011295	0.011362	0.011329	0.011194	0.011530
0.011295	0.011262	0.011262	0.011429	0.011262	0.011362	0.011597
0.011295	0.011671	0.011664	0.011426	0.011173	0.011332	0.011215
0.011170	0.011644	0.011166	0.011373	0.011207	0.011315	0.011326
0.011417	0.011126	0.011283	0.011126	0.011168	0.011328	0.011303
0.011324	0.011218	0.011307	0.011236	0.011296	0.011429	0.011497
0.011497	0.011363	0.011466	0.011467	0.011603	0.011625	0.011295
0.011530	0.011396	0.011262	0.011362	0.011161	0.011396	0.011195
0.011262	0.011262	0.011228	0.011195	0.011228	0.011195	0.011295
0.011235	0.011270	0.011231	0.011300	0.011436	0.011402	0.011402
0.011401	0.011329	0.011097	0.011096	0.011263	0.011433	0.011433
0.011228	0.011297	0.011531	0.011500	0.011430	0.011464	0.011396
0.011329	0.011396	0.011396	0.011295	0.011866	0.011463	0.011530
0.011564	0.011630	0.011265	0.011337	0.011228	0.011429	0.011396
0.011430	0.011463	0.011463	0.011463	0.011610	0.011597	0.011234
0.011128	0.011130	0.011230	0.011196	0.011299	0.011135	0.011468
0.011266	0.011411	0.011156	0.011285	0.011262	0.011334	0.011228
0.011195	0.011195	0.011262	0.011228	0.011429	0.011396	0.011329
0.011429	0.011497	0.011228	0.011228	0.011262	0.011262	0.011263
0.011331	0.011262	0.011262	0.011296	0.011429	0.011497	0.011497
0.011363	0.011466	0.011467	0.011603	0.011625	0.011295	0.011463
0.011396	0.011396	0.011229	0.011408	0.011338	0.011367	0.011235
0.011404	0.011607	0.011399	0.011298	0.011195	0.011396	0.011262
0.011362	0.011295	0.011195	0.011296	0.011396	0.011398	0.011295
0.011195	0.011128	0.011262	0.011195	0.011127	0.011230	0.011396
0.011295	0.011228	0.011262	0.011295	0.011262	0.011195	0.011396
0.011262	0.011295	0.011295	0.011228	0.011295	0.011530	0.011194
0.011228	0.011631	0.011228	0.011262	0.011228	0.011597	0.011146
0.011622	0.011145	0.011211	0.011382	0.011467	0.011383	0.011102
0.011165	0.011396	0.011496	0.011603	0.011464	0.011601	0.011733
0.011632	0.011669	0.011631	0.011880	0.011597	0.012472	0.011633
0.012372	0.012139	0.012273	0.012545	0.011597	0.011295	0.011598
0.012036	0.011971	0.011564	0.011463	0.011531	0.011531	0.011500
0.011396	0.011399	0.011463	0.011430	0.011800	0.011866	0.011867
0.011766	0.011867	0.011970	0.011737	0.012036	0.012042	0.012103
0.012104	0.012138	0.012050	0.012104	0.012105	0.011329	0.011631
0.011699	0.012202	0.011704	0.011933	0.012406	0.011468	0.011363
0.011329	0.011329	0.011799	0.011842	0.011904	0.011664	0.011330
S080	0.011698	0.011698	0.011967	0.011732	0.011732	0.011799
0.011637	0.011570	0.011799	0.011295	0.011362	0.011329	0.011295
0.011262	0.011497	0.011228	0.011329	0.011396	0.011362	0.011329
0.011295	0.011329	0.011362	0.011295	0.011329	0.011329	0.011603
0.011496	0.011329	0.011228	0.011396	0.011396	0.011362	0.011396
0.011362	0.011463	0.011362	0.011362	0.011396	0.011362	0.011530
0.011228	0.011362	0.011396	0.011329	0.011362	0.011463	0.011396
0.011362	0.011530	0.011430	0.011631	0.011362	0.011631	0.011362
0.011329	0.011362	0.011497	0.011631	0.011664	0.011362	0.011463
0.011396	0.011463	0.011362	0.011597	0.011765	0.011362	0.011429
0.011329	0.011429	0.011396	0.011463	0.011396	0.011396	0.011396
0.011295	0.011329	0.011295	0.000000	0.011362	0.011397	0.011463
0.011396	0.011362	0.011429	0.011329	0.011396	0.011295	0.011362
0.011530	0.011463	0.011698	0.011530	0.011362	0.011597	0.011362
0.011468	0.011620	0.011551	0.011216	0.011247	0.011355	0.011336
0.012011	0.011416	0.011184	0.011597	0.011597	0.011329	0.011430
0.011429	0.011396	0.011463	0.011362	0.011463	0.011396	0.011396
0.011362	0.011396	0.011664	0.011530	0.011429	0.011396	0.011329
0.011295	0.011228	0.011362	0.011329	0.011463	0.011430	0.011362
0.011463	0.011530	0.011329	0.011329	0.011362	0.011362	0.011364

0.011432	0.011362	0.011362	0.011204	0.011277	0.011271	0.011363
0.011365	0.011266	0.011282	0.011302	0.011264	0.011300	0.011330
0.011492	0.011370	0.011370	0.011296	0.011268	0.011430	0.011329
0.011397	0.011397	0.011396	0.011496	0.011429	0.011799	0.011531
0.011564	0.011430	0.011362	0.011396	0.011262	0.011429	0.011295
0.011362	0.011362	0.011329	0.011295	0.011329	0.011295	0.011396
0.011362	0.011463	0.011396	0.011295	0.011397	0.011497	0.011500
0.011497	0.011396	0.011295	0.011229	0.011362	0.011295	0.011228
0.011332	0.011497	0.011295	0.011497	0.011262	0.011363	0.011329
0.011430	0.011329	0.011362	0.011329	0.011362	0.011228	0.011362
0.011396	0.011329	0.011396	0.011329	0.011362	0.011396	0.011631
0.011665	0.011597	0.011665	0.011498	0.011665	0.011362	0.011329
0.011297	0.011545	0.011426	0.011266	0.011671	0.011231	0.011336
0.011671	0.011570	0.011530	0.011732	0.011436	0.011597	0.011564
0.011497	0.011664	0.011522	0.011307	0.011354	0.011396	0.011466
0.011564	0.011465	0.011601	0.011669	0.011597	0.011665	0.011772
0.011500	0.011671	0.011602	0.011534	0.011800	0.011262	0.011465
0.011429	0.011362	0.011262	0.011262	0.011329	0.011329	0.011396
0.011329	0.011396	0.011295	0.011329	0.011329	0.011329	0.011396
0.011329	0.011698	0.011597	0.011631	0.011228	0.011329	0.011362
0.011362	0.011329	0.011396	0.011362	0.011295	0.011295	0.011295
0.011765	0.011329	0.011463	0.011262	0.011362	0.011530	0.011295
0.011295	0.011362	0.011329	0.011497	0.011295	0.011429	0.011564
0.011497	0.011329	0.011329	0.011262	0.011262	0.011430	0.011362
0.011262	0.011362	0.011228	0.011262	0.011396	0.011463	0.011295
0.011262	0.011396	0.011329	0.011228	0.011497	0.011228	0.011396
0.011471	0.011295	0.011530	0.011430	0.011396	0.011329	0.011429
0.011295	0.011631	0.011228	0.011430	0.011228	0.011228	0.011228
0.011463	0.011598	0.011362	0.011429	0.011262	0.011597	0.011228
0.011295	0.011295	0.011329	0.011429	0.011329	0.011429	0.011497
0.011497	0.011463	0.011362	0.011463	0.011429	0.011295	0.011262
0.011295	0.011295	0.011362	0.011228	0.011262	0.011429	0.011295
0.011262	0.011329	0.011463	0.011429	0.011396	0.011329	0.011329
0.011396	0.011329	0.011429	0.011295	0.011429	0.011530	0.011765
0.011329	0.011665	0.011302	0.017823	0.011257	0.011366	0.011583
0.011545	0.011588	0.011374	0.011408	0.011664	0.011613	0.011364
0.011487	0.011487	0.011138	0.011312	0.011216	0.011307	0.011275
0.011483	0.011358	0.011394	0.011126	0.011463	0.011497	0.011500
0.011369	0.011463	0.011463	0.011362	0.011396	0.011396	0.011363
0.011396	0.011329	0.011362	0.011396	0.011363	0.011295	0.011329
0.011329	0.011396	0.011295	0.011396	0.011430	0.011463	0.011362
0.011262	0.011295	0.011631	0.011631	0.011430	0.011464	0.011497
0.011262	0.011262	0.011295	0.011396	0.011262	0.011497	0.011497
0.011530	0.011530	0.011463	0.011564	0.011665	0.011565	0.011396
0.011396	0.011463	0.011497	0.011329	0.011396	0.011362	0.011396
0.011463	0.011463	0.011497	0.011463	0.011363	0.011564	0.011530
0.011399	0.011295	0.011396	0.011497	0.011396	0.011463	0.011564
0.011732	0.011436	0.011597	0.011564	0.011497	0.011664	0.011295
0.011464	0.011363	0.011329	0.011329	0.011396	0.011295	0.011396
0.011430	0.011463	0.011362	0.011262	0.011295	0.011631	0.011631
0.011430	0.011464	0.011530	0.011463	0.012036	0.011489	0.011447
0.011517	0.011442	0.011587	0.011476	0.011550	0.011508	0.011439
0.011987	0.011372	0.011505	0.011441	0.011714	0.011570	0.011673
0.012012	0.011475	0.011635	0.011698	0.011564	0.011497	0.011601
0.011262	0.011429	0.011564	0.011530	0.011533	0.011463	0.011431
0.011362	0.011295	0.011329	0.011362	0.011262	0.011329	0.011228
0.011295	0.011329	0.011295	0.011262	0.011362	0.011295	0.011329
0.011329	0.011262	0.011329	0.011362	0.011262	0.011463	0.011497
0.011396	0.011396	0.011396	0.011396	0.011329	0.011664	0.011362
0.011339	0.011329	0.011497	0.011501	0.011430	0.011429	0.011429

0.011429	0.011362	0.011329	0.011362	0.011463	0.011463	0.011396
0.011396	0.011530	0.011631	0.011429	0.011631	0.011631	0.011496
0.011329	0.011396	0.011429	0.011765	0.011396	0.011430	0.011396
0.011429	0.011295	0.011295	0.011598	0.011597	0.011564	0.011362
0.011463	0.011530	0.011396	0.011463	0.011429	0.011295	0.011564
0.011396	0.011362	0.011295	0.011530	0.011362	0.011463	0.011698
0.011396	0.011705	0.011698	0.011385	0.011117	0.011366	0.011215
0.011225	0.011605	0.011271	0.011328	0.011158	0.011315	0.011273
0.011366	0.011192	0.011339	0.011192	0.011222	0.011395	0.011385
0.011399	0.011296	0.011307	0.011342	0.011397	0.011463	0.011530
0.011530	0.011397	0.011500	0.011501	0.011637	0.011661	0.011396
0.011564	0.011430	0.011362	0.011396	0.011262	0.011429	0.011295
0.011362	0.011362	0.011329	0.011295	0.011329	0.011295	0.011396
0.011305	0.011340	0.011334	0.011403	0.011539	0.011470	0.011504
0.011503	0.011430	0.011208	0.011204	0.011365	0.011535	0.011535
0.011329	0.011399	0.011633	0.011567	0.011531	0.011565	0.011496
0.011429	0.011497	0.011496	0.011396	0.011900	0.011564	0.011631
0.011665	0.011666	0.011367	0.011441	0.011329	0.011530	0.011497
0.011531	0.011564	0.011564	0.011564	0.011729	0.011698	0.011338
0.011229	0.011233	0.011333	0.011298	0.011334	0.011247	0.011502
0.011369	0.011367	0.011203	0.011397	0.011363	0.011437	0.011329
0.011295	0.011228	0.011362	0.011329	0.011463	0.011430	0.011362
0.011463	0.011530	0.011329	0.011329	0.011362	0.011362	0.011364
0.011432	0.011362	0.011362	0.011397	0.011463	0.011530	0.011530
0.011397	0.011500	0.011501	0.011637	0.011661	0.011396	0.011564
0.011497	0.011497	0.011331	0.011513	0.011442	0.011469	0.011341
0.011508	0.011698	0.011484	0.011400	0.011295	0.011497	0.011362
0.011463	0.011396	0.011295	0.011397	0.011497	0.011500	0.011396
0.011295	0.011229	0.011362	0.011295	0.011228	0.011332	0.011497
0.011396	0.011329	0.011362	0.011396	0.011363	0.011295	0.011430
0.011362	0.011396	0.011396	0.011329	0.011396	0.011631	0.011228
0.011329	0.011664	0.011329	0.011362	0.011329	0.011631	0.011189
0.011566	0.011188	0.011110	0.011342	0.011427	0.011383	0.011144
0.011165	0.011429	0.011530	0.011637	0.011498	0.011635	0.011766
0.011666	0.011703	0.011664	0.011915	0.011631	0.012505	0.011667
0.012406	0.012173	0.012307	0.012579	0.011631	0.011329	0.011631
0.012070	0.012005	0.011597	0.011497	0.011565	0.011565	0.011534
0.011429	0.011433	0.011496	0.011464	0.011834	0.011900	0.011901
0.011767	0.011901	0.012004	0.011771	0.012070	0.012076	0.012137
0.012138	0.012172	0.012084	0.012138	0.012140	0.011429	0.011665
0.011733	0.012236	0.011738	0.011967	0.012440	0.011502	0.011396
0.011362	0.011362	0.011833	0.011842	0.011940	0.011698	0.011363
S081 0.011664	0.011664	0.012000	0.011765	0.011765	0.011832	
0.011671	0.011603	0.011832	0.011262	0.011329	0.011296	0.011262
0.011295	0.011463	0.011262	0.011295	0.011362	0.011329	0.011295
0.011262	0.011295	0.011396	0.011262	0.011295	0.011295	0.011637
0.011530	0.011295	0.011194	0.011429	0.011362	0.011329	0.011362
0.011329	0.011429	0.011329	0.011329	0.011362	0.011329	0.011496
0.011262	0.011329	0.011362	0.011295	0.011396	0.011429	0.011362
0.011329	0.011497	0.011396	0.011664	0.011329	0.011597	0.011329
0.011295	0.011396	0.011463	0.011597	0.011698	0.011396	0.011496
0.011362	0.011429	0.011329	0.011563	0.011799	0.011329	0.011396
0.011295	0.011396	0.011362	0.011429	0.011362	0.011362	0.011362
0.011262	0.011295	0.011262	0.011362	0.000000	0.011363	0.011429
0.011362	0.011329	0.011396	0.011295	0.011429	0.011262	0.011329
0.011496	0.011429	0.011664	0.011496	0.011329	0.011564	0.011329
0.011505	0.011732	0.011551	0.011216	0.011247	0.011355	0.011454
0.012085	0.011416	0.011266	0.011563	0.011564	0.011362	0.011463
0.011396	0.011362	0.011429	0.011329	0.011429	0.011362	0.011362
0.011329	0.011429	0.011631	0.011496	0.011396	0.011362	0.011295

0.011262	0.011262	0.011329	0.011295	0.011497	0.011463	0.011396
0.011497	0.011564	0.011295	0.011295	0.011329	0.011329	0.011330
0.011398	0.011329	0.011329	0.011204	0.011277	0.011235	0.011329
0.011331	0.011266	0.011318	0.011302	0.011230	0.011266	0.011297
0.011564	0.011336	0.011370	0.011262	0.011268	0.011397	0.011363
0.011430	0.011363	0.011363	0.011463	0.011396	0.011765	0.011564
0.011597	0.011463	0.011329	0.011429	0.011228	0.011463	0.011262
0.011329	0.011329	0.011295	0.011262	0.011295	0.011262	0.011362
0.011329	0.011429	0.011362	0.011262	0.011363	0.011463	0.011466
0.011463	0.011362	0.011262	0.011195	0.011329	0.011262	0.011195
0.011298	0.011463	0.011262	0.011463	0.011228	0.011329	0.011295
0.011396	0.011295	0.011329	0.011295	0.011329	0.011195	0.011329
0.011362	0.011295	0.011362	0.011295	0.011329	0.011362	0.011597
0.011698	0.011631	0.011698	0.011465	0.011698	0.011329	0.011295
0.011330	0.011586	0.011426	0.011232	0.011705	0.011197	0.011296
0.011705	0.011604	0.011496	0.011698	0.011402	0.011564	0.011530
0.011463	0.011631	0.011486	0.011266	0.011354	0.011362	0.011432
0.011530	0.011431	0.011567	0.011635	0.011564	0.011631	0.011738
0.011466	0.011637	0.011568	0.011500	0.011766	0.011228	0.011431
0.011396	0.011329	0.011228	0.011228	0.011295	0.011295	0.011362
0.011295	0.011362	0.011262	0.011362	0.011295	0.011295	0.011362
0.011295	0.011731	0.011631	0.011664	0.011194	0.011295	0.011329
0.011329	0.011295	0.011362	0.011396	0.011262	0.011262	0.011262
0.011799	0.011429	0.011396	0.011228	0.011329	0.011497	0.011262
0.011262	0.011329	0.011295	0.011463	0.011262	0.011396	0.011530
0.011463	0.011295	0.011295	0.011228	0.011228	0.011396	0.011329
0.011228	0.011329	0.011194	0.011228	0.011362	0.011497	0.011262
0.011228	0.011362	0.011295	0.011194	0.011530	0.011195	0.011362
0.011437	0.011262	0.011564	0.011396	0.011429	0.011295	0.011396
0.011262	0.011597	0.011194	0.011463	0.011262	0.011262	0.011194
0.011429	0.011631	0.011329	0.011362	0.011228	0.011631	0.011262
0.011262	0.011262	0.011295	0.011396	0.011295	0.011396	0.011463
0.011463	0.011429	0.011329	0.011429	0.011396	0.011262	0.011228
0.011262	0.011262	0.011329	0.011262	0.011228	0.011396	0.011262
0.011295	0.011295	0.011429	0.011463	0.011362	0.011295	0.011295
0.011362	0.011295	0.011396	0.011262	0.011396	0.011496	0.011799
0.011295	0.011698	0.011302	0.017823	0.011257	0.011366	0.011653
0.011614	0.011658	0.011322	0.011234	0.011664	0.011682	0.011303
0.011487	0.011487	0.011216	0.011228	0.011294	0.011258	0.011232
0.011482	0.011470	0.011468	0.011325	0.011429	0.011463	0.011466
0.011335	0.011429	0.011429	0.011396	0.011362	0.011362	0.011329
0.011362	0.011295	0.011329	0.011362	0.011329	0.011262	0.011295
0.011295	0.011362	0.011262	0.011362	0.011396	0.011429	0.011329
0.011362	0.011329	0.011665	0.011665	0.011396	0.011430	0.011463
0.011228	0.011228	0.011262	0.011362	0.011228	0.011463	0.011463
0.011497	0.011496	0.011429	0.011530	0.011698	0.011532	0.011362
0.011362	0.011429	0.011463	0.011295	0.011362	0.011329	0.011362
0.011430	0.011430	0.011463	0.011430	0.011330	0.011530	0.011497
0.011365	0.011262	0.011362	0.011463	0.011362	0.011497	0.011530
0.011698	0.011402	0.011564	0.011530	0.011463	0.011631	0.011262
0.011431	0.011329	0.011295	0.011295	0.011362	0.011262	0.011362
0.011396	0.011429	0.011329	0.011362	0.011329	0.011665	0.011665
0.011396	0.011430	0.011496	0.011429	0.012069	0.011489	0.011412
0.011482	0.011441	0.011552	0.011441	0.011550	0.011474	0.011439
0.012021	0.011337	0.011471	0.011406	0.011680	0.011536	0.011638
0.012046	0.011512	0.011601	0.011664	0.011530	0.011530	0.011567
0.011228	0.011396	0.011530	0.011496	0.011499	0.011496	0.011464
0.011329	0.011262	0.011295	0.011329	0.011228	0.011295	0.011194
0.011329	0.011362	0.011262	0.011228	0.011329	0.011262	0.011295
0.011295	0.011228	0.011295	0.011329	0.011228	0.011429	0.011463

0.011362	0.011429	0.011362	0.011362	0.011295	0.011698	0.011329
0.011373	0.011295	0.011530	0.011535	0.011396	0.011396	0.011396
0.011396	0.011329	0.011295	0.011329	0.011429	0.011429	0.011362
0.011362	0.011496	0.011664	0.011329	0.011597	0.011597	0.011463
0.011295	0.011362	0.011396	0.011799	0.011362	0.011396	0.011362
0.011396	0.011329	0.011329	0.011631	0.011564	0.011597	0.011329
0.011429	0.011496	0.011362	0.011429	0.011396	0.011262	0.011597
0.011362	0.011329	0.011329	0.011496	0.011329	0.011396	0.011664
0.011362	0.011739	0.011731	0.011507	0.011286	0.011434	0.011215
0.011170	0.011761	0.011271	0.011462	0.011353	0.011416	0.011379
0.011468	0.011192	0.011283	0.011126	0.011222	0.011328	0.011425
0.011437	0.011336	0.011412	0.011342	0.011363	0.011497	0.011564
0.011564	0.011430	0.011533	0.011535	0.011670	0.011696	0.011362
0.011597	0.011463	0.011329	0.011429	0.011228	0.011463	0.011262
0.011329	0.011329	0.011295	0.011262	0.011295	0.011262	0.011362
0.011340	0.011305	0.011334	0.011368	0.011505	0.011470	0.011470
0.011469	0.011396	0.011171	0.011204	0.011331	0.011501	0.011501
0.011296	0.011365	0.011599	0.011567	0.011497	0.011531	0.011463
0.011396	0.011463	0.011463	0.011362	0.011933	0.011530	0.011597
0.011631	0.011701	0.011333	0.011372	0.011295	0.011497	0.011463
0.011497	0.011530	0.011530	0.011530	0.011650	0.011664	0.011303
0.011195	0.011199	0.011298	0.011264	0.011368	0.011209	0.011536
0.011335	0.011499	0.011156	0.011360	0.011329	0.011402	0.011295
0.011262	0.011262	0.011329	0.011295	0.011497	0.011463	0.011396
0.011497	0.011564	0.011295	0.011295	0.011329	0.011329	0.011330
0.011398	0.011329	0.011329	0.011363	0.011497	0.011564	0.011564
0.011430	0.011533	0.011535	0.011670	0.011696	0.011362	0.011530
0.011463	0.011463	0.011297	0.011478	0.011408	0.011401	0.011305
0.011473	0.011652	0.011441	0.011366	0.011262	0.011463	0.011329
0.011429	0.011362	0.011262	0.011363	0.011463	0.011466	0.011362
0.011262	0.011195	0.011329	0.011262	0.011195	0.011298	0.011463
0.011362	0.011295	0.011329	0.011362	0.011329	0.011262	0.011463
0.011329	0.011362	0.011362	0.011295	0.011362	0.011597	0.011262
0.011295	0.011698	0.011295	0.011329	0.011295	0.011664	0.011232
0.011735	0.011230	0.011261	0.011503	0.011589	0.011491	0.011186
0.011218	0.011463	0.011564	0.011671	0.011531	0.011669	0.011800
0.011699	0.011737	0.011698	0.011949	0.011664	0.012539	0.011700
0.012439	0.012207	0.012341	0.012612	0.011664	0.011362	0.011665
0.012103	0.012038	0.011631	0.011530	0.011598	0.011598	0.011567
0.011463	0.011467	0.011530	0.011497	0.011867	0.011933	0.011935
0.011834	0.011934	0.012038	0.011804	0.012104	0.012110	0.012171
0.012171	0.012205	0.012152	0.012172	0.012173	0.011396	0.011699
0.011766	0.012202	0.011772	0.012001	0.012474	0.011536	0.011430
0.011396	0.011396	0.011867	0.011924	0.012010	0.011731	0.011397
S082	0.011700	0.011700	0.012037	0.011801	0.011801	0.011868
0.011673	0.011571	0.011801	0.011296	0.011363	0.011330	0.011296
0.011329	0.011498	0.011296	0.011329	0.011397	0.011363	0.011329
0.011296	0.011329	0.011430	0.011296	0.011330	0.011329	0.011639
0.011565	0.011329	0.011228	0.011464	0.011363	0.011363	0.011397
0.011363	0.011464	0.011363	0.011363	0.011397	0.011363	0.011531
0.011296	0.011363	0.011397	0.011329	0.011430	0.011464	0.011397
0.011363	0.011531	0.011431	0.011700	0.011363	0.011632	0.011363
0.011329	0.011430	0.011498	0.011632	0.011733	0.011430	0.011531
0.011397	0.011464	0.011363	0.011599	0.011835	0.011363	0.011430
0.011329	0.011430	0.011397	0.011464	0.011397	0.011397	0.011397
0.011296	0.011329	0.011296	0.011397	0.011363	0.000000	0.011464
0.011397	0.011363	0.011430	0.011329	0.011464	0.011296	0.011363
0.011531	0.011464	0.011700	0.011464	0.011363	0.011599	0.011363
0.011505	0.011732	0.011513	0.011216	0.011209	0.011355	0.011454
0.012048	0.011467	0.011184	0.011599	0.011599	0.011363	0.011498

0.011430	0.011397	0.011464	0.011363	0.011464	0.011363	0.011397
0.011363	0.011464	0.011666	0.011531	0.011430	0.011397	0.011329
0.011296	0.011296	0.011363	0.011329	0.011531	0.011498	0.011430
0.011531	0.011599	0.011329	0.011329	0.011363	0.011363	0.011331
0.011399	0.011363	0.011363	0.011204	0.011278	0.011271	0.011363
0.011366	0.011267	0.011318	0.011337	0.011264	0.011300	0.011331
0.011601	0.011371	0.011405	0.011296	0.011303	0.011431	0.011397
0.011465	0.011398	0.011397	0.011498	0.011430	0.011733	0.011599
0.011632	0.011498	0.011363	0.011464	0.011262	0.011498	0.011296
0.011363	0.011363	0.011329	0.011296	0.011329	0.011296	0.011397
0.011363	0.011464	0.011397	0.011296	0.011398	0.011498	0.011500
0.011498	0.011397	0.011296	0.011229	0.011363	0.011296	0.011229
0.011298	0.011498	0.011296	0.011498	0.011262	0.011363	0.011329
0.011430	0.011329	0.011363	0.011329	0.011363	0.011228	0.011363
0.011397	0.011329	0.011397	0.011329	0.011363	0.011397	0.011599
0.011733	0.011666	0.011733	0.011498	0.011733	0.011363	0.011329
0.011364	0.011586	0.011381	0.011266	0.011740	0.011231	0.011336
0.011707	0.011639	0.011531	0.011733	0.011437	0.011599	0.011565
0.011498	0.011666	0.011488	0.011391	0.011355	0.011397	0.011467
0.011565	0.011466	0.011603	0.011671	0.011599	0.011666	0.011774
0.011501	0.011673	0.011603	0.011535	0.011802	0.011262	0.011466
0.011430	0.011363	0.011262	0.011262	0.011329	0.011329	0.011397
0.011329	0.011397	0.011296	0.011397	0.011329	0.011329	0.011397
0.011329	0.011767	0.011666	0.011700	0.011228	0.011329	0.011363
0.011363	0.011329	0.011397	0.011430	0.011296	0.011296	0.011296
0.011834	0.011464	0.011464	0.011262	0.011363	0.011531	0.011296
0.011296	0.011363	0.011329	0.011498	0.011296	0.011430	0.011565
0.011498	0.011329	0.011329	0.011262	0.011262	0.011430	0.011363
0.011262	0.011363	0.011229	0.011262	0.011397	0.011532	0.011296
0.011262	0.011397	0.011330	0.011229	0.011565	0.011229	0.011397
0.011472	0.011296	0.011599	0.011431	0.011464	0.011329	0.011430
0.011296	0.011632	0.011228	0.011498	0.011296	0.011296	0.011228
0.011464	0.011666	0.011363	0.011430	0.011262	0.011666	0.011296
0.011296	0.011296	0.011329	0.011430	0.011329	0.011430	0.011498
0.011430	0.011464	0.011363	0.011464	0.011430	0.011296	0.011262
0.011296	0.011296	0.011363	0.011296	0.011262	0.011430	0.011296
0.011329	0.011329	0.011464	0.011498	0.011397	0.011329	0.011330
0.011397	0.011329	0.011430	0.011296	0.011430	0.011531	0.011834
0.011329	0.011733	0.011302	0.017823	0.011191	0.011488	0.011653
0.011648	0.011658	0.011374	0.011292	0.011774	0.011716	0.011364
0.011487	0.011610	0.011216	0.011270	0.011255	0.011356	0.011275
0.011482	0.011470	0.011431	0.011193	0.011464	0.011498	0.011501
0.011370	0.011464	0.011464	0.011430	0.011363	0.011363	0.011363
0.011397	0.011329	0.011363	0.011397	0.011363	0.011296	0.011329
0.011329	0.011397	0.011296	0.011397	0.011430	0.011464	0.011363
0.011329	0.011363	0.011700	0.011700	0.011431	0.011465	0.011498
0.011262	0.011262	0.011296	0.011397	0.011262	0.011498	0.011498
0.011531	0.011531	0.011464	0.011565	0.011733	0.011566	0.011397
0.011397	0.011464	0.011498	0.011329	0.011397	0.011363	0.011397
0.011464	0.011464	0.011498	0.011464	0.011364	0.011565	0.011531
0.011400	0.011296	0.011397	0.011498	0.011397	0.011531	0.011565
0.011733	0.011437	0.011599	0.011565	0.011498	0.011666	0.011296
0.011465	0.011363	0.011329	0.011329	0.011397	0.011296	0.011397
0.011430	0.011464	0.011363	0.011329	0.011363	0.011700	0.011700
0.011431	0.011465	0.011531	0.011464	0.012106	0.011526	0.011448
0.011483	0.011477	0.011589	0.011477	0.011587	0.011510	0.011475
0.012059	0.011373	0.011507	0.011442	0.011716	0.011572	0.011674
0.012083	0.011551	0.011365	0.011397	0.011397	0.011430	0.011331
0.011262	0.011296	0.011430	0.011397	0.011365	0.011531	0.011498
0.011363	0.011296	0.011329	0.011363	0.011262	0.011329	0.011228

0.011363	0.011397	0.011296	0.011262	0.011363	0.011296	0.011329
0.011329	0.011262	0.011329	0.011363	0.011262	0.011464	0.011498
0.011329	0.011464	0.011397	0.011397	0.011329	0.011733	0.011363
0.011409	0.011329	0.011565	0.011570	0.011430	0.011430	0.011430
0.011430	0.011363	0.011329	0.011363	0.011464	0.011464	0.011397
0.011397	0.011531	0.011700	0.011430	0.011632	0.011632	0.011498
0.011329	0.011397	0.011430	0.011835	0.011397	0.011430	0.011397
0.011430	0.011363	0.011363	0.011666	0.011599	0.011632	0.011363
0.011464	0.011531	0.011397	0.011464	0.011430	0.011296	0.011632
0.011397	0.011363	0.011363	0.011531	0.011363	0.011464	0.011700
0.011397	0.011741	0.011767	0.011548	0.011229	0.011469	0.011267
0.011280	0.011722	0.011271	0.011417	0.011255	0.011366	0.011379
0.011468	0.011126	0.011339	0.011192	0.011222	0.011395	0.011425
0.011437	0.011336	0.011412	0.011342	0.011398	0.011531	0.011599
0.011599	0.011464	0.011568	0.011570	0.011706	0.011698	0.011363
0.011632	0.011498	0.011363	0.011464	0.011262	0.011498	0.011296
0.011363	0.011363	0.011329	0.011296	0.011329	0.011296	0.011397
0.011375	0.011376	0.011369	0.011403	0.011540	0.011505	0.011505
0.011504	0.011431	0.011245	0.011241	0.011366	0.011535	0.011536
0.011330	0.011400	0.011634	0.011534	0.011532	0.011566	0.011498
0.011430	0.011498	0.011498	0.011397	0.011969	0.011565	0.011633
0.011666	0.011703	0.011368	0.011442	0.011329	0.011531	0.011498
0.011532	0.011565	0.011565	0.011565	0.011690	0.011700	0.011338
0.011230	0.011199	0.011333	0.011299	0.011403	0.011210	0.011571
0.011370	0.011412	0.011156	0.011361	0.011363	0.011437	0.011329
0.011296	0.011296	0.011363	0.011329	0.011531	0.011498	0.011430
0.011531	0.011599	0.011329	0.011329	0.011363	0.011363	0.011331
0.011399	0.011363	0.011363	0.011398	0.011531	0.011599	0.011599
0.011464	0.011568	0.011570	0.011706	0.011698	0.011363	0.011565
0.011498	0.011498	0.011331	0.011479	0.011443	0.011470	0.011340
0.011508	0.011744	0.011442	0.011401	0.011296	0.011498	0.011363
0.011464	0.011397	0.011296	0.011398	0.011498	0.011500	0.011397
0.011296	0.011229	0.011363	0.011296	0.011229	0.011298	0.011498
0.011397	0.011329	0.011363	0.011397	0.011363	0.011296	0.011464
0.011363	0.011397	0.011397	0.011329	0.011397	0.011632	0.011296
0.011329	0.011733	0.011329	0.011363	0.011329	0.011700	0.011189
0.011678	0.011230	0.011261	0.011463	0.011549	0.011491	0.011144
0.011218	0.011498	0.011599	0.011706	0.011565	0.011703	0.011834
0.011735	0.011772	0.011733	0.011949	0.011700	0.012577	0.011734
0.012476	0.012241	0.012378	0.012646	0.011700	0.011397	0.011700
0.012139	0.011836	0.011666	0.011565	0.011633	0.011633	0.011603
0.011498	0.011501	0.011565	0.011532	0.011902	0.011969	0.011970
0.011870	0.011969	0.012072	0.011838	0.012139	0.012146	0.012207
0.012207	0.012241	0.012186	0.012140	0.012207	0.011430	0.011734
0.011802	0.012273	0.011806	0.012037	0.011801	0.011570	0.011464
0.011430	0.011430	0.011902	0.011926	0.012012	0.011733	0.011431
S083	0.011765	0.011765	0.012101	0.011866	0.011866	0.011933
0.011739	0.011671	0.011933	0.011362	0.011429	0.011397	0.011362
0.011396	0.011564	0.011362	0.011396	0.011463	0.011429	0.011396
0.011362	0.011396	0.011496	0.011363	0.011396	0.011396	0.011705
0.011631	0.011396	0.011295	0.011530	0.011463	0.011429	0.011463
0.011429	0.011530	0.011429	0.011429	0.011463	0.011429	0.011597
0.011362	0.011429	0.011463	0.011396	0.011496	0.011530	0.011463
0.011429	0.011597	0.011497	0.011765	0.011429	0.011698	0.011429
0.011396	0.011497	0.011564	0.011698	0.011799	0.011496	0.011597
0.011463	0.011530	0.011429	0.011664	0.011900	0.011429	0.011496
0.011396	0.011496	0.011463	0.011530	0.011463	0.011463	0.011463
0.011362	0.011396	0.011362	0.011463	0.011429	0.011464	0.000000
0.011463	0.011429	0.011496	0.011396	0.011530	0.011362	0.011429
0.011262	0.011530	0.011765	0.011597	0.011429	0.011664	0.011429

0.011616	0.011845	0.011664	0.011373	0.011321	0.011465	0.011533
0.012158	0.011670	0.011348	0.011664	0.011664	0.011463	0.011564
0.011496	0.011463	0.011530	0.011429	0.011530	0.011463	0.011463
0.011429	0.011530	0.011731	0.011597	0.011496	0.011463	0.011396
0.011362	0.011362	0.011429	0.011396	0.011597	0.011564	0.011497
0.011597	0.011665	0.011396	0.011396	0.011430	0.011429	0.011432
0.011500	0.011429	0.011429	0.011312	0.011349	0.011306	0.011430
0.011433	0.011369	0.011429	0.011405	0.011332	0.011368	0.011398
0.011636	0.011439	0.011473	0.011363	0.011371	0.011497	0.011464
0.011531	0.011465	0.011464	0.011564	0.011496	0.011866	0.011665
0.011698	0.011564	0.011429	0.011530	0.011329	0.011564	0.011362
0.011429	0.011429	0.011396	0.011362	0.011396	0.011362	0.011463
0.011429	0.011530	0.011463	0.011362	0.011464	0.011564	0.011567
0.011564	0.011463	0.011362	0.011296	0.011429	0.011362	0.011295
0.011400	0.011564	0.011362	0.011564	0.011329	0.011430	0.011396
0.011497	0.011396	0.011429	0.011396	0.011429	0.011295	0.011429
0.011463	0.011396	0.011463	0.011396	0.011429	0.011463	0.011698
0.011799	0.011732	0.011799	0.011566	0.011799	0.011429	0.011396
0.011432	0.011747	0.011609	0.011335	0.011807	0.011299	0.011414
0.011773	0.011706	0.011597	0.011799	0.011504	0.011664	0.011631
0.011564	0.011732	0.011557	0.011431	0.011437	0.011463	0.011500
0.011631	0.011532	0.011669	0.011737	0.011664	0.011732	0.011839
0.011568	0.011739	0.011669	0.011601	0.011867	0.011329	0.011532
0.011496	0.011429	0.011329	0.011329	0.011396	0.011396	0.011463
0.011396	0.011463	0.011362	0.011463	0.011396	0.011396	0.011463
0.011396	0.011832	0.011731	0.011765	0.011295	0.011396	0.011429
0.011429	0.011396	0.011463	0.011496	0.011362	0.011362	0.011362
0.011899	0.011530	0.011530	0.011329	0.011429	0.011597	0.011362
0.011362	0.011429	0.011396	0.011564	0.011362	0.011496	0.011631
0.011564	0.011396	0.011396	0.011329	0.011329	0.011496	0.011429
0.011329	0.011429	0.011295	0.011329	0.011463	0.011598	0.011362
0.011329	0.011463	0.011396	0.011295	0.011631	0.011295	0.011463
0.011539	0.011362	0.011664	0.011497	0.011530	0.011396	0.011496
0.011362	0.011698	0.011295	0.011564	0.011362	0.011362	0.011295
0.011530	0.011732	0.011429	0.011496	0.011329	0.011731	0.011362
0.011362	0.011362	0.011396	0.011496	0.011396	0.011496	0.011564
0.011564	0.011530	0.011429	0.011530	0.011496	0.011362	0.011329
0.011362	0.011362	0.011429	0.011362	0.011329	0.011496	0.011362
0.011396	0.011396	0.011530	0.011564	0.011463	0.011396	0.011396
0.011463	0.011396	0.011496	0.011362	0.011496	0.011597	0.011899
0.011396	0.011799	0.011405	0.017898	0.011387	0.011549	0.011758
0.011718	0.011764	0.011427	0.011350	0.011884	0.011785	0.011485
0.011629	0.011732	0.011333	0.011437	0.011411	0.011455	0.011404
0.011623	0.011544	0.011542	0.011325	0.011530	0.011564	0.011567
0.011438	0.011530	0.011530	0.011497	0.011463	0.011463	0.011430
0.011463	0.011396	0.011429	0.011463	0.011430	0.011362	0.011396
0.011396	0.011463	0.011362	0.011463	0.011497	0.011530	0.011429
0.011463	0.011429	0.011765	0.011765	0.011497	0.011531	0.011564
0.011329	0.011329	0.011362	0.011463	0.011329	0.011564	0.011564
0.011597	0.011597	0.011530	0.011631	0.011799	0.011599	0.011463
0.011463	0.011530	0.011564	0.011396	0.011463	0.011429	0.011463
0.011531	0.011530	0.011564	0.011530	0.011431	0.011631	0.011597
0.011399	0.011362	0.011463	0.011564	0.011463	0.011597	0.011631
0.011799	0.011504	0.011664	0.011631	0.011564	0.011732	0.011363
0.011532	0.011430	0.011396	0.011396	0.011463	0.011362	0.011463
0.011497	0.011530	0.011429	0.011463	0.011429	0.011765	0.011765
0.011497	0.011531	0.011597	0.011530	0.012170	0.011596	0.011482
0.011587	0.011545	0.011657	0.011545	0.011655	0.011577	0.011543
0.012124	0.011441	0.011574	0.011510	0.011783	0.011638	0.011741
0.012148	0.011588	0.011703	0.011765	0.011631	0.011631	0.011668

0.011329	0.011497	0.011631	0.011597	0.011601	0.011597	0.011565
0.011429	0.011362	0.011396	0.011429	0.011329	0.011396	0.011295
0.011429	0.011463	0.011362	0.011329	0.011429	0.011362	0.011396
0.011396	0.011329	0.011396	0.011429	0.011329	0.011530	0.011564
0.011463	0.011530	0.011463	0.011463	0.011396	0.011799	0.011429
0.011443	0.011396	0.011631	0.011637	0.011497	0.011496	0.011496
0.011496	0.011429	0.011396	0.011429	0.011530	0.011530	0.011463
0.011463	0.011597	0.011765	0.011496	0.011698	0.011698	0.011496
0.011396	0.011463	0.011496	0.011900	0.011463	0.011497	0.011463
0.011496	0.011429	0.011429	0.011732	0.011664	0.011698	0.011429
0.011530	0.011597	0.011463	0.011530	0.011496	0.011362	0.011698
0.011463	0.011429	0.011429	0.011597	0.011429	0.011530	0.011765
0.011463	0.011807	0.011832	0.011629	0.011399	0.011536	0.011421
0.011335	0.011839	0.011376	0.011596	0.011451	0.011518	0.011591
0.011621	0.011324	0.011505	0.011324	0.011437	0.011596	0.011547
0.011550	0.011414	0.011518	0.011412	0.011465	0.011597	0.011665
0.011665	0.011531	0.011635	0.011603	0.011772	0.011802	0.011463
0.011698	0.011564	0.011429	0.011530	0.011329	0.011564	0.011362
0.011429	0.011429	0.011396	0.011362	0.011396	0.011362	0.011463
0.011444	0.011445	0.011437	0.011471	0.011608	0.011573	0.011572
0.011571	0.011497	0.011319	0.011312	0.011433	0.011602	0.011603
0.011396	0.011467	0.011700	0.011668	0.011598	0.011632	0.011564
0.011496	0.011564	0.011563	0.011463	0.012034	0.011631	0.011698
0.011732	0.011772	0.011435	0.011511	0.011396	0.011597	0.011564
0.011598	0.011631	0.011631	0.011631	0.011768	0.011765	0.011372
0.011297	0.011268	0.011401	0.011366	0.011470	0.011284	0.011638
0.011437	0.011543	0.011298	0.011435	0.011430	0.011505	0.011396
0.011362	0.011362	0.011429	0.011396	0.011597	0.011564	0.011497
0.011597	0.011665	0.011396	0.011396	0.011430	0.011429	0.011432
0.011500	0.011429	0.011429	0.011465	0.011597	0.011665	0.011665
0.011531	0.011635	0.011603	0.011772	0.011802	0.011463	0.011631
0.011564	0.011564	0.011398	0.011582	0.011477	0.011537	0.011411
0.011577	0.011698	0.011526	0.011468	0.011362	0.011564	0.011429
0.011530	0.011463	0.011362	0.011464	0.011564	0.011567	0.011463
0.011362	0.011296	0.011429	0.011362	0.011295	0.011400	0.011564
0.011463	0.011396	0.011429	0.011463	0.011430	0.011362	0.011564
0.011429	0.011463	0.011463	0.011396	0.011463	0.011698	0.011362
0.011396	0.011799	0.011396	0.011429	0.011396	0.011765	0.011317
0.011847	0.011358	0.011411	0.011583	0.011671	0.011598	0.011311
0.011375	0.011564	0.011664	0.011773	0.011632	0.011771	0.011901
0.011800	0.011838	0.011799	0.012051	0.011765	0.012640	0.011801
0.012540	0.012308	0.012442	0.012714	0.011765	0.011463	0.011766
0.012204	0.012140	0.011732	0.011631	0.011699	0.011699	0.011669
0.011564	0.011569	0.011631	0.011598	0.011968	0.012034	0.012036
0.011935	0.012035	0.012139	0.011906	0.012205	0.012211	0.012272
0.012273	0.012306	0.012255	0.012273	0.012274	0.011496	0.011799
0.011867	0.012371	0.011874	0.012102	0.012575	0.011637	0.011531
0.011496	0.011496	0.011968	0.011965	0.012116	0.011832	0.011498
S084 0.011698	0.011698	0.012034	0.011799	0.011799	0.011866	
0.011705	0.011637	0.011866	0.011295	0.011362	0.011329	0.011295
0.011329	0.011497	0.011295	0.011329	0.011396	0.011362	0.011329
0.011295	0.011329	0.011429	0.011295	0.011329	0.011329	0.011671
0.011564	0.011329	0.011228	0.011463	0.011396	0.011362	0.011396
0.011362	0.011463	0.011362	0.011362	0.011396	0.011362	0.011530
0.011295	0.011362	0.011396	0.011329	0.011429	0.011463	0.011396
0.011362	0.011530	0.011430	0.011698	0.011362	0.011631	0.011362
0.011329	0.011430	0.011497	0.011631	0.011732	0.011429	0.011530
0.011396	0.011463	0.011362	0.011597	0.011833	0.011362	0.011429
0.011329	0.011429	0.011396	0.011463	0.011396	0.011396	0.011396
0.011295	0.011329	0.011295	0.011396	0.011362	0.011397	0.011463

0.000000	0.011362	0.011429	0.011329	0.011463	0.011295	0.011362
0.011530	0.011463	0.011698	0.011530	0.011362	0.011597	0.011362
0.011542	0.011770	0.011588	0.011295	0.011284	0.011355	0.011493
0.012121	0.011518	0.011266	0.011597	0.011597	0.011396	0.011497
0.011429	0.011396	0.011463	0.011362	0.011463	0.011396	0.011396
0.011362	0.011463	0.011664	0.011530	0.011295	0.011396	0.011329
0.011295	0.011295	0.011362	0.011329	0.011530	0.011497	0.011430
0.011530	0.011597	0.011329	0.011329	0.011362	0.011362	0.011364
0.011432	0.011362	0.011362	0.011240	0.011313	0.011271	0.011363
0.011365	0.011301	0.011318	0.011336	0.011264	0.011300	0.011330
0.011600	0.011370	0.011405	0.011296	0.011302	0.011430	0.011397
0.011464	0.011397	0.011396	0.011496	0.011429	0.011799	0.011598
0.011631	0.011497	0.011362	0.011463	0.011262	0.011497	0.011295
0.011362	0.011362	0.011329	0.011295	0.011329	0.011295	0.011396
0.011362	0.011463	0.011396	0.011295	0.011397	0.011497	0.011500
0.011497	0.011396	0.011295	0.011229	0.011362	0.011295	0.011228
0.011298	0.011497	0.011295	0.011497	0.011262	0.011228	0.011329
0.011430	0.011329	0.011362	0.011329	0.011362	0.011228	0.011362
0.011396	0.011329	0.011396	0.011329	0.011362	0.011396	0.011631
0.011732	0.011664	0.011732	0.011498	0.011732	0.011362	0.011329
0.011364	0.011667	0.011472	0.011266	0.011739	0.011231	0.011375
0.011739	0.011638	0.011530	0.011732	0.011436	0.011597	0.011564
0.011497	0.011664	0.011522	0.011349	0.011354	0.011396	0.011466
0.011564	0.011465	0.011601	0.011669	0.011597	0.011665	0.011772
0.011500	0.011671	0.011602	0.011534	0.011800	0.011262	0.011465
0.011429	0.011362	0.011262	0.011262	0.011329	0.011329	0.011396
0.011329	0.011329	0.011295	0.011396	0.011329	0.011329	0.011396
0.011329	0.011765	0.011664	0.011698	0.011228	0.011329	0.011362
0.011228	0.011329	0.011396	0.011429	0.011295	0.011295	0.011295
0.011832	0.011463	0.011463	0.011262	0.011362	0.011530	0.011295
0.011295	0.011362	0.011329	0.011497	0.011295	0.011429	0.011564
0.011497	0.011329	0.011329	0.011262	0.011262	0.011430	0.011362
0.011262	0.011362	0.011228	0.011262	0.011396	0.011530	0.011295
0.011262	0.011396	0.011329	0.011228	0.011564	0.011228	0.011396
0.011471	0.011295	0.011597	0.011430	0.011463	0.011262	0.011429
0.011295	0.011631	0.011228	0.011497	0.011295	0.011295	0.011228
0.011463	0.011665	0.011362	0.011429	0.011262	0.011664	0.011295
0.011295	0.011295	0.011329	0.011429	0.011329	0.011429	0.011497
0.011497	0.011463	0.011362	0.011463	0.011429	0.011295	0.011262
0.011295	0.011295	0.011362	0.011295	0.011262	0.011429	0.011295
0.011329	0.011329	0.011463	0.011497	0.011396	0.011329	0.011329
0.011396	0.011329	0.011429	0.011295	0.011429	0.011530	0.011832
0.011329	0.011732	0.011336	0.017861	0.011322	0.011549	0.011688
0.011648	0.011693	0.011427	0.011350	0.011774	0.011716	0.011425
0.011630	0.011610	0.011216	0.011353	0.011333	0.011455	0.011361
0.011623	0.011470	0.011505	0.011193	0.011329	0.011497	0.011500
0.011369	0.011463	0.011463	0.011430	0.011396	0.011396	0.011363
0.011396	0.011329	0.011362	0.011396	0.011228	0.011295	0.011329
0.011329	0.011396	0.011295	0.011396	0.011430	0.011463	0.011362
0.011396	0.011362	0.011698	0.011698	0.011362	0.011464	0.011497
0.011262	0.011262	0.011295	0.011396	0.011262	0.011497	0.011497
0.011530	0.011530	0.011329	0.011564	0.011732	0.011565	0.011396
0.011396	0.011463	0.011497	0.011329	0.011396	0.011362	0.011396
0.011463	0.011463	0.011497	0.011463	0.011363	0.011564	0.011530
0.011399	0.011295	0.011396	0.011497	0.011396	0.011530	0.011564
0.011732	0.011436	0.011597	0.011564	0.011497	0.011664	0.011295
0.011464	0.011363	0.011329	0.011329	0.011396	0.011295	0.011396
0.011430	0.011463	0.011362	0.011396	0.011362	0.011698	0.011698
0.011362	0.011464	0.011530	0.011463	0.012103	0.011525	0.011447
0.011517	0.011476	0.011587	0.011476	0.011585	0.011508	0.011474

0.012056	0.011372	0.011505	0.011441	0.011714	0.011570	0.011673
0.012080	0.011550	0.011635	0.011698	0.011564	0.011564	0.011601
0.011262	0.011429	0.011564	0.011530	0.011533	0.011530	0.011498
0.011362	0.011295	0.011329	0.011362	0.011262	0.011329	0.011228
0.011362	0.011396	0.011295	0.011262	0.011362	0.011295	0.011329
0.011329	0.011262	0.011329	0.011362	0.011262	0.011463	0.011497
0.011396	0.011463	0.011396	0.011396	0.011329	0.011732	0.011362
0.011408	0.011329	0.011564	0.011569	0.011430	0.011429	0.011429
0.011429	0.011362	0.011329	0.011362	0.011463	0.011463	0.011262
0.011396	0.011530	0.011698	0.011429	0.011631	0.011631	0.011496
0.011329	0.011396	0.011429	0.011833	0.011396	0.011430	0.000000
0.011429	0.011362	0.011362	0.011665	0.011597	0.011631	0.011362
0.011463	0.011530	0.011396	0.011463	0.011429	0.011295	0.011631
0.011396	0.011362	0.011362	0.011530	0.011362	0.011463	0.011698
0.011396	0.011773	0.011765	0.011548	0.011342	0.011468	0.011318
0.011335	0.011761	0.011306	0.011507	0.011353	0.011467	0.011485
0.011570	0.011258	0.011450	0.011324	0.011330	0.011529	0.011466
0.011475	0.011375	0.011448	0.011377	0.011397	0.011530	0.011598
0.011597	0.011464	0.011567	0.011569	0.011704	0.011732	0.011396
0.011631	0.011497	0.011362	0.011463	0.011262	0.011497	0.011295
0.011362	0.011362	0.011329	0.011295	0.011329	0.011295	0.011396
0.011375	0.011375	0.011368	0.011403	0.011539	0.011504	0.011504
0.011503	0.011430	0.011245	0.011240	0.011365	0.011535	0.011535
0.011329	0.011399	0.011633	0.011601	0.011531	0.011565	0.011496
0.011429	0.011497	0.011496	0.011396	0.011967	0.011564	0.011631
0.011665	0.011737	0.011333	0.011337	0.011329	0.011530	0.011497
0.011531	0.011564	0.011564	0.011564	0.011729	0.011698	0.011338
0.011229	0.011233	0.011333	0.011298	0.011402	0.011247	0.011570
0.011369	0.011499	0.011298	0.011397	0.011363	0.011437	0.011329
0.011295	0.011295	0.011362	0.011329	0.011530	0.011497	0.011430
0.011530	0.011597	0.011329	0.011329	0.011362	0.011362	0.011364
0.011432	0.011362	0.011362	0.011397	0.011530	0.011598	0.011597
0.011464	0.011567	0.011569	0.011704	0.011732	0.011396	0.011564
0.011497	0.011497	0.011331	0.011513	0.011442	0.011469	0.011341
0.011508	0.011744	0.011526	0.011400	0.011295	0.011497	0.011362
0.011463	0.011396	0.011295	0.011397	0.011497	0.011500	0.011396
0.011295	0.011229	0.011362	0.011295	0.011228	0.011298	0.011497
0.011396	0.011329	0.011362	0.011396	0.011228	0.011295	0.011497
0.011362	0.011396	0.011396	0.011329	0.011396	0.011631	0.011295
0.011329	0.011732	0.011329	0.011362	0.011329	0.011698	0.011275
0.011791	0.011273	0.011361	0.011503	0.011589	0.011527	0.011227
0.011323	0.011496	0.011597	0.011705	0.011565	0.011703	0.011834
0.011733	0.011770	0.011732	0.011983	0.011698	0.012573	0.011734
0.012473	0.012241	0.012375	0.012646	0.011698	0.011396	0.011699
0.012137	0.012072	0.011664	0.011564	0.011632	0.011632	0.011601
0.011496	0.011501	0.011564	0.011531	0.011901	0.011967	0.011968
0.011868	0.011968	0.012072	0.011838	0.012138	0.012144	0.012205
0.012206	0.012239	0.012186	0.012206	0.012207	0.011429	0.011732
0.011800	0.012303	0.011806	0.012034	0.012508	0.011570	0.011464
0.011429	0.011429	0.011900	0.011883	0.012045	0.011765	0.011431
S085	0.011664	0.011664	0.012000	0.011765	0.011765	0.011832
0.011637	0.011569	0.011832	0.011262	0.011329	0.011296	0.011262
0.011295	0.011463	0.011262	0.011295	0.011362	0.011329	0.011295
0.011262	0.011295	0.011396	0.011262	0.011295	0.011295	0.011603
0.011530	0.011295	0.011194	0.011429	0.011362	0.011329	0.011362
0.011329	0.011429	0.011329	0.011329	0.011362	0.011329	0.011496
0.011262	0.011329	0.011362	0.011295	0.011396	0.011429	0.011362
0.011329	0.011497	0.011396	0.011664	0.011329	0.011597	0.011329
0.011295	0.011396	0.011463	0.011597	0.011698	0.011396	0.011496
0.011362	0.011429	0.011329	0.011563	0.011799	0.011329	0.011396

0.011295	0.011396	0.011362	0.011429	0.011362	0.011362	0.011362
0.011262	0.011295	0.011262	0.011362	0.011329	0.011363	0.011429
0.011362	0.000000	0.011396	0.011295	0.011429	0.011262	0.011329
0.011496	0.011429	0.011664	0.011496	0.011329	0.011564	0.011329
0.011431	0.011658	0.011513	0.011177	0.011247	0.011355	0.011375
0.012085	0.011467	0.011225	0.011564	0.011564	0.011362	0.011463
0.011396	0.011362	0.011429	0.011329	0.011429	0.011362	0.011362
0.011329	0.011429	0.011631	0.011496	0.011396	0.011362	0.011295
0.011262	0.011262	0.011329	0.011295	0.011497	0.011463	0.011396
0.011497	0.011564	0.011295	0.011295	0.011329	0.011329	0.011330
0.011398	0.011329	0.011329	0.011168	0.011241	0.011200	0.011329
0.011331	0.011266	0.011318	0.011302	0.011196	0.011231	0.011263
0.011528	0.011301	0.011336	0.011262	0.011268	0.011397	0.011363
0.011430	0.011363	0.011363	0.011396	0.011396	0.011765	0.011564
0.011597	0.011463	0.011329	0.011430	0.011228	0.011463	0.011262
0.011329	0.011329	0.011295	0.011262	0.011295	0.011262	0.011362
0.011329	0.011429	0.011362	0.011262	0.011363	0.011463	0.011466
0.011463	0.011362	0.011262	0.011195	0.011329	0.011262	0.011195
0.011298	0.011463	0.011262	0.011463	0.011228	0.011329	0.011295
0.011396	0.011295	0.011329	0.011295	0.011329	0.011195	0.011329
0.011362	0.011295	0.011362	0.011295	0.011329	0.011362	0.011597
0.011698	0.011631	0.011698	0.011465	0.011698	0.011329	0.011295
0.011330	0.011586	0.011380	0.011232	0.011705	0.011197	0.011336
0.011671	0.011604	0.011496	0.011698	0.011402	0.011564	0.011530
0.011463	0.011631	0.011451	0.011307	0.011354	0.011362	0.011432
0.011530	0.011431	0.011567	0.011635	0.011564	0.011631	0.011738
0.011466	0.011637	0.011534	0.011466	0.011766	0.011228	0.011431
0.011396	0.011329	0.011228	0.011228	0.011295	0.011295	0.011362
0.011295	0.011362	0.011262	0.011362	0.011295	0.011295	0.011362
0.011295	0.011731	0.011631	0.011664	0.011194	0.011295	0.011329
0.011329	0.011295	0.011362	0.011396	0.011262	0.011262	0.011262
0.011799	0.011429	0.011429	0.011228	0.011329	0.011497	0.011262
0.011262	0.011329	0.011295	0.011463	0.011262	0.011396	0.011530
0.011463	0.011295	0.011295	0.011228	0.011228	0.011396	0.011329
0.011228	0.011329	0.011194	0.011228	0.011362	0.011497	0.011262
0.011228	0.011362	0.011295	0.011194	0.011530	0.011195	0.011362
0.011437	0.011262	0.011564	0.011396	0.011429	0.011295	0.011396
0.011262	0.011597	0.011194	0.011463	0.011262	0.011262	0.011194
0.011429	0.011631	0.011329	0.011396	0.011228	0.011631	0.011262
0.011262	0.011262	0.011295	0.011396	0.011295	0.011396	0.011463
0.011463	0.011429	0.011329	0.011429	0.011396	0.011262	0.011228
0.011262	0.011262	0.011329	0.011262	0.011228	0.011396	0.011262
0.011295	0.011295	0.011429	0.011463	0.011362	0.011295	0.011295
0.011362	0.011295	0.011396	0.011262	0.011396	0.011429	0.011799
0.011295	0.011698	0.011302	0.017823	0.011257	0.011305	0.011653
0.011614	0.011658	0.011322	0.011292	0.011719	0.011682	0.011303
0.011534	0.011426	0.011138	0.011270	0.011294	0.011307	0.011189
0.011482	0.011470	0.011468	0.011391	0.011429	0.011463	0.011466
0.011335	0.011429	0.011429	0.011396	0.011362	0.011362	0.011329
0.011362	0.011295	0.011329	0.011362	0.011329	0.011262	0.011295
0.011295	0.011362	0.011262	0.011362	0.011396	0.011429	0.011329
0.011362	0.011329	0.011665	0.011665	0.011396	0.011430	0.011430
0.011228	0.011228	0.011262	0.011362	0.011228	0.011463	0.011463
0.011497	0.011496	0.011429	0.011530	0.011698	0.011532	0.011362
0.011362	0.011429	0.011463	0.011295	0.011362	0.011329	0.011362
0.011430	0.011430	0.011463	0.011430	0.011330	0.011530	0.011497
0.011365	0.011262	0.011362	0.011463	0.011362	0.011497	0.011530
0.011698	0.011402	0.011564	0.011530	0.011463	0.011631	0.011262
0.011431	0.011329	0.011295	0.011295	0.011362	0.011262	0.011362
0.011396	0.011429	0.011329	0.011362	0.011329	0.011665	0.011665

0.011396	0.011430	0.011496	0.011429	0.012069	0.011453	0.011412
0.011447	0.011407	0.011517	0.011441	0.011515	0.011474	0.011405
0.011987	0.011302	0.011437	0.011371	0.011645	0.011502	0.011639
0.012046	0.011475	0.011601	0.011664	0.011530	0.011530	0.011567
0.011228	0.011396	0.011530	0.011496	0.011499	0.011496	0.011464
0.011329	0.011262	0.011295	0.011329	0.011228	0.011295	0.011194
0.011329	0.011362	0.011262	0.011228	0.011329	0.011262	0.011295
0.011295	0.011228	0.011295	0.011329	0.011228	0.011429	0.011463
0.011362	0.011429	0.011362	0.011362	0.011295	0.011698	0.011329
0.011373	0.011295	0.011530	0.011535	0.011396	0.011396	0.011396
0.011396	0.011329	0.011295	0.011329	0.011429	0.011429	0.011362
0.011362	0.011496	0.011664	0.011396	0.011597	0.011597	0.011463
0.011295	0.011362	0.011396	0.011799	0.011362	0.011396	0.011362
0.011396	0.011329	0.011329	0.011631	0.011564	0.011597	0.011329
0.011429	0.011496	0.011362	0.011429	0.011396	0.011262	0.011597
0.011362	0.011329	0.011329	0.011496	0.011329	0.011429	0.011664
0.011362	0.011705	0.011731	0.011467	0.011173	0.011434	0.011318
0.011115	0.011683	0.011271	0.011417	0.011255	0.011366	0.011379
0.011468	0.011192	0.011338	0.011192	0.011222	0.011395	0.011385
0.011399	0.011296	0.011412	0.011342	0.011364	0.011497	0.011564
0.011564	0.011430	0.011533	0.011535	0.011670	0.011696	0.011362
0.011597	0.011463	0.011329	0.011430	0.011228	0.011463	0.011262
0.011329	0.011329	0.011295	0.011262	0.011295	0.011262	0.011362
0.011305	0.011305	0.011334	0.011334	0.011471	0.011470	0.011436
0.011435	0.011396	0.011134	0.011132	0.011331	0.011501	0.011501
0.011296	0.011365	0.011599	0.011567	0.011497	0.011531	0.011463
0.011396	0.011463	0.011463	0.011362	0.011933	0.011530	0.011597
0.011631	0.011701	0.011333	0.011407	0.011295	0.011497	0.011463
0.011497	0.011530	0.011530	0.011530	0.011650	0.011664	0.011303
0.011195	0.011199	0.011298	0.011264	0.011368	0.011172	0.011536
0.011335	0.011455	0.011203	0.011285	0.011329	0.011368	0.011295
0.011262	0.011262	0.011329	0.011295	0.011497	0.011463	0.011396
0.011497	0.011564	0.011295	0.011295	0.011329	0.011329	0.011330
0.011398	0.011329	0.011329	0.011364	0.011497	0.011564	0.011564
0.011430	0.011533	0.011535	0.011670	0.011696	0.011362	0.011530
0.011463	0.011463	0.011297	0.011478	0.011408	0.011435	0.011305
0.011473	0.011698	0.011399	0.011366	0.011262	0.011463	0.011329
0.011429	0.011362	0.011262	0.011363	0.011463	0.011466	0.011362
0.011262	0.011195	0.011329	0.011262	0.011195	0.011298	0.011463
0.011362	0.011295	0.011329	0.011362	0.011329	0.011262	0.011463
0.011329	0.011362	0.011362	0.011295	0.011362	0.011597	0.011262
0.011295	0.011698	0.011295	0.011329	0.011295	0.011664	0.011232
0.011622	0.011188	0.011261	0.011463	0.011549	0.011491	0.011186
0.011218	0.011463	0.011564	0.011671	0.011531	0.011669	0.011800
0.011699	0.011737	0.011698	0.011949	0.011664	0.012539	0.011700
0.012439	0.012207	0.012341	0.012612	0.011664	0.011362	0.011665
0.012103	0.012038	0.011631	0.011530	0.011598	0.011598	0.011567
0.011463	0.011467	0.011530	0.011497	0.011867	0.011933	0.011935
0.011834	0.011934	0.012038	0.011804	0.012104	0.012110	0.012171
0.012171	0.012205	0.012152	0.012172	0.012173	0.011396	0.011699
0.011766	0.012270	0.011772	0.012001	0.012474	0.011536	0.011430
0.011396	0.011396	0.011867	0.011883	0.012010	0.011731	0.011397
S086	0.011732	0.011732	0.012068	0.011832	0.011832	0.011900
0.011739	0.011671	0.011899	0.011329	0.011396	0.011363	0.011329
0.011362	0.011530	0.011329	0.011362	0.011429	0.011396	0.011362
0.011329	0.011362	0.011463	0.011329	0.011295	0.011362	0.011705
0.011597	0.011362	0.011262	0.011496	0.011362	0.011396	0.011362
0.011396	0.011429	0.011396	0.011396	0.011429	0.011396	0.011496
0.011329	0.011396	0.011429	0.011362	0.011463	0.011496	0.011429
0.011396	0.011564	0.011463	0.011732	0.011396	0.011597	0.011396

0.011362	0.011463	0.011530	0.011597	0.011765	0.011463	0.011564
0.011362	0.011429	0.011396	0.011563	0.011866	0.011396	0.011463
0.011362	0.011463	0.011429	0.011496	0.011429	0.011429	0.011429
0.011329	0.011362	0.011329	0.011429	0.011396	0.011430	0.011496
0.011429	0.011396	0.000000	0.011362	0.011496	0.011262	0.011396
0.011564	0.011496	0.011731	0.011564	0.011396	0.011564	0.011396
0.011579	0.011807	0.011626	0.011334	0.011321	0.011428	0.011533
0.012158	0.011569	0.011348	0.011631	0.011631	0.011429	0.011530
0.011463	0.011429	0.011496	0.011396	0.011497	0.011362	0.011429
0.011396	0.011496	0.011698	0.011564	0.011463	0.011430	0.011295
0.011329	0.011329	0.011262	0.011362	0.011564	0.011530	0.011463
0.011564	0.011631	0.011362	0.011362	0.011396	0.011396	0.011398
0.011466	0.011396	0.011396	0.011240	0.011349	0.011306	0.011396
0.011399	0.011335	0.011392	0.011371	0.011298	0.011334	0.011364
0.011636	0.011405	0.011301	0.011329	0.011337	0.011464	0.011430
0.011497	0.011431	0.011430	0.011530	0.011463	0.011765	0.011631
0.011664	0.011530	0.011396	0.011497	0.011295	0.011530	0.011329
0.011396	0.011396	0.011362	0.011329	0.011362	0.011329	0.011429
0.011329	0.011429	0.011430	0.011329	0.011431	0.011530	0.011533
0.011396	0.011430	0.011329	0.011263	0.011396	0.011329	0.011262
0.011366	0.011396	0.011262	0.011396	0.011295	0.011396	0.011362
0.011396	0.011362	0.011329	0.011362	0.011396	0.011262	0.011329
0.011362	0.011362	0.011430	0.011362	0.011396	0.011430	0.011665
0.011765	0.011698	0.011765	0.011532	0.011766	0.011396	0.011362
0.011398	0.011626	0.011517	0.011300	0.011739	0.011265	0.011414
0.011773	0.011672	0.011564	0.011765	0.011470	0.011631	0.011530
0.011530	0.011698	0.011558	0.011307	0.011354	0.011430	0.011500
0.011597	0.011499	0.011635	0.011635	0.011631	0.011698	0.011805
0.011534	0.011637	0.011636	0.011567	0.011834	0.011295	0.011498
0.011396	0.011396	0.011295	0.011295	0.011295	0.011362	0.011429
0.011295	0.011362	0.011262	0.011429	0.011295	0.011362	0.011429
0.011362	0.011799	0.011698	0.011732	0.011262	0.011362	0.011396
0.011396	0.011362	0.011429	0.011463	0.011329	0.011329	0.011329
0.011866	0.011496	0.011496	0.011295	0.011396	0.011497	0.011329
0.011329	0.011396	0.011362	0.011463	0.011329	0.011463	0.011530
0.011530	0.011362	0.011362	0.011295	0.011295	0.011463	0.011396
0.011295	0.011396	0.011262	0.011295	0.011429	0.011564	0.011329
0.011295	0.011429	0.011363	0.011262	0.011597	0.011262	0.011429
0.011437	0.011329	0.011631	0.011463	0.011496	0.011362	0.011463
0.011329	0.011597	0.011262	0.011530	0.011329	0.011329	0.011262
0.011429	0.011698	0.011396	0.011463	0.011295	0.011698	0.011329
0.011329	0.011329	0.011362	0.011396	0.011362	0.011463	0.011530
0.011530	0.011496	0.011396	0.011497	0.011396	0.011329	0.011295
0.011329	0.011329	0.011396	0.011329	0.011228	0.011463	0.011329
0.011362	0.011362	0.011496	0.011530	0.011429	0.011295	0.011362
0.011429	0.011362	0.011463	0.011329	0.011463	0.011564	0.011866
0.011362	0.011765	0.011371	0.017861	0.011257	0.011488	0.011723
0.011683	0.011729	0.011322	0.011292	0.011774	0.011751	0.011425
0.011629	0.011610	0.011294	0.011353	0.011333	0.011356	0.011361
0.011623	0.011544	0.011505	0.011325	0.011497	0.011530	0.011534
0.011266	0.011497	0.011497	0.011463	0.011429	0.011430	0.011396
0.011429	0.011362	0.011396	0.011430	0.011396	0.011329	0.011362
0.011362	0.011430	0.011329	0.011430	0.011463	0.011497	0.011396
0.011429	0.011396	0.011732	0.011732	0.011463	0.011497	0.011530
0.011295	0.011295	0.011329	0.011362	0.011295	0.011530	0.011530
0.011564	0.011564	0.011497	0.011597	0.011765	0.011599	0.011362
0.011430	0.011497	0.011463	0.011362	0.011430	0.011396	0.011429
0.011497	0.011497	0.011530	0.011430	0.011330	0.011597	0.011497
0.011433	0.011329	0.011429	0.011530	0.011430	0.011564	0.011597
0.011765	0.011470	0.011631	0.011530	0.011530	0.011698	0.011329

0.011498	0.011396	0.011362	0.011362	0.011430	0.011329	0.011430
0.011463	0.011497	0.011396	0.011429	0.011396	0.011732	0.011732
0.011463	0.011497	0.011564	0.011496	0.012137	0.011560	0.011482
0.011552	0.011511	0.011622	0.011510	0.011620	0.011543	0.011508
0.012090	0.011406	0.011540	0.011475	0.011749	0.011604	0.011639
0.012114	0.011588	0.011669	0.011732	0.011597	0.011597	0.011635
0.011295	0.011396	0.011597	0.011564	0.011567	0.011564	0.011532
0.011396	0.011262	0.011295	0.011396	0.011228	0.011362	0.011262
0.011396	0.011429	0.011329	0.011295	0.011396	0.011329	0.011362
0.011362	0.011295	0.011362	0.011396	0.011295	0.011429	0.011530
0.011429	0.011496	0.011429	0.011429	0.011362	0.011765	0.011396
0.011443	0.011362	0.011598	0.011603	0.011396	0.011463	0.011463
0.011463	0.011329	0.011362	0.011329	0.011496	0.011497	0.011429
0.011429	0.011564	0.011731	0.011463	0.011664	0.011664	0.011530
0.011362	0.011429	0.011463	0.011866	0.011430	0.011396	0.011429
0.011463	0.011396	0.011396	0.011698	0.011564	0.011664	0.011396
0.011497	0.011564	0.011429	0.011497	0.011463	0.011329	0.011664
0.011429	0.011396	0.011396	0.011564	0.011396	0.011496	0.011732
0.011429	0.011807	0.011799	0.011629	0.011229	0.011502	0.011370
0.011280	0.011839	0.011341	0.011596	0.011402	0.011569	0.011538
0.011570	0.011192	0.011450	0.011126	0.011384	0.011395	0.011466
0.011475	0.011336	0.011483	0.011377	0.011431	0.011564	0.011631
0.011631	0.011497	0.011601	0.011603	0.011738	0.011767	0.011429
0.011664	0.011530	0.011396	0.011497	0.011295	0.011530	0.011329
0.011396	0.011396	0.011362	0.011329	0.011362	0.011329	0.011429
0.011409	0.011410	0.011403	0.011437	0.011573	0.011470	0.011538
0.011537	0.011464	0.011282	0.011276	0.011399	0.011568	0.011569
0.011363	0.011433	0.011666	0.011635	0.011565	0.011599	0.011530
0.011463	0.011530	0.011530	0.011429	0.012001	0.011597	0.011665
0.011698	0.011737	0.011401	0.011476	0.011362	0.011564	0.011530
0.011565	0.011597	0.011597	0.011597	0.011729	0.011732	0.011372
0.011263	0.011268	0.011367	0.011332	0.011436	0.011284	0.011604
0.011403	0.011367	0.011108	0.011435	0.011396	0.011471	0.011295
0.011329	0.011329	0.011262	0.011362	0.011564	0.011530	0.011463
0.011564	0.011631	0.011362	0.011362	0.011396	0.011396	0.011398
0.011466	0.011396	0.011396	0.011431	0.011564	0.011631	0.011631
0.011497	0.011601	0.011603	0.011738	0.011767	0.011429	0.011597
0.011530	0.011530	0.011364	0.011547	0.011477	0.011503	0.011375
0.011473	0.011698	0.011526	0.011434	0.011262	0.011396	0.011329
0.011429	0.011430	0.011329	0.011431	0.011530	0.011533	0.011430
0.011329	0.011263	0.011396	0.011329	0.011262	0.011366	0.011396
0.011429	0.011362	0.011396	0.011430	0.011396	0.011329	0.011530
0.011396	0.011429	0.011429	0.011362	0.011429	0.011664	0.011329
0.011362	0.011765	0.011362	0.011329	0.011362	0.011732	0.011360
0.011678	0.011358	0.011411	0.011584	0.011671	0.011563	0.011311
0.011323	0.011530	0.011631	0.011739	0.011599	0.011737	0.011867
0.011767	0.011804	0.011765	0.012017	0.011731	0.012606	0.011768
0.012507	0.012274	0.012408	0.012680	0.011731	0.011430	0.011732
0.012171	0.012106	0.011698	0.011597	0.011665	0.011665	0.011635
0.011530	0.011535	0.011597	0.011565	0.011935	0.012001	0.012002
0.011901	0.012002	0.012106	0.011872	0.012171	0.012178	0.012238
0.012239	0.012273	0.012221	0.012239	0.012241	0.011463	0.011766
0.011834	0.012337	0.011840	0.012068	0.012541	0.011604	0.011497
0.011463	0.011463	0.011934	0.011965	0.012081	0.011799	0.011465
S087	0.011631	0.011631	0.011967	0.011731	0.011731	0.011799
0.011603	0.011535	0.011799	0.011228	0.011295	0.011262	0.011228
0.011262	0.011429	0.011228	0.011262	0.011329	0.011295	0.011262
0.011228	0.011262	0.011362	0.011228	0.011262	0.011262	0.011569
0.011496	0.011262	0.011161	0.011396	0.011329	0.011295	0.011329
0.011295	0.011396	0.011295	0.011295	0.011329	0.011295	0.011463

0.011228	0.011295	0.011329	0.011262	0.011362	0.011396	0.011329
0.011295	0.011463	0.011362	0.011631	0.011295	0.011564	0.011295
0.011262	0.011362	0.011429	0.011564	0.011664	0.011362	0.011463
0.011329	0.011396	0.011295	0.011530	0.011765	0.011295	0.011362
0.011262	0.011362	0.011329	0.011396	0.011194	0.011329	0.011329
0.011228	0.011262	0.011228	0.011329	0.011295	0.011329	0.011396
0.011329	0.011295	0.011362	0.000000	0.011396	0.011228	0.011295
0.011463	0.011396	0.011631	0.011463	0.011295	0.011530	0.011295
0.011357	0.011583	0.011400	0.011099	0.011172	0.011281	0.011296
0.012011	0.011365	0.011102	0.011530	0.011530	0.011329	0.011429
0.011362	0.011329	0.011396	0.011295	0.011396	0.011329	0.011329
0.011295	0.011262	0.011597	0.011463	0.011362	0.011329	0.011262
0.011228	0.011228	0.011295	0.011262	0.011463	0.011429	0.011362
0.011463	0.011530	0.011262	0.011262	0.011295	0.011295	0.011297
0.011364	0.011295	0.011295	0.011132	0.011241	0.011200	0.011296
0.011297	0.011232	0.011245	0.011267	0.011196	0.011231	0.011263
0.011528	0.011233	0.011336	0.011228	0.011233	0.011363	0.011329
0.011396	0.011330	0.011329	0.011429	0.011362	0.011731	0.011530
0.011563	0.011429	0.011295	0.011396	0.011195	0.011429	0.011228
0.011295	0.011295	0.011262	0.011228	0.011262	0.011228	0.011329
0.011295	0.011396	0.011329	0.011228	0.011330	0.011429	0.011432
0.011430	0.011329	0.011228	0.011161	0.011295	0.011228	0.011161
0.011264	0.011430	0.011228	0.011430	0.011195	0.011295	0.011262
0.011362	0.011262	0.011295	0.011262	0.011295	0.011161	0.011295
0.011329	0.011262	0.011329	0.011262	0.011295	0.011329	0.011564
0.011664	0.011597	0.011665	0.011397	0.011665	0.011295	0.011262
0.011297	0.011545	0.011426	0.011163	0.011671	0.011163	0.011257
0.011637	0.011570	0.011463	0.011664	0.011367	0.011530	0.011497
0.011429	0.011597	0.011451	0.011307	0.011354	0.011329	0.011398
0.011497	0.011397	0.011533	0.011601	0.011530	0.011597	0.011704
0.011432	0.011603	0.011534	0.011466	0.011733	0.011194	0.011397
0.011362	0.011295	0.011194	0.011194	0.011262	0.011262	0.011329
0.011262	0.011329	0.011228	0.011329	0.011262	0.011262	0.011329
0.011262	0.011698	0.011597	0.011631	0.011161	0.011262	0.011295
0.011295	0.011262	0.011329	0.011362	0.011228	0.011228	0.011228
0.011765	0.011396	0.011396	0.011194	0.011295	0.011463	0.011228
0.011228	0.011295	0.011262	0.011429	0.011228	0.011362	0.011496
0.011429	0.011262	0.011262	0.011194	0.011194	0.011362	0.011295
0.011194	0.011295	0.011161	0.011195	0.011329	0.011463	0.011228
0.011195	0.011329	0.011262	0.011161	0.011496	0.011161	0.011329
0.011402	0.011228	0.011530	0.011362	0.011396	0.011262	0.011362
0.011228	0.011564	0.011161	0.011429	0.011228	0.011228	0.011161
0.011396	0.011597	0.011295	0.011362	0.011194	0.011597	0.011228
0.011228	0.011228	0.011262	0.011362	0.011262	0.011362	0.011429
0.011429	0.011396	0.011295	0.011396	0.011362	0.011228	0.011194
0.011228	0.011228	0.011295	0.011228	0.011194	0.011362	0.011228
0.011262	0.011262	0.011396	0.011429	0.011329	0.011262	0.011262
0.011262	0.011262	0.011362	0.011228	0.011362	0.011463	0.011765
0.011262	0.011664	0.011233	0.017747	0.011126	0.011305	0.011583
0.011545	0.011588	0.011217	0.011176	0.011609	0.011612	0.011242
0.011392	0.011426	0.000000	0.011144	0.011216	0.011208	0.011146
0.011389	0.011395	0.011394	0.011259	0.011396	0.011429	0.011432
0.011301	0.011396	0.011396	0.011362	0.011329	0.011329	0.011295
0.011329	0.011262	0.011295	0.011329	0.011295	0.011228	0.011262
0.011262	0.011329	0.011228	0.011329	0.011362	0.011396	0.011295
0.011329	0.011295	0.011631	0.011631	0.011362	0.011396	0.011430
0.011195	0.011195	0.011228	0.011329	0.011195	0.011429	0.011429
0.011463	0.011463	0.011396	0.011496	0.011665	0.011498	0.011329
0.011329	0.011396	0.011429	0.011262	0.011329	0.011295	0.011329
0.011396	0.011396	0.011429	0.011396	0.011296	0.011497	0.011463

0.011331	0.011228	0.011329	0.011430	0.011329	0.011463	0.011496
0.011664	0.011367	0.011530	0.011497	0.011429	0.011597	0.011228
0.011397	0.011295	0.011262	0.011262	0.011329	0.011228	0.011329
0.011362	0.011396	0.011295	0.011329	0.011295	0.011631	0.011631
0.011362	0.011396	0.011463	0.011396	0.012035	0.011453	0.011377
0.011447	0.011407	0.011517	0.011406	0.011515	0.011439	0.011405
0.011987	0.011302	0.011437	0.011371	0.011645	0.011502	0.011604
0.012012	0.011475	0.011567	0.011631	0.011496	0.011497	0.011533
0.011194	0.011362	0.011496	0.011463	0.011466	0.011463	0.011397
0.011295	0.011228	0.011262	0.011295	0.011194	0.011262	0.011161
0.011295	0.011329	0.011228	0.011194	0.011295	0.011228	0.011262
0.011262	0.011194	0.011262	0.011295	0.011194	0.011396	0.011429
0.011329	0.011396	0.011329	0.011329	0.011262	0.011664	0.011295
0.011338	0.011262	0.011497	0.011501	0.011362	0.011362	0.011362
0.011362	0.011295	0.011262	0.011295	0.011396	0.011396	0.011329
0.011329	0.011463	0.011631	0.011362	0.011564	0.011564	0.011429
0.011262	0.011329	0.011362	0.011765	0.011329	0.011362	0.011329
0.011362	0.011295	0.011295	0.011597	0.011530	0.011564	0.011295
0.011396	0.011463	0.011329	0.011396	0.011362	0.011228	0.011564
0.011329	0.011295	0.011295	0.011463	0.011295	0.011396	0.011631
0.011329	0.011671	0.011698	0.011385	0.011117	0.011400	0.011163
0.011115	0.011605	0.011201	0.011328	0.011158	0.011264	0.011273
0.011366	0.000000	0.011227	0.000000	0.011114	0.011261	0.011263
0.011286	0.011178	0.011342	0.011272	0.011330	0.011463	0.011530
0.011530	0.011396	0.011500	0.011501	0.011636	0.011661	0.011329
0.011563	0.011429	0.011295	0.011396	0.011195	0.011429	0.011228
0.011295	0.011295	0.011262	0.011228	0.011262	0.011228	0.011329
0.011305	0.011305	0.011300	0.011197	0.011471	0.011436	0.011436
0.011435	0.011228	0.011134	0.011132	0.011297	0.011467	0.011467
0.011262	0.011331	0.011565	0.011533	0.011464	0.011498	0.011429
0.011362	0.011429	0.011429	0.011329	0.011900	0.011496	0.011564
0.011597	0.011665	0.011299	0.011372	0.011262	0.011463	0.011429
0.011329	0.011496	0.011496	0.011496	0.011650	0.011631	0.011268
0.011162	0.011164	0.011264	0.011230	0.011333	0.011172	0.011502
0.011300	0.011455	0.011203	0.011285	0.011296	0.011368	0.011262
0.011228	0.011228	0.011295	0.011262	0.011463	0.011429	0.011362
0.011463	0.011530	0.011262	0.011262	0.011295	0.011295	0.011297
0.011364	0.011295	0.011295	0.011330	0.011463	0.011530	0.011530
0.011396	0.011500	0.011501	0.011636	0.011661	0.011329	0.011496
0.011429	0.011429	0.011263	0.011443	0.011373	0.011265	0.011235
0.011404	0.011607	0.011441	0.011332	0.011228	0.011430	0.011295
0.011396	0.011329	0.011228	0.011330	0.011429	0.011432	0.011329
0.011228	0.011161	0.011295	0.011228	0.011161	0.011264	0.011430
0.011329	0.011262	0.011295	0.011329	0.011295	0.011228	0.011430
0.011295	0.011329	0.011329	0.011262	0.011329	0.011564	0.011228
0.011262	0.011664	0.011262	0.011295	0.011262	0.011631	0.011103
0.011566	0.011103	0.011161	0.011422	0.011426	0.011419	0.000000
0.011113	0.011429	0.011530	0.011637	0.011498	0.011601	0.011766
0.011666	0.011703	0.011664	0.011880	0.011631	0.012505	0.011666
0.012405	0.012139	0.012307	0.012578	0.011631	0.011329	0.011631
0.012070	0.011971	0.011597	0.011497	0.011564	0.011564	0.011534
0.011429	0.011399	0.011496	0.011464	0.011833	0.011900	0.011901
0.011800	0.011901	0.011970	0.011737	0.012070	0.012076	0.012137
0.012138	0.012171	0.012084	0.012138	0.012139	0.011362	0.011665
0.011733	0.012236	0.011738	0.011967	0.012440	0.011502	0.011396
0.011362	0.011362	0.011833	0.011883	0.011975	0.011698	0.011363
S088 0.011765	0.011765	0.012034	0.011799	0.011799	0.011866	
0.011502	0.011434	0.011866	0.011362	0.011429	0.011397	0.011362
0.011329	0.011564	0.011295	0.011396	0.011463	0.011429	0.011396
0.011362	0.011396	0.011429	0.011363	0.011396	0.011396	0.011467

0.011564	0.011396	0.011295	0.011463	0.011463	0.011429	0.011463
0.011429	0.011530	0.011429	0.011429	0.011463	0.011429	0.011597
0.011295	0.011429	0.011463	0.011396	0.011429	0.011530	0.011463
0.011429	0.011597	0.011497	0.011496	0.011429	0.011698	0.011429
0.011396	0.011429	0.011564	0.011698	0.011597	0.011429	0.011530
0.011463	0.011530	0.011429	0.011664	0.011631	0.011429	0.011496
0.011396	0.011496	0.011463	0.011530	0.011463	0.011463	0.011463
0.011362	0.011396	0.011362	0.011396	0.011429	0.011464	0.011530
0.011463	0.011429	0.011496	0.011396	0.000000	0.011362	0.011429
0.011597	0.011530	0.011631	0.011597	0.011429	0.011664	0.011429
0.011616	0.011546	0.011664	0.011373	0.011359	0.011428	0.011493
0.012121	0.011467	0.011390	0.011664	0.011664	0.011396	0.011429
0.011496	0.011463	0.011530	0.011429	0.011530	0.011463	0.011463
0.011429	0.011463	0.011731	0.011597	0.011496	0.011463	0.011396
0.011362	0.011295	0.011429	0.011396	0.011530	0.011497	0.011429
0.011530	0.011597	0.011396	0.011396	0.011430	0.011429	0.011432
0.011500	0.011429	0.011429	0.011312	0.011386	0.011341	0.011430
0.011433	0.011369	0.011429	0.011405	0.011332	0.011368	0.011398
0.011528	0.011439	0.011473	0.011363	0.011371	0.011497	0.011396
0.011464	0.011465	0.011464	0.011563	0.011496	0.011866	0.011597
0.011631	0.011497	0.011429	0.011463	0.011329	0.011496	0.011362
0.011429	0.011429	0.011396	0.011362	0.011396	0.011362	0.011463
0.011429	0.011530	0.011463	0.011362	0.011464	0.011564	0.011567
0.011564	0.011463	0.011362	0.011296	0.011429	0.011362	0.011295
0.011400	0.011564	0.011362	0.011564	0.011329	0.011430	0.011329
0.011497	0.011396	0.011429	0.011396	0.011429	0.011295	0.011429
0.011463	0.011396	0.011463	0.011396	0.011429	0.011463	0.011698
0.011530	0.011463	0.011530	0.011566	0.011597	0.011429	0.011396
0.011364	0.011626	0.011426	0.011335	0.011535	0.011299	0.011454
0.011535	0.011570	0.011597	0.011799	0.011504	0.011664	0.011631
0.011564	0.011732	0.011593	0.011472	0.011479	0.011463	0.011534
0.011631	0.011532	0.011669	0.011737	0.011664	0.011732	0.011839
0.011568	0.011739	0.011602	0.011601	0.011867	0.011329	0.011532
0.011496	0.011429	0.011329	0.011329	0.011396	0.011396	0.011463
0.011396	0.011463	0.011362	0.011396	0.011396	0.011396	0.011463
0.011396	0.011564	0.011463	0.011496	0.011295	0.011396	0.011429
0.011429	0.011329	0.011463	0.011429	0.011362	0.011362	0.011362
0.011631	0.011463	0.011530	0.011329	0.011429	0.011597	0.011362
0.011362	0.011429	0.011396	0.011564	0.011362	0.011496	0.011631
0.011564	0.011396	0.011396	0.011329	0.011329	0.011496	0.011362
0.011329	0.011362	0.011295	0.011329	0.011463	0.011463	0.011362
0.011329	0.011463	0.011396	0.011295	0.011429	0.011295	0.011463
0.011539	0.011362	0.011396	0.011497	0.011463	0.011396	0.011496
0.011362	0.011698	0.011295	0.011362	0.011295	0.011295	0.011295
0.011530	0.011598	0.011429	0.011496	0.011329	0.011463	0.011295
0.011362	0.011362	0.011396	0.011496	0.011396	0.011496	0.011564
0.011564	0.011530	0.011429	0.011530	0.011496	0.011362	0.011329
0.011362	0.011362	0.011429	0.011295	0.011329	0.011496	0.011362
0.011329	0.011396	0.011530	0.011496	0.011463	0.011396	0.011396
0.011463	0.011396	0.011496	0.011362	0.011496	0.011597	0.011698
0.011396	0.011597	0.011405	0.017898	0.011387	0.011488	0.011479
0.011441	0.011482	0.011479	0.011466	0.011774	0.011509	0.011485
0.011582	0.011549	0.011333	0.011437	0.011294	0.011307	0.011404
0.011482	0.011507	0.011431	0.011325	0.011530	0.011564	0.011567
0.011438	0.011530	0.011530	0.011429	0.011463	0.011463	0.011430
0.011463	0.011396	0.011429	0.011463	0.011430	0.011362	0.011396
0.011396	0.011463	0.011362	0.011463	0.011497	0.011530	0.011429
0.011396	0.011362	0.011564	0.011564	0.011497	0.011531	0.011564
0.011329	0.011329	0.011362	0.011463	0.011329	0.011564	0.011564
0.011597	0.011597	0.011530	0.011631	0.011597	0.011633	0.011463

0.011463	0.011530	0.011564	0.011396	0.011463	0.011429	0.011463
0.011531	0.011530	0.011564	0.011530	0.011431	0.011631	0.011597
0.011467	0.011362	0.011463	0.011564	0.011463	0.011530	0.011631
0.011799	0.011504	0.011664	0.011631	0.011564	0.011732	0.011363
0.011532	0.011430	0.011396	0.011396	0.011463	0.011362	0.011463
0.011497	0.011530	0.011429	0.011396	0.011362	0.011564	0.011564
0.011497	0.011531	0.011597	0.011530	0.012103	0.011596	0.011517
0.011587	0.011546	0.011657	0.011545	0.011655	0.011577	0.011543
0.012056	0.011441	0.011574	0.011510	0.011783	0.011638	0.011741
0.012080	0.011512	0.011703	0.011765	0.011631	0.011564	0.011668
0.011329	0.011497	0.011631	0.011597	0.011601	0.011530	0.011498
0.011429	0.011362	0.011396	0.011429	0.011329	0.011396	0.011295
0.011362	0.011396	0.011362	0.011329	0.011429	0.011362	0.011396
0.011396	0.011329	0.011396	0.011429	0.011329	0.011530	0.011564
0.011463	0.011463	0.011463	0.011463	0.011396	0.011597	0.011429
0.011408	0.011396	0.011564	0.011569	0.011429	0.011429	0.011496
0.011496	0.011429	0.011396	0.011429	0.011530	0.011530	0.011463
0.011463	0.011597	0.011429	0.011496	0.011698	0.011698	0.011564
0.011396	0.011463	0.011496	0.011698	0.011463	0.011429	0.011463
0.011496	0.011362	0.011362	0.011665	0.011664	0.011631	0.011429
0.011530	0.011597	0.011463	0.011530	0.011496	0.011362	0.011429
0.011463	0.011429	0.011362	0.011597	0.011429	0.011530	0.011564
0.011463	0.011569	0.011564	0.011588	0.011399	0.011468	0.011370
0.011280	0.011800	0.011376	0.011462	0.011402	0.011416	0.011432
0.011570	0.011258	0.011505	0.011324	0.011384	0.011596	0.011547
0.011550	0.011454	0.011448	0.011448	0.011465	0.011530	0.011597
0.011597	0.011464	0.011567	0.011569	0.011704	0.011732	0.011463
0.011631	0.011497	0.011429	0.011463	0.011329	0.011496	0.011362
0.011429	0.011429	0.011396	0.011362	0.011396	0.011362	0.011463
0.011444	0.011445	0.011437	0.011471	0.011608	0.011573	0.011572
0.011571	0.011497	0.011319	0.011312	0.011433	0.011602	0.011603
0.011396	0.011467	0.011700	0.011668	0.011598	0.011632	0.011564
0.011429	0.011564	0.011563	0.011463	0.011832	0.011631	0.011698
0.011732	0.011523	0.011367	0.011511	0.011396	0.011597	0.011564
0.011598	0.011631	0.011631	0.011631	0.011689	0.011765	0.011407
0.011297	0.011303	0.011401	0.011366	0.011402	0.011284	0.011366
0.011437	0.011411	0.011346	0.011435	0.011430	0.011505	0.011396
0.011362	0.011295	0.011429	0.011396	0.011530	0.011497	0.011429
0.011530	0.011597	0.011396	0.011396	0.011430	0.011429	0.011432
0.011500	0.011429	0.011429	0.011465	0.011530	0.011597	0.011597
0.011464	0.011567	0.011569	0.011704	0.011732	0.011463	0.011631
0.011564	0.011564	0.011398	0.011582	0.011512	0.011537	0.011411
0.011577	0.011744	0.011568	0.011468	0.011362	0.011564	0.011429
0.011530	0.011463	0.011362	0.011464	0.011564	0.011567	0.011463
0.011362	0.011296	0.011429	0.011362	0.011295	0.011400	0.011564
0.011463	0.011396	0.011429	0.011463	0.011430	0.011362	0.011497
0.011429	0.011463	0.011463	0.011396	0.011463	0.011698	0.011295
0.011396	0.011597	0.011396	0.011429	0.011396	0.011496	0.011403
0.011735	0.011400	0.011361	0.011543	0.011630	0.011527	0.011353
0.011323	0.011496	0.011597	0.011705	0.011565	0.011703	0.011834
0.011733	0.011770	0.011731	0.011983	0.011698	0.012573	0.011734
0.012473	0.012241	0.012375	0.012646	0.011698	0.011396	0.011698
0.012137	0.012072	0.011664	0.011564	0.011632	0.011632	0.011567
0.011496	0.011501	0.011564	0.011531	0.011901	0.011967	0.011968
0.011867	0.011968	0.012072	0.011838	0.012138	0.012144	0.012205
0.012205	0.012239	0.012186	0.012205	0.012207	0.011496	0.011732
0.011800	0.012303	0.011806	0.011967	0.012440	0.011502	0.011396
0.011362	0.011362	0.011900	0.011924	0.012045	0.011765	0.011431
S089	0.011597	0.011597	0.011933	0.011698	0.011698	0.011765
0.011603	0.011535	0.011765	0.011195	0.011262	0.011229	0.011194

0.011228	0.011396	0.011194	0.011228	0.011295	0.011262	0.011228
0.011194	0.011228	0.011329	0.011195	0.011161	0.011228	0.011569
0.011463	0.011228	0.011127	0.011362	0.011228	0.011262	0.011228
0.011262	0.011295	0.011262	0.011262	0.011295	0.011262	0.011429
0.011194	0.011262	0.011295	0.011228	0.011329	0.011362	0.011295
0.011262	0.011429	0.011329	0.011597	0.011262	0.011463	0.011262
0.011228	0.011329	0.011396	0.011463	0.011631	0.011329	0.011429
0.011228	0.011295	0.011262	0.011429	0.011732	0.011262	0.011329
0.011161	0.011329	0.011295	0.011362	0.011295	0.011295	0.011295
0.011194	0.011228	0.011194	0.011295	0.011262	0.011296	0.011362
0.011295	0.011262	0.011262	0.011228	0.011362	0.000000	0.011262
0.011429	0.011362	0.011597	0.011429	0.011262	0.011429	0.011262
0.011431	0.011658	0.011475	0.011177	0.011172	0.011281	0.011375
0.012011	0.011416	0.011184	0.011496	0.011496	0.011295	0.011396
0.011329	0.011295	0.011362	0.011262	0.011362	0.011228	0.011295
0.011262	0.011362	0.011564	0.011429	0.011329	0.011295	0.011161
0.011195	0.011195	0.011195	0.011228	0.011429	0.011396	0.011329
0.011429	0.011497	0.011228	0.011228	0.011262	0.011262	0.011263
0.011331	0.011262	0.011262	0.011096	0.011205	0.011165	0.011262
0.011263	0.011198	0.011245	0.011233	0.011162	0.011197	0.011229
0.011492	0.011267	0.011232	0.011195	0.011199	0.011329	0.011296
0.011363	0.011296	0.011296	0.011396	0.011329	0.011631	0.011497
0.011530	0.011396	0.011262	0.011362	0.011161	0.011396	0.011195
0.011262	0.011262	0.011228	0.011195	0.011228	0.011195	0.011295
0.011195	0.011295	0.011295	0.011195	0.011296	0.011396	0.011398
0.011329	0.011295	0.011195	0.011128	0.011262	0.011195	0.011128
0.011230	0.011329	0.011127	0.011329	0.011161	0.011262	0.011228
0.011262	0.011228	0.011195	0.011228	0.011262	0.011127	0.011195
0.011228	0.011228	0.011295	0.011228	0.011262	0.011295	0.011530
0.011631	0.011564	0.011631	0.011397	0.011631	0.011262	0.011228
0.011263	0.011505	0.011335	0.011163	0.011637	0.011129	0.011257
0.011637	0.011536	0.011429	0.011631	0.011333	0.011497	0.011396
0.011396	0.011564	0.011380	0.011225	0.011270	0.011295	0.011365
0.011463	0.011364	0.011500	0.011500	0.011497	0.011564	0.011670
0.011399	0.011501	0.011500	0.011432	0.011699	0.011161	0.011363
0.011262	0.011262	0.011161	0.011161	0.011161	0.011228	0.011295
0.011161	0.011228	0.011127	0.011295	0.011161	0.011228	0.011295
0.011228	0.011664	0.011564	0.011597	0.011127	0.011228	0.011262
0.011262	0.011228	0.011295	0.011329	0.011194	0.011194	0.011194
0.011731	0.011362	0.011362	0.011161	0.011262	0.011362	0.011195
0.011194	0.011262	0.011228	0.011329	0.011194	0.011329	0.011396
0.011396	0.011228	0.011228	0.011161	0.011161	0.011329	0.011262
0.011161	0.011262	0.011127	0.011161	0.011295	0.011430	0.011195
0.011161	0.011295	0.011228	0.011127	0.011463	0.011127	0.011295
0.011266	0.011195	0.011497	0.011329	0.011362	0.011228	0.011329
0.011194	0.011463	0.011127	0.011396	0.011194	0.011194	0.011127
0.011295	0.011564	0.011262	0.011329	0.011161	0.011564	0.011194
0.011195	0.011194	0.011228	0.011262	0.011228	0.011329	0.011396
0.011396	0.011362	0.011262	0.011362	0.011262	0.011194	0.011161
0.011194	0.011194	0.011262	0.011194	0.011094	0.011329	0.011194
0.011228	0.011228	0.011362	0.011396	0.011295	0.011161	0.011228
0.011295	0.011228	0.011329	0.011194	0.011329	0.011429	0.011732
0.011228	0.011631	0.011233	0.017748	0.011126	0.011366	0.011583
0.011545	0.011588	0.011217	0.011176	0.011664	0.011613	0.011242
0.011440	0.011487	0.011138	0.011144	0.011177	0.011307	0.011232
0.011436	0.011395	0.011357	0.011193	0.011362	0.011396	0.011398
0.011198	0.011362	0.011362	0.011329	0.011295	0.011295	0.011262
0.011295	0.011228	0.011262	0.011295	0.011262	0.011195	0.011228
0.011228	0.011295	0.011195	0.011295	0.011329	0.011362	0.011262
0.011295	0.011262	0.011597	0.011597	0.011329	0.011363	0.011396

0.011161	0.011161	0.011195	0.011228	0.011161	0.011396	0.011396
0.011429	0.011429	0.011362	0.011463	0.011631	0.011464	0.011228
0.011295	0.011362	0.011329	0.011228	0.011295	0.011262	0.011295
0.011363	0.011362	0.011396	0.011295	0.011195	0.011463	0.011362
0.011297	0.011195	0.011295	0.011396	0.011295	0.011430	0.011463
0.011631	0.011333	0.011497	0.011396	0.011396	0.011564	0.011195
0.011363	0.011262	0.011228	0.011228	0.011295	0.011195	0.011295
0.011329	0.011362	0.011262	0.011295	0.011262	0.011597	0.011597
0.011329	0.011363	0.011429	0.011362	0.012002	0.011417	0.011306
0.011412	0.011372	0.011447	0.011337	0.011480	0.011405	0.011370
0.011918	0.011268	0.011403	0.011337	0.011611	0.011468	0.011502
0.011978	0.011399	0.011533	0.011597	0.011463	0.011463	0.011499
0.011161	0.011262	0.011463	0.011429	0.011432	0.011429	0.011397
0.011262	0.011127	0.011161	0.011262	0.011094	0.011228	0.011127
0.011262	0.011295	0.011194	0.011161	0.011262	0.011194	0.011228
0.011228	0.011161	0.011228	0.011262	0.011161	0.011295	0.011396
0.011295	0.011362	0.011295	0.011295	0.011228	0.011631	0.011262
0.011269	0.011228	0.011463	0.011467	0.011262	0.011329	0.011329
0.011329	0.011195	0.011228	0.011195	0.011362	0.011362	0.011295
0.011295	0.011429	0.011597	0.011329	0.011530	0.011530	0.011396
0.011228	0.011295	0.011329	0.011732	0.011295	0.011262	0.011295
0.011329	0.011262	0.011262	0.011564	0.011429	0.011530	0.011262
0.011362	0.011429	0.011295	0.011362	0.011329	0.011195	0.011530
0.011295	0.011262	0.011262	0.011429	0.011262	0.011362	0.011597
0.011295	0.011671	0.011664	0.011467	0.011173	0.011366	0.011215
0.011225	0.011683	0.011201	0.011417	0.011207	0.011366	0.011326
0.011417	0.011126	0.011283	0.011126	0.011168	0.011328	0.011303
0.011324	0.011178	0.011342	0.011272	0.011296	0.011429	0.011497
0.011497	0.011363	0.011466	0.011467	0.011603	0.011626	0.011295
0.011530	0.011396	0.011262	0.011362	0.011161	0.011396	0.011195
0.011262	0.011262	0.011228	0.011195	0.011228	0.011195	0.011295
0.011270	0.011270	0.011266	0.011300	0.011437	0.011333	0.011402
0.011401	0.011329	0.011134	0.011132	0.011263	0.011433	0.011433
0.011228	0.011297	0.011531	0.011500	0.011430	0.011464	0.011396
0.011329	0.011396	0.011396	0.011295	0.011866	0.011463	0.011530
0.011564	0.011630	0.011265	0.011337	0.011228	0.011429	0.011396
0.011430	0.011463	0.011463	0.011463	0.011611	0.011597	0.011234
0.011128	0.011130	0.011230	0.011196	0.011299	0.011135	0.011468
0.011266	0.011323	0.000000	0.011248	0.011262	0.011334	0.011161
0.011195	0.011195	0.011195	0.011228	0.011429	0.011396	0.011329
0.011429	0.011497	0.011228	0.011228	0.011262	0.011262	0.011263
0.011331	0.011262	0.011262	0.011296	0.011429	0.011497	0.011497
0.011363	0.011466	0.011467	0.011603	0.011626	0.011295	0.011463
0.011396	0.011396	0.011229	0.011408	0.011338	0.011367	0.011235
0.011336	0.011561	0.011357	0.011298	0.011127	0.011329	0.011195
0.011295	0.011295	0.011195	0.011296	0.011396	0.011398	0.011295
0.011195	0.011128	0.011262	0.011195	0.011128	0.011230	0.011329
0.011295	0.011228	0.011262	0.011295	0.011262	0.011195	0.011396
0.011262	0.011295	0.011295	0.011228	0.011295	0.011530	0.011194
0.011161	0.011631	0.011228	0.011194	0.011228	0.011597	0.011189
0.011622	0.011188	0.011211	0.011422	0.011508	0.011419	0.011144
0.011165	0.011396	0.011496	0.011603	0.011464	0.011601	0.011733
0.011632	0.011669	0.011631	0.011880	0.011597	0.012472	0.011633
0.012372	0.012139	0.012273	0.012545	0.011597	0.011295	0.011598
0.012036	0.011971	0.011564	0.011463	0.011531	0.011531	0.011500
0.011396	0.011399	0.011463	0.011430	0.011800	0.011866	0.011867
0.011767	0.011867	0.011970	0.011737	0.012037	0.012042	0.012104
0.012104	0.012138	0.012084	0.012104	0.012106	0.011329	0.011631
0.011699	0.012202	0.011704	0.011933	0.012406	0.011468	0.011363
0.011329	0.011329	0.011799	0.011800	0.011940	0.011664	0.011330

S090	0.011664	0.011664	0.012001	0.011765	0.011765	0.011832
0.011671	0.011603	0.011832	0.011262	0.011329	0.011296	0.011262
0.011295	0.011463	0.011262	0.011295	0.011362	0.011329	0.011295
0.011262	0.011295	0.011396	0.011262	0.011295	0.011295	0.011637
0.011530	0.011295	0.011194	0.011429	0.011362	0.011329	0.011362
0.011329	0.011429	0.011329	0.011329	0.011362	0.011329	0.011496
0.011262	0.011329	0.011362	0.011295	0.011396	0.011429	0.011362
0.011329	0.011497	0.011396	0.011664	0.011329	0.011530	0.011329
0.011295	0.011396	0.011463	0.011530	0.011698	0.011396	0.011496
0.011362	0.011429	0.011329	0.011564	0.011799	0.011329	0.011396
0.011295	0.011396	0.011362	0.011429	0.011362	0.011362	0.011362
0.011262	0.011295	0.011262	0.011362	0.011329	0.011363	0.011429
0.011362	0.011329	0.011396	0.011295	0.011429	0.011262	0.000000
0.011496	0.011429	0.011664	0.011497	0.011329	0.011564	0.011329
0.011468	0.011695	0.011475	0.011138	0.011172	0.011318	0.011375
0.012011	0.011416	0.011184	0.011564	0.011564	0.011362	0.011463
0.011396	0.011362	0.011429	0.011329	0.011429	0.011362	0.011362
0.011329	0.011429	0.011631	0.011496	0.011396	0.011362	0.011295
0.011262	0.011262	0.011329	0.011295	0.011497	0.011463	0.011396
0.011497	0.011564	0.011295	0.011295	0.011329	0.011329	0.011330
0.011398	0.011329	0.011329	0.011168	0.011241	0.011200	0.011329
0.011297	0.011232	0.011245	0.011267	0.011230	0.011232	0.011297
0.011528	0.011302	0.011336	0.011262	0.011233	0.011397	0.011363
0.011430	0.011330	0.011363	0.011463	0.011396	0.011765	0.011564
0.011597	0.011463	0.011329	0.011430	0.011228	0.011463	0.011262
0.011329	0.011329	0.011295	0.011262	0.011295	0.011262	0.011362
0.011329	0.011429	0.011362	0.011262	0.011363	0.011463	0.011466
0.011463	0.011362	0.011262	0.011195	0.011329	0.011262	0.011195
0.011298	0.011463	0.011262	0.011463	0.011228	0.011329	0.011295
0.011396	0.011295	0.011329	0.011295	0.011329	0.011195	0.011329
0.011362	0.011295	0.011362	0.011295	0.011329	0.011362	0.011597
0.011698	0.011631	0.011698	0.011431	0.011698	0.011329	0.011295
0.011330	0.011586	0.011426	0.011197	0.011705	0.011197	0.011296
0.011705	0.011604	0.011496	0.011698	0.011402	0.011564	0.011530
0.011463	0.011631	0.011486	0.011307	0.011354	0.011362	0.011432
0.011530	0.011431	0.011567	0.011635	0.011564	0.011631	0.011738
0.011466	0.011637	0.011568	0.011500	0.011767	0.011228	0.011431
0.011396	0.011329	0.011228	0.011228	0.011295	0.011295	0.011362
0.011295	0.011362	0.011262	0.011362	0.011295	0.011295	0.011362
0.011295	0.011731	0.011631	0.011664	0.011194	0.011295	0.011329
0.011329	0.011295	0.011362	0.011396	0.011262	0.011262	0.011262
0.011799	0.011429	0.011429	0.011228	0.011329	0.011497	0.011262
0.011262	0.011329	0.011295	0.011463	0.011262	0.011396	0.011530
0.011463	0.011295	0.011295	0.011228	0.011228	0.011396	0.011329
0.011228	0.011329	0.011195	0.011228	0.011362	0.011497	0.011262
0.011228	0.011362	0.011295	0.011195	0.011530	0.011195	0.011362
0.011437	0.011262	0.011564	0.011396	0.011429	0.011295	0.011396
0.011262	0.011530	0.011194	0.011463	0.011262	0.011262	0.011194
0.011429	0.011597	0.011329	0.011396	0.011228	0.011631	0.011262
0.011262	0.011262	0.011295	0.011396	0.011295	0.011396	0.011463
0.011463	0.011429	0.011329	0.011429	0.011396	0.011262	0.011228
0.011262	0.011262	0.011329	0.011262	0.011228	0.011396	0.011262
0.011295	0.011295	0.011429	0.011463	0.011362	0.011295	0.011295
0.011362	0.011295	0.011396	0.011262	0.011396	0.011496	0.011799
0.011295	0.011698	0.011267	0.017785	0.011191	0.011305	0.011618
0.011579	0.011623	0.011322	0.011292	0.011664	0.011647	0.011303
0.011440	0.011487	0.011138	0.011228	0.011216	0.011258	0.011189
0.011436	0.011395	0.011394	0.011259	0.011429	0.011463	0.011466
0.011335	0.011429	0.011429	0.011396	0.011362	0.011362	0.011329
0.011362	0.011295	0.011329	0.011362	0.011329	0.011262	0.011295

0.011295	0.011362	0.011262	0.011362	0.011396	0.011429	0.011329
0.011362	0.011329	0.011665	0.011665	0.011396	0.011430	0.011463
0.011228	0.011228	0.011262	0.011362	0.011228	0.011463	0.011463
0.011497	0.011496	0.011429	0.011530	0.011698	0.011532	0.011362
0.011362	0.011429	0.011463	0.011295	0.011362	0.011329	0.011362
0.011430	0.011430	0.011463	0.011430	0.011330	0.011530	0.011497
0.011365	0.011262	0.011362	0.011463	0.011362	0.011497	0.011530
0.011698	0.011402	0.011564	0.011530	0.011463	0.011631	0.011262
0.011431	0.011295	0.011295	0.011295	0.011362	0.011262	0.011362
0.011396	0.011429	0.011329	0.011362	0.011329	0.011665	0.011665
0.011396	0.011430	0.011496	0.011429	0.012069	0.011489	0.011412
0.011482	0.011442	0.011552	0.011441	0.011550	0.011474	0.011405
0.012021	0.011337	0.011471	0.011406	0.011680	0.011536	0.011639
0.012046	0.011512	0.011601	0.011664	0.011530	0.011530	0.011567
0.011228	0.011396	0.011530	0.011496	0.011499	0.011496	0.011431
0.011329	0.011262	0.011295	0.011194	0.011228	0.011295	0.011194
0.011329	0.011362	0.011262	0.011228	0.011329	0.011262	0.011295
0.011295	0.011228	0.011295	0.011329	0.011228	0.011429	0.011463
0.011362	0.011429	0.011362	0.011362	0.011295	0.011698	0.011329
0.011373	0.011295	0.011530	0.011535	0.011396	0.011396	0.011396
0.011396	0.011329	0.011295	0.011329	0.011429	0.011429	0.011362
0.011362	0.011496	0.011664	0.011396	0.011597	0.011597	0.011463
0.011295	0.011362	0.011396	0.011799	0.011362	0.011396	0.011362
0.011396	0.011329	0.011329	0.011631	0.011564	0.011597	0.011329
0.011429	0.011496	0.011362	0.011429	0.011396	0.011262	0.011597
0.011362	0.011329	0.011329	0.011496	0.011329	0.011429	0.011664
0.011362	0.011739	0.011731	0.011467	0.011173	0.011434	0.011215
0.011170	0.011683	0.011236	0.011328	0.011207	0.011315	0.011326
0.011468	0.011126	0.011283	0.011126	0.011168	0.011328	0.011385
0.011399	0.011296	0.011377	0.011307	0.011364	0.011497	0.011564
0.011564	0.011430	0.011533	0.011535	0.011670	0.011696	0.011362
0.011597	0.011463	0.011329	0.011430	0.011228	0.011463	0.011262
0.011329	0.011329	0.011295	0.011262	0.011295	0.011262	0.011362
0.011305	0.011305	0.011300	0.011334	0.011471	0.011436	0.011436
0.011435	0.011396	0.011171	0.011168	0.011297	0.011467	0.011467
0.011296	0.011331	0.011599	0.011533	0.011497	0.011531	0.011463
0.011396	0.011463	0.011463	0.011362	0.011933	0.011530	0.011597
0.011631	0.011701	0.011333	0.011407	0.011295	0.011497	0.011463
0.011497	0.011530	0.011530	0.011530	0.011689	0.011664	0.011303
0.011195	0.011199	0.011298	0.011264	0.011368	0.011209	0.011536
0.011335	0.011455	0.011203	0.011322	0.011329	0.011368	0.011295
0.011262	0.011262	0.011329	0.011295	0.011497	0.011463	0.011396
0.011497	0.011564	0.011295	0.011295	0.011329	0.011329	0.011330
0.011398	0.011329	0.011329	0.011364	0.011497	0.011564	0.011564
0.011430	0.011533	0.011535	0.011670	0.011696	0.011362	0.011530
0.011463	0.011463	0.011297	0.011478	0.011408	0.011435	0.011270
0.011473	0.011652	0.011441	0.011366	0.011262	0.011463	0.011329
0.011429	0.011362	0.011262	0.011363	0.011463	0.011466	0.011362
0.011262	0.011195	0.011329	0.011262	0.011195	0.011298	0.011463
0.011362	0.011295	0.011329	0.011362	0.011329	0.011262	0.011463
0.011329	0.011362	0.011362	0.011295	0.011362	0.011597	0.011262
0.011295	0.011698	0.011295	0.011329	0.011295	0.011664	0.011189
0.011678	0.011188	0.011211	0.011422	0.011508	0.011455	0.011102
0.011165	0.011463	0.011564	0.011671	0.011531	0.011635	0.011800
0.011699	0.011737	0.011698	0.011914	0.011664	0.012539	0.011700
0.012439	0.012207	0.012341	0.012612	0.011664	0.011362	0.011665
0.012103	0.012004	0.011631	0.011530	0.011598	0.011598	0.011567
0.011463	0.011433	0.011530	0.011497	0.011867	0.011933	0.011935
0.011834	0.011934	0.012038	0.011770	0.012104	0.012110	0.012171
0.012172	0.012205	0.012118	0.012172	0.012173	0.011396	0.011699

0.011766	0.012270	0.011772	0.012001	0.012474	0.011536	0.011430
0.011396	0.011396	0.011867	0.011883	0.011975	0.011731	0.011397
S091	0.011832	0.011832	0.012169	0.011933	0.011933	0.012000
0.011807	0.011739	0.012000	0.011429	0.011496	0.011464	0.011429
0.011463	0.011631	0.011429	0.011463	0.011530	0.011496	0.011463
0.011429	0.011463	0.011564	0.011430	0.011463	0.011463	0.011773
0.011698	0.011463	0.011362	0.011597	0.011530	0.011497	0.011530
0.011496	0.011597	0.011496	0.011496	0.011530	0.011496	0.011664
0.011429	0.011496	0.011530	0.011463	0.011564	0.011597	0.011530
0.011496	0.011664	0.011564	0.011832	0.011496	0.011765	0.011496
0.011463	0.011564	0.011631	0.011765	0.011866	0.011564	0.011664
0.011530	0.011597	0.011496	0.011731	0.011967	0.011496	0.011564
0.011463	0.011564	0.011530	0.011597	0.011530	0.011530	0.011530
0.011429	0.011463	0.011429	0.011530	0.011496	0.011531	0.011262
0.011530	0.011496	0.011564	0.011463	0.011597	0.011429	0.011496
0.000000	0.011597	0.011832	0.011664	0.011496	0.011731	0.011496
0.011653	0.011882	0.011702	0.011412	0.011359	0.011502	0.011572
0.012195	0.011721	0.011390	0.011731	0.011731	0.011530	0.011631
0.011564	0.011530	0.011597	0.011496	0.011597	0.011530	0.011530
0.011496	0.011597	0.011799	0.011664	0.011564	0.011530	0.011463
0.011429	0.011429	0.011497	0.011463	0.011665	0.011631	0.011564
0.011665	0.011732	0.011463	0.011463	0.011497	0.011497	0.011465
0.011533	0.011497	0.011497	0.011348	0.011422	0.011411	0.011497
0.011500	0.011404	0.011503	0.011474	0.011400	0.011437	0.011465
0.011744	0.011508	0.011542	0.011430	0.011440	0.011565	0.011531
0.011598	0.011532	0.011531	0.011631	0.011564	0.011933	0.011732
0.011765	0.011631	0.011496	0.011597	0.011396	0.011631	0.011430
0.011496	0.011496	0.011463	0.011429	0.011463	0.011429	0.011530
0.011497	0.011597	0.011530	0.011429	0.011532	0.011631	0.011635
0.011631	0.011530	0.011429	0.011364	0.011497	0.011429	0.011362
0.011468	0.011631	0.011430	0.011631	0.011396	0.011497	0.011463
0.011564	0.011463	0.011497	0.011463	0.011497	0.011362	0.011497
0.011530	0.011463	0.011530	0.011463	0.011497	0.011530	0.011765
0.011866	0.011799	0.011866	0.011633	0.011866	0.011497	0.011463
0.011499	0.011828	0.011609	0.011403	0.011875	0.011367	0.011493
0.011841	0.011774	0.011664	0.011866	0.011572	0.011732	0.011698
0.011631	0.011799	0.011664	0.011555	0.011563	0.011530	0.011601
0.011698	0.011600	0.011736	0.011805	0.011732	0.011799	0.011907
0.011635	0.011807	0.011737	0.011669	0.011935	0.011396	0.011599
0.011564	0.011496	0.011396	0.011396	0.011463	0.011463	0.011530
0.011463	0.011530	0.011429	0.011530	0.011463	0.011463	0.011530
0.011463	0.011899	0.011799	0.011832	0.011362	0.011463	0.011496
0.011496	0.011463	0.011530	0.011564	0.011429	0.011429	0.011429
0.011967	0.011597	0.011597	0.011396	0.011496	0.011664	0.011429
0.011429	0.011496	0.011463	0.011631	0.011429	0.011564	0.011698
0.011631	0.011463	0.011463	0.011396	0.011396	0.011564	0.011496
0.011396	0.011496	0.011362	0.011396	0.011530	0.011665	0.011429
0.011396	0.011530	0.011463	0.011362	0.011698	0.011362	0.011530
0.011608	0.011429	0.011732	0.011564	0.011597	0.011463	0.011564
0.011429	0.011765	0.011362	0.011631	0.011429	0.011429	0.011362
0.011597	0.011799	0.011496	0.011564	0.011396	0.011799	0.011429
0.011429	0.011429	0.011463	0.011564	0.011463	0.011564	0.011631
0.011631	0.011597	0.011496	0.011597	0.011564	0.011429	0.011396
0.011429	0.011429	0.011496	0.011429	0.011396	0.011564	0.011429
0.011463	0.011463	0.011597	0.011631	0.011530	0.011463	0.011463
0.011530	0.011463	0.011564	0.011429	0.011564	0.011664	0.011967
0.011463	0.011866	0.011440	0.017898	0.011387	0.011488	0.011793
0.011787	0.011799	0.011479	0.011466	0.011829	0.011854	0.011425
0.011724	0.011671	0.011372	0.011479	0.011450	0.011406	0.011447
0.011717	0.011581	0.011579	0.011457	0.011597	0.011631	0.011635

0.011507	0.011597	0.011597	0.011564	0.011530	0.011530	0.011497
0.011530	0.011463	0.011497	0.011530	0.011497	0.011429	0.011463
0.011463	0.011530	0.011429	0.011530	0.011564	0.011597	0.011496
0.011530	0.011497	0.011833	0.011833	0.011564	0.011598	0.011631
0.011396	0.011396	0.011429	0.011530	0.011396	0.011631	0.011631
0.011665	0.011664	0.011597	0.011698	0.011866	0.011666	0.011530
0.011530	0.011597	0.011631	0.011463	0.011530	0.011497	0.011530
0.011598	0.011598	0.011631	0.011598	0.011498	0.011698	0.011665
0.011500	0.011429	0.011530	0.011631	0.011530	0.011665	0.011698
0.011866	0.011572	0.011732	0.011698	0.011631	0.011799	0.011430
0.011599	0.011497	0.011463	0.011463	0.011530	0.011429	0.011530
0.011564	0.011597	0.011496	0.011530	0.011497	0.011833	0.011833
0.011564	0.011598	0.011664	0.011597	0.012238	0.011667	0.011588
0.011623	0.011615	0.011728	0.011614	0.011725	0.011646	0.011612
0.012193	0.011510	0.011642	0.011579	0.011852	0.011706	0.011809
0.012216	0.011701	0.011736	0.011832	0.011698	0.011698	0.011702
0.011396	0.011564	0.011698	0.011664	0.011634	0.011664	0.011633
0.011496	0.011429	0.011463	0.011496	0.011396	0.011463	0.011362
0.011496	0.011530	0.011429	0.011396	0.011496	0.011429	0.011463
0.011463	0.011396	0.011463	0.011496	0.011396	0.011597	0.011631
0.011530	0.011597	0.011530	0.011530	0.011463	0.011866	0.011496
0.011547	0.011463	0.011698	0.011705	0.011564	0.011564	0.011564
0.011564	0.011496	0.011463	0.011496	0.011597	0.011597	0.011530
0.011530	0.011664	0.011832	0.011563	0.011765	0.011765	0.011564
0.011463	0.011530	0.011564	0.011967	0.011530	0.011564	0.011530
0.011564	0.011496	0.011496	0.011799	0.011732	0.011765	0.011496
0.011597	0.011664	0.011530	0.011597	0.011564	0.011429	0.011765
0.011530	0.011496	0.011496	0.011664	0.011496	0.011597	0.011832
0.011530	0.011875	0.011899	0.011670	0.011455	0.011604	0.011525
0.011280	0.011878	0.011411	0.011596	0.011499	0.011467	0.011538
0.011672	0.011258	0.011505	0.011324	0.011437	0.011596	0.011587
0.011588	0.011454	0.011553	0.011448	0.011532	0.011665	0.011732
0.011732	0.011598	0.011703	0.011636	0.011840	0.011873	0.011530
0.011765	0.011631	0.011496	0.011597	0.011396	0.011631	0.011430
0.011496	0.011496	0.011463	0.011429	0.011463	0.011429	0.011530
0.011514	0.011515	0.011505	0.011540	0.011676	0.011641	0.011641
0.011639	0.011564	0.011392	0.011384	0.011500	0.011670	0.011671
0.011464	0.011534	0.011767	0.011736	0.011666	0.011699	0.011631
0.011564	0.011631	0.011631	0.011530	0.012101	0.011698	0.011765
0.011799	0.011843	0.011503	0.011580	0.011463	0.011664	0.011631
0.011666	0.011698	0.011698	0.011698	0.011807	0.011832	0.011476
0.011364	0.011372	0.011469	0.011434	0.011538	0.011396	0.011706
0.011506	0.011630	0.011394	0.011547	0.011497	0.011573	0.011463
0.011429	0.011429	0.011497	0.011463	0.011665	0.011631	0.011564
0.011665	0.011732	0.011463	0.011463	0.011497	0.011497	0.011465
0.011533	0.011497	0.011497	0.011532	0.011665	0.011732	0.011732
0.011598	0.011703	0.011636	0.011840	0.011873	0.011530	0.011698
0.011631	0.011631	0.011466	0.011652	0.011547	0.011605	0.011481
0.011646	0.011835	0.011610	0.011536	0.011430	0.011631	0.011497
0.011597	0.011530	0.011429	0.011532	0.011631	0.011635	0.011530
0.011429	0.011364	0.011497	0.011429	0.011362	0.011468	0.011631
0.011530	0.011463	0.011497	0.011530	0.011497	0.011429	0.011631
0.011496	0.011530	0.011530	0.011463	0.011530	0.011765	0.011429
0.011463	0.011866	0.011463	0.011496	0.011463	0.011832	0.011403
0.011903	0.011400	0.011511	0.011624	0.011711	0.011634	0.011353
0.011480	0.011631	0.011731	0.011841	0.011700	0.011838	0.011968
0.011868	0.011906	0.011866	0.012120	0.011832	0.012707	0.011869
0.012608	0.012376	0.012510	0.012781	0.011832	0.011530	0.011833
0.012272	0.012207	0.011799	0.011698	0.011766	0.011766	0.011737
0.011631	0.011637	0.011698	0.011665	0.012035	0.012101	0.012103

0.012002	0.012103	0.012207	0.011974	0.012272	0.012279	0.012339
0.012340	0.012374	0.012323	0.012340	0.012342	0.011564	0.011867
0.011935	0.012438	0.011942	0.012169	0.012643	0.011705	0.011598
0.011564	0.011563	0.012035	0.012089	0.012151	0.011899	0.011566
S092	0.011765	0.011765	0.012101	0.011866	0.011866	0.011933
0.011773	0.011705	0.011933	0.011362	0.011429	0.011397	0.011362
0.011396	0.011564	0.011362	0.011396	0.011463	0.011429	0.011396
0.011362	0.011396	0.011496	0.011363	0.011396	0.011396	0.011739
0.011631	0.011396	0.011295	0.011530	0.011463	0.011429	0.011463
0.011429	0.011530	0.011429	0.011429	0.011463	0.011429	0.011597
0.011362	0.011429	0.011463	0.011396	0.011496	0.011530	0.011463
0.011429	0.011597	0.011497	0.011765	0.011429	0.011698	0.011429
0.011396	0.011497	0.011564	0.011698	0.011799	0.011496	0.011597
0.011463	0.011530	0.011429	0.011664	0.011900	0.011429	0.011496
0.011396	0.011496	0.011463	0.011530	0.011463	0.011463	0.011463
0.011362	0.011396	0.011362	0.011463	0.011429	0.011464	0.011530
0.011463	0.011429	0.011496	0.011396	0.011530	0.011362	0.011429
0.011597	0.000000	0.011631	0.011597	0.011429	0.011664	0.011429
0.011616	0.011845	0.011664	0.011334	0.011359	0.011428	0.011572
0.012195	0.011620	0.011348	0.011664	0.011664	0.011463	0.011564
0.011496	0.011463	0.011530	0.011429	0.011530	0.011463	0.011463
0.011429	0.011530	0.011731	0.011597	0.011496	0.011463	0.011396
0.011362	0.011362	0.011429	0.011396	0.011597	0.011564	0.011497
0.011597	0.011665	0.011396	0.011396	0.011430	0.011429	0.011432
0.011500	0.011429	0.011429	0.011276	0.011349	0.011341	0.011430
0.011433	0.011369	0.011392	0.011405	0.011332	0.011368	0.011398
0.011636	0.011439	0.011473	0.011363	0.011371	0.011497	0.011464
0.011531	0.011465	0.011464	0.011564	0.011496	0.011866	0.011665
0.011698	0.011564	0.011429	0.011530	0.011329	0.011564	0.011362
0.011429	0.011429	0.011396	0.011362	0.011396	0.011362	0.011463
0.011429	0.011530	0.011463	0.011362	0.011464	0.011564	0.011567
0.011564	0.011463	0.011362	0.011296	0.011429	0.011362	0.011295
0.011400	0.011564	0.011362	0.011564	0.011329	0.011430	0.011396
0.011497	0.011396	0.011429	0.011396	0.011429	0.011295	0.011429
0.011463	0.011396	0.011463	0.011396	0.011429	0.011463	0.011698
0.011799	0.011732	0.011799	0.011566	0.011799	0.011429	0.011329
0.011432	0.011707	0.011517	0.011335	0.011807	0.011299	0.011454
0.011807	0.011706	0.011597	0.011799	0.011504	0.011664	0.011631
0.011564	0.011732	0.011593	0.011390	0.011479	0.011463	0.011534
0.011631	0.011532	0.011669	0.011737	0.011664	0.011732	0.011839
0.011568	0.011739	0.011669	0.011601	0.011867	0.011329	0.011532
0.011496	0.011429	0.011329	0.011329	0.011396	0.011396	0.011463
0.011396	0.011463	0.011362	0.011463	0.011396	0.011396	0.011463
0.011396	0.011832	0.011731	0.011765	0.011295	0.011396	0.011429
0.011429	0.011396	0.011463	0.011496	0.011362	0.011362	0.011362
0.011899	0.011530	0.011530	0.011329	0.011429	0.011597	0.011362
0.011362	0.011429	0.011396	0.011564	0.011362	0.011496	0.011631
0.011564	0.011396	0.011396	0.011329	0.011329	0.011496	0.011429
0.011329	0.011429	0.011295	0.011329	0.011463	0.011598	0.011362
0.011329	0.011463	0.011396	0.011295	0.011631	0.011295	0.011463
0.011539	0.011362	0.011664	0.011497	0.011530	0.011396	0.011496
0.011362	0.011698	0.011295	0.011564	0.011362	0.011362	0.011295
0.011530	0.011732	0.011429	0.011496	0.011329	0.011731	0.011362
0.011362	0.011362	0.011396	0.011496	0.011396	0.011496	0.011564
0.011564	0.011530	0.011429	0.011530	0.011496	0.011362	0.011329
0.011362	0.011362	0.011429	0.011362	0.011329	0.011496	0.011362
0.011396	0.011396	0.011530	0.011564	0.011463	0.011396	0.011396
0.011463	0.011396	0.011496	0.011362	0.011496	0.011597	0.011899
0.011396	0.011799	0.011405	0.017936	0.011322	0.011427	0.011758
0.011718	0.011764	0.011532	0.011524	0.011774	0.011785	0.011364

0.011629	0.011549	0.011294	0.011437	0.011372	0.011406	0.011361
0.011623	0.011544	0.011579	0.011457	0.011530	0.011564	0.011567
0.011438	0.011530	0.011530	0.011497	0.011463	0.011463	0.011430
0.011463	0.011396	0.011429	0.011463	0.011430	0.011362	0.011396
0.011396	0.011463	0.011362	0.011463	0.011497	0.011530	0.011429
0.011463	0.011429	0.011765	0.011765	0.011497	0.011531	0.011564
0.011329	0.011329	0.011362	0.011463	0.011329	0.011564	0.011564
0.011597	0.011597	0.011530	0.011631	0.011799	0.011633	0.011463
0.011463	0.011530	0.011564	0.011396	0.011463	0.011429	0.011463
0.011531	0.011530	0.011564	0.011530	0.011431	0.011631	0.011597
0.011466	0.011362	0.011463	0.011564	0.011463	0.011597	0.011631
0.011799	0.011504	0.011664	0.011631	0.011564	0.011732	0.011363
0.011498	0.011430	0.011396	0.011396	0.011463	0.011362	0.011463
0.011497	0.011530	0.011429	0.011463	0.011429	0.011765	0.011765
0.011497	0.011531	0.011597	0.011530	0.012170	0.011596	0.011517
0.011587	0.011545	0.011657	0.011545	0.011655	0.011577	0.011543
0.012124	0.011441	0.011574	0.011510	0.011783	0.011638	0.011741
0.012148	0.011625	0.011703	0.011765	0.011631	0.011631	0.011668
0.011329	0.011497	0.011631	0.011597	0.011601	0.011597	0.011565
0.011429	0.011362	0.011396	0.011429	0.011329	0.011396	0.011295
0.011429	0.011463	0.011362	0.011329	0.011429	0.011362	0.011396
0.011396	0.011329	0.011396	0.011429	0.011329	0.011530	0.011564
0.011463	0.011530	0.011463	0.011463	0.011396	0.011799	0.011429
0.011478	0.011396	0.011631	0.011637	0.011497	0.011496	0.011295
0.011496	0.011429	0.011396	0.011429	0.011530	0.011530	0.011463
0.011463	0.011597	0.011765	0.011496	0.011698	0.011698	0.011564
0.011396	0.011463	0.011496	0.011900	0.011195	0.011497	0.011463
0.011496	0.011429	0.011429	0.011732	0.011664	0.011698	0.011429
0.011530	0.011597	0.011463	0.011530	0.011496	0.011362	0.011698
0.011463	0.011429	0.011429	0.011597	0.011429	0.011530	0.011765
0.011463	0.011841	0.011832	0.011629	0.011342	0.011536	0.011370
0.011280	0.011839	0.011376	0.011596	0.011402	0.011467	0.011485
0.011621	0.011258	0.011450	0.011258	0.011383	0.011462	0.011547
0.011550	0.011454	0.011518	0.011448	0.011465	0.011597	0.011665
0.011665	0.011531	0.011635	0.011636	0.011772	0.011802	0.011463
0.011698	0.011564	0.011429	0.011530	0.011329	0.011564	0.011362
0.011429	0.011429	0.011396	0.011362	0.011396	0.011362	0.011463
0.011444	0.011445	0.011437	0.011471	0.011608	0.011573	0.011572
0.011571	0.011497	0.011282	0.011276	0.011433	0.011602	0.011603
0.011396	0.011467	0.011700	0.011668	0.011598	0.011632	0.011564
0.011496	0.011564	0.011563	0.011463	0.012034	0.011631	0.011698
0.011732	0.011772	0.011435	0.011511	0.011396	0.011597	0.011564
0.011598	0.011631	0.011631	0.011631	0.011807	0.011765	0.011407
0.011297	0.011303	0.011401	0.011366	0.011470	0.011321	0.011638
0.011437	0.011586	0.011346	0.011472	0.011430	0.011505	0.011396
0.011362	0.011362	0.011429	0.011396	0.011597	0.011564	0.011497
0.011597	0.011665	0.011396	0.011396	0.011430	0.011429	0.011432
0.011500	0.011429	0.011429	0.011465	0.011597	0.011665	0.011665
0.011531	0.011635	0.011636	0.011772	0.011802	0.011463	0.011631
0.011564	0.011564	0.011398	0.011582	0.011512	0.011537	0.011411
0.011577	0.011789	0.011568	0.011468	0.011362	0.011564	0.011429
0.011530	0.011463	0.011362	0.011464	0.011564	0.011567	0.011463
0.011362	0.011296	0.011429	0.011362	0.011295	0.011400	0.011564
0.011463	0.011396	0.011429	0.011463	0.011430	0.011362	0.011564
0.011429	0.011463	0.011463	0.011396	0.011463	0.011698	0.011362
0.011396	0.011799	0.011396	0.011429	0.011396	0.011765	0.011360
0.011847	0.011358	0.011411	0.011583	0.011671	0.011598	0.011269
0.011323	0.011564	0.011664	0.011773	0.011632	0.011771	0.011901
0.011800	0.011838	0.011799	0.012051	0.011765	0.012640	0.011801
0.012540	0.012308	0.012442	0.012714	0.011765	0.011463	0.011766

0.012204	0.012140	0.011732	0.011631	0.011699	0.011699	0.011669
0.011564	0.011569	0.011631	0.011598	0.011968	0.012034	0.012036
0.011935	0.012035	0.012139	0.011906	0.012205	0.012211	0.012272
0.012273	0.012306	0.012255	0.012273	0.012274	0.011496	0.011799
0.011867	0.012371	0.011874	0.012102	0.012575	0.011637	0.011531
0.011496	0.011496	0.011968	0.012048	0.012116	0.011832	0.011498
S093	0.012000	0.012000	0.012135	0.011900	0.011900	0.011967
0.011535	0.011468	0.011967	0.011597	0.011664	0.011632	0.011597
0.011631	0.011799	0.011597	0.011631	0.011698	0.011664	0.011631
0.011597	0.011631	0.011530	0.011598	0.011631	0.011631	0.011501
0.011664	0.011631	0.011530	0.011564	0.011698	0.011664	0.011698
0.011664	0.011765	0.011664	0.011664	0.011698	0.011664	0.011832
0.011597	0.011664	0.011698	0.011631	0.011731	0.011765	0.011698
0.011664	0.011832	0.011732	0.011530	0.011664	0.011933	0.011664
0.011631	0.011732	0.011799	0.011933	0.011564	0.011732	0.011832
0.011698	0.011765	0.011664	0.011899	0.011664	0.011664	0.011732
0.011631	0.011731	0.011698	0.011765	0.011698	0.011698	0.011698
0.011597	0.011631	0.011597	0.011698	0.011664	0.011700	0.011765
0.011698	0.011664	0.011731	0.011631	0.011631	0.011597	0.011664
0.011832	0.011631	0.000000	0.011832	0.011664	0.011900	0.011664
0.011839	0.011471	0.011890	0.011607	0.011583	0.011649	0.011572
0.012195	0.011518	0.011554	0.011899	0.011899	0.011698	0.011664
0.011732	0.011698	0.011765	0.011664	0.011765	0.011698	0.011698
0.011664	0.011765	0.011967	0.011832	0.011732	0.011698	0.011631
0.011597	0.011597	0.011665	0.011631	0.011631	0.011598	0.011530
0.011631	0.011698	0.011631	0.011631	0.011665	0.011665	0.011668
0.011736	0.011665	0.011665	0.011492	0.011603	0.011586	0.011665
0.011670	0.011610	0.011614	0.011612	0.011570	0.011608	0.011634
0.011564	0.011681	0.011715	0.011598	0.011579	0.011733	0.011699
0.011767	0.011701	0.011699	0.011799	0.011731	0.012101	0.011900
0.011732	0.011799	0.011664	0.011765	0.011564	0.011597	0.011598
0.011664	0.011664	0.011631	0.011597	0.011631	0.011597	0.011698
0.011665	0.011765	0.011631	0.011597	0.011700	0.011799	0.011804
0.011799	0.011698	0.011597	0.011532	0.011665	0.011597	0.011530
0.011536	0.011799	0.011597	0.011799	0.011564	0.011665	0.011631
0.011732	0.011631	0.011665	0.011631	0.011665	0.011530	0.011665
0.011698	0.011631	0.011698	0.011631	0.011665	0.011698	0.011934
0.011564	0.011496	0.011564	0.011802	0.011497	0.011665	0.011631
0.011668	0.011950	0.011838	0.011575	0.011569	0.011538	0.011729
0.011569	0.011638	0.011832	0.012034	0.011743	0.011900	0.011866
0.011799	0.011967	0.011842	0.011719	0.011773	0.011698	0.011771
0.011866	0.011769	0.011906	0.011974	0.011900	0.011967	0.012077
0.011805	0.011977	0.011839	0.011838	0.012103	0.011564	0.011768
0.011731	0.011664	0.011564	0.011564	0.011631	0.011631	0.011698
0.011631	0.011698	0.011597	0.011698	0.011631	0.011631	0.011698
0.011631	0.011597	0.011496	0.011530	0.011530	0.011631	0.011664
0.011664	0.011564	0.011698	0.011732	0.011597	0.011597	0.011597
0.011664	0.011765	0.011765	0.011564	0.011664	0.011832	0.011597
0.011597	0.011664	0.011631	0.011799	0.011597	0.011530	0.011664
0.011597	0.011631	0.011631	0.011564	0.011564	0.011732	0.011664
0.011564	0.011664	0.011530	0.011564	0.011698	0.011564	0.011597
0.011564	0.011698	0.011631	0.011530	0.011396	0.011530	0.011698
0.011779	0.011597	0.011429	0.011732	0.011765	0.011631	0.011731
0.011597	0.011933	0.011530	0.011732	0.011597	0.011597	0.011530
0.011765	0.011698	0.011664	0.011732	0.011564	0.011496	0.011597
0.011597	0.011597	0.011631	0.011732	0.011631	0.011731	0.011799
0.011799	0.011765	0.011664	0.011765	0.011731	0.011597	0.011564
0.011597	0.011597	0.011664	0.011597	0.011564	0.011732	0.011597
0.011631	0.011631	0.011765	0.011799	0.011698	0.011631	0.011631
0.011698	0.011631	0.011731	0.011597	0.011731	0.011832	0.011664

0.011631	0.011564	0.011647	0.018125	0.011387	0.011794	0.011514
0.011475	0.011482	0.011846	0.011582	0.011554	0.011543	0.011728
0.011772	0.011549	0.011528	0.011688	0.011646	0.011702	0.011706
0.011436	0.011544	0.011431	0.011457	0.011765	0.011799	0.011804
0.011678	0.011765	0.011765	0.011732	0.011698	0.011698	0.011665
0.011698	0.011631	0.011664	0.011698	0.011665	0.011597	0.011631
0.011631	0.011698	0.011597	0.011698	0.011732	0.011765	0.011664
0.011698	0.011665	0.011530	0.011530	0.011732	0.011766	0.011799
0.011564	0.011564	0.011597	0.011698	0.011564	0.011799	0.011799
0.011833	0.011832	0.011765	0.011866	0.011564	0.011869	0.011698
0.011698	0.011765	0.011799	0.011631	0.011698	0.011665	0.011698
0.011766	0.011766	0.011799	0.011766	0.011666	0.011866	0.011833
0.011704	0.011597	0.011698	0.011799	0.011698	0.011833	0.011866
0.012034	0.011743	0.011900	0.011866	0.011799	0.011967	0.011598
0.011768	0.011665	0.011631	0.011631	0.011698	0.011597	0.011698
0.011732	0.011765	0.011664	0.011698	0.011665	0.011530	0.011530
0.011732	0.011766	0.011832	0.011765	0.012406	0.011846	0.011764
0.011834	0.011788	0.011904	0.011788	0.011900	0.011819	0.011784
0.012365	0.011683	0.011814	0.011752	0.012025	0.011877	0.011979
0.012386	0.011890	0.011939	0.012000	0.011866	0.011664	0.011905
0.011564	0.011732	0.011866	0.011832	0.011837	0.011631	0.011599
0.011664	0.011597	0.011631	0.011664	0.011564	0.011631	0.011530
0.011664	0.011698	0.011597	0.011564	0.011664	0.011597	0.011631
0.011631	0.011564	0.011631	0.011664	0.011564	0.011765	0.011799
0.011698	0.011765	0.011698	0.011698	0.011631	0.011564	0.011664
0.011512	0.011631	0.011665	0.011671	0.011664	0.011664	0.011463
0.011732	0.011664	0.011631	0.011664	0.011765	0.011765	0.011698
0.011698	0.011832	0.011530	0.011731	0.011933	0.011933	0.011799
0.011631	0.011698	0.011732	0.011463	0.011698	0.011664	0.011698
0.011731	0.011664	0.011664	0.011967	0.011900	0.011933	0.011664
0.011765	0.011832	0.011698	0.011765	0.011731	0.011597	0.011463
0.011698	0.011664	0.011664	0.011832	0.011664	0.011765	0.011597
0.011698	0.011603	0.011597	0.011629	0.011455	0.011774	0.011473
0.011609	0.011839	0.011587	0.011820	0.011695	0.011518	0.011591
0.011621	0.011323	0.011561	0.011390	0.011437	0.011596	0.011750
0.011776	0.011690	0.011765	0.011694	0.011701	0.011631	0.011698
0.011631	0.011766	0.011872	0.011670	0.012010	0.012015	0.011698
0.011732	0.011799	0.011664	0.011765	0.011564	0.011597	0.011598
0.011664	0.011664	0.011631	0.011597	0.011631	0.011597	0.011698
0.011654	0.011690	0.011677	0.011711	0.011847	0.011812	0.011811
0.011810	0.011733	0.011503	0.011492	0.011670	0.011840	0.011841
0.011632	0.011704	0.011936	0.011905	0.011834	0.011868	0.011799
0.011731	0.011799	0.011799	0.011698	0.011799	0.011866	0.011933
0.011967	0.011558	0.011606	0.011753	0.011631	0.011832	0.011799
0.011834	0.011866	0.011866	0.011866	0.012004	0.012000	0.011650
0.011533	0.011545	0.011639	0.011604	0.011709	0.011583	0.011400
0.011678	0.011543	0.011680	0.011735	0.011665	0.011745	0.011631
0.011597	0.011597	0.011665	0.011631	0.011631	0.011598	0.011530
0.011631	0.011698	0.011631	0.011631	0.011665	0.011665	0.011668
0.011736	0.011665	0.011665	0.011701	0.011631	0.011698	0.011631
0.011766	0.011872	0.011670	0.012010	0.012015	0.011698	0.011866
0.011799	0.011799	0.011635	0.011826	0.011755	0.011775	0.011656
0.011818	0.012109	0.011865	0.011706	0.011597	0.011799	0.011665
0.011765	0.011631	0.011597	0.011700	0.011799	0.011804	0.011698
0.011597	0.011532	0.011665	0.011597	0.011530	0.011536	0.011799
0.011698	0.011631	0.011664	0.011698	0.011665	0.011597	0.011597
0.011664	0.011698	0.011698	0.011631	0.011698	0.011933	0.011597
0.011631	0.011564	0.011631	0.011664	0.011631	0.011530	0.011574
0.011903	0.011400	0.011511	0.011543	0.011630	0.011599	0.011395
0.011480	0.011597	0.011698	0.011807	0.011666	0.011805	0.011935

0.011834	0.011872	0.011832	0.012086	0.011799	0.012674	0.011835
0.012574	0.012342	0.012476	0.012748	0.011731	0.011698	0.011800
0.012440	0.012174	0.011765	0.011665	0.011733	0.011733	0.011703
0.011597	0.011603	0.011664	0.011632	0.012002	0.012068	0.012070
0.011969	0.012069	0.012173	0.011940	0.012441	0.012449	0.012508
0.012509	0.012543	0.012494	0.012509	0.012511	0.011731	0.012035
0.012103	0.012606	0.012112	0.012068	0.012541	0.011604	0.011497
0.011396	0.011463	0.012002	0.012089	0.012151	0.011799	0.011532
S094	0.011832	0.011832	0.012169	0.011933	0.011933	0.012000
0.011841	0.011773	0.012000	0.011430	0.011496	0.011464	0.011429
0.011463	0.011631	0.011429	0.011463	0.011530	0.011497	0.011463
0.011429	0.011463	0.011564	0.011430	0.011463	0.011463	0.011807
0.011698	0.011463	0.011362	0.011597	0.011530	0.011497	0.011530
0.011497	0.011530	0.011497	0.011497	0.011530	0.011295	0.011665
0.011429	0.011496	0.011530	0.011463	0.011564	0.011597	0.011530
0.011497	0.011665	0.011564	0.011832	0.011497	0.011765	0.011497
0.011463	0.011564	0.011631	0.011765	0.011866	0.011564	0.011664
0.011530	0.011530	0.011497	0.011664	0.011967	0.011497	0.011564
0.011463	0.011564	0.011530	0.011597	0.011530	0.011530	0.011530
0.011429	0.011463	0.011429	0.011530	0.011496	0.011464	0.011597
0.011530	0.011496	0.011564	0.011463	0.011597	0.011429	0.011497
0.011664	0.011597	0.011832	0.000000	0.011497	0.011732	0.011496
0.011505	0.011882	0.011664	0.011334	0.011359	0.011355	0.011572
0.012195	0.011620	0.011390	0.011732	0.011732	0.011530	0.011631
0.011564	0.011530	0.011597	0.011497	0.011597	0.011530	0.011530
0.011496	0.011597	0.011799	0.011664	0.011564	0.011530	0.011463
0.011430	0.011430	0.011497	0.011463	0.011665	0.011631	0.011564
0.011665	0.011732	0.011463	0.011463	0.011497	0.011497	0.011499
0.011567	0.011497	0.011497	0.011312	0.011386	0.011376	0.011497
0.011264	0.011404	0.011429	0.011405	0.011400	0.011403	0.011432
0.011672	0.011474	0.011474	0.011430	0.011371	0.011531	0.011531
0.011598	0.011498	0.011531	0.011631	0.011564	0.011933	0.011732
0.011765	0.011631	0.011497	0.011598	0.011396	0.011631	0.011430
0.011497	0.011497	0.011463	0.011429	0.011463	0.011430	0.011530
0.011497	0.011598	0.011530	0.011430	0.011532	0.011631	0.011635
0.011631	0.011531	0.011430	0.011364	0.011497	0.011430	0.011363
0.011434	0.011631	0.011430	0.011631	0.011396	0.011497	0.011463
0.011564	0.011463	0.011497	0.011463	0.011497	0.011362	0.011497
0.011530	0.011463	0.011530	0.011463	0.011497	0.011530	0.011765
0.011866	0.011799	0.011866	0.011633	0.011867	0.011497	0.011463
0.011466	0.011788	0.011518	0.011403	0.011875	0.011333	0.011454
0.011875	0.011774	0.011664	0.011866	0.011572	0.011732	0.011698
0.011631	0.011799	0.011664	0.011513	0.011605	0.011530	0.011602
0.011698	0.011600	0.011736	0.011805	0.011732	0.011799	0.011907
0.011636	0.011807	0.011737	0.011466	0.011935	0.011396	0.011599
0.011564	0.011497	0.011396	0.011396	0.011463	0.011463	0.011530
0.011463	0.011530	0.011429	0.011530	0.011463	0.011463	0.011530
0.011463	0.011900	0.011799	0.011832	0.011362	0.011463	0.011497
0.011497	0.011463	0.011530	0.011564	0.011429	0.011429	0.011429
0.011967	0.011597	0.011597	0.011396	0.011496	0.011597	0.011429
0.011429	0.011496	0.011463	0.011631	0.011429	0.011564	0.011698
0.011631	0.011463	0.011463	0.011396	0.011396	0.011564	0.011496
0.011396	0.011496	0.011362	0.011396	0.011329	0.011665	0.011430
0.011396	0.011329	0.011463	0.011362	0.011698	0.011362	0.011530
0.011608	0.011429	0.011732	0.011564	0.011463	0.011463	0.011564
0.011429	0.011765	0.011362	0.011631	0.011429	0.011429	0.011362
0.011597	0.011799	0.011497	0.011564	0.011396	0.011799	0.011429
0.011429	0.011429	0.011463	0.011497	0.011463	0.011564	0.011631
0.011094	0.011597	0.011496	0.011597	0.011564	0.011429	0.011396
0.011429	0.011429	0.011497	0.011429	0.011396	0.011564	0.011429

0.011463	0.011463	0.011597	0.011631	0.011530	0.011463	0.011463
0.011530	0.011463	0.011564	0.011429	0.011564	0.011664	0.011967
0.011463	0.011866	0.011440	0.017974	0.011257	0.011549	0.011793
0.011752	0.011799	0.011427	0.011466	0.011885	0.011820	0.011485
0.011630	0.011671	0.011333	0.011437	0.011373	0.011455	0.011404
0.011623	0.011582	0.011579	0.011325	0.011597	0.011631	0.011635
0.011507	0.011597	0.011597	0.011564	0.011530	0.011530	0.011497
0.011530	0.011463	0.011497	0.011530	0.011497	0.011430	0.011463
0.011463	0.011530	0.011430	0.011531	0.011564	0.011396	0.011497
0.011530	0.011497	0.011833	0.011833	0.011564	0.011598	0.011631
0.011396	0.011396	0.011430	0.011530	0.011396	0.011631	0.011631
0.011665	0.011664	0.011597	0.011698	0.011866	0.011700	0.011530
0.011530	0.011598	0.011631	0.011463	0.011530	0.011497	0.011530
0.011598	0.011598	0.011631	0.011598	0.011498	0.011698	0.011665
0.011534	0.011430	0.011530	0.011631	0.011531	0.011665	0.011698
0.011866	0.011572	0.011732	0.011698	0.011631	0.011799	0.011430
0.011599	0.011497	0.011463	0.011463	0.011530	0.011430	0.011531
0.011564	0.011396	0.011497	0.011530	0.011497	0.011833	0.011833
0.011564	0.011598	0.011664	0.011597	0.012238	0.011668	0.011588
0.011447	0.011615	0.011728	0.011614	0.011725	0.011647	0.011612
0.012194	0.011510	0.011642	0.011579	0.011852	0.011706	0.011809
0.012216	0.011701	0.011770	0.011832	0.011698	0.011698	0.011736
0.011396	0.011564	0.011698	0.011664	0.011668	0.011664	0.011633
0.011496	0.011429	0.011463	0.011497	0.011396	0.011463	0.011362
0.011429	0.011463	0.011429	0.011396	0.011497	0.011429	0.011463
0.011463	0.011396	0.011463	0.011497	0.011396	0.011530	0.011497
0.011329	0.011463	0.011329	0.011463	0.011463	0.011866	0.011496
0.011547	0.011463	0.011699	0.011705	0.011564	0.011564	0.011564
0.011564	0.011497	0.011463	0.011497	0.011597	0.011597	0.011530
0.011530	0.011664	0.011832	0.011564	0.011765	0.011765	0.011631
0.011463	0.011530	0.011564	0.011967	0.011530	0.011564	0.011530
0.011564	0.011496	0.011496	0.011799	0.011732	0.011765	0.011497
0.011597	0.011664	0.011530	0.011597	0.011564	0.011429	0.011765
0.011530	0.011497	0.011496	0.011664	0.011497	0.011597	0.011833
0.011530	0.011909	0.011900	0.011670	0.011399	0.011570	0.011422
0.011335	0.011878	0.011411	0.011552	0.011499	0.011519	0.011485
0.011570	0.011258	0.011450	0.011258	0.011384	0.011462	0.011587
0.011588	0.011493	0.011553	0.011483	0.011532	0.011665	0.011732
0.011732	0.011598	0.011703	0.011704	0.011840	0.011873	0.011530
0.011765	0.011631	0.011497	0.011598	0.011396	0.011631	0.011430
0.011497	0.011497	0.011463	0.011429	0.011463	0.011430	0.011530
0.011444	0.011480	0.011437	0.011505	0.011642	0.011573	0.011607
0.011605	0.011565	0.011319	0.011312	0.011467	0.011636	0.011637
0.011430	0.011501	0.011734	0.011703	0.011632	0.011666	0.011631
0.011564	0.011631	0.011631	0.011530	0.012102	0.011698	0.011766
0.011799	0.011844	0.011504	0.011545	0.011463	0.011664	0.011631
0.011666	0.011698	0.011698	0.011698	0.011847	0.011832	0.011477
0.011364	0.011372	0.011469	0.011434	0.011538	0.011396	0.011706
0.011506	0.011674	0.011394	0.011510	0.011497	0.011539	0.011463
0.011430	0.011430	0.011497	0.011463	0.011665	0.011631	0.011564
0.011665	0.011732	0.011463	0.011463	0.011497	0.011497	0.011499
0.011567	0.011497	0.011497	0.011532	0.011665	0.011732	0.011732
0.011598	0.011703	0.011704	0.011840	0.011873	0.011530	0.011698
0.011631	0.011631	0.011432	0.011617	0.011582	0.011605	0.011446
0.011646	0.011789	0.011526	0.011536	0.011430	0.011631	0.011497
0.011598	0.011530	0.011430	0.011532	0.011631	0.011635	0.011531
0.011430	0.011364	0.011497	0.011430	0.011363	0.011434	0.011631
0.011530	0.011463	0.011497	0.011530	0.011497	0.011430	0.011631
0.011497	0.011530	0.011530	0.011463	0.011329	0.011698	0.011429
0.011463	0.011866	0.011463	0.011497	0.011463	0.011832	0.011403

0.011848	0.011400	0.011411	0.011624	0.011712	0.011634	0.011353
0.011323	0.011631	0.011732	0.011841	0.011700	0.011839	0.011968
0.011868	0.011906	0.011866	0.012120	0.011832	0.012708	0.011869
0.012608	0.012376	0.012510	0.012782	0.011832	0.011531	0.011833
0.012272	0.012207	0.011799	0.011698	0.011766	0.011766	0.011737
0.011631	0.011637	0.011698	0.011666	0.012036	0.012102	0.012103
0.012002	0.012103	0.012207	0.011974	0.012273	0.012280	0.012340
0.012341	0.012374	0.012289	0.012341	0.012342	0.011564	0.011867
0.011935	0.012438	0.011942	0.012169	0.012643	0.011706	0.011598
0.011564	0.011564	0.012035	0.012006	0.012116	0.011900	0.011566
S095	0.011664	0.011664	0.012001	0.011765	0.011765	0.011832
0.011637	0.011569	0.011832	0.011262	0.011329	0.011296	0.011262
0.011295	0.011463	0.011262	0.011295	0.011362	0.011329	0.011295
0.011262	0.011295	0.011396	0.011262	0.011295	0.011295	0.011603
0.011530	0.011295	0.011194	0.011429	0.011362	0.011329	0.011362
0.011329	0.011429	0.011329	0.011329	0.011362	0.011329	0.011497
0.011262	0.011329	0.011362	0.011295	0.011396	0.011429	0.011362
0.011329	0.011497	0.011396	0.011664	0.011329	0.011597	0.011329
0.011295	0.011396	0.011463	0.011597	0.011698	0.011396	0.011496
0.011362	0.011429	0.011329	0.011564	0.011799	0.011329	0.011396
0.011295	0.011396	0.011362	0.011429	0.011362	0.011362	0.011362
0.011262	0.011295	0.011262	0.011362	0.011329	0.011363	0.011429
0.011362	0.011329	0.011396	0.011295	0.011429	0.011262	0.011329
0.011496	0.011429	0.011664	0.011497	0.000000	0.011564	0.011329
0.011468	0.011695	0.011551	0.011216	0.011247	0.011355	0.011415
0.012085	0.011569	0.011225	0.011564	0.011564	0.011362	0.011463
0.011396	0.011362	0.011429	0.011329	0.011429	0.011362	0.011362
0.011329	0.011429	0.011631	0.011496	0.011396	0.011362	0.011295
0.011262	0.011262	0.011329	0.011295	0.011497	0.011463	0.011396
0.011497	0.011564	0.011295	0.011295	0.011329	0.011329	0.011330
0.011398	0.011262	0.011329	0.011204	0.011277	0.011236	0.011329
0.011331	0.011266	0.011318	0.011302	0.011230	0.011266	0.011297
0.011564	0.011336	0.011370	0.011262	0.011268	0.011397	0.011363
0.011430	0.011364	0.011363	0.011463	0.011396	0.011765	0.011564
0.011597	0.011463	0.011329	0.011430	0.011228	0.011463	0.011262
0.011329	0.011329	0.011295	0.011262	0.011295	0.011262	0.011362
0.011329	0.011430	0.011362	0.011262	0.011363	0.011463	0.011466
0.011463	0.011362	0.011262	0.011195	0.011329	0.011262	0.011195
0.011298	0.011463	0.011262	0.011463	0.011228	0.011329	0.011295
0.011329	0.011295	0.011262	0.011295	0.011329	0.011195	0.011329
0.011362	0.011295	0.011362	0.011295	0.011329	0.011362	0.011597
0.011698	0.011631	0.011698	0.011465	0.011698	0.011329	0.011295
0.011297	0.011626	0.011380	0.011232	0.011705	0.011197	0.011336
0.011671	0.011604	0.011496	0.011698	0.011402	0.011564	0.011530
0.011463	0.011631	0.011486	0.011307	0.011312	0.011362	0.011432
0.011530	0.011431	0.011567	0.011635	0.011564	0.011631	0.011670
0.011466	0.011637	0.011568	0.011500	0.011767	0.011228	0.011431
0.011329	0.011329	0.011228	0.011228	0.011295	0.011295	0.011362
0.011295	0.011362	0.011262	0.011362	0.011295	0.011295	0.011362
0.011295	0.011731	0.011631	0.011664	0.011194	0.011295	0.011329
0.011329	0.011295	0.011362	0.011396	0.011262	0.011262	0.011262
0.011799	0.011429	0.011429	0.011228	0.011329	0.011497	0.011262
0.011262	0.011329	0.011295	0.011463	0.011262	0.011396	0.011530
0.011463	0.011295	0.011295	0.011228	0.011228	0.011396	0.011329
0.011228	0.011329	0.011195	0.011228	0.011362	0.011497	0.011262
0.011228	0.011362	0.011228	0.011195	0.011530	0.011195	0.011362
0.011437	0.011262	0.011564	0.011396	0.011429	0.011295	0.011396
0.011262	0.011597	0.011194	0.011463	0.011262	0.011262	0.011194
0.011429	0.011631	0.011329	0.011396	0.011228	0.011631	0.011262
0.011262	0.011262	0.011295	0.011396	0.011295	0.011396	0.011463

0.011463	0.011429	0.011329	0.011430	0.011396	0.011262	0.011228
0.011262	0.011262	0.011329	0.011262	0.011228	0.011396	0.011262
0.011295	0.011295	0.011429	0.011463	0.011362	0.011295	0.011295
0.011362	0.011295	0.011396	0.011262	0.011396	0.011496	0.011799
0.011295	0.011698	0.011302	0.017823	0.011322	0.011427	0.011653
0.011614	0.011658	0.011374	0.011408	0.011774	0.011682	0.011364
0.011582	0.011549	0.011177	0.011312	0.011294	0.011357	0.011275
0.011576	0.011470	0.011468	0.011193	0.011429	0.011463	0.011466
0.011335	0.011430	0.011429	0.011396	0.011362	0.011362	0.011329
0.011362	0.011295	0.011329	0.011362	0.011329	0.011262	0.011295
0.011295	0.011362	0.011262	0.011363	0.011396	0.011430	0.011329
0.011362	0.011329	0.011665	0.011665	0.011396	0.011430	0.011463
0.011228	0.011228	0.011262	0.011362	0.011228	0.011463	0.011463
0.011429	0.011496	0.011429	0.011463	0.011698	0.011464	0.011362
0.011362	0.011430	0.011463	0.011295	0.011362	0.011329	0.011362
0.011430	0.011430	0.011463	0.011430	0.011330	0.011530	0.011497
0.011365	0.011262	0.011362	0.011463	0.011363	0.011497	0.011530
0.011698	0.011402	0.011564	0.011530	0.011463	0.011631	0.011262
0.011431	0.011329	0.011295	0.011295	0.011362	0.011262	0.011363
0.011396	0.011430	0.011329	0.011362	0.011329	0.011665	0.011665
0.011396	0.011430	0.011496	0.011429	0.012069	0.011453	0.011412
0.011482	0.011407	0.011552	0.011441	0.011515	0.011474	0.011405
0.012022	0.011337	0.011471	0.011406	0.011680	0.011536	0.011639
0.012046	0.011437	0.011601	0.011664	0.011530	0.011530	0.011567
0.011228	0.011396	0.011530	0.011496	0.011499	0.011496	0.011464
0.011329	0.011262	0.011295	0.011329	0.011228	0.011295	0.011194
0.011329	0.011362	0.011262	0.011228	0.011329	0.011262	0.011295
0.011295	0.011228	0.011295	0.011329	0.011228	0.011429	0.011463
0.011362	0.011429	0.011362	0.011362	0.011295	0.011698	0.011329
0.011373	0.011295	0.011530	0.011535	0.011396	0.011396	0.011396
0.011396	0.011329	0.011295	0.011329	0.011429	0.011429	0.011362
0.011362	0.011497	0.011664	0.011396	0.011597	0.011597	0.011463
0.011295	0.011362	0.011396	0.011799	0.011362	0.011396	0.011362
0.011396	0.011329	0.011329	0.011631	0.011564	0.011597	0.011329
0.011429	0.011429	0.011362	0.011429	0.011396	0.011262	0.011597
0.011362	0.011329	0.011329	0.011497	0.011329	0.011429	0.011664
0.011362	0.011705	0.011732	0.011507	0.011286	0.011434	0.011370
0.011225	0.011722	0.011271	0.011462	0.011304	0.011417	0.011326
0.011519	0.011258	0.011394	0.011258	0.011276	0.011462	0.011385
0.011399	0.011296	0.011377	0.011342	0.011364	0.011497	0.011564
0.011564	0.011430	0.011534	0.011535	0.011670	0.011696	0.011362
0.011597	0.011463	0.011329	0.011430	0.011228	0.011463	0.011262
0.011329	0.011329	0.011295	0.011262	0.011295	0.011262	0.011362
0.011305	0.011340	0.011334	0.011368	0.011505	0.011470	0.011470
0.011469	0.011396	0.011208	0.011204	0.011331	0.011433	0.011501
0.011296	0.011365	0.011599	0.011567	0.011497	0.011531	0.011463
0.011396	0.011463	0.011463	0.011362	0.011933	0.011530	0.011597
0.011631	0.011701	0.011333	0.011407	0.011295	0.011497	0.011463
0.011497	0.011530	0.011530	0.011530	0.011689	0.011664	0.011303
0.011195	0.011199	0.011299	0.011264	0.011368	0.011172	0.011536
0.011335	0.011411	0.011156	0.011322	0.011329	0.011402	0.011295
0.011262	0.011262	0.011329	0.011295	0.011497	0.011463	0.011396
0.011497	0.011564	0.011295	0.011295	0.011329	0.011329	0.011330
0.011398	0.011262	0.011329	0.011364	0.011497	0.011564	0.011564
0.011430	0.011534	0.011535	0.011670	0.011696	0.011362	0.011530
0.011463	0.011463	0.011297	0.011478	0.011408	0.011435	0.011305
0.011473	0.011653	0.011441	0.011366	0.011262	0.011463	0.011329
0.011430	0.011362	0.011262	0.011363	0.011463	0.011466	0.011362
0.011262	0.011195	0.011329	0.011262	0.011195	0.011298	0.011463
0.011362	0.011295	0.011329	0.011362	0.011329	0.011262	0.011463

0.011329	0.011362	0.011362	0.011295	0.011362	0.011597	0.011262
0.011295	0.011698	0.011295	0.011329	0.011295	0.011664	0.011232
0.011735	0.011230	0.011361	0.011463	0.011549	0.011455	0.011186
0.011323	0.011463	0.011564	0.011671	0.011531	0.011669	0.011800
0.011699	0.011737	0.011698	0.011880	0.011664	0.012472	0.011700
0.012439	0.012207	0.012307	0.012613	0.011664	0.011363	0.011665
0.012103	0.012038	0.011631	0.011463	0.011598	0.011598	0.011500
0.011463	0.011467	0.011530	0.011464	0.011867	0.011933	0.011935
0.011834	0.011934	0.012038	0.011804	0.012104	0.012110	0.012171
0.012172	0.012205	0.012152	0.012172	0.012173	0.011396	0.011699
0.011767	0.012270	0.011772	0.012001	0.012474	0.011536	0.011430
0.011396	0.011396	0.011867	0.011842	0.012010	0.011732	0.011397
S096	0.011900	0.011900	0.012236	0.012000	0.012000	0.012068
0.011875	0.011807	0.012068	0.011497	0.011564	0.011531	0.011463
0.011530	0.011698	0.011496	0.011530	0.011597	0.011564	0.011530
0.011496	0.011530	0.011631	0.011497	0.011463	0.011530	0.011841
0.011731	0.011530	0.011429	0.011664	0.011530	0.011564	0.011530
0.011564	0.011597	0.011564	0.011564	0.011597	0.011564	0.011732
0.011496	0.011564	0.011597	0.011530	0.011631	0.011664	0.011597
0.011564	0.011732	0.011631	0.011900	0.011564	0.011765	0.011564
0.011530	0.011631	0.011698	0.011765	0.011933	0.011631	0.011732
0.011530	0.011597	0.011564	0.011731	0.012034	0.011564	0.011631
0.011530	0.011631	0.011597	0.011664	0.011597	0.011597	0.011597
0.011496	0.011530	0.011496	0.011597	0.011564	0.011599	0.011664
0.011597	0.011564	0.011564	0.011530	0.011664	0.011429	0.011564
0.011731	0.011664	0.011900	0.011732	0.011564	0.000000	0.011564
0.011728	0.011957	0.011777	0.011451	0.011508	0.011612	0.011690
0.012341	0.011721	0.011513	0.011799	0.011799	0.011597	0.011698
0.011631	0.011597	0.011664	0.011564	0.011664	0.011530	0.011597
0.011564	0.011664	0.011866	0.011731	0.011631	0.011597	0.011463
0.011497	0.011497	0.011497	0.011530	0.011732	0.011698	0.011631
0.011732	0.011799	0.011530	0.011530	0.011564	0.011564	0.011567
0.011635	0.011564	0.011564	0.011420	0.011530	0.011481	0.011565
0.011568	0.011507	0.011577	0.011543	0.011468	0.011506	0.011533
0.011816	0.011577	0.011542	0.011497	0.011475	0.011632	0.011598
0.011666	0.011599	0.011598	0.011698	0.011631	0.011866	0.011799
0.011765	0.011698	0.011564	0.011665	0.011463	0.011631	0.011497
0.011564	0.011564	0.011530	0.011497	0.011530	0.011497	0.011597
0.011497	0.011597	0.011598	0.011497	0.011599	0.011698	0.011702
0.011631	0.011598	0.011497	0.011431	0.011564	0.011497	0.011430
0.011536	0.011631	0.011430	0.011631	0.011463	0.011564	0.011530
0.011564	0.011530	0.011497	0.011530	0.011564	0.011430	0.011497
0.011530	0.011530	0.011598	0.011530	0.011564	0.011597	0.011833
0.011933	0.011866	0.011934	0.011701	0.011934	0.011564	0.011530
0.011567	0.011788	0.011701	0.011472	0.011943	0.011435	0.011493
0.011909	0.011842	0.011731	0.011933	0.011607	0.011799	0.011497
0.011631	0.011866	0.011735	0.011555	0.011605	0.011597	0.011669
0.011765	0.011667	0.011804	0.011805	0.011799	0.011866	0.011975
0.011703	0.011807	0.011805	0.011737	0.012002	0.011463	0.011667
0.011564	0.011564	0.011463	0.011463	0.011463	0.011530	0.011597
0.011463	0.011530	0.011429	0.011597	0.011396	0.011530	0.011597
0.011530	0.011967	0.011866	0.011900	0.011429	0.011530	0.011564
0.011564	0.011530	0.011597	0.011631	0.011496	0.011496	0.011496
0.012034	0.011664	0.011664	0.011463	0.011564	0.011664	0.011497
0.011496	0.011564	0.011530	0.011631	0.011496	0.011631	0.011698
0.011698	0.011530	0.011530	0.011463	0.011463	0.011631	0.011564
0.011463	0.011564	0.011429	0.011463	0.011597	0.011732	0.011497
0.011463	0.011597	0.011531	0.011429	0.011765	0.011429	0.011597
0.011608	0.011497	0.011799	0.011631	0.011664	0.011530	0.011631
0.011496	0.011765	0.011429	0.011698	0.011496	0.011496	0.011429

0.011597	0.011866	0.011564	0.011631	0.011463	0.011866	0.011496
0.011497	0.011496	0.011530	0.011564	0.011530	0.011631	0.011698
0.011698	0.011664	0.011564	0.011665	0.011564	0.011496	0.011463
0.011496	0.011496	0.011564	0.011496	0.011396	0.011631	0.011496
0.011530	0.011530	0.011664	0.011698	0.011597	0.011463	0.011530
0.011597	0.011530	0.011631	0.011496	0.011631	0.011732	0.012034
0.011530	0.011933	0.011474	0.018050	0.011453	0.011733	0.011898
0.011856	0.011905	0.011636	0.011582	0.012050	0.011924	0.011729
0.011724	0.011976	0.011450	0.011395	0.011529	0.011653	0.011491
0.011764	0.011656	0.011691	0.011523	0.011664	0.011698	0.011703
0.011507	0.011665	0.011665	0.011631	0.011597	0.011598	0.011564
0.011597	0.011530	0.011564	0.011598	0.011564	0.011497	0.011530
0.011530	0.011598	0.011497	0.011598	0.011631	0.011665	0.011564
0.011597	0.011564	0.011900	0.011900	0.011564	0.011666	0.011698
0.011463	0.011463	0.011497	0.011530	0.011463	0.011698	0.011698
0.011732	0.011731	0.011664	0.011765	0.011934	0.011767	0.011530
0.011598	0.011665	0.011631	0.011530	0.011597	0.011564	0.011597
0.011665	0.011665	0.011698	0.011598	0.011498	0.011765	0.011665
0.011602	0.011497	0.011597	0.011698	0.011598	0.011732	0.011765
0.011933	0.011607	0.011799	0.011497	0.011631	0.011866	0.011497
0.011667	0.011564	0.011530	0.011530	0.011598	0.011497	0.011598
0.011631	0.011665	0.011564	0.011597	0.011564	0.011900	0.011900
0.011564	0.011666	0.011731	0.011664	0.012305	0.011703	0.011658
0.011728	0.011684	0.011798	0.011684	0.011795	0.011715	0.011681
0.012262	0.011579	0.011711	0.011648	0.011921	0.011774	0.011809
0.012284	0.011776	0.011838	0.011900	0.011765	0.011765	0.011804
0.011463	0.011564	0.011765	0.011731	0.011736	0.011664	0.011700
0.011564	0.011429	0.011463	0.011564	0.011396	0.011530	0.011429
0.011564	0.011597	0.011496	0.011463	0.011497	0.011496	0.011530
0.011530	0.011463	0.011530	0.011564	0.011463	0.011597	0.011698
0.011597	0.011664	0.011597	0.011597	0.011530	0.011933	0.011564
0.011617	0.011530	0.011732	0.011739	0.011564	0.011631	0.011631
0.011631	0.011497	0.011530	0.011497	0.011664	0.011664	0.011564
0.011597	0.011664	0.011900	0.011631	0.011833	0.011833	0.011698
0.011530	0.011597	0.011631	0.012034	0.011598	0.011564	0.011597
0.011631	0.011564	0.011564	0.011866	0.011732	0.011833	0.011564
0.011664	0.011732	0.011597	0.011664	0.011631	0.011497	0.011832
0.011597	0.011564	0.011564	0.011732	0.011564	0.011664	0.011900
0.011597	0.011943	0.011967	0.011711	0.011455	0.011672	0.011473
0.011500	0.011917	0.011517	0.011730	0.011548	0.011620	0.011591
0.011723	0.011324	0.011506	0.011390	0.011438	0.011663	0.011628
0.011625	0.011493	0.011659	0.011589	0.011600	0.011665	0.011732
0.011799	0.011665	0.011770	0.011772	0.011908	0.011944	0.011597
0.011765	0.011698	0.011564	0.011665	0.011463	0.011631	0.011497
0.011564	0.011564	0.011530	0.011497	0.011530	0.011497	0.011597
0.011584	0.011585	0.011574	0.011608	0.011745	0.011641	0.011709
0.011708	0.011632	0.011466	0.011456	0.011568	0.011738	0.011739
0.011531	0.011602	0.011835	0.011804	0.011733	0.011767	0.011698
0.011631	0.011698	0.011698	0.011597	0.012169	0.011765	0.011833
0.011866	0.011950	0.011572	0.011649	0.011530	0.011732	0.011698
0.011733	0.011765	0.011765	0.011765	0.011847	0.011900	0.011546
0.011432	0.011441	0.011537	0.011502	0.011607	0.011471	0.011774
0.011575	0.011718	0.011489	0.011585	0.011565	0.011574	0.011463
0.011497	0.011497	0.011497	0.011530	0.011732	0.011698	0.011631
0.011732	0.011799	0.011530	0.011530	0.011564	0.011564	0.011567
0.011635	0.011564	0.011564	0.011600	0.011665	0.011732	0.011799
0.011665	0.011770	0.011772	0.011908	0.011944	0.011597	0.011765
0.011698	0.011698	0.011534	0.011687	0.011651	0.011639	0.011551
0.011646	0.011926	0.011568	0.011604	0.011430	0.011631	0.011497
0.011597	0.011598	0.011497	0.011599	0.011698	0.011702	0.011598

0.011497	0.011431	0.011564	0.011497	0.011430	0.011536	0.011631
0.011597	0.011530	0.011564	0.011598	0.011564	0.011497	0.011698
0.011564	0.011597	0.011597	0.011530	0.011597	0.011832	0.011496
0.011530	0.011933	0.011530	0.011496	0.011530	0.011900	0.011532
0.012016	0.011485	0.011511	0.011704	0.011752	0.011670	0.011436
0.011480	0.011698	0.011799	0.011841	0.011767	0.011838	0.011968
0.011868	0.011906	0.011866	0.012120	0.011900	0.012775	0.011936
0.012675	0.012443	0.012577	0.012781	0.011899	0.011598	0.011900
0.012339	0.012275	0.011866	0.011766	0.011834	0.011834	0.011804
0.011698	0.011705	0.011765	0.011733	0.012103	0.012169	0.012171
0.012070	0.012170	0.012274	0.012041	0.012340	0.012347	0.012407
0.012408	0.012442	0.012392	0.012408	0.012410	0.011631	0.011934
0.012002	0.012505	0.012010	0.012236	0.012710	0.011773	0.011665
0.011631	0.011631	0.012102	0.012130	0.012257	0.011967	0.011633
S097	0.011664	0.011664	0.012001	0.011765	0.011765	0.011832
0.011671	0.011603	0.011765	0.011262	0.011329	0.011296	0.011262
0.011295	0.011396	0.011262	0.011295	0.011362	0.011329	0.011295
0.011262	0.011295	0.011396	0.011262	0.011295	0.011295	0.011637
0.011530	0.011295	0.011194	0.011429	0.011362	0.011329	0.011362
0.011329	0.011429	0.011262	0.011329	0.011295	0.011329	0.011496
0.011262	0.011329	0.011295	0.011228	0.011396	0.011429	0.011362
0.011329	0.011429	0.011396	0.011664	0.011262	0.011597	0.011262
0.011295	0.011396	0.011396	0.011597	0.011698	0.011396	0.011496
0.011362	0.011429	0.011329	0.011563	0.011799	0.011262	0.011396
0.011295	0.011396	0.011362	0.011429	0.011362	0.011295	0.011295
0.011262	0.011295	0.011262	0.011362	0.011329	0.011363	0.011429
0.011362	0.011329	0.011396	0.011295	0.011429	0.011262	0.011329
0.011496	0.011429	0.011664	0.011496	0.011329	0.011564	0.000000
0.011505	0.011732	0.011551	0.011255	0.011247	0.011355	0.011454
0.012085	0.011518	0.011266	0.011563	0.011564	0.011362	0.011463
0.011396	0.011362	0.011429	0.011329	0.011429	0.011362	0.011362
0.011329	0.011429	0.011631	0.011429	0.011396	0.011362	0.011295
0.011262	0.011262	0.011329	0.011295	0.011497	0.011463	0.011396
0.011497	0.011564	0.011295	0.011295	0.011329	0.011329	0.011330
0.011398	0.011329	0.011329	0.011204	0.011277	0.011235	0.011329
0.011331	0.011266	0.011318	0.011302	0.011230	0.011266	0.011297
0.011564	0.011336	0.011370	0.011262	0.011268	0.011397	0.011363
0.011430	0.011363	0.011363	0.011463	0.011396	0.011765	0.011564
0.011597	0.011463	0.011329	0.011429	0.011228	0.011463	0.011262
0.011329	0.011329	0.011295	0.011262	0.011295	0.011262	0.011362
0.011329	0.011429	0.011362	0.011262	0.011296	0.011463	0.011466
0.011463	0.011362	0.011262	0.011195	0.011329	0.011262	0.011195
0.011298	0.011463	0.011262	0.011463	0.011228	0.011329	0.011295
0.011396	0.011295	0.011329	0.011295	0.011329	0.011195	0.011329
0.011362	0.011295	0.011362	0.011295	0.011329	0.011362	0.011597
0.011698	0.011631	0.011698	0.011397	0.011698	0.011329	0.011295
0.011297	0.011626	0.011426	0.011232	0.011637	0.011197	0.011257
0.011705	0.011604	0.011496	0.011698	0.011402	0.011564	0.011530
0.011463	0.011631	0.011486	0.011307	0.011395	0.011362	0.011432
0.011530	0.011431	0.011567	0.011635	0.011564	0.011631	0.011738
0.011466	0.011637	0.011568	0.011500	0.011766	0.011228	0.011431
0.011396	0.011329	0.011228	0.011228	0.011295	0.011228	0.011295
0.011295	0.011362	0.011262	0.011362	0.011295	0.011295	0.011362
0.011295	0.011731	0.011631	0.011664	0.011194	0.011295	0.011329
0.011329	0.011295	0.011362	0.011396	0.011262	0.011262	0.011262
0.011799	0.011429	0.011429	0.011228	0.011329	0.011497	0.011262
0.011262	0.011329	0.011295	0.011463	0.011262	0.011396	0.011530
0.011463	0.011295	0.011295	0.011228	0.011228	0.011396	0.011329
0.011228	0.011329	0.011195	0.011228	0.011362	0.011497	0.011228
0.011228	0.011362	0.011295	0.011195	0.011530	0.011195	0.011295

0.011437	0.011262	0.011564	0.011396	0.011429	0.011295	0.011329
0.011262	0.011597	0.011194	0.011463	0.011262	0.011262	0.011194
0.011429	0.011631	0.011329	0.011396	0.011228	0.011631	0.011262
0.011262	0.011262	0.011295	0.011396	0.011295	0.011396	0.011463
0.011463	0.011429	0.011262	0.011429	0.011396	0.011194	0.011228
0.011262	0.011194	0.011329	0.011262	0.011228	0.011396	0.011262
0.011295	0.011295	0.011429	0.011463	0.011362	0.011295	0.011295
0.011362	0.011295	0.011396	0.011262	0.011396	0.011429	0.011799
0.011295	0.011698	0.011302	0.017823	0.011322	0.011366	0.011653
0.011614	0.011658	0.011374	0.011292	0.011719	0.011681	0.011303
0.011534	0.011487	0.011216	0.011270	0.011294	0.011307	0.011275
0.011529	0.011470	0.011468	0.011325	0.011429	0.011463	0.011466
0.011335	0.011429	0.011429	0.011396	0.011362	0.011362	0.011329
0.011362	0.011295	0.011329	0.011362	0.011329	0.011262	0.011295
0.011295	0.011362	0.011262	0.011362	0.011396	0.011429	0.011329
0.011362	0.011329	0.011665	0.011665	0.011396	0.011363	0.011463
0.011228	0.011228	0.011262	0.011362	0.011228	0.011463	0.011463
0.011497	0.011496	0.011429	0.011530	0.011698	0.011532	0.011362
0.011362	0.011429	0.011463	0.011295	0.011362	0.011329	0.011362
0.011430	0.011430	0.011463	0.011430	0.011330	0.011530	0.011497
0.011365	0.011262	0.011362	0.011396	0.011362	0.011497	0.011530
0.011698	0.011402	0.011564	0.011530	0.011463	0.011631	0.011262
0.011431	0.011262	0.011295	0.011295	0.011362	0.011262	0.011362
0.011396	0.011429	0.011329	0.011362	0.011329	0.011665	0.011665
0.011396	0.011363	0.011496	0.011429	0.012069	0.011489	0.011412
0.011482	0.011441	0.011552	0.011441	0.011550	0.011474	0.011439
0.012021	0.011337	0.011471	0.011406	0.011680	0.011536	0.011638
0.012046	0.011512	0.011601	0.011664	0.011530	0.011530	0.011567
0.011228	0.011396	0.011530	0.011496	0.011499	0.011496	0.011464
0.011329	0.011262	0.011295	0.011329	0.011228	0.011295	0.011194
0.011329	0.011362	0.011262	0.011228	0.011329	0.011262	0.011295
0.011295	0.011228	0.011295	0.011329	0.011228	0.011429	0.011463
0.011362	0.011429	0.011362	0.011362	0.011295	0.011698	0.011329
0.011373	0.011295	0.011530	0.011535	0.011396	0.011329	0.011396
0.011396	0.011329	0.011295	0.011329	0.011362	0.011429	0.011362
0.011362	0.011496	0.011664	0.011396	0.011597	0.011597	0.011463
0.011295	0.011362	0.011396	0.011799	0.011362	0.011396	0.011362
0.011396	0.011329	0.011329	0.011631	0.011564	0.011597	0.011329
0.011429	0.011496	0.011362	0.011429	0.011396	0.011262	0.011597
0.011362	0.011329	0.011329	0.011496	0.011329	0.011429	0.011664
0.011362	0.011739	0.011731	0.011548	0.011342	0.011434	0.011318
0.011170	0.011761	0.011271	0.011507	0.011353	0.011366	0.011432
0.011519	0.011192	0.011394	0.011258	0.011330	0.011462	0.011425
0.011437	0.011336	0.011412	0.011342	0.011363	0.011497	0.011564
0.011564	0.011430	0.011533	0.011535	0.011670	0.011696	0.011362
0.011597	0.011463	0.011329	0.011429	0.011228	0.011463	0.011262
0.011329	0.011329	0.011295	0.011262	0.011295	0.011262	0.011362
0.011340	0.011340	0.011334	0.011368	0.011505	0.011470	0.011470
0.011469	0.011396	0.011208	0.011204	0.011263	0.011501	0.011501
0.011296	0.011365	0.011599	0.011567	0.011497	0.011464	0.011463
0.011396	0.011463	0.011463	0.011362	0.011933	0.011530	0.011597
0.011631	0.011701	0.011333	0.011407	0.011295	0.011497	0.011463
0.011497	0.011530	0.011530	0.011530	0.011650	0.011664	0.011303
0.011195	0.011199	0.011298	0.011264	0.011368	0.011209	0.011536
0.011335	0.011499	0.011203	0.011360	0.011329	0.011402	0.011295
0.011262	0.011262	0.011329	0.011295	0.011497	0.011463	0.011396
0.011497	0.011564	0.011295	0.011295	0.011329	0.011329	0.011330
0.011398	0.011329	0.011329	0.011363	0.011497	0.011564	0.011564
0.011430	0.011533	0.011535	0.011670	0.011696	0.011362	0.011530
0.011463	0.011463	0.011229	0.011478	0.011338	0.011435	0.011270

0.011439	0.011653	0.011399	0.011366	0.011262	0.011463	0.011329
0.011429	0.011362	0.011262	0.011296	0.011463	0.011466	0.011362
0.011262	0.011195	0.011329	0.011262	0.011195	0.011298	0.011463
0.011362	0.011295	0.011329	0.011362	0.011329	0.011262	0.011463
0.011329	0.011362	0.011362	0.011295	0.011362	0.011597	0.011262
0.011295	0.011698	0.011295	0.011329	0.011295	0.011664	0.011232
0.011735	0.011273	0.011311	0.011503	0.011589	0.011491	0.011186
0.011270	0.011463	0.011564	0.011671	0.011531	0.011669	0.011800
0.011699	0.011737	0.011698	0.011949	0.011664	0.012539	0.011700
0.012439	0.012207	0.012341	0.012612	0.011664	0.011362	0.011665
0.012103	0.012038	0.011631	0.011530	0.011598	0.011598	0.011567
0.011463	0.011467	0.011530	0.011497	0.011867	0.011933	0.011935
0.011834	0.011934	0.012038	0.011804	0.012104	0.012110	0.012171
0.012171	0.012205	0.012152	0.012172	0.012173	0.011396	0.011699
0.011766	0.012270	0.011772	0.012001	0.012474	0.011536	0.011430
0.011396	0.011396	0.011867	0.011924	0.012010	0.011732	0.011397
S098	0.011616	0.011616	0.011988	0.011950	0.011950	0.011988
0.011806	0.011732	0.011913	0.011431	0.011468	0.011431	0.011394
0.011431	0.011653	0.011431	0.011468	0.011468	0.011468	0.011431
0.011431	0.011468	0.011579	0.011394	0.011468	0.011431	0.011806
0.011728	0.011468	0.011357	0.011616	0.011505	0.011505	0.011468
0.011468	0.011616	0.011505	0.011505	0.011505	0.011357	0.011691
0.011431	0.011505	0.011505	0.011468	0.011579	0.011616	0.011542
0.011505	0.011654	0.011506	0.011876	0.011468	0.011765	0.011468
0.011468	0.011580	0.011542	0.011765	0.011913	0.011579	0.011691
0.011542	0.011579	0.011468	0.011653	0.011988	0.011505	0.011579
0.011468	0.011542	0.011542	0.011579	0.011468	0.011505	0.011505
0.011431	0.011431	0.011394	0.011468	0.011505	0.011505	0.011616
0.011542	0.011431	0.011579	0.011357	0.011616	0.011431	0.011468
0.011653	0.011616	0.011839	0.011505	0.011468	0.011728	0.011505
0.000000	0.011845	0.011678	0.011376	0.011337	0.011255	0.011533
0.012221	0.011636	0.011311	0.011728	0.011728	0.011505	0.011579
0.011542	0.011542	0.011616	0.011505	0.011542	0.011505	0.011468
0.011431	0.011542	0.011802	0.011653	0.011542	0.011542	0.011468
0.011394	0.011431	0.011505	0.011394	0.011691	0.011653	0.011579
0.011691	0.011765	0.011468	0.011468	0.011505	0.011505	0.011506
0.011580	0.011505	0.011505	0.011331	0.011415	0.011410	0.011468
0.011322	0.011434	0.011496	0.011471	0.011398	0.011439	0.011470
0.011725	0.011441	0.011477	0.011431	0.011397	0.011542	0.011542
0.011617	0.011543	0.011542	0.011579	0.011542	0.011876	0.011728
0.011765	0.011654	0.011468	0.011616	0.011394	0.011653	0.011394
0.011505	0.011505	0.011431	0.011431	0.011431	0.011431	0.011542
0.011468	0.011616	0.011542	0.011357	0.011544	0.011654	0.011620
0.011654	0.011542	0.011431	0.011358	0.011505	0.011431	0.011357
0.011472	0.011654	0.011431	0.011654	0.011394	0.011468	0.011468
0.011579	0.011431	0.011505	0.011468	0.011505	0.011357	0.011468
0.011505	0.011468	0.011505	0.011431	0.011468	0.011542	0.011728
0.011913	0.011765	0.011913	0.011616	0.011914	0.011505	0.011468
0.011470	0.011736	0.011628	0.011399	0.011923	0.011362	0.011410
0.011918	0.011810	0.011653	0.011876	0.011513	0.011765	0.011654
0.011616	0.011802	0.011687	0.011511	0.011429	0.011542	0.011621
0.011728	0.011580	0.011765	0.011805	0.011542	0.011802	0.011919
0.011582	0.011810	0.011659	0.011546	0.011951	0.011394	0.011544
0.011542	0.011468	0.011357	0.011357	0.011431	0.011468	0.011542
0.011468	0.011542	0.011431	0.011505	0.011468	0.011468	0.011542
0.011468	0.011950	0.011839	0.011876	0.011357	0.011468	0.011505
0.011505	0.011468	0.011542	0.011579	0.011394	0.011394	0.011394
0.011988	0.011579	0.011616	0.011394	0.011505	0.011654	0.011394
0.011394	0.011431	0.011468	0.011616	0.011431	0.011579	0.011728
0.011653	0.011468	0.011468	0.011394	0.011394	0.011505	0.011468

0.011394	0.011505	0.011357	0.011394	0.011394	0.011691	0.011394
0.011394	0.011394	0.011394	0.011357	0.011728	0.011357	0.011542
0.011628	0.011431	0.011765	0.011542	0.011542	0.011431	0.011542
0.011431	0.011765	0.011357	0.011616	0.011431	0.011431	0.011357
0.011542	0.011839	0.011468	0.011579	0.011394	0.011839	0.011431
0.011431	0.011431	0.011468	0.011542	0.011468	0.011579	0.011653
0.011505	0.011616	0.011505	0.011617	0.011579	0.011431	0.011394
0.011431	0.011431	0.011468	0.011431	0.011394	0.011542	0.011431
0.011468	0.011431	0.011542	0.011653	0.011505	0.011468	0.011468
0.011505	0.011468	0.011505	0.011394	0.011579	0.011691	0.011950
0.011431	0.011876	0.011431	0.018175	0.011257	0.011567	0.011765
0.011765	0.011765	0.011217	0.011420	0.011885	0.011802	0.011485
0.011696	0.011610	0.011333	0.011452	0.011387	0.011406	0.011452
0.011686	0.011612	0.011570	0.011342	0.011616	0.011616	0.011546
0.011475	0.011579	0.011579	0.011542	0.011505	0.011506	0.011505
0.011505	0.011431	0.011505	0.011542	0.011468	0.011431	0.011468
0.011468	0.011505	0.011357	0.011505	0.011579	0.011468	0.011505
0.011542	0.011505	0.011839	0.011839	0.011580	0.011617	0.011616
0.011394	0.011394	0.011394	0.011542	0.011394	0.011653	0.011653
0.011691	0.011653	0.011579	0.011728	0.011913	0.011693	0.011542
0.011542	0.011616	0.011653	0.011468	0.011468	0.011431	0.011542
0.011579	0.011579	0.011616	0.011616	0.011506	0.011653	0.011653
0.011547	0.011394	0.011505	0.011654	0.011505	0.011691	0.011691
0.011876	0.011513	0.011765	0.011654	0.011616	0.011802	0.011431
0.011618	0.011505	0.011468	0.011468	0.011505	0.011357	0.011505
0.011579	0.011468	0.011505	0.011542	0.011505	0.011839	0.011839
0.011580	0.011617	0.011616	0.011579	0.012211	0.011644	0.011564
0.011482	0.011627	0.011717	0.011557	0.011746	0.011666	0.011587
0.012154	0.011516	0.011624	0.011553	0.011853	0.011695	0.011807
0.012225	0.011683	0.011543	0.011542	0.011542	0.011579	0.011506
0.011394	0.011431	0.011579	0.011542	0.011543	0.011691	0.011653
0.011468	0.011431	0.011468	0.011468	0.011394	0.011431	0.011357
0.011431	0.011468	0.011431	0.011394	0.011505	0.011431	0.011468
0.011468	0.011394	0.011468	0.011505	0.011394	0.011616	0.011579
0.011431	0.011542	0.011394	0.011431	0.011468	0.011913	0.011505
0.011562	0.011431	0.011728	0.011735	0.011542	0.011505	0.011579
0.011579	0.011505	0.011468	0.011505	0.011616	0.011616	0.011505
0.011542	0.011654	0.011876	0.011505	0.011691	0.011691	0.011579
0.011431	0.011542	0.011579	0.011988	0.011543	0.011542	0.011542
0.011579	0.011431	0.011431	0.011839	0.011728	0.011802	0.011505
0.011579	0.011654	0.011505	0.011579	0.011505	0.011431	0.011765
0.011542	0.011468	0.011431	0.011579	0.011505	0.011579	0.011839
0.011468	0.011881	0.011950	0.011670	0.011289	0.011579	0.011380
0.011343	0.011905	0.011360	0.011552	0.011451	0.011468	0.011432
0.011525	0.011324	0.011394	0.011258	0.011330	0.011395	0.011548
0.011551	0.011419	0.011505	0.011401	0.011544	0.011691	0.011728
0.011728	0.011617	0.011693	0.011734	0.011808	0.011843	0.011468
0.011765	0.011654	0.011468	0.011616	0.011394	0.011653	0.011394
0.011505	0.011505	0.011431	0.011431	0.011431	0.011431	0.011542
0.011512	0.011523	0.011509	0.011476	0.011664	0.011622	0.011587
0.011587	0.011468	0.011341	0.011333	0.011509	0.011659	0.011622
0.011468	0.011546	0.011803	0.011693	0.011691	0.011654	0.011579
0.011579	0.011654	0.011653	0.011505	0.012174	0.011728	0.011765
0.011839	0.011846	0.011437	0.011595	0.011468	0.011653	0.011616
0.011580	0.011616	0.011691	0.011691	0.011539	0.011802	0.011481
0.011359	0.011365	0.011472	0.011435	0.011551	0.011308	0.011734
0.011513	0.011591	0.011323	0.011471	0.011505	0.011589	0.011468
0.011394	0.011431	0.011505	0.011394	0.011691	0.011653	0.011579
0.011691	0.011765	0.011468	0.011468	0.011505	0.011505	0.011506
0.011580	0.011505	0.011505	0.011544	0.011691	0.011728	0.011728

0.011617	0.011693	0.011734	0.011808	0.011843	0.011468	0.011690
0.011616	0.011616	0.011434	0.011636	0.011598	0.011550	0.011485
0.011592	0.011982	0.011631	0.011548	0.011431	0.011654	0.011468
0.011616	0.011542	0.011357	0.011544	0.011654	0.011620	0.011542
0.011431	0.011358	0.011505	0.011431	0.011357	0.011472	0.011654
0.011505	0.011431	0.011505	0.011542	0.011468	0.011431	0.011616
0.011505	0.011542	0.011542	0.011468	0.011394	0.011691	0.011431
0.011468	0.011913	0.011468	0.011505	0.011431	0.011876	0.011368
0.011859	0.011319	0.011473	0.011561	0.011638	0.011623	0.011317
0.011384	0.011653	0.011765	0.011885	0.011728	0.011877	0.012025
0.011914	0.011919	0.011913	0.012073	0.011802	0.012657	0.011913
0.012657	0.012285	0.012512	0.012696	0.011802	0.011505	0.011876
0.012249	0.012025	0.011839	0.011728	0.011802	0.011802	0.011770
0.011653	0.011656	0.011728	0.011691	0.012062	0.012099	0.012099
0.011951	0.012025	0.012211	0.011913	0.012249	0.012256	0.012361
0.012361	0.012323	0.012249	0.012286	0.012286	0.011542	0.011839
0.011951	0.012471	0.011920	0.012062	0.011877	0.011734	0.011616
0.011579	0.011579	0.011988	0.011999	0.012165	0.011913	0.011580
S099	0.011845	0.011845	0.011882	0.011845	0.011845	0.011882
0.011286	0.011210	0.011807	0.011658	0.011695	0.011658	0.011620
0.011583	0.011882	0.011583	0.011695	0.011695	0.011695	0.011658
0.011658	0.011695	0.011508	0.011621	0.011696	0.011658	0.011286
0.011658	0.011695	0.011583	0.011508	0.011733	0.011733	0.011695
0.011695	0.011845	0.011733	0.011733	0.011732	0.011732	0.011920
0.011583	0.011732	0.011733	0.011695	0.011733	0.011845	0.011770
0.011733	0.011882	0.011733	0.011359	0.011695	0.011994	0.011695
0.011695	0.011733	0.011770	0.011994	0.011471	0.011733	0.011807
0.011770	0.011807	0.011695	0.011957	0.011434	0.011733	0.011770
0.011695	0.011770	0.011770	0.011807	0.011695	0.011733	0.011733
0.011658	0.011658	0.011620	0.011620	0.011732	0.011732	0.011845
0.011770	0.011658	0.011807	0.011583	0.011546	0.011658	0.011695
0.011882	0.011845	0.011471	0.011882	0.011695	0.011957	0.011732
0.011845	0.000000	0.011917	0.011573	0.011617	0.011765	0.011493
0.012190	0.011479	0.011650	0.011957	0.011957	0.011658	0.011508
0.011770	0.011770	0.011845	0.011733	0.011770	0.011733	0.011695
0.011658	0.011695	0.012032	0.011845	0.011770	0.011770	0.011695
0.011620	0.011583	0.011733	0.011620	0.011621	0.011583	0.011508
0.011621	0.011695	0.011695	0.011695	0.011733	0.011732	0.011733
0.011809	0.011733	0.011733	0.011567	0.011617	0.011608	0.011695
0.011663	0.011625	0.011699	0.011663	0.011627	0.011632	0.011698
0.011573	0.011674	0.011671	0.011658	0.011589	0.011770	0.011695
0.011770	0.011772	0.011770	0.011807	0.011770	0.012107	0.011882
0.011695	0.011807	0.011695	0.011770	0.011620	0.011583	0.011621
0.011733	0.011733	0.011658	0.011658	0.011658	0.011658	0.011770
0.011695	0.011845	0.011695	0.011658	0.011773	0.011882	0.011849
0.011882	0.011770	0.011658	0.011586	0.011732	0.011658	0.011583
0.011588	0.011882	0.011658	0.011882	0.011620	0.011695	0.011695
0.011807	0.011658	0.011733	0.011695	0.011733	0.011583	0.011695
0.011658	0.011695	0.011733	0.011658	0.011695	0.011732	0.011957
0.011396	0.011247	0.011396	0.011845	0.011471	0.011732	0.011695
0.011660	0.011878	0.011783	0.011630	0.011400	0.011592	0.011809
0.011398	0.011664	0.011882	0.012107	0.011783	0.011957	0.011882
0.011845	0.012032	0.011929	0.011834	0.011712	0.011770	0.011852
0.011957	0.011809	0.011995	0.012035	0.011770	0.012032	0.012152
0.011811	0.012043	0.011814	0.011927	0.012183	0.011620	0.011735
0.011733	0.011695	0.011583	0.011583	0.011658	0.011695	0.011733
0.011695	0.011770	0.011658	0.011658	0.011695	0.011695	0.011770
0.011695	0.011434	0.011322	0.011359	0.011583	0.011695	0.011733
0.011733	0.011620	0.011770	0.011733	0.011620	0.011620	0.011620
0.011471	0.011733	0.011845	0.011620	0.011732	0.011882	0.011620

0.011620	0.011658	0.011695	0.011845	0.011658	0.011583	0.011733
0.011658	0.011695	0.011695	0.011620	0.011620	0.011733	0.011695
0.011620	0.011732	0.011583	0.011620	0.011770	0.011509	0.011621
0.011621	0.011770	0.011621	0.011583	0.011284	0.011583	0.011770
0.011861	0.011658	0.011247	0.011770	0.011770	0.011658	0.011770
0.011658	0.011994	0.011583	0.011546	0.011583	0.011583	0.011583
0.011770	0.011695	0.011695	0.011807	0.011620	0.011322	0.011583
0.011658	0.011658	0.011695	0.011770	0.011695	0.011807	0.011882
0.011882	0.011845	0.011732	0.011845	0.011807	0.011658	0.011620
0.011658	0.011658	0.011695	0.011583	0.011620	0.011770	0.011658
0.011620	0.011658	0.011770	0.011807	0.011733	0.011695	0.011695
0.011733	0.011695	0.011733	0.011620	0.011807	0.011919	0.011508
0.011658	0.011434	0.011695	0.018507	0.011388	0.011693	0.011284
0.011284	0.011284	0.011899	0.011659	0.011445	0.011322	0.011728
0.011652	0.011427	0.011568	0.011719	0.011596	0.011505	0.011669
0.011397	0.011537	0.011376	0.011489	0.011845	0.011845	0.011813
0.011706	0.011807	0.011807	0.011695	0.011733	0.011733	0.011733
0.011733	0.011658	0.011733	0.011770	0.011695	0.011658	0.011695
0.011695	0.011733	0.011583	0.011733	0.011807	0.011845	0.011732
0.011695	0.011658	0.011396	0.011396	0.011808	0.011846	0.011845
0.011620	0.011620	0.011620	0.011770	0.011620	0.011882	0.011882
0.011919	0.011882	0.011807	0.011957	0.011471	0.011960	0.011770
0.011770	0.011845	0.011882	0.011658	0.011695	0.011658	0.011770
0.011807	0.011807	0.011845	0.011845	0.011734	0.011882	0.011882
0.011777	0.011620	0.011733	0.011882	0.011733	0.011845	0.011919
0.012107	0.011783	0.011957	0.011882	0.011845	0.012032	0.011658
0.011847	0.011733	0.011695	0.011695	0.011733	0.011583	0.011733
0.011807	0.011845	0.011732	0.011695	0.011658	0.011396	0.011396
0.011808	0.011846	0.011845	0.011807	0.012370	0.011923	0.011802
0.011911	0.011859	0.011956	0.011792	0.011982	0.011900	0.011781
0.012315	0.011749	0.011856	0.011825	0.012088	0.011925	0.012039
0.012385	0.011856	0.011771	0.011770	0.011770	0.011508	0.011733
0.011620	0.011658	0.011807	0.011770	0.011771	0.011620	0.011546
0.011695	0.011658	0.011695	0.011695	0.011620	0.011658	0.011583
0.011658	0.011695	0.011658	0.011620	0.011733	0.011658	0.011695
0.011695	0.011620	0.011695	0.011733	0.011620	0.011845	0.011882
0.011733	0.011770	0.011770	0.011733	0.011695	0.011471	0.011732
0.011488	0.011658	0.011658	0.011664	0.011695	0.011658	0.011733
0.011807	0.011733	0.011695	0.011733	0.011845	0.011845	0.011733
0.011770	0.011882	0.011359	0.011732	0.011882	0.011882	0.011807
0.011658	0.011770	0.011807	0.011546	0.011771	0.011695	0.011770
0.011807	0.011583	0.011583	0.011957	0.011957	0.011920	0.011733
0.011807	0.011882	0.011733	0.011807	0.011733	0.011658	0.011247
0.011770	0.011695	0.011583	0.011807	0.011733	0.011807	0.011546
0.011695	0.011361	0.011434	0.011589	0.011518	0.011770	0.011491
0.011513	0.011784	0.011626	0.011596	0.011695	0.011417	0.011432
0.011577	0.011324	0.011506	0.011390	0.011384	0.011596	0.011840
0.011822	0.011743	0.011695	0.011747	0.011735	0.011620	0.011621
0.011583	0.011733	0.011847	0.011663	0.011926	0.011927	0.011695
0.011695	0.011807	0.011695	0.011770	0.011620	0.011583	0.011621
0.011733	0.011733	0.011658	0.011658	0.011658	0.011658	0.011770
0.011705	0.011720	0.011701	0.011670	0.011859	0.011815	0.011781
0.011780	0.011695	0.011586	0.011570	0.011700	0.011851	0.011815
0.011695	0.011738	0.012033	0.011922	0.011920	0.011883	0.011807
0.011807	0.011882	0.011882	0.011732	0.011733	0.011957	0.011995
0.012069	0.011337	0.011630	0.011830	0.011695	0.011882	0.011845
0.011809	0.011845	0.011920	0.011920	0.011848	0.012032	0.011715
0.011587	0.011597	0.011702	0.011665	0.011706	0.011601	0.011211
0.011745	0.011449	0.011696	0.011722	0.011733	0.011784	0.011695
0.011620	0.011583	0.011733	0.011620	0.011621	0.011583	0.011508

0.011621	0.011695	0.011695	0.011695	0.011733	0.011732	0.011733
0.011809	0.011733	0.011733	0.011735	0.011620	0.011621	0.011583
0.011733	0.011847	0.011663	0.011926	0.011927	0.011695	0.011919
0.011845	0.011845	0.011662	0.011872	0.011833	0.011781	0.011721
0.011826	0.012135	0.011870	0.011779	0.011658	0.011882	0.011695
0.011845	0.011695	0.011658	0.011773	0.011882	0.011849	0.011770
0.011658	0.011586	0.011732	0.011658	0.011583	0.011588	0.011882
0.011733	0.011658	0.011733	0.011770	0.011695	0.011658	0.011546
0.011695	0.011770	0.011770	0.011695	0.011770	0.011995	0.011583
0.011695	0.011471	0.011695	0.011733	0.011658	0.011359	0.011681
0.011916	0.011495	0.011473	0.011565	0.011601	0.011589	0.011449
0.011438	0.011546	0.011658	0.011778	0.011620	0.011771	0.011920
0.011808	0.011813	0.011807	0.011967	0.011695	0.012631	0.011808
0.012557	0.012257	0.012485	0.012671	0.011658	0.011620	0.011770
0.012370	0.011920	0.011733	0.011621	0.011695	0.011695	0.011663
0.011546	0.011548	0.011620	0.011583	0.011995	0.011995	0.012032
0.011845	0.011957	0.012144	0.011807	0.012408	0.012416	0.012520
0.012520	0.012483	0.012407	0.012370	0.012445	0.011770	0.011995
0.012107	0.012631	0.012077	0.011920	0.011733	0.011588	0.011471
0.011359	0.011434	0.011920	0.011962	0.012059	0.011770	0.011509
S100	0.011664	0.011664	0.012004	0.011966	0.011966	0.012004
0.011819	0.011743	0.011928	0.011475	0.011513	0.011475	0.011437
0.011475	0.011702	0.011475	0.011475	0.011513	0.011513	0.011475
0.011475	0.011513	0.011626	0.011438	0.011513	0.011437	0.011819
0.011739	0.011513	0.011400	0.011664	0.011551	0.011551	0.011437
0.011513	0.011664	0.011551	0.011551	0.011551	0.011551	0.011739
0.011437	0.011551	0.011551	0.011513	0.011626	0.011664	0.011588
0.011551	0.011664	0.011513	0.011890	0.011513	0.011777	0.011513
0.011513	0.011626	0.011626	0.011777	0.011928	0.011626	0.011739
0.011588	0.011664	0.011513	0.011777	0.011966	0.011551	0.011626
0.011513	0.011588	0.011588	0.011626	0.011513	0.011551	0.011551
0.011475	0.011475	0.011437	0.011551	0.011551	0.011513	0.011664
0.011588	0.011513	0.011626	0.011400	0.011664	0.011475	0.011475
0.011702	0.011664	0.011890	0.011664	0.011551	0.011777	0.011551
0.011678	0.011917	0.000000	0.011459	0.011446	0.011536	0.011658
0.011600	0.011772	0.011431	0.011777	0.011664	0.011551	0.011588
0.011588	0.011551	0.011664	0.011551	0.011626	0.011551	0.011513
0.011475	0.011588	0.011815	0.011739	0.011588	0.011589	0.011513
0.011475	0.011475	0.011551	0.011437	0.011739	0.011702	0.011626
0.011739	0.011815	0.011475	0.011475	0.011513	0.011513	0.011513
0.011589	0.011551	0.011551	0.011372	0.011411	0.011413	0.011513
0.011551	0.011475	0.011524	0.011513	0.011404	0.011442	0.011477
0.011690	0.011483	0.011514	0.011475	0.011437	0.011588	0.011589
0.011664	0.011588	0.011588	0.011626	0.011588	0.011966	0.011777
0.011777	0.011702	0.011513	0.011664	0.011437	0.011664	0.011438
0.011513	0.011513	0.011475	0.011475	0.011475	0.011475	0.011588
0.011513	0.011626	0.011551	0.011475	0.011590	0.011702	0.011629
0.011664	0.011588	0.011437	0.011401	0.011551	0.011437	0.011400
0.011517	0.011664	0.011475	0.011664	0.011400	0.011513	0.011475
0.011626	0.011475	0.011551	0.011475	0.011513	0.011400	0.011513
0.011551	0.011513	0.011551	0.011475	0.011513	0.011588	0.011739
0.011928	0.011777	0.011928	0.011664	0.011928	0.011551	0.011513
0.011553	0.011792	0.011685	0.011444	0.011938	0.011405	0.011586
0.011933	0.011784	0.011664	0.011891	0.011598	0.011777	0.011740
0.011702	0.011777	0.011659	0.011435	0.011432	0.011589	0.011631
0.011777	0.011627	0.011740	0.011894	0.011588	0.011853	0.011972
0.011629	0.011900	0.011707	0.011707	0.012005	0.011437	0.011590
0.011588	0.011513	0.011400	0.011400	0.011475	0.011513	0.011588
0.011513	0.011588	0.011475	0.011551	0.011513	0.011513	0.011513
0.011513	0.011966	0.011853	0.011890	0.011400	0.011513	0.011551

0.011551	0.011475	0.011588	0.011626	0.011475	0.011475	0.011475
0.012004	0.011626	0.011664	0.011437	0.011513	0.011740	0.011437
0.011437	0.011437	0.011513	0.011626	0.011475	0.011626	0.011739
0.011702	0.011513	0.011513	0.011437	0.011437	0.011513	0.011551
0.011437	0.011551	0.011400	0.011437	0.011588	0.011740	0.011475
0.011438	0.011588	0.011476	0.011400	0.011739	0.011400	0.011588
0.011677	0.011475	0.011777	0.011589	0.011664	0.011475	0.011588
0.011475	0.011777	0.011400	0.011664	0.011475	0.011475	0.011400
0.011588	0.011815	0.011513	0.011626	0.011437	0.011853	0.011475
0.011475	0.011475	0.011475	0.011626	0.011513	0.011626	0.011702
0.011664	0.011588	0.011551	0.011588	0.011626	0.011475	0.011437
0.011475	0.011475	0.011513	0.011437	0.011437	0.011588	0.011475
0.011513	0.011475	0.011588	0.011702	0.011551	0.011513	0.011513
0.011551	0.011475	0.011551	0.011475	0.011626	0.011702	0.011966
0.011513	0.011891	0.011513	0.018388	0.011519	0.011651	0.011815
0.011815	0.011815	0.011591	0.011467	0.012050	0.011853	0.011678
0.011725	0.011793	0.011419	0.011500	0.011387	0.011561	0.011506
0.011765	0.011683	0.011684	0.011552	0.011588	0.011664	0.011668
0.011521	0.011664	0.011626	0.011588	0.011551	0.011551	0.011551
0.011551	0.011475	0.011475	0.011588	0.011513	0.011475	0.011513
0.011513	0.011551	0.011437	0.011551	0.011626	0.011664	0.011551
0.011588	0.011551	0.011815	0.011815	0.011626	0.011665	0.011664
0.011400	0.011437	0.011437	0.011588	0.011437	0.011702	0.011702
0.011664	0.011664	0.011626	0.011702	0.011928	0.011704	0.011588
0.011551	0.011664	0.011702	0.011513	0.011513	0.011475	0.011588
0.011626	0.011626	0.011664	0.011588	0.011552	0.011664	0.011702
0.011594	0.011437	0.011588	0.011702	0.011513	0.011740	0.011739
0.011891	0.011598	0.011777	0.011740	0.011702	0.011777	0.011475
0.011666	0.011551	0.011513	0.011513	0.011551	0.011437	0.011551
0.011626	0.011664	0.011551	0.011588	0.011551	0.011815	0.011815
0.011626	0.011665	0.011664	0.011626	0.012269	0.011661	0.011613
0.011685	0.011603	0.011729	0.011605	0.011686	0.011715	0.011560
0.012174	0.011524	0.011634	0.011601	0.011868	0.011706	0.011896
0.012245	0.011740	0.011552	0.011551	0.011551	0.011626	0.011514
0.011437	0.011475	0.011588	0.011551	0.011551	0.011702	0.011702
0.011513	0.011475	0.011513	0.011513	0.011437	0.011475	0.011400
0.011551	0.011588	0.011475	0.011437	0.011551	0.011475	0.011513
0.011513	0.011437	0.011513	0.011551	0.011437	0.011664	0.011702
0.011551	0.011664	0.011588	0.011551	0.011437	0.011928	0.011551
0.011610	0.011475	0.011739	0.011747	0.011589	0.011551	0.011626
0.011626	0.011551	0.011513	0.011551	0.011664	0.011664	0.011551
0.011588	0.011702	0.011890	0.011551	0.011740	0.011740	0.011664
0.011475	0.011588	0.011626	0.011966	0.011589	0.011589	0.011588
0.011588	0.011513	0.011513	0.011815	0.011778	0.011777	0.011551
0.011626	0.011702	0.011551	0.011626	0.011513	0.011475	0.011777
0.011588	0.011513	0.011513	0.011664	0.011551	0.011664	0.011891
0.011513	0.011895	0.011966	0.011760	0.011399	0.011626	0.011473
0.011528	0.011947	0.011437	0.011686	0.011548	0.011620	0.011645
0.011724	0.011455	0.011617	0.011457	0.011546	0.011664	0.011640
0.011649	0.011542	0.011594	0.011523	0.011591	0.011702	0.011777
0.011739	0.011665	0.011783	0.011708	0.011858	0.011934	0.011513
0.011777	0.011702	0.011513	0.011664	0.011437	0.011664	0.011438
0.011513	0.011513	0.011475	0.011475	0.011475	0.011475	0.011588
0.011483	0.011525	0.011551	0.011480	0.011670	0.011627	0.011592
0.011630	0.011513	0.011342	0.011373	0.011551	0.011702	0.011665
0.011513	0.011588	0.011853	0.011740	0.011702	0.011664	0.011626
0.011588	0.011702	0.011702	0.011551	0.012193	0.011664	0.011778
0.011890	0.011860	0.011520	0.011604	0.011513	0.011702	0.011664
0.011627	0.011702	0.011702	0.011702	0.011853	0.011890	0.011526
0.011402	0.011408	0.011518	0.011480	0.011521	0.011394	0.011746

0.011559	0.011546	0.011273	0.011558	0.011551	0.011594	0.011513
0.011475	0.011475	0.011551	0.011437	0.011739	0.011702	0.011626
0.011739	0.011815	0.011475	0.011475	0.011513	0.011513	0.011513
0.011589	0.011551	0.011551	0.011591	0.011702	0.011777	0.011739
0.011665	0.011783	0.011708	0.011858	0.011934	0.011513	0.011739
0.011664	0.011664	0.011440	0.011645	0.011645	0.011597	0.011532
0.011524	0.011883	0.011787	0.011595	0.011475	0.011664	0.011513
0.011626	0.011551	0.011475	0.011590	0.011702	0.011629	0.011588
0.011437	0.011401	0.011551	0.011437	0.011400	0.011517	0.011664
0.011551	0.011475	0.011475	0.011588	0.011513	0.011475	0.011664
0.011551	0.011588	0.011588	0.011513	0.011588	0.011777	0.011437
0.011513	0.011928	0.011513	0.011513	0.011513	0.011890	0.011450
0.011847	0.011405	0.011562	0.011644	0.011720	0.011671	0.011398
0.011480	0.011702	0.011815	0.011938	0.011777	0.011929	0.012079
0.011967	0.011972	0.011928	0.012126	0.011815	0.012798	0.011966
0.012610	0.012420	0.012651	0.012839	0.011815	0.011551	0.011928
0.012307	0.012080	0.011890	0.011778	0.011853	0.011853	0.011820
0.011702	0.011704	0.011777	0.011740	0.012080	0.012117	0.012117
0.011966	0.011966	0.012193	0.011966	0.012308	0.012277	0.012421
0.012383	0.012383	0.012307	0.012345	0.012346	0.011588	0.011891
0.011928	0.012458	0.011973	0.012117	0.011966	0.011744	0.011664
0.011626	0.011626	0.012042	0.012113	0.012176	0.011928	0.011626
S101	0.011373	0.011373	0.011685	0.011646	0.011646	0.011685
0.011532	0.011453	0.011607	0.011177	0.011217	0.011177	0.011138
0.011177	0.011412	0.011177	0.011177	0.011216	0.011216	0.011177
0.011177	0.011216	0.011334	0.011139	0.011217	0.011138	0.011532
0.011490	0.011216	0.011099	0.011334	0.011216	0.011255	0.011177
0.011216	0.011334	0.011256	0.011255	0.011295	0.011255	0.011451
0.011138	0.011255	0.011295	0.011216	0.011334	0.011373	0.011295
0.011256	0.011451	0.011256	0.011568	0.011255	0.011451	0.011255
0.011216	0.011334	0.011334	0.011451	0.011646	0.011295	0.011412
0.011295	0.011295	0.011216	0.011490	0.011646	0.011256	0.011295
0.011216	0.011295	0.011295	0.011334	0.011216	0.011256	0.011256
0.011177	0.011177	0.011138	0.011216	0.011216	0.011216	0.011373
0.011295	0.011177	0.011334	0.011099	0.011373	0.011177	0.011138
0.011412	0.011334	0.011607	0.011334	0.011216	0.011451	0.011255
0.011376	0.011573	0.011459	0.000000	0.011141	0.011183	0.011346
0.011343	0.011427	0.011147	0.011451	0.011373	0.011255	0.011295
0.011295	0.011255	0.011334	0.011256	0.011295	0.011256	0.011177
0.011138	0.011295	0.011490	0.011412	0.011295	0.011295	0.011216
0.011138	0.011177	0.011255	0.011138	0.011451	0.011373	0.011334
0.011412	0.011490	0.011177	0.011177	0.011217	0.011216	0.011217
0.011256	0.011255	0.011255	0.011101	0.011142	0.011142	0.011216
0.011216	0.011177	0.011222	0.011216	0.011139	0.011179	0.011217
0.011388	0.011220	0.011219	0.011177	0.011099	0.011295	0.011256
0.011334	0.011295	0.011295	0.011295	0.011295	0.011607	0.011451
0.011529	0.011412	0.011216	0.011373	0.011138	0.011412	0.011138
0.011216	0.011216	0.011177	0.011177	0.011177	0.011177	0.011295
0.011216	0.011334	0.011255	0.011177	0.011295	0.011412	0.011335
0.011373	0.011295	0.011138	0.011100	0.011255	0.011138	0.011099
0.011218	0.011373	0.011177	0.011373	0.011099	0.011216	0.011138
0.011334	0.011138	0.011255	0.011177	0.011216	0.011099	0.011216
0.011255	0.011216	0.011255	0.011177	0.011177	0.011255	0.011451
0.011607	0.011490	0.011607	0.011412	0.011646	0.011216	0.011177
0.011256	0.011440	0.011326	0.011140	0.011653	0.011100	0.011197
0.011650	0.011454	0.011334	0.011568	0.011339	0.011490	0.011373
0.011334	0.011529	0.011352	0.011250	0.011206	0.011295	0.011297
0.011490	0.011296	0.011493	0.011572	0.011255	0.011529	0.011652
0.011296	0.011574	0.011375	0.011454	0.011647	0.011138	0.011295
0.011295	0.011216	0.011099	0.011099	0.011177	0.011216	0.011256

0.011216	0.011295	0.011177	0.011216	0.011216	0.011216	0.011255
0.011216	0.011685	0.011529	0.011529	0.011099	0.011216	0.011256
0.011255	0.011177	0.011295	0.011334	0.011138	0.011138	0.011138
0.011724	0.011334	0.011373	0.011138	0.011216	0.011373	0.011138
0.011138	0.011138	0.011216	0.011412	0.011177	0.011334	0.011490
0.011412	0.011216	0.011216	0.011138	0.011138	0.011216	0.011216
0.011138	0.011255	0.011099	0.011138	0.011295	0.011412	0.011138
0.011139	0.011295	0.011139	0.011099	0.011451	0.011099	0.011295
0.011380	0.011177	0.011490	0.011295	0.011373	0.011177	0.011334
0.011177	0.011451	0.011099	0.011295	0.011177	0.011177	0.011099
0.011295	0.011490	0.011216	0.011334	0.011138	0.011529	0.011177
0.011177	0.011177	0.011216	0.011255	0.011216	0.011334	0.011373
0.011334	0.011334	0.011255	0.011373	0.011334	0.011177	0.011138
0.011177	0.011177	0.011216	0.011138	0.011138	0.011295	0.011177
0.011216	0.011177	0.011295	0.011373	0.011216	0.011216	0.011217
0.011256	0.011177	0.011255	0.011138	0.011295	0.011451	0.011685
0.011177	0.011607	0.011138	0.018410	0.011191	0.011378	0.011529
0.011490	0.011529	0.011277	0.011244	0.011665	0.011568	0.011303
0.011455	0.011427	0.011101	0.011151	0.011187	0.011208	0.011191
0.011446	0.011386	0.011383	0.011137	0.011334	0.011373	0.011296
0.011221	0.011334	0.011334	0.011255	0.011256	0.011256	0.011256
0.011256	0.011138	0.011216	0.011295	0.011216	0.011177	0.011177
0.011177	0.011255	0.011099	0.011256	0.011334	0.011373	0.011217
0.011295	0.011216	0.011529	0.011529	0.011295	0.011373	0.011412
0.011138	0.011099	0.011138	0.011295	0.011099	0.011373	0.011373
0.011412	0.011334	0.011334	0.011451	0.011646	0.011452	0.011295
0.011295	0.011373	0.011412	0.011216	0.011216	0.011177	0.011295
0.011334	0.011334	0.011373	0.011334	0.011256	0.011412	0.011412
0.011297	0.011138	0.011255	0.011373	0.011216	0.011412	0.011451
0.011568	0.011339	0.011490	0.011373	0.011334	0.011529	0.011138
0.011374	0.011256	0.011177	0.011177	0.011255	0.011099	0.011256
0.011334	0.011373	0.011217	0.011295	0.011216	0.011529	0.011529
0.011295	0.011373	0.011373	0.011334	0.011920	0.011392	0.011266
0.011428	0.011341	0.011431	0.011303	0.011466	0.011383	0.011336
0.011862	0.011222	0.011340	0.011303	0.011583	0.011457	0.011575
0.011891	0.011461	0.011256	0.011255	0.011255	0.011334	0.011217
0.011138	0.011177	0.011295	0.011255	0.011256	0.011451	0.011373
0.011216	0.011177	0.011216	0.011177	0.011138	0.011177	0.011099
0.011255	0.011295	0.011138	0.011138	0.011256	0.011177	0.011216
0.011216	0.011138	0.011216	0.011256	0.011138	0.011334	0.011412
0.011255	0.011373	0.011295	0.011255	0.011177	0.011646	0.011255
0.011305	0.011177	0.011490	0.011495	0.011295	0.011216	0.011295
0.011295	0.011256	0.011216	0.011256	0.011373	0.011334	0.011255
0.011295	0.011412	0.011607	0.011216	0.011334	0.011334	0.011334
0.011177	0.011295	0.011295	0.011724	0.011295	0.011295	0.011295
0.011334	0.011177	0.011177	0.011490	0.011490	0.011451	0.011255
0.011334	0.011412	0.011216	0.011334	0.011216	0.011177	0.011490
0.011295	0.011216	0.011177	0.011334	0.011255	0.011334	0.011568
0.011216	0.011610	0.011646	0.011440	0.011177	0.011295	0.011222
0.011174	0.011638	0.011139	0.011332	0.011209	0.011319	0.011326
0.011428	0.011126	0.011283	0.011126	0.011168	0.011328	0.011318
0.011342	0.011229	0.011295	0.011220	0.011296	0.011451	0.011490
0.011451	0.011373	0.011452	0.011376	0.011532	0.011596	0.011216
0.011529	0.011412	0.011216	0.011373	0.011138	0.011412	0.011138
0.011216	0.011216	0.011177	0.011177	0.011177	0.011177	0.011295
0.011257	0.011261	0.011256	0.011179	0.011415	0.011373	0.011336
0.011335	0.011216	0.011102	0.011101	0.011256	0.011412	0.011373
0.011216	0.011295	0.011568	0.011412	0.011412	0.011373	0.011334
0.011334	0.011373	0.011373	0.011216	0.011881	0.011373	0.011529
0.011607	0.011560	0.011219	0.011342	0.011216	0.011373	0.011373

0.011295	0.011334	0.011451	0.011451	0.011426	0.011529	0.011222
0.011100	0.011101	0.011219	0.011179	0.011260	0.011104	0.011456
0.011260	0.011415	0.011116	0.011278	0.011177	0.011258	0.011216
0.011138	0.011177	0.011255	0.011138	0.011451	0.011373	0.011334
0.011412	0.011490	0.011177	0.011177	0.011217	0.011216	0.011217
0.011256	0.011255	0.011255	0.011296	0.011451	0.011490	0.011451
0.011373	0.011452	0.011376	0.011532	0.011596	0.011216	0.011451
0.011373	0.011373	0.011178	0.011424	0.011342	0.011298	0.011223
0.011341	0.011592	0.011407	0.011298	0.011177	0.011373	0.011216
0.011334	0.011255	0.011177	0.011295	0.011412	0.011335	0.011295
0.011138	0.011100	0.011255	0.011138	0.011099	0.011218	0.011373
0.011256	0.011138	0.011216	0.011295	0.011216	0.011177	0.011373
0.011216	0.011295	0.011295	0.011216	0.011295	0.011490	0.011138
0.011216	0.011646	0.011216	0.011216	0.011177	0.011568	0.011150
0.011592	0.011151	0.011215	0.011407	0.011449	0.011416	0.011104
0.011168	0.011373	0.011490	0.011574	0.011451	0.011608	0.011763
0.011647	0.011611	0.011607	0.011848	0.011490	0.012468	0.011646
0.012312	0.012076	0.012315	0.012508	0.011568	0.011216	0.011607
0.011960	0.011764	0.011529	0.011451	0.011490	0.011490	0.011493
0.011373	0.011374	0.011451	0.011412	0.011803	0.011802	0.011881
0.011646	0.011763	0.011920	0.011607	0.011960	0.011966	0.012077
0.012038	0.012038	0.011960	0.011999	0.011999	0.011295	0.011529
0.011646	0.012116	0.011611	0.011803	0.011646	0.011453	0.011373
0.011334	0.011334	0.011724	0.011871	0.011937	0.011685	0.011334
S102	0.011620	0.011620	0.011695	0.011620	0.011620	0.011695
0.011589	0.011476	0.011657	0.011172	0.011210	0.011172	0.011172
0.011209	0.011359	0.011172	0.011172	0.011247	0.011247	0.011172
0.011172	0.011210	0.011321	0.011172	0.011210	0.011135	0.011551
0.011433	0.011210	0.011098	0.011359	0.011247	0.011210	0.011135
0.011247	0.011321	0.011247	0.011247	0.011284	0.011247	0.011433
0.011135	0.011247	0.011284	0.011210	0.011321	0.011359	0.011284
0.011247	0.011396	0.011247	0.011583	0.011247	0.011508	0.011247
0.011210	0.011322	0.011359	0.011508	0.011620	0.011321	0.011433
0.011284	0.011284	0.011210	0.011433	0.011657	0.011247	0.011321
0.011210	0.011247	0.011284	0.011321	0.011210	0.011284	0.011284
0.011172	0.011172	0.011135	0.011247	0.011247	0.011209	0.011321
0.011284	0.011247	0.011321	0.011172	0.011359	0.011172	0.011172
0.011359	0.011359	0.011583	0.011359	0.011247	0.011508	0.011247
0.011337	0.011617	0.011446	0.011141	0.000000	0.011219	0.011342
0.011324	0.011416	0.011144	0.011433	0.011359	0.011247	0.011321
0.011321	0.011247	0.011359	0.011247	0.011321	0.011247	0.011284
0.011247	0.011284	0.011471	0.011433	0.011321	0.011284	0.011210
0.011172	0.011172	0.011247	0.011172	0.011433	0.011396	0.011321
0.011433	0.011508	0.011135	0.011135	0.011172	0.011172	0.011172
0.011247	0.011247	0.011247	0.011099	0.011137	0.011138	0.011247
0.011247	0.011172	0.011213	0.011210	0.011136	0.011174	0.011210
0.011411	0.011212	0.011247	0.011172	0.011172	0.011321	0.011284
0.011359	0.011247	0.011247	0.011321	0.011321	0.011695	0.011471
0.011545	0.011396	0.011209	0.011359	0.011135	0.011359	0.011098
0.011210	0.011172	0.011172	0.011172	0.011172	0.011172	0.011284
0.011210	0.011321	0.011247	0.011172	0.011248	0.011396	0.011359
0.011359	0.011284	0.011098	0.011098	0.011247	0.011098	0.011098
0.011173	0.011359	0.011172	0.011359	0.011098	0.011210	0.011172
0.011321	0.011209	0.011247	0.011172	0.011210	0.011098	0.011210
0.011247	0.011209	0.011284	0.011210	0.011247	0.011284	0.011433
0.011620	0.011471	0.011620	0.011396	0.011620	0.011210	0.011209
0.011248	0.011555	0.011314	0.011136	0.011627	0.011098	0.011233
0.011589	0.011474	0.011359	0.011545	0.011326	0.011471	0.011471
0.011396	0.011471	0.011378	0.011201	0.011246	0.011284	0.011324
0.011471	0.011360	0.011436	0.011512	0.011321	0.011583	0.011701

0.011360	0.011513	0.011474	0.011436	0.011695	0.011135	0.011322
0.011247	0.011210	0.011098	0.011098	0.011210	0.011210	0.011284
0.011210	0.011284	0.011172	0.011247	0.011210	0.011209	0.011210
0.011210	0.011620	0.011545	0.011583	0.011098	0.011210	0.011247
0.011247	0.011172	0.011284	0.011321	0.011172	0.011172	0.011172
0.011695	0.011321	0.011359	0.011135	0.011210	0.011359	0.011135
0.011135	0.011172	0.011210	0.011359	0.011172	0.011321	0.011433
0.011396	0.011210	0.011209	0.011135	0.011135	0.011210	0.011247
0.011135	0.011247	0.011098	0.011135	0.011284	0.011434	0.011172
0.011135	0.011284	0.011210	0.011098	0.011433	0.011098	0.011284
0.011365	0.011172	0.011471	0.011284	0.011359	0.011210	0.011321
0.011172	0.011508	0.011098	0.011321	0.011172	0.011172	0.011098
0.011284	0.011508	0.011210	0.011321	0.011135	0.011545	0.011172
0.011172	0.011172	0.011172	0.011247	0.011210	0.011321	0.011396
0.011359	0.011284	0.011247	0.011359	0.011321	0.011172	0.011135
0.011172	0.011172	0.011210	0.011135	0.011135	0.011284	0.011172
0.011210	0.011172	0.011284	0.011396	0.011247	0.011210	0.011210
0.011247	0.011172	0.011284	0.011172	0.011321	0.011396	0.011657
0.011210	0.011583	0.011210	0.017989	0.011191	0.011389	0.011513
0.011513	0.011513	0.011273	0.011176	0.011675	0.011589	0.011307
0.011440	0.011434	0.011101	0.011194	0.011183	0.011213	0.011194
0.011436	0.011364	0.011365	0.011129	0.011284	0.011396	0.011399
0.011213	0.011359	0.011321	0.011284	0.011247	0.011247	0.011247
0.011284	0.011209	0.011172	0.011284	0.011210	0.011172	0.011210
0.011210	0.011247	0.011135	0.011247	0.011321	0.011359	0.011247
0.011284	0.011247	0.011508	0.011508	0.011322	0.011359	0.011396
0.011098	0.011135	0.011135	0.011284	0.011135	0.011359	0.011359
0.011321	0.011359	0.011321	0.011359	0.011620	0.011360	0.011284
0.011247	0.011321	0.011396	0.011210	0.011210	0.011172	0.011284
0.011284	0.011284	0.011321	0.011247	0.011247	0.011359	0.011433
0.011286	0.011135	0.011284	0.011396	0.011210	0.011396	0.011433
0.011545	0.011326	0.011471	0.011471	0.011396	0.011471	0.011172
0.011360	0.011247	0.011210	0.011210	0.011247	0.011135	0.011247
0.011321	0.011359	0.011247	0.011284	0.011247	0.011508	0.011508
0.011322	0.011359	0.011396	0.011359	0.011994	0.011380	0.011296
0.011411	0.011331	0.011452	0.011292	0.011412	0.011406	0.011326
0.011978	0.011253	0.011404	0.011291	0.011635	0.011439	0.011514
0.011967	0.011442	0.011210	0.011210	0.011209	0.011321	0.011172
0.011135	0.011172	0.011247	0.011210	0.011210	0.011359	0.011396
0.011210	0.011172	0.011210	0.011210	0.011135	0.011172	0.011098
0.011247	0.011284	0.011172	0.011135	0.011247	0.011172	0.011210
0.011210	0.011135	0.011210	0.011247	0.011135	0.011321	0.011396
0.011247	0.011359	0.011284	0.011247	0.011135	0.011620	0.011247
0.011293	0.011135	0.011433	0.011437	0.011322	0.011247	0.011321
0.011321	0.011247	0.011172	0.011247	0.011359	0.011359	0.011284
0.011284	0.011359	0.011583	0.011284	0.011433	0.011433	0.011359
0.011172	0.011284	0.011321	0.011657	0.011284	0.011322	0.011284
0.011247	0.011209	0.011209	0.011471	0.011471	0.011433	0.011210
0.011359	0.011396	0.011247	0.011359	0.011247	0.011172	0.011508
0.011284	0.011209	0.011209	0.011396	0.011210	0.011359	0.011620
0.011209	0.011665	0.011657	0.011430	0.011173	0.011284	0.011163
0.011178	0.011657	0.011135	0.011377	0.011207	0.011315	0.011326
0.011417	0.011126	0.011283	0.011126	0.011168	0.011328	0.011272
0.011341	0.011223	0.011289	0.011211	0.011285	0.011359	0.011471
0.011433	0.011359	0.011474	0.011399	0.011549	0.011608	0.011210
0.011545	0.011396	0.011209	0.011359	0.011135	0.011359	0.011098
0.011210	0.011172	0.011172	0.011172	0.011172	0.011172	0.011284
0.011212	0.011252	0.011247	0.011211	0.011437	0.011359	0.011361
0.011398	0.011210	0.011100	0.011099	0.011247	0.011433	0.011359
0.011172	0.011284	0.011545	0.011471	0.011396	0.011471	0.011396

0.011284	0.011396	0.011359	0.011247	0.011882	0.011321	0.011508
0.011583	0.011577	0.011212	0.011291	0.011210	0.011433	0.011396
0.011359	0.011396	0.011396	0.011396	0.011411	0.011620	0.011215
0.011098	0.011099	0.011212	0.011174	0.011250	0.011102	0.011438
0.011251	0.011399	0.011114	0.011269	0.011247	0.011324	0.011210
0.011172	0.011172	0.011247	0.011172	0.011433	0.011396	0.011321
0.011433	0.011508	0.011135	0.011135	0.011172	0.011172	0.011172
0.011247	0.011247	0.011247	0.011285	0.011359	0.011471	0.011433
0.011359	0.011474	0.011399	0.011549	0.011608	0.011210	0.011433
0.011396	0.011396	0.011173	0.011369	0.011330	0.011288	0.011216
0.011250	0.011563	0.011391	0.011287	0.011172	0.011359	0.011210
0.011321	0.011247	0.011172	0.011248	0.011396	0.011359	0.011284
0.011098	0.011098	0.011247	0.011098	0.011098	0.011173	0.011359
0.011284	0.011209	0.011172	0.011284	0.011210	0.011172	0.011359
0.011247	0.011284	0.011284	0.011210	0.011284	0.011433	0.011135
0.011210	0.011620	0.011210	0.011210	0.011209	0.011583	0.011146
0.011622	0.011146	0.011211	0.011390	0.011431	0.011400	0.011102
0.011165	0.011396	0.011508	0.011626	0.011471	0.011620	0.011769
0.011658	0.011699	0.011620	0.011925	0.011545	0.012480	0.011657
0.012293	0.012143	0.012334	0.012519	0.011545	0.011247	0.011620
0.012069	0.011807	0.011545	0.011471	0.011545	0.011545	0.011511
0.011396	0.011396	0.011471	0.011433	0.011695	0.011807	0.011769
0.011657	0.011732	0.011844	0.011658	0.011995	0.012038	0.012144
0.012107	0.012144	0.012069	0.012107	0.012107	0.011321	0.011583
0.011620	0.012143	0.011737	0.011844	0.011695	0.011436	0.011359
0.011321	0.011321	0.011732	0.011755	0.011897	0.011545	0.011321
S103	0.011686	0.011686	0.011797	0.011760	0.011760	0.011797
0.011694	0.011619	0.011760	0.011244	0.011318	0.011318	0.011281
0.011318	0.011502	0.011281	0.011281	0.011391	0.011355	0.011318
0.011281	0.011318	0.011391	0.011244	0.011318	0.011281	0.011694
0.011576	0.011281	0.011207	0.011428	0.011355	0.011355	0.011318
0.011355	0.011465	0.011355	0.011355	0.011355	0.011207	0.011539
0.011281	0.011318	0.011355	0.011318	0.011428	0.011428	0.011391
0.011355	0.011502	0.011392	0.011723	0.011318	0.011649	0.011318
0.011318	0.011428	0.011465	0.011649	0.011760	0.011428	0.011539
0.011391	0.011465	0.011318	0.011502	0.011797	0.011355	0.011428
0.011318	0.011391	0.011391	0.011428	0.011355	0.011355	0.011355
0.011281	0.011281	0.011244	0.011355	0.011355	0.011355	0.011465
0.011355	0.011355	0.011428	0.011281	0.011428	0.011281	0.011318
0.011502	0.011428	0.011649	0.011355	0.011355	0.011612	0.011355
0.011255	0.011765	0.011536	0.011183	0.011219	0.000000	0.011433
0.012108	0.011488	0.011234	0.011575	0.011576	0.011355	0.011465
0.011428	0.011391	0.011465	0.011355	0.011428	0.011355	0.011391
0.011355	0.011428	0.011649	0.011539	0.011428	0.011392	0.011318
0.011244	0.011281	0.011355	0.011281	0.011539	0.011502	0.011428
0.011539	0.011539	0.011318	0.011318	0.011355	0.011355	0.011355
0.011429	0.011355	0.011355	0.011175	0.011217	0.011214	0.011355
0.011209	0.011283	0.011258	0.011283	0.011246	0.011286	0.011319
0.011565	0.011287	0.011399	0.011281	0.011283	0.011428	0.011392
0.011465	0.011392	0.011391	0.011465	0.011428	0.011760	0.011539
0.011649	0.011465	0.011355	0.011465	0.011244	0.011502	0.011244
0.011355	0.011355	0.011281	0.011281	0.011281	0.011281	0.011391
0.011318	0.011465	0.011391	0.011207	0.011392	0.011391	0.011393
0.011502	0.011355	0.011281	0.011208	0.011355	0.011281	0.011208
0.011321	0.011502	0.011281	0.011502	0.011244	0.011318	0.011318
0.011428	0.011318	0.011355	0.011318	0.011355	0.011207	0.011318
0.011355	0.011318	0.011391	0.011318	0.011355	0.011391	0.011539
0.011760	0.011612	0.011723	0.011502	0.011760	0.011355	0.011318
0.011319	0.011589	0.011461	0.011246	0.011768	0.011210	0.011359
0.011768	0.011655	0.011539	0.011723	0.011434	0.011612	0.011539

0.011465	0.011649	0.011530	0.011379	0.011383	0.011392	0.011469
0.011539	0.011467	0.011617	0.011691	0.011465	0.011649	0.011804
0.011430	0.011693	0.011506	0.011394	0.011797	0.011244	0.011393
0.011391	0.011318	0.011207	0.011207	0.011281	0.011318	0.011391
0.011318	0.011355	0.011281	0.011391	0.011318	0.011318	0.011355
0.011318	0.011796	0.011649	0.011686	0.011207	0.011318	0.011355
0.011355	0.011318	0.011391	0.011428	0.011244	0.011244	0.011244
0.011870	0.011391	0.011428	0.011244	0.011355	0.011465	0.011244
0.011244	0.011318	0.011318	0.011502	0.011244	0.011428	0.011575
0.011502	0.011318	0.011318	0.011244	0.011244	0.011355	0.011355
0.011244	0.011355	0.011207	0.011244	0.011244	0.011539	0.011281
0.011244	0.011244	0.011318	0.011207	0.011539	0.011207	0.011391
0.011474	0.011281	0.011612	0.011392	0.011391	0.011281	0.011391
0.011281	0.011649	0.011207	0.011465	0.011281	0.011281	0.011207
0.011391	0.011686	0.011318	0.011391	0.011244	0.011649	0.011281
0.011244	0.011281	0.011318	0.011428	0.011318	0.011428	0.011502
0.011355	0.011465	0.011355	0.011465	0.011428	0.011281	0.011244
0.011281	0.011281	0.011318	0.011281	0.011244	0.011391	0.011281
0.011318	0.011281	0.011391	0.011502	0.011355	0.011318	0.011318
0.011391	0.011318	0.011428	0.011281	0.011428	0.011539	0.011760
0.011318	0.011723	0.011318	0.018162	0.011128	0.011514	0.011649
0.011649	0.011649	0.011115	0.011242	0.011754	0.011686	0.011382
0.011458	0.011513	0.011183	0.011325	0.011221	0.011267	0.011333
0.011552	0.011456	0.011493	0.011199	0.011465	0.011465	0.011468
0.011362	0.011465	0.011391	0.011428	0.011355	0.011355	0.011355
0.011391	0.011318	0.011355	0.011391	0.011318	0.011244	0.011318
0.011318	0.011355	0.011244	0.011355	0.011428	0.011318	0.011355
0.011391	0.011355	0.011686	0.011686	0.011391	0.011428	0.011465
0.011244	0.011244	0.011281	0.011391	0.011244	0.011502	0.011502
0.011539	0.011539	0.011428	0.011576	0.011760	0.011577	0.011391
0.011391	0.011465	0.011465	0.011281	0.011318	0.011244	0.011355
0.011465	0.011465	0.011502	0.011465	0.011355	0.011539	0.011539
0.011395	0.011244	0.011391	0.011502	0.011392	0.011502	0.011502
0.011723	0.011434	0.011612	0.011539	0.011465	0.011649	0.011281
0.011429	0.011355	0.011318	0.011318	0.011355	0.011244	0.011355
0.011428	0.011318	0.011355	0.011391	0.011355	0.011686	0.011686
0.011391	0.011428	0.011502	0.011465	0.012129	0.011533	0.011409
0.011368	0.011480	0.011601	0.011365	0.011599	0.011515	0.011438
0.012041	0.011364	0.011512	0.011440	0.011703	0.011508	0.011695
0.012104	0.011516	0.011392	0.011391	0.011391	0.011428	0.011355
0.011244	0.011281	0.011428	0.011391	0.011392	0.011539	0.011465
0.011318	0.011281	0.011281	0.011318	0.011244	0.011281	0.011207
0.011281	0.011318	0.011281	0.011244	0.011355	0.011281	0.011318
0.011281	0.011244	0.011318	0.011355	0.011244	0.011465	0.011391
0.011281	0.011391	0.011244	0.011281	0.011318	0.011760	0.011318
0.011405	0.011281	0.011576	0.011581	0.011428	0.011391	0.011391
0.011428	0.011355	0.011281	0.011355	0.011465	0.011428	0.011391
0.011391	0.011502	0.011686	0.011391	0.011539	0.011539	0.011465
0.011281	0.011391	0.011428	0.011834	0.011392	0.011428	0.011355
0.011428	0.011318	0.011318	0.011649	0.011576	0.011612	0.011355
0.011465	0.011539	0.011355	0.011465	0.011355	0.011281	0.011649
0.011391	0.011318	0.011318	0.011428	0.011355	0.011428	0.011723
0.011318	0.011806	0.011797	0.011531	0.011180	0.011465	0.011223
0.011292	0.011682	0.011283	0.011391	0.011315	0.011332	0.011340
0.011431	0.011129	0.011236	0.000000	0.011228	0.011269	0.011398
0.011455	0.011309	0.011395	0.011324	0.011356	0.011539	0.011575
0.011539	0.011429	0.011579	0.011581	0.011692	0.011681	0.011318
0.011649	0.011465	0.011355	0.011465	0.011244	0.011502	0.011244
0.011355	0.011355	0.011281	0.011281	0.011281	0.011281	0.011391
0.011362	0.011328	0.011320	0.011360	0.011548	0.011506	0.011471

0.011471	0.011355	0.011180	0.011177	0.011357	0.011543	0.011469
0.011318	0.011394	0.011650	0.011540	0.011539	0.011502	0.011465
0.011391	0.011502	0.011502	0.011318	0.012018	0.011576	0.011613
0.011686	0.011646	0.011360	0.011439	0.011318	0.011539	0.011465
0.011503	0.011465	0.011539	0.011539	0.011574	0.011686	0.011327
0.011209	0.011213	0.011284	0.011284	0.011398	0.011226	0.011582
0.011361	0.011542	0.011216	0.011348	0.011355	0.011435	0.011318
0.011244	0.011281	0.011355	0.011281	0.011539	0.011502	0.011428
0.011539	0.011539	0.011318	0.011318	0.011355	0.011355	0.011355
0.011429	0.011355	0.011355	0.011356	0.011539	0.011575	0.011539
0.011429	0.011579	0.011581	0.011692	0.011681	0.011318	0.011502
0.011465	0.011465	0.011282	0.011519	0.011442	0.011434	0.011328
0.011437	0.011712	0.011517	0.011396	0.011281	0.011502	0.011318
0.011465	0.011391	0.011207	0.011392	0.011391	0.011393	0.011355
0.011281	0.011208	0.011355	0.011281	0.011208	0.011321	0.011502
0.011391	0.011318	0.011355	0.011391	0.011318	0.011244	0.011465
0.011318	0.011391	0.011355	0.011318	0.011244	0.011539	0.011281
0.011318	0.011760	0.011318	0.011355	0.011318	0.011723	0.011242
0.011644	0.011240	0.011324	0.011484	0.011532	0.011517	0.011193
0.011169	0.011465	0.011539	0.011655	0.011502	0.011650	0.011797
0.011687	0.011727	0.011686	0.011991	0.011612	0.012350	0.011723
0.012461	0.012055	0.012205	0.012647	0.011686	0.011318	0.011612
0.012092	0.011797	0.011649	0.011539	0.011612	0.011612	0.011617
0.011465	0.011467	0.011539	0.011502	0.011907	0.011981	0.011907
0.011834	0.011944	0.012055	0.011797	0.012129	0.012173	0.012240
0.012240	0.012203	0.012203	0.012203	0.012203	0.011391	0.011723
0.011797	0.012202	0.011839	0.011981	0.011797	0.011580	0.011465
0.011428	0.011428	0.011760	0.011923	0.011968	0.011760	0.011429
S104	0.011572	0.011572	0.011533	0.011454	0.011454	0.011533
0.011417	0.011338	0.011533	0.011375	0.011415	0.011375	0.011336
0.011296	0.011572	0.011296	0.011414	0.011414	0.011415	0.011375
0.011375	0.011415	0.011139	0.011336	0.011415	0.011336	0.011417
0.011336	0.011415	0.011296	0.011139	0.011454	0.011415	0.011336
0.011415	0.011572	0.011454	0.011454	0.011454	0.011454	0.011651
0.011257	0.011454	0.011454	0.011415	0.011454	0.011572	0.011493
0.011454	0.011612	0.011415	0.011493	0.011414	0.011690	0.011414
0.011414	0.011454	0.011493	0.011690	0.011533	0.011454	0.011533
0.011493	0.011493	0.011415	0.011690	0.011533	0.011454	0.011493
0.011415	0.011415	0.011493	0.011533	0.011415	0.011454	0.011454
0.011375	0.011375	0.011336	0.011336	0.011454	0.011454	0.011533
0.011493	0.011375	0.011533	0.011296	0.011493	0.011375	0.011375
0.011572	0.011572	0.011572	0.011572	0.011415	0.011690	0.011454
0.011533	0.011493	0.011658	0.011346	0.011342	0.011433	0.000000
0.000000	0.011270	0.011358	0.011611	0.011572	0.011375	0.011415
0.011493	0.011454	0.011572	0.011454	0.011493	0.011454	0.011415
0.011375	0.011415	0.011769	0.011611	0.011493	0.011454	0.011415
0.011336	0.011296	0.011454	0.011336	0.011257	0.011218	0.011139
0.011257	0.011336	0.011375	0.011375	0.011415	0.011414	0.011415
0.011494	0.011415	0.011454	0.011305	0.011346	0.011347	0.011414
0.011414	0.011375	0.011428	0.011415	0.011339	0.011379	0.011416
0.011348	0.011422	0.011416	0.011375	0.011336	0.011493	0.011415
0.011493	0.011415	0.011415	0.011493	0.011493	0.011848	0.011612
0.011415	0.011533	0.011414	0.011493	0.011336	0.011257	0.011296
0.011415	0.011414	0.011375	0.011375	0.011375	0.011375	0.011493
0.011415	0.011533	0.011493	0.011336	0.011455	0.011611	0.011575
0.011572	0.011493	0.011375	0.011298	0.011454	0.011375	0.011297
0.011299	0.011572	0.011375	0.011572	0.011296	0.011415	0.011375
0.011533	0.011375	0.011454	0.011375	0.011415	0.011296	0.011415
0.011454	0.011414	0.011454	0.011375	0.011414	0.011454	0.011651
0.011533	0.011375	0.011493	0.011572	0.011533	0.011415	0.011414

0.011377	0.011639	0.011550	0.011300	0.011539	0.011300	0.011474
0.011536	0.011418	0.011533	0.011848	0.011501	0.011651	0.011612
0.011572	0.011690	0.011644	0.011448	0.011453	0.011493	0.011577
0.011690	0.011534	0.011612	0.011693	0.011493	0.011769	0.011893
0.011535	0.011698	0.011616	0.011656	0.011888	0.011336	0.011495
0.011454	0.011415	0.011296	0.011296	0.011375	0.011415	0.011454
0.011415	0.011493	0.011375	0.011375	0.011415	0.011414	0.011415
0.011415	0.011533	0.011454	0.011493	0.011296	0.011415	0.011454
0.011454	0.011375	0.011454	0.011454	0.011336	0.011336	0.011336
0.011572	0.011454	0.011572	0.011336	0.011414	0.011572	0.011336
0.011336	0.011336	0.011414	0.011533	0.011375	0.011296	0.011415
0.011375	0.011415	0.011414	0.011336	0.011336	0.011454	0.011415
0.011336	0.011454	0.011296	0.011336	0.011493	0.011297	0.011336
0.011336	0.011493	0.011336	0.011296	0.011336	0.011296	0.011493
0.011583	0.011375	0.011375	0.011493	0.011493	0.011375	0.011493
0.011375	0.011690	0.011296	0.011415	0.011296	0.011296	0.011296
0.011493	0.011454	0.011415	0.011533	0.011336	0.011454	0.011296
0.011375	0.011375	0.011375	0.011454	0.011414	0.011533	0.011611
0.011572	0.011493	0.011454	0.011572	0.011533	0.011375	0.011336
0.011375	0.011375	0.011415	0.011257	0.011336	0.011493	0.011375
0.011336	0.011375	0.011493	0.011533	0.011454	0.011414	0.011415
0.011454	0.011375	0.011454	0.011336	0.011533	0.011611	0.011572
0.011375	0.011494	0.011415	0.018505	0.011257	0.011675	0.011415
0.011415	0.011415	0.011492	0.011304	0.011507	0.011454	0.011555
0.011456	0.011123	0.011261	0.011430	0.011317	0.011365	0.011427
0.011302	0.011261	0.011264	0.000000	0.011533	0.011572	0.011536
0.011421	0.011533	0.011533	0.011415	0.011454	0.011454	0.011415
0.011454	0.011375	0.011375	0.011493	0.011415	0.011375	0.011415
0.011415	0.011454	0.011296	0.011454	0.011533	0.011572	0.011454
0.011415	0.011375	0.011415	0.011415	0.011533	0.011573	0.011572
0.011296	0.011336	0.011336	0.011493	0.011336	0.011572	0.011572
0.011533	0.011533	0.011533	0.011572	0.011533	0.011574	0.011493
0.011454	0.011533	0.011611	0.011415	0.011415	0.011375	0.011493
0.011454	0.011454	0.011493	0.011454	0.011455	0.011572	0.011611
0.011498	0.011336	0.011454	0.011612	0.011415	0.011533	0.011651
0.011848	0.011501	0.011651	0.011612	0.011572	0.011690	0.011375
0.011574	0.011454	0.011415	0.011415	0.011454	0.011296	0.011454
0.011533	0.011572	0.011454	0.011415	0.011375	0.011415	0.011415
0.011533	0.011573	0.011572	0.011533	0.012125	0.011599	0.011514
0.011632	0.011542	0.011677	0.011508	0.011587	0.011624	0.011497
0.012063	0.011464	0.011580	0.011544	0.011823	0.011655	0.011695
0.012138	0.011593	0.011455	0.011454	0.011454	0.011139	0.011415
0.011336	0.011375	0.011493	0.011454	0.011454	0.011257	0.011296
0.011415	0.011375	0.011415	0.011414	0.011336	0.011375	0.011296
0.011375	0.011415	0.011375	0.011336	0.011454	0.011375	0.011415
0.011414	0.011336	0.011415	0.011454	0.011336	0.011572	0.011611
0.011454	0.011493	0.011493	0.011454	0.011336	0.011533	0.011454
0.011101	0.011375	0.011336	0.011339	0.011493	0.011375	0.011533
0.011533	0.011454	0.011375	0.011454	0.011572	0.011572	0.011454
0.011493	0.011572	0.011493	0.011414	0.011611	0.011611	0.011493
0.011375	0.011493	0.011533	0.011572	0.011494	0.011493	0.011493
0.011493	0.011296	0.011296	0.011612	0.011690	0.011572	0.011415
0.011533	0.011533	0.011454	0.011533	0.011414	0.011375	0.011375
0.011493	0.011414	0.011296	0.011533	0.011415	0.011533	0.011572
0.011414	0.011496	0.011572	0.011307	0.011118	0.011415	0.011168
0.011424	0.011548	0.011296	0.011467	0.011304	0.011315	0.011273
0.011267	0.011192	0.011339	0.011126	0.011222	0.011328	0.011437
0.011537	0.011384	0.011415	0.011418	0.011495	0.011257	0.011336
0.011296	0.011494	0.011574	0.011219	0.011654	0.011724	0.011415
0.011415	0.011533	0.011414	0.011493	0.011336	0.011257	0.011296

0.011415	0.011414	0.011375	0.011375	0.011375	0.011375	0.011493
0.011417	0.011465	0.011454	0.011419	0.011617	0.011534	0.011536
0.011536	0.011415	0.011313	0.011305	0.011454	0.011612	0.011573
0.011375	0.011493	0.011769	0.011651	0.011611	0.011611	0.011533
0.011493	0.011611	0.011611	0.011454	0.011809	0.011572	0.011690
0.011808	0.011479	0.011381	0.011507	0.011414	0.011611	0.011572
0.011534	0.011572	0.011533	0.011533	0.011706	0.011769	0.011426
0.011298	0.011303	0.011418	0.011379	0.011381	0.011324	0.011339
0.011461	0.011266	0.011342	0.011495	0.011454	0.011538	0.011415
0.011336	0.011296	0.011454	0.011336	0.011257	0.011218	0.011139
0.011257	0.011336	0.011375	0.011375	0.011415	0.011414	0.011415
0.011494	0.011415	0.011454	0.011495	0.011257	0.011336	0.011296
0.011494	0.011574	0.011219	0.011654	0.011724	0.011415	0.011651
0.011572	0.011572	0.011338	0.011547	0.011548	0.011500	0.011430
0.011464	0.011812	0.011612	0.011459	0.011375	0.011572	0.011415
0.011533	0.011493	0.011336	0.011455	0.011611	0.011575	0.011493
0.011375	0.011298	0.011454	0.011375	0.011297	0.011299	0.011572
0.011454	0.011375	0.011375	0.011493	0.011415	0.011375	0.011257
0.011414	0.011493	0.011493	0.011414	0.011493	0.011651	0.011257
0.011415	0.011533	0.011414	0.011415	0.011375	0.011493	0.011374
0.011581	0.011236	0.011215	0.011273	0.011313	0.011257	0.011191
0.011169	0.011296	0.011415	0.011538	0.011375	0.011533	0.011690
0.011573	0.011576	0.011572	0.011735	0.011415	0.012440	0.011572
0.012243	0.012045	0.012286	0.012363	0.011415	0.011375	0.011533
0.012164	0.011691	0.011454	0.011375	0.011454	0.011454	0.011378
0.011296	0.011297	0.011375	0.011336	0.011612	0.011651	0.011690
0.011493	0.011612	0.011769	0.011493	0.012125	0.012172	0.012283
0.012283	0.012244	0.012164	0.012204	0.012204	0.011493	0.011730
0.011769	0.012243	0.011814	0.011730	0.011533	0.011338	0.011257
0.011218	0.011218	0.011651	0.011631	0.011783	0.011454	0.011218
S105 0.012452	0.012452	0.012158	0.012085	0.012085	0.012158	
0.012135	0.012024	0.012195	0.012011	0.012048	0.012011	0.012011
0.011975	0.012195	0.011938	0.012048	0.012085	0.012085	0.012011
0.012011	0.012048	0.011792	0.012013	0.012049	0.012011	0.012098
0.011975	0.012048	0.011938	0.011828	0.012085	0.012011	0.012011
0.012085	0.012195	0.012085	0.012085	0.012121	0.012085	0.012268
0.011938	0.012085	0.012121	0.012048	0.012085	0.012195	0.012121
0.012085	0.012268	0.012086	0.012121	0.012085	0.012341	0.012085
0.012048	0.012085	0.012195	0.012341	0.012121	0.012048	0.012195
0.012121	0.012158	0.012048	0.012305	0.012195	0.012085	0.012158
0.012048	0.012085	0.012121	0.012158	0.012085	0.012121	0.012121
0.012011	0.012011	0.011975	0.012011	0.012085	0.012048	0.012158
0.012121	0.012085	0.012158	0.012011	0.012121	0.012011	0.012011
0.012195	0.012195	0.012195	0.012195	0.012085	0.012341	0.012085
0.012221	0.012190	0.011600	0.011343	0.011324	0.012108	0.000000
0.000000	0.011264	0.011348	0.012268	0.012268	0.012011	0.012085
0.012158	0.012085	0.012195	0.012085	0.012158	0.012085	0.012121
0.012085	0.012085	0.012378	0.012268	0.012158	0.012122	0.012048
0.012011	0.011938	0.012085	0.012011	0.011902	0.011865	0.011792
0.011902	0.011975	0.012011	0.012011	0.012048	0.012048	0.012049
0.012123	0.012085	0.012085	0.011891	0.011899	0.012010	0.012085
0.012085	0.012011	0.011404	0.012048	0.011985	0.012023	0.012052
0.012052	0.012066	0.012088	0.012011	0.011719	0.012158	0.012011
0.012085	0.012085	0.012085	0.012158	0.012158	0.012525	0.012231
0.012085	0.012158	0.012048	0.012121	0.011975	0.011902	0.011938
0.012048	0.012048	0.012011	0.012011	0.012011	0.012011	0.012121
0.012048	0.012195	0.012121	0.012011	0.012089	0.012231	0.012203
0.012231	0.012121	0.012011	0.011942	0.012085	0.012011	0.011939
0.011947	0.012231	0.012012	0.012231	0.011975	0.012048	0.012048
0.012158	0.012048	0.012085	0.012048	0.012085	0.011938	0.012048

0.012085	0.012048	0.012121	0.012048	0.012085	0.012121	0.012305
0.012158	0.012011	0.012158	0.012231	0.012121	0.012048	0.012048
0.012015	0.012474	0.012413	0.011991	0.012171	0.011952	0.011444
0.012134	0.012095	0.012231	0.012451	0.012178	0.012305	0.012305
0.012231	0.012341	0.012297	0.012245	0.012288	0.012122	0.012204
0.012305	0.012199	0.012278	0.012352	0.012158	0.012415	0.012539
0.012201	0.012355	0.012315	0.012277	0.012526	0.011975	0.012162
0.012048	0.012048	0.011938	0.011938	0.012048	0.012048	0.012121
0.012048	0.012121	0.012011	0.012011	0.012048	0.012048	0.012085
0.012048	0.012158	0.012085	0.012121	0.011938	0.012048	0.012085
0.012085	0.012048	0.012121	0.012085	0.012011	0.012011	0.012011
0.012231	0.012085	0.012195	0.011975	0.012085	0.012231	0.011975
0.011975	0.012011	0.012048	0.012195	0.012011	0.011938	0.012048
0.012011	0.012048	0.012048	0.011975	0.011975	0.012085	0.012085
0.011975	0.012085	0.011938	0.011975	0.012121	0.011939	0.012012
0.011976	0.012121	0.012049	0.011938	0.011975	0.011938	0.012121
0.012218	0.012012	0.012011	0.012122	0.012121	0.012048	0.012158
0.012011	0.012341	0.011938	0.012085	0.011938	0.011938	0.011938
0.012121	0.012121	0.012048	0.012158	0.011975	0.012085	0.011938
0.012011	0.012011	0.012011	0.012121	0.012048	0.012158	0.012231
0.012195	0.012158	0.012085	0.012195	0.012158	0.012011	0.011975
0.012011	0.012011	0.012048	0.011938	0.011975	0.012121	0.012011
0.011975	0.012011	0.012121	0.012121	0.012085	0.012048	0.012048
0.012121	0.012011	0.012121	0.012011	0.012158	0.012231	0.012158
0.012048	0.012085	0.012048	0.018730	0.011257	0.012842	0.012059
0.012059	0.012059	0.011492	0.011292	0.011515	0.012133	0.011554
0.011440	0.011124	0.011262	0.012206	0.011300	0.011369	0.012224
0.011295	0.011248	0.011249	0.000000	0.012158	0.012231	0.012240
0.012072	0.012195	0.012158	0.012048	0.012085	0.012086	0.012086
0.012121	0.012048	0.012048	0.012121	0.012048	0.012011	0.012048
0.012048	0.012085	0.011975	0.012085	0.012158	0.012195	0.012048
0.012048	0.012011	0.012011	0.012011	0.012159	0.012196	0.012231
0.011938	0.011975	0.011975	0.012121	0.011975	0.012195	0.012195
0.012195	0.012231	0.012158	0.012231	0.012121	0.012235	0.012121
0.012085	0.012158	0.012231	0.012048	0.012048	0.012011	0.012121
0.012122	0.012121	0.012158	0.012121	0.012088	0.012231	0.012268
0.012132	0.011975	0.012121	0.012231	0.012048	0.012158	0.012268
0.012451	0.012178	0.012305	0.012305	0.012231	0.012341	0.012012
0.012198	0.012085	0.012048	0.012048	0.012085	0.011975	0.012085
0.012158	0.012195	0.012048	0.012048	0.012011	0.012011	0.012011
0.012159	0.012196	0.012231	0.012195	0.012783	0.012273	0.012177
0.012283	0.012198	0.012367	0.012158	0.012285	0.012266	0.012178
0.012786	0.012119	0.012257	0.012155	0.012489	0.012285	0.012359
0.012803	0.012304	0.012087	0.012085	0.012085	0.011792	0.012050
0.011975	0.012011	0.012121	0.012085	0.012086	0.011902	0.011938
0.012048	0.012011	0.012048	0.012048	0.011975	0.012011	0.011938
0.012011	0.012048	0.012011	0.011975	0.012085	0.012011	0.012048
0.012048	0.011975	0.012048	0.012085	0.011975	0.012195	0.012231
0.012085	0.012121	0.012121	0.012085	0.011975	0.012121	0.012085
0.011783	0.012012	0.011975	0.011985	0.012158	0.012121	0.012158
0.012158	0.012085	0.012011	0.012085	0.012195	0.012195	0.012121
0.012121	0.012195	0.012121	0.012158	0.012268	0.012268	0.012195
0.012011	0.012121	0.012158	0.012158	0.012122	0.012158	0.012121
0.012121	0.011975	0.011975	0.012305	0.012305	0.012268	0.012048
0.012195	0.012231	0.012085	0.012195	0.012085	0.012011	0.012048
0.012121	0.012048	0.011975	0.012231	0.012048	0.012195	0.012231
0.012048	0.012209	0.012195	0.011307	0.011117	0.012085	0.011164
0.012412	0.011527	0.011975	0.011462	0.011304	0.011315	0.011273
0.011264	0.011192	0.011339	0.011126	0.011222	0.011328	0.011438
0.012240	0.011381	0.012070	0.012058	0.012126	0.011902	0.012011

0.011938	0.012122	0.012240	0.011947	0.011650	0.012407	0.012011
0.012085	0.012158	0.012048	0.012121	0.011975	0.011902	0.011938
0.012048	0.012048	0.012011	0.012011	0.012011	0.012011	0.012121
0.011917	0.011474	0.012086	0.012097	0.012280	0.012124	0.012203
0.012239	0.012085	0.011295	0.011289	0.012085	0.012268	0.012197
0.012011	0.012121	0.012378	0.012305	0.012231	0.012305	0.012231
0.012121	0.012231	0.012195	0.012085	0.012378	0.012231	0.012341
0.012415	0.012145	0.012065	0.012154	0.012048	0.012268	0.012231
0.012234	0.012231	0.012231	0.012231	0.012478	0.012451	0.012083
0.011945	0.011968	0.012062	0.012023	0.012030	0.012045	0.011986
0.012106	0.012107	0.012254	0.012204	0.012085	0.012133	0.012048
0.012011	0.011938	0.012085	0.012011	0.011902	0.011865	0.011792
0.011902	0.011975	0.012011	0.012011	0.012048	0.012048	0.012049
0.012123	0.012085	0.012085	0.012126	0.011902	0.012011	0.011938
0.012122	0.012240	0.011947	0.011650	0.012407	0.012011	0.012268
0.012231	0.012231	0.012018	0.012232	0.012194	0.012174	0.012087
0.012107	0.012601	0.012403	0.012135	0.012012	0.012231	0.012048
0.012195	0.012121	0.012011	0.012089	0.012231	0.012203	0.012121
0.012011	0.011942	0.012085	0.012011	0.011939	0.011947	0.012231
0.012121	0.012048	0.012048	0.012121	0.012048	0.012011	0.011902
0.012085	0.012121	0.012121	0.012048	0.012121	0.012268	0.011938
0.012048	0.012121	0.012048	0.012048	0.012048	0.012121	0.011360
0.011566	0.011230	0.011211	0.011262	0.011305	0.011949	0.011186
0.011165	0.011938	0.012048	0.012170	0.012011	0.012158	0.012305
0.012196	0.012239	0.012195	0.012125	0.012085	0.013002	0.012195
0.012856	0.012672	0.012860	0.013041	0.012085	0.012011	0.012158
0.012820	0.012342	0.012085	0.012012	0.012085	0.012085	0.011982
0.011938	0.011939	0.012011	0.011975	0.012232	0.012378	0.012305
0.012231	0.012341	0.012378	0.012196	0.012783	0.012868	0.012931
0.012930	0.012930	0.012856	0.012856	0.012893	0.012158	0.012378
0.012415	0.012966	0.012462	0.012415	0.012232	0.011983	0.011902
0.011865	0.011865	0.012268	0.012417	0.012440	0.012085	0.011866
S106 0.011620	0.011620	0.011671	0.011620	0.011620	0.011671	
0.011419	0.011317	0.011620	0.011467	0.011467	0.011467	0.011416
0.011365	0.011670	0.011365	0.011416	0.011467	0.011467	0.011467
0.011467	0.011467	0.011213	0.011417	0.011467	0.011416	0.011419
0.011416	0.011518	0.011365	0.011213	0.011467	0.011467	0.011365
0.011518	0.011569	0.011518	0.011569	0.011569	0.011518	0.011772
0.011314	0.011518	0.011569	0.011416	0.011518	0.011721	0.011620
0.011569	0.011670	0.011467	0.011467	0.011518	0.011670	0.011518
0.011467	0.011518	0.011569	0.011670	0.011467	0.011365	0.011569
0.011518	0.011467	0.011518	0.011772	0.011416	0.011569	0.011518
0.011518	0.011518	0.011620	0.011670	0.011467	0.011467	0.011467
0.011467	0.011467	0.011416	0.011416	0.011416	0.011467	0.011670
0.011518	0.011467	0.011569	0.011365	0.011467	0.011416	0.011416
0.011721	0.011620	0.011518	0.011620	0.011569	0.011721	0.011518
0.011636	0.011479	0.011772	0.011427	0.011416	0.011488	0.011270
0.011264	0.000000	0.011427	0.011619	0.011518	0.011467	0.011518
0.011518	0.011467	0.011620	0.011518	0.011671	0.011518	0.011467
0.011416	0.011467	0.011721	0.011721	0.011569	0.011569	0.011467
0.011416	0.011365	0.011467	0.011365	0.011365	0.011213	0.011213
0.011314	0.011314	0.011416	0.011416	0.011416	0.011416	0.011417
0.011417	0.011518	0.011518	0.011376	0.011432	0.011433	0.011467
0.011518	0.011467	0.011519	0.011518	0.011370	0.011474	0.011521
0.011167	0.011528	0.011520	0.011467	0.011365	0.011518	0.011416
0.011518	0.011467	0.011467	0.011569	0.011518	0.011874	0.011620
0.011416	0.011518	0.011467	0.011518	0.011416	0.011264	0.011365
0.011467	0.011467	0.011416	0.011467	0.011467	0.011467	0.011569
0.011467	0.011620	0.011518	0.011365	0.011569	0.011620	0.011570
0.011620	0.011518	0.011365	0.011365	0.011518	0.011365	0.011365

0.011369	0.011620	0.011416	0.011620	0.011365	0.011467	0.011416
0.011569	0.011365	0.011518	0.011467	0.011467	0.011365	0.011416
0.011518	0.011467	0.011467	0.011416	0.011467	0.011518	0.011721
0.011467	0.011365	0.011416	0.011619	0.011366	0.011467	0.011467
0.011468	0.011703	0.011517	0.011419	0.011522	0.011371	0.011580
0.011419	0.011266	0.011620	0.011772	0.011523	0.011721	0.011671
0.011721	0.011721	0.011656	0.011431	0.011507	0.011569	0.011626
0.011721	0.011570	0.011671	0.011724	0.011518	0.011772	0.012087
0.011572	0.011731	0.011729	0.011677	0.011824	0.011416	0.011622
0.011467	0.011467	0.011365	0.011365	0.011416	0.011518	0.011467
0.011467	0.011518	0.011416	0.011416	0.011416	0.011467	0.011416
0.011467	0.011569	0.011365	0.011365	0.011365	0.011467	0.011569
0.011518	0.011314	0.011569	0.011467	0.011467	0.011467	0.011467
0.011620	0.011518	0.011620	0.011416	0.011518	0.011518	0.011416
0.011416	0.011416	0.011518	0.011620	0.011416	0.011365	0.011416
0.011467	0.011518	0.011518	0.011416	0.011365	0.011416	0.011569
0.011416	0.011518	0.011365	0.011365	0.011569	0.000000	0.011467
0.011417	0.011569	0.011468	0.011365	0.011264	0.011365	0.011620
0.011479	0.011467	0.011365	0.011569	0.011518	0.011467	0.011569
0.011467	0.011670	0.011365	0.011314	0.011365	0.011365	0.011365
0.011518	0.011264	0.011518	0.011620	0.011365	0.011365	0.011365
0.011365	0.011467	0.011467	0.011416	0.011518	0.011670	0.011620
0.011620	0.011569	0.011467	0.011620	0.011569	0.011467	0.011416
0.011416	0.011467	0.011518	0.011314	0.011365	0.011569	0.011416
0.011365	0.011467	0.011518	0.011518	0.011467	0.011416	0.011518
0.011467	0.011467	0.011518	0.011467	0.011569	0.011670	0.011416
0.011518	0.011416	0.011416	0.019854	0.011281	0.011575	0.011365
0.011416	0.011416	0.011565	0.011388	0.011461	0.011518	0.011574
0.011492	0.011213	0.011379	0.011448	0.011379	0.011316	0.011512
0.011215	0.011264	0.011213	0.011248	0.011569	0.011620	0.011521
0.011476	0.011670	0.011620	0.011467	0.011518	0.011518	0.011467
0.011467	0.011365	0.011416	0.011569	0.011467	0.011467	0.011467
0.011467	0.011569	0.011365	0.011569	0.011620	0.011569	0.011467
0.011518	0.011416	0.011315	0.011315	0.011569	0.011671	0.011670
0.011365	0.011365	0.011416	0.011467	0.011365	0.011620	0.011620
0.011467	0.011620	0.011619	0.011518	0.011416	0.011520	0.011467
0.011518	0.011518	0.011619	0.011518	0.011518	0.011365	0.011518
0.011518	0.011518	0.011569	0.011467	0.011519	0.011670	0.011569
0.011575	0.011365	0.011518	0.011670	0.011518	0.011620	0.011721
0.011772	0.011523	0.011721	0.011671	0.011721	0.011721	0.011416
0.011724	0.011569	0.011467	0.011467	0.011569	0.011365	0.011569
0.011620	0.011569	0.011467	0.011518	0.011416	0.011315	0.011315
0.011569	0.011671	0.011569	0.011569	0.012130	0.011546	0.011434
0.011689	0.011535	0.011702	0.011584	0.011647	0.011687	0.011581
0.012101	0.011480	0.011682	0.011480	0.011843	0.011727	0.011779
0.012147	0.011582	0.011417	0.011467	0.011467	0.011213	0.011417
0.011416	0.011365	0.011518	0.011467	0.011417	0.011264	0.011314
0.011518	0.011416	0.011467	0.011467	0.011365	0.011467	0.011365
0.011467	0.011467	0.011365	0.011416	0.011518	0.011467	0.011518
0.011467	0.011416	0.011416	0.011467	0.011365	0.011569	0.011670
0.011467	0.011518	0.011518	0.011518	0.011365	0.011467	0.011518
0.011219	0.011416	0.011416	0.011421	0.011569	0.011569	0.011569
0.011620	0.011467	0.011416	0.011467	0.011721	0.011569	0.011467
0.011518	0.011671	0.011467	0.011569	0.011620	0.011620	0.011670
0.011467	0.011518	0.011620	0.011416	0.011569	0.011569	0.011518
0.011467	0.011365	0.011365	0.011620	0.011721	0.011620	0.011467
0.011670	0.011671	0.011467	0.011670	0.011518	0.011467	0.011315
0.011518	0.011518	0.011365	0.011620	0.011467	0.011620	0.011467
0.011518	0.011316	0.011416	0.011322	0.011188	0.011416	0.011236
0.011356	0.011474	0.011365	0.011461	0.011355	0.011249	0.011307

0.011228	0.011214	0.011374	0.011203	0.011245	0.011438	0.011607
0.011636	0.011485	0.011525	0.011518	0.011572	0.011264	0.011365
0.011365	0.011518	0.011676	0.011266	0.011778	0.011704	0.011467
0.011416	0.011518	0.011467	0.011518	0.011416	0.011264	0.011365
0.011467	0.011467	0.011416	0.011467	0.011467	0.011467	0.011569
0.011475	0.011530	0.011569	0.011421	0.011730	0.011519	0.011572
0.011676	0.011416	0.011386	0.011374	0.011569	0.011772	0.011620
0.011467	0.011620	0.011925	0.011722	0.011569	0.011772	0.011467
0.011518	0.011620	0.011467	0.011467	0.011773	0.011467	0.011620
0.012027	0.011381	0.011371	0.011586	0.011467	0.011467	0.011620
0.011467	0.011670	0.011518	0.011518	0.011585	0.011823	0.011482
0.011369	0.011376	0.011524	0.011474	0.011476	0.011399	0.011318
0.011528	0.000000	0.011487	0.011515	0.011467	0.011473	0.011467
0.011416	0.011365	0.011467	0.011365	0.011365	0.011213	0.011213
0.011314	0.011314	0.011416	0.011416	0.011416	0.011416	0.011417
0.011417	0.011518	0.011518	0.011572	0.011264	0.011365	0.011365
0.011518	0.011676	0.011266	0.011778	0.011704	0.011467	0.011670
0.011620	0.011620	0.011418	0.011583	0.011634	0.011520	0.011533
0.011472	0.011909	0.011669	0.011574	0.011416	0.011620	0.011467
0.011620	0.011518	0.011365	0.011569	0.011620	0.011570	0.011518
0.011365	0.011365	0.011518	0.011365	0.011365	0.011369	0.011620
0.011467	0.011365	0.011416	0.011569	0.011467	0.011467	0.011314
0.011467	0.011569	0.011620	0.011467	0.011518	0.011721	0.011314
0.011518	0.011467	0.011467	0.011467	0.011467	0.011467	0.011428
0.011586	0.011224	0.011217	0.011273	0.011319	0.011267	0.011215
0.011170	0.011314	0.011467	0.011569	0.011416	0.011620	0.011670
0.011569	0.011625	0.011518	0.011926	0.011467	0.012486	0.011569
0.012231	0.012027	0.012286	0.012437	0.011365	0.011416	0.011569
0.012334	0.011671	0.011467	0.011416	0.011416	0.011416	0.011368
0.011314	0.011315	0.011365	0.011315	0.011722	0.011671	0.011772
0.011518	0.011620	0.011772	0.011518	0.012181	0.012138	0.012232
0.012283	0.012232	0.012232	0.012181	0.012232	0.011518	0.011721
0.011721	0.012231	0.011877	0.011569	0.011467	0.011162	0.011111
0.011162	0.011111	0.011722	0.011742	0.011886	0.011518	0.011162
S107	0.011348	0.011348	0.011761	0.011678	0.011678	0.011761
0.011558	0.011475	0.011678	0.011184	0.011225	0.011184	0.011143
0.011184	0.011390	0.011184	0.011184	0.011184	0.011225	0.011102
0.011184	0.011225	0.011348	0.011143	0.011225	0.011143	0.011557
0.011472	0.011225	0.011102	0.011390	0.011266	0.011184	0.011143
0.011225	0.011390	0.011266	0.011266	0.011266	0.011266	0.011472
0.011143	0.011225	0.011266	0.011225	0.011348	0.011390	0.011307
0.011266	0.011431	0.011225	0.011637	0.011225	0.011431	0.011225
0.011225	0.011349	0.011307	0.011431	0.011637	0.011266	0.011472
0.011307	0.011349	0.011225	0.011513	0.011637	0.011266	0.011307
0.011225	0.011225	0.011307	0.011349	0.011225	0.011266	0.011266
0.011184	0.011184	0.011143	0.011184	0.011266	0.011184	0.011348
0.011266	0.011225	0.011348	0.011102	0.011390	0.011184	0.011184
0.011390	0.011348	0.011554	0.011390	0.011225	0.011513	0.011266
0.011311	0.011650	0.011431	0.011147	0.011144	0.011234	0.011358
0.011348	0.011427	0.000000	0.011431	0.011348	0.011266	0.011307
0.011266	0.011266	0.011390	0.011266	0.011307	0.011266	0.011225
0.011184	0.011307	0.011513	0.011431	0.011307	0.011307	0.011225
0.011184	0.011184	0.011266	0.011143	0.011472	0.011431	0.011348
0.011472	0.011554	0.011143	0.011143	0.011143	0.011143	0.011143
0.011184	0.011266	0.011266	0.011103	0.011146	0.011146	0.011225
0.011266	0.011184	0.011227	0.011225	0.011144	0.011186	0.011226
0.011448	0.011228	0.011226	0.011102	0.011143	0.011266	0.011266
0.011349	0.011266	0.011266	0.011307	0.011307	0.011719	0.011472
0.011596	0.011431	0.011225	0.011390	0.011143	0.011390	0.011102
0.011184	0.011184	0.011184	0.011184	0.011184	0.011184	0.011307

0.011225	0.011348	0.011225	0.011184	0.011267	0.011431	0.011392
0.011390	0.011307	0.011143	0.011102	0.011266	0.011143	0.011102
0.011144	0.011390	0.011184	0.011390	0.011102	0.011225	0.011184
0.011349	0.011184	0.011266	0.011184	0.011225	0.011102	0.011225
0.011266	0.011143	0.011225	0.011184	0.011225	0.011307	0.011390
0.011678	0.011513	0.011637	0.011307	0.011596	0.011184	0.011143
0.011267	0.011510	0.011346	0.011144	0.011684	0.011102	0.011253
0.011641	0.011518	0.011348	0.011555	0.011310	0.011472	0.011472
0.011390	0.011472	0.011407	0.011162	0.011110	0.011225	0.011351
0.011472	0.011308	0.011431	0.011474	0.011307	0.011554	0.011724
0.011350	0.011477	0.011476	0.011476	0.011679	0.011143	0.011308
0.011225	0.011184	0.011102	0.011102	0.011184	0.011225	0.011307
0.011225	0.011307	0.011184	0.011266	0.011225	0.011143	0.011225
0.011225	0.011678	0.011554	0.011596	0.011102	0.011184	0.011266
0.011266	0.011184	0.011307	0.011349	0.011184	0.011184	0.011184
0.011719	0.011307	0.011348	0.011143	0.011225	0.011431	0.011143
0.011143	0.011143	0.011184	0.011349	0.011184	0.011348	0.011472
0.011431	0.011225	0.011143	0.011143	0.011143	0.011225	0.011266
0.011143	0.011266	0.011102	0.011102	0.011307	0.011390	0.011184
0.011143	0.011307	0.011143	0.011102	0.011472	0.011102	0.011307
0.011313	0.011184	0.011513	0.011266	0.011390	0.011184	0.011266
0.011184	0.011431	0.011102	0.011307	0.011184	0.011184	0.011102
0.011307	0.011554	0.011225	0.011307	0.011102	0.011554	0.011184
0.011184	0.011102	0.011184	0.011307	0.011225	0.011348	0.011431
0.011390	0.011266	0.011266	0.011390	0.011348	0.011184	0.011143
0.011143	0.011184	0.011225	0.011143	0.011143	0.011307	0.011143
0.011184	0.011143	0.011266	0.011390	0.011266	0.011225	0.011225
0.011266	0.011184	0.011266	0.011143	0.011307	0.011390	0.011678
0.011225	0.011596	0.011225	0.018031	0.011193	0.011430	0.011555
0.011554	0.011555	0.011286	0.011246	0.011724	0.011596	0.011325
0.011456	0.011461	0.011103	0.011204	0.011192	0.011227	0.011207
0.011456	0.011390	0.011393	0.011132	0.011307	0.011348	0.011267
0.011228	0.011307	0.011348	0.011307	0.011266	0.011266	0.011266
0.011225	0.011184	0.011143	0.011307	0.011225	0.011184	0.011184
0.011184	0.011266	0.011143	0.011266	0.011348	0.011348	0.011184
0.011307	0.011266	0.011472	0.011472	0.011349	0.011390	0.011390
0.011102	0.011143	0.011143	0.011266	0.011143	0.011390	0.011390
0.011307	0.011348	0.011348	0.011348	0.011554	0.011350	0.011266
0.011225	0.011307	0.011431	0.011225	0.011225	0.011184	0.011266
0.011266	0.011266	0.011307	0.011266	0.011267	0.011348	0.011390
0.011310	0.011143	0.011184	0.011431	0.011225	0.011431	0.011431
0.011555	0.011310	0.011472	0.011472	0.011390	0.011472	0.011184
0.011391	0.011266	0.011184	0.011184	0.011266	0.011143	0.011266
0.011348	0.011348	0.011184	0.011307	0.011266	0.011472	0.011472
0.011349	0.011390	0.011348	0.011349	0.012009	0.011363	0.011276
0.011443	0.011354	0.011448	0.011316	0.011400	0.011440	0.011310
0.011942	0.011272	0.011395	0.011313	0.011607	0.011434	0.011517
0.011979	0.011338	0.011184	0.011225	0.011184	0.011348	0.011143
0.011143	0.011184	0.011266	0.011225	0.011184	0.011390	0.011431
0.011225	0.011102	0.011225	0.011225	0.011143	0.011184	0.011102
0.011266	0.011307	0.011184	0.011143	0.011266	0.011184	0.011225
0.011225	0.011143	0.011225	0.011266	0.011143	0.011390	0.011431
0.011266	0.011390	0.011307	0.011266	0.011143	0.011637	0.011225
0.011318	0.011184	0.011472	0.011477	0.011307	0.011266	0.011307
0.011349	0.011266	0.011184	0.011266	0.011390	0.011348	0.011266
0.011266	0.011349	0.011637	0.011266	0.011472	0.011472	0.011348
0.011184	0.011266	0.011349	0.011596	0.011266	0.011307	0.011266
0.011266	0.011225	0.011225	0.011513	0.011431	0.011513	0.011225
0.011349	0.011390	0.011266	0.011349	0.011225	0.011184	0.011513
0.011307	0.011225	0.011225	0.011348	0.011225	0.011348	0.011637

0.011225	0.011599	0.011719	0.011448	0.011173	0.011307	0.011220
0.011190	0.011685	0.011102	0.011381	0.011161	0.011322	0.011330
0.011417	0.011127	0.011286	0.011128	0.011170	0.011328	0.011280
0.011358	0.011230	0.011307	0.011185	0.011308	0.011390	0.011513
0.011472	0.011390	0.011475	0.011435	0.011516	0.011582	0.011143
0.011596	0.011431	0.011225	0.011390	0.011143	0.011390	0.011102
0.011184	0.011184	0.011184	0.011184	0.011184	0.011184	0.011307
0.011185	0.011230	0.011184	0.011227	0.011435	0.011308	0.011309
0.011351	0.011184	0.011104	0.011103	0.011266	0.011431	0.011390
0.011184	0.011307	0.011596	0.011431	0.011390	0.011431	0.011348
0.011266	0.011431	0.011390	0.011266	0.011843	0.011348	0.011431
0.011637	0.011580	0.011144	0.011315	0.011225	0.011431	0.011348
0.011349	0.011390	0.011390	0.011390	0.011391	0.011513	0.011230
0.011102	0.011103	0.011227	0.011185	0.011270	0.000000	0.011476
0.011186	0.011372	0.000000	0.011241	0.011266	0.011310	0.011225
0.011184	0.011184	0.011266	0.011143	0.011472	0.011431	0.011348
0.011472	0.011554	0.011143	0.011143	0.011143	0.011143	0.011143
0.011184	0.011266	0.011266	0.011308	0.011390	0.011513	0.011472
0.011390	0.011475	0.011435	0.011516	0.011582	0.011143	0.011431
0.011348	0.011348	0.011143	0.011400	0.011359	0.011269	0.011232
0.011271	0.011624	0.011428	0.011310	0.011184	0.011390	0.011225
0.011348	0.011225	0.011184	0.011267	0.011431	0.011392	0.011307
0.011143	0.011102	0.011266	0.011143	0.011102	0.011144	0.011390
0.011225	0.011184	0.011143	0.011307	0.011225	0.011184	0.011390
0.011266	0.011266	0.011307	0.011225	0.011307	0.011472	0.011143
0.011225	0.011637	0.011225	0.011225	0.011184	0.011637	0.011148
0.011622	0.011147	0.011215	0.011398	0.011440	0.011390	0.011104
0.011169	0.011431	0.011554	0.011685	0.011513	0.011678	0.011843
0.011720	0.011724	0.011678	0.011926	0.011554	0.012586	0.011719
0.012339	0.012173	0.012425	0.012546	0.011513	0.011266	0.011678
0.012050	0.011843	0.011596	0.011514	0.011554	0.011554	0.011475
0.011431	0.011431	0.011472	0.011431	0.011761	0.011761	0.011843
0.011678	0.011761	0.011884	0.011678	0.012009	0.012016	0.012009
0.012091	0.012092	0.012050	0.012050	0.012092	0.011307	0.011596
0.011596	0.012215	0.011640	0.011843	0.011678	0.011473	0.011390
0.011348	0.011348	0.011761	0.011705	0.011852	0.011596	0.011348
S108	0.011899	0.011899	0.012236	0.012000	0.012000	0.012067
0.011909	0.011841	0.012067	0.011497	0.011563	0.011531	0.011496
0.011530	0.011698	0.011496	0.011463	0.011597	0.011564	0.011530
0.011429	0.011530	0.011631	0.011497	0.011530	0.011530	0.011875
0.011765	0.011530	0.011429	0.011664	0.011597	0.011564	0.011597
0.011564	0.011664	0.011564	0.011564	0.011530	0.011564	0.011731
0.011496	0.011564	0.011530	0.011530	0.011631	0.011530	0.011597
0.011564	0.011664	0.011631	0.011899	0.011496	0.011832	0.011496
0.011530	0.011631	0.011698	0.011832	0.011933	0.011631	0.011731
0.011597	0.011664	0.011564	0.011799	0.012034	0.011564	0.011631
0.011530	0.011631	0.011597	0.011664	0.011597	0.011597	0.011597
0.011496	0.011530	0.011496	0.011597	0.011563	0.011599	0.011664
0.011597	0.011564	0.011631	0.011530	0.011664	0.011496	0.011564
0.011731	0.011664	0.011899	0.011732	0.011564	0.011799	0.011563
0.011728	0.011957	0.011777	0.011451	0.011433	0.011575	0.011611
0.012268	0.011619	0.011431	0.000000	0.011597	0.011597	0.011698
0.011631	0.011597	0.011664	0.011564	0.011664	0.011597	0.011597
0.011563	0.011664	0.011866	0.011731	0.011631	0.011597	0.011530
0.011497	0.011497	0.011564	0.011530	0.011732	0.011698	0.011631
0.011732	0.011799	0.011530	0.011530	0.011564	0.011564	0.011567
0.011635	0.011564	0.011564	0.011456	0.011494	0.011481	0.011564
0.011568	0.011507	0.011540	0.011543	0.011468	0.011505	0.011533
0.011780	0.011577	0.011577	0.011497	0.011475	0.011632	0.011598
0.011666	0.011599	0.011598	0.011698	0.011631	0.011933	0.011799

0.011832	0.011698	0.011564	0.011665	0.011463	0.011698	0.011497
0.011564	0.011564	0.011530	0.011496	0.011530	0.011497	0.011597
0.011564	0.011665	0.011597	0.011497	0.011599	0.011698	0.011702
0.011698	0.011598	0.011497	0.011431	0.011564	0.011497	0.011430
0.011536	0.011698	0.011497	0.011698	0.011463	0.011564	0.011530
0.011631	0.011530	0.011564	0.011530	0.011564	0.011429	0.011564
0.011597	0.011530	0.011597	0.011530	0.011564	0.011597	0.011833
0.011933	0.011866	0.011933	0.011633	0.011934	0.011564	0.011530
0.011567	0.011869	0.011609	0.011437	0.011942	0.011435	0.011493
0.011943	0.011842	0.011530	0.011933	0.011641	0.011597	0.011765
0.011631	0.011664	0.011735	0.011349	0.011647	0.011597	0.011669
0.011765	0.011667	0.011669	0.011872	0.011799	0.011866	0.011975
0.011703	0.011875	0.011737	0.011703	0.011969	0.011463	0.011667
0.011631	0.011564	0.011463	0.011463	0.011530	0.011530	0.011597
0.011530	0.011597	0.011496	0.011597	0.011530	0.011530	0.011530
0.011530	0.011967	0.011866	0.011899	0.011429	0.011530	0.011564
0.011564	0.011530	0.011597	0.011631	0.011496	0.011496	0.011496
0.012034	0.011664	0.011664	0.011463	0.011563	0.011664	0.011496
0.011496	0.011563	0.011530	0.011698	0.011496	0.011631	0.011765
0.011698	0.011530	0.011530	0.011463	0.011463	0.011631	0.011564
0.011463	0.011563	0.011429	0.011463	0.011597	0.011732	0.011497
0.011463	0.011597	0.011531	0.011429	0.011765	0.011429	0.011597
0.011676	0.011496	0.011799	0.011631	0.011664	0.011530	0.011564
0.011496	0.011832	0.011429	0.011698	0.011496	0.011496	0.011429
0.011664	0.011866	0.011564	0.011631	0.011463	0.011866	0.011496
0.011496	0.011496	0.011530	0.011631	0.011530	0.011631	0.011698
0.011698	0.011664	0.011563	0.011664	0.011631	0.011496	0.011463
0.011496	0.011496	0.011564	0.011496	0.011463	0.011631	0.011496
0.011530	0.011530	0.011664	0.011698	0.011564	0.011530	0.011530
0.011597	0.011530	0.011631	0.011496	0.011631	0.011530	0.012034
0.011530	0.011933	0.011543	0.017936	0.011257	0.011611	0.011897
0.011856	0.011904	0.011532	0.011524	0.011884	0.011923	0.011485
0.011629	0.011671	0.011450	0.011479	0.011529	0.011504	0.011490
0.011670	0.011656	0.011653	0.011523	0.011664	0.011698	0.011703
0.011575	0.011665	0.011597	0.011631	0.011597	0.011598	0.011564
0.011530	0.011530	0.011362	0.011597	0.011564	0.011497	0.011530
0.011530	0.011597	0.011497	0.011597	0.011631	0.011665	0.011564
0.011597	0.011564	0.011900	0.011900	0.011631	0.011598	0.011698
0.011463	0.011463	0.011497	0.011597	0.011463	0.011698	0.011698
0.011530	0.011530	0.011664	0.011564	0.011933	0.011565	0.011597
0.011396	0.011665	0.011698	0.011530	0.011597	0.011564	0.011463
0.011195	0.011195	0.011228	0.011665	0.011565	0.011765	0.011732
0.011602	0.011497	0.011597	0.011698	0.011597	0.011732	0.011765
0.011933	0.011641	0.011597	0.011765	0.011631	0.011664	0.011497
0.011532	0.011564	0.011530	0.011530	0.011597	0.011497	0.011597
0.011631	0.011665	0.011564	0.011597	0.011564	0.011900	0.011900
0.011631	0.011598	0.011731	0.011664	0.012305	0.011560	0.011658
0.011693	0.011546	0.011798	0.011684	0.011655	0.011715	0.011543
0.012262	0.011579	0.011711	0.011648	0.011921	0.011774	0.011877
0.012284	0.011776	0.011838	0.011899	0.011765	0.011765	0.011804
0.011463	0.011631	0.011765	0.011731	0.011736	0.011731	0.011700
0.011563	0.011496	0.011530	0.011564	0.011463	0.011530	0.011429
0.011564	0.011597	0.011496	0.011463	0.011564	0.011496	0.011530
0.011463	0.011463	0.011530	0.011564	0.011463	0.011664	0.011698
0.011597	0.011664	0.011597	0.011597	0.011530	0.011933	0.011564
0.011617	0.011530	0.011766	0.011773	0.011631	0.011631	0.011631
0.011631	0.011564	0.011530	0.011564	0.011664	0.011664	0.011597
0.011597	0.011731	0.011899	0.011631	0.011832	0.011832	0.011698
0.011530	0.011597	0.011631	0.012034	0.011598	0.011631	0.011597
0.011429	0.011563	0.011563	0.011866	0.011799	0.011832	0.011564

0.011664	0.011731	0.011564	0.011664	0.011631	0.011496	0.011832
0.011597	0.011564	0.011563	0.011731	0.011564	0.011664	0.011900
0.011597	0.011977	0.011967	0.011710	0.011399	0.011672	0.011473
0.011390	0.011917	0.011482	0.011641	0.011499	0.011518	0.011538
0.011672	0.011258	0.011450	0.011258	0.011437	0.011462	0.011628
0.011625	0.011532	0.011659	0.011518	0.011599	0.011732	0.011799
0.011799	0.011665	0.011736	0.011772	0.011908	0.011873	0.011597
0.011832	0.011698	0.011564	0.011665	0.011463	0.011698	0.011497
0.011564	0.011564	0.011530	0.011496	0.011530	0.011497	0.011597
0.011409	0.011550	0.011505	0.011608	0.011744	0.011709	0.011572
0.011708	0.011632	0.011429	0.011348	0.011568	0.011738	0.011739
0.011531	0.011602	0.011835	0.011804	0.011531	0.011767	0.011698
0.011631	0.011698	0.011698	0.011597	0.012169	0.011564	0.011631
0.011866	0.011915	0.011572	0.011649	0.011530	0.011732	0.011698
0.011733	0.011765	0.011564	0.011564	0.011886	0.011899	0.011546
0.011432	0.011441	0.011537	0.011502	0.011607	0.011471	0.011774
0.011575	0.011762	0.011489	0.011622	0.011564	0.011642	0.011530
0.011497	0.011497	0.011564	0.011530	0.011732	0.011698	0.011631
0.011732	0.011799	0.011530	0.011530	0.011564	0.011564	0.011567
0.011635	0.011564	0.011564	0.011599	0.011732	0.011799	0.011799
0.011665	0.011736	0.011772	0.011908	0.011873	0.011597	0.011765
0.011698	0.011698	0.011500	0.011687	0.011616	0.011673	0.011516
0.011714	0.011880	0.011695	0.011604	0.011497	0.011698	0.011564
0.011665	0.011597	0.011497	0.011599	0.011698	0.011702	0.011598
0.011497	0.011431	0.011564	0.011497	0.011430	0.011536	0.011698
0.011530	0.011530	0.011362	0.011597	0.011564	0.011497	0.011698
0.011564	0.011597	0.011597	0.011530	0.011597	0.011832	0.011496
0.011530	0.011933	0.011530	0.011564	0.011530	0.011899	0.011403
0.011847	0.011485	0.011461	0.011704	0.011793	0.011706	0.011353
0.011375	0.011698	0.011731	0.011841	0.011700	0.011838	0.011968
0.011868	0.011906	0.011866	0.012188	0.011899	0.012775	0.011936
0.012607	0.012443	0.012577	0.012849	0.011899	0.011598	0.011833
0.012339	0.012207	0.011866	0.011765	0.011834	0.011834	0.011736
0.011698	0.011671	0.011765	0.011733	0.012103	0.012169	0.012170
0.012069	0.012170	0.012274	0.012041	0.012340	0.012347	0.012407
0.012408	0.012441	0.012391	0.012408	0.012409	0.011631	0.011934
0.012002	0.012505	0.012010	0.012236	0.012710	0.011773	0.011665
0.011597	0.011631	0.012102	0.012171	0.012257	0.011967	0.011633
S109	0.011900	0.011900	0.012236	0.012000	0.012000	0.012068
0.011909	0.011841	0.012068	0.011497	0.011564	0.011531	0.011496
0.011530	0.011698	0.011496	0.011530	0.011597	0.011564	0.011530
0.011496	0.011530	0.011631	0.011497	0.011531	0.011463	0.011875
0.011765	0.011530	0.011429	0.011664	0.011597	0.011564	0.011597
0.011564	0.011664	0.011564	0.011564	0.011597	0.011564	0.011732
0.011496	0.011564	0.011597	0.011530	0.011631	0.011597	0.011597
0.011564	0.011732	0.011631	0.011900	0.011564	0.011832	0.011564
0.011530	0.011631	0.011698	0.011832	0.011933	0.011631	0.011732
0.011597	0.011664	0.011564	0.011799	0.012034	0.011564	0.011631
0.011530	0.011631	0.011597	0.011664	0.011597	0.011597	0.011597
0.011496	0.011530	0.011496	0.011597	0.011564	0.011599	0.011664
0.011597	0.011564	0.011631	0.011530	0.011664	0.011496	0.011564
0.011731	0.011664	0.011899	0.011732	0.011564	0.011799	0.011564
0.011728	0.011957	0.011664	0.011373	0.011359	0.011576	0.011572
0.012268	0.011518	0.011348	0.011597	0.000000	0.011597	0.011698
0.011631	0.011597	0.011664	0.011564	0.011664	0.011597	0.011597
0.011564	0.011664	0.011866	0.011731	0.011631	0.011597	0.011530
0.011497	0.011497	0.011564	0.011530	0.011732	0.011698	0.011631
0.011732	0.011799	0.011530	0.011530	0.011564	0.011564	0.011533
0.011601	0.011564	0.011564	0.011456	0.011422	0.011481	0.011564
0.011568	0.011507	0.011429	0.011543	0.011468	0.011506	0.011533

0.011780	0.011577	0.011577	0.011497	0.011475	0.011632	0.011598
0.011666	0.011599	0.011598	0.011698	0.011631	0.012000	0.011799
0.011832	0.011698	0.011564	0.011665	0.011463	0.011698	0.011497
0.011564	0.011564	0.011530	0.011496	0.011530	0.011497	0.011597
0.011564	0.011665	0.011598	0.011497	0.011599	0.011698	0.011702
0.011698	0.011598	0.011497	0.011431	0.011564	0.011497	0.011430
0.011536	0.011698	0.011497	0.011698	0.011429	0.011564	0.011530
0.011631	0.011530	0.011564	0.011530	0.011564	0.011430	0.011564
0.011598	0.011530	0.011597	0.011530	0.011564	0.011597	0.011833
0.011933	0.011866	0.011934	0.011599	0.011934	0.011564	0.011530
0.011567	0.011869	0.011609	0.011403	0.011942	0.011435	0.011532
0.011943	0.011842	0.011530	0.011933	0.011641	0.011530	0.011765
0.011698	0.011597	0.011735	0.011431	0.011647	0.011597	0.011669
0.011765	0.011667	0.011669	0.011872	0.011799	0.011866	0.011975
0.011703	0.011875	0.011805	0.011737	0.012002	0.011463	0.011667
0.011631	0.011564	0.011463	0.011463	0.011530	0.011530	0.011597
0.011530	0.011597	0.011496	0.011597	0.011530	0.011530	0.011597
0.011530	0.011967	0.011866	0.011900	0.011429	0.011530	0.011564
0.011564	0.011530	0.011597	0.011631	0.011496	0.011496	0.011496
0.012034	0.011664	0.011664	0.011463	0.011564	0.011732	0.011496
0.011496	0.011564	0.011530	0.011698	0.011496	0.011631	0.011765
0.011698	0.011530	0.011530	0.011463	0.011463	0.011631	0.011564
0.011463	0.011564	0.011429	0.011463	0.011597	0.011732	0.011497
0.011463	0.011597	0.011531	0.011429	0.011765	0.011429	0.011597
0.011676	0.011497	0.011799	0.011631	0.011664	0.011530	0.011631
0.011496	0.011832	0.011429	0.011698	0.011496	0.011496	0.011429
0.011664	0.011833	0.011564	0.011631	0.011463	0.011866	0.011496
0.011496	0.011496	0.011530	0.011631	0.011530	0.011631	0.011698
0.011698	0.011664	0.011564	0.011665	0.011631	0.011496	0.011463
0.011496	0.011496	0.011564	0.011496	0.011463	0.011631	0.011496
0.011530	0.011530	0.011664	0.011698	0.011597	0.011530	0.011530
0.011597	0.011530	0.011631	0.011496	0.011631	0.011530	0.012034
0.011530	0.011933	0.011509	0.017936	0.011257	0.011672	0.011863
0.011822	0.011869	0.011479	0.011408	0.011774	0.011889	0.011485
0.011629	0.011610	0.011372	0.011521	0.011411	0.011406	0.011533
0.011529	0.011582	0.011579	0.011457	0.011664	0.011698	0.011703
0.011575	0.011665	0.011597	0.011631	0.011597	0.011598	0.011564
0.011530	0.011530	0.011362	0.011597	0.011564	0.011497	0.011530
0.011530	0.011597	0.011497	0.011598	0.011631	0.011665	0.011564
0.011597	0.011564	0.011900	0.011900	0.011631	0.011665	0.011698
0.011463	0.011463	0.011497	0.011598	0.011463	0.011698	0.011698
0.011463	0.011530	0.011597	0.011496	0.011934	0.011498	0.011597
0.011396	0.011665	0.011698	0.011530	0.011597	0.011564	0.011530
0.011463	0.011463	0.011497	0.011665	0.011565	0.011765	0.011732
0.011602	0.011497	0.011597	0.011698	0.011598	0.011732	0.011765
0.011933	0.011641	0.011530	0.011765	0.011698	0.011597	0.011497
0.011599	0.011564	0.011530	0.011530	0.011597	0.011497	0.011598
0.011631	0.011665	0.011564	0.011597	0.011564	0.011900	0.011900
0.011631	0.011665	0.011731	0.011664	0.012305	0.011489	0.011658
0.011728	0.011476	0.011798	0.011684	0.011655	0.011715	0.011474
0.012262	0.011579	0.011711	0.011648	0.011921	0.011774	0.011877
0.012284	0.011776	0.011804	0.011900	0.011765	0.011765	0.011770
0.011463	0.011631	0.011765	0.011731	0.011702	0.011731	0.011700
0.011564	0.011496	0.011530	0.011564	0.011463	0.011530	0.011429
0.011564	0.011597	0.011496	0.011463	0.011564	0.011496	0.011530
0.011530	0.011463	0.011530	0.011564	0.011463	0.011664	0.011698
0.011597	0.011664	0.011597	0.011597	0.011530	0.011933	0.011564
0.011617	0.011530	0.011766	0.011773	0.011631	0.011631	0.011631
0.011631	0.011564	0.011530	0.011564	0.011664	0.011664	0.011597
0.011597	0.011732	0.011899	0.011631	0.011832	0.011832	0.011698

0.011530	0.011597	0.011631	0.012034	0.011598	0.011631	0.011597
0.011429	0.011564	0.011564	0.011866	0.011732	0.011832	0.011564
0.011664	0.011732	0.011597	0.011664	0.011631	0.011496	0.011832
0.011597	0.011564	0.011564	0.011732	0.011564	0.011664	0.011900
0.011597	0.011977	0.011967	0.011670	0.011342	0.011672	0.011421
0.011445	0.011878	0.011446	0.011551	0.011499	0.011569	0.011538
0.011519	0.011258	0.011505	0.011258	0.011384	0.011462	0.011547
0.011663	0.011454	0.011624	0.011553	0.011600	0.011732	0.011799
0.011799	0.011665	0.011770	0.011772	0.011908	0.011944	0.011597
0.011832	0.011698	0.011564	0.011665	0.011463	0.011698	0.011497
0.011564	0.011564	0.011530	0.011496	0.011530	0.011497	0.011597
0.011409	0.011550	0.011574	0.011608	0.011745	0.011709	0.011641
0.011708	0.011632	0.011356	0.011348	0.011568	0.011738	0.011739
0.011531	0.011602	0.011835	0.011804	0.011464	0.011767	0.011698
0.011631	0.011698	0.011698	0.011597	0.012169	0.011496	0.011564
0.011866	0.011879	0.011572	0.011649	0.011530	0.011732	0.011698
0.011733	0.011765	0.011496	0.011496	0.011925	0.011900	0.011546
0.011432	0.011441	0.011537	0.011502	0.011607	0.011471	0.011774
0.011575	0.011718	0.011489	0.011622	0.011565	0.011642	0.011530
0.011497	0.011497	0.011564	0.011530	0.011732	0.011698	0.011631
0.011732	0.011799	0.011530	0.011530	0.011564	0.011564	0.011533
0.011601	0.011564	0.011564	0.011600	0.011732	0.011799	0.011799
0.011665	0.011770	0.011772	0.011908	0.011944	0.011597	0.011765
0.011698	0.011698	0.011534	0.011687	0.011651	0.011639	0.011516
0.011715	0.011926	0.011653	0.011604	0.011497	0.011698	0.011564
0.011665	0.011598	0.011497	0.011599	0.011698	0.011702	0.011598
0.011497	0.011431	0.011564	0.011497	0.011430	0.011536	0.011698
0.011530	0.011530	0.011362	0.011597	0.011564	0.011497	0.011698
0.011564	0.011597	0.011597	0.011530	0.011597	0.011832	0.011496
0.011530	0.011933	0.011530	0.011564	0.011530	0.011900	0.011360
0.011735	0.011400	0.011311	0.011624	0.011712	0.011670	0.011353
0.011270	0.011698	0.011799	0.011909	0.011767	0.011872	0.012036
0.011935	0.011974	0.011933	0.012154	0.011899	0.012775	0.011936
0.012608	0.012443	0.012577	0.012849	0.011899	0.011598	0.011900
0.012339	0.012241	0.011866	0.011765	0.011834	0.011834	0.011737
0.011698	0.011671	0.011765	0.011733	0.012103	0.012169	0.012170
0.012070	0.012170	0.012274	0.012008	0.012340	0.012347	0.012407
0.012408	0.012442	0.012357	0.012408	0.012410	0.011631	0.011934
0.012002	0.012505	0.012010	0.012236	0.012710	0.011739	0.011665
0.011597	0.011631	0.012102	0.012212	0.012257	0.011967	0.011633
S110	0.011698	0.011698	0.011967	0.011732	0.011732	0.011799
0.011637	0.011569	0.011799	0.011295	0.011362	0.011329	0.011295
0.011262	0.011496	0.011161	0.011329	0.011396	0.011362	0.011329
0.011295	0.011329	0.011362	0.011295	0.011329	0.011329	0.011603
0.011496	0.011329	0.011228	0.011396	0.011362	0.011362	0.011396
0.011362	0.011463	0.011362	0.011362	0.011396	0.011362	0.011530
0.011228	0.011362	0.011396	0.011329	0.011362	0.011463	0.011396
0.011362	0.011530	0.011430	0.011631	0.011362	0.011631	0.011362
0.011329	0.011362	0.011496	0.011631	0.011664	0.011362	0.011463
0.011396	0.011463	0.011362	0.011597	0.011765	0.011362	0.011429
0.011329	0.011429	0.011396	0.011463	0.011396	0.011396	0.011396
0.011295	0.011329	0.011295	0.011329	0.011362	0.011363	0.011463
0.011396	0.011362	0.011429	0.011329	0.011396	0.011295	0.011362
0.011530	0.011463	0.011698	0.011530	0.011362	0.011597	0.011362
0.011505	0.011658	0.011551	0.011255	0.011247	0.011355	0.011375
0.012011	0.011467	0.011266	0.011597	0.011597	0.000000	0.011429
0.011429	0.011396	0.011463	0.011362	0.011463	0.011262	0.011396
0.011362	0.011329	0.011664	0.011463	0.011429	0.011396	0.011329
0.011295	0.011228	0.011362	0.011329	0.011463	0.011430	0.011362
0.011463	0.011530	0.011329	0.011329	0.011362	0.011362	0.011364

0.011432	0.011362	0.011362	0.011240	0.011313	0.011271	0.011363
0.011365	0.011301	0.011318	0.011336	0.011264	0.011300	0.011330
0.011528	0.011370	0.011405	0.011296	0.011302	0.011430	0.011329
0.011396	0.011397	0.011396	0.011496	0.011429	0.011799	0.011530
0.011564	0.011429	0.011362	0.011396	0.011262	0.011429	0.011295
0.011362	0.011362	0.011329	0.011295	0.011329	0.011295	0.011396
0.011362	0.011463	0.011396	0.011295	0.011397	0.011497	0.011499
0.011497	0.011396	0.011295	0.011229	0.011362	0.011295	0.011228
0.011332	0.011497	0.011295	0.011497	0.011262	0.011362	0.011329
0.011429	0.011329	0.011362	0.011329	0.011362	0.011228	0.011362
0.011396	0.011329	0.011396	0.011329	0.011362	0.011396	0.011631
0.011664	0.011597	0.011665	0.011465	0.011665	0.011362	0.011329
0.000000	0.011545	0.011472	0.011232	0.011671	0.011197	0.011336
0.011671	0.011570	0.011530	0.011732	0.011436	0.011597	0.011564
0.011496	0.011664	0.011522	0.011390	0.011395	0.011396	0.011466
0.011564	0.011465	0.011601	0.011669	0.011597	0.011664	0.011771
0.011500	0.011671	0.011602	0.011533	0.011800	0.011262	0.011464
0.011429	0.011362	0.011262	0.011262	0.011329	0.011329	0.011396
0.011329	0.011396	0.011295	0.011329	0.011329	0.011329	0.011396
0.011329	0.011698	0.011597	0.011631	0.011228	0.011329	0.011362
0.011362	0.011329	0.011396	0.011362	0.011295	0.011295	0.011295
0.011765	0.011396	0.011463	0.011262	0.011362	0.011530	0.011295
0.011295	0.011362	0.011329	0.011496	0.011295	0.011429	0.011564
0.011496	0.011329	0.011329	0.011262	0.011262	0.011429	0.011362
0.011262	0.011362	0.011228	0.011262	0.011396	0.011463	0.011295
0.011262	0.011396	0.011329	0.011228	0.011496	0.011228	0.011396
0.011471	0.011295	0.011530	0.011430	0.011396	0.011329	0.011429
0.011295	0.011631	0.011228	0.011429	0.011161	0.011161	0.011228
0.011463	0.011598	0.011362	0.011429	0.011262	0.011597	0.011161
0.011295	0.011295	0.011329	0.011429	0.011329	0.011429	0.011496
0.011496	0.011463	0.011362	0.011463	0.011429	0.011295	0.011262
0.011295	0.011295	0.011362	0.011228	0.011262	0.011429	0.011295
0.011194	0.011329	0.011463	0.011429	0.011396	0.011329	0.011329
0.011396	0.011329	0.011429	0.011295	0.011429	0.011530	0.011765
0.011329	0.011664	0.011302	0.017823	0.011322	0.011366	0.011583
0.011545	0.011588	0.011427	0.011408	0.011664	0.011612	0.011425
0.011487	0.011549	0.011216	0.011311	0.011216	0.011307	0.011318
0.011482	0.011395	0.011394	0.011193	0.011463	0.011497	0.011500
0.011369	0.011463	0.011463	0.011362	0.011396	0.011396	0.011362
0.011396	0.011329	0.011362	0.011396	0.011362	0.011295	0.011329
0.011329	0.011396	0.011295	0.011396	0.011429	0.011463	0.011362
0.011329	0.011295	0.011631	0.011631	0.011429	0.011464	0.011497
0.011262	0.011262	0.011295	0.011396	0.011262	0.011497	0.011497
0.011530	0.011530	0.011463	0.011564	0.011665	0.011565	0.011396
0.011396	0.011463	0.011497	0.011329	0.011396	0.011362	0.011396
0.011463	0.011463	0.011497	0.011463	0.011363	0.011564	0.011530
0.011399	0.011295	0.011396	0.011497	0.011396	0.011463	0.011564
0.011732	0.011436	0.011597	0.011564	0.011496	0.011664	0.011295
0.011464	0.011363	0.011329	0.011329	0.011396	0.011295	0.011396
0.011429	0.011463	0.011362	0.011329	0.011295	0.011631	0.011631
0.011429	0.011464	0.011530	0.011463	0.012035	0.011524	0.011447
0.011517	0.011476	0.011587	0.011476	0.011585	0.011508	0.011474
0.011987	0.011372	0.011505	0.011440	0.011714	0.011570	0.011672
0.012012	0.011475	0.011635	0.011698	0.011564	0.011497	0.011601
0.011262	0.011429	0.011563	0.011530	0.011533	0.011463	0.011397
0.011362	0.011295	0.011329	0.011362	0.011262	0.011329	0.011228
0.011295	0.011329	0.011295	0.011262	0.011362	0.011295	0.011329
0.011329	0.011262	0.011329	0.011362	0.011262	0.011463	0.011496
0.011396	0.011396	0.011396	0.011396	0.011329	0.011664	0.011362
0.011338	0.011329	0.011497	0.011501	0.011429	0.011429	0.011429

0.011429	0.011362	0.011329	0.011362	0.011463	0.011463	0.011396
0.011396	0.011530	0.011631	0.011429	0.011631	0.011631	0.011496
0.011329	0.011396	0.011429	0.011765	0.011396	0.011429	0.011396
0.011429	0.011295	0.011295	0.011597	0.011597	0.011564	0.011362
0.011463	0.011530	0.011396	0.011463	0.011429	0.011295	0.011564
0.011396	0.011362	0.011295	0.011530	0.011362	0.011463	0.011698
0.011396	0.011705	0.011698	0.011467	0.011173	0.011400	0.011267
0.011225	0.011683	0.011271	0.011373	0.011207	0.011315	0.011326
0.011417	0.011258	0.011394	0.011258	0.011276	0.011462	0.011425
0.011437	0.011336	0.011272	0.011342	0.011397	0.011463	0.011530
0.011530	0.011396	0.011500	0.011467	0.011569	0.011625	0.011396
0.011564	0.011429	0.011362	0.011396	0.011262	0.011429	0.011295
0.011362	0.011362	0.011329	0.011295	0.011329	0.011295	0.011396
0.011375	0.011375	0.011368	0.011403	0.011539	0.011504	0.011504
0.011503	0.011430	0.011245	0.011240	0.011365	0.011534	0.011535
0.011329	0.011399	0.011632	0.011601	0.011531	0.011565	0.011496
0.011429	0.011496	0.011496	0.011396	0.011900	0.011564	0.011631
0.011665	0.011665	0.011367	0.011441	0.011329	0.011530	0.011496
0.011531	0.011563	0.011564	0.011564	0.011689	0.011698	0.011338
0.011229	0.011233	0.011332	0.011298	0.011333	0.011247	0.011502
0.011369	0.011411	0.011251	0.011397	0.011363	0.011437	0.011329
0.011295	0.011228	0.011362	0.011329	0.011463	0.011430	0.011362
0.011463	0.011530	0.011329	0.011329	0.011362	0.011362	0.011364
0.011432	0.011362	0.011362	0.011397	0.011463	0.011530	0.011530
0.011396	0.011500	0.011467	0.011569	0.011625	0.011396	0.011564
0.011496	0.011496	0.011331	0.011513	0.011442	0.011469	0.011305
0.011473	0.011653	0.011441	0.011400	0.011295	0.011497	0.011362
0.011463	0.011396	0.011295	0.011397	0.011497	0.011499	0.011396
0.011295	0.011229	0.011362	0.011295	0.011228	0.011332	0.011497
0.011396	0.011329	0.011362	0.011396	0.011362	0.011295	0.011430
0.011362	0.011396	0.011396	0.011329	0.011396	0.011631	0.011228
0.011329	0.011664	0.011329	0.011362	0.011329	0.011631	0.011274
0.011622	0.011273	0.011261	0.011422	0.011508	0.011419	0.011227
0.011218	0.011429	0.011530	0.011637	0.011464	0.011601	0.011766
0.011666	0.011703	0.011664	0.011880	0.011631	0.012505	0.011666
0.012405	0.012139	0.012307	0.012545	0.011631	0.011329	0.011631
0.012070	0.011971	0.011597	0.011497	0.011565	0.011565	0.011534
0.011429	0.011399	0.011496	0.011464	0.011833	0.011900	0.011901
0.011800	0.011901	0.011970	0.011737	0.012070	0.012076	0.012137
0.012138	0.012171	0.012084	0.012138	0.012105	0.011429	0.011665
0.011733	0.012236	0.011704	0.011967	0.012440	0.011468	0.011396
0.011362	0.011362	0.011833	0.011883	0.011975	0.011631	0.011330
S111	0.011799	0.011799	0.012068	0.011832	0.011832	0.011900
0.011501	0.011434	0.011900	0.011396	0.011463	0.011430	0.011396
0.011362	0.011597	0.011329	0.011429	0.011496	0.011463	0.011429
0.011396	0.011429	0.011463	0.011396	0.011430	0.011429	0.011467
0.011597	0.011429	0.011329	0.011496	0.011497	0.011463	0.011497
0.011463	0.011564	0.011463	0.011463	0.011496	0.011463	0.011631
0.011329	0.011463	0.011497	0.011429	0.011463	0.011564	0.011497
0.011463	0.011631	0.011531	0.011530	0.011463	0.011732	0.011463
0.011429	0.011463	0.011597	0.011732	0.011564	0.011463	0.011564
0.011496	0.011564	0.011463	0.011698	0.011664	0.011463	0.011530
0.011429	0.011530	0.011497	0.011564	0.011496	0.011497	0.011497
0.011396	0.011429	0.011396	0.011430	0.011463	0.011498	0.011564
0.011497	0.011463	0.011530	0.011429	0.011429	0.011396	0.011463
0.011631	0.011564	0.011664	0.011631	0.011463	0.011698	0.011463
0.011579	0.011508	0.011588	0.011295	0.011321	0.011465	0.011415
0.012085	0.011518	0.011307	0.011698	0.011698	0.011429	0.000000
0.011530	0.011496	0.011564	0.011463	0.011564	0.011497	0.011496
0.011463	0.011496	0.011765	0.011631	0.011530	0.011497	0.011430

0.011396	0.011329	0.011463	0.011430	0.011564	0.011530	0.011463
0.011564	0.011631	0.011429	0.011429	0.011463	0.011463	0.011432
0.011500	0.011463	0.011463	0.011276	0.011349	0.011341	0.011464
0.011433	0.011335	0.011429	0.011405	0.011366	0.011369	0.011432
0.011564	0.011439	0.011473	0.011396	0.011371	0.011531	0.011430
0.011498	0.011498	0.011497	0.011597	0.011530	0.011900	0.011631
0.011664	0.011530	0.011463	0.011497	0.011362	0.011530	0.011396
0.011463	0.011463	0.011430	0.011396	0.011429	0.011396	0.011497
0.011463	0.011564	0.011497	0.011396	0.011498	0.011598	0.011601
0.011598	0.011497	0.011396	0.011330	0.011463	0.011396	0.011329
0.011434	0.011598	0.011396	0.011598	0.011362	0.011463	0.011430
0.011530	0.011430	0.011463	0.011430	0.011463	0.011329	0.011463
0.011497	0.011430	0.011497	0.011429	0.011463	0.011497	0.011732
0.011564	0.011496	0.011564	0.011600	0.011564	0.011463	0.011430
0.011398	0.011707	0.011563	0.011369	0.011569	0.011333	0.011493
0.011535	0.011672	0.011631	0.011833	0.011538	0.011698	0.011665
0.011597	0.011765	0.011629	0.011513	0.011521	0.011497	0.011568
0.011665	0.011566	0.011703	0.011771	0.011698	0.011765	0.011873
0.011602	0.011773	0.011636	0.011635	0.011901	0.011362	0.011566
0.011530	0.011463	0.011362	0.011362	0.011429	0.011429	0.011496
0.011429	0.011497	0.011396	0.011429	0.011429	0.011429	0.011496
0.011429	0.011597	0.011496	0.011530	0.011329	0.011429	0.011463
0.011463	0.011429	0.011497	0.011463	0.011396	0.011396	0.011396
0.011664	0.011497	0.011564	0.011362	0.011463	0.011631	0.011396
0.011396	0.011429	0.011429	0.011597	0.011396	0.011530	0.011664
0.011597	0.011429	0.011429	0.011362	0.011362	0.011530	0.011463
0.011362	0.011463	0.011329	0.011362	0.011497	0.011564	0.011396
0.011363	0.011497	0.011430	0.011329	0.011396	0.011329	0.011497
0.011574	0.011396	0.011429	0.011530	0.011497	0.011429	0.011530
0.011396	0.011732	0.011329	0.011463	0.011329	0.011329	0.011329
0.011564	0.011698	0.011463	0.011530	0.011362	0.011496	0.011329
0.011396	0.011396	0.011429	0.011530	0.011429	0.011530	0.011597
0.011597	0.011564	0.011463	0.011564	0.011530	0.011396	0.011362
0.011396	0.011396	0.011463	0.011329	0.011362	0.011530	0.011396
0.011362	0.011429	0.011564	0.011530	0.011496	0.011429	0.011430
0.011497	0.011429	0.011530	0.011396	0.011530	0.011631	0.011664
0.011429	0.011564	0.011405	0.017937	0.011388	0.011427	0.011479
0.011441	0.011482	0.011479	0.011466	0.011664	0.011543	0.011485
0.011535	0.011610	0.011255	0.011353	0.011294	0.011357	0.011361
0.011436	0.011470	0.011394	0.011325	0.011564	0.011597	0.011601
0.011472	0.011564	0.011564	0.011463	0.011497	0.011497	0.011463
0.011497	0.011429	0.011463	0.011497	0.011463	0.011396	0.011429
0.011429	0.011497	0.011396	0.011497	0.011530	0.011564	0.011463
0.011429	0.011396	0.011530	0.011530	0.011530	0.011565	0.011598
0.011362	0.011362	0.011396	0.011497	0.011362	0.011598	0.011598
0.011631	0.011631	0.011564	0.011665	0.011564	0.011666	0.011497
0.011497	0.011564	0.011598	0.011430	0.011497	0.011463	0.011497
0.011564	0.011564	0.011597	0.011564	0.011464	0.011665	0.011631
0.011500	0.011396	0.011497	0.011598	0.011497	0.011564	0.011664
0.011833	0.011538	0.011698	0.011665	0.011597	0.011765	0.011396
0.011566	0.011464	0.011429	0.011429	0.011497	0.011396	0.011497
0.011530	0.011564	0.011463	0.011429	0.011396	0.011530	0.011530
0.011530	0.011565	0.011631	0.011564	0.012137	0.011632	0.011553
0.011623	0.011580	0.011693	0.011580	0.011690	0.011612	0.011543
0.012090	0.011475	0.011608	0.011544	0.011818	0.011672	0.011775
0.012114	0.011588	0.011703	0.011799	0.011664	0.011597	0.011668
0.011362	0.011530	0.011664	0.011631	0.011601	0.011564	0.011532
0.011463	0.011396	0.011429	0.011463	0.011362	0.011429	0.011329
0.011396	0.011429	0.011396	0.011362	0.011463	0.011396	0.011429
0.011429	0.011362	0.011429	0.011463	0.011362	0.011564	0.011597

0.011497	0.011497	0.011497	0.011497	0.011429	0.011564	0.011463
0.011443	0.011429	0.011598	0.011603	0.011463	0.011463	0.011530
0.011530	0.011463	0.011429	0.011463	0.011564	0.011564	0.011497
0.011496	0.011631	0.011530	0.011530	0.011732	0.011732	0.011597
0.011429	0.011497	0.011530	0.011664	0.011497	0.011463	0.011497
0.011530	0.011396	0.011396	0.011698	0.011698	0.011665	0.011463
0.011564	0.011564	0.011496	0.011564	0.011530	0.011396	0.011463
0.011496	0.011463	0.011396	0.011631	0.011463	0.011564	0.011732
0.011496	0.011569	0.011597	0.011507	0.011286	0.011502	0.011318
0.011280	0.011722	0.011376	0.011462	0.011304	0.011417	0.011432
0.011519	0.011324	0.011506	0.011391	0.011384	0.011596	0.011506
0.011512	0.011414	0.011448	0.011448	0.011498	0.011564	0.011631
0.011631	0.011497	0.011601	0.011603	0.011738	0.011767	0.011497
0.011664	0.011530	0.011463	0.011497	0.011362	0.011530	0.011396
0.011463	0.011463	0.011430	0.011396	0.011429	0.011396	0.011497
0.011444	0.011445	0.011437	0.011471	0.011608	0.011573	0.011572
0.011571	0.011531	0.011282	0.011276	0.011433	0.011602	0.011603
0.011430	0.011467	0.011734	0.011703	0.011632	0.011666	0.011597
0.011530	0.011597	0.011597	0.011497	0.011799	0.011664	0.011732
0.011766	0.011558	0.011469	0.011545	0.011429	0.011631	0.011597
0.011632	0.011664	0.011664	0.011664	0.011807	0.011799	0.011442
0.011331	0.011337	0.011435	0.011400	0.011436	0.011359	0.011400
0.011472	0.011543	0.011394	0.011435	0.011464	0.011505	0.011430
0.011396	0.011329	0.011463	0.011430	0.011564	0.011530	0.011463
0.011564	0.011631	0.011429	0.011429	0.011463	0.011463	0.011432
0.011500	0.011463	0.011463	0.011498	0.011564	0.011631	0.011631
0.011497	0.011601	0.011603	0.011738	0.011767	0.011497	0.011664
0.011597	0.011597	0.011432	0.011617	0.011547	0.011571	0.011446
0.011611	0.011789	0.011568	0.011502	0.011396	0.011598	0.011463
0.011564	0.011497	0.011396	0.011498	0.011598	0.011601	0.011497
0.011396	0.011330	0.011463	0.011396	0.011329	0.011434	0.011598
0.011497	0.011429	0.011463	0.011497	0.011463	0.011396	0.011530
0.011463	0.011497	0.011496	0.011429	0.011497	0.011732	0.011329
0.011429	0.011564	0.011429	0.011463	0.011429	0.011530	0.011317
0.011735	0.011315	0.011311	0.011503	0.011549	0.011527	0.011227
0.011270	0.011530	0.011631	0.011739	0.011599	0.011737	0.011867
0.011767	0.011804	0.011765	0.012017	0.011732	0.012607	0.011768
0.012507	0.012275	0.012409	0.012680	0.011732	0.011430	0.011732
0.012171	0.012106	0.011698	0.011598	0.011666	0.011666	0.011635
0.011530	0.011535	0.011597	0.011565	0.011935	0.012001	0.012002
0.011901	0.012002	0.012106	0.011872	0.012172	0.012178	0.012239
0.012239	0.012273	0.012221	0.012240	0.012241	0.011530	0.011766
0.011834	0.012337	0.011840	0.012068	0.012541	0.011604	0.011497
0.011396	0.011463	0.011934	0.011965	0.012010	0.011799	0.011465
S112 0.011732	0.011732	0.012068	0.011832	0.011832	0.011900	
0.011705	0.011637	0.011899	0.011329	0.011396	0.011363	0.011329
0.011362	0.011530	0.011329	0.011362	0.011429	0.011396	0.011362
0.011329	0.011362	0.011463	0.011329	0.011363	0.011362	0.011671
0.011597	0.011362	0.011262	0.011496	0.011429	0.011396	0.011429
0.011396	0.011497	0.011396	0.011396	0.011429	0.011396	0.011564
0.011329	0.011396	0.011429	0.011362	0.011463	0.011496	0.011429
0.011396	0.011564	0.011463	0.011732	0.011396	0.011664	0.011396
0.011362	0.011463	0.011530	0.011664	0.011765	0.011463	0.011564
0.011429	0.011497	0.011396	0.011631	0.011866	0.011396	0.011463
0.011362	0.011463	0.011429	0.011497	0.011429	0.011429	0.011429
0.011329	0.011362	0.011329	0.011429	0.011396	0.011430	0.011496
0.011429	0.011396	0.011463	0.011362	0.011496	0.011329	0.011396
0.011564	0.011496	0.011732	0.011564	0.011396	0.011631	0.011396
0.011542	0.011770	0.011588	0.011295	0.011321	0.011428	0.011493
0.012158	0.011518	0.011266	0.011631	0.011631	0.011429	0.011530

0.000000	0.011429	0.011496	0.011396	0.011497	0.011429	0.011429
0.011396	0.011496	0.011698	0.011564	0.011463	0.011430	0.011362
0.011329	0.011329	0.011396	0.011362	0.011564	0.011530	0.011463
0.011564	0.011631	0.011362	0.011362	0.011396	0.011396	0.011398
0.011466	0.011396	0.011396	0.011276	0.011349	0.011306	0.011396
0.011399	0.011335	0.011392	0.011371	0.011298	0.011334	0.011364
0.011636	0.011405	0.011439	0.011329	0.011337	0.000000	0.011430
0.011497	0.011431	0.011430	0.011530	0.011463	0.011832	0.011631
0.011664	0.011530	0.011396	0.011497	0.011295	0.011530	0.011329
0.011396	0.011396	0.011362	0.011329	0.011362	0.011329	0.011430
0.011396	0.011497	0.011430	0.011329	0.011431	0.011530	0.011533
0.011530	0.011430	0.011329	0.011263	0.011396	0.011329	0.011262
0.011366	0.011531	0.011329	0.011530	0.011295	0.011396	0.011362
0.011463	0.011362	0.011396	0.011362	0.011396	0.011262	0.011396
0.011430	0.011362	0.011094	0.011362	0.011396	0.011430	0.011665
0.011765	0.011698	0.011766	0.011532	0.011766	0.011396	0.011362
0.011398	0.011667	0.011426	0.011300	0.011773	0.011265	0.011336
0.011739	0.011672	0.011564	0.011765	0.011436	0.011631	0.011597
0.011530	0.011698	0.011558	0.011431	0.011479	0.011430	0.011500
0.011597	0.011499	0.011635	0.011703	0.011631	0.011698	0.011806
0.011534	0.011705	0.011636	0.011567	0.011834	0.011295	0.011498
0.011463	0.011396	0.011295	0.011295	0.011362	0.011362	0.011429
0.011362	0.011429	0.011329	0.011429	0.011362	0.011362	0.011429
0.011362	0.011799	0.011698	0.011732	0.011262	0.011362	0.011396
0.011396	0.011362	0.011429	0.011463	0.011329	0.011329	0.011329
0.011866	0.011496	0.011497	0.011295	0.011396	0.011564	0.011329
0.011329	0.011396	0.011362	0.011530	0.011329	0.011463	0.011597
0.011530	0.011362	0.011362	0.011295	0.011295	0.011463	0.011396
0.011295	0.011329	0.011262	0.011295	0.011429	0.011564	0.011329
0.011295	0.011429	0.011363	0.011262	0.011597	0.011262	0.011429
0.011505	0.011329	0.011631	0.011463	0.011497	0.011362	0.011396
0.011329	0.011664	0.011262	0.011530	0.011329	0.011329	0.011262
0.011496	0.011698	0.011396	0.011463	0.011295	0.011698	0.011329
0.011329	0.011329	0.011362	0.011463	0.011362	0.011463	0.011530
0.011530	0.011497	0.011396	0.011497	0.011463	0.011329	0.011295
0.011329	0.011329	0.011396	0.011329	0.011295	0.011463	0.011329
0.011362	0.011362	0.011497	0.011530	0.011429	0.011362	0.011362
0.011429	0.011362	0.011463	0.011329	0.011463	0.011564	0.011866
0.011362	0.011765	0.011371	0.017861	0.011387	0.011488	0.011723
0.011683	0.011729	0.011427	0.011408	0.011829	0.011751	0.011485
0.011582	0.011610	0.011255	0.011312	0.011372	0.011406	0.011361
0.011623	0.011507	0.011542	0.011391	0.011497	0.011530	0.011500
0.011404	0.011497	0.011497	0.011463	0.011429	0.011430	0.011396
0.011430	0.011362	0.011396	0.011430	0.011396	0.011329	0.011362
0.011362	0.011430	0.011329	0.011430	0.011463	0.011497	0.011396
0.011429	0.011396	0.011732	0.011732	0.011463	0.011497	0.011530
0.011295	0.011295	0.011329	0.011430	0.011295	0.011530	0.011530
0.011564	0.011564	0.011497	0.011597	0.011766	0.011599	0.011430
0.011430	0.011497	0.011530	0.011362	0.011430	0.011396	0.011430
0.011497	0.011497	0.011530	0.011497	0.011397	0.011597	0.011564
0.011433	0.011329	0.011430	0.011530	0.011430	0.011564	0.011597
0.011765	0.011436	0.011631	0.011597	0.011530	0.011698	0.011329
0.011498	0.011396	0.011362	0.011362	0.011430	0.011329	0.011430
0.011463	0.011497	0.011396	0.011429	0.011396	0.011732	0.011732
0.011463	0.011497	0.011564	0.011496	0.012137	0.011560	0.011482
0.011552	0.011511	0.011622	0.011510	0.011620	0.011543	0.011508
0.012090	0.011406	0.011540	0.011475	0.011749	0.011604	0.011707
0.012114	0.011588	0.011669	0.011732	0.011597	0.011597	0.011635
0.011295	0.011463	0.011597	0.011564	0.011567	0.011564	0.011532
0.011396	0.011329	0.011362	0.011396	0.011295	0.011362	0.011262

0.011396	0.011429	0.011329	0.011295	0.011396	0.011329	0.011362
0.011362	0.011295	0.011362	0.011396	0.011295	0.011497	0.011530
0.011429	0.011497	0.011429	0.011429	0.011362	0.011765	0.011396
0.011443	0.011362	0.011598	0.011603	0.011463	0.011463	0.011463
0.011463	0.011396	0.011362	0.011396	0.011497	0.011497	0.011429
0.011429	0.011564	0.011732	0.011463	0.011665	0.011665	0.011530
0.011362	0.011429	0.011463	0.011866	0.011430	0.011463	0.011429
0.011463	0.011396	0.011396	0.011698	0.011631	0.011665	0.011396
0.011497	0.011564	0.011429	0.011497	0.011463	0.011329	0.011664
0.011429	0.011396	0.011396	0.011564	0.011396	0.011496	0.011732
0.011429	0.011773	0.011799	0.011548	0.011342	0.011502	0.011370
0.011280	0.011761	0.011341	0.011507	0.011353	0.011467	0.011485
0.011570	0.011324	0.011450	0.011324	0.011330	0.011462	0.011425
0.011475	0.011336	0.011483	0.011412	0.011431	0.011564	0.011631
0.011631	0.011497	0.011601	0.011603	0.011738	0.011767	0.011429
0.011664	0.011530	0.011396	0.011497	0.011295	0.011530	0.011329
0.011396	0.011396	0.011362	0.011329	0.011362	0.011329	0.011430
0.011410	0.011410	0.011403	0.011437	0.011573	0.011538	0.011538
0.011469	0.011464	0.011245	0.011240	0.011399	0.011568	0.011569
0.011363	0.011433	0.011666	0.011635	0.011565	0.011599	0.011530
0.011463	0.011530	0.011530	0.011429	0.012001	0.011597	0.011665
0.011698	0.011737	0.011401	0.011476	0.011362	0.011564	0.011530
0.011565	0.011597	0.011597	0.011597	0.011729	0.011732	0.011372
0.011263	0.011268	0.011367	0.011332	0.011436	0.011284	0.011604
0.011403	0.011586	0.011346	0.011397	0.011396	0.011471	0.011362
0.011329	0.011329	0.011396	0.011362	0.011564	0.011530	0.011463
0.011564	0.011631	0.011362	0.011362	0.011396	0.011396	0.011398
0.011466	0.011396	0.011396	0.011431	0.011564	0.011631	0.011631
0.011497	0.011601	0.011603	0.011738	0.011767	0.011429	0.011597
0.011530	0.011530	0.011365	0.011547	0.011477	0.011503	0.011376
0.011542	0.011744	0.011568	0.011434	0.011329	0.011530	0.011396
0.011497	0.011430	0.011329	0.011431	0.011530	0.011533	0.011430
0.011329	0.011263	0.011396	0.011329	0.011262	0.011366	0.011531
0.011430	0.011362	0.011396	0.011430	0.011396	0.011329	0.011530
0.011396	0.011429	0.011429	0.011362	0.011429	0.011597	0.011329
0.011362	0.011765	0.011362	0.011396	0.011362	0.011732	0.011274
0.011791	0.011273	0.011311	0.011543	0.011589	0.011563	0.011227
0.011270	0.011530	0.011631	0.011739	0.011599	0.011737	0.011867
0.011767	0.011804	0.011765	0.012017	0.011732	0.012606	0.011768
0.012507	0.012274	0.012409	0.012680	0.011731	0.011430	0.011732
0.012171	0.012106	0.011698	0.011597	0.011665	0.011665	0.011635
0.011530	0.011535	0.011597	0.011565	0.011935	0.012001	0.012002
0.011901	0.012002	0.012106	0.011872	0.012171	0.012178	0.012238
0.012239	0.012273	0.012221	0.012239	0.012241	0.011463	0.011766
0.011834	0.012337	0.011806	0.012068	0.012541	0.011604	0.011497
0.011463	0.011463	0.011934	0.012006	0.012045	0.011799	0.011465
S113	0.011631	0.011631	0.012034	0.011799	0.011799	0.011866
0.011705	0.011637	0.011866	0.011295	0.011362	0.011329	0.011295
0.011329	0.011496	0.011295	0.011329	0.011396	0.011362	0.011329
0.011295	0.011329	0.011429	0.011295	0.011329	0.011329	0.011671
0.011564	0.011329	0.011228	0.011463	0.011396	0.011362	0.011396
0.011362	0.011463	0.011362	0.011362	0.011396	0.011362	0.011530
0.011295	0.011362	0.011396	0.011329	0.011429	0.011463	0.011396
0.011362	0.011530	0.011430	0.011698	0.011362	0.011631	0.011362
0.011329	0.011429	0.011496	0.011631	0.011731	0.011429	0.011530
0.011396	0.011463	0.011362	0.011597	0.011832	0.011362	0.011429
0.011329	0.011429	0.011396	0.011463	0.011396	0.011396	0.011396
0.011295	0.011329	0.011295	0.011396	0.011362	0.011397	0.011463
0.011396	0.011362	0.011429	0.011329	0.011463	0.011295	0.011362
0.011530	0.011463	0.011698	0.011530	0.011362	0.011597	0.011362

0.011542	0.011770	0.011551	0.011255	0.011247	0.011391	0.011454
0.012085	0.011467	0.011266	0.011597	0.011597	0.011396	0.011496
0.011429	0.000000	0.011396	0.011362	0.011463	0.011396	0.011329
0.011295	0.011463	0.011463	0.011530	0.011429	0.011396	0.011329
0.011295	0.011295	0.011362	0.011329	0.011530	0.011497	0.011429
0.011530	0.011597	0.011329	0.011329	0.011362	0.011362	0.011364
0.011432	0.011362	0.011362	0.011240	0.011277	0.011271	0.011363
0.011365	0.011301	0.011318	0.011336	0.011264	0.011300	0.011330
0.011600	0.011370	0.011370	0.011296	0.011302	0.011430	0.011396
0.011464	0.011397	0.011396	0.011429	0.011362	0.011799	0.011597
0.011631	0.011497	0.011362	0.011463	0.011262	0.011496	0.011295
0.011362	0.011362	0.011329	0.011295	0.011329	0.011295	0.011396
0.011362	0.011463	0.011396	0.011295	0.011397	0.011497	0.011499
0.011497	0.011396	0.011295	0.011229	0.011362	0.011295	0.011228
0.011332	0.011497	0.011295	0.011497	0.011262	0.011362	0.011329
0.011430	0.011329	0.011362	0.011329	0.011362	0.011228	0.011362
0.011396	0.011329	0.011396	0.011329	0.011295	0.011396	0.011631
0.011732	0.011664	0.011732	0.011498	0.011732	0.011295	0.011329
0.011364	0.011626	0.011380	0.011266	0.011739	0.011231	0.011336
0.011739	0.011638	0.011496	0.011530	0.011436	0.011597	0.011564
0.011496	0.011664	0.011451	0.011349	0.011395	0.011396	0.011398
0.011564	0.011397	0.011601	0.011669	0.011530	0.011463	0.011704
0.011432	0.011671	0.011534	0.011533	0.011598	0.011262	0.011464
0.011429	0.011362	0.011262	0.011262	0.011329	0.011329	0.011396
0.011329	0.011396	0.011295	0.011396	0.011329	0.011329	0.011329
0.011329	0.011765	0.011664	0.011698	0.011228	0.011329	0.011362
0.011362	0.011329	0.011396	0.011429	0.011295	0.011295	0.011295
0.011832	0.011463	0.011463	0.011262	0.011362	0.011530	0.011295
0.011295	0.011362	0.011329	0.011497	0.011295	0.011429	0.011564
0.011496	0.011329	0.011329	0.011262	0.011262	0.011429	0.011362
0.011262	0.011362	0.011228	0.011262	0.011396	0.011530	0.011295
0.011262	0.011396	0.011329	0.011228	0.011564	0.011228	0.011396
0.011471	0.011295	0.011597	0.011429	0.011463	0.011329	0.011429
0.011295	0.011631	0.011228	0.011496	0.011295	0.011295	0.011228
0.011463	0.011665	0.011362	0.011429	0.011262	0.011664	0.011295
0.011295	0.011295	0.011329	0.011429	0.011329	0.011429	0.011496
0.011496	0.011463	0.011362	0.011463	0.011429	0.011295	0.011262
0.011295	0.011295	0.011362	0.011295	0.011262	0.011429	0.011295
0.011329	0.011329	0.011463	0.011496	0.011396	0.011329	0.011329
0.011396	0.011329	0.011429	0.011295	0.011429	0.011530	0.011832
0.011329	0.011732	0.011336	0.017823	0.011191	0.011427	0.011688
0.011648	0.011693	0.011427	0.011350	0.011664	0.011716	0.011303
0.011487	0.011487	0.011255	0.011311	0.011294	0.011356	0.011318
0.011482	0.011470	0.011468	0.011325	0.011463	0.011429	0.011500
0.011369	0.011463	0.011463	0.011429	0.011396	0.011396	0.011362
0.011396	0.011329	0.011362	0.011396	0.011362	0.011295	0.011262
0.011262	0.011396	0.011295	0.011396	0.011429	0.011463	0.011295
0.011396	0.011362	0.011698	0.011698	0.011430	0.011464	0.011430
0.011262	0.011262	0.011295	0.011396	0.011262	0.011497	0.011497
0.011530	0.011496	0.011463	0.011564	0.011732	0.011565	0.011396
0.011396	0.011463	0.011497	0.011329	0.011396	0.011362	0.011396
0.011463	0.011463	0.011497	0.011463	0.011363	0.011564	0.011530
0.011399	0.011295	0.011396	0.011497	0.011329	0.011530	0.011564
0.011530	0.011436	0.011597	0.011564	0.011496	0.011664	0.011295
0.011464	0.011363	0.011262	0.011262	0.011396	0.011295	0.011396
0.011429	0.011463	0.011295	0.011396	0.011362	0.011698	0.011698
0.011430	0.011464	0.011463	0.011396	0.012069	0.011524	0.011377
0.011517	0.011476	0.011587	0.011476	0.011585	0.011302	0.011474
0.012021	0.011302	0.011437	0.011233	0.011508	0.011570	0.011673
0.012046	0.011550	0.011635	0.011698	0.011564	0.011564	0.011601

0.011262	0.011429	0.011564	0.011530	0.011533	0.011530	0.011498
0.011362	0.011295	0.011329	0.011362	0.011262	0.011329	0.011228
0.011362	0.011396	0.011295	0.011262	0.011362	0.011295	0.011329
0.011329	0.011262	0.011329	0.011362	0.011262	0.011463	0.011496
0.011396	0.011463	0.011396	0.011396	0.011329	0.011731	0.011362
0.011408	0.011329	0.011564	0.011569	0.011429	0.011429	0.011429
0.011429	0.011362	0.011329	0.011362	0.011463	0.011463	0.011396
0.011396	0.011530	0.011698	0.011429	0.011631	0.011631	0.011496
0.011329	0.011396	0.011429	0.011832	0.011396	0.011429	0.011396
0.011429	0.011362	0.011362	0.011665	0.011597	0.011631	0.011362
0.011463	0.011530	0.011396	0.011463	0.011429	0.011295	0.011631
0.011396	0.011362	0.011362	0.011530	0.011362	0.011463	0.011698
0.011396	0.011773	0.011765	0.011548	0.011229	0.011468	0.011267
0.011225	0.011761	0.011271	0.011507	0.011255	0.011366	0.011379
0.011519	0.011126	0.011339	0.011192	0.011222	0.011395	0.011385
0.011362	0.011336	0.011448	0.011377	0.011397	0.011530	0.011597
0.011597	0.011464	0.011567	0.011569	0.011704	0.011732	0.011396
0.011631	0.011497	0.011362	0.011463	0.011262	0.011496	0.011295
0.011362	0.011362	0.011329	0.011295	0.011329	0.011295	0.011396
0.011375	0.011375	0.011368	0.011403	0.011539	0.011504	0.011504
0.011503	0.011430	0.011245	0.011240	0.011365	0.011535	0.011535
0.011329	0.011399	0.011632	0.011601	0.011531	0.011565	0.011429
0.011429	0.011295	0.011295	0.011396	0.011933	0.011564	0.011631
0.011665	0.011737	0.011367	0.011441	0.011329	0.011463	0.011429
0.011531	0.011496	0.011564	0.011564	0.011532	0.011631	0.011338
0.011229	0.011233	0.011332	0.011298	0.011402	0.011247	0.011570
0.011369	0.011499	0.011251	0.011360	0.011363	0.011437	0.011329
0.011295	0.011295	0.011362	0.011329	0.011530	0.011497	0.011429
0.011530	0.011597	0.011329	0.011329	0.011362	0.011362	0.011364
0.011432	0.011362	0.011362	0.011397	0.011530	0.011597	0.011597
0.011464	0.011567	0.011569	0.011704	0.011732	0.011396	0.011564
0.011429	0.011429	0.011331	0.011513	0.011442	0.011469	0.011340
0.011508	0.011653	0.011484	0.011400	0.011295	0.011497	0.011362
0.011463	0.011396	0.011295	0.011397	0.011497	0.011499	0.011396
0.011295	0.011229	0.011362	0.011295	0.011228	0.011332	0.011497
0.011396	0.011329	0.011362	0.011396	0.011362	0.011295	0.011497
0.011362	0.011396	0.011396	0.011329	0.011396	0.011631	0.011295
0.011329	0.011731	0.011329	0.011362	0.011329	0.011698	0.011274
0.011735	0.011273	0.011261	0.011503	0.011589	0.011491	0.011186
0.011218	0.011496	0.011597	0.011705	0.011565	0.011703	0.011834
0.011733	0.011770	0.011731	0.011983	0.011698	0.012505	0.011734
0.012473	0.012173	0.012307	0.012578	0.011698	0.011396	0.011698
0.012103	0.012072	0.011664	0.011564	0.011632	0.011632	0.011601
0.011496	0.011501	0.011564	0.011531	0.011901	0.011967	0.011968
0.011867	0.011968	0.012072	0.011838	0.012104	0.012110	0.012171
0.012172	0.012205	0.012152	0.012172	0.012173	0.011362	0.011699
0.011800	0.012303	0.011772	0.012034	0.012508	0.011570	0.011464
0.011429	0.011429	0.011900	0.011965	0.012045	0.011765	0.011431
S114	0.011564	0.011564	0.012101	0.011866	0.011866	0.011933
0.011773	0.011705	0.011933	0.011362	0.011429	0.011397	0.011362
0.011396	0.011564	0.011362	0.011396	0.011463	0.011429	0.011396
0.011362	0.011396	0.011496	0.011363	0.011396	0.011396	0.011739
0.011631	0.011396	0.011295	0.011530	0.011463	0.011429	0.011463
0.011429	0.011530	0.011429	0.011429	0.011463	0.011429	0.011597
0.011362	0.011429	0.011463	0.011396	0.011496	0.011530	0.011463
0.011429	0.011597	0.011497	0.011765	0.011429	0.011698	0.011429
0.011396	0.011497	0.011564	0.011698	0.011799	0.011496	0.011597
0.011463	0.011530	0.011429	0.011664	0.011900	0.011429	0.011496
0.011396	0.011496	0.011463	0.011530	0.011463	0.011463	0.011463
0.011362	0.011396	0.011362	0.011463	0.011429	0.011464	0.011530

0.011463	0.011429	0.011496	0.011396	0.011530	0.011362	0.011429
0.011597	0.011530	0.011765	0.011597	0.011429	0.011664	0.011429
0.011616	0.011845	0.011664	0.011334	0.011359	0.011465	0.011572
0.012195	0.011620	0.011390	0.011664	0.011664	0.011463	0.011564
0.011496	0.011396	0.000000	0.011429	0.011530	0.011463	0.011329
0.011362	0.011530	0.011664	0.011597	0.011496	0.011463	0.011396
0.011362	0.011362	0.011429	0.011396	0.011597	0.011564	0.011497
0.011597	0.011665	0.011396	0.011396	0.011430	0.011429	0.011432
0.011500	0.011429	0.011429	0.011312	0.011386	0.011341	0.011430
0.011433	0.011369	0.011429	0.011405	0.011332	0.011368	0.011398
0.011672	0.011439	0.011473	0.011363	0.011371	0.011497	0.011464
0.011531	0.011465	0.011464	0.011429	0.011295	0.011866	0.011665
0.011698	0.011564	0.011429	0.011530	0.011329	0.011564	0.011362
0.011429	0.011429	0.011396	0.011362	0.011396	0.011362	0.011463
0.011429	0.011530	0.011463	0.011362	0.011464	0.011564	0.011567
0.011564	0.011463	0.011362	0.011296	0.011429	0.011362	0.011295
0.011400	0.011564	0.011362	0.011564	0.011329	0.011430	0.011396
0.011497	0.011396	0.011429	0.011396	0.011429	0.011295	0.011429
0.011463	0.011396	0.011463	0.011396	0.011295	0.011463	0.011698
0.011799	0.011732	0.011799	0.011566	0.011799	0.011295	0.011396
0.011432	0.011747	0.011426	0.011335	0.011807	0.011299	0.011375
0.011807	0.011706	0.011564	0.011732	0.011367	0.011664	0.011631
0.011564	0.011732	0.011451	0.011349	0.011437	0.011463	0.011466
0.011631	0.011397	0.011669	0.011737	0.011530	0.011665	0.011704
0.011432	0.011705	0.011500	0.011601	0.011800	0.011329	0.011532
0.011496	0.011429	0.011329	0.011329	0.011396	0.011396	0.011463
0.011396	0.011463	0.011362	0.011463	0.011396	0.011396	0.011463
0.011396	0.011832	0.011731	0.011765	0.011295	0.011396	0.011429
0.011429	0.011396	0.011463	0.011496	0.011362	0.011362	0.011362
0.011899	0.011530	0.011530	0.011329	0.011429	0.011597	0.011362
0.011362	0.011429	0.011396	0.011564	0.011362	0.011496	0.011631
0.011564	0.011396	0.011396	0.011329	0.011329	0.011496	0.011429
0.011329	0.011429	0.011295	0.011329	0.011463	0.011598	0.011362
0.011329	0.011463	0.011396	0.011295	0.011631	0.011295	0.011463
0.011471	0.011362	0.011664	0.011497	0.011530	0.011396	0.011496
0.011362	0.011698	0.011295	0.011564	0.011362	0.011362	0.011295
0.011530	0.011732	0.011429	0.011496	0.011329	0.011731	0.011362
0.011362	0.011362	0.011396	0.011496	0.011396	0.011496	0.011564
0.011564	0.011530	0.011429	0.011530	0.011496	0.011362	0.011329
0.011362	0.011362	0.011429	0.011362	0.011329	0.011496	0.011362
0.011396	0.011396	0.011530	0.011564	0.011463	0.011396	0.011396
0.011463	0.011396	0.011496	0.011362	0.011496	0.011597	0.011899
0.011396	0.011799	0.011405	0.017936	0.011387	0.011549	0.011758
0.011718	0.011764	0.011532	0.011466	0.011939	0.011785	0.011546
0.011677	0.011732	0.011333	0.011395	0.011411	0.011504	0.011361
0.011623	0.011581	0.011579	0.011325	0.011530	0.011362	0.011500
0.011438	0.011530	0.011530	0.011497	0.011463	0.011463	0.011430
0.011463	0.011396	0.011429	0.011463	0.011430	0.011362	0.011262
0.011262	0.011463	0.011362	0.011463	0.011497	0.011530	0.011295
0.011463	0.011429	0.011765	0.011765	0.011497	0.011531	0.011564
0.011329	0.011329	0.011362	0.011463	0.011329	0.011564	0.011564
0.011597	0.011564	0.011530	0.011631	0.011799	0.011633	0.011463
0.011463	0.011530	0.011564	0.011396	0.011463	0.011429	0.011463
0.011531	0.011530	0.011564	0.011530	0.011431	0.011631	0.011597
0.011467	0.011362	0.011463	0.011564	0.011463	0.011597	0.011631
0.011732	0.011367	0.011664	0.011631	0.011564	0.011732	0.011363
0.011532	0.011430	0.011262	0.011262	0.011463	0.011362	0.011463
0.011497	0.011530	0.011295	0.011463	0.011429	0.011765	0.011765
0.011497	0.011531	0.011396	0.011329	0.012136	0.011560	0.011377
0.011587	0.011546	0.011622	0.011476	0.011620	0.011508	0.011543

0.012056	0.011302	0.011437	0.011406	0.011714	0.011638	0.011741
0.012046	0.011550	0.011703	0.011765	0.011631	0.011631	0.011668
0.011329	0.011497	0.011631	0.011597	0.011601	0.011597	0.011565
0.011429	0.011362	0.011396	0.011429	0.011329	0.011396	0.011295
0.011429	0.011463	0.011362	0.011329	0.011429	0.011362	0.011396
0.011396	0.011329	0.011396	0.011429	0.011329	0.011530	0.011564
0.011463	0.011530	0.011463	0.011463	0.011396	0.011799	0.011429
0.011443	0.011396	0.011631	0.011637	0.011497	0.011496	0.011496
0.011496	0.011429	0.011396	0.011429	0.011530	0.011530	0.011463
0.011463	0.011597	0.011765	0.011496	0.011698	0.011698	0.011564
0.011396	0.011463	0.011496	0.011900	0.011463	0.011497	0.011463
0.011496	0.011429	0.011429	0.011732	0.011664	0.011698	0.011429
0.011530	0.011597	0.011463	0.011530	0.011496	0.011362	0.011698
0.011463	0.011429	0.011429	0.011530	0.011429	0.011530	0.011765
0.011463	0.011841	0.011832	0.011670	0.011399	0.011536	0.011421
0.011390	0.011878	0.011376	0.011641	0.011451	0.011569	0.011591
0.011672	0.011389	0.011561	0.011390	0.011437	0.011596	0.011547
0.011550	0.011454	0.011518	0.011448	0.011465	0.011597	0.011665
0.011665	0.011531	0.011635	0.011636	0.011772	0.011802	0.011463
0.011698	0.011564	0.011429	0.011530	0.011329	0.011564	0.011362
0.011429	0.011429	0.011396	0.011362	0.011396	0.011362	0.011463
0.011444	0.011445	0.011437	0.011471	0.011608	0.011573	0.011572
0.011571	0.011497	0.011282	0.011312	0.011433	0.011602	0.011603
0.011396	0.011467	0.011700	0.011668	0.011598	0.011632	0.011362
0.011496	0.011496	0.011496	0.011463	0.012000	0.011631	0.011698
0.011732	0.011808	0.011435	0.011511	0.011396	0.011463	0.011362
0.011598	0.011496	0.011631	0.011631	0.011728	0.011631	0.011407
0.011297	0.011303	0.011401	0.011366	0.011470	0.011321	0.011638
0.011437	0.011455	0.011203	0.011322	0.011430	0.011505	0.011396
0.011362	0.011362	0.011429	0.011396	0.011597	0.011564	0.011497
0.011597	0.011665	0.011396	0.011396	0.011430	0.011429	0.011432
0.011500	0.011429	0.011429	0.011465	0.011597	0.011665	0.011665
0.011531	0.011635	0.011636	0.011772	0.011802	0.011463	0.011631
0.011362	0.011362	0.011398	0.011582	0.011512	0.011537	0.011411
0.011577	0.011789	0.011526	0.011468	0.011362	0.011564	0.011429
0.011530	0.011463	0.011362	0.011464	0.011564	0.011567	0.011463
0.011362	0.011296	0.011429	0.011362	0.011295	0.011400	0.011564
0.011463	0.011396	0.011429	0.011463	0.011430	0.011362	0.011564
0.011429	0.011463	0.011463	0.011396	0.011463	0.011698	0.011362
0.011396	0.011799	0.011396	0.011429	0.011396	0.011765	0.011403
0.011903	0.011400	0.011411	0.011624	0.011711	0.011598	0.011311
0.011375	0.011564	0.011664	0.011773	0.011632	0.011771	0.011901
0.011800	0.011838	0.011799	0.012051	0.011765	0.012573	0.011801
0.012540	0.012241	0.012375	0.012714	0.011765	0.011463	0.011766
0.012171	0.012140	0.011732	0.011631	0.011699	0.011699	0.011669
0.011564	0.011569	0.011631	0.011598	0.011968	0.012034	0.012036
0.011935	0.012035	0.012139	0.011906	0.012171	0.012178	0.012238
0.012239	0.012273	0.012220	0.012239	0.012240	0.011295	0.011766
0.011867	0.012371	0.011840	0.012101	0.012575	0.011637	0.011531
0.011496	0.011496	0.011968	0.011924	0.012116	0.011832	0.011498
S115	0.011664	0.011664	0.012001	0.011765	0.011765	0.011832
0.011671	0.011603	0.011832	0.011262	0.011329	0.011296	0.011262
0.011295	0.011463	0.011262	0.011295	0.011362	0.011329	0.011295
0.011262	0.011295	0.011396	0.011262	0.011295	0.011295	0.011637
0.011530	0.011295	0.011194	0.011429	0.011362	0.011329	0.011362
0.011329	0.011429	0.011329	0.011329	0.011362	0.011329	0.011496
0.011262	0.011329	0.011362	0.011295	0.011396	0.011429	0.011362
0.011329	0.011497	0.011396	0.011664	0.011329	0.011597	0.011329
0.011295	0.011396	0.011463	0.011597	0.011698	0.011396	0.011496
0.011362	0.011429	0.011329	0.011564	0.011799	0.011329	0.011396

0.011295	0.011396	0.011362	0.011429	0.011362	0.011362	0.011362
0.011262	0.011295	0.011262	0.011362	0.011329	0.011363	0.011429
0.011362	0.011329	0.011396	0.011295	0.011429	0.011262	0.011329
0.011496	0.011429	0.011664	0.011497	0.011329	0.011564	0.011329
0.011505	0.011733	0.011551	0.011256	0.011247	0.011355	0.011454
0.012085	0.011518	0.011266	0.011564	0.011564	0.011362	0.011463
0.011396	0.011362	0.011429	0.000000	0.011429	0.011362	0.011362
0.011329	0.011429	0.011631	0.011496	0.011396	0.011362	0.011295
0.011262	0.011262	0.011329	0.011295	0.011497	0.011463	0.011396
0.011497	0.011564	0.011295	0.011295	0.011329	0.011329	0.011330
0.011398	0.011329	0.011329	0.011204	0.011277	0.011236	0.011329
0.011331	0.011266	0.011318	0.011302	0.011230	0.011266	0.011297
0.011564	0.011336	0.011370	0.011262	0.011268	0.011397	0.011363
0.011430	0.011363	0.011363	0.011463	0.011396	0.011765	0.011564
0.011597	0.011463	0.011329	0.011430	0.011228	0.011463	0.011262
0.011329	0.011329	0.011295	0.011262	0.011295	0.011262	0.011362
0.011329	0.011430	0.011362	0.011262	0.011363	0.011463	0.011466
0.011463	0.011362	0.011262	0.011195	0.011329	0.011262	0.011195
0.011298	0.011463	0.011262	0.011463	0.011228	0.011329	0.011295
0.011396	0.011295	0.011329	0.011295	0.011329	0.011195	0.011329
0.011362	0.011295	0.011362	0.011295	0.011329	0.011362	0.011597
0.011698	0.011631	0.011698	0.011465	0.011698	0.011329	0.011295
0.011330	0.011586	0.011472	0.011232	0.011705	0.011197	0.011336
0.011705	0.011604	0.011496	0.011698	0.011402	0.011564	0.011530
0.011463	0.011631	0.011486	0.011307	0.011312	0.011362	0.011432
0.011530	0.011431	0.011567	0.011635	0.011564	0.011631	0.011738
0.011466	0.011637	0.011568	0.011500	0.011767	0.011228	0.011431
0.011396	0.011329	0.011228	0.011228	0.011295	0.011295	0.011362
0.011295	0.011362	0.011262	0.011362	0.011295	0.011295	0.011362
0.011295	0.011731	0.011631	0.011664	0.011194	0.011295	0.011329
0.011329	0.011295	0.011362	0.011396	0.011262	0.011262	0.011262
0.011799	0.011429	0.011429	0.011228	0.011329	0.011497	0.011262
0.011262	0.011329	0.011295	0.011463	0.011262	0.011396	0.011530
0.011463	0.011295	0.011295	0.011228	0.011228	0.011396	0.011329
0.011228	0.011329	0.011195	0.011228	0.011362	0.011497	0.011262
0.011228	0.011362	0.011295	0.011195	0.011530	0.011195	0.011362
0.011437	0.011262	0.011564	0.011396	0.011429	0.011295	0.011396
0.011262	0.011597	0.011194	0.011463	0.011262	0.011262	0.011194
0.011429	0.011631	0.011329	0.011396	0.011228	0.011631	0.011262
0.011262	0.011262	0.011295	0.011396	0.011295	0.011396	0.011463
0.011463	0.011429	0.011329	0.011429	0.011396	0.011262	0.011228
0.011262	0.011262	0.011329	0.011262	0.011228	0.011396	0.011262
0.011295	0.011295	0.011429	0.011463	0.011362	0.011295	0.011295
0.011362	0.011295	0.011396	0.011262	0.011396	0.011496	0.011799
0.011295	0.011698	0.011302	0.017823	0.011322	0.011427	0.011653
0.011614	0.011658	0.011374	0.011292	0.011774	0.011682	0.011425
0.011534	0.011610	0.011216	0.011270	0.011294	0.011356	0.011318
0.011529	0.011470	0.011468	0.011259	0.011429	0.011463	0.011466
0.011335	0.011429	0.011429	0.011396	0.011362	0.011362	0.011329
0.011362	0.011295	0.011329	0.011362	0.011329	0.011262	0.011295
0.011295	0.011362	0.011262	0.011362	0.011396	0.011429	0.011329
0.011362	0.011329	0.011665	0.011665	0.011396	0.011430	0.011463
0.011228	0.011228	0.011262	0.011362	0.011228	0.011463	0.011463
0.011497	0.011496	0.011429	0.011530	0.011698	0.011532	0.011362
0.011362	0.011429	0.011463	0.011295	0.011362	0.011329	0.011362
0.011430	0.011430	0.011463	0.011430	0.011330	0.011530	0.011497
0.011365	0.011262	0.011362	0.011463	0.011362	0.011497	0.011530
0.011698	0.011402	0.011564	0.011530	0.011463	0.011631	0.011262
0.011431	0.011329	0.011295	0.011295	0.011362	0.011262	0.011362
0.011396	0.011429	0.011329	0.011362	0.011329	0.011665	0.011665

0.011396	0.011430	0.011496	0.011429	0.012069	0.011453	0.011412
0.011482	0.011442	0.011552	0.011441	0.011550	0.011474	0.011439
0.012021	0.011337	0.011471	0.011406	0.011680	0.011536	0.011639
0.012046	0.011475	0.011601	0.011664	0.011530	0.011530	0.011567
0.011228	0.011396	0.011530	0.011496	0.011499	0.011496	0.011464
0.011329	0.011262	0.011295	0.011329	0.011228	0.011295	0.011194
0.011329	0.011362	0.011262	0.011228	0.011329	0.011262	0.011295
0.011295	0.011228	0.011295	0.011329	0.011228	0.011429	0.011463
0.011362	0.011429	0.011362	0.011362	0.011295	0.011698	0.011329
0.011373	0.011295	0.011530	0.011535	0.011396	0.011396	0.011396
0.011396	0.011329	0.011295	0.011329	0.011429	0.011429	0.011362
0.011362	0.011496	0.011664	0.011396	0.011597	0.011597	0.011463
0.011295	0.011362	0.011396	0.011799	0.011362	0.011396	0.011362
0.011396	0.011329	0.011329	0.011631	0.011564	0.011597	0.011329
0.011429	0.011496	0.011362	0.011429	0.011396	0.011262	0.011597
0.011362	0.011329	0.011329	0.011496	0.011329	0.011429	0.011664
0.011362	0.011739	0.011732	0.011548	0.011286	0.011434	0.011318
0.011280	0.011761	0.011271	0.011507	0.011304	0.011416	0.011432
0.011519	0.011258	0.011394	0.011258	0.011276	0.011462	0.011425
0.011437	0.011336	0.011412	0.011342	0.011364	0.011497	0.011564
0.011564	0.011430	0.011533	0.011535	0.011670	0.011661	0.011362
0.011597	0.011463	0.011329	0.011430	0.011228	0.011463	0.011262
0.011329	0.011329	0.011295	0.011262	0.011295	0.011262	0.011362
0.011340	0.011340	0.011334	0.011368	0.011505	0.011470	0.011470
0.011469	0.011396	0.011208	0.011204	0.011331	0.011501	0.011501
0.011296	0.011365	0.011599	0.011567	0.011497	0.011531	0.011463
0.011396	0.011463	0.011463	0.011362	0.011933	0.011530	0.011597
0.011631	0.011701	0.011333	0.011407	0.011295	0.011497	0.011463
0.011497	0.011530	0.011530	0.011530	0.011689	0.011664	0.011303
0.011195	0.011199	0.011298	0.011264	0.011368	0.011209	0.011536
0.011335	0.011455	0.011203	0.011360	0.011329	0.011402	0.011295
0.011262	0.011262	0.011329	0.011295	0.011497	0.011463	0.011396
0.011497	0.011564	0.011295	0.011295	0.011329	0.011329	0.011330
0.011398	0.011329	0.011329	0.011364	0.011497	0.011564	0.011564
0.011430	0.011533	0.011535	0.011670	0.011661	0.011362	0.011530
0.011463	0.011463	0.011297	0.011478	0.011408	0.011435	0.011305
0.011473	0.011698	0.011484	0.011366	0.011262	0.011463	0.011329
0.011430	0.011362	0.011262	0.011363	0.011463	0.011466	0.011362
0.011262	0.011195	0.011329	0.011262	0.011195	0.011298	0.011463
0.011362	0.011295	0.011329	0.011362	0.011329	0.011262	0.011463
0.011329	0.011362	0.011362	0.011295	0.011362	0.011597	0.011262
0.011295	0.011698	0.011295	0.011329	0.011295	0.011664	0.011232
0.011735	0.011273	0.011311	0.011463	0.011549	0.011491	0.011186
0.011270	0.011463	0.011564	0.011671	0.011531	0.011669	0.011800
0.011699	0.011737	0.011698	0.011949	0.011664	0.012539	0.011700
0.012439	0.012207	0.012341	0.012612	0.011664	0.011362	0.011665
0.012103	0.012038	0.011631	0.011530	0.011598	0.011598	0.011534
0.011463	0.011467	0.011530	0.011497	0.011867	0.011933	0.011935
0.011834	0.011934	0.012038	0.011804	0.012104	0.012110	0.012171
0.012172	0.012205	0.012152	0.012172	0.012173	0.011396	0.011699
0.011766	0.012270	0.011772	0.012001	0.012474	0.011536	0.011430
0.011396	0.011396	0.011867	0.011883	0.012010	0.011732	0.011397
S116	0.011765	0.011765	0.012068	0.011832	0.011832	0.011900
0.011773	0.011705	0.011900	0.011362	0.011429	0.011397	0.011362
0.011396	0.011564	0.011362	0.011396	0.011463	0.011429	0.011396
0.011362	0.011396	0.011496	0.011363	0.011396	0.011396	0.011739
0.011631	0.011396	0.011295	0.011530	0.011463	0.011430	0.011463
0.011429	0.011530	0.011429	0.011429	0.011463	0.011429	0.011597
0.011362	0.011429	0.011463	0.011396	0.011496	0.011530	0.011463
0.011429	0.011597	0.011497	0.011765	0.011429	0.011698	0.011429

0.011396	0.011497	0.011497	0.011698	0.011799	0.011497	0.011597
0.011463	0.011530	0.011430	0.011664	0.011900	0.011429	0.011497
0.011396	0.011496	0.011463	0.011530	0.011463	0.011463	0.011463
0.011362	0.011396	0.011362	0.011463	0.011429	0.011464	0.011530
0.011463	0.011429	0.011497	0.011396	0.011530	0.011362	0.011429
0.011597	0.011530	0.011765	0.011597	0.011429	0.011664	0.011429
0.011542	0.011770	0.011626	0.011295	0.011321	0.011428	0.011493
0.012158	0.011671	0.011307	0.011664	0.011664	0.011463	0.011564
0.011497	0.011463	0.011530	0.011429	0.000000	0.011463	0.011463
0.011429	0.011530	0.011732	0.011597	0.011497	0.011463	0.011396
0.011362	0.011362	0.011430	0.011396	0.011597	0.011564	0.011497
0.011597	0.011665	0.011396	0.011396	0.011430	0.011429	0.011432
0.011500	0.011430	0.011430	0.011312	0.011386	0.011341	0.011430
0.011433	0.011370	0.011392	0.011405	0.011332	0.011369	0.011398
0.011672	0.011439	0.011474	0.011363	0.011371	0.011498	0.011464
0.011531	0.011465	0.011464	0.011564	0.011496	0.011866	0.011665
0.011698	0.011564	0.011429	0.011530	0.011329	0.011564	0.011362
0.011429	0.011429	0.011396	0.011362	0.011396	0.011362	0.011463
0.011430	0.011530	0.011463	0.011362	0.011465	0.011564	0.011567
0.011564	0.011463	0.011362	0.011296	0.011430	0.011362	0.011295
0.011400	0.011564	0.011362	0.011564	0.011329	0.011430	0.011396
0.011497	0.011396	0.011430	0.011396	0.011430	0.011295	0.011430
0.011463	0.011396	0.011463	0.011396	0.011430	0.011463	0.011698
0.011799	0.011732	0.011799	0.011532	0.011799	0.011429	0.011396
0.011432	0.011667	0.011563	0.011300	0.011773	0.011265	0.011414
0.011807	0.011706	0.011597	0.011799	0.011504	0.011665	0.011631
0.011564	0.011732	0.011558	0.011349	0.011354	0.011463	0.011534
0.011631	0.011532	0.011669	0.011737	0.011665	0.011732	0.011839
0.011568	0.011739	0.011670	0.011601	0.011868	0.011329	0.011532
0.011496	0.011429	0.011262	0.011329	0.011396	0.011396	0.011463
0.011396	0.011463	0.011362	0.011463	0.011396	0.011396	0.011463
0.011396	0.011832	0.011732	0.011765	0.011295	0.011396	0.011430
0.011429	0.011396	0.011463	0.011497	0.011362	0.011362	0.011362
0.011900	0.011530	0.011530	0.011329	0.011429	0.011597	0.011362
0.011362	0.011429	0.011396	0.011564	0.011362	0.011497	0.011631
0.011564	0.011396	0.011396	0.011329	0.011329	0.011497	0.011429
0.011329	0.011429	0.011295	0.011329	0.011463	0.011598	0.011362
0.011329	0.011463	0.011396	0.011295	0.011631	0.011295	0.011463
0.011540	0.011362	0.011664	0.011497	0.011530	0.011396	0.011497
0.011362	0.011698	0.011295	0.011564	0.011362	0.011362	0.011295
0.011530	0.011698	0.011430	0.011497	0.011329	0.011732	0.011362
0.011362	0.011362	0.011396	0.011497	0.011396	0.011497	0.011564
0.011564	0.011530	0.011429	0.011530	0.011496	0.011362	0.011329
0.011362	0.011362	0.011430	0.011362	0.011329	0.011497	0.011362
0.011396	0.011396	0.011530	0.011564	0.011463	0.011396	0.011396
0.011463	0.011396	0.011496	0.011362	0.011497	0.011597	0.011900
0.011396	0.011799	0.011371	0.017899	0.011322	0.011427	0.011723
0.011683	0.011729	0.011374	0.011408	0.011885	0.011751	0.011425
0.011677	0.011610	0.011255	0.011312	0.011373	0.011455	0.011318
0.011670	0.011544	0.011542	0.011325	0.011530	0.011564	0.011568
0.011438	0.011463	0.011530	0.011497	0.011463	0.011463	0.011430
0.011463	0.011396	0.011429	0.011463	0.011430	0.011362	0.011396
0.011396	0.011463	0.011362	0.011463	0.011497	0.011530	0.011429
0.011463	0.011430	0.011766	0.011766	0.011497	0.011531	0.011564
0.011329	0.011329	0.011362	0.011463	0.011329	0.011564	0.011564
0.011597	0.011597	0.011530	0.011631	0.011799	0.011633	0.011463
0.011463	0.011530	0.011564	0.011396	0.011463	0.011430	0.011463
0.011531	0.011530	0.011564	0.011531	0.011431	0.011631	0.011597
0.011467	0.011362	0.011463	0.011564	0.011463	0.011598	0.011631
0.011799	0.011504	0.011665	0.011631	0.011564	0.011732	0.011363

0.011532	0.011430	0.011396	0.011396	0.011463	0.011362	0.011463
0.011497	0.011530	0.011429	0.011463	0.011430	0.011766	0.011766
0.011497	0.011531	0.011597	0.011530	0.012170	0.011525	0.011482
0.011588	0.011511	0.011622	0.011545	0.011620	0.011577	0.011508
0.012125	0.011441	0.011574	0.011510	0.011783	0.011638	0.011741
0.012148	0.011550	0.011703	0.011765	0.011631	0.011631	0.011668
0.011329	0.011497	0.011631	0.011597	0.011601	0.011597	0.011565
0.011429	0.011362	0.011396	0.011429	0.011329	0.011396	0.011295
0.011429	0.011463	0.011362	0.011329	0.011429	0.011362	0.011396
0.011396	0.011329	0.011396	0.011430	0.011329	0.011530	0.011564
0.011463	0.011530	0.011463	0.011463	0.011396	0.011799	0.011429
0.011478	0.011396	0.011631	0.011637	0.011497	0.011496	0.011497
0.011497	0.011430	0.011396	0.011430	0.011530	0.011530	0.011463
0.011463	0.011597	0.011765	0.011496	0.011698	0.011698	0.011564
0.011396	0.011463	0.011497	0.011900	0.011463	0.011497	0.011463
0.011497	0.011429	0.011429	0.011732	0.011665	0.011698	0.011429
0.011530	0.011597	0.011463	0.011530	0.011496	0.011362	0.011698
0.011463	0.011429	0.011429	0.011597	0.011429	0.011530	0.011765
0.011463	0.011841	0.011832	0.011589	0.011286	0.011536	0.011473
0.011335	0.011800	0.011341	0.011552	0.011353	0.011468	0.011538
0.011621	0.011258	0.011506	0.011391	0.011384	0.011529	0.011466
0.011475	0.011375	0.011448	0.011412	0.011465	0.011597	0.011665
0.011665	0.011531	0.011635	0.011636	0.011772	0.011802	0.011463
0.011698	0.011564	0.011429	0.011530	0.011329	0.011564	0.011362
0.011429	0.011429	0.011396	0.011362	0.011396	0.011362	0.011463
0.011410	0.011445	0.011437	0.011471	0.011608	0.011573	0.011572
0.011571	0.011497	0.011319	0.011312	0.011433	0.011602	0.011603
0.011397	0.011467	0.011700	0.011669	0.011598	0.011632	0.011564
0.011496	0.011564	0.011564	0.011463	0.012034	0.011631	0.011698
0.011732	0.011808	0.011435	0.011511	0.011396	0.011597	0.011564
0.011598	0.011631	0.011631	0.011631	0.011768	0.011765	0.011407
0.011297	0.011268	0.011401	0.011366	0.011470	0.011284	0.011638
0.011403	0.011499	0.011203	0.011397	0.011430	0.011505	0.011396
0.011362	0.011362	0.011430	0.011396	0.011597	0.011564	0.011497
0.011597	0.011665	0.011396	0.011396	0.011430	0.011429	0.011432
0.011500	0.011430	0.011430	0.011465	0.011597	0.011665	0.011665
0.011531	0.011635	0.011636	0.011772	0.011802	0.011463	0.011631
0.011564	0.011564	0.011398	0.011547	0.011512	0.011537	0.011376
0.011577	0.011744	0.011526	0.011468	0.011362	0.011564	0.011430
0.011530	0.011463	0.011362	0.011465	0.011564	0.011567	0.011463
0.011362	0.011296	0.011430	0.011362	0.011295	0.011400	0.011564
0.011463	0.011396	0.011429	0.011463	0.011430	0.011362	0.011564
0.011429	0.011463	0.011463	0.011396	0.011463	0.011698	0.011362
0.011396	0.011799	0.011396	0.011429	0.011396	0.011765	0.011317
0.011791	0.011315	0.011461	0.011543	0.011630	0.011527	0.011269
0.011428	0.011564	0.011664	0.011773	0.011633	0.011737	0.011901
0.011801	0.011838	0.011799	0.012017	0.011732	0.012607	0.011802
0.012507	0.012275	0.012409	0.012714	0.011765	0.011396	0.011766
0.012205	0.012106	0.011732	0.011631	0.011699	0.011699	0.011669
0.011564	0.011535	0.011631	0.011598	0.011968	0.012034	0.012036
0.011935	0.012036	0.012139	0.011872	0.012205	0.012212	0.012272
0.012273	0.012307	0.012221	0.012273	0.012275	0.011496	0.011800
0.011868	0.012371	0.011874	0.012102	0.012575	0.011638	0.011531
0.011496	0.011496	0.011968	0.011883	0.012116	0.011832	0.011498
S117	0.011698	0.011698	0.012034	0.011799	0.011799	0.011866
0.011705	0.011637	0.011866	0.011295	0.011362	0.011329	0.011295
0.011329	0.011496	0.011295	0.011329	0.011396	0.011362	0.011329
0.011295	0.011329	0.011429	0.011295	0.011262	0.011329	0.011671
0.011564	0.011329	0.011228	0.011463	0.011295	0.011362	0.011329
0.011362	0.011396	0.011362	0.011362	0.011396	0.011362	0.011530

0.011295	0.011362	0.011396	0.011329	0.011429	0.011463	0.011396
0.011362	0.011530	0.011430	0.011698	0.011362	0.011564	0.011362
0.011329	0.011429	0.011497	0.011564	0.011732	0.011429	0.011530
0.011329	0.011396	0.011362	0.011530	0.011832	0.011362	0.011429
0.011329	0.011429	0.011396	0.011463	0.011396	0.011396	0.011396
0.011295	0.011329	0.011295	0.011396	0.011362	0.011363	0.011463
0.011396	0.011362	0.011362	0.011329	0.011463	0.011228	0.011362
0.011530	0.011463	0.011698	0.011530	0.011362	0.011530	0.011362
0.011505	0.011733	0.011551	0.011256	0.011247	0.011355	0.011454
0.012085	0.011518	0.011266	0.011597	0.011597	0.011262	0.011497
0.011429	0.011396	0.011463	0.011362	0.011463	0.000000	0.011396
0.011362	0.011463	0.011664	0.011530	0.011429	0.011396	0.011262
0.011295	0.011295	0.011295	0.011329	0.011530	0.011497	0.011429
0.011530	0.011597	0.011329	0.011329	0.011362	0.011362	0.011364
0.011432	0.011362	0.011362	0.011204	0.011313	0.011271	0.011363
0.011365	0.011301	0.011318	0.011336	0.011264	0.011300	0.011330
0.011600	0.011370	0.011336	0.011296	0.011302	0.011430	0.011396
0.011464	0.011397	0.011396	0.011496	0.011429	0.011732	0.011598
0.011631	0.011497	0.011362	0.011463	0.011262	0.011496	0.011295
0.011362	0.011362	0.011329	0.011295	0.011329	0.011295	0.011396
0.011295	0.011396	0.011396	0.011295	0.011397	0.011497	0.011499
0.011430	0.011396	0.011295	0.011229	0.011362	0.011295	0.011228
0.011332	0.011430	0.011228	0.011430	0.011262	0.011362	0.011329
0.011362	0.011329	0.011295	0.011329	0.011362	0.011228	0.011295
0.011329	0.011329	0.011396	0.011329	0.011362	0.011396	0.011631
0.011732	0.011664	0.011732	0.011465	0.011732	0.011362	0.011329
0.011263	0.011586	0.011472	0.011232	0.011739	0.011231	0.011336
0.011739	0.011638	0.011530	0.011732	0.011436	0.011597	0.011497
0.011496	0.011664	0.011522	0.011390	0.011395	0.011396	0.011466
0.011564	0.011465	0.011601	0.011601	0.011597	0.011665	0.011772
0.011500	0.011603	0.011602	0.011534	0.011800	0.011262	0.011465
0.011362	0.011362	0.011262	0.011262	0.011262	0.011329	0.011396
0.011262	0.011329	0.011228	0.011396	0.011262	0.011329	0.011396
0.011329	0.011765	0.011664	0.011698	0.011228	0.011329	0.011362
0.011362	0.011329	0.011396	0.011429	0.011295	0.011295	0.011295
0.011832	0.011463	0.011463	0.011262	0.011362	0.011463	0.011295
0.011295	0.011362	0.011329	0.011429	0.011295	0.011429	0.011496
0.011496	0.011329	0.011329	0.011262	0.011262	0.011429	0.011362
0.011262	0.011362	0.011228	0.011262	0.011396	0.011530	0.011295
0.011262	0.011396	0.011329	0.011228	0.011564	0.011228	0.011396
0.011402	0.011295	0.011597	0.011430	0.011463	0.011329	0.011429
0.011295	0.011564	0.011228	0.011497	0.011295	0.011295	0.011228
0.011396	0.011665	0.011362	0.011429	0.011262	0.011664	0.011295
0.011295	0.011295	0.011329	0.011362	0.011329	0.011429	0.011496
0.011497	0.011463	0.011362	0.011463	0.011362	0.011295	0.011262
0.011295	0.011295	0.011362	0.011295	0.011194	0.011429	0.011295
0.011329	0.011329	0.011463	0.011496	0.011396	0.011262	0.011329
0.011396	0.011329	0.011429	0.011295	0.011429	0.011530	0.011832
0.011329	0.011732	0.011302	0.017823	0.011257	0.011488	0.011653
0.011614	0.011658	0.011374	0.011292	0.011774	0.011682	0.011425
0.011534	0.011671	0.011216	0.011270	0.011255	0.011406	0.011318
0.011529	0.011470	0.011431	0.011259	0.011463	0.011497	0.011500
0.011301	0.011463	0.011463	0.011429	0.011396	0.011396	0.011363
0.011396	0.011329	0.011362	0.011396	0.011362	0.011295	0.011329
0.011329	0.011396	0.011295	0.011396	0.011430	0.011463	0.011362
0.011396	0.011362	0.011698	0.011698	0.011430	0.011464	0.011497
0.011262	0.011262	0.011295	0.011329	0.011262	0.011497	0.011497
0.011530	0.011530	0.011463	0.011564	0.011732	0.011565	0.011329
0.011396	0.011463	0.011429	0.011329	0.011396	0.011362	0.011396
0.011463	0.011463	0.011497	0.011396	0.011296	0.011564	0.011463

0.011399	0.011295	0.011396	0.011497	0.011396	0.011530	0.011564
0.011732	0.011436	0.011597	0.011497	0.011496	0.011664	0.011295
0.011464	0.011363	0.011329	0.011329	0.011396	0.011295	0.011396
0.011430	0.011463	0.011362	0.011396	0.011362	0.011698	0.011698
0.011430	0.011464	0.011530	0.011463	0.012103	0.011524	0.011447
0.011517	0.011476	0.011587	0.011476	0.011585	0.011508	0.011474
0.012056	0.011372	0.011505	0.011441	0.011714	0.011570	0.011604
0.012080	0.011550	0.011635	0.011698	0.011564	0.011564	0.011601
0.011262	0.011362	0.011564	0.011530	0.011533	0.011530	0.011464
0.011362	0.011228	0.011262	0.011362	0.011194	0.011329	0.011228
0.011362	0.011396	0.011295	0.011262	0.011362	0.011295	0.011329
0.011329	0.011262	0.011329	0.011362	0.011262	0.011396	0.011496
0.011396	0.011463	0.011396	0.011396	0.011329	0.011732	0.011362
0.011408	0.011329	0.011564	0.011569	0.011362	0.011429	0.011429
0.011429	0.011295	0.011329	0.011295	0.011463	0.011463	0.011396
0.011396	0.011530	0.011698	0.011429	0.011631	0.011631	0.011496
0.011329	0.011396	0.011429	0.011832	0.011396	0.011362	0.011396
0.011429	0.011362	0.011362	0.011665	0.011530	0.011631	0.011362
0.011463	0.011530	0.011396	0.011463	0.011429	0.011295	0.011631
0.011396	0.011362	0.011362	0.011530	0.011362	0.011463	0.011698
0.011396	0.011773	0.011765	0.011548	0.011286	0.011468	0.011267
0.011335	0.011761	0.011271	0.011507	0.011304	0.011467	0.011432
0.011519	0.011258	0.011394	0.011258	0.011276	0.011462	0.011385
0.011399	0.011257	0.011412	0.011342	0.011397	0.011530	0.011597
0.011597	0.011464	0.011567	0.011569	0.011704	0.011696	0.011396
0.011631	0.011497	0.011362	0.011463	0.011262	0.011496	0.011295
0.011362	0.011362	0.011329	0.011295	0.011329	0.011295	0.011396
0.011375	0.011375	0.011368	0.011403	0.011539	0.011436	0.011504
0.011503	0.011430	0.011245	0.011240	0.011365	0.011535	0.011535
0.011329	0.011399	0.011632	0.011601	0.011531	0.011565	0.011496
0.011429	0.011496	0.011496	0.011396	0.011967	0.011564	0.011631
0.011665	0.011737	0.011367	0.011441	0.011329	0.011530	0.011497
0.011531	0.011564	0.011564	0.011564	0.011728	0.011698	0.011338
0.011229	0.011233	0.011333	0.011298	0.011402	0.011247	0.011570
0.011369	0.011455	0.011203	0.011397	0.011363	0.011437	0.011262
0.011295	0.011295	0.011295	0.011329	0.011530	0.011497	0.011429
0.011530	0.011597	0.011329	0.011329	0.011362	0.011362	0.011364
0.011432	0.011362	0.011362	0.011397	0.011530	0.011597	0.011597
0.011464	0.011567	0.011569	0.011704	0.011696	0.011396	0.011564
0.011497	0.011497	0.011331	0.011513	0.011442	0.011469	0.011305
0.011404	0.011607	0.011484	0.011400	0.011228	0.011430	0.011295
0.011396	0.011396	0.011295	0.011397	0.011497	0.011499	0.011396
0.011295	0.011229	0.011362	0.011295	0.011228	0.011332	0.011430
0.011396	0.011329	0.011362	0.011396	0.011362	0.011295	0.011497
0.011362	0.011396	0.011396	0.011329	0.011396	0.011631	0.011295
0.011329	0.011732	0.011329	0.011295	0.011329	0.011698	0.011274
0.011735	0.011273	0.011311	0.011503	0.011589	0.011491	0.011227
0.011270	0.011496	0.011597	0.011705	0.011531	0.011669	0.011834
0.011733	0.011770	0.011732	0.011949	0.011698	0.012573	0.011734
0.012473	0.012207	0.012375	0.012613	0.011698	0.011396	0.011699
0.012137	0.012038	0.011664	0.011564	0.011632	0.011632	0.011601
0.011496	0.011467	0.011564	0.011531	0.011901	0.011967	0.011968
0.011868	0.011968	0.012038	0.011804	0.012138	0.012144	0.012205
0.012205	0.012239	0.012152	0.012206	0.012173	0.011429	0.011732
0.011800	0.012303	0.011772	0.012034	0.012508	0.011536	0.011464
0.011429	0.011429	0.011900	0.011965	0.012045	0.011698	0.011397
S118 0.011564	0.011564	0.012034	0.011799	0.011799	0.011866	
0.011637	0.011569	0.011866	0.011295	0.011362	0.011329	0.011295
0.011329	0.011496	0.011295	0.011329	0.011396	0.011362	0.011329
0.011295	0.011329	0.011429	0.011295	0.011329	0.011329	0.011603

0.011564	0.011329	0.011228	0.011463	0.011396	0.011362	0.011396
0.011362	0.011463	0.011362	0.011362	0.011396	0.011362	0.011530
0.011295	0.011362	0.011396	0.011329	0.011429	0.011463	0.011396
0.011362	0.011530	0.011430	0.011698	0.011362	0.011631	0.011362
0.011329	0.011429	0.011496	0.011631	0.011731	0.011429	0.011530
0.011396	0.011463	0.011362	0.011597	0.011832	0.011362	0.011429
0.011329	0.011429	0.011396	0.011463	0.011396	0.011396	0.011396
0.011295	0.011329	0.011295	0.011396	0.011362	0.011397	0.011463
0.011396	0.011362	0.011429	0.011329	0.011463	0.011295	0.011362
0.011530	0.011463	0.011698	0.011530	0.011362	0.011597	0.011362
0.011468	0.011695	0.011513	0.011177	0.011284	0.011391	0.011415
0.012121	0.011467	0.011225	0.011597	0.011597	0.011396	0.011496
0.011429	0.011329	0.011329	0.011362	0.011463	0.011396	0.000000
0.011094	0.011463	0.011597	0.011530	0.011429	0.011396	0.011329
0.011295	0.011295	0.011362	0.011329	0.011530	0.011497	0.011429
0.011530	0.011597	0.011329	0.011329	0.011362	0.011362	0.011364
0.011432	0.011362	0.011362	0.011240	0.011313	0.011271	0.011363
0.011365	0.011301	0.011355	0.011336	0.011264	0.011300	0.011330
0.011600	0.011370	0.011404	0.011296	0.011302	0.011430	0.011396
0.011464	0.011397	0.011396	0.011228	0.011295	0.011799	0.011597
0.011631	0.011497	0.011362	0.011463	0.011262	0.011496	0.011295
0.011362	0.011362	0.011329	0.011295	0.011329	0.011295	0.011396
0.011362	0.011463	0.011396	0.011295	0.011397	0.011497	0.011499
0.011497	0.011396	0.011295	0.011229	0.011362	0.011295	0.011228
0.011332	0.011497	0.011295	0.011497	0.011262	0.011362	0.011329
0.011429	0.011262	0.011362	0.011329	0.011362	0.011228	0.011362
0.011396	0.011329	0.011396	0.011329	0.011161	0.011396	0.011631
0.011732	0.011664	0.011732	0.011498	0.011732	0.011228	0.011329
0.011364	0.011666	0.011426	0.011266	0.011739	0.011231	0.011336
0.011671	0.011638	0.011496	0.011664	0.011367	0.011597	0.011564
0.011496	0.011664	0.011415	0.011307	0.011395	0.011396	0.011398
0.011564	0.011263	0.011601	0.011669	0.011396	0.011597	0.011636
0.011297	0.011671	0.011466	0.011533	0.011733	0.011262	0.011464
0.011429	0.011362	0.011262	0.011262	0.011329	0.011329	0.011396
0.011329	0.011396	0.011295	0.011396	0.011329	0.011329	0.011396
0.011329	0.011765	0.011664	0.011698	0.011228	0.011329	0.011362
0.011362	0.011329	0.011396	0.011429	0.011295	0.011295	0.011295
0.011832	0.011463	0.011463	0.011262	0.011362	0.011530	0.011295
0.011295	0.011362	0.011329	0.011496	0.011295	0.011429	0.011564
0.011496	0.011329	0.011329	0.011262	0.011262	0.011429	0.011362
0.011262	0.011362	0.011228	0.011262	0.011396	0.011530	0.011295
0.011262	0.011396	0.011329	0.011228	0.011564	0.011228	0.011396
0.011437	0.011295	0.011597	0.011430	0.011463	0.011329	0.011429
0.011295	0.011631	0.011228	0.011496	0.011295	0.011295	0.011228
0.011463	0.011665	0.011362	0.011429	0.011262	0.011664	0.011295
0.011295	0.011295	0.011329	0.011429	0.011329	0.011429	0.011496
0.011496	0.011463	0.011362	0.011463	0.011429	0.011295	0.011262
0.011295	0.011295	0.011362	0.011295	0.011262	0.011429	0.011295
0.011329	0.011329	0.011463	0.011496	0.011396	0.011329	0.011329
0.011396	0.011329	0.011429	0.011295	0.011429	0.011530	0.011832
0.011329	0.011731	0.011336	0.017860	0.011191	0.011305	0.011688
0.011648	0.011693	0.011322	0.011292	0.011664	0.011716	0.011303
0.011487	0.011426	0.011177	0.011228	0.011333	0.011307	0.011232
0.011482	0.011507	0.011505	0.011457	0.011463	0.011362	0.011432
0.011369	0.011463	0.011463	0.011429	0.011396	0.011396	0.011363
0.011396	0.011262	0.011362	0.011396	0.011362	0.011295	0.011195
0.011195	0.011396	0.011295	0.011396	0.011429	0.011463	0.011228
0.011396	0.011362	0.011698	0.011698	0.011429	0.011464	0.011497
0.011262	0.011262	0.011295	0.011396	0.011262	0.011497	0.011497
0.011530	0.011496	0.011463	0.011564	0.011732	0.011565	0.011396

0.011396	0.011463	0.011497	0.011329	0.011396	0.011362	0.011396
0.011463	0.011463	0.011497	0.011463	0.011363	0.011564	0.011463
0.011399	0.011295	0.011396	0.011497	0.011396	0.011530	0.011564
0.011664	0.011367	0.011597	0.011564	0.011496	0.011664	0.011295
0.011464	0.011363	0.011195	0.011195	0.011396	0.011295	0.011396
0.011429	0.011463	0.011228	0.011396	0.011362	0.011698	0.011698
0.011429	0.011464	0.011396	0.011329	0.012069	0.011524	0.011341
0.011517	0.011476	0.011587	0.011441	0.011585	0.011439	0.011474
0.012021	0.011233	0.011368	0.011371	0.011645	0.011570	0.011672
0.012012	0.011512	0.011635	0.011698	0.011564	0.011564	0.011601
0.011262	0.011429	0.011564	0.011530	0.011533	0.011530	0.011498
0.011362	0.011295	0.011329	0.011362	0.011262	0.011329	0.011228
0.011362	0.011396	0.011295	0.011262	0.011362	0.011295	0.011329
0.011329	0.011262	0.011329	0.011362	0.011262	0.011463	0.011496
0.011396	0.011463	0.011396	0.011396	0.011329	0.011731	0.011362
0.011408	0.011329	0.011564	0.011569	0.011429	0.011429	0.011429
0.011429	0.011362	0.011329	0.011362	0.011463	0.011463	0.011396
0.011396	0.011530	0.011698	0.011429	0.011631	0.011631	0.011496
0.011329	0.011396	0.011429	0.011832	0.011396	0.011429	0.011396
0.011429	0.011362	0.011362	0.011664	0.011597	0.011631	0.011362
0.011463	0.011530	0.011396	0.011463	0.011429	0.011295	0.011631
0.011396	0.011362	0.011362	0.011530	0.011362	0.011463	0.011698
0.011396	0.011705	0.011765	0.011507	0.011229	0.011468	0.011267
0.011225	0.011722	0.011306	0.011462	0.011255	0.011366	0.011379
0.011519	0.011192	0.011339	0.011192	0.011222	0.011395	0.011385
0.011399	0.011296	0.011448	0.011377	0.011397	0.011530	0.011597
0.011597	0.011464	0.011567	0.011569	0.011704	0.011731	0.011396
0.011631	0.011497	0.011362	0.011463	0.011262	0.011496	0.011295
0.011362	0.011362	0.011329	0.011295	0.011329	0.011295	0.011396
0.011375	0.011375	0.011368	0.011403	0.011539	0.011470	0.011504
0.011503	0.011430	0.011171	0.011168	0.011365	0.011534	0.011535
0.011329	0.011399	0.011632	0.011601	0.011531	0.011565	0.011362
0.011429	0.011429	0.011429	0.011396	0.011933	0.011564	0.011631
0.011665	0.011736	0.011367	0.011441	0.011329	0.011262	0.011362
0.011531	0.011429	0.011564	0.011564	0.011650	0.011564	0.011338
0.011229	0.011233	0.011332	0.011298	0.011402	0.011247	0.011570
0.011369	0.011499	0.011251	0.011285	0.011363	0.011437	0.011329
0.011295	0.011295	0.011362	0.011329	0.011530	0.011497	0.011429
0.011530	0.011597	0.011329	0.011329	0.011362	0.011362	0.011364
0.011432	0.011362	0.011362	0.011397	0.011530	0.011597	0.011597
0.011464	0.011567	0.011569	0.011704	0.011731	0.011396	0.011564
0.011362	0.011362	0.011331	0.011513	0.011442	0.011469	0.011340
0.011508	0.011698	0.011526	0.011400	0.011295	0.011497	0.011362
0.011463	0.011396	0.011295	0.011397	0.011497	0.011499	0.011396
0.011295	0.011229	0.011362	0.011295	0.011228	0.011332	0.011497
0.011396	0.011262	0.011362	0.011396	0.011362	0.011295	0.011497
0.011362	0.011396	0.011396	0.011329	0.011396	0.011631	0.011295
0.011329	0.011731	0.011329	0.011362	0.011329	0.011698	0.011232
0.011734	0.011230	0.011261	0.011543	0.011549	0.011527	0.011144
0.011218	0.011496	0.011597	0.011705	0.011565	0.011703	0.011834
0.011733	0.011770	0.011731	0.011983	0.011698	0.012505	0.011734
0.012473	0.012173	0.012307	0.012646	0.011698	0.011396	0.011698
0.012103	0.012072	0.011664	0.011564	0.011632	0.011632	0.011601
0.011496	0.011501	0.011564	0.011531	0.011901	0.011967	0.011968
0.011867	0.011968	0.012072	0.011838	0.012104	0.012110	0.012171
0.012171	0.012205	0.012084	0.012171	0.012173	0.011295	0.011699
0.011800	0.012303	0.011772	0.012034	0.012508	0.011570	0.011464
0.011429	0.011429	0.011900	0.011924	0.012045	0.011765	0.011431
S119	0.011597	0.011597	0.012001	0.011765	0.011765	0.011832
0.011603	0.011535	0.011832	0.011262	0.011329	0.011296	0.011262

0.011295	0.011463	0.011262	0.011295	0.011362	0.011329	0.011295
0.011262	0.011295	0.011396	0.011262	0.011295	0.011295	0.011569
0.011530	0.011295	0.011194	0.011429	0.011362	0.011329	0.011362
0.011329	0.011429	0.011329	0.011329	0.011362	0.011329	0.011496
0.011262	0.011329	0.011362	0.011295	0.011396	0.011429	0.011362
0.011329	0.011496	0.011396	0.011664	0.011329	0.011597	0.011329
0.011295	0.011396	0.011463	0.011597	0.011698	0.011396	0.011496
0.011362	0.011429	0.011329	0.011563	0.011799	0.011329	0.011396
0.011295	0.011396	0.011362	0.011429	0.011362	0.011362	0.011362
0.011262	0.011295	0.011262	0.011362	0.011329	0.011363	0.011429
0.011362	0.011329	0.011396	0.011295	0.011429	0.011262	0.011329
0.011496	0.011429	0.011664	0.011496	0.011329	0.011564	0.011329
0.011431	0.011658	0.011475	0.011138	0.011247	0.011355	0.011375
0.012085	0.011416	0.011184	0.011563	0.011564	0.011362	0.011463
0.011396	0.011295	0.011362	0.011329	0.011429	0.011362	0.011094
0.000000	0.011429	0.011564	0.011496	0.011396	0.011362	0.011295
0.011262	0.011262	0.011329	0.011295	0.011497	0.011463	0.011396
0.011497	0.011564	0.011295	0.011295	0.011329	0.011329	0.011330
0.011398	0.011329	0.011329	0.011204	0.011277	0.011235	0.011329
0.011331	0.011266	0.011318	0.011302	0.011230	0.011266	0.011297
0.011564	0.011336	0.011370	0.011262	0.011268	0.011397	0.011363
0.011430	0.011363	0.011363	0.011262	0.011329	0.011765	0.011564
0.011597	0.011463	0.011329	0.011429	0.011228	0.011463	0.011262
0.011329	0.011329	0.011295	0.011262	0.011295	0.011262	0.011362
0.011329	0.011429	0.011362	0.011262	0.011363	0.011463	0.011466
0.011463	0.011362	0.011262	0.011195	0.011329	0.011262	0.011195
0.011298	0.011463	0.011262	0.011463	0.011228	0.011329	0.011295
0.011396	0.011228	0.011329	0.011295	0.011329	0.011195	0.011329
0.011362	0.011295	0.011362	0.011295	0.011195	0.011362	0.011597
0.011698	0.011631	0.011698	0.011465	0.011698	0.011262	0.011295
0.011330	0.011626	0.011426	0.011232	0.011705	0.011197	0.011336
0.011637	0.011604	0.011463	0.011631	0.011402	0.011564	0.011530
0.011463	0.011631	0.011415	0.011307	0.011395	0.011362	0.011365
0.011530	0.011296	0.011567	0.011635	0.011429	0.011564	0.011670
0.011331	0.011637	0.011500	0.011500	0.011699	0.011228	0.011431
0.011396	0.011329	0.011228	0.011228	0.011295	0.011295	0.011362
0.011295	0.011362	0.011262	0.011362	0.011295	0.011295	0.011362
0.011295	0.011731	0.011631	0.011664	0.011194	0.011295	0.011329
0.011329	0.011295	0.011362	0.011396	0.011262	0.011262	0.011262
0.011799	0.011429	0.011429	0.011228	0.011329	0.011496	0.011262
0.011262	0.011329	0.011295	0.011463	0.011262	0.011396	0.011530
0.011463	0.011295	0.011295	0.011228	0.011228	0.011396	0.011329
0.011228	0.011329	0.011195	0.011228	0.011362	0.011497	0.011262
0.011228	0.011362	0.011295	0.011195	0.011530	0.011195	0.011362
0.011437	0.011262	0.011564	0.011396	0.011429	0.011295	0.011396
0.011262	0.011597	0.011194	0.011463	0.011262	0.011262	0.011194
0.011429	0.011631	0.011329	0.011396	0.011228	0.011631	0.011262
0.011262	0.011262	0.011295	0.011396	0.011295	0.011396	0.011463
0.011463	0.011429	0.011329	0.011429	0.011396	0.011262	0.011228
0.011262	0.011262	0.011329	0.011262	0.011228	0.011396	0.011262
0.011295	0.011295	0.011429	0.011463	0.011362	0.011295	0.011295
0.011362	0.011295	0.011396	0.011262	0.011396	0.011496	0.011799
0.011295	0.011698	0.011302	0.017823	0.011191	0.011305	0.011653
0.011614	0.011658	0.011322	0.011292	0.011609	0.011681	0.011303
0.011440	0.011426	0.011138	0.011228	0.011294	0.011258	0.011189
0.011436	0.011470	0.011468	0.011391	0.011429	0.011396	0.011466
0.011335	0.011429	0.011429	0.011396	0.011362	0.011362	0.011329
0.011362	0.011228	0.011329	0.011362	0.011329	0.011262	0.011228
0.011228	0.011362	0.011262	0.011362	0.011396	0.011429	0.011262
0.011362	0.011329	0.011665	0.011665	0.011396	0.011430	0.011463

0.011228	0.011228	0.011262	0.011362	0.011228	0.011463	0.011463
0.011497	0.011463	0.011429	0.011530	0.011698	0.011531	0.011362
0.011362	0.011429	0.011463	0.011295	0.011362	0.011329	0.011362
0.011430	0.011430	0.011463	0.011430	0.011330	0.011530	0.011429
0.011365	0.011262	0.011362	0.011463	0.011362	0.011497	0.011530
0.011631	0.011402	0.011564	0.011530	0.011463	0.011631	0.011262
0.011431	0.011329	0.011228	0.011228	0.011362	0.011262	0.011362
0.011396	0.011429	0.011262	0.011362	0.011329	0.011665	0.011665
0.011396	0.011430	0.011429	0.011362	0.012035	0.011489	0.011341
0.011482	0.011441	0.011552	0.011441	0.011550	0.011405	0.011439
0.011987	0.011268	0.011403	0.011337	0.011611	0.011536	0.011638
0.012012	0.011512	0.011601	0.011664	0.011530	0.011530	0.011567
0.011228	0.011396	0.011530	0.011496	0.011499	0.011496	0.011464
0.011329	0.011262	0.011295	0.011329	0.011228	0.011295	0.011194
0.011329	0.011362	0.011262	0.011228	0.011329	0.011262	0.011295
0.011295	0.011228	0.011295	0.011329	0.011228	0.011429	0.011463
0.011362	0.011429	0.011362	0.011362	0.011295	0.011698	0.011329
0.011373	0.011295	0.011530	0.011535	0.011396	0.011396	0.011396
0.011396	0.011329	0.011295	0.011329	0.011429	0.011429	0.011362
0.011362	0.011496	0.011664	0.011396	0.011597	0.011597	0.011463
0.011295	0.011362	0.011396	0.011799	0.011362	0.011396	0.011362
0.011396	0.011329	0.011329	0.011631	0.011564	0.011597	0.011329
0.011429	0.011496	0.011362	0.011429	0.011396	0.011262	0.011597
0.011362	0.011329	0.011329	0.011496	0.011329	0.011429	0.011664
0.011362	0.011671	0.011731	0.011467	0.011173	0.011434	0.011215
0.011170	0.011683	0.011271	0.011417	0.011207	0.011315	0.011326
0.011468	0.011126	0.011283	0.011126	0.011168	0.011328	0.011344
0.011362	0.011257	0.011412	0.011342	0.011363	0.011497	0.011564
0.011564	0.011430	0.011533	0.011535	0.011670	0.011696	0.011362
0.011597	0.011463	0.011329	0.011429	0.011228	0.011463	0.011262
0.011329	0.011329	0.011295	0.011262	0.011295	0.011262	0.011362
0.011340	0.011340	0.011334	0.011368	0.011505	0.011436	0.011470
0.011469	0.011396	0.011134	0.011132	0.011331	0.011501	0.011501
0.011296	0.011365	0.011599	0.011567	0.011497	0.011531	0.011396
0.011396	0.011396	0.011396	0.011362	0.011900	0.011530	0.011597
0.011631	0.011701	0.011333	0.011407	0.011295	0.011295	0.011396
0.011497	0.011463	0.011530	0.011530	0.011610	0.011597	0.011303
0.011195	0.011199	0.011298	0.011264	0.011368	0.011209	0.011536
0.011334	0.011499	0.011251	0.011285	0.011329	0.011402	0.011295
0.011262	0.011262	0.011329	0.011295	0.011497	0.011463	0.011396
0.011497	0.011564	0.011295	0.011295	0.011329	0.011329	0.011330
0.011398	0.011329	0.011329	0.011363	0.011497	0.011564	0.011564
0.011430	0.011533	0.011535	0.011670	0.011696	0.011362	0.011530
0.011396	0.011396	0.011297	0.011478	0.011408	0.011435	0.011305
0.011473	0.011698	0.011484	0.011366	0.011262	0.011463	0.011329
0.011429	0.011362	0.011262	0.011363	0.011463	0.011466	0.011362
0.011262	0.011195	0.011329	0.011262	0.011195	0.011298	0.011463
0.011362	0.011228	0.011329	0.011362	0.011329	0.011262	0.011463
0.011329	0.011362	0.011362	0.011295	0.011362	0.011597	0.011262
0.011295	0.011698	0.011295	0.011329	0.011295	0.011664	0.011189
0.011678	0.011188	0.011211	0.011503	0.011508	0.011491	0.011102
0.011165	0.011463	0.011564	0.011671	0.011531	0.011669	0.011800
0.011699	0.011737	0.011698	0.011948	0.011664	0.012471	0.011700
0.012439	0.012139	0.012273	0.012612	0.011664	0.011362	0.011665
0.012069	0.012038	0.011631	0.011530	0.011598	0.011598	0.011567
0.011463	0.011467	0.011530	0.011497	0.011867	0.011933	0.011935
0.011834	0.011934	0.012038	0.011804	0.012070	0.012076	0.012137
0.012138	0.012171	0.012049	0.012138	0.012139	0.011329	0.011665
0.011766	0.012270	0.011738	0.012001	0.012474	0.011536	0.011430
0.011396	0.011396	0.011867	0.011924	0.012010	0.011731	0.011397

S120	0.011765	0.011765	0.012034	0.011799	0.011799	0.011866
0.011671	0.011603	0.011866	0.011362	0.011429	0.011397	0.011362
0.011329	0.011564	0.011295	0.011396	0.011463	0.011429	0.011396
0.011362	0.011396	0.011429	0.011363	0.011396	0.011396	0.011637
0.011564	0.011396	0.011295	0.011463	0.011463	0.011429	0.011463
0.011429	0.011530	0.011429	0.011429	0.011463	0.011429	0.011597
0.011295	0.011429	0.011463	0.011396	0.011429	0.011530	0.011463
0.011429	0.011597	0.011497	0.011698	0.011429	0.011698	0.011429
0.011396	0.011429	0.011564	0.011698	0.011731	0.011429	0.011530
0.011463	0.011530	0.011429	0.011664	0.011832	0.011429	0.011496
0.011396	0.011496	0.011463	0.011530	0.011262	0.011463	0.011463
0.011362	0.011396	0.011362	0.011396	0.011429	0.011464	0.011530
0.011463	0.011429	0.011496	0.011262	0.011463	0.011362	0.011429
0.011597	0.011530	0.011765	0.011597	0.011429	0.011664	0.011429
0.011542	0.011695	0.011588	0.011295	0.011284	0.011428	0.011415
0.012085	0.011467	0.011307	0.011664	0.011664	0.011329	0.011496
0.011496	0.011463	0.011530	0.011429	0.011530	0.011463	0.011463
0.011429	0.000000	0.011731	0.011597	0.011496	0.011463	0.011396
0.011362	0.011295	0.011429	0.011396	0.011530	0.011497	0.011429
0.011530	0.011597	0.011396	0.011396	0.011430	0.011429	0.011432
0.011500	0.011429	0.011429	0.011276	0.011386	0.011341	0.011430
0.011433	0.011369	0.011392	0.011405	0.011332	0.011368	0.011398
0.011600	0.011370	0.011473	0.011363	0.011371	0.011497	0.011396
0.011464	0.011465	0.011464	0.011564	0.011496	0.011866	0.011597
0.011631	0.011497	0.011429	0.011463	0.011329	0.011496	0.011362
0.011429	0.011429	0.011396	0.011362	0.011396	0.011362	0.011463
0.011429	0.011530	0.011463	0.011362	0.011464	0.011564	0.011533
0.011564	0.011463	0.011362	0.011263	0.011429	0.011362	0.011295
0.011366	0.011564	0.011362	0.011564	0.011329	0.011429	0.011396
0.011497	0.011396	0.011429	0.011396	0.011429	0.011295	0.011429
0.011463	0.011396	0.011463	0.011396	0.011429	0.011463	0.011698
0.011732	0.011664	0.011732	0.011532	0.011732	0.011429	0.011396
0.011297	0.011626	0.011472	0.011300	0.011739	0.011265	0.011375
0.011705	0.011638	0.011597	0.011799	0.011504	0.011664	0.011631
0.011564	0.011664	0.011593	0.011472	0.011521	0.011463	0.011534
0.011631	0.011532	0.011669	0.011737	0.011664	0.011732	0.011839
0.011568	0.011739	0.011669	0.011601	0.011867	0.011329	0.011532
0.011496	0.011429	0.011329	0.011329	0.011396	0.011396	0.011463
0.011396	0.011463	0.011362	0.011396	0.011396	0.011396	0.011463
0.011396	0.011765	0.011664	0.011698	0.011295	0.011396	0.011429
0.011429	0.011396	0.011463	0.011429	0.011362	0.011362	0.011362
0.011832	0.011463	0.011530	0.011329	0.011429	0.011597	0.011362
0.011362	0.011429	0.011396	0.011564	0.011362	0.011496	0.011631
0.011564	0.011396	0.011396	0.011329	0.011329	0.011496	0.011429
0.011329	0.011429	0.011295	0.011329	0.011463	0.011530	0.011362
0.011329	0.011463	0.011396	0.011295	0.011564	0.011295	0.011463
0.011539	0.011362	0.011597	0.011497	0.011463	0.011396	0.011496
0.011362	0.011698	0.011295	0.011496	0.011295	0.011295	0.011295
0.011530	0.011665	0.011429	0.011496	0.011329	0.011664	0.011295
0.011362	0.011362	0.011396	0.011496	0.011396	0.011496	0.011564
0.011564	0.011530	0.011429	0.011530	0.011496	0.011362	0.011329
0.011362	0.011362	0.011429	0.011295	0.011329	0.011496	0.011362
0.011329	0.011396	0.011530	0.011496	0.011463	0.011396	0.011396
0.011329	0.011396	0.011496	0.011362	0.011496	0.011597	0.011832
0.011396	0.011731	0.011371	0.017898	0.011387	0.011366	0.011653
0.011614	0.011658	0.011427	0.011408	0.011719	0.011681	0.011425
0.011487	0.011487	0.011255	0.011353	0.011294	0.011307	0.011318
0.011482	0.011470	0.011468	0.011325	0.011530	0.011564	0.011567
0.011438	0.011530	0.011530	0.011429	0.011463	0.011463	0.011430
0.011463	0.011396	0.011429	0.011463	0.011429	0.011362	0.011396

0.011396	0.011463	0.011362	0.011463	0.011497	0.011530	0.011429
0.011396	0.011362	0.011698	0.011698	0.011497	0.011531	0.011564
0.011329	0.011329	0.011362	0.011463	0.011329	0.011564	0.011564
0.011597	0.011597	0.011530	0.011631	0.011732	0.011633	0.011463
0.011463	0.011530	0.011564	0.011396	0.011463	0.011429	0.011463
0.011531	0.011530	0.011564	0.011530	0.011431	0.011631	0.011597
0.011466	0.011362	0.011463	0.011564	0.011463	0.011530	0.011631
0.011799	0.011504	0.011664	0.011631	0.011564	0.011664	0.011363
0.011532	0.011430	0.011396	0.011396	0.011463	0.011362	0.011463
0.011497	0.011530	0.011429	0.011396	0.011362	0.011698	0.011698
0.011497	0.011531	0.011597	0.011530	0.012103	0.011596	0.011517
0.011587	0.011545	0.011657	0.011545	0.011655	0.011577	0.011543
0.012056	0.011441	0.011574	0.011510	0.011783	0.011638	0.011741
0.012080	0.011550	0.011703	0.011765	0.011631	0.011564	0.011668
0.011329	0.011496	0.011631	0.011597	0.011601	0.011530	0.011464
0.011429	0.011362	0.011396	0.011429	0.011329	0.011396	0.011295
0.011362	0.011396	0.011362	0.011329	0.011429	0.011362	0.011396
0.011396	0.011329	0.011396	0.011429	0.011329	0.011530	0.011564
0.011463	0.011463	0.011463	0.011463	0.011396	0.011731	0.011429
0.011408	0.011396	0.011564	0.011569	0.011496	0.011496	0.011496
0.011496	0.011429	0.011396	0.011429	0.011530	0.011530	0.011463
0.011463	0.011597	0.011698	0.011496	0.011698	0.011698	0.011563
0.011396	0.011463	0.011496	0.011832	0.011463	0.011496	0.011463
0.011496	0.011362	0.011362	0.011664	0.011664	0.011631	0.011429
0.011530	0.011597	0.011463	0.011530	0.011496	0.011362	0.011631
0.011463	0.011429	0.011362	0.011597	0.011429	0.011530	0.011765
0.011463	0.011739	0.011765	0.011467	0.011229	0.011468	0.011267
0.011170	0.011722	0.011341	0.011462	0.011255	0.011366	0.011379
0.011468	0.011323	0.011450	0.011324	0.011330	0.011529	0.011466
0.011475	0.011375	0.011412	0.011412	0.011465	0.011530	0.011597
0.011597	0.011464	0.011567	0.011569	0.011704	0.011732	0.011463
0.011631	0.011497	0.011429	0.011463	0.011329	0.011496	0.011362
0.011429	0.011429	0.011396	0.011362	0.011396	0.011362	0.011463
0.011444	0.011445	0.011437	0.011266	0.011607	0.011573	0.011572
0.011571	0.011296	0.011282	0.011276	0.011433	0.011602	0.011603
0.011396	0.011467	0.011700	0.011668	0.011598	0.011632	0.011564
0.011496	0.011564	0.011563	0.011463	0.011967	0.011631	0.011698
0.011732	0.011701	0.011435	0.011510	0.011396	0.011597	0.011564
0.011397	0.011631	0.011631	0.011631	0.011807	0.011765	0.011407
0.011297	0.011303	0.011400	0.011366	0.011402	0.011321	0.011570
0.011437	0.011543	0.011394	0.011435	0.011430	0.011505	0.011396
0.011362	0.011295	0.011429	0.011396	0.011530	0.011497	0.011429
0.011530	0.011597	0.011396	0.011396	0.011430	0.011429	0.011432
0.011500	0.011429	0.011429	0.011465	0.011530	0.011597	0.011597
0.011464	0.011567	0.011569	0.011704	0.011732	0.011463	0.011631
0.011564	0.011564	0.011398	0.011582	0.011512	0.011333	0.011375
0.011508	0.011698	0.011441	0.011468	0.011362	0.011564	0.011429
0.011530	0.011463	0.011362	0.011464	0.011564	0.011533	0.011463
0.011362	0.011263	0.011429	0.011362	0.011295	0.011366	0.011564
0.011463	0.011396	0.011429	0.011463	0.011429	0.011362	0.011497
0.011429	0.011463	0.011463	0.011396	0.011463	0.011698	0.011295
0.011396	0.011731	0.011396	0.011429	0.011396	0.011698	0.011274
0.011678	0.011273	0.011261	0.011503	0.011508	0.011491	0.011227
0.011218	0.011496	0.011597	0.011705	0.011565	0.011669	0.011834
0.011733	0.011770	0.011731	0.011949	0.011698	0.012572	0.011734
0.012473	0.012207	0.012375	0.012578	0.011698	0.011396	0.011698
0.012137	0.012038	0.011664	0.011564	0.011632	0.011632	0.011601
0.011496	0.011467	0.011564	0.011531	0.011901	0.011967	0.011968
0.011867	0.011968	0.012038	0.011804	0.012137	0.012144	0.012205
0.012205	0.012239	0.012152	0.012205	0.012207	0.011496	0.011732

0.011800	0.012303	0.011806	0.012034	0.012508	0.011570	0.011464
0.011429	0.011429	0.011900	0.011965	0.012045	0.011765	0.011431
S121	0.011900	0.011900	0.012303	0.012068	0.012068	0.012135
0.011977	0.011909	0.012135	0.011564	0.011631	0.011599	0.011564
0.011597	0.011765	0.011564	0.011597	0.011664	0.011631	0.011597
0.011564	0.011597	0.011698	0.011564	0.011598	0.011597	0.011943
0.011832	0.011597	0.011496	0.011731	0.011664	0.011631	0.011664
0.011631	0.011732	0.011631	0.011631	0.011664	0.011631	0.011799
0.011564	0.011631	0.011664	0.011597	0.011698	0.011732	0.011664
0.011631	0.011799	0.011699	0.011967	0.011631	0.011900	0.011631
0.011597	0.011698	0.011765	0.011900	0.012000	0.011698	0.011799
0.011664	0.011732	0.011631	0.011866	0.012102	0.011631	0.011698
0.011597	0.011698	0.011664	0.011664	0.011664	0.011664	0.011664
0.011564	0.011597	0.011564	0.011664	0.011631	0.011666	0.011731
0.011664	0.011631	0.011698	0.011597	0.011731	0.011564	0.011631
0.011799	0.011731	0.011967	0.011799	0.011631	0.011866	0.011631
0.011802	0.012032	0.011815	0.011490	0.011471	0.011649	0.011769
0.012378	0.011721	0.011513	0.011866	0.011866	0.011664	0.011765
0.011698	0.011463	0.011664	0.011631	0.011732	0.011664	0.011597
0.011564	0.011731	0.000000	0.011799	0.011698	0.011665	0.011598
0.011564	0.011564	0.011631	0.011597	0.011799	0.011766	0.011698
0.011799	0.011866	0.011597	0.011597	0.011631	0.011631	0.011634
0.011703	0.011631	0.011631	0.011528	0.011603	0.011551	0.011632
0.011636	0.011576	0.011577	0.011612	0.011536	0.011574	0.011601
0.011888	0.011646	0.011680	0.011565	0.011579	0.011700	0.011666
0.011733	0.011667	0.011666	0.011698	0.011631	0.012068	0.011867
0.011899	0.011766	0.011631	0.011732	0.011530	0.011765	0.011564
0.011631	0.011631	0.011597	0.011564	0.011597	0.011564	0.011665
0.011631	0.011732	0.011665	0.011564	0.011667	0.011766	0.011736
0.011766	0.011665	0.011564	0.011499	0.011631	0.011564	0.011497
0.011604	0.011766	0.011564	0.011766	0.011530	0.011631	0.011597
0.011698	0.011597	0.011631	0.011598	0.011631	0.011497	0.011631
0.011665	0.011597	0.011665	0.011597	0.011564	0.011665	0.011900
0.012001	0.011933	0.012001	0.011768	0.012001	0.011564	0.011597
0.011635	0.011869	0.011609	0.011540	0.011977	0.011504	0.011611
0.012011	0.011911	0.011765	0.011127	0.011675	0.011866	0.011833
0.011765	0.011933	0.011735	0.011637	0.011563	0.011665	0.011669
0.011833	0.011667	0.011872	0.011940	0.011799	0.011732	0.011975
0.011703	0.011943	0.011805	0.011804	0.011868	0.011530	0.011734
0.011698	0.011631	0.011530	0.011530	0.011597	0.011597	0.011664
0.011597	0.011664	0.011564	0.011664	0.011597	0.011597	0.011664
0.011597	0.012034	0.011933	0.011967	0.011496	0.011597	0.011631
0.011631	0.011597	0.011664	0.011698	0.011564	0.011564	0.011564
0.012101	0.011732	0.011732	0.011530	0.011631	0.011799	0.011564
0.011564	0.011631	0.011597	0.011765	0.011564	0.011698	0.011832
0.011765	0.011597	0.011597	0.011530	0.011530	0.011698	0.011631
0.011530	0.011631	0.011497	0.011530	0.011665	0.011799	0.011564
0.011531	0.011664	0.011598	0.011497	0.011832	0.011497	0.011664
0.011745	0.011564	0.011866	0.011698	0.011732	0.011597	0.011698
0.011564	0.011900	0.011496	0.011765	0.011564	0.011564	0.011496
0.011732	0.011934	0.011631	0.011698	0.011530	0.011933	0.011564
0.011564	0.011564	0.011597	0.011698	0.011597	0.011698	0.011765
0.011765	0.011732	0.011631	0.011732	0.011698	0.011564	0.011530
0.011564	0.011564	0.011631	0.011564	0.011530	0.011698	0.011564
0.011597	0.011597	0.011732	0.011765	0.011664	0.011597	0.011597
0.011664	0.011597	0.011698	0.011564	0.011698	0.011799	0.012101
0.011597	0.012001	0.011578	0.018088	0.011388	0.011549	0.011933
0.011891	0.011940	0.011479	0.011466	0.011885	0.011958	0.011546
0.011677	0.011732	0.011528	0.011479	0.011490	0.011455	0.011447
0.011717	0.011768	0.011653	0.011656	0.011732	0.011698	0.011737

0.011644	0.011732	0.011732	0.011698	0.011665	0.011665	0.011631
0.011665	0.011597	0.011631	0.011665	0.011631	0.011564	0.011530
0.011530	0.011597	0.011564	0.011665	0.011698	0.011732	0.011564
0.011664	0.011631	0.011967	0.011967	0.011698	0.011733	0.011698
0.011530	0.011530	0.011564	0.011665	0.011530	0.011766	0.011766
0.011799	0.011765	0.011732	0.011833	0.012001	0.011835	0.011665
0.011665	0.011732	0.011766	0.011597	0.011665	0.011631	0.011665
0.011732	0.011732	0.011765	0.011732	0.011633	0.011833	0.011799
0.011670	0.011564	0.011665	0.011766	0.011598	0.011799	0.011832
0.011127	0.011675	0.011866	0.011833	0.011765	0.011933	0.011564
0.011734	0.011632	0.011530	0.011530	0.011597	0.011564	0.011665
0.011698	0.011732	0.011564	0.011664	0.011631	0.011967	0.011967
0.011698	0.011733	0.011732	0.011664	0.012339	0.011775	0.011658
0.011799	0.011754	0.011869	0.011753	0.011865	0.011439	0.011750
0.012297	0.011579	0.011711	0.011510	0.011783	0.011843	0.011945
0.012318	0.011814	0.011906	0.011967	0.011832	0.011832	0.011871
0.011530	0.011698	0.011832	0.011799	0.011804	0.011799	0.011768
0.011631	0.011564	0.011597	0.011631	0.011530	0.011597	0.011496
0.011631	0.011664	0.011564	0.011530	0.011631	0.011564	0.011597
0.011597	0.011530	0.011597	0.011631	0.011530	0.011732	0.011765
0.011664	0.011732	0.011664	0.011664	0.011597	0.012000	0.011631
0.011686	0.011597	0.011833	0.011841	0.011698	0.011698	0.011698
0.011698	0.011631	0.011597	0.011631	0.011732	0.011732	0.011664
0.011664	0.011799	0.011967	0.011698	0.011900	0.011900	0.011765
0.011597	0.011664	0.011698	0.012102	0.011665	0.011698	0.011664
0.011698	0.011631	0.011631	0.011934	0.011866	0.011900	0.011631
0.011732	0.011799	0.011664	0.011732	0.011698	0.011564	0.011900
0.011664	0.011631	0.011631	0.011799	0.011631	0.011732	0.011967
0.011664	0.012045	0.012034	0.011833	0.011399	0.011740	0.011473
0.011335	0.012073	0.011552	0.011775	0.011548	0.011518	0.011592
0.011723	0.011192	0.011561	0.011391	0.011492	0.011664	0.011709
0.011625	0.011650	0.011694	0.011624	0.011667	0.011799	0.011866
0.011866	0.011733	0.011838	0.011840	0.011976	0.011979	0.011664
0.011899	0.011766	0.011631	0.011732	0.011530	0.011765	0.011564
0.011631	0.011631	0.011597	0.011564	0.011597	0.011564	0.011665
0.011654	0.011655	0.011643	0.011677	0.011813	0.011778	0.011777
0.011776	0.011699	0.011540	0.011528	0.011636	0.011806	0.011807
0.011598	0.011670	0.011902	0.011872	0.011800	0.011834	0.011698
0.011698	0.011564	0.011564	0.011664	0.012203	0.011832	0.011900
0.011934	0.011986	0.011640	0.011719	0.011597	0.011732	0.011698
0.011800	0.011765	0.011832	0.011832	0.011768	0.011900	0.011615
0.011500	0.011510	0.011605	0.011570	0.011675	0.011433	0.011842
0.011643	0.011718	0.011489	0.011660	0.011632	0.011710	0.011598
0.011564	0.011564	0.011631	0.011597	0.011799	0.011766	0.011698
0.011799	0.011866	0.011597	0.011597	0.011631	0.011631	0.011634
0.011703	0.011631	0.011631	0.011667	0.011799	0.011866	0.011866
0.011733	0.011838	0.011840	0.011976	0.011979	0.011664	0.011832
0.011698	0.011698	0.011601	0.011722	0.011721	0.011707	0.011621
0.011784	0.011926	0.011738	0.011672	0.011564	0.011766	0.011631
0.011732	0.011665	0.011564	0.011667	0.011766	0.011736	0.011665
0.011564	0.011499	0.011631	0.011564	0.011497	0.011604	0.011766
0.011665	0.011597	0.011631	0.011665	0.011631	0.011564	0.011766
0.011631	0.011664	0.011664	0.011597	0.011664	0.011900	0.011564
0.011597	0.012000	0.011597	0.011631	0.011597	0.011967	0.011489
0.011904	0.011570	0.011512	0.011825	0.011875	0.011778	0.011437
0.011428	0.011765	0.011866	0.011977	0.011835	0.011940	0.012103
0.012003	0.012041	0.012000	0.012223	0.011967	0.012775	0.012004
0.012743	0.012444	0.012578	0.012849	0.011967	0.011665	0.011968
0.012373	0.012343	0.011933	0.011833	0.011901	0.011901	0.011872
0.011765	0.011739	0.011832	0.011800	0.012170	0.012236	0.012238

0.012137	0.012238	0.012342	0.012075	0.012374	0.012381	0.012441
0.012442	0.012475	0.012392	0.012442	0.012443	0.011631	0.011968
0.012070	0.012573	0.012044	0.012304	0.012778	0.011842	0.011733
0.011698	0.011698	0.012170	0.012254	0.012327	0.012034	0.011701
S122	0.011832	0.011832	0.012169	0.011933	0.011933	0.012000
0.011841	0.011773	0.011933	0.011429	0.011496	0.011464	0.011429
0.011463	0.011564	0.011362	0.011463	0.011530	0.011496	0.011463
0.011429	0.011463	0.011564	0.011430	0.011463	0.011463	0.011807
0.011698	0.011463	0.011362	0.011597	0.011530	0.011497	0.011530
0.011496	0.011597	0.011496	0.011496	0.011530	0.011496	0.011664
0.011429	0.011496	0.011530	0.011463	0.011563	0.011597	0.011530
0.011496	0.011664	0.011564	0.011832	0.011496	0.011765	0.011496
0.011463	0.011564	0.011631	0.011765	0.011866	0.011564	0.011664
0.011530	0.011597	0.011496	0.011731	0.011967	0.011429	0.011564
0.011463	0.011564	0.011530	0.011597	0.011530	0.011530	0.011530
0.011429	0.011463	0.011429	0.011530	0.011496	0.011531	0.011597
0.011530	0.011496	0.011564	0.011463	0.011597	0.011429	0.011496
0.011664	0.011597	0.011832	0.011664	0.011496	0.011731	0.011429
0.011653	0.011845	0.011739	0.011412	0.011433	0.011539	0.011611
0.012268	0.011721	0.011431	0.011731	0.011731	0.011463	0.011631
0.011564	0.011530	0.011597	0.011496	0.011597	0.011530	0.011530
0.011496	0.011597	0.011799	0.000000	0.011564	0.011530	0.011463
0.011429	0.011429	0.011497	0.011463	0.011665	0.011631	0.011564
0.011665	0.011732	0.011463	0.011463	0.011497	0.011497	0.011499
0.011567	0.011497	0.011497	0.011384	0.011458	0.011411	0.011497
0.011500	0.011438	0.011503	0.011474	0.011400	0.011437	0.011465
0.011708	0.011508	0.011542	0.011430	0.011440	0.011565	0.011531
0.011598	0.011532	0.011531	0.011631	0.011564	0.011933	0.011732
0.011765	0.011631	0.011496	0.011597	0.011396	0.011631	0.011430
0.011496	0.011496	0.011463	0.011429	0.011463	0.011429	0.011530
0.011497	0.011597	0.011530	0.011429	0.011464	0.011631	0.011635
0.011631	0.011530	0.011429	0.011364	0.011497	0.011430	0.011363
0.011468	0.011631	0.011430	0.011631	0.011396	0.011497	0.011463
0.011564	0.011463	0.011497	0.011463	0.011497	0.011362	0.011497
0.011530	0.011463	0.011530	0.011463	0.011497	0.011530	0.011765
0.011866	0.011799	0.011866	0.011633	0.011866	0.011497	0.011463
0.011432	0.011707	0.011517	0.011369	0.011807	0.011367	0.011414
0.011875	0.011774	0.011664	0.011866	0.011572	0.011731	0.011698
0.011631	0.011799	0.011664	0.011431	0.011521	0.011530	0.011601
0.011698	0.011600	0.011736	0.011804	0.011732	0.011799	0.011907
0.011635	0.011807	0.011737	0.011669	0.011935	0.011396	0.011599
0.011496	0.011496	0.011396	0.011396	0.011463	0.011396	0.011530
0.011463	0.011530	0.011429	0.011530	0.011463	0.011463	0.011530
0.011463	0.011899	0.011799	0.011832	0.011362	0.011463	0.011496
0.011496	0.011463	0.011530	0.011564	0.011429	0.011429	0.011429
0.011967	0.011597	0.011597	0.011396	0.011496	0.011664	0.011429
0.011429	0.011496	0.011463	0.011631	0.011429	0.011564	0.011698
0.011631	0.011463	0.011463	0.011396	0.011396	0.011564	0.011496
0.011396	0.011496	0.011362	0.011396	0.011530	0.011665	0.011396
0.011396	0.011530	0.011463	0.011362	0.011698	0.011362	0.011463
0.011608	0.011429	0.011732	0.011564	0.011597	0.011463	0.011564
0.011429	0.011765	0.011362	0.011631	0.011362	0.011362	0.011362
0.011597	0.011799	0.011496	0.011564	0.011396	0.011799	0.011362
0.011429	0.011429	0.011463	0.011564	0.011463	0.011564	0.011631
0.011631	0.011597	0.011496	0.011597	0.011564	0.011429	0.011396
0.011429	0.011429	0.011496	0.011429	0.011396	0.011564	0.011429
0.011396	0.011463	0.011597	0.011631	0.011530	0.011463	0.011463
0.011530	0.011463	0.011564	0.011429	0.011564	0.011597	0.011967
0.011463	0.011866	0.011474	0.017974	0.011387	0.011549	0.011828
0.011787	0.011834	0.011532	0.011582	0.011774	0.011854	0.011485

0.011772	0.011671	0.011372	0.011479	0.011490	0.011455	0.011447
0.011717	0.011656	0.011653	0.011523	0.011597	0.011631	0.011635
0.011507	0.011597	0.011597	0.011564	0.011530	0.011530	0.011497
0.011530	0.011463	0.011496	0.011530	0.011497	0.011429	0.011463
0.011463	0.011530	0.011429	0.011530	0.011564	0.011597	0.011497
0.011530	0.011497	0.011833	0.011833	0.011564	0.011531	0.011631
0.011396	0.011396	0.011429	0.011463	0.011396	0.011631	0.011631
0.011665	0.011664	0.011597	0.011698	0.011866	0.011700	0.011530
0.011530	0.011597	0.011631	0.011463	0.011530	0.011497	0.011530
0.011598	0.011598	0.011631	0.011598	0.011498	0.011698	0.011665
0.011534	0.011429	0.011463	0.011631	0.011530	0.011665	0.011698
0.011866	0.011572	0.011731	0.011698	0.011631	0.011799	0.011430
0.011599	0.011430	0.011463	0.011463	0.011530	0.011429	0.011530
0.011564	0.011597	0.011497	0.011530	0.011497	0.011833	0.011833
0.011564	0.011531	0.011664	0.011597	0.012237	0.011632	0.011588
0.011658	0.011580	0.011728	0.011614	0.011690	0.011646	0.011577
0.012193	0.011510	0.011642	0.011579	0.011852	0.011706	0.011809
0.012216	0.011663	0.011770	0.011832	0.011698	0.011698	0.011736
0.011396	0.011564	0.011698	0.011664	0.011668	0.011664	0.011633
0.011496	0.011429	0.011463	0.011496	0.011396	0.011463	0.011362
0.011496	0.011530	0.011429	0.011396	0.011496	0.011429	0.011463
0.011463	0.011396	0.011463	0.011496	0.011396	0.011597	0.011631
0.011530	0.011597	0.011530	0.011530	0.011463	0.011866	0.011496
0.011547	0.011463	0.011698	0.011705	0.011564	0.011564	0.011564
0.011564	0.011496	0.011463	0.011496	0.011530	0.011597	0.011530
0.011530	0.011664	0.011832	0.011564	0.011765	0.011765	0.011631
0.011463	0.011530	0.011564	0.011967	0.011530	0.011564	0.011530
0.011564	0.011496	0.011496	0.011799	0.011732	0.011765	0.011496
0.011597	0.011664	0.011530	0.011597	0.011563	0.011429	0.011765
0.011530	0.011496	0.011496	0.011664	0.011496	0.011597	0.011832
0.011530	0.011909	0.011899	0.011710	0.011399	0.011604	0.011628
0.011335	0.011917	0.011446	0.011596	0.011499	0.011518	0.011538
0.011621	0.011389	0.011505	0.011258	0.011437	0.011529	0.011587
0.011588	0.011493	0.011483	0.011518	0.011532	0.011665	0.011732
0.011732	0.011598	0.011702	0.011670	0.011772	0.011838	0.011530
0.011765	0.011631	0.011496	0.011597	0.011396	0.011631	0.011430
0.011496	0.011496	0.011463	0.011429	0.011463	0.011429	0.011530
0.011479	0.011515	0.011505	0.011540	0.011676	0.011641	0.011640
0.011639	0.011564	0.011393	0.011384	0.011433	0.011670	0.011671
0.011464	0.011534	0.011767	0.011736	0.011665	0.011632	0.011631
0.011564	0.011631	0.011631	0.011530	0.012101	0.011698	0.011765
0.011799	0.011879	0.011504	0.011580	0.011463	0.011664	0.011631
0.011666	0.011698	0.011698	0.011698	0.011768	0.011832	0.011476
0.011364	0.011372	0.011469	0.011434	0.011538	0.011396	0.011706
0.011506	0.011630	0.011298	0.011547	0.011497	0.011573	0.011463
0.011429	0.011429	0.011497	0.011463	0.011665	0.011631	0.011564
0.011665	0.011732	0.011463	0.011463	0.011497	0.011497	0.011499
0.011567	0.011497	0.011497	0.011532	0.011665	0.011732	0.011732
0.011598	0.011702	0.011670	0.011772	0.011838	0.011530	0.011698
0.011631	0.011631	0.011466	0.011652	0.011512	0.011605	0.011481
0.011646	0.011881	0.011568	0.011536	0.011430	0.011631	0.011497
0.011597	0.011530	0.011429	0.011464	0.011631	0.011635	0.011530
0.011429	0.011364	0.011497	0.011430	0.011363	0.011468	0.011631
0.011530	0.011463	0.011496	0.011530	0.011497	0.011429	0.011631
0.011496	0.011530	0.011530	0.011463	0.011530	0.011765	0.011429
0.011463	0.011866	0.011463	0.011496	0.011463	0.011832	0.011403
0.011847	0.011443	0.011511	0.011664	0.011752	0.011634	0.011353
0.011480	0.011631	0.011731	0.011841	0.011700	0.011838	0.011968
0.011868	0.011906	0.011866	0.012120	0.011832	0.012707	0.011869
0.012608	0.012376	0.012510	0.012781	0.011832	0.011530	0.011833

0.012272	0.012207	0.011799	0.011698	0.011766	0.011766	0.011737
0.011631	0.011637	0.011698	0.011665	0.012035	0.012101	0.012103
0.012002	0.012103	0.012207	0.011974	0.012272	0.012279	0.012339
0.012340	0.012374	0.012323	0.012340	0.012342	0.011564	0.011867
0.011935	0.012438	0.011942	0.012169	0.012643	0.011705	0.011598
0.011564	0.011564	0.012035	0.012089	0.012186	0.011900	0.011566
S123	0.011732	0.011732	0.012068	0.011832	0.011832	0.011900
0.011705	0.011637	0.011899	0.011329	0.011396	0.011363	0.011329
0.011362	0.011530	0.011329	0.011362	0.011429	0.011396	0.011362
0.011329	0.011362	0.011463	0.011329	0.011363	0.011362	0.011671
0.011597	0.011362	0.011262	0.011496	0.011429	0.011396	0.011429
0.011396	0.011497	0.011396	0.011396	0.011429	0.011396	0.011564
0.011329	0.011396	0.011429	0.011362	0.011463	0.011496	0.011429
0.011396	0.011564	0.011463	0.011732	0.011396	0.011664	0.011396
0.011362	0.011463	0.011530	0.011664	0.011765	0.011463	0.011564
0.011429	0.011497	0.011396	0.011631	0.011866	0.011396	0.011463
0.011362	0.011463	0.011429	0.011497	0.011429	0.011429	0.011429
0.011329	0.011362	0.011329	0.011429	0.011396	0.011430	0.011496
0.011295	0.011396	0.011463	0.011362	0.011496	0.011329	0.011396
0.011564	0.011496	0.011732	0.011564	0.011396	0.011631	0.011396
0.011542	0.011770	0.011588	0.011295	0.011321	0.011428	0.011493
0.012158	0.011569	0.011307	0.011631	0.011631	0.011429	0.011530
0.011463	0.011429	0.011496	0.011396	0.011497	0.011429	0.011429
0.011396	0.011496	0.011698	0.011564	0.000000	0.011430	0.011362
0.011329	0.011329	0.011396	0.011362	0.011564	0.011530	0.011463
0.011564	0.011631	0.011362	0.011362	0.011396	0.011396	0.011398
0.011466	0.011396	0.011396	0.011276	0.011349	0.011306	0.011396
0.011399	0.011335	0.011392	0.011371	0.011298	0.011334	0.011364
0.011636	0.011405	0.011439	0.011329	0.011337	0.011464	0.011430
0.011497	0.011431	0.011430	0.011530	0.011463	0.011832	0.011631
0.011664	0.011530	0.011396	0.011497	0.011295	0.011530	0.011329
0.011396	0.011396	0.011362	0.011329	0.011362	0.011329	0.011430
0.011396	0.011497	0.011430	0.011329	0.011431	0.011530	0.011533
0.011530	0.011430	0.011329	0.011263	0.011396	0.011329	0.011262
0.011332	0.011531	0.011329	0.011530	0.011295	0.011262	0.011362
0.011463	0.011362	0.011396	0.011362	0.011396	0.011262	0.011396
0.011430	0.011362	0.011430	0.011362	0.011396	0.011430	0.011665
0.011765	0.011698	0.011766	0.011532	0.011766	0.011396	0.011362
0.011364	0.011707	0.011518	0.011300	0.011773	0.011231	0.011414
0.011773	0.011672	0.011564	0.011765	0.011470	0.011631	0.011597
0.011530	0.011698	0.011522	0.011390	0.011479	0.011430	0.011466
0.011597	0.011499	0.011635	0.011703	0.011631	0.011698	0.011806
0.011534	0.011705	0.011636	0.011567	0.011834	0.011295	0.011498
0.011463	0.011396	0.011295	0.011295	0.011362	0.011362	0.011429
0.011362	0.011429	0.011329	0.011429	0.011362	0.011362	0.011429
0.011362	0.011799	0.011698	0.011732	0.011262	0.011362	0.011396
0.011262	0.011362	0.011429	0.011463	0.011329	0.011329	0.011329
0.011866	0.011496	0.011497	0.011295	0.011396	0.011564	0.011329
0.011329	0.011396	0.011362	0.011530	0.011329	0.011463	0.011597
0.011530	0.011362	0.011362	0.011295	0.011295	0.011463	0.011396
0.011295	0.011396	0.011262	0.011295	0.011429	0.011564	0.011329
0.011295	0.011429	0.011363	0.011262	0.011597	0.011262	0.011429
0.011505	0.011329	0.011631	0.011463	0.011497	0.011362	0.011463
0.011329	0.011664	0.011262	0.011530	0.011329	0.011329	0.011262
0.011496	0.011698	0.011396	0.011463	0.011295	0.011698	0.011329
0.011329	0.011329	0.011362	0.011463	0.011362	0.011463	0.011530
0.011530	0.011497	0.011396	0.011497	0.011463	0.011329	0.011295
0.011329	0.011329	0.011396	0.011329	0.011295	0.011463	0.011329
0.011362	0.011362	0.011497	0.011530	0.011429	0.011362	0.011362
0.011429	0.011362	0.011463	0.011329	0.011463	0.011564	0.011866

0.011362	0.011765	0.011371	0.017899	0.011388	0.011427	0.011723
0.011683	0.011729	0.011374	0.011292	0.011719	0.011751	0.011303
0.011630	0.011549	0.011255	0.011312	0.011372	0.011406	0.011361
0.011623	0.011544	0.011542	0.011391	0.011362	0.011530	0.011534
0.011404	0.011497	0.011497	0.011463	0.011429	0.011430	0.011396
0.011430	0.011362	0.011396	0.011430	0.011262	0.011329	0.011362
0.011362	0.011430	0.011329	0.011430	0.011463	0.011497	0.011396
0.011429	0.011396	0.011732	0.011732	0.011463	0.011497	0.011530
0.011295	0.011295	0.011329	0.011430	0.011295	0.011530	0.011530
0.011564	0.011564	0.011362	0.011597	0.011766	0.011599	0.011430
0.011430	0.011497	0.011530	0.011362	0.011430	0.011396	0.011430
0.011497	0.011497	0.011530	0.011497	0.011397	0.011597	0.011564
0.011433	0.011329	0.011430	0.011530	0.011430	0.011564	0.011597
0.011765	0.011470	0.011631	0.011597	0.011530	0.011698	0.011329
0.011498	0.011396	0.011362	0.011362	0.011430	0.011329	0.011430
0.011463	0.011497	0.011396	0.011429	0.011396	0.011732	0.011732
0.011463	0.011497	0.011564	0.011496	0.012069	0.011560	0.011482
0.011552	0.011511	0.011622	0.011510	0.011620	0.011543	0.011508
0.012022	0.011406	0.011540	0.011475	0.011749	0.011604	0.011707
0.012046	0.011588	0.011669	0.011732	0.011597	0.011597	0.011635
0.011295	0.011463	0.011597	0.011564	0.011567	0.011564	0.011532
0.011396	0.011329	0.011362	0.011396	0.011295	0.011362	0.011262
0.011396	0.011429	0.011329	0.011295	0.011396	0.011329	0.011362
0.011362	0.011295	0.011362	0.011396	0.011295	0.011497	0.011530
0.011429	0.011497	0.011429	0.011429	0.011362	0.011765	0.011396
0.011443	0.011362	0.011598	0.011603	0.011463	0.011463	0.011463
0.011463	0.011396	0.011362	0.011396	0.011497	0.011497	0.011295
0.011429	0.011564	0.011732	0.011463	0.011665	0.011665	0.011496
0.011362	0.011429	0.011463	0.011866	0.011430	0.011463	0.011295
0.011463	0.011396	0.011396	0.011698	0.011631	0.011665	0.011396
0.011497	0.011564	0.011429	0.011497	0.011463	0.011329	0.011664
0.011429	0.011396	0.011396	0.011564	0.011396	0.011496	0.011732
0.011429	0.011773	0.011799	0.011589	0.011399	0.011502	0.011267
0.011225	0.011800	0.011341	0.011507	0.011353	0.011467	0.011485
0.011621	0.011324	0.011450	0.011324	0.011330	0.011462	0.011425
0.011475	0.011336	0.011483	0.011412	0.011431	0.011564	0.011631
0.011631	0.011497	0.011601	0.011603	0.011738	0.011767	0.011429
0.011664	0.011530	0.011396	0.011497	0.011295	0.011530	0.011329
0.011396	0.011396	0.011362	0.011329	0.011362	0.011329	0.011430
0.011410	0.011410	0.011403	0.011437	0.011573	0.011538	0.011538
0.011537	0.011464	0.011282	0.011276	0.011399	0.011568	0.011569
0.011363	0.011433	0.011666	0.011635	0.011565	0.011599	0.011530
0.011463	0.011530	0.011530	0.011429	0.012001	0.011597	0.011665
0.011698	0.011772	0.011401	0.011372	0.011362	0.011564	0.011530
0.011565	0.011597	0.011597	0.011597	0.011768	0.011732	0.011372
0.011263	0.011268	0.011367	0.011332	0.011436	0.011284	0.011604
0.011403	0.011586	0.011298	0.011397	0.011396	0.011471	0.011362
0.011329	0.011329	0.011396	0.011362	0.011564	0.011530	0.011463
0.011564	0.011631	0.011362	0.011362	0.011396	0.011396	0.011398
0.011466	0.011396	0.011396	0.011431	0.011564	0.011631	0.011631
0.011497	0.011601	0.011603	0.011738	0.011767	0.011429	0.011597
0.011530	0.011530	0.011365	0.011547	0.011442	0.011503	0.011376
0.011508	0.011744	0.011526	0.011434	0.011329	0.011530	0.011396
0.011497	0.011430	0.011329	0.011431	0.011530	0.011533	0.011430
0.011329	0.011263	0.011396	0.011329	0.011262	0.011332	0.011531
0.011430	0.011362	0.011396	0.011430	0.011262	0.011329	0.011530
0.011396	0.011429	0.011429	0.011362	0.011429	0.011664	0.011329
0.011362	0.011765	0.011362	0.011396	0.011362	0.011732	0.011317
0.011791	0.011315	0.011411	0.011584	0.011630	0.011563	0.011269
0.011375	0.011530	0.011631	0.011739	0.011599	0.011737	0.011867

0.011767	0.011770	0.011765	0.012017	0.011732	0.012606	0.011768
0.012473	0.012274	0.012409	0.012680	0.011731	0.011430	0.011732
0.012104	0.012106	0.011698	0.011597	0.011665	0.011665	0.011635
0.011530	0.011535	0.011597	0.011565	0.011935	0.012001	0.012002
0.011901	0.012002	0.012106	0.011872	0.012104	0.012110	0.012171
0.012172	0.012206	0.012152	0.012172	0.012173	0.011463	0.011732
0.011834	0.012337	0.011840	0.012068	0.012541	0.011604	0.011497
0.011463	0.011463	0.011934	0.011965	0.012081	0.011799	0.011465
S124	0.011698	0.011698	0.012035	0.011799	0.011799	0.011866
0.011706	0.011638	0.011866	0.011295	0.011362	0.011330	0.011295
0.011329	0.011497	0.011295	0.011329	0.011396	0.011362	0.011329
0.011295	0.011329	0.011429	0.011295	0.011329	0.011329	0.011672
0.011564	0.011329	0.011228	0.011463	0.011396	0.011362	0.011396
0.011362	0.011463	0.011362	0.011362	0.011396	0.011362	0.011530
0.011295	0.011362	0.011396	0.011329	0.011429	0.011463	0.011396
0.011362	0.011530	0.011430	0.011698	0.011362	0.011631	0.011362
0.011329	0.011430	0.011497	0.011631	0.011732	0.011430	0.011530
0.011396	0.011463	0.011362	0.011597	0.011833	0.011362	0.011430
0.011329	0.011429	0.011396	0.011463	0.011396	0.011396	0.011396
0.011295	0.011329	0.011295	0.011396	0.011362	0.011397	0.011463
0.011396	0.011362	0.011430	0.011329	0.011463	0.011295	0.011362
0.011530	0.011463	0.011698	0.011530	0.011362	0.011597	0.011362
0.011542	0.011770	0.011589	0.011295	0.011284	0.011392	0.011454
0.012122	0.011569	0.011307	0.011597	0.011597	0.011396	0.011497
0.011430	0.011396	0.011463	0.011362	0.011463	0.011396	0.011396
0.011362	0.011463	0.011665	0.011530	0.011430	0.000000	0.011329
0.011295	0.011295	0.011363	0.011329	0.011530	0.011497	0.011430
0.011530	0.011598	0.011329	0.011329	0.011363	0.011362	0.011364
0.011432	0.011363	0.011363	0.011240	0.011313	0.011271	0.011363
0.011365	0.011301	0.011355	0.011336	0.011264	0.011300	0.011331
0.011600	0.011371	0.011405	0.011296	0.011302	0.011430	0.011397
0.011464	0.011397	0.011397	0.011497	0.011429	0.011799	0.011598
0.011631	0.011497	0.011362	0.011463	0.011262	0.011497	0.011295
0.011362	0.011362	0.011329	0.011295	0.011329	0.011295	0.011396
0.011363	0.011463	0.011396	0.011295	0.011397	0.011497	0.011500
0.011497	0.011396	0.011295	0.011229	0.011362	0.011295	0.011228
0.011332	0.011497	0.011295	0.011497	0.011262	0.011363	0.011329
0.011430	0.011329	0.011363	0.011329	0.011363	0.011228	0.011363
0.011396	0.011329	0.011396	0.011329	0.011362	0.011396	0.011631
0.011732	0.011665	0.011732	0.011499	0.011732	0.011362	0.011329
0.011364	0.011667	0.011426	0.011266	0.011739	0.011231	0.011375
0.011740	0.011638	0.011530	0.011732	0.011436	0.011597	0.011564
0.011497	0.011665	0.011522	0.011390	0.011438	0.011396	0.011466
0.011564	0.011465	0.011567	0.011669	0.011598	0.011665	0.011772
0.011500	0.011671	0.011602	0.011534	0.011801	0.011262	0.011465
0.011429	0.011362	0.011262	0.011262	0.011329	0.011329	0.011396
0.011329	0.011396	0.011295	0.011396	0.011329	0.011329	0.011396
0.011329	0.011765	0.011665	0.011698	0.011228	0.011329	0.011362
0.011362	0.011329	0.011396	0.011430	0.011295	0.011295	0.011295
0.011833	0.011463	0.011463	0.011262	0.011362	0.011530	0.011295
0.011295	0.011362	0.011329	0.011497	0.011295	0.011429	0.011564
0.011497	0.011329	0.011329	0.011262	0.011262	0.011430	0.011362
0.011262	0.011362	0.011228	0.011262	0.011396	0.011531	0.011295
0.011262	0.011396	0.011329	0.011228	0.011564	0.011228	0.011396
0.011471	0.011295	0.011597	0.011430	0.011463	0.011329	0.011429
0.011295	0.011631	0.011228	0.011497	0.011295	0.011295	0.011228
0.011463	0.011665	0.011362	0.011430	0.011262	0.011665	0.011295
0.011295	0.011295	0.011329	0.011430	0.011329	0.011429	0.011497
0.011497	0.011463	0.011362	0.011463	0.011429	0.011295	0.011262
0.011295	0.011295	0.011362	0.011295	0.011262	0.011430	0.011295

0.011329	0.011329	0.011463	0.011497	0.011396	0.011329	0.011329
0.011396	0.011329	0.011429	0.011295	0.011430	0.011530	0.011833
0.011329	0.011732	0.011336	0.017864	0.011322	0.011611	0.011688
0.011649	0.011694	0.011480	0.011467	0.011775	0.011716	0.011425
0.011582	0.011671	0.011255	0.011354	0.011334	0.011406	0.011362
0.011577	0.011507	0.011505	0.011259	0.011463	0.011497	0.011500
0.011369	0.011463	0.011463	0.011430	0.011396	0.011396	0.011363
0.011396	0.011329	0.011362	0.011396	0.011363	0.011295	0.011329
0.011329	0.011396	0.011295	0.011396	0.011362	0.011463	0.011362
0.011396	0.011363	0.011699	0.011699	0.011430	0.011464	0.011497
0.011262	0.011262	0.011295	0.011396	0.011262	0.011497	0.011497
0.011530	0.011530	0.011463	0.011564	0.011732	0.011565	0.011396
0.011396	0.011463	0.011497	0.011329	0.011396	0.011362	0.011396
0.011464	0.011463	0.011497	0.011463	0.011363	0.011564	0.011530
0.011365	0.011295	0.011396	0.011497	0.011396	0.011531	0.011564
0.011732	0.011436	0.011597	0.011564	0.011497	0.011665	0.011296
0.011465	0.011363	0.011329	0.011329	0.011396	0.011295	0.011396
0.011362	0.011463	0.011362	0.011396	0.011363	0.011699	0.011699
0.011430	0.011464	0.011530	0.011463	0.012103	0.011525	0.011447
0.011517	0.011476	0.011587	0.011476	0.011550	0.011509	0.011474
0.012056	0.011372	0.011506	0.011441	0.011715	0.011570	0.011673
0.012080	0.011550	0.011635	0.011698	0.011564	0.011564	0.011601
0.011262	0.011430	0.011564	0.011530	0.011533	0.011530	0.011498
0.011362	0.011295	0.011329	0.011362	0.011262	0.011329	0.011228
0.011362	0.011396	0.011295	0.011262	0.011362	0.011295	0.011329
0.011329	0.011262	0.011329	0.011362	0.011262	0.011463	0.011497
0.011396	0.011463	0.011396	0.011396	0.011329	0.011732	0.011362
0.011408	0.011329	0.011564	0.011569	0.011430	0.011429	0.011429
0.011430	0.011362	0.011329	0.011362	0.011463	0.011463	0.011396
0.011396	0.011530	0.011698	0.011429	0.011631	0.011631	0.011497
0.011329	0.011396	0.011430	0.011833	0.011396	0.011430	0.011396
0.011430	0.011362	0.011362	0.011665	0.011598	0.011631	0.011362
0.011463	0.011530	0.011396	0.011463	0.011429	0.011295	0.011631
0.011396	0.011362	0.011362	0.011530	0.011362	0.011463	0.011698
0.011396	0.011774	0.011765	0.011589	0.011286	0.011468	0.011370
0.011390	0.011800	0.011306	0.011507	0.011304	0.011417	0.011432
0.011520	0.011258	0.011394	0.011259	0.011276	0.011462	0.011425
0.011437	0.011336	0.011448	0.011377	0.011397	0.011530	0.011598
0.011598	0.011464	0.011568	0.011569	0.011705	0.011732	0.011396
0.011631	0.011497	0.011362	0.011463	0.011262	0.011497	0.011295
0.011362	0.011362	0.011329	0.011295	0.011329	0.011295	0.011396
0.011375	0.011375	0.011369	0.011403	0.011539	0.011504	0.011504
0.011503	0.011430	0.011245	0.011240	0.011365	0.011535	0.011535
0.011329	0.011399	0.011633	0.011601	0.011531	0.011565	0.011497
0.011429	0.011497	0.011497	0.011396	0.011967	0.011564	0.011564
0.011665	0.011737	0.011367	0.011441	0.011329	0.011530	0.011497
0.011531	0.011564	0.011564	0.011564	0.011689	0.011698	0.011338
0.011229	0.011233	0.011333	0.011298	0.011402	0.011247	0.011570
0.011369	0.011543	0.011299	0.011398	0.011363	0.011437	0.011329
0.011295	0.011295	0.011363	0.011329	0.011530	0.011497	0.011430
0.011530	0.011598	0.011329	0.011329	0.011363	0.011362	0.011364
0.011432	0.011363	0.011363	0.011397	0.011530	0.011598	0.011598
0.011464	0.011568	0.011569	0.011705	0.011732	0.011396	0.011564
0.011497	0.011497	0.011331	0.011513	0.011443	0.011469	0.011341
0.011508	0.011698	0.011441	0.011400	0.011295	0.011497	0.011363
0.011463	0.011396	0.011295	0.011397	0.011497	0.011500	0.011396
0.011295	0.011229	0.011362	0.011295	0.011228	0.011332	0.011497
0.011396	0.011329	0.011362	0.011396	0.011363	0.011295	0.011497
0.011362	0.011396	0.011396	0.011329	0.011396	0.011631	0.011295
0.011329	0.011732	0.011329	0.011362	0.011329	0.011698	0.011317

0.011791	0.011315	0.011361	0.011544	0.011630	0.011527	0.011269
0.011323	0.011497	0.011597	0.011706	0.011565	0.011703	0.011834
0.011733	0.011771	0.011732	0.011983	0.011698	0.012573	0.011734
0.012474	0.012241	0.012375	0.012647	0.011698	0.011396	0.011699
0.012138	0.012073	0.011665	0.011564	0.011632	0.011632	0.011601
0.011497	0.011501	0.011564	0.011531	0.011901	0.011900	0.011969
0.011868	0.011969	0.012072	0.011839	0.012138	0.012110	0.012205
0.012206	0.012240	0.012187	0.012206	0.012207	0.011429	0.011733
0.011801	0.012304	0.011806	0.012035	0.012508	0.011570	0.011464
0.011429	0.011429	0.011901	0.011966	0.012046	0.011765	0.011431
S125	0.011631	0.011631	0.011967	0.011732	0.011732	0.011799
0.011638	0.011570	0.011732	0.011228	0.011295	0.011262	0.011228
0.011262	0.011430	0.011228	0.011262	0.011329	0.011295	0.011262
0.011228	0.011262	0.011362	0.011228	0.011195	0.011262	0.011604
0.011497	0.011262	0.011161	0.011396	0.011262	0.011295	0.011262
0.011295	0.011329	0.011295	0.011295	0.011329	0.011295	0.011463
0.011228	0.011295	0.011329	0.011262	0.011362	0.011396	0.011329
0.011295	0.011463	0.011363	0.011631	0.011295	0.011497	0.011295
0.011262	0.011363	0.011430	0.011497	0.011665	0.011362	0.011463
0.011262	0.011329	0.011295	0.011463	0.011766	0.011295	0.011362
0.011262	0.011362	0.011329	0.011396	0.011329	0.011329	0.011329
0.011228	0.011262	0.011228	0.011329	0.011295	0.011329	0.011396
0.011329	0.011295	0.011295	0.011262	0.011396	0.011161	0.011295
0.011463	0.011396	0.011631	0.011463	0.011295	0.011463	0.011295
0.011468	0.011695	0.011513	0.011216	0.011210	0.011318	0.011415
0.012048	0.011467	0.011225	0.011530	0.011530	0.011329	0.011430
0.011362	0.011329	0.011396	0.011295	0.011396	0.011262	0.011329
0.011295	0.011396	0.011598	0.011463	0.011362	0.011329	0.000000
0.011228	0.011228	0.011228	0.011262	0.011463	0.011430	0.011362
0.011463	0.011530	0.011262	0.011262	0.011295	0.011295	0.011297
0.011364	0.011295	0.011295	0.011132	0.011241	0.011201	0.011296
0.011297	0.011232	0.011282	0.011267	0.011196	0.011232	0.011263
0.011528	0.011302	0.011267	0.011228	0.011233	0.011363	0.011329
0.011397	0.011330	0.011329	0.011429	0.011362	0.011665	0.011531
0.011564	0.011430	0.011295	0.011396	0.011195	0.011429	0.011228
0.011295	0.011295	0.011262	0.011228	0.011262	0.011228	0.011329
0.011228	0.011329	0.011329	0.011228	0.011330	0.011430	0.011432
0.011362	0.011329	0.011228	0.011161	0.011295	0.011228	0.011161
0.011264	0.011362	0.011161	0.011362	0.011195	0.011295	0.011262
0.011295	0.011262	0.011228	0.011262	0.011295	0.011161	0.011228
0.011262	0.011262	0.011329	0.011262	0.011295	0.011329	0.011564
0.011665	0.011597	0.011665	0.011431	0.011665	0.011295	0.011262
0.011297	0.011545	0.011335	0.011198	0.011671	0.011163	0.011296
0.011637	0.011570	0.011463	0.011665	0.011368	0.011530	0.011430
0.011430	0.011597	0.011451	0.011307	0.011354	0.011329	0.011399
0.011497	0.011398	0.011534	0.011534	0.011530	0.011598	0.011704
0.011433	0.011536	0.011534	0.011466	0.011733	0.011195	0.011397
0.011295	0.011295	0.011195	0.011195	0.011195	0.011262	0.011329
0.011195	0.011262	0.011161	0.011329	0.011195	0.011262	0.011329
0.011262	0.011698	0.011598	0.011631	0.011161	0.011262	0.011295
0.011295	0.011262	0.011329	0.011362	0.011228	0.011228	0.011228
0.011765	0.011396	0.011396	0.011195	0.011295	0.011396	0.011228
0.011228	0.011295	0.011262	0.011362	0.011228	0.011362	0.011429
0.011430	0.011262	0.011262	0.011195	0.011195	0.011363	0.011295
0.011195	0.011295	0.011161	0.011195	0.011329	0.011463	0.011228
0.011195	0.011329	0.011262	0.011161	0.011497	0.011161	0.011329
0.011334	0.011228	0.011530	0.011363	0.011396	0.011262	0.011362
0.011228	0.011497	0.011161	0.011430	0.011228	0.011228	0.011161
0.011329	0.011598	0.011295	0.011362	0.011195	0.011598	0.011228
0.011228	0.011228	0.011262	0.011295	0.011262	0.011362	0.011430

0.011430	0.011396	0.011295	0.011396	0.011295	0.011228	0.011195
0.011228	0.011228	0.011295	0.011228	0.011127	0.011362	0.011228
0.011262	0.011262	0.011396	0.011430	0.011329	0.011195	0.011262
0.011329	0.011262	0.011362	0.011228	0.011362	0.011463	0.011766
0.011262	0.011665	0.011267	0.017785	0.011257	0.011488	0.011618
0.011579	0.011623	0.011322	0.011292	0.011719	0.011647	0.011364
0.011487	0.011610	0.011177	0.011228	0.011216	0.011357	0.011275
0.011482	0.011433	0.011394	0.011126	0.011396	0.011430	0.011432
0.011232	0.011396	0.011396	0.011362	0.011329	0.011329	0.011295
0.011329	0.011262	0.011295	0.011329	0.011295	0.011228	0.011262
0.011262	0.011329	0.011228	0.011329	0.011362	0.011396	0.011295
0.011329	0.011295	0.011631	0.011631	0.011363	0.011397	0.011430
0.011195	0.011195	0.011228	0.011262	0.011195	0.011430	0.011430
0.011463	0.011463	0.011396	0.011497	0.011665	0.011498	0.011262
0.011329	0.011396	0.011362	0.011262	0.011329	0.011295	0.011329
0.011396	0.011396	0.011430	0.011329	0.011229	0.011497	0.011396
0.011331	0.011228	0.011329	0.011430	0.011329	0.011463	0.011497
0.011665	0.011368	0.011530	0.011430	0.011430	0.011597	0.011228
0.011397	0.011296	0.011262	0.011262	0.011329	0.011228	0.011329
0.011362	0.011396	0.011295	0.011329	0.011295	0.011631	0.011631
0.011363	0.011397	0.011463	0.011396	0.012036	0.011453	0.011377
0.011447	0.011407	0.011517	0.011407	0.011515	0.011440	0.011405
0.011988	0.011303	0.011437	0.011372	0.011646	0.011502	0.011537
0.012012	0.011475	0.011331	0.011329	0.011329	0.011362	0.011297
0.011195	0.011161	0.011362	0.011329	0.011331	0.011463	0.011431
0.011295	0.011161	0.011195	0.011295	0.011127	0.011262	0.011161
0.011295	0.011329	0.011228	0.011195	0.011295	0.011228	0.011262
0.011262	0.011195	0.011262	0.011295	0.011195	0.011329	0.011430
0.011329	0.011396	0.011329	0.011329	0.011262	0.011665	0.011295
0.011339	0.011262	0.011497	0.011501	0.011295	0.011362	0.011362
0.011362	0.011228	0.011262	0.011228	0.011396	0.011396	0.011329
0.011329	0.011463	0.011631	0.011362	0.011564	0.011564	0.011430
0.011262	0.011329	0.011362	0.011766	0.011329	0.011295	0.011329
0.011362	0.011295	0.011295	0.011598	0.011463	0.011564	0.011295
0.011396	0.011463	0.011329	0.011396	0.011362	0.011228	0.011564
0.011329	0.011295	0.011295	0.011463	0.011295	0.011396	0.011631
0.011329	0.011706	0.011698	0.011507	0.011229	0.011400	0.011267
0.011280	0.011722	0.011236	0.011462	0.011255	0.011417	0.011379
0.011468	0.011192	0.011339	0.011192	0.011222	0.011395	0.011344
0.011362	0.011218	0.011377	0.011307	0.011330	0.011463	0.011530
0.011530	0.011396	0.011500	0.011501	0.011636	0.011661	0.011329
0.011564	0.011430	0.011295	0.011396	0.011195	0.011429	0.011228
0.011295	0.011295	0.011262	0.011228	0.011262	0.011228	0.011329
0.011305	0.011305	0.011300	0.011334	0.011471	0.011368	0.011436
0.011435	0.011363	0.011171	0.011168	0.011297	0.011467	0.011467
0.011262	0.011331	0.011565	0.011533	0.011464	0.011498	0.011430
0.011362	0.011430	0.011429	0.011329	0.011900	0.011497	0.011564
0.011598	0.011666	0.011299	0.011372	0.011262	0.011463	0.011430
0.011464	0.011497	0.011497	0.011497	0.011650	0.011631	0.011268
0.011162	0.011164	0.011265	0.011230	0.011334	0.011172	0.011502
0.011300	0.011411	0.011156	0.011322	0.011296	0.011368	0.000000
0.011228	0.011228	0.011228	0.011262	0.011463	0.011430	0.011362
0.011463	0.011530	0.011262	0.011262	0.011295	0.011295	0.011297
0.011364	0.011295	0.011295	0.011330	0.011463	0.011530	0.011530
0.011396	0.011500	0.011501	0.011636	0.011661	0.011329	0.011497
0.011430	0.011430	0.011263	0.011443	0.011373	0.011401	0.011270
0.011370	0.011607	0.011399	0.011332	0.011161	0.011362	0.011228
0.011329	0.011329	0.011228	0.011330	0.011430	0.011432	0.011329
0.011228	0.011161	0.011295	0.011228	0.011161	0.011264	0.011362
0.011329	0.011262	0.011295	0.011329	0.011295	0.011228	0.011430

0.011295	0.011329	0.011329	0.011262	0.011329	0.011564	0.011228
0.011262	0.011665	0.011262	0.011228	0.011262	0.011631	0.011232
0.011679	0.011230	0.011261	0.011463	0.011549	0.011455	0.011186
0.011218	0.011430	0.011530	0.011637	0.011498	0.011635	0.011766
0.011666	0.011703	0.011665	0.011915	0.011631	0.012506	0.011667
0.012406	0.012173	0.012307	0.012579	0.011631	0.011329	0.011631
0.012070	0.012005	0.011598	0.011497	0.011565	0.011565	0.011534
0.011430	0.011433	0.011497	0.011464	0.011834	0.011900	0.011901
0.011801	0.011901	0.012004	0.011771	0.012070	0.012076	0.012137
0.012138	0.012172	0.012118	0.012138	0.012139	0.011362	0.011665
0.011733	0.012237	0.011738	0.011968	0.012440	0.011502	0.011396
0.011362	0.011362	0.011833	0.011883	0.011975	0.011698	0.011363
S126	0.011597	0.011597	0.011934	0.011698	0.011698	0.011765
0.011604	0.011536	0.011698	0.011195	0.011262	0.011228	0.011195
0.011228	0.011396	0.011195	0.011228	0.011295	0.011262	0.011228
0.011195	0.011228	0.011329	0.011195	0.011228	0.011228	0.011570
0.011463	0.011228	0.011127	0.011362	0.011295	0.011262	0.011295
0.011262	0.011362	0.011262	0.011262	0.011295	0.011262	0.011430
0.011195	0.011262	0.011295	0.011228	0.011329	0.011362	0.011295
0.011262	0.011430	0.011329	0.011597	0.011262	0.011530	0.011262
0.011228	0.011329	0.011396	0.011530	0.011631	0.011329	0.011430
0.011295	0.011362	0.011262	0.011497	0.011732	0.011262	0.011329
0.011228	0.011329	0.011295	0.011362	0.011295	0.011295	0.011295
0.011195	0.011228	0.011195	0.011295	0.011262	0.011296	0.011362
0.011295	0.011262	0.011329	0.011228	0.011362	0.011195	0.011262
0.011429	0.011362	0.011597	0.011430	0.011262	0.011497	0.011262
0.011394	0.011620	0.011475	0.011138	0.011172	0.011244	0.011336
0.012011	0.011416	0.011184	0.011497	0.011497	0.011295	0.011396
0.011329	0.011295	0.011362	0.011262	0.011362	0.011295	0.011295
0.011262	0.011362	0.011564	0.011429	0.011329	0.011295	0.011228
0.000000	0.011195	0.011262	0.011228	0.011429	0.011396	0.011329
0.011429	0.011497	0.011228	0.011228	0.011262	0.011262	0.011263
0.011331	0.011262	0.011262	0.011132	0.011205	0.011165	0.011262
0.011263	0.011198	0.011245	0.011233	0.011162	0.011197	0.011229
0.011492	0.011267	0.011301	0.011195	0.011199	0.011329	0.011296
0.011363	0.011296	0.011296	0.011396	0.011329	0.011698	0.011497
0.011530	0.011396	0.011262	0.011362	0.011161	0.011396	0.011195
0.011262	0.011262	0.011228	0.011195	0.011228	0.011195	0.011295
0.011262	0.011362	0.011295	0.011195	0.011296	0.011396	0.011398
0.011396	0.011295	0.011195	0.011128	0.011262	0.011195	0.011127
0.011230	0.011396	0.011195	0.011396	0.011161	0.011262	0.011228
0.011329	0.011228	0.011262	0.011228	0.011262	0.011127	0.011262
0.011295	0.011228	0.011295	0.011228	0.011262	0.011228	0.011530
0.011631	0.011564	0.011631	0.011397	0.011631	0.011262	0.011228
0.011263	0.011505	0.011380	0.011129	0.011637	0.011129	0.011257
0.011637	0.011536	0.011429	0.011631	0.011333	0.011497	0.011463
0.011396	0.011564	0.011416	0.011266	0.011312	0.011295	0.011365
0.011463	0.011364	0.011500	0.011568	0.011497	0.011564	0.011670
0.011399	0.011569	0.011500	0.011432	0.011700	0.011161	0.011364
0.011329	0.011262	0.011161	0.011161	0.011228	0.011228	0.011295
0.011228	0.011295	0.011195	0.011295	0.011228	0.011228	0.011295
0.011228	0.011665	0.011564	0.011597	0.011127	0.011228	0.011262
0.011262	0.011228	0.011295	0.011329	0.011195	0.011195	0.011195
0.011732	0.011362	0.011362	0.011161	0.011262	0.011430	0.011195
0.011195	0.011262	0.011228	0.011396	0.011195	0.011329	0.011463
0.011396	0.011228	0.011228	0.011161	0.011161	0.011329	0.011262
0.011161	0.011262	0.011127	0.011161	0.011295	0.011430	0.011195
0.011161	0.011295	0.011228	0.011127	0.011463	0.011127	0.011295
0.011368	0.011195	0.011497	0.011329	0.011362	0.011228	0.011329
0.011195	0.011530	0.011127	0.011396	0.011195	0.011195	0.011127

0.011362	0.011564	0.011262	0.011329	0.011161	0.011564	0.011195
0.011195	0.011195	0.011228	0.011329	0.011228	0.011329	0.011396
0.011396	0.011362	0.011262	0.011362	0.011329	0.011195	0.011161
0.011195	0.011195	0.011262	0.011195	0.011161	0.011329	0.011195
0.011228	0.011228	0.011362	0.011396	0.011295	0.011228	0.011228
0.011295	0.011228	0.011329	0.011195	0.011329	0.011429	0.011732
0.011228	0.011631	0.011233	0.017747	0.011126	0.011366	0.011583
0.011545	0.011588	0.011217	0.011234	0.011609	0.011612	0.011242
0.011440	0.011487	0.011099	0.011144	0.011177	0.011258	0.011146
0.011436	0.011395	0.011357	0.011193	0.011362	0.011396	0.011398
0.011266	0.011362	0.011362	0.011329	0.011295	0.011295	0.011262
0.011295	0.011228	0.011262	0.011295	0.011262	0.011195	0.011228
0.011228	0.011295	0.011195	0.011295	0.011329	0.011362	0.011262
0.011295	0.011262	0.011597	0.011597	0.011329	0.011363	0.011396
0.011161	0.011161	0.011195	0.011295	0.011161	0.011396	0.011396
0.011429	0.011429	0.011362	0.011463	0.011631	0.011464	0.011295
0.011295	0.011362	0.011396	0.011228	0.011295	0.011262	0.011295
0.011363	0.011362	0.011396	0.011363	0.011262	0.011463	0.011429
0.011297	0.011195	0.011295	0.011396	0.011295	0.011430	0.011396
0.011631	0.011333	0.011497	0.011463	0.011396	0.011564	0.011195
0.011363	0.011262	0.011228	0.011228	0.011295	0.011195	0.011295
0.011329	0.011362	0.011262	0.011295	0.011262	0.011597	0.011597
0.011329	0.011363	0.011429	0.011362	0.012002	0.011417	0.011342
0.011412	0.011372	0.011482	0.011372	0.011480	0.011405	0.011371
0.011953	0.011268	0.011403	0.011337	0.011611	0.011468	0.011571
0.011978	0.011437	0.011297	0.011295	0.011295	0.011329	0.011263
0.011161	0.011195	0.011329	0.011295	0.011297	0.011429	0.011397
0.011262	0.011195	0.011228	0.011262	0.011161	0.011228	0.011127
0.011262	0.011295	0.011195	0.011161	0.011262	0.011195	0.011228
0.011228	0.011161	0.011228	0.011262	0.011161	0.011362	0.011396
0.011295	0.011362	0.011295	0.011295	0.011228	0.011631	0.011262
0.011304	0.011228	0.011463	0.011467	0.011329	0.011329	0.011329
0.011329	0.011262	0.011228	0.011262	0.011362	0.011362	0.011295
0.011295	0.011430	0.011597	0.011329	0.011530	0.011530	0.011396
0.011228	0.011295	0.011329	0.011732	0.011295	0.011329	0.011295
0.011329	0.011262	0.011262	0.011564	0.011497	0.011530	0.011262
0.011362	0.011430	0.011295	0.011362	0.011329	0.011195	0.011530
0.011295	0.011262	0.011262	0.011430	0.011262	0.011362	0.011598
0.011295	0.011672	0.011665	0.011426	0.011173	0.011366	0.011215
0.011170	0.011683	0.011201	0.011373	0.011158	0.011315	0.011273
0.011417	0.000000	0.011227	0.000000	0.011114	0.011261	0.011263
0.011286	0.011257	0.011342	0.011272	0.011296	0.011429	0.011497
0.011497	0.011363	0.011466	0.011467	0.011602	0.011626	0.011295
0.011530	0.011396	0.011262	0.011362	0.011161	0.011396	0.011195
0.011262	0.011262	0.011228	0.011195	0.011228	0.011195	0.011295
0.011270	0.011270	0.011266	0.011300	0.011437	0.011402	0.011402
0.011401	0.011329	0.011134	0.011132	0.011263	0.011433	0.011433
0.011228	0.011297	0.011531	0.011500	0.011430	0.011464	0.011396
0.011329	0.011396	0.011396	0.011295	0.011866	0.011463	0.011530
0.011564	0.011630	0.011265	0.011337	0.011228	0.011430	0.011396
0.011430	0.011463	0.011463	0.011463	0.011610	0.011597	0.011234
0.011128	0.011130	0.011231	0.011196	0.011299	0.011135	0.011468
0.011266	0.011411	0.011156	0.011285	0.011262	0.011334	0.011228
0.000000	0.011195	0.011262	0.011228	0.011429	0.011396	0.011329
0.011429	0.011497	0.011228	0.011228	0.011262	0.011262	0.011263
0.011331	0.011262	0.011262	0.011296	0.011429	0.011497	0.011497
0.011363	0.011466	0.011467	0.011602	0.011626	0.011295	0.011396
0.011396	0.011396	0.011229	0.011408	0.011338	0.011367	0.011235
0.011405	0.011561	0.011399	0.011298	0.011195	0.011396	0.011262
0.011362	0.011295	0.011195	0.011296	0.011396	0.011398	0.011295

0.011195	0.011128	0.011262	0.011195	0.011127	0.011230	0.011396
0.011295	0.011228	0.011262	0.011295	0.011262	0.011195	0.011396
0.011262	0.011295	0.011295	0.011228	0.011295	0.011530	0.011195
0.011228	0.011631	0.011228	0.011262	0.011228	0.011597	0.011189
0.011622	0.011188	0.011211	0.011422	0.011508	0.011419	0.011144
0.011165	0.011396	0.011497	0.011603	0.011464	0.011601	0.011733
0.011632	0.011669	0.011631	0.011880	0.011597	0.012472	0.011633
0.012372	0.012139	0.012273	0.012545	0.011597	0.011295	0.011598
0.012036	0.011971	0.011564	0.011463	0.011531	0.011531	0.011500
0.011396	0.011399	0.011463	0.011430	0.011800	0.011866	0.011867
0.011767	0.011867	0.011970	0.011737	0.012036	0.012042	0.012104
0.012104	0.012138	0.012084	0.012104	0.012105	0.011329	0.011631
0.011699	0.012203	0.011704	0.011934	0.012406	0.011468	0.011363
0.011329	0.011329	0.011799	0.011842	0.011940	0.011665	0.011330
S127	0.011597	0.011597	0.011866	0.011631	0.011631	0.011698
0.011536	0.011468	0.011631	0.011195	0.011262	0.011228	0.011195
0.011161	0.011396	0.011127	0.011228	0.011295	0.011262	0.011228
0.011195	0.011228	0.011262	0.011195	0.011228	0.011228	0.011502
0.011396	0.011228	0.011127	0.011295	0.011295	0.011262	0.011295
0.011262	0.011362	0.011262	0.011262	0.011295	0.011262	0.011429
0.011127	0.011262	0.011295	0.011228	0.011262	0.011362	0.011295
0.011262	0.011430	0.011329	0.011530	0.011262	0.011530	0.011262
0.011228	0.011262	0.011396	0.011530	0.011564	0.011262	0.011362
0.011295	0.011362	0.011262	0.011497	0.011665	0.011262	0.011329
0.011228	0.011329	0.011295	0.011362	0.011295	0.011295	0.011295
0.011195	0.011228	0.011195	0.011228	0.011262	0.011296	0.011362
0.011295	0.011262	0.011329	0.011228	0.011295	0.011195	0.011262
0.011429	0.011362	0.011597	0.011430	0.011262	0.011497	0.011262
0.011431	0.011583	0.011475	0.011177	0.011172	0.011281	0.011296
0.011938	0.011365	0.011184	0.011497	0.011497	0.011228	0.011329
0.011329	0.011295	0.011362	0.011262	0.011362	0.011295	0.011295
0.011262	0.011295	0.011564	0.011429	0.011329	0.011295	0.011228
0.011195	0.000000	0.011262	0.011228	0.011362	0.011329	0.011262
0.011362	0.011429	0.011228	0.011228	0.011262	0.011262	0.011263
0.011331	0.011262	0.011262	0.011132	0.011205	0.011165	0.011262
0.011263	0.011198	0.011245	0.011233	0.011162	0.011197	0.011229
0.011420	0.011267	0.011301	0.011195	0.011199	0.011329	0.011228
0.011296	0.011296	0.011296	0.011396	0.011329	0.011698	0.011430
0.011463	0.011329	0.011262	0.011295	0.011161	0.011329	0.011195
0.011262	0.011262	0.011228	0.011195	0.011228	0.011195	0.011295
0.011262	0.011362	0.011295	0.011195	0.011296	0.011396	0.011398
0.011396	0.011295	0.011195	0.011128	0.011262	0.011195	0.011127
0.011230	0.011396	0.011195	0.011396	0.011161	0.011262	0.011228
0.011329	0.011228	0.011262	0.011228	0.011262	0.011127	0.011262
0.011295	0.011228	0.011295	0.011228	0.011262	0.011295	0.011530
0.011564	0.011497	0.011564	0.011397	0.011564	0.011262	0.011228
0.011195	0.011464	0.011335	0.011163	0.011569	0.011129	0.011257
0.011569	0.011434	0.011429	0.011631	0.011333	0.011497	0.011463
0.011396	0.011564	0.011416	0.011266	0.011312	0.011295	0.011365
0.011463	0.011364	0.011500	0.011568	0.011497	0.011564	0.011670
0.011399	0.011569	0.011500	0.011432	0.011699	0.011161	0.011364
0.011329	0.011262	0.011161	0.011161	0.011228	0.011228	0.011295
0.011228	0.011295	0.011195	0.011228	0.011228	0.011228	0.011295
0.011228	0.011597	0.011497	0.011530	0.011127	0.011228	0.011262
0.011262	0.011228	0.011295	0.011262	0.011195	0.011195	0.011195
0.011665	0.011295	0.011362	0.011161	0.011262	0.011430	0.011195
0.011195	0.011262	0.011228	0.011396	0.011195	0.011329	0.011463
0.011396	0.011228	0.011228	0.011161	0.011161	0.011329	0.011262
0.011161	0.011262	0.011127	0.011161	0.011295	0.011363	0.011195
0.011161	0.011295	0.011228	0.011127	0.011396	0.011127	0.011295

0.011368	0.011195	0.011430	0.011329	0.011295	0.011228	0.011329
0.011195	0.011530	0.011127	0.011329	0.011127	0.011127	0.011127
0.011362	0.011497	0.011262	0.011329	0.011161	0.011497	0.011127
0.011195	0.011195	0.011228	0.011329	0.011228	0.011329	0.011396
0.011396	0.011362	0.011262	0.011362	0.011329	0.011195	0.011161
0.011195	0.011195	0.011262	0.011127	0.011161	0.011329	0.011195
0.011161	0.011228	0.011362	0.011329	0.011295	0.011228	0.011228
0.011295	0.011228	0.011329	0.011195	0.011329	0.011429	0.011665
0.011228	0.011564	0.011233	0.017747	0.011257	0.011305	0.011514
0.011475	0.011517	0.011270	0.011234	0.011609	0.011543	0.011303
0.011392	0.011426	0.011138	0.011228	0.011138	0.011208	0.011232
0.011389	0.011321	0.011320	0.011126	0.011362	0.011396	0.011398
0.011266	0.011362	0.011362	0.011262	0.011295	0.011295	0.011262
0.011295	0.011228	0.011262	0.011295	0.011262	0.011195	0.011228
0.011228	0.011295	0.011195	0.011295	0.011329	0.011362	0.011262
0.011228	0.011195	0.011530	0.011530	0.011329	0.011363	0.011396
0.011161	0.011161	0.011195	0.011295	0.011161	0.011396	0.011396
0.011429	0.011429	0.011362	0.011463	0.011564	0.011464	0.011295
0.011295	0.011362	0.011396	0.011228	0.011295	0.011262	0.011295
0.011363	0.011362	0.011396	0.011362	0.011262	0.011463	0.011429
0.011297	0.011195	0.011295	0.011396	0.011295	0.011362	0.011463
0.011631	0.011333	0.011497	0.011463	0.011396	0.011564	0.011195
0.011363	0.011262	0.011228	0.011228	0.011295	0.011195	0.011295
0.011329	0.011362	0.011262	0.011228	0.011195	0.011530	0.011530
0.011329	0.011363	0.011429	0.011362	0.011935	0.011417	0.011342
0.011412	0.011372	0.011482	0.011372	0.011480	0.011405	0.011371
0.011884	0.011268	0.011403	0.011337	0.011611	0.011468	0.011571
0.011910	0.011362	0.011297	0.011295	0.011295	0.011262	0.011263
0.011161	0.011195	0.011329	0.011295	0.011297	0.011362	0.011330
0.011262	0.011195	0.011228	0.011262	0.011161	0.011228	0.011127
0.011195	0.011228	0.011195	0.011161	0.011262	0.011195	0.011228
0.011228	0.011161	0.011228	0.011262	0.011161	0.011362	0.011396
0.011295	0.011295	0.011295	0.011295	0.011228	0.011564	0.011262
0.011234	0.011228	0.011396	0.011400	0.011329	0.011329	0.011329
0.011329	0.011262	0.011228	0.011262	0.011362	0.011362	0.011295
0.011295	0.011429	0.011530	0.011329	0.011530	0.011530	0.011396
0.011228	0.011295	0.011329	0.011665	0.011295	0.011329	0.011295
0.011329	0.011195	0.011195	0.011497	0.011497	0.011463	0.011262
0.011362	0.011429	0.011295	0.011362	0.011329	0.011195	0.011463
0.011295	0.011262	0.011195	0.011429	0.011262	0.011362	0.011597
0.011295	0.011604	0.011597	0.011385	0.011117	0.011298	0.011163
0.011115	0.011605	0.011201	0.011328	0.011158	0.011315	0.011273
0.011366	0.011126	0.011338	0.011126	0.011222	0.011395	0.011344
0.011362	0.011257	0.011272	0.011272	0.011296	0.011362	0.011429
0.011429	0.011296	0.011398	0.011399	0.011535	0.011555	0.011295
0.011463	0.011329	0.011262	0.011295	0.011161	0.011329	0.011195
0.011262	0.011262	0.011228	0.011195	0.011228	0.011195	0.011295
0.011270	0.011270	0.011266	0.011300	0.011436	0.011402	0.011402
0.011401	0.011329	0.011134	0.011132	0.011263	0.011433	0.011433
0.011228	0.011297	0.011531	0.011499	0.011430	0.011464	0.011396
0.011329	0.011396	0.011396	0.011295	0.011799	0.011463	0.011530
0.011564	0.011559	0.011231	0.011337	0.011228	0.011430	0.011396
0.011430	0.011463	0.011463	0.011463	0.011610	0.011597	0.011234
0.011128	0.011130	0.011230	0.011196	0.011231	0.011135	0.011400
0.011266	0.011323	0.011156	0.011285	0.011262	0.011334	0.011228
0.011195	0.000000	0.011262	0.011228	0.011362	0.011329	0.011262
0.011362	0.011429	0.011228	0.011228	0.011262	0.011262	0.011263
0.011331	0.011262	0.011262	0.011296	0.011362	0.011429	0.011429
0.011296	0.011398	0.011399	0.011535	0.011555	0.011295	0.011463
0.011396	0.011396	0.011229	0.011408	0.011338	0.011367	0.011235

0.011404	0.011561	0.011357	0.011298	0.011195	0.011396	0.011262
0.011362	0.011295	0.011195	0.011296	0.011396	0.011398	0.011295
0.011195	0.011128	0.011262	0.011195	0.011127	0.011230	0.011396
0.011295	0.011228	0.011262	0.011295	0.011262	0.011195	0.011329
0.011262	0.011295	0.011295	0.011228	0.011295	0.011530	0.011127
0.011228	0.011564	0.011228	0.011262	0.011228	0.011530	0.011189
0.011566	0.011188	0.011161	0.011342	0.011426	0.011347	0.011144
0.011113	0.011329	0.011430	0.011535	0.011397	0.011534	0.011665
0.011565	0.011601	0.011564	0.011812	0.011530	0.012405	0.011565
0.012304	0.012072	0.012206	0.012477	0.011530	0.011228	0.011530
0.011969	0.011903	0.011497	0.011396	0.011464	0.011464	0.011432
0.011329	0.011331	0.011396	0.011363	0.011733	0.011799	0.011800
0.011699	0.011800	0.011903	0.011669	0.011969	0.011974	0.012036
0.012037	0.012070	0.012015	0.012037	0.012038	0.011329	0.011564
0.011632	0.012136	0.011636	0.011867	0.012339	0.011400	0.011295
0.011262	0.011262	0.011732	0.011759	0.011869	0.011597	0.011262
S128	0.011665	0.011665	0.012001	0.011765	0.011765	0.011833
0.011672	0.011604	0.011765	0.011262	0.011329	0.011296	0.011262
0.011295	0.011463	0.011262	0.011295	0.011362	0.011329	0.011295
0.011262	0.011295	0.011396	0.011262	0.011228	0.011295	0.011638
0.011530	0.011295	0.011195	0.011429	0.011295	0.011329	0.011295
0.011329	0.011362	0.011329	0.011329	0.011362	0.011329	0.011497
0.011262	0.011329	0.011362	0.011295	0.011396	0.011430	0.011362
0.011329	0.011497	0.011396	0.011665	0.011329	0.011530	0.011329
0.011295	0.011396	0.011463	0.011530	0.011698	0.011396	0.011497
0.011295	0.011362	0.011329	0.011497	0.011799	0.011329	0.011396
0.011295	0.011396	0.011362	0.011430	0.011362	0.011362	0.011362
0.011262	0.011295	0.011262	0.011362	0.011329	0.011363	0.011429
0.011362	0.011329	0.011262	0.011295	0.011429	0.011195	0.011329
0.011497	0.011429	0.011665	0.011497	0.011329	0.011497	0.011329
0.011505	0.011733	0.011551	0.011255	0.011247	0.011355	0.011454
0.012085	0.011467	0.011266	0.011564	0.011564	0.011362	0.011463
0.011396	0.011362	0.011429	0.011329	0.011430	0.011295	0.011362
0.011329	0.011429	0.011631	0.011497	0.011396	0.011363	0.011228
0.011262	0.011262	0.000000	0.011295	0.011497	0.011463	0.011396
0.011497	0.011564	0.011295	0.011295	0.011329	0.011329	0.011330
0.011398	0.011329	0.011329	0.011168	0.011277	0.011236	0.011329
0.011331	0.011266	0.011318	0.011302	0.011230	0.011266	0.011297
0.011564	0.011336	0.011301	0.011262	0.011268	0.011397	0.011363
0.011430	0.011364	0.011363	0.011463	0.011396	0.011698	0.011564
0.011597	0.011463	0.011329	0.011430	0.011228	0.011463	0.011262
0.011329	0.011329	0.011295	0.011262	0.011295	0.011262	0.011362
0.011262	0.011362	0.011362	0.011262	0.011363	0.011463	0.011466
0.011329	0.011362	0.011262	0.011195	0.011329	0.011262	0.011195
0.011298	0.011329	0.011195	0.011329	0.011228	0.011329	0.011295
0.011329	0.011295	0.011262	0.011295	0.011329	0.011195	0.011262
0.011295	0.011295	0.011362	0.011295	0.011329	0.011362	0.011597
0.011698	0.011631	0.011698	0.011465	0.011698	0.011329	0.011295
0.011330	0.011505	0.011380	0.011232	0.011671	0.011197	0.011336
0.011705	0.011604	0.011497	0.011698	0.011402	0.011564	0.011463
0.011463	0.011631	0.011487	0.011349	0.011396	0.011363	0.011433
0.011530	0.011431	0.011568	0.011568	0.011564	0.011631	0.011738
0.011466	0.011569	0.011568	0.011500	0.011767	0.011228	0.011431
0.011329	0.011329	0.011228	0.011228	0.011228	0.011295	0.011362
0.011228	0.011295	0.011195	0.011295	0.011228	0.011295	0.011362
0.011295	0.011732	0.011631	0.011665	0.011195	0.011295	0.011329
0.011329	0.011295	0.011362	0.011396	0.011262	0.011262	0.011262
0.011799	0.011430	0.011430	0.011228	0.011329	0.011430	0.011262
0.011262	0.011329	0.011295	0.011396	0.011262	0.011396	0.011463
0.011463	0.011295	0.011295	0.011228	0.011228	0.011396	0.011329

0.011228	0.011329	0.011195	0.011228	0.011362	0.011497	0.011262
0.011228	0.011362	0.011296	0.011195	0.011530	0.011195	0.011362
0.011368	0.011262	0.011564	0.011396	0.011362	0.011295	0.011396
0.011262	0.011530	0.011195	0.011463	0.011262	0.011262	0.011195
0.011362	0.011631	0.011329	0.011396	0.011228	0.011631	0.011262
0.011262	0.011262	0.011295	0.011329	0.011295	0.011396	0.011463
0.011463	0.011430	0.011329	0.011430	0.011329	0.011262	0.011228
0.011262	0.011262	0.011329	0.011262	0.011161	0.011396	0.011262
0.011295	0.011295	0.011430	0.011463	0.011362	0.011228	0.011295
0.011362	0.011295	0.011396	0.011262	0.011396	0.011497	0.011799
0.011295	0.011698	0.011302	0.017823	0.011257	0.011427	0.011653
0.011614	0.011658	0.011374	0.011292	0.011719	0.011682	0.011364
0.011535	0.011610	0.011216	0.011270	0.011255	0.011356	0.011318
0.011529	0.011470	0.011431	0.011259	0.011430	0.011463	0.011466
0.011266	0.011430	0.011430	0.011396	0.011362	0.011363	0.011329
0.011362	0.011295	0.011329	0.011362	0.011329	0.011262	0.011295
0.011295	0.011362	0.011262	0.011363	0.011396	0.011430	0.011329
0.011362	0.011329	0.011665	0.011665	0.011396	0.011430	0.011463
0.011228	0.011228	0.011262	0.011295	0.011228	0.011463	0.011463
0.011497	0.011497	0.011430	0.011530	0.011698	0.011532	0.011295
0.011362	0.011430	0.011396	0.011295	0.011362	0.011329	0.011362
0.011430	0.011430	0.011463	0.011363	0.011262	0.011530	0.011429
0.011365	0.011262	0.011362	0.011463	0.011363	0.011497	0.011530
0.011698	0.011402	0.011564	0.011463	0.011463	0.011631	0.011262
0.011431	0.011329	0.011295	0.011295	0.011362	0.011262	0.011363
0.011396	0.011430	0.011329	0.011362	0.011329	0.011665	0.011665
0.011396	0.011430	0.011497	0.011429	0.012070	0.011489	0.011412
0.011482	0.011442	0.011552	0.011441	0.011550	0.011474	0.011440
0.012022	0.011337	0.011471	0.011406	0.011680	0.011537	0.011571
0.012047	0.011512	0.011365	0.011362	0.011362	0.011396	0.011331
0.011228	0.011195	0.011396	0.011362	0.011364	0.011497	0.011464
0.011329	0.011195	0.011228	0.011329	0.011161	0.011295	0.011195
0.011262	0.011295	0.011262	0.011228	0.011329	0.011262	0.011295
0.011295	0.011228	0.011295	0.011329	0.011228	0.011362	0.011463
0.011362	0.011362	0.011362	0.011362	0.011295	0.011698	0.011329
0.011373	0.011295	0.011531	0.011535	0.011329	0.011396	0.011396
0.011396	0.011262	0.011295	0.011262	0.011430	0.011430	0.011362
0.011362	0.011497	0.011665	0.011396	0.011598	0.011598	0.011463
0.011295	0.011362	0.011396	0.011799	0.011363	0.011329	0.011362
0.011396	0.011329	0.011329	0.011631	0.011497	0.011598	0.011329
0.011430	0.011497	0.011362	0.011430	0.011396	0.011262	0.011597
0.011362	0.011329	0.011329	0.011497	0.011329	0.011430	0.011665
0.011362	0.011740	0.011732	0.011548	0.011229	0.011434	0.011267
0.011225	0.011761	0.011271	0.011507	0.011304	0.011467	0.011432
0.011468	0.011258	0.011339	0.011192	0.011276	0.011395	0.011385
0.011399	0.011257	0.011412	0.011307	0.011364	0.011497	0.011564
0.011564	0.011430	0.011534	0.011535	0.011670	0.011696	0.011362
0.011597	0.011463	0.011329	0.011430	0.011228	0.011463	0.011262
0.011329	0.011329	0.011295	0.011262	0.011295	0.011262	0.011362
0.011340	0.011340	0.011266	0.011368	0.011505	0.011402	0.011470
0.011469	0.011396	0.011208	0.011204	0.011331	0.011501	0.011501
0.011296	0.011365	0.011599	0.011567	0.011497	0.011531	0.011463
0.011396	0.011463	0.011463	0.011362	0.011934	0.011530	0.011598
0.011631	0.011666	0.011333	0.011407	0.011295	0.011497	0.011463
0.011498	0.011530	0.011530	0.011530	0.011650	0.011665	0.011303
0.011196	0.011199	0.011299	0.011264	0.011368	0.011210	0.011536
0.011335	0.011411	0.011156	0.011360	0.011329	0.011402	0.011228
0.011262	0.011262	0.000000	0.011295	0.011497	0.011463	0.011396
0.011497	0.011564	0.011295	0.011295	0.011329	0.011329	0.011330
0.011398	0.011329	0.011329	0.011364	0.011497	0.011564	0.011564

0.011430	0.011534	0.011535	0.011670	0.011696	0.011362	0.011530
0.011463	0.011463	0.011297	0.011478	0.011408	0.011435	0.011305
0.011405	0.011561	0.011441	0.011366	0.011195	0.011329	0.011262
0.011362	0.011362	0.011262	0.011363	0.011463	0.011466	0.011362
0.011262	0.011195	0.011329	0.011262	0.011195	0.011298	0.011329
0.011362	0.011295	0.011329	0.011362	0.011329	0.011262	0.011463
0.011329	0.011362	0.011362	0.011295	0.011362	0.011598	0.011262
0.011295	0.011698	0.011295	0.011262	0.011295	0.011665	0.011275
0.011735	0.011273	0.011311	0.011503	0.011589	0.011491	0.011227
0.011218	0.011463	0.011564	0.011671	0.011531	0.011669	0.011800
0.011699	0.011669	0.011698	0.011949	0.011665	0.012540	0.011700
0.012439	0.012207	0.012341	0.012613	0.011665	0.011363	0.011665
0.012103	0.012038	0.011631	0.011531	0.011598	0.011598	0.011568
0.011463	0.011467	0.011530	0.011497	0.011867	0.011934	0.011935
0.011834	0.011934	0.012038	0.011804	0.012104	0.012110	0.012171
0.012172	0.012205	0.012152	0.012172	0.012173	0.011396	0.011699
0.011767	0.012270	0.011772	0.012001	0.012474	0.011536	0.011430
0.011396	0.011396	0.011867	0.011883	0.012010	0.011732	0.011397
S129	0.011631	0.011631	0.011967	0.011732	0.011732	0.011799
0.011604	0.011536	0.011732	0.011228	0.011295	0.011262	0.011228
0.011262	0.011429	0.011228	0.011262	0.011329	0.011295	0.011262
0.011228	0.011262	0.011362	0.011228	0.011262	0.011262	0.011570
0.011497	0.011262	0.011161	0.011396	0.011329	0.011295	0.011329
0.011295	0.011396	0.011295	0.011295	0.011329	0.011295	0.011463
0.011228	0.011295	0.011329	0.011262	0.011362	0.011396	0.011329
0.011295	0.011463	0.011363	0.011631	0.011295	0.011564	0.011295
0.011262	0.011362	0.011430	0.011564	0.011665	0.011362	0.011463
0.011329	0.011396	0.011295	0.011530	0.011766	0.011295	0.011362
0.011262	0.011362	0.011329	0.011396	0.011329	0.011329	0.011329
0.011228	0.011262	0.011228	0.011329	0.011295	0.011329	0.011396
0.011329	0.011295	0.011362	0.011262	0.011396	0.011228	0.011295
0.011463	0.011396	0.011631	0.011463	0.011295	0.011530	0.011295
0.011394	0.011620	0.011437	0.011138	0.011172	0.011281	0.011336
0.012011	0.011365	0.011143	0.011530	0.011530	0.011329	0.011430
0.011362	0.011329	0.011396	0.011295	0.011396	0.011329	0.011329
0.011295	0.011396	0.011597	0.011463	0.011362	0.011329	0.011262
0.011228	0.011228	0.011295	0.000000	0.011463	0.011429	0.011362
0.011463	0.011530	0.011262	0.011262	0.011295	0.011295	0.011297
0.011364	0.011295	0.011295	0.011168	0.011241	0.011200	0.011296
0.011297	0.011232	0.011245	0.011267	0.011196	0.011231	0.011263
0.011528	0.011301	0.011336	0.011228	0.011233	0.011363	0.011329
0.011396	0.011330	0.011329	0.011429	0.011362	0.011698	0.011530
0.011564	0.011430	0.011295	0.011396	0.011195	0.011429	0.011228
0.011295	0.011295	0.011262	0.011228	0.011262	0.011228	0.011329
0.011295	0.011396	0.011329	0.011228	0.011330	0.011430	0.011432
0.011430	0.011329	0.011228	0.011161	0.011295	0.011228	0.011161
0.011264	0.011430	0.011228	0.011430	0.011195	0.011295	0.011262
0.011362	0.011262	0.011295	0.011262	0.011295	0.011161	0.011295
0.011329	0.011262	0.011329	0.011262	0.011295	0.011329	0.011564
0.011665	0.011597	0.011665	0.011397	0.011665	0.011295	0.011262
0.011263	0.011545	0.011335	0.011163	0.011671	0.011163	0.011257
0.011637	0.011570	0.011463	0.011665	0.011368	0.011530	0.011497
0.011430	0.011597	0.011451	0.011307	0.011354	0.011329	0.011399
0.011497	0.011398	0.011534	0.011602	0.011530	0.011598	0.011704
0.011433	0.011603	0.011534	0.011466	0.011733	0.011195	0.011397
0.011362	0.011295	0.011195	0.011195	0.011262	0.011262	0.011329
0.011262	0.011329	0.011228	0.011329	0.011262	0.011262	0.011329
0.011262	0.011698	0.011597	0.011631	0.011161	0.011262	0.011295
0.011295	0.011262	0.011329	0.011362	0.011228	0.011228	0.011228
0.011765	0.011396	0.011396	0.011195	0.011295	0.011463	0.011228

0.011228	0.011295	0.011262	0.011430	0.011228	0.011362	0.011497
0.011429	0.011262	0.011262	0.011195	0.011195	0.011362	0.011295
0.011195	0.011295	0.011161	0.011195	0.011329	0.011463	0.011228
0.011195	0.011329	0.011262	0.011161	0.011497	0.011161	0.011329
0.011403	0.011228	0.011530	0.011362	0.011396	0.011262	0.011362
0.011228	0.011564	0.011161	0.011430	0.011228	0.011228	0.011161
0.011396	0.011564	0.011295	0.011362	0.011195	0.011597	0.011228
0.011228	0.011228	0.011262	0.011362	0.011262	0.011362	0.011429
0.011430	0.011396	0.011295	0.011396	0.011362	0.011228	0.011195
0.011228	0.011228	0.011295	0.011228	0.011195	0.011295	0.011228
0.011262	0.011262	0.011396	0.011429	0.011329	0.011262	0.011262
0.011329	0.011262	0.011362	0.011228	0.011362	0.011463	0.011765
0.011262	0.011665	0.011233	0.017747	0.011126	0.011366	0.011583
0.011545	0.011588	0.011270	0.011234	0.011664	0.011612	0.011303
0.011440	0.011487	0.011099	0.011186	0.011216	0.011258	0.011189
0.011389	0.011395	0.011394	0.011259	0.011396	0.011430	0.011432
0.011301	0.011396	0.011396	0.011362	0.011329	0.011329	0.011295
0.011329	0.011262	0.011295	0.011329	0.011295	0.011228	0.011262
0.011262	0.011329	0.011228	0.011329	0.011362	0.011396	0.011295
0.011329	0.011295	0.011631	0.011631	0.011362	0.011396	0.011430
0.011195	0.011195	0.011228	0.011329	0.011195	0.011429	0.011429
0.011463	0.011463	0.011396	0.011497	0.011665	0.011498	0.011329
0.011329	0.011396	0.011429	0.011262	0.011329	0.011161	0.011329
0.011396	0.011396	0.011429	0.011396	0.011296	0.011497	0.011463
0.011331	0.011228	0.011329	0.011430	0.011329	0.011463	0.011497
0.011665	0.011368	0.011530	0.011497	0.011430	0.011597	0.011228
0.011397	0.011295	0.011262	0.011262	0.011329	0.011228	0.011329
0.011362	0.011396	0.011295	0.011329	0.011295	0.011631	0.011631
0.011362	0.011396	0.011463	0.011396	0.012036	0.011453	0.011377
0.011447	0.011407	0.011517	0.011407	0.011515	0.011440	0.011405
0.011988	0.011303	0.011437	0.011372	0.011646	0.011502	0.011605
0.012012	0.011475	0.011331	0.011329	0.011329	0.011362	0.011297
0.011195	0.011228	0.011362	0.011329	0.011331	0.011463	0.011431
0.011295	0.011228	0.011262	0.011295	0.011195	0.011262	0.011161
0.011295	0.011329	0.011228	0.011195	0.011295	0.011228	0.011262
0.011262	0.011195	0.011262	0.011295	0.011195	0.011396	0.011429
0.011329	0.011396	0.011329	0.011329	0.011262	0.011665	0.011295
0.011339	0.011262	0.011497	0.011501	0.011362	0.011362	0.011362
0.011362	0.011295	0.011262	0.011295	0.011396	0.011396	0.011329
0.011329	0.011463	0.011631	0.011362	0.011564	0.011564	0.011429
0.011262	0.011329	0.011362	0.011766	0.011329	0.011362	0.011329
0.011362	0.011295	0.011295	0.011598	0.011530	0.011564	0.011295
0.011396	0.011463	0.011329	0.011396	0.011362	0.011228	0.011564
0.011329	0.011295	0.011295	0.011463	0.011295	0.011396	0.011631
0.011329	0.011672	0.011698	0.011426	0.011173	0.011400	0.011215
0.011170	0.011644	0.011201	0.011373	0.011207	0.011315	0.011326
0.011366	0.011126	0.011283	0.000000	0.011168	0.011261	0.011303
0.011324	0.011218	0.011342	0.011272	0.011330	0.011463	0.011530
0.011530	0.011396	0.011500	0.011501	0.011636	0.011661	0.011329
0.011564	0.011430	0.011295	0.011396	0.011195	0.011429	0.011228
0.011295	0.011295	0.011262	0.011228	0.011262	0.011228	0.011329
0.011270	0.011270	0.011300	0.011334	0.011471	0.011436	0.011436
0.011435	0.011363	0.011134	0.011132	0.011297	0.011467	0.011467
0.011262	0.011331	0.011565	0.011533	0.011464	0.011498	0.011429
0.011362	0.011429	0.011429	0.011329	0.011900	0.011497	0.011564
0.011597	0.011666	0.011299	0.011372	0.011262	0.011463	0.011430
0.011464	0.011497	0.011497	0.011497	0.011650	0.011631	0.011268
0.011162	0.011164	0.011265	0.011230	0.011334	0.011172	0.011502
0.011300	0.011455	0.011203	0.011285	0.011296	0.011368	0.011262
0.011228	0.011228	0.011295	0.000000	0.011463	0.011429	0.011362

0.011463	0.011530	0.011262	0.011262	0.011295	0.011295	0.011297
0.011364	0.011295	0.011295	0.011330	0.011463	0.011530	0.011530
0.011396	0.011500	0.011501	0.011636	0.011661	0.011329	0.011497
0.011430	0.011430	0.011263	0.011443	0.011373	0.011401	0.011235
0.011439	0.011561	0.011357	0.011332	0.011228	0.011430	0.011295
0.011396	0.011329	0.011228	0.011330	0.011430	0.011432	0.011329
0.011228	0.011161	0.011295	0.011228	0.011161	0.011264	0.011430
0.011329	0.011262	0.011295	0.011329	0.011295	0.011228	0.011430
0.011295	0.011329	0.011329	0.011262	0.011329	0.011564	0.011228
0.011262	0.011665	0.011262	0.011295	0.011262	0.011631	0.011146
0.011566	0.011145	0.011161	0.011422	0.011467	0.011419	0.011102
0.011113	0.011429	0.011530	0.011637	0.011498	0.011601	0.011766
0.011666	0.011703	0.011665	0.011880	0.011631	0.012506	0.011666
0.012405	0.012173	0.012307	0.012578	0.011631	0.011329	0.011631
0.012070	0.011971	0.011597	0.011497	0.011564	0.011564	0.011534
0.011429	0.011399	0.011497	0.011464	0.011833	0.011900	0.011901
0.011800	0.011901	0.012004	0.011737	0.012070	0.012076	0.012137
0.012138	0.012172	0.012084	0.012138	0.012139	0.011362	0.011665
0.011733	0.012237	0.011738	0.011967	0.012440	0.011502	0.011396
0.011362	0.011362	0.011833	0.011883	0.011975	0.011698	0.011363
S130	0.011833	0.011833	0.011833	0.011597	0.011597	0.011665
0.011570	0.011502	0.011665	0.011430	0.011497	0.011464	0.011429
0.011396	0.011631	0.011362	0.011463	0.011530	0.011497	0.011463
0.011429	0.011463	0.011228	0.011430	0.011463	0.011463	0.011536
0.011430	0.011463	0.011362	0.011262	0.011530	0.011497	0.011530
0.011497	0.011597	0.011497	0.011497	0.011530	0.011497	0.011665
0.011362	0.011497	0.011530	0.011463	0.011497	0.011597	0.011530
0.011497	0.011665	0.011564	0.011564	0.011497	0.011765	0.011497
0.011463	0.011497	0.011631	0.011765	0.011598	0.011497	0.011597
0.011530	0.011597	0.011497	0.011732	0.011699	0.011497	0.011564
0.011463	0.011564	0.011530	0.011597	0.011530	0.011530	0.011530
0.011429	0.011463	0.011429	0.011463	0.011497	0.011531	0.011597
0.011530	0.011497	0.011564	0.011463	0.011530	0.011429	0.011497
0.011665	0.011597	0.011631	0.011665	0.011497	0.011732	0.011497
0.011691	0.011621	0.011739	0.011451	0.011433	0.011539	0.011257
0.011902	0.011365	0.011472	0.011732	0.011732	0.011463	0.011564
0.011564	0.011530	0.011597	0.011497	0.011597	0.011530	0.011530
0.011497	0.011530	0.011799	0.011665	0.011564	0.011530	0.011463
0.011429	0.011362	0.011497	0.011463	0.000000	0.011228	0.011161
0.011262	0.011329	0.011463	0.011463	0.011497	0.011497	0.011499
0.011567	0.011497	0.011497	0.011384	0.011458	0.011411	0.011497
0.011500	0.011438	0.011503	0.011474	0.011400	0.011437	0.011465
0.011456	0.011508	0.011542	0.011430	0.011440	0.011565	0.011464
0.011531	0.011532	0.011531	0.011631	0.011564	0.011934	0.011665
0.011497	0.011564	0.011497	0.011530	0.011396	0.011362	0.011430
0.011497	0.011497	0.011463	0.011429	0.011463	0.011429	0.011530
0.011497	0.011597	0.011530	0.011429	0.011532	0.011631	0.011635
0.011631	0.011530	0.011429	0.011364	0.011497	0.011429	0.011362
0.011332	0.011631	0.011430	0.011631	0.011396	0.011497	0.011463
0.011564	0.011463	0.011497	0.011463	0.011497	0.011362	0.011497
0.011530	0.011463	0.011530	0.011463	0.011497	0.011530	0.011765
0.011598	0.011530	0.011598	0.011633	0.011598	0.011497	0.011463
0.011432	0.011747	0.011609	0.011403	0.011603	0.011367	0.011532
0.011604	0.011502	0.011665	0.011866	0.011572	0.011732	0.011698
0.011631	0.011799	0.011664	0.011513	0.011563	0.011530	0.011602
0.011698	0.011600	0.011737	0.011805	0.011732	0.011799	0.011908
0.011636	0.011807	0.011738	0.011669	0.011935	0.011396	0.011600
0.011564	0.011497	0.011396	0.011396	0.011463	0.011463	0.011530
0.011463	0.011530	0.011429	0.011463	0.011463	0.011463	0.011530
0.011463	0.011631	0.011530	0.011564	0.011362	0.011463	0.011497

0.011497	0.011463	0.011530	0.011497	0.011429	0.011429	0.011429
0.011698	0.011530	0.011597	0.011396	0.011497	0.011665	0.011429
0.011429	0.011497	0.011463	0.011631	0.011429	0.011362	0.011497
0.011430	0.011463	0.011463	0.011396	0.011396	0.011564	0.011497
0.011396	0.011497	0.011362	0.011396	0.011530	0.011396	0.011430
0.011396	0.011530	0.011464	0.011362	0.011430	0.011362	0.011530
0.011608	0.011430	0.011463	0.011564	0.011530	0.011463	0.011564
0.011429	0.011765	0.011362	0.011564	0.011362	0.011362	0.011362
0.011597	0.011531	0.011497	0.011564	0.011396	0.011530	0.011362
0.011429	0.011429	0.011463	0.011564	0.011463	0.011564	0.011631
0.011631	0.011597	0.011497	0.011598	0.011564	0.011429	0.011396
0.011429	0.011429	0.011497	0.011362	0.011396	0.011564	0.011429
0.011396	0.011463	0.011597	0.011564	0.011530	0.011463	0.011463
0.011530	0.011463	0.011564	0.011429	0.011564	0.011665	0.011698
0.011463	0.011598	0.011474	0.018012	0.011322	0.011672	0.011549
0.011510	0.011553	0.011584	0.011292	0.011665	0.011578	0.011668
0.011440	0.011304	0.011411	0.011479	0.011411	0.011554	0.011490
0.011389	0.011358	0.011357	0.011193	0.011597	0.011631	0.011635
0.011507	0.011597	0.011597	0.011497	0.011530	0.011531	0.011497
0.011530	0.011463	0.011497	0.011530	0.011497	0.011429	0.011463
0.011463	0.011530	0.011429	0.011530	0.011564	0.011597	0.011497
0.011463	0.011430	0.011564	0.011564	0.011564	0.011598	0.011631
0.011396	0.011396	0.011429	0.011530	0.011396	0.011631	0.011631
0.011665	0.011665	0.011597	0.011698	0.011598	0.011700	0.011530
0.011530	0.011597	0.011631	0.011463	0.011530	0.011497	0.011530
0.011598	0.011598	0.011631	0.011598	0.011498	0.011698	0.011665
0.011534	0.011429	0.011530	0.011631	0.011530	0.011597	0.011698
0.011866	0.011572	0.011732	0.011698	0.011631	0.011799	0.011430
0.011599	0.011497	0.011463	0.011463	0.011530	0.011429	0.011530
0.011564	0.011597	0.011497	0.011463	0.011430	0.011564	0.011564
0.011564	0.011598	0.011665	0.011597	0.012171	0.011668	0.011588
0.011658	0.011615	0.011728	0.011615	0.011725	0.011647	0.011612
0.012125	0.011510	0.011643	0.011579	0.011852	0.011707	0.011809
0.012149	0.011625	0.011534	0.011530	0.011530	0.011161	0.011500
0.011396	0.011429	0.011564	0.011530	0.011533	0.011396	0.011363
0.011497	0.011429	0.011463	0.011497	0.011396	0.011463	0.011362
0.011429	0.011463	0.011429	0.011396	0.011497	0.011429	0.011463
0.011463	0.011396	0.011463	0.011497	0.011396	0.011597	0.011631
0.011530	0.011530	0.011530	0.011530	0.011463	0.011598	0.011497
0.011199	0.011463	0.011430	0.011434	0.011564	0.011564	0.011564
0.011564	0.011497	0.011463	0.011497	0.011597	0.011597	0.011530
0.011530	0.011665	0.011564	0.011564	0.011766	0.011766	0.011631
0.011463	0.011530	0.011564	0.011699	0.011531	0.011564	0.011530
0.011564	0.011429	0.011429	0.011732	0.011732	0.011698	0.011497
0.011597	0.011665	0.011530	0.011597	0.011564	0.011429	0.011497
0.011530	0.011497	0.011429	0.011665	0.011497	0.011597	0.011631
0.011530	0.011638	0.011631	0.011385	0.011117	0.011536	0.011267
0.011445	0.011644	0.011446	0.011641	0.011499	0.011366	0.011326
0.011366	0.011258	0.011394	0.011192	0.011276	0.011395	0.011628
0.011625	0.011532	0.011518	0.011518	0.011532	0.011396	0.011463
0.011396	0.011531	0.011635	0.011297	0.011772	0.011802	0.011530
0.011497	0.011564	0.011497	0.011530	0.011396	0.011362	0.011430
0.011497	0.011497	0.011463	0.011429	0.011463	0.011429	0.011530
0.011514	0.011515	0.011505	0.011540	0.011676	0.011641	0.011641
0.011639	0.011564	0.011392	0.011384	0.011500	0.011670	0.011671
0.011464	0.011534	0.011767	0.011736	0.011666	0.011700	0.011631
0.011564	0.011631	0.011631	0.011530	0.011833	0.011698	0.011766
0.011799	0.011595	0.011504	0.011580	0.011463	0.011665	0.011631
0.011666	0.011698	0.011698	0.011698	0.011846	0.011833	0.011477
0.011365	0.011372	0.011469	0.011434	0.011470	0.011396	0.011434

0.011506	0.011323	0.011394	0.011547	0.011497	0.011573	0.011463
0.011429	0.011362	0.011497	0.011463	0.000000	0.011228	0.011161
0.011262	0.011329	0.011463	0.011463	0.011497	0.011497	0.011499
0.011567	0.011497	0.011497	0.011532	0.011396	0.011463	0.011396
0.011531	0.011635	0.011297	0.011772	0.011802	0.011530	0.011698
0.011631	0.011631	0.011466	0.011652	0.011581	0.011605	0.011481
0.011646	0.011789	0.011653	0.011536	0.011430	0.011631	0.011497
0.011597	0.011530	0.011429	0.011532	0.011631	0.011635	0.011530
0.011429	0.011364	0.011497	0.011429	0.011362	0.011332	0.011631
0.011530	0.011463	0.011497	0.011530	0.011497	0.011429	0.011362
0.011497	0.011530	0.011530	0.011463	0.011530	0.011765	0.011362
0.011463	0.011598	0.011463	0.011497	0.011463	0.011564	0.011446
0.011566	0.011230	0.011211	0.011382	0.011467	0.011383	0.011269
0.011165	0.011362	0.011463	0.011569	0.011431	0.011568	0.011699
0.011599	0.011635	0.011597	0.011846	0.011564	0.012439	0.011599
0.012338	0.012106	0.012240	0.012511	0.011564	0.011463	0.011564
0.012204	0.011937	0.011530	0.011430	0.011497	0.011497	0.011466
0.011362	0.011365	0.011430	0.011396	0.011767	0.011833	0.011834
0.011733	0.011834	0.011937	0.011703	0.012205	0.012212	0.012272
0.012273	0.012306	0.012255	0.012273	0.012274	0.011564	0.011799
0.011867	0.012371	0.011874	0.011900	0.012373	0.011434	0.011329
0.011295	0.011295	0.011766	0.011800	0.011905	0.011631	0.011296
S131	0.011799	0.011799	0.011799	0.011564	0.011564	0.011631
0.011536	0.011468	0.011631	0.011396	0.011463	0.011430	0.011396
0.011362	0.011597	0.011329	0.011429	0.011497	0.011463	0.011429
0.011396	0.011430	0.011195	0.011396	0.011430	0.011429	0.011502
0.011396	0.011430	0.011329	0.011228	0.011497	0.011463	0.011497
0.011463	0.011564	0.011463	0.011463	0.011497	0.011463	0.011631
0.011329	0.011463	0.011497	0.011430	0.011463	0.011564	0.011497
0.011463	0.011631	0.011531	0.011530	0.011463	0.011732	0.011463
0.011429	0.011463	0.011597	0.011732	0.011564	0.011463	0.011564
0.011497	0.011564	0.011463	0.011698	0.011665	0.011463	0.011530
0.011430	0.011530	0.011497	0.011564	0.011497	0.011497	0.011497
0.011396	0.011430	0.011396	0.011430	0.011463	0.011498	0.011564
0.011497	0.011463	0.011530	0.011429	0.011497	0.011396	0.011463
0.011631	0.011564	0.011598	0.011631	0.011463	0.011698	0.011463
0.011653	0.011583	0.011702	0.011373	0.011396	0.011502	0.011218
0.011865	0.011213	0.011431	0.011698	0.011698	0.011430	0.011530
0.011530	0.011497	0.011564	0.011463	0.011564	0.011497	0.011497
0.011463	0.011497	0.011766	0.011631	0.011530	0.011497	0.011430
0.011396	0.011329	0.011463	0.011429	0.011228	0.000000	0.011127
0.011161	0.011228	0.011429	0.011429	0.011463	0.011463	0.011465
0.011533	0.011463	0.011463	0.011348	0.011422	0.011376	0.011464
0.011467	0.011404	0.011466	0.011440	0.011366	0.011403	0.011432
0.011420	0.011474	0.011508	0.011396	0.011406	0.011531	0.011430
0.011497	0.011498	0.011497	0.011597	0.011530	0.011900	0.011631
0.011463	0.011530	0.011463	0.011497	0.011362	0.011329	0.011396
0.011463	0.011463	0.011429	0.011396	0.011429	0.011396	0.011497
0.011463	0.011564	0.011497	0.011396	0.011498	0.011597	0.011601
0.011598	0.011497	0.011396	0.011330	0.011463	0.011396	0.011329
0.011332	0.011598	0.011396	0.011598	0.011362	0.011463	0.011429
0.011530	0.011429	0.011463	0.011430	0.011463	0.011329	0.011463
0.011497	0.011429	0.011497	0.011429	0.011463	0.011497	0.011732
0.011564	0.011497	0.011564	0.011599	0.011564	0.011463	0.011429
0.011398	0.011667	0.011563	0.011369	0.011569	0.011333	0.011454
0.011570	0.011468	0.011631	0.011833	0.011538	0.011698	0.011665
0.011597	0.011765	0.011629	0.011431	0.011522	0.011497	0.011568
0.011665	0.011566	0.011703	0.011771	0.011698	0.011766	0.011874
0.011602	0.011773	0.011704	0.011635	0.011902	0.011362	0.011566
0.011530	0.011463	0.011362	0.011362	0.011429	0.011430	0.011497

0.011430	0.011497	0.011396	0.011430	0.011430	0.011429	0.011497
0.011430	0.011597	0.011497	0.011530	0.011329	0.011429	0.011463
0.011463	0.011429	0.011497	0.011463	0.011396	0.011396	0.011396
0.011665	0.011497	0.011564	0.011362	0.011463	0.011631	0.011396
0.011396	0.011463	0.011429	0.011598	0.011396	0.011329	0.011463
0.011396	0.011430	0.011429	0.011362	0.011362	0.011530	0.011463
0.011362	0.011463	0.011329	0.011362	0.011497	0.011363	0.011396
0.011363	0.011497	0.011430	0.011329	0.011396	0.011329	0.011497
0.011574	0.011396	0.011430	0.011531	0.011497	0.011429	0.011530
0.011396	0.011732	0.011329	0.011530	0.011329	0.011329	0.011329
0.011564	0.011497	0.011463	0.011530	0.011362	0.011497	0.011329
0.011396	0.011396	0.011429	0.011530	0.011429	0.011530	0.011597
0.011597	0.011564	0.011463	0.011564	0.011530	0.011396	0.011362
0.011396	0.011396	0.011463	0.011329	0.011362	0.011530	0.011396
0.011362	0.011430	0.011564	0.011530	0.011497	0.011429	0.011430
0.011497	0.011430	0.011530	0.011396	0.011530	0.011631	0.011665
0.011429	0.011564	0.011440	0.017974	0.011322	0.011549	0.011514
0.011475	0.011518	0.011479	0.011234	0.011555	0.011544	0.011607
0.011392	0.011243	0.011372	0.011395	0.011372	0.011455	0.011404
0.011248	0.011321	0.011320	0.011259	0.011564	0.011598	0.011601
0.011473	0.011564	0.011564	0.011463	0.011497	0.011497	0.011463
0.011497	0.011429	0.011463	0.011497	0.011463	0.011396	0.011429
0.011429	0.011497	0.011396	0.011497	0.011530	0.011564	0.011463
0.011429	0.011396	0.011530	0.011530	0.011530	0.011565	0.011598
0.011362	0.011362	0.011396	0.011497	0.011362	0.011597	0.011597
0.011631	0.011631	0.011564	0.011665	0.011564	0.011666	0.011497
0.011497	0.011564	0.011597	0.011429	0.011497	0.011463	0.011497
0.011564	0.011564	0.011597	0.011564	0.011464	0.011665	0.011631
0.011501	0.011396	0.011497	0.011598	0.011497	0.011564	0.011665
0.011833	0.011538	0.011698	0.011665	0.011597	0.011765	0.011396
0.011565	0.011464	0.011429	0.011429	0.011497	0.011396	0.011497
0.011530	0.011564	0.011463	0.011429	0.011396	0.011530	0.011530
0.011530	0.011565	0.011631	0.011564	0.012137	0.011632	0.011553
0.011623	0.011580	0.011693	0.011580	0.011691	0.011612	0.011578
0.012091	0.011476	0.011608	0.011545	0.011818	0.011673	0.011775
0.012115	0.011588	0.011500	0.011497	0.011497	0.011127	0.011466
0.011362	0.011396	0.011530	0.011497	0.011500	0.011362	0.011330
0.011463	0.011396	0.011429	0.011463	0.011362	0.011430	0.011329
0.011396	0.011429	0.011396	0.011362	0.011463	0.011396	0.011429
0.011429	0.011362	0.011430	0.011463	0.011362	0.011564	0.011597
0.011497	0.011497	0.011497	0.011497	0.011429	0.011564	0.011463
0.011165	0.011430	0.011396	0.011400	0.011530	0.011530	0.011530
0.011530	0.011463	0.011429	0.011463	0.011564	0.011564	0.011497
0.011497	0.011631	0.011530	0.011530	0.011732	0.011732	0.011597
0.011429	0.011497	0.011530	0.011665	0.011497	0.011530	0.011497
0.011530	0.011396	0.011396	0.011698	0.011698	0.011665	0.011463
0.011564	0.011631	0.011497	0.011564	0.011530	0.011396	0.011463
0.011497	0.011463	0.011396	0.011631	0.011463	0.011564	0.011598
0.011497	0.011604	0.011598	0.011345	0.011173	0.011502	0.011215
0.011335	0.011605	0.011411	0.011552	0.011451	0.011417	0.011326
0.011264	0.011258	0.011394	0.011192	0.011276	0.011395	0.011587
0.011588	0.011493	0.011483	0.011483	0.011498	0.011362	0.011430
0.011362	0.011497	0.011601	0.011196	0.011738	0.011767	0.011497
0.011463	0.011530	0.011463	0.011497	0.011362	0.011329	0.011396
0.011463	0.011463	0.011429	0.011396	0.011429	0.011396	0.011497
0.011479	0.011480	0.011471	0.011505	0.011642	0.011607	0.011606
0.011605	0.011531	0.011356	0.011348	0.011467	0.011636	0.011637
0.011430	0.011500	0.011733	0.011702	0.011632	0.011666	0.011597
0.011530	0.011597	0.011597	0.011497	0.011800	0.011665	0.011732
0.011766	0.011559	0.011435	0.011545	0.011430	0.011631	0.011598

0.011632	0.011665	0.011665	0.011665	0.011807	0.011799	0.011442
0.011331	0.011337	0.011435	0.011400	0.011436	0.011359	0.011400
0.011472	0.011279	0.011346	0.011510	0.011464	0.011539	0.011430
0.011396	0.011329	0.011463	0.011429	0.011228	0.000000	0.011127
0.011161	0.011228	0.011429	0.011429	0.011463	0.011463	0.011465
0.011533	0.011463	0.011463	0.011498	0.011362	0.011430	0.011362
0.011497	0.011601	0.011196	0.011738	0.011767	0.011497	0.011665
0.011598	0.011598	0.011432	0.011617	0.011547	0.011571	0.011446
0.011611	0.011698	0.011611	0.011502	0.011396	0.011598	0.011463
0.011564	0.011497	0.011396	0.011498	0.011597	0.011601	0.011497
0.011396	0.011330	0.011463	0.011396	0.011329	0.011332	0.011598
0.011497	0.011429	0.011463	0.011497	0.011463	0.011396	0.011329
0.011463	0.011497	0.011497	0.011429	0.011497	0.011732	0.011329
0.011430	0.011564	0.011429	0.011463	0.011429	0.011530	0.011403
0.011622	0.011273	0.011161	0.011342	0.011427	0.011347	0.011227
0.011113	0.011329	0.011430	0.011536	0.011397	0.011534	0.011666
0.011565	0.011601	0.011564	0.011812	0.011530	0.012405	0.011566
0.012305	0.012072	0.012206	0.012478	0.011530	0.011430	0.011531
0.012171	0.011903	0.011497	0.011396	0.011464	0.011464	0.011432
0.011329	0.011332	0.011396	0.011363	0.011733	0.011799	0.011800
0.011700	0.011800	0.011903	0.011669	0.012171	0.012178	0.012238
0.012239	0.012273	0.012220	0.012239	0.012241	0.011530	0.011766
0.011834	0.012338	0.011840	0.011867	0.012339	0.011400	0.011296
0.011262	0.011262	0.011732	0.011759	0.011869	0.011598	0.011262
S132	0.011732	0.011732	0.011732	0.011497	0.011497	0.011564
0.011468	0.011400	0.011564	0.011329	0.011396	0.011363	0.011329
0.011295	0.011530	0.011262	0.011362	0.011429	0.011396	0.011362
0.011329	0.011362	0.011127	0.011329	0.011363	0.011362	0.011434
0.011329	0.011362	0.011262	0.011161	0.011429	0.011396	0.011430
0.011396	0.011497	0.011396	0.011396	0.011429	0.011396	0.011564
0.011262	0.011396	0.011429	0.011362	0.011396	0.011497	0.011429
0.011396	0.011564	0.011463	0.011463	0.011396	0.011665	0.011396
0.011362	0.011396	0.011530	0.011665	0.011497	0.011396	0.011497
0.011429	0.011497	0.011396	0.011631	0.011598	0.011396	0.011463
0.011362	0.011463	0.011430	0.011497	0.011429	0.011429	0.011429
0.011329	0.011362	0.011329	0.011362	0.011396	0.011430	0.011497
0.011430	0.011396	0.011463	0.011362	0.011429	0.011329	0.011396
0.011564	0.011497	0.011530	0.011564	0.011396	0.011631	0.011396
0.011579	0.011508	0.011626	0.011334	0.011321	0.011428	0.011139
0.011792	0.011213	0.011348	0.011631	0.011631	0.011362	0.011463
0.011463	0.011429	0.011497	0.011396	0.011497	0.011429	0.011429
0.011396	0.011429	0.011698	0.011564	0.011463	0.011430	0.011362
0.011329	0.011262	0.011396	0.011362	0.011161	0.011127	0.000000
0.011161	0.011228	0.011362	0.011362	0.011396	0.011396	0.011398
0.011466	0.011396	0.011396	0.011276	0.011349	0.011306	0.011396
0.011399	0.011335	0.011392	0.011371	0.011298	0.011334	0.011364
0.011348	0.011405	0.011439	0.011329	0.011337	0.011464	0.011363
0.011430	0.011431	0.011430	0.011530	0.011463	0.011833	0.011564
0.011396	0.011463	0.011396	0.011430	0.011295	0.011262	0.011329
0.011396	0.011396	0.011362	0.011329	0.011362	0.011329	0.011429
0.011396	0.011497	0.011430	0.011329	0.011431	0.011530	0.011533
0.011530	0.011430	0.011329	0.011263	0.011396	0.011329	0.011262
0.011264	0.011530	0.011329	0.011530	0.011295	0.011396	0.011362
0.011463	0.011362	0.011396	0.011362	0.011396	0.011262	0.011396
0.011430	0.011362	0.011429	0.011362	0.011396	0.011429	0.011665
0.011497	0.011430	0.011497	0.011532	0.011497	0.011396	0.011362
0.011330	0.011626	0.011472	0.011300	0.011501	0.011265	0.011414
0.011502	0.011400	0.011564	0.011766	0.011470	0.011631	0.011598
0.011530	0.011698	0.011558	0.011390	0.011438	0.011430	0.011500
0.011598	0.011499	0.011635	0.011703	0.011631	0.011698	0.011806

0.011534	0.011705	0.011636	0.011568	0.011834	0.011295	0.011498
0.011463	0.011396	0.011295	0.011295	0.011362	0.011362	0.011429
0.011362	0.011430	0.011329	0.011362	0.011362	0.011362	0.011429
0.011362	0.011530	0.011430	0.011463	0.011262	0.011362	0.011396
0.011396	0.011362	0.011430	0.011396	0.011329	0.011329	0.011329
0.011598	0.011429	0.011497	0.011295	0.011396	0.011564	0.011329
0.011329	0.011396	0.011362	0.011530	0.011329	0.011262	0.011396
0.011329	0.011362	0.011362	0.011295	0.011295	0.011463	0.011396
0.011295	0.011396	0.011262	0.011295	0.011430	0.011295	0.011329
0.011295	0.011430	0.011363	0.011262	0.011329	0.011262	0.011430
0.011505	0.011329	0.011362	0.011463	0.011430	0.011362	0.011463
0.011329	0.011665	0.011262	0.011463	0.011262	0.011262	0.011262
0.011497	0.011430	0.011396	0.011463	0.011295	0.011430	0.011262
0.011329	0.011329	0.011362	0.011463	0.011362	0.011463	0.011530
0.011530	0.011497	0.011396	0.011497	0.011463	0.011329	0.011295
0.011329	0.011329	0.011396	0.011262	0.011295	0.011463	0.011329
0.011295	0.011362	0.011497	0.011463	0.011429	0.011362	0.011362
0.011429	0.011362	0.011463	0.011329	0.011463	0.011564	0.011598
0.011362	0.011497	0.011371	0.017898	0.011257	0.011549	0.011444
0.011406	0.011447	0.011427	0.011234	0.011500	0.011475	0.011546
0.011392	0.011182	0.011294	0.011353	0.011294	0.011406	0.011361
0.011248	0.011246	0.011246	0.011126	0.011497	0.011530	0.011534
0.011404	0.011497	0.011497	0.011396	0.011430	0.011430	0.011396
0.011430	0.011362	0.011396	0.011429	0.011396	0.011329	0.011362
0.011362	0.011429	0.011329	0.011430	0.011463	0.011497	0.011396
0.011362	0.011329	0.011463	0.011463	0.011463	0.011497	0.011530
0.011295	0.011295	0.011329	0.011430	0.011295	0.011530	0.011530
0.011564	0.011564	0.011497	0.011597	0.011497	0.011599	0.011430
0.011429	0.011497	0.011530	0.011362	0.011429	0.011396	0.011430
0.011497	0.011497	0.011530	0.011497	0.011397	0.011597	0.011564
0.011433	0.011329	0.011429	0.011530	0.011430	0.011497	0.011597
0.011766	0.011470	0.011631	0.011598	0.011530	0.011698	0.011329
0.011498	0.011396	0.011362	0.011362	0.011429	0.011329	0.011430
0.011463	0.011497	0.011396	0.011362	0.011329	0.011463	0.011463
0.011463	0.011497	0.011564	0.011497	0.012070	0.011560	0.011482
0.011552	0.011511	0.011623	0.011511	0.011620	0.011543	0.011508
0.012022	0.011406	0.011540	0.011475	0.011749	0.011605	0.011707
0.012046	0.011512	0.011432	0.011429	0.011429	0.000000	0.011398
0.011295	0.011329	0.011463	0.011429	0.011432	0.011295	0.011262
0.011396	0.011329	0.011362	0.011396	0.011295	0.011362	0.011262
0.011329	0.011362	0.011329	0.011295	0.011396	0.011329	0.011362
0.011362	0.011295	0.011362	0.011396	0.011295	0.011497	0.011530
0.011430	0.011430	0.011429	0.011430	0.011362	0.011497	0.011396
0.011095	0.011362	0.011329	0.011332	0.011463	0.011463	0.011463
0.011463	0.011396	0.011362	0.011396	0.011497	0.011497	0.011429
0.011429	0.011564	0.011463	0.011463	0.011665	0.011665	0.011530
0.011362	0.011429	0.011463	0.011598	0.011430	0.011463	0.011430
0.011463	0.011329	0.011329	0.011631	0.011631	0.011598	0.011396
0.011497	0.011564	0.011429	0.011497	0.011463	0.011329	0.011396
0.011429	0.011396	0.011329	0.011564	0.011396	0.011497	0.011530
0.011429	0.011536	0.011530	0.011263	0.000000	0.011434	0.011215
0.011335	0.011527	0.011341	0.011507	0.011353	0.011315	0.011273
0.011213	0.011192	0.011339	0.011126	0.011222	0.011328	0.011506
0.011512	0.011414	0.011412	0.011412	0.011431	0.011295	0.011362
0.011295	0.011430	0.011533	0.011196	0.011670	0.011696	0.011430
0.011396	0.011463	0.011396	0.011430	0.011295	0.011262	0.011329
0.011396	0.011396	0.011362	0.011329	0.011362	0.011329	0.011429
0.011409	0.011410	0.011403	0.011437	0.011573	0.011538	0.011538
0.011537	0.011464	0.011282	0.011276	0.011399	0.011568	0.011569
0.011363	0.011433	0.011666	0.011635	0.011565	0.011599	0.011530

0.011463	0.011530	0.011530	0.011429	0.011732	0.011597	0.011665
0.011698	0.011488	0.011401	0.011476	0.011362	0.011564	0.011530
0.011565	0.011597	0.011597	0.011597	0.011728	0.011732	0.011372
0.011263	0.011268	0.011367	0.011332	0.011368	0.011284	0.011332
0.011403	0.011192	0.011251	0.011435	0.011396	0.011471	0.011362
0.011329	0.011262	0.011396	0.011362	0.011161	0.011127	0.000000
0.011161	0.011228	0.011362	0.011362	0.011396	0.011396	0.011398
0.011466	0.011396	0.011396	0.011431	0.011295	0.011362	0.011295
0.011430	0.011533	0.011196	0.011670	0.011696	0.011430	0.011597
0.011530	0.011530	0.011365	0.011547	0.011477	0.011503	0.011375
0.011542	0.011698	0.011526	0.011434	0.011329	0.011530	0.011396
0.011497	0.011430	0.011329	0.011431	0.011530	0.011533	0.011430
0.011329	0.011263	0.011396	0.011329	0.011262	0.011264	0.011530
0.011430	0.011362	0.011396	0.011429	0.011396	0.011329	0.011262
0.011396	0.011430	0.011429	0.011362	0.011429	0.011665	0.011262
0.011362	0.011497	0.011362	0.011396	0.011362	0.011463	0.011317
0.011510	0.011188	0.011161	0.011262	0.011345	0.011276	0.011144
0.011113	0.011262	0.011362	0.011468	0.011330	0.011466	0.011598
0.011498	0.011534	0.011497	0.011744	0.011463	0.012338	0.011498
0.012237	0.012004	0.012139	0.012410	0.011463	0.011362	0.011463
0.012103	0.011836	0.011430	0.011329	0.011396	0.011396	0.011365
0.011262	0.011264	0.011329	0.011296	0.011666	0.011732	0.011733
0.011632	0.011733	0.011836	0.011601	0.012104	0.012110	0.012171
0.012172	0.012205	0.012152	0.012172	0.012173	0.011463	0.011699
0.011766	0.012270	0.011772	0.011799	0.012272	0.011332	0.011228
0.011195	0.011195	0.011665	0.011677	0.011799	0.011530	0.011195
S133	0.011833	0.011833	0.011833	0.011597	0.011597	0.011665
0.011570	0.011502	0.011665	0.011430	0.011497	0.011464	0.011429
0.011396	0.011631	0.011362	0.011463	0.011530	0.011497	0.011463
0.011429	0.011463	0.011228	0.011430	0.011463	0.011463	0.011536
0.011430	0.011463	0.011362	0.011262	0.011530	0.011497	0.011530
0.011497	0.011597	0.011497	0.011497	0.011530	0.011497	0.011665
0.011362	0.011497	0.011530	0.011463	0.011497	0.011597	0.011530
0.011497	0.011665	0.011564	0.011564	0.011497	0.011765	0.011497
0.011463	0.011497	0.011631	0.011765	0.011598	0.011497	0.011597
0.011530	0.011597	0.011497	0.011732	0.011699	0.011497	0.011564
0.011463	0.011564	0.011530	0.011597	0.011530	0.011530	0.011530
0.011429	0.011463	0.011429	0.011463	0.011497	0.011531	0.011597
0.011530	0.011497	0.011564	0.011463	0.011530	0.011429	0.011497
0.011665	0.011597	0.011631	0.011665	0.011497	0.011732	0.011497
0.011691	0.011621	0.011739	0.011412	0.011433	0.011539	0.011257
0.011902	0.011314	0.011472	0.011732	0.011732	0.011463	0.011564
0.011564	0.011530	0.011597	0.011497	0.011597	0.011530	0.011530
0.011497	0.011530	0.011799	0.011665	0.011564	0.011530	0.011463
0.011429	0.011362	0.011497	0.011463	0.011262	0.011161	0.011161
0.000000	0.011262	0.011463	0.011463	0.011497	0.011497	0.011499
0.011567	0.011497	0.011497	0.011384	0.011458	0.011411	0.011497
0.011500	0.011438	0.011503	0.011474	0.011400	0.011437	0.011465
0.011456	0.011508	0.011542	0.011430	0.011440	0.011565	0.011464
0.011531	0.011532	0.011531	0.011631	0.011564	0.011934	0.011665
0.011497	0.011564	0.011497	0.011530	0.011396	0.011362	0.011430
0.011497	0.011497	0.011463	0.011429	0.011463	0.011429	0.011530
0.011497	0.011597	0.011530	0.011429	0.011532	0.011631	0.011635
0.011631	0.011530	0.011429	0.011364	0.011497	0.011429	0.011362
0.011366	0.011631	0.011430	0.011631	0.011396	0.011497	0.011463
0.011564	0.011463	0.011497	0.011463	0.011497	0.011362	0.011497
0.011530	0.011463	0.011530	0.011463	0.011497	0.011530	0.011765
0.011598	0.011530	0.011598	0.011633	0.011598	0.011497	0.011463
0.011432	0.011747	0.011609	0.011403	0.011603	0.011367	0.011532
0.011604	0.011502	0.011665	0.011866	0.011572	0.011732	0.011698

0.011631	0.011799	0.011664	0.011472	0.011563	0.011530	0.011602
0.011698	0.011600	0.011737	0.011805	0.011732	0.011799	0.011908
0.011636	0.011807	0.011738	0.011669	0.011935	0.011396	0.011600
0.011564	0.011497	0.011396	0.011396	0.011463	0.011463	0.011530
0.011463	0.011530	0.011429	0.011463	0.011463	0.011463	0.011530
0.011463	0.011631	0.011530	0.011564	0.011362	0.011463	0.011497
0.011497	0.011463	0.011530	0.011497	0.011429	0.011429	0.011429
0.011698	0.011530	0.011597	0.011396	0.011497	0.011665	0.011429
0.011429	0.011497	0.011463	0.011631	0.011429	0.011362	0.011497
0.011430	0.011463	0.011463	0.011396	0.011396	0.011564	0.011497
0.011396	0.011497	0.011362	0.011396	0.011530	0.011396	0.011430
0.011396	0.011530	0.011464	0.011362	0.011430	0.011362	0.011530
0.011608	0.011430	0.011463	0.011564	0.011530	0.011463	0.011564
0.011429	0.011765	0.011362	0.011564	0.011362	0.011362	0.011362
0.011597	0.011531	0.011497	0.011564	0.011396	0.011530	0.011362
0.011429	0.011429	0.011463	0.011564	0.011463	0.011564	0.011631
0.011631	0.011597	0.011497	0.011597	0.011564	0.011429	0.011396
0.011429	0.011429	0.011497	0.011362	0.011396	0.011564	0.011429
0.011396	0.011463	0.011597	0.011564	0.011530	0.011463	0.011463
0.011530	0.011463	0.011564	0.011429	0.011564	0.011665	0.011698
0.011463	0.011598	0.011474	0.018012	0.011387	0.011672	0.011549
0.011510	0.011553	0.011584	0.011350	0.011610	0.011578	0.011668
0.011487	0.011304	0.011411	0.011479	0.011411	0.011504	0.011447
0.011342	0.011358	0.011357	0.011193	0.011597	0.011631	0.011635
0.011507	0.011597	0.011597	0.011497	0.011530	0.011531	0.011497
0.011530	0.011463	0.011497	0.011530	0.011497	0.011429	0.011463
0.011463	0.011530	0.011429	0.011530	0.011564	0.011597	0.011497
0.011463	0.011430	0.011564	0.011564	0.011564	0.011598	0.011631
0.011396	0.011396	0.011429	0.011530	0.011396	0.011631	0.011631
0.011665	0.011665	0.011597	0.011698	0.011598	0.011700	0.011530
0.011530	0.011597	0.011631	0.011463	0.011530	0.011497	0.011530
0.011598	0.011598	0.011631	0.011598	0.011498	0.011698	0.011665
0.011534	0.011429	0.011530	0.011631	0.011530	0.011597	0.011698
0.011866	0.011572	0.011732	0.011698	0.011631	0.011799	0.011430
0.011599	0.011497	0.011463	0.011463	0.011530	0.011429	0.011530
0.011564	0.011597	0.011497	0.011463	0.011430	0.011564	0.011564
0.011564	0.011598	0.011665	0.011597	0.012171	0.011668	0.011588
0.011658	0.011615	0.011728	0.011615	0.011726	0.011647	0.011612
0.012125	0.011510	0.011643	0.011579	0.011852	0.011707	0.011809
0.012149	0.011625	0.011534	0.011530	0.011530	0.011161	0.011500
0.011396	0.011429	0.011564	0.011530	0.011533	0.011396	0.011363
0.011497	0.011429	0.011463	0.011497	0.011396	0.011463	0.011362
0.011429	0.011463	0.011429	0.011396	0.011497	0.011429	0.011463
0.011463	0.011396	0.011463	0.011497	0.011396	0.011597	0.011631
0.011530	0.011530	0.011530	0.011530	0.011463	0.011598	0.011497
0.011199	0.011463	0.011430	0.011434	0.011564	0.011564	0.011564
0.011564	0.011497	0.011463	0.011497	0.011597	0.011597	0.011530
0.011530	0.011665	0.011564	0.011564	0.011766	0.011766	0.011631
0.011463	0.011530	0.011564	0.011698	0.011531	0.011564	0.011530
0.011564	0.011429	0.011429	0.011732	0.011732	0.011698	0.011497
0.011597	0.011665	0.011530	0.011597	0.011564	0.011429	0.011497
0.011530	0.011497	0.011429	0.011665	0.011497	0.011597	0.011631
0.011530	0.011638	0.011631	0.011385	0.011229	0.011536	0.011318
0.011445	0.011644	0.011446	0.011596	0.011499	0.011467	0.011326
0.011315	0.011324	0.011450	0.011258	0.011330	0.011462	0.011628
0.011625	0.011532	0.011518	0.011518	0.011532	0.011396	0.011463
0.011396	0.011531	0.011635	0.011230	0.011772	0.011802	0.011530
0.011497	0.011564	0.011497	0.011530	0.011396	0.011362	0.011430
0.011497	0.011497	0.011463	0.011429	0.011463	0.011429	0.011530
0.011514	0.011515	0.011505	0.011540	0.011676	0.011641	0.011641

0.011639	0.011564	0.011392	0.011384	0.011500	0.011670	0.011671
0.011464	0.011534	0.011767	0.011736	0.011665	0.011700	0.011631
0.011564	0.011631	0.011631	0.011530	0.011833	0.011698	0.011766
0.011799	0.011595	0.011504	0.011580	0.011463	0.011665	0.011631
0.011666	0.011698	0.011698	0.011698	0.011846	0.011833	0.011477
0.011365	0.011372	0.011469	0.011434	0.011470	0.011396	0.011434
0.011506	0.011323	0.011394	0.011547	0.011497	0.011573	0.011463
0.011429	0.011362	0.011497	0.011463	0.011262	0.011161	0.011161
0.000000	0.011262	0.011463	0.011463	0.011497	0.011497	0.011499
0.011567	0.011497	0.011497	0.011532	0.011396	0.011463	0.011396
0.011531	0.011635	0.011230	0.011772	0.011802	0.011530	0.011698
0.011631	0.011631	0.011466	0.011652	0.011581	0.011605	0.011481
0.011646	0.011789	0.011653	0.011536	0.011430	0.011631	0.011497
0.011597	0.011530	0.011429	0.011532	0.011631	0.011635	0.011530
0.011429	0.011364	0.011497	0.011429	0.011362	0.011366	0.011631
0.011530	0.011463	0.011497	0.011530	0.011497	0.011429	0.011362
0.011497	0.011530	0.011530	0.011463	0.011530	0.011765	0.011362
0.011463	0.011598	0.011463	0.011497	0.011463	0.011564	0.011446
0.011678	0.011315	0.011261	0.011382	0.011467	0.011383	0.011269
0.011218	0.011362	0.011463	0.011569	0.011430	0.011568	0.011699
0.011599	0.011635	0.011597	0.011846	0.011564	0.012439	0.011599
0.012338	0.012106	0.012240	0.012511	0.011564	0.011463	0.011564
0.012204	0.011937	0.011530	0.011430	0.011497	0.011497	0.011466
0.011362	0.011365	0.011430	0.011396	0.011766	0.011833	0.011834
0.011733	0.011834	0.011937	0.011703	0.012205	0.012212	0.012272
0.012273	0.012306	0.012255	0.012273	0.012274	0.011564	0.011799
0.011867	0.012371	0.011874	0.011900	0.012373	0.011434	0.011329
0.011295	0.011295	0.011766	0.011801	0.011905	0.011631	0.011296
S134	0.011900	0.011900	0.011900	0.011665	0.011665	0.011732
0.011638	0.011570	0.011732	0.011497	0.011564	0.011531	0.011497
0.011463	0.011698	0.011429	0.011530	0.011597	0.011564	0.011530
0.011497	0.011530	0.011295	0.011497	0.011531	0.011530	0.011604
0.011497	0.011530	0.011429	0.011329	0.011597	0.011564	0.011597
0.011564	0.011665	0.011564	0.011564	0.011597	0.011564	0.011732
0.011429	0.011564	0.011597	0.011530	0.011564	0.011665	0.011597
0.011564	0.011732	0.011631	0.011631	0.011564	0.011833	0.011564
0.011530	0.011564	0.011698	0.011833	0.011665	0.011564	0.011665
0.011597	0.011665	0.011564	0.011799	0.011766	0.011564	0.011631
0.011530	0.011631	0.011597	0.011665	0.011597	0.011597	0.011597
0.011497	0.011530	0.011497	0.011530	0.011564	0.011599	0.011665
0.011597	0.011564	0.011631	0.011530	0.011597	0.011497	0.011564
0.011732	0.011665	0.011698	0.011732	0.011564	0.011799	0.011564
0.011765	0.011695	0.011815	0.011490	0.011508	0.011539	0.011336
0.011975	0.011314	0.011554	0.011799	0.011799	0.011530	0.011631
0.011631	0.011597	0.011665	0.011564	0.011665	0.011597	0.011597
0.011564	0.011597	0.011866	0.011732	0.011631	0.011598	0.011530
0.011497	0.011429	0.011564	0.011530	0.011329	0.011228	0.011228
0.011262	0.000000	0.011530	0.011530	0.011564	0.011564	0.011567
0.011635	0.011564	0.011564	0.011456	0.011530	0.011481	0.011564
0.011568	0.011507	0.011577	0.011543	0.011468	0.011505	0.011533
0.011528	0.011577	0.011611	0.011497	0.011510	0.011632	0.011531
0.011598	0.011599	0.011598	0.011698	0.011631	0.012001	0.011732
0.011564	0.011631	0.011564	0.011597	0.011463	0.011429	0.011497
0.011564	0.011564	0.011530	0.011497	0.011530	0.011497	0.011597
0.011564	0.011665	0.011597	0.011497	0.011532	0.011698	0.011702
0.011698	0.011598	0.011497	0.011364	0.011564	0.011497	0.011430
0.011434	0.011698	0.011497	0.011698	0.011463	0.011564	0.011530
0.011631	0.011530	0.011564	0.011530	0.011564	0.011430	0.011564
0.011597	0.011530	0.011597	0.011530	0.011564	0.011597	0.011833
0.011665	0.011597	0.011665	0.011701	0.011665	0.011564	0.011530

0.011499	0.011707	0.011563	0.011403	0.011671	0.011435	0.011572
0.011672	0.011570	0.011732	0.011934	0.011641	0.011799	0.011766
0.011698	0.011866	0.011736	0.011555	0.011647	0.011598	0.011669
0.011766	0.011668	0.011804	0.011873	0.011799	0.011867	0.011975
0.011703	0.011875	0.011805	0.011737	0.012003	0.011463	0.011667
0.011631	0.011564	0.011463	0.011463	0.011530	0.011530	0.011597
0.011530	0.011597	0.011497	0.011530	0.011530	0.011530	0.011597
0.011530	0.011698	0.011598	0.011631	0.011429	0.011530	0.011564
0.011564	0.011530	0.011597	0.011564	0.011497	0.011497	0.011497
0.011765	0.011597	0.011665	0.011463	0.011564	0.011732	0.011497
0.011497	0.011564	0.011530	0.011698	0.011497	0.011430	0.011564
0.011497	0.011530	0.011530	0.011463	0.011463	0.011631	0.011564
0.011463	0.011564	0.011430	0.011463	0.011597	0.011463	0.011497
0.011464	0.011597	0.011531	0.011430	0.011497	0.011430	0.011597
0.011677	0.011497	0.011530	0.011631	0.011597	0.011530	0.011631
0.011497	0.011833	0.011429	0.011631	0.011430	0.011430	0.011429
0.011665	0.011598	0.011564	0.011631	0.011463	0.011598	0.011430
0.011497	0.011497	0.011530	0.011631	0.011530	0.011631	0.011698
0.011698	0.011665	0.011564	0.011665	0.011631	0.011497	0.011463
0.011497	0.011497	0.011564	0.011429	0.011463	0.011631	0.011497
0.011463	0.011530	0.011665	0.011631	0.011597	0.011530	0.011530
0.011597	0.011530	0.011631	0.011497	0.011631	0.011732	0.011766
0.011530	0.011665	0.011543	0.018087	0.011518	0.011794	0.011618
0.011579	0.011623	0.011584	0.011350	0.011719	0.011647	0.011789
0.011487	0.011426	0.011489	0.011563	0.011411	0.011603	0.011533
0.011342	0.011433	0.011431	0.011193	0.011665	0.011698	0.011703
0.011576	0.011665	0.011665	0.011564	0.011597	0.011598	0.011564
0.011597	0.011530	0.011564	0.011597	0.011564	0.011497	0.011530
0.011530	0.011597	0.011497	0.011597	0.011631	0.011665	0.011564
0.011530	0.011497	0.011631	0.011631	0.011631	0.011598	0.011698
0.011463	0.011463	0.011497	0.011597	0.011463	0.011698	0.011698
0.011732	0.011732	0.011665	0.011765	0.011665	0.011768	0.011597
0.011597	0.011665	0.011698	0.011530	0.011597	0.011564	0.011597
0.011665	0.011665	0.011698	0.011665	0.011565	0.011765	0.011732
0.011602	0.011497	0.011597	0.011698	0.011597	0.011665	0.011765
0.011934	0.011641	0.011799	0.011766	0.011698	0.011866	0.011497
0.011667	0.011564	0.011530	0.011530	0.011597	0.011497	0.011597
0.011631	0.011665	0.011564	0.011530	0.011497	0.011631	0.011631
0.011631	0.011598	0.011732	0.011665	0.012238	0.011739	0.011658
0.011728	0.011685	0.011798	0.011684	0.011796	0.011716	0.011681
0.012194	0.011580	0.011711	0.011648	0.011921	0.011775	0.011877
0.012217	0.011701	0.011601	0.011597	0.011597	0.011228	0.011567
0.011463	0.011497	0.011631	0.011597	0.011601	0.011463	0.011431
0.011564	0.011497	0.011530	0.011564	0.011463	0.011530	0.011429
0.011497	0.011530	0.011497	0.011463	0.011564	0.011497	0.011530
0.011530	0.011463	0.011530	0.011564	0.011463	0.011665	0.011698
0.011597	0.011597	0.011597	0.011597	0.011530	0.011665	0.011564
0.011269	0.011530	0.011497	0.011501	0.011631	0.011631	0.011631
0.011631	0.011564	0.011530	0.011564	0.011665	0.011665	0.011597
0.011597	0.011732	0.011631	0.011631	0.011833	0.011833	0.011698
0.011530	0.011597	0.011631	0.011766	0.011598	0.011631	0.011597
0.011631	0.011497	0.011497	0.011799	0.011799	0.011765	0.011564
0.011665	0.011732	0.011597	0.011665	0.011631	0.011497	0.011564
0.011597	0.011564	0.011497	0.011732	0.011564	0.011665	0.011698
0.011597	0.011706	0.011698	0.011467	0.011342	0.011604	0.011318
0.011554	0.011722	0.011517	0.011686	0.011597	0.011569	0.011485
0.011315	0.011455	0.011561	0.011390	0.011437	0.011596	0.011709
0.011701	0.011611	0.011589	0.011589	0.011599	0.011463	0.011530
0.011463	0.011598	0.011703	0.011297	0.011840	0.011838	0.011597
0.011564	0.011631	0.011564	0.011597	0.011463	0.011429	0.011497

0.011564	0.011564	0.011530	0.011497	0.011530	0.011497	0.011597
0.011584	0.011585	0.011574	0.011608	0.011744	0.011709	0.011709
0.011708	0.011632	0.011466	0.011456	0.011568	0.011738	0.011739
0.011531	0.011602	0.011835	0.011804	0.011733	0.011767	0.011698
0.011631	0.011698	0.011698	0.011597	0.011900	0.011765	0.011833
0.011866	0.011595	0.011572	0.011649	0.011530	0.011698	0.011698
0.011733	0.011765	0.011765	0.011765	0.011886	0.011900	0.011546
0.011432	0.011441	0.011537	0.011502	0.011539	0.011471	0.011502
0.011575	0.011411	0.011394	0.011622	0.011564	0.011642	0.011530
0.011497	0.011429	0.011564	0.011530	0.011329	0.011228	0.011228
0.011262	0.000000	0.011530	0.011530	0.011564	0.011564	0.011567
0.011635	0.011564	0.011564	0.011599	0.011463	0.011530	0.011463
0.011598	0.011703	0.011297	0.011840	0.011838	0.011597	0.011765
0.011698	0.011698	0.011534	0.011721	0.011651	0.011673	0.011481
0.011714	0.011698	0.011653	0.011604	0.011497	0.011698	0.011564
0.011665	0.011597	0.011497	0.011532	0.011698	0.011702	0.011598
0.011497	0.011364	0.011564	0.011497	0.011430	0.011434	0.011698
0.011597	0.011530	0.011564	0.011597	0.011564	0.011497	0.011430
0.011564	0.011597	0.011597	0.011530	0.011597	0.011833	0.011429
0.011530	0.011665	0.011530	0.011564	0.011530	0.011631	0.011532
0.011678	0.011400	0.011261	0.011463	0.011549	0.011455	0.011353
0.011218	0.011430	0.011530	0.011637	0.011498	0.011635	0.011766
0.011666	0.011703	0.011665	0.011915	0.011631	0.012506	0.011667
0.012406	0.012173	0.012307	0.012579	0.011631	0.011530	0.011631
0.012272	0.012005	0.011598	0.011497	0.011565	0.011565	0.011534
0.011430	0.011433	0.011497	0.011464	0.011834	0.011900	0.011901
0.011801	0.011901	0.012004	0.011771	0.012272	0.012279	0.012340
0.012340	0.012374	0.012323	0.012340	0.012342	0.011631	0.011867
0.011935	0.012439	0.011942	0.011968	0.012440	0.011502	0.011396
0.011362	0.011362	0.011833	0.011883	0.011975	0.011698	0.011363
S135 0.011631	0.011631	0.011967	0.011732	0.011732	0.011799	
0.011638	0.011570	0.011732	0.011228	0.011295	0.011262	0.011228
0.011262	0.011429	0.011228	0.011262	0.011329	0.011295	0.011262
0.011228	0.011262	0.011362	0.011228	0.011262	0.011262	0.011604
0.011497	0.011262	0.011161	0.011396	0.011329	0.011295	0.011329
0.011295	0.011396	0.011295	0.011295	0.011329	0.011295	0.011463
0.011228	0.011295	0.011329	0.011262	0.011362	0.011396	0.011329
0.011295	0.011463	0.011363	0.011631	0.011295	0.011564	0.011295
0.011262	0.011362	0.011429	0.011564	0.011665	0.011362	0.011463
0.011329	0.011396	0.011295	0.011530	0.011765	0.011295	0.011362
0.011262	0.011362	0.011329	0.011396	0.011329	0.011329	0.011329
0.011228	0.011262	0.011228	0.011329	0.011295	0.011329	0.011396
0.011329	0.011295	0.011362	0.011262	0.011396	0.011228	0.011295
0.011463	0.011396	0.011631	0.011463	0.011295	0.011530	0.011295
0.011468	0.011695	0.011475	0.011177	0.011135	0.011318	0.011375
0.012011	0.011416	0.011143	0.011530	0.011530	0.011329	0.011429
0.011362	0.011329	0.011396	0.011295	0.011396	0.011329	0.011329
0.011295	0.011396	0.011597	0.011463	0.011362	0.011329	0.011262
0.011228	0.011228	0.011295	0.011262	0.011463	0.011429	0.011362
0.011463	0.011530	0.000000	0.000000	0.011094	0.011094	0.011094
0.011162	0.011295	0.011295	0.011168	0.011241	0.011200	0.011296
0.011297	0.011232	0.011245	0.011267	0.011196	0.011231	0.011263
0.011456	0.011301	0.011336	0.011228	0.011233	0.011363	0.011329
0.011396	0.011330	0.011329	0.011429	0.011362	0.011732	0.011530
0.011564	0.011429	0.011295	0.011396	0.011195	0.011429	0.011228
0.011295	0.011295	0.011262	0.011228	0.011262	0.011228	0.011329
0.011295	0.011396	0.011329	0.011228	0.011330	0.011429	0.011432
0.011430	0.011329	0.011228	0.011161	0.011295	0.011228	0.011161
0.011264	0.011430	0.011228	0.011430	0.011195	0.011295	0.011262
0.011362	0.011262	0.011295	0.011262	0.011295	0.011161	0.011295

0.011329	0.011262	0.011329	0.011262	0.011295	0.011329	0.011564
0.011665	0.011597	0.011665	0.011431	0.011665	0.011295	0.011262
0.011297	0.011545	0.011380	0.011197	0.011671	0.011163	0.011257
0.011671	0.011502	0.011463	0.011665	0.011368	0.011530	0.011497
0.011429	0.011597	0.011451	0.011307	0.011270	0.011329	0.011399
0.011497	0.011398	0.011534	0.011602	0.011530	0.011598	0.011704
0.011433	0.011603	0.011534	0.011466	0.011733	0.011195	0.011397
0.011362	0.011295	0.011195	0.011195	0.011262	0.011262	0.011329
0.011262	0.011329	0.011228	0.011329	0.011262	0.011262	0.011329
0.011262	0.011698	0.011597	0.011631	0.011161	0.011262	0.011295
0.011295	0.011262	0.011329	0.011362	0.011228	0.011228	0.011228
0.011765	0.011396	0.011396	0.011195	0.011295	0.011463	0.011228
0.011228	0.011295	0.011262	0.011430	0.011228	0.011362	0.011497
0.011429	0.011262	0.011262	0.011195	0.011195	0.011362	0.011295
0.011195	0.011295	0.011161	0.011195	0.011329	0.011463	0.011228
0.011195	0.011329	0.011262	0.011161	0.011497	0.011161	0.011329
0.011403	0.011228	0.011530	0.011363	0.011396	0.011262	0.011362
0.011228	0.011564	0.011161	0.011429	0.011228	0.011228	0.011161
0.011396	0.011531	0.011295	0.011362	0.011195	0.011597	0.011228
0.011228	0.011228	0.011262	0.011362	0.011262	0.011362	0.011429
0.011429	0.011396	0.011295	0.011396	0.011362	0.011228	0.011195
0.011228	0.011228	0.011295	0.011228	0.011195	0.011362	0.011228
0.011262	0.011262	0.011396	0.011429	0.011329	0.011262	0.011262
0.011329	0.011262	0.011362	0.011228	0.011362	0.011463	0.011765
0.011262	0.011665	0.011267	0.017747	0.011191	0.011427	0.011618
0.011579	0.011623	0.011270	0.011234	0.011719	0.011647	0.011303
0.011440	0.011549	0.011177	0.011228	0.011255	0.011307	0.011232
0.011436	0.011395	0.011357	0.011193	0.011396	0.011430	0.011432
0.011301	0.011396	0.011396	0.011362	0.011329	0.011329	0.011295
0.011329	0.011262	0.011295	0.011329	0.011295	0.011228	0.011262
0.011262	0.011329	0.011228	0.011329	0.011362	0.011396	0.011295
0.011329	0.011295	0.011631	0.011631	0.011362	0.011396	0.011430
0.011195	0.011195	0.011228	0.011329	0.011195	0.011429	0.011429
0.011463	0.011463	0.011396	0.011497	0.011665	0.011498	0.011329
0.011329	0.011396	0.011429	0.011262	0.011329	0.011295	0.011329
0.011396	0.011396	0.011429	0.011396	0.011296	0.011497	0.011463
0.011331	0.011228	0.011329	0.011430	0.011329	0.011463	0.011497
0.011665	0.011368	0.011530	0.011497	0.011429	0.011597	0.011228
0.011397	0.011295	0.011262	0.011262	0.011329	0.011228	0.011329
0.011362	0.011396	0.011295	0.011329	0.011295	0.011631	0.011631
0.011362	0.011396	0.011463	0.011396	0.012036	0.011453	0.011377
0.011447	0.011407	0.011517	0.011407	0.011515	0.011440	0.011405
0.011987	0.011303	0.011437	0.011372	0.011646	0.011502	0.011605
0.012012	0.011475	0.011128	0.011127	0.011127	0.011362	0.011094
0.011195	0.011228	0.011161	0.011127	0.011128	0.011463	0.011431
0.011295	0.011228	0.011262	0.011295	0.011195	0.011262	0.011161
0.011295	0.011329	0.011228	0.011195	0.011295	0.011228	0.011262
0.011262	0.011195	0.011262	0.011295	0.011195	0.011396	0.011429
0.011329	0.011396	0.011329	0.011329	0.011262	0.011665	0.011295
0.011339	0.011262	0.011497	0.011501	0.011362	0.011362	0.011362
0.011362	0.011295	0.011262	0.011295	0.011396	0.011396	0.011329
0.011329	0.011463	0.011631	0.011362	0.011564	0.011564	0.011429
0.011262	0.011329	0.011362	0.011765	0.011329	0.011362	0.011329
0.011362	0.011295	0.011295	0.011598	0.011530	0.011564	0.011295
0.011396	0.011463	0.011329	0.011396	0.011362	0.011228	0.011564
0.011329	0.011295	0.011295	0.011463	0.011295	0.011396	0.011631
0.011329	0.011705	0.011698	0.011467	0.011173	0.011400	0.011215
0.011225	0.011683	0.011236	0.011373	0.011207	0.011315	0.011326
0.011417	0.011126	0.011283	0.011126	0.011168	0.011328	0.011385
0.011399	0.011257	0.011377	0.011272	0.011330	0.011463	0.011530

0.011530	0.011396	0.011500	0.011501	0.011636	0.011661	0.011329
0.011564	0.011429	0.011295	0.011396	0.011195	0.011429	0.011228
0.011295	0.011295	0.011262	0.011228	0.011262	0.011228	0.011329
0.011305	0.011305	0.011300	0.011334	0.011471	0.011436	0.011436
0.011435	0.011363	0.011171	0.011168	0.011297	0.011467	0.011467
0.011262	0.011331	0.011565	0.011533	0.011464	0.011498	0.011429
0.011362	0.011429	0.011429	0.011329	0.011900	0.011497	0.011564
0.011597	0.011666	0.011299	0.011372	0.011262	0.011463	0.011430
0.011464	0.011497	0.011497	0.011497	0.011650	0.011631	0.011268
0.011162	0.011164	0.011265	0.011230	0.011334	0.011135	0.011502
0.011300	0.011367	0.011108	0.011322	0.011296	0.011368	0.011262
0.011228	0.011228	0.011295	0.011262	0.011463	0.011429	0.011362
0.011463	0.011530	0.000000	0.000000	0.011094	0.011094	0.011094
0.011162	0.011295	0.011295	0.011330	0.011463	0.011530	0.011530
0.011396	0.011500	0.011501	0.011636	0.011661	0.011329	0.011497
0.011430	0.011430	0.011263	0.011443	0.011373	0.011401	0.011270
0.011439	0.011652	0.011399	0.011332	0.011228	0.011430	0.011295
0.011396	0.011329	0.011228	0.011330	0.011429	0.011432	0.011329
0.011228	0.011161	0.011295	0.011228	0.011161	0.011264	0.011430
0.011329	0.011262	0.011295	0.011329	0.011295	0.011228	0.011430
0.011295	0.011329	0.011329	0.011262	0.011329	0.011564	0.011228
0.011262	0.011665	0.011262	0.011295	0.011262	0.011631	0.011146
0.011622	0.011188	0.011211	0.011422	0.011508	0.011455	0.011102
0.011165	0.011429	0.011530	0.011637	0.011498	0.011635	0.011766
0.011666	0.011703	0.011665	0.011914	0.011631	0.012506	0.011666
0.012405	0.012173	0.012307	0.012578	0.011631	0.011329	0.011631
0.012070	0.012004	0.011597	0.011497	0.011564	0.011564	0.011534
0.011429	0.011433	0.011497	0.011464	0.011833	0.011900	0.011901
0.011800	0.011901	0.012004	0.011770	0.012070	0.012076	0.012137
0.012138	0.012171	0.012118	0.012138	0.012139	0.011362	0.011665
0.011733	0.012236	0.011738	0.011967	0.012406	0.011502	0.011396
0.011362	0.011362	0.011833	0.011842	0.011975	0.011698	0.011363
S136	0.011631	0.011631	0.011967	0.011732	0.011732	0.011799
0.011638	0.011570	0.011732	0.011228	0.011295	0.011262	0.011228
0.011262	0.011429	0.011228	0.011262	0.011329	0.011295	0.011262
0.011228	0.011262	0.011362	0.011228	0.011262	0.011262	0.011604
0.011497	0.011262	0.011161	0.011396	0.011329	0.011295	0.011329
0.011295	0.011396	0.011295	0.011295	0.011329	0.011295	0.011463
0.011228	0.011295	0.011329	0.011262	0.011362	0.011396	0.011329
0.011295	0.011463	0.011363	0.011631	0.011295	0.011564	0.011295
0.011262	0.011362	0.011429	0.011564	0.011665	0.011362	0.011463
0.011329	0.011396	0.011295	0.011530	0.011765	0.011295	0.011362
0.011262	0.011362	0.011329	0.011396	0.011329	0.011329	0.011329
0.011228	0.011262	0.011228	0.011329	0.011295	0.011329	0.011396
0.011329	0.011295	0.011362	0.011262	0.011396	0.011228	0.011295
0.011463	0.011396	0.011631	0.011463	0.011295	0.011530	0.011295
0.011468	0.011695	0.011475	0.011177	0.011135	0.011318	0.011375
0.012011	0.011416	0.011143	0.011530	0.011530	0.011329	0.011429
0.011362	0.011329	0.011396	0.011295	0.011396	0.011329	0.011329
0.011295	0.011396	0.011597	0.011463	0.011362	0.011329	0.011262
0.011228	0.011228	0.011295	0.011262	0.011463	0.011429	0.011362
0.011463	0.011530	0.000000	0.000000	0.011094	0.011094	0.011094
0.011162	0.011295	0.011295	0.011168	0.011241	0.011200	0.011296
0.011297	0.011232	0.011245	0.011267	0.011196	0.011231	0.011263
0.011456	0.011301	0.011336	0.011228	0.011233	0.011363	0.011329
0.011396	0.011330	0.011329	0.011429	0.011362	0.011732	0.011530
0.011564	0.011429	0.011295	0.011396	0.011195	0.011429	0.011228
0.011295	0.011295	0.011262	0.011228	0.011262	0.011228	0.011329
0.011295	0.011396	0.011329	0.011228	0.011330	0.011429	0.011432
0.011430	0.011329	0.011228	0.011161	0.011295	0.011228	0.011161

0.011264	0.011430	0.011228	0.011430	0.011195	0.011295	0.011262
0.011362	0.011262	0.011295	0.011262	0.011295	0.011161	0.011295
0.011329	0.011262	0.011329	0.011262	0.011295	0.011329	0.011564
0.011665	0.011597	0.011665	0.011431	0.011665	0.011295	0.011262
0.011297	0.011545	0.011380	0.011197	0.011671	0.011163	0.011257
0.011671	0.011502	0.011463	0.011665	0.011368	0.011530	0.011497
0.011429	0.011597	0.011451	0.011307	0.011270	0.011329	0.011399
0.011497	0.011398	0.011534	0.011602	0.011530	0.011598	0.011704
0.011433	0.011603	0.011534	0.011466	0.011733	0.011195	0.011397
0.011362	0.011295	0.011195	0.011195	0.011262	0.011262	0.011329
0.011262	0.011329	0.011228	0.011329	0.011262	0.011262	0.011329
0.011262	0.011698	0.011597	0.011631	0.011161	0.011262	0.011295
0.011295	0.011262	0.011329	0.011362	0.011228	0.011228	0.011228
0.011765	0.011396	0.011396	0.011195	0.011295	0.011463	0.011228
0.011228	0.011295	0.011262	0.011430	0.011228	0.011362	0.011497
0.011429	0.011262	0.011262	0.011195	0.011195	0.011362	0.011295
0.011195	0.011295	0.011161	0.011195	0.011329	0.011463	0.011228
0.011195	0.011329	0.011262	0.011161	0.011497	0.011161	0.011329
0.011403	0.011228	0.011530	0.011363	0.011396	0.011262	0.011362
0.011228	0.011564	0.011161	0.011429	0.011228	0.011228	0.011161
0.011396	0.011531	0.011295	0.011362	0.011195	0.011597	0.011228
0.011228	0.011228	0.011262	0.011362	0.011262	0.011362	0.011429
0.011429	0.011396	0.011295	0.011396	0.011362	0.011228	0.011195
0.011228	0.011228	0.011295	0.011228	0.011195	0.011362	0.011228
0.011262	0.011262	0.011396	0.011429	0.011329	0.011262	0.011262
0.011329	0.011262	0.011362	0.011228	0.011362	0.011463	0.011765
0.011262	0.011665	0.011267	0.017747	0.011191	0.011427	0.011618
0.011579	0.011623	0.011270	0.011234	0.011719	0.011647	0.011303
0.011440	0.011549	0.011177	0.011228	0.011255	0.011307	0.011232
0.011436	0.011395	0.011357	0.011193	0.011396	0.011430	0.011432
0.011301	0.011396	0.011396	0.011362	0.011329	0.011329	0.011295
0.011329	0.011262	0.011295	0.011329	0.011295	0.011228	0.011262
0.011262	0.011329	0.011228	0.011329	0.011362	0.011396	0.011295
0.011329	0.011295	0.011631	0.011631	0.011362	0.011396	0.011430
0.011195	0.011195	0.011228	0.011329	0.011195	0.011429	0.011429
0.011463	0.011463	0.011396	0.011497	0.011665	0.011498	0.011329
0.011329	0.011396	0.011429	0.011262	0.011329	0.011295	0.011329
0.011396	0.011396	0.011429	0.011396	0.011296	0.011497	0.011463
0.011331	0.011228	0.011329	0.011430	0.011329	0.011463	0.011497
0.011665	0.011368	0.011530	0.011497	0.011429	0.011597	0.011228
0.011397	0.011295	0.011262	0.011262	0.011329	0.011228	0.011329
0.011362	0.011396	0.011295	0.011329	0.011295	0.011631	0.011631
0.011362	0.011396	0.011463	0.011396	0.012036	0.011453	0.011377
0.011447	0.011407	0.011517	0.011407	0.011515	0.011440	0.011405
0.011987	0.011303	0.011437	0.011372	0.011646	0.011502	0.011605
0.012012	0.011475	0.011128	0.011127	0.011127	0.011362	0.011094
0.011195	0.011228	0.011161	0.011127	0.011128	0.011463	0.011431
0.011295	0.011228	0.011262	0.011295	0.011195	0.011262	0.011161
0.011295	0.011329	0.011228	0.011195	0.011295	0.011228	0.011262
0.011262	0.011195	0.011262	0.011295	0.011195	0.011396	0.011429
0.011329	0.011396	0.011329	0.011329	0.011262	0.011665	0.011295
0.011339	0.011262	0.011497	0.011501	0.011362	0.011362	0.011362
0.011362	0.011295	0.011262	0.011295	0.011396	0.011396	0.011329
0.011329	0.011463	0.011631	0.011362	0.011564	0.011564	0.011429
0.011262	0.011329	0.011362	0.011765	0.011329	0.011362	0.011329
0.011362	0.011295	0.011295	0.011598	0.011530	0.011564	0.011295
0.011396	0.011463	0.011329	0.011396	0.011362	0.011228	0.011564
0.011329	0.011295	0.011295	0.011463	0.011295	0.011396	0.011631
0.011329	0.011705	0.011698	0.011467	0.011173	0.011400	0.011215
0.011225	0.011683	0.011236	0.011373	0.011207	0.011315	0.011326

0.011417	0.011126	0.011283	0.011126	0.011168	0.011328	0.011385
0.011399	0.011257	0.011377	0.011272	0.011330	0.011463	0.011530
0.011530	0.011396	0.011500	0.011501	0.011636	0.011661	0.011329
0.011564	0.011429	0.011295	0.011396	0.011195	0.011429	0.011228
0.011295	0.011295	0.011262	0.011228	0.011262	0.011228	0.011329
0.011305	0.011305	0.011300	0.011334	0.011471	0.011436	0.011436
0.011435	0.011363	0.011171	0.011168	0.011297	0.011467	0.011467
0.011262	0.011331	0.011565	0.011533	0.011464	0.011498	0.011429
0.011362	0.011429	0.011429	0.011329	0.011900	0.011497	0.011564
0.011597	0.011666	0.011299	0.011372	0.011262	0.011463	0.011430
0.011464	0.011497	0.011497	0.011497	0.011650	0.011631	0.011268
0.011162	0.011164	0.011265	0.011230	0.011334	0.011135	0.011502
0.011300	0.011367	0.011108	0.011322	0.011296	0.011368	0.011262
0.011228	0.011228	0.011295	0.011262	0.011463	0.011429	0.011362
0.011463	0.011530	0.000000	0.000000	0.011094	0.011094	0.011094
0.011162	0.011295	0.011295	0.011330	0.011463	0.011530	0.011530
0.011396	0.011500	0.011501	0.011636	0.011661	0.011329	0.011497
0.011430	0.011430	0.011263	0.011443	0.011373	0.011401	0.011270
0.011439	0.011652	0.011399	0.011332	0.011228	0.011430	0.011295
0.011396	0.011329	0.011228	0.011330	0.011429	0.011432	0.011329
0.011228	0.011161	0.011295	0.011228	0.011161	0.011264	0.011430
0.011329	0.011262	0.011295	0.011329	0.011295	0.011228	0.011430
0.011295	0.011329	0.011329	0.011262	0.011329	0.011564	0.011228
0.011262	0.011665	0.011262	0.011295	0.011262	0.011631	0.011146
0.011622	0.011188	0.011211	0.011422	0.011508	0.011455	0.011102
0.011165	0.011429	0.011530	0.011637	0.011498	0.011635	0.011766
0.011666	0.011703	0.011665	0.011914	0.011631	0.012506	0.011666
0.012405	0.012173	0.012307	0.012578	0.011631	0.011329	0.011631
0.012070	0.012004	0.011597	0.011497	0.011564	0.011564	0.011534
0.011429	0.011433	0.011497	0.011464	0.011833	0.011900	0.011901
0.011800	0.011901	0.012004	0.011770	0.012070	0.012076	0.012137
0.012138	0.012171	0.012118	0.012138	0.012139	0.011362	0.011665
0.011733	0.012236	0.011738	0.011967	0.012406	0.011502	0.011396
0.011362	0.011362	0.011833	0.011842	0.011975	0.011698	0.011363
S137 0.011665	0.011665	0.012001	0.011766	0.011766	0.011833	
0.011672	0.011604	0.011766	0.011262	0.011329	0.011296	0.011262
0.011295	0.011463	0.011262	0.011295	0.011362	0.011329	0.011295
0.011262	0.011295	0.011396	0.011262	0.011295	0.011295	0.011638
0.011530	0.011295	0.011195	0.011430	0.011362	0.011329	0.011362
0.011329	0.011430	0.011329	0.011329	0.011362	0.011329	0.011497
0.011262	0.011329	0.011362	0.011295	0.011396	0.011430	0.011362
0.011329	0.011497	0.011396	0.011665	0.011329	0.011597	0.011329
0.011295	0.011396	0.011463	0.011597	0.011698	0.011396	0.011497
0.011362	0.011430	0.011329	0.011564	0.011799	0.011329	0.011396
0.011295	0.011396	0.011362	0.011430	0.011362	0.011362	0.011362
0.011262	0.011295	0.011262	0.011362	0.011329	0.011363	0.011430
0.011362	0.011329	0.011396	0.011295	0.011430	0.011262	0.011329
0.011497	0.011430	0.011665	0.011497	0.011329	0.011564	0.011329
0.011505	0.011733	0.011513	0.011217	0.011172	0.011355	0.011415
0.012048	0.011416	0.011143	0.011564	0.011564	0.011362	0.011463
0.011396	0.011362	0.011430	0.011329	0.011430	0.011362	0.011362
0.011329	0.011430	0.011631	0.011497	0.011396	0.011363	0.011295
0.011262	0.011262	0.011329	0.011295	0.011497	0.011463	0.011396
0.011497	0.011564	0.011094	0.011094	0.000000	0.000000	0.000000
0.011128	0.011329	0.011329	0.011204	0.011277	0.011236	0.011329
0.011331	0.011266	0.011282	0.011302	0.011230	0.011266	0.011297
0.011492	0.011336	0.011370	0.011262	0.011268	0.011397	0.011363
0.011430	0.011364	0.011363	0.011463	0.011396	0.011766	0.011564
0.011597	0.011463	0.011329	0.011430	0.011228	0.011463	0.011262
0.011329	0.011329	0.011295	0.011262	0.011295	0.011262	0.011362

0.011329	0.011430	0.011362	0.011262	0.011363	0.011463	0.011466
0.011463	0.011363	0.011262	0.011195	0.011329	0.011262	0.011195
0.011298	0.011463	0.011262	0.011463	0.011228	0.011329	0.011295
0.011396	0.011295	0.011329	0.011295	0.011329	0.011195	0.011329
0.011362	0.011295	0.011362	0.011295	0.011329	0.011362	0.011598
0.011698	0.011631	0.011698	0.011465	0.011698	0.011329	0.011295
0.011330	0.011586	0.011380	0.011197	0.011705	0.011197	0.011296
0.011706	0.011536	0.011497	0.011698	0.011402	0.011564	0.011530
0.011463	0.011631	0.011487	0.011349	0.011312	0.011363	0.011433
0.011531	0.011431	0.011568	0.011636	0.011564	0.011631	0.011738
0.011466	0.011637	0.011568	0.011500	0.011767	0.011228	0.011431
0.011396	0.011329	0.011228	0.011228	0.011295	0.011295	0.011362
0.011295	0.011362	0.011262	0.011362	0.011295	0.011295	0.011362
0.011295	0.011732	0.011631	0.011665	0.011195	0.011295	0.011329
0.011329	0.011295	0.011362	0.011396	0.011262	0.011262	0.011262
0.011799	0.011430	0.011430	0.011228	0.011329	0.011497	0.011262
0.011262	0.011329	0.011295	0.011463	0.011262	0.011396	0.011530
0.011463	0.011295	0.011295	0.011228	0.011228	0.011396	0.011329
0.011228	0.011329	0.011195	0.011228	0.011362	0.011497	0.011262
0.011228	0.011362	0.011296	0.011195	0.011530	0.011195	0.011362
0.011437	0.011262	0.011564	0.011396	0.011430	0.011295	0.011396
0.011262	0.011597	0.011195	0.011463	0.011262	0.011262	0.011195
0.011430	0.011564	0.011329	0.011396	0.011228	0.011631	0.011262
0.011262	0.011262	0.011295	0.011396	0.011295	0.011396	0.011463
0.011463	0.011430	0.011329	0.011430	0.011396	0.011262	0.011228
0.011262	0.011262	0.011329	0.011262	0.011228	0.011396	0.011262
0.011295	0.011295	0.011430	0.011463	0.011362	0.011295	0.011295
0.011362	0.011295	0.011396	0.011262	0.011396	0.011497	0.011799
0.011295	0.011698	0.011302	0.017749	0.011191	0.011488	0.011653
0.011614	0.011658	0.011270	0.011234	0.011774	0.011682	0.011364
0.011440	0.011610	0.011216	0.011228	0.011255	0.011356	0.011275
0.011436	0.011433	0.011357	0.011193	0.011430	0.011463	0.011466
0.011335	0.011430	0.011430	0.011396	0.011362	0.011363	0.011329
0.011362	0.011295	0.011329	0.011362	0.011329	0.011262	0.011295
0.011295	0.011362	0.011262	0.011362	0.011396	0.011430	0.011329
0.011362	0.011329	0.011665	0.011665	0.011396	0.011430	0.011463
0.011228	0.011228	0.011262	0.011362	0.011228	0.011463	0.011463
0.011497	0.011497	0.011430	0.011530	0.011698	0.011532	0.011362
0.011362	0.011430	0.011463	0.011295	0.011362	0.011329	0.011362
0.011430	0.011430	0.011463	0.011430	0.011330	0.011530	0.011497
0.011365	0.011262	0.011362	0.011463	0.011362	0.011497	0.011530
0.011698	0.011402	0.011564	0.011530	0.011463	0.011631	0.011262
0.011431	0.011329	0.011295	0.011295	0.011362	0.011262	0.011362
0.011396	0.011430	0.011329	0.011362	0.011329	0.011665	0.011665
0.011396	0.011430	0.011497	0.011430	0.012070	0.011489	0.011412
0.011482	0.011442	0.011552	0.011441	0.011550	0.011474	0.011440
0.012022	0.011337	0.011471	0.011406	0.011680	0.011537	0.011639
0.012047	0.011512	0.011094	0.011161	0.011094	0.011396	0.000000
0.011228	0.011262	0.011195	0.011161	0.011094	0.011497	0.011464
0.011329	0.011262	0.011295	0.011329	0.011228	0.011295	0.011195
0.011329	0.011362	0.011262	0.011228	0.011329	0.011262	0.011295
0.011295	0.011228	0.011295	0.011329	0.011228	0.011430	0.011463
0.011362	0.011430	0.011362	0.011362	0.011295	0.011698	0.011329
0.011373	0.011295	0.011531	0.011535	0.011396	0.011396	0.011396
0.011396	0.011329	0.011295	0.011329	0.011430	0.011430	0.011362
0.011362	0.011497	0.011665	0.011396	0.011598	0.011598	0.011463
0.011295	0.011362	0.011396	0.011799	0.011363	0.011396	0.011362
0.011396	0.011329	0.011329	0.011631	0.011564	0.011598	0.011329
0.011430	0.011497	0.011362	0.011430	0.011396	0.011262	0.011597
0.011362	0.011329	0.011329	0.011497	0.011329	0.011430	0.011665

0.011362	0.011740	0.011732	0.011507	0.011173	0.011434	0.011215
0.011280	0.011722	0.011271	0.011373	0.011255	0.011366	0.011326
0.011417	0.011126	0.011283	0.011126	0.011168	0.011328	0.011425
0.011437	0.011296	0.011412	0.011307	0.011364	0.011497	0.011564
0.011564	0.011430	0.011534	0.011535	0.011670	0.011696	0.011362
0.011597	0.011463	0.011329	0.011430	0.011228	0.011463	0.011262
0.011329	0.011329	0.011295	0.011262	0.011295	0.011262	0.011362
0.011340	0.011340	0.011334	0.011368	0.011505	0.011470	0.011470
0.011469	0.011396	0.011208	0.011204	0.011331	0.011501	0.011501
0.011296	0.011365	0.011599	0.011567	0.011497	0.011531	0.011463
0.011396	0.011463	0.011463	0.011362	0.011934	0.011530	0.011598
0.011631	0.011666	0.011333	0.011407	0.011295	0.011497	0.011463
0.011498	0.011530	0.011530	0.011530	0.011689	0.011665	0.011303
0.011196	0.011199	0.011299	0.011264	0.011368	0.011172	0.011536
0.011335	0.011367	0.011108	0.011360	0.011329	0.011402	0.011295
0.011262	0.011262	0.011329	0.011295	0.011497	0.011463	0.011396
0.011497	0.011564	0.011094	0.011094	0.000000	0.000000	0.000000
0.011128	0.011329	0.011329	0.011364	0.011497	0.011564	0.011564
0.011430	0.011534	0.011535	0.011670	0.011696	0.011362	0.011530
0.011463	0.011463	0.011297	0.011478	0.011408	0.011435	0.011305
0.011473	0.011698	0.011399	0.011366	0.011262	0.011463	0.011329
0.011430	0.011362	0.011262	0.011363	0.011463	0.011466	0.011363
0.011262	0.011195	0.011329	0.011262	0.011195	0.011298	0.011463
0.011362	0.011295	0.011329	0.011362	0.011329	0.011262	0.011463
0.011329	0.011362	0.011362	0.011295	0.011362	0.011598	0.011262
0.011295	0.011698	0.011295	0.011329	0.011295	0.011665	0.011146
0.011622	0.011230	0.011211	0.011463	0.011549	0.011491	0.011144
0.011165	0.011463	0.011564	0.011671	0.011532	0.011669	0.011800
0.011700	0.011737	0.011698	0.011949	0.011665	0.012540	0.011700
0.012439	0.012207	0.012341	0.012612	0.011665	0.011362	0.011665
0.012103	0.012038	0.011631	0.011531	0.011598	0.011598	0.011568
0.011463	0.011467	0.011530	0.011497	0.011867	0.011934	0.011935
0.011834	0.011935	0.012038	0.011804	0.012104	0.012110	0.012171
0.012172	0.012205	0.012152	0.012172	0.012173	0.011396	0.011699
0.011699	0.012270	0.011772	0.012001	0.012441	0.011536	0.011430
0.011396	0.011396	0.011867	0.011883	0.012010	0.011732	0.011397
S138	0.011665	0.011665	0.012001	0.011765	0.011765	0.011833
0.011672	0.011604	0.011765	0.011262	0.011329	0.011296	0.011262
0.011295	0.011463	0.011262	0.011295	0.011362	0.011329	0.011295
0.011262	0.011295	0.011396	0.011262	0.011295	0.011295	0.011638
0.011530	0.011295	0.011195	0.011429	0.011362	0.011329	0.011362
0.011329	0.011429	0.011329	0.011329	0.011362	0.011329	0.011497
0.011262	0.011329	0.011362	0.011295	0.011396	0.011429	0.011362
0.011329	0.011497	0.011396	0.011665	0.011329	0.011597	0.011329
0.011295	0.011396	0.011463	0.011597	0.011698	0.011396	0.011497
0.011362	0.011429	0.011329	0.011564	0.011799	0.011329	0.011396
0.011295	0.011396	0.011362	0.011429	0.011362	0.011362	0.011362
0.011262	0.011295	0.011262	0.011362	0.011329	0.011363	0.011429
0.011362	0.011329	0.011396	0.011295	0.011429	0.011262	0.011329
0.011497	0.011429	0.011665	0.011497	0.011329	0.011564	0.011329
0.011505	0.011732	0.011513	0.011216	0.011172	0.011355	0.011414
0.012048	0.011416	0.011143	0.011564	0.011564	0.011362	0.011463
0.011396	0.011362	0.011429	0.011329	0.011429	0.011362	0.011362
0.011329	0.011429	0.011631	0.011497	0.011396	0.011362	0.011295
0.011262	0.011262	0.011329	0.011295	0.011497	0.011463	0.011396
0.011497	0.011564	0.011094	0.011094	0.000000	0.000000	0.000000
0.011128	0.011329	0.011329	0.011204	0.011277	0.011235	0.011329
0.011331	0.011266	0.011282	0.011302	0.011230	0.011266	0.011297
0.011492	0.011336	0.011370	0.011262	0.011268	0.011397	0.011363
0.011430	0.011363	0.011363	0.011463	0.011396	0.011765	0.011564

0.011597	0.011463	0.011329	0.011429	0.011228	0.011463	0.011262
0.011329	0.011329	0.011295	0.011262	0.011295	0.011262	0.011362
0.011329	0.011429	0.011362	0.011262	0.011363	0.011463	0.011466
0.011463	0.011362	0.011262	0.011195	0.011329	0.011262	0.011195
0.011298	0.011463	0.011262	0.011463	0.011228	0.011329	0.011295
0.011396	0.011295	0.011329	0.011295	0.011329	0.011195	0.011329
0.011362	0.011295	0.011362	0.011295	0.011329	0.011362	0.011597
0.011698	0.011631	0.011698	0.011465	0.011698	0.011329	0.011295
0.011330	0.011586	0.011380	0.011197	0.011705	0.011197	0.011296
0.011705	0.011536	0.011497	0.011698	0.011402	0.011564	0.011530
0.011463	0.011631	0.011487	0.011349	0.011312	0.011362	0.011432
0.011530	0.011431	0.011567	0.011636	0.011564	0.011631	0.011738
0.011466	0.011637	0.011568	0.011500	0.011767	0.011228	0.011431
0.011396	0.011329	0.011228	0.011228	0.011295	0.011295	0.011362
0.011295	0.011362	0.011262	0.011362	0.011295	0.011295	0.011362
0.011295	0.011732	0.011631	0.011665	0.011195	0.011295	0.011329
0.011329	0.011295	0.011362	0.011396	0.011262	0.011262	0.011262
0.011799	0.011429	0.011429	0.011228	0.011329	0.011497	0.011262
0.011262	0.011329	0.011295	0.011463	0.011262	0.011396	0.011530
0.011463	0.011295	0.011295	0.011228	0.011228	0.011396	0.011329
0.011228	0.011329	0.011195	0.011228	0.011362	0.011497	0.011262
0.011228	0.011362	0.011296	0.011195	0.011530	0.011195	0.011362
0.011437	0.011262	0.011564	0.011396	0.011429	0.011295	0.011396
0.011262	0.011597	0.011195	0.011463	0.011262	0.011262	0.011195
0.011429	0.011564	0.011329	0.011396	0.011228	0.011631	0.011262
0.011262	0.011262	0.011295	0.011396	0.011295	0.011396	0.011463
0.011463	0.011429	0.011329	0.011430	0.011396	0.011262	0.011228
0.011262	0.011262	0.011329	0.011262	0.011228	0.011396	0.011262
0.011295	0.011295	0.011429	0.011463	0.011362	0.011295	0.011295
0.011362	0.011295	0.011396	0.011262	0.011396	0.011497	0.011799
0.011295	0.011698	0.011302	0.017747	0.011191	0.011488	0.011653
0.011614	0.011658	0.011270	0.011234	0.011774	0.011681	0.011364
0.011440	0.011610	0.011216	0.011228	0.011255	0.011356	0.011275
0.011436	0.011433	0.011357	0.011193	0.011429	0.011463	0.011466
0.011335	0.011429	0.011429	0.011396	0.011362	0.011363	0.011329
0.011362	0.011295	0.011329	0.011362	0.011329	0.011262	0.011295
0.011295	0.011362	0.011262	0.011362	0.011396	0.011429	0.011329
0.011362	0.011329	0.011665	0.011665	0.011396	0.011430	0.011463
0.011228	0.011228	0.011262	0.011362	0.011228	0.011463	0.011463
0.011497	0.011497	0.011429	0.011530	0.011698	0.011532	0.011362
0.011362	0.011429	0.011463	0.011295	0.011362	0.011329	0.011362
0.011430	0.011430	0.011463	0.011430	0.011330	0.011530	0.011497
0.011365	0.011262	0.011362	0.011463	0.011362	0.011497	0.011530
0.011698	0.011402	0.011564	0.011530	0.011463	0.011631	0.011262
0.011431	0.011329	0.011295	0.011295	0.011362	0.011262	0.011362
0.011396	0.011429	0.011329	0.011362	0.011329	0.011665	0.011665
0.011396	0.011430	0.011497	0.011430	0.012070	0.011489	0.011412
0.011482	0.011442	0.011552	0.011441	0.011550	0.011474	0.011439
0.012022	0.011337	0.011471	0.011406	0.011680	0.011536	0.011639
0.012046	0.011512	0.011094	0.011161	0.011094	0.011396	0.000000
0.011228	0.011262	0.011195	0.011161	0.011094	0.011497	0.011464
0.011329	0.011262	0.011295	0.011329	0.011228	0.011295	0.011195
0.011329	0.011362	0.011262	0.011228	0.011329	0.011262	0.011295
0.011295	0.011228	0.011295	0.011329	0.011228	0.011429	0.011463
0.011362	0.011429	0.011362	0.011362	0.011295	0.011698	0.011329
0.011373	0.011295	0.011531	0.011535	0.011396	0.011396	0.011396
0.011396	0.011329	0.011295	0.011329	0.011429	0.011429	0.011362
0.011362	0.011497	0.011665	0.011396	0.011597	0.011597	0.011463
0.011295	0.011362	0.011396	0.011799	0.011363	0.011396	0.011362
0.011396	0.011329	0.011329	0.011631	0.011564	0.011597	0.011329

0.011429	0.011497	0.011362	0.011429	0.011396	0.011262	0.011597
0.011362	0.011329	0.011329	0.011497	0.011329	0.011429	0.011665
0.011362	0.011739	0.011732	0.011507	0.011173	0.011434	0.011215
0.011280	0.011722	0.011271	0.011373	0.011255	0.011366	0.011326
0.011417	0.011126	0.011283	0.011126	0.011168	0.011328	0.011425
0.011437	0.011296	0.011412	0.011307	0.011363	0.011497	0.011564
0.011564	0.011430	0.011533	0.011535	0.011670	0.011696	0.011362
0.011597	0.011463	0.011329	0.011429	0.011228	0.011463	0.011262
0.011329	0.011329	0.011295	0.011262	0.011295	0.011262	0.011362
0.011340	0.011340	0.011334	0.011368	0.011505	0.011470	0.011470
0.011469	0.011396	0.011208	0.011204	0.011331	0.011501	0.011501
0.011296	0.011365	0.011599	0.011567	0.011497	0.011531	0.011463
0.011396	0.011463	0.011463	0.011362	0.011934	0.011530	0.011598
0.011631	0.011666	0.011333	0.011407	0.011295	0.011497	0.011463
0.011498	0.011530	0.011530	0.011530	0.011689	0.011665	0.011303
0.011196	0.011199	0.011299	0.011264	0.011368	0.011172	0.011536
0.011335	0.011367	0.011108	0.011360	0.011329	0.011402	0.011295
0.011262	0.011262	0.011329	0.011295	0.011497	0.011463	0.011396
0.011497	0.011564	0.011094	0.011094	0.000000	0.000000	0.000000
0.011128	0.011329	0.011329	0.011363	0.011497	0.011564	0.011564
0.011430	0.011533	0.011535	0.011670	0.011696	0.011362	0.011530
0.011463	0.011463	0.011297	0.011478	0.011408	0.011435	0.011305
0.011473	0.011698	0.011399	0.011366	0.011262	0.011463	0.011329
0.011429	0.011362	0.011262	0.011363	0.011463	0.011466	0.011362
0.011262	0.011195	0.011329	0.011262	0.011195	0.011298	0.011463
0.011362	0.011295	0.011329	0.011362	0.011329	0.011262	0.011463
0.011329	0.011362	0.011362	0.011295	0.011362	0.011597	0.011262
0.011295	0.011698	0.011295	0.011329	0.011295	0.011665	0.011146
0.011622	0.011230	0.011211	0.011463	0.011549	0.011491	0.011144
0.011165	0.011463	0.011564	0.011671	0.011531	0.011669	0.011800
0.011699	0.011737	0.011698	0.011948	0.011665	0.012539	0.011700
0.012439	0.012207	0.012341	0.012612	0.011665	0.011362	0.011665
0.012103	0.012038	0.011631	0.011530	0.011598	0.011598	0.011568
0.011463	0.011467	0.011530	0.011497	0.011867	0.011934	0.011935
0.011834	0.011934	0.012038	0.011804	0.012104	0.012110	0.012171
0.012171	0.012205	0.012152	0.012172	0.012173	0.011396	0.011699
0.011699	0.012270	0.011772	0.012001	0.012440	0.011536	0.011430
0.011396	0.011396	0.011867	0.011883	0.012010	0.011732	0.011397
S139	0.011668	0.011668	0.012007	0.011770	0.011770	0.011837
0.011675	0.011607	0.011770	0.011263	0.011330	0.011297	0.011263
0.011297	0.011465	0.011263	0.011297	0.011364	0.011330	0.011297
0.011263	0.011297	0.011398	0.011263	0.011297	0.011297	0.011641
0.011533	0.011297	0.011195	0.011432	0.011364	0.011330	0.011364
0.011330	0.011432	0.011330	0.011330	0.011364	0.011330	0.011499
0.011263	0.011330	0.011364	0.011297	0.011398	0.011432	0.011364
0.011330	0.011499	0.011398	0.011668	0.011330	0.011601	0.011330
0.011297	0.011398	0.011465	0.011601	0.011702	0.011398	0.011499
0.011364	0.011432	0.011330	0.011567	0.011803	0.011330	0.011398
0.011297	0.011398	0.011364	0.011432	0.011364	0.011364	0.011364
0.011263	0.011297	0.011263	0.011364	0.011330	0.011331	0.011432
0.011364	0.011330	0.011398	0.011297	0.011432	0.011263	0.011330
0.011465	0.011432	0.011668	0.011499	0.011330	0.011567	0.011330
0.011506	0.011733	0.011513	0.011217	0.011172	0.011355	0.011415
0.012049	0.011417	0.011143	0.011567	0.011533	0.011364	0.011432
0.011398	0.011364	0.011432	0.011330	0.011432	0.011364	0.011364
0.011330	0.011432	0.011634	0.011499	0.011398	0.011364	0.011297
0.011263	0.011263	0.011330	0.011297	0.011499	0.011465	0.011398
0.011499	0.011567	0.011094	0.011094	0.000000	0.000000	0.000000
0.011128	0.011330	0.011330	0.011204	0.011277	0.011237	0.011331
0.011333	0.011267	0.011283	0.011303	0.011231	0.011267	0.011298

0.011495	0.011338	0.011372	0.011263	0.011269	0.011399	0.011365
0.011432	0.011365	0.011365	0.011465	0.011398	0.011770	0.011567
0.011601	0.011465	0.011330	0.011432	0.011229	0.011465	0.011263
0.011330	0.011330	0.011297	0.011263	0.011297	0.011263	0.011364
0.011330	0.011432	0.011364	0.011263	0.011365	0.011465	0.011468
0.011466	0.011364	0.011263	0.011196	0.011330	0.011263	0.011195
0.011299	0.011466	0.011263	0.011466	0.011229	0.011330	0.011297
0.011398	0.011297	0.011330	0.011297	0.011330	0.011195	0.011330
0.011364	0.011297	0.011364	0.011297	0.011330	0.011364	0.011601
0.011702	0.011634	0.011702	0.011467	0.011702	0.011330	0.011297
0.011332	0.011589	0.011383	0.011198	0.011709	0.011197	0.011298
0.011709	0.011539	0.011499	0.011702	0.011404	0.011567	0.011533
0.011465	0.011634	0.011489	0.011351	0.011314	0.011364	0.011435
0.011533	0.011433	0.011570	0.011639	0.011567	0.011635	0.011742
0.011469	0.011641	0.011571	0.011503	0.011771	0.011229	0.011433
0.011398	0.011330	0.011229	0.011229	0.011297	0.011297	0.011364
0.011297	0.011364	0.011263	0.011364	0.011297	0.011297	0.011364
0.011297	0.011736	0.011634	0.011668	0.011195	0.011297	0.011330
0.011330	0.011297	0.011364	0.011398	0.011263	0.011263	0.011263
0.011803	0.011432	0.011432	0.011229	0.011330	0.011499	0.011263
0.011263	0.011330	0.011297	0.011465	0.011263	0.011398	0.011533
0.011465	0.011297	0.011297	0.011229	0.011229	0.011330	0.011330
0.011229	0.011330	0.011195	0.011229	0.011364	0.011500	0.011263
0.011229	0.011364	0.011297	0.011195	0.011533	0.011195	0.011364
0.011439	0.011263	0.011567	0.011398	0.011432	0.011297	0.011398
0.011263	0.011601	0.011195	0.011465	0.011263	0.011263	0.011195
0.011398	0.011567	0.011330	0.011398	0.011229	0.011634	0.011263
0.011263	0.011263	0.011297	0.011398	0.011297	0.011398	0.011465
0.011465	0.011432	0.011330	0.011432	0.011398	0.011263	0.011229
0.011263	0.011263	0.011330	0.011263	0.011229	0.011398	0.011263
0.011297	0.011297	0.011432	0.011465	0.011364	0.011297	0.011297
0.011364	0.011297	0.011398	0.011263	0.011364	0.011499	0.011803
0.011297	0.011702	0.011302	0.017755	0.011191	0.011488	0.011654
0.011616	0.011659	0.011270	0.011235	0.011774	0.011685	0.011364
0.011440	0.011610	0.011216	0.011228	0.011256	0.011356	0.011276
0.011436	0.011433	0.011357	0.011193	0.011432	0.011466	0.011468
0.011337	0.011432	0.011432	0.011398	0.011364	0.011364	0.011331
0.011364	0.011297	0.011330	0.011364	0.011330	0.011263	0.011297
0.011297	0.011364	0.011263	0.011364	0.011398	0.011432	0.011330
0.011364	0.011330	0.011668	0.011668	0.011398	0.011432	0.011465
0.011229	0.011229	0.011263	0.011364	0.011229	0.011465	0.011465
0.011499	0.011499	0.011432	0.011533	0.011702	0.011534	0.011364
0.011364	0.011432	0.011465	0.011297	0.011364	0.011330	0.011364
0.011432	0.011432	0.011465	0.011432	0.011331	0.011533	0.011499
0.011367	0.011263	0.011364	0.011465	0.011364	0.011499	0.011533
0.011702	0.011404	0.011567	0.011533	0.011465	0.011634	0.011263
0.011433	0.011331	0.011297	0.011297	0.011364	0.011263	0.011364
0.011398	0.011432	0.011330	0.011364	0.011330	0.011668	0.011668
0.011398	0.011432	0.011499	0.011432	0.012076	0.011492	0.011379
0.011483	0.011444	0.011555	0.011409	0.011553	0.011477	0.011442
0.012028	0.011339	0.011474	0.011408	0.011684	0.011539	0.011642
0.012052	0.011515	0.011094	0.011162	0.011094	0.011398	0.000000
0.011229	0.011263	0.011195	0.011162	0.011094	0.011499	0.011467
0.011330	0.011263	0.011297	0.011330	0.011229	0.011297	0.011195
0.011330	0.011364	0.011263	0.011229	0.011330	0.011263	0.011297
0.011297	0.011229	0.011297	0.011330	0.011229	0.011432	0.011465
0.011364	0.011432	0.011364	0.011364	0.011297	0.011702	0.011330
0.011375	0.011297	0.011533	0.011538	0.011398	0.011398	0.011398
0.011398	0.011330	0.011297	0.011330	0.011432	0.011432	0.011364
0.011364	0.011499	0.011668	0.011398	0.011601	0.011601	0.011465

0.011297	0.011364	0.011398	0.011803	0.011364	0.011398	0.011364
0.011398	0.011330	0.011330	0.011635	0.011533	0.011601	0.011330
0.011432	0.011499	0.011364	0.011432	0.011398	0.011263	0.011601
0.011364	0.011330	0.011330	0.011465	0.011330	0.011432	0.011668
0.011364	0.011744	0.011736	0.011508	0.011173	0.011436	0.011215
0.011280	0.011723	0.011271	0.011373	0.011256	0.011366	0.011326
0.011418	0.011126	0.011283	0.011126	0.011168	0.011329	0.011426
0.011437	0.011296	0.011413	0.011307	0.011365	0.011499	0.011567
0.011567	0.011432	0.011536	0.011537	0.011674	0.011700	0.011364
0.011601	0.011465	0.011330	0.011432	0.011229	0.011465	0.011263
0.011330	0.011330	0.011297	0.011263	0.011297	0.011263	0.011364
0.011341	0.011342	0.011336	0.011370	0.011508	0.011473	0.011472
0.011471	0.011398	0.011209	0.011205	0.011333	0.011503	0.011504
0.011297	0.011367	0.011602	0.011536	0.011500	0.011534	0.011465
0.011398	0.011465	0.011465	0.011330	0.011939	0.011533	0.011601
0.011634	0.011669	0.011335	0.011409	0.011297	0.011499	0.011466
0.011500	0.011533	0.011533	0.011533	0.011654	0.011668	0.011305
0.011196	0.011200	0.011300	0.011265	0.011370	0.011173	0.011539
0.011336	0.011368	0.011108	0.011362	0.011331	0.011404	0.011297
0.011263	0.011263	0.011330	0.011297	0.011499	0.011465	0.011398
0.011499	0.011567	0.011094	0.011094	0.000000	0.000000	0.000000
0.011128	0.011330	0.011330	0.011365	0.011499	0.011567	0.011567
0.011432	0.011536	0.011537	0.011674	0.011700	0.011364	0.011533
0.011466	0.011466	0.011298	0.011480	0.011410	0.011437	0.011307
0.011476	0.011703	0.011401	0.011368	0.011263	0.011466	0.011330
0.011432	0.011364	0.011263	0.011365	0.011465	0.011468	0.011364
0.011263	0.011196	0.011330	0.011263	0.011195	0.011299	0.011466
0.011364	0.011297	0.011330	0.011364	0.011330	0.011263	0.011466
0.011330	0.011364	0.011364	0.011297	0.011364	0.011601	0.011263
0.011297	0.011702	0.011297	0.011330	0.011297	0.011668	0.011146
0.011623	0.011230	0.011211	0.011463	0.011549	0.011491	0.011144
0.011165	0.011465	0.011567	0.011675	0.011534	0.011673	0.011804
0.011703	0.011741	0.011702	0.011954	0.011668	0.012548	0.011704
0.012413	0.012214	0.012348	0.012621	0.011668	0.011364	0.011668
0.012076	0.012044	0.011634	0.011533	0.011601	0.011601	0.011571
0.011465	0.011470	0.011533	0.011500	0.011872	0.011939	0.011940
0.011839	0.011906	0.012044	0.011809	0.012110	0.012116	0.012178
0.012178	0.012212	0.012159	0.012178	0.012179	0.011398	0.011702
0.011703	0.012277	0.011776	0.012007	0.012448	0.011538	0.011432
0.011398	0.011398	0.011871	0.011889	0.012011	0.011736	0.011399
S140	0.011737	0.011737	0.012075	0.011838	0.011838	0.011906
0.011744	0.011676	0.011838	0.011331	0.011398	0.011365	0.011331
0.011364	0.011533	0.011331	0.011364	0.011432	0.011398	0.011364
0.011331	0.011364	0.011466	0.011331	0.011365	0.011364	0.011710
0.011601	0.011364	0.011263	0.011500	0.011432	0.011398	0.011432
0.011398	0.011500	0.011398	0.011398	0.011432	0.011398	0.011567
0.011331	0.011398	0.011432	0.011364	0.011466	0.011500	0.011432
0.011398	0.011567	0.011466	0.011736	0.011398	0.011669	0.011398
0.011364	0.011466	0.011533	0.011669	0.011770	0.011466	0.011567
0.011432	0.011500	0.011398	0.011635	0.011872	0.011398	0.011466
0.011364	0.011466	0.011432	0.011500	0.011432	0.011432	0.011432
0.011331	0.011364	0.011331	0.011432	0.011398	0.011399	0.011500
0.011432	0.011398	0.011466	0.011364	0.011500	0.011331	0.011398
0.011533	0.011500	0.011736	0.011567	0.011398	0.011635	0.011398
0.011580	0.011809	0.011589	0.011256	0.011247	0.011429	0.011494
0.012123	0.011417	0.011184	0.011635	0.011601	0.011432	0.011500
0.011466	0.011432	0.011500	0.011398	0.011500	0.011432	0.011432
0.011398	0.011500	0.011703	0.011567	0.011466	0.011432	0.011364
0.011331	0.011331	0.011398	0.011364	0.011567	0.011533	0.011466
0.011567	0.011635	0.011162	0.011162	0.011128	0.011128	0.011128

0.000000	0.011398	0.011398	0.011276	0.011350	0.011307	0.011399
0.011401	0.011336	0.011357	0.011373	0.011300	0.011336	0.011366
0.011568	0.011407	0.011442	0.011331	0.011339	0.011467	0.011433
0.011500	0.011433	0.011433	0.011533	0.011466	0.011838	0.011635
0.011669	0.011533	0.011398	0.011500	0.011297	0.011533	0.011331
0.011398	0.011398	0.011364	0.011331	0.011364	0.011331	0.011432
0.011398	0.011500	0.011432	0.011331	0.011433	0.011533	0.011537
0.011534	0.011432	0.011331	0.011264	0.011398	0.011331	0.011263
0.011368	0.011534	0.011331	0.011534	0.011297	0.011398	0.011364
0.011466	0.011364	0.011398	0.011364	0.011398	0.011263	0.011398
0.011398	0.011364	0.011432	0.011364	0.011398	0.011432	0.011669
0.011770	0.011703	0.011770	0.011501	0.011770	0.011398	0.011364
0.011400	0.011672	0.011476	0.011267	0.011778	0.011266	0.011377
0.011778	0.011608	0.011567	0.011770	0.011473	0.011635	0.011601
0.011533	0.011703	0.011561	0.011393	0.011356	0.011432	0.011503
0.011601	0.011502	0.011639	0.011708	0.011635	0.011703	0.011811
0.011537	0.011710	0.011640	0.011537	0.011806	0.011297	0.011501
0.011466	0.011398	0.011297	0.011297	0.011364	0.011364	0.011432
0.011364	0.011432	0.011331	0.011432	0.011364	0.011364	0.011432
0.011364	0.011804	0.011703	0.011736	0.011263	0.011364	0.011398
0.011398	0.011364	0.011432	0.011466	0.011331	0.011331	0.011331
0.011872	0.011500	0.011500	0.011297	0.011398	0.011567	0.011331
0.011331	0.011398	0.011364	0.011534	0.011331	0.011466	0.011601
0.011533	0.011364	0.011364	0.011297	0.011297	0.011398	0.011398
0.011297	0.011398	0.011263	0.011297	0.011432	0.011568	0.011331
0.011297	0.011432	0.011365	0.011263	0.011601	0.011263	0.011432
0.011508	0.011331	0.011635	0.011466	0.011500	0.011364	0.011466
0.011331	0.011669	0.011263	0.011533	0.011331	0.011331	0.011263
0.011466	0.011635	0.011398	0.011466	0.011297	0.011703	0.011331
0.011331	0.011331	0.011364	0.011466	0.011364	0.011466	0.011533
0.011533	0.011500	0.011398	0.011500	0.011466	0.011331	0.011297
0.011331	0.011331	0.011398	0.011331	0.011297	0.011466	0.011331
0.011364	0.011364	0.011500	0.011533	0.011432	0.011364	0.011365
0.011432	0.011364	0.011466	0.011331	0.011432	0.011567	0.011872
0.011364	0.011770	0.011371	0.017798	0.011192	0.011549	0.011724
0.011686	0.011730	0.011323	0.011293	0.011829	0.011755	0.011425
0.011440	0.011671	0.011295	0.011270	0.011256	0.011406	0.011319
0.011436	0.011508	0.011394	0.011259	0.011500	0.011534	0.011537
0.011406	0.011500	0.011500	0.011466	0.011432	0.011432	0.011398
0.011432	0.011364	0.011398	0.011398	0.011398	0.011331	0.011364
0.011364	0.011432	0.011331	0.011432	0.011466	0.011500	0.011398
0.011432	0.011398	0.011736	0.011736	0.011466	0.011500	0.011534
0.011297	0.011297	0.011331	0.011432	0.011297	0.011533	0.011533
0.011567	0.011567	0.011500	0.011601	0.011770	0.011603	0.011432
0.011432	0.011500	0.011533	0.011364	0.011432	0.011398	0.011432
0.011500	0.011500	0.011533	0.011500	0.011399	0.011601	0.011567
0.011435	0.011331	0.011432	0.011534	0.011432	0.011567	0.011601
0.011770	0.011473	0.011635	0.011601	0.011533	0.011703	0.011331
0.011501	0.011399	0.011364	0.011364	0.011432	0.011331	0.011432
0.011466	0.011500	0.011398	0.011432	0.011398	0.011736	0.011736
0.011466	0.011500	0.011567	0.011500	0.012144	0.011564	0.011450
0.011519	0.011514	0.011627	0.011479	0.011624	0.011547	0.011512
0.012098	0.011409	0.011543	0.011478	0.011754	0.011608	0.011711
0.012122	0.011592	0.011162	0.011229	0.011162	0.011466	0.011128
0.011297	0.011331	0.011263	0.011229	0.011162	0.011567	0.011535
0.011398	0.011331	0.011364	0.011398	0.011297	0.011364	0.011263
0.011398	0.011432	0.011331	0.011297	0.011398	0.011331	0.011364
0.011364	0.011297	0.011364	0.011398	0.011297	0.011500	0.011533
0.011432	0.011500	0.011432	0.011432	0.011297	0.011770	0.011398
0.011446	0.011365	0.011602	0.011607	0.011466	0.011466	0.011466

0.011466	0.011398	0.011364	0.011398	0.011500	0.011500	0.011432
0.011432	0.011567	0.011703	0.011466	0.011669	0.011669	0.011533
0.011364	0.011432	0.011466	0.011872	0.011432	0.011466	0.011432
0.011466	0.011398	0.011398	0.011703	0.011601	0.011669	0.011398
0.011500	0.011533	0.011432	0.011500	0.011466	0.011331	0.011669
0.011432	0.011398	0.011398	0.011533	0.011398	0.011500	0.011736
0.011432	0.011813	0.011804	0.011590	0.011173	0.011505	0.011215
0.011335	0.011801	0.011342	0.011418	0.011305	0.011417	0.011326
0.011469	0.011126	0.011283	0.011127	0.011168	0.011329	0.011507
0.011513	0.011376	0.011484	0.011378	0.011433	0.011567	0.011635
0.011635	0.011500	0.011605	0.011606	0.011743	0.011772	0.011432
0.011669	0.011533	0.011398	0.011500	0.011297	0.011533	0.011331
0.011398	0.011398	0.011364	0.011331	0.011364	0.011331	0.011432
0.011412	0.011413	0.011405	0.011439	0.011577	0.011542	0.011541
0.011540	0.011466	0.011283	0.011278	0.011401	0.011572	0.011573
0.011365	0.011435	0.011670	0.011605	0.011568	0.011568	0.011533
0.011466	0.011533	0.011533	0.011398	0.012007	0.011601	0.011669
0.011703	0.011742	0.011404	0.011479	0.011364	0.011567	0.011534
0.011568	0.011601	0.011601	0.011601	0.011694	0.011736	0.011375
0.011265	0.011269	0.011369	0.011334	0.011439	0.011211	0.011608
0.011406	0.011412	0.011156	0.011438	0.011399	0.011439	0.011364
0.011331	0.011331	0.011398	0.011364	0.011567	0.011533	0.011466
0.011567	0.011635	0.011162	0.011162	0.011128	0.011128	0.011128
0.000000	0.011398	0.011398	0.011433	0.011567	0.011635	0.011635
0.011500	0.011605	0.011606	0.011743	0.011772	0.011432	0.011601
0.011534	0.011534	0.011367	0.011551	0.011480	0.011506	0.011378
0.011511	0.011750	0.011487	0.011436	0.011331	0.011534	0.011398
0.011500	0.011432	0.011331	0.011433	0.011533	0.011537	0.011432
0.011331	0.011264	0.011398	0.011331	0.011263	0.011368	0.011534
0.011432	0.011364	0.011398	0.011398	0.011398	0.011331	0.011534
0.011398	0.011432	0.011432	0.011364	0.011432	0.011669	0.011331
0.011364	0.011770	0.011364	0.011398	0.011364	0.011736	0.011189
0.011680	0.011316	0.011211	0.011544	0.011631	0.011564	0.011186
0.011166	0.011533	0.011635	0.011744	0.011602	0.011741	0.011873
0.011772	0.011810	0.011770	0.012024	0.011736	0.012617	0.011772
0.012483	0.012283	0.012418	0.012691	0.011736	0.011432	0.011737
0.012144	0.012113	0.011703	0.011601	0.011670	0.011670	0.011639
0.011533	0.011538	0.011601	0.011568	0.011940	0.012007	0.012008
0.011907	0.011974	0.012113	0.011878	0.012179	0.012185	0.012246
0.012247	0.012281	0.012228	0.012247	0.012249	0.011466	0.011771
0.011771	0.012346	0.011845	0.012075	0.012518	0.011607	0.011500
0.011466	0.011466	0.011940	0.011973	0.012082	0.011804	0.011467
S141 0.011665	0.011665	0.012001	0.011765	0.011765	0.011833	
0.011638	0.011570	0.011765	0.011262	0.011329	0.011296	0.011262
0.011295	0.011463	0.011262	0.011295	0.011362	0.011329	0.011295
0.011262	0.011295	0.011396	0.011262	0.011295	0.011295	0.011604
0.011530	0.011295	0.011195	0.011429	0.011362	0.011329	0.011362
0.011329	0.011430	0.011329	0.011329	0.011362	0.011329	0.011497
0.011262	0.011329	0.011362	0.011295	0.011396	0.011429	0.011362
0.011329	0.011497	0.011396	0.011665	0.011329	0.011597	0.011329
0.011295	0.011396	0.011463	0.011597	0.011698	0.011396	0.011497
0.011362	0.011430	0.011329	0.011564	0.011799	0.011329	0.011396
0.011295	0.011396	0.011362	0.011430	0.011362	0.011362	0.011362
0.011262	0.011295	0.011262	0.011362	0.011329	0.011363	0.011429
0.011362	0.011329	0.011396	0.011295	0.011429	0.011262	0.011329
0.011497	0.011429	0.011665	0.011497	0.011262	0.011564	0.011329
0.011505	0.011733	0.011551	0.011255	0.011247	0.011355	0.011415
0.012085	0.011518	0.011266	0.011564	0.011564	0.011362	0.011463
0.011396	0.011362	0.011429	0.011329	0.011430	0.011362	0.011362
0.011329	0.011429	0.011631	0.011497	0.011396	0.011363	0.011295

0.011262	0.011262	0.011329	0.011295	0.011497	0.011463	0.011396
0.011497	0.011564	0.011295	0.011295	0.011329	0.011329	0.011330
0.011398	0.000000	0.011329	0.011204	0.011277	0.011236	0.011329
0.011331	0.011266	0.011318	0.011302	0.011230	0.011266	0.011297
0.011564	0.011336	0.011370	0.011262	0.011268	0.011397	0.011363
0.011430	0.011363	0.011363	0.011463	0.011396	0.011766	0.011564
0.011597	0.011463	0.011329	0.011430	0.011228	0.011463	0.011262
0.011329	0.011329	0.011295	0.011262	0.011295	0.011262	0.011362
0.011329	0.011429	0.011362	0.011262	0.011363	0.011463	0.011466
0.011463	0.011362	0.011262	0.011195	0.011329	0.011262	0.011195
0.011298	0.011463	0.011262	0.011463	0.011228	0.011329	0.011295
0.011329	0.011295	0.011262	0.011295	0.011329	0.011195	0.011329
0.011362	0.011295	0.011362	0.011295	0.011329	0.011362	0.011597
0.011698	0.011631	0.011698	0.011465	0.011698	0.011329	0.011295
0.011330	0.011586	0.011289	0.011232	0.011705	0.011163	0.011296
0.011671	0.011604	0.011497	0.011698	0.011368	0.011564	0.011530
0.011463	0.011631	0.011487	0.011349	0.011396	0.011363	0.011432
0.011530	0.011431	0.011567	0.011636	0.011564	0.011631	0.011670
0.011466	0.011637	0.011568	0.011500	0.011767	0.011228	0.011431
0.011329	0.011329	0.011228	0.011228	0.011295	0.011295	0.011362
0.011295	0.011362	0.011262	0.011362	0.011295	0.011295	0.011362
0.011295	0.011732	0.011631	0.011665	0.011195	0.011295	0.011329
0.011329	0.011295	0.011362	0.011396	0.011262	0.011262	0.011262
0.011799	0.011430	0.011430	0.011228	0.011329	0.011497	0.011262
0.011262	0.011329	0.011295	0.011463	0.011262	0.011396	0.011530
0.011463	0.011295	0.011295	0.011228	0.011228	0.011396	0.011329
0.011228	0.011329	0.011195	0.011228	0.011362	0.011497	0.011262
0.011228	0.011362	0.011296	0.011195	0.011530	0.011195	0.011362
0.011437	0.011262	0.011564	0.011396	0.011430	0.011295	0.011396
0.011262	0.011597	0.011195	0.011463	0.011262	0.011262	0.011195
0.011429	0.011631	0.011329	0.011396	0.011228	0.011631	0.011262
0.011262	0.011262	0.011295	0.011396	0.011295	0.011396	0.011463
0.011463	0.011430	0.011329	0.011430	0.011396	0.011262	0.011228
0.011262	0.011262	0.011329	0.011262	0.011228	0.011396	0.011262
0.011295	0.011295	0.011430	0.011463	0.011362	0.011295	0.011295
0.011362	0.011295	0.011396	0.011262	0.011396	0.011497	0.011799
0.011295	0.011698	0.011302	0.017823	0.011322	0.011488	0.011653
0.011614	0.011658	0.011427	0.011350	0.011774	0.011682	0.011425
0.011535	0.011610	0.011177	0.011311	0.011294	0.011356	0.011318
0.011529	0.011470	0.011468	0.011259	0.011430	0.011463	0.011466
0.011335	0.011429	0.011429	0.011396	0.011362	0.011363	0.011329
0.011362	0.011295	0.011329	0.011362	0.011329	0.011262	0.011295
0.011295	0.011362	0.011262	0.011362	0.011396	0.011429	0.011329
0.011362	0.011329	0.011665	0.011665	0.011396	0.011430	0.011463
0.011228	0.011228	0.011262	0.011362	0.011228	0.011463	0.011463
0.011497	0.011497	0.011430	0.011530	0.011698	0.011532	0.011362
0.011362	0.011429	0.011463	0.011295	0.011362	0.011329	0.011362
0.011430	0.011430	0.011463	0.011430	0.011330	0.011463	0.011497
0.011365	0.011262	0.011362	0.011463	0.011362	0.011497	0.011530
0.011698	0.011368	0.011564	0.011530	0.011463	0.011631	0.011262
0.011431	0.011329	0.011295	0.011295	0.011362	0.011262	0.011362
0.011396	0.011429	0.011329	0.011362	0.011329	0.011665	0.011665
0.011396	0.011430	0.011497	0.011429	0.012070	0.011489	0.011412
0.011482	0.011442	0.011552	0.011441	0.011550	0.011474	0.011440
0.012022	0.011337	0.011471	0.011406	0.011680	0.011536	0.011639
0.012046	0.011512	0.011365	0.011362	0.011362	0.011396	0.011331
0.011228	0.011262	0.011396	0.011362	0.011364	0.011497	0.011464
0.011329	0.011262	0.011295	0.011329	0.011228	0.011295	0.011195
0.011329	0.011362	0.011262	0.011228	0.011329	0.011262	0.011295
0.011295	0.011228	0.011295	0.011329	0.011228	0.011430	0.011463

0.011362	0.011430	0.011362	0.011362	0.011295	0.011698	0.011329
0.011373	0.011295	0.011531	0.011535	0.011396	0.011396	0.011396
0.011396	0.011329	0.011295	0.011329	0.011430	0.011430	0.011362
0.011362	0.011497	0.011665	0.011396	0.011598	0.011598	0.011463
0.011295	0.011362	0.011396	0.011799	0.011363	0.011396	0.011362
0.011396	0.011329	0.011329	0.011631	0.011564	0.011598	0.011329
0.011430	0.011429	0.011362	0.011430	0.011396	0.011262	0.011597
0.011362	0.011329	0.011329	0.011497	0.011329	0.011429	0.011665
0.011362	0.011706	0.011732	0.011548	0.011286	0.011434	0.011267
0.011335	0.011761	0.011271	0.011507	0.011304	0.011417	0.011326
0.011519	0.011258	0.011394	0.011258	0.011276	0.011462	0.011385
0.011399	0.011296	0.011412	0.011342	0.011364	0.011497	0.011564
0.011564	0.011430	0.011533	0.011535	0.011670	0.011661	0.011362
0.011597	0.011463	0.011329	0.011430	0.011228	0.011463	0.011262
0.011329	0.011329	0.011295	0.011262	0.011295	0.011262	0.011362
0.011340	0.011340	0.011334	0.011368	0.011505	0.011470	0.011470
0.011469	0.011396	0.011208	0.011204	0.011331	0.011433	0.011501
0.011296	0.011365	0.011599	0.011567	0.011497	0.011531	0.011463
0.011396	0.011463	0.011463	0.011362	0.011934	0.011530	0.011598
0.011631	0.011701	0.011333	0.011407	0.011295	0.011497	0.011463
0.011498	0.011530	0.011530	0.011530	0.011650	0.011665	0.011303
0.011196	0.011199	0.011299	0.011264	0.011368	0.011210	0.011536
0.011335	0.011499	0.011203	0.011360	0.011329	0.011402	0.011295
0.011262	0.011262	0.011329	0.011295	0.011497	0.011463	0.011396
0.011497	0.011564	0.011295	0.011295	0.011329	0.011329	0.011330
0.011398	0.000000	0.011329	0.011364	0.011497	0.011564	0.011564
0.011430	0.011533	0.011535	0.011670	0.011661	0.011362	0.011530
0.011463	0.011463	0.011297	0.011478	0.011408	0.011435	0.011305
0.011473	0.011652	0.011441	0.011366	0.011262	0.011463	0.011329
0.011429	0.011362	0.011262	0.011363	0.011463	0.011466	0.011362
0.011262	0.011195	0.011329	0.011262	0.011195	0.011298	0.011463
0.011362	0.011295	0.011329	0.011362	0.011329	0.011262	0.011463
0.011329	0.011362	0.011362	0.011295	0.011362	0.011598	0.011262
0.011295	0.011698	0.011295	0.011329	0.011295	0.011665	0.011274
0.011791	0.011273	0.011311	0.011503	0.011589	0.011491	0.011227
0.011270	0.011463	0.011564	0.011671	0.011531	0.011669	0.011800
0.011699	0.011737	0.011698	0.011880	0.011665	0.012472	0.011700
0.012439	0.012207	0.012307	0.012612	0.011665	0.011362	0.011665
0.012103	0.012038	0.011631	0.011463	0.011598	0.011598	0.011500
0.011463	0.011467	0.011530	0.011464	0.011867	0.011934	0.011935
0.011834	0.011934	0.012038	0.011804	0.012104	0.012110	0.012171
0.012172	0.012205	0.012152	0.012172	0.012173	0.011396	0.011699
0.011766	0.012270	0.011772	0.012001	0.012474	0.011536	0.011430
0.011396	0.011396	0.011867	0.011924	0.012010	0.011732	0.011397
S142	0.011665	0.011665	0.012001	0.011765	0.011765	0.011833
0.011672	0.011604	0.011765	0.011262	0.011329	0.011296	0.011262
0.011295	0.011463	0.011262	0.011295	0.011362	0.011329	0.011295
0.011262	0.011295	0.011396	0.011262	0.011296	0.011295	0.011638
0.011530	0.011295	0.011195	0.011429	0.011362	0.011329	0.011362
0.011329	0.011430	0.011329	0.011329	0.011362	0.011329	0.011497
0.011262	0.011329	0.011362	0.011295	0.011396	0.011430	0.011362
0.011329	0.011497	0.011396	0.011665	0.011329	0.011597	0.011329
0.011295	0.011396	0.011463	0.011597	0.011698	0.011396	0.011497
0.011362	0.011430	0.011329	0.011564	0.011799	0.011329	0.011396
0.011295	0.011396	0.011362	0.011430	0.011362	0.011362	0.011362
0.011262	0.011295	0.011262	0.011362	0.011329	0.011363	0.011429
0.011362	0.011329	0.011396	0.011295	0.011429	0.011262	0.011329
0.011497	0.011429	0.011665	0.011497	0.011329	0.011564	0.011329
0.011505	0.011733	0.011551	0.011255	0.011247	0.011355	0.011454
0.012085	0.011518	0.011266	0.011564	0.011564	0.011362	0.011463

0.011396	0.011362	0.011429	0.011329	0.011430	0.011362	0.011362
0.011329	0.011429	0.011631	0.011497	0.011396	0.011363	0.011295
0.011262	0.011262	0.011329	0.011295	0.011497	0.011463	0.011396
0.011497	0.011564	0.011295	0.011295	0.011329	0.011329	0.011330
0.011398	0.011329	0.000000	0.011204	0.011277	0.011236	0.011329
0.011331	0.011266	0.011318	0.011302	0.011230	0.011266	0.011297
0.011564	0.011336	0.011370	0.011262	0.011268	0.011397	0.011363
0.011430	0.011363	0.011363	0.011463	0.011396	0.011766	0.011564
0.011597	0.011463	0.011329	0.011430	0.011228	0.011463	0.011262
0.011329	0.011329	0.011295	0.011262	0.011295	0.011262	0.011094
0.011329	0.011430	0.011362	0.011262	0.011363	0.011463	0.011466
0.011463	0.011362	0.011262	0.011195	0.011329	0.011262	0.011195
0.011298	0.011463	0.011262	0.011463	0.011228	0.011329	0.011295
0.011396	0.011295	0.011329	0.011295	0.011329	0.011195	0.011329
0.011362	0.011295	0.011362	0.011295	0.011329	0.011362	0.011597
0.011698	0.011631	0.011698	0.011465	0.011698	0.011329	0.011295
0.011330	0.011586	0.011426	0.011232	0.011705	0.011163	0.011257
0.011705	0.011570	0.011497	0.011698	0.011402	0.011564	0.011530
0.011463	0.011631	0.011487	0.011349	0.011396	0.011363	0.011433
0.011530	0.011431	0.011568	0.011636	0.011564	0.011631	0.011738
0.011466	0.011637	0.011568	0.011500	0.011767	0.011228	0.011431
0.011396	0.011329	0.011228	0.011228	0.011295	0.011295	0.011362
0.011295	0.011362	0.011262	0.011362	0.011295	0.011295	0.011362
0.011295	0.011732	0.011631	0.011665	0.011195	0.011295	0.011329
0.011329	0.011295	0.011362	0.011396	0.011262	0.011262	0.011262
0.011799	0.011430	0.011430	0.011228	0.011329	0.011497	0.011262
0.011262	0.011329	0.011295	0.011463	0.011262	0.011396	0.011530
0.011463	0.011295	0.011295	0.011228	0.011228	0.011396	0.011329
0.011228	0.011329	0.011195	0.011228	0.011362	0.011497	0.011262
0.011161	0.011362	0.011296	0.011195	0.011530	0.011195	0.011295
0.011437	0.011262	0.011564	0.011396	0.011430	0.011295	0.011396
0.011262	0.011597	0.011195	0.011463	0.011262	0.011262	0.011195
0.011430	0.011631	0.011329	0.011396	0.011228	0.011631	0.011262
0.011262	0.011262	0.011295	0.011396	0.011295	0.011396	0.011463
0.011463	0.011430	0.011329	0.011430	0.011396	0.011262	0.011228
0.011262	0.011262	0.011329	0.011262	0.011228	0.011396	0.011262
0.011295	0.011295	0.011430	0.011463	0.011362	0.011295	0.011295
0.011362	0.011295	0.011396	0.011262	0.011396	0.011497	0.011799
0.011295	0.011698	0.011302	0.017823	0.011322	0.011549	0.011653
0.011614	0.011658	0.011322	0.011292	0.011774	0.011682	0.011425
0.011582	0.011610	0.011216	0.011311	0.011294	0.011307	0.011318
0.011529	0.011470	0.011468	0.011325	0.011430	0.011463	0.011466
0.011335	0.011430	0.011430	0.011396	0.011362	0.011363	0.011329
0.011362	0.011295	0.011329	0.011295	0.011329	0.011262	0.011295
0.011295	0.011362	0.011262	0.011362	0.011396	0.011430	0.011329
0.011362	0.011329	0.011665	0.011665	0.011396	0.011430	0.011463
0.011228	0.011228	0.011262	0.011362	0.011228	0.011463	0.011463
0.011497	0.011497	0.011430	0.011530	0.011698	0.011532	0.011362
0.011362	0.011430	0.011463	0.011295	0.011362	0.011329	0.011362
0.011430	0.011430	0.011463	0.011430	0.011330	0.011530	0.011497
0.011365	0.011262	0.011362	0.011463	0.011362	0.011497	0.011530
0.011698	0.011402	0.011564	0.011530	0.011463	0.011631	0.011262
0.011431	0.011329	0.011295	0.011295	0.011362	0.011262	0.011362
0.011396	0.011430	0.011329	0.011362	0.011329	0.011665	0.011665
0.011396	0.011430	0.011497	0.011430	0.012070	0.011489	0.011412
0.011482	0.011442	0.011552	0.011441	0.011550	0.011474	0.011440
0.012022	0.011337	0.011471	0.011406	0.011680	0.011537	0.011639
0.012047	0.011512	0.011365	0.011362	0.011362	0.011396	0.011331
0.011228	0.011262	0.011396	0.011362	0.011364	0.011497	0.011464
0.011329	0.011262	0.011295	0.011329	0.011228	0.011295	0.011195

0.011329	0.011362	0.011262	0.011228	0.011329	0.011262	0.011295
0.011295	0.011228	0.011295	0.011329	0.011228	0.011430	0.011463
0.011362	0.011430	0.011362	0.011362	0.011295	0.011698	0.011329
0.011373	0.011295	0.011531	0.011535	0.011396	0.011396	0.011396
0.011396	0.011329	0.011295	0.011329	0.011430	0.011430	0.011362
0.011362	0.011497	0.011665	0.011396	0.011530	0.011530	0.011463
0.011295	0.011362	0.011396	0.011799	0.011363	0.011396	0.011362
0.011396	0.011329	0.011329	0.011631	0.011564	0.011598	0.011329
0.011430	0.011497	0.011362	0.011430	0.011396	0.011262	0.011597
0.011362	0.011329	0.011329	0.011497	0.011329	0.011430	0.011665
0.011362	0.011740	0.011732	0.011548	0.011286	0.011434	0.011267
0.011280	0.011761	0.011271	0.011507	0.011353	0.011467	0.011485
0.011519	0.011324	0.011394	0.011258	0.011330	0.011328	0.011425
0.011437	0.011336	0.011412	0.011342	0.011094	0.011497	0.011564
0.011564	0.011430	0.011533	0.011535	0.011670	0.011626	0.011329
0.011597	0.011463	0.011329	0.011430	0.011228	0.011463	0.011262
0.011329	0.011329	0.011295	0.011262	0.011295	0.011262	0.011094
0.011340	0.011340	0.011334	0.011368	0.011505	0.011470	0.011470
0.011469	0.011396	0.011134	0.011168	0.011331	0.011501	0.011501
0.011296	0.011365	0.011599	0.011567	0.011497	0.011531	0.011463
0.011396	0.011463	0.011463	0.011362	0.011934	0.011530	0.011598
0.011631	0.011701	0.011333	0.011407	0.011295	0.011497	0.011463
0.011498	0.011530	0.011530	0.011530	0.011650	0.011665	0.011303
0.011196	0.011199	0.011299	0.011264	0.011368	0.011210	0.011536
0.011335	0.011411	0.011108	0.011360	0.011329	0.011402	0.011295
0.011262	0.011262	0.011329	0.011295	0.011497	0.011463	0.011396
0.011497	0.011564	0.011295	0.011295	0.011329	0.011329	0.011330
0.011398	0.011329	0.000000	0.011094	0.011497	0.011564	0.011564
0.011430	0.011533	0.011535	0.011670	0.011626	0.011329	0.011530
0.011463	0.011463	0.011297	0.011478	0.011408	0.011435	0.011270
0.011439	0.011698	0.011399	0.011366	0.011262	0.011463	0.011329
0.011430	0.011362	0.011262	0.011363	0.011463	0.011466	0.011362
0.011262	0.011195	0.011329	0.011262	0.011195	0.011298	0.011463
0.011362	0.011295	0.011329	0.011295	0.011329	0.011262	0.011463
0.011329	0.011362	0.011362	0.011295	0.011362	0.011598	0.011262
0.011295	0.011698	0.011295	0.011329	0.011295	0.011665	0.011274
0.011735	0.011273	0.011311	0.011503	0.011589	0.011491	0.011227
0.011270	0.011463	0.011564	0.011671	0.011531	0.011669	0.011800
0.011699	0.011737	0.011698	0.011949	0.011665	0.012506	0.011700
0.012439	0.012173	0.012307	0.012612	0.011665	0.011362	0.011665
0.012103	0.012038	0.011631	0.011531	0.011598	0.011598	0.011568
0.011463	0.011467	0.011530	0.011497	0.011867	0.011934	0.011935
0.011834	0.011934	0.012038	0.011804	0.012104	0.012110	0.012171
0.012172	0.012205	0.012152	0.012172	0.012173	0.011396	0.011699
0.011766	0.012270	0.011772	0.012001	0.012474	0.011536	0.011430
0.011396	0.011396	0.011867	0.011924	0.012010	0.011732	0.011397
S143 0.011564	0.011564	0.011672	0.011636	0.011636	0.011672	
0.011533	0.011424	0.011636	0.011096	0.011204	0.011168	0.011132
0.011168	0.011348	0.011132	0.011168	0.011240	0.011204	0.011168
0.011132	0.011168	0.011276	0.011132	0.011132	0.011168	0.011497
0.011420	0.011168	0.000000	0.011312	0.011168	0.011204	0.011096
0.011168	0.011276	0.011204	0.011204	0.011240	0.011204	0.011384
0.011132	0.011204	0.011240	0.011168	0.011276	0.011312	0.011204
0.011204	0.011384	0.011276	0.011528	0.011204	0.011456	0.011204
0.011168	0.011276	0.011348	0.011456	0.011528	0.011240	0.011384
0.011204	0.011276	0.011204	0.011384	0.011600	0.011204	0.011276
0.011168	0.011276	0.011204	0.011312	0.011204	0.011240	0.011240
0.011132	0.011168	0.011096	0.011204	0.011204	0.011204	0.011312
0.011240	0.011168	0.011240	0.011132	0.011312	0.011096	0.011168
0.011348	0.011276	0.011492	0.011312	0.011204	0.011420	0.011204

0.011331	0.011567	0.011372	0.011101	0.011099	0.011175	0.011305
0.011891	0.011376	0.011103	0.011456	0.011456	0.011240	0.011276
0.011276	0.011240	0.011312	0.011204	0.011312	0.011204	0.011240
0.011204	0.011276	0.011528	0.011384	0.011276	0.011240	0.011132
0.011132	0.011132	0.011168	0.011168	0.011384	0.011348	0.011276
0.011384	0.011456	0.011168	0.011168	0.011204	0.011204	0.011204
0.011276	0.011204	0.011204	0.000000	0.011134	0.011097	0.011204
0.011204	0.011132	0.011175	0.011168	0.011096	0.011132	0.011168
0.011425	0.011168	0.011205	0.011132	0.011132	0.011276	0.011240
0.011312	0.011240	0.011240	0.011276	0.011276	0.011600	0.011420
0.011492	0.011312	0.011204	0.011312	0.011096	0.011348	0.011132
0.011168	0.011204	0.011168	0.011132	0.011132	0.011132	0.011240
0.011132	0.011276	0.011240	0.011096	0.011241	0.011348	0.011314
0.011312	0.011240	0.011132	0.000000	0.011204	0.011132	0.000000
0.011169	0.011312	0.011096	0.011312	0.011096	0.011168	0.011168
0.011240	0.011168	0.011168	0.011168	0.011204	0.000000	0.011168
0.011168	0.011168	0.011240	0.011168	0.011204	0.011240	0.011492
0.011528	0.011420	0.011528	0.011349	0.011564	0.011204	0.011168
0.011205	0.011493	0.011307	0.011097	0.011569	0.000000	0.011187
0.011570	0.011496	0.011348	0.011600	0.011243	0.011456	0.011384
0.011348	0.011528	0.011362	0.011193	0.011238	0.011240	0.011314
0.011420	0.011313	0.011423	0.011495	0.011384	0.011528	0.011641
0.011348	0.011495	0.011420	0.011384	0.011672	0.011096	0.011312
0.011204	0.011204	0.011096	0.000000	0.011132	0.011168	0.011240
0.011132	0.011204	0.011096	0.011240	0.011132	0.011168	0.011240
0.011168	0.011600	0.011456	0.011492	0.000000	0.011168	0.011204
0.011204	0.011168	0.011204	0.011276	0.011132	0.011132	0.011132
0.011672	0.011276	0.011312	0.011096	0.011204	0.011348	0.011096
0.011096	0.011168	0.011168	0.011312	0.011132	0.011276	0.011348
0.011348	0.011132	0.011168	0.000000	0.011096	0.011204	0.011204
0.000000	0.011168	0.000000	0.011096	0.011240	0.011384	0.011132
0.011096	0.011240	0.011168	0.000000	0.011384	0.000000	0.011240
0.011280	0.011132	0.011420	0.011240	0.011312	0.011168	0.011276
0.011132	0.011456	0.000000	0.011276	0.011132	0.011132	0.000000
0.011168	0.011528	0.011204	0.011276	0.011096	0.011456	0.011132
0.011132	0.011132	0.011168	0.011240	0.011168	0.011276	0.011312
0.011312	0.011312	0.011204	0.011276	0.011240	0.011132	0.011096
0.011132	0.011132	0.011204	0.011132	0.000000	0.011276	0.011132
0.011168	0.011168	0.011240	0.011348	0.011240	0.011132	0.011168
0.011240	0.011096	0.011276	0.011132	0.011276	0.011384	0.011600
0.011168	0.011564	0.011169	0.017794	0.011127	0.011250	0.011499
0.011463	0.011499	0.011169	0.011181	0.011634	0.011536	0.011249
0.011405	0.011439	0.000000	0.011147	0.011141	0.011215	0.011105
0.011401	0.011329	0.011328	0.011128	0.011276	0.011348	0.011350
0.011170	0.011240	0.011312	0.011276	0.011240	0.011240	0.011204
0.011240	0.011168	0.011204	0.011240	0.011168	0.011132	0.011168
0.011168	0.011240	0.011132	0.011240	0.011240	0.011312	0.011204
0.011240	0.011204	0.011456	0.011456	0.011276	0.011312	0.011348
0.011096	0.011096	0.011132	0.011204	0.011096	0.011348	0.011348
0.011348	0.011348	0.011312	0.011384	0.011564	0.011385	0.011204
0.011240	0.011276	0.011312	0.011168	0.011204	0.011168	0.011240
0.011312	0.011312	0.011348	0.011276	0.011168	0.011420	0.011348
0.011242	0.011096	0.011240	0.011312	0.011204	0.011384	0.011420
0.011600	0.011243	0.011456	0.011384	0.011348	0.011528	0.011132
0.011313	0.011204	0.011168	0.011168	0.011240	0.011132	0.011240
0.011240	0.011312	0.011204	0.011240	0.011204	0.011456	0.011456
0.011276	0.011312	0.011348	0.011312	0.011960	0.011364	0.011249
0.011357	0.011320	0.011434	0.011283	0.011396	0.011354	0.011314
0.011905	0.011208	0.011352	0.011281	0.011572	0.011386	0.011497
0.011933	0.011379	0.011240	0.011240	0.011240	0.011276	0.011204

0.011096	0.011096	0.011276	0.011240	0.011240	0.011384	0.011349
0.011204	0.011096	0.011132	0.011204	0.000000	0.011132	0.000000
0.011204	0.011240	0.011132	0.011096	0.011204	0.011132	0.011168
0.011168	0.011096	0.011168	0.011168	0.011096	0.011276	0.011348
0.011204	0.011312	0.011240	0.011204	0.011132	0.011528	0.011204
0.011246	0.011168	0.011420	0.011423	0.011240	0.011276	0.011240
0.011276	0.011168	0.011168	0.011168	0.011312	0.011312	0.011240
0.011240	0.011348	0.011528	0.011276	0.011384	0.011384	0.011348
0.011168	0.011240	0.011276	0.011636	0.011240	0.011240	0.011240
0.011240	0.011204	0.011204	0.011528	0.011348	0.011492	0.011204
0.011312	0.011384	0.011240	0.011312	0.011240	0.011132	0.011456
0.011240	0.011168	0.011204	0.011348	0.011204	0.011312	0.011564
0.011168	0.011570	0.011600	0.011396	0.011117	0.011312	0.011167
0.011117	0.011624	0.011133	0.011339	0.011162	0.011273	0.011279
0.011377	0.000000	0.011231	0.000000	0.011117	0.011266	0.011272
0.011296	0.011142	0.011282	0.011171	0.011241	0.011384	0.011456
0.011420	0.011312	0.011386	0.011423	0.011496	0.011553	0.011168
0.011492	0.011312	0.011204	0.011312	0.011096	0.011348	0.011132
0.011168	0.011204	0.011168	0.011132	0.011132	0.011132	0.011240
0.011206	0.011206	0.011204	0.011204	0.011384	0.011312	0.011349
0.011348	0.011240	0.000000	0.000000	0.011204	0.011384	0.011312
0.011168	0.011204	0.011492	0.011420	0.011384	0.011420	0.011348
0.011276	0.011348	0.011312	0.011204	0.011816	0.011384	0.011492
0.011528	0.011518	0.011206	0.011245	0.011168	0.011384	0.011348
0.011349	0.011348	0.011384	0.011384	0.011481	0.011564	0.011171
0.000000	0.000000	0.011169	0.011133	0.011206	0.000000	0.011387
0.011207	0.011335	0.000000	0.011219	0.011204	0.011276	0.011132
0.011132	0.011132	0.011168	0.011168	0.011384	0.011348	0.011276
0.011384	0.011456	0.011168	0.011168	0.011204	0.011204	0.011204
0.011276	0.011204	0.011204	0.011241	0.011384	0.011456	0.011420
0.011312	0.011386	0.011423	0.011496	0.011553	0.011168	0.011420
0.011348	0.011348	0.011169	0.011358	0.011282	0.011279	0.011173
0.011208	0.011552	0.011332	0.011206	0.011096	0.011312	0.011132
0.011276	0.011240	0.011096	0.011241	0.011348	0.011314	0.011240
0.011132	0.000000	0.011204	0.011132	0.000000	0.011169	0.011312
0.011240	0.011168	0.011204	0.011240	0.011168	0.011132	0.011348
0.011204	0.011240	0.011240	0.011168	0.011240	0.011456	0.011132
0.011168	0.011528	0.011168	0.011132	0.011132	0.011528	0.011105
0.011572	0.011103	0.011164	0.011349	0.011440	0.011359	0.000000
0.011115	0.011348	0.011456	0.011569	0.011420	0.011567	0.011708
0.011600	0.011602	0.011600	0.011865	0.011528	0.012392	0.011601
0.012248	0.012106	0.012215	0.012469	0.011528	0.011240	0.011564
0.011960	0.011963	0.011528	0.011420	0.011492	0.011492	0.011459
0.011348	0.011351	0.011420	0.011384	0.011744	0.011816	0.011780
0.011672	0.011780	0.011853	0.011675	0.011961	0.012003	0.012069
0.012069	0.012069	0.012002	0.012033	0.012035	0.011276	0.011564
0.011600	0.012104	0.011677	0.011852	0.012394	0.011424	0.011312
0.011276	0.011276	0.011708	0.011788	0.011866	0.011636	0.011277
S144 0.011639	0.011639	0.011711	0.011675	0.011675	0.011747	
0.011609	0.011499	0.011675	0.011169	0.011277	0.011241	0.011205
0.011241	0.011422	0.011205	0.011241	0.011313	0.011277	0.011241
0.011205	0.011241	0.011349	0.011205	0.011241	0.011205	0.011573
0.011458	0.011205	0.011133	0.011386	0.011277	0.011277	0.011241
0.011241	0.011386	0.011277	0.011277	0.011313	0.011277	0.011422
0.011169	0.011277	0.011313	0.011241	0.011349	0.011386	0.011277
0.011277	0.011458	0.011314	0.011603	0.011277	0.011530	0.011277
0.011241	0.011350	0.011422	0.011530	0.011603	0.011313	0.011458
0.011313	0.011386	0.011277	0.011530	0.011675	0.011277	0.011349
0.011241	0.011349	0.011241	0.011386	0.011313	0.011313	0.011313
0.011205	0.011241	0.011205	0.011277	0.011277	0.011278	0.011349

0.011313	0.011241	0.011349	0.011241	0.011386	0.011205	0.011241
0.011422	0.011349	0.011603	0.011386	0.011277	0.011530	0.011277
0.011415	0.011617	0.011411	0.011142	0.011137	0.011217	0.011346
0.011899	0.011432	0.011146	0.011494	0.011422	0.011313	0.011349
0.011349	0.011277	0.011386	0.011277	0.011386	0.011313	0.011313
0.011277	0.011386	0.011603	0.011458	0.011349	0.011313	0.011241
0.011205	0.011205	0.011277	0.011241	0.011458	0.011422	0.011349
0.011458	0.011530	0.011241	0.011241	0.011277	0.011277	0.011277
0.011350	0.011277	0.011277	0.011134	0.000000	0.011169	0.011277
0.011277	0.011205	0.011175	0.011207	0.011169	0.011205	0.011241
0.011469	0.011278	0.011313	0.011205	0.011207	0.011349	0.011313
0.011386	0.011313	0.011313	0.011386	0.011349	0.011747	0.011494
0.011566	0.011422	0.011277	0.011386	0.011169	0.011386	0.011205
0.011241	0.011277	0.011241	0.011205	0.011205	0.011205	0.011313
0.011241	0.011313	0.011313	0.011205	0.011278	0.011422	0.011388
0.011386	0.011313	0.011205	0.011133	0.011277	0.011205	0.011133
0.011243	0.011386	0.011205	0.011386	0.011133	0.011277	0.011205
0.011349	0.011241	0.011277	0.011205	0.011241	0.011133	0.011277
0.011313	0.011241	0.011313	0.011241	0.011277	0.011313	0.011530
0.011603	0.011566	0.011603	0.011387	0.011639	0.011277	0.011241
0.011278	0.011543	0.011357	0.011134	0.011645	0.011097	0.011229
0.011646	0.011535	0.011349	0.011675	0.011318	0.011494	0.011494
0.011422	0.011530	0.011437	0.011236	0.011327	0.011313	0.011386
0.011458	0.011387	0.011461	0.011607	0.011458	0.011566	0.011717
0.011423	0.011608	0.011494	0.011458	0.011748	0.011169	0.011386
0.011313	0.011277	0.011169	0.011169	0.011241	0.011241	0.011313
0.011241	0.011313	0.011205	0.011313	0.011241	0.011241	0.011241
0.011241	0.011675	0.011530	0.011566	0.011133	0.011241	0.011277
0.011277	0.011205	0.011313	0.011349	0.011205	0.011205	0.011205
0.011747	0.011349	0.011386	0.011169	0.011241	0.011458	0.011169
0.011169	0.011241	0.011241	0.011386	0.011205	0.011349	0.011422
0.011422	0.011205	0.011241	0.011133	0.011169	0.011277	0.011277
0.011133	0.011241	0.011133	0.011133	0.011313	0.011458	0.011205
0.011169	0.011313	0.011241	0.011133	0.011458	0.011133	0.011313
0.011392	0.011205	0.011494	0.011350	0.011386	0.011241	0.011349
0.011205	0.011530	0.011133	0.011349	0.011205	0.011205	0.011133
0.011349	0.011567	0.011277	0.011349	0.011169	0.011530	0.011205
0.011205	0.011205	0.011205	0.011349	0.011241	0.011349	0.011422
0.011386	0.011313	0.011277	0.011386	0.011349	0.011205	0.011169
0.011205	0.011205	0.011277	0.011169	0.011169	0.011349	0.011205
0.011241	0.011241	0.011349	0.011422	0.011313	0.011241	0.011205
0.011277	0.011169	0.011349	0.011205	0.011349	0.011422	0.011675
0.011241	0.011566	0.011207	0.018067	0.011194	0.011323	0.011541
0.011504	0.011545	0.011280	0.011240	0.011636	0.011578	0.011316
0.011451	0.011440	0.011101	0.011194	0.011183	0.011165	0.011151
0.011446	0.011368	0.011372	0.011195	0.011313	0.011422	0.011424
0.011281	0.011313	0.011386	0.011349	0.011313	0.011314	0.011277
0.011313	0.011241	0.011205	0.011313	0.011277	0.011205	0.011241
0.011241	0.011313	0.011205	0.011313	0.011349	0.011386	0.011277
0.011313	0.011277	0.011530	0.011530	0.011350	0.011350	0.011422
0.011133	0.011169	0.011205	0.011313	0.011169	0.011422	0.011422
0.011386	0.011349	0.011386	0.011422	0.011639	0.011423	0.011313
0.011277	0.011349	0.011422	0.011241	0.011277	0.011241	0.011313
0.011350	0.011349	0.011386	0.011313	0.011278	0.011494	0.011458
0.011279	0.011169	0.011313	0.011386	0.011277	0.011458	0.011494
0.011675	0.011318	0.011494	0.011494	0.011422	0.011530	0.011205
0.011387	0.011277	0.011241	0.011241	0.011313	0.011205	0.011313
0.011349	0.011386	0.011277	0.011313	0.011277	0.011530	0.011530
0.011350	0.011350	0.011422	0.011386	0.012038	0.011404	0.011324
0.011434	0.011359	0.011509	0.011359	0.011435	0.011430	0.011353

0.011985	0.011283	0.011428	0.011356	0.011612	0.011461	0.011610
0.012012	0.011457	0.011314	0.011313	0.011313	0.011349	0.011277
0.011169	0.011205	0.011349	0.011313	0.011314	0.011422	0.011387
0.011277	0.011205	0.011241	0.011277	0.011169	0.011241	0.011133
0.011277	0.011313	0.011205	0.011169	0.011277	0.011205	0.011241
0.011241	0.011169	0.011241	0.011277	0.011169	0.011386	0.011422
0.011277	0.011386	0.011313	0.011277	0.011169	0.011603	0.011277
0.011319	0.011241	0.011458	0.011462	0.011350	0.011313	0.011313
0.011349	0.011277	0.011241	0.011277	0.011386	0.011386	0.011313
0.011313	0.011422	0.011603	0.011313	0.011494	0.011494	0.011422
0.011241	0.011313	0.011349	0.011711	0.011313	0.011350	0.011313
0.011313	0.011277	0.011277	0.011530	0.011458	0.011494	0.011277
0.011386	0.011458	0.011313	0.011386	0.011313	0.011205	0.011530
0.011313	0.011241	0.011277	0.011422	0.011277	0.011386	0.011639
0.011241	0.011682	0.011675	0.011439	0.011173	0.011387	0.011219
0.011119	0.011664	0.011171	0.011385	0.011213	0.011273	0.011334
0.011428	0.011130	0.011287	0.011128	0.011173	0.011334	0.011315
0.011341	0.011225	0.011322	0.011210	0.011314	0.011422	0.011494
0.011494	0.011386	0.011497	0.011425	0.011571	0.011670	0.011241
0.011566	0.011422	0.011277	0.011386	0.011169	0.011386	0.011205
0.011241	0.011277	0.011241	0.011205	0.011205	0.011205	0.011313
0.011245	0.011281	0.011278	0.011313	0.011458	0.011386	0.011423
0.011422	0.011313	0.011138	0.011136	0.011277	0.011458	0.011422
0.011241	0.011313	0.011530	0.011494	0.011422	0.011494	0.011422
0.011313	0.011422	0.011422	0.011277	0.011892	0.011386	0.011530
0.011603	0.011598	0.011244	0.011283	0.011241	0.011458	0.011422
0.011459	0.011422	0.011422	0.011422	0.011611	0.011639	0.011245
0.011133	0.011134	0.011207	0.011206	0.011279	0.011139	0.011462
0.011282	0.011425	0.011160	0.011297	0.011277	0.011349	0.011241
0.011205	0.011205	0.011277	0.011241	0.011458	0.011422	0.011349
0.011458	0.011530	0.011241	0.011241	0.011277	0.011277	0.011277
0.011350	0.011277	0.011277	0.011314	0.011422	0.011494	0.011494
0.011386	0.011497	0.011425	0.011571	0.011670	0.011241	0.011494
0.011422	0.011422	0.011206	0.011433	0.011358	0.011390	0.011211
0.011357	0.011552	0.011381	0.011317	0.011205	0.011386	0.011241
0.011313	0.011313	0.011205	0.011278	0.011422	0.011388	0.011313
0.011205	0.011133	0.011277	0.011205	0.011133	0.011243	0.011386
0.011313	0.011241	0.011205	0.011313	0.011277	0.011205	0.011422
0.011277	0.011313	0.011313	0.011241	0.011313	0.011530	0.011169
0.011241	0.011603	0.011241	0.011241	0.011241	0.011603	0.011148
0.011624	0.011146	0.011215	0.011390	0.011482	0.011400	0.011104
0.011168	0.011422	0.011530	0.011645	0.011495	0.011606	0.011784
0.011675	0.011715	0.011675	0.011867	0.011566	0.012544	0.011604
0.012255	0.012148	0.012294	0.012476	0.011566	0.011313	0.011639
0.012038	0.012004	0.011603	0.011494	0.011566	0.011566	0.011533
0.011422	0.011389	0.011494	0.011458	0.011820	0.011892	0.011892
0.011747	0.011856	0.011930	0.011752	0.012074	0.012081	0.012147
0.012147	0.012183	0.012085	0.012147	0.012149	0.011349	0.011639
0.011675	0.012110	0.011754	0.011965	0.012510	0.011461	0.011386
0.011349	0.011349	0.011711	0.011833	0.011912	0.011675	0.011350
S145	0.011586	0.011586	0.011656	0.011621	0.011621	0.011691
0.011557	0.011450	0.011621	0.011130	0.011236	0.011201	0.011165
0.011200	0.011376	0.011165	0.011200	0.011271	0.011236	0.011200
0.011165	0.011200	0.011306	0.011166	0.011201	0.011200	0.011521
0.011446	0.000000	0.011095	0.011341	0.011236	0.011236	0.011271
0.011201	0.011341	0.011236	0.011236	0.011271	0.011235	0.011376
0.011165	0.011235	0.011271	0.011200	0.011306	0.011341	0.011236
0.011236	0.011411	0.011306	0.011551	0.011236	0.011516	0.011236
0.011200	0.011306	0.011376	0.011516	0.011551	0.011271	0.011411
0.011271	0.011341	0.011236	0.011481	0.011691	0.011236	0.011306

0.011200	0.011306	0.011201	0.011341	0.011271	0.011271	0.011271
0.011165	0.011200	0.011165	0.011271	0.011235	0.011271	0.011306
0.011271	0.011200	0.011306	0.011200	0.011341	0.011165	0.011200
0.011411	0.011341	0.011586	0.011376	0.011236	0.011481	0.011235
0.011410	0.011608	0.011413	0.011142	0.011138	0.011214	0.011347
0.012010	0.011433	0.011146	0.011481	0.011481	0.011271	0.011341
0.011306	0.011271	0.011341	0.011236	0.011341	0.011271	0.011271
0.011235	0.011341	0.011551	0.011411	0.011306	0.011271	0.011201
0.011165	0.011165	0.011236	0.011200	0.011411	0.011376	0.011306
0.011411	0.011481	0.011200	0.011200	0.011236	0.011235	0.011237
0.011307	0.011236	0.011236	0.011097	0.011169	0.000000	0.011235
0.011236	0.011167	0.000000	0.000000	0.011130	0.011165	0.011200
0.011458	0.011236	0.011274	0.011165	0.011168	0.011306	0.011271
0.011341	0.011271	0.011271	0.011306	0.011306	0.011691	0.011446
0.011516	0.011341	0.011236	0.011341	0.011130	0.011376	0.011165
0.011200	0.011236	0.011200	0.011165	0.011200	0.011165	0.011271
0.011236	0.011306	0.011271	0.011165	0.011236	0.011376	0.011378
0.011376	0.011271	0.011165	0.011096	0.011235	0.011165	0.011095
0.011202	0.011376	0.011165	0.011376	0.011130	0.011236	0.011200
0.011306	0.011200	0.011236	0.011201	0.011236	0.011095	0.011236
0.011271	0.011200	0.011271	0.011200	0.011235	0.011271	0.011516
0.011551	0.011516	0.011586	0.011377	0.011586	0.011235	0.011200
0.011237	0.011524	0.011345	0.011132	0.011592	0.011096	0.011225
0.011592	0.011521	0.011376	0.011621	0.011274	0.011481	0.011446
0.011376	0.011551	0.011388	0.011230	0.011275	0.011271	0.011341
0.011411	0.011342	0.011449	0.011555	0.011481	0.011516	0.011661
0.011377	0.011555	0.011481	0.011411	0.011692	0.011130	0.011341
0.011306	0.011236	0.011130	0.011130	0.011200	0.011200	0.011271
0.011200	0.011271	0.011165	0.011271	0.011200	0.011200	0.011271
0.011200	0.011621	0.011516	0.011551	0.011095	0.011200	0.011236
0.011236	0.011200	0.011236	0.011306	0.011165	0.011165	0.011165
0.011691	0.011306	0.011341	0.011130	0.011235	0.011411	0.011165
0.011165	0.011200	0.011200	0.011376	0.011165	0.011306	0.011411
0.011376	0.011165	0.011200	0.011095	0.011130	0.011306	0.011235
0.011095	0.011200	0.011095	0.011130	0.011271	0.011411	0.011165
0.011130	0.011271	0.011200	0.011095	0.011411	0.011095	0.011271
0.011346	0.011165	0.011446	0.011306	0.011341	0.011200	0.011306
0.011165	0.011516	0.011095	0.011341	0.011165	0.011165	0.011095
0.011341	0.011551	0.011236	0.011306	0.011130	0.011516	0.011165
0.011165	0.011165	0.011200	0.011306	0.011200	0.011306	0.011341
0.011341	0.011341	0.011235	0.011341	0.011306	0.011165	0.011130
0.011165	0.011165	0.011236	0.011165	0.011130	0.011306	0.011165
0.011200	0.011200	0.011341	0.011376	0.011271	0.011200	0.011165
0.011236	0.011130	0.011306	0.011165	0.011306	0.011411	0.011621
0.011200	0.011586	0.011204	0.018027	0.011194	0.011380	0.011534
0.011494	0.011538	0.011278	0.011241	0.011700	0.011565	0.011315
0.011453	0.011507	0.011101	0.011191	0.011182	0.011216	0.011150
0.011449	0.011370	0.011368	0.011128	0.011306	0.011376	0.011378
0.011238	0.011271	0.011341	0.011306	0.011271	0.011271	0.011236
0.011271	0.011200	0.011236	0.011271	0.011236	0.011165	0.011200
0.011200	0.011271	0.011165	0.011271	0.011306	0.011341	0.011236
0.011271	0.011236	0.011516	0.011516	0.011306	0.011306	0.011376
0.011130	0.011130	0.011165	0.011271	0.011130	0.011376	0.011376
0.011411	0.011376	0.011341	0.011446	0.011586	0.011447	0.011271
0.011236	0.011306	0.011376	0.011200	0.011271	0.011236	0.011271
0.011341	0.011341	0.011376	0.011341	0.011236	0.011446	0.011411
0.011237	0.011165	0.011271	0.011341	0.011236	0.011411	0.011446
0.011621	0.011274	0.011481	0.011446	0.011376	0.011551	0.011165
0.011342	0.011236	0.011200	0.011200	0.011271	0.011165	0.011271
0.011306	0.011341	0.011236	0.011271	0.011236	0.011516	0.011516

0.011306	0.011306	0.011411	0.011341	0.011973	0.011393	0.011315
0.011387	0.011349	0.011458	0.011349	0.011423	0.011383	0.011344
0.011919	0.011239	0.011380	0.011311	0.011558	0.011413	0.011557
0.011946	0.011405	0.011272	0.011271	0.011271	0.011306	0.011237
0.011130	0.011165	0.011306	0.011271	0.011272	0.011411	0.011377
0.011235	0.011165	0.011200	0.011236	0.011130	0.011200	0.011095
0.011235	0.011271	0.011165	0.011130	0.011236	0.011165	0.011200
0.011200	0.011130	0.011200	0.011236	0.011130	0.011341	0.011376
0.011271	0.011341	0.011271	0.011271	0.011200	0.011551	0.011235
0.011275	0.011200	0.011446	0.011449	0.011306	0.011306	0.011306
0.011306	0.011236	0.011200	0.011236	0.011341	0.011341	0.011271
0.011271	0.011376	0.011551	0.011306	0.011481	0.011481	0.011376
0.011200	0.011271	0.011306	0.011691	0.011271	0.011306	0.011271
0.011306	0.011235	0.011235	0.011551	0.011446	0.011516	0.011236
0.011341	0.011411	0.011271	0.011341	0.011271	0.011165	0.011481
0.011271	0.011236	0.011235	0.011411	0.011236	0.011341	0.011586
0.011200	0.011628	0.011621	0.011441	0.011174	0.011344	0.011220
0.011175	0.011668	0.011170	0.011387	0.011214	0.011328	0.011337
0.011429	0.011130	0.011288	0.011128	0.011174	0.011335	0.011316
0.011337	0.011226	0.011318	0.011207	0.011271	0.011411	0.011481
0.011446	0.011341	0.011449	0.011450	0.011520	0.011615	0.011236
0.011516	0.011341	0.011236	0.011341	0.011130	0.011376	0.011165
0.011200	0.011236	0.011200	0.011165	0.011200	0.011165	0.011271
0.011241	0.011238	0.011237	0.011271	0.011411	0.011378	0.011377
0.011376	0.011271	0.011098	0.011097	0.011236	0.011411	0.011376
0.011200	0.011271	0.011446	0.011482	0.011411	0.011446	0.011376
0.011306	0.011376	0.011376	0.011271	0.011832	0.011411	0.011516
0.011516	0.011581	0.011238	0.011276	0.011200	0.011411	0.011376
0.011412	0.011411	0.011446	0.011446	0.011593	0.011586	0.011203
0.011096	0.011096	0.000000	0.011166	0.011237	0.011098	0.011414
0.011239	0.011371	0.011108	0.011251	0.011236	0.011306	0.011201
0.011165	0.011165	0.011236	0.011200	0.011411	0.011376	0.011306
0.011411	0.011481	0.011200	0.011200	0.011236	0.011235	0.011237
0.011307	0.011236	0.011236	0.011271	0.011411	0.011481	0.011446
0.011341	0.011449	0.011450	0.011520	0.011615	0.011236	0.011446
0.011376	0.011376	0.011201	0.011387	0.011312	0.011345	0.011206
0.011384	0.011537	0.011368	0.011238	0.011165	0.011376	0.011236
0.011306	0.011271	0.011165	0.011236	0.011376	0.011378	0.011271
0.011165	0.011096	0.011235	0.011165	0.011095	0.011202	0.011376
0.011271	0.011200	0.011236	0.011271	0.011236	0.011165	0.011376
0.011236	0.011271	0.011271	0.011200	0.011271	0.011481	0.011165
0.011200	0.011551	0.011200	0.011200	0.011200	0.011551	0.011149
0.011628	0.011147	0.011216	0.011392	0.011484	0.011398	0.011104
0.011169	0.011376	0.011481	0.011556	0.011446	0.011590	0.011727
0.011621	0.011659	0.011621	0.011844	0.011516	0.012464	0.011552
0.012253	0.012115	0.012221	0.012398	0.011586	0.011271	0.011586
0.011973	0.011975	0.011551	0.011446	0.011516	0.011516	0.011484
0.011376	0.011379	0.011446	0.011411	0.011797	0.011867	0.011867
0.011763	0.011867	0.011904	0.011730	0.012008	0.012014	0.012079
0.012079	0.012114	0.012022	0.012079	0.012045	0.011306	0.011621
0.011656	0.012148	0.011697	0.011937	0.012430	0.011450	0.011341
0.011306	0.011306	0.011797	0.011801	0.011899	0.011656	0.011306
S146	0.011665	0.011665	0.011800	0.011766	0.011766	0.011834
0.011638	0.011536	0.011766	0.011262	0.011329	0.011296	0.011262
0.011296	0.011464	0.011262	0.011296	0.011363	0.011329	0.011296
0.011262	0.011296	0.011396	0.011262	0.011296	0.011296	0.011604
0.011531	0.011296	0.011195	0.011430	0.011363	0.011329	0.011363
0.011329	0.011430	0.011329	0.011329	0.011363	0.011329	0.011497
0.011262	0.011329	0.011363	0.011296	0.011396	0.011430	0.011363
0.011329	0.011497	0.011397	0.011665	0.011329	0.011598	0.011329

0.011296	0.011396	0.011464	0.011598	0.011699	0.011396	0.011497
0.011363	0.011430	0.011329	0.011564	0.011800	0.011329	0.011396
0.011296	0.011396	0.011363	0.011430	0.011363	0.011363	0.011363
0.011262	0.011296	0.011262	0.011363	0.011329	0.011363	0.011430
0.011363	0.011329	0.011396	0.011296	0.011430	0.011262	0.011329
0.011497	0.011430	0.011665	0.011497	0.011329	0.011565	0.011329
0.011468	0.011695	0.011513	0.011216	0.011247	0.011355	0.011414
0.012085	0.011467	0.011225	0.011564	0.011564	0.011363	0.011464
0.011396	0.011363	0.011430	0.011329	0.011430	0.011363	0.011363
0.011329	0.011430	0.011632	0.011497	0.011396	0.011363	0.011296
0.011262	0.011262	0.011329	0.011296	0.011497	0.011464	0.011396
0.011497	0.011564	0.011296	0.011296	0.011329	0.011329	0.011331
0.011399	0.011329	0.011329	0.011204	0.011277	0.011235	0.000000
0.011331	0.011266	0.011318	0.011302	0.011230	0.011266	0.011297
0.011564	0.011336	0.011370	0.011262	0.011268	0.011397	0.011363
0.011430	0.011363	0.011363	0.011464	0.011396	0.011766	0.011531
0.011598	0.011464	0.011329	0.011430	0.011228	0.011464	0.011262
0.011329	0.011329	0.011296	0.011262	0.011296	0.011262	0.011363
0.011329	0.011430	0.011363	0.011262	0.011364	0.011464	0.011466
0.011464	0.011363	0.011262	0.011195	0.011329	0.011262	0.011195
0.011298	0.011464	0.011262	0.011464	0.011228	0.011329	0.011296
0.011396	0.011296	0.011329	0.011296	0.011329	0.011195	0.011329
0.011363	0.011296	0.011363	0.011296	0.011329	0.011363	0.011598
0.011699	0.011632	0.011699	0.011465	0.011699	0.011329	0.011296
0.011331	0.011586	0.011426	0.011232	0.011706	0.011197	0.011296
0.011672	0.011604	0.011497	0.011699	0.011402	0.011565	0.011531
0.011464	0.011632	0.011487	0.011349	0.011396	0.011363	0.011433
0.011531	0.011431	0.011568	0.011636	0.011564	0.011632	0.011739
0.011467	0.011638	0.011568	0.011500	0.011767	0.011228	0.011431
0.011396	0.011329	0.011228	0.011228	0.011296	0.011296	0.011363
0.011296	0.011363	0.011262	0.011363	0.011296	0.011296	0.011363
0.011296	0.011733	0.011632	0.011665	0.011195	0.011296	0.011329
0.011329	0.011296	0.011363	0.011396	0.011262	0.011262	0.011262
0.011800	0.011396	0.011430	0.011228	0.011329	0.011497	0.011262
0.011262	0.011329	0.011296	0.011464	0.011262	0.011396	0.011531
0.011464	0.011296	0.011296	0.011228	0.011228	0.011396	0.011329
0.011228	0.011329	0.011195	0.011228	0.011363	0.011497	0.011262
0.011228	0.011363	0.011296	0.011195	0.011531	0.011195	0.011363
0.011437	0.011262	0.011564	0.011396	0.011430	0.011296	0.011396
0.011262	0.011598	0.011195	0.011464	0.011262	0.011262	0.011195
0.011430	0.011632	0.011329	0.011396	0.011228	0.011632	0.011262
0.011262	0.011262	0.011296	0.011396	0.011296	0.011396	0.011464
0.011464	0.011430	0.011329	0.011430	0.011396	0.011262	0.011228
0.011262	0.011262	0.011329	0.011262	0.011228	0.011396	0.011262
0.011296	0.011296	0.011430	0.011464	0.011363	0.011296	0.011296
0.011363	0.011296	0.011396	0.011262	0.011396	0.011497	0.011733
0.011296	0.011699	0.011302	0.017823	0.011191	0.011488	0.011653
0.011614	0.011658	0.011322	0.011234	0.011719	0.011682	0.011364
0.011440	0.011610	0.011177	0.011228	0.011255	0.011356	0.011232
0.011482	0.011470	0.011431	0.011259	0.011430	0.011464	0.011466
0.011335	0.011430	0.011430	0.011396	0.011363	0.011363	0.011329
0.011363	0.011296	0.011329	0.011363	0.011329	0.011262	0.011296
0.011296	0.011363	0.011262	0.011363	0.011396	0.011430	0.011329
0.011363	0.011329	0.011665	0.011665	0.011397	0.011431	0.011464
0.011228	0.011228	0.011262	0.011363	0.011228	0.011464	0.011464
0.011497	0.011497	0.011430	0.011531	0.011699	0.011532	0.011363
0.011363	0.011430	0.011464	0.011296	0.011363	0.011329	0.011363
0.011430	0.011430	0.011464	0.011430	0.011330	0.011531	0.011497
0.011365	0.011262	0.011363	0.011464	0.011363	0.011497	0.011531
0.011699	0.011402	0.011565	0.011531	0.011464	0.011632	0.011262

0.011431	0.011329	0.011296	0.011296	0.011363	0.011262	0.011363
0.011396	0.011430	0.011329	0.011363	0.011329	0.011665	0.011665
0.011397	0.011431	0.011497	0.011430	0.012070	0.011489	0.011412
0.011482	0.011442	0.011552	0.011442	0.011551	0.011475	0.011440
0.012023	0.011337	0.011472	0.011406	0.011680	0.011537	0.011639
0.012047	0.011512	0.011365	0.011363	0.011363	0.011396	0.011331
0.011228	0.011262	0.011396	0.011363	0.011365	0.011497	0.011465
0.011329	0.011262	0.011296	0.011329	0.011228	0.011296	0.011195
0.011329	0.011363	0.011262	0.011228	0.011329	0.011262	0.011296
0.011296	0.011228	0.011296	0.011329	0.011228	0.011430	0.011464
0.011363	0.011430	0.011363	0.011363	0.011296	0.011699	0.011329
0.011374	0.011296	0.011531	0.011535	0.011396	0.011396	0.011396
0.011396	0.011329	0.011296	0.011329	0.011430	0.011430	0.011363
0.011363	0.011497	0.011665	0.011396	0.011598	0.011598	0.011464
0.011296	0.011363	0.011396	0.011800	0.011363	0.011396	0.011363
0.011396	0.011329	0.011329	0.011632	0.011565	0.011598	0.011329
0.011430	0.011497	0.011363	0.011430	0.011396	0.011262	0.011598
0.011363	0.011329	0.011329	0.011497	0.011329	0.011430	0.011665
0.011296	0.011706	0.011733	0.011507	0.011173	0.011434	0.011215
0.011280	0.011722	0.011271	0.011462	0.011255	0.011366	0.011326
0.011468	0.011126	0.011283	0.011126	0.011168	0.011328	0.011344
0.011362	0.011296	0.011412	0.011342	0.011364	0.011497	0.011564
0.011531	0.011430	0.011534	0.011535	0.011671	0.011662	0.011329
0.011598	0.011464	0.011329	0.011430	0.011228	0.011464	0.011262
0.011329	0.011329	0.011296	0.011262	0.011296	0.011262	0.011363
0.011340	0.011340	0.011334	0.011368	0.011505	0.011470	0.011470
0.011469	0.011396	0.011171	0.011204	0.011331	0.011501	0.011501
0.011296	0.011365	0.011599	0.011567	0.011497	0.011531	0.011464
0.011396	0.011464	0.011464	0.011363	0.011935	0.011531	0.011598
0.011632	0.011702	0.011333	0.011372	0.011296	0.011497	0.011464
0.011498	0.011497	0.011531	0.011531	0.011689	0.011665	0.011303
0.011196	0.011199	0.011299	0.011264	0.011368	0.011210	0.011536
0.011335	0.011455	0.011203	0.011322	0.011329	0.011402	0.011296
0.011262	0.011262	0.011329	0.011296	0.011497	0.011464	0.011396
0.011497	0.011564	0.011296	0.011296	0.011329	0.011329	0.011331
0.011399	0.011329	0.011329	0.011364	0.011497	0.011564	0.011531
0.011430	0.011534	0.011535	0.011671	0.011662	0.011329	0.011531
0.011464	0.011464	0.011297	0.011478	0.011408	0.011435	0.011305
0.011474	0.011653	0.011441	0.011366	0.011262	0.011464	0.011329
0.011430	0.011363	0.011262	0.011364	0.011464	0.011466	0.011363
0.011262	0.011195	0.011329	0.011262	0.011195	0.011298	0.011464
0.011363	0.011296	0.011329	0.011363	0.011329	0.011262	0.011464
0.011329	0.011363	0.011363	0.011296	0.011363	0.011598	0.011262
0.011296	0.011699	0.011296	0.011329	0.011296	0.011665	0.011232
0.011622	0.011188	0.011211	0.011503	0.011549	0.011491	0.011186
0.011165	0.011464	0.011564	0.011671	0.011532	0.011669	0.011801
0.011700	0.011737	0.011699	0.011949	0.011665	0.012541	0.011700
0.012407	0.012207	0.012342	0.012613	0.011665	0.011363	0.011665
0.012104	0.012039	0.011632	0.011531	0.011598	0.011598	0.011568
0.011464	0.011467	0.011531	0.011497	0.011868	0.011935	0.011935
0.011835	0.011935	0.012038	0.011804	0.012104	0.012110	0.012172
0.012171	0.012205	0.012153	0.012172	0.012174	0.011396	0.011699
0.011766	0.012272	0.011772	0.012002	0.012475	0.011536	0.011430
0.011396	0.011396	0.011867	0.011924	0.012010	0.011733	0.011397
S147	0.011670	0.011670	0.011772	0.011738	0.011738	0.011806
0.011643	0.011540	0.011738	0.011263	0.011331	0.011298	0.011263
0.011297	0.011467	0.011263	0.011297	0.011365	0.011331	0.011297
0.011263	0.011297	0.011399	0.011264	0.011298	0.011297	0.011608
0.011534	0.011297	0.011196	0.011433	0.011365	0.011331	0.011365
0.011331	0.011433	0.011331	0.011331	0.011365	0.011128	0.011501

0.011263	0.011331	0.011365	0.011297	0.011399	0.011433	0.011365
0.011331	0.011501	0.011399	0.011636	0.011331	0.011602	0.011331
0.011297	0.011399	0.011467	0.011602	0.011670	0.011399	0.011500
0.011365	0.011433	0.011331	0.011500	0.011772	0.011331	0.011399
0.011297	0.011399	0.011365	0.011433	0.011365	0.011365	0.011365
0.011263	0.011297	0.011263	0.011365	0.011331	0.011366	0.011433
0.011365	0.011331	0.011399	0.011297	0.011433	0.011263	0.011297
0.011500	0.011433	0.011670	0.011264	0.011331	0.011568	0.011331
0.011322	0.011663	0.011551	0.011216	0.011247	0.011209	0.011414
0.012085	0.011518	0.011266	0.011568	0.011568	0.011365	0.011433
0.011399	0.011365	0.011433	0.011331	0.011433	0.011365	0.011365
0.011331	0.011433	0.011636	0.011500	0.011399	0.011365	0.011297
0.011263	0.011263	0.011331	0.011297	0.011500	0.011467	0.011399
0.011500	0.011568	0.011297	0.011297	0.011331	0.011331	0.011333
0.011401	0.011331	0.011331	0.011204	0.011277	0.011236	0.011331
0.000000	0.011266	0.011318	0.011302	0.011231	0.011266	0.011298
0.011564	0.011336	0.011370	0.011263	0.011268	0.011399	0.011365
0.011433	0.011365	0.011365	0.011467	0.011399	0.011772	0.011534
0.011602	0.011467	0.011331	0.011433	0.011230	0.011467	0.011263
0.011297	0.011331	0.011297	0.011263	0.011297	0.011263	0.011365
0.011331	0.011433	0.011365	0.011263	0.011366	0.011467	0.011469
0.011467	0.011365	0.011263	0.011196	0.011331	0.011263	0.011196
0.011300	0.011467	0.011263	0.011467	0.011230	0.011331	0.011297
0.011399	0.011297	0.011331	0.011297	0.011331	0.011196	0.011331
0.011365	0.011297	0.011365	0.011297	0.011331	0.011365	0.011602
0.011670	0.011602	0.011670	0.011468	0.011670	0.011331	0.011297
0.011333	0.011631	0.011384	0.011233	0.011676	0.011198	0.011298
0.011677	0.011608	0.011500	0.011704	0.011405	0.011568	0.011535
0.011467	0.011636	0.011491	0.011309	0.011397	0.011365	0.011436
0.011534	0.011434	0.011538	0.011641	0.011568	0.011636	0.011744
0.011470	0.011642	0.011572	0.011299	0.011772	0.011230	0.011434
0.011399	0.011331	0.011230	0.011230	0.011297	0.011297	0.011365
0.011297	0.011365	0.011263	0.011365	0.011297	0.011297	0.011365
0.011297	0.011704	0.011602	0.011636	0.011196	0.011297	0.011331
0.011331	0.011297	0.011365	0.011399	0.011263	0.011263	0.011263
0.011772	0.011399	0.011433	0.011230	0.011331	0.011501	0.011263
0.011263	0.011297	0.011297	0.011467	0.011263	0.011399	0.011534
0.011467	0.011297	0.011297	0.011230	0.011230	0.011399	0.011331
0.011230	0.011331	0.011196	0.011230	0.011162	0.011501	0.011263
0.011230	0.011162	0.011298	0.011196	0.011500	0.011196	0.011365
0.011440	0.011263	0.011534	0.011399	0.011297	0.011297	0.011399
0.011263	0.011602	0.011196	0.011433	0.011263	0.011263	0.011196
0.011433	0.011636	0.011331	0.011399	0.011230	0.011602	0.011263
0.011263	0.011263	0.011297	0.011399	0.011297	0.011399	0.011467
0.011230	0.011433	0.011331	0.011433	0.011399	0.011263	0.011230
0.011263	0.011263	0.011331	0.011263	0.011230	0.011399	0.011263
0.011297	0.011297	0.011433	0.011467	0.011365	0.011297	0.011297
0.011365	0.011263	0.011399	0.011263	0.011399	0.011500	0.011704
0.011297	0.011670	0.011304	0.017879	0.011126	0.011427	0.011622
0.011583	0.011627	0.011217	0.011350	0.011719	0.011651	0.011364
0.011535	0.011549	0.011177	0.011270	0.011255	0.011307	0.011275
0.011529	0.011470	0.011468	0.011259	0.011433	0.011467	0.011470
0.011337	0.011433	0.011433	0.011399	0.011365	0.011365	0.011331
0.011365	0.011297	0.011331	0.011365	0.011331	0.011263	0.011297
0.011297	0.011365	0.011263	0.011365	0.011399	0.011230	0.011331
0.011365	0.011331	0.011602	0.011602	0.011399	0.011433	0.011467
0.011230	0.011230	0.011263	0.011365	0.011230	0.011467	0.011467
0.011500	0.011500	0.011433	0.011534	0.011670	0.011536	0.011365
0.011365	0.011433	0.011467	0.011297	0.011365	0.011331	0.011365
0.011433	0.011433	0.011467	0.011433	0.011332	0.011534	0.011500

0.011368	0.011263	0.011365	0.011467	0.011331	0.011501	0.011534
0.011704	0.011405	0.011568	0.011535	0.011467	0.011636	0.011264
0.011434	0.011331	0.011297	0.011297	0.011365	0.011263	0.011365
0.011399	0.011230	0.011331	0.011365	0.011331	0.011602	0.011602
0.011399	0.011433	0.011500	0.011433	0.012078	0.011493	0.011415
0.011273	0.011445	0.011556	0.011445	0.011519	0.011478	0.011440
0.012030	0.011339	0.011475	0.011409	0.011685	0.011541	0.011643
0.012054	0.011516	0.011367	0.011365	0.011365	0.011399	0.011333
0.011230	0.011263	0.011399	0.011365	0.011367	0.011500	0.011468
0.011331	0.011263	0.011297	0.011331	0.011230	0.011297	0.011196
0.011263	0.011297	0.011263	0.011230	0.011331	0.011263	0.011297
0.011297	0.011230	0.011297	0.011331	0.011230	0.011433	0.011331
0.011230	0.011297	0.011162	0.011297	0.011297	0.011670	0.011331
0.011376	0.011297	0.011534	0.011539	0.011399	0.011399	0.011399
0.011399	0.011331	0.011297	0.011331	0.011433	0.011433	0.011365
0.011365	0.011501	0.011636	0.011399	0.011602	0.011602	0.011467
0.011297	0.011365	0.011399	0.011772	0.011365	0.011399	0.011365
0.011399	0.011331	0.011331	0.011636	0.011568	0.011602	0.011331
0.011433	0.011501	0.011365	0.011433	0.011365	0.011263	0.011568
0.011365	0.011331	0.011331	0.011501	0.011331	0.011433	0.011670
0.011297	0.011711	0.011704	0.011507	0.011229	0.011437	0.011318
0.011225	0.011761	0.011273	0.011417	0.011304	0.011315	0.011326
0.011468	0.011126	0.011283	0.000000	0.011222	0.011261	0.011387
0.011402	0.011338	0.011415	0.011309	0.011366	0.011500	0.011568
0.011534	0.011433	0.011503	0.011539	0.011641	0.011702	0.011331
0.011602	0.011467	0.011331	0.011433	0.011230	0.011467	0.011263
0.011297	0.011331	0.011297	0.011263	0.011297	0.011263	0.011365
0.011340	0.011340	0.011334	0.011368	0.011505	0.011470	0.011470
0.011469	0.011399	0.011208	0.011204	0.011331	0.011501	0.011501
0.011297	0.011365	0.011602	0.011570	0.011500	0.011534	0.011467
0.011399	0.011467	0.011467	0.011365	0.011907	0.011501	0.011602
0.011636	0.011635	0.011336	0.011409	0.011297	0.011500	0.011467
0.011501	0.011500	0.011534	0.011534	0.011652	0.011670	0.011305
0.011197	0.011200	0.011300	0.011266	0.011336	0.011211	0.011505
0.011336	0.011456	0.011203	0.011360	0.011331	0.011402	0.011297
0.011263	0.011263	0.011331	0.011297	0.011500	0.011467	0.011399
0.011500	0.011568	0.011297	0.011297	0.011331	0.011331	0.011333
0.011401	0.011331	0.011331	0.011366	0.011500	0.011568	0.011534
0.011433	0.011503	0.011539	0.011641	0.011702	0.011331	0.011534
0.011467	0.011467	0.011299	0.011481	0.011410	0.011438	0.011307
0.011477	0.011705	0.011443	0.011369	0.011263	0.011467	0.011331
0.011433	0.011365	0.011263	0.011366	0.011467	0.011469	0.011365
0.011263	0.011196	0.011331	0.011263	0.011196	0.011300	0.011467
0.011365	0.011297	0.011331	0.011365	0.011331	0.011263	0.011467
0.011331	0.011365	0.011365	0.011297	0.011162	0.011501	0.011263
0.011297	0.011670	0.011297	0.011297	0.011297	0.011636	0.011274
0.011678	0.011273	0.011311	0.011503	0.011589	0.011494	0.011227
0.011218	0.011467	0.011568	0.011676	0.011535	0.011674	0.011806
0.011704	0.011742	0.011704	0.011955	0.011636	0.012552	0.011705
0.012383	0.012215	0.012352	0.012591	0.011670	0.011365	0.011670
0.012112	0.012047	0.011636	0.011535	0.011602	0.011602	0.011572
0.011467	0.011471	0.011534	0.011501	0.011874	0.011941	0.011941
0.011841	0.011942	0.011977	0.011810	0.012112	0.012118	0.012180
0.012180	0.012214	0.012161	0.012180	0.012182	0.011399	0.011704
0.011738	0.012281	0.011778	0.012009	0.012486	0.011539	0.011433
0.011399	0.011399	0.011874	0.011924	0.012010	0.011738	0.011400
S148	0.011610	0.011610	0.011713	0.011679	0.011679	0.011748
0.011582	0.011477	0.011679	0.011198	0.011266	0.011232	0.011198
0.011232	0.011404	0.011198	0.011232	0.011301	0.011266	0.011232
0.011198	0.011232	0.011335	0.011198	0.011232	0.011232	0.011547

0.011473	0.011232	0.011129	0.011369	0.011301	0.011266	0.011301
0.011266	0.011370	0.011266	0.011266	0.011301	0.011266	0.011438
0.011198	0.011266	0.011301	0.011232	0.011335	0.011369	0.011301
0.011266	0.011438	0.011335	0.011576	0.011266	0.011541	0.011266
0.011232	0.011335	0.011404	0.011541	0.011610	0.011335	0.011438
0.011301	0.011370	0.011266	0.011507	0.011713	0.011266	0.011335
0.011232	0.011335	0.011301	0.011370	0.011301	0.011301	0.011301
0.011198	0.011232	0.011198	0.011266	0.011266	0.011267	0.011369
0.011301	0.011266	0.011335	0.011232	0.011369	0.011198	0.011232
0.011404	0.011369	0.011610	0.011404	0.011266	0.011507	0.011266
0.011434	0.011625	0.011475	0.011177	0.011172	0.011283	0.011375
0.012011	0.011467	0.011184	0.011507	0.011507	0.011301	0.011335
0.011335	0.011301	0.011369	0.011266	0.011370	0.011301	0.011301
0.011266	0.011369	0.011576	0.011438	0.011335	0.011301	0.011232
0.011198	0.011198	0.011266	0.011232	0.011438	0.011404	0.011335
0.011438	0.011507	0.011232	0.011232	0.011266	0.011266	0.011267
0.011336	0.011266	0.011266	0.011132	0.011205	0.011167	0.011266
0.011266	0.000000	0.011246	0.011235	0.011164	0.011199	0.011233
0.011496	0.011270	0.011304	0.011198	0.011200	0.011335	0.011301
0.011370	0.011301	0.011301	0.011404	0.011335	0.011713	0.011473
0.011541	0.011404	0.011266	0.011370	0.011163	0.011404	0.011198
0.011232	0.011266	0.011232	0.011198	0.011198	0.011198	0.011301
0.011232	0.011369	0.011301	0.011198	0.011302	0.011404	0.011372
0.011404	0.011301	0.011198	0.011129	0.011266	0.011198	0.011129
0.011234	0.011404	0.011198	0.011404	0.011163	0.011266	0.011232
0.011335	0.011232	0.011266	0.011232	0.011266	0.011129	0.011266
0.011301	0.011232	0.011301	0.011232	0.011266	0.011301	0.011541
0.011610	0.011541	0.011610	0.011405	0.011610	0.011266	0.011232
0.011268	0.011558	0.011390	0.011166	0.011616	0.011130	0.011221
0.011616	0.011547	0.011438	0.011645	0.011340	0.011507	0.011473
0.011404	0.011576	0.011424	0.011272	0.011317	0.011301	0.011372
0.011473	0.011371	0.011476	0.011580	0.011438	0.011576	0.011685
0.011407	0.011581	0.011476	0.011441	0.011714	0.011163	0.011370
0.011301	0.011266	0.011163	0.011163	0.011232	0.011232	0.011301
0.011232	0.011301	0.011198	0.011301	0.011232	0.011232	0.011301
0.011232	0.011644	0.011541	0.011576	0.011129	0.011232	0.011266
0.011266	0.011232	0.011301	0.011335	0.011198	0.011198	0.011198
0.011713	0.011335	0.011370	0.011163	0.011266	0.011438	0.011163
0.011163	0.011232	0.011232	0.011404	0.011198	0.011335	0.011473
0.011404	0.011232	0.011232	0.011163	0.011163	0.011266	0.011266
0.011163	0.011266	0.011129	0.011163	0.011301	0.011438	0.011198
0.011163	0.011301	0.011232	0.011129	0.011438	0.011129	0.011301
0.011376	0.011198	0.011473	0.011335	0.011370	0.011232	0.011335
0.011198	0.011541	0.011129	0.011370	0.011198	0.011198	0.011129
0.011335	0.011576	0.011266	0.011335	0.011163	0.011541	0.011198
0.011198	0.011198	0.011232	0.011335	0.011232	0.011335	0.011404
0.011370	0.011369	0.011266	0.011370	0.011335	0.011198	0.011163
0.011198	0.011198	0.011266	0.011198	0.011163	0.011335	0.011198
0.011232	0.011232	0.011335	0.011404	0.011301	0.011232	0.011232
0.011301	0.011198	0.011335	0.011198	0.011335	0.011438	0.011644
0.011232	0.011610	0.011234	0.017802	0.011257	0.011427	0.011552
0.011517	0.011556	0.011322	0.011292	0.011719	0.011587	0.011364
0.011487	0.011548	0.011138	0.011228	0.011216	0.011307	0.011232
0.011482	0.011395	0.011394	0.011193	0.011370	0.011404	0.011406
0.011271	0.011369	0.011369	0.011335	0.011301	0.011301	0.011267
0.011301	0.011232	0.011266	0.011301	0.011266	0.011198	0.011232
0.011232	0.011301	0.011198	0.011301	0.011335	0.011369	0.011266
0.011301	0.011266	0.011507	0.011507	0.011335	0.011370	0.011404
0.011163	0.011163	0.011198	0.011301	0.011163	0.011404	0.011404
0.011438	0.011438	0.011370	0.011473	0.011610	0.011474	0.011301

0.011301	0.011369	0.011404	0.011232	0.011266	0.011232	0.011301
0.011370	0.011369	0.011404	0.011370	0.011267	0.011473	0.011438
0.011303	0.011163	0.011301	0.011404	0.011266	0.011438	0.011473
0.011645	0.011340	0.011507	0.011473	0.011404	0.011576	0.011198
0.011370	0.011267	0.011232	0.011232	0.011301	0.011198	0.011301
0.011335	0.011369	0.011266	0.011301	0.011266	0.011507	0.011507
0.011335	0.011370	0.011404	0.011369	0.012024	0.011426	0.011312
0.011418	0.011380	0.011492	0.011344	0.011454	0.011413	0.011376
0.011975	0.011273	0.011411	0.011343	0.011624	0.011478	0.011582
0.011999	0.011446	0.011301	0.011301	0.011301	0.011335	0.011267
0.011163	0.011198	0.011335	0.011301	0.011301	0.011438	0.011405
0.011266	0.011198	0.011232	0.011266	0.011163	0.011232	0.011129
0.011266	0.011301	0.011198	0.011163	0.011266	0.011198	0.011232
0.011232	0.011163	0.011232	0.011266	0.011163	0.011370	0.011404
0.011266	0.011370	0.011301	0.011266	0.011232	0.011610	0.011266
0.011310	0.011232	0.011473	0.011477	0.011335	0.011335	0.011335
0.011335	0.011266	0.011232	0.011266	0.011370	0.011370	0.011301
0.011301	0.011438	0.011576	0.011335	0.011473	0.011473	0.011404
0.011232	0.011301	0.011335	0.011713	0.011301	0.011335	0.011301
0.011335	0.011266	0.011266	0.011576	0.011473	0.011541	0.011266
0.011370	0.011438	0.011301	0.011370	0.011301	0.011198	0.011507
0.011301	0.011232	0.011266	0.011404	0.011266	0.011369	0.011610
0.011232	0.011651	0.011644	0.011467	0.011229	0.011371	0.011267
0.011225	0.011683	0.011202	0.011417	0.011255	0.011366	0.011379
0.011468	0.011192	0.011339	0.011192	0.011222	0.011395	0.011347
0.011364	0.011259	0.011344	0.011238	0.011302	0.011438	0.011507
0.011473	0.011370	0.011475	0.011476	0.011581	0.011639	0.011232
0.011541	0.011404	0.011266	0.011370	0.011163	0.011404	0.011198
0.011232	0.011266	0.011232	0.011198	0.011198	0.011198	0.011301
0.011272	0.011273	0.011268	0.011303	0.011442	0.011405	0.011406
0.011406	0.011335	0.011135	0.011133	0.011266	0.011438	0.011439
0.011232	0.011301	0.011541	0.011473	0.011438	0.011473	0.011404
0.011335	0.011335	0.011404	0.011266	0.011851	0.011438	0.011542
0.011576	0.011607	0.011270	0.011344	0.011232	0.011438	0.011404
0.011439	0.011404	0.011438	0.011438	0.011582	0.011610	0.011238
0.011130	0.011131	0.011234	0.011199	0.011270	0.011137	0.011443
0.011270	0.011371	0.011108	0.011289	0.011266	0.011338	0.011232
0.011198	0.011198	0.011266	0.011232	0.011438	0.011404	0.011335
0.011438	0.011507	0.011232	0.011232	0.011266	0.011266	0.011267
0.011336	0.011266	0.011266	0.011302	0.011438	0.011507	0.011473
0.011370	0.011475	0.011476	0.011581	0.011639	0.011232	0.011473
0.011404	0.011404	0.011233	0.011416	0.011344	0.011374	0.011204
0.011413	0.011622	0.011364	0.011304	0.011198	0.011404	0.011232
0.011369	0.011301	0.011198	0.011302	0.011404	0.011372	0.011301
0.011198	0.011129	0.011266	0.011198	0.011129	0.011234	0.011404
0.011301	0.011232	0.011266	0.011301	0.011266	0.011198	0.011404
0.011266	0.011301	0.011301	0.011232	0.011301	0.011507	0.011198
0.011232	0.011610	0.011232	0.011232	0.011232	0.011576	0.011189
0.011678	0.011188	0.011261	0.011422	0.011508	0.011422	0.011144
0.011218	0.011404	0.011507	0.011616	0.011473	0.011614	0.011748
0.011645	0.011683	0.011644	0.011899	0.011576	0.012505	0.011645
0.012333	0.012163	0.012302	0.012544	0.011610	0.011301	0.011610
0.012024	0.011992	0.011576	0.011473	0.011541	0.011541	0.011510
0.011404	0.011407	0.011473	0.011438	0.011748	0.011816	0.011782
0.011679	0.011782	0.011853	0.011682	0.012059	0.012064	0.012127
0.012127	0.012162	0.012104	0.012127	0.012129	0.011335	0.011610
0.011679	0.012195	0.011719	0.011920	0.012438	0.011477	0.011369
0.011335	0.011335	0.011748	0.011814	0.011940	0.011679	0.011336
S149	0.011688	0.011688	0.011761	0.011724	0.011724	0.011761
0.011658	0.011546	0.011724	0.011245	0.011282	0.011282	0.011245

0.011282	0.011466	0.011245	0.011245	0.011355	0.011318	0.011245
0.011245	0.011282	0.011392	0.011245	0.011282	0.011208	0.011621
0.011503	0.000000	0.011171	0.011429	0.011318	0.011318	0.011208
0.011318	0.011429	0.011318	0.011318	0.011355	0.011318	0.011503
0.011208	0.011318	0.011355	0.011282	0.011392	0.011429	0.011355
0.011318	0.011503	0.011282	0.011651	0.011318	0.011577	0.011318
0.011282	0.011392	0.011429	0.011577	0.011687	0.011355	0.011503
0.011355	0.011429	0.011282	0.011540	0.011688	0.011318	0.011392
0.011282	0.011355	0.011319	0.011392	0.011318	0.011355	0.011355
0.011245	0.011245	0.011208	0.011282	0.011318	0.011318	0.011429
0.011318	0.011318	0.011392	0.011245	0.011429	0.011245	0.011245
0.011503	0.011392	0.011614	0.011429	0.011318	0.011577	0.011318
0.011496	0.011699	0.011524	0.011222	0.011213	0.011258	0.011428
0.011404	0.011519	0.011227	0.011540	0.011429	0.011318	0.011429
0.011392	0.011318	0.011429	0.011318	0.011392	0.011318	0.011355
0.011318	0.011392	0.011577	0.011503	0.011392	0.011355	0.011282
0.011245	0.011245	0.011318	0.011245	0.011503	0.011466	0.011392
0.011503	0.011577	0.011245	0.011245	0.011282	0.011282	0.011283
0.011357	0.011318	0.011318	0.011175	0.011175	0.000000	0.011318
0.011318	0.011246	0.000000	0.000000	0.011209	0.011247	0.011283
0.011482	0.011285	0.011319	0.011245	0.011245	0.011392	0.011355
0.011429	0.011355	0.011355	0.011429	0.011392	0.011798	0.011503
0.011614	0.011466	0.011318	0.011429	0.011208	0.011429	0.011208
0.011282	0.011282	0.011245	0.011245	0.011245	0.011245	0.011355
0.011282	0.011392	0.011282	0.011245	0.011356	0.011466	0.011432
0.011429	0.011355	0.011208	0.011171	0.011318	0.011208	0.011171
0.011284	0.011429	0.011245	0.011429	0.011171	0.011282	0.011245
0.011392	0.011282	0.011318	0.011245	0.011282	0.011171	0.011282
0.011318	0.011282	0.011355	0.011282	0.011318	0.011355	0.011429
0.011687	0.011540	0.011651	0.011466	0.011688	0.011318	0.011282
0.011319	0.011635	0.011363	0.011210	0.011695	0.011173	0.011317
0.011695	0.011544	0.011429	0.011651	0.011398	0.011540	0.011540
0.011466	0.011540	0.011414	0.011153	0.011243	0.011355	0.011358
0.011540	0.011431	0.011469	0.011618	0.011503	0.011614	0.011768
0.011431	0.011620	0.011581	0.011506	0.011725	0.011208	0.011393
0.011355	0.011282	0.011171	0.011171	0.011282	0.011282	0.011355
0.011282	0.011355	0.011245	0.011318	0.011282	0.011282	0.011282
0.011282	0.011724	0.011577	0.011614	0.011171	0.011282	0.011319
0.011318	0.011245	0.011355	0.011392	0.011245	0.011245	0.011245
0.011798	0.011355	0.011429	0.011208	0.011282	0.011503	0.011208
0.011208	0.011245	0.011282	0.011429	0.011245	0.011392	0.011466
0.011466	0.011282	0.011282	0.011208	0.011208	0.011355	0.011318
0.011208	0.011318	0.011171	0.011171	0.011355	0.011503	0.011245
0.011208	0.011355	0.011282	0.011171	0.011503	0.011171	0.011355
0.011437	0.011245	0.011540	0.011355	0.011429	0.011282	0.011392
0.011245	0.011577	0.011171	0.011392	0.011245	0.011245	0.011171
0.011392	0.011577	0.011282	0.011392	0.011208	0.011577	0.011245
0.011245	0.011245	0.011245	0.011392	0.011282	0.011392	0.011429
0.011429	0.011355	0.011318	0.011392	0.011392	0.011245	0.011208
0.011245	0.011245	0.011282	0.011208	0.011208	0.011355	0.011245
0.011245	0.011245	0.011392	0.011466	0.011318	0.011282	0.011282
0.011355	0.011245	0.011392	0.011245	0.011392	0.011466	0.011724
0.011282	0.011651	0.011283	0.018171	0.011261	0.011452	0.011624
0.011584	0.011624	0.011330	0.011296	0.011777	0.011657	0.011432
0.011500	0.011612	0.011182	0.011282	0.011265	0.011370	0.011242
0.011488	0.011442	0.011446	0.011198	0.011355	0.011466	0.011469
0.011249	0.011392	0.011392	0.011355	0.011319	0.011319	0.011319
0.011355	0.011282	0.011245	0.011355	0.011282	0.011245	0.011282
0.011282	0.011318	0.011208	0.011319	0.011392	0.011429	0.011318
0.011355	0.011318	0.011577	0.011577	0.011392	0.011430	0.011466

0.011171	0.011208	0.011245	0.011355	0.011208	0.011466	0.011466
0.011429	0.011429	0.011392	0.011466	0.011687	0.011467	0.011355
0.011318	0.011429	0.011466	0.011282	0.011282	0.011245	0.011355
0.011392	0.011392	0.011429	0.011355	0.011319	0.011466	0.011466
0.011358	0.011208	0.011318	0.011466	0.011319	0.011503	0.011503
0.011651	0.011398	0.011540	0.011540	0.011466	0.011540	0.011245
0.011430	0.011319	0.011282	0.011282	0.011318	0.011208	0.011319
0.011392	0.011429	0.011318	0.011355	0.011318	0.011577	0.011577
0.011392	0.011430	0.011466	0.011429	0.012095	0.011455	0.011332
0.011486	0.011404	0.011525	0.011404	0.011446	0.011478	0.011398
0.012081	0.011327	0.011475	0.011403	0.011667	0.011509	0.011621
0.012069	0.011478	0.011320	0.011318	0.011318	0.011392	0.011283
0.011208	0.011208	0.011355	0.011318	0.011320	0.011466	0.011466
0.011282	0.011245	0.011282	0.011282	0.011208	0.011245	0.011171
0.011318	0.011355	0.011245	0.011208	0.011318	0.011245	0.011282
0.011282	0.011208	0.011282	0.011319	0.011208	0.011429	0.011466
0.011355	0.011429	0.011355	0.011355	0.011208	0.011687	0.011318
0.011329	0.011245	0.011503	0.011508	0.011392	0.011318	0.011355
0.011392	0.011319	0.011282	0.011319	0.011429	0.011429	0.011355
0.011355	0.011466	0.011651	0.011355	0.011503	0.011503	0.011466
0.011245	0.011318	0.011392	0.011725	0.011356	0.011392	0.011318
0.011355	0.011282	0.011282	0.011577	0.011503	0.011540	0.011318
0.011429	0.011503	0.011318	0.011429	0.011318	0.011245	0.011577
0.011355	0.011282	0.011282	0.011466	0.011318	0.011429	0.011688
0.011282	0.011733	0.011724	0.011519	0.011229	0.011392	0.011270
0.011235	0.011739	0.011246	0.011469	0.011308	0.011423	0.011439
0.011475	0.011262	0.011343	0.011192	0.011276	0.011402	0.011401
0.011423	0.011307	0.011402	0.011288	0.011357	0.011466	0.011540
0.011503	0.011430	0.011543	0.011470	0.011618	0.011682	0.011282
0.011614	0.011466	0.011318	0.011429	0.011208	0.011429	0.011208
0.011282	0.011282	0.011245	0.011245	0.011245	0.011245	0.011355
0.011286	0.011325	0.011319	0.011321	0.011508	0.011430	0.011469
0.011468	0.011318	0.011178	0.011175	0.011318	0.011503	0.011430
0.011282	0.011355	0.011614	0.011540	0.011466	0.011540	0.011466
0.011355	0.011466	0.011466	0.011355	0.011946	0.011429	0.011540
0.011651	0.011611	0.011285	0.011364	0.011245	0.011503	0.011466
0.011467	0.011466	0.011466	0.011466	0.011622	0.011688	0.011251
0.011172	0.011136	0.000000	0.011247	0.011286	0.011142	0.011508
0.011324	0.011445	0.011113	0.011307	0.011318	0.011396	0.011282
0.011245	0.011245	0.011318	0.011245	0.011503	0.011466	0.011392
0.011503	0.011577	0.011245	0.011245	0.011282	0.011282	0.011283
0.011357	0.011318	0.011318	0.011357	0.011466	0.011540	0.011503
0.011430	0.011543	0.011470	0.011618	0.011682	0.011282	0.011503
0.011466	0.011466	0.011246	0.011479	0.011365	0.011397	0.011291
0.011325	0.011562	0.011483	0.011359	0.011245	0.011429	0.011282
0.011392	0.011282	0.011245	0.011356	0.011466	0.011432	0.011355
0.011208	0.011171	0.011318	0.011208	0.011171	0.011284	0.011429
0.011355	0.011282	0.011245	0.011355	0.011282	0.011245	0.011429
0.011318	0.011355	0.011355	0.011282	0.011355	0.011540	0.011208
0.011282	0.011687	0.011282	0.011282	0.011282	0.011651	0.011191
0.011689	0.011235	0.011261	0.011474	0.011562	0.011477	0.011188
0.011218	0.011466	0.011577	0.011694	0.011540	0.011688	0.011835
0.011725	0.011766	0.011687	0.011990	0.011614	0.012538	0.011725
0.012353	0.012242	0.012393	0.012614	0.011577	0.011318	0.011688
0.012095	0.011873	0.011651	0.011540	0.011577	0.011577	0.011580
0.011466	0.011468	0.011503	0.011503	0.011836	0.011909	0.011909
0.011761	0.011909	0.011872	0.011799	0.012132	0.012139	0.012243
0.012206	0.012243	0.012169	0.012206	0.012132	0.011392	0.011688
0.011688	0.012205	0.011804	0.011909	0.011761	0.011506	0.011429
0.011392	0.011392	0.011835	0.011881	0.011970	0.011687	0.011392

S150	0.011647	0.011647	0.011750	0.011716	0.011716	0.011750
0.011619	0.011514	0.011716	0.011233	0.011302	0.011267	0.011233
0.011267	0.011440	0.011233	0.011267	0.011336	0.011302	0.011267
0.011233	0.011267	0.011371	0.011233	0.011267	0.011267	0.011584
0.011509	0.000000	0.011164	0.011405	0.011336	0.011302	0.011267
0.011302	0.011405	0.011302	0.011302	0.011336	0.011302	0.011474
0.011233	0.011302	0.011336	0.011267	0.011371	0.011405	0.011336
0.011302	0.011474	0.011371	0.011612	0.011302	0.011578	0.011302
0.011267	0.011371	0.011440	0.011578	0.011647	0.011371	0.011474
0.011336	0.011405	0.011302	0.011509	0.011716	0.011302	0.011371
0.011267	0.011371	0.011336	0.011405	0.011336	0.011336	0.011336
0.011233	0.011267	0.011198	0.011302	0.011302	0.011337	0.011405
0.011336	0.011302	0.011371	0.011267	0.011405	0.011233	0.011267
0.011474	0.011405	0.011612	0.011405	0.011302	0.011543	0.011302
0.011471	0.011663	0.011513	0.011216	0.011210	0.011283	0.011415
0.012048	0.011518	0.011225	0.011543	0.011543	0.011336	0.011405
0.011371	0.011336	0.011405	0.011302	0.011405	0.011336	0.011336
0.011302	0.011405	0.011612	0.011474	0.011371	0.011336	0.011267
0.011233	0.011233	0.011302	0.011267	0.011474	0.011440	0.011371
0.011474	0.011543	0.011267	0.011267	0.011302	0.011302	0.011303
0.011373	0.011302	0.011302	0.011168	0.011207	0.000000	0.011302
0.011302	0.011235	0.000000	0.000000	0.011200	0.011235	0.011268
0.011528	0.011306	0.011340	0.011233	0.011233	0.011371	0.011336
0.011405	0.011336	0.011336	0.011440	0.011371	0.011750	0.011509
0.011578	0.011440	0.011302	0.011405	0.011198	0.011440	0.011233
0.011267	0.011302	0.011267	0.011233	0.011267	0.011233	0.011336
0.011267	0.011405	0.011336	0.011233	0.011337	0.011440	0.011442
0.011440	0.011336	0.011233	0.011164	0.011302	0.011233	0.011164
0.011270	0.011440	0.011233	0.011440	0.011198	0.011267	0.011267
0.011371	0.011267	0.011302	0.011267	0.011302	0.011164	0.011302
0.011302	0.011267	0.011336	0.011267	0.011302	0.011336	0.011578
0.011647	0.011509	0.011647	0.011441	0.011647	0.011302	0.011267
0.011303	0.011602	0.011390	0.011201	0.011653	0.011165	0.011302
0.011653	0.011583	0.011474	0.011681	0.011376	0.011543	0.011509
0.011440	0.011612	0.011426	0.011230	0.011317	0.011336	0.011373
0.011509	0.011407	0.011512	0.011617	0.011543	0.011612	0.011722
0.011441	0.011618	0.011547	0.011477	0.011751	0.011198	0.011406
0.011371	0.011302	0.011198	0.011164	0.011267	0.011267	0.011336
0.011267	0.011336	0.011233	0.011336	0.011267	0.011267	0.011336
0.011267	0.011681	0.011578	0.011612	0.011164	0.011267	0.011302
0.011302	0.011267	0.011336	0.011371	0.011233	0.011233	0.011233
0.011750	0.011371	0.011405	0.011198	0.011302	0.011474	0.011233
0.011198	0.011267	0.011267	0.011440	0.011233	0.011371	0.011509
0.011440	0.011267	0.011267	0.011198	0.011198	0.011336	0.011302
0.011198	0.011302	0.011164	0.011198	0.011336	0.011474	0.011233
0.011198	0.011336	0.011267	0.011164	0.011474	0.011164	0.011336
0.011412	0.011233	0.011509	0.011336	0.011405	0.011267	0.011371
0.011233	0.011578	0.011164	0.011405	0.011233	0.011233	0.011164
0.011405	0.011613	0.011302	0.011371	0.011198	0.011578	0.011233
0.011233	0.011233	0.011267	0.011371	0.011267	0.011371	0.011440
0.011405	0.011405	0.011302	0.011405	0.011371	0.011233	0.011198
0.011233	0.011233	0.011302	0.011233	0.011198	0.011371	0.011233
0.011267	0.011267	0.011371	0.011440	0.011336	0.011267	0.011267
0.011336	0.011233	0.011371	0.011233	0.011371	0.011474	0.011681
0.011267	0.011647	0.011271	0.017841	0.011257	0.011427	0.011592
0.011552	0.011592	0.011322	0.011292	0.011774	0.011621	0.011425
0.011487	0.011610	0.011177	0.011270	0.011255	0.011356	0.011275
0.011483	0.011433	0.011431	0.011193	0.011405	0.011440	0.011442
0.011272	0.011405	0.011405	0.011371	0.011336	0.011336	0.011302
0.011336	0.011267	0.011302	0.011336	0.011267	0.011233	0.011267

0.011267	0.011336	0.011233	0.011336	0.011371	0.011405	0.011302
0.011336	0.011302	0.011578	0.011578	0.011371	0.011406	0.011440
0.011198	0.011198	0.011233	0.011336	0.011198	0.011440	0.011440
0.011474	0.011474	0.011405	0.011509	0.011647	0.011510	0.011336
0.011336	0.011405	0.011440	0.011267	0.011336	0.011267	0.011336
0.011405	0.011405	0.011440	0.011405	0.011302	0.011509	0.011474
0.011339	0.011233	0.011336	0.011440	0.011302	0.011474	0.011509
0.011681	0.011376	0.011543	0.011509	0.011440	0.011612	0.011233
0.011406	0.011302	0.011267	0.011267	0.011336	0.011233	0.011336
0.011371	0.011405	0.011302	0.011336	0.011302	0.011578	0.011578
0.011371	0.011406	0.011474	0.011405	0.012062	0.011464	0.011350
0.011458	0.011417	0.011494	0.011416	0.011492	0.011450	0.011411
0.012013	0.011309	0.011448	0.011380	0.011662	0.011480	0.011619
0.012038	0.011448	0.011338	0.011336	0.011336	0.011371	0.011303
0.011198	0.011233	0.011371	0.011336	0.011338	0.011474	0.011441
0.011302	0.011233	0.011267	0.011302	0.011198	0.011233	0.011164
0.011302	0.011336	0.011233	0.011198	0.011302	0.011233	0.011267
0.011267	0.011198	0.011267	0.011302	0.011198	0.011405	0.011440
0.011336	0.011405	0.011336	0.011336	0.011267	0.011647	0.011302
0.011311	0.011267	0.011509	0.011513	0.011371	0.011371	0.011371
0.011371	0.011302	0.011267	0.011302	0.011405	0.011405	0.011336
0.011336	0.011474	0.011612	0.011371	0.011543	0.011543	0.011440
0.011267	0.011336	0.011371	0.011716	0.011336	0.011371	0.011336
0.011371	0.011302	0.011302	0.011612	0.011543	0.011578	0.011302
0.011405	0.011474	0.011336	0.011405	0.011336	0.011233	0.011543
0.011336	0.011302	0.011302	0.011474	0.011302	0.011405	0.011647
0.011267	0.011688	0.011681	0.011507	0.011229	0.011405	0.011267
0.011225	0.011722	0.011237	0.011462	0.011304	0.011417	0.011432
0.011468	0.011258	0.011339	0.011192	0.011276	0.011395	0.011387
0.011402	0.011298	0.011382	0.011275	0.011337	0.011474	0.011543
0.011509	0.011406	0.011512	0.011513	0.011617	0.011641	0.011302
0.011578	0.011440	0.011302	0.011405	0.011198	0.011440	0.011233
0.011267	0.011302	0.011267	0.011233	0.011267	0.011233	0.011336
0.011306	0.011309	0.011302	0.011339	0.011478	0.011440	0.011442
0.011442	0.011371	0.011171	0.011168	0.011302	0.011474	0.011440
0.011267	0.011336	0.011578	0.011544	0.011474	0.011509	0.011440
0.011371	0.011440	0.011440	0.011336	0.011889	0.011474	0.011578
0.011612	0.011647	0.011306	0.011380	0.011267	0.011474	0.011440
0.011475	0.011440	0.011509	0.011509	0.011583	0.011647	0.011239
0.011165	0.011132	0.000000	0.011235	0.011271	0.011137	0.011479
0.011306	0.011421	0.011109	0.011290	0.011302	0.011374	0.011267
0.011233	0.011233	0.011302	0.011267	0.011474	0.011440	0.011371
0.011474	0.011543	0.011267	0.011267	0.011302	0.011302	0.011303
0.011373	0.011302	0.011302	0.011337	0.011474	0.011543	0.011509
0.011406	0.011512	0.011513	0.011617	0.011641	0.011302	0.011509
0.011440	0.011440	0.011268	0.011453	0.011345	0.011410	0.011276
0.011379	0.011579	0.011452	0.011340	0.011233	0.011440	0.011267
0.011405	0.011336	0.011233	0.011337	0.011440	0.011442	0.011336
0.011233	0.011164	0.011302	0.011233	0.011164	0.011270	0.011440
0.011336	0.011267	0.011302	0.011336	0.011267	0.011233	0.011440
0.011302	0.011336	0.011336	0.011267	0.011336	0.011543	0.011233
0.011267	0.011647	0.011267	0.011267	0.011267	0.011612	0.011189
0.011679	0.011230	0.011261	0.011463	0.011549	0.011458	0.011186
0.011218	0.011440	0.011543	0.011652	0.011509	0.011651	0.011785
0.011682	0.011720	0.011681	0.011937	0.011612	0.012476	0.011682
0.012373	0.012202	0.012341	0.012585	0.011647	0.011336	0.011647
0.012027	0.012030	0.011612	0.011509	0.011578	0.011578	0.011547
0.011440	0.011443	0.011509	0.011474	0.011820	0.011889	0.011854
0.011785	0.011889	0.011925	0.011754	0.012062	0.012103	0.012166
0.012166	0.012166	0.012137	0.012131	0.012064	0.011371	0.011647

0.011681	0.012269	0.011756	0.011923	0.012443	0.011513	0.011405
0.011371	0.011371	0.011854	0.011859	0.011983	0.011716	0.011372
S151	0.011570	0.011570	0.011706	0.011672	0.011672	0.011740
0.011576	0.011473	0.011672	0.011162	0.011230	0.011196	0.011162
0.011196	0.011366	0.011162	0.011196	0.011264	0.011230	0.011196
0.011162	0.011196	0.011298	0.011162	0.011196	0.011196	0.011541
0.011434	0.011196	0.011094	0.011332	0.011230	0.011230	0.011264
0.011196	0.011332	0.011230	0.011230	0.011264	0.011230	0.011400
0.011162	0.011230	0.011264	0.011196	0.011298	0.011332	0.011230
0.011230	0.011400	0.011298	0.011570	0.011230	0.011502	0.011230
0.011196	0.011298	0.011366	0.011502	0.011570	0.011298	0.011400
0.011264	0.011332	0.011230	0.011468	0.011706	0.011230	0.011298
0.011196	0.011298	0.011264	0.011332	0.011264	0.011264	0.011264
0.011162	0.011196	0.011162	0.011264	0.011230	0.011264	0.011332
0.011264	0.011196	0.011298	0.011196	0.011332	0.011162	0.011230
0.011400	0.011332	0.011570	0.011400	0.011230	0.011468	0.011230
0.011398	0.011627	0.011404	0.011139	0.011136	0.011246	0.011339
0.011985	0.011370	0.011144	0.011468	0.011468	0.011264	0.011366
0.011298	0.011264	0.011332	0.011230	0.011332	0.011264	0.011264
0.011230	0.011332	0.011536	0.011400	0.011298	0.011264	0.011196
0.011162	0.011162	0.011230	0.011196	0.011400	0.011366	0.011298
0.011400	0.011468	0.011196	0.011196	0.011230	0.011230	0.011231
0.011300	0.011230	0.011230	0.011096	0.011169	0.011130	0.011230
0.011231	0.011164	0.011209	0.011200	0.000000	0.011163	0.011196
0.011456	0.011233	0.011268	0.011162	0.011165	0.011298	0.011264
0.011332	0.011265	0.011264	0.011332	0.011298	0.011672	0.011434
0.011502	0.011366	0.011230	0.011332	0.011128	0.011366	0.011162
0.011230	0.011230	0.011196	0.011162	0.011196	0.011162	0.011264
0.011230	0.011332	0.011264	0.011162	0.011265	0.011366	0.011368
0.011366	0.011264	0.011162	0.011094	0.011230	0.011162	0.011094
0.011198	0.011366	0.011162	0.011366	0.011128	0.011230	0.011196
0.011298	0.011196	0.011230	0.011196	0.011230	0.011094	0.011230
0.011264	0.011196	0.011264	0.011196	0.011230	0.011264	0.011502
0.011604	0.011536	0.011604	0.011367	0.011604	0.011230	0.011196
0.011231	0.011509	0.011292	0.011130	0.011610	0.011095	0.011220
0.011610	0.011507	0.011366	0.011604	0.011268	0.011468	0.011434
0.011366	0.011536	0.011382	0.011184	0.011270	0.011264	0.011334
0.011434	0.011333	0.011471	0.011540	0.011468	0.011536	0.011643
0.011367	0.011540	0.011468	0.011400	0.011673	0.011128	0.011332
0.011298	0.011230	0.011128	0.011128	0.011196	0.011196	0.011264
0.011196	0.011264	0.011162	0.011264	0.011196	0.011196	0.011264
0.011196	0.011638	0.011536	0.011570	0.011094	0.011196	0.011230
0.011230	0.011196	0.011264	0.011298	0.011162	0.011162	0.011162
0.011706	0.011298	0.011332	0.011128	0.011230	0.011400	0.011162
0.011162	0.011230	0.011196	0.011366	0.011162	0.011298	0.011400
0.011366	0.011162	0.011196	0.011094	0.011128	0.011298	0.011230
0.011094	0.011196	0.011094	0.011128	0.011264	0.011400	0.011162
0.011128	0.011264	0.011196	0.011094	0.011434	0.011094	0.011264
0.011337	0.011162	0.011468	0.011298	0.011332	0.011196	0.011298
0.011162	0.011502	0.011094	0.011366	0.011162	0.011162	0.011094
0.011332	0.011536	0.011230	0.011298	0.011128	0.011536	0.011162
0.011162	0.011162	0.011196	0.011298	0.011196	0.011298	0.011366
0.011366	0.011332	0.011230	0.011332	0.011298	0.011162	0.011128
0.011162	0.011162	0.011230	0.011162	0.011128	0.011298	0.011162
0.011196	0.011196	0.011332	0.011366	0.011264	0.011196	0.011196
0.011264	0.011162	0.011298	0.011162	0.011298	0.011400	0.011638
0.011196	0.011604	0.011200	0.017789	0.011127	0.011305	0.011554
0.011515	0.011558	0.011220	0.011178	0.011619	0.011584	0.011246
0.011397	0.011427	0.011100	0.011145	0.011179	0.011211	0.011147
0.011393	0.011362	0.011360	0.011195	0.011332	0.011366	0.011368

0.011233	0.011298	0.011332	0.011298	0.011264	0.011264	0.011230
0.011264	0.011196	0.011230	0.011264	0.011230	0.011162	0.011196
0.011196	0.011264	0.011162	0.011264	0.011298	0.011332	0.011230
0.011264	0.011230	0.011570	0.011570	0.011298	0.011332	0.011366
0.011128	0.011128	0.011162	0.011264	0.011128	0.011366	0.011366
0.011400	0.011366	0.011332	0.011434	0.011604	0.011435	0.011264
0.011264	0.011298	0.011366	0.011196	0.011264	0.011230	0.011264
0.011332	0.011332	0.011366	0.011332	0.011231	0.011434	0.011400
0.011266	0.011162	0.011264	0.011332	0.011264	0.011400	0.011434
0.011604	0.011268	0.011468	0.011434	0.011366	0.011536	0.011162
0.011333	0.011230	0.011196	0.011196	0.011264	0.011162	0.011264
0.011298	0.011332	0.011230	0.011264	0.011230	0.011570	0.011570
0.011298	0.011332	0.011400	0.011332	0.011945	0.011383	0.011309
0.011377	0.011340	0.011448	0.011340	0.011447	0.011373	0.011337
0.011893	0.011234	0.011371	0.011303	0.011578	0.011437	0.011541
0.011919	0.011400	0.011266	0.011264	0.011264	0.011298	0.011231
0.011128	0.011162	0.011298	0.011264	0.011265	0.011400	0.011367
0.011230	0.011162	0.011196	0.011230	0.011128	0.011196	0.011094
0.011230	0.011264	0.011162	0.011128	0.011230	0.011162	0.011196
0.011196	0.011128	0.011196	0.011230	0.011128	0.011332	0.011366
0.011264	0.011332	0.011264	0.011264	0.011196	0.011570	0.011230
0.011270	0.011196	0.011434	0.011437	0.011298	0.011298	0.011298
0.011298	0.011230	0.011196	0.011230	0.011332	0.011332	0.011264
0.011264	0.011366	0.011570	0.011298	0.011502	0.011502	0.011366
0.011196	0.011264	0.011298	0.011706	0.011264	0.011298	0.011264
0.011298	0.011230	0.011230	0.011536	0.011434	0.011502	0.011230
0.011332	0.011400	0.011264	0.011332	0.011298	0.011162	0.011502
0.011264	0.011230	0.011230	0.011400	0.011230	0.011332	0.011570
0.011196	0.011645	0.011638	0.011431	0.011173	0.011335	0.011217
0.011115	0.011651	0.011167	0.011377	0.011209	0.011319	0.011276
0.011371	0.000000	0.011230	0.000000	0.011170	0.011265	0.011307
0.011327	0.011220	0.011309	0.011238	0.011265	0.011400	0.011468
0.011434	0.011332	0.011436	0.011403	0.011540	0.011597	0.011230
0.011502	0.011366	0.011230	0.011332	0.011128	0.011366	0.011162
0.011230	0.011230	0.011196	0.011162	0.011196	0.011162	0.011264
0.011236	0.011235	0.011233	0.011266	0.011403	0.011371	0.011370
0.011369	0.011298	0.011097	0.011096	0.011231	0.011403	0.011369
0.011196	0.011266	0.011502	0.011470	0.011400	0.011434	0.011366
0.011298	0.011366	0.011366	0.011264	0.011842	0.011434	0.011502
0.011536	0.011601	0.011233	0.011304	0.011196	0.011400	0.011366
0.011401	0.011400	0.011434	0.011434	0.011578	0.011570	0.011200
0.011095	0.011095	0.011198	0.011163	0.011266	0.011098	0.011438
0.011234	0.011368	0.000000	0.011248	0.011230	0.011300	0.011196
0.011162	0.011162	0.011230	0.011196	0.011400	0.011366	0.011298
0.011400	0.011468	0.011196	0.011196	0.011230	0.011230	0.011231
0.011300	0.011230	0.011230	0.011265	0.011400	0.011468	0.011434
0.011332	0.011436	0.011403	0.011540	0.011597	0.011230	0.011434
0.011366	0.011366	0.011197	0.011377	0.011306	0.011336	0.011166
0.011374	0.011522	0.011360	0.011267	0.011162	0.011366	0.011230
0.011332	0.011264	0.011162	0.011265	0.011366	0.011368	0.011264
0.011162	0.011094	0.011230	0.011162	0.011094	0.011198	0.011366
0.011264	0.011196	0.011230	0.011264	0.011230	0.011162	0.011366
0.011230	0.011264	0.011264	0.011196	0.011264	0.011502	0.011162
0.011196	0.011570	0.011196	0.011196	0.011196	0.011570	0.011104
0.011567	0.011145	0.011162	0.011383	0.011472	0.011387	0.000000
0.011114	0.011366	0.011468	0.011575	0.011435	0.011573	0.011706
0.011604	0.011641	0.011604	0.011854	0.011570	0.012455	0.011605
0.012285	0.012117	0.012219	0.012493	0.011570	0.011264	0.011570
0.011979	0.011947	0.011536	0.011434	0.011502	0.011502	0.011471
0.011366	0.011369	0.011434	0.011400	0.011774	0.011842	0.011842

0.011741	0.011842	0.011946	0.011710	0.011979	0.011984	0.012047
0.012047	0.012081	0.012026	0.012047	0.012049	0.011298	0.011604
0.011672	0.012114	0.011677	0.011910	0.012388	0.011438	0.011332
0.011298	0.011298	0.011774	0.011801	0.011878	0.011638	0.011299
S152	0.011608	0.011608	0.011711	0.011677	0.011677	0.011746
0.011580	0.011476	0.011677	0.011197	0.011266	0.011232	0.011197
0.011231	0.011403	0.011197	0.011231	0.011300	0.011266	0.011231
0.011197	0.011232	0.011334	0.011197	0.011232	0.011232	0.011545
0.011471	0.011232	0.011129	0.011368	0.011266	0.011266	0.011300
0.011232	0.011369	0.011266	0.011266	0.011300	0.011266	0.011437
0.011197	0.011266	0.011300	0.011231	0.011334	0.011368	0.011266
0.011266	0.011437	0.011334	0.011574	0.011266	0.011540	0.011266
0.011231	0.011334	0.011403	0.011540	0.011574	0.011334	0.011437
0.011266	0.011369	0.011266	0.011471	0.011711	0.011266	0.011334
0.011232	0.011334	0.011300	0.011368	0.011300	0.011300	0.011300
0.011197	0.011231	0.011197	0.011300	0.011266	0.011300	0.011368
0.011300	0.011231	0.011334	0.011231	0.011368	0.011197	0.011232
0.011437	0.011368	0.011608	0.011403	0.011266	0.011506	0.011266
0.011439	0.011632	0.011442	0.011179	0.011174	0.011286	0.011379
0.012023	0.011474	0.011186	0.011505	0.011506	0.011300	0.011369
0.011334	0.011300	0.011368	0.011266	0.011369	0.011300	0.011300
0.011266	0.011368	0.011574	0.011437	0.011334	0.011300	0.011232
0.011197	0.011197	0.011266	0.011231	0.011437	0.011403	0.011334
0.011437	0.011505	0.011231	0.011231	0.011266	0.011266	0.011267
0.011336	0.011266	0.011266	0.011132	0.011205	0.011165	0.011266
0.011266	0.011199	0.011247	0.011235	0.011163	0.000000	0.011232
0.011492	0.011267	0.011303	0.011197	0.011200	0.011334	0.011300
0.011369	0.011300	0.011300	0.011334	0.011334	0.011711	0.011471
0.011540	0.011403	0.011266	0.011369	0.011163	0.011403	0.011197
0.011232	0.011266	0.011231	0.011197	0.011231	0.011197	0.011300
0.011266	0.011368	0.011300	0.011197	0.011301	0.011403	0.011405
0.011403	0.011300	0.011197	0.011129	0.011266	0.011197	0.011129
0.011234	0.011403	0.011197	0.011403	0.011163	0.011266	0.011232
0.011334	0.011231	0.011266	0.011232	0.011266	0.011129	0.011266
0.011300	0.011231	0.011300	0.011231	0.011266	0.011300	0.011540
0.011608	0.011540	0.011608	0.011404	0.011608	0.011266	0.011231
0.011267	0.011554	0.011341	0.011165	0.011614	0.011130	0.011261
0.011615	0.011545	0.011403	0.011643	0.011304	0.011506	0.011471
0.011403	0.011574	0.011421	0.011269	0.011313	0.011300	0.011371
0.011471	0.011370	0.011474	0.011578	0.011506	0.011574	0.011682
0.011404	0.011578	0.011506	0.011437	0.011712	0.011163	0.011369
0.011334	0.011266	0.011163	0.011163	0.011232	0.011232	0.011300
0.011231	0.011300	0.011197	0.011300	0.011232	0.011231	0.011300
0.011232	0.011643	0.011540	0.011574	0.011129	0.011232	0.011266
0.011266	0.011231	0.011266	0.011334	0.011197	0.011197	0.011197
0.011711	0.011334	0.011368	0.011163	0.011266	0.011437	0.011197
0.011197	0.011231	0.011231	0.011403	0.011197	0.011334	0.011437
0.011403	0.011197	0.011231	0.011129	0.011163	0.011334	0.011266
0.011129	0.011231	0.011129	0.011163	0.011300	0.011437	0.011197
0.011163	0.011300	0.011231	0.011129	0.011437	0.000000	0.011300
0.011374	0.011197	0.011471	0.011334	0.011369	0.011232	0.011334
0.011197	0.011540	0.011129	0.011369	0.011197	0.011197	0.011129
0.011368	0.011574	0.011266	0.011334	0.011163	0.011540	0.011197
0.011197	0.011197	0.011231	0.011334	0.011231	0.011334	0.011403
0.011369	0.011368	0.011266	0.011369	0.011300	0.011197	0.011163
0.011197	0.011197	0.011266	0.011197	0.011163	0.011334	0.011197
0.011231	0.011232	0.011368	0.011403	0.011300	0.011231	0.011232
0.011300	0.011163	0.011334	0.011197	0.011334	0.011437	0.011643
0.011232	0.011608	0.011236	0.017889	0.011261	0.011367	0.011558
0.011519	0.011562	0.011327	0.011297	0.011676	0.011588	0.011370

0.011494	0.011489	0.011139	0.011230	0.011218	0.011311	0.011234
0.011489	0.011400	0.011398	0.011195	0.011369	0.011403	0.011405
0.011269	0.011334	0.011368	0.011334	0.011300	0.011300	0.011266
0.011300	0.011231	0.011266	0.011300	0.011266	0.011197	0.011231
0.011231	0.011300	0.011197	0.011300	0.011334	0.011368	0.011266
0.011300	0.011266	0.011540	0.011540	0.011334	0.011369	0.011403
0.011163	0.011163	0.011197	0.011300	0.011163	0.011403	0.011403
0.011437	0.011403	0.011369	0.011471	0.011608	0.011473	0.011300
0.011300	0.011334	0.011403	0.011232	0.011300	0.011266	0.011300
0.011369	0.011368	0.011403	0.011369	0.011266	0.011471	0.011437
0.011302	0.011197	0.011300	0.011369	0.011266	0.011437	0.011471
0.011643	0.011304	0.011506	0.011471	0.011403	0.011574	0.011197
0.011369	0.011266	0.011231	0.011231	0.011300	0.011197	0.011300
0.011334	0.011368	0.011266	0.011300	0.011266	0.011540	0.011540
0.011334	0.011369	0.011437	0.011368	0.011986	0.011421	0.011347
0.011415	0.011377	0.011486	0.011378	0.011450	0.011410	0.011372
0.011935	0.011270	0.011408	0.011340	0.011617	0.011474	0.011580
0.011960	0.011441	0.011302	0.011300	0.011300	0.011334	0.011267
0.011163	0.011197	0.011334	0.011300	0.011302	0.011437	0.011404
0.011266	0.011197	0.011232	0.011266	0.011163	0.011232	0.011129
0.011266	0.011300	0.011197	0.011163	0.011266	0.011197	0.011232
0.011231	0.011163	0.011232	0.011266	0.011163	0.011369	0.011403
0.011300	0.011369	0.011300	0.011300	0.011231	0.011574	0.011266
0.011307	0.011232	0.011471	0.011475	0.011334	0.011334	0.011334
0.011334	0.011266	0.011231	0.011266	0.011368	0.011369	0.011300
0.011300	0.011403	0.011574	0.011334	0.011506	0.011506	0.011403
0.011231	0.011300	0.011334	0.011711	0.011300	0.011334	0.011300
0.011334	0.011266	0.011266	0.011574	0.011471	0.011540	0.011266
0.011369	0.011437	0.011300	0.011369	0.011300	0.011197	0.011505
0.011300	0.011266	0.011266	0.011437	0.011266	0.011368	0.011608
0.011231	0.011649	0.011643	0.011472	0.011230	0.011372	0.011270
0.011226	0.011691	0.011204	0.011423	0.011259	0.011371	0.011385
0.011475	0.011195	0.011344	0.011195	0.011225	0.011403	0.011351
0.011368	0.011261	0.011348	0.011240	0.011301	0.011437	0.011506
0.011471	0.011369	0.011474	0.011475	0.011579	0.011601	0.011266
0.011540	0.011403	0.011266	0.011369	0.011163	0.011403	0.011197
0.011232	0.011266	0.011231	0.011197	0.011231	0.011197	0.011300
0.011272	0.011270	0.011268	0.011300	0.011437	0.011405	0.011405
0.011403	0.011334	0.011134	0.011132	0.011266	0.011437	0.011403
0.011231	0.011300	0.011540	0.011507	0.011437	0.011471	0.011403
0.011334	0.011403	0.011403	0.011300	0.011848	0.011437	0.011540
0.011574	0.011606	0.011269	0.011342	0.011231	0.011437	0.011403
0.011438	0.011437	0.011471	0.011471	0.011620	0.011608	0.011237
0.011129	0.000000	0.011233	0.011199	0.011268	0.011136	0.011441
0.011270	0.011413	0.011156	0.011286	0.011266	0.011334	0.011232
0.011197	0.011197	0.011266	0.011231	0.011437	0.011403	0.011334
0.011437	0.011505	0.011231	0.011231	0.011266	0.011266	0.011267
0.011336	0.011266	0.011266	0.011301	0.011437	0.011506	0.011471
0.011369	0.011474	0.011475	0.011579	0.011601	0.011266	0.011471
0.011403	0.011403	0.011233	0.011415	0.011343	0.011373	0.011238
0.011412	0.011620	0.011404	0.011268	0.011197	0.011403	0.011266
0.011368	0.011300	0.011197	0.011301	0.011403	0.011405	0.011300
0.011197	0.011129	0.011266	0.011197	0.011129	0.011234	0.011403
0.011300	0.011231	0.011266	0.011300	0.011266	0.011197	0.011403
0.011266	0.011300	0.011300	0.011231	0.011300	0.011506	0.011197
0.011232	0.011574	0.011231	0.011232	0.011231	0.011574	0.011191
0.011680	0.011188	0.011264	0.011423	0.011514	0.011426	0.011145
0.011220	0.011403	0.011506	0.011614	0.011472	0.011612	0.011746
0.011643	0.011680	0.011643	0.011896	0.011574	0.012501	0.011644
0.012295	0.012160	0.012263	0.012505	0.011608	0.011300	0.011608

0.012021	0.011989	0.011574	0.011471	0.011540	0.011540	0.011509
0.011403	0.011406	0.011471	0.011437	0.011814	0.011883	0.011883
0.011781	0.011883	0.011919	0.011749	0.012021	0.012027	0.012090
0.012090	0.012124	0.012035	0.012090	0.012092	0.011334	0.011643
0.011677	0.012157	0.011717	0.011952	0.012433	0.011476	0.011369
0.011334	0.011334	0.011814	0.011842	0.011914	0.011677	0.011335
S153	0.011634	0.011634	0.011770	0.011736	0.011736	0.011804
0.011641	0.011539	0.011736	0.011229	0.011297	0.011196	0.011229
0.011263	0.011432	0.011229	0.011263	0.011330	0.011297	0.011263
0.011229	0.011263	0.011364	0.011229	0.011263	0.011263	0.011607
0.011499	0.011263	0.011162	0.011398	0.011297	0.011297	0.011330
0.011297	0.011398	0.011297	0.011297	0.011330	0.011297	0.011466
0.011229	0.011297	0.011330	0.011263	0.011364	0.011398	0.011330
0.011297	0.011466	0.011364	0.011634	0.011297	0.011567	0.011297
0.011263	0.011364	0.011432	0.011567	0.011634	0.011364	0.011466
0.011330	0.011398	0.011297	0.011533	0.011770	0.011297	0.011364
0.011263	0.011364	0.011330	0.011398	0.011330	0.011330	0.011330
0.011229	0.011263	0.011229	0.011330	0.011297	0.011331	0.011398
0.011330	0.011263	0.011364	0.011263	0.011398	0.011229	0.011297
0.011465	0.011398	0.011634	0.011432	0.011297	0.011533	0.011297
0.011470	0.011698	0.011477	0.011217	0.011210	0.011319	0.011416
0.012052	0.011521	0.011226	0.011533	0.011533	0.011330	0.011432
0.011364	0.011330	0.011398	0.011297	0.011398	0.011330	0.011330
0.011297	0.011398	0.011601	0.011465	0.011364	0.011331	0.011263
0.011229	0.011229	0.011297	0.011263	0.011465	0.011432	0.011364
0.011465	0.011533	0.011263	0.011263	0.011297	0.011297	0.011298
0.011366	0.011297	0.011297	0.011168	0.011241	0.011200	0.011297
0.011298	0.011233	0.011283	0.011268	0.011196	0.011232	0.000000
0.011528	0.011302	0.011336	0.011229	0.011234	0.011364	0.011330
0.011398	0.011331	0.011330	0.011398	0.011364	0.011736	0.011499
0.011567	0.011432	0.011297	0.011398	0.011195	0.011432	0.011229
0.011297	0.011297	0.011263	0.011229	0.011263	0.011229	0.011330
0.011297	0.011398	0.011330	0.011229	0.011331	0.011432	0.011434
0.011432	0.011330	0.011229	0.011162	0.011297	0.011229	0.011162
0.011265	0.011432	0.011229	0.011432	0.011195	0.011297	0.011263
0.011364	0.011263	0.011297	0.011263	0.011297	0.011162	0.011297
0.011330	0.011263	0.011330	0.011263	0.011297	0.011330	0.011567
0.011668	0.011601	0.011668	0.011433	0.011668	0.011297	0.011263
0.011298	0.011587	0.011382	0.011198	0.011675	0.011163	0.011258
0.011675	0.011572	0.011465	0.011668	0.011335	0.011533	0.011499
0.011432	0.011601	0.011453	0.011309	0.011355	0.011330	0.011401
0.011499	0.011399	0.011537	0.011605	0.011533	0.011601	0.011708
0.011435	0.011606	0.011536	0.011468	0.011737	0.011195	0.011399
0.011364	0.011297	0.011195	0.011195	0.011263	0.011263	0.011330
0.011263	0.011330	0.011229	0.011330	0.011263	0.011263	0.011330
0.011263	0.011702	0.011601	0.011634	0.011162	0.011263	0.011297
0.011297	0.011263	0.011330	0.011364	0.011229	0.011229	0.011229
0.011770	0.011364	0.011398	0.011195	0.011297	0.011466	0.011229
0.011229	0.011297	0.011263	0.011432	0.011229	0.011364	0.011465
0.011432	0.011263	0.011263	0.011195	0.011195	0.011364	0.011297
0.011195	0.011297	0.011162	0.011195	0.011330	0.011466	0.011229
0.011195	0.011330	0.011263	0.011162	0.011499	0.011162	0.011330
0.011404	0.000000	0.011533	0.011364	0.011398	0.011263	0.011364
0.011229	0.011567	0.011162	0.011432	0.011229	0.011229	0.011162
0.011398	0.011601	0.011297	0.011364	0.011195	0.011601	0.011229
0.011229	0.011229	0.011263	0.011364	0.011263	0.011364	0.011432
0.011398	0.011398	0.011297	0.011398	0.011364	0.011229	0.011195
0.011229	0.011229	0.011297	0.011229	0.011195	0.011364	0.011229
0.011263	0.011263	0.011398	0.011432	0.011330	0.011263	0.011263
0.011330	0.011229	0.011364	0.011229	0.011364	0.011465	0.011702

0.011263	0.011668	0.011268	0.017820	0.011192	0.011367	0.011621
0.011582	0.011626	0.011376	0.011352	0.011668	0.011650	0.011305
0.011537	0.011488	0.011178	0.011271	0.011256	0.011259	0.011276
0.011532	0.011434	0.011433	0.011326	0.011398	0.011432	0.011434
0.011302	0.011398	0.011398	0.011364	0.011330	0.011331	0.011297
0.011330	0.011263	0.011297	0.011330	0.011297	0.011229	0.011263
0.011263	0.011330	0.011229	0.011331	0.011364	0.011398	0.011297
0.011330	0.011297	0.011634	0.011634	0.011364	0.011399	0.011432
0.011195	0.011195	0.011229	0.011330	0.011195	0.011432	0.011432
0.011465	0.011465	0.011398	0.011499	0.011668	0.011501	0.011330
0.011330	0.011364	0.011432	0.011263	0.011330	0.011297	0.011330
0.011398	0.011398	0.011432	0.011398	0.011297	0.011499	0.011465
0.011333	0.011229	0.011330	0.011398	0.011331	0.011466	0.011499
0.011668	0.011335	0.011533	0.011499	0.011432	0.011601	0.011229
0.011399	0.011297	0.011263	0.011263	0.011330	0.011229	0.011331
0.011364	0.011398	0.011297	0.011330	0.011297	0.011634	0.011634
0.011364	0.011399	0.011465	0.011398	0.012041	0.011454	0.011379
0.011448	0.011409	0.011518	0.011409	0.011517	0.011442	0.011406
0.011992	0.011303	0.011439	0.011372	0.011647	0.011505	0.011607
0.012017	0.011476	0.011332	0.011330	0.011330	0.011364	0.011298
0.011195	0.011229	0.011364	0.011330	0.011332	0.011465	0.011433
0.011297	0.011229	0.011263	0.011297	0.011195	0.011263	0.011162
0.011297	0.011330	0.011229	0.011195	0.011297	0.011229	0.011263
0.011263	0.011195	0.011263	0.011297	0.011195	0.011398	0.011432
0.011330	0.011398	0.011330	0.011330	0.011263	0.011634	0.011297
0.011340	0.011263	0.011499	0.011503	0.011364	0.011364	0.011364
0.011364	0.011297	0.011263	0.011297	0.011398	0.011398	0.011330
0.011330	0.011432	0.011634	0.011364	0.011567	0.011567	0.011432
0.011263	0.011330	0.011364	0.011770	0.011331	0.011364	0.011330
0.011364	0.011297	0.011297	0.011533	0.011533	0.011567	0.011297
0.011398	0.011466	0.011330	0.011398	0.011364	0.011229	0.011567
0.011330	0.011297	0.011297	0.011466	0.011297	0.011398	0.011635
0.011263	0.011709	0.011702	0.011509	0.011286	0.011402	0.011319
0.011170	0.011725	0.011237	0.011419	0.011306	0.011316	0.011381
0.011522	0.011127	0.011340	0.011193	0.011223	0.011398	0.011386
0.011401	0.011298	0.011379	0.011273	0.011331	0.011465	0.011533
0.011499	0.011398	0.011502	0.011503	0.011572	0.011629	0.011297
0.011567	0.011432	0.011297	0.011398	0.011195	0.011432	0.011229
0.011297	0.011297	0.011263	0.011229	0.011263	0.011229	0.011330
0.011305	0.011305	0.011301	0.011334	0.011471	0.011437	0.011436
0.011435	0.011364	0.011171	0.011168	0.011298	0.011468	0.011469
0.011263	0.011332	0.011567	0.011535	0.011466	0.011499	0.011432
0.011364	0.011432	0.011432	0.011330	0.011905	0.011499	0.011567
0.011601	0.011669	0.011300	0.011373	0.011263	0.011465	0.011432
0.011466	0.011465	0.011499	0.011499	0.011613	0.011634	0.011269
0.011162	0.011165	0.011265	0.000000	0.011335	0.011173	0.011504
0.011302	0.011456	0.011204	0.011323	0.011297	0.011368	0.011263
0.011229	0.011229	0.011297	0.011263	0.011465	0.011432	0.011364
0.011465	0.011533	0.011263	0.011263	0.011297	0.011297	0.011298
0.011366	0.011297	0.011297	0.011331	0.011465	0.011533	0.011499
0.011398	0.011502	0.011503	0.011572	0.011629	0.011297	0.011499
0.011432	0.011432	0.011230	0.011445	0.011375	0.011368	0.011236
0.011406	0.011611	0.011442	0.011334	0.011229	0.011432	0.011297
0.011398	0.011330	0.011229	0.011331	0.011432	0.011434	0.011330
0.011229	0.011162	0.011297	0.011229	0.011162	0.011265	0.011432
0.011330	0.011263	0.011297	0.011330	0.011297	0.011229	0.011432
0.011297	0.011330	0.011330	0.011263	0.011330	0.011567	0.011229
0.011263	0.011634	0.011263	0.011263	0.011263	0.011634	0.011233
0.011736	0.011231	0.011312	0.011463	0.011551	0.011457	0.011186
0.011271	0.011432	0.011533	0.011640	0.011500	0.011638	0.011770

0.011668	0.011705	0.011668	0.011918	0.011634	0.012514	0.011669
0.012379	0.012178	0.012314	0.012587	0.011634	0.011330	0.011634
0.012075	0.012010	0.011601	0.011499	0.011567	0.011567	0.011537
0.011432	0.011435	0.011499	0.011466	0.011838	0.011905	0.011905
0.011805	0.011905	0.012009	0.011774	0.012075	0.012081	0.012143
0.012143	0.012177	0.012124	0.012143	0.012145	0.011364	0.011668
0.011736	0.012244	0.011742	0.011973	0.012448	0.011504	0.011398
0.011364	0.011364	0.011837	0.011883	0.011943	0.011702	0.011365
S154	0.011924	0.011924	0.011744	0.011708	0.011708	0.011744
0.011534	0.011425	0.011708	0.011456	0.011564	0.011529	0.011492
0.011456	0.011708	0.011420	0.011528	0.011600	0.011564	0.011528
0.011492	0.011528	0.011348	0.011493	0.011529	0.011528	0.011498
0.011492	0.011528	0.011420	0.011384	0.011564	0.011564	0.011456
0.011528	0.011636	0.011564	0.011564	0.011600	0.011564	0.011744
0.011420	0.011564	0.011600	0.011528	0.011564	0.011672	0.011564
0.011564	0.011744	0.011637	0.011528	0.011564	0.011852	0.011564
0.011528	0.011564	0.011708	0.011852	0.011528	0.011528	0.011636
0.011600	0.011672	0.011564	0.011780	0.011600	0.011564	0.011636
0.011528	0.011636	0.011528	0.011672	0.011600	0.011600	0.011600
0.011492	0.011528	0.011456	0.011492	0.011564	0.011601	0.011636
0.011600	0.011528	0.011636	0.011528	0.011528	0.011492	0.011528
0.011744	0.011636	0.011564	0.011672	0.011564	0.011816	0.011564
0.011725	0.011573	0.011690	0.011388	0.011411	0.011565	0.011348
0.012052	0.011167	0.011448	0.011780	0.011780	0.011528	0.011564
0.011636	0.011600	0.011672	0.011564	0.011672	0.011600	0.011600
0.011564	0.011600	0.011888	0.011708	0.011636	0.011600	0.011528
0.011492	0.011420	0.011564	0.011528	0.011456	0.011420	0.011348
0.011456	0.011528	0.011456	0.011456	0.011492	0.011492	0.011495
0.011568	0.011564	0.011564	0.011425	0.011469	0.011458	0.011564
0.011564	0.011496	0.011482	0.011528	0.011456	0.011492	0.011528
0.000000	0.011564	0.011604	0.011492	0.011492	0.011636	0.011528
0.011600	0.011600	0.011600	0.011636	0.011636	0.012033	0.011708
0.011564	0.011564	0.011564	0.011564	0.011456	0.011420	0.011492
0.011528	0.011564	0.011528	0.011492	0.011528	0.011492	0.011600
0.011528	0.011636	0.011600	0.011456	0.011566	0.011672	0.011676
0.011708	0.011600	0.011492	0.011422	0.011564	0.011492	0.011420
0.011425	0.011708	0.011492	0.011708	0.011456	0.011528	0.011456
0.011636	0.011528	0.011564	0.011528	0.011564	0.011420	0.011564
0.011564	0.011528	0.011600	0.011528	0.011564	0.011600	0.011852
0.011528	0.011420	0.011528	0.011710	0.011564	0.011564	0.011528
0.011494	0.011797	0.011550	0.011465	0.011570	0.011425	0.011610
0.011570	0.011206	0.011672	0.011961	0.011610	0.011780	0.011780
0.011708	0.011852	0.011737	0.011500	0.011591	0.011600	0.011675
0.011744	0.011675	0.011713	0.011895	0.011816	0.011816	0.012004
0.011708	0.011895	0.011816	0.011744	0.012034	0.011456	0.011673
0.011636	0.011564	0.011456	0.011420	0.011528	0.011492	0.011600
0.011528	0.011600	0.011492	0.011528	0.011528	0.011528	0.011600
0.011528	0.011600	0.011456	0.011492	0.011420	0.011528	0.011564
0.011564	0.011456	0.011564	0.011528	0.011492	0.011492	0.011492
0.011672	0.011564	0.011672	0.011456	0.011564	0.011744	0.011492
0.011456	0.011528	0.011528	0.011708	0.011492	0.011420	0.011492
0.011492	0.011492	0.011528	0.011420	0.011456	0.011564	0.011564
0.011420	0.011528	0.011420	0.011420	0.011600	0.011384	0.011492
0.011456	0.011600	0.011528	0.011420	0.011384	0.011420	0.011600
0.011684	0.011492	0.011420	0.011600	0.011600	0.011528	0.011636
0.011492	0.011852	0.011420	0.011492	0.011420	0.011420	0.011420
0.011672	0.011240	0.011564	0.011636	0.011456	0.011456	0.011420
0.011492	0.011492	0.011492	0.011636	0.011492	0.011600	0.011672
0.011672	0.011672	0.011564	0.011672	0.011636	0.011492	0.011456
0.011492	0.011492	0.011564	0.011420	0.011456	0.011636	0.011492

0.011456	0.011528	0.011636	0.011636	0.011600	0.011528	0.011492
0.011600	0.011456	0.011636	0.011492	0.011636	0.011708	0.011600
0.011528	0.011564	0.011536	0.017306	0.011262	0.011513	0.011504
0.011464	0.011504	0.011557	0.011425	0.011525	0.011536	0.011574
0.011457	0.011316	0.011428	0.011501	0.011387	0.011427	0.011421
0.011207	0.011371	0.011254	0.011399	0.011636	0.011708	0.011713
0.011573	0.011600	0.011672	0.011564	0.011600	0.011601	0.011564
0.011600	0.011528	0.011528	0.011600	0.011528	0.011492	0.011528
0.011528	0.011600	0.011492	0.011600	0.011600	0.011672	0.011564
0.011528	0.011492	0.011492	0.011492	0.011636	0.011637	0.011708
0.011420	0.011456	0.011492	0.011600	0.011456	0.011672	0.011672
0.011672	0.011672	0.011672	0.011708	0.011564	0.011710	0.011600
0.011564	0.011636	0.011708	0.011528	0.011600	0.011528	0.011600
0.011636	0.011636	0.011672	0.011672	0.011565	0.011780	0.011744
0.011604	0.011456	0.011600	0.011672	0.011564	0.011672	0.011780
0.011961	0.011610	0.011780	0.011780	0.011708	0.011852	0.011492
0.011674	0.011564	0.011528	0.011528	0.011600	0.011492	0.011600
0.011600	0.011672	0.011564	0.011528	0.011492	0.011492	0.011492
0.011636	0.011637	0.011744	0.011672	0.012250	0.011706	0.011662
0.011733	0.011653	0.011807	0.011692	0.011694	0.011723	0.011642
0.012200	0.011576	0.011718	0.011649	0.011865	0.011713	0.011898
0.012225	0.011694	0.011532	0.011528	0.011528	0.011348	0.011495
0.011456	0.011492	0.011564	0.011528	0.011531	0.011456	0.011421
0.011564	0.011492	0.011528	0.011564	0.011456	0.011492	0.011420
0.011492	0.011528	0.011492	0.011456	0.011564	0.011492	0.011528
0.011528	0.011456	0.011528	0.011564	0.011456	0.011636	0.011708
0.011600	0.011600	0.011600	0.011600	0.011492	0.011528	0.011564
0.011319	0.011528	0.011492	0.011496	0.011636	0.011636	0.011600
0.011636	0.011564	0.011528	0.011564	0.011672	0.011672	0.011600
0.011600	0.011708	0.011528	0.011636	0.011780	0.011780	0.011636
0.011528	0.011600	0.011636	0.011600	0.011601	0.011636	0.011600
0.011564	0.011492	0.011492	0.011780	0.011780	0.011744	0.011564
0.011672	0.011744	0.011600	0.011672	0.011600	0.011492	0.011456
0.011600	0.011564	0.011492	0.011744	0.011564	0.011672	0.011636
0.011528	0.011607	0.011600	0.011442	0.011231	0.011600	0.011274
0.011407	0.011669	0.011502	0.011576	0.011421	0.011382	0.011282
0.011325	0.011200	0.011346	0.011196	0.011289	0.011404	0.011660
0.011697	0.011558	0.011584	0.011544	0.011602	0.011456	0.011528
0.011492	0.011565	0.011676	0.011497	0.011787	0.011819	0.011564
0.011564	0.011564	0.011564	0.011564	0.011456	0.011420	0.011492
0.011528	0.011564	0.011528	0.011492	0.011528	0.011492	0.011600
0.011535	0.011568	0.011564	0.011600	0.011744	0.011709	0.011710
0.011708	0.011636	0.011437	0.011428	0.011564	0.011744	0.011673
0.011528	0.011600	0.011780	0.011817	0.011708	0.011780	0.011708
0.011636	0.011708	0.011672	0.011600	0.011817	0.011708	0.011816
0.011852	0.011519	0.011499	0.011615	0.011528	0.011744	0.011708
0.011746	0.011708	0.011744	0.011744	0.011902	0.011924	0.011539
0.011423	0.011429	0.011533	0.011496	0.011496	0.011453	0.011387
0.011574	0.011244	0.011409	0.011613	0.011564	0.011636	0.011528
0.011492	0.011420	0.011564	0.011528	0.011456	0.011420	0.011348
0.011456	0.011528	0.011456	0.011456	0.011492	0.011492	0.011495
0.011568	0.011564	0.011564	0.011602	0.011456	0.011528	0.011492
0.011565	0.011676	0.011497	0.011787	0.011819	0.011564	0.011780
0.011708	0.011708	0.011531	0.011730	0.011653	0.011681	0.011473
0.011614	0.011947	0.011694	0.011570	0.011492	0.011708	0.011528
0.011636	0.011600	0.011456	0.011566	0.011672	0.011676	0.011600
0.011492	0.011422	0.011564	0.011492	0.011420	0.011425	0.011708
0.011600	0.011528	0.011528	0.011600	0.011528	0.011492	0.011420
0.011564	0.011600	0.011600	0.011528	0.011600	0.011816	0.011420
0.011528	0.011528	0.011528	0.011528	0.011492	0.011528	0.011417

0.011688	0.011365	0.011218	0.011393	0.011486	0.011437	0.011192
0.011169	0.011420	0.011528	0.011642	0.011493	0.011640	0.011781
0.011672	0.011711	0.011672	0.011939	0.011600	0.012466	0.011673
0.012322	0.012180	0.012289	0.012507	0.011564	0.011528	0.011636
0.012286	0.012036	0.011600	0.011492	0.011564	0.011564	0.011531
0.011420	0.011424	0.011492	0.011456	0.011817	0.011889	0.011852
0.011781	0.011853	0.011927	0.011749	0.012251	0.012294	0.012359
0.012359	0.012358	0.012293	0.012322	0.012325	0.011636	0.011852
0.011888	0.012394	0.011969	0.011853	0.012359	0.011388	0.011312
0.011276	0.011204	0.011817	0.011827	0.011912	0.011672	0.011349
S155	0.011681	0.011681	0.011784	0.011750	0.011750	0.011819
0.011583	0.011549	0.011750	0.011267	0.011336	0.011302	0.011267
0.011301	0.011474	0.011267	0.011301	0.011370	0.011336	0.011301
0.011267	0.011302	0.011405	0.011267	0.011302	0.011301	0.011618
0.011543	0.011302	0.011198	0.011439	0.011336	0.011336	0.011370
0.011302	0.011439	0.011336	0.011336	0.011370	0.011336	0.011508
0.011267	0.011336	0.011370	0.011301	0.011405	0.011439	0.011336
0.011336	0.011508	0.011405	0.011646	0.011336	0.011612	0.011336
0.011301	0.011405	0.011474	0.011612	0.011646	0.011370	0.011508
0.011370	0.011439	0.011336	0.011577	0.011784	0.011336	0.011405
0.011302	0.011405	0.011336	0.011439	0.011302	0.011370	0.011370
0.011267	0.011301	0.011267	0.011370	0.011336	0.011371	0.011439
0.011370	0.011301	0.011405	0.011233	0.011439	0.011267	0.011302
0.011508	0.011439	0.011681	0.011474	0.011336	0.011577	0.011336
0.011441	0.011674	0.011483	0.011220	0.011212	0.011287	0.011422
0.012066	0.011528	0.011228	0.011577	0.011577	0.011370	0.011439
0.011405	0.011370	0.011439	0.011336	0.011439	0.011370	0.011370
0.011336	0.011370	0.011646	0.011508	0.011405	0.011371	0.011302
0.011267	0.011267	0.011336	0.011301	0.011508	0.011474	0.011405
0.011508	0.011577	0.011301	0.011301	0.011336	0.011336	0.011338
0.011407	0.011336	0.011336	0.011168	0.011278	0.011236	0.011336
0.011336	0.011270	0.011285	0.011306	0.011233	0.011267	0.011302
0.011564	0.000000	0.011375	0.011267	0.011271	0.011405	0.011371
0.011440	0.011370	0.011370	0.011405	0.011405	0.011784	0.011543
0.011612	0.011439	0.011336	0.011439	0.011233	0.011474	0.011267
0.011302	0.011336	0.011301	0.011267	0.011301	0.011267	0.011370
0.011336	0.011439	0.011370	0.011233	0.011372	0.011474	0.011442
0.011474	0.011370	0.011267	0.011199	0.011336	0.011267	0.011198
0.011304	0.011474	0.011267	0.011474	0.011233	0.011336	0.011302
0.011405	0.011301	0.011336	0.011302	0.011336	0.011198	0.011336
0.011370	0.011301	0.011370	0.011301	0.011336	0.011370	0.011612
0.011681	0.011612	0.011681	0.011441	0.011681	0.011336	0.011301
0.011337	0.011557	0.011437	0.011201	0.011687	0.011200	0.011303
0.011688	0.011618	0.011474	0.011715	0.011376	0.011577	0.011543
0.011474	0.011646	0.011495	0.011354	0.011357	0.011371	0.011442
0.011543	0.011441	0.011546	0.011651	0.011577	0.011646	0.011756
0.011475	0.011651	0.011577	0.011508	0.011785	0.011233	0.011440
0.011405	0.011336	0.011233	0.011233	0.011302	0.011302	0.011370
0.011301	0.011370	0.011267	0.011370	0.011302	0.011301	0.011370
0.011302	0.011715	0.011612	0.011646	0.011198	0.011302	0.011336
0.011336	0.011301	0.011336	0.011405	0.011267	0.011267	0.011267
0.011784	0.011405	0.011439	0.011233	0.011336	0.011508	0.011267
0.011267	0.011301	0.011301	0.011474	0.011267	0.011405	0.011508
0.011474	0.011267	0.011301	0.011198	0.011233	0.011405	0.011336
0.011198	0.011301	0.011198	0.011233	0.011370	0.011508	0.011267
0.011233	0.011370	0.011301	0.011198	0.011508	0.011198	0.011370
0.011447	0.011267	0.011543	0.011405	0.011439	0.011302	0.011405
0.011267	0.011612	0.011198	0.011439	0.011267	0.011267	0.011198
0.011439	0.011647	0.011336	0.011405	0.011233	0.011612	0.011267
0.011267	0.011267	0.011301	0.011405	0.011301	0.011405	0.011439

0.011439	0.011439	0.011336	0.011440	0.011405	0.011267	0.011233
0.011267	0.011267	0.011336	0.011267	0.011233	0.011405	0.011267
0.011301	0.011302	0.011439	0.011474	0.011370	0.011301	0.011302
0.011370	0.011233	0.011405	0.011267	0.011405	0.011508	0.011715
0.011302	0.011681	0.011272	0.017969	0.011261	0.011370	0.011597
0.011557	0.011601	0.011328	0.011356	0.011738	0.011627	0.011373
0.011544	0.011555	0.011180	0.011274	0.011259	0.011313	0.011235
0.011539	0.011439	0.011438	0.011195	0.011439	0.011474	0.011477
0.011341	0.011370	0.011439	0.011405	0.011371	0.011371	0.011336
0.011370	0.011301	0.011336	0.011370	0.011336	0.011267	0.011301
0.011301	0.011370	0.011267	0.011371	0.011370	0.011439	0.011336
0.011370	0.011336	0.011612	0.011612	0.011405	0.011440	0.011474
0.011233	0.011233	0.011267	0.011370	0.011233	0.011474	0.011474
0.011474	0.011474	0.011439	0.011508	0.011681	0.011510	0.011370
0.011370	0.011405	0.011474	0.011302	0.011370	0.011336	0.011370
0.011440	0.011439	0.011474	0.011439	0.011337	0.011543	0.011508
0.011373	0.011233	0.011370	0.011439	0.011336	0.011508	0.011543
0.011715	0.011376	0.011577	0.011543	0.011474	0.011646	0.011267
0.011441	0.011336	0.011301	0.011301	0.011370	0.011267	0.011371
0.011370	0.011439	0.011336	0.011370	0.011336	0.011612	0.011612
0.011405	0.011440	0.011508	0.011439	0.012061	0.011496	0.011421
0.011489	0.011450	0.011561	0.011451	0.011524	0.011483	0.011444
0.012011	0.011342	0.011480	0.011413	0.011690	0.011546	0.011653
0.012036	0.011481	0.011373	0.011370	0.011370	0.011405	0.011338
0.011233	0.011267	0.011405	0.011370	0.011373	0.011508	0.011440
0.011336	0.011267	0.011302	0.011336	0.011233	0.011302	0.011198
0.011336	0.011370	0.011267	0.011233	0.011336	0.011267	0.011302
0.011301	0.011233	0.011302	0.011336	0.011233	0.011439	0.011474
0.011370	0.011439	0.011370	0.011370	0.011301	0.011646	0.011336
0.011380	0.011302	0.011543	0.011547	0.011405	0.011405	0.011405
0.011405	0.011336	0.011301	0.011336	0.011439	0.011439	0.011370
0.011370	0.011474	0.011646	0.011405	0.011577	0.011577	0.011474
0.011301	0.011370	0.011405	0.011784	0.011371	0.011405	0.011370
0.011370	0.011336	0.011336	0.011647	0.011543	0.011612	0.011336
0.011439	0.011508	0.011370	0.011439	0.011370	0.011267	0.011577
0.011370	0.011336	0.011336	0.011508	0.011336	0.011439	0.011681
0.011301	0.011723	0.011715	0.011516	0.011287	0.011443	0.011322
0.011227	0.011735	0.011240	0.011425	0.011310	0.011373	0.011387
0.011527	0.011196	0.011344	0.011195	0.011226	0.011403	0.011394
0.011409	0.011303	0.011385	0.011277	0.011372	0.011508	0.011577
0.011543	0.011440	0.011546	0.011547	0.011652	0.011714	0.011336
0.011612	0.011439	0.011336	0.011439	0.011233	0.011474	0.011267
0.011302	0.011336	0.011301	0.011267	0.011301	0.011267	0.011370
0.011344	0.011341	0.011339	0.011302	0.011509	0.011477	0.011475
0.011474	0.011336	0.011208	0.011205	0.011336	0.011508	0.011474
0.011301	0.011370	0.011577	0.011579	0.011508	0.011543	0.011474
0.011405	0.011474	0.011439	0.011370	0.011923	0.011508	0.011612
0.011646	0.011682	0.011340	0.011414	0.011301	0.011508	0.011474
0.011440	0.011508	0.011543	0.011543	0.011663	0.011681	0.011309
0.011199	0.011202	0.011304	0.011269	0.011339	0.011174	0.011513
0.011341	0.011459	0.011204	0.011325	0.011336	0.011405	0.011302
0.011267	0.011267	0.011336	0.011301	0.011508	0.011474	0.011405
0.011508	0.011577	0.011301	0.011301	0.011336	0.011336	0.011338
0.011407	0.011336	0.011336	0.011372	0.011508	0.011577	0.011543
0.011440	0.011546	0.011547	0.011652	0.011714	0.011336	0.011543
0.011474	0.011474	0.011303	0.011488	0.011416	0.011375	0.011276
0.011449	0.011671	0.011493	0.011339	0.011267	0.011474	0.011336
0.011439	0.011370	0.011233	0.011372	0.011474	0.011442	0.011370
0.011267	0.011199	0.011336	0.011267	0.011198	0.011304	0.011474
0.011370	0.011301	0.011336	0.011370	0.011336	0.011267	0.011474

0.011336	0.011370	0.011370	0.011301	0.011370	0.011577	0.011267
0.011302	0.011646	0.011301	0.011302	0.011267	0.011646	0.011235
0.011740	0.011232	0.011316	0.011466	0.011559	0.011465	0.011188
0.011274	0.011474	0.011577	0.011687	0.011544	0.011650	0.011820
0.011715	0.011753	0.011715	0.011936	0.011646	0.012544	0.011716
0.012372	0.012167	0.012306	0.012584	0.011681	0.011370	0.011681
0.012096	0.012029	0.011646	0.011543	0.011612	0.011612	0.011581
0.011474	0.011443	0.011543	0.011508	0.011888	0.011957	0.011957
0.011855	0.011957	0.011959	0.011789	0.012096	0.012102	0.012165
0.012165	0.012200	0.012076	0.012165	0.012167	0.011405	0.011716
0.011750	0.012199	0.011791	0.012026	0.012511	0.011548	0.011439
0.011405	0.011405	0.011854	0.011884	0.011991	0.011750	0.011406
S156	0.011715	0.011715	0.011818	0.011784	0.011784	0.011853
0.011688	0.011583	0.011784	0.011301	0.011370	0.011336	0.011301
0.011336	0.011508	0.011301	0.011336	0.011405	0.011370	0.011336
0.011301	0.011336	0.011439	0.011302	0.011267	0.011336	0.011653
0.011542	0.011336	0.011232	0.011473	0.011301	0.011370	0.011301
0.011370	0.011405	0.011370	0.011370	0.011405	0.011370	0.011473
0.011301	0.011370	0.011405	0.011336	0.011439	0.011473	0.011405
0.011370	0.011543	0.011405	0.011680	0.011370	0.011577	0.011370
0.011336	0.011439	0.011508	0.011577	0.011680	0.011439	0.011542
0.011336	0.011405	0.011370	0.011542	0.011784	0.011370	0.011439
0.011336	0.011439	0.011405	0.011473	0.011405	0.011405	0.011405
0.011301	0.011336	0.011301	0.011370	0.011370	0.011405	0.011473
0.011405	0.011336	0.011301	0.011336	0.011473	0.011232	0.011336
0.011542	0.011473	0.011715	0.011474	0.011370	0.011542	0.011370
0.011477	0.011671	0.011514	0.011219	0.011247	0.011399	0.011416
0.012088	0.011520	0.011226	0.011577	0.011577	0.011405	0.011473
0.011439	0.011370	0.011473	0.011370	0.011474	0.011336	0.011404
0.011370	0.011473	0.011680	0.011542	0.011439	0.011405	0.011267
0.011301	0.011301	0.011301	0.011336	0.011542	0.011508	0.011439
0.011542	0.011611	0.011336	0.011336	0.011370	0.011370	0.011372
0.011442	0.011370	0.011370	0.011205	0.011313	0.011274	0.011370
0.011370	0.011304	0.011319	0.011340	0.011268	0.011303	0.011336
0.011604	0.011375	0.000000	0.011301	0.011305	0.011439	0.011405
0.011474	0.011405	0.011405	0.011473	0.011439	0.011749	0.011577
0.011646	0.011508	0.011370	0.011474	0.011267	0.011473	0.011301
0.011336	0.011370	0.011336	0.011301	0.011336	0.011301	0.011405
0.011267	0.011405	0.011405	0.011301	0.011406	0.011508	0.011511
0.011439	0.011405	0.011301	0.011233	0.011370	0.011301	0.011233
0.011339	0.011439	0.011232	0.011439	0.011267	0.011370	0.011336
0.011370	0.011336	0.011301	0.011336	0.011370	0.011232	0.011301
0.011336	0.011336	0.011405	0.011336	0.011370	0.011405	0.011646
0.011715	0.011646	0.011715	0.011509	0.011715	0.011370	0.011336
0.011372	0.011642	0.011436	0.011271	0.011722	0.011235	0.011381
0.011722	0.011652	0.011508	0.011749	0.011410	0.011577	0.011508
0.011508	0.011646	0.011534	0.011357	0.011402	0.011405	0.011477
0.011577	0.011475	0.011546	0.011616	0.011611	0.011680	0.011790
0.011511	0.011617	0.011615	0.011545	0.011819	0.011267	0.011475
0.011370	0.011370	0.011267	0.011267	0.011267	0.011336	0.011405
0.011267	0.011336	0.011232	0.011336	0.011267	0.011336	0.011370
0.011336	0.011749	0.011646	0.011680	0.011232	0.011336	0.011370
0.011370	0.011336	0.011405	0.011439	0.011301	0.011301	0.011301
0.011818	0.011439	0.011473	0.011267	0.011370	0.011474	0.011301
0.011267	0.011336	0.011336	0.011405	0.011301	0.011439	0.011439
0.011508	0.011336	0.011336	0.011267	0.011267	0.011439	0.011370
0.011267	0.011370	0.011232	0.011267	0.011405	0.011542	0.011301
0.011267	0.011405	0.011336	0.011232	0.011542	0.011232	0.011405
0.011411	0.011301	0.011577	0.011439	0.011473	0.011336	0.011439
0.011301	0.011577	0.011232	0.011473	0.011301	0.011301	0.011232

0.011404	0.011681	0.011370	0.011439	0.011267	0.011646	0.011301
0.011301	0.011301	0.011301	0.011370	0.011336	0.011439	0.011508
0.011474	0.011439	0.011370	0.011474	0.011370	0.011301	0.011267
0.011301	0.011301	0.011370	0.011301	0.011198	0.011439	0.011301
0.011336	0.011336	0.011473	0.011508	0.011405	0.011267	0.011336
0.011405	0.011267	0.011439	0.011301	0.011439	0.011508	0.011749
0.011336	0.011715	0.011342	0.017901	0.011192	0.011558	0.011665
0.011625	0.011670	0.011327	0.011293	0.011667	0.011694	0.011308
0.011536	0.011610	0.011179	0.011315	0.011258	0.011362	0.011278
0.011531	0.011471	0.011436	0.011193	0.011439	0.011508	0.011511
0.000000	0.011473	0.011473	0.011439	0.011405	0.011405	0.011370
0.011405	0.011336	0.011336	0.011405	0.011370	0.011301	0.011336
0.011336	0.011405	0.011301	0.011405	0.011439	0.011473	0.011370
0.011405	0.011370	0.011646	0.011646	0.011439	0.011474	0.011508
0.011232	0.011267	0.011301	0.011336	0.011267	0.011508	0.011508
0.011508	0.011508	0.011473	0.011542	0.011715	0.011544	0.011336
0.011370	0.011473	0.011439	0.011336	0.011405	0.011336	0.011405
0.011439	0.011439	0.011473	0.011370	0.011302	0.011577	0.011473
0.011408	0.011301	0.011405	0.011474	0.011370	0.011542	0.011577
0.011749	0.011410	0.011577	0.011508	0.011508	0.011646	0.011301
0.011475	0.011370	0.011336	0.011336	0.011405	0.011301	0.011405
0.011439	0.011473	0.011370	0.011405	0.011370	0.011646	0.011646
0.011439	0.011474	0.011542	0.011473	0.012129	0.011463	0.011458
0.011528	0.011452	0.011600	0.011487	0.011526	0.011520	0.011446
0.012081	0.011379	0.011517	0.011450	0.011730	0.011583	0.011618
0.012106	0.011524	0.011407	0.011405	0.011404	0.011439	0.011372
0.011267	0.011232	0.011439	0.011405	0.011407	0.011508	0.011509
0.011370	0.011232	0.011267	0.011370	0.011198	0.011336	0.011232
0.011370	0.011405	0.011301	0.011267	0.011370	0.011301	0.011336
0.011336	0.011267	0.011336	0.011370	0.011267	0.011405	0.011508
0.011405	0.011473	0.011405	0.011405	0.011267	0.011680	0.011370
0.011417	0.011336	0.011542	0.011547	0.011370	0.011439	0.011439
0.011439	0.011301	0.011336	0.011301	0.011473	0.011473	0.011405
0.011405	0.011508	0.011680	0.011439	0.011611	0.011611	0.011508
0.011336	0.011405	0.011439	0.011784	0.011405	0.011370	0.011405
0.011405	0.011370	0.011370	0.011646	0.011542	0.011611	0.011370
0.011474	0.011542	0.011405	0.011474	0.011405	0.011301	0.011611
0.011404	0.011370	0.011370	0.011542	0.011370	0.011473	0.011715
0.011336	0.011757	0.011749	0.011509	0.011229	0.011476	0.011319
0.011339	0.011724	0.011309	0.011464	0.011207	0.011418	0.011380
0.011470	0.011127	0.011340	0.011127	0.011169	0.011330	0.011348
0.011368	0.011220	0.011420	0.011348	0.011406	0.011508	0.011577
0.011577	0.011474	0.011580	0.011582	0.011686	0.011748	0.011370
0.011646	0.011508	0.011370	0.011474	0.011267	0.011473	0.011301
0.011336	0.011370	0.011336	0.011301	0.011336	0.011301	0.011405
0.011307	0.011378	0.011372	0.011407	0.011546	0.011406	0.011510
0.011509	0.011439	0.011246	0.011241	0.011370	0.011543	0.011543
0.011336	0.011405	0.011646	0.011612	0.011508	0.011577	0.011508
0.011405	0.011508	0.011508	0.011404	0.011956	0.011508	0.011612
0.011680	0.011718	0.011340	0.011415	0.011336	0.011542	0.011508
0.011543	0.011508	0.011542	0.011542	0.011743	0.011715	0.011345
0.011234	0.011238	0.011339	0.011304	0.011376	0.011252	0.011548
0.011376	0.011509	0.011207	0.011403	0.011370	0.011442	0.011267
0.011301	0.011301	0.011301	0.011336	0.011542	0.011508	0.011439
0.011542	0.011611	0.011336	0.011336	0.011370	0.011370	0.011372
0.011442	0.011370	0.011370	0.011406	0.011508	0.011577	0.011577
0.011474	0.011580	0.011582	0.011686	0.011748	0.011370	0.011577
0.011508	0.011508	0.011303	0.011522	0.011451	0.011479	0.011348
0.011378	0.011666	0.011451	0.011409	0.011232	0.011439	0.011267
0.011405	0.011405	0.011301	0.011406	0.011508	0.011511	0.011405

0.011301	0.011233	0.011370	0.011301	0.011233	0.011339	0.011439
0.011405	0.011336	0.011336	0.011405	0.011370	0.011301	0.011508
0.011370	0.011405	0.011405	0.011336	0.011405	0.011611	0.011301
0.011336	0.011680	0.011336	0.011267	0.011336	0.011680	0.011232
0.011622	0.011230	0.011312	0.011463	0.011550	0.011459	0.011186
0.011271	0.011508	0.011611	0.011721	0.011578	0.011719	0.011853
0.011750	0.011788	0.011749	0.012006	0.011680	0.012612	0.011750
0.012405	0.012269	0.012408	0.012651	0.011680	0.011405	0.011715
0.012164	0.012098	0.011680	0.011577	0.011646	0.011646	0.011615
0.011508	0.011512	0.011577	0.011542	0.011922	0.011991	0.011956
0.011887	0.011991	0.012027	0.011857	0.012164	0.012171	0.012233
0.012233	0.012268	0.012211	0.012233	0.012236	0.011439	0.011715
0.011784	0.012336	0.011825	0.012025	0.012544	0.011547	0.011473
0.011439	0.011439	0.011922	0.011897	0.011986	0.011749	0.011440
S157	0.011598	0.011598	0.011733	0.011699	0.011699	0.011766
0.011604	0.011502	0.011699	0.011195	0.011262	0.011229	0.011195
0.011228	0.011396	0.011195	0.011228	0.011296	0.011262	0.011161
0.011195	0.011228	0.011329	0.011195	0.011229	0.011228	0.011570
0.011464	0.011228	0.011128	0.011363	0.011296	0.011262	0.011296
0.011262	0.011363	0.011262	0.011262	0.011296	0.011262	0.011430
0.011195	0.011262	0.011296	0.011228	0.011329	0.011363	0.011296
0.011262	0.011430	0.011329	0.011598	0.011262	0.011531	0.011262
0.011228	0.011329	0.011396	0.011531	0.011632	0.011329	0.011430
0.011296	0.011363	0.011262	0.011497	0.011733	0.011262	0.011329
0.011228	0.011329	0.011296	0.011363	0.011296	0.011296	0.011296
0.011195	0.011228	0.011195	0.011296	0.011262	0.011296	0.011363
0.011296	0.011262	0.011329	0.011228	0.011363	0.011195	0.011262
0.011430	0.011363	0.011598	0.011430	0.011262	0.011497	0.011262
0.011431	0.011658	0.011475	0.011177	0.011172	0.011281	0.011375
0.012011	0.011467	0.011102	0.011497	0.011497	0.011296	0.011396
0.011329	0.011296	0.011363	0.011262	0.011363	0.011296	0.011296
0.011262	0.011363	0.011565	0.011430	0.011329	0.011296	0.011228
0.011195	0.011195	0.011262	0.011228	0.011430	0.011396	0.011329
0.011430	0.011497	0.011228	0.011228	0.011262	0.011262	0.011263
0.011331	0.011262	0.011262	0.011132	0.011205	0.011165	0.011262
0.011263	0.011198	0.011245	0.011233	0.011162	0.011197	0.011229
0.011492	0.011267	0.011301	0.000000	0.011199	0.011329	0.011296
0.011363	0.011296	0.011296	0.011396	0.011329	0.011699	0.011464
0.011531	0.011396	0.011262	0.011363	0.011161	0.011396	0.011195
0.011262	0.011262	0.011228	0.011195	0.011228	0.011195	0.011296
0.011262	0.011363	0.011296	0.011195	0.011296	0.011396	0.011399
0.011396	0.011296	0.011195	0.011128	0.011262	0.011195	0.011128
0.011162	0.011397	0.011195	0.011396	0.011161	0.011262	0.011228
0.011329	0.011228	0.011262	0.011228	0.011262	0.011128	0.011262
0.011296	0.011161	0.011296	0.011228	0.011262	0.011296	0.011531
0.011632	0.011564	0.011632	0.011398	0.011632	0.011262	0.011161
0.011263	0.011545	0.011335	0.011163	0.011638	0.011129	0.011257
0.011638	0.011536	0.011430	0.011632	0.011334	0.011497	0.011464
0.011396	0.011564	0.011416	0.011266	0.011270	0.011228	0.011365
0.011464	0.011364	0.011500	0.011568	0.011497	0.011565	0.011671
0.011399	0.011570	0.011501	0.011433	0.011700	0.011161	0.011364
0.011329	0.011262	0.011161	0.011161	0.011228	0.011228	0.011296
0.011228	0.011296	0.011195	0.011296	0.011228	0.011161	0.011296
0.011228	0.011665	0.011565	0.011598	0.011128	0.011228	0.011262
0.011262	0.011228	0.011296	0.011329	0.011195	0.011195	0.011195
0.011733	0.011329	0.011363	0.011161	0.011262	0.011430	0.011195
0.011195	0.011262	0.011228	0.011396	0.011195	0.011329	0.011464
0.011396	0.011228	0.011161	0.011161	0.011161	0.011329	0.011262
0.011161	0.011262	0.011128	0.011161	0.011296	0.011430	0.011195
0.011161	0.011296	0.011229	0.011128	0.011464	0.011128	0.011296

0.011369	0.011195	0.011497	0.011329	0.011363	0.011228	0.011329
0.011195	0.011531	0.011128	0.011396	0.011195	0.011195	0.011128
0.011363	0.011565	0.011262	0.011329	0.011161	0.011565	0.011195
0.011195	0.011128	0.011228	0.011329	0.011228	0.011329	0.011396
0.011396	0.011363	0.011262	0.011363	0.011329	0.011195	0.011161
0.011195	0.011195	0.011262	0.011195	0.011161	0.011329	0.011195
0.011228	0.011228	0.011363	0.011396	0.011296	0.011228	0.011228
0.011296	0.011228	0.011329	0.011195	0.011329	0.011430	0.011665
0.011228	0.011632	0.011233	0.017710	0.011191	0.011366	0.011583
0.011545	0.011588	0.011322	0.011292	0.011664	0.011612	0.011303
0.011487	0.011487	0.011138	0.011228	0.011216	0.011258	0.011232
0.011482	0.011395	0.011394	0.011193	0.011363	0.011396	0.011399
0.011266	0.011363	0.011363	0.011329	0.011296	0.011296	0.011262
0.011296	0.011228	0.011262	0.011296	0.011262	0.011195	0.011228
0.011228	0.011296	0.011195	0.011296	0.011329	0.011363	0.011262
0.011296	0.011262	0.011598	0.011598	0.011329	0.011363	0.011396
0.011161	0.011161	0.011195	0.011296	0.011161	0.011396	0.011396
0.011430	0.011430	0.011363	0.011464	0.011632	0.011465	0.011296
0.011296	0.011363	0.011396	0.011228	0.011296	0.011262	0.011296
0.011363	0.011363	0.011396	0.011363	0.011262	0.011464	0.011430
0.011298	0.011195	0.011296	0.011396	0.011296	0.011430	0.011464
0.011632	0.011334	0.011497	0.011464	0.011396	0.011564	0.011195
0.011364	0.011262	0.011228	0.011228	0.011296	0.011195	0.011296
0.011329	0.011363	0.011262	0.011296	0.011262	0.011598	0.011598
0.011329	0.011363	0.011430	0.011363	0.012003	0.011418	0.011342
0.011412	0.011373	0.011482	0.011372	0.011480	0.011406	0.011371
0.011954	0.011268	0.011403	0.011337	0.011611	0.011469	0.011571
0.011979	0.011399	0.011297	0.011296	0.011296	0.011329	0.011263
0.011161	0.011195	0.011329	0.011296	0.011297	0.011430	0.011397
0.011262	0.011128	0.011228	0.011262	0.011161	0.011228	0.011128
0.011262	0.011296	0.011195	0.011161	0.011262	0.011195	0.011228
0.011228	0.011161	0.011228	0.011262	0.011161	0.011363	0.011396
0.011296	0.011363	0.011296	0.011296	0.011228	0.011632	0.011262
0.011304	0.011228	0.011464	0.011468	0.011329	0.011329	0.011329
0.011329	0.011262	0.011228	0.011262	0.011363	0.011363	0.011296
0.011296	0.011430	0.011598	0.011329	0.011531	0.011531	0.011396
0.011228	0.011296	0.011329	0.011733	0.011296	0.011329	0.011296
0.011329	0.011262	0.011262	0.011565	0.011497	0.011531	0.011262
0.011363	0.011430	0.011296	0.011363	0.011329	0.011195	0.011531
0.011296	0.011262	0.011262	0.011430	0.011262	0.011363	0.011598
0.011228	0.011672	0.011665	0.011467	0.011229	0.011366	0.011267
0.011170	0.011683	0.011201	0.011373	0.011255	0.011315	0.011326
0.011468	0.011126	0.011283	0.011126	0.011168	0.011328	0.011344
0.011362	0.011257	0.011342	0.011272	0.011296	0.011430	0.011497
0.011464	0.011363	0.011466	0.011467	0.011603	0.011626	0.011262
0.011531	0.011396	0.011262	0.011363	0.011161	0.011396	0.011195
0.011262	0.011262	0.011228	0.011195	0.011228	0.011195	0.011296
0.011270	0.011270	0.011197	0.011300	0.011437	0.011402	0.011402
0.011401	0.011329	0.011134	0.011132	0.011263	0.011433	0.011433
0.011228	0.011297	0.011531	0.011500	0.011430	0.011464	0.011396
0.011329	0.011396	0.011396	0.011296	0.011868	0.011464	0.011531
0.011565	0.011631	0.011265	0.011337	0.011228	0.011430	0.011396
0.011431	0.011430	0.011464	0.011464	0.011571	0.011598	0.011234
0.011128	0.011130	0.011231	0.011196	0.011300	0.011098	0.011468
0.011198	0.011367	0.011108	0.011248	0.011262	0.011334	0.011228
0.011195	0.011195	0.011262	0.011228	0.011430	0.011396	0.011329
0.011430	0.011497	0.011228	0.011228	0.011262	0.011262	0.011263
0.011331	0.011262	0.011262	0.011296	0.011430	0.011497	0.011464
0.011363	0.011466	0.011467	0.011603	0.011626	0.011262	0.011464
0.011396	0.011396	0.011229	0.011408	0.011338	0.011367	0.011235

0.011405	0.011607	0.011399	0.011298	0.011195	0.011396	0.011262
0.011363	0.011296	0.011195	0.011296	0.011396	0.011399	0.011296
0.011195	0.011128	0.011262	0.011195	0.011128	0.011162	0.011397
0.011296	0.011228	0.011262	0.011296	0.011262	0.011195	0.011396
0.011262	0.011296	0.011296	0.011228	0.011296	0.011531	0.011195
0.011228	0.011632	0.011228	0.011262	0.011228	0.011598	0.011189
0.011678	0.011188	0.011261	0.011422	0.011508	0.011419	0.011144
0.011218	0.011396	0.011497	0.011603	0.011464	0.011602	0.011733
0.011632	0.011669	0.011632	0.011880	0.011598	0.012474	0.011633
0.012339	0.012139	0.012275	0.012546	0.011598	0.011296	0.011598
0.012037	0.011972	0.011565	0.011464	0.011531	0.011531	0.011501
0.011396	0.011400	0.011464	0.011430	0.011800	0.011867	0.011867
0.011768	0.011868	0.011970	0.011737	0.012037	0.012042	0.012104
0.012104	0.012138	0.012084	0.012104	0.012106	0.011329	0.011632
0.011699	0.012204	0.011705	0.011935	0.012408	0.011468	0.011363
0.011329	0.011329	0.011800	0.011800	0.011940	0.011665	0.011330
S158	0.011613	0.011613	0.011717	0.011683	0.011683	0.011717
0.011550	0.011445	0.011683	0.011199	0.011268	0.011233	0.011129
0.011233	0.011406	0.011199	0.011233	0.011302	0.011268	0.011233
0.011199	0.011233	0.011337	0.011199	0.011233	0.011233	0.011515
0.011475	0.011233	0.011129	0.011371	0.011302	0.011268	0.011233
0.011268	0.011371	0.011268	0.011268	0.011302	0.011268	0.011440
0.011199	0.011268	0.011302	0.011233	0.011337	0.011371	0.011302
0.011268	0.011441	0.011337	0.011579	0.011268	0.011544	0.011268
0.011233	0.011337	0.011406	0.011544	0.011613	0.011337	0.011440
0.011302	0.011371	0.011268	0.011475	0.011683	0.011268	0.011337
0.011233	0.011337	0.011302	0.011371	0.011302	0.011302	0.011302
0.011199	0.011233	0.011164	0.011268	0.011268	0.011303	0.011371
0.011302	0.011268	0.011337	0.011233	0.011371	0.011199	0.011233
0.011440	0.011371	0.011579	0.011371	0.011268	0.011475	0.011268
0.011397	0.011589	0.011437	0.011099	0.011172	0.011283	0.011336
0.011719	0.011365	0.011143	0.011475	0.011475	0.011302	0.011371
0.011337	0.011302	0.011371	0.011268	0.011371	0.011302	0.011302
0.011268	0.011371	0.011579	0.011440	0.011337	0.011302	0.011233
0.011199	0.011199	0.011268	0.011233	0.011440	0.011406	0.011337
0.011440	0.011510	0.011233	0.011233	0.011268	0.011268	0.011269
0.011339	0.011268	0.011268	0.011132	0.011207	0.011168	0.011268
0.011268	0.011200	0.011245	0.011233	0.011165	0.011200	0.011234
0.011492	0.011271	0.011305	0.011199	0.000000	0.011337	0.011302
0.011371	0.011302	0.011302	0.011406	0.011337	0.011717	0.011475
0.011544	0.011406	0.011268	0.011371	0.011164	0.011406	0.011199
0.011233	0.011268	0.011233	0.011199	0.011233	0.011199	0.011302
0.011233	0.011371	0.011302	0.011199	0.011303	0.011406	0.011408
0.011406	0.011302	0.011199	0.011130	0.011268	0.011199	0.011129
0.011235	0.011406	0.011199	0.011406	0.011164	0.011233	0.011233
0.011337	0.011233	0.011268	0.011233	0.011268	0.011129	0.011268
0.011268	0.011233	0.011302	0.011233	0.011268	0.011302	0.011544
0.011613	0.011475	0.011613	0.011407	0.011613	0.011268	0.011233
0.011269	0.011562	0.011390	0.011166	0.011619	0.011130	0.011262
0.011585	0.011549	0.011406	0.011648	0.011341	0.011475	0.011475
0.011406	0.011544	0.011427	0.011188	0.011318	0.011302	0.011374
0.011475	0.011373	0.011443	0.011583	0.011510	0.011579	0.011688
0.011408	0.011584	0.011513	0.011443	0.011718	0.011164	0.011372
0.011337	0.011268	0.011164	0.011129	0.011233	0.011233	0.011302
0.011233	0.011302	0.011199	0.011302	0.011233	0.011233	0.011302
0.011233	0.011648	0.011544	0.011579	0.011129	0.011233	0.011268
0.011268	0.011233	0.011302	0.011337	0.011199	0.011199	0.011199
0.011717	0.011337	0.011371	0.011164	0.011268	0.011441	0.011199
0.011164	0.011233	0.011233	0.011406	0.011199	0.011337	0.011475
0.011406	0.011233	0.011233	0.011164	0.011164	0.011337	0.011268

0.011164	0.011268	0.011129	0.011164	0.011302	0.011441	0.011199
0.011164	0.011302	0.011233	0.011129	0.011440	0.011129	0.011302
0.011377	0.011199	0.011475	0.011302	0.011371	0.011233	0.011337
0.011199	0.011544	0.011129	0.011371	0.011199	0.011199	0.011129
0.011371	0.011579	0.011268	0.011337	0.011164	0.011544	0.011199
0.011199	0.011199	0.011233	0.011337	0.011233	0.011337	0.011337
0.011371	0.011371	0.011268	0.011371	0.011337	0.011199	0.011164
0.011199	0.011199	0.011268	0.011199	0.011164	0.011337	0.011199
0.011233	0.011233	0.011337	0.011406	0.011302	0.011233	0.011233
0.011302	0.011199	0.011337	0.011199	0.011337	0.011406	0.011648
0.011233	0.011613	0.011236	0.017806	0.011126	0.011306	0.011557
0.011518	0.011558	0.011270	0.011234	0.011609	0.011587	0.011243
0.011392	0.011426	0.011099	0.011186	0.011217	0.011209	0.011147
0.011389	0.011395	0.011394	0.011259	0.011371	0.011406	0.011408
0.011272	0.011371	0.011371	0.011337	0.011302	0.011302	0.011268
0.011302	0.011233	0.011233	0.011302	0.011233	0.011199	0.011233
0.011233	0.011302	0.011199	0.011302	0.011337	0.011371	0.011268
0.011302	0.011268	0.011544	0.011544	0.011337	0.011372	0.011406
0.011164	0.011164	0.011199	0.011302	0.011164	0.011406	0.011406
0.011406	0.011406	0.011371	0.011440	0.011613	0.011442	0.011302
0.011268	0.011371	0.011406	0.011233	0.011302	0.011233	0.011302
0.011337	0.011337	0.011371	0.011371	0.011268	0.011475	0.011440
0.011304	0.011199	0.011302	0.011406	0.011268	0.011441	0.011475
0.011648	0.011341	0.011475	0.011475	0.011406	0.011544	0.011199
0.011372	0.011268	0.011233	0.011233	0.011302	0.011199	0.011302
0.011337	0.011371	0.011268	0.011302	0.011268	0.011544	0.011544
0.011337	0.011372	0.011440	0.011371	0.012029	0.011392	0.011350
0.011422	0.011346	0.011422	0.011382	0.011421	0.011416	0.011342
0.011980	0.011274	0.011413	0.011345	0.011627	0.011446	0.011586
0.012005	0.011449	0.011304	0.011302	0.011302	0.011337	0.011269
0.011164	0.011199	0.011337	0.011302	0.011304	0.011440	0.011407
0.011268	0.011199	0.011233	0.011268	0.011164	0.011199	0.011129
0.011268	0.011302	0.011129	0.011164	0.011268	0.011199	0.011233
0.011233	0.011164	0.011233	0.011268	0.011164	0.011371	0.011406
0.011302	0.011371	0.011302	0.011302	0.011164	0.011613	0.011268
0.011311	0.011233	0.011475	0.011479	0.011337	0.011337	0.011337
0.011337	0.011268	0.011233	0.011268	0.011371	0.011371	0.011233
0.011302	0.011440	0.011579	0.011337	0.011510	0.011510	0.011406
0.011233	0.011302	0.011337	0.011683	0.011302	0.011337	0.011302
0.011302	0.011268	0.011268	0.011544	0.011510	0.011510	0.011268
0.011371	0.011440	0.011302	0.011371	0.011302	0.011199	0.011510
0.011302	0.011268	0.011268	0.011440	0.011268	0.011371	0.011613
0.011233	0.011620	0.011648	0.011426	0.011117	0.011372	0.011163
0.011115	0.011644	0.011202	0.011373	0.011158	0.011264	0.011273
0.011417	0.000000	0.011227	0.000000	0.011114	0.011261	0.011306
0.011327	0.011219	0.011347	0.011240	0.011303	0.011440	0.011510
0.011475	0.011372	0.011478	0.011479	0.011583	0.011606	0.011268
0.011544	0.011406	0.011268	0.011371	0.011164	0.011406	0.011199
0.011233	0.011268	0.011233	0.011199	0.011233	0.011199	0.011302
0.011236	0.011273	0.011268	0.011305	0.011444	0.011407	0.011408
0.011408	0.011337	0.011134	0.011132	0.011268	0.011441	0.011406
0.011233	0.011302	0.011544	0.011510	0.011406	0.011475	0.011406
0.011337	0.011406	0.011406	0.011233	0.011856	0.011406	0.011510
0.011579	0.011611	0.011271	0.011345	0.011233	0.011441	0.011406
0.011441	0.011406	0.011440	0.011440	0.011584	0.011613	0.011239
0.011130	0.011132	0.011235	0.011200	0.011271	0.011137	0.011445
0.011272	0.011422	0.011159	0.011252	0.011268	0.011270	0.011233
0.011199	0.011199	0.011268	0.011233	0.011440	0.011406	0.011337
0.011440	0.011510	0.011233	0.011233	0.011268	0.011268	0.011269
0.011339	0.011268	0.011268	0.011303	0.011440	0.011510	0.011475

0.011372	0.011478	0.011479	0.011583	0.011606	0.011268	0.011475
0.011406	0.011406	0.011234	0.011418	0.011346	0.011376	0.011241
0.011344	0.011580	0.011410	0.011305	0.011199	0.011406	0.011233
0.011371	0.011302	0.011199	0.011303	0.011406	0.011408	0.011302
0.011199	0.011130	0.011268	0.011199	0.011129	0.011235	0.011406
0.011302	0.011233	0.011233	0.011302	0.011233	0.011199	0.011406
0.011268	0.011302	0.011302	0.011233	0.011302	0.011510	0.011199
0.011233	0.011613	0.011233	0.011233	0.011233	0.011579	0.011146
0.011622	0.011145	0.011161	0.011422	0.011467	0.011422	0.000000
0.011113	0.011406	0.011510	0.011619	0.011476	0.011617	0.011752
0.011648	0.011687	0.011648	0.011904	0.011579	0.012445	0.011649
0.012306	0.012170	0.012309	0.012553	0.011579	0.011302	0.011613
0.012064	0.011998	0.011579	0.011475	0.011544	0.011544	0.011513
0.011406	0.011409	0.011475	0.011441	0.011787	0.011856	0.011821
0.011752	0.011856	0.011892	0.011721	0.012030	0.012070	0.012134
0.012134	0.012134	0.012104	0.012099	0.012101	0.011337	0.011614
0.011648	0.012237	0.011723	0.011890	0.012411	0.011444	0.011371
0.011337	0.011337	0.011821	0.011819	0.011914	0.011648	0.011338
S159 0.011733	0.011733	0.011868	0.011834	0.011834	0.011902	
0.011707	0.011605	0.011834	0.011329	0.011397	0.011363	0.011329
0.011363	0.011531	0.011329	0.011363	0.011430	0.011397	0.011363
0.011329	0.011363	0.011464	0.011330	0.011363	0.011363	0.011673
0.011598	0.011363	0.011262	0.011497	0.011430	0.011397	0.011430
0.011397	0.011498	0.011397	0.011397	0.011430	0.011397	0.011565
0.011329	0.011397	0.011430	0.011363	0.011464	0.011497	0.011430
0.011397	0.011565	0.011464	0.011733	0.011397	0.011666	0.011397
0.011363	0.011464	0.011531	0.011666	0.011767	0.011464	0.011565
0.011430	0.011498	0.011397	0.011632	0.011868	0.011397	0.011464
0.011363	0.011464	0.011430	0.011498	0.011430	0.011430	0.011430
0.011329	0.011363	0.011329	0.011430	0.011397	0.011431	0.011497
0.011430	0.011397	0.011464	0.011363	0.011497	0.011329	0.011397
0.011565	0.011497	0.011733	0.011531	0.011397	0.011632	0.011397
0.011542	0.011770	0.011588	0.011295	0.011321	0.011428	0.011493
0.012158	0.011518	0.011266	0.011632	0.011632	0.011430	0.011531
0.000000	0.011430	0.011497	0.011397	0.011498	0.011430	0.011430
0.011397	0.011497	0.011700	0.011565	0.011464	0.011430	0.011363
0.011329	0.011329	0.011397	0.011363	0.011565	0.011531	0.011464
0.011565	0.011632	0.011363	0.011363	0.011397	0.011397	0.011399
0.011467	0.011397	0.011397	0.011276	0.011349	0.011306	0.011397
0.011399	0.011335	0.011392	0.011371	0.011298	0.011334	0.011364
0.011636	0.011405	0.011439	0.011329	0.011337	0.000000	0.011430
0.011498	0.011431	0.011430	0.011531	0.011464	0.011834	0.011599
0.011666	0.011531	0.011397	0.011498	0.011296	0.011531	0.011329
0.011397	0.011397	0.011363	0.011329	0.011363	0.011329	0.011430
0.011397	0.011497	0.011430	0.011329	0.011432	0.011531	0.011534
0.011531	0.011430	0.011329	0.011263	0.011397	0.011329	0.011262
0.011366	0.011531	0.011329	0.011531	0.011296	0.011397	0.011363
0.011464	0.011363	0.011397	0.011363	0.011397	0.011262	0.011397
0.011430	0.011363	0.011094	0.011363	0.011397	0.011430	0.011666
0.011767	0.011699	0.011767	0.011533	0.011767	0.011397	0.011363
0.011398	0.011667	0.011426	0.011301	0.011774	0.011265	0.011336
0.011741	0.011673	0.011565	0.011767	0.011436	0.011632	0.011599
0.011531	0.011699	0.011559	0.011432	0.011480	0.011430	0.011501
0.011599	0.011499	0.011636	0.011705	0.011632	0.011700	0.011807
0.011535	0.011706	0.011636	0.011569	0.011835	0.011296	0.011499
0.011464	0.011397	0.011296	0.011296	0.011363	0.011363	0.011430
0.011363	0.011430	0.011329	0.011430	0.011363	0.011363	0.011430
0.011363	0.011800	0.011700	0.011733	0.011262	0.011363	0.011397
0.011397	0.011363	0.011430	0.011464	0.011329	0.011329	0.011329
0.011868	0.011464	0.011498	0.011296	0.011397	0.011565	0.011329

0.011329	0.011397	0.011363	0.011531	0.011329	0.011464	0.011598
0.011531	0.011363	0.011363	0.011296	0.011296	0.011464	0.011397
0.011296	0.011329	0.011262	0.011296	0.011430	0.011565	0.011329
0.011296	0.011430	0.011363	0.011262	0.011598	0.011262	0.011430
0.011506	0.011329	0.011632	0.011464	0.011498	0.011363	0.011397
0.011329	0.011666	0.011262	0.011531	0.011329	0.011329	0.011262
0.011497	0.011700	0.011397	0.011464	0.011296	0.011700	0.011329
0.011329	0.011329	0.011363	0.011464	0.011363	0.011464	0.011531
0.011498	0.011498	0.011397	0.011498	0.011464	0.011329	0.011296
0.011329	0.011329	0.011397	0.011329	0.011296	0.011464	0.011329
0.011363	0.011363	0.011498	0.011531	0.011430	0.011363	0.011363
0.011430	0.011363	0.011464	0.011329	0.011464	0.011565	0.011800
0.011363	0.011767	0.011371	0.017864	0.011388	0.011488	0.011723
0.011683	0.011729	0.011427	0.011408	0.011829	0.011751	0.011485
0.011582	0.011610	0.011255	0.011312	0.011373	0.011406	0.011361
0.011623	0.011507	0.011542	0.011391	0.011498	0.011531	0.011501
0.011404	0.011497	0.011498	0.011464	0.011430	0.011431	0.011397
0.011430	0.011363	0.011397	0.011430	0.011397	0.011329	0.011363
0.011363	0.011430	0.011329	0.011430	0.011464	0.011497	0.011397
0.011430	0.011397	0.011733	0.011733	0.011464	0.011498	0.011531
0.011296	0.011296	0.011329	0.011430	0.011296	0.011531	0.011531
0.011565	0.011565	0.011498	0.011598	0.011767	0.011600	0.011430
0.011430	0.011497	0.011531	0.011363	0.011430	0.011397	0.011430
0.011498	0.011497	0.011531	0.011498	0.011397	0.011598	0.011565
0.011434	0.011329	0.011430	0.011531	0.011430	0.011565	0.011598
0.011767	0.011436	0.011632	0.011599	0.011531	0.011699	0.011329
0.011499	0.011397	0.011363	0.011363	0.011430	0.011329	0.011430
0.011464	0.011497	0.011397	0.011430	0.011397	0.011733	0.011733
0.011464	0.011498	0.011565	0.011497	0.012138	0.011561	0.011483
0.011553	0.011512	0.011623	0.011511	0.011621	0.011544	0.011509
0.012093	0.011407	0.011541	0.011476	0.011750	0.011606	0.011708
0.012116	0.011588	0.011433	0.011430	0.011430	0.011464	0.011399
0.011296	0.011329	0.011464	0.011430	0.011433	0.011565	0.011532
0.011397	0.011329	0.011363	0.011397	0.011296	0.011363	0.011262
0.011397	0.011430	0.011329	0.011296	0.011397	0.011329	0.011363
0.011363	0.011296	0.011363	0.011397	0.011296	0.011498	0.011531
0.011430	0.011498	0.011430	0.011430	0.011363	0.011767	0.011397
0.011444	0.011363	0.011598	0.011604	0.011464	0.011464	0.011464
0.011464	0.011397	0.011363	0.011397	0.011498	0.011498	0.011430
0.011430	0.011565	0.011733	0.011464	0.011666	0.011666	0.011531
0.011363	0.011430	0.011464	0.011868	0.011430	0.011464	0.011430
0.011464	0.011397	0.011397	0.011700	0.011632	0.011666	0.011397
0.011498	0.011565	0.011430	0.011498	0.011464	0.011329	0.011666
0.011430	0.011397	0.011397	0.011565	0.011397	0.011497	0.011733
0.011363	0.011775	0.011800	0.011548	0.011342	0.011502	0.011370
0.011280	0.011761	0.011341	0.011507	0.011353	0.011467	0.011485
0.011570	0.011324	0.011450	0.011324	0.011330	0.011462	0.011425
0.011475	0.011336	0.011483	0.011377	0.011432	0.011565	0.011632
0.011598	0.011498	0.011602	0.011604	0.011705	0.011768	0.011397
0.011666	0.011531	0.011397	0.011498	0.011296	0.011531	0.011329
0.011397	0.011397	0.011363	0.011329	0.011363	0.011329	0.011430
0.011410	0.011410	0.011403	0.011437	0.011573	0.011538	0.011538
0.011469	0.011464	0.011245	0.011240	0.011399	0.011569	0.011569
0.011363	0.011433	0.011666	0.011635	0.011565	0.011599	0.011531
0.011464	0.011531	0.011531	0.011430	0.012003	0.011599	0.011666
0.011699	0.011738	0.011402	0.011476	0.011363	0.011565	0.011531
0.011566	0.011565	0.011599	0.011599	0.011729	0.011733	0.011373
0.011263	0.011268	0.011367	0.011332	0.011437	0.011284	0.011605
0.011404	0.011587	0.011346	0.011397	0.011397	0.011471	0.011363
0.011329	0.011329	0.011397	0.011363	0.011565	0.011531	0.011464

0.011565	0.011632	0.011363	0.011363	0.011397	0.011397	0.011399
0.011467	0.011397	0.011397	0.011432	0.011565	0.011632	0.011598
0.011498	0.011602	0.011604	0.011705	0.011768	0.011397	0.011598
0.011531	0.011531	0.011365	0.011548	0.011478	0.011504	0.011376
0.011543	0.011745	0.011568	0.011435	0.011329	0.011531	0.011397
0.011497	0.011430	0.011329	0.011432	0.011531	0.011534	0.011430
0.011329	0.011263	0.011397	0.011329	0.011262	0.011366	0.011531
0.011430	0.011363	0.011397	0.011430	0.011397	0.011329	0.011531
0.011397	0.011430	0.011430	0.011363	0.011430	0.011599	0.011329
0.011363	0.011767	0.011363	0.011363	0.011363	0.011733	0.011275
0.011791	0.011273	0.011311	0.011543	0.011589	0.011563	0.011227
0.011270	0.011531	0.011632	0.011740	0.011599	0.011738	0.011869
0.011767	0.011805	0.011767	0.012018	0.011733	0.012610	0.011768
0.012475	0.012275	0.012411	0.012682	0.011733	0.011430	0.011733
0.012172	0.012107	0.011699	0.011599	0.011666	0.011666	0.011636
0.011531	0.011536	0.011598	0.011565	0.011936	0.012003	0.012003
0.011903	0.012003	0.012106	0.011873	0.012173	0.012179	0.012240
0.012240	0.012274	0.012222	0.012240	0.012242	0.011464	0.011767
0.011834	0.012340	0.011807	0.012070	0.012544	0.011604	0.011498
0.011464	0.011464	0.011935	0.012006	0.012010	0.011800	0.011465
S160	0.011699	0.011699	0.011767	0.011733	0.011733	0.011800
0.011639	0.011536	0.011733	0.011296	0.011363	0.011330	0.011296
0.011262	0.011497	0.011228	0.011329	0.011396	0.011363	0.011329
0.011296	0.011329	0.011363	0.011296	0.011330	0.011329	0.011604
0.011497	0.011329	0.011228	0.011396	0.011396	0.011363	0.011397
0.011363	0.011464	0.011363	0.011363	0.011396	0.011363	0.011531
0.011228	0.011363	0.011396	0.011329	0.011363	0.011464	0.011396
0.011363	0.011531	0.011430	0.011632	0.011363	0.011632	0.011363
0.011329	0.011363	0.011497	0.011632	0.011666	0.011363	0.011464
0.011396	0.011464	0.011363	0.011598	0.011767	0.011363	0.011430
0.011329	0.011430	0.011397	0.011464	0.011396	0.011396	0.011396
0.011296	0.011329	0.011296	0.011329	0.011363	0.011397	0.011464
0.011397	0.011363	0.011430	0.011329	0.011396	0.011296	0.011363
0.011531	0.011464	0.011699	0.011531	0.011363	0.011598	0.011363
0.011542	0.011695	0.011589	0.011256	0.011284	0.011392	0.011415
0.012011	0.011416	0.011266	0.011598	0.011598	0.011329	0.011430
0.011430	0.011396	0.011464	0.011363	0.011464	0.011396	0.011396
0.011363	0.011396	0.011666	0.011531	0.011430	0.011397	0.011329
0.011296	0.011228	0.011363	0.011329	0.011464	0.011430	0.011363
0.011464	0.011531	0.011329	0.011329	0.011363	0.011363	0.011365
0.011433	0.011363	0.011363	0.011240	0.011313	0.011271	0.011363
0.011365	0.011301	0.011355	0.011336	0.011264	0.011300	0.011330
0.011528	0.011371	0.011405	0.011296	0.011302	0.011430	0.000000
0.011094	0.011397	0.011396	0.011497	0.011430	0.011800	0.011498
0.011565	0.011430	0.011363	0.011397	0.011262	0.011430	0.011296
0.011363	0.011363	0.011329	0.011296	0.011329	0.011296	0.011396
0.011363	0.011464	0.011397	0.011296	0.011398	0.011497	0.011500
0.011498	0.011396	0.011296	0.011229	0.011363	0.011296	0.011228
0.011332	0.011498	0.011296	0.011498	0.011262	0.011363	0.011329
0.011430	0.011329	0.011363	0.011329	0.011363	0.011228	0.011363
0.011397	0.011329	0.011396	0.011329	0.011363	0.011396	0.011632
0.011666	0.011598	0.011666	0.011499	0.011666	0.011363	0.011329
0.011297	0.011586	0.011426	0.011266	0.011672	0.011231	0.011375
0.011672	0.011570	0.011531	0.011733	0.011436	0.011598	0.011565
0.011497	0.011665	0.011487	0.011225	0.011312	0.011397	0.011467
0.011565	0.011465	0.011602	0.011670	0.011598	0.011666	0.011773
0.011501	0.011672	0.011602	0.011534	0.011801	0.011262	0.011465
0.011430	0.011363	0.011262	0.011262	0.011329	0.011329	0.011396
0.011329	0.011397	0.011296	0.011329	0.011329	0.011329	0.011396
0.011329	0.011699	0.011598	0.011632	0.011228	0.011329	0.011363

0.011363	0.011329	0.011397	0.011363	0.011296	0.011296	0.011296
0.011767	0.011363	0.011464	0.011262	0.011363	0.011531	0.011296
0.011296	0.011363	0.011329	0.011497	0.011296	0.011430	0.011565
0.011497	0.011329	0.011329	0.011262	0.011262	0.011430	0.011363
0.011262	0.011363	0.011228	0.011262	0.011396	0.011464	0.011296
0.011262	0.011396	0.011329	0.011228	0.011497	0.011228	0.011396
0.011471	0.011296	0.011531	0.011430	0.011397	0.011296	0.011430
0.011296	0.011632	0.011228	0.011430	0.011228	0.011228	0.011228
0.011464	0.011599	0.011363	0.011430	0.011262	0.011598	0.011228
0.011296	0.011296	0.011329	0.011430	0.011329	0.011430	0.011497
0.011497	0.011464	0.011363	0.011464	0.011430	0.011296	0.011262
0.011296	0.011296	0.011363	0.011228	0.011262	0.011430	0.011296
0.011262	0.011329	0.011464	0.011430	0.011396	0.011329	0.011329
0.011396	0.011329	0.011430	0.011296	0.011430	0.011531	0.011699
0.011329	0.011666	0.011336	0.017748	0.011191	0.011366	0.011618
0.011579	0.011623	0.011374	0.011408	0.011664	0.011647	0.011303
0.011440	0.011487	0.011255	0.011353	0.011177	0.011258	0.011318
0.011436	0.011433	0.011431	0.011259	0.011464	0.011497	0.011500
0.011370	0.011464	0.011464	0.011296	0.011397	0.011397	0.011363
0.011397	0.011329	0.011363	0.011396	0.011363	0.011296	0.011329
0.011329	0.011396	0.011296	0.011397	0.011430	0.011464	0.011363
0.011329	0.011296	0.011632	0.011632	0.011430	0.011464	0.011497
0.011262	0.011262	0.011296	0.011397	0.011262	0.011497	0.011497
0.011531	0.011531	0.011464	0.011565	0.011666	0.011566	0.011397
0.011396	0.011464	0.011497	0.011329	0.011396	0.011363	0.011397
0.011464	0.011464	0.011497	0.011464	0.011364	0.011565	0.011531
0.011400	0.011296	0.011396	0.011497	0.011397	0.011464	0.011565
0.011733	0.011436	0.011598	0.011565	0.011497	0.011665	0.011296
0.011465	0.011363	0.011329	0.011329	0.011396	0.011296	0.011397
0.011430	0.011464	0.011363	0.011329	0.011296	0.011632	0.011632
0.011430	0.011464	0.011531	0.011464	0.012037	0.011525	0.011448
0.011518	0.011477	0.011588	0.011477	0.011586	0.011509	0.011474
0.011989	0.011372	0.011506	0.011441	0.011715	0.011571	0.011673
0.012013	0.011475	0.011399	0.011396	0.011396	0.011363	0.011365
0.011262	0.011296	0.011430	0.011396	0.011399	0.011464	0.011431
0.011363	0.011296	0.011329	0.011363	0.011262	0.011329	0.011228
0.011296	0.011329	0.011296	0.011262	0.011363	0.011296	0.011329
0.011329	0.011262	0.011329	0.011363	0.011262	0.011464	0.011497
0.011397	0.011397	0.011396	0.011397	0.011329	0.011666	0.011363
0.011339	0.011329	0.011497	0.011502	0.011430	0.011430	0.011430
0.011430	0.011363	0.011329	0.011363	0.011464	0.011464	0.011396
0.011396	0.011531	0.011632	0.011430	0.011632	0.011632	0.011497
0.011329	0.011396	0.011430	0.011767	0.011397	0.011430	0.011397
0.011430	0.011296	0.011296	0.011531	0.011599	0.011497	0.011363
0.011464	0.011531	0.011396	0.011464	0.011430	0.011296	0.011565
0.011396	0.011363	0.011296	0.011531	0.011363	0.011464	0.011699
0.011329	0.011707	0.011699	0.011507	0.000000	0.011400	0.011215
0.011170	0.011683	0.011306	0.011418	0.011207	0.011366	0.011326
0.011468	0.011126	0.011394	0.011126	0.011276	0.011328	0.011466
0.011475	0.011375	0.011377	0.011377	0.011398	0.011464	0.011531
0.011497	0.011397	0.011500	0.011501	0.011637	0.011662	0.011363
0.011565	0.011430	0.011363	0.011397	0.011262	0.011430	0.011296
0.011363	0.011363	0.011329	0.011296	0.011329	0.011296	0.011396
0.011375	0.011375	0.011369	0.011403	0.011539	0.011504	0.011504
0.011503	0.011430	0.011245	0.011240	0.011365	0.011535	0.011535
0.011329	0.011399	0.011633	0.011601	0.011531	0.011565	0.011497
0.011430	0.011497	0.011497	0.011396	0.011902	0.011565	0.011632
0.011666	0.011631	0.011368	0.011441	0.011329	0.011531	0.011497
0.011532	0.011531	0.011565	0.011565	0.011729	0.011699	0.011338
0.011230	0.011234	0.011333	0.011298	0.011334	0.011247	0.011502

0.011369	0.011323	0.011156	0.011397	0.011363	0.011437	0.011329
0.011296	0.011228	0.011363	0.011329	0.011464	0.011430	0.011363
0.011464	0.011531	0.011329	0.011329	0.011363	0.011363	0.011365
0.011433	0.011363	0.011363	0.011398	0.011464	0.011531	0.011497
0.011397	0.011500	0.011501	0.011637	0.011662	0.011363	0.011564
0.011497	0.011497	0.011331	0.011513	0.011443	0.011469	0.011341
0.011508	0.011699	0.011441	0.011401	0.011296	0.011498	0.011363
0.011464	0.011397	0.011296	0.011398	0.011497	0.011500	0.011396
0.011296	0.011229	0.011363	0.011296	0.011228	0.011332	0.011498
0.011397	0.011329	0.011363	0.011396	0.011363	0.011296	0.011430
0.011363	0.011397	0.011396	0.011329	0.011396	0.011632	0.011228
0.011329	0.011666	0.011329	0.011363	0.011329	0.011632	0.011275
0.011566	0.011273	0.011211	0.011423	0.011508	0.011455	0.011186
0.011165	0.011430	0.011531	0.011637	0.011498	0.011636	0.011767
0.011666	0.011703	0.011666	0.011880	0.011632	0.012508	0.011667
0.012373	0.012173	0.012309	0.012580	0.011632	0.011329	0.011632
0.012071	0.012006	0.011598	0.011498	0.011565	0.011565	0.011535
0.011430	0.011434	0.011497	0.011464	0.011834	0.011901	0.011901
0.011802	0.011902	0.012004	0.011771	0.012071	0.012077	0.012138
0.012138	0.012172	0.012119	0.012138	0.012140	0.011430	0.011666
0.011733	0.012238	0.011739	0.011969	0.012442	0.011502	0.011397
0.011363	0.011363	0.011834	0.011883	0.011975	0.011699	0.011364
S161	0.011767	0.011767	0.011834	0.011800	0.011800	0.011868
0.011673	0.011571	0.011800	0.011363	0.011430	0.011397	0.011363
0.011329	0.011565	0.011296	0.011396	0.011464	0.011430	0.011396
0.011363	0.011397	0.011430	0.011363	0.011397	0.011396	0.011639
0.011565	0.011397	0.011296	0.011464	0.011464	0.011430	0.011464
0.011430	0.011531	0.011430	0.011430	0.011464	0.011430	0.011598
0.011296	0.011430	0.011464	0.011396	0.011430	0.011531	0.011464
0.011430	0.011599	0.011498	0.011699	0.011430	0.011699	0.011430
0.011396	0.011430	0.011565	0.011699	0.011733	0.011430	0.011531
0.011464	0.011531	0.011430	0.011665	0.011834	0.011430	0.011497
0.011397	0.011497	0.011464	0.011531	0.011464	0.011464	0.011464
0.011363	0.011396	0.011363	0.011397	0.011430	0.011465	0.011531
0.011464	0.011430	0.011497	0.011396	0.011464	0.011363	0.011430
0.011598	0.011531	0.011767	0.011598	0.011430	0.011666	0.011430
0.011617	0.011770	0.011664	0.011334	0.011359	0.011465	0.011493
0.012085	0.011518	0.011349	0.011666	0.011666	0.011396	0.011498
0.011497	0.011464	0.011531	0.011430	0.011531	0.011464	0.011464
0.011430	0.011464	0.011733	0.011598	0.011497	0.011464	0.011397
0.011363	0.011296	0.011430	0.011396	0.011531	0.011497	0.011430
0.011531	0.011598	0.011396	0.011396	0.011430	0.011430	0.011432
0.011500	0.011430	0.011430	0.011312	0.011386	0.011341	0.011430
0.011433	0.011370	0.011429	0.011405	0.011332	0.011369	0.011398
0.011600	0.011440	0.011474	0.011363	0.011371	0.011498	0.011094
0.000000	0.011465	0.011464	0.011565	0.011497	0.011868	0.011565
0.011632	0.011498	0.011430	0.011464	0.011329	0.011497	0.011363
0.011430	0.011430	0.011396	0.011363	0.011396	0.011363	0.011464
0.011430	0.011531	0.011464	0.011363	0.011465	0.011565	0.011568
0.011565	0.011464	0.011363	0.011297	0.011430	0.011363	0.011296
0.011400	0.011565	0.011363	0.011565	0.011329	0.011430	0.011397
0.011497	0.011396	0.011430	0.011397	0.011430	0.011296	0.011430
0.011464	0.011396	0.011464	0.011396	0.011430	0.011464	0.011699
0.011733	0.011666	0.011733	0.011566	0.011733	0.011430	0.011396
0.011365	0.011667	0.011518	0.011335	0.011740	0.011299	0.011454
0.011706	0.011638	0.011598	0.011800	0.011505	0.011666	0.011632
0.011565	0.011733	0.011559	0.011308	0.011396	0.011464	0.011535
0.011632	0.011533	0.011670	0.011738	0.011666	0.011733	0.011841
0.011569	0.011740	0.011670	0.011602	0.011869	0.011329	0.011532
0.011497	0.011430	0.011329	0.011329	0.011397	0.011397	0.011464

0.011396	0.011464	0.011363	0.011397	0.011397	0.011396	0.011464
0.011397	0.011767	0.011666	0.011699	0.011296	0.011397	0.011430
0.011430	0.011396	0.011464	0.011430	0.011363	0.011363	0.011363
0.011834	0.011430	0.011531	0.011329	0.011430	0.011599	0.011363
0.011363	0.011430	0.011396	0.011565	0.011363	0.011497	0.011632
0.011565	0.011397	0.011396	0.011329	0.011329	0.011498	0.011430
0.011329	0.011430	0.011296	0.011329	0.011464	0.011531	0.011363
0.011330	0.011464	0.011397	0.011296	0.011565	0.011296	0.011464
0.011540	0.011363	0.011598	0.011498	0.011464	0.011329	0.011497
0.011363	0.011699	0.011296	0.011498	0.011296	0.011296	0.011296
0.011531	0.011666	0.011430	0.011497	0.011329	0.011666	0.011296
0.011363	0.011363	0.011396	0.011497	0.011396	0.011497	0.011565
0.011565	0.011531	0.011430	0.011531	0.011497	0.011363	0.011329
0.011363	0.011363	0.011430	0.011296	0.011329	0.011497	0.011363
0.011329	0.011397	0.011531	0.011497	0.011464	0.011396	0.011397
0.011464	0.011396	0.011497	0.011363	0.011497	0.011598	0.011767
0.011397	0.011733	0.011405	0.017823	0.011322	0.011488	0.011688
0.011649	0.011694	0.011479	0.011525	0.011774	0.011716	0.011425
0.011535	0.011610	0.011333	0.011437	0.011255	0.011357	0.011404
0.011530	0.011507	0.011505	0.011325	0.011531	0.011565	0.011568
0.011438	0.011531	0.011531	0.011363	0.011464	0.011464	0.011430
0.011464	0.011396	0.011430	0.011464	0.011430	0.011363	0.011396
0.011396	0.011464	0.011363	0.011464	0.011497	0.011531	0.011430
0.011397	0.011363	0.011699	0.011699	0.011498	0.011532	0.011565
0.011329	0.011329	0.011363	0.011464	0.011329	0.011565	0.011565
0.011598	0.011598	0.011531	0.011632	0.011733	0.011634	0.011464
0.011464	0.011531	0.011565	0.011397	0.011464	0.011430	0.011464
0.011531	0.011531	0.011565	0.011531	0.011431	0.011632	0.011598
0.011467	0.011363	0.011464	0.011565	0.011464	0.011531	0.011632
0.011800	0.011505	0.011666	0.011632	0.011565	0.011733	0.011363
0.011532	0.011430	0.011396	0.011396	0.011464	0.011363	0.011464
0.011497	0.011531	0.011430	0.011397	0.011363	0.011699	0.011699
0.011498	0.011532	0.011598	0.011531	0.012104	0.011597	0.011518
0.011588	0.011547	0.011658	0.011546	0.011656	0.011578	0.011543
0.012058	0.011441	0.011575	0.011510	0.011784	0.011639	0.011742
0.012081	0.011550	0.011467	0.011464	0.011464	0.011430	0.011433
0.011329	0.011363	0.011497	0.011464	0.011466	0.011531	0.011499
0.011430	0.011363	0.011397	0.011430	0.011329	0.011397	0.011296
0.011363	0.011397	0.011363	0.011329	0.011430	0.011363	0.011397
0.011396	0.011329	0.011397	0.011430	0.011329	0.011531	0.011565
0.011464	0.011464	0.011464	0.011464	0.011396	0.011733	0.011430
0.011409	0.011397	0.011565	0.011570	0.011498	0.011497	0.011497
0.011498	0.011430	0.011396	0.011430	0.011531	0.011531	0.011464
0.011464	0.011598	0.011699	0.011497	0.011700	0.011700	0.011565
0.011396	0.011464	0.011498	0.011834	0.011464	0.011498	0.011464
0.011497	0.011363	0.011363	0.011599	0.011666	0.011565	0.011430
0.011531	0.011598	0.011464	0.011531	0.011497	0.011363	0.011632
0.011464	0.011430	0.011363	0.011598	0.011430	0.011531	0.011767
0.011396	0.011741	0.011767	0.011589	0.011173	0.011468	0.011318
0.011280	0.011761	0.011376	0.011507	0.011304	0.011468	0.011432
0.011570	0.011258	0.011506	0.011258	0.011384	0.011462	0.011547
0.011550	0.011454	0.011448	0.011448	0.011465	0.011531	0.011598
0.011565	0.011464	0.011568	0.011569	0.011705	0.011733	0.011430
0.011632	0.011498	0.011430	0.011464	0.011329	0.011497	0.011363
0.011430	0.011430	0.011396	0.011363	0.011396	0.011363	0.011464
0.011445	0.011445	0.011437	0.011471	0.011608	0.011573	0.011573
0.011571	0.011497	0.011319	0.011312	0.011433	0.011603	0.011603
0.011397	0.011467	0.011700	0.011669	0.011598	0.011632	0.011565
0.011497	0.011565	0.011565	0.011464	0.011969	0.011632	0.011700
0.011733	0.011702	0.011436	0.011511	0.011396	0.011598	0.011565

0.011600	0.011598	0.011632	0.011632	0.011808	0.011767	0.011407
0.011297	0.011303	0.011401	0.011366	0.011403	0.011322	0.011570
0.011438	0.011411	0.011251	0.011435	0.011430	0.011505	0.011397
0.011363	0.011296	0.011430	0.011396	0.011531	0.011497	0.011430
0.011531	0.011598	0.011396	0.011396	0.011430	0.011430	0.011432
0.011500	0.011430	0.011430	0.011465	0.011531	0.011598	0.011565
0.011464	0.011568	0.011569	0.011705	0.011733	0.011430	0.011632
0.011565	0.011565	0.011399	0.011583	0.011512	0.011538	0.011411
0.011577	0.011790	0.011526	0.011469	0.011363	0.011565	0.011430
0.011531	0.011464	0.011363	0.011465	0.011565	0.011568	0.011464
0.011363	0.011297	0.011430	0.011363	0.011296	0.011400	0.011565
0.011464	0.011396	0.011430	0.011464	0.011430	0.011363	0.011497
0.011430	0.011464	0.011464	0.011396	0.011464	0.011699	0.011296
0.011397	0.011733	0.011396	0.011430	0.011396	0.011699	0.011360
0.011679	0.011358	0.011311	0.011503	0.011590	0.011527	0.011269
0.011270	0.011497	0.011598	0.011705	0.011566	0.011704	0.011835
0.011734	0.011771	0.011733	0.011949	0.011699	0.012576	0.011734
0.012441	0.012241	0.012376	0.012648	0.011699	0.011397	0.011699
0.012138	0.012073	0.011666	0.011565	0.011632	0.011632	0.011602
0.011497	0.011502	0.011565	0.011531	0.011902	0.011969	0.011969
0.011869	0.011969	0.012072	0.011838	0.012139	0.012145	0.012206
0.012206	0.012240	0.012187	0.012206	0.012208	0.011497	0.011733
0.011801	0.012306	0.011807	0.012036	0.012509	0.011570	0.011464
0.011430	0.011430	0.011902	0.011965	0.012046	0.011767	0.011431
S162	0.011701	0.011701	0.011836	0.011802	0.011802	0.011869
0.011708	0.011606	0.011802	0.011296	0.011363	0.011330	0.011296
0.011330	0.011498	0.011296	0.011330	0.011397	0.011364	0.011330
0.011296	0.011330	0.011431	0.011296	0.011330	0.011330	0.011674
0.011566	0.011330	0.011229	0.011465	0.011397	0.011363	0.011397
0.011364	0.011465	0.011363	0.011364	0.011397	0.011363	0.011532
0.011296	0.011363	0.011397	0.011330	0.011431	0.011465	0.011397
0.011364	0.011532	0.011431	0.011701	0.011363	0.011633	0.011363
0.011330	0.011431	0.011498	0.011633	0.011734	0.011431	0.011532
0.011397	0.011465	0.011364	0.011599	0.011836	0.011364	0.011431
0.011330	0.011431	0.011397	0.011465	0.011397	0.011397	0.011397
0.011296	0.011330	0.011296	0.011397	0.011363	0.011398	0.011465
0.011397	0.011363	0.011431	0.011330	0.011465	0.011296	0.011330
0.011532	0.011465	0.011701	0.011498	0.011364	0.011599	0.011363
0.011543	0.011772	0.011588	0.011295	0.011247	0.011392	0.011415
0.012085	0.011467	0.011266	0.011599	0.011599	0.011397	0.011498
0.011431	0.011397	0.011465	0.011363	0.011465	0.011397	0.011397
0.011363	0.011465	0.011667	0.011532	0.011431	0.011397	0.011330
0.011296	0.011296	0.011364	0.011330	0.011532	0.011498	0.011431
0.011532	0.011599	0.011330	0.011330	0.011364	0.011363	0.011365
0.011433	0.011363	0.011363	0.011240	0.011313	0.011271	0.011363
0.011365	0.011301	0.011355	0.011336	0.011265	0.011300	0.011331
0.011600	0.011370	0.011405	0.011296	0.011302	0.011431	0.011397
0.011465	0.000000	0.000000	0.011498	0.011431	0.011802	0.011566
0.011633	0.011498	0.011363	0.011465	0.011262	0.011498	0.011296
0.011364	0.011363	0.011330	0.011296	0.011330	0.011296	0.011397
0.011364	0.011465	0.011397	0.011229	0.011398	0.011498	0.011501
0.011498	0.011397	0.011296	0.011229	0.011363	0.011296	0.011229
0.011333	0.011498	0.011296	0.011498	0.011262	0.011364	0.011330
0.011431	0.011330	0.011364	0.011330	0.011364	0.011229	0.011364
0.011397	0.011330	0.011397	0.011330	0.011363	0.011397	0.011633
0.011734	0.011667	0.011734	0.011500	0.011735	0.011363	0.011330
0.011365	0.011668	0.011427	0.011267	0.011741	0.011231	0.011336
0.011742	0.011640	0.011532	0.011735	0.011437	0.011600	0.011566
0.011498	0.011667	0.011524	0.011391	0.011439	0.011397	0.011468
0.011566	0.011466	0.011603	0.011672	0.011599	0.011667	0.011774

0.011502	0.011673	0.011604	0.011535	0.011803	0.011262	0.011466
0.011431	0.011364	0.011262	0.011262	0.011330	0.011330	0.011397
0.011330	0.011397	0.011296	0.011397	0.011330	0.011330	0.011397
0.011330	0.011768	0.011667	0.011701	0.011229	0.011330	0.011364
0.011364	0.011330	0.011397	0.011431	0.011296	0.011296	0.011296
0.011836	0.011431	0.011465	0.011262	0.011363	0.011532	0.011296
0.011296	0.011330	0.011330	0.011498	0.011296	0.011431	0.011566
0.011498	0.011330	0.011330	0.011262	0.011262	0.011431	0.011363
0.011262	0.011363	0.011229	0.011262	0.011397	0.011532	0.011296
0.011263	0.011397	0.011330	0.011229	0.011566	0.011229	0.011397
0.011472	0.011296	0.011599	0.011431	0.011465	0.011330	0.011431
0.011296	0.011633	0.011229	0.011498	0.011296	0.011296	0.011229
0.011465	0.011667	0.011364	0.011431	0.011262	0.011667	0.011296
0.011296	0.011296	0.011330	0.011431	0.011330	0.011431	0.011498
0.011465	0.011465	0.011363	0.011465	0.011431	0.011296	0.011262
0.011296	0.011296	0.011364	0.011296	0.011262	0.011431	0.011296
0.011330	0.011330	0.011465	0.011498	0.011397	0.011330	0.011330
0.011397	0.011330	0.011431	0.011296	0.011431	0.011532	0.011768
0.011330	0.011734	0.011337	0.017841	0.011322	0.011488	0.011690
0.011650	0.011695	0.011374	0.011350	0.011829	0.011718	0.011364
0.011487	0.011610	0.011255	0.011311	0.011333	0.011406	0.011361
0.011482	0.011470	0.011468	0.011259	0.011465	0.011498	0.011501
0.011370	0.011465	0.011465	0.011431	0.011397	0.011397	0.011364
0.011397	0.011330	0.011363	0.011397	0.011364	0.011296	0.011330
0.011330	0.011397	0.011296	0.011397	0.011431	0.011465	0.011363
0.011397	0.011364	0.011701	0.011701	0.011431	0.011465	0.011498
0.011262	0.011262	0.011296	0.011397	0.011262	0.011498	0.011498
0.011532	0.011532	0.011465	0.011566	0.011734	0.011567	0.011397
0.011397	0.011465	0.011498	0.011330	0.011397	0.011363	0.011397
0.011465	0.011465	0.011498	0.011465	0.011364	0.011566	0.011532
0.011400	0.011296	0.011397	0.011498	0.011397	0.011532	0.011566
0.011735	0.011437	0.011600	0.011566	0.011498	0.011667	0.011296
0.011466	0.011364	0.011330	0.011330	0.011397	0.011296	0.011397
0.011431	0.011465	0.011363	0.011397	0.011364	0.011701	0.011701
0.011431	0.011465	0.011532	0.011465	0.012106	0.011526	0.011449
0.011519	0.011478	0.011589	0.011478	0.011587	0.011510	0.011475
0.012060	0.011373	0.011507	0.011442	0.011717	0.011572	0.011675
0.012083	0.011551	0.011400	0.011397	0.011397	0.011431	0.011366
0.011262	0.011296	0.011431	0.011397	0.011399	0.011532	0.011499
0.011363	0.011296	0.011330	0.011363	0.011262	0.011330	0.011229
0.011363	0.011397	0.011296	0.011262	0.011364	0.011296	0.011330
0.011330	0.011262	0.011330	0.011364	0.011262	0.011465	0.011498
0.011397	0.011465	0.011397	0.011397	0.011330	0.011734	0.011363
0.011410	0.011330	0.011566	0.011571	0.011431	0.011431	0.011431
0.011431	0.011364	0.011330	0.011364	0.011465	0.011465	0.011397
0.011397	0.011532	0.011701	0.011431	0.011633	0.011633	0.011498
0.011330	0.011397	0.011431	0.011836	0.011397	0.011431	0.011397
0.011431	0.011363	0.011363	0.011667	0.011600	0.011633	0.011364
0.011465	0.011397	0.011397	0.011465	0.011431	0.011296	0.011633
0.011397	0.011363	0.011363	0.011532	0.011364	0.011465	0.011701
0.011330	0.011776	0.011768	0.011548	0.011286	0.011469	0.011267
0.011280	0.011761	0.011307	0.011507	0.011304	0.011417	0.011432
0.011519	0.011258	0.011394	0.011258	0.011276	0.011462	0.011426
0.011438	0.011297	0.011449	0.011307	0.011399	0.011532	0.011599
0.011566	0.011465	0.011569	0.011571	0.011673	0.011699	0.011364
0.011633	0.011498	0.011363	0.011465	0.011262	0.011498	0.011296
0.011364	0.011363	0.011330	0.011296	0.011330	0.011296	0.011397
0.011375	0.011375	0.011368	0.011403	0.011539	0.011504	0.011504
0.011503	0.011431	0.011245	0.011240	0.011365	0.011535	0.011535
0.011330	0.011399	0.011633	0.011601	0.011532	0.011566	0.011498

0.011431	0.011498	0.011498	0.011397	0.011971	0.011566	0.011633
0.011667	0.011740	0.011368	0.011442	0.011330	0.011532	0.011498
0.011533	0.011532	0.011566	0.011566	0.011729	0.011701	0.011339
0.011230	0.011234	0.011333	0.011299	0.011403	0.011210	0.011571
0.011370	0.011499	0.011251	0.011398	0.011364	0.011437	0.011330
0.011296	0.011296	0.011364	0.011330	0.011532	0.011498	0.011431
0.011532	0.011599	0.011330	0.011330	0.011364	0.011363	0.011365
0.011433	0.011363	0.011363	0.011399	0.011532	0.011599	0.011566
0.011465	0.011569	0.011571	0.011673	0.011699	0.011364	0.011566
0.011498	0.011498	0.011331	0.011514	0.011443	0.011470	0.011341
0.011509	0.011655	0.011399	0.011401	0.011296	0.011498	0.011364
0.011465	0.011397	0.011229	0.011398	0.011498	0.011501	0.011397
0.011296	0.011229	0.011363	0.011296	0.011229	0.011333	0.011498
0.011397	0.011330	0.011363	0.011397	0.011364	0.011296	0.011498
0.011363	0.011397	0.011397	0.011330	0.011397	0.011633	0.011296
0.011330	0.011734	0.011330	0.011330	0.011330	0.011701	0.011275
0.011791	0.011273	0.011261	0.011503	0.011589	0.011528	0.011227
0.011218	0.011498	0.011599	0.011707	0.011567	0.011705	0.011836
0.011735	0.011773	0.011734	0.011985	0.011701	0.012579	0.011736
0.012444	0.012243	0.012379	0.012617	0.011701	0.011397	0.011701
0.012140	0.012075	0.011667	0.011566	0.011633	0.011633	0.011603
0.011498	0.011503	0.011566	0.011532	0.011903	0.011971	0.011970
0.011871	0.011971	0.012040	0.011840	0.012141	0.012147	0.012208
0.012208	0.012242	0.012190	0.012208	0.012210	0.011431	0.011735
0.011802	0.012309	0.011808	0.012038	0.012512	0.011571	0.011465
0.011431	0.011431	0.011903	0.011924	0.012045	0.011768	0.011432
S163	0.011699	0.011699	0.011834	0.011800	0.011800	0.011867
0.011707	0.011604	0.011800	0.011296	0.011363	0.011329	0.011296
0.011329	0.011497	0.011296	0.011329	0.011396	0.011363	0.011329
0.011296	0.011329	0.011430	0.011296	0.011329	0.011329	0.011672
0.011564	0.011329	0.011228	0.011464	0.011396	0.011363	0.011396
0.011363	0.011464	0.011363	0.011363	0.011396	0.011363	0.011531
0.011296	0.011363	0.011396	0.011329	0.011430	0.011464	0.011396
0.011363	0.011531	0.011430	0.011699	0.011363	0.011632	0.011363
0.011329	0.011430	0.011497	0.011632	0.011733	0.011430	0.011531
0.011396	0.011464	0.011363	0.011598	0.011834	0.011363	0.011430
0.011329	0.011430	0.011396	0.011464	0.011396	0.011396	0.011396
0.011296	0.011329	0.011296	0.011396	0.011363	0.011397	0.011464
0.011396	0.011363	0.011430	0.011329	0.011464	0.011296	0.011363
0.011531	0.011464	0.011699	0.011531	0.011363	0.011598	0.011363
0.011542	0.011770	0.011588	0.011295	0.011247	0.011391	0.011415
0.012085	0.011467	0.011266	0.011598	0.011598	0.011396	0.011497
0.011430	0.011396	0.011464	0.011363	0.011464	0.011396	0.011396
0.011363	0.011464	0.011666	0.011531	0.011430	0.011397	0.011329
0.011296	0.011296	0.011363	0.011329	0.011531	0.011497	0.011430
0.011531	0.011598	0.011329	0.011329	0.011363	0.011363	0.011365
0.011433	0.011363	0.011363	0.011240	0.011313	0.011271	0.011363
0.011365	0.011301	0.011355	0.011336	0.011264	0.011300	0.011330
0.011600	0.011370	0.011405	0.011296	0.011302	0.011430	0.011396
0.011464	0.000000	0.000000	0.011497	0.011430	0.011800	0.011565
0.011632	0.011497	0.011363	0.011464	0.011262	0.011497	0.011296
0.011363	0.011363	0.011329	0.011296	0.011329	0.011296	0.011396
0.011363	0.011464	0.011396	0.011228	0.011398	0.011497	0.011500
0.011497	0.011396	0.011296	0.011229	0.011363	0.011296	0.011228
0.011332	0.011497	0.011296	0.011497	0.011262	0.011363	0.011329
0.011430	0.011329	0.011363	0.011329	0.011363	0.011228	0.011363
0.011396	0.011329	0.011396	0.011329	0.011363	0.011396	0.011632
0.011733	0.011665	0.011733	0.011499	0.011733	0.011363	0.011329
0.011364	0.011667	0.011426	0.011266	0.011740	0.011231	0.011336
0.011740	0.011638	0.011531	0.011733	0.011436	0.011598	0.011565

0.011497	0.011665	0.011523	0.011390	0.011438	0.011396	0.011467
0.011565	0.011465	0.011602	0.011670	0.011598	0.011666	0.011773
0.011501	0.011672	0.011602	0.011534	0.011801	0.011262	0.011465
0.011430	0.011363	0.011262	0.011262	0.011329	0.011329	0.011396
0.011329	0.011396	0.011296	0.011396	0.011329	0.011329	0.011396
0.011329	0.011766	0.011666	0.011699	0.011228	0.011329	0.011363
0.011363	0.011329	0.011396	0.011430	0.011296	0.011296	0.011296
0.011834	0.011430	0.011464	0.011262	0.011363	0.011531	0.011296
0.011296	0.011363	0.011329	0.011497	0.011296	0.011430	0.011564
0.011497	0.011329	0.011329	0.011262	0.011262	0.011430	0.011363
0.011262	0.011363	0.011228	0.011262	0.011396	0.011531	0.011296
0.011262	0.011396	0.011329	0.011228	0.011565	0.011228	0.011396
0.011471	0.011296	0.011598	0.011430	0.011464	0.011329	0.011430
0.011296	0.011632	0.011228	0.011497	0.011296	0.011296	0.011228
0.011464	0.011666	0.011363	0.011430	0.011262	0.011666	0.011296
0.011296	0.011296	0.011329	0.011430	0.011329	0.011430	0.011497
0.011497	0.011464	0.011363	0.011464	0.011430	0.011296	0.011262
0.011296	0.011296	0.011363	0.011296	0.011262	0.011430	0.011296
0.011329	0.011329	0.011464	0.011497	0.011396	0.011329	0.011329
0.011396	0.011329	0.011430	0.011296	0.011430	0.011531	0.011766
0.011329	0.011733	0.011336	0.017823	0.011322	0.011488	0.011688
0.011648	0.011693	0.011374	0.011350	0.011829	0.011716	0.011364
0.011487	0.011610	0.011255	0.011311	0.011333	0.011406	0.011361
0.011482	0.011470	0.011468	0.011259	0.011464	0.011497	0.011500
0.011370	0.011464	0.011464	0.011430	0.011396	0.011397	0.011363
0.011396	0.011329	0.011363	0.011396	0.011363	0.011296	0.011329
0.011329	0.011396	0.011296	0.011397	0.011430	0.011464	0.011363
0.011396	0.011363	0.011699	0.011699	0.011430	0.011464	0.011497
0.011262	0.011262	0.011296	0.011396	0.011262	0.011497	0.011497
0.011531	0.011531	0.011464	0.011565	0.011733	0.011566	0.011396
0.011396	0.011464	0.011497	0.011329	0.011396	0.011363	0.011396
0.011464	0.011464	0.011497	0.011464	0.011364	0.011564	0.011531
0.011399	0.011296	0.011396	0.011497	0.011397	0.011531	0.011564
0.011733	0.011436	0.011598	0.011565	0.011497	0.011665	0.011296
0.011465	0.011363	0.011329	0.011329	0.011396	0.011296	0.011397
0.011430	0.011464	0.011363	0.011396	0.011363	0.011699	0.011699
0.011430	0.011464	0.011531	0.011464	0.012104	0.011525	0.011448
0.011517	0.011477	0.011588	0.011477	0.011586	0.011509	0.011474
0.012058	0.011372	0.011506	0.011441	0.011715	0.011571	0.011673
0.012081	0.011550	0.011399	0.011396	0.011396	0.011430	0.011365
0.011262	0.011296	0.011430	0.011396	0.011399	0.011531	0.011498
0.011363	0.011296	0.011329	0.011363	0.011262	0.011329	0.011228
0.011363	0.011396	0.011296	0.011262	0.011363	0.011296	0.011329
0.011329	0.011262	0.011329	0.011363	0.011262	0.011464	0.011497
0.011396	0.011464	0.011396	0.011396	0.011329	0.011733	0.011363
0.011409	0.011329	0.011564	0.011569	0.011430	0.011430	0.011430
0.011430	0.011363	0.011329	0.011363	0.011464	0.011464	0.011396
0.011396	0.011531	0.011699	0.011430	0.011632	0.011632	0.011497
0.011329	0.011396	0.011430	0.011834	0.011397	0.011430	0.011396
0.011430	0.011363	0.011363	0.011666	0.011598	0.011632	0.011363
0.011464	0.011397	0.011396	0.011464	0.011430	0.011296	0.011632
0.011396	0.011363	0.011363	0.011531	0.011363	0.011464	0.011699
0.011329	0.011775	0.011766	0.011548	0.011286	0.011468	0.011267
0.011280	0.011761	0.011306	0.011507	0.011304	0.011417	0.011432
0.011519	0.011258	0.011394	0.011258	0.011276	0.011462	0.011425
0.011437	0.011296	0.011448	0.011342	0.011398	0.011531	0.011598
0.011564	0.011464	0.011568	0.011569	0.011705	0.011697	0.011363
0.011632	0.011497	0.011363	0.011464	0.011262	0.011497	0.011296
0.011363	0.011363	0.011329	0.011296	0.011329	0.011296	0.011396
0.011375	0.011375	0.011368	0.011403	0.011539	0.011504	0.011504

0.011503	0.011430	0.011245	0.011240	0.011365	0.011535	0.011535
0.011329	0.011399	0.011632	0.011601	0.011531	0.011565	0.011497
0.011430	0.011497	0.011497	0.011396	0.011969	0.011565	0.011632
0.011665	0.011738	0.011368	0.011441	0.011329	0.011531	0.011497
0.011532	0.011531	0.011565	0.011565	0.011729	0.011699	0.011338
0.011230	0.011234	0.011333	0.011298	0.011402	0.011210	0.011570
0.011369	0.011499	0.011251	0.011397	0.011363	0.011437	0.011329
0.011296	0.011296	0.011363	0.011329	0.011531	0.011497	0.011430
0.011531	0.011598	0.011329	0.011329	0.011363	0.011363	0.011365
0.011433	0.011363	0.011363	0.011398	0.011531	0.011598	0.011564
0.011464	0.011568	0.011569	0.011705	0.011697	0.011363	0.011564
0.011497	0.011497	0.011331	0.011513	0.011443	0.011469	0.011340
0.011508	0.011653	0.011399	0.011400	0.011296	0.011497	0.011363
0.011464	0.011396	0.011228	0.011398	0.011497	0.011500	0.011396
0.011296	0.011229	0.011363	0.011296	0.011228	0.011332	0.011497
0.011396	0.011329	0.011363	0.011396	0.011363	0.011296	0.011497
0.011363	0.011396	0.011396	0.011329	0.011396	0.011632	0.011296
0.011329	0.011733	0.011329	0.011363	0.011329	0.011699	0.011275
0.011791	0.011273	0.011261	0.011503	0.011589	0.011527	0.011227
0.011218	0.011497	0.011598	0.011705	0.011565	0.011703	0.011834
0.011733	0.011771	0.011733	0.011983	0.011699	0.012575	0.011734
0.012441	0.012241	0.012376	0.012647	0.011699	0.011396	0.011699
0.012138	0.012073	0.011665	0.011565	0.011632	0.011632	0.011602
0.011497	0.011501	0.011564	0.011531	0.011901	0.011969	0.011968
0.011869	0.011969	0.012072	0.011838	0.012138	0.012144	0.012205
0.012205	0.012239	0.012187	0.012206	0.012208	0.011430	0.011733
0.011800	0.012306	0.011806	0.012036	0.012509	0.011570	0.011464
0.011430	0.011430	0.011901	0.011924	0.012045	0.011766	0.011431
S164 0.011664	0.011664	0.012135	0.011899	0.011899	0.011967	
0.011773	0.011705	0.011967	0.011396	0.011463	0.011430	0.011396
0.011429	0.011597	0.011396	0.011429	0.011496	0.011463	0.011429
0.011396	0.011429	0.011530	0.011396	0.011430	0.011429	0.011739
0.011664	0.011429	0.011329	0.011564	0.011496	0.011463	0.011496
0.011463	0.011564	0.011463	0.011463	0.011496	0.011463	0.011631
0.011396	0.011463	0.011496	0.011429	0.011530	0.011563	0.011496
0.011463	0.011631	0.011463	0.011799	0.011463	0.011731	0.011463
0.011429	0.011530	0.011597	0.011731	0.011832	0.011530	0.011631
0.011496	0.011564	0.011463	0.011698	0.011933	0.011463	0.011530
0.011429	0.011530	0.011496	0.011564	0.011496	0.011496	0.011496
0.011396	0.011429	0.011396	0.011496	0.011463	0.011498	0.011564
0.011496	0.011396	0.011530	0.011429	0.011563	0.011396	0.011463
0.011631	0.011564	0.011799	0.011631	0.011463	0.011698	0.011463
0.011579	0.011807	0.011626	0.011295	0.011321	0.011465	0.011493
0.012158	0.011569	0.011307	0.011698	0.011698	0.011496	0.011597
0.011530	0.011429	0.011429	0.011463	0.011564	0.011496	0.011228
0.011262	0.011564	0.011698	0.011631	0.011530	0.011497	0.011429
0.011396	0.011396	0.011463	0.011429	0.011631	0.011597	0.011530
0.011631	0.011698	0.011429	0.011429	0.011463	0.011463	0.011465
0.011533	0.011463	0.011463	0.011276	0.011386	0.011306	0.011464
0.011467	0.011404	0.011429	0.011440	0.011332	0.011334	0.011398
0.011636	0.011405	0.011473	0.011396	0.011406	0.011531	0.011497
0.011565	0.011498	0.011497	0.000000	0.011396	0.011899	0.011698
0.011731	0.011597	0.011463	0.011564	0.011362	0.011597	0.011396
0.011463	0.011463	0.011429	0.011396	0.011429	0.011396	0.011497
0.011463	0.011564	0.011497	0.011396	0.011498	0.011597	0.011601
0.011597	0.011497	0.011396	0.011330	0.011463	0.011396	0.011329
0.011434	0.011597	0.011396	0.011597	0.011362	0.011463	0.011429
0.011530	0.011362	0.011463	0.011429	0.011463	0.011329	0.011463
0.011497	0.011429	0.011497	0.011429	0.011262	0.011497	0.011732
0.011832	0.011765	0.011833	0.011599	0.011833	0.011329	0.011429

0.011465	0.011666	0.011517	0.011335	0.011841	0.011333	0.011414
0.011807	0.011740	0.011597	0.011765	0.011470	0.011698	0.011664
0.011597	0.011765	0.011451	0.011349	0.011437	0.011497	0.011500
0.011665	0.011364	0.011702	0.011771	0.011497	0.011698	0.011704
0.011399	0.011739	0.011534	0.011601	0.011834	0.011362	0.011532
0.011530	0.011463	0.011362	0.011362	0.011429	0.011429	0.011496
0.011429	0.011496	0.011396	0.011496	0.011429	0.011429	0.011496
0.011429	0.011866	0.011765	0.011799	0.011329	0.011429	0.011463
0.011463	0.011429	0.011496	0.011530	0.011396	0.011396	0.011396
0.011933	0.011563	0.011564	0.011362	0.011463	0.011631	0.011396
0.011396	0.011463	0.011429	0.011597	0.011396	0.011530	0.011664
0.011597	0.011429	0.011429	0.011362	0.011362	0.011530	0.011463
0.011362	0.011463	0.011329	0.011362	0.011496	0.011631	0.011396
0.011363	0.011496	0.011430	0.011329	0.011664	0.011329	0.011496
0.011505	0.011396	0.011698	0.011530	0.011564	0.011429	0.011530
0.011396	0.011731	0.011329	0.011597	0.011396	0.011396	0.011329
0.011563	0.011766	0.011463	0.011530	0.011362	0.011765	0.011396
0.011396	0.011396	0.011429	0.011530	0.011429	0.011530	0.011597
0.011597	0.011564	0.011463	0.011564	0.011530	0.011396	0.011362
0.011396	0.011396	0.011463	0.011396	0.011362	0.011530	0.011396
0.011429	0.011429	0.011564	0.011597	0.011496	0.011429	0.011429
0.011496	0.011429	0.011530	0.011396	0.011530	0.011631	0.011933
0.011429	0.011832	0.011405	0.017898	0.011322	0.011427	0.011758
0.011718	0.011764	0.011427	0.011408	0.011829	0.011785	0.011425
0.011582	0.011549	0.011294	0.011353	0.011411	0.011455	0.011318
0.011576	0.011544	0.011542	0.011457	0.011564	0.011463	0.011534
0.011438	0.011564	0.011564	0.011530	0.011496	0.011497	0.011463
0.011497	0.011362	0.011463	0.011497	0.011463	0.011396	0.011295
0.011295	0.011497	0.011396	0.011497	0.011530	0.011564	0.011329
0.011496	0.011463	0.011799	0.011799	0.011530	0.011564	0.011597
0.011362	0.011362	0.011396	0.011497	0.011362	0.011597	0.011597
0.011631	0.011597	0.011564	0.011664	0.011833	0.011666	0.011496
0.011497	0.011564	0.011597	0.011429	0.011497	0.011463	0.011496
0.011564	0.011564	0.011597	0.011564	0.011464	0.011665	0.011564
0.011500	0.011396	0.011497	0.011597	0.011497	0.011631	0.011664
0.011765	0.011470	0.011698	0.011664	0.011597	0.011765	0.011396
0.011565	0.011464	0.011295	0.011295	0.011497	0.011396	0.011497
0.011530	0.011564	0.011329	0.011496	0.011463	0.011799	0.011799
0.011530	0.011564	0.011496	0.011429	0.012170	0.011560	0.011412
0.011552	0.011511	0.011622	0.011476	0.011620	0.011508	0.011543
0.012056	0.011268	0.011403	0.011406	0.011680	0.011638	0.011740
0.012080	0.011550	0.011736	0.011799	0.011664	0.011664	0.011702
0.011362	0.011530	0.011664	0.011631	0.011635	0.011631	0.011599
0.011463	0.011396	0.011429	0.011463	0.011362	0.011429	0.011329
0.011463	0.011496	0.011396	0.011362	0.011463	0.011396	0.011429
0.011429	0.011362	0.011429	0.011463	0.011362	0.011564	0.011597
0.011496	0.011564	0.011496	0.011496	0.011429	0.011832	0.011463
0.011478	0.011429	0.011665	0.011637	0.011530	0.011530	0.011530
0.011530	0.011463	0.011429	0.011463	0.011564	0.011564	0.011496
0.011496	0.011631	0.011799	0.011530	0.011664	0.011664	0.011597
0.011429	0.011496	0.011530	0.011933	0.011497	0.011530	0.011496
0.011530	0.011463	0.011463	0.011765	0.011698	0.011731	0.011463
0.011564	0.011631	0.011496	0.011564	0.011530	0.011396	0.011731
0.011496	0.011463	0.011463	0.011631	0.011463	0.011563	0.011799
0.011496	0.011841	0.011866	0.011588	0.011286	0.011570	0.011318
0.011335	0.011800	0.011376	0.011551	0.011353	0.011467	0.011485
0.011621	0.011324	0.011450	0.011324	0.011330	0.011529	0.011506
0.011512	0.011375	0.011518	0.011412	0.011498	0.011631	0.011698
0.011698	0.011564	0.011669	0.011670	0.011806	0.011838	0.011496
0.011731	0.011597	0.011463	0.011564	0.011362	0.011597	0.011396

0.011463	0.011463	0.011429	0.011396	0.011429	0.011396	0.011497
0.011444	0.011410	0.011471	0.011471	0.011607	0.011607	0.011572
0.011537	0.011531	0.011245	0.011240	0.011467	0.011636	0.011637
0.011430	0.011500	0.011733	0.011702	0.011632	0.011666	0.011463
0.011530	0.011530	0.011530	0.011497	0.012034	0.011664	0.011732
0.011765	0.011843	0.011469	0.011545	0.011429	0.011362	0.011463
0.011632	0.011530	0.011664	0.011664	0.011768	0.011664	0.011407
0.011331	0.011337	0.011400	0.011366	0.011470	0.011284	0.011672
0.011437	0.011543	0.011298	0.011360	0.011464	0.011505	0.011429
0.011396	0.011396	0.011463	0.011429	0.011631	0.011597	0.011530
0.011631	0.011698	0.011429	0.011429	0.011463	0.011463	0.011465
0.011533	0.011463	0.011463	0.011498	0.011631	0.011698	0.011698
0.011564	0.011669	0.011670	0.011806	0.011838	0.011496	0.011664
0.011463	0.011463	0.011432	0.011617	0.011547	0.011571	0.011375
0.011611	0.011698	0.011568	0.011502	0.011396	0.011597	0.011463
0.011564	0.011497	0.011396	0.011498	0.011597	0.011601	0.011497
0.011396	0.011330	0.011463	0.011396	0.011329	0.011434	0.011597
0.011497	0.011362	0.011463	0.011497	0.011463	0.011396	0.011598
0.011463	0.011496	0.011496	0.011429	0.011496	0.011731	0.011396
0.011429	0.011832	0.011429	0.011463	0.011429	0.011799	0.011317
0.011791	0.011273	0.011361	0.011543	0.011630	0.011599	0.011227
0.011323	0.011597	0.011698	0.011807	0.011666	0.011771	0.011935
0.011834	0.011838	0.011832	0.012051	0.011799	0.012606	0.011835
0.012574	0.012274	0.012408	0.012747	0.011799	0.011497	0.011799
0.012204	0.012139	0.011765	0.011665	0.011733	0.011733	0.011703
0.011597	0.011569	0.011664	0.011632	0.012002	0.012068	0.012069
0.011968	0.012069	0.012173	0.011906	0.012205	0.012211	0.012272
0.012272	0.012306	0.012152	0.012273	0.012274	0.011396	0.011799
0.011901	0.012404	0.011874	0.012135	0.012609	0.011671	0.011564
0.011530	0.011530	0.012001	0.011965	0.012116	0.011866	0.011532
S165	0.011463	0.011463	0.012068	0.011832	0.011832	0.011900
0.011739	0.011671	0.011899	0.011329	0.011396	0.011363	0.011329
0.011362	0.011530	0.011329	0.011362	0.011429	0.011396	0.011362
0.011329	0.011362	0.011463	0.011329	0.011362	0.011362	0.011705
0.011597	0.011362	0.011262	0.011496	0.011429	0.011396	0.011429
0.011396	0.011496	0.011396	0.011396	0.011429	0.011396	0.011564
0.011329	0.011396	0.011429	0.011362	0.011463	0.011496	0.011429
0.011396	0.011564	0.011463	0.011731	0.011396	0.011664	0.011396
0.011362	0.011463	0.011530	0.011664	0.011765	0.011463	0.011564
0.011429	0.011496	0.011396	0.011631	0.011866	0.011396	0.011463
0.011362	0.011463	0.011429	0.011496	0.011429	0.011429	0.011429
0.011329	0.011362	0.011329	0.011429	0.011396	0.011430	0.011496
0.011429	0.011396	0.011463	0.011362	0.011496	0.011329	0.011396
0.011564	0.011496	0.011731	0.011564	0.011396	0.011631	0.011396
0.011542	0.011770	0.011588	0.011295	0.011321	0.011428	0.011493
0.012158	0.011518	0.011307	0.011631	0.011631	0.011429	0.011530
0.011463	0.011362	0.011295	0.011396	0.011496	0.011429	0.011295
0.011329	0.011496	0.011631	0.011564	0.011463	0.011429	0.011362
0.011329	0.011329	0.011396	0.011362	0.011564	0.011530	0.011463
0.011564	0.011631	0.011362	0.011362	0.011396	0.011396	0.011398
0.011466	0.011396	0.011396	0.011276	0.011349	0.011306	0.011396
0.011399	0.011335	0.011392	0.011371	0.011298	0.011334	0.011364
0.011636	0.011405	0.011439	0.011329	0.011337	0.011464	0.011430
0.011497	0.011431	0.011430	0.011396	0.000000	0.011832	0.011631
0.011664	0.011530	0.011396	0.011497	0.011295	0.011530	0.011329
0.011396	0.011396	0.011362	0.011329	0.011362	0.011329	0.011429
0.011396	0.011497	0.011429	0.011329	0.011431	0.011530	0.011533
0.011530	0.011430	0.011329	0.011263	0.011396	0.011329	0.011262
0.011366	0.011530	0.011329	0.011530	0.011295	0.011396	0.011362
0.011463	0.011362	0.011396	0.011362	0.011396	0.011262	0.011396

0.011429	0.011362	0.011429	0.011362	0.011262	0.011429	0.011665
0.011765	0.011698	0.011765	0.011532	0.011765	0.011262	0.011362
0.011398	0.011707	0.011426	0.011300	0.011773	0.011265	0.011336
0.011773	0.011672	0.011530	0.011698	0.011197	0.011631	0.011597
0.011530	0.011698	0.011380	0.011225	0.011270	0.011429	0.011432
0.011597	0.011364	0.011601	0.011703	0.011497	0.011631	0.011670
0.011399	0.011671	0.011399	0.011567	0.011766	0.011295	0.011498
0.011463	0.011396	0.011295	0.011295	0.011362	0.011362	0.011429
0.011362	0.011429	0.011329	0.011429	0.011362	0.011362	0.011429
0.011362	0.011799	0.011698	0.011731	0.011262	0.011362	0.011396
0.011396	0.011362	0.011429	0.011463	0.011329	0.011329	0.011329
0.011866	0.011496	0.011496	0.011295	0.011396	0.011564	0.011329
0.011329	0.011396	0.011362	0.011530	0.011329	0.011463	0.011597
0.011530	0.011362	0.011362	0.011295	0.011295	0.011463	0.011396
0.011295	0.011396	0.011262	0.011295	0.011429	0.011564	0.011329
0.011295	0.011429	0.011363	0.011262	0.011597	0.011262	0.011429
0.011437	0.011329	0.011631	0.011463	0.011496	0.011362	0.011463
0.011329	0.011664	0.011262	0.011530	0.011329	0.011329	0.011262
0.011496	0.011698	0.011396	0.011463	0.011295	0.011698	0.011329
0.011329	0.011329	0.011362	0.011463	0.011362	0.011463	0.011530
0.011530	0.011496	0.011396	0.011496	0.011463	0.011329	0.011295
0.011329	0.011329	0.011396	0.011329	0.011295	0.011463	0.011329
0.011362	0.011362	0.011496	0.011530	0.011429	0.011362	0.011362
0.011429	0.011362	0.011463	0.011329	0.011463	0.011564	0.011866
0.011362	0.011765	0.011371	0.017898	0.011257	0.011488	0.011723
0.011683	0.011728	0.011374	0.011350	0.011774	0.011751	0.011364
0.011534	0.011549	0.011255	0.011311	0.011372	0.011356	0.011318
0.011529	0.011544	0.011542	0.011325	0.011496	0.011262	0.011466
0.011369	0.011497	0.011497	0.011463	0.011429	0.011430	0.011396
0.011429	0.011362	0.011396	0.011429	0.011396	0.011329	0.011228
0.011228	0.011429	0.011329	0.011430	0.011463	0.011497	0.011262
0.011429	0.011396	0.011732	0.011732	0.011463	0.011497	0.011530
0.011295	0.011295	0.011329	0.011429	0.011295	0.011530	0.011530
0.011564	0.011530	0.011496	0.011597	0.011765	0.011599	0.011429
0.011429	0.011497	0.011530	0.011362	0.011429	0.011396	0.011429
0.011497	0.011497	0.011530	0.011497	0.011397	0.011597	0.011564
0.011433	0.011329	0.011429	0.011530	0.011430	0.011564	0.011597
0.011698	0.011197	0.011631	0.011597	0.011530	0.011698	0.011329
0.011498	0.011396	0.011228	0.011228	0.011429	0.011329	0.011430
0.011463	0.011497	0.011262	0.011429	0.011396	0.011732	0.011732
0.011463	0.011497	0.011228	0.011161	0.012103	0.011489	0.011236
0.011517	0.011476	0.011552	0.011441	0.011550	0.011439	0.011508
0.011987	0.011164	0.011300	0.011337	0.011645	0.011570	0.011672
0.012012	0.011437	0.011669	0.011731	0.011597	0.011597	0.011635
0.011295	0.011463	0.011597	0.011564	0.011567	0.011564	0.011532
0.011396	0.011329	0.011362	0.011396	0.011295	0.011362	0.011262
0.011396	0.011429	0.011329	0.011295	0.011396	0.011329	0.011362
0.011362	0.011295	0.011362	0.011396	0.011295	0.011496	0.011530
0.011429	0.011496	0.011429	0.011429	0.011362	0.011765	0.011396
0.011408	0.011362	0.011598	0.011603	0.011463	0.011463	0.011463
0.011463	0.011396	0.011362	0.011396	0.011496	0.011496	0.011429
0.011429	0.011564	0.011731	0.011463	0.011664	0.011664	0.011530
0.011362	0.011429	0.011463	0.011866	0.011430	0.011463	0.011429
0.011463	0.011396	0.011396	0.011698	0.011631	0.011664	0.011396
0.011496	0.011564	0.011429	0.011496	0.011463	0.011329	0.011664
0.011429	0.011396	0.011396	0.011564	0.011396	0.011496	0.011732
0.011429	0.011807	0.011799	0.011548	0.011286	0.011502	0.011318
0.011335	0.011839	0.011341	0.011551	0.011353	0.011416	0.011432
0.011570	0.011258	0.011394	0.011258	0.011276	0.011462	0.011466
0.011475	0.011375	0.011483	0.011412	0.011431	0.011564	0.011631

0.011631	0.011497	0.011601	0.011603	0.011738	0.011767	0.011429
0.011664	0.011530	0.011396	0.011497	0.011295	0.011530	0.011329
0.011396	0.011396	0.011362	0.011329	0.011362	0.011329	0.011429
0.011409	0.011410	0.011403	0.011437	0.011573	0.011538	0.011538
0.011537	0.011464	0.011245	0.011276	0.011399	0.011568	0.011569
0.011363	0.011433	0.011666	0.011635	0.011565	0.011599	0.011194
0.011463	0.011463	0.011463	0.011429	0.011967	0.011597	0.011664
0.011698	0.011772	0.011401	0.011476	0.011362	0.011429	0.011262
0.011565	0.011463	0.011597	0.011597	0.011689	0.011597	0.011372
0.011263	0.011233	0.011332	0.011332	0.011436	0.011209	0.011604
0.011403	0.011411	0.011108	0.011285	0.011396	0.011471	0.011362
0.011329	0.011329	0.011396	0.011362	0.011564	0.011530	0.011463
0.011564	0.011631	0.011362	0.011362	0.011396	0.011396	0.011398
0.011466	0.011396	0.011396	0.011431	0.011564	0.011631	0.011631
0.011497	0.011601	0.011603	0.011738	0.011767	0.011429	0.011597
0.011262	0.011262	0.011365	0.011547	0.011477	0.011503	0.011376
0.011542	0.011744	0.011568	0.011434	0.011329	0.011530	0.011396
0.011497	0.011429	0.011329	0.011431	0.011530	0.011533	0.011430
0.011329	0.011263	0.011396	0.011329	0.011262	0.011366	0.011530
0.011429	0.011362	0.011396	0.011429	0.011396	0.011329	0.011530
0.011396	0.011429	0.011429	0.011362	0.011429	0.011664	0.011329
0.011362	0.011765	0.011362	0.011396	0.011362	0.011731	0.011317
0.011791	0.011273	0.011311	0.011584	0.011630	0.011563	0.011227
0.011270	0.011530	0.011631	0.011739	0.011599	0.011737	0.011867
0.011767	0.011804	0.011765	0.012017	0.011732	0.012539	0.011768
0.012507	0.012207	0.012341	0.012680	0.011731	0.011430	0.011732
0.012137	0.012106	0.011698	0.011597	0.011665	0.011665	0.011635
0.011530	0.011535	0.011597	0.011565	0.011934	0.012000	0.012002
0.011901	0.012002	0.012105	0.011872	0.012137	0.012144	0.012205
0.012205	0.012239	0.012186	0.012205	0.012206	0.011127	0.011732
0.011834	0.012337	0.011806	0.012068	0.012541	0.011604	0.011497
0.011463	0.011463	0.011934	0.011842	0.012080	0.011799	0.011465
S166	0.012101	0.012101	0.012438	0.012202	0.012202	0.012269
0.012045	0.011977	0.012269	0.011698	0.011765	0.011733	0.011698
0.011732	0.011900	0.011698	0.011664	0.011799	0.011765	0.011732
0.011698	0.011732	0.011832	0.011699	0.011665	0.011732	0.012011
0.011900	0.011732	0.011631	0.011866	0.011732	0.011765	0.011732
0.011765	0.011799	0.011765	0.011765	0.011732	0.011765	0.011933
0.011698	0.011698	0.011732	0.011731	0.011832	0.011799	0.011799
0.011765	0.011866	0.011833	0.012101	0.011698	0.011967	0.011698
0.011732	0.011833	0.011900	0.011967	0.012135	0.011832	0.011933
0.011731	0.011799	0.011765	0.011933	0.012236	0.011765	0.011832
0.011732	0.011832	0.011799	0.011866	0.011799	0.011799	0.011799
0.011698	0.011731	0.011698	0.011799	0.011765	0.011733	0.011866
0.011799	0.011765	0.011765	0.011731	0.011866	0.011631	0.011765
0.011933	0.011866	0.012101	0.011933	0.011765	0.011866	0.011765
0.011876	0.012107	0.011966	0.011607	0.011695	0.011760	0.011848
0.012525	0.011874	0.011719	0.011933	0.012000	0.011799	0.011900
0.011832	0.011799	0.011866	0.011765	0.011866	0.011732	0.011799
0.011765	0.011866	0.012068	0.011933	0.011832	0.011799	0.011665
0.011698	0.011698	0.011698	0.011698	0.011934	0.011900	0.011833
0.011934	0.012001	0.011732	0.011732	0.011766	0.011765	0.011770
0.011838	0.011766	0.011766	0.011600	0.011747	0.011691	0.011766
0.011772	0.011713	0.011798	0.011750	0.011672	0.011711	0.011736
0.012033	0.011784	0.011749	0.011699	0.011717	0.011834	0.011800
0.011868	0.011802	0.011800	0.011899	0.011832	0.000000	0.012001
0.011967	0.011900	0.011765	0.011866	0.011665	0.011832	0.011698
0.011765	0.011765	0.011732	0.011698	0.011732	0.011698	0.011799
0.011698	0.011799	0.011799	0.011698	0.011802	0.011900	0.011905
0.011833	0.011799	0.011698	0.011634	0.011765	0.011698	0.011631

0.011740	0.011833	0.011631	0.011833	0.011665	0.011766	0.011732
0.011766	0.011732	0.011698	0.011732	0.011766	0.011631	0.011698
0.011732	0.011732	0.011799	0.011732	0.011765	0.011799	0.012034
0.012135	0.012068	0.012136	0.011903	0.012136	0.011765	0.011732
0.011770	0.012031	0.011792	0.011643	0.012146	0.011640	0.011769
0.012079	0.012047	0.011933	0.012135	0.011846	0.012001	0.011833
0.011765	0.012068	0.011913	0.011761	0.011899	0.011799	0.011872
0.011967	0.011870	0.012007	0.012008	0.012001	0.012068	0.012179
0.011906	0.012011	0.011941	0.011940	0.012171	0.011664	0.011869
0.011765	0.011765	0.011664	0.011664	0.011664	0.011732	0.011799
0.011664	0.011732	0.011631	0.011799	0.011664	0.011732	0.011731
0.011732	0.012168	0.012068	0.012101	0.011631	0.011732	0.011765
0.011765	0.011732	0.011799	0.011832	0.011698	0.011698	0.011698
0.012236	0.011866	0.011866	0.011664	0.011765	0.011799	0.011698
0.011698	0.011765	0.011732	0.011832	0.011698	0.011832	0.011899
0.011900	0.011732	0.011732	0.011664	0.011664	0.011833	0.011765
0.011664	0.011765	0.011631	0.011664	0.011799	0.011934	0.011698
0.011665	0.011799	0.011732	0.011631	0.011967	0.011631	0.011799
0.011779	0.011698	0.012001	0.011833	0.011866	0.011732	0.011765
0.011698	0.011967	0.011631	0.011900	0.011698	0.011698	0.011631
0.011799	0.012068	0.011765	0.011832	0.011664	0.012068	0.011698
0.011698	0.011698	0.011732	0.011765	0.011732	0.011832	0.011900
0.011900	0.011866	0.011765	0.011866	0.011765	0.011698	0.011664
0.011698	0.011698	0.011765	0.011698	0.011597	0.011832	0.011698
0.011732	0.011732	0.011866	0.011900	0.011799	0.011664	0.011732
0.011799	0.011731	0.011832	0.011698	0.011832	0.011933	0.012236
0.011732	0.012135	0.011681	0.018276	0.011649	0.011672	0.012107
0.012064	0.012116	0.011689	0.011814	0.011994	0.012131	0.011546
0.011914	0.011793	0.011606	0.011521	0.011724	0.011653	0.011619
0.011952	0.011842	0.011876	0.011920	0.011866	0.011900	0.011906
0.011713	0.011866	0.011866	0.011833	0.011799	0.011799	0.011766
0.011799	0.011732	0.011765	0.011799	0.011766	0.011698	0.011732
0.011732	0.011799	0.011698	0.011799	0.011833	0.011866	0.011765
0.011732	0.011766	0.012102	0.012102	0.011766	0.011800	0.011900
0.011665	0.011665	0.011698	0.011732	0.011665	0.011900	0.011900
0.011934	0.011933	0.011866	0.011967	0.012136	0.011970	0.011732
0.011799	0.011866	0.011833	0.011732	0.011799	0.011765	0.011732
0.011867	0.011867	0.011900	0.011799	0.011700	0.011967	0.011866
0.011805	0.011698	0.011799	0.011900	0.011799	0.011934	0.011967
0.012135	0.011846	0.012001	0.011833	0.011765	0.012068	0.011699
0.011802	0.011766	0.011732	0.011732	0.011799	0.011698	0.011799
0.011833	0.011866	0.011765	0.011732	0.011766	0.012102	0.012102
0.011766	0.011800	0.011933	0.011866	0.012507	0.011954	0.011834
0.011939	0.011893	0.011974	0.011857	0.012006	0.011923	0.011888
0.012469	0.011787	0.011917	0.011856	0.012128	0.011979	0.012013
0.012489	0.011927	0.012041	0.012101	0.011967	0.011967	0.012007
0.011664	0.011765	0.011967	0.011933	0.011939	0.011866	0.011903
0.011765	0.011631	0.011664	0.011765	0.011597	0.011732	0.011631
0.011765	0.011799	0.011698	0.011664	0.011765	0.011698	0.011732
0.011664	0.011664	0.011732	0.011765	0.011664	0.011799	0.011900
0.011799	0.011866	0.011799	0.011799	0.011732	0.012135	0.011698
0.011826	0.011732	0.011900	0.011909	0.011765	0.011832	0.011832
0.011833	0.011698	0.011732	0.011698	0.011866	0.011866	0.011799
0.011799	0.011933	0.012101	0.011832	0.012034	0.012034	0.011900
0.011731	0.011799	0.011833	0.012236	0.011799	0.011765	0.011799
0.011832	0.011765	0.011765	0.012068	0.011933	0.012034	0.011765
0.011866	0.011933	0.011799	0.011866	0.011765	0.011698	0.012034
0.011799	0.011765	0.011765	0.011933	0.011765	0.011866	0.012101
0.011799	0.012113	0.012169	0.011833	0.011737	0.011876	0.011731
0.011500	0.012151	0.011728	0.011909	0.011695	0.011773	0.011804

0.011876	0.011653	0.011784	0.011655	0.011599	0.011864	0.011790
0.011776	0.011690	0.011871	0.011800	0.011802	0.011866	0.011934
0.012001	0.011867	0.011973	0.011976	0.012112	0.012156	0.011799
0.011967	0.011900	0.011765	0.011866	0.011665	0.011832	0.011698
0.011765	0.011765	0.011732	0.011698	0.011732	0.011698	0.011799
0.011759	0.011795	0.011711	0.011814	0.011950	0.011846	0.011846
0.011912	0.011834	0.011651	0.011600	0.011772	0.011942	0.011943
0.011733	0.011738	0.012037	0.012007	0.011935	0.011969	0.011899
0.011832	0.011900	0.011899	0.011799	0.012371	0.011967	0.012034
0.012068	0.012164	0.011777	0.011857	0.011732	0.011933	0.011900
0.011935	0.011967	0.011967	0.011967	0.012122	0.012101	0.011754
0.011635	0.011649	0.011741	0.011706	0.011812	0.011695	0.011978
0.011781	0.011894	0.011680	0.011735	0.011767	0.011779	0.011665
0.011698	0.011698	0.011698	0.011698	0.011934	0.011900	0.011833
0.011934	0.012001	0.011732	0.011732	0.011766	0.011765	0.011770
0.011838	0.011766	0.011766	0.011802	0.011866	0.011934	0.012001
0.011867	0.011973	0.011976	0.012112	0.012156	0.011799	0.011967
0.011900	0.011900	0.011737	0.011931	0.011860	0.011878	0.011761
0.011852	0.012246	0.011949	0.011808	0.011631	0.011833	0.011698
0.011799	0.011799	0.011698	0.011802	0.011900	0.011905	0.011799
0.011698	0.011634	0.011765	0.011698	0.011631	0.011740	0.011833
0.011799	0.011732	0.011765	0.011799	0.011766	0.011698	0.011900
0.011765	0.011799	0.011799	0.011732	0.011799	0.012034	0.011698
0.011732	0.012135	0.011732	0.011698	0.011731	0.012101	0.011703
0.012185	0.011698	0.011712	0.011906	0.011956	0.011850	0.011604
0.011742	0.011899	0.011933	0.012011	0.011902	0.012042	0.012171
0.012070	0.012109	0.012068	0.012394	0.012101	0.012977	0.012139
0.012878	0.012646	0.012780	0.013053	0.012101	0.011799	0.012035
0.012542	0.012343	0.012068	0.011967	0.012036	0.012036	0.012008
0.011899	0.011908	0.011967	0.011935	0.012305	0.012371	0.012373
0.012272	0.012372	0.012477	0.012245	0.012543	0.012551	0.012610
0.012611	0.012644	0.012597	0.012611	0.012613	0.011832	0.012136
0.012205	0.012708	0.012214	0.012438	0.012913	0.011978	0.011867
0.011832	0.011832	0.012304	0.012336	0.012468	0.012169	0.011835
S167	0.011900	0.011900	0.012169	0.011934	0.011934	0.012001
0.011808	0.011740	0.011934	0.011497	0.011564	0.011498	0.011497
0.011463	0.011698	0.011430	0.011530	0.011598	0.011564	0.011530
0.011497	0.011530	0.011564	0.011497	0.011531	0.011530	0.011774
0.011698	0.011530	0.011430	0.011597	0.011598	0.011564	0.011598
0.011564	0.011665	0.011564	0.011564	0.011597	0.011564	0.011665
0.011430	0.011564	0.011598	0.011530	0.011564	0.011665	0.011598
0.011564	0.011732	0.011632	0.011833	0.011564	0.011833	0.011564
0.011530	0.011564	0.011698	0.011833	0.011867	0.011564	0.011665
0.011597	0.011665	0.011564	0.011799	0.011968	0.011564	0.011631
0.011530	0.011631	0.011598	0.011665	0.011597	0.011598	0.011598
0.011497	0.011530	0.011497	0.011531	0.011564	0.011599	0.011665
0.011598	0.011564	0.011631	0.011530	0.011597	0.011497	0.011564
0.011732	0.011665	0.011900	0.011732	0.011564	0.011799	0.011564
0.011728	0.011882	0.011777	0.011451	0.011471	0.011539	0.011612
0.012231	0.011620	0.011472	0.011799	0.011799	0.011530	0.011631
0.011631	0.011597	0.011665	0.011564	0.011665	0.011598	0.011597
0.011564	0.011597	0.011867	0.011732	0.011631	0.011598	0.011531
0.011497	0.011430	0.011564	0.011530	0.011665	0.011631	0.011564
0.011665	0.011732	0.011530	0.011530	0.011564	0.011564	0.011567
0.011635	0.011564	0.011564	0.011420	0.011494	0.011446	0.011531
0.011534	0.011473	0.011503	0.011509	0.011434	0.011471	0.011499
0.011708	0.011543	0.011577	0.011464	0.011475	0.011599	0.011498
0.011565	0.011566	0.011565	0.011698	0.011631	0.012001	0.000000
0.011766	0.011631	0.011564	0.011598	0.011463	0.011631	0.011497
0.011564	0.011564	0.011530	0.011497	0.011530	0.011497	0.011598

0.011564	0.011665	0.011598	0.011497	0.011600	0.011699	0.011702
0.011699	0.011598	0.011497	0.011431	0.011564	0.011497	0.011430
0.011536	0.011699	0.011497	0.011699	0.011463	0.011564	0.011530
0.011631	0.011530	0.011564	0.011531	0.011564	0.011430	0.011564
0.011598	0.011530	0.011598	0.011530	0.011564	0.011598	0.011833
0.011867	0.011799	0.011867	0.011701	0.011867	0.011564	0.011530
0.011499	0.011545	0.011701	0.011472	0.011875	0.011435	0.011532
0.011841	0.011740	0.011732	0.011934	0.011641	0.011799	0.011766
0.011698	0.011866	0.011736	0.011555	0.011564	0.011598	0.011670
0.011766	0.011634	0.011805	0.011873	0.011799	0.011867	0.011976
0.011704	0.011841	0.011772	0.011737	0.011969	0.011463	0.011633
0.011631	0.011564	0.011463	0.011463	0.011530	0.011530	0.011597
0.011530	0.011598	0.011497	0.011530	0.011530	0.011530	0.011597
0.011530	0.011900	0.011799	0.011833	0.011430	0.011530	0.011564
0.011564	0.011530	0.011598	0.011564	0.011497	0.011497	0.011497
0.011967	0.011598	0.011665	0.011463	0.011564	0.011732	0.011497
0.011497	0.011564	0.011530	0.011699	0.011497	0.011631	0.011765
0.011698	0.011530	0.011530	0.011463	0.011463	0.011631	0.011564
0.011463	0.011564	0.011430	0.011463	0.011598	0.011665	0.011497
0.011464	0.011598	0.011531	0.011430	0.011698	0.011430	0.011598
0.011643	0.011497	0.011732	0.011631	0.011598	0.011530	0.011631
0.011497	0.011833	0.011430	0.011631	0.011430	0.011430	0.011430
0.011665	0.011800	0.011564	0.011631	0.011463	0.011799	0.011430
0.011497	0.011497	0.011530	0.011631	0.011530	0.011631	0.011698
0.011698	0.011665	0.011564	0.011665	0.011631	0.011497	0.011463
0.011497	0.011497	0.011564	0.011430	0.011463	0.011631	0.011497
0.011463	0.011530	0.011665	0.011564	0.011598	0.011530	0.011531
0.011598	0.011530	0.011631	0.011497	0.011631	0.011732	0.011967
0.011530	0.011867	0.011509	0.017975	0.011584	0.011488	0.011793
0.011753	0.011799	0.011532	0.011582	0.011940	0.011820	0.011668
0.011677	0.011732	0.011450	0.011521	0.011451	0.011554	0.011534
0.011623	0.011619	0.011616	0.011457	0.011665	0.011698	0.011703
0.011541	0.011665	0.011665	0.011564	0.011598	0.011598	0.011564
0.011598	0.011530	0.011564	0.011598	0.011564	0.011497	0.011530
0.011530	0.011598	0.011497	0.011598	0.011631	0.011665	0.011564
0.011530	0.011497	0.011833	0.011833	0.011631	0.011666	0.011699
0.011463	0.011463	0.011497	0.011598	0.011463	0.011698	0.011698
0.011732	0.011732	0.011665	0.011766	0.011867	0.011768	0.011598
0.011598	0.011665	0.011698	0.011530	0.011598	0.011564	0.011598
0.011665	0.011665	0.011698	0.011665	0.011565	0.011765	0.011732
0.011535	0.011497	0.011598	0.011699	0.011598	0.011463	0.011765
0.011934	0.011641	0.011799	0.011766	0.011698	0.011866	0.011497
0.011667	0.011565	0.011530	0.011530	0.011598	0.011497	0.011598
0.011631	0.011665	0.011564	0.011530	0.011497	0.011833	0.011833
0.011631	0.011666	0.011732	0.011665	0.012205	0.011668	0.011658
0.011658	0.011685	0.011763	0.011684	0.011761	0.011716	0.011647
0.012194	0.011545	0.011677	0.011579	0.011887	0.011775	0.011843
0.012183	0.011437	0.011703	0.011665	0.011698	0.011631	0.011669
0.011463	0.011564	0.011698	0.011665	0.011669	0.011665	0.011633
0.011564	0.011497	0.011530	0.011564	0.011463	0.011530	0.011430
0.011497	0.011530	0.011497	0.011463	0.011564	0.011497	0.011530
0.011530	0.011463	0.011530	0.011564	0.011463	0.011665	0.011698
0.011598	0.011598	0.011598	0.011598	0.011530	0.011867	0.011564
0.011548	0.011530	0.011665	0.011671	0.011631	0.011631	0.011631
0.011631	0.011564	0.011530	0.011564	0.011665	0.011665	0.011598
0.011597	0.011732	0.011833	0.011631	0.011833	0.011833	0.011698
0.011530	0.011598	0.011631	0.011968	0.011598	0.011631	0.011598
0.011631	0.011497	0.011497	0.011800	0.011800	0.011766	0.011564
0.011665	0.011732	0.011598	0.011665	0.011631	0.011497	0.011766
0.011597	0.011564	0.011497	0.011732	0.011564	0.011665	0.011900

0.011597	0.011876	0.011900	0.011711	0.011399	0.011570	0.011473
0.011335	0.011917	0.011482	0.011641	0.011548	0.011671	0.011645
0.011672	0.011521	0.011729	0.011523	0.011545	0.011730	0.011669
0.011663	0.011572	0.011554	0.011553	0.011600	0.011665	0.011732
0.011665	0.011598	0.011703	0.011704	0.011840	0.011873	0.011598
0.011766	0.011631	0.011564	0.011598	0.011463	0.011631	0.011497
0.011564	0.011564	0.011530	0.011497	0.011530	0.011497	0.011598
0.011549	0.011550	0.011540	0.011574	0.011711	0.011675	0.011675
0.011674	0.011598	0.011430	0.011420	0.011535	0.011704	0.011705
0.011497	0.011568	0.011801	0.011770	0.011699	0.011733	0.011698
0.011631	0.011698	0.011698	0.011597	0.012102	0.011766	0.011833
0.011867	0.011880	0.011572	0.011615	0.011530	0.011732	0.011698
0.011733	0.011765	0.011766	0.011766	0.011807	0.011900	0.011511
0.011432	0.011441	0.011503	0.011468	0.011505	0.011396	0.011672
0.011541	0.011587	0.011441	0.011510	0.011531	0.011608	0.011531
0.011497	0.011430	0.011564	0.011530	0.011665	0.011631	0.011564
0.011665	0.011732	0.011530	0.011530	0.011564	0.011564	0.011567
0.011635	0.011564	0.011564	0.011600	0.011665	0.011732	0.011665
0.011598	0.011703	0.011704	0.011840	0.011873	0.011598	0.011765
0.011698	0.011698	0.011534	0.011652	0.011651	0.011673	0.011516
0.011715	0.011881	0.011568	0.011604	0.011497	0.011699	0.011564
0.011665	0.011598	0.011497	0.011600	0.011699	0.011702	0.011598
0.011497	0.011431	0.011564	0.011497	0.011430	0.011536	0.011699
0.011598	0.011530	0.011564	0.011598	0.011564	0.011497	0.011631
0.011564	0.011598	0.011597	0.011530	0.011598	0.011833	0.011430
0.011530	0.011867	0.011530	0.011564	0.011530	0.011833	0.011532
0.011904	0.011485	0.011411	0.011624	0.011753	0.011635	0.011478
0.011375	0.011631	0.011732	0.011807	0.011700	0.011839	0.011969
0.011834	0.011872	0.011866	0.012086	0.011833	0.012708	0.011835
0.012541	0.012342	0.012510	0.012782	0.011833	0.011531	0.011833
0.012272	0.012208	0.011799	0.011699	0.011733	0.011733	0.011737
0.011631	0.011637	0.011698	0.011632	0.012036	0.012035	0.012070
0.012003	0.012103	0.012173	0.011940	0.012273	0.012280	0.012340
0.012307	0.012341	0.012324	0.012307	0.012343	0.011631	0.011867
0.011901	0.012439	0.011942	0.012102	0.012643	0.011672	0.011598
0.011564	0.011564	0.012035	0.012006	0.012151	0.011900	0.011566
S168 0.011933	0.011933	0.012001	0.011765	0.011765	0.011832	
0.011671	0.011603	0.011832	0.011530	0.011597	0.011565	0.011530
0.011496	0.011731	0.011463	0.011564	0.011631	0.011597	0.011564
0.011530	0.011564	0.011396	0.011530	0.011564	0.011564	0.011637
0.011429	0.011564	0.011463	0.011429	0.011631	0.011597	0.011631
0.011597	0.011698	0.011597	0.011597	0.011631	0.011597	0.011765
0.011463	0.011597	0.011631	0.011564	0.011597	0.011698	0.011631
0.011597	0.011765	0.011665	0.011664	0.011597	0.011866	0.011597
0.011564	0.011597	0.011731	0.011866	0.011698	0.011597	0.011698
0.011631	0.011698	0.011597	0.011832	0.011799	0.011597	0.011664
0.011564	0.011664	0.011631	0.011698	0.011631	0.011631	0.011631
0.011530	0.011564	0.011530	0.011564	0.011597	0.011632	0.011698
0.011631	0.011597	0.011664	0.011563	0.011631	0.011530	0.011597
0.011765	0.011698	0.011732	0.011765	0.011597	0.011765	0.011597
0.011765	0.011695	0.011777	0.011529	0.011545	0.011649	0.011415
0.012085	0.011416	0.011596	0.011832	0.011832	0.011564	0.011664
0.011664	0.011631	0.011698	0.011597	0.011698	0.011631	0.011631
0.011597	0.011631	0.011899	0.011765	0.011664	0.011631	0.011564
0.011530	0.011463	0.011597	0.011564	0.011497	0.011463	0.011396
0.011497	0.011564	0.011564	0.011564	0.011597	0.011597	0.011601
0.011669	0.011597	0.011597	0.011492	0.011566	0.011516	0.011598
0.011602	0.011541	0.011614	0.011578	0.011502	0.011540	0.011567
0.011564	0.011612	0.011646	0.011531	0.011544	0.011666	0.011565
0.011632	0.011633	0.011632	0.011731	0.011664	0.011967	0.011766

0.000000	0.011665	0.011597	0.011631	0.011497	0.011329	0.011530
0.011597	0.011597	0.011564	0.011530	0.011564	0.011530	0.011631
0.011597	0.011698	0.011631	0.011530	0.011633	0.011732	0.011736
0.011732	0.011631	0.011530	0.011465	0.011597	0.011530	0.011463
0.011468	0.011732	0.011530	0.011732	0.011497	0.011597	0.011564
0.011665	0.011564	0.011597	0.011564	0.011597	0.011463	0.011597
0.011631	0.011564	0.011631	0.011564	0.011597	0.011631	0.011866
0.011698	0.011631	0.011698	0.011734	0.011699	0.011597	0.011564
0.011533	0.011828	0.011609	0.011506	0.011705	0.011469	0.011611
0.011705	0.011604	0.011765	0.011967	0.011675	0.011832	0.011799
0.011731	0.011900	0.011771	0.011596	0.011731	0.011631	0.011703
0.011799	0.011701	0.011838	0.011906	0.011832	0.011900	0.012009
0.011737	0.011909	0.011839	0.011770	0.012036	0.011496	0.011700
0.011664	0.011597	0.011496	0.011496	0.011564	0.011564	0.011631
0.011564	0.011631	0.011530	0.011564	0.011496	0.011564	0.011631
0.011564	0.011732	0.011631	0.011664	0.011463	0.011564	0.011597
0.011597	0.011564	0.011631	0.011597	0.011530	0.011530	0.011530
0.011799	0.011631	0.011698	0.011496	0.011597	0.011765	0.011530
0.011530	0.011597	0.011564	0.011732	0.011530	0.011463	0.011597
0.011530	0.011564	0.011564	0.011496	0.011496	0.011664	0.011597
0.011496	0.011597	0.011463	0.011496	0.011631	0.011497	0.011530
0.011497	0.011631	0.011564	0.011463	0.011530	0.011463	0.011631
0.011711	0.011530	0.011564	0.011665	0.011631	0.011564	0.011664
0.011530	0.011866	0.011463	0.011664	0.011463	0.011463	0.011463
0.011698	0.011631	0.011597	0.011664	0.011496	0.011631	0.011463
0.011530	0.011530	0.011564	0.011664	0.011564	0.011664	0.011731
0.011731	0.011698	0.011597	0.011698	0.011664	0.011530	0.011496
0.011530	0.011530	0.011597	0.011463	0.011496	0.011664	0.011530
0.011496	0.011564	0.011698	0.011664	0.011631	0.011564	0.011564
0.011631	0.011564	0.011664	0.011530	0.011664	0.011765	0.011799
0.011564	0.011698	0.011578	0.018125	0.011649	0.011672	0.011653
0.011614	0.011658	0.011741	0.011582	0.011720	0.011682	0.011789
0.011724	0.011488	0.011489	0.011646	0.011529	0.011702	0.011619
0.011530	0.011321	0.011468	0.011391	0.011698	0.011732	0.011737
0.011610	0.011698	0.011698	0.011597	0.011631	0.011631	0.011598
0.011631	0.011564	0.011597	0.011631	0.011597	0.011530	0.011564
0.011564	0.011631	0.011530	0.011631	0.011665	0.011698	0.011597
0.011564	0.011530	0.011665	0.011665	0.011665	0.011699	0.011732
0.011497	0.011497	0.011530	0.011631	0.011497	0.011732	0.011732
0.011765	0.011765	0.011698	0.011799	0.011698	0.011801	0.011631
0.011631	0.011698	0.011732	0.011564	0.011631	0.011597	0.011631
0.011699	0.011698	0.011732	0.011698	0.011599	0.011799	0.011765
0.011636	0.011530	0.011631	0.011732	0.011631	0.011698	0.011799
0.011967	0.011675	0.011832	0.011799	0.011731	0.011900	0.011531
0.011700	0.011598	0.011564	0.011564	0.011631	0.011530	0.011631
0.011665	0.011698	0.011597	0.011564	0.011530	0.011665	0.011665
0.011665	0.011699	0.011765	0.011698	0.012271	0.011775	0.011693
0.011763	0.011719	0.011833	0.011718	0.011830	0.011750	0.011715
0.012228	0.011614	0.011745	0.011683	0.011955	0.011808	0.011911
0.012250	0.011739	0.011872	0.011933	0.011799	0.011530	0.011837
0.011496	0.011664	0.011799	0.011765	0.011770	0.011362	0.011464
0.011597	0.011530	0.011564	0.011597	0.011496	0.011564	0.011463
0.011530	0.011564	0.011530	0.011496	0.011597	0.011530	0.011564
0.011564	0.011496	0.011564	0.011597	0.011496	0.011698	0.011731
0.011631	0.011631	0.011631	0.011631	0.011564	0.011698	0.011597
0.011373	0.011564	0.011430	0.011433	0.011664	0.011664	0.011664
0.011664	0.011597	0.011564	0.011597	0.011698	0.011698	0.011631
0.011631	0.011765	0.011664	0.011664	0.011866	0.011866	0.011731
0.011563	0.011631	0.011664	0.011799	0.011631	0.011664	0.011631
0.011664	0.011530	0.011530	0.011832	0.011832	0.011799	0.011597

0.011698	0.011765	0.011631	0.011698	0.011664	0.011530	0.011597
0.011631	0.011597	0.011530	0.011765	0.011597	0.011698	0.011732
0.011631	0.011739	0.011732	0.011345	0.011455	0.011638	0.011370
0.011555	0.011722	0.011552	0.011730	0.011597	0.011620	0.011591
0.011315	0.011587	0.011617	0.011522	0.011545	0.011797	0.011750
0.011738	0.011650	0.011624	0.011624	0.011633	0.011362	0.011430
0.011564	0.011632	0.011736	0.011535	0.011874	0.011908	0.011631
0.000000	0.011665	0.011597	0.011631	0.011497	0.011329	0.011530
0.011597	0.011597	0.011564	0.011530	0.011564	0.011530	0.011631
0.011619	0.011620	0.011608	0.011642	0.011779	0.011743	0.011743
0.011742	0.011665	0.011503	0.011492	0.011602	0.011772	0.011773
0.011565	0.011636	0.011868	0.011838	0.011766	0.011801	0.011731
0.011664	0.011731	0.011731	0.011631	0.011934	0.011799	0.011866
0.011900	0.011701	0.011606	0.011684	0.011564	0.011765	0.011732
0.011767	0.011799	0.011799	0.011799	0.011925	0.011933	0.011580
0.011466	0.011476	0.011571	0.011536	0.011573	0.011508	0.011536
0.011609	0.011499	0.011632	0.011660	0.011598	0.011676	0.011564
0.011530	0.011463	0.011597	0.011564	0.011497	0.011463	0.011396
0.011497	0.011564	0.011564	0.011564	0.011597	0.011597	0.011601
0.011669	0.011597	0.011597	0.011633	0.011362	0.011430	0.011564
0.011632	0.011736	0.011535	0.011874	0.011908	0.011631	0.011799
0.011732	0.011732	0.011567	0.011756	0.011686	0.011673	0.011586
0.011749	0.012017	0.011738	0.011638	0.011530	0.011732	0.011597
0.011698	0.011631	0.011530	0.011633	0.011732	0.011736	0.011631
0.011530	0.011465	0.011597	0.011530	0.011463	0.011468	0.011732
0.011631	0.011564	0.011597	0.011631	0.011597	0.011530	0.011463
0.011597	0.011631	0.011631	0.011564	0.011631	0.011866	0.011463
0.011564	0.011698	0.011564	0.011597	0.011563	0.011664	0.011617
0.011904	0.011485	0.011461	0.011342	0.011427	0.011347	0.011436
0.011428	0.011463	0.011564	0.011671	0.011532	0.011669	0.011800
0.011700	0.011737	0.011698	0.011949	0.011664	0.012539	0.011700
0.012439	0.012207	0.012341	0.012613	0.011664	0.011564	0.011665
0.012306	0.012038	0.011631	0.011530	0.011598	0.011598	0.011568
0.011463	0.011467	0.011530	0.011497	0.011867	0.011933	0.011935
0.011834	0.011935	0.012038	0.011804	0.012306	0.012313	0.012373
0.012374	0.012408	0.012357	0.012374	0.012376	0.011664	0.011900
0.011968	0.012472	0.011976	0.012001	0.012474	0.011536	0.011430
0.011396	0.011396	0.011867	0.011883	0.012010	0.011732	0.011397
S169	0.011799	0.011799	0.012068	0.011833	0.011833	0.011900
0.011740	0.011672	0.011833	0.011396	0.011463	0.011430	0.011396
0.011362	0.011597	0.011329	0.011430	0.011497	0.011463	0.011430
0.011396	0.011430	0.011463	0.011396	0.011430	0.011430	0.011706
0.011597	0.011430	0.011329	0.011497	0.011497	0.011463	0.011497
0.011463	0.011564	0.011463	0.011463	0.011497	0.011463	0.011631
0.011329	0.011463	0.011497	0.011429	0.011463	0.011564	0.011497
0.011463	0.011631	0.011531	0.011732	0.011463	0.011732	0.011463
0.011429	0.011463	0.011598	0.011732	0.011766	0.011463	0.011295
0.011497	0.011564	0.011463	0.011698	0.011867	0.011463	0.011530
0.011430	0.011530	0.011497	0.011564	0.011497	0.011497	0.011497
0.011396	0.011429	0.011396	0.011430	0.011463	0.011498	0.011564
0.011497	0.011463	0.011530	0.011429	0.011497	0.011396	0.011463
0.011631	0.011564	0.011799	0.011631	0.011463	0.011698	0.011463
0.011654	0.011807	0.011702	0.011412	0.011396	0.011465	0.011533
0.012158	0.011518	0.011431	0.011698	0.011698	0.011429	0.011530
0.011530	0.011497	0.011564	0.011463	0.011564	0.011497	0.011497
0.011463	0.011497	0.011766	0.011631	0.011530	0.011497	0.011430
0.011396	0.011329	0.011463	0.011430	0.011564	0.011530	0.011463
0.011564	0.011631	0.011429	0.011429	0.011463	0.011463	0.011465
0.011533	0.011463	0.011463	0.011312	0.011422	0.011341	0.011464
0.011467	0.011404	0.011466	0.011440	0.011366	0.011403	0.011432

0.011564	0.011439	0.011508	0.011396	0.011406	0.011531	0.011430
0.011498	0.011498	0.011497	0.011597	0.011530	0.011900	0.011631
0.011665	0.000000	0.011463	0.011228	0.011362	0.011530	0.011396
0.011463	0.011463	0.011430	0.011396	0.011430	0.011396	0.011497
0.011463	0.011564	0.011497	0.011396	0.011498	0.011598	0.011601
0.011598	0.011497	0.011396	0.011330	0.011463	0.011396	0.011329
0.011434	0.011598	0.011396	0.011598	0.011362	0.011463	0.011430
0.011530	0.011430	0.011463	0.011362	0.011396	0.011329	0.011463
0.011497	0.011430	0.011497	0.011429	0.011463	0.011497	0.011732
0.011698	0.011698	0.011766	0.011600	0.011766	0.011463	0.011430
0.011398	0.011586	0.011518	0.011369	0.011739	0.011333	0.011414
0.011773	0.011672	0.011631	0.011833	0.011538	0.011698	0.011665
0.011598	0.011765	0.011629	0.011390	0.011480	0.011497	0.011568
0.011665	0.011566	0.011703	0.011771	0.011698	0.011698	0.011874
0.011602	0.011773	0.011704	0.011635	0.011902	0.011362	0.011566
0.011530	0.011463	0.011362	0.011362	0.011430	0.011430	0.011497
0.011429	0.011497	0.011396	0.011430	0.011430	0.011430	0.011497
0.011430	0.011799	0.011698	0.011732	0.011329	0.011430	0.011463
0.011463	0.011430	0.011497	0.011195	0.011396	0.011396	0.011396
0.011866	0.011497	0.011564	0.011362	0.011463	0.011631	0.011396
0.011396	0.011463	0.011429	0.011598	0.011396	0.011530	0.011665
0.011597	0.011430	0.011430	0.011362	0.011362	0.011530	0.011463
0.011362	0.011463	0.011329	0.011362	0.011497	0.011564	0.011396
0.011363	0.011497	0.011430	0.011329	0.011597	0.011329	0.011497
0.011574	0.011396	0.011631	0.011530	0.011497	0.011430	0.011530
0.011396	0.011732	0.011329	0.011530	0.011329	0.011329	0.011329
0.011564	0.011699	0.011463	0.011530	0.011362	0.011698	0.011329
0.011396	0.011396	0.011430	0.011530	0.011362	0.011530	0.011597
0.011598	0.011564	0.011463	0.011564	0.011530	0.011396	0.011362
0.011396	0.011396	0.011463	0.011329	0.011362	0.011530	0.011396
0.011362	0.011430	0.011564	0.011530	0.011497	0.011429	0.011430
0.011497	0.011429	0.011530	0.011396	0.011530	0.011631	0.011866
0.011430	0.011766	0.011440	0.017974	0.011388	0.011549	0.011723
0.011683	0.011729	0.011584	0.011466	0.011884	0.011751	0.011607
0.011677	0.011610	0.011372	0.011479	0.011294	0.011455	0.011447
0.011623	0.011544	0.011542	0.011325	0.011564	0.011597	0.011601
0.011473	0.011564	0.011564	0.011463	0.011497	0.011497	0.011463
0.011497	0.011429	0.011463	0.011497	0.011463	0.011396	0.011429
0.011429	0.011497	0.011396	0.011497	0.011530	0.011564	0.011463
0.011430	0.011396	0.011732	0.011732	0.011531	0.011565	0.011598
0.011362	0.011362	0.011396	0.011497	0.011362	0.011598	0.011598
0.011631	0.011631	0.011564	0.011665	0.011766	0.011666	0.011497
0.011497	0.011564	0.011598	0.011430	0.011497	0.011463	0.011497
0.011564	0.011564	0.011597	0.011564	0.011464	0.011665	0.011631
0.011501	0.011396	0.011497	0.011598	0.011497	0.011564	0.011665
0.011833	0.011538	0.011698	0.011665	0.011598	0.011765	0.011396
0.011566	0.011464	0.011429	0.011429	0.011497	0.011396	0.011497
0.011530	0.011564	0.011463	0.011430	0.011396	0.011732	0.011732
0.011531	0.011565	0.011631	0.011564	0.012137	0.011632	0.011553
0.011623	0.011581	0.011693	0.011580	0.011691	0.011612	0.011578
0.012091	0.011476	0.011608	0.011545	0.011749	0.011673	0.011775
0.012115	0.011588	0.011534	0.011497	0.011530	0.011463	0.011500
0.011362	0.011396	0.011530	0.011497	0.011500	0.011564	0.011532
0.011463	0.011396	0.011430	0.011463	0.011362	0.011430	0.011329
0.011396	0.011430	0.011396	0.011362	0.011463	0.011396	0.011430
0.011429	0.011362	0.011430	0.011463	0.011362	0.011564	0.011597
0.011497	0.011497	0.011497	0.011497	0.011430	0.011766	0.011463
0.011443	0.011430	0.011598	0.011603	0.011531	0.011530	0.011530
0.011530	0.011463	0.011429	0.011463	0.011564	0.011564	0.011497
0.011497	0.011631	0.011732	0.011530	0.011732	0.011732	0.011597

0.011429	0.011497	0.011530	0.011867	0.011497	0.011531	0.011497
0.011530	0.011396	0.011396	0.011699	0.011699	0.011665	0.011463
0.011564	0.011631	0.011497	0.011564	0.011530	0.011396	0.011665
0.011497	0.011463	0.011396	0.011631	0.011463	0.011564	0.011799
0.011497	0.011808	0.011799	0.011629	0.011286	0.011502	0.011370
0.011390	0.011839	0.011411	0.011596	0.011402	0.011569	0.011538
0.011519	0.011389	0.011506	0.011258	0.011491	0.011462	0.011587
0.011588	0.011493	0.011412	0.011412	0.011498	0.011564	0.011631
0.011631	0.011228	0.011331	0.011603	0.011670	0.011484	0.011497
0.011665	0.000000	0.011463	0.011228	0.011362	0.011530	0.011396
0.011463	0.011463	0.011430	0.011396	0.011430	0.011396	0.011497
0.011479	0.011410	0.011471	0.011505	0.011642	0.011607	0.011607
0.011605	0.011531	0.011319	0.011312	0.011467	0.011636	0.011637
0.011430	0.011501	0.011734	0.011703	0.011632	0.011666	0.011597
0.011530	0.011597	0.011597	0.011497	0.012001	0.011665	0.011732
0.011766	0.011701	0.011470	0.011511	0.011430	0.011631	0.011597
0.011632	0.011665	0.011665	0.011665	0.011768	0.011799	0.011442
0.011331	0.011337	0.011435	0.011400	0.011436	0.011322	0.011604
0.011472	0.011411	0.011203	0.011472	0.011464	0.011539	0.011430
0.011396	0.011329	0.011463	0.011430	0.011564	0.011530	0.011463
0.011564	0.011631	0.011429	0.011429	0.011463	0.011463	0.011465
0.011533	0.011463	0.011463	0.011498	0.011564	0.011631	0.011631
0.011228	0.011331	0.011603	0.011670	0.011484	0.011497	0.011665
0.011597	0.011597	0.011432	0.011617	0.011547	0.011571	0.011446
0.011611	0.011789	0.011483	0.011502	0.011396	0.011598	0.011463
0.011564	0.011497	0.011396	0.011498	0.011598	0.011601	0.011497
0.011396	0.011330	0.011463	0.011396	0.011329	0.011434	0.011598
0.011497	0.011429	0.011463	0.011497	0.011463	0.011396	0.011530
0.011463	0.011497	0.011497	0.011429	0.011497	0.011732	0.011329
0.011430	0.011766	0.011429	0.011463	0.011429	0.011732	0.011446
0.011791	0.011443	0.011411	0.011584	0.011671	0.011563	0.011395
0.011270	0.011530	0.011631	0.011739	0.011599	0.011737	0.011867
0.011767	0.011804	0.011765	0.012017	0.011732	0.012607	0.011768
0.012507	0.012275	0.012409	0.012680	0.011732	0.011430	0.011732
0.012171	0.012106	0.011698	0.011598	0.011666	0.011666	0.011635
0.011530	0.011535	0.011597	0.011565	0.011935	0.012001	0.012002
0.011902	0.012002	0.012106	0.011872	0.012172	0.012178	0.012239
0.012239	0.012273	0.012221	0.012240	0.012241	0.011530	0.011766
0.011834	0.012338	0.011840	0.012069	0.012541	0.011604	0.011497
0.011463	0.011463	0.011934	0.011924	0.012081	0.011799	0.011465
S170	0.011664	0.011664	0.012001	0.011765	0.011765	0.011832
0.011671	0.011603	0.011799	0.011262	0.011329	0.011296	0.011262
0.011295	0.011463	0.011262	0.011295	0.011362	0.011329	0.011295
0.011262	0.011295	0.011396	0.011262	0.011295	0.011295	0.011637
0.011530	0.011295	0.011195	0.011429	0.011362	0.011329	0.011362
0.011329	0.011429	0.011329	0.011329	0.011362	0.011329	0.011497
0.011262	0.011329	0.011362	0.011295	0.011396	0.011429	0.011362
0.011329	0.011497	0.011396	0.011664	0.011329	0.011597	0.011329
0.011295	0.011396	0.011463	0.011597	0.011698	0.011396	0.011497
0.011362	0.011429	0.011329	0.011564	0.011799	0.011329	0.011396
0.011295	0.011396	0.011362	0.011429	0.011362	0.011362	0.011362
0.011262	0.011295	0.011262	0.011362	0.011329	0.011363	0.011429
0.011362	0.011329	0.011396	0.011295	0.011429	0.011262	0.011329
0.011496	0.011429	0.011664	0.011497	0.011329	0.011564	0.011329
0.011468	0.011695	0.011513	0.011216	0.011209	0.011355	0.011414
0.012048	0.011467	0.011225	0.011564	0.011564	0.011362	0.011463
0.011396	0.011362	0.011429	0.011329	0.011429	0.011362	0.011362
0.011329	0.011429	0.011631	0.011496	0.011396	0.011362	0.011295
0.011262	0.011262	0.011329	0.011295	0.011497	0.011463	0.011396
0.011497	0.011564	0.011295	0.011295	0.011329	0.011329	0.011330

0.011398	0.011329	0.011329	0.011204	0.011277	0.011236	0.011329
0.011331	0.011266	0.011318	0.011302	0.011230	0.011266	0.011297
0.011564	0.011336	0.011370	0.011262	0.011268	0.011397	0.011363
0.011430	0.011363	0.011363	0.011463	0.011396	0.011765	0.011564
0.011597	0.011463	0.000000	0.011430	0.011228	0.011463	0.011262
0.011329	0.011329	0.011295	0.011262	0.011295	0.011262	0.011362
0.011329	0.011429	0.011362	0.011262	0.011363	0.011463	0.011466
0.011463	0.011362	0.011262	0.011195	0.011329	0.011262	0.011195
0.011298	0.011463	0.011262	0.011463	0.011228	0.011329	0.011295
0.011396	0.011295	0.011329	0.011295	0.011329	0.011195	0.011329
0.011362	0.011295	0.011362	0.011295	0.011329	0.011362	0.011597
0.011698	0.011631	0.011698	0.011465	0.011698	0.011329	0.011295
0.011330	0.011586	0.011472	0.011232	0.011705	0.011197	0.011336
0.011705	0.011604	0.011496	0.011698	0.011402	0.011564	0.011530
0.011463	0.011631	0.011487	0.011349	0.011396	0.011362	0.011432
0.011530	0.011431	0.011567	0.011635	0.011564	0.011631	0.011738
0.011466	0.011637	0.011568	0.011500	0.011767	0.011228	0.011431
0.011396	0.011329	0.011228	0.011228	0.011295	0.011295	0.011362
0.011295	0.011362	0.011262	0.011362	0.011295	0.011295	0.011362
0.011295	0.011732	0.011631	0.011664	0.011195	0.011295	0.011329
0.011329	0.011295	0.011362	0.011396	0.011262	0.011262	0.011262
0.011799	0.011429	0.011429	0.011228	0.011329	0.011497	0.011262
0.011262	0.011329	0.011295	0.011463	0.011262	0.011396	0.011530
0.011396	0.011295	0.011295	0.011228	0.011228	0.011396	0.011329
0.011228	0.011329	0.011195	0.011228	0.011362	0.011497	0.011262
0.011228	0.011362	0.011295	0.011195	0.011530	0.011195	0.011362
0.011437	0.011262	0.011564	0.011396	0.011429	0.011295	0.011396
0.011262	0.011597	0.011195	0.011463	0.011262	0.011262	0.011195
0.011429	0.011631	0.011329	0.011396	0.011228	0.011631	0.011262
0.011262	0.011262	0.011295	0.011396	0.011295	0.011396	0.011463
0.011463	0.011429	0.011329	0.011430	0.011396	0.011262	0.011228
0.011262	0.011262	0.011329	0.011262	0.011228	0.011396	0.011262
0.011295	0.011295	0.011429	0.011463	0.011362	0.011295	0.011295
0.011362	0.011295	0.011396	0.011262	0.011396	0.011497	0.011799
0.011295	0.011698	0.011302	0.017747	0.011126	0.011427	0.011653
0.011614	0.011658	0.011270	0.011234	0.011664	0.011682	0.011303
0.011440	0.011487	0.011177	0.011270	0.011216	0.011258	0.011275
0.011482	0.011433	0.011431	0.011259	0.011429	0.011463	0.011466
0.011335	0.011429	0.011429	0.011396	0.011362	0.011363	0.011329
0.011362	0.011295	0.011329	0.011362	0.011329	0.011262	0.011295
0.011295	0.011362	0.011262	0.011362	0.011396	0.011429	0.011329
0.011362	0.011329	0.011665	0.011665	0.011396	0.011430	0.011463
0.011228	0.011228	0.011262	0.011362	0.011228	0.011463	0.011463
0.011497	0.011496	0.011429	0.011530	0.011698	0.011532	0.011362
0.011362	0.011429	0.011463	0.011228	0.011362	0.011329	0.011362
0.011430	0.011430	0.011463	0.011430	0.011330	0.011530	0.011497
0.011365	0.011262	0.011362	0.011463	0.011362	0.011497	0.011530
0.011698	0.011402	0.011564	0.011530	0.011463	0.011631	0.011262
0.011431	0.011329	0.011295	0.011295	0.011362	0.011262	0.011362
0.011396	0.011429	0.011329	0.011362	0.011329	0.011665	0.011665
0.011396	0.011430	0.011496	0.011429	0.012069	0.011489	0.011412
0.011482	0.011442	0.011552	0.011441	0.011550	0.011405	0.011439
0.012022	0.011337	0.011471	0.011406	0.011680	0.011536	0.011639
0.012046	0.011475	0.011432	0.011497	0.011396	0.011396	0.011398
0.011228	0.011262	0.011396	0.011362	0.011364	0.011496	0.011464
0.011329	0.011262	0.011295	0.011329	0.011228	0.011295	0.011195
0.011329	0.011362	0.011262	0.011228	0.011329	0.011262	0.011295
0.011295	0.011228	0.011295	0.011329	0.011228	0.011429	0.011463
0.011362	0.011429	0.011362	0.011362	0.011295	0.011698	0.011329
0.011373	0.011295	0.011531	0.011535	0.011396	0.011396	0.011396

0.011396	0.011329	0.011295	0.011329	0.011429	0.011429	0.011362
0.011362	0.011497	0.011664	0.011396	0.011597	0.011597	0.011463
0.011295	0.011362	0.011396	0.011799	0.011362	0.011396	0.011362
0.011396	0.011329	0.011329	0.011631	0.011564	0.011597	0.011329
0.011429	0.011497	0.011362	0.011429	0.011396	0.011262	0.011597
0.011362	0.011329	0.011329	0.011497	0.011329	0.011429	0.011665
0.011362	0.011739	0.011732	0.011507	0.011173	0.011434	0.011215
0.011225	0.011722	0.011271	0.011417	0.011255	0.011264	0.011273
0.011417	0.000000	0.011227	0.000000	0.011168	0.011261	0.011385
0.011399	0.011296	0.011412	0.011342	0.011364	0.011497	0.011564
0.011564	0.011430	0.011533	0.011535	0.011670	0.011661	0.011362
0.011597	0.011463	0.000000	0.011430	0.011228	0.011463	0.011262
0.011329	0.011329	0.011295	0.011262	0.011295	0.011262	0.011362
0.011340	0.011340	0.011334	0.011368	0.011505	0.011470	0.011470
0.011469	0.011396	0.011208	0.011204	0.011331	0.011501	0.011501
0.011296	0.011365	0.011599	0.011567	0.011497	0.011531	0.011463
0.011396	0.011463	0.011463	0.011362	0.011933	0.011530	0.011597
0.011631	0.011701	0.011333	0.011407	0.011295	0.011497	0.011463
0.011497	0.011530	0.011530	0.011530	0.011689	0.011664	0.011303
0.011195	0.011199	0.011299	0.011264	0.011368	0.011210	0.011536
0.011335	0.011499	0.011251	0.011360	0.011329	0.011402	0.011295
0.011262	0.011262	0.011329	0.011295	0.011497	0.011463	0.011396
0.011497	0.011564	0.011295	0.011295	0.011329	0.011329	0.011330
0.011398	0.011329	0.011329	0.011364	0.011497	0.011564	0.011564
0.011430	0.011533	0.011535	0.011670	0.011661	0.011362	0.011530
0.011463	0.011463	0.011297	0.011478	0.011408	0.011435	0.011305
0.011473	0.011698	0.011441	0.011366	0.011262	0.011463	0.011329
0.011429	0.011362	0.011262	0.011363	0.011463	0.011466	0.011362
0.011262	0.011195	0.011329	0.011262	0.011195	0.011298	0.011463
0.011362	0.011295	0.011329	0.011362	0.011329	0.011262	0.011463
0.011329	0.011362	0.011362	0.011295	0.011362	0.011597	0.011262
0.011295	0.011698	0.011295	0.011329	0.011295	0.011664	0.011189
0.011622	0.011230	0.011261	0.011422	0.011508	0.011455	0.011144
0.011165	0.011463	0.011564	0.011671	0.011531	0.011669	0.011800
0.011699	0.011737	0.011698	0.011949	0.011664	0.012539	0.011700
0.012439	0.012207	0.012341	0.012612	0.011664	0.011362	0.011665
0.012103	0.012038	0.011631	0.011530	0.011598	0.011598	0.011567
0.011463	0.011467	0.011530	0.011497	0.011867	0.011933	0.011935
0.011834	0.011934	0.012038	0.011804	0.012104	0.012110	0.012171
0.012172	0.012205	0.012152	0.012172	0.012173	0.011396	0.011699
0.011766	0.012270	0.011772	0.012001	0.012474	0.011536	0.011430
0.011396	0.011396	0.011867	0.011883	0.012010	0.011732	0.011397
S171	0.011766	0.011766	0.012035	0.011799	0.011799	0.011866
0.011706	0.011638	0.011799	0.011363	0.011429	0.011397	0.011362
0.011329	0.011564	0.011295	0.011396	0.011463	0.011430	0.011396
0.011362	0.011396	0.011430	0.011363	0.011396	0.011396	0.011672
0.011564	0.011396	0.011295	0.011463	0.011463	0.011430	0.011463
0.011430	0.011530	0.011430	0.011430	0.011463	0.011430	0.011598
0.011295	0.011430	0.011463	0.011396	0.011430	0.011530	0.011463
0.011430	0.011598	0.011497	0.011698	0.011430	0.011698	0.011430
0.011396	0.011430	0.011564	0.011698	0.011732	0.011430	0.011262
0.011463	0.011530	0.011430	0.011665	0.011833	0.011430	0.011497
0.011396	0.011497	0.011463	0.011530	0.011463	0.011463	0.011463
0.011362	0.011396	0.011362	0.011396	0.011429	0.011464	0.011530
0.011463	0.011430	0.011497	0.011396	0.011463	0.011362	0.011430
0.011597	0.011530	0.011765	0.011598	0.011430	0.011665	0.011429
0.011616	0.011770	0.011664	0.011373	0.011359	0.011465	0.011493
0.012121	0.011518	0.011390	0.011665	0.011665	0.011396	0.011497
0.011497	0.011463	0.011530	0.011430	0.011530	0.011463	0.011463
0.011429	0.011463	0.011732	0.011597	0.011497	0.011463	0.011396

0.011362	0.011295	0.011430	0.011396	0.011530	0.011497	0.011430
0.011530	0.011597	0.011396	0.011396	0.011430	0.011429	0.011432
0.011500	0.011430	0.011430	0.011312	0.011386	0.011341	0.011430
0.011433	0.011370	0.011429	0.011405	0.011332	0.011369	0.011398
0.011564	0.011439	0.011474	0.011363	0.011371	0.011498	0.011397
0.011464	0.011465	0.011464	0.011564	0.011497	0.011866	0.011598
0.011631	0.011228	0.011430	0.000000	0.011329	0.011497	0.011362
0.011430	0.011430	0.011396	0.011362	0.011396	0.011362	0.011463
0.011430	0.011530	0.011463	0.011362	0.011465	0.011564	0.011567
0.011564	0.011463	0.011362	0.011296	0.011430	0.011362	0.011295
0.011400	0.011564	0.011362	0.011564	0.011329	0.011430	0.011396
0.011497	0.011396	0.011430	0.011396	0.011430	0.011295	0.011430
0.011463	0.011396	0.011463	0.011396	0.011430	0.011463	0.011698
0.011665	0.011665	0.011732	0.011566	0.011732	0.011429	0.011396
0.011364	0.011626	0.011518	0.011335	0.011705	0.011299	0.011375
0.011739	0.011638	0.011597	0.011799	0.011504	0.011665	0.011631
0.011564	0.011732	0.011593	0.011431	0.011522	0.011463	0.011534
0.011631	0.011533	0.011669	0.011737	0.011665	0.011665	0.011840
0.011568	0.011739	0.011670	0.011602	0.011868	0.011329	0.011532
0.011497	0.011430	0.011329	0.011329	0.011396	0.011396	0.011463
0.011396	0.011463	0.011362	0.011396	0.011396	0.011396	0.011463
0.011396	0.011765	0.011665	0.011698	0.011295	0.011396	0.011430
0.011430	0.011396	0.011463	0.011161	0.011362	0.011362	0.011362
0.011833	0.011463	0.011530	0.011329	0.011429	0.011598	0.011362
0.011362	0.011429	0.011396	0.011564	0.011362	0.011497	0.011631
0.011564	0.011396	0.011396	0.011329	0.011329	0.011497	0.011429
0.011329	0.011429	0.011295	0.011329	0.011463	0.011531	0.011362
0.011329	0.011463	0.011396	0.011295	0.011564	0.011295	0.011463
0.011540	0.011362	0.011598	0.011497	0.011463	0.011396	0.011497
0.011362	0.011698	0.011295	0.011497	0.011295	0.011295	0.011295
0.011530	0.011665	0.011430	0.011497	0.011329	0.011665	0.011295
0.011362	0.011362	0.011396	0.011497	0.011329	0.011497	0.011564
0.011564	0.011530	0.011429	0.011531	0.011497	0.011362	0.011329
0.011362	0.011362	0.011430	0.011295	0.011329	0.011497	0.011362
0.011329	0.011396	0.011530	0.011497	0.011463	0.011396	0.011396
0.011463	0.011396	0.011497	0.011362	0.011497	0.011597	0.011833
0.011396	0.011732	0.011405	0.017937	0.011453	0.011549	0.011688
0.011649	0.011694	0.011584	0.011525	0.011885	0.011716	0.011546
0.011630	0.011610	0.011333	0.011437	0.011255	0.011505	0.011447
0.011576	0.011507	0.011505	0.011259	0.011530	0.011564	0.011568
0.011438	0.011530	0.011530	0.011430	0.011463	0.011463	0.011430
0.011463	0.011396	0.011430	0.011463	0.011430	0.011362	0.011396
0.011396	0.011463	0.011362	0.011463	0.011497	0.011530	0.011429
0.011396	0.011363	0.011698	0.011698	0.011497	0.011531	0.011564
0.011329	0.011329	0.011362	0.011463	0.011329	0.011564	0.011564
0.011597	0.011597	0.011530	0.011631	0.011732	0.011633	0.011463
0.011463	0.011530	0.011564	0.011396	0.011463	0.011430	0.011463
0.011531	0.011530	0.011564	0.011531	0.011431	0.011631	0.011597
0.011467	0.011362	0.011463	0.011564	0.011463	0.011531	0.011631
0.011799	0.011504	0.011665	0.011631	0.011564	0.011732	0.011363
0.011532	0.011430	0.011396	0.011396	0.011463	0.011362	0.011463
0.011497	0.011530	0.011429	0.011396	0.011363	0.011698	0.011698
0.011497	0.011531	0.011597	0.011530	0.012104	0.011596	0.011518
0.011588	0.011546	0.011658	0.011545	0.011655	0.011578	0.011543
0.012057	0.011441	0.011574	0.011510	0.011715	0.011639	0.011741
0.012081	0.011550	0.011500	0.011463	0.011497	0.011430	0.011466
0.011329	0.011362	0.011497	0.011463	0.011466	0.011530	0.011498
0.011429	0.011362	0.011396	0.011430	0.011329	0.011396	0.011295
0.011362	0.011396	0.011362	0.011329	0.011430	0.011362	0.011396
0.011396	0.011329	0.011396	0.011430	0.011329	0.011530	0.011564

0.011463	0.011463	0.011463	0.011463	0.011396	0.011732	0.011430
0.011408	0.011396	0.011564	0.011569	0.011497	0.011497	0.011497
0.011497	0.011430	0.011396	0.011430	0.011530	0.011530	0.011463
0.011463	0.011598	0.011698	0.011497	0.011698	0.011698	0.011564
0.011396	0.011463	0.011497	0.011833	0.011463	0.011497	0.011463
0.011497	0.011362	0.011362	0.011665	0.011665	0.011631	0.011430
0.011530	0.011598	0.011463	0.011530	0.011497	0.011362	0.011631
0.011463	0.011430	0.011362	0.011598	0.011430	0.011530	0.011766
0.011463	0.011774	0.011766	0.011589	0.011286	0.011468	0.011370
0.011390	0.011800	0.011376	0.011552	0.011402	0.011518	0.011538
0.011519	0.011521	0.011561	0.011391	0.011492	0.011529	0.011547
0.011550	0.011454	0.011448	0.011412	0.011465	0.011530	0.011598
0.011597	0.011128	0.011229	0.011569	0.011636	0.011378	0.011463
0.011631	0.011228	0.011430	0.000000	0.011329	0.011497	0.011362
0.011430	0.011430	0.011396	0.011362	0.011396	0.011362	0.011463
0.011444	0.011410	0.011437	0.011471	0.011608	0.011573	0.011572
0.011571	0.011497	0.011319	0.011312	0.011433	0.011602	0.011603
0.011397	0.011467	0.011700	0.011669	0.011598	0.011632	0.011564
0.011497	0.011564	0.011564	0.011463	0.011968	0.011631	0.011699
0.011732	0.011701	0.011435	0.011511	0.011396	0.011597	0.011564
0.011599	0.011631	0.011631	0.011631	0.011768	0.011766	0.011407
0.011297	0.011303	0.011401	0.011366	0.011402	0.011284	0.011570
0.011438	0.011411	0.011251	0.011472	0.011430	0.011505	0.011396
0.011362	0.011295	0.011430	0.011396	0.011530	0.011497	0.011430
0.011530	0.011597	0.011396	0.011396	0.011430	0.011429	0.011432
0.011500	0.011430	0.011430	0.011465	0.011530	0.011598	0.011597
0.011128	0.011229	0.011569	0.011636	0.011378	0.011463	0.011631
0.011564	0.011564	0.011398	0.011582	0.011512	0.011537	0.011411
0.011577	0.011744	0.011483	0.011468	0.011362	0.011564	0.011430
0.011530	0.011463	0.011362	0.011465	0.011564	0.011567	0.011463
0.011362	0.011296	0.011430	0.011362	0.011295	0.011400	0.011564
0.011463	0.011396	0.011430	0.011463	0.011430	0.011362	0.011497
0.011430	0.011463	0.011463	0.011396	0.011463	0.011698	0.011295
0.011396	0.011732	0.011396	0.011430	0.011396	0.011698	0.011403
0.011791	0.011400	0.011361	0.011543	0.011630	0.011527	0.011353
0.011270	0.011497	0.011598	0.011705	0.011565	0.011703	0.011834
0.011733	0.011771	0.011732	0.011983	0.011698	0.012573	0.011734
0.012473	0.012241	0.012375	0.012647	0.011698	0.011396	0.011699
0.012137	0.012072	0.011665	0.011564	0.011632	0.011632	0.011602
0.011497	0.011501	0.011564	0.011531	0.011901	0.011967	0.011968
0.011868	0.011968	0.012072	0.011838	0.012138	0.012144	0.012205
0.012206	0.012239	0.012187	0.012206	0.012207	0.011497	0.011732
0.011800	0.012304	0.011806	0.012035	0.012508	0.011570	0.011464
0.011430	0.011429	0.011901	0.011883	0.012046	0.011765	0.011431
S172 0.011564	0.011564	0.011900	0.011665	0.011665	0.011732	
0.011570	0.011502	0.011665	0.011161	0.011228	0.011195	0.011161
0.011195	0.011362	0.011161	0.011195	0.011262	0.011228	0.011195
0.011161	0.011195	0.011295	0.011161	0.011195	0.011195	0.011536
0.011429	0.011195	0.011094	0.011329	0.011262	0.011228	0.011262
0.011228	0.011329	0.011228	0.011228	0.011262	0.011228	0.011396
0.011161	0.011228	0.011262	0.011195	0.011295	0.011329	0.011262
0.011228	0.011396	0.011295	0.011564	0.011228	0.011497	0.011228
0.011195	0.011295	0.011362	0.011497	0.011597	0.011295	0.011396
0.011262	0.011329	0.011228	0.011463	0.011698	0.011228	0.011295
0.011195	0.011295	0.011262	0.011329	0.011262	0.011262	0.011262
0.011161	0.011195	0.011161	0.011262	0.011228	0.011262	0.011329
0.011262	0.011228	0.011295	0.011195	0.011329	0.011161	0.011228
0.011396	0.011329	0.011564	0.011396	0.011228	0.011463	0.011228
0.011394	0.011620	0.011437	0.011138	0.011135	0.011244	0.011336
0.011975	0.011416	0.011143	0.011463	0.011463	0.011262	0.011362

0.011295	0.011262	0.011329	0.011228	0.011329	0.011262	0.011262
0.011228	0.011329	0.011530	0.011396	0.011295	0.011262	0.011195
0.011161	0.011161	0.011228	0.011195	0.011396	0.011362	0.011295
0.011396	0.011463	0.011195	0.011195	0.011228	0.011228	0.011229
0.011297	0.011228	0.011228	0.011096	0.011169	0.011130	0.011228
0.011230	0.011163	0.011208	0.011198	0.011128	0.011163	0.011195
0.011456	0.011233	0.011267	0.011161	0.011164	0.011296	0.011262
0.011329	0.011262	0.011262	0.011362	0.011295	0.011665	0.011463
0.011497	0.011362	0.011228	0.011329	0.000000	0.011362	0.011161
0.011228	0.011228	0.011195	0.011094	0.011195	0.011094	0.011262
0.011228	0.011329	0.011262	0.011161	0.011262	0.011362	0.011364
0.011362	0.011262	0.011161	0.011094	0.011228	0.011161	0.011094
0.011196	0.011362	0.011161	0.011362	0.011127	0.011228	0.011195
0.011295	0.011195	0.011228	0.011195	0.011228	0.011094	0.011228
0.011262	0.011195	0.011262	0.011195	0.011228	0.011262	0.011497
0.011597	0.011530	0.011597	0.011363	0.011597	0.011228	0.011195
0.011229	0.011505	0.011335	0.011129	0.011603	0.011094	0.011218
0.011603	0.011502	0.011396	0.011597	0.011299	0.011463	0.011430
0.011362	0.011530	0.011380	0.011225	0.011270	0.011262	0.011331
0.011430	0.011330	0.011466	0.011534	0.011463	0.011530	0.011636
0.011365	0.011535	0.011466	0.011398	0.011666	0.011127	0.011330
0.011295	0.011228	0.011127	0.011127	0.011195	0.011195	0.011262
0.011195	0.011262	0.011161	0.011262	0.011195	0.011195	0.011262
0.011195	0.011631	0.011530	0.011564	0.011094	0.011195	0.011228
0.011228	0.011195	0.011262	0.011295	0.011161	0.011161	0.011161
0.011698	0.011329	0.011329	0.011127	0.011228	0.011396	0.011161
0.011161	0.011228	0.011195	0.011362	0.011161	0.011295	0.011429
0.011362	0.011195	0.011195	0.011127	0.011127	0.011295	0.011228
0.011127	0.011228	0.011094	0.011127	0.011262	0.011396	0.011161
0.011128	0.011262	0.011195	0.011094	0.011429	0.011094	0.011262
0.011334	0.011161	0.011463	0.011295	0.011329	0.011195	0.011295
0.011161	0.011497	0.011094	0.011362	0.011161	0.011161	0.011094
0.011329	0.011531	0.011228	0.011295	0.011127	0.011530	0.011161
0.011161	0.011161	0.011195	0.011295	0.011195	0.011295	0.011362
0.011362	0.011329	0.011228	0.011329	0.011295	0.011161	0.011127
0.011161	0.011161	0.011228	0.011161	0.011127	0.011295	0.011161
0.011195	0.011195	0.011329	0.011362	0.011262	0.011195	0.011195
0.011262	0.011195	0.011295	0.011161	0.011295	0.011396	0.011698
0.011195	0.011597	0.011198	0.017710	0.011191	0.011366	0.011548
0.011510	0.011553	0.011270	0.011234	0.011664	0.011578	0.011303
0.011440	0.011487	0.011099	0.011186	0.011177	0.011258	0.011189
0.011436	0.011358	0.011357	0.011126	0.011329	0.011362	0.011365
0.011232	0.011329	0.011329	0.011295	0.011262	0.011262	0.011228
0.011262	0.011195	0.011228	0.011262	0.011228	0.011161	0.011195
0.011195	0.011262	0.011161	0.011262	0.011295	0.011329	0.011228
0.011262	0.011228	0.011564	0.011564	0.011295	0.011329	0.011362
0.011127	0.011127	0.011161	0.011262	0.011127	0.011362	0.011362
0.011396	0.011396	0.011329	0.011429	0.011597	0.011431	0.011262
0.011262	0.011329	0.011362	0.011195	0.011262	0.011228	0.011262
0.011329	0.011329	0.011362	0.011329	0.011229	0.011430	0.011396
0.011263	0.011161	0.011262	0.011362	0.011262	0.011396	0.011429
0.011597	0.011299	0.011463	0.011430	0.011362	0.011530	0.011161
0.011330	0.011228	0.011195	0.011195	0.011262	0.011161	0.011262
0.011295	0.011329	0.011228	0.011262	0.011228	0.011564	0.011564
0.011295	0.011329	0.011396	0.011329	0.011968	0.011382	0.011306
0.011377	0.011338	0.011447	0.011337	0.011445	0.011371	0.011336
0.011919	0.011233	0.011369	0.011302	0.011577	0.011434	0.011537
0.011944	0.011399	0.011297	0.011262	0.011295	0.011295	0.011263
0.011127	0.011161	0.011295	0.011262	0.011263	0.011396	0.011363
0.011228	0.011161	0.011195	0.011228	0.011127	0.011195	0.011094

0.011228	0.011262	0.011161	0.011127	0.011228	0.011161	0.011195
0.011195	0.011127	0.011195	0.011228	0.011127	0.011329	0.011362
0.011262	0.011329	0.011262	0.011262	0.011195	0.011597	0.011228
0.011269	0.011195	0.011430	0.011433	0.011295	0.011295	0.011295
0.011295	0.011228	0.011195	0.011228	0.011329	0.011329	0.011262
0.011262	0.011396	0.011564	0.011295	0.011497	0.011497	0.011362
0.011195	0.011262	0.011295	0.011698	0.011262	0.011295	0.011262
0.011295	0.011228	0.011228	0.011530	0.011463	0.011497	0.011228
0.011329	0.011396	0.011262	0.011329	0.011295	0.011161	0.011497
0.011262	0.011228	0.011228	0.011396	0.011228	0.011329	0.011564
0.011262	0.011638	0.011631	0.011426	0.011173	0.011332	0.011215
0.011170	0.011644	0.011166	0.011373	0.011207	0.011315	0.011326
0.011417	0.011126	0.011283	0.011126	0.011168	0.011328	0.011304
0.011324	0.011218	0.011307	0.011236	0.011262	0.011396	0.011463
0.011463	0.011329	0.011432	0.011433	0.011569	0.011590	0.011262
0.011497	0.011362	0.011228	0.011329	0.000000	0.011362	0.011161
0.011228	0.011228	0.011195	0.011094	0.011195	0.011094	0.011262
0.011235	0.011235	0.011231	0.011266	0.011402	0.011368	0.011367
0.011367	0.011296	0.011097	0.011096	0.011230	0.011399	0.011399
0.011195	0.011263	0.011498	0.011466	0.011396	0.011430	0.011362
0.011295	0.011362	0.011362	0.011262	0.011833	0.011430	0.011497
0.011530	0.011594	0.011231	0.011303	0.011195	0.011396	0.011362
0.011397	0.011429	0.011429	0.011429	0.011571	0.011564	0.011199
0.011094	0.011095	0.011196	0.011162	0.011265	0.011098	0.011434
0.011232	0.011323	0.011108	0.011248	0.011228	0.011300	0.011195
0.011161	0.011161	0.011228	0.011195	0.011396	0.011362	0.011295
0.011396	0.011463	0.011195	0.011195	0.011228	0.011228	0.011229
0.011297	0.011228	0.011228	0.011262	0.011396	0.011463	0.011463
0.011329	0.011432	0.011433	0.011569	0.011590	0.011262	0.011429
0.011362	0.011362	0.011196	0.011373	0.011303	0.011333	0.011200
0.011370	0.011561	0.011357	0.011264	0.011161	0.011362	0.011228
0.011329	0.011262	0.011161	0.011262	0.011362	0.011364	0.011262
0.011161	0.011094	0.011228	0.011161	0.011094	0.011196	0.011362
0.011262	0.011195	0.011228	0.011262	0.011228	0.011161	0.011362
0.011228	0.011262	0.011262	0.011195	0.011262	0.011497	0.011161
0.011195	0.011597	0.011195	0.011228	0.011195	0.011564	0.011146
0.011622	0.011145	0.011211	0.011382	0.011467	0.011383	0.011102
0.011165	0.011362	0.011463	0.011569	0.011430	0.011568	0.011699
0.011598	0.011635	0.011597	0.011846	0.011564	0.012438	0.011599
0.012338	0.012105	0.012240	0.012511	0.011564	0.011262	0.011564
0.012002	0.011937	0.011530	0.011430	0.011497	0.011497	0.011466
0.011362	0.011365	0.011429	0.011396	0.011766	0.011833	0.011834
0.011733	0.011833	0.011937	0.011703	0.012003	0.012008	0.012070
0.012070	0.012104	0.012050	0.012070	0.012072	0.011295	0.011598
0.011665	0.012169	0.011670	0.011900	0.012373	0.011434	0.011329
0.011295	0.011295	0.011766	0.011801	0.011904	0.011631	0.011296
S173	0.011799	0.011799	0.011866	0.011631	0.011631	0.011698
0.011536	0.011468	0.011631	0.011396	0.011463	0.011430	0.011396
0.011362	0.011597	0.011329	0.011429	0.011496	0.011463	0.011429
0.011396	0.011429	0.011262	0.011396	0.011430	0.011429	0.011502
0.011161	0.011429	0.011329	0.011295	0.011496	0.011463	0.011497
0.011463	0.011564	0.011463	0.011463	0.011496	0.011463	0.011631
0.011329	0.011463	0.011496	0.011429	0.011463	0.011564	0.011496
0.011463	0.011631	0.011530	0.011530	0.011463	0.011732	0.011463
0.011429	0.011463	0.011597	0.011732	0.011564	0.011463	0.011564
0.011496	0.011564	0.011463	0.011698	0.011665	0.011463	0.011530
0.011429	0.011530	0.011497	0.011564	0.011496	0.011497	0.011497
0.011396	0.011429	0.011396	0.011429	0.011463	0.011498	0.011564
0.011497	0.011463	0.011530	0.011429	0.011496	0.011396	0.011463
0.011631	0.011564	0.011597	0.011631	0.011463	0.011631	0.011463

0.011653	0.011583	0.011664	0.011412	0.011359	0.011502	0.011257
0.011902	0.011264	0.011390	0.011698	0.011698	0.011429	0.011530
0.011530	0.011496	0.011564	0.011463	0.011564	0.011496	0.011496
0.011463	0.011496	0.011765	0.011631	0.011530	0.011497	0.011429
0.011396	0.011329	0.011463	0.011429	0.011362	0.011329	0.011262
0.011362	0.011429	0.011429	0.011429	0.011463	0.011463	0.011465
0.011533	0.011463	0.011463	0.011348	0.011386	0.011376	0.011464
0.011467	0.011404	0.011429	0.011440	0.011366	0.011403	0.011432
0.011420	0.011474	0.011473	0.011396	0.011406	0.011531	0.011430
0.011497	0.011498	0.011497	0.011597	0.011530	0.011832	0.011631
0.011329	0.011530	0.011463	0.011497	0.011362	0.000000	0.011396
0.011463	0.011463	0.011429	0.011396	0.011429	0.011396	0.011497
0.011463	0.011564	0.011497	0.011396	0.011498	0.011597	0.011601
0.011597	0.011497	0.011396	0.011330	0.011463	0.011396	0.011329
0.011332	0.011598	0.011396	0.011597	0.011362	0.011463	0.011429
0.011530	0.011429	0.011463	0.011429	0.011463	0.011329	0.011463
0.011497	0.011429	0.011497	0.011429	0.011463	0.011497	0.011732
0.011564	0.011497	0.011564	0.011599	0.011564	0.011463	0.011429
0.011398	0.011667	0.011517	0.011369	0.011569	0.011333	0.011414
0.011569	0.011468	0.011631	0.011833	0.011538	0.011698	0.011665
0.011597	0.011765	0.011629	0.011513	0.011563	0.011497	0.011568
0.011665	0.011566	0.011703	0.011771	0.011698	0.011765	0.011873
0.011602	0.011773	0.011703	0.011635	0.011901	0.011362	0.011566
0.011530	0.011463	0.011362	0.011362	0.011429	0.011429	0.011496
0.011429	0.011497	0.011396	0.011429	0.011362	0.011429	0.011496
0.011429	0.011597	0.011497	0.011530	0.011329	0.011429	0.011463
0.011463	0.011429	0.011497	0.011463	0.011396	0.011396	0.011396
0.011665	0.011496	0.011564	0.011362	0.011463	0.011631	0.011396
0.011396	0.011463	0.011429	0.011597	0.011396	0.011329	0.011463
0.011396	0.011429	0.011429	0.011362	0.011362	0.011530	0.011463
0.011362	0.011463	0.011329	0.011362	0.011497	0.011363	0.011396
0.011363	0.011497	0.011430	0.011329	0.011396	0.011329	0.011497
0.011574	0.011396	0.011430	0.011530	0.011497	0.011429	0.011530
0.011396	0.011732	0.011329	0.011530	0.011329	0.011329	0.011329
0.011564	0.011497	0.011463	0.011530	0.011362	0.011497	0.011329
0.011396	0.011396	0.011429	0.011530	0.011429	0.011530	0.011597
0.011597	0.011564	0.011463	0.011564	0.011530	0.011396	0.011362
0.011396	0.011396	0.011463	0.011329	0.011362	0.011530	0.011396
0.011362	0.011429	0.011564	0.011530	0.011496	0.011429	0.011430
0.011496	0.011429	0.011530	0.011396	0.011530	0.011631	0.011665
0.011429	0.011564	0.011440	0.017936	0.011387	0.011672	0.011514
0.011475	0.011518	0.011636	0.011408	0.011610	0.011544	0.011668
0.011487	0.011365	0.011372	0.011479	0.011372	0.011554	0.011490
0.011342	0.011135	0.011320	0.011126	0.011564	0.011597	0.011601
0.011472	0.011564	0.011564	0.011463	0.011497	0.011497	0.011463
0.011497	0.011429	0.011463	0.011497	0.011463	0.011396	0.011429
0.011429	0.011497	0.011396	0.011497	0.011530	0.011564	0.011463
0.011429	0.011396	0.011530	0.011530	0.011530	0.011564	0.011597
0.011362	0.011362	0.011396	0.011497	0.011362	0.011597	0.011597
0.011631	0.011631	0.011564	0.011664	0.011564	0.011666	0.011497
0.011497	0.011564	0.011597	0.011429	0.011497	0.011463	0.011497
0.011564	0.011564	0.011597	0.011564	0.011464	0.011665	0.011631
0.011500	0.011396	0.011497	0.011597	0.011497	0.011564	0.011664
0.011833	0.011538	0.011698	0.011665	0.011597	0.011765	0.011396
0.011565	0.011463	0.011429	0.011429	0.011497	0.011396	0.011497
0.011530	0.011564	0.011463	0.011429	0.011396	0.011530	0.011530
0.011530	0.011564	0.011631	0.011564	0.012137	0.011632	0.011553
0.011623	0.011580	0.011693	0.011580	0.011690	0.011612	0.011577
0.012090	0.011476	0.011608	0.011544	0.011818	0.011672	0.011775
0.012114	0.011588	0.011669	0.011698	0.011664	0.011396	0.011635

0.011362	0.011530	0.011664	0.011631	0.011635	0.011094	0.011330
0.011463	0.011396	0.011429	0.011463	0.011362	0.011429	0.011329
0.011396	0.011429	0.011396	0.011362	0.011463	0.011396	0.011429
0.011429	0.011362	0.011429	0.011463	0.011362	0.011564	0.011597
0.011497	0.011497	0.011496	0.011497	0.011429	0.011564	0.011463
0.011234	0.011429	0.011161	0.011162	0.011530	0.011530	0.011530
0.011530	0.011463	0.011429	0.011463	0.011564	0.011564	0.011496
0.011496	0.011631	0.011530	0.011530	0.011732	0.011732	0.011597
0.011429	0.011497	0.011530	0.011665	0.011497	0.011530	0.011497
0.011530	0.011396	0.011396	0.011698	0.011698	0.011665	0.011463
0.011564	0.011631	0.011496	0.011564	0.011530	0.011396	0.011463
0.011496	0.011463	0.011396	0.011631	0.011463	0.011564	0.011597
0.011496	0.011604	0.011597	0.011101	0.011229	0.011502	0.011112
0.011445	0.011566	0.011376	0.011551	0.011402	0.011417	0.011379
0.011111	0.011324	0.011394	0.011258	0.011330	0.011529	0.011547
0.011588	0.011454	0.011483	0.011483	0.011498	0.011094	0.011161
0.011430	0.011497	0.011601	0.011399	0.011738	0.011767	0.011497
0.011329	0.011530	0.011463	0.011497	0.011362	0.000000	0.011396
0.011463	0.011463	0.011429	0.011396	0.011429	0.011396	0.011497
0.011479	0.011480	0.011471	0.011505	0.011642	0.011607	0.011606
0.011605	0.011531	0.011356	0.011348	0.011467	0.011636	0.011637
0.011430	0.011500	0.011733	0.011702	0.011632	0.011666	0.011597
0.011530	0.011597	0.011597	0.011497	0.011799	0.011664	0.011732
0.011765	0.011559	0.011469	0.011545	0.011429	0.011631	0.011597
0.011632	0.011664	0.011664	0.011664	0.011807	0.011799	0.011442
0.011331	0.011337	0.011435	0.011400	0.011436	0.011359	0.011400
0.011472	0.011323	0.011441	0.011510	0.011464	0.011539	0.011429
0.011396	0.011329	0.011463	0.011429	0.011362	0.011329	0.011262
0.011362	0.011429	0.011429	0.011429	0.011463	0.011463	0.011465
0.011533	0.011463	0.011463	0.011498	0.011094	0.011161	0.011430
0.011497	0.011601	0.011399	0.011738	0.011767	0.011497	0.011664
0.011597	0.011597	0.011432	0.011617	0.011547	0.011537	0.011446
0.011611	0.011789	0.011568	0.011502	0.011396	0.011597	0.011463
0.011564	0.011497	0.011396	0.011498	0.011597	0.011601	0.011497
0.011396	0.011330	0.011463	0.011396	0.011329	0.011332	0.011598
0.011497	0.011429	0.011463	0.011497	0.011463	0.011396	0.011329
0.011463	0.011497	0.011497	0.011429	0.011496	0.011732	0.011329
0.011429	0.011564	0.011429	0.011463	0.011429	0.011530	0.011403
0.011622	0.011273	0.011261	0.000000	0.011142	0.011096	0.011227
0.011218	0.011329	0.011429	0.011535	0.011397	0.011534	0.011665
0.011565	0.011601	0.011564	0.011812	0.011530	0.012404	0.011566
0.012305	0.012072	0.012206	0.012477	0.011530	0.011430	0.011531
0.012171	0.011903	0.011497	0.011396	0.011464	0.011464	0.011432
0.011329	0.011332	0.011396	0.011363	0.011733	0.011799	0.011800
0.011699	0.011800	0.011903	0.011669	0.012171	0.012178	0.012238
0.012239	0.012273	0.012220	0.012239	0.012240	0.011530	0.011766
0.011834	0.012337	0.011840	0.011866	0.012339	0.011400	0.011296
0.011262	0.011262	0.011732	0.011759	0.011869	0.011597	0.011262
S174	0.011598	0.011598	0.011934	0.011698	0.011698	0.011766
0.011604	0.011536	0.011698	0.011195	0.011262	0.011229	0.011195
0.011228	0.011396	0.011195	0.011228	0.011295	0.011262	0.011228
0.011195	0.011228	0.011329	0.011195	0.011228	0.011228	0.011570
0.011463	0.011228	0.011127	0.011362	0.011295	0.011262	0.011295
0.011262	0.011362	0.011262	0.011262	0.011295	0.011262	0.011430
0.011195	0.011262	0.011295	0.011228	0.011329	0.011362	0.011295
0.011262	0.011430	0.011329	0.011598	0.011262	0.011530	0.011262
0.011228	0.011329	0.011396	0.011530	0.011631	0.011329	0.011430
0.011295	0.011362	0.011262	0.011497	0.011732	0.011262	0.011329
0.011228	0.011329	0.011295	0.011362	0.011295	0.011295	0.011295
0.011195	0.011228	0.011195	0.011295	0.011262	0.011296	0.011362

0.011295	0.011262	0.011329	0.011228	0.011362	0.011195	0.011262
0.011430	0.011362	0.011598	0.011430	0.011262	0.011497	0.011262
0.011394	0.011621	0.011438	0.011138	0.011098	0.011244	0.011296
0.011938	0.011365	0.011102	0.011497	0.011497	0.011295	0.011396
0.011329	0.011295	0.011362	0.011262	0.011362	0.011295	0.011295
0.011262	0.011362	0.011564	0.011430	0.011329	0.011295	0.011228
0.011195	0.011195	0.011262	0.011228	0.011430	0.011396	0.011329
0.011430	0.011497	0.011228	0.011228	0.011262	0.011262	0.011263
0.011331	0.011262	0.011262	0.011132	0.011205	0.011165	0.011262
0.011263	0.011198	0.011208	0.011233	0.011162	0.011197	0.011229
0.011492	0.011267	0.011301	0.011195	0.011199	0.011329	0.011296
0.011363	0.011296	0.011296	0.011396	0.011329	0.011698	0.011497
0.011530	0.011396	0.011262	0.011362	0.011161	0.011396	0.000000
0.011262	0.011262	0.011228	0.011195	0.011228	0.011195	0.011295
0.011262	0.011362	0.011295	0.011195	0.011296	0.011396	0.011398
0.011396	0.011295	0.011195	0.011128	0.011262	0.011195	0.011128
0.011230	0.011396	0.011195	0.011396	0.011161	0.011262	0.011228
0.011329	0.011228	0.011262	0.011228	0.011262	0.011127	0.011262
0.011295	0.011228	0.011295	0.011228	0.011262	0.011295	0.011530
0.011631	0.011564	0.011631	0.011397	0.011631	0.011262	0.011228
0.011263	0.011545	0.011335	0.011129	0.011637	0.011129	0.011257
0.011638	0.011536	0.011430	0.011631	0.011333	0.011497	0.011463
0.011396	0.011564	0.011416	0.011266	0.011228	0.011295	0.011365
0.011463	0.011364	0.011500	0.011568	0.011497	0.011564	0.011670
0.011399	0.011570	0.011501	0.011432	0.011700	0.011161	0.011364
0.011329	0.011262	0.011161	0.011161	0.011228	0.011228	0.011295
0.011228	0.011295	0.011195	0.011295	0.011228	0.011228	0.011295
0.011228	0.011665	0.011564	0.011598	0.011127	0.011228	0.011262
0.011262	0.011228	0.011295	0.011329	0.011195	0.011195	0.011195
0.011732	0.011362	0.011362	0.011161	0.011262	0.011430	0.011195
0.011195	0.011262	0.011228	0.011396	0.011195	0.011329	0.011463
0.011396	0.011228	0.011228	0.011161	0.011161	0.011329	0.011262
0.011161	0.011262	0.011127	0.011161	0.011295	0.011430	0.011195
0.011161	0.011295	0.011228	0.011127	0.011463	0.011127	0.011295
0.011368	0.011195	0.011497	0.011329	0.011362	0.011228	0.011329
0.011195	0.011530	0.011127	0.011396	0.011195	0.011195	0.011127
0.011362	0.011564	0.011262	0.011329	0.011161	0.011564	0.011195
0.011195	0.011195	0.011228	0.011329	0.011228	0.011329	0.011396
0.011396	0.011362	0.011262	0.011363	0.011329	0.011195	0.011161
0.011195	0.011195	0.011262	0.011195	0.011161	0.011329	0.011195
0.011228	0.011228	0.011362	0.011396	0.011295	0.011228	0.011228
0.011295	0.011228	0.011329	0.011195	0.011329	0.011430	0.011732
0.011228	0.011631	0.011198	0.017674	0.011126	0.011366	0.011549
0.011510	0.011553	0.011217	0.011176	0.011665	0.011578	0.011243
0.011392	0.011488	0.000000	0.011186	0.011177	0.011258	0.011189
0.011389	0.011321	0.011320	0.011126	0.011362	0.011396	0.011398
0.011266	0.011362	0.011362	0.011329	0.011295	0.011295	0.011262
0.011295	0.011228	0.011262	0.011295	0.011262	0.011195	0.011228
0.011228	0.011295	0.011195	0.011295	0.011329	0.011362	0.011262
0.011295	0.011262	0.011598	0.011598	0.011329	0.011363	0.011396
0.011161	0.011161	0.011195	0.011295	0.011161	0.011396	0.011396
0.011430	0.011430	0.011362	0.011463	0.011631	0.011464	0.011295
0.011295	0.011362	0.011396	0.011228	0.011295	0.011262	0.011295
0.011363	0.011363	0.011396	0.011363	0.011262	0.011463	0.011430
0.011297	0.011195	0.011295	0.011396	0.011295	0.011430	0.011463
0.011631	0.011333	0.011497	0.011463	0.011396	0.011564	0.011195
0.011363	0.011262	0.011228	0.011228	0.011295	0.011195	0.011295
0.011329	0.011362	0.011262	0.011295	0.011262	0.011598	0.011598
0.011329	0.011363	0.011430	0.011362	0.012002	0.011418	0.011342
0.011412	0.011372	0.011482	0.011372	0.011480	0.011405	0.011371

0.011953	0.011268	0.011403	0.011337	0.011611	0.011469	0.011571
0.011979	0.011437	0.011331	0.011295	0.011329	0.011329	0.011297
0.011161	0.011195	0.011329	0.011295	0.011297	0.011430	0.011397
0.011262	0.011195	0.011228	0.011262	0.011161	0.011228	0.011127
0.011262	0.011295	0.011195	0.011161	0.011262	0.011195	0.011228
0.011228	0.011161	0.011228	0.011262	0.011161	0.011362	0.011396
0.011295	0.011362	0.011295	0.011295	0.011228	0.011631	0.011262
0.011304	0.011228	0.011464	0.011467	0.011329	0.011329	0.011329
0.011329	0.011262	0.011228	0.011262	0.011362	0.011362	0.011295
0.011295	0.011430	0.011598	0.011329	0.011530	0.011530	0.011396
0.011228	0.011295	0.011329	0.011732	0.011295	0.011329	0.011295
0.011329	0.011262	0.011262	0.011564	0.011497	0.011530	0.011262
0.011362	0.011430	0.011295	0.011362	0.011329	0.011195	0.011530
0.011295	0.011262	0.011262	0.011430	0.011262	0.011362	0.011598
0.011295	0.011672	0.011665	0.011385	0.011117	0.011366	0.011164
0.011170	0.011605	0.011166	0.011328	0.011158	0.011264	0.011273
0.011366	0.000000	0.011227	0.000000	0.011114	0.011261	0.011304
0.011324	0.011178	0.011307	0.011201	0.011296	0.011430	0.011497
0.011497	0.011363	0.011466	0.011467	0.011603	0.011626	0.011295
0.011530	0.011396	0.011262	0.011362	0.011161	0.011396	0.000000
0.011262	0.011262	0.011228	0.011195	0.011228	0.011195	0.011295
0.011270	0.011270	0.011266	0.011300	0.011437	0.011402	0.011402
0.011401	0.011329	0.011134	0.011132	0.011263	0.011433	0.011433
0.011228	0.011297	0.011532	0.011500	0.011430	0.011464	0.011396
0.011329	0.011396	0.011396	0.011295	0.011867	0.011463	0.011531
0.011564	0.011630	0.011265	0.011337	0.011228	0.011430	0.011396
0.011430	0.011463	0.011463	0.011463	0.011611	0.011598	0.011234
0.011128	0.011130	0.011231	0.011196	0.011299	0.011135	0.011468
0.011266	0.011411	0.011156	0.011285	0.011262	0.011334	0.011228
0.011195	0.011195	0.011262	0.011228	0.011430	0.011396	0.011329
0.011430	0.011497	0.011228	0.011228	0.011262	0.011262	0.011263
0.011331	0.011262	0.011262	0.011296	0.011430	0.011497	0.011497
0.011363	0.011466	0.011467	0.011603	0.011626	0.011295	0.011463
0.011396	0.011396	0.011229	0.011408	0.011338	0.011367	0.011235
0.011405	0.011607	0.011399	0.011298	0.011195	0.011396	0.011262
0.011362	0.011295	0.011195	0.011296	0.011396	0.011398	0.011295
0.011195	0.011128	0.011262	0.011195	0.011128	0.011230	0.011396
0.011295	0.011228	0.011262	0.011295	0.011262	0.011195	0.011396
0.011262	0.011295	0.011295	0.011228	0.011295	0.011530	0.011195
0.011228	0.011631	0.011228	0.011262	0.011228	0.011598	0.011103
0.011566	0.011103	0.011161	0.011342	0.011427	0.011383	0.000000
0.011113	0.011396	0.011497	0.011603	0.011464	0.011568	0.011733
0.011632	0.011669	0.011631	0.011846	0.011598	0.012472	0.011633
0.012372	0.012140	0.012274	0.012545	0.011598	0.011295	0.011598
0.012036	0.011971	0.011564	0.011463	0.011531	0.011531	0.011500
0.011396	0.011365	0.011463	0.011430	0.011800	0.011867	0.011868
0.011767	0.011867	0.011971	0.011703	0.012037	0.012042	0.012104
0.012104	0.012138	0.012050	0.012105	0.012106	0.011329	0.011632
0.011699	0.012203	0.011704	0.011934	0.012407	0.011468	0.011363
0.011329	0.011329	0.011800	0.011842	0.011940	0.011665	0.011330
S175	0.011664	0.011664	0.012001	0.011765	0.011765	0.011832
0.011671	0.011603	0.011832	0.011262	0.011329	0.011296	0.011262
0.011295	0.011463	0.011262	0.011295	0.011362	0.011329	0.011295
0.011262	0.011295	0.011396	0.011262	0.011295	0.011295	0.011637
0.011530	0.011295	0.011194	0.011429	0.011362	0.011329	0.011362
0.011329	0.011429	0.011329	0.011329	0.011362	0.011329	0.011497
0.011262	0.011329	0.011362	0.011295	0.011396	0.011429	0.011362
0.011329	0.011497	0.011396	0.011664	0.011329	0.011597	0.011329
0.011295	0.011396	0.011463	0.011597	0.011698	0.011329	0.011496
0.011362	0.011429	0.011329	0.011564	0.011799	0.011329	0.011396

0.011295	0.011396	0.011362	0.011429	0.011362	0.011362	0.011362
0.011262	0.011295	0.011262	0.011362	0.011329	0.011363	0.011429
0.011362	0.011329	0.011396	0.011295	0.011429	0.011262	0.011329
0.011496	0.011429	0.011664	0.011497	0.011329	0.011564	0.011329
0.011505	0.011733	0.011513	0.011216	0.011210	0.011355	0.011415
0.012048	0.011467	0.011184	0.011564	0.011564	0.011362	0.011463
0.011396	0.011362	0.011429	0.011329	0.011429	0.011362	0.011362
0.011329	0.011429	0.011631	0.011496	0.011396	0.011362	0.011295
0.011262	0.011262	0.011329	0.011295	0.011497	0.011463	0.011396
0.011497	0.011564	0.011295	0.011295	0.011329	0.011329	0.011330
0.011398	0.011329	0.011329	0.011168	0.011241	0.011200	0.011329
0.011297	0.011232	0.011282	0.011267	0.011230	0.011232	0.011297
0.011528	0.011302	0.011336	0.011262	0.011233	0.011397	0.011363
0.011430	0.011364	0.011363	0.011463	0.011396	0.011765	0.011564
0.011597	0.011463	0.011329	0.011430	0.011228	0.011463	0.011262
0.000000	0.011329	0.011295	0.011262	0.011295	0.011262	0.011362
0.011329	0.011430	0.011362	0.011262	0.011363	0.011463	0.011466
0.011463	0.011362	0.011262	0.011195	0.011329	0.011262	0.011195
0.011298	0.011463	0.011262	0.011463	0.011195	0.011329	0.011295
0.011396	0.011295	0.011329	0.011295	0.011329	0.011195	0.011329
0.011362	0.011295	0.011362	0.011295	0.011329	0.011362	0.011597
0.011698	0.011631	0.011698	0.011465	0.011698	0.011329	0.011295
0.011330	0.011626	0.011472	0.011232	0.011671	0.011197	0.011336
0.011705	0.011604	0.011496	0.011698	0.011402	0.011564	0.011530
0.011463	0.011631	0.011486	0.011307	0.011228	0.011362	0.011432
0.011530	0.011431	0.011567	0.011635	0.011564	0.011631	0.011738
0.011466	0.011637	0.011568	0.011500	0.011767	0.011228	0.011431
0.011396	0.011329	0.011228	0.011228	0.011295	0.011295	0.011362
0.011295	0.011362	0.011262	0.011362	0.011295	0.011295	0.011362
0.011295	0.011731	0.011631	0.011664	0.011194	0.011295	0.011329
0.011329	0.011295	0.011362	0.011396	0.011262	0.011262	0.011262
0.011799	0.011429	0.011429	0.011228	0.011329	0.011497	0.011262
0.011262	0.011329	0.011295	0.011463	0.011262	0.011396	0.011530
0.011463	0.011295	0.011295	0.011228	0.011228	0.011396	0.011329
0.011228	0.011329	0.011195	0.011228	0.011362	0.011497	0.011262
0.011228	0.011362	0.011295	0.011195	0.011530	0.011195	0.011362
0.011437	0.011262	0.011564	0.011396	0.011429	0.011295	0.011396
0.011262	0.011597	0.011194	0.011463	0.011262	0.011262	0.011194
0.011429	0.011631	0.011329	0.011396	0.011228	0.011631	0.011262
0.011262	0.011262	0.011295	0.011396	0.011295	0.011396	0.011463
0.011463	0.011429	0.011329	0.011430	0.011396	0.011262	0.011228
0.011262	0.011262	0.011329	0.011262	0.011228	0.011396	0.011262
0.011295	0.011295	0.011429	0.011463	0.011362	0.011295	0.011295
0.011362	0.011295	0.011396	0.011262	0.011396	0.011496	0.011799
0.011295	0.011698	0.011302	0.017785	0.011126	0.011427	0.011653
0.011614	0.011658	0.011270	0.011292	0.011719	0.011682	0.011364
0.011440	0.011548	0.011177	0.011228	0.011216	0.011307	0.011275
0.011482	0.011433	0.011394	0.011193	0.011429	0.011463	0.011466
0.011335	0.011430	0.011429	0.011396	0.011362	0.011362	0.011329
0.011362	0.011295	0.011329	0.011362	0.011329	0.011262	0.011295
0.011295	0.011362	0.011262	0.011363	0.011396	0.011430	0.011329
0.011362	0.011329	0.011665	0.011665	0.011396	0.011430	0.011463
0.011228	0.011228	0.011262	0.011362	0.011228	0.011463	0.011463
0.011497	0.011496	0.011429	0.011530	0.011698	0.011532	0.011362
0.011362	0.011430	0.011463	0.011295	0.011362	0.011329	0.011362
0.011430	0.011430	0.011463	0.011430	0.011330	0.011530	0.011497
0.011365	0.011262	0.011362	0.011463	0.011295	0.011497	0.011530
0.011698	0.011402	0.011564	0.011530	0.011463	0.011631	0.011262
0.011431	0.011329	0.011295	0.011295	0.011362	0.011262	0.011363
0.011396	0.011430	0.011329	0.011362	0.011329	0.011665	0.011665

0.011396	0.011430	0.011429	0.011429	0.012069	0.011489	0.011412
0.011482	0.011442	0.011552	0.011441	0.011550	0.011474	0.011405
0.012022	0.011337	0.011471	0.011406	0.011680	0.011536	0.011639
0.012046	0.011512	0.011601	0.011664	0.011530	0.011530	0.011567
0.011228	0.011396	0.011530	0.011496	0.011499	0.011496	0.011464
0.011329	0.011262	0.011295	0.011329	0.011228	0.011295	0.011194
0.011329	0.011362	0.011262	0.011228	0.011329	0.011262	0.011295
0.011295	0.011228	0.011295	0.011329	0.011228	0.011429	0.011463
0.011362	0.011429	0.011362	0.011362	0.011295	0.011698	0.011329
0.011373	0.011295	0.011530	0.011535	0.011396	0.011396	0.011396
0.011396	0.011329	0.011295	0.011329	0.011429	0.011429	0.011362
0.011362	0.011497	0.011664	0.011396	0.011597	0.011597	0.011463
0.011295	0.011362	0.011396	0.011799	0.011362	0.011396	0.011362
0.011396	0.011329	0.011329	0.011631	0.011564	0.011597	0.011329
0.011429	0.011497	0.011362	0.011429	0.011396	0.011262	0.011597
0.011362	0.011262	0.011329	0.011497	0.011329	0.011429	0.011664
0.011362	0.011739	0.011732	0.011507	0.011173	0.011434	0.011215
0.011225	0.011722	0.011271	0.011373	0.011255	0.011366	0.011326
0.011468	0.000000	0.011283	0.000000	0.011168	0.011261	0.011425
0.011437	0.011336	0.011412	0.011342	0.011364	0.011497	0.011564
0.011564	0.011430	0.011534	0.011535	0.011670	0.011696	0.011362
0.011597	0.011463	0.011329	0.011430	0.011228	0.011463	0.011262
0.000000	0.011329	0.011295	0.011262	0.011295	0.011262	0.011362
0.011305	0.011305	0.011300	0.011334	0.011471	0.011436	0.011436
0.011435	0.011396	0.011171	0.011168	0.011297	0.011467	0.011467
0.011296	0.011331	0.011531	0.011567	0.011497	0.011531	0.011463
0.011396	0.011463	0.011463	0.011362	0.011933	0.011530	0.011597
0.011564	0.011630	0.011333	0.011407	0.011295	0.011497	0.011463
0.011497	0.011530	0.011530	0.011530	0.011611	0.011664	0.011303
0.011195	0.011199	0.011299	0.011264	0.011368	0.011172	0.011536
0.011300	0.011367	0.011108	0.011322	0.011329	0.011368	0.011295
0.011262	0.011262	0.011329	0.011295	0.011497	0.011463	0.011396
0.011497	0.011564	0.011295	0.011295	0.011329	0.011329	0.011330
0.011398	0.011329	0.011329	0.011364	0.011497	0.011564	0.011564
0.011430	0.011534	0.011535	0.011670	0.011696	0.011362	0.011530
0.011463	0.011463	0.011297	0.011443	0.011408	0.011435	0.011305
0.011473	0.011698	0.011483	0.011366	0.011262	0.011463	0.011329
0.011430	0.011362	0.011262	0.011363	0.011463	0.011466	0.011362
0.011262	0.011195	0.011329	0.011262	0.011195	0.011298	0.011463
0.011362	0.011295	0.011329	0.011362	0.011329	0.011262	0.011463
0.011329	0.011362	0.011362	0.011295	0.011362	0.011597	0.011262
0.011295	0.011698	0.011295	0.011329	0.011295	0.011664	0.011189
0.011622	0.011230	0.011261	0.011463	0.011549	0.011491	0.011186
0.011218	0.011463	0.011564	0.011671	0.011531	0.011669	0.011800
0.011699	0.011737	0.011698	0.011949	0.011664	0.012539	0.011700
0.012439	0.012207	0.012341	0.012613	0.011664	0.011363	0.011665
0.012103	0.012038	0.011631	0.011530	0.011598	0.011598	0.011567
0.011463	0.011467	0.011530	0.011497	0.011867	0.011933	0.011935
0.011834	0.011934	0.012038	0.011804	0.012104	0.012110	0.012171
0.012172	0.012205	0.012152	0.012172	0.012173	0.011396	0.011699
0.011767	0.012270	0.011772	0.012001	0.012474	0.011536	0.011430
0.011396	0.011396	0.011867	0.011883	0.011940	0.011732	0.011397
S176	0.011664	0.011664	0.012001	0.011765	0.011765	0.011832
0.011671	0.011603	0.011832	0.011262	0.011329	0.011296	0.011262
0.011295	0.011463	0.011262	0.011295	0.011362	0.011329	0.011295
0.011262	0.011295	0.011396	0.011262	0.011295	0.011295	0.011637
0.011530	0.011295	0.011194	0.011429	0.011362	0.011329	0.011362
0.011329	0.011429	0.011329	0.011329	0.011362	0.011329	0.011496
0.011262	0.011329	0.011362	0.011295	0.011396	0.011429	0.011362
0.011329	0.011497	0.011396	0.011664	0.011329	0.011597	0.011329

0.011295	0.011396	0.011463	0.011597	0.011698	0.011396	0.011496
0.011362	0.011429	0.011329	0.011564	0.011799	0.011329	0.011396
0.011295	0.011396	0.011362	0.011429	0.011362	0.011362	0.011362
0.011262	0.011295	0.011262	0.011362	0.011329	0.011363	0.011429
0.011362	0.011329	0.011396	0.011295	0.011429	0.011262	0.011329
0.011496	0.011429	0.011664	0.011497	0.011329	0.011564	0.011329
0.011505	0.011733	0.011513	0.011216	0.011172	0.011355	0.011414
0.012048	0.011467	0.011184	0.011564	0.011564	0.011362	0.011463
0.011396	0.011362	0.011429	0.011329	0.011429	0.011362	0.011362
0.011329	0.011429	0.011631	0.011496	0.011396	0.011362	0.011295
0.011262	0.011262	0.011329	0.011295	0.011497	0.011463	0.011396
0.011497	0.011564	0.011295	0.011295	0.011329	0.011329	0.011330
0.011398	0.011329	0.011329	0.011204	0.011277	0.011236	0.011329
0.011331	0.011266	0.011282	0.011302	0.011230	0.011266	0.011297
0.011564	0.011336	0.011370	0.011262	0.011268	0.011397	0.011363
0.011430	0.011363	0.011363	0.011463	0.011396	0.011765	0.011564
0.011597	0.011463	0.011329	0.011430	0.011228	0.011463	0.011262
0.011329	0.000000	0.011295	0.011262	0.011295	0.011262	0.011362
0.011329	0.011429	0.011362	0.011262	0.011363	0.011463	0.011466
0.011463	0.011362	0.011262	0.011195	0.011329	0.011262	0.011195
0.011298	0.011463	0.011262	0.011463	0.011228	0.011329	0.011295
0.011396	0.011295	0.011329	0.011295	0.011329	0.011195	0.011329
0.011362	0.011295	0.011362	0.011295	0.011329	0.011362	0.011597
0.011698	0.011631	0.011698	0.011465	0.011698	0.011329	0.011295
0.011330	0.011545	0.011380	0.011232	0.011705	0.011197	0.011296
0.011705	0.011604	0.011496	0.011698	0.011402	0.011564	0.011530
0.011463	0.011631	0.011486	0.011349	0.011354	0.011362	0.011432
0.011530	0.011431	0.011567	0.011635	0.011564	0.011631	0.011738
0.011466	0.011637	0.011568	0.011500	0.011767	0.011228	0.011431
0.011396	0.011329	0.011228	0.011228	0.011295	0.011295	0.011362
0.011295	0.011362	0.011262	0.011362	0.011295	0.011295	0.011362
0.011295	0.011731	0.011631	0.011664	0.011194	0.011295	0.011329
0.011329	0.011295	0.011362	0.011396	0.011262	0.011262	0.011262
0.011799	0.011429	0.011429	0.011228	0.011329	0.011497	0.011262
0.011262	0.011329	0.011295	0.011463	0.011262	0.011396	0.011530
0.011463	0.011295	0.011295	0.011228	0.011228	0.011396	0.011329
0.011228	0.011329	0.011195	0.011228	0.011362	0.011497	0.011262
0.011228	0.011362	0.011295	0.011195	0.011530	0.011195	0.011362
0.011437	0.011262	0.011564	0.011396	0.011429	0.011295	0.011396
0.011262	0.011597	0.011194	0.011463	0.011262	0.011262	0.011194
0.011429	0.011631	0.011329	0.011396	0.011228	0.011631	0.011262
0.011262	0.011262	0.011295	0.011396	0.011295	0.011396	0.011463
0.011463	0.011429	0.011329	0.011429	0.011396	0.011262	0.011228
0.011262	0.011262	0.011329	0.011262	0.011228	0.011396	0.011262
0.011295	0.011295	0.011429	0.011463	0.011362	0.011295	0.011295
0.011362	0.011295	0.011396	0.011262	0.011396	0.011496	0.011799
0.011295	0.011698	0.011302	0.017785	0.011191	0.011427	0.011653
0.011614	0.011658	0.011270	0.011234	0.011774	0.011682	0.011303
0.011487	0.011548	0.011216	0.011228	0.011294	0.011356	0.011232
0.011482	0.011433	0.011394	0.011259	0.011429	0.011463	0.011466
0.011335	0.011429	0.011429	0.011396	0.011362	0.011362	0.011329
0.011362	0.011295	0.011329	0.011362	0.011329	0.011262	0.011295
0.011295	0.011362	0.011262	0.011362	0.011396	0.011429	0.011329
0.011362	0.011329	0.011665	0.011665	0.011396	0.011430	0.011463
0.011228	0.011228	0.011262	0.011362	0.011228	0.011463	0.011463
0.011497	0.011496	0.011429	0.011530	0.011698	0.011532	0.011362
0.011362	0.011429	0.011463	0.011295	0.011362	0.011329	0.011362
0.011430	0.011430	0.011463	0.011430	0.011330	0.011530	0.011497
0.011365	0.011262	0.011362	0.011463	0.011362	0.011497	0.011530
0.011698	0.011402	0.011564	0.011530	0.011463	0.011631	0.011262

0.011431	0.011329	0.011295	0.011295	0.011362	0.011262	0.011362
0.011396	0.011429	0.011329	0.011362	0.011329	0.011665	0.011665
0.011396	0.011430	0.011496	0.011429	0.012069	0.011489	0.011412
0.011482	0.011442	0.011552	0.011441	0.011550	0.011474	0.011439
0.012021	0.011337	0.011471	0.011406	0.011680	0.011536	0.011639
0.012046	0.011512	0.011601	0.011664	0.011530	0.011530	0.011567
0.011228	0.011396	0.011530	0.011496	0.011499	0.011496	0.011464
0.011329	0.011262	0.011295	0.011329	0.011228	0.011295	0.011194
0.011329	0.011362	0.011262	0.011228	0.011329	0.011262	0.011295
0.011295	0.011228	0.011295	0.011329	0.011228	0.011429	0.011463
0.011362	0.011429	0.011362	0.011362	0.011295	0.011698	0.011329
0.011373	0.011295	0.011530	0.011535	0.011396	0.011396	0.011396
0.011396	0.011329	0.011295	0.011329	0.011429	0.011429	0.011362
0.011362	0.011496	0.011664	0.011396	0.011597	0.011597	0.011463
0.011295	0.011362	0.011396	0.011799	0.011362	0.011396	0.011362
0.011396	0.011329	0.011329	0.011631	0.011564	0.011597	0.011329
0.011429	0.011496	0.011362	0.011429	0.011396	0.011262	0.011597
0.011362	0.011329	0.011329	0.011496	0.011329	0.011429	0.011664
0.011362	0.011739	0.011731	0.011507	0.011229	0.011434	0.011267
0.011225	0.011722	0.011271	0.011417	0.011255	0.011366	0.011379
0.011468	0.011126	0.011338	0.011192	0.011222	0.011395	0.011425
0.011437	0.011296	0.011412	0.011307	0.011364	0.011497	0.011564
0.011564	0.011430	0.011533	0.011535	0.011670	0.011696	0.011362
0.011597	0.011463	0.011329	0.011430	0.011228	0.011463	0.011262
0.011329	0.000000	0.011295	0.011262	0.011295	0.011262	0.011362
0.011340	0.011340	0.011334	0.011368	0.011505	0.011470	0.011470
0.011469	0.011396	0.011208	0.011204	0.011331	0.011501	0.011501
0.011296	0.011365	0.011599	0.011567	0.011497	0.011531	0.011463
0.011396	0.011463	0.011463	0.011362	0.011933	0.011530	0.011597
0.011631	0.011701	0.011299	0.011407	0.011295	0.011497	0.011463
0.011497	0.011530	0.011530	0.011530	0.011650	0.011664	0.011303
0.011195	0.011164	0.011298	0.011264	0.011368	0.011172	0.011536
0.011335	0.011455	0.011203	0.011360	0.011329	0.011402	0.011295
0.011262	0.011262	0.011329	0.011295	0.011497	0.011463	0.011396
0.011497	0.011564	0.011295	0.011295	0.011329	0.011329	0.011330
0.011398	0.011329	0.011329	0.011364	0.011497	0.011564	0.011564
0.011430	0.011533	0.011535	0.011670	0.011696	0.011362	0.011530
0.011463	0.011463	0.011297	0.011443	0.011408	0.011435	0.011305
0.011473	0.011698	0.011441	0.011366	0.011262	0.011463	0.011329
0.011429	0.011362	0.011262	0.011363	0.011463	0.011466	0.011362
0.011262	0.011195	0.011329	0.011262	0.011195	0.011298	0.011463
0.011362	0.011295	0.011329	0.011362	0.011329	0.011262	0.011463
0.011329	0.011362	0.011362	0.011295	0.011362	0.011597	0.011262
0.011295	0.011698	0.011295	0.011329	0.011295	0.011664	0.011189
0.011678	0.011230	0.011261	0.011463	0.011549	0.011491	0.011144
0.011218	0.011463	0.011564	0.011671	0.011531	0.011669	0.011800
0.011699	0.011737	0.011698	0.011949	0.011664	0.012539	0.011700
0.012439	0.012207	0.012341	0.012545	0.011664	0.011362	0.011665
0.012103	0.012038	0.011631	0.011530	0.011598	0.011598	0.011567
0.011463	0.011467	0.011530	0.011497	0.011867	0.011933	0.011935
0.011834	0.011934	0.012038	0.011804	0.012104	0.012110	0.012171
0.012172	0.012205	0.012152	0.012172	0.012173	0.011396	0.011699
0.011766	0.012270	0.011772	0.012001	0.012474	0.011536	0.011430
0.011396	0.011396	0.011867	0.011883	0.012010	0.011731	0.011397
S177	0.011631	0.011631	0.011967	0.011732	0.011732	0.011799
0.011638	0.011570	0.011732	0.011228	0.011295	0.011262	0.011228
0.011262	0.011429	0.011228	0.011262	0.011329	0.011295	0.011262
0.011228	0.011262	0.011362	0.011228	0.011262	0.011262	0.011604
0.011497	0.011262	0.011161	0.011396	0.011329	0.011295	0.011329
0.011295	0.011396	0.011295	0.011295	0.011329	0.011295	0.011463

0.011228	0.011295	0.011329	0.011262	0.011362	0.011396	0.011329
0.011295	0.011463	0.011363	0.011631	0.011295	0.011564	0.011295
0.011262	0.011362	0.011430	0.011564	0.011665	0.011362	0.011463
0.011329	0.011396	0.011295	0.011530	0.011766	0.011295	0.011362
0.011262	0.011362	0.011329	0.011396	0.011329	0.011329	0.011329
0.011228	0.011262	0.011228	0.011329	0.011295	0.011329	0.011396
0.011329	0.011295	0.011362	0.011262	0.011396	0.011228	0.011295
0.011463	0.011396	0.011631	0.011463	0.011295	0.011530	0.011295
0.011431	0.011658	0.011475	0.011177	0.011172	0.011281	0.011375
0.012011	0.011416	0.011184	0.011530	0.011530	0.011329	0.011430
0.011362	0.011329	0.011396	0.011295	0.011396	0.011329	0.011329
0.011295	0.011396	0.011597	0.011463	0.011362	0.011329	0.011262
0.011228	0.011228	0.011295	0.011262	0.011463	0.011429	0.011362
0.011463	0.011530	0.011262	0.011262	0.011295	0.011295	0.011297
0.011364	0.011295	0.011295	0.011168	0.011241	0.011200	0.011296
0.011297	0.011232	0.011245	0.011267	0.011196	0.011231	0.011263
0.011528	0.011301	0.011336	0.011228	0.011233	0.011363	0.011329
0.011396	0.011330	0.011329	0.011429	0.011362	0.011732	0.011530
0.011564	0.011430	0.011295	0.011396	0.011195	0.011429	0.011228
0.011295	0.011295	0.000000	0.011228	0.011262	0.011228	0.011329
0.011295	0.011396	0.011329	0.011228	0.011330	0.011430	0.011432
0.011430	0.011329	0.011228	0.011161	0.011295	0.011228	0.011161
0.011264	0.011430	0.011228	0.011430	0.011195	0.011295	0.011262
0.011362	0.011262	0.011295	0.011262	0.011295	0.011161	0.011295
0.011329	0.011262	0.011329	0.011262	0.011295	0.011329	0.011564
0.011665	0.011597	0.011665	0.011397	0.011665	0.011295	0.011262
0.011297	0.011505	0.011380	0.011163	0.011671	0.011163	0.011257
0.011671	0.011570	0.011463	0.011665	0.011368	0.011530	0.011497
0.011429	0.011597	0.011451	0.011266	0.011312	0.011329	0.011399
0.011497	0.011398	0.011534	0.011602	0.011530	0.011598	0.011704
0.011433	0.011603	0.011534	0.011466	0.011733	0.011195	0.011397
0.011362	0.011295	0.011195	0.011195	0.011262	0.011262	0.011329
0.011262	0.011329	0.011228	0.011329	0.011262	0.011262	0.011329
0.011262	0.011698	0.011597	0.011631	0.011161	0.011262	0.011295
0.011295	0.011262	0.011329	0.011362	0.011228	0.011228	0.011228
0.011765	0.011396	0.011396	0.011195	0.011295	0.011463	0.011228
0.011228	0.011295	0.011262	0.011430	0.011228	0.011362	0.011497
0.011429	0.011262	0.011262	0.011195	0.011195	0.011362	0.011295
0.011195	0.011295	0.011161	0.011195	0.011329	0.011463	0.011228
0.011195	0.011329	0.011262	0.011161	0.011497	0.011161	0.011329
0.011403	0.011228	0.011530	0.011362	0.011396	0.011262	0.011362
0.011228	0.011564	0.011161	0.011430	0.011228	0.011228	0.011161
0.011396	0.011564	0.011295	0.011362	0.011195	0.011597	0.011228
0.011228	0.011228	0.011262	0.011362	0.011262	0.011362	0.011429
0.011430	0.011396	0.011295	0.011396	0.011362	0.011228	0.011195
0.011228	0.011228	0.011295	0.011228	0.011195	0.011362	0.011228
0.011262	0.011262	0.011396	0.011429	0.011329	0.011262	0.011262
0.011329	0.011262	0.011362	0.011228	0.011362	0.011463	0.011765
0.011262	0.011665	0.011233	0.017747	0.011191	0.011305	0.011583
0.011545	0.011588	0.011217	0.011234	0.011609	0.011612	0.011242
0.011487	0.011426	0.011138	0.011144	0.011216	0.011208	0.011189
0.011482	0.011395	0.011394	0.011259	0.011396	0.011430	0.011432
0.011301	0.011396	0.011396	0.011362	0.011329	0.011329	0.011295
0.011329	0.011262	0.011295	0.011329	0.011295	0.011228	0.011262
0.011262	0.011329	0.011228	0.011329	0.011362	0.011396	0.011295
0.011329	0.011295	0.011631	0.011631	0.011362	0.011396	0.011430
0.011195	0.011195	0.011228	0.011329	0.011195	0.011429	0.011429
0.011463	0.011463	0.011396	0.011497	0.011665	0.011498	0.011329
0.011329	0.011396	0.011429	0.011262	0.011329	0.011295	0.011329
0.011396	0.011396	0.011429	0.011396	0.011296	0.011497	0.011463

0.011331	0.011228	0.011329	0.011430	0.011329	0.011463	0.011497
0.011665	0.011368	0.011530	0.011497	0.011429	0.011597	0.011228
0.011397	0.011295	0.011262	0.011262	0.011329	0.011228	0.011329
0.011362	0.011396	0.011295	0.011329	0.011295	0.011631	0.011631
0.011362	0.011396	0.011463	0.011396	0.012036	0.011417	0.011342
0.011412	0.011407	0.011482	0.011372	0.011515	0.011440	0.011405
0.011988	0.011303	0.011437	0.011372	0.011646	0.011502	0.011605
0.012012	0.011437	0.011365	0.011329	0.011362	0.011362	0.011331
0.011195	0.011228	0.011362	0.011329	0.011331	0.011463	0.011431
0.011295	0.011228	0.011262	0.011295	0.011195	0.011262	0.011161
0.011295	0.011329	0.011228	0.011195	0.011295	0.011228	0.011262
0.011262	0.011195	0.011262	0.011295	0.011195	0.011396	0.011429
0.011329	0.011396	0.011329	0.011329	0.011262	0.011665	0.011295
0.011304	0.011262	0.011497	0.011501	0.011362	0.011362	0.011362
0.011362	0.011295	0.011262	0.011295	0.011396	0.011396	0.011329
0.011329	0.011463	0.011631	0.011362	0.011564	0.011564	0.011429
0.011262	0.011329	0.011362	0.011765	0.011329	0.011362	0.011329
0.011362	0.011295	0.011295	0.011598	0.011530	0.011564	0.011295
0.011396	0.011463	0.011329	0.011396	0.011362	0.011228	0.011564
0.011329	0.011295	0.011295	0.011463	0.011295	0.011396	0.011631
0.011329	0.011705	0.011698	0.011467	0.011173	0.011400	0.011215
0.011115	0.011683	0.011201	0.011417	0.011207	0.011366	0.011379
0.011417	0.011126	0.011339	0.011126	0.011168	0.011261	0.011344
0.011362	0.011257	0.011342	0.011272	0.011330	0.011463	0.011530
0.011530	0.011396	0.011500	0.011467	0.011636	0.011661	0.011329
0.011564	0.011430	0.011295	0.011396	0.011195	0.011429	0.011228
0.011295	0.011295	0.000000	0.011228	0.011262	0.011228	0.011329
0.011305	0.011305	0.011300	0.011334	0.011471	0.011436	0.011436
0.011435	0.011363	0.011171	0.011168	0.011297	0.011467	0.011467
0.011262	0.011331	0.011565	0.011533	0.011464	0.011498	0.011429
0.011362	0.011429	0.011429	0.011329	0.011900	0.011497	0.011564
0.011597	0.011666	0.011299	0.011372	0.011262	0.011463	0.011430
0.011464	0.011497	0.011497	0.011497	0.011650	0.011631	0.011268
0.011162	0.011164	0.011265	0.011230	0.011334	0.011135	0.011502
0.011300	0.011367	0.011156	0.011285	0.011296	0.011368	0.011262
0.011228	0.011228	0.011295	0.011262	0.011463	0.011429	0.011362
0.011463	0.011530	0.011262	0.011262	0.011295	0.011295	0.011297
0.011364	0.011295	0.011295	0.011330	0.011463	0.011530	0.011530
0.011396	0.011500	0.011467	0.011636	0.011661	0.011329	0.011497
0.011430	0.011430	0.011263	0.011443	0.011338	0.011401	0.011200
0.011439	0.011607	0.011441	0.011332	0.011228	0.011430	0.011295
0.011396	0.011329	0.011228	0.011330	0.011430	0.011432	0.011329
0.011228	0.011161	0.011295	0.011228	0.011161	0.011264	0.011430
0.011329	0.011262	0.011295	0.011329	0.011295	0.011228	0.011430
0.011228	0.011329	0.011329	0.011262	0.011329	0.011564	0.011228
0.011262	0.011665	0.011262	0.011295	0.011262	0.011631	0.011189
0.011622	0.011188	0.011211	0.011422	0.011508	0.011419	0.011144
0.011218	0.011429	0.011530	0.011603	0.011498	0.011601	0.011766
0.011666	0.011703	0.011665	0.011880	0.011631	0.012506	0.011666
0.012405	0.012173	0.012307	0.012578	0.011631	0.011329	0.011631
0.012070	0.011971	0.011597	0.011497	0.011564	0.011564	0.011534
0.011429	0.011399	0.011497	0.011464	0.011833	0.011900	0.011901
0.011800	0.011901	0.012004	0.011737	0.012070	0.012076	0.012137
0.012138	0.012172	0.012084	0.012138	0.012139	0.011362	0.011665
0.011733	0.012236	0.011738	0.011967	0.012440	0.011502	0.011396
0.011362	0.011362	0.011833	0.011842	0.011975	0.011698	0.011363
S178	0.011597	0.011597	0.011933	0.011698	0.011698	0.011765
0.011603	0.011536	0.011765	0.011195	0.011262	0.011228	0.011194
0.011228	0.011396	0.011194	0.011228	0.011295	0.011262	0.011228
0.011194	0.011228	0.011329	0.011195	0.011228	0.011228	0.011569

0.011463	0.011228	0.011127	0.011362	0.011295	0.011262	0.011295
0.011262	0.011362	0.011262	0.011262	0.011295	0.011262	0.011429
0.011194	0.011262	0.011295	0.011228	0.011329	0.011362	0.011295
0.011262	0.011429	0.011329	0.011597	0.011262	0.011530	0.011262
0.011228	0.011329	0.011396	0.011530	0.011631	0.011329	0.011429
0.011295	0.011362	0.011262	0.011496	0.011732	0.011262	0.011329
0.011228	0.011329	0.011295	0.011362	0.011295	0.011295	0.011295
0.011194	0.011228	0.011195	0.011295	0.011262	0.011296	0.011362
0.011295	0.011262	0.011329	0.011228	0.011362	0.011195	0.011262
0.011429	0.011362	0.011597	0.011429	0.011262	0.011497	0.011262
0.011431	0.011658	0.011475	0.011177	0.011172	0.011281	0.011375
0.012011	0.011467	0.011184	0.011496	0.011496	0.011295	0.011396
0.011329	0.011295	0.011362	0.011262	0.011362	0.011295	0.011295
0.011262	0.011362	0.011564	0.011429	0.011329	0.011295	0.011228
0.011195	0.011195	0.011262	0.011228	0.011429	0.011396	0.011329
0.011429	0.011497	0.011228	0.011228	0.011262	0.011262	0.011263
0.011331	0.011262	0.011262	0.011132	0.011205	0.011165	0.011262
0.011263	0.011198	0.011245	0.011233	0.011162	0.011197	0.011229
0.011492	0.011267	0.011301	0.011195	0.011199	0.011329	0.011296
0.011363	0.011296	0.011296	0.011396	0.011329	0.011698	0.011497
0.011530	0.011396	0.011262	0.011362	0.011094	0.011396	0.011195
0.011262	0.011262	0.011228	0.000000	0.011228	0.011127	0.011295
0.011262	0.011362	0.011295	0.011195	0.011296	0.011396	0.011398
0.011396	0.011295	0.011195	0.011128	0.011262	0.011195	0.011127
0.011230	0.011396	0.011195	0.011396	0.011161	0.011262	0.011228
0.011329	0.011228	0.011262	0.011228	0.011262	0.011127	0.011262
0.011295	0.011228	0.011295	0.011228	0.011262	0.011295	0.011530
0.011631	0.011564	0.011631	0.011397	0.011631	0.011262	0.011228
0.011263	0.011545	0.011380	0.011163	0.011637	0.011129	0.011257
0.011637	0.011536	0.011429	0.011631	0.011333	0.011497	0.011463
0.011396	0.011564	0.011415	0.011266	0.011312	0.011295	0.011365
0.011463	0.011364	0.011500	0.011568	0.011497	0.011564	0.011670
0.011399	0.011569	0.011500	0.011432	0.011699	0.011161	0.011363
0.011329	0.011262	0.011161	0.011161	0.011228	0.011228	0.011295
0.011228	0.011295	0.011195	0.011295	0.011228	0.011228	0.011295
0.011228	0.011664	0.011564	0.011597	0.011127	0.011228	0.011262
0.011262	0.011228	0.011295	0.011329	0.011194	0.011194	0.011194
0.011732	0.011362	0.011362	0.011161	0.011262	0.011429	0.011195
0.011194	0.011262	0.011228	0.011396	0.011194	0.011329	0.011463
0.011396	0.011228	0.011228	0.011161	0.011161	0.011329	0.011262
0.011161	0.011262	0.011127	0.011161	0.011295	0.011430	0.011195
0.011161	0.011295	0.011228	0.011127	0.011463	0.011127	0.011295
0.011368	0.011195	0.011497	0.011329	0.011362	0.011228	0.011329
0.011194	0.011530	0.011127	0.011396	0.011195	0.011195	0.011127
0.011362	0.011564	0.011262	0.011329	0.011161	0.011564	0.011195
0.011195	0.011194	0.011228	0.011329	0.011228	0.011329	0.011396
0.011396	0.011362	0.011262	0.011362	0.011329	0.011195	0.011161
0.011194	0.011195	0.011262	0.011194	0.011161	0.011329	0.011194
0.011228	0.011228	0.011362	0.011396	0.011295	0.011228	0.011228
0.011295	0.011228	0.011329	0.011194	0.011329	0.011429	0.011732
0.011228	0.011631	0.011233	0.017747	0.011257	0.011427	0.011583
0.011545	0.011588	0.011322	0.011292	0.011719	0.011612	0.011364
0.011487	0.011549	0.011138	0.011228	0.011216	0.011307	0.011232
0.011482	0.011395	0.011394	0.011193	0.011362	0.011396	0.011398
0.011266	0.011362	0.011362	0.011329	0.011295	0.011295	0.011262
0.011295	0.011228	0.011262	0.011295	0.011262	0.011195	0.011228
0.011228	0.011295	0.011195	0.011295	0.011329	0.011362	0.011262
0.011295	0.011262	0.011597	0.011597	0.011329	0.011363	0.011396
0.011161	0.011161	0.011195	0.011295	0.011161	0.011396	0.011396
0.011429	0.011429	0.011362	0.011463	0.011631	0.011464	0.011295

0.011295	0.011362	0.011396	0.011228	0.011295	0.011262	0.011295
0.011363	0.011362	0.011396	0.011363	0.011262	0.011463	0.011429
0.011297	0.011195	0.011295	0.011396	0.011295	0.011430	0.011463
0.011631	0.011333	0.011497	0.011463	0.011396	0.011564	0.011195
0.011363	0.011262	0.011228	0.011228	0.011295	0.011195	0.011295
0.011329	0.011362	0.011262	0.011295	0.011262	0.011597	0.011597
0.011329	0.011363	0.011429	0.011362	0.012002	0.011417	0.011342
0.011412	0.011372	0.011482	0.011372	0.011480	0.011405	0.011370
0.011953	0.011268	0.011403	0.011337	0.011611	0.011468	0.011571
0.011978	0.011437	0.011398	0.011463	0.011329	0.011329	0.011364
0.011161	0.011195	0.011329	0.011295	0.011297	0.011429	0.011397
0.011262	0.011194	0.011228	0.011262	0.011161	0.011228	0.011127
0.011262	0.011295	0.011195	0.011161	0.011262	0.011194	0.011228
0.011228	0.011161	0.011228	0.011262	0.011161	0.011362	0.011396
0.011295	0.011362	0.011295	0.011295	0.011228	0.011631	0.011262
0.011304	0.011228	0.011463	0.011467	0.011329	0.011329	0.011329
0.011329	0.011262	0.011228	0.011262	0.011362	0.011362	0.011295
0.011295	0.011429	0.011597	0.011329	0.011530	0.011530	0.011396
0.011228	0.011295	0.011329	0.011732	0.011295	0.011329	0.011295
0.011329	0.011262	0.011262	0.011564	0.011497	0.011530	0.011194
0.011295	0.011429	0.011295	0.011295	0.011329	0.011195	0.011530
0.011295	0.011262	0.011262	0.011429	0.011194	0.011362	0.011597
0.011295	0.011671	0.011664	0.011467	0.011229	0.011366	0.011267
0.011225	0.011683	0.011201	0.011417	0.011255	0.011366	0.011379
0.011468	0.011192	0.011339	0.011192	0.011222	0.011395	0.011344
0.011362	0.011257	0.011342	0.011272	0.011296	0.011429	0.011497
0.011497	0.011363	0.011466	0.011467	0.011603	0.011625	0.011295
0.011530	0.011396	0.011262	0.011362	0.011094	0.011396	0.011195
0.011262	0.011262	0.011228	0.000000	0.011228	0.011127	0.011295
0.011270	0.011270	0.011266	0.011300	0.011436	0.011402	0.011402
0.011401	0.011329	0.011134	0.011132	0.011263	0.011433	0.011433
0.011228	0.011297	0.011531	0.011500	0.011430	0.011464	0.011396
0.011329	0.011396	0.011396	0.011295	0.011799	0.011463	0.011530
0.011564	0.011630	0.011265	0.011337	0.011228	0.011429	0.011396
0.011430	0.011463	0.011463	0.011463	0.011610	0.011597	0.011234
0.011128	0.011130	0.011230	0.011196	0.011299	0.011135	0.011468
0.011266	0.011367	0.011156	0.011285	0.011262	0.011334	0.011228
0.011195	0.011195	0.011262	0.011228	0.011429	0.011396	0.011329
0.011429	0.011497	0.011228	0.011228	0.011262	0.011262	0.011263
0.011331	0.011262	0.011262	0.011296	0.011429	0.011497	0.011497
0.011363	0.011466	0.011467	0.011603	0.011625	0.011295	0.011463
0.011396	0.011396	0.011229	0.011408	0.011338	0.011367	0.011235
0.011404	0.011607	0.011399	0.011298	0.011195	0.011396	0.011262
0.011362	0.011295	0.011195	0.011296	0.011396	0.011398	0.011295
0.011195	0.011128	0.011262	0.011195	0.011127	0.011230	0.011396
0.011295	0.011228	0.011262	0.011295	0.011262	0.011195	0.011396
0.011262	0.011295	0.011295	0.011228	0.011295	0.011463	0.011194
0.011228	0.011631	0.011228	0.011262	0.011228	0.011597	0.011189
0.011678	0.011188	0.011261	0.011422	0.011508	0.011419	0.011144
0.011218	0.011396	0.011497	0.011603	0.011464	0.011601	0.011733
0.011632	0.011669	0.011631	0.011880	0.011597	0.012472	0.011633
0.012372	0.012139	0.012273	0.012545	0.011597	0.011295	0.011598
0.012036	0.011971	0.011564	0.011463	0.011531	0.011531	0.011500
0.011396	0.011399	0.011463	0.011430	0.011800	0.011866	0.011867
0.011767	0.011867	0.011970	0.011737	0.012036	0.012042	0.012103
0.012104	0.012138	0.012084	0.012104	0.012105	0.011329	0.011631
0.011699	0.012202	0.011704	0.011934	0.012406	0.011468	0.011363
0.011329	0.011329	0.011799	0.011842	0.011940	0.011664	0.011330
S179	0.011631	0.011631	0.011967	0.011731	0.011731	0.011799
0.011637	0.011569	0.011799	0.011228	0.011295	0.011262	0.011228

0.011262	0.011429	0.011228	0.011262	0.011329	0.011295	0.011262
0.011228	0.011262	0.011362	0.011228	0.011262	0.011262	0.011603
0.011496	0.011262	0.011161	0.011396	0.011329	0.011295	0.011329
0.011295	0.011396	0.011295	0.011295	0.011329	0.011295	0.011463
0.011228	0.011295	0.011329	0.011262	0.011362	0.011396	0.011329
0.011295	0.011463	0.011362	0.011631	0.011295	0.011564	0.011295
0.011262	0.011362	0.011429	0.011564	0.011664	0.011362	0.011463
0.011329	0.011396	0.011295	0.011530	0.011765	0.011295	0.011362
0.011262	0.011362	0.011329	0.011396	0.011329	0.011329	0.011329
0.011228	0.011262	0.011228	0.011329	0.011295	0.011329	0.011396
0.011329	0.011295	0.011362	0.011262	0.011396	0.011228	0.011295
0.011463	0.011396	0.011631	0.011463	0.011295	0.011530	0.011295
0.011431	0.011658	0.011475	0.011177	0.011172	0.011281	0.011375
0.012011	0.011467	0.011184	0.011530	0.011530	0.011329	0.011429
0.011362	0.011329	0.011396	0.011295	0.011396	0.011329	0.011329
0.011295	0.011396	0.011597	0.011463	0.011362	0.011329	0.011262
0.011228	0.011228	0.011295	0.011262	0.011463	0.011429	0.011362
0.011463	0.011530	0.011262	0.011262	0.011295	0.011295	0.011297
0.011364	0.011295	0.011295	0.011132	0.011205	0.011200	0.011296
0.011297	0.011198	0.011245	0.011267	0.011196	0.011231	0.011263
0.011528	0.011301	0.011336	0.011228	0.011233	0.011363	0.011329
0.011396	0.011330	0.011329	0.011429	0.011362	0.011732	0.011530
0.011564	0.011430	0.011295	0.011396	0.011195	0.011429	0.011228
0.011295	0.011295	0.011262	0.011228	0.000000	0.011228	0.011329
0.011295	0.011396	0.011329	0.011228	0.011330	0.011430	0.011432
0.011430	0.011329	0.011228	0.011161	0.011295	0.011228	0.011161
0.011264	0.011430	0.011228	0.011430	0.011195	0.011295	0.011262
0.011362	0.011262	0.011295	0.011262	0.011295	0.011161	0.011295
0.011329	0.011262	0.011329	0.011262	0.011295	0.011329	0.011564
0.011664	0.011597	0.011665	0.011431	0.011665	0.011295	0.011262
0.011297	0.011586	0.011426	0.011197	0.011671	0.011163	0.011296
0.011671	0.011570	0.011463	0.011664	0.011367	0.011530	0.011497
0.011429	0.011597	0.011451	0.011307	0.011354	0.011329	0.011398
0.011497	0.011397	0.011533	0.011601	0.011530	0.011597	0.011704
0.011432	0.011603	0.011534	0.011466	0.011733	0.011194	0.011397
0.011362	0.011295	0.011194	0.011194	0.011262	0.011262	0.011329
0.011262	0.011329	0.011228	0.011329	0.011262	0.011262	0.011329
0.011262	0.011698	0.011597	0.011631	0.011161	0.011262	0.011295
0.011295	0.011262	0.011329	0.011362	0.011228	0.011228	0.011228
0.011765	0.011396	0.011396	0.011194	0.011295	0.011463	0.011161
0.011228	0.011295	0.011262	0.011429	0.011228	0.011362	0.011496
0.011429	0.011262	0.011262	0.011194	0.011194	0.011362	0.011295
0.011194	0.011295	0.011161	0.011194	0.011329	0.011463	0.011228
0.011195	0.011329	0.011262	0.011161	0.011496	0.011161	0.011329
0.011402	0.011228	0.011530	0.011362	0.011396	0.011262	0.011362
0.011228	0.011564	0.011161	0.011429	0.011228	0.011228	0.011161
0.011396	0.011597	0.011295	0.011362	0.011194	0.011597	0.011228
0.011228	0.011228	0.011262	0.011362	0.011262	0.011362	0.011429
0.011429	0.011396	0.011295	0.011396	0.011362	0.011228	0.011194
0.011228	0.011228	0.011295	0.011228	0.011194	0.011362	0.011228
0.011262	0.011262	0.011396	0.011429	0.011329	0.011262	0.011262
0.011329	0.011262	0.011362	0.011228	0.011362	0.011463	0.011765
0.011262	0.011664	0.011233	0.017747	0.011257	0.011427	0.011583
0.011579	0.011588	0.011322	0.011292	0.011719	0.011647	0.011364
0.011487	0.011548	0.011138	0.011228	0.011216	0.011307	0.011232
0.011482	0.011395	0.011394	0.011193	0.011396	0.011429	0.011432
0.011301	0.011396	0.011396	0.011362	0.011329	0.011329	0.011295
0.011329	0.011262	0.011295	0.011329	0.011295	0.011228	0.011262
0.011262	0.011329	0.011228	0.011329	0.011362	0.011396	0.011295
0.011329	0.011295	0.011631	0.011631	0.011362	0.011396	0.011430

0.011195	0.011195	0.011228	0.011329	0.011195	0.011429	0.011429
0.011463	0.011463	0.011396	0.011497	0.011665	0.011498	0.011329
0.011329	0.011396	0.011429	0.011262	0.011329	0.011295	0.011329
0.011396	0.011396	0.011429	0.011396	0.011296	0.011497	0.011463
0.011331	0.011228	0.011329	0.011430	0.011329	0.011463	0.011496
0.011664	0.011367	0.011530	0.011497	0.011429	0.011597	0.011228
0.011397	0.011295	0.011262	0.011262	0.011329	0.011228	0.011329
0.011362	0.011396	0.011295	0.011329	0.011295	0.011631	0.011631
0.011362	0.011396	0.011463	0.011396	0.012035	0.011453	0.011377
0.011447	0.011407	0.011517	0.011406	0.011515	0.011439	0.011405
0.011987	0.011302	0.011437	0.011371	0.011645	0.011502	0.011604
0.012012	0.011475	0.011567	0.011631	0.011496	0.011496	0.011533
0.011194	0.011362	0.011496	0.011463	0.011466	0.011463	0.011431
0.011295	0.011228	0.011262	0.011295	0.011194	0.011262	0.011161
0.011295	0.011329	0.011228	0.011194	0.011295	0.011228	0.011262
0.011262	0.011194	0.011262	0.011295	0.011194	0.011396	0.011429
0.011329	0.011396	0.011329	0.011329	0.011262	0.011664	0.011295
0.011338	0.011262	0.011497	0.011501	0.011362	0.011362	0.011362
0.011362	0.011295	0.011262	0.011295	0.011396	0.011396	0.011329
0.011329	0.011463	0.011631	0.011362	0.011564	0.011564	0.011429
0.011262	0.011329	0.011362	0.011765	0.011329	0.011362	0.011329
0.011362	0.011295	0.011295	0.011597	0.011530	0.011564	0.011295
0.011396	0.011463	0.011329	0.011396	0.011362	0.011228	0.011564
0.011329	0.011228	0.011295	0.011463	0.011295	0.011396	0.011631
0.011329	0.011705	0.011698	0.011467	0.011229	0.011400	0.011267
0.011225	0.011683	0.011201	0.011417	0.011255	0.011366	0.011379
0.011468	0.011192	0.011338	0.011192	0.011222	0.011395	0.011344
0.011362	0.011257	0.011342	0.011272	0.011330	0.011463	0.011530
0.011530	0.011396	0.011500	0.011501	0.011636	0.011661	0.011329
0.011564	0.011430	0.011295	0.011396	0.011195	0.011429	0.011228
0.011295	0.011295	0.011262	0.011228	0.000000	0.011228	0.011329
0.011305	0.011305	0.011300	0.011334	0.011471	0.011436	0.011436
0.011435	0.011363	0.011171	0.011168	0.011297	0.011467	0.011467
0.011262	0.011331	0.011565	0.011533	0.011464	0.011498	0.011429
0.011362	0.011429	0.011429	0.011329	0.011900	0.011496	0.011564
0.011597	0.011665	0.011299	0.011372	0.011262	0.011463	0.011429
0.011464	0.011496	0.011496	0.011496	0.011650	0.011631	0.011268
0.011162	0.011164	0.011264	0.011230	0.011333	0.011172	0.011502
0.011300	0.011455	0.011203	0.011322	0.011296	0.011368	0.011262
0.011228	0.011228	0.011295	0.011262	0.011463	0.011429	0.011362
0.011463	0.011530	0.011262	0.011262	0.011295	0.011295	0.011297
0.011364	0.011295	0.011295	0.011330	0.011463	0.011530	0.011530
0.011396	0.011500	0.011501	0.011636	0.011661	0.011329	0.011496
0.011429	0.011429	0.011263	0.011443	0.011373	0.011401	0.011270
0.011439	0.011652	0.011399	0.011332	0.011228	0.011430	0.011295
0.011396	0.011329	0.011228	0.011330	0.011430	0.011432	0.011329
0.011228	0.011161	0.011295	0.011228	0.011161	0.011264	0.011430
0.011329	0.011262	0.011295	0.011329	0.011295	0.011228	0.011430
0.011295	0.011329	0.011329	0.011262	0.011329	0.011564	0.011228
0.011262	0.011664	0.011262	0.011295	0.011262	0.011631	0.011189
0.011678	0.011188	0.011261	0.011422	0.011508	0.011419	0.011144
0.011218	0.011429	0.011530	0.011637	0.011498	0.011635	0.011766
0.011666	0.011703	0.011664	0.011914	0.011631	0.012438	0.011666
0.012405	0.012105	0.012240	0.012578	0.011631	0.011329	0.011631
0.012070	0.012004	0.011597	0.011497	0.011564	0.011564	0.011534
0.011429	0.011433	0.011496	0.011464	0.011833	0.011900	0.011901
0.011733	0.011901	0.011970	0.011770	0.012070	0.012076	0.012137
0.012138	0.012172	0.012118	0.012138	0.012139	0.011362	0.011665
0.011733	0.012236	0.011738	0.011967	0.012440	0.011502	0.011396
0.011362	0.011362	0.011833	0.011883	0.011939	0.011698	0.011363

S180	0.011597	0.011597	0.011934	0.011698	0.011698	0.011765
0.011604	0.011536	0.011698	0.011195	0.011262	0.011228	0.011195
0.011228	0.011396	0.011195	0.011228	0.011295	0.011262	0.011228
0.011195	0.011228	0.011329	0.011195	0.011228	0.011228	0.011570
0.011463	0.011228	0.011127	0.011362	0.011295	0.011262	0.011295
0.011262	0.011362	0.011262	0.011262	0.011295	0.011262	0.011430
0.011195	0.011262	0.011295	0.011228	0.011329	0.011362	0.011295
0.011262	0.011430	0.011329	0.011597	0.011262	0.011530	0.011262
0.011228	0.011329	0.011396	0.011530	0.011631	0.011329	0.011429
0.011295	0.011362	0.011262	0.011497	0.011732	0.011262	0.011329
0.011228	0.011329	0.011295	0.011362	0.011295	0.011295	0.011295
0.011195	0.011228	0.011195	0.011295	0.011262	0.011296	0.011362
0.011295	0.011262	0.011329	0.011228	0.011362	0.011195	0.011262
0.011429	0.011362	0.011597	0.011430	0.011262	0.011497	0.011262
0.011431	0.011658	0.011475	0.011177	0.011172	0.011281	0.011375
0.012011	0.011467	0.011184	0.011497	0.011497	0.011295	0.011396
0.011329	0.011295	0.011362	0.011262	0.011362	0.011295	0.011295
0.011262	0.011362	0.011564	0.011429	0.011329	0.011295	0.011228
0.011195	0.011195	0.011262	0.011228	0.011429	0.011396	0.011329
0.011429	0.011497	0.011228	0.011228	0.011262	0.011262	0.011263
0.011331	0.011262	0.011262	0.011132	0.011205	0.011165	0.011262
0.011263	0.011198	0.011245	0.011233	0.011162	0.011197	0.011229
0.011492	0.011267	0.011301	0.011195	0.011199	0.011329	0.011296
0.011363	0.011296	0.011296	0.011396	0.011329	0.011698	0.011497
0.011530	0.011396	0.011262	0.011362	0.011094	0.011396	0.011195
0.011262	0.011262	0.011228	0.011127	0.011228	0.000000	0.011295
0.011262	0.011362	0.011295	0.011195	0.011296	0.011396	0.011398
0.011396	0.011295	0.011195	0.011128	0.011262	0.011195	0.011127
0.011230	0.011396	0.011195	0.011396	0.011161	0.011262	0.011228
0.011329	0.011228	0.011262	0.011228	0.011262	0.011127	0.011262
0.011295	0.011228	0.011295	0.011228	0.011262	0.011295	0.011530
0.011631	0.011564	0.011631	0.011397	0.011631	0.011262	0.011228
0.011263	0.011545	0.011380	0.011163	0.011637	0.011129	0.011257
0.011637	0.011536	0.011429	0.011631	0.011333	0.011497	0.011463
0.011396	0.011564	0.011416	0.011266	0.011312	0.011295	0.011365
0.011463	0.011364	0.011500	0.011568	0.011497	0.011564	0.011670
0.011399	0.011569	0.011500	0.011432	0.011700	0.011161	0.011364
0.011329	0.011262	0.011161	0.011161	0.011228	0.011228	0.011295
0.011228	0.011295	0.011195	0.011295	0.011228	0.011228	0.011295
0.011228	0.011665	0.011564	0.011597	0.011127	0.011228	0.011262
0.011262	0.011228	0.011295	0.011329	0.011195	0.011195	0.011195
0.011665	0.011362	0.011362	0.011161	0.011262	0.011430	0.011195
0.011195	0.011262	0.011228	0.011396	0.011195	0.011329	0.011463
0.011396	0.011228	0.011228	0.011161	0.011161	0.011329	0.011262
0.011161	0.011262	0.011127	0.011161	0.011295	0.011430	0.011195
0.011161	0.011295	0.011228	0.011127	0.011463	0.011127	0.011295
0.011368	0.011195	0.011497	0.011329	0.011362	0.011228	0.011329
0.011195	0.011530	0.011127	0.011396	0.011195	0.011195	0.011127
0.011362	0.011564	0.011262	0.011329	0.011161	0.011564	0.011195
0.011195	0.011195	0.011228	0.011329	0.011228	0.011329	0.011396
0.011396	0.011362	0.011262	0.011362	0.011329	0.011195	0.011161
0.011195	0.011195	0.011262	0.011195	0.011161	0.011329	0.011195
0.011228	0.011228	0.011362	0.011396	0.011295	0.011228	0.011228
0.011295	0.011228	0.011329	0.011195	0.011329	0.011429	0.011732
0.011228	0.011631	0.011233	0.017747	0.011257	0.011427	0.011583
0.011545	0.011588	0.011322	0.011292	0.011719	0.011612	0.011364
0.011487	0.011549	0.011138	0.011228	0.011216	0.011307	0.011232
0.011482	0.011395	0.011394	0.011126	0.011362	0.011396	0.011398
0.011266	0.011362	0.011362	0.011329	0.011295	0.011295	0.011262
0.011295	0.011228	0.011262	0.011295	0.011262	0.011195	0.011228

0.011228	0.011295	0.011195	0.011295	0.011329	0.011362	0.011262
0.011295	0.011262	0.011597	0.011597	0.011329	0.011363	0.011396
0.011161	0.011161	0.011195	0.011295	0.011161	0.011396	0.011396
0.011429	0.011429	0.011362	0.011463	0.011631	0.011464	0.011295
0.011295	0.011362	0.011396	0.011228	0.011295	0.011262	0.011295
0.011363	0.011362	0.011396	0.011363	0.011262	0.011463	0.011429
0.011297	0.011195	0.011295	0.011396	0.011295	0.011430	0.011463
0.011631	0.011333	0.011497	0.011463	0.011396	0.011564	0.011195
0.011363	0.011262	0.011228	0.011228	0.011295	0.011195	0.011295
0.011329	0.011362	0.011262	0.011295	0.011262	0.011597	0.011597
0.011329	0.011363	0.011429	0.011362	0.012002	0.011417	0.011342
0.011412	0.011372	0.011482	0.011372	0.011480	0.011405	0.011371
0.011953	0.011268	0.011403	0.011337	0.011611	0.011468	0.011571
0.011978	0.011437	0.011297	0.011295	0.011295	0.011329	0.011263
0.011161	0.011195	0.011329	0.011295	0.011297	0.011429	0.011397
0.011262	0.011195	0.011228	0.011262	0.011161	0.011228	0.011127
0.011262	0.011295	0.011195	0.011161	0.011262	0.011195	0.011228
0.011228	0.011161	0.011228	0.011262	0.011161	0.011362	0.011396
0.011295	0.011362	0.011295	0.011295	0.011228	0.011631	0.011262
0.011304	0.011228	0.011463	0.011467	0.011329	0.011329	0.011329
0.011329	0.011262	0.011228	0.011262	0.011362	0.011362	0.011295
0.011295	0.011430	0.011597	0.011329	0.011530	0.011530	0.011396
0.011228	0.011295	0.011329	0.011732	0.011295	0.011329	0.011295
0.011329	0.011262	0.011262	0.011564	0.011497	0.011530	0.011262
0.011362	0.011430	0.011295	0.011362	0.011329	0.011195	0.011530
0.011295	0.011262	0.011262	0.011430	0.011262	0.011362	0.011597
0.011295	0.011672	0.011665	0.011467	0.011229	0.011366	0.011267
0.011225	0.011683	0.011201	0.011417	0.011255	0.011366	0.011379
0.011468	0.011192	0.011339	0.011192	0.011222	0.011395	0.011344
0.011362	0.011257	0.011342	0.011272	0.011296	0.011429	0.011497
0.011497	0.011363	0.011466	0.011467	0.011603	0.011625	0.011295
0.011530	0.011396	0.011262	0.011362	0.011094	0.011396	0.011195
0.011262	0.011262	0.011228	0.011127	0.011228	0.000000	0.011295
0.011270	0.011270	0.011266	0.011300	0.011436	0.011402	0.011402
0.011401	0.011329	0.011134	0.011132	0.011263	0.011433	0.011433
0.011228	0.011297	0.011531	0.011500	0.011430	0.011464	0.011396
0.011329	0.011396	0.011396	0.011295	0.011866	0.011463	0.011530
0.011564	0.011630	0.011265	0.011337	0.011228	0.011430	0.011396
0.011430	0.011463	0.011463	0.011463	0.011610	0.011597	0.011234
0.011128	0.011130	0.011231	0.011196	0.011299	0.011135	0.011468
0.011266	0.011367	0.011156	0.011285	0.011262	0.011334	0.011228
0.011195	0.011195	0.011262	0.011228	0.011429	0.011396	0.011329
0.011429	0.011497	0.011228	0.011228	0.011262	0.011262	0.011263
0.011331	0.011262	0.011262	0.011296	0.011429	0.011497	0.011497
0.011363	0.011466	0.011467	0.011603	0.011625	0.011295	0.011463
0.011396	0.011396	0.011229	0.011408	0.011338	0.011367	0.011235
0.011404	0.011607	0.011399	0.011298	0.011195	0.011396	0.011262
0.011362	0.011295	0.011195	0.011296	0.011396	0.011398	0.011295
0.011195	0.011128	0.011262	0.011195	0.011127	0.011230	0.011396
0.011295	0.011228	0.011262	0.011295	0.011262	0.011195	0.011396
0.011262	0.011295	0.011295	0.011228	0.011295	0.011530	0.011195
0.011228	0.011631	0.011228	0.011262	0.011228	0.011597	0.011189
0.011622	0.011188	0.011261	0.011422	0.011508	0.011419	0.011144
0.011218	0.011396	0.011497	0.011603	0.011464	0.011601	0.011733
0.011632	0.011669	0.011631	0.011880	0.011597	0.012472	0.011633
0.012372	0.012139	0.012273	0.012545	0.011597	0.011295	0.011598
0.012036	0.011971	0.011564	0.011463	0.011531	0.011531	0.011500
0.011396	0.011399	0.011463	0.011430	0.011800	0.011866	0.011867
0.011767	0.011867	0.011970	0.011737	0.012036	0.012042	0.012103
0.012104	0.012138	0.012084	0.012104	0.012105	0.011329	0.011631

0.011699	0.012203	0.011704	0.011934	0.012406	0.011468	0.011363
0.011329	0.011329	0.011799	0.011842	0.011940	0.011665	0.011330
S181	0.011698	0.011698	0.012035	0.011799	0.011799	0.011866
0.011706	0.011638	0.011799	0.011295	0.011362	0.011329	0.011295
0.011329	0.011497	0.011295	0.011329	0.011396	0.011362	0.011329
0.011295	0.011329	0.011429	0.011295	0.011329	0.011329	0.011672
0.011564	0.011329	0.011228	0.011463	0.011396	0.011362	0.011396
0.011362	0.011463	0.011362	0.011362	0.011396	0.011362	0.011530
0.011295	0.011362	0.011396	0.011329	0.011429	0.011463	0.011396
0.011362	0.011530	0.011430	0.011698	0.011362	0.011631	0.011362
0.011329	0.011430	0.011497	0.011631	0.011732	0.011430	0.011530
0.011396	0.011463	0.011362	0.011597	0.011833	0.011362	0.011430
0.011329	0.011429	0.011396	0.011463	0.011396	0.011396	0.011396
0.011295	0.011329	0.011295	0.011396	0.011362	0.011397	0.011463
0.011396	0.011362	0.011429	0.011329	0.011463	0.011295	0.011362
0.011530	0.011463	0.011698	0.011530	0.011362	0.011597	0.011362
0.011542	0.011770	0.011588	0.011295	0.011284	0.011391	0.011493
0.012121	0.011569	0.011307	0.011597	0.011597	0.011396	0.011497
0.011430	0.011396	0.011463	0.011362	0.011463	0.011396	0.011396
0.011362	0.011463	0.011665	0.011530	0.011430	0.011396	0.011329
0.011295	0.011295	0.011362	0.011329	0.011530	0.011497	0.011429
0.011530	0.011597	0.011329	0.011329	0.011362	0.011362	0.011364
0.011432	0.011362	0.011094	0.011240	0.011313	0.011271	0.011363
0.011365	0.011301	0.011355	0.011336	0.011264	0.011300	0.011330
0.011600	0.011370	0.011405	0.011296	0.011302	0.011430	0.011396
0.011464	0.011397	0.011396	0.011497	0.011429	0.011799	0.011598
0.011631	0.011497	0.011362	0.011463	0.011262	0.011497	0.011295
0.011362	0.011362	0.011329	0.011295	0.011329	0.011295	0.000000
0.011362	0.011463	0.011396	0.011295	0.011397	0.011497	0.011499
0.011497	0.011396	0.011295	0.011229	0.011362	0.011295	0.011228
0.011332	0.011497	0.011295	0.011497	0.011262	0.011362	0.011329
0.011430	0.011329	0.011362	0.011329	0.011362	0.011228	0.011362
0.011396	0.011329	0.011396	0.011329	0.011362	0.011396	0.011631
0.011732	0.011665	0.011732	0.011498	0.011732	0.011362	0.011329
0.011364	0.011626	0.011472	0.011266	0.011739	0.011197	0.011296
0.011739	0.011604	0.011530	0.011732	0.011436	0.011597	0.011564
0.011497	0.011664	0.011522	0.011390	0.011438	0.011396	0.011466
0.011564	0.011465	0.011601	0.011669	0.011598	0.011665	0.011772
0.011500	0.011671	0.011602	0.011534	0.011801	0.011262	0.011465
0.011429	0.011362	0.011262	0.011262	0.011329	0.011329	0.011396
0.011329	0.011396	0.011295	0.011396	0.011329	0.011329	0.011396
0.011329	0.011765	0.011665	0.011698	0.011228	0.011329	0.011362
0.011362	0.011329	0.011396	0.011430	0.011295	0.011295	0.011295
0.011833	0.011463	0.011463	0.011262	0.011362	0.011530	0.011295
0.011295	0.011362	0.011329	0.011497	0.011295	0.011429	0.011564
0.011497	0.011329	0.011329	0.011262	0.011262	0.011430	0.011362
0.011262	0.011362	0.011228	0.011262	0.011396	0.011531	0.011295
0.011195	0.011396	0.011329	0.011228	0.011564	0.011228	0.011329
0.011471	0.011295	0.011597	0.011430	0.011463	0.011329	0.011429
0.011295	0.011631	0.011228	0.011497	0.011295	0.011295	0.011228
0.011463	0.011665	0.011362	0.011430	0.011262	0.011665	0.011295
0.011295	0.011295	0.011329	0.011430	0.011329	0.011429	0.011497
0.011497	0.011463	0.011362	0.011463	0.011429	0.011295	0.011262
0.011295	0.011295	0.011362	0.011295	0.011262	0.011430	0.011295
0.011329	0.011329	0.011463	0.011497	0.011396	0.011329	0.011329
0.011396	0.011329	0.011429	0.011295	0.011429	0.011530	0.011833
0.011329	0.011732	0.011336	0.017861	0.011388	0.011549	0.011688
0.011648	0.011693	0.011374	0.011350	0.011829	0.011716	0.011485
0.011629	0.011671	0.011255	0.011353	0.011333	0.011356	0.011361
0.011576	0.011507	0.011505	0.011391	0.011463	0.011497	0.011500

0.011369	0.011463	0.011463	0.011429	0.011396	0.011396	0.011363
0.011396	0.011329	0.011362	0.011329	0.011362	0.011295	0.011329
0.011329	0.011396	0.011295	0.011396	0.011430	0.011463	0.011362
0.011396	0.011362	0.011698	0.011698	0.011430	0.011464	0.011497
0.011262	0.011262	0.011295	0.011396	0.011262	0.011497	0.011497
0.011530	0.011530	0.011463	0.011564	0.011732	0.011565	0.011396
0.011396	0.011463	0.011497	0.011329	0.011396	0.011362	0.011396
0.011463	0.011463	0.011497	0.011463	0.011363	0.011564	0.011530
0.011399	0.011295	0.011396	0.011497	0.011396	0.011530	0.011564
0.011732	0.011436	0.011597	0.011564	0.011497	0.011664	0.011295
0.011464	0.011363	0.011329	0.011329	0.011396	0.011295	0.011396
0.011430	0.011463	0.011362	0.011396	0.011362	0.011698	0.011698
0.011430	0.011464	0.011530	0.011463	0.012103	0.011453	0.011447
0.011517	0.011407	0.011587	0.011476	0.011585	0.011509	0.011474
0.012056	0.011372	0.011506	0.011441	0.011715	0.011570	0.011673
0.012080	0.011550	0.011432	0.011429	0.011429	0.011429	0.011398
0.011262	0.011295	0.011429	0.011396	0.011398	0.011530	0.011498
0.011362	0.011295	0.011329	0.011362	0.011262	0.011329	0.011228
0.011362	0.011396	0.011295	0.011262	0.011362	0.011295	0.011329
0.011329	0.011262	0.011329	0.011362	0.011262	0.011463	0.011497
0.011396	0.011463	0.011396	0.011396	0.011329	0.011732	0.011362
0.011408	0.011329	0.011564	0.011569	0.011430	0.011429	0.011429
0.011430	0.011362	0.011329	0.011362	0.011463	0.011463	0.011396
0.011396	0.011530	0.011698	0.011429	0.011564	0.011564	0.011497
0.011329	0.011396	0.011430	0.011833	0.011396	0.011430	0.011396
0.011429	0.011362	0.011362	0.011665	0.011598	0.011631	0.011362
0.011463	0.011530	0.011396	0.011463	0.011429	0.011295	0.011631
0.011396	0.011362	0.011362	0.011530	0.011362	0.011463	0.011698
0.011396	0.011773	0.011765	0.011589	0.011342	0.011468	0.011318
0.011335	0.011800	0.011306	0.011551	0.011402	0.011518	0.011538
0.011570	0.011389	0.011450	0.011324	0.011384	0.011395	0.011466
0.011475	0.011375	0.011448	0.011377	0.011128	0.011530	0.011597
0.011597	0.011464	0.011567	0.011569	0.011704	0.011661	0.011396
0.011631	0.011497	0.011362	0.011463	0.011262	0.011497	0.011295
0.011362	0.011362	0.011329	0.011295	0.011329	0.011295	0.000000
0.011375	0.011375	0.011368	0.011403	0.011539	0.011504	0.011504
0.011503	0.011430	0.011171	0.011204	0.011365	0.011535	0.011535
0.011329	0.011399	0.011632	0.011601	0.011531	0.011565	0.011497
0.011429	0.011497	0.011497	0.011396	0.011967	0.011564	0.011631
0.011665	0.011737	0.011367	0.011441	0.011329	0.011530	0.011497
0.011531	0.011564	0.011564	0.011564	0.011689	0.011698	0.011338
0.011229	0.011233	0.011333	0.011298	0.011402	0.011247	0.011570
0.011369	0.011455	0.011156	0.011397	0.011363	0.011437	0.011329
0.011295	0.011295	0.011362	0.011329	0.011530	0.011497	0.011429
0.011530	0.011597	0.011329	0.011329	0.011362	0.011362	0.011364
0.011432	0.011362	0.011094	0.011128	0.011530	0.011597	0.011597
0.011464	0.011567	0.011569	0.011704	0.011661	0.011396	0.011564
0.011497	0.011497	0.011331	0.011513	0.011442	0.011469	0.011305
0.011473	0.011744	0.011441	0.011400	0.011295	0.011497	0.011362
0.011463	0.011396	0.011295	0.011397	0.011497	0.011499	0.011396
0.011295	0.011229	0.011362	0.011295	0.011228	0.011332	0.011497
0.011396	0.011329	0.011362	0.011329	0.011362	0.011295	0.011497
0.011362	0.011396	0.011396	0.011329	0.011396	0.011631	0.011295
0.011329	0.011732	0.011329	0.011362	0.011329	0.011698	0.011317
0.011791	0.011315	0.011361	0.011543	0.011630	0.011527	0.011269
0.011323	0.011497	0.011597	0.011705	0.011565	0.011703	0.011834
0.011733	0.011771	0.011732	0.011983	0.011698	0.012539	0.011734
0.012473	0.012207	0.012341	0.012646	0.011698	0.011396	0.011699
0.012137	0.012072	0.011665	0.011564	0.011632	0.011632	0.011601
0.011497	0.011501	0.011564	0.011531	0.011901	0.011967	0.011968

0.011868	0.011968	0.012072	0.011838	0.012138	0.012144	0.012205
0.012205	0.012239	0.012186	0.012206	0.012207	0.011429	0.011732
0.011800	0.012304	0.011806	0.012035	0.012508	0.011570	0.011464
0.011429	0.011429	0.011900	0.011965	0.012045	0.011765	0.011431
S182	0.011665	0.011665	0.012001	0.011765	0.011765	0.011833
0.011672	0.011604	0.011765	0.011262	0.011329	0.011296	0.011262
0.011295	0.011463	0.011262	0.011295	0.011362	0.011329	0.011295
0.011262	0.011295	0.011396	0.011262	0.011228	0.011295	0.011638
0.011530	0.011295	0.011195	0.011429	0.011295	0.011329	0.011295
0.011329	0.011362	0.011329	0.011329	0.011362	0.011329	0.011497
0.011262	0.011329	0.011362	0.011295	0.011396	0.011430	0.011362
0.011329	0.011497	0.011396	0.011665	0.011329	0.011530	0.011329
0.011295	0.011396	0.011463	0.011530	0.011698	0.011396	0.011497
0.011295	0.011362	0.011329	0.011497	0.011799	0.011329	0.011396
0.011295	0.011396	0.011362	0.011430	0.011362	0.011362	0.011362
0.011262	0.011295	0.011262	0.011362	0.011329	0.011363	0.011429
0.011362	0.011329	0.011329	0.011295	0.011429	0.011195	0.011329
0.011497	0.011429	0.011665	0.011497	0.011329	0.011497	0.011329
0.011468	0.011695	0.011513	0.011216	0.011210	0.011318	0.011415
0.012048	0.011467	0.011225	0.011564	0.011564	0.011362	0.011463
0.011396	0.011362	0.011429	0.011329	0.011430	0.011295	0.011362
0.011329	0.011429	0.011631	0.011497	0.011396	0.011363	0.011228
0.011262	0.011262	0.011262	0.011295	0.011497	0.011463	0.011396
0.011497	0.011564	0.011295	0.011295	0.011329	0.011329	0.011330
0.011398	0.011329	0.011329	0.011132	0.011241	0.011236	0.011329
0.011331	0.011232	0.011282	0.011267	0.011230	0.011266	0.011297
0.011528	0.011336	0.011267	0.011262	0.011233	0.011397	0.011363
0.011430	0.011364	0.011363	0.011463	0.011396	0.011698	0.011564
0.011597	0.011463	0.011329	0.011430	0.011228	0.011463	0.011262
0.011329	0.011329	0.011295	0.011262	0.011295	0.011262	0.011362
0.000000	0.011362	0.011362	0.011262	0.011363	0.011463	0.011466
0.011396	0.011362	0.011262	0.011195	0.011329	0.011262	0.011195
0.011298	0.011396	0.011195	0.011396	0.011228	0.011329	0.011295
0.011329	0.011295	0.011262	0.011295	0.011329	0.011195	0.011262
0.011295	0.011295	0.011362	0.011295	0.011329	0.011362	0.011597
0.011698	0.011631	0.011698	0.011465	0.011698	0.011329	0.011295
0.011330	0.011586	0.011426	0.011232	0.011705	0.011197	0.011296
0.011705	0.011604	0.011497	0.011698	0.011402	0.011564	0.011463
0.011463	0.011631	0.011487	0.011307	0.011312	0.011363	0.011433
0.011530	0.011431	0.011568	0.011568	0.011564	0.011631	0.011738
0.011466	0.011569	0.011568	0.011500	0.011767	0.011228	0.011431
0.011329	0.011329	0.011228	0.011228	0.011228	0.011295	0.011362
0.011228	0.011295	0.011195	0.011362	0.011228	0.011295	0.011362
0.011295	0.011732	0.011631	0.011665	0.011195	0.011295	0.011329
0.011329	0.011295	0.011362	0.011396	0.011262	0.011262	0.011262
0.011799	0.011430	0.011430	0.011228	0.011329	0.011430	0.011262
0.011262	0.011329	0.011295	0.011396	0.011262	0.011396	0.011463
0.011463	0.011295	0.011295	0.011228	0.011228	0.011396	0.011329
0.011228	0.011329	0.011195	0.011228	0.011362	0.011497	0.011262
0.011228	0.011362	0.011296	0.011195	0.011530	0.011195	0.011362
0.011368	0.011262	0.011564	0.011396	0.011430	0.011295	0.011396
0.011262	0.011530	0.011195	0.011463	0.011262	0.011262	0.011195
0.011362	0.011631	0.011329	0.011396	0.011228	0.011631	0.011262
0.011262	0.011262	0.011295	0.011329	0.011295	0.011396	0.011463
0.011463	0.011430	0.011329	0.011430	0.011329	0.011262	0.011228
0.011262	0.011262	0.011329	0.011262	0.011161	0.011396	0.011262
0.011295	0.011295	0.011430	0.011463	0.011362	0.011228	0.011295
0.011362	0.011295	0.011396	0.011262	0.011396	0.011497	0.011799
0.011295	0.011698	0.011267	0.017785	0.011191	0.011366	0.011618
0.011579	0.011623	0.011270	0.011234	0.011664	0.011647	0.011303

0.011487	0.011549	0.011177	0.011186	0.011177	0.011307	0.011275
0.011482	0.011433	0.011394	0.011259	0.011430	0.011463	0.011466
0.011266	0.011430	0.011430	0.011396	0.011362	0.011363	0.011329
0.011362	0.011295	0.011329	0.011362	0.011329	0.011262	0.011295
0.011295	0.011362	0.011262	0.011363	0.011396	0.011430	0.011329
0.011362	0.011329	0.011665	0.011665	0.011396	0.011430	0.011463
0.011228	0.011228	0.011262	0.011295	0.011228	0.011463	0.011463
0.011497	0.011497	0.011430	0.011530	0.011698	0.011532	0.011295
0.011362	0.011430	0.011396	0.011295	0.011362	0.011329	0.011362
0.011430	0.011430	0.011463	0.011363	0.011262	0.011530	0.011429
0.011365	0.011262	0.011362	0.011463	0.011363	0.011497	0.011530
0.011698	0.011402	0.011564	0.011463	0.011463	0.011631	0.011262
0.011431	0.011329	0.011295	0.011295	0.011362	0.011262	0.011363
0.011396	0.011430	0.011329	0.011362	0.011329	0.011665	0.011665
0.011396	0.011430	0.011497	0.011429	0.012070	0.011489	0.011412
0.011482	0.011442	0.011552	0.011441	0.011550	0.011474	0.011440
0.012022	0.011337	0.011471	0.011406	0.011680	0.011537	0.011571
0.012047	0.011512	0.011365	0.011362	0.011362	0.011396	0.011331
0.011228	0.011195	0.011396	0.011362	0.011364	0.011497	0.011464
0.011329	0.011195	0.011228	0.011329	0.011161	0.011295	0.011195
0.011329	0.011362	0.011262	0.011228	0.011329	0.011262	0.011295
0.011295	0.011228	0.011295	0.011329	0.011228	0.011362	0.011463
0.011362	0.011430	0.011362	0.011362	0.011295	0.011698	0.011329
0.011373	0.011295	0.011531	0.011535	0.011329	0.011396	0.011396
0.011396	0.011262	0.011295	0.011262	0.011430	0.011430	0.011362
0.011362	0.011497	0.011665	0.011396	0.011598	0.011598	0.011463
0.011295	0.011362	0.011396	0.011799	0.011363	0.011329	0.011362
0.011396	0.011329	0.011329	0.011631	0.011497	0.011598	0.011329
0.011430	0.011497	0.011362	0.011430	0.011396	0.011262	0.011597
0.011362	0.011329	0.011329	0.011497	0.011329	0.011430	0.011665
0.011362	0.011740	0.011732	0.011507	0.011173	0.011400	0.011267
0.011225	0.011722	0.011236	0.011462	0.011255	0.011417	0.011379
0.011468	0.011126	0.011339	0.011126	0.011222	0.011328	0.011344
0.011362	0.011218	0.011377	0.011307	0.011364	0.011497	0.011564
0.011564	0.011430	0.011534	0.011535	0.011670	0.011696	0.011362
0.011597	0.011463	0.011329	0.011430	0.011228	0.011463	0.011262
0.011329	0.011329	0.011295	0.011262	0.011295	0.011262	0.011362
0.011305	0.011305	0.011300	0.011368	0.011505	0.011368	0.011470
0.011469	0.011396	0.011171	0.011168	0.011331	0.011501	0.011501
0.011296	0.011365	0.011599	0.011533	0.011497	0.011531	0.011463
0.011396	0.011463	0.011463	0.011362	0.011934	0.011530	0.011598
0.011631	0.011701	0.011333	0.011407	0.011295	0.011497	0.011463
0.011498	0.011530	0.011530	0.011530	0.011650	0.011665	0.011303
0.011196	0.011199	0.011299	0.011264	0.011368	0.011210	0.011536
0.011335	0.011367	0.011108	0.011360	0.011329	0.011402	0.011228
0.011262	0.011262	0.011262	0.011295	0.011497	0.011463	0.011396
0.011497	0.011564	0.011295	0.011295	0.011329	0.011329	0.011330
0.011398	0.011329	0.011329	0.011364	0.011497	0.011564	0.011564
0.011430	0.011534	0.011535	0.011670	0.011696	0.011362	0.011530
0.011463	0.011463	0.011297	0.011478	0.011408	0.011435	0.011305
0.011405	0.011653	0.011441	0.011366	0.011195	0.011396	0.000000
0.011362	0.011362	0.011262	0.011363	0.011463	0.011466	0.011362
0.011262	0.011195	0.011329	0.011262	0.011195	0.011298	0.011396
0.011362	0.011295	0.011329	0.011362	0.011329	0.011262	0.011463
0.011329	0.011362	0.011362	0.011295	0.011362	0.011598	0.011262
0.011295	0.011698	0.011295	0.011262	0.011295	0.011665	0.011232
0.011622	0.011230	0.011261	0.011463	0.011549	0.011455	0.011186
0.011218	0.011463	0.011564	0.011671	0.011531	0.011669	0.011800
0.011699	0.011737	0.011698	0.011949	0.011665	0.012540	0.011700
0.012439	0.012207	0.012341	0.012613	0.011665	0.011363	0.011665

0.012103	0.012038	0.011631	0.011531	0.011598	0.011598	0.011568
0.011463	0.011467	0.011530	0.011497	0.011867	0.011934	0.011935
0.011801	0.011934	0.012038	0.011804	0.012104	0.012110	0.012171
0.012172	0.012205	0.012118	0.012172	0.012173	0.011396	0.011699
0.011767	0.012270	0.011772	0.012001	0.012474	0.011536	0.011430
0.011396	0.011396	0.011867	0.011924	0.011975	0.011732	0.011397
S183	0.011766	0.011766	0.012102	0.011866	0.011866	0.011934
0.011774	0.011706	0.011866	0.011362	0.011429	0.011397	0.011362
0.011396	0.011564	0.011362	0.011396	0.011463	0.011430	0.011396
0.011362	0.011396	0.011497	0.011363	0.011329	0.011396	0.011740
0.011631	0.011396	0.011295	0.011530	0.011396	0.011430	0.011396
0.011430	0.011463	0.011430	0.011430	0.011463	0.011429	0.011597
0.011362	0.011429	0.011463	0.011396	0.011497	0.011530	0.011463
0.011430	0.011598	0.011497	0.011765	0.011429	0.011631	0.011429
0.011396	0.011497	0.011564	0.011631	0.011799	0.011497	0.011597
0.011396	0.011463	0.011430	0.011597	0.011900	0.011430	0.011497
0.011396	0.011497	0.011463	0.011530	0.011463	0.011463	0.011463
0.011362	0.011396	0.011362	0.011463	0.011429	0.011464	0.011530
0.011463	0.011429	0.011429	0.011396	0.011530	0.011295	0.011429
0.011597	0.011530	0.011765	0.011598	0.011430	0.011597	0.011429
0.011616	0.011845	0.011626	0.011334	0.011321	0.011465	0.011533
0.012195	0.011620	0.011348	0.011665	0.011665	0.011463	0.011564
0.011497	0.011463	0.011530	0.011430	0.011530	0.011396	0.011463
0.011429	0.011530	0.011732	0.011597	0.011497	0.011463	0.011329
0.011362	0.011362	0.011362	0.011396	0.011597	0.011564	0.011497
0.011597	0.011665	0.011396	0.011396	0.011430	0.011429	0.011432
0.011500	0.011429	0.011430	0.011276	0.011313	0.011306	0.011430
0.011433	0.011369	0.011392	0.011405	0.011332	0.011368	0.011398
0.011636	0.011439	0.011405	0.011363	0.011371	0.011497	0.011464
0.011531	0.011465	0.011464	0.011564	0.011497	0.011799	0.011665
0.011698	0.011564	0.011429	0.011530	0.011329	0.011564	0.011362
0.011430	0.011429	0.011396	0.011362	0.011396	0.011362	0.011463
0.011362	0.000000	0.011463	0.011362	0.011465	0.011564	0.011567
0.011497	0.011463	0.011362	0.011296	0.011429	0.011362	0.011295
0.011400	0.011497	0.011295	0.011497	0.011329	0.011430	0.011396
0.011429	0.011396	0.011362	0.011396	0.011430	0.011295	0.011362
0.011396	0.011396	0.011463	0.011396	0.011429	0.011463	0.011698
0.011799	0.011732	0.011799	0.011566	0.011799	0.011429	0.011396
0.011432	0.011667	0.011472	0.011335	0.011807	0.011299	0.011454
0.011807	0.011706	0.011597	0.011799	0.011504	0.011665	0.011564
0.011564	0.011732	0.011558	0.011431	0.011480	0.011463	0.011500
0.011631	0.011533	0.011669	0.011669	0.011665	0.011732	0.011840
0.011568	0.011671	0.011670	0.011601	0.011868	0.011329	0.011532
0.011429	0.011430	0.011329	0.011329	0.011329	0.011396	0.011463
0.011262	0.011396	0.011295	0.011463	0.011329	0.011396	0.011463
0.011396	0.011833	0.011732	0.011765	0.011295	0.011396	0.011430
0.011430	0.011396	0.011463	0.011497	0.011362	0.011362	0.011362
0.011900	0.011530	0.011530	0.011329	0.011429	0.011530	0.011362
0.011362	0.011429	0.011396	0.011497	0.011362	0.011497	0.011497
0.011564	0.011396	0.011396	0.011329	0.011329	0.011497	0.011429
0.011329	0.011429	0.011295	0.011329	0.011463	0.011598	0.011362
0.011329	0.011463	0.011396	0.011295	0.011631	0.011295	0.011463
0.011471	0.011362	0.011665	0.011497	0.011530	0.011396	0.011497
0.011362	0.011631	0.011295	0.011564	0.011362	0.011362	0.011295
0.011463	0.011732	0.011430	0.011497	0.011329	0.011732	0.011362
0.011362	0.011362	0.011396	0.011429	0.011396	0.011497	0.011564
0.011564	0.011530	0.011429	0.011530	0.011429	0.011362	0.011329
0.011362	0.011362	0.011430	0.011362	0.011262	0.011497	0.011362
0.011396	0.011396	0.011530	0.011564	0.011463	0.011329	0.011396
0.011463	0.011396	0.011497	0.011362	0.011497	0.011597	0.011900

0.011396	0.011799	0.011405	0.017936	0.011322	0.011672	0.011758
0.011718	0.011764	0.011374	0.011350	0.011774	0.011785	0.011425
0.011582	0.011671	0.011294	0.011353	0.011333	0.011406	0.011447
0.011576	0.011544	0.011505	0.011325	0.011530	0.011564	0.011567
0.011369	0.011530	0.011530	0.011497	0.011463	0.011463	0.011430
0.011463	0.011396	0.011429	0.011463	0.011430	0.011362	0.011396
0.011396	0.011463	0.011362	0.011463	0.011497	0.011530	0.011429
0.011463	0.011430	0.011765	0.011765	0.011497	0.011531	0.011564
0.011329	0.011329	0.011362	0.011396	0.011329	0.011564	0.011564
0.011597	0.011597	0.011530	0.011631	0.011799	0.011633	0.011396
0.011463	0.011530	0.011497	0.011396	0.011463	0.011429	0.011463
0.011531	0.011530	0.011564	0.011396	0.011363	0.011631	0.011530
0.011467	0.011362	0.011463	0.011564	0.011463	0.011598	0.011631
0.011799	0.011504	0.011665	0.011564	0.011564	0.011732	0.011363
0.011498	0.011430	0.011396	0.011396	0.011463	0.011362	0.011463
0.011497	0.011530	0.011429	0.011463	0.011430	0.011765	0.011765
0.011497	0.011531	0.011597	0.011530	0.012171	0.011596	0.011482
0.011588	0.011546	0.011623	0.011545	0.011655	0.011578	0.011543
0.012125	0.011441	0.011574	0.011510	0.011784	0.011639	0.011673
0.012149	0.011588	0.011466	0.011463	0.011463	0.011497	0.011432
0.011329	0.011295	0.011497	0.011463	0.011466	0.011597	0.011565
0.011429	0.011295	0.011329	0.011429	0.011262	0.011396	0.011295
0.011429	0.011463	0.011362	0.011329	0.011430	0.011362	0.011396
0.011396	0.011329	0.011396	0.011430	0.011329	0.011463	0.011564
0.011463	0.011530	0.011463	0.011463	0.011396	0.011799	0.011429
0.011443	0.011396	0.011632	0.011637	0.011430	0.011497	0.011497
0.011497	0.011362	0.011396	0.011362	0.011530	0.011530	0.011463
0.011463	0.011597	0.011765	0.011497	0.011698	0.011698	0.011564
0.011396	0.011463	0.011497	0.011900	0.011463	0.011430	0.011463
0.011497	0.011429	0.011429	0.011732	0.011598	0.011698	0.011430
0.011530	0.011597	0.011463	0.011530	0.011497	0.011362	0.011698
0.011463	0.011429	0.011429	0.011597	0.011430	0.011530	0.011766
0.011463	0.011842	0.011833	0.011629	0.011342	0.011536	0.011318
0.011445	0.011839	0.011376	0.011596	0.011402	0.011518	0.011485
0.011570	0.011258	0.011283	0.011258	0.011330	0.011395	0.011466
0.011512	0.011336	0.011518	0.011448	0.011465	0.011597	0.011665
0.011665	0.011531	0.011635	0.011636	0.011772	0.011802	0.011463
0.011698	0.011564	0.011429	0.011530	0.011329	0.011564	0.011362
0.011430	0.011429	0.011396	0.011362	0.011396	0.011362	0.011463
0.011444	0.011445	0.011437	0.011471	0.011608	0.011504	0.011572
0.011571	0.011497	0.011319	0.011312	0.011433	0.011602	0.011603
0.011396	0.011467	0.011700	0.011669	0.011598	0.011632	0.011564
0.011497	0.011564	0.011564	0.011463	0.012035	0.011631	0.011698
0.011732	0.011808	0.011435	0.011511	0.011396	0.011597	0.011564
0.011599	0.011631	0.011631	0.011631	0.011768	0.011766	0.011373
0.011297	0.011268	0.011401	0.011332	0.011470	0.011284	0.011638
0.011438	0.011543	0.011298	0.011435	0.011430	0.011505	0.011329
0.011362	0.011362	0.011362	0.011396	0.011597	0.011564	0.011497
0.011597	0.011665	0.011396	0.011396	0.011430	0.011429	0.011432
0.011500	0.011429	0.011430	0.011465	0.011597	0.011665	0.011665
0.011531	0.011635	0.011636	0.011772	0.011802	0.011463	0.011631
0.011564	0.011564	0.011398	0.011582	0.011512	0.011537	0.011376
0.011508	0.011744	0.011484	0.011434	0.011295	0.011497	0.011362
0.000000	0.011463	0.011362	0.011465	0.011564	0.011567	0.011463
0.011362	0.011296	0.011429	0.011362	0.011295	0.011400	0.011497
0.011463	0.011396	0.011429	0.011463	0.011430	0.011362	0.011564
0.011429	0.011463	0.011463	0.011396	0.011463	0.011698	0.011362
0.011396	0.011799	0.011396	0.011295	0.011396	0.011765	0.011317
0.011735	0.011358	0.011361	0.011584	0.011671	0.011599	0.011311
0.011323	0.011564	0.011665	0.011773	0.011632	0.011771	0.011901

0.011800	0.011838	0.011799	0.012051	0.011765	0.012641	0.011801
0.012540	0.012308	0.012442	0.012714	0.011765	0.011463	0.011766
0.012204	0.012140	0.011732	0.011631	0.011699	0.011699	0.011669
0.011564	0.011569	0.011631	0.011598	0.011968	0.012035	0.012036
0.011935	0.012035	0.012139	0.011906	0.012205	0.012212	0.012272
0.012273	0.012307	0.012255	0.012273	0.012274	0.011497	0.011800
0.011867	0.012371	0.011874	0.012102	0.012575	0.011638	0.011531
0.011497	0.011497	0.011968	0.012006	0.012116	0.011833	0.011498
S184	0.011698	0.011698	0.012035	0.011799	0.011799	0.011866
0.011706	0.011638	0.011799	0.011295	0.011362	0.011329	0.011295
0.011329	0.011497	0.011295	0.011329	0.011396	0.011362	0.011329
0.011295	0.011329	0.011429	0.011296	0.011329	0.011329	0.011672
0.011564	0.011329	0.011228	0.011463	0.011396	0.011363	0.011396
0.011362	0.011463	0.011362	0.011362	0.011396	0.011362	0.011530
0.011295	0.011362	0.011396	0.011329	0.011430	0.011463	0.011396
0.011362	0.011531	0.011430	0.011698	0.011362	0.011631	0.011362
0.011329	0.011430	0.011497	0.011631	0.011732	0.011430	0.011530
0.011396	0.011463	0.011363	0.011597	0.011833	0.011362	0.011430
0.011329	0.011430	0.011396	0.011463	0.011396	0.011396	0.011396
0.011295	0.011329	0.011295	0.011396	0.011362	0.011397	0.011463
0.011396	0.011362	0.011430	0.011329	0.011463	0.011295	0.011362
0.011530	0.011463	0.011631	0.011530	0.011362	0.011598	0.011362
0.011542	0.011695	0.011551	0.011255	0.011247	0.011391	0.011493
0.012121	0.011518	0.011225	0.011597	0.011598	0.011396	0.011497
0.011430	0.011396	0.011463	0.011362	0.011463	0.011396	0.011396
0.011362	0.011463	0.011665	0.011530	0.011430	0.011396	0.011329
0.011295	0.011295	0.011362	0.011329	0.011530	0.011497	0.011430
0.011530	0.011597	0.011329	0.011329	0.011362	0.011362	0.011364
0.011432	0.011362	0.011362	0.011240	0.011313	0.011271	0.011363
0.011365	0.011301	0.011282	0.011336	0.011264	0.011300	0.011330
0.011600	0.011370	0.011405	0.011296	0.011302	0.011430	0.011397
0.011464	0.011397	0.011396	0.011497	0.011429	0.011799	0.011598
0.011631	0.011497	0.011362	0.011463	0.011262	0.011497	0.011295
0.011362	0.011362	0.011329	0.011295	0.011329	0.011295	0.011396
0.011362	0.011463	0.000000	0.011295	0.011397	0.011497	0.011500
0.011497	0.011396	0.011295	0.011229	0.011362	0.011295	0.011228
0.011332	0.011497	0.011295	0.011497	0.011262	0.011363	0.011329
0.011430	0.011329	0.011362	0.011329	0.011362	0.011228	0.011362
0.011396	0.011329	0.011396	0.011329	0.011362	0.011396	0.011631
0.011732	0.011665	0.011732	0.011498	0.011732	0.011362	0.011329
0.011364	0.011626	0.011426	0.011266	0.011739	0.011231	0.011336
0.011739	0.011638	0.011530	0.011732	0.011436	0.011598	0.011564
0.011497	0.011665	0.011522	0.011349	0.011396	0.011396	0.011466
0.011564	0.011465	0.011601	0.011670	0.011598	0.011665	0.011772
0.011500	0.011672	0.011602	0.011534	0.011801	0.011262	0.011465
0.011430	0.011362	0.011262	0.011262	0.011329	0.011329	0.011396
0.011329	0.011396	0.011295	0.011396	0.011329	0.011329	0.011396
0.011329	0.011765	0.011665	0.011698	0.011228	0.011329	0.011363
0.011362	0.011329	0.011396	0.011430	0.011295	0.011295	0.011295
0.011833	0.011463	0.011463	0.011262	0.011362	0.011531	0.011295
0.011295	0.011362	0.011329	0.011497	0.011295	0.011430	0.011564
0.011497	0.011329	0.011329	0.011262	0.011262	0.011430	0.011362
0.011262	0.011362	0.011228	0.011262	0.011396	0.011531	0.011295
0.011262	0.011396	0.011329	0.011228	0.011564	0.011228	0.011396
0.011471	0.011295	0.011598	0.011430	0.011463	0.011329	0.011430
0.011295	0.011631	0.011228	0.011497	0.011295	0.011295	0.011228
0.011463	0.011665	0.011363	0.011430	0.011262	0.011665	0.011295
0.011295	0.011295	0.011329	0.011430	0.011329	0.011430	0.011497
0.011497	0.011463	0.011362	0.011463	0.011430	0.011295	0.011262
0.011295	0.011295	0.011363	0.011295	0.011262	0.011430	0.011295

0.011329	0.011329	0.011463	0.011497	0.011396	0.011329	0.011329
0.011396	0.011329	0.011430	0.011295	0.011430	0.011530	0.011833
0.011329	0.011732	0.011336	0.017861	0.011322	0.011427	0.011688
0.011648	0.011693	0.011427	0.011408	0.011719	0.011716	0.011364
0.011582	0.011548	0.011216	0.011312	0.011333	0.011356	0.011318
0.011576	0.011470	0.011468	0.011325	0.011463	0.011497	0.011500
0.011370	0.011463	0.011463	0.011430	0.011396	0.011396	0.011363
0.011396	0.011329	0.011362	0.011396	0.011363	0.011295	0.011329
0.011329	0.011396	0.011295	0.011396	0.011430	0.011463	0.011362
0.011396	0.011362	0.011698	0.011698	0.011430	0.011464	0.011497
0.011262	0.011262	0.011295	0.011396	0.011262	0.011497	0.011497
0.011530	0.011530	0.011463	0.011564	0.011732	0.011565	0.011396
0.011396	0.011463	0.011497	0.011329	0.011396	0.011362	0.011396
0.011463	0.011463	0.011497	0.011463	0.011363	0.011564	0.011530
0.011399	0.011295	0.011396	0.011497	0.011396	0.011530	0.011564
0.011732	0.011436	0.011598	0.011564	0.011497	0.011665	0.011295
0.011464	0.011363	0.011329	0.011329	0.011396	0.011295	0.011396
0.011430	0.011463	0.011362	0.011396	0.011362	0.011698	0.011698
0.011430	0.011464	0.011530	0.011463	0.012104	0.011525	0.011447
0.011517	0.011477	0.011588	0.011476	0.011585	0.011509	0.011474
0.012057	0.011372	0.011506	0.011441	0.011715	0.011571	0.011673
0.012081	0.011512	0.011398	0.011396	0.011396	0.011430	0.011364
0.011262	0.011295	0.011430	0.011396	0.011398	0.011530	0.011498
0.011362	0.011295	0.011329	0.011362	0.011262	0.011329	0.011228
0.011362	0.011396	0.011295	0.011262	0.011362	0.011295	0.011329
0.011329	0.011262	0.011329	0.011363	0.011262	0.011463	0.011497
0.011396	0.011463	0.011396	0.011396	0.011329	0.011732	0.011362
0.011408	0.011329	0.011564	0.011569	0.011430	0.011430	0.011362
0.011430	0.011363	0.011329	0.011363	0.011463	0.011463	0.011396
0.011396	0.011530	0.011698	0.011429	0.011631	0.011631	0.011497
0.011329	0.011396	0.011430	0.011833	0.011396	0.011430	0.011396
0.011430	0.011362	0.011362	0.011665	0.011598	0.011631	0.011362
0.011463	0.011530	0.011396	0.011463	0.011430	0.011295	0.011631
0.011396	0.011362	0.011362	0.011530	0.011362	0.011463	0.011698
0.011396	0.011774	0.011766	0.011548	0.011286	0.011468	0.011318
0.011225	0.011761	0.011306	0.011462	0.011304	0.011417	0.011432
0.011519	0.011258	0.011394	0.011258	0.011276	0.011462	0.011466
0.011475	0.011375	0.011448	0.011377	0.011397	0.011530	0.011598
0.011530	0.011464	0.011567	0.011569	0.011704	0.011696	0.011396
0.011631	0.011497	0.011362	0.011463	0.011262	0.011497	0.011295
0.011362	0.011362	0.011329	0.011295	0.011329	0.011295	0.011396
0.011375	0.011375	0.011368	0.011403	0.011539	0.011504	0.011504
0.011503	0.011430	0.011245	0.011240	0.011365	0.011535	0.011535
0.011329	0.011399	0.011633	0.011601	0.011531	0.011565	0.011497
0.011430	0.011497	0.011497	0.011396	0.011968	0.011564	0.011631
0.011665	0.011701	0.011367	0.011441	0.011329	0.011530	0.011497
0.011531	0.011564	0.011564	0.011564	0.011729	0.011698	0.011338
0.011229	0.011233	0.011333	0.011298	0.011402	0.011210	0.011570
0.011369	0.011499	0.011251	0.011397	0.011363	0.011437	0.011329
0.011295	0.011295	0.011362	0.011329	0.011530	0.011497	0.011430
0.011530	0.011597	0.011329	0.011329	0.011362	0.011362	0.011364
0.011432	0.011362	0.011362	0.011397	0.011530	0.011598	0.011530
0.011464	0.011567	0.011569	0.011704	0.011696	0.011396	0.011564
0.011497	0.011497	0.011331	0.011513	0.011442	0.011469	0.011341
0.011508	0.011698	0.011483	0.011400	0.011295	0.011497	0.011362
0.011463	0.000000	0.011295	0.011397	0.011497	0.011500	0.011396
0.011295	0.011229	0.011362	0.011295	0.011228	0.011332	0.011497
0.011396	0.011329	0.011362	0.011396	0.011363	0.011295	0.011497
0.011362	0.011396	0.011396	0.011329	0.011396	0.011631	0.011295
0.011329	0.011732	0.011329	0.011362	0.011329	0.011698	0.011232

0.011735	0.011273	0.011311	0.011503	0.011589	0.011527	0.011186
0.011270	0.011497	0.011597	0.011705	0.011565	0.011703	0.011834
0.011733	0.011771	0.011732	0.011983	0.011698	0.012573	0.011734
0.012473	0.012241	0.012375	0.012646	0.011698	0.011396	0.011699
0.012137	0.012072	0.011665	0.011564	0.011632	0.011632	0.011602
0.011497	0.011501	0.011564	0.011531	0.011901	0.011967	0.011968
0.011868	0.011968	0.012072	0.011838	0.012138	0.012144	0.012205
0.012206	0.012239	0.012186	0.012206	0.012207	0.011429	0.011732
0.011800	0.012304	0.011806	0.012035	0.012508	0.011570	0.011464
0.011430	0.011429	0.011900	0.011965	0.012045	0.011765	0.011431
S185	0.011597	0.011597	0.011934	0.011698	0.011698	0.011765
0.011604	0.011536	0.011698	0.011195	0.011262	0.011228	0.011195
0.011228	0.011396	0.011195	0.011228	0.011295	0.011262	0.011228
0.011195	0.011228	0.011329	0.011195	0.011228	0.011228	0.011570
0.011463	0.011228	0.011127	0.011362	0.011295	0.011262	0.011295
0.011262	0.011362	0.011262	0.011262	0.011295	0.011262	0.011430
0.011195	0.011262	0.011295	0.011228	0.011329	0.011362	0.011295
0.011262	0.011430	0.011329	0.011597	0.011262	0.011530	0.011262
0.011228	0.011329	0.011396	0.011530	0.011631	0.011329	0.011429
0.011295	0.011362	0.011262	0.011497	0.011732	0.011262	0.011329
0.011228	0.011329	0.011295	0.011362	0.011295	0.011295	0.011295
0.011195	0.011228	0.011195	0.011295	0.011262	0.011296	0.011362
0.011295	0.011262	0.011329	0.011228	0.011362	0.011195	0.011262
0.011429	0.011362	0.011597	0.011430	0.011262	0.011497	0.011262
0.011357	0.011658	0.011475	0.011177	0.011172	0.011207	0.011336
0.012011	0.011365	0.011184	0.011497	0.011497	0.011295	0.011396
0.011329	0.011295	0.011362	0.011262	0.011362	0.011295	0.011295
0.011262	0.011362	0.011564	0.011429	0.011329	0.011295	0.011228
0.011195	0.011195	0.011262	0.011228	0.011429	0.011396	0.011329
0.011429	0.011497	0.011228	0.011228	0.011262	0.011262	0.011263
0.011331	0.011262	0.011262	0.011096	0.011205	0.011165	0.011262
0.011263	0.011198	0.011245	0.011233	0.011162	0.011197	0.011229
0.011456	0.011233	0.011301	0.011195	0.011199	0.011329	0.011296
0.011363	0.011229	0.011228	0.011396	0.011329	0.011698	0.011497
0.011530	0.011396	0.011262	0.011362	0.011161	0.011396	0.011195
0.011262	0.011262	0.011228	0.011195	0.011228	0.011195	0.011295
0.011262	0.011362	0.011295	0.000000	0.011296	0.011396	0.011398
0.011396	0.011295	0.011195	0.011128	0.011262	0.011195	0.011127
0.011230	0.011396	0.011195	0.011396	0.011161	0.011262	0.011228
0.011329	0.011228	0.011262	0.011228	0.011262	0.011127	0.011262
0.011295	0.011228	0.011295	0.011228	0.011262	0.011295	0.011530
0.011631	0.011564	0.011631	0.011397	0.011631	0.011262	0.011228
0.011229	0.011505	0.011335	0.011163	0.011637	0.011129	0.011257
0.011637	0.011536	0.011429	0.011631	0.011333	0.011497	0.011463
0.011396	0.011564	0.011416	0.011266	0.011312	0.011295	0.011365
0.011463	0.011364	0.011500	0.011568	0.011497	0.011564	0.011670
0.011399	0.011569	0.011500	0.011432	0.011699	0.011161	0.011364
0.011329	0.011262	0.011161	0.011161	0.011228	0.011228	0.011295
0.011228	0.011295	0.011195	0.011295	0.011228	0.011228	0.011295
0.011228	0.011665	0.011564	0.011597	0.011127	0.011228	0.011262
0.011262	0.011228	0.011295	0.011329	0.011195	0.011195	0.011195
0.011732	0.011362	0.011362	0.011161	0.011262	0.011430	0.011195
0.011195	0.011262	0.011228	0.011396	0.011195	0.011329	0.011463
0.011396	0.011228	0.011228	0.011161	0.011161	0.011329	0.011262
0.011161	0.011262	0.011127	0.011161	0.011295	0.011430	0.011195
0.011161	0.011295	0.011228	0.011127	0.011463	0.011127	0.011295
0.011368	0.011195	0.011497	0.011329	0.011362	0.011228	0.011329
0.011195	0.011530	0.011127	0.011396	0.011195	0.011195	0.011127
0.011362	0.011564	0.011262	0.011329	0.011161	0.011564	0.011195
0.011195	0.011195	0.011228	0.011329	0.011228	0.011329	0.011396

0.011396	0.011362	0.011262	0.011362	0.011329	0.011195	0.011161
0.011195	0.011195	0.011262	0.011195	0.011161	0.011329	0.011195
0.011228	0.011228	0.011362	0.011396	0.011295	0.011228	0.011228
0.011295	0.011228	0.011329	0.011195	0.011329	0.011429	0.011732
0.011228	0.011631	0.011233	0.017747	0.011126	0.011427	0.011583
0.011545	0.011588	0.011217	0.011234	0.011664	0.011612	0.011303
0.011440	0.011487	0.011138	0.011228	0.011216	0.011258	0.011232
0.011436	0.011395	0.011394	0.011126	0.011362	0.011396	0.011398
0.011266	0.011362	0.011362	0.011329	0.011295	0.011295	0.011262
0.011295	0.011228	0.011262	0.011295	0.011262	0.011195	0.011228
0.011228	0.011295	0.011195	0.011295	0.011329	0.011362	0.011262
0.011295	0.011262	0.011597	0.011597	0.011329	0.011363	0.011396
0.011161	0.011161	0.011195	0.011295	0.011161	0.011396	0.011396
0.011429	0.011429	0.011362	0.011463	0.011631	0.011464	0.011295
0.011295	0.011362	0.011396	0.011228	0.011295	0.011262	0.011295
0.011363	0.011362	0.011396	0.011362	0.011262	0.011463	0.011429
0.011297	0.011195	0.011295	0.011396	0.011295	0.011430	0.011463
0.011631	0.011333	0.011497	0.011463	0.011396	0.011564	0.011195
0.011363	0.011262	0.011228	0.011228	0.011295	0.011195	0.011295
0.011329	0.011362	0.011262	0.011295	0.011262	0.011597	0.011597
0.011329	0.011363	0.011429	0.011362	0.012002	0.011417	0.011342
0.011412	0.011372	0.011482	0.011372	0.011480	0.011405	0.011371
0.011953	0.011268	0.011403	0.011337	0.011611	0.011468	0.011571
0.011978	0.011437	0.011297	0.011295	0.011295	0.011329	0.011263
0.011161	0.011195	0.011329	0.011295	0.011297	0.011429	0.011397
0.011262	0.011195	0.011228	0.011262	0.011161	0.011228	0.011127
0.011262	0.011295	0.011195	0.011161	0.011262	0.011195	0.011228
0.011228	0.011161	0.011228	0.011262	0.011161	0.011362	0.011396
0.011295	0.011362	0.011295	0.011295	0.011228	0.011631	0.011262
0.011304	0.011228	0.011463	0.011467	0.011329	0.011329	0.011329
0.011329	0.011262	0.011228	0.011262	0.011362	0.011362	0.011295
0.011295	0.011429	0.011597	0.011329	0.011530	0.011530	0.011396
0.011228	0.011295	0.011329	0.011732	0.011295	0.011329	0.011295
0.011329	0.011262	0.011262	0.011564	0.011497	0.011530	0.011262
0.011362	0.011362	0.011295	0.011362	0.011329	0.011195	0.011530
0.011295	0.011262	0.011262	0.011429	0.011262	0.011362	0.011597
0.011295	0.011672	0.011665	0.011467	0.011117	0.011366	0.011163
0.011225	0.011683	0.011201	0.011417	0.011207	0.011315	0.011326
0.011366	0.011126	0.011227	0.000000	0.011168	0.011261	0.011303
0.011324	0.011218	0.011342	0.011236	0.011296	0.011429	0.011497
0.011497	0.011363	0.011466	0.011467	0.011602	0.011625	0.011295
0.011530	0.011396	0.011262	0.011362	0.011161	0.011396	0.011195
0.011262	0.011262	0.011228	0.011195	0.011228	0.011195	0.011295
0.011270	0.011270	0.011266	0.011300	0.011436	0.011402	0.011402
0.011401	0.011329	0.011097	0.011096	0.011263	0.011433	0.011433
0.011228	0.011297	0.011531	0.011499	0.011430	0.011464	0.011396
0.011329	0.011396	0.011396	0.011295	0.011866	0.011463	0.011530
0.011564	0.011630	0.011265	0.011337	0.011228	0.011430	0.011396
0.011430	0.011463	0.011463	0.011463	0.011571	0.011597	0.011234
0.011128	0.011130	0.011230	0.011196	0.011299	0.011135	0.011468
0.011266	0.011411	0.011108	0.011285	0.011262	0.011334	0.011228
0.011195	0.011195	0.011262	0.011228	0.011429	0.011396	0.011329
0.011429	0.011497	0.011228	0.011228	0.011262	0.011262	0.011263
0.011331	0.011262	0.011262	0.011296	0.011429	0.011497	0.011497
0.011363	0.011466	0.011467	0.011602	0.011625	0.011295	0.011463
0.011396	0.011396	0.011229	0.011408	0.011338	0.011367	0.011235
0.011404	0.011607	0.011357	0.011298	0.011195	0.011396	0.011262
0.011362	0.011295	0.000000	0.011296	0.011396	0.011398	0.011295
0.011195	0.011128	0.011262	0.011195	0.011127	0.011230	0.011396
0.011295	0.011228	0.011262	0.011295	0.011262	0.011195	0.011396

0.011262	0.011295	0.011295	0.011228	0.011295	0.011530	0.011195
0.011228	0.011631	0.011228	0.011262	0.011228	0.011597	0.011189
0.011678	0.011188	0.011211	0.011422	0.011508	0.011419	0.011144
0.011113	0.011396	0.011497	0.011603	0.011464	0.011601	0.011733
0.011632	0.011669	0.011631	0.011880	0.011597	0.012405	0.011633
0.012372	0.012072	0.012206	0.012545	0.011597	0.011295	0.011598
0.012036	0.011971	0.011564	0.011463	0.011531	0.011531	0.011500
0.011396	0.011399	0.011463	0.011430	0.011800	0.011866	0.011867
0.011767	0.011867	0.011970	0.011737	0.012036	0.012042	0.012103
0.012104	0.012138	0.012084	0.012104	0.012105	0.011329	0.011631
0.011699	0.012203	0.011704	0.011934	0.012406	0.011468	0.011363
0.011329	0.011329	0.011799	0.011800	0.011939	0.011665	0.011330
S186	0.011701	0.011701	0.012038	0.011802	0.011802	0.011869
0.011708	0.011640	0.011734	0.011296	0.011363	0.011330	0.011296
0.011330	0.011431	0.011296	0.011330	0.011363	0.011363	0.011330
0.011296	0.011330	0.011397	0.011296	0.011330	0.011330	0.011674
0.011566	0.011330	0.011229	0.011431	0.011397	0.011364	0.011397
0.011363	0.011465	0.011363	0.011363	0.011397	0.011363	0.011532
0.011296	0.011363	0.011397	0.011330	0.011431	0.011464	0.011397
0.011363	0.011532	0.011431	0.011700	0.011363	0.011633	0.011363
0.011330	0.011431	0.011498	0.011633	0.011734	0.011431	0.011532
0.011397	0.011465	0.011363	0.011599	0.011836	0.011296	0.011431
0.011330	0.011431	0.011330	0.011465	0.011397	0.011397	0.011397
0.011296	0.011330	0.011296	0.011397	0.011363	0.011398	0.011464
0.011397	0.011363	0.011431	0.011330	0.011464	0.011296	0.011363
0.011532	0.011464	0.011700	0.011532	0.011363	0.011599	0.011296
0.011544	0.011773	0.011590	0.011295	0.011248	0.011392	0.011455
0.012089	0.011569	0.011267	0.011599	0.011599	0.011397	0.011498
0.011431	0.011397	0.011464	0.011363	0.011465	0.011397	0.011397
0.011363	0.011464	0.011667	0.011464	0.011431	0.011397	0.011330
0.011296	0.011296	0.011363	0.011330	0.011532	0.011498	0.011431
0.011532	0.011532	0.011330	0.011330	0.011363	0.011363	0.011365
0.011433	0.011363	0.011363	0.011241	0.011278	0.011236	0.011364
0.011366	0.011302	0.011356	0.011337	0.011265	0.011301	0.011331
0.011566	0.011372	0.011406	0.011296	0.011303	0.011432	0.011398
0.011465	0.011398	0.011398	0.011498	0.011431	0.011802	0.011600
0.011633	0.011498	0.011363	0.011465	0.011262	0.011498	0.011296
0.011363	0.011363	0.011330	0.011296	0.011330	0.011296	0.011397
0.011363	0.011465	0.011397	0.011296	0.000000	0.011431	0.011467
0.011498	0.011364	0.011296	0.011229	0.011363	0.011296	0.011229
0.011333	0.011498	0.011296	0.011498	0.011262	0.011363	0.011330
0.011431	0.011330	0.011363	0.011330	0.011363	0.011229	0.011363
0.011397	0.011330	0.011397	0.011330	0.011363	0.011397	0.011633
0.011734	0.011667	0.011734	0.011500	0.011734	0.011363	0.011330
0.011365	0.011667	0.011426	0.011266	0.011673	0.011231	0.011336
0.011742	0.011640	0.011532	0.011734	0.011437	0.011599	0.011566
0.011498	0.011667	0.011488	0.011350	0.011397	0.011397	0.011434
0.011566	0.011466	0.011603	0.011672	0.011599	0.011667	0.011774
0.011502	0.011674	0.011604	0.011535	0.011803	0.011262	0.011466
0.011431	0.011363	0.011262	0.011262	0.011330	0.011262	0.011397
0.011330	0.011397	0.011296	0.011397	0.011330	0.011330	0.011397
0.011330	0.011768	0.011667	0.011701	0.011229	0.011330	0.011363
0.011363	0.011330	0.011397	0.011431	0.011296	0.011296	0.011296
0.011835	0.011464	0.011465	0.011262	0.011363	0.011532	0.011296
0.011296	0.011363	0.011330	0.011498	0.011262	0.011431	0.011566
0.011498	0.011330	0.011330	0.011262	0.011262	0.011431	0.011363
0.011262	0.011363	0.011229	0.011262	0.011397	0.011532	0.011262
0.011263	0.011397	0.011330	0.011229	0.011566	0.011229	0.011330
0.011473	0.011296	0.011599	0.011431	0.011465	0.011330	0.011431
0.011296	0.011633	0.011229	0.011498	0.011296	0.011296	0.011229

0.011464	0.011667	0.011363	0.011431	0.011262	0.011667	0.011296
0.011296	0.011296	0.011330	0.011431	0.011330	0.011431	0.011498
0.011498	0.011465	0.011363	0.011465	0.011431	0.011296	0.011262
0.011296	0.011296	0.011363	0.011296	0.011262	0.011431	0.011296
0.011330	0.011330	0.011465	0.011498	0.011397	0.011330	0.011330
0.011397	0.011330	0.011431	0.011296	0.011431	0.011465	0.011802
0.011330	0.011734	0.011337	0.017849	0.011258	0.011491	0.011690
0.011651	0.011696	0.011374	0.011292	0.011778	0.011719	0.011427
0.011487	0.011613	0.011256	0.011355	0.011333	0.011358	0.011363
0.011529	0.011471	0.011470	0.011259	0.011465	0.011498	0.011501
0.011371	0.011464	0.011465	0.011431	0.011397	0.011397	0.011364
0.011397	0.011330	0.011363	0.011397	0.011363	0.011296	0.011330
0.011330	0.011397	0.011296	0.011397	0.011431	0.011464	0.011363
0.011397	0.011363	0.011700	0.011700	0.011431	0.011330	0.011498
0.011262	0.011262	0.011296	0.011397	0.011262	0.011498	0.011498
0.011532	0.011532	0.011465	0.011566	0.011734	0.011567	0.011397
0.011397	0.011464	0.011498	0.011330	0.011397	0.011363	0.011397
0.011465	0.011465	0.011498	0.011465	0.011364	0.011566	0.011532
0.011366	0.011296	0.011397	0.011498	0.011397	0.011532	0.011566
0.011734	0.011437	0.011599	0.011566	0.011498	0.011667	0.011296
0.011466	0.011296	0.011330	0.011330	0.011397	0.011296	0.011397
0.011431	0.011464	0.011363	0.011397	0.011363	0.011700	0.011700
0.011431	0.011330	0.011532	0.011465	0.012107	0.011526	0.011413
0.011519	0.011478	0.011589	0.011477	0.011587	0.011510	0.011476
0.012060	0.011373	0.011507	0.011442	0.011717	0.011572	0.011675
0.012084	0.011514	0.011400	0.011397	0.011397	0.011431	0.011365
0.011262	0.011296	0.011431	0.011397	0.011399	0.011532	0.011500
0.011363	0.011296	0.011330	0.011363	0.011262	0.011330	0.011229
0.011363	0.011397	0.011296	0.011262	0.011363	0.011296	0.011330
0.011330	0.011262	0.011330	0.011363	0.011262	0.011465	0.011498
0.011397	0.011465	0.011397	0.011397	0.011330	0.011734	0.011363
0.011375	0.011330	0.011566	0.011571	0.011431	0.011431	0.011431
0.011431	0.011363	0.011330	0.011363	0.011397	0.011431	0.011397
0.011397	0.011532	0.011700	0.011431	0.011633	0.011633	0.011498
0.011330	0.011397	0.011431	0.011836	0.011397	0.011431	0.011397
0.011431	0.011363	0.011363	0.011667	0.011600	0.011633	0.011363
0.011465	0.011532	0.011397	0.011465	0.011431	0.011296	0.011633
0.011397	0.011363	0.011363	0.011532	0.011363	0.011464	0.011701
0.011397	0.011776	0.011768	0.011550	0.011230	0.011469	0.011267
0.011281	0.011764	0.011307	0.011509	0.011354	0.011367	0.011434
0.011519	0.011259	0.011340	0.011193	0.011277	0.011397	0.011467
0.011476	0.011337	0.011449	0.011343	0.011398	0.011532	0.011599
0.011566	0.011465	0.011569	0.011570	0.011707	0.011734	0.011397
0.011633	0.011498	0.011363	0.011465	0.011262	0.011498	0.011296
0.011363	0.011363	0.011330	0.011296	0.011330	0.011296	0.011397
0.011376	0.011376	0.011370	0.011404	0.011541	0.011506	0.011506
0.011505	0.011431	0.011246	0.011241	0.011298	0.011536	0.011537
0.011330	0.011400	0.011634	0.011603	0.011533	0.011499	0.011498
0.011431	0.011498	0.011498	0.011397	0.011971	0.011566	0.011633
0.011599	0.011739	0.011368	0.011443	0.011330	0.011532	0.011498
0.011533	0.011566	0.011566	0.011566	0.011731	0.011701	0.011304
0.011230	0.011199	0.011334	0.011299	0.011403	0.011210	0.011572
0.011370	0.011500	0.011251	0.011361	0.011364	0.011438	0.011330
0.011296	0.011296	0.011363	0.011330	0.011532	0.011498	0.011431
0.011532	0.011532	0.011330	0.011330	0.011363	0.011363	0.011365
0.011433	0.011363	0.011363	0.011398	0.011532	0.011599	0.011566
0.011465	0.011569	0.011570	0.011707	0.011734	0.011397	0.011566
0.011498	0.011498	0.011332	0.011514	0.011374	0.011470	0.011341
0.011509	0.011698	0.011484	0.011401	0.011296	0.011498	0.011363
0.011465	0.011397	0.011296	0.000000	0.011431	0.011467	0.011364

0.011296	0.011229	0.011363	0.011296	0.011229	0.011333	0.011498
0.011397	0.011330	0.011363	0.011397	0.011363	0.011296	0.011498
0.011363	0.011397	0.011397	0.011330	0.011397	0.011633	0.011296
0.011330	0.011734	0.011330	0.011363	0.011330	0.011700	0.011232
0.011678	0.011231	0.011261	0.011505	0.011591	0.011528	0.011228
0.011218	0.011498	0.011599	0.011708	0.011567	0.011705	0.011836
0.011735	0.011773	0.011734	0.011986	0.011701	0.012578	0.011736
0.012478	0.012245	0.012379	0.012652	0.011700	0.011397	0.011667
0.012141	0.012042	0.011667	0.011566	0.011634	0.011634	0.011603
0.011498	0.011469	0.011566	0.011533	0.011904	0.011971	0.011972
0.011871	0.011971	0.012075	0.011841	0.012141	0.012148	0.012209
0.012209	0.012209	0.012190	0.012176	0.012177	0.011431	0.011735
0.011803	0.012308	0.011809	0.012038	0.012513	0.011571	0.011465
0.011431	0.011431	0.011903	0.011928	0.012049	0.011768	0.011432
S187	0.011799	0.011799	0.012136	0.011900	0.011900	0.011967
0.011808	0.011740	0.011900	0.011396	0.011463	0.011430	0.011396
0.011430	0.011598	0.011396	0.011430	0.011497	0.011463	0.011430
0.011396	0.011430	0.011530	0.011396	0.011396	0.011430	0.011774
0.011665	0.011430	0.011329	0.011564	0.011497	0.011463	0.011497
0.011463	0.011564	0.011463	0.011463	0.011497	0.011463	0.011631
0.011396	0.011463	0.011497	0.011430	0.011530	0.011564	0.011497
0.011463	0.011631	0.011531	0.011799	0.011463	0.011732	0.011463
0.011430	0.011530	0.011598	0.011732	0.011833	0.011530	0.011631
0.011497	0.011564	0.011463	0.011698	0.011934	0.011463	0.011530
0.011430	0.011530	0.011497	0.011564	0.011497	0.011497	0.011497
0.011396	0.011430	0.011396	0.011497	0.011463	0.011498	0.011564
0.011497	0.011463	0.011530	0.011429	0.011564	0.011396	0.011463
0.011631	0.011564	0.011799	0.011631	0.011463	0.011698	0.011463
0.011654	0.011882	0.011702	0.011412	0.011396	0.011391	0.011611
0.012231	0.011620	0.011431	0.011698	0.011698	0.011497	0.011598
0.011530	0.011497	0.011564	0.011463	0.011564	0.011497	0.011497
0.011463	0.011564	0.011766	0.011631	0.011530	0.011497	0.011430
0.011396	0.011396	0.011463	0.011430	0.011631	0.011597	0.011530
0.011631	0.011698	0.011429	0.011429	0.011463	0.011463	0.011465
0.011533	0.011463	0.011463	0.011348	0.011422	0.011376	0.011464
0.011467	0.011404	0.011466	0.011440	0.011366	0.011403	0.011432
0.011672	0.011474	0.011508	0.011396	0.011406	0.011531	0.011497
0.011565	0.011498	0.011497	0.011597	0.011530	0.011900	0.011699
0.011732	0.011598	0.011463	0.011564	0.011362	0.011597	0.011396
0.011463	0.011463	0.011430	0.011396	0.011430	0.011396	0.011497
0.011463	0.011564	0.011497	0.011396	0.011431	0.000000	0.011466
0.011598	0.011497	0.011396	0.011263	0.011463	0.011396	0.011329
0.011434	0.011598	0.011396	0.011598	0.011362	0.011463	0.011430
0.011530	0.011430	0.011463	0.011430	0.011463	0.011329	0.011463
0.011497	0.011430	0.011497	0.011429	0.011463	0.011497	0.011732
0.011833	0.011765	0.011833	0.011600	0.011833	0.011463	0.011430
0.011466	0.011586	0.011380	0.011266	0.011841	0.011333	0.011454
0.011841	0.011740	0.011631	0.011833	0.011538	0.011698	0.011665
0.011598	0.011765	0.011593	0.011472	0.011480	0.011497	0.011568
0.011665	0.011566	0.011703	0.011771	0.011698	0.011766	0.011874
0.011602	0.011773	0.011704	0.011635	0.011902	0.011362	0.011566
0.011530	0.011463	0.011362	0.011362	0.011430	0.011430	0.011497
0.011430	0.011497	0.011396	0.011497	0.011430	0.011430	0.011497
0.011430	0.011866	0.011766	0.011799	0.011329	0.011430	0.011463
0.011463	0.011430	0.011497	0.011530	0.011396	0.011396	0.011396
0.011934	0.011564	0.011564	0.011362	0.011463	0.011631	0.011396
0.011396	0.011463	0.011430	0.011598	0.011396	0.011530	0.011665
0.011597	0.011430	0.011430	0.011362	0.011362	0.011530	0.011463
0.011362	0.011463	0.011329	0.011362	0.011497	0.011631	0.011396
0.011363	0.011497	0.011430	0.011329	0.011665	0.011329	0.011497

0.011540	0.011396	0.011698	0.011531	0.011564	0.011430	0.011530
0.011396	0.011732	0.011329	0.011598	0.011396	0.011396	0.011329
0.011564	0.011766	0.011463	0.011530	0.011362	0.011766	0.011396
0.011396	0.011396	0.011430	0.011530	0.011430	0.011195	0.011597
0.011598	0.011564	0.011463	0.011564	0.011530	0.011396	0.011362
0.011396	0.011396	0.011463	0.011396	0.011362	0.011530	0.011396
0.011430	0.011430	0.011564	0.011598	0.011497	0.011430	0.011430
0.011497	0.011430	0.011530	0.011396	0.011530	0.011631	0.011934
0.011430	0.011833	0.011440	0.017937	0.011453	0.011672	0.011793
0.011752	0.011799	0.011374	0.011408	0.011994	0.011820	0.011607
0.011582	0.011793	0.011372	0.011437	0.011333	0.011554	0.011490
0.011623	0.011619	0.011616	0.011325	0.011564	0.011598	0.011601
0.011473	0.011564	0.011564	0.011530	0.011497	0.011497	0.011463
0.011497	0.011430	0.011463	0.011497	0.011463	0.011396	0.011429
0.011429	0.011497	0.011396	0.011497	0.011530	0.011564	0.011463
0.011497	0.011463	0.011799	0.011799	0.011531	0.011531	0.011598
0.011362	0.011362	0.011396	0.011497	0.011362	0.011597	0.011597
0.011631	0.011631	0.011564	0.011665	0.011833	0.011667	0.011497
0.011497	0.011564	0.011598	0.011430	0.011497	0.011463	0.011497
0.011564	0.011564	0.011597	0.011564	0.011431	0.011665	0.011631
0.011501	0.011396	0.011497	0.011598	0.011497	0.011631	0.011665
0.011833	0.011538	0.011698	0.011665	0.011598	0.011765	0.011396
0.011566	0.011464	0.011429	0.011429	0.011497	0.011396	0.011497
0.011530	0.011564	0.011463	0.011497	0.011463	0.011799	0.011799
0.011531	0.011531	0.011631	0.011564	0.012205	0.011632	0.011518
0.011623	0.011581	0.011658	0.011545	0.011691	0.011612	0.011578
0.012160	0.011476	0.011608	0.011545	0.011818	0.011673	0.011775
0.012149	0.011626	0.011500	0.011497	0.011497	0.011530	0.011466
0.011362	0.011396	0.011530	0.011497	0.011500	0.011631	0.011599
0.011463	0.011396	0.011430	0.011463	0.011362	0.011430	0.011329
0.011463	0.011497	0.011396	0.011362	0.011463	0.011396	0.011430
0.011430	0.011362	0.011430	0.011463	0.011362	0.011564	0.011597
0.011497	0.011564	0.011497	0.011497	0.011430	0.011833	0.011463
0.011513	0.011430	0.011665	0.011671	0.011530	0.011530	0.011530
0.011530	0.011463	0.011430	0.011463	0.011564	0.011564	0.011497
0.011497	0.011631	0.011799	0.011530	0.011732	0.011732	0.011597
0.011430	0.011497	0.011530	0.011934	0.011497	0.011531	0.011497
0.011530	0.011463	0.011463	0.011766	0.011699	0.011732	0.011463
0.011564	0.011631	0.011497	0.011564	0.011530	0.011396	0.011732
0.011497	0.011463	0.011463	0.011631	0.011463	0.011564	0.011799
0.011497	0.011876	0.011866	0.011711	0.011512	0.011570	0.011370
0.011500	0.011917	0.011411	0.011641	0.011499	0.011620	0.011645
0.011621	0.011455	0.011617	0.011457	0.011491	0.011663	0.011587
0.011588	0.011493	0.011553	0.011483	0.011465	0.011631	0.011698
0.011698	0.011565	0.011669	0.011670	0.011772	0.011767	0.011497
0.011732	0.011598	0.011463	0.011564	0.011362	0.011597	0.011396
0.011463	0.011463	0.011430	0.011396	0.011430	0.011396	0.011497
0.011479	0.011480	0.011471	0.011505	0.011642	0.011607	0.011607
0.011605	0.011531	0.011356	0.011348	0.011467	0.011636	0.011637
0.011430	0.011501	0.011734	0.011702	0.011632	0.011666	0.011597
0.011530	0.011598	0.011597	0.011497	0.012068	0.011665	0.011732
0.011766	0.011844	0.011470	0.011545	0.011430	0.011631	0.011598
0.011632	0.011665	0.011665	0.011665	0.011807	0.011799	0.011442
0.011331	0.011337	0.011435	0.011400	0.011505	0.011359	0.011672
0.011472	0.011630	0.011156	0.011472	0.011464	0.011539	0.011430
0.011396	0.011396	0.011463	0.011430	0.011631	0.011597	0.011530
0.011631	0.011698	0.011429	0.011429	0.011463	0.011463	0.011465
0.011533	0.011463	0.011463	0.011465	0.011631	0.011698	0.011698
0.011565	0.011669	0.011670	0.011772	0.011767	0.011497	0.011665
0.011598	0.011598	0.011432	0.011617	0.011547	0.011571	0.011305

0.011611	0.011652	0.011483	0.011502	0.011396	0.011598	0.011463
0.011564	0.011497	0.011396	0.011431	0.000000	0.011466	0.011497
0.011396	0.011263	0.011463	0.011396	0.011329	0.011434	0.011598
0.011497	0.011430	0.011463	0.011497	0.011463	0.011396	0.011597
0.011463	0.011497	0.011497	0.011430	0.011497	0.011732	0.011396
0.011430	0.011833	0.011430	0.011463	0.011430	0.011799	0.011446
0.011791	0.011443	0.011411	0.011664	0.011752	0.011634	0.011395
0.011375	0.011597	0.011698	0.011807	0.011666	0.011805	0.011935
0.011834	0.011872	0.011833	0.012086	0.011799	0.012674	0.011835
0.012574	0.012342	0.012476	0.012748	0.011799	0.011497	0.011799
0.012238	0.012174	0.011766	0.011665	0.011733	0.011733	0.011703
0.011597	0.011603	0.011665	0.011632	0.012002	0.012068	0.012069
0.011969	0.012069	0.012173	0.011940	0.012239	0.012246	0.012306
0.012307	0.012340	0.012289	0.012307	0.012308	0.011530	0.011833
0.011901	0.012405	0.011908	0.012136	0.012609	0.011672	0.011564
0.011530	0.011530	0.012001	0.012006	0.012116	0.011866	0.011532
S188	0.011804	0.011804	0.012143	0.011905	0.011905	0.011973
0.011813	0.011744	0.011905	0.011398	0.011466	0.011433	0.011398
0.011432	0.011601	0.011398	0.011432	0.011499	0.011466	0.011432
0.011398	0.011432	0.011533	0.011365	0.011432	0.011432	0.011778
0.011669	0.011432	0.011331	0.011567	0.011499	0.011466	0.011500
0.011466	0.011533	0.011466	0.011466	0.011499	0.011466	0.011635
0.011398	0.011466	0.011499	0.011432	0.011533	0.011567	0.011499
0.011466	0.011635	0.011534	0.011804	0.011466	0.011736	0.011466
0.011432	0.011533	0.011601	0.011736	0.011838	0.011533	0.011635
0.011499	0.011533	0.011466	0.011702	0.011939	0.011466	0.011533
0.011432	0.011533	0.011499	0.011567	0.011466	0.011499	0.011499
0.011398	0.011432	0.011398	0.011500	0.011466	0.011500	0.011567
0.011500	0.011466	0.011533	0.011432	0.011567	0.011398	0.011466
0.011635	0.011567	0.011804	0.011635	0.011466	0.011702	0.011466
0.011620	0.011849	0.011629	0.011335	0.011359	0.011393	0.011575
0.012203	0.011570	0.011392	0.011702	0.011702	0.011499	0.011601
0.011533	0.011499	0.011567	0.011466	0.011567	0.011499	0.011499
0.011466	0.011533	0.011736	0.011635	0.011533	0.011500	0.011432
0.011398	0.011398	0.011466	0.011432	0.011635	0.011601	0.011533
0.011635	0.011702	0.011432	0.011432	0.011466	0.011466	0.011468
0.011537	0.011466	0.011466	0.011314	0.011388	0.011378	0.011466
0.011469	0.011372	0.011432	0.011442	0.011368	0.011405	0.011434
0.011676	0.011442	0.011511	0.011399	0.011408	0.011534	0.011500
0.011568	0.011501	0.011500	0.011601	0.011533	0.011905	0.011702
0.011736	0.011601	0.011466	0.011567	0.011364	0.011601	0.011398
0.011466	0.011466	0.011432	0.011398	0.011432	0.011398	0.011499
0.011466	0.011567	0.011500	0.011398	0.011467	0.011466	0.000000
0.011601	0.011500	0.011398	0.011297	0.011466	0.011398	0.011331
0.011401	0.011601	0.011398	0.011601	0.011364	0.011466	0.011432
0.011533	0.011432	0.011466	0.011432	0.011466	0.011331	0.011466
0.011500	0.011432	0.011499	0.011432	0.011466	0.011499	0.011736
0.011838	0.011770	0.011838	0.011603	0.011838	0.011466	0.011432
0.011468	0.011670	0.011473	0.011302	0.011846	0.011335	0.011457
0.011846	0.011745	0.011635	0.011804	0.011541	0.011635	0.011669
0.011601	0.011770	0.011633	0.011434	0.011567	0.011500	0.011571
0.011669	0.011570	0.011707	0.011776	0.011702	0.011770	0.011879
0.011605	0.011778	0.011708	0.011639	0.011907	0.011364	0.011569
0.011533	0.011466	0.011364	0.011364	0.011432	0.011432	0.011499
0.011432	0.011500	0.011398	0.011499	0.011432	0.011432	0.011499
0.011432	0.011871	0.011770	0.011804	0.011331	0.011432	0.011466
0.011466	0.011432	0.011500	0.011533	0.011398	0.011398	0.011398
0.011939	0.011567	0.011567	0.011364	0.011466	0.011601	0.011398
0.011398	0.011466	0.011432	0.011601	0.011398	0.011533	0.011668
0.011601	0.011432	0.011432	0.011364	0.011364	0.011533	0.011466

0.011364	0.011466	0.011331	0.011364	0.011500	0.011635	0.011398
0.011365	0.011500	0.011432	0.011331	0.011669	0.011331	0.011500
0.011577	0.011398	0.011702	0.011534	0.011567	0.011432	0.011533
0.011398	0.011736	0.011331	0.011601	0.011398	0.011398	0.011331
0.011567	0.011770	0.011466	0.011533	0.011364	0.011770	0.011398
0.011398	0.011398	0.011432	0.011500	0.011432	0.011533	0.011601
0.011601	0.011567	0.011466	0.011567	0.011533	0.011398	0.011364
0.011398	0.011398	0.011466	0.011398	0.011364	0.011533	0.011398
0.011432	0.011432	0.011567	0.011601	0.011499	0.011432	0.011432
0.011466	0.011432	0.011533	0.011398	0.011533	0.011635	0.011939
0.011432	0.011838	0.011408	0.017940	0.011454	0.011673	0.011762
0.011756	0.011768	0.011480	0.011234	0.011886	0.011824	0.011547
0.011583	0.011733	0.011335	0.011440	0.011335	0.011456	0.011405
0.011577	0.011585	0.011583	0.011328	0.011567	0.011601	0.011605
0.011475	0.011567	0.011567	0.011533	0.011500	0.011500	0.011466
0.011499	0.011432	0.011466	0.011499	0.011466	0.011398	0.011432
0.011432	0.011499	0.011398	0.011500	0.011533	0.011567	0.011466
0.011499	0.011466	0.011804	0.011804	0.011534	0.011568	0.011601
0.011364	0.011364	0.011398	0.011500	0.011364	0.011601	0.011601
0.011635	0.011635	0.011567	0.011669	0.011838	0.011670	0.011500
0.011499	0.011567	0.011601	0.011432	0.011499	0.011466	0.011500
0.011567	0.011567	0.011601	0.011567	0.011433	0.011668	0.011635
0.011503	0.011398	0.011499	0.011601	0.011500	0.011635	0.011668
0.011804	0.011541	0.011635	0.011669	0.011601	0.011770	0.011398
0.011569	0.011466	0.011432	0.011432	0.011499	0.011398	0.011500
0.011533	0.011567	0.011466	0.011499	0.011466	0.011804	0.011804
0.011534	0.011568	0.011635	0.011567	0.012178	0.011636	0.011556
0.011627	0.011584	0.011662	0.011514	0.011695	0.011616	0.011581
0.012132	0.011478	0.011612	0.011513	0.011823	0.011677	0.011780
0.012156	0.011668	0.011503	0.011499	0.011499	0.011533	0.011468
0.011364	0.011398	0.011533	0.011499	0.011502	0.011635	0.011603
0.011466	0.011398	0.011432	0.011466	0.011364	0.011432	0.011331
0.011466	0.011499	0.011398	0.011364	0.011466	0.011398	0.011398
0.011432	0.011364	0.011432	0.011466	0.011364	0.011533	0.011601
0.011500	0.011567	0.011499	0.011500	0.011432	0.011838	0.011466
0.011516	0.011398	0.011669	0.011675	0.011533	0.011533	0.011533
0.011533	0.011466	0.011432	0.011466	0.011567	0.011567	0.011499
0.011499	0.011635	0.011804	0.011533	0.011736	0.011736	0.011601
0.011432	0.011499	0.011533	0.011939	0.011500	0.011533	0.011500
0.011499	0.011466	0.011466	0.011770	0.011703	0.011736	0.011466
0.011567	0.011635	0.011499	0.011567	0.011533	0.011398	0.011736
0.011432	0.011466	0.011466	0.011635	0.011466	0.011567	0.011804
0.011499	0.011881	0.011871	0.011671	0.011512	0.011539	0.011318
0.011445	0.011845	0.011378	0.011600	0.011500	0.011570	0.011539
0.011571	0.011390	0.011506	0.011391	0.011384	0.011597	0.011550
0.011553	0.011456	0.011521	0.011450	0.011501	0.011635	0.011702
0.011702	0.011568	0.011673	0.011674	0.011811	0.011806	0.011500
0.011736	0.011601	0.011466	0.011567	0.011364	0.011601	0.011398
0.011466	0.011466	0.011432	0.011398	0.011432	0.011398	0.011499
0.011482	0.011483	0.011474	0.011439	0.011646	0.011610	0.011610
0.011609	0.011500	0.011321	0.011314	0.011469	0.011640	0.011641
0.011433	0.011503	0.011738	0.011706	0.011636	0.011602	0.011601
0.011533	0.011601	0.011567	0.011499	0.012075	0.011635	0.011736
0.011770	0.011849	0.011472	0.011548	0.011432	0.011635	0.011601
0.011602	0.011668	0.011669	0.011669	0.011811	0.011804	0.011444
0.011333	0.011339	0.011437	0.011402	0.011507	0.011361	0.011676
0.011475	0.011635	0.011349	0.011513	0.011466	0.011542	0.011432
0.011398	0.011398	0.011466	0.011432	0.011635	0.011601	0.011533
0.011635	0.011702	0.011432	0.011432	0.011466	0.011466	0.011468
0.011537	0.011466	0.011466	0.011501	0.011635	0.011702	0.011702

0.011568	0.011673	0.011674	0.011811	0.011806	0.011500	0.011668
0.011601	0.011601	0.011435	0.011621	0.011550	0.011540	0.011378
0.011580	0.011700	0.011527	0.011505	0.011398	0.011601	0.011466
0.011567	0.011500	0.011398	0.011467	0.011466	0.000000	0.011500
0.011398	0.011297	0.011466	0.011398	0.011331	0.011401	0.011601
0.011499	0.011432	0.011466	0.011499	0.011466	0.011398	0.011601
0.011466	0.011500	0.011499	0.011432	0.011499	0.011736	0.011398
0.011432	0.011838	0.011432	0.011466	0.011432	0.011804	0.011404
0.011848	0.011401	0.011361	0.011627	0.011712	0.011602	0.011353
0.011323	0.011601	0.011669	0.011778	0.011636	0.011775	0.011906
0.011805	0.011843	0.011804	0.012092	0.011770	0.012685	0.011840
0.012584	0.012350	0.012485	0.012758	0.011804	0.011500	0.011804
0.012246	0.012181	0.011770	0.011669	0.011737	0.011737	0.011707
0.011601	0.011606	0.011668	0.011635	0.012008	0.012075	0.012076
0.011975	0.012042	0.012180	0.011946	0.012212	0.012219	0.012280
0.012281	0.012314	0.012262	0.012281	0.012248	0.011533	0.011804
0.011873	0.012414	0.011913	0.012143	0.012619	0.011675	0.011568
0.011533	0.011533	0.012007	0.012054	0.012087	0.011871	0.011535
S189	0.011799	0.011799	0.012136	0.011900	0.011900	0.011967
0.011808	0.011740	0.011900	0.011396	0.011463	0.011430	0.011396
0.011430	0.011598	0.011396	0.011430	0.011497	0.011463	0.011430
0.011396	0.011430	0.011530	0.011396	0.011363	0.011430	0.011774
0.011665	0.011396	0.011329	0.011564	0.011430	0.011463	0.011430
0.011463	0.011497	0.011463	0.011463	0.011497	0.011463	0.011631
0.011396	0.011463	0.011497	0.011430	0.011530	0.011564	0.011497
0.011463	0.011631	0.011531	0.011799	0.011463	0.011665	0.011463
0.011430	0.011531	0.011598	0.011665	0.011833	0.011530	0.011631
0.011430	0.011497	0.011463	0.011631	0.011934	0.011463	0.011530
0.011430	0.011530	0.011497	0.011564	0.011497	0.011497	0.011497
0.011396	0.011430	0.011396	0.011497	0.011463	0.011498	0.011564
0.011497	0.011463	0.011396	0.011430	0.011564	0.011329	0.011463
0.011631	0.011564	0.011799	0.011631	0.011463	0.011631	0.011463
0.011654	0.011882	0.011664	0.011373	0.011359	0.011502	0.011572
0.012231	0.011620	0.011390	0.011698	0.011698	0.011497	0.011598
0.011530	0.011497	0.011564	0.011463	0.011564	0.011430	0.011497
0.011463	0.011564	0.011766	0.011631	0.011530	0.011497	0.011362
0.011396	0.011396	0.011329	0.011430	0.011631	0.011598	0.011530
0.011631	0.011698	0.011430	0.011430	0.011463	0.011463	0.011466
0.011534	0.011463	0.011463	0.011312	0.011386	0.011376	0.011464
0.011467	0.011404	0.011429	0.011440	0.011366	0.011403	0.011432
0.011708	0.011474	0.011439	0.011396	0.011406	0.011531	0.011498
0.011565	0.011498	0.011497	0.011597	0.011530	0.011833	0.011699
0.011732	0.011598	0.011463	0.011564	0.011362	0.011597	0.011396
0.011463	0.011463	0.011430	0.011396	0.011430	0.011396	0.011497
0.011396	0.011497	0.011497	0.011396	0.011498	0.011598	0.011601
0.000000	0.011497	0.011396	0.011330	0.011463	0.011396	0.011329
0.011434	0.000000	0.011329	0.000000	0.011363	0.011463	0.011430
0.011463	0.011430	0.011396	0.011430	0.011463	0.011329	0.011396
0.011430	0.011430	0.011497	0.011430	0.011463	0.011497	0.011732
0.011833	0.011765	0.011833	0.011600	0.011833	0.011463	0.011430
0.011466	0.011667	0.011563	0.011369	0.011807	0.011333	0.011454
0.011842	0.011740	0.011631	0.011833	0.011538	0.011699	0.011598
0.011598	0.011765	0.011629	0.011514	0.011564	0.011497	0.011568
0.011665	0.011566	0.011703	0.011703	0.011699	0.011766	0.011874
0.011602	0.011705	0.011704	0.011635	0.011902	0.011362	0.011566
0.011463	0.011463	0.011362	0.011362	0.011362	0.011430	0.011497
0.011362	0.011430	0.011329	0.011497	0.011362	0.011430	0.011497
0.011430	0.011866	0.011766	0.011799	0.011329	0.011430	0.011463
0.011463	0.011430	0.011497	0.011530	0.011396	0.011396	0.011396
0.011934	0.011564	0.011564	0.011362	0.011463	0.011564	0.011396

0.011396	0.011463	0.011430	0.011530	0.011396	0.011530	0.011597
0.011598	0.011430	0.011430	0.011362	0.011362	0.011531	0.011463
0.011362	0.011463	0.011329	0.011362	0.011497	0.011632	0.011396
0.011363	0.011497	0.011430	0.011329	0.011665	0.011329	0.011497
0.011506	0.011396	0.011698	0.011531	0.011564	0.011430	0.011530
0.011396	0.011665	0.011329	0.011598	0.011396	0.011396	0.011329
0.011497	0.011766	0.011463	0.011530	0.011362	0.011766	0.011396
0.011396	0.011396	0.011430	0.011463	0.011430	0.011530	0.011598
0.011598	0.011564	0.011463	0.011564	0.011463	0.011396	0.011362
0.011396	0.011396	0.011463	0.011396	0.011295	0.011530	0.011396
0.011430	0.011430	0.011564	0.011598	0.011497	0.011362	0.011430
0.011497	0.011430	0.011530	0.011396	0.011530	0.011631	0.011934
0.011430	0.011833	0.011440	0.017974	0.011322	0.011733	0.011793
0.011752	0.011799	0.011479	0.011408	0.011829	0.011820	0.011546
0.011629	0.011732	0.011294	0.011437	0.011333	0.011505	0.011491
0.011670	0.011582	0.011579	0.011193	0.011564	0.011598	0.011601
0.011404	0.011497	0.011564	0.011530	0.011497	0.011497	0.011463
0.011497	0.011430	0.011463	0.011497	0.011463	0.011396	0.011430
0.011430	0.011497	0.011396	0.011497	0.011530	0.011564	0.011463
0.011497	0.011463	0.011799	0.011799	0.011531	0.011565	0.011598
0.011362	0.011362	0.011396	0.011430	0.011362	0.011598	0.011598
0.011631	0.011631	0.011564	0.011665	0.011833	0.011667	0.011430
0.011497	0.011564	0.011530	0.011430	0.011497	0.011463	0.011497
0.011564	0.011564	0.011598	0.011497	0.011397	0.011665	0.011564
0.011501	0.011396	0.011497	0.011598	0.011497	0.011631	0.011665
0.011833	0.011538	0.011699	0.011598	0.011598	0.011765	0.011396
0.011566	0.011464	0.011430	0.011430	0.011497	0.011396	0.011497
0.011530	0.011564	0.011463	0.011497	0.011463	0.011799	0.011799
0.011531	0.011565	0.011631	0.011564	0.012205	0.011632	0.011553
0.011623	0.011581	0.011693	0.011580	0.011691	0.011612	0.011578
0.012160	0.011476	0.011608	0.011545	0.011818	0.011673	0.011707
0.012183	0.011663	0.011500	0.011497	0.011497	0.011530	0.011466
0.011362	0.011329	0.011530	0.011497	0.011500	0.011631	0.011599
0.011463	0.011329	0.011362	0.011463	0.011295	0.011430	0.011329
0.011463	0.011497	0.011396	0.011362	0.011463	0.011396	0.011430
0.011430	0.011362	0.011430	0.011463	0.011362	0.011497	0.011598
0.011497	0.011564	0.011497	0.011497	0.011430	0.011833	0.011463
0.011513	0.011430	0.011665	0.011671	0.011463	0.011530	0.011530
0.011531	0.011396	0.011430	0.011396	0.011564	0.011564	0.011497
0.011497	0.011631	0.011799	0.011530	0.011732	0.011732	0.011598
0.011430	0.011497	0.011531	0.011934	0.011497	0.011463	0.011497
0.011530	0.011463	0.011463	0.011766	0.011631	0.011732	0.011463
0.011564	0.011631	0.011497	0.011564	0.011530	0.011396	0.011732
0.011497	0.011463	0.011463	0.011631	0.011463	0.011564	0.011799
0.011497	0.011876	0.011866	0.011670	0.011342	0.011570	0.011318
0.011555	0.011878	0.011411	0.011596	0.011402	0.011518	0.011485
0.011570	0.011324	0.011394	0.011258	0.011384	0.011462	0.011466
0.011475	0.011336	0.011553	0.011448	0.011498	0.011631	0.011698
0.011698	0.011565	0.011669	0.011670	0.011806	0.011838	0.011497
0.011732	0.011598	0.011463	0.011564	0.011362	0.011597	0.011396
0.011463	0.011463	0.011430	0.011396	0.011430	0.011396	0.011497
0.011479	0.011480	0.011471	0.011505	0.011642	0.011538	0.011607
0.011605	0.011531	0.011319	0.011348	0.011467	0.011636	0.011637
0.011430	0.011501	0.011734	0.011703	0.011632	0.011666	0.011597
0.011530	0.011598	0.011597	0.011497	0.012069	0.011665	0.011732
0.011766	0.011808	0.011470	0.011545	0.011430	0.011631	0.011598
0.011632	0.011665	0.011665	0.011665	0.011807	0.011799	0.011442
0.011331	0.011337	0.011435	0.011400	0.011505	0.011359	0.011672
0.011472	0.011586	0.011346	0.011510	0.011464	0.011539	0.011362
0.011396	0.011396	0.011329	0.011430	0.011631	0.011598	0.011530

0.011631	0.011698	0.011430	0.011430	0.011463	0.011463	0.011466
0.011534	0.011463	0.011463	0.011498	0.011631	0.011698	0.011698
0.011565	0.011669	0.011670	0.011806	0.011838	0.011497	0.011665
0.011598	0.011598	0.011432	0.011617	0.011547	0.011571	0.011446
0.011542	0.011835	0.011568	0.011502	0.011329	0.000000	0.011396
0.011497	0.011497	0.011396	0.011498	0.011598	0.011601	0.011497
0.011396	0.011330	0.011463	0.011396	0.011329	0.011434	0.000000
0.011497	0.011430	0.011463	0.011497	0.011463	0.011396	0.011597
0.011463	0.011497	0.011497	0.011430	0.011497	0.011732	0.011396
0.011430	0.011833	0.011430	0.011396	0.011430	0.011799	0.011403
0.011847	0.011400	0.011461	0.011624	0.011712	0.011635	0.011353
0.011323	0.011597	0.011698	0.011807	0.011666	0.011805	0.011935
0.011834	0.011872	0.011833	0.012086	0.011799	0.012675	0.011835
0.012574	0.012342	0.012476	0.012748	0.011799	0.011497	0.011800
0.012239	0.012174	0.011766	0.011665	0.011733	0.011733	0.011703
0.011597	0.011603	0.011665	0.011632	0.012002	0.012068	0.012070
0.011969	0.012069	0.012173	0.011940	0.012239	0.012246	0.012306
0.012307	0.012341	0.012289	0.012307	0.012309	0.011530	0.011833
0.011901	0.012405	0.011908	0.012136	0.012609	0.011672	0.011564
0.011530	0.011530	0.012002	0.012048	0.012151	0.011866	0.011532
S190	0.011699	0.011699	0.012035	0.011799	0.011799	0.011867
0.011672	0.011570	0.011799	0.011295	0.011362	0.011329	0.011295
0.011329	0.011430	0.011295	0.011329	0.011396	0.011362	0.011329
0.011295	0.011329	0.011362	0.011296	0.011329	0.011329	0.011638
0.011564	0.011329	0.011228	0.011396	0.011396	0.011363	0.011396
0.011362	0.011463	0.011362	0.011362	0.011396	0.011362	0.011530
0.011295	0.011362	0.011396	0.011329	0.011430	0.011463	0.011396
0.011362	0.011531	0.011430	0.011698	0.011362	0.011631	0.011362
0.011329	0.011430	0.011497	0.011631	0.011732	0.011430	0.011530
0.011396	0.011463	0.011363	0.011598	0.011833	0.011362	0.011430
0.011329	0.011430	0.011396	0.011463	0.011396	0.011396	0.011396
0.011295	0.011329	0.011295	0.011396	0.011362	0.011397	0.011463
0.011396	0.011362	0.011430	0.011329	0.011463	0.011295	0.011362
0.011530	0.011463	0.011698	0.011531	0.011362	0.011598	0.011362
0.011542	0.011770	0.011588	0.011295	0.011284	0.011355	0.011493
0.012121	0.011518	0.011307	0.011598	0.011598	0.011396	0.011497
0.011430	0.011396	0.011463	0.011362	0.011463	0.011396	0.011396
0.011362	0.011463	0.011665	0.011530	0.011430	0.011396	0.011329
0.011295	0.011295	0.011362	0.011329	0.011530	0.011497	0.011430
0.011530	0.011598	0.011329	0.011329	0.011363	0.011362	0.011364
0.011432	0.011362	0.011362	0.011240	0.011313	0.011271	0.011363
0.011365	0.011301	0.011355	0.011336	0.011264	0.011300	0.011330
0.011600	0.011370	0.011405	0.011296	0.011302	0.011430	0.011396
0.011464	0.011397	0.011396	0.011497	0.011430	0.011799	0.011598
0.011631	0.011497	0.011362	0.011463	0.011262	0.011497	0.011295
0.011362	0.011362	0.011329	0.011295	0.011329	0.011295	0.011396
0.011362	0.011463	0.011396	0.011295	0.011364	0.011497	0.011500
0.011497	0.000000	0.011295	0.011229	0.011362	0.011295	0.011228
0.011332	0.011497	0.011295	0.011497	0.011262	0.011363	0.011329
0.011430	0.011329	0.011362	0.011329	0.011362	0.011228	0.011362
0.011396	0.011329	0.011396	0.011329	0.011362	0.011396	0.011631
0.011732	0.011665	0.011732	0.011498	0.011732	0.011362	0.011329
0.011364	0.011626	0.011472	0.011266	0.011739	0.011231	0.011336
0.011706	0.011638	0.011530	0.011732	0.011436	0.011598	0.011564
0.011497	0.011665	0.011522	0.011349	0.011396	0.011396	0.011466
0.011564	0.011465	0.011602	0.011670	0.011598	0.011665	0.011772
0.011500	0.011672	0.011602	0.011534	0.011801	0.011262	0.011465
0.011430	0.011362	0.011262	0.011262	0.011329	0.011329	0.011396
0.011329	0.011396	0.011295	0.011396	0.011329	0.011329	0.011396
0.011329	0.011766	0.011665	0.011698	0.011228	0.011329	0.011363

0.011362	0.011329	0.011396	0.011430	0.011295	0.011295	0.011295
0.011833	0.011463	0.011463	0.011262	0.011362	0.011531	0.011295
0.011295	0.011362	0.011329	0.011497	0.011295	0.011430	0.011564
0.011497	0.011329	0.011329	0.011262	0.011262	0.011430	0.011362
0.011262	0.011362	0.011228	0.011262	0.011396	0.011531	0.011295
0.011262	0.011396	0.011329	0.011228	0.011564	0.011228	0.011396
0.011471	0.011295	0.011598	0.011430	0.011463	0.011329	0.011430
0.011295	0.011631	0.011228	0.011497	0.011295	0.011295	0.011228
0.011463	0.011665	0.011363	0.011430	0.011262	0.011665	0.011295
0.011295	0.011295	0.011329	0.011430	0.011329	0.011430	0.011497
0.011497	0.011463	0.011362	0.011463	0.011430	0.011295	0.011262
0.011295	0.011295	0.011363	0.011295	0.011262	0.011430	0.011295
0.011329	0.011329	0.011463	0.011497	0.011396	0.011329	0.011329
0.011396	0.011329	0.011430	0.011295	0.011430	0.011530	0.011766
0.011329	0.011732	0.011336	0.017861	0.011322	0.011549	0.011688
0.011648	0.011693	0.011322	0.011350	0.011829	0.011716	0.011485
0.011535	0.011671	0.011255	0.011353	0.011255	0.011406	0.011361
0.011576	0.011507	0.011505	0.011193	0.011463	0.011497	0.011500
0.011370	0.011463	0.011463	0.011430	0.011195	0.011195	0.011363
0.011396	0.011329	0.011362	0.011396	0.011363	0.011295	0.011329
0.011329	0.011396	0.011295	0.011396	0.011430	0.011463	0.011362
0.011396	0.011362	0.011698	0.011698	0.011430	0.011464	0.011497
0.011262	0.011262	0.011295	0.011396	0.011262	0.011497	0.011497
0.011530	0.011530	0.011463	0.011564	0.011732	0.011566	0.011396
0.011396	0.011463	0.011497	0.011329	0.011396	0.011362	0.011396
0.011463	0.011463	0.011497	0.011463	0.011363	0.011564	0.011530
0.011399	0.011295	0.011396	0.011497	0.011396	0.011531	0.011564
0.011732	0.011436	0.011598	0.011564	0.011497	0.011665	0.011296
0.011464	0.011363	0.011329	0.011329	0.011396	0.011295	0.011396
0.011430	0.011463	0.011362	0.011396	0.011362	0.011698	0.011698
0.011430	0.011464	0.011530	0.011463	0.012104	0.011525	0.011447
0.011517	0.011477	0.011588	0.011476	0.011586	0.011509	0.011474
0.012057	0.011372	0.011506	0.011441	0.011715	0.011571	0.011673
0.012081	0.011550	0.011399	0.011396	0.011396	0.011430	0.011364
0.011262	0.011295	0.011430	0.011396	0.011398	0.011530	0.011498
0.011362	0.011295	0.011329	0.011362	0.011262	0.011329	0.011228
0.011362	0.011396	0.011295	0.011262	0.011363	0.011295	0.011329
0.011329	0.011262	0.011329	0.011363	0.011262	0.011463	0.011497
0.011396	0.011463	0.011396	0.011396	0.011329	0.011732	0.011362
0.011408	0.011329	0.011564	0.011569	0.011430	0.011430	0.011430
0.011430	0.011363	0.011329	0.011363	0.011463	0.011463	0.011396
0.011396	0.011530	0.011698	0.011430	0.011631	0.011631	0.011497
0.011329	0.011396	0.011430	0.011833	0.011396	0.011430	0.011396
0.011430	0.011362	0.011362	0.011665	0.011598	0.011631	0.011362
0.011463	0.011530	0.011396	0.011463	0.011430	0.011295	0.011631
0.011396	0.011362	0.011362	0.011530	0.011362	0.011463	0.011699
0.011396	0.011740	0.011766	0.011588	0.011286	0.011468	0.011267
0.011335	0.011800	0.011306	0.011551	0.011402	0.011467	0.011485
0.011519	0.011258	0.011394	0.011192	0.011384	0.011462	0.011466
0.011475	0.011375	0.011448	0.011377	0.011397	0.011530	0.011598
0.011530	0.011464	0.011567	0.011569	0.011705	0.011732	0.011362
0.011631	0.011497	0.011362	0.011463	0.011262	0.011497	0.011295
0.011362	0.011362	0.011329	0.011295	0.011329	0.011295	0.011396
0.011375	0.011375	0.011368	0.011403	0.011539	0.011504	0.011504
0.011503	0.011430	0.011245	0.011240	0.011365	0.011535	0.011535
0.011329	0.011399	0.011632	0.011601	0.011531	0.011565	0.011497
0.011430	0.011497	0.011497	0.011396	0.011968	0.011564	0.011631
0.011665	0.011737	0.011367	0.011441	0.011329	0.011530	0.011497
0.011531	0.011564	0.011564	0.011564	0.011728	0.011698	0.011338
0.011229	0.011233	0.011333	0.011298	0.011402	0.011247	0.011570

0.011369	0.011499	0.011251	0.011360	0.011363	0.011437	0.011329
0.011295	0.011295	0.011362	0.011329	0.011530	0.011497	0.011430
0.011530	0.011598	0.011329	0.011329	0.011363	0.011362	0.011364
0.011432	0.011362	0.011362	0.011397	0.011530	0.011598	0.011530
0.011464	0.011567	0.011569	0.011705	0.011732	0.011362	0.011564
0.011497	0.011497	0.011331	0.011513	0.011442	0.011469	0.011340
0.011473	0.011698	0.011483	0.011400	0.011295	0.011497	0.011362
0.011463	0.011396	0.011295	0.011364	0.011497	0.011500	0.000000
0.011295	0.011229	0.011362	0.011295	0.011228	0.011332	0.011497
0.011396	0.011329	0.011362	0.011396	0.011363	0.011295	0.011497
0.011362	0.011396	0.011396	0.011329	0.011396	0.011631	0.011295
0.011329	0.011732	0.011329	0.011362	0.011329	0.011698	0.011317
0.011679	0.011315	0.011361	0.011543	0.011630	0.011527	0.011269
0.011270	0.011497	0.011598	0.011705	0.011565	0.011703	0.011834
0.011733	0.011771	0.011732	0.011983	0.011698	0.012574	0.011734
0.012473	0.012241	0.012375	0.012646	0.011698	0.011396	0.011698
0.012137	0.012072	0.011665	0.011564	0.011632	0.011632	0.011602
0.011497	0.011501	0.011564	0.011531	0.011901	0.011968	0.011968
0.011868	0.011968	0.012072	0.011838	0.012138	0.012144	0.012205
0.012205	0.012239	0.012186	0.012205	0.012207	0.011430	0.011732
0.011800	0.012304	0.011806	0.012035	0.012508	0.011570	0.011464
0.011430	0.011430	0.011901	0.011965	0.012045	0.011766	0.011431
S191 0.011597	0.011597	0.011934	0.011698	0.011698	0.011765	
0.011604	0.011536	0.011698	0.011195	0.011262	0.011228	0.011195
0.011228	0.011396	0.011195	0.011228	0.011295	0.011262	0.011228
0.011195	0.011228	0.011329	0.011195	0.011228	0.011228	0.011570
0.011463	0.011228	0.011127	0.011362	0.011295	0.011262	0.011295
0.011262	0.011362	0.011262	0.011262	0.011295	0.011262	0.011430
0.011195	0.011262	0.011295	0.011228	0.011329	0.011362	0.011295
0.011262	0.011430	0.011329	0.011597	0.011262	0.011530	0.011262
0.011228	0.011329	0.011396	0.011530	0.011631	0.011329	0.011429
0.011295	0.011362	0.011262	0.011497	0.011732	0.011262	0.011329
0.011228	0.011329	0.011295	0.011362	0.011295	0.011295	0.011295
0.011195	0.011228	0.011195	0.011295	0.011262	0.011296	0.011362
0.011295	0.011262	0.011329	0.011228	0.011362	0.011195	0.011262
0.011429	0.011362	0.011597	0.011430	0.011262	0.011497	0.011262
0.011431	0.011658	0.011437	0.011138	0.011098	0.011281	0.011375
0.012011	0.011365	0.011143	0.011497	0.011497	0.011295	0.011396
0.011329	0.011295	0.011362	0.011262	0.011362	0.011295	0.011295
0.011262	0.011362	0.011564	0.011429	0.011329	0.011295	0.011228
0.011195	0.011195	0.011262	0.011228	0.011429	0.011396	0.011329
0.011429	0.011497	0.011228	0.011228	0.011262	0.011262	0.011263
0.011331	0.011262	0.011262	0.011132	0.011205	0.011165	0.011262
0.011263	0.011198	0.011208	0.011233	0.011162	0.011197	0.011229
0.011492	0.011267	0.011301	0.011195	0.011199	0.011329	0.011296
0.011363	0.011296	0.011296	0.011396	0.011329	0.011698	0.011497
0.011530	0.011396	0.011262	0.011362	0.011161	0.011396	0.011195
0.011262	0.011262	0.011228	0.011195	0.011228	0.011195	0.011295
0.011262	0.011362	0.011295	0.011195	0.011296	0.011396	0.011398
0.011396	0.011295	0.000000	0.011128	0.011262	0.000000	0.011127
0.011230	0.011396	0.011195	0.011396	0.011161	0.011262	0.011228
0.011329	0.011228	0.011262	0.011228	0.011262	0.011127	0.011262
0.011295	0.011228	0.011295	0.011228	0.011262	0.011295	0.011530
0.011631	0.011564	0.011631	0.011397	0.011631	0.011262	0.011228
0.011263	0.011505	0.011289	0.011163	0.011637	0.011129	0.011218
0.011637	0.011536	0.011429	0.011631	0.011333	0.011497	0.011463
0.011396	0.011564	0.011416	0.011266	0.011270	0.011295	0.011365
0.011463	0.011364	0.011500	0.011568	0.011497	0.011564	0.011670
0.011399	0.011569	0.011500	0.011432	0.011700	0.011161	0.011364
0.011329	0.011262	0.011161	0.011161	0.011228	0.011228	0.011295

0.011228	0.011295	0.011195	0.011295	0.011228	0.011228	0.011295
0.011228	0.011665	0.011564	0.011597	0.011127	0.011228	0.011262
0.011262	0.011228	0.011295	0.011329	0.011195	0.011195	0.011195
0.011732	0.011362	0.011362	0.011161	0.011262	0.011430	0.011195
0.011195	0.011262	0.011228	0.011396	0.011195	0.011329	0.011463
0.011396	0.011228	0.011228	0.011161	0.011161	0.011262	0.011262
0.011161	0.011262	0.011127	0.011161	0.011295	0.011430	0.011195
0.011161	0.011295	0.011228	0.011127	0.011463	0.011127	0.011295
0.011368	0.011195	0.011497	0.011329	0.011362	0.011228	0.011329
0.011195	0.011530	0.011127	0.011396	0.011195	0.011195	0.011127
0.011362	0.011564	0.011262	0.011329	0.011161	0.011564	0.011195
0.011195	0.011195	0.011228	0.011329	0.011228	0.011329	0.011396
0.011396	0.011362	0.011262	0.011362	0.011329	0.011195	0.011161
0.011195	0.011195	0.011262	0.011195	0.011161	0.011329	0.011195
0.011228	0.011228	0.011362	0.011396	0.011295	0.011228	0.011228
0.011295	0.011228	0.011329	0.011195	0.011329	0.011429	0.011732
0.011228	0.011631	0.011233	0.017747	0.011126	0.011305	0.011583
0.011545	0.011588	0.011217	0.011176	0.011609	0.011612	0.011242
0.011392	0.011426	0.011138	0.011186	0.011216	0.011208	0.011146
0.011389	0.011395	0.011357	0.011259	0.011362	0.011396	0.011398
0.011266	0.011362	0.011362	0.011329	0.011295	0.011295	0.011262
0.011295	0.011228	0.011262	0.011295	0.011262	0.011195	0.011228
0.011228	0.011295	0.011195	0.011295	0.011329	0.011362	0.011262
0.011295	0.011262	0.011597	0.011597	0.011329	0.011363	0.011396
0.011161	0.011161	0.011195	0.011295	0.011161	0.011396	0.011396
0.011429	0.011429	0.011362	0.011463	0.011631	0.011464	0.011295
0.011295	0.011362	0.011396	0.011228	0.011295	0.011262	0.011295
0.011363	0.011362	0.011396	0.011363	0.011262	0.011463	0.011429
0.011297	0.011195	0.011295	0.011396	0.011295	0.011430	0.011463
0.011631	0.011333	0.011497	0.011463	0.011396	0.011564	0.011195
0.011363	0.011262	0.011228	0.011228	0.011295	0.011195	0.011295
0.011329	0.011362	0.011262	0.011295	0.011262	0.011597	0.011597
0.011329	0.011363	0.011429	0.011362	0.012002	0.011417	0.011342
0.011412	0.011372	0.011482	0.011372	0.011480	0.011405	0.011371
0.011953	0.011268	0.011403	0.011337	0.011611	0.011468	0.011571
0.011978	0.011437	0.011297	0.011295	0.011295	0.011329	0.011263
0.011161	0.011195	0.011329	0.011295	0.011297	0.011429	0.011397
0.011262	0.011195	0.011228	0.011262	0.011161	0.011228	0.011127
0.011262	0.011295	0.011195	0.011161	0.011262	0.011195	0.011228
0.011228	0.011161	0.011228	0.011262	0.011161	0.011362	0.011396
0.011295	0.011362	0.011295	0.011295	0.011228	0.011631	0.011262
0.011304	0.011228	0.011463	0.011467	0.011329	0.011329	0.011329
0.011329	0.011262	0.011228	0.011262	0.011362	0.011362	0.011295
0.011295	0.011430	0.011597	0.011329	0.011530	0.011530	0.011396
0.011228	0.011295	0.011329	0.011732	0.011295	0.011329	0.011295
0.011329	0.011262	0.011262	0.011564	0.011497	0.011530	0.011262
0.011362	0.011430	0.011295	0.011362	0.011329	0.011195	0.011530
0.011295	0.011262	0.011262	0.011430	0.011262	0.011362	0.011597
0.011295	0.011672	0.011665	0.011426	0.011117	0.011366	0.011163
0.011115	0.011683	0.011201	0.011373	0.011158	0.011264	0.011273
0.011366	0.000000	0.011227	0.000000	0.011114	0.011261	0.011344
0.011362	0.011257	0.011342	0.011272	0.011296	0.011429	0.011497
0.011497	0.011363	0.011466	0.011467	0.011603	0.011625	0.011295
0.011530	0.011396	0.011262	0.011362	0.011161	0.011396	0.011195
0.011262	0.011262	0.011228	0.011195	0.011228	0.011195	0.011295
0.011270	0.011270	0.011266	0.011300	0.011436	0.011402	0.011402
0.011401	0.011329	0.011134	0.011132	0.011263	0.011433	0.011433
0.011228	0.011297	0.011531	0.011500	0.011430	0.011464	0.011396
0.011329	0.011396	0.011396	0.011295	0.011866	0.011463	0.011530
0.011564	0.011630	0.011265	0.011337	0.011228	0.011430	0.011396

0.011430	0.011463	0.011463	0.011463	0.011571	0.011597	0.011234
0.011128	0.011130	0.011231	0.011196	0.011299	0.011135	0.011468
0.011266	0.011367	0.011108	0.011285	0.011262	0.011334	0.011228
0.011195	0.011195	0.011262	0.011228	0.011429	0.011396	0.011329
0.011429	0.011497	0.011228	0.011228	0.011262	0.011262	0.011263
0.011331	0.011262	0.011262	0.011296	0.011429	0.011497	0.011497
0.011363	0.011466	0.011467	0.011603	0.011625	0.011295	0.011463
0.011396	0.011396	0.011229	0.011408	0.011338	0.011367	0.011235
0.011370	0.011607	0.011399	0.011298	0.011195	0.011396	0.011262
0.011362	0.011295	0.011195	0.011296	0.011396	0.011398	0.011295
0.000000	0.011128	0.011262	0.000000	0.011127	0.011230	0.011396
0.011295	0.011228	0.011262	0.011295	0.011262	0.011195	0.011396
0.011262	0.011295	0.011295	0.011228	0.011295	0.011530	0.011195
0.011228	0.011631	0.011228	0.011262	0.011228	0.011597	0.011103
0.011566	0.011145	0.011161	0.011422	0.011467	0.011419	0.000000
0.011113	0.011396	0.011497	0.011603	0.011464	0.011601	0.011733
0.011632	0.011669	0.011631	0.011880	0.011597	0.012472	0.011633
0.012372	0.012139	0.012273	0.012545	0.011597	0.011295	0.011598
0.012036	0.011971	0.011564	0.011463	0.011531	0.011531	0.011500
0.011396	0.011399	0.011463	0.011430	0.011800	0.011866	0.011867
0.011767	0.011867	0.011970	0.011737	0.012036	0.012042	0.012103
0.012104	0.012138	0.012084	0.012104	0.012105	0.011329	0.011631
0.011699	0.012203	0.011704	0.011934	0.012406	0.011468	0.011363
0.011329	0.011329	0.011799	0.011842	0.011940	0.011665	0.011330
S192	0.011532	0.011532	0.011870	0.011633	0.011633	0.011701
0.011538	0.011470	0.011633	0.011128	0.011195	0.011162	0.011128
0.011161	0.011330	0.011128	0.011161	0.011229	0.011195	0.011161
0.011128	0.011161	0.011263	0.011128	0.011162	0.011161	0.011504
0.011397	0.011161	0.000000	0.011296	0.011229	0.011195	0.011229
0.011195	0.011296	0.011195	0.011195	0.011229	0.011195	0.011364
0.011128	0.011195	0.011229	0.011161	0.011263	0.011296	0.011229
0.011195	0.011364	0.011263	0.011532	0.011195	0.011465	0.011195
0.011161	0.011263	0.011330	0.011465	0.011566	0.011263	0.011364
0.011229	0.011296	0.011195	0.011431	0.011667	0.011195	0.011263
0.011161	0.011263	0.011229	0.011296	0.011195	0.011229	0.011229
0.011128	0.011161	0.011128	0.011229	0.011195	0.011229	0.011296
0.011229	0.011195	0.011263	0.011161	0.011296	0.011128	0.011195
0.011364	0.011296	0.011532	0.011364	0.011195	0.011431	0.011195
0.011358	0.011586	0.011401	0.011100	0.011098	0.011208	0.011298
0.011942	0.011365	0.011102	0.011431	0.011431	0.011229	0.011330
0.011263	0.011229	0.011296	0.011195	0.011296	0.011229	0.011229
0.011195	0.011263	0.011499	0.011364	0.011263	0.011229	0.011161
0.011128	0.011128	0.011195	0.011161	0.011364	0.011330	0.011263
0.011364	0.011364	0.011161	0.011161	0.011195	0.011195	0.011196
0.011264	0.011195	0.011195	0.000000	0.011133	0.011096	0.011195
0.011196	0.011129	0.011171	0.011164	0.011094	0.011129	0.011162
0.011422	0.011199	0.011233	0.011128	0.011130	0.011263	0.011229
0.011297	0.011229	0.011229	0.011330	0.011263	0.011634	0.011431
0.011465	0.011330	0.011195	0.011296	0.011094	0.011330	0.011128
0.011195	0.011195	0.011161	0.011128	0.011161	0.011128	0.011229
0.011195	0.011296	0.011229	0.011128	0.011229	0.011263	0.011297
0.011330	0.011229	0.011128	0.000000	0.011195	0.011128	0.000000
0.011162	0.011330	0.011128	0.011330	0.011094	0.011195	0.011161
0.011263	0.011161	0.011195	0.011161	0.011195	0.000000	0.011195
0.011229	0.011161	0.011229	0.011161	0.011195	0.011229	0.011465
0.011566	0.011498	0.011566	0.011331	0.011566	0.011195	0.011161
0.011196	0.011465	0.011289	0.011095	0.011571	0.000000	0.011179
0.011572	0.011470	0.011364	0.011566	0.011266	0.011431	0.011398
0.011330	0.011498	0.011346	0.011185	0.011229	0.011229	0.011298
0.011397	0.011297	0.011434	0.011502	0.011431	0.011499	0.011605

0.011332	0.011503	0.011434	0.011366	0.011635	0.011094	0.011297
0.011263	0.011195	0.011094	0.011094	0.011161	0.011161	0.011229
0.011161	0.011229	0.011128	0.011229	0.011161	0.011161	0.011229
0.011161	0.011600	0.011499	0.011532	0.000000	0.011161	0.011195
0.011195	0.011161	0.011229	0.011263	0.011128	0.011128	0.011128
0.011667	0.011296	0.011296	0.011094	0.011195	0.011364	0.011128
0.011128	0.011195	0.011161	0.011330	0.011094	0.011263	0.011397
0.011330	0.011161	0.011161	0.011094	0.011094	0.011263	0.011195
0.011094	0.011195	0.000000	0.011094	0.011229	0.011364	0.011128
0.011094	0.011229	0.011162	0.000000	0.011397	0.000000	0.011229
0.011301	0.011128	0.011431	0.011263	0.011296	0.011161	0.011263
0.011128	0.011465	0.000000	0.011330	0.011128	0.011128	0.000000
0.011296	0.011499	0.011195	0.011263	0.011094	0.011499	0.011128
0.011128	0.011128	0.011161	0.011263	0.011161	0.011263	0.011330
0.011330	0.011296	0.011195	0.011296	0.011263	0.011128	0.011094
0.011128	0.011128	0.011195	0.011128	0.011094	0.011263	0.011128
0.011161	0.011161	0.011296	0.011330	0.011229	0.011161	0.011161
0.011195	0.011161	0.011263	0.011128	0.011263	0.011364	0.011667
0.011161	0.011566	0.011164	0.017704	0.011126	0.011306	0.011516
0.011477	0.011519	0.011217	0.011176	0.011612	0.011546	0.011243
0.011392	0.011428	0.000000	0.011144	0.011139	0.011209	0.011147
0.011389	0.011322	0.011321	0.011127	0.011296	0.011330	0.011332
0.011198	0.011296	0.011296	0.011263	0.011229	0.011229	0.011195
0.011229	0.011161	0.011195	0.011229	0.011195	0.011128	0.011161
0.011161	0.011229	0.011128	0.011229	0.011263	0.011296	0.011195
0.011229	0.011195	0.011532	0.011532	0.011263	0.011296	0.011330
0.011094	0.011094	0.011128	0.011229	0.011094	0.011330	0.011330
0.011364	0.011364	0.011296	0.011397	0.011566	0.011398	0.011229
0.011229	0.011296	0.011330	0.011161	0.011229	0.011195	0.011229
0.011296	0.011296	0.011330	0.011296	0.011196	0.011397	0.011364
0.011230	0.011128	0.011229	0.011330	0.011229	0.011364	0.011397
0.011566	0.011266	0.011431	0.011398	0.011330	0.011498	0.011128
0.011297	0.011195	0.011161	0.011161	0.011229	0.011128	0.011229
0.011263	0.011296	0.011195	0.011229	0.011195	0.011532	0.011532
0.011263	0.011296	0.011364	0.011296	0.011905	0.011347	0.011272
0.011343	0.011304	0.011378	0.011304	0.011412	0.011337	0.011303
0.011853	0.011199	0.011335	0.011269	0.011544	0.011402	0.011505
0.011880	0.011363	0.011230	0.011229	0.011229	0.011263	0.011196
0.011094	0.011128	0.011263	0.011229	0.011230	0.011364	0.011331
0.011195	0.011128	0.011161	0.011195	0.011094	0.011161	0.000000
0.011195	0.011229	0.011128	0.011094	0.011195	0.011128	0.011161
0.011161	0.011094	0.011161	0.011195	0.011094	0.011296	0.011330
0.011229	0.011296	0.011229	0.011229	0.011161	0.011566	0.011195
0.011235	0.011161	0.011398	0.011401	0.011263	0.011263	0.011263
0.011263	0.011195	0.011161	0.011195	0.011296	0.011296	0.011229
0.011229	0.011364	0.011532	0.011263	0.011465	0.011465	0.011330
0.011161	0.011229	0.011263	0.011667	0.011229	0.011263	0.011229
0.011263	0.011195	0.011195	0.011499	0.011431	0.011465	0.011195
0.011296	0.011364	0.011229	0.011296	0.011263	0.011128	0.011465
0.011229	0.011195	0.011195	0.011364	0.011195	0.011296	0.011532
0.011229	0.011606	0.011600	0.011386	0.011117	0.011299	0.011164
0.011115	0.011608	0.011131	0.011330	0.011158	0.011265	0.011274
0.011366	0.000000	0.011228	0.000000	0.011114	0.011262	0.011264
0.011287	0.011179	0.011272	0.011202	0.011229	0.011364	0.011431
0.011431	0.011297	0.011400	0.011401	0.011537	0.011556	0.011229
0.011465	0.011330	0.011195	0.011296	0.011094	0.011330	0.011128
0.011195	0.011195	0.011161	0.011128	0.011161	0.011128	0.011229
0.011201	0.011201	0.011198	0.011198	0.011369	0.011335	0.011334
0.011334	0.011229	0.000000	0.000000	0.011196	0.011366	0.011367
0.011162	0.011230	0.011466	0.011434	0.011364	0.011398	0.011330

0.011263	0.011330	0.011330	0.011229	0.011802	0.011397	0.011465
0.011499	0.011561	0.011197	0.011269	0.011161	0.011364	0.011330
0.011331	0.011397	0.011397	0.011397	0.011534	0.011532	0.011165
0.000000	0.000000	0.011163	0.011129	0.011232	0.000000	0.011402
0.011198	0.011325	0.000000	0.011211	0.011195	0.011266	0.011161
0.011128	0.011128	0.011195	0.011161	0.011364	0.011330	0.011263
0.011364	0.011364	0.011161	0.011161	0.011195	0.011195	0.011196
0.011264	0.011195	0.011195	0.011229	0.011364	0.011431	0.011431
0.011297	0.011400	0.011401	0.011537	0.011556	0.011229	0.011397
0.011330	0.011330	0.011162	0.011340	0.011270	0.011265	0.011166
0.011337	0.011516	0.011314	0.011231	0.011128	0.011330	0.011195
0.011296	0.011229	0.011128	0.011229	0.011263	0.011297	0.011229
0.011128	0.000000	0.011195	0.011128	0.000000	0.011162	0.011330
0.011229	0.011161	0.011195	0.011229	0.011195	0.011128	0.011330
0.011195	0.011229	0.011229	0.011161	0.011229	0.011465	0.011128
0.011161	0.011566	0.011161	0.011195	0.011161	0.011532	0.011103
0.011566	0.011103	0.011161	0.011343	0.011428	0.011349	0.000000
0.011113	0.011330	0.011431	0.011537	0.011398	0.011536	0.011668
0.011567	0.011604	0.011566	0.011815	0.011532	0.012410	0.011568
0.012310	0.012076	0.012211	0.012483	0.011532	0.011229	0.011499
0.011972	0.011873	0.011499	0.011398	0.011465	0.011465	0.011434
0.011330	0.011333	0.011397	0.011364	0.011735	0.011802	0.011803
0.011702	0.011803	0.011907	0.011672	0.011939	0.011944	0.012006
0.012007	0.012041	0.011985	0.012007	0.012008	0.011263	0.011533
0.011634	0.012140	0.011639	0.011870	0.012345	0.011401	0.011296
0.011263	0.011263	0.011735	0.011763	0.011873	0.011600	0.011263
S193 0.011665	0.011665	0.012001	0.011765	0.011765	0.011833	
0.011672	0.011604	0.011765	0.011262	0.011329	0.011296	0.011262
0.011295	0.011463	0.011262	0.011295	0.011362	0.011329	0.011295
0.011262	0.011295	0.011396	0.011262	0.011295	0.011295	0.011638
0.011530	0.011295	0.011195	0.011429	0.011362	0.011329	0.011362
0.011329	0.011429	0.011329	0.011329	0.011362	0.011329	0.011497
0.011262	0.011329	0.011362	0.011295	0.011396	0.011429	0.011362
0.011329	0.011497	0.011396	0.011665	0.011329	0.011597	0.011329
0.011295	0.011396	0.011463	0.011597	0.011698	0.011396	0.011497
0.011295	0.011430	0.011329	0.011497	0.011799	0.011329	0.011396
0.011295	0.011396	0.011362	0.011429	0.011362	0.011362	0.011362
0.011262	0.011295	0.011262	0.011362	0.011329	0.011363	0.011429
0.011362	0.011329	0.011396	0.011295	0.011429	0.011262	0.011329
0.011497	0.011429	0.011665	0.011497	0.011329	0.011564	0.011329
0.011505	0.011732	0.011551	0.011255	0.011247	0.011355	0.011454
0.012085	0.011518	0.011266	0.011564	0.011564	0.011362	0.011463
0.011396	0.011362	0.011429	0.011329	0.011430	0.011362	0.011362
0.011329	0.011429	0.011631	0.011497	0.011396	0.011362	0.011295
0.011262	0.011262	0.011329	0.011295	0.011497	0.011463	0.011396
0.011497	0.011564	0.011295	0.011295	0.011329	0.011329	0.011330
0.011398	0.011329	0.011329	0.011204	0.011277	0.011235	0.011329
0.011331	0.011266	0.011318	0.011302	0.011230	0.011266	0.011297
0.011564	0.011336	0.011370	0.011262	0.011268	0.011397	0.011363
0.011430	0.011363	0.011363	0.011463	0.011396	0.011765	0.011564
0.011597	0.011463	0.011329	0.011430	0.011228	0.011463	0.011262
0.011329	0.011329	0.011295	0.011262	0.011295	0.011262	0.011362
0.011329	0.011429	0.011362	0.011262	0.011363	0.011463	0.011466
0.011463	0.011362	0.011262	0.011195	0.000000	0.011262	0.011195
0.011298	0.011463	0.011262	0.011463	0.011228	0.011329	0.011295
0.011396	0.011295	0.011329	0.011295	0.011329	0.011195	0.011329
0.011362	0.011295	0.011362	0.011295	0.011329	0.011362	0.011597
0.011698	0.011631	0.011698	0.011465	0.011698	0.011329	0.011295
0.011330	0.011586	0.011426	0.011232	0.011705	0.011197	0.011296
0.011705	0.011604	0.011497	0.011698	0.011402	0.011564	0.011530

0.011463	0.011631	0.011487	0.011349	0.011396	0.011362	0.011432
0.011530	0.011431	0.011567	0.011636	0.011564	0.011631	0.011738
0.011466	0.011637	0.011568	0.011500	0.011767	0.011228	0.011431
0.011396	0.011329	0.011228	0.011228	0.011295	0.011295	0.011362
0.011295	0.011362	0.011262	0.011362	0.011295	0.011295	0.011362
0.011295	0.011732	0.011631	0.011665	0.011195	0.011295	0.011329
0.011329	0.011295	0.011362	0.011396	0.011262	0.011262	0.011262
0.011799	0.011429	0.011429	0.011228	0.011329	0.011497	0.011262
0.011262	0.011329	0.011295	0.011463	0.011262	0.011396	0.011530
0.011463	0.011295	0.011295	0.011228	0.011228	0.011396	0.011329
0.011228	0.011329	0.011195	0.011228	0.011362	0.011497	0.011262
0.011228	0.011362	0.011296	0.011195	0.011530	0.011195	0.011362
0.011437	0.011262	0.011564	0.011396	0.011429	0.011295	0.011396
0.011262	0.011597	0.011195	0.011463	0.011262	0.011262	0.011195
0.011429	0.011631	0.011329	0.011396	0.011228	0.011631	0.011262
0.011262	0.011262	0.011295	0.011396	0.011295	0.011396	0.011463
0.011463	0.011429	0.011329	0.011430	0.011329	0.011262	0.011228
0.011262	0.011262	0.011329	0.011262	0.011228	0.011396	0.011262
0.011295	0.011295	0.011429	0.011463	0.011362	0.011295	0.011295
0.011362	0.011295	0.011396	0.011262	0.011396	0.011497	0.011799
0.011295	0.011698	0.011302	0.017823	0.011387	0.011488	0.011653
0.011614	0.011658	0.011427	0.011408	0.011829	0.011681	0.011425
0.011582	0.011609	0.011216	0.011311	0.011294	0.011406	0.011318
0.011576	0.011470	0.011468	0.011193	0.011429	0.011463	0.011466
0.011335	0.011429	0.011429	0.011396	0.011362	0.011363	0.011329
0.011362	0.011295	0.011329	0.011362	0.011329	0.011262	0.011295
0.011295	0.011362	0.011262	0.011362	0.011396	0.011429	0.011329
0.011362	0.011329	0.011665	0.011665	0.011396	0.011430	0.011463
0.011228	0.011228	0.011262	0.011362	0.011228	0.011463	0.011463
0.011497	0.011497	0.011429	0.011530	0.011698	0.011532	0.011362
0.011362	0.011429	0.011463	0.011295	0.011362	0.011329	0.011362
0.011430	0.011430	0.011463	0.011430	0.011330	0.011530	0.011497
0.011365	0.011262	0.011362	0.011463	0.011362	0.011497	0.011530
0.011698	0.011402	0.011564	0.011530	0.011463	0.011631	0.011262
0.011431	0.011329	0.011295	0.011295	0.011362	0.011262	0.011362
0.011396	0.011429	0.011329	0.011362	0.011329	0.011665	0.011665
0.011396	0.011430	0.011497	0.011429	0.012070	0.011489	0.011412
0.011482	0.011442	0.011552	0.011441	0.011550	0.011474	0.011440
0.012022	0.011337	0.011471	0.011406	0.011680	0.011536	0.011639
0.012046	0.011512	0.011365	0.011362	0.011362	0.011396	0.011331
0.011228	0.011262	0.011396	0.011362	0.011364	0.011497	0.011464
0.011329	0.011262	0.011295	0.011329	0.011228	0.011295	0.011195
0.011329	0.011362	0.011262	0.011228	0.011329	0.011262	0.011295
0.011295	0.011228	0.011295	0.011329	0.011228	0.011429	0.011463
0.011362	0.011429	0.011362	0.011362	0.011295	0.011698	0.011329
0.011373	0.011295	0.011531	0.011535	0.011396	0.011396	0.011396
0.011396	0.011329	0.011295	0.011329	0.011429	0.011430	0.011362
0.011362	0.011497	0.011665	0.011396	0.011597	0.011597	0.011463
0.011295	0.011362	0.011396	0.011799	0.011363	0.011396	0.011362
0.011396	0.011329	0.011329	0.011631	0.011564	0.011597	0.011329
0.011430	0.011497	0.011362	0.011430	0.011396	0.011262	0.011597
0.011362	0.011329	0.011329	0.011497	0.011329	0.011429	0.011665
0.011362	0.011739	0.011732	0.011548	0.011342	0.011434	0.011318
0.011280	0.011761	0.011271	0.011507	0.011353	0.011467	0.011485
0.011570	0.011323	0.011394	0.011258	0.011330	0.011529	0.011425
0.011437	0.011336	0.011412	0.011342	0.011363	0.011497	0.011564
0.011564	0.011430	0.011533	0.011535	0.011670	0.011696	0.011362
0.011597	0.011463	0.011329	0.011430	0.011228	0.011463	0.011262
0.011329	0.011329	0.011295	0.011262	0.011295	0.011262	0.011362
0.011340	0.011340	0.011334	0.011368	0.011505	0.011470	0.011470

0.011469	0.011396	0.011208	0.011204	0.011331	0.011501	0.011501
0.011296	0.011365	0.011599	0.011567	0.011497	0.011531	0.011463
0.011396	0.011463	0.011463	0.011362	0.011934	0.011530	0.011598
0.011631	0.011701	0.011333	0.011407	0.011295	0.011497	0.011463
0.011498	0.011530	0.011530	0.011530	0.011650	0.011665	0.011303
0.011196	0.011199	0.011299	0.011264	0.011368	0.011210	0.011536
0.011335	0.011499	0.011251	0.011360	0.011329	0.011402	0.011295
0.011262	0.011262	0.011329	0.011295	0.011497	0.011463	0.011396
0.011497	0.011564	0.011295	0.011295	0.011329	0.011329	0.011330
0.011398	0.011329	0.011329	0.011363	0.011497	0.011564	0.011564
0.011430	0.011533	0.011535	0.011670	0.011696	0.011362	0.011530
0.011463	0.011463	0.011297	0.011478	0.011408	0.011401	0.011305
0.011473	0.011652	0.011441	0.011366	0.011262	0.011463	0.011329
0.011429	0.011362	0.011262	0.011363	0.011463	0.011466	0.011362
0.011262	0.011195	0.000000	0.011262	0.011195	0.011298	0.011463
0.011362	0.011295	0.011329	0.011362	0.011329	0.011262	0.011463
0.011329	0.011362	0.011362	0.011295	0.011362	0.011597	0.011262
0.011295	0.011698	0.011295	0.011329	0.011295	0.011665	0.011274
0.011791	0.011273	0.011361	0.011503	0.011589	0.011491	0.011227
0.011323	0.011463	0.011564	0.011671	0.011531	0.011669	0.011800
0.011699	0.011737	0.011698	0.011948	0.011665	0.012539	0.011700
0.012439	0.012207	0.012341	0.012612	0.011665	0.011362	0.011665
0.012103	0.012038	0.011631	0.011530	0.011598	0.011598	0.011568
0.011463	0.011467	0.011530	0.011497	0.011867	0.011934	0.011935
0.011834	0.011934	0.012038	0.011804	0.012104	0.012110	0.012171
0.012171	0.012205	0.012152	0.012172	0.012173	0.011396	0.011699
0.011766	0.012270	0.011772	0.012001	0.012474	0.011536	0.011430
0.011396	0.011396	0.011867	0.011924	0.012010	0.011732	0.011397
S194	0.011598	0.011598	0.011934	0.011698	0.011698	0.011766
0.011604	0.011536	0.011698	0.011195	0.011262	0.011229	0.011195
0.011228	0.011396	0.011195	0.011228	0.011295	0.011262	0.011228
0.011195	0.011228	0.011329	0.011195	0.011228	0.011228	0.011570
0.011463	0.011228	0.011127	0.011362	0.011295	0.011262	0.011295
0.011262	0.011362	0.011262	0.011262	0.011295	0.011262	0.011430
0.011195	0.011262	0.011295	0.011228	0.011329	0.011362	0.011295
0.011262	0.011430	0.011329	0.011597	0.011262	0.011530	0.011262
0.011228	0.011329	0.011396	0.011530	0.011631	0.011329	0.011430
0.011295	0.011362	0.011262	0.011497	0.011732	0.011262	0.011329
0.011228	0.011329	0.011295	0.011362	0.011295	0.011295	0.011295
0.011195	0.011228	0.011195	0.011295	0.011262	0.011296	0.011362
0.011295	0.011262	0.011329	0.011228	0.011362	0.011195	0.011262
0.011429	0.011362	0.011597	0.011430	0.011262	0.011497	0.011262
0.011431	0.011658	0.011437	0.011138	0.011098	0.011281	0.011375
0.012011	0.011365	0.011143	0.011497	0.011497	0.011295	0.011396
0.011329	0.011295	0.011362	0.011262	0.011362	0.011295	0.011295
0.011262	0.011362	0.011564	0.011430	0.011329	0.011295	0.011228
0.011195	0.011195	0.011262	0.011228	0.011429	0.011396	0.011329
0.011429	0.011497	0.011228	0.011228	0.011262	0.011262	0.011263
0.011331	0.011262	0.011262	0.011132	0.011205	0.011165	0.011262
0.011263	0.011198	0.011208	0.011233	0.011162	0.011197	0.011229
0.011492	0.011267	0.011301	0.011195	0.011199	0.011329	0.011296
0.011363	0.011296	0.011296	0.011396	0.011329	0.011698	0.011497
0.011530	0.011396	0.011262	0.011362	0.011161	0.011396	0.011195
0.011262	0.011262	0.011228	0.011195	0.011228	0.011195	0.011295
0.011262	0.011362	0.011295	0.011195	0.011296	0.011396	0.011398
0.011396	0.011295	0.000000	0.011128	0.011262	0.000000	0.011127
0.011230	0.011396	0.011195	0.011396	0.011161	0.011262	0.011228
0.011329	0.011228	0.011262	0.011228	0.011262	0.011127	0.011262
0.011295	0.011228	0.011295	0.011228	0.011262	0.011295	0.011530
0.011631	0.011564	0.011631	0.011397	0.011631	0.011262	0.011228

0.011263	0.011505	0.011289	0.011163	0.011637	0.011129	0.011218
0.011637	0.011536	0.011429	0.011631	0.011333	0.011497	0.011463
0.011396	0.011564	0.011416	0.011266	0.011270	0.011295	0.011365
0.011463	0.011364	0.011500	0.011568	0.011497	0.011564	0.011670
0.011399	0.011569	0.011500	0.011432	0.011700	0.011161	0.011364
0.011329	0.011262	0.011161	0.011161	0.011228	0.011228	0.011295
0.011228	0.011295	0.011195	0.011295	0.011228	0.011228	0.011295
0.011228	0.011665	0.011564	0.011597	0.011127	0.011228	0.011262
0.011262	0.011228	0.011295	0.011329	0.011195	0.011195	0.011195
0.011732	0.011362	0.011362	0.011161	0.011262	0.011430	0.011195
0.011195	0.011262	0.011228	0.011396	0.011195	0.011329	0.011463
0.011396	0.011228	0.011228	0.011161	0.011161	0.011262	0.011262
0.011161	0.011262	0.011127	0.011161	0.011295	0.011430	0.011195
0.011161	0.011295	0.011228	0.011127	0.011463	0.011127	0.011295
0.011368	0.011195	0.011497	0.011329	0.011362	0.011228	0.011329
0.011195	0.011530	0.011127	0.011396	0.011195	0.011195	0.011127
0.011362	0.011564	0.011262	0.011329	0.011161	0.011564	0.011195
0.011195	0.011195	0.011228	0.011329	0.011228	0.011329	0.011396
0.011396	0.011362	0.011262	0.011362	0.011329	0.011195	0.011161
0.011195	0.011195	0.011262	0.011195	0.011161	0.011329	0.011195
0.011228	0.011228	0.011362	0.011396	0.011295	0.011228	0.011228
0.011295	0.011228	0.011329	0.011195	0.011329	0.011430	0.011732
0.011228	0.011631	0.011233	0.017748	0.011126	0.011305	0.011583
0.011545	0.011588	0.011217	0.011176	0.011609	0.011613	0.011242
0.011392	0.011426	0.011138	0.011186	0.011216	0.011208	0.011146
0.011389	0.011395	0.011357	0.011259	0.011362	0.011396	0.011398
0.011266	0.011362	0.011362	0.011329	0.011295	0.011295	0.011262
0.011295	0.011228	0.011262	0.011295	0.011262	0.011195	0.011228
0.011228	0.011295	0.011195	0.011295	0.011329	0.011362	0.011262
0.011295	0.011262	0.011597	0.011597	0.011329	0.011363	0.011396
0.011161	0.011161	0.011195	0.011295	0.011161	0.011396	0.011396
0.011429	0.011429	0.011362	0.011463	0.011631	0.011464	0.011295
0.011295	0.011362	0.011396	0.011228	0.011295	0.011262	0.011295
0.011363	0.011362	0.011396	0.011363	0.011262	0.011463	0.011430
0.011297	0.011195	0.011295	0.011396	0.011295	0.011430	0.011463
0.011631	0.011333	0.011497	0.011463	0.011396	0.011564	0.011195
0.011363	0.011262	0.011228	0.011228	0.011295	0.011195	0.011295
0.011329	0.011362	0.011262	0.011295	0.011262	0.011597	0.011597
0.011329	0.011363	0.011429	0.011362	0.012002	0.011418	0.011342
0.011412	0.011372	0.011482	0.011372	0.011480	0.011405	0.011371
0.011953	0.011268	0.011403	0.011337	0.011611	0.011468	0.011571
0.011978	0.011437	0.011297	0.011295	0.011295	0.011329	0.011263
0.011161	0.011195	0.011329	0.011295	0.011297	0.011429	0.011397
0.011262	0.011195	0.011228	0.011262	0.011161	0.011228	0.011127
0.011262	0.011295	0.011195	0.011161	0.011262	0.011195	0.011228
0.011228	0.011161	0.011228	0.011262	0.011161	0.011362	0.011396
0.011295	0.011362	0.011295	0.011295	0.011228	0.011631	0.011262
0.011304	0.011228	0.011463	0.011467	0.011329	0.011329	0.011329
0.011329	0.011262	0.011228	0.011262	0.011362	0.011362	0.011295
0.011295	0.011430	0.011597	0.011329	0.011530	0.011530	0.011396
0.011228	0.011295	0.011329	0.011732	0.011295	0.011329	0.011295
0.011329	0.011262	0.011262	0.011564	0.011497	0.011530	0.011262
0.011362	0.011430	0.011295	0.011362	0.011329	0.011195	0.011530
0.011295	0.011262	0.011262	0.011430	0.011262	0.011362	0.011598
0.011295	0.011672	0.011665	0.011426	0.011117	0.011366	0.011164
0.011115	0.011683	0.011201	0.011373	0.011158	0.011264	0.011273
0.011366	0.000000	0.011227	0.000000	0.011114	0.011261	0.011344
0.011362	0.011257	0.011342	0.011272	0.011296	0.011429	0.011497
0.011497	0.011363	0.011466	0.011467	0.011603	0.011626	0.011295
0.011530	0.011396	0.011262	0.011362	0.011161	0.011396	0.011195

0.011262	0.011262	0.011228	0.011195	0.011228	0.011195	0.011295
0.011270	0.011270	0.011266	0.011300	0.011437	0.011402	0.011402
0.011401	0.011329	0.011134	0.011132	0.011263	0.011433	0.011433
0.011228	0.011297	0.011531	0.011500	0.011430	0.011464	0.011396
0.011329	0.011396	0.011396	0.011295	0.011867	0.011463	0.011530
0.011564	0.011630	0.011265	0.011337	0.011228	0.011430	0.011396
0.011430	0.011463	0.011463	0.011463	0.011571	0.011597	0.011234
0.011128	0.011130	0.011231	0.011196	0.011299	0.011135	0.011468
0.011266	0.011367	0.011108	0.011285	0.011262	0.011334	0.011228
0.011195	0.011195	0.011262	0.011228	0.011429	0.011396	0.011329
0.011429	0.011497	0.011228	0.011228	0.011262	0.011262	0.011263
0.011331	0.011262	0.011262	0.011296	0.011429	0.011497	0.011497
0.011363	0.011466	0.011467	0.011603	0.011626	0.011295	0.011463
0.011396	0.011396	0.011229	0.011408	0.011338	0.011367	0.011235
0.011370	0.011607	0.011399	0.011298	0.011195	0.011396	0.011262
0.011362	0.011295	0.011195	0.011296	0.011396	0.011398	0.011295
0.000000	0.011128	0.011262	0.000000	0.011127	0.011230	0.011396
0.011295	0.011228	0.011262	0.011295	0.011262	0.011195	0.011396
0.011262	0.011295	0.011295	0.011228	0.011295	0.011530	0.011195
0.011228	0.011631	0.011228	0.011262	0.011228	0.011597	0.011103
0.011566	0.011145	0.011161	0.011422	0.011467	0.011419	0.000000
0.011113	0.011396	0.011497	0.011603	0.011464	0.011601	0.011733
0.011632	0.011669	0.011631	0.011880	0.011597	0.012472	0.011633
0.012372	0.012139	0.012273	0.012545	0.011597	0.011295	0.011598
0.012036	0.011971	0.011564	0.011463	0.011531	0.011531	0.011500
0.011396	0.011399	0.011463	0.011430	0.011800	0.011866	0.011867
0.011767	0.011867	0.011970	0.011737	0.012037	0.012042	0.012104
0.012104	0.012138	0.012084	0.012104	0.012105	0.011329	0.011631
0.011666	0.012203	0.011704	0.011934	0.012407	0.011468	0.011363
0.011329	0.011329	0.011799	0.011842	0.011940	0.011665	0.011330
S195	0.011531	0.011531	0.011867	0.011631	0.011631	0.011699
0.011536	0.011468	0.011631	0.011128	0.011195	0.011161	0.011127
0.011161	0.011329	0.011127	0.011161	0.011228	0.011195	0.011161
0.011127	0.011161	0.011262	0.011128	0.011161	0.011161	0.011502
0.011396	0.011161	0.000000	0.011295	0.011228	0.011195	0.011228
0.011195	0.011295	0.011195	0.011195	0.011228	0.011195	0.011363
0.011127	0.011195	0.011228	0.011161	0.011262	0.011295	0.011228
0.011195	0.011363	0.011262	0.011530	0.011195	0.011463	0.011195
0.011161	0.011262	0.011329	0.011463	0.011564	0.011262	0.011363
0.011228	0.011295	0.011195	0.011430	0.011665	0.011195	0.011262
0.011161	0.011262	0.011228	0.011295	0.011228	0.011228	0.011228
0.011127	0.011161	0.011127	0.011228	0.011195	0.011229	0.011295
0.011228	0.011195	0.011262	0.011161	0.011295	0.011128	0.011195
0.011362	0.011295	0.011530	0.011363	0.011195	0.011430	0.011195
0.011357	0.011583	0.011400	0.011099	0.011098	0.011208	0.011297
0.011939	0.011365	0.011102	0.011430	0.011430	0.011228	0.011329
0.011262	0.011228	0.011295	0.011195	0.011295	0.011228	0.011228
0.011195	0.011295	0.011497	0.011363	0.011262	0.011228	0.011161
0.011127	0.011127	0.011195	0.011161	0.011362	0.011329	0.011262
0.011362	0.011430	0.011161	0.011161	0.011195	0.011195	0.011195
0.011263	0.011195	0.011195	0.000000	0.011133	0.011095	0.011195
0.011196	0.011129	0.011171	0.011164	0.011094	0.011129	0.011162
0.011420	0.011198	0.011233	0.011128	0.011129	0.011262	0.011228
0.011296	0.011229	0.011228	0.011329	0.011262	0.011631	0.011430
0.011463	0.011329	0.011195	0.011295	0.011094	0.011329	0.011128
0.011195	0.011195	0.011161	0.011127	0.011161	0.011127	0.011228
0.011195	0.011295	0.011228	0.011127	0.011229	0.011329	0.011331
0.011329	0.011228	0.011127	0.000000	0.011195	0.011127	0.000000
0.011162	0.011329	0.011128	0.011329	0.011094	0.011195	0.011161
0.011262	0.011161	0.011195	0.011161	0.011195	0.000000	0.011195

0.011228	0.011161	0.011228	0.011161	0.011195	0.011228	0.011463
0.011564	0.011497	0.011564	0.011330	0.011564	0.011195	0.011161
0.011195	0.011465	0.011289	0.011095	0.011569	0.000000	0.011178
0.011570	0.011468	0.011362	0.011564	0.011265	0.011430	0.011396
0.011329	0.011497	0.011345	0.011184	0.011228	0.011228	0.011297
0.011396	0.011296	0.011432	0.011500	0.011430	0.011497	0.011603
0.011331	0.011502	0.011433	0.011365	0.011633	0.011094	0.011296
0.011262	0.011195	0.011094	0.011094	0.011161	0.011161	0.011228
0.011161	0.011228	0.011127	0.011228	0.011161	0.011161	0.011228
0.011161	0.011598	0.011497	0.011531	0.000000	0.011161	0.011195
0.011195	0.011161	0.011228	0.011262	0.011127	0.011127	0.011127
0.011665	0.011295	0.011295	0.011094	0.011195	0.011363	0.011128
0.011127	0.011195	0.011161	0.011329	0.011127	0.011262	0.011396
0.011329	0.011161	0.011161	0.011094	0.011094	0.011262	0.011195
0.011094	0.011195	0.000000	0.011094	0.011228	0.011363	0.011128
0.011094	0.011228	0.011161	0.000000	0.011396	0.000000	0.011228
0.011300	0.011128	0.011430	0.011262	0.011295	0.011161	0.011262
0.011127	0.011463	0.000000	0.011329	0.011127	0.011127	0.000000
0.011295	0.011497	0.011195	0.011262	0.011094	0.011497	0.011127
0.011128	0.011127	0.011161	0.011262	0.011161	0.011262	0.011329
0.011329	0.011295	0.011195	0.011295	0.011262	0.011127	0.011094
0.011127	0.011127	0.011195	0.011127	0.011094	0.011262	0.011127
0.011161	0.011161	0.011295	0.011329	0.011228	0.011161	0.011161
0.011228	0.011161	0.011262	0.011127	0.011262	0.011363	0.011665
0.011161	0.011564	0.011164	0.017638	0.011126	0.011305	0.011514
0.011476	0.011518	0.011217	0.011176	0.011610	0.011544	0.011242
0.011392	0.011427	0.000000	0.011144	0.011138	0.011208	0.011146
0.011389	0.011321	0.011320	0.011126	0.011295	0.011329	0.011331
0.011198	0.011295	0.011295	0.011262	0.011228	0.011228	0.011195
0.011228	0.011161	0.011195	0.011228	0.011195	0.011128	0.011161
0.011161	0.011228	0.011127	0.011228	0.011262	0.011295	0.011195
0.011228	0.011195	0.011530	0.011530	0.011262	0.011296	0.011329
0.011094	0.011094	0.011127	0.011228	0.011094	0.011329	0.011329
0.011362	0.011362	0.011295	0.011396	0.011564	0.011397	0.011228
0.011228	0.011295	0.011329	0.011161	0.011228	0.011195	0.011228
0.011296	0.011295	0.011329	0.011295	0.011195	0.011396	0.011363
0.011230	0.011128	0.011228	0.011329	0.011228	0.011363	0.011396
0.011564	0.011265	0.011430	0.011396	0.011329	0.011497	0.011128
0.011296	0.011195	0.011161	0.011161	0.011228	0.011127	0.011228
0.011262	0.011295	0.011195	0.011228	0.011195	0.011530	0.011530
0.011262	0.011296	0.011362	0.011295	0.011935	0.011346	0.011271
0.011342	0.011303	0.011412	0.011303	0.011410	0.011336	0.011302
0.011885	0.011199	0.011334	0.011268	0.011543	0.011401	0.011503
0.011911	0.011362	0.011229	0.011228	0.011228	0.011262	0.011196
0.011094	0.011128	0.011262	0.011228	0.011229	0.011362	0.011330
0.011195	0.011127	0.011161	0.011195	0.011094	0.011161	0.000000
0.011195	0.011228	0.011127	0.011094	0.011195	0.011127	0.011161
0.011161	0.011094	0.011161	0.011195	0.011094	0.011295	0.011329
0.011228	0.011295	0.011228	0.011228	0.011161	0.011564	0.011195
0.011234	0.011161	0.011396	0.011400	0.011262	0.011262	0.011262
0.011262	0.011195	0.011161	0.011195	0.011295	0.011295	0.011228
0.011228	0.011363	0.011530	0.011262	0.011463	0.011463	0.011329
0.011161	0.011228	0.011262	0.011665	0.011228	0.011262	0.011228
0.011262	0.011195	0.011195	0.011497	0.011430	0.011463	0.011195
0.011295	0.011363	0.011228	0.011295	0.011262	0.011128	0.011463
0.011228	0.011195	0.011195	0.011363	0.011195	0.011295	0.011531
0.011228	0.011604	0.011598	0.011385	0.011117	0.011298	0.011164
0.011115	0.011566	0.011131	0.011328	0.011158	0.011264	0.011273
0.011366	0.000000	0.011227	0.000000	0.011114	0.011261	0.011263
0.011286	0.011178	0.011272	0.011201	0.011229	0.011362	0.011430

0.011430	0.011296	0.011398	0.011399	0.011535	0.011555	0.011228
0.011463	0.011329	0.011195	0.011295	0.011094	0.011329	0.011128
0.011195	0.011195	0.011161	0.011127	0.011161	0.011127	0.011228
0.011200	0.011200	0.011197	0.011232	0.011368	0.011334	0.011333
0.011333	0.011262	0.000000	0.000000	0.011196	0.011365	0.011366
0.011161	0.011230	0.011464	0.011432	0.011363	0.011397	0.011329
0.011262	0.011329	0.011329	0.011228	0.011800	0.011396	0.011463
0.011497	0.011559	0.011197	0.011268	0.011161	0.011363	0.011329
0.011363	0.011396	0.011396	0.011396	0.011532	0.011531	0.011164
0.000000	0.000000	0.011163	0.011128	0.011231	0.000000	0.011400
0.011198	0.011323	0.000000	0.011210	0.011195	0.011266	0.011161
0.011127	0.011127	0.011195	0.011161	0.011362	0.011329	0.011262
0.011362	0.011430	0.011161	0.011161	0.011195	0.011195	0.011195
0.011263	0.011195	0.011195	0.011229	0.011362	0.011430	0.011430
0.011296	0.011398	0.011399	0.011535	0.011555	0.011228	0.011396
0.011329	0.011329	0.011162	0.011339	0.011269	0.011299	0.011165
0.011336	0.011516	0.011314	0.011230	0.011128	0.011329	0.011195
0.011295	0.011228	0.011127	0.011229	0.011329	0.011331	0.011228
0.011127	0.000000	0.011195	0.011127	0.000000	0.011162	0.011329
0.011228	0.011161	0.011195	0.011228	0.011195	0.011128	0.011329
0.011195	0.011228	0.011228	0.011161	0.011228	0.011463	0.011127
0.011161	0.011564	0.011161	0.011195	0.011161	0.011530	0.011103
0.011566	0.011103	0.011161	0.011342	0.011427	0.011347	0.000000
0.011113	0.011329	0.011396	0.011502	0.011363	0.011500	0.011632
0.011531	0.011568	0.011531	0.011812	0.011531	0.012405	0.011566
0.012305	0.012072	0.012206	0.012478	0.011530	0.011228	0.011531
0.011969	0.011904	0.011497	0.011396	0.011464	0.011464	0.011432
0.011329	0.011332	0.011396	0.011363	0.011733	0.011800	0.011800
0.011700	0.011800	0.011903	0.011669	0.011970	0.011975	0.012037
0.012037	0.012071	0.012016	0.012037	0.012038	0.011262	0.011564
0.011632	0.012136	0.011637	0.011867	0.012340	0.011400	0.011296
0.011262	0.011262	0.011733	0.011759	0.011870	0.011598	0.011263
S196 0.011638	0.011638	0.011842	0.011604	0.011604	0.011672	
0.011541	0.011473	0.011604	0.011230	0.011298	0.011264	0.011230
0.011264	0.011434	0.011230	0.011264	0.011332	0.011298	0.011196
0.011230	0.011264	0.011264	0.011230	0.011264	0.011264	0.011507
0.011400	0.011264	0.011162	0.011298	0.011332	0.011298	0.011332
0.011298	0.011400	0.011298	0.011298	0.011332	0.011298	0.011468
0.011230	0.011298	0.011332	0.011264	0.011366	0.011400	0.011332
0.011298	0.011468	0.011366	0.011468	0.011298	0.011570	0.011298
0.011264	0.011366	0.011434	0.011570	0.011570	0.011366	0.011468
0.011332	0.011400	0.011264	0.011536	0.011672	0.011298	0.011366
0.011264	0.011366	0.011332	0.011400	0.011298	0.011332	0.011332
0.011230	0.011264	0.011230	0.011332	0.011298	0.011298	0.011400
0.011298	0.011298	0.011366	0.011264	0.011400	0.011230	0.011298
0.011468	0.011400	0.011536	0.011434	0.011298	0.011536	0.011298
0.011472	0.011588	0.011517	0.011218	0.011173	0.011321	0.011299
0.011947	0.011369	0.011144	0.011536	0.011536	0.011332	0.011434
0.011366	0.011332	0.011400	0.011298	0.011400	0.011332	0.011332
0.011298	0.011366	0.011604	0.011468	0.011332	0.011332	0.011264
0.011230	0.011230	0.011298	0.011264	0.011332	0.011332	0.011264
0.011366	0.011434	0.011264	0.011264	0.011298	0.011298	0.011299
0.011368	0.011298	0.011298	0.011169	0.011243	0.011202	0.011298
0.011300	0.011234	0.011284	0.011270	0.011198	0.011234	0.011265
0.011425	0.011304	0.011339	0.011162	0.011235	0.011366	0.011332
0.011400	0.011333	0.011332	0.011434	0.011366	0.011740	0.011536
0.011468	0.011434	0.011298	0.011400	0.011196	0.011332	0.011230
0.011298	0.011298	0.011264	0.011230	0.011264	0.011230	0.011332
0.011298	0.011400	0.011332	0.011230	0.011333	0.011434	0.011401
0.011434	0.011332	0.011230	0.011162	0.011298	0.011230	0.011162

0.000000	0.011434	0.011230	0.011434	0.011196	0.011264	0.011264
0.011366	0.011264	0.011298	0.011264	0.011298	0.011162	0.011298
0.011332	0.011196	0.011332	0.011264	0.011298	0.011332	0.011570
0.011570	0.011502	0.011570	0.011435	0.011570	0.011298	0.011196
0.011299	0.011591	0.011430	0.011199	0.011575	0.011164	0.011299
0.011575	0.011473	0.011468	0.011672	0.011371	0.011536	0.011502
0.011434	0.011604	0.011456	0.011311	0.011315	0.011230	0.011403
0.011502	0.011401	0.011539	0.011539	0.011502	0.011536	0.011711
0.011437	0.011541	0.011540	0.011471	0.011741	0.011196	0.011401
0.011366	0.011298	0.011196	0.011196	0.011264	0.011264	0.011332
0.011264	0.011332	0.011230	0.011332	0.011264	0.011196	0.011332
0.011264	0.011604	0.011502	0.011536	0.011162	0.011264	0.011298
0.011264	0.011264	0.011332	0.011366	0.011196	0.011196	0.011196
0.011672	0.011400	0.011400	0.011196	0.011298	0.011434	0.011230
0.011230	0.011298	0.011264	0.011434	0.011230	0.011264	0.011400
0.011332	0.011264	0.011162	0.011196	0.011196	0.011366	0.011298
0.011196	0.011298	0.011162	0.011196	0.011332	0.011366	0.011230
0.011196	0.011332	0.011264	0.011162	0.011400	0.011162	0.011332
0.011407	0.011230	0.011434	0.011366	0.011400	0.011264	0.011366
0.011230	0.011570	0.011162	0.011434	0.011230	0.011230	0.011162
0.011400	0.011502	0.011264	0.011366	0.011196	0.011502	0.011230
0.011230	0.011162	0.011264	0.011366	0.011264	0.011366	0.011434
0.011400	0.011400	0.011298	0.011400	0.011366	0.011230	0.011196
0.011230	0.011230	0.011264	0.011230	0.011196	0.011366	0.011230
0.011264	0.011264	0.011400	0.011434	0.011332	0.011264	0.011264
0.011298	0.011264	0.011332	0.011230	0.011366	0.011468	0.011672
0.011264	0.011570	0.011270	0.017851	0.011258	0.011434	0.011519
0.011480	0.011523	0.011378	0.011351	0.011616	0.011549	0.011429
0.011537	0.011431	0.011179	0.011273	0.011257	0.011359	0.011277
0.011392	0.011323	0.011322	0.011260	0.011366	0.011434	0.011436
0.011304	0.011400	0.011400	0.011366	0.011332	0.011332	0.011298
0.011332	0.011264	0.011298	0.011332	0.011264	0.011230	0.011264
0.011264	0.011332	0.011230	0.011298	0.011332	0.011400	0.011298
0.011332	0.011298	0.011536	0.011536	0.011366	0.011400	0.011434
0.011196	0.011196	0.011230	0.011332	0.011196	0.011434	0.011434
0.011468	0.011468	0.011366	0.011502	0.011570	0.011503	0.011332
0.011298	0.011400	0.011434	0.011264	0.011332	0.011298	0.011332
0.011400	0.011400	0.011434	0.011400	0.011265	0.011502	0.011468
0.011334	0.011230	0.011332	0.011400	0.011332	0.011468	0.011502
0.011672	0.011371	0.011536	0.011502	0.011434	0.011604	0.011230
0.011401	0.011298	0.011264	0.011264	0.011332	0.011230	0.011298
0.011332	0.011400	0.011298	0.011332	0.011298	0.011536	0.011536
0.011366	0.011400	0.011468	0.011400	0.012013	0.011458	0.011381
0.011452	0.011411	0.011451	0.011376	0.011521	0.011444	0.011409
0.011964	0.011305	0.011441	0.011375	0.011583	0.011508	0.011542
0.011989	0.011404	0.011334	0.011332	0.011332	0.011264	0.011300
0.011196	0.011230	0.011332	0.011332	0.011334	0.011366	0.011333
0.011298	0.011162	0.011264	0.011298	0.011196	0.011264	0.011162
0.011298	0.011332	0.011230	0.011196	0.011264	0.011230	0.011264
0.011264	0.011196	0.011264	0.011298	0.011196	0.011400	0.011434
0.011298	0.011400	0.011332	0.011332	0.011264	0.011570	0.011298
0.011236	0.011264	0.011400	0.011403	0.011366	0.011366	0.011366
0.011366	0.011298	0.011264	0.011298	0.011400	0.011400	0.011298
0.011298	0.011468	0.011536	0.011366	0.011570	0.011570	0.011434
0.011264	0.011332	0.011366	0.011672	0.011332	0.011366	0.011298
0.011366	0.011298	0.011298	0.011604	0.011536	0.011570	0.011298
0.011400	0.011468	0.011332	0.011400	0.011366	0.011230	0.011468
0.011298	0.011298	0.011298	0.011468	0.011298	0.011400	0.011536
0.011332	0.011610	0.011604	0.011388	0.011287	0.011404	0.011319
0.011283	0.011611	0.011238	0.011467	0.011307	0.011368	0.011381

0.011369	0.011193	0.011340	0.011193	0.011223	0.011398	0.011388
0.011403	0.011299	0.011381	0.011310	0.011333	0.011366	0.011434
0.011434	0.011400	0.011505	0.011403	0.011643	0.011667	0.011332
0.011468	0.011434	0.011298	0.011400	0.011196	0.011332	0.011230
0.011298	0.011298	0.011264	0.011230	0.011264	0.011230	0.011332
0.011308	0.011308	0.011233	0.011303	0.011475	0.011440	0.011440
0.011439	0.011332	0.011172	0.011170	0.011300	0.011471	0.011472
0.011264	0.011334	0.011571	0.011539	0.011468	0.011503	0.011434
0.011366	0.011434	0.011434	0.011332	0.011808	0.011502	0.011570
0.011604	0.011565	0.011302	0.011376	0.011264	0.011468	0.011434
0.011435	0.011502	0.011502	0.011502	0.011617	0.011638	0.011271
0.011163	0.011165	0.011267	0.011232	0.011337	0.011136	0.011404
0.011234	0.011283	0.011157	0.011288	0.011298	0.011372	0.011264
0.011230	0.011230	0.011298	0.011264	0.011332	0.011332	0.011264
0.011366	0.011434	0.011264	0.011264	0.011298	0.011298	0.011299
0.011368	0.011298	0.011298	0.011333	0.011366	0.011434	0.011434
0.011400	0.011505	0.011403	0.011643	0.011667	0.011332	0.011502
0.011434	0.011434	0.011265	0.011447	0.011377	0.011370	0.011273
0.011443	0.011660	0.011446	0.011335	0.011230	0.011434	0.011298
0.011400	0.011332	0.011230	0.011333	0.011434	0.011401	0.011332
0.011230	0.011162	0.011298	0.011230	0.011162	0.000000	0.011434
0.011332	0.011264	0.011298	0.011332	0.011264	0.011230	0.011332
0.011298	0.011332	0.011332	0.011264	0.011332	0.011570	0.011230
0.011264	0.011570	0.011264	0.011298	0.011264	0.011468	0.011233
0.011739	0.011231	0.011312	0.011345	0.011430	0.011350	0.011187
0.011271	0.011332	0.011434	0.011507	0.011401	0.011539	0.011672
0.011571	0.011608	0.011570	0.011821	0.011502	0.012386	0.011571
0.012285	0.012049	0.012185	0.012459	0.011536	0.011332	0.011536
0.012081	0.011913	0.011502	0.011400	0.011468	0.011468	0.011402
0.011332	0.011335	0.011366	0.011366	0.011740	0.011808	0.011809
0.011707	0.011808	0.011879	0.011642	0.012048	0.012053	0.012115
0.012116	0.012150	0.012096	0.012116	0.012117	0.011366	0.011638
0.011741	0.012182	0.011746	0.011842	0.012320	0.011404	0.011298
0.011264	0.011264	0.011740	0.011685	0.011879	0.011570	0.011265
S197 0.011799	0.011799	0.012136	0.011900	0.011900	0.011967	
0.011808	0.011740	0.011900	0.011396	0.011463	0.011431	0.011396
0.011430	0.011598	0.011396	0.011430	0.011497	0.011463	0.011430
0.011396	0.011430	0.011530	0.011396	0.011363	0.011430	0.011774
0.011665	0.011396	0.011329	0.011564	0.011430	0.011463	0.011430
0.011463	0.011497	0.011463	0.011463	0.011497	0.011463	0.011631
0.011396	0.011463	0.011497	0.011430	0.011530	0.011564	0.011497
0.011463	0.011631	0.011531	0.011799	0.011463	0.011665	0.011463
0.011430	0.011531	0.011598	0.011665	0.011833	0.011531	0.011631
0.011430	0.011497	0.011463	0.011631	0.011934	0.011463	0.011530
0.011430	0.011530	0.011497	0.011564	0.011497	0.011497	0.011497
0.011396	0.011430	0.011396	0.011497	0.011463	0.011498	0.011564
0.011497	0.011463	0.011396	0.011430	0.011564	0.011329	0.011463
0.011631	0.011564	0.011799	0.011631	0.011463	0.011631	0.011463
0.011654	0.011882	0.011664	0.011373	0.011359	0.011502	0.011572
0.012231	0.011620	0.011390	0.011698	0.011698	0.011497	0.011598
0.011531	0.011497	0.011564	0.011463	0.011564	0.011430	0.011497
0.011463	0.011564	0.011766	0.011631	0.011531	0.011497	0.011362
0.011396	0.011396	0.011329	0.011430	0.011631	0.011598	0.011530
0.011631	0.011698	0.011430	0.011430	0.011463	0.011463	0.011466
0.011534	0.011463	0.011463	0.011312	0.011386	0.011376	0.011464
0.011467	0.011404	0.011429	0.011440	0.011366	0.011403	0.011432
0.011708	0.011474	0.011439	0.011397	0.011406	0.011531	0.011498
0.011565	0.011498	0.011497	0.011597	0.011530	0.011833	0.011699
0.011732	0.011598	0.011463	0.011564	0.011362	0.011598	0.011396
0.011463	0.011463	0.011430	0.011396	0.011430	0.011396	0.011497

0.011396	0.011497	0.011497	0.011396	0.011498	0.011598	0.011601
0.000000	0.011497	0.011396	0.011330	0.011463	0.011396	0.011329
0.011434	0.000000	0.011329	0.000000	0.011363	0.011463	0.011430
0.011463	0.011430	0.011396	0.011430	0.011463	0.011329	0.011396
0.011430	0.011430	0.011497	0.011430	0.011463	0.011497	0.011732
0.011833	0.011766	0.011833	0.011600	0.011833	0.011463	0.011430
0.011466	0.011667	0.011563	0.011369	0.011807	0.011333	0.011454
0.011842	0.011740	0.011631	0.011833	0.011538	0.011699	0.011598
0.011598	0.011765	0.011629	0.011514	0.011564	0.011497	0.011568
0.011665	0.011566	0.011703	0.011704	0.011699	0.011766	0.011874
0.011602	0.011706	0.011704	0.011636	0.011902	0.011362	0.011566
0.011463	0.011463	0.011362	0.011362	0.011362	0.011430	0.011497
0.011362	0.011430	0.011329	0.011497	0.011362	0.011430	0.011497
0.011430	0.011866	0.011766	0.011799	0.011329	0.011430	0.011463
0.011463	0.011430	0.011497	0.011531	0.011396	0.011396	0.011396
0.011934	0.011564	0.011564	0.011362	0.011463	0.011564	0.011396
0.011396	0.011463	0.011430	0.011530	0.011396	0.011530	0.011597
0.011598	0.011430	0.011430	0.011362	0.011362	0.011531	0.011463
0.011362	0.011463	0.011329	0.011362	0.011497	0.011632	0.011396
0.011363	0.011497	0.011430	0.011329	0.011665	0.011329	0.011497
0.011506	0.011396	0.011698	0.011531	0.011564	0.011430	0.011530
0.011396	0.011665	0.011329	0.011598	0.011396	0.011396	0.011329
0.011497	0.011766	0.011463	0.011530	0.011362	0.011766	0.011396
0.011396	0.011396	0.011430	0.011463	0.011430	0.011530	0.011598
0.011598	0.011564	0.011463	0.011564	0.011463	0.011396	0.011362
0.011396	0.011396	0.011463	0.011396	0.011295	0.011530	0.011396
0.011430	0.011430	0.011564	0.011598	0.011497	0.011362	0.011430
0.011497	0.011430	0.011530	0.011396	0.011530	0.011631	0.011934
0.011430	0.011833	0.011440	0.017937	0.011322	0.011733	0.011793
0.011753	0.011799	0.011479	0.011408	0.011830	0.011820	0.011546
0.011630	0.011732	0.011294	0.011437	0.011333	0.011505	0.011491
0.011670	0.011582	0.011579	0.011193	0.011564	0.011598	0.011601
0.011404	0.011497	0.011564	0.011530	0.011497	0.011497	0.011463
0.011497	0.011430	0.011463	0.011497	0.011463	0.011396	0.011430
0.011430	0.011497	0.011396	0.011497	0.011530	0.011564	0.011463
0.011497	0.011463	0.011799	0.011799	0.011531	0.011565	0.011598
0.011362	0.011362	0.011396	0.011430	0.011362	0.011598	0.011598
0.011631	0.011631	0.011564	0.011665	0.011833	0.011667	0.011430
0.011497	0.011564	0.011530	0.011430	0.011497	0.011463	0.011497
0.011564	0.011564	0.011598	0.011497	0.011397	0.011665	0.011564
0.011501	0.011396	0.011497	0.011598	0.011497	0.011631	0.011665
0.011833	0.011538	0.011699	0.011598	0.011598	0.011765	0.011396
0.011566	0.011464	0.011430	0.011430	0.011497	0.011396	0.011497
0.011530	0.011564	0.011463	0.011497	0.011463	0.011799	0.011799
0.011531	0.011565	0.011631	0.011564	0.012205	0.011632	0.011553
0.011623	0.011581	0.011693	0.011580	0.011691	0.011612	0.011578
0.012160	0.011476	0.011609	0.011545	0.011818	0.011673	0.011707
0.012183	0.011663	0.011500	0.011497	0.011497	0.011530	0.011466
0.011362	0.011329	0.011530	0.011497	0.011500	0.011631	0.011599
0.011463	0.011329	0.011362	0.011463	0.011295	0.011430	0.011329
0.011463	0.011497	0.011396	0.011362	0.011463	0.011396	0.011430
0.011430	0.011362	0.011430	0.011463	0.011362	0.011497	0.011598
0.011497	0.011564	0.011497	0.011497	0.011430	0.011833	0.011463
0.011513	0.011430	0.011665	0.011671	0.011463	0.011530	0.011530
0.011531	0.011396	0.011430	0.011396	0.011564	0.011564	0.011497
0.011497	0.011631	0.011799	0.011530	0.011732	0.011732	0.011598
0.011430	0.011497	0.011531	0.011934	0.011497	0.011463	0.011497
0.011530	0.011463	0.011463	0.011766	0.011631	0.011732	0.011463
0.011564	0.011631	0.011497	0.011564	0.011530	0.011396	0.011732
0.011497	0.011463	0.011463	0.011631	0.011463	0.011564	0.011799

0.011497	0.011876	0.011867	0.011670	0.011342	0.011570	0.011318
0.011555	0.011839	0.011412	0.011596	0.011402	0.011519	0.011485
0.011570	0.011324	0.011394	0.011258	0.011384	0.011462	0.011466
0.011475	0.011336	0.011554	0.011448	0.011499	0.011631	0.011698
0.011698	0.011565	0.011669	0.011670	0.011806	0.011838	0.011497
0.011732	0.011598	0.011463	0.011564	0.011362	0.011598	0.011396
0.011463	0.011463	0.011430	0.011396	0.011430	0.011396	0.011497
0.011479	0.011480	0.011471	0.011505	0.011642	0.011539	0.011607
0.011605	0.011531	0.011319	0.011348	0.011467	0.011636	0.011637
0.011430	0.011501	0.011734	0.011703	0.011632	0.011666	0.011598
0.011530	0.011598	0.011598	0.011497	0.012069	0.011665	0.011732
0.011766	0.011809	0.011470	0.011545	0.011430	0.011631	0.011598
0.011632	0.011665	0.011665	0.011665	0.011808	0.011799	0.011442
0.011331	0.011337	0.011435	0.011400	0.011505	0.011359	0.011672
0.011472	0.011587	0.011346	0.011510	0.011464	0.011539	0.011362
0.011396	0.011396	0.011329	0.011430	0.011631	0.011598	0.011530
0.011631	0.011698	0.011430	0.011430	0.011463	0.011463	0.011466
0.011534	0.011463	0.011463	0.011499	0.011631	0.011698	0.011698
0.011565	0.011669	0.011670	0.011806	0.011838	0.011497	0.011665
0.011598	0.011598	0.011432	0.011617	0.011547	0.011571	0.011446
0.011542	0.011835	0.011568	0.011502	0.011329	0.000000	0.011396
0.011497	0.011497	0.011396	0.011498	0.011598	0.011601	0.011497
0.011396	0.011330	0.011463	0.011396	0.011329	0.011434	0.000000
0.011497	0.011430	0.011463	0.011497	0.011463	0.011396	0.011598
0.011463	0.011497	0.011497	0.011430	0.011497	0.011732	0.011396
0.011430	0.011833	0.011430	0.011396	0.011430	0.011799	0.011403
0.011847	0.011400	0.011461	0.011624	0.011712	0.011635	0.011353
0.011323	0.011598	0.011665	0.011773	0.011633	0.011771	0.011901
0.011801	0.011839	0.011799	0.012086	0.011799	0.012675	0.011835
0.012574	0.012342	0.012476	0.012748	0.011799	0.011497	0.011800
0.012239	0.012174	0.011766	0.011665	0.011733	0.011733	0.011703
0.011598	0.011603	0.011665	0.011632	0.012002	0.012069	0.012070
0.011969	0.012069	0.012173	0.011940	0.012239	0.012246	0.012306
0.012307	0.012341	0.012289	0.012307	0.012309	0.011530	0.011833
0.011901	0.012405	0.011908	0.012136	0.012609	0.011672	0.011565
0.011530	0.011530	0.012002	0.012048	0.012151	0.011866	0.011532
S198	0.011598	0.011598	0.011934	0.011698	0.011698	0.011766
0.011604	0.011536	0.011698	0.011195	0.011262	0.011229	0.011195
0.011228	0.011396	0.011195	0.011228	0.011295	0.011262	0.011228
0.011195	0.011228	0.011329	0.011195	0.011161	0.011228	0.011570
0.011463	0.011228	0.011127	0.011362	0.011228	0.011262	0.011228
0.011262	0.011295	0.011262	0.011262	0.011295	0.011262	0.011430
0.011195	0.011262	0.011295	0.011228	0.011329	0.011362	0.011295
0.011262	0.011430	0.011329	0.011597	0.011262	0.011463	0.011262
0.011228	0.011329	0.011396	0.011463	0.011631	0.011329	0.011430
0.011228	0.011295	0.011262	0.011429	0.011732	0.011262	0.011329
0.011228	0.011329	0.011295	0.011362	0.011295	0.011295	0.011295
0.011195	0.011228	0.011195	0.011295	0.011262	0.011296	0.011362
0.011295	0.011262	0.011262	0.011228	0.011362	0.011127	0.011262
0.011430	0.011362	0.011597	0.011430	0.011262	0.011430	0.011262
0.011431	0.011658	0.011475	0.011177	0.011172	0.011281	0.011375
0.012012	0.011416	0.011184	0.011497	0.011497	0.011295	0.011396
0.011329	0.011295	0.011362	0.011262	0.011362	0.011228	0.011295
0.011262	0.011362	0.011564	0.011430	0.011329	0.011295	0.011161
0.011195	0.011195	0.011195	0.011228	0.011430	0.011396	0.011329
0.011430	0.011497	0.011228	0.011228	0.011262	0.011262	0.011263
0.011331	0.011262	0.011262	0.011096	0.011205	0.011165	0.011262
0.011263	0.011198	0.011245	0.011233	0.011162	0.011197	0.011229
0.011492	0.011267	0.011232	0.011195	0.011199	0.011329	0.011296
0.011363	0.011296	0.011296	0.011396	0.011329	0.011631	0.011497

0.011530	0.011396	0.011262	0.011362	0.011161	0.011396	0.011195
0.011262	0.011262	0.011228	0.011195	0.011228	0.011195	0.011295
0.011195	0.011295	0.011295	0.011195	0.011296	0.011396	0.011398
0.011329	0.011295	0.011195	0.011128	0.011262	0.011195	0.011128
0.011230	0.011329	0.000000	0.011329	0.011161	0.011262	0.011228
0.011262	0.011228	0.011195	0.011228	0.011262	0.011127	0.011195
0.011228	0.011228	0.011295	0.011228	0.011262	0.011295	0.011463
0.011631	0.011564	0.011631	0.011397	0.011631	0.011262	0.011228
0.011263	0.011505	0.011335	0.011163	0.011637	0.011129	0.011257
0.011638	0.011536	0.011430	0.011631	0.011333	0.011497	0.011396
0.011396	0.011564	0.011416	0.011225	0.011270	0.011295	0.011365
0.011463	0.011364	0.011500	0.011500	0.011497	0.011564	0.011670
0.011399	0.011502	0.011500	0.011432	0.011700	0.011161	0.011364
0.011262	0.011262	0.011161	0.011161	0.011161	0.011228	0.011295
0.011161	0.011228	0.011127	0.011295	0.011161	0.011228	0.011295
0.011228	0.011665	0.011564	0.011598	0.011127	0.011228	0.011262
0.011262	0.011228	0.011295	0.011329	0.011195	0.011195	0.011195
0.011732	0.011362	0.011362	0.011161	0.011262	0.011362	0.011195
0.011195	0.011262	0.011228	0.011329	0.011195	0.011329	0.011396
0.011396	0.011228	0.011228	0.011161	0.011161	0.011329	0.011262
0.011161	0.011262	0.011127	0.011161	0.011295	0.011430	0.011195
0.011161	0.011295	0.011228	0.011127	0.011463	0.011127	0.011295
0.011300	0.011195	0.011497	0.011329	0.011362	0.011228	0.011329
0.011195	0.011463	0.011127	0.011396	0.011195	0.011195	0.011127
0.011295	0.011564	0.011262	0.011329	0.011161	0.011564	0.011195
0.011195	0.011195	0.011228	0.011262	0.011228	0.011329	0.011396
0.011396	0.011362	0.011262	0.011363	0.011262	0.011195	0.011161
0.011195	0.011195	0.011262	0.011195	0.011094	0.011329	0.011195
0.011228	0.011228	0.011362	0.011396	0.011295	0.011161	0.011228
0.011295	0.011228	0.011329	0.011195	0.011329	0.011430	0.011732
0.011228	0.011631	0.011233	0.017748	0.011191	0.011366	0.011583
0.011545	0.011588	0.011270	0.011234	0.011664	0.011613	0.011303
0.011440	0.011549	0.011138	0.011186	0.011177	0.011307	0.011232
0.011436	0.011395	0.011357	0.011193	0.011362	0.011396	0.011398
0.011198	0.011362	0.011362	0.011329	0.011295	0.011295	0.011262
0.011295	0.011228	0.011262	0.011295	0.011262	0.011195	0.011228
0.011228	0.011295	0.011195	0.011295	0.011329	0.011362	0.011262
0.011295	0.011262	0.011598	0.011597	0.011329	0.011363	0.011396
0.011161	0.011161	0.011195	0.011195	0.011161	0.011396	0.011396
0.011430	0.011430	0.011362	0.011463	0.011631	0.011464	0.011228
0.011295	0.011362	0.011329	0.011228	0.011295	0.011262	0.011295
0.011363	0.011362	0.011396	0.011295	0.011195	0.011463	0.011362
0.011297	0.011195	0.011295	0.011396	0.011295	0.011430	0.011463
0.011631	0.011333	0.011497	0.011396	0.011396	0.011564	0.011195
0.011363	0.011262	0.011228	0.011228	0.011295	0.011195	0.011295
0.011329	0.011362	0.011262	0.011295	0.011262	0.011598	0.011597
0.011329	0.011363	0.011430	0.011362	0.011969	0.011418	0.011342
0.011412	0.011372	0.011482	0.011372	0.011480	0.011405	0.011371
0.011919	0.011268	0.011403	0.011337	0.011611	0.011468	0.011503
0.011944	0.011399	0.011297	0.011295	0.011295	0.011329	0.011263
0.011161	0.011127	0.011329	0.011295	0.011297	0.011430	0.011397
0.011262	0.011127	0.011161	0.011262	0.011094	0.011228	0.011127
0.011262	0.011295	0.011195	0.011161	0.011262	0.011195	0.011228
0.011228	0.011161	0.011228	0.011262	0.011161	0.011295	0.011396
0.011295	0.011362	0.011295	0.011295	0.011228	0.011631	0.011262
0.011304	0.011228	0.011464	0.011467	0.011262	0.011329	0.011329
0.011329	0.011195	0.011228	0.011195	0.011362	0.011362	0.011295
0.011295	0.011430	0.011597	0.011329	0.011530	0.011530	0.011396
0.011228	0.011295	0.011329	0.011732	0.011295	0.011262	0.011295
0.011329	0.011262	0.011262	0.011564	0.011430	0.011530	0.011262

0.011362	0.011430	0.011295	0.011362	0.011329	0.011195	0.011530
0.011295	0.011262	0.011262	0.011430	0.011262	0.011362	0.011598
0.011295	0.011672	0.011665	0.011467	0.011173	0.011366	0.011215
0.011170	0.011683	0.011201	0.011417	0.011207	0.011366	0.011326
0.011417	0.011126	0.011283	0.011126	0.011168	0.011328	0.011304
0.011324	0.011178	0.011342	0.011272	0.011296	0.011430	0.011497
0.011497	0.011363	0.011466	0.011467	0.011603	0.011626	0.011295
0.011530	0.011396	0.011262	0.011362	0.011161	0.011396	0.011195
0.011262	0.011262	0.011228	0.011195	0.011228	0.011195	0.011295
0.011270	0.011270	0.011266	0.011300	0.011437	0.011333	0.011402
0.011401	0.011329	0.011134	0.011132	0.011263	0.011433	0.011400
0.011228	0.011297	0.011531	0.011500	0.011430	0.011464	0.011396
0.011329	0.011396	0.011396	0.011295	0.011867	0.011463	0.011497
0.011564	0.011630	0.011265	0.011337	0.011228	0.011430	0.011396
0.011430	0.011463	0.011463	0.011463	0.011611	0.011598	0.011234
0.011128	0.011130	0.011231	0.011196	0.011299	0.011135	0.011468
0.011266	0.011367	0.000000	0.011285	0.011262	0.011334	0.011161
0.011195	0.011195	0.011195	0.011228	0.011430	0.011396	0.011329
0.011430	0.011497	0.011228	0.011228	0.011262	0.011262	0.011263
0.011331	0.011262	0.011262	0.011296	0.011430	0.011497	0.011497
0.011363	0.011466	0.011467	0.011603	0.011626	0.011295	0.011463
0.011396	0.011396	0.011229	0.011408	0.011338	0.011367	0.011235
0.011336	0.011561	0.011357	0.011298	0.000000	0.011329	0.011195
0.011295	0.011295	0.011195	0.011296	0.011396	0.011398	0.011295
0.011195	0.011128	0.011262	0.011195	0.011128	0.011230	0.011329
0.011295	0.011228	0.011262	0.011295	0.011262	0.011195	0.011396
0.011262	0.011262	0.011295	0.011228	0.011295	0.011530	0.011195
0.011228	0.011631	0.011228	0.011195	0.011228	0.011597	0.011189
0.011622	0.011188	0.011211	0.011422	0.011508	0.011419	0.011144
0.011165	0.011396	0.011497	0.011603	0.011464	0.011601	0.011733
0.011632	0.011669	0.011631	0.011880	0.011598	0.012472	0.011633
0.012372	0.012139	0.012274	0.012545	0.011597	0.011295	0.011598
0.012002	0.011971	0.011564	0.011463	0.011531	0.011531	0.011500
0.011396	0.011399	0.011463	0.011430	0.011800	0.011867	0.011867
0.011767	0.011867	0.011971	0.011737	0.012003	0.012008	0.012070
0.012070	0.012104	0.012050	0.012071	0.012072	0.011329	0.011598
0.011699	0.012203	0.011670	0.011934	0.012407	0.011468	0.011363
0.011329	0.011329	0.011800	0.011842	0.011940	0.011665	0.011330
S199	0.011799	0.011799	0.012136	0.011900	0.011900	0.011967
0.011808	0.011740	0.011900	0.011396	0.011463	0.011431	0.011396
0.011430	0.011598	0.011396	0.011430	0.011497	0.011463	0.011430
0.011396	0.011430	0.011530	0.011396	0.011363	0.011430	0.011774
0.011665	0.011396	0.011329	0.011564	0.011430	0.011463	0.011430
0.011463	0.011497	0.011463	0.011463	0.011497	0.011463	0.011631
0.011396	0.011463	0.011497	0.011430	0.011530	0.011564	0.011497
0.011463	0.011631	0.011531	0.011799	0.011463	0.011665	0.011463
0.011430	0.011531	0.011598	0.011665	0.011833	0.011530	0.011631
0.011430	0.011497	0.011463	0.011631	0.011934	0.011463	0.011530
0.011430	0.011530	0.011497	0.011564	0.011497	0.011497	0.011497
0.011396	0.011430	0.011396	0.011497	0.011463	0.011498	0.011564
0.011497	0.011463	0.011396	0.011430	0.011564	0.011329	0.011463
0.011631	0.011564	0.011799	0.011631	0.011463	0.011631	0.011463
0.011654	0.011882	0.011664	0.011373	0.011359	0.011502	0.011572
0.012231	0.011620	0.011390	0.011698	0.011698	0.011497	0.011598
0.011530	0.011497	0.011564	0.011463	0.011564	0.011430	0.011497
0.011463	0.011564	0.011766	0.011631	0.011530	0.011497	0.011362
0.011396	0.011396	0.011329	0.011430	0.011631	0.011598	0.011530
0.011631	0.011698	0.011430	0.011430	0.011463	0.011463	0.011466
0.011534	0.011463	0.011463	0.011312	0.011386	0.011376	0.011464
0.011467	0.011404	0.011429	0.011440	0.011366	0.011403	0.011432

0.011708	0.011474	0.011439	0.011396	0.011406	0.011531	0.011498
0.011565	0.011498	0.011497	0.011597	0.011530	0.011833	0.011699
0.011732	0.011598	0.011463	0.011564	0.011362	0.011597	0.011396
0.011463	0.011463	0.011430	0.011396	0.011430	0.011396	0.011497
0.011396	0.011497	0.011497	0.011396	0.011498	0.011598	0.011601
0.000000	0.011497	0.011396	0.011330	0.011463	0.011396	0.011329
0.011434	0.000000	0.011329	0.000000	0.011363	0.011463	0.011430
0.011463	0.011430	0.011396	0.011430	0.011463	0.011329	0.011396
0.011430	0.011430	0.011497	0.011430	0.011463	0.011497	0.011732
0.011833	0.011766	0.011833	0.011600	0.011833	0.011463	0.011430
0.011466	0.011667	0.011563	0.011369	0.011807	0.011333	0.011454
0.011842	0.011740	0.011631	0.011833	0.011538	0.011699	0.011598
0.011598	0.011765	0.011629	0.011514	0.011564	0.011497	0.011568
0.011665	0.011566	0.011703	0.011704	0.011699	0.011766	0.011874
0.011602	0.011706	0.011704	0.011635	0.011902	0.011362	0.011566
0.011463	0.011463	0.011362	0.011362	0.011362	0.011430	0.011497
0.011362	0.011430	0.011329	0.011497	0.011362	0.011430	0.011497
0.011430	0.011866	0.011766	0.011799	0.011329	0.011430	0.011463
0.011463	0.011430	0.011497	0.011530	0.011396	0.011396	0.011396
0.011934	0.011564	0.011564	0.011362	0.011463	0.011564	0.011396
0.011396	0.011463	0.011430	0.011530	0.011396	0.011530	0.011597
0.011598	0.011430	0.011430	0.011362	0.011362	0.011531	0.011463
0.011362	0.011463	0.011329	0.011362	0.011497	0.011632	0.011396
0.011363	0.011497	0.011430	0.011329	0.011665	0.011329	0.011497
0.011506	0.011396	0.011698	0.011531	0.011564	0.011430	0.011530
0.011396	0.011665	0.011329	0.011598	0.011396	0.011396	0.011329
0.011497	0.011766	0.011463	0.011530	0.011362	0.011766	0.011396
0.011396	0.011396	0.011430	0.011463	0.011430	0.011530	0.011598
0.011598	0.011564	0.011463	0.011564	0.011463	0.011396	0.011362
0.011396	0.011396	0.011463	0.011396	0.011295	0.011530	0.011396
0.011430	0.011430	0.011564	0.011598	0.011497	0.011362	0.011430
0.011497	0.011430	0.011530	0.011396	0.011530	0.011631	0.011934
0.011430	0.011833	0.011440	0.017975	0.011322	0.011733	0.011793
0.011752	0.011799	0.011479	0.011408	0.011830	0.011820	0.011546
0.011629	0.011732	0.011294	0.011437	0.011333	0.011505	0.011491
0.011670	0.011582	0.011579	0.011193	0.011564	0.011598	0.011601
0.011404	0.011497	0.011564	0.011530	0.011497	0.011497	0.011463
0.011497	0.011430	0.011463	0.011497	0.011463	0.011396	0.011430
0.011430	0.011497	0.011396	0.011497	0.011530	0.011564	0.011463
0.011497	0.011463	0.011799	0.011799	0.011531	0.011565	0.011598
0.011362	0.011362	0.011396	0.011430	0.011362	0.011598	0.011598
0.011631	0.011631	0.011564	0.011665	0.011833	0.011667	0.011430
0.011497	0.011564	0.011530	0.011430	0.011497	0.011463	0.011497
0.011564	0.011564	0.011598	0.011497	0.011397	0.011665	0.011564
0.011501	0.011396	0.011497	0.011598	0.011497	0.011631	0.011665
0.011833	0.011538	0.011699	0.011598	0.011598	0.011765	0.011396
0.011566	0.011464	0.011430	0.011430	0.011497	0.011396	0.011497
0.011530	0.011564	0.011463	0.011497	0.011463	0.011799	0.011799
0.011531	0.011565	0.011631	0.011564	0.012205	0.011632	0.011553
0.011623	0.011581	0.011693	0.011580	0.011691	0.011612	0.011578
0.012160	0.011476	0.011609	0.011545	0.011818	0.011673	0.011707
0.012183	0.011663	0.011500	0.011497	0.011497	0.011530	0.011466
0.011362	0.011329	0.011530	0.011497	0.011500	0.011631	0.011599
0.011463	0.011329	0.011362	0.011463	0.011295	0.011430	0.011329
0.011463	0.011497	0.011396	0.011362	0.011463	0.011396	0.011430
0.011430	0.011362	0.011430	0.011463	0.011362	0.011497	0.011598
0.011497	0.011564	0.011497	0.011497	0.011430	0.011833	0.011463
0.011513	0.011430	0.011665	0.011671	0.011463	0.011530	0.011530
0.011531	0.011396	0.011430	0.011396	0.011564	0.011564	0.011497
0.011497	0.011631	0.011799	0.011530	0.011732	0.011732	0.011598

0.011430	0.011497	0.011531	0.011934	0.011497	0.011463	0.011497
0.011530	0.011463	0.011463	0.011766	0.011631	0.011732	0.011463
0.011564	0.011631	0.011497	0.011564	0.011530	0.011396	0.011732
0.011497	0.011463	0.011463	0.011631	0.011463	0.011564	0.011799
0.011497	0.011876	0.011867	0.011670	0.011342	0.011570	0.011318
0.011555	0.011878	0.011412	0.011596	0.011402	0.011518	0.011485
0.011570	0.011324	0.011394	0.011258	0.011384	0.011462	0.011466
0.011475	0.011336	0.011554	0.011448	0.011499	0.011631	0.011698
0.011698	0.011565	0.011669	0.011670	0.011806	0.011838	0.011497
0.011732	0.011598	0.011463	0.011564	0.011362	0.011597	0.011396
0.011463	0.011463	0.011430	0.011396	0.011430	0.011396	0.011497
0.011479	0.011480	0.011471	0.011505	0.011642	0.011539	0.011607
0.011605	0.011531	0.011319	0.011348	0.011467	0.011636	0.011637
0.011430	0.011501	0.011734	0.011703	0.011632	0.011666	0.011598
0.011530	0.011598	0.011597	0.011497	0.012069	0.011665	0.011732
0.011766	0.011809	0.011470	0.011545	0.011430	0.011631	0.011598
0.011632	0.011665	0.011665	0.011665	0.011807	0.011799	0.011442
0.011331	0.011337	0.011435	0.011400	0.011505	0.011359	0.011672
0.011472	0.011587	0.011346	0.011510	0.011464	0.011539	0.011362
0.011396	0.011396	0.011329	0.011430	0.011631	0.011598	0.011530
0.011631	0.011698	0.011430	0.011430	0.011463	0.011463	0.011466
0.011534	0.011463	0.011463	0.011499	0.011631	0.011698	0.011698
0.011565	0.011669	0.011670	0.011806	0.011838	0.011497	0.011665
0.011598	0.011598	0.011432	0.011617	0.011547	0.011571	0.011446
0.011542	0.011835	0.011568	0.011502	0.011329	0.000000	0.011396
0.011497	0.011497	0.011396	0.011498	0.011598	0.011601	0.011497
0.011396	0.011330	0.011463	0.011396	0.011329	0.011434	0.000000
0.011497	0.011430	0.011463	0.011497	0.011463	0.011396	0.011598
0.011463	0.011463	0.011497	0.011430	0.011497	0.011732	0.011396
0.011430	0.011833	0.011430	0.011396	0.011430	0.011799	0.011403
0.011847	0.011400	0.011461	0.011624	0.011712	0.011635	0.011353
0.011323	0.011598	0.011698	0.011807	0.011666	0.011805	0.011935
0.011834	0.011872	0.011833	0.012086	0.011799	0.012675	0.011835
0.012574	0.012342	0.012476	0.012748	0.011799	0.011497	0.011800
0.012239	0.012174	0.011766	0.011665	0.011733	0.011733	0.011703
0.011598	0.011603	0.011665	0.011632	0.012002	0.012069	0.012070
0.011969	0.012069	0.012173	0.011940	0.012239	0.012246	0.012306
0.012307	0.012341	0.012289	0.012307	0.012309	0.011530	0.011833
0.011901	0.012405	0.011908	0.012136	0.012609	0.011672	0.011565
0.011530	0.011530	0.012002	0.012048	0.012151	0.011866	0.011532
S200	0.011564	0.011564	0.011900	0.011664	0.011664	0.011732
0.011570	0.011502	0.011732	0.011161	0.011228	0.011195	0.011161
0.011195	0.011362	0.011161	0.011195	0.011262	0.011228	0.011195
0.011161	0.011195	0.011295	0.011161	0.011195	0.011161	0.011535
0.011429	0.011195	0.011094	0.011329	0.011262	0.011228	0.011262
0.011228	0.011329	0.011228	0.011228	0.011262	0.011228	0.011396
0.011161	0.011228	0.011262	0.011195	0.011295	0.011329	0.011262
0.011228	0.011396	0.011295	0.011564	0.011228	0.011429	0.011228
0.011195	0.011295	0.011362	0.011429	0.011597	0.011295	0.011396
0.011262	0.011329	0.011228	0.011463	0.011698	0.011228	0.011295
0.011195	0.011295	0.011262	0.011329	0.011262	0.011262	0.011262
0.011161	0.011195	0.011161	0.011262	0.011228	0.011262	0.011329
0.011262	0.011228	0.011295	0.011195	0.011329	0.011161	0.011228
0.011396	0.011329	0.011564	0.011396	0.011228	0.011463	0.011228
0.011394	0.011620	0.011400	0.011099	0.011098	0.011244	0.011296
0.011975	0.011365	0.011102	0.011463	0.011429	0.011262	0.011362
0.011295	0.011262	0.011329	0.011228	0.011329	0.011262	0.011262
0.011228	0.011329	0.011530	0.011396	0.011295	0.011262	0.011195
0.011161	0.011161	0.011228	0.011195	0.011396	0.011362	0.011295
0.011396	0.011463	0.011195	0.011195	0.011228	0.011228	0.011229

0.011297	0.011228	0.011228	0.011096	0.011133	0.011130	0.011228
0.011230	0.011163	0.011171	0.011198	0.011128	0.011163	0.011195
0.011456	0.011233	0.011267	0.011161	0.011164	0.011296	0.011262
0.011329	0.011262	0.011262	0.011362	0.011295	0.011665	0.011463
0.011497	0.011362	0.011228	0.011329	0.011127	0.011362	0.011161
0.011195	0.011228	0.011195	0.011161	0.011195	0.011161	0.011262
0.011228	0.011329	0.011262	0.011161	0.011262	0.011362	0.011364
0.011363	0.011262	0.011161	0.011094	0.011228	0.011161	0.011094
0.011196	0.011363	0.011161	0.011363	0.000000	0.011228	0.011127
0.011295	0.011195	0.011228	0.011127	0.011161	0.011094	0.011228
0.011262	0.011195	0.011262	0.011195	0.011228	0.011262	0.011497
0.011597	0.011530	0.011598	0.011364	0.011598	0.011228	0.011195
0.011229	0.011505	0.011335	0.011129	0.011603	0.011094	0.011218
0.011603	0.011502	0.011396	0.011597	0.011299	0.011463	0.011430
0.011362	0.011530	0.011380	0.011225	0.011270	0.011262	0.011331
0.011430	0.011330	0.011466	0.011534	0.011463	0.011530	0.011636
0.011365	0.011535	0.011466	0.011398	0.011666	0.011127	0.011330
0.011295	0.011228	0.011127	0.011127	0.011195	0.011195	0.011262
0.011195	0.011262	0.011161	0.011262	0.011195	0.011195	0.011262
0.011195	0.011631	0.011530	0.011564	0.011094	0.011195	0.011228
0.011228	0.011127	0.011262	0.011295	0.011161	0.011161	0.011161
0.011698	0.011329	0.011329	0.011127	0.011228	0.011396	0.011161
0.011161	0.011228	0.011195	0.011362	0.011161	0.011295	0.011429
0.011362	0.011195	0.011195	0.011127	0.011127	0.011295	0.011228
0.011127	0.011228	0.011094	0.011127	0.011262	0.011396	0.011161
0.011128	0.011262	0.011195	0.011094	0.011429	0.011094	0.011262
0.011334	0.011161	0.011463	0.011295	0.011329	0.011195	0.011295
0.011161	0.011429	0.011094	0.011362	0.011161	0.011161	0.011094
0.011329	0.011530	0.011228	0.011295	0.011127	0.011530	0.011161
0.011161	0.011161	0.011195	0.011295	0.011195	0.011295	0.011362
0.011362	0.011262	0.011228	0.011329	0.011295	0.011161	0.011094
0.011161	0.011161	0.011228	0.011161	0.011127	0.011295	0.011161
0.011195	0.011195	0.011329	0.011362	0.011262	0.011195	0.011195
0.011262	0.011195	0.011295	0.011161	0.011295	0.011396	0.011698
0.011195	0.011597	0.011198	0.017711	0.011126	0.011366	0.011548
0.011510	0.011553	0.011217	0.011176	0.011610	0.011578	0.011242
0.011392	0.011426	0.000000	0.011186	0.011138	0.011208	0.011189
0.011389	0.011321	0.011357	0.011126	0.011329	0.011362	0.011365
0.011232	0.011329	0.011329	0.011295	0.011262	0.011262	0.011228
0.011262	0.011195	0.011161	0.011262	0.011228	0.011161	0.011195
0.011195	0.011262	0.011161	0.011262	0.011295	0.011329	0.011228
0.011262	0.011228	0.011564	0.011564	0.011295	0.011329	0.011362
0.011127	0.011127	0.011161	0.011262	0.011127	0.011362	0.011362
0.011396	0.011396	0.011295	0.011429	0.011598	0.011431	0.011262
0.011262	0.011329	0.011362	0.011195	0.011262	0.011228	0.011262
0.011329	0.011329	0.011362	0.011329	0.011229	0.011430	0.011396
0.011263	0.011161	0.011262	0.011362	0.011262	0.011396	0.011429
0.011597	0.011299	0.011463	0.011430	0.011362	0.011530	0.011161
0.011330	0.011228	0.011195	0.011195	0.011262	0.011161	0.011262
0.011295	0.011329	0.011228	0.011262	0.011228	0.011564	0.011564
0.011295	0.011329	0.011362	0.011329	0.011968	0.011382	0.011306
0.011377	0.011338	0.011447	0.011337	0.011445	0.011371	0.011336
0.011919	0.011233	0.011368	0.011302	0.011577	0.011434	0.011537
0.011944	0.011399	0.011500	0.011564	0.011429	0.011429	0.011466
0.011127	0.011295	0.011429	0.011396	0.011398	0.011396	0.011363
0.011228	0.011161	0.011195	0.011228	0.011127	0.011195	0.011094
0.011228	0.011262	0.011161	0.011127	0.011228	0.011161	0.011195
0.011195	0.011127	0.011195	0.011228	0.011127	0.011329	0.011362
0.011262	0.011329	0.011262	0.011262	0.011195	0.011597	0.011228
0.011269	0.011195	0.011430	0.011433	0.011295	0.011295	0.011295

0.011295	0.011228	0.011195	0.011228	0.011329	0.011329	0.011262
0.011262	0.011396	0.011564	0.011228	0.011497	0.011497	0.011362
0.011195	0.011262	0.011295	0.011698	0.011262	0.011295	0.011262
0.011295	0.011228	0.011228	0.011463	0.011463	0.011429	0.011228
0.011329	0.011396	0.011262	0.011329	0.011295	0.011161	0.011497
0.011262	0.011127	0.011228	0.011396	0.011228	0.011329	0.011564
0.011262	0.011637	0.011631	0.011385	0.011117	0.011332	0.011164
0.011170	0.011605	0.011166	0.011328	0.011158	0.011264	0.011273
0.011366	0.000000	0.011227	0.000000	0.011114	0.011261	0.011263
0.011324	0.011178	0.011307	0.011236	0.011262	0.011396	0.011463
0.011463	0.011329	0.011432	0.011433	0.011569	0.011590	0.011262
0.011497	0.011362	0.011228	0.011329	0.011127	0.011362	0.011161
0.011195	0.011228	0.011195	0.011161	0.011195	0.011161	0.011262
0.011235	0.011235	0.011232	0.011266	0.011402	0.011368	0.011368
0.011367	0.011296	0.011097	0.011096	0.011230	0.011399	0.011400
0.011195	0.011263	0.011397	0.011466	0.011397	0.011430	0.011362
0.011295	0.011362	0.011362	0.011262	0.011833	0.011362	0.011497
0.011430	0.011594	0.011231	0.011303	0.011195	0.011396	0.011362
0.011397	0.011429	0.011429	0.011429	0.011571	0.011564	0.011199
0.011094	0.011095	0.011196	0.011162	0.011265	0.011098	0.011434
0.011232	0.011367	0.011108	0.011248	0.011228	0.011300	0.011195
0.011161	0.011161	0.011228	0.011195	0.011396	0.011362	0.011295
0.011396	0.011463	0.011195	0.011195	0.011228	0.011228	0.011229
0.011297	0.011228	0.011228	0.011262	0.011396	0.011463	0.011463
0.011329	0.011432	0.011433	0.011569	0.011590	0.011262	0.011429
0.011362	0.011362	0.011196	0.011373	0.011303	0.011333	0.011200
0.011370	0.011561	0.011357	0.011264	0.011161	0.011363	0.011228
0.011329	0.011262	0.011161	0.011262	0.011362	0.011364	0.011262
0.011161	0.011094	0.011228	0.011161	0.011094	0.011196	0.011363
0.011262	0.011195	0.011161	0.011262	0.011228	0.011161	0.011362
0.011228	0.011262	0.011262	0.011195	0.011262	0.011497	0.011161
0.011195	0.011597	0.011195	0.011228	0.011195	0.011564	0.011103
0.011566	0.011103	0.011161	0.011342	0.011427	0.011383	0.000000
0.011113	0.011362	0.011463	0.011569	0.011431	0.011568	0.011699
0.011599	0.011635	0.011597	0.011846	0.011564	0.012438	0.011599
0.012338	0.012106	0.012240	0.012511	0.011564	0.011262	0.011564
0.012002	0.011937	0.011530	0.011430	0.011497	0.011497	0.011466
0.011362	0.011365	0.011429	0.011396	0.011766	0.011833	0.011834
0.011733	0.011834	0.011937	0.011703	0.012003	0.012008	0.012070
0.012071	0.012104	0.012050	0.012071	0.012072	0.011295	0.011598
0.011666	0.012169	0.011670	0.011900	0.012373	0.011434	0.011329
0.011295	0.011295	0.011766	0.011801	0.011905	0.011631	0.011296
S201 0.011665	0.011665	0.012001	0.011766	0.011766	0.011833	
0.011672	0.011604	0.011766	0.011262	0.011329	0.011296	0.011262
0.011295	0.011463	0.011262	0.011295	0.011362	0.011329	0.011295
0.011262	0.011295	0.011396	0.011262	0.011296	0.011295	0.011638
0.011530	0.011295	0.011195	0.011430	0.011362	0.011329	0.011363
0.011329	0.011430	0.011329	0.011329	0.011362	0.011329	0.011497
0.011262	0.011329	0.011362	0.011295	0.011396	0.011430	0.011362
0.011329	0.011497	0.011396	0.011665	0.011329	0.011598	0.011329
0.011295	0.011396	0.011463	0.011598	0.011698	0.011396	0.011497
0.011362	0.011430	0.011329	0.011564	0.011799	0.011329	0.011396
0.011295	0.011396	0.011362	0.011430	0.011362	0.011362	0.011362
0.011262	0.011295	0.011262	0.011363	0.011329	0.011363	0.011430
0.011228	0.011329	0.011396	0.011295	0.011430	0.011262	0.011329
0.011497	0.011430	0.011665	0.011497	0.011329	0.011564	0.011329
0.011468	0.011695	0.011513	0.011216	0.011210	0.011318	0.011415
0.012048	0.011467	0.011225	0.011564	0.011564	0.011362	0.011463
0.011396	0.011362	0.011430	0.011329	0.011430	0.011362	0.011362
0.011329	0.011429	0.011631	0.011497	0.011262	0.011363	0.011295

0.011262	0.011262	0.011329	0.011295	0.011497	0.011463	0.011396
0.011497	0.011564	0.011295	0.011295	0.011329	0.011329	0.011330
0.011398	0.011329	0.011329	0.011168	0.011277	0.011236	0.011329
0.011331	0.011266	0.011282	0.011267	0.011230	0.011266	0.011297
0.011528	0.011336	0.011370	0.011262	0.011233	0.011397	0.011363
0.011430	0.011364	0.011363	0.011463	0.011396	0.011766	0.011564
0.011597	0.011463	0.011329	0.011430	0.011228	0.011463	0.011262
0.011329	0.011329	0.011295	0.011262	0.011295	0.011262	0.011362
0.011329	0.011430	0.011363	0.011262	0.011363	0.011463	0.011466
0.011463	0.011363	0.011262	0.011195	0.011329	0.011262	0.011195
0.011264	0.011463	0.011262	0.011463	0.011228	0.000000	0.011295
0.011396	0.011295	0.011329	0.011295	0.011329	0.011195	0.011329
0.011363	0.011295	0.011362	0.011295	0.011329	0.011362	0.011597
0.011698	0.011631	0.011698	0.011465	0.011699	0.011329	0.011295
0.011330	0.011626	0.011426	0.011232	0.011705	0.011197	0.011336
0.011706	0.011604	0.011497	0.011699	0.011402	0.011564	0.011531
0.011463	0.011631	0.011487	0.011307	0.011354	0.011363	0.011433
0.011530	0.011431	0.011568	0.011636	0.011564	0.011631	0.011738
0.011466	0.011638	0.011568	0.011500	0.011767	0.011228	0.011431
0.011396	0.011329	0.011228	0.011228	0.011295	0.011295	0.011362
0.011295	0.011363	0.011262	0.011362	0.011295	0.011295	0.011362
0.011295	0.011732	0.011631	0.011665	0.011195	0.011295	0.011329
0.011195	0.011295	0.011363	0.011396	0.011262	0.011262	0.011262
0.011799	0.011430	0.011430	0.011228	0.011329	0.011497	0.011262
0.011262	0.011329	0.011295	0.011463	0.011262	0.011396	0.011530
0.011463	0.011295	0.011295	0.011228	0.011228	0.011396	0.011329
0.011228	0.011329	0.011195	0.011228	0.011363	0.011497	0.011262
0.011228	0.011362	0.011296	0.011195	0.011530	0.011195	0.011362
0.011437	0.011262	0.011564	0.011396	0.011430	0.011295	0.011396
0.011262	0.011598	0.011195	0.011463	0.011262	0.011262	0.011195
0.011430	0.011631	0.011329	0.011396	0.011228	0.011631	0.011262
0.011262	0.011262	0.011295	0.011396	0.011295	0.011396	0.011463
0.011463	0.011430	0.011329	0.011430	0.011396	0.011262	0.011228
0.011262	0.011262	0.011329	0.011262	0.011228	0.011396	0.011262
0.011295	0.011295	0.011430	0.011463	0.011362	0.011295	0.011295
0.011362	0.011295	0.011396	0.011262	0.011396	0.011497	0.011799
0.011295	0.011698	0.011267	0.017785	0.011322	0.011427	0.011618
0.011579	0.011623	0.011322	0.011234	0.011719	0.011647	0.011364
0.011535	0.011549	0.011177	0.011270	0.011255	0.011357	0.011275
0.011529	0.011433	0.011431	0.011193	0.011295	0.011463	0.011466
0.011335	0.011430	0.011430	0.011396	0.011363	0.011363	0.011329
0.011362	0.011295	0.011329	0.011362	0.000000	0.011262	0.011295
0.011295	0.011362	0.011262	0.011363	0.011396	0.011430	0.011329
0.011362	0.011329	0.011665	0.011665	0.011396	0.011430	0.011463
0.011228	0.011228	0.011262	0.011363	0.011228	0.011463	0.011463
0.011497	0.011497	0.011295	0.011530	0.011698	0.011532	0.011363
0.011362	0.011430	0.011463	0.011295	0.011362	0.011329	0.011363
0.011430	0.011430	0.011463	0.011430	0.011330	0.011530	0.011497
0.011365	0.011262	0.011362	0.011463	0.011363	0.011497	0.011530
0.011699	0.011402	0.011564	0.011531	0.011463	0.011631	0.011262
0.011431	0.011329	0.011295	0.011295	0.011362	0.011262	0.011363
0.011396	0.011430	0.011329	0.011362	0.011329	0.011665	0.011665
0.011396	0.011430	0.011497	0.011430	0.012070	0.011489	0.011412
0.011482	0.011442	0.011552	0.011441	0.011550	0.011474	0.011440
0.012022	0.011337	0.011471	0.011406	0.011680	0.011537	0.011639
0.012047	0.011513	0.011365	0.011362	0.011362	0.011396	0.011331
0.011228	0.011262	0.011396	0.011362	0.011364	0.011497	0.011464
0.011329	0.011262	0.011295	0.011329	0.011228	0.011295	0.011195
0.011329	0.011362	0.011262	0.011228	0.011329	0.011262	0.011295
0.011295	0.011228	0.011295	0.011329	0.011228	0.011430	0.011463

0.011363	0.011430	0.011362	0.011363	0.011295	0.011698	0.011329
0.011373	0.011295	0.011531	0.011535	0.011396	0.011396	0.011396
0.011396	0.011329	0.011295	0.011329	0.011430	0.011430	0.011228
0.011362	0.011497	0.011665	0.011396	0.011598	0.011598	0.011463
0.011295	0.011362	0.011396	0.011799	0.011363	0.011396	0.011228
0.011396	0.011329	0.011329	0.011631	0.011564	0.011598	0.011329
0.011430	0.011497	0.011362	0.011430	0.011396	0.011262	0.011598
0.011362	0.011329	0.011329	0.011497	0.011329	0.011430	0.011665
0.011362	0.011740	0.011732	0.011507	0.011286	0.011400	0.011267
0.011225	0.011722	0.011236	0.011462	0.011304	0.011417	0.011432
0.011519	0.011258	0.011394	0.011258	0.011276	0.011462	0.011385
0.011399	0.011296	0.011377	0.011307	0.011364	0.011497	0.011564
0.011564	0.011430	0.011534	0.011535	0.011670	0.011696	0.011363
0.011597	0.011463	0.011329	0.011430	0.011228	0.011463	0.011262
0.011329	0.011329	0.011295	0.011262	0.011295	0.011262	0.011362
0.011340	0.011340	0.011300	0.011368	0.011505	0.011470	0.011470
0.011469	0.011396	0.011171	0.011168	0.011331	0.011501	0.011501
0.011296	0.011365	0.011599	0.011567	0.011497	0.011531	0.011463
0.011396	0.011463	0.011463	0.011362	0.011934	0.011530	0.011598
0.011631	0.011701	0.011333	0.011303	0.011295	0.011497	0.011463
0.011498	0.011530	0.011530	0.011530	0.011689	0.011665	0.011303
0.011196	0.011199	0.011299	0.011264	0.011368	0.011210	0.011536
0.011335	0.011499	0.011251	0.011360	0.011329	0.011402	0.011295
0.011262	0.011262	0.011329	0.011295	0.011497	0.011463	0.011396
0.011497	0.011564	0.011295	0.011295	0.011329	0.011329	0.011330
0.011398	0.011329	0.011329	0.011364	0.011497	0.011564	0.011564
0.011430	0.011534	0.011535	0.011670	0.011696	0.011363	0.011530
0.011463	0.011463	0.011297	0.011478	0.011408	0.011435	0.011305
0.011473	0.011698	0.011484	0.011366	0.011262	0.011463	0.011329
0.011430	0.011363	0.011262	0.011363	0.011463	0.011466	0.011363
0.011262	0.011195	0.011329	0.011262	0.011195	0.011264	0.011463
0.011362	0.011295	0.011329	0.011362	0.000000	0.011262	0.011463
0.011329	0.011363	0.011362	0.011295	0.011362	0.011598	0.011262
0.011295	0.011698	0.011295	0.011329	0.011295	0.011665	0.011232
0.011735	0.011230	0.011311	0.011463	0.011549	0.011455	0.011186
0.011270	0.011463	0.011564	0.011671	0.011532	0.011669	0.011800
0.011700	0.011737	0.011698	0.011949	0.011665	0.012540	0.011700
0.012439	0.012207	0.012341	0.012613	0.011665	0.011363	0.011665
0.012104	0.012038	0.011631	0.011531	0.011598	0.011598	0.011568
0.011463	0.011467	0.011530	0.011497	0.011867	0.011934	0.011935
0.011834	0.011935	0.012038	0.011804	0.012104	0.012110	0.012171
0.012172	0.012206	0.012118	0.012172	0.012173	0.011396	0.011699
0.011767	0.012270	0.011772	0.012001	0.012474	0.011536	0.011430
0.011396	0.011396	0.011867	0.011883	0.011975	0.011732	0.011397
S202 0.011631	0.011631	0.011967	0.011732	0.011732	0.011799	
0.011638	0.011570	0.011732	0.011228	0.011295	0.011262	0.011228
0.011262	0.011430	0.011228	0.011262	0.011329	0.011295	0.011262
0.011228	0.011262	0.011362	0.011228	0.011262	0.011262	0.011604
0.011497	0.011262	0.011161	0.011396	0.011329	0.011295	0.011329
0.011295	0.011396	0.011295	0.011295	0.011329	0.011295	0.011463
0.011228	0.011295	0.011329	0.011262	0.011362	0.011396	0.011329
0.011295	0.011463	0.011363	0.011631	0.011295	0.011497	0.011295
0.011262	0.011362	0.011430	0.011497	0.011665	0.011362	0.011463
0.011329	0.011396	0.011295	0.011530	0.011766	0.011295	0.011362
0.011262	0.011362	0.011329	0.011396	0.011329	0.011329	0.011329
0.011228	0.011262	0.011228	0.011329	0.011295	0.011329	0.011396
0.011329	0.011295	0.011362	0.011262	0.011329	0.011228	0.011295
0.011463	0.011396	0.011631	0.011463	0.011295	0.011530	0.011295
0.011468	0.011695	0.011475	0.011138	0.011172	0.011318	0.011375
0.012048	0.011416	0.011184	0.011530	0.011530	0.011329	0.011430

0.011362	0.011329	0.011396	0.011295	0.011396	0.011329	0.011329
0.011295	0.011396	0.011597	0.011463	0.011362	0.011329	0.011262
0.011228	0.011228	0.011295	0.011262	0.011463	0.011429	0.011362
0.011463	0.011530	0.011262	0.011262	0.011295	0.011295	0.011297
0.011364	0.011295	0.011295	0.011168	0.011205	0.011200	0.011296
0.011297	0.011232	0.011245	0.011267	0.011196	0.011232	0.011263
0.011456	0.011302	0.011336	0.011228	0.011233	0.011363	0.011329
0.011397	0.011330	0.011329	0.011429	0.011362	0.011732	0.011530
0.011564	0.011430	0.011295	0.011396	0.011195	0.011429	0.011228
0.011295	0.011295	0.011262	0.011228	0.011262	0.011228	0.011329
0.011295	0.011396	0.011329	0.011228	0.011330	0.011430	0.011432
0.011430	0.011329	0.011228	0.011161	0.011295	0.011228	0.011161
0.011264	0.011430	0.011228	0.011430	0.011127	0.011295	0.000000
0.011362	0.011262	0.011295	0.011195	0.011228	0.011161	0.011295
0.011329	0.011262	0.011329	0.011262	0.011295	0.011329	0.011564
0.011665	0.011597	0.011665	0.011431	0.011665	0.011295	0.011262
0.011297	0.011586	0.011380	0.011197	0.011671	0.011163	0.011296
0.011671	0.011502	0.011463	0.011665	0.011368	0.011530	0.011497
0.011430	0.011597	0.011451	0.011266	0.011312	0.011329	0.011399
0.011497	0.011398	0.011534	0.011602	0.011530	0.011598	0.011704
0.011433	0.011603	0.011534	0.011466	0.011733	0.011195	0.011397
0.011362	0.011295	0.011195	0.011195	0.011262	0.011262	0.011329
0.011262	0.011329	0.011228	0.011329	0.011262	0.011262	0.011329
0.011262	0.011698	0.011598	0.011631	0.011161	0.011262	0.011295
0.011295	0.011195	0.011329	0.011362	0.011228	0.011228	0.011228
0.011765	0.011396	0.011396	0.011195	0.011295	0.011463	0.011228
0.011228	0.011295	0.011262	0.011430	0.011228	0.011362	0.011497
0.011430	0.011262	0.011262	0.011195	0.011195	0.011362	0.011228
0.011195	0.011228	0.011161	0.011195	0.011329	0.011463	0.011228
0.011195	0.011329	0.011262	0.011161	0.011497	0.011161	0.011329
0.011403	0.011228	0.011530	0.011363	0.011396	0.011262	0.011362
0.011228	0.011497	0.011161	0.011430	0.011228	0.011228	0.011161
0.011396	0.011531	0.011295	0.011362	0.011195	0.011598	0.011228
0.011228	0.011228	0.011262	0.011362	0.011262	0.011362	0.011430
0.011430	0.011329	0.011295	0.011396	0.011362	0.011228	0.011195
0.011228	0.011228	0.011295	0.011228	0.011195	0.011362	0.011228
0.011262	0.011262	0.011396	0.011430	0.011329	0.011262	0.011262
0.011329	0.011262	0.011362	0.011228	0.011362	0.011463	0.011765
0.011262	0.011665	0.011267	0.017785	0.011191	0.011427	0.011618
0.011579	0.011623	0.011322	0.011292	0.011664	0.011647	0.011303
0.011440	0.011487	0.011138	0.011270	0.011216	0.011258	0.011232
0.011436	0.011395	0.011431	0.011193	0.011396	0.011430	0.011432
0.011301	0.011396	0.011396	0.011362	0.011329	0.011329	0.011295
0.011329	0.011262	0.011228	0.011329	0.011295	0.011228	0.011262
0.011262	0.011329	0.011228	0.011329	0.011362	0.011396	0.011295
0.011329	0.011295	0.011631	0.011631	0.011363	0.011397	0.011430
0.011195	0.011195	0.011228	0.011329	0.011195	0.011430	0.011430
0.011463	0.011463	0.011396	0.011497	0.011665	0.011498	0.011329
0.011329	0.011396	0.011430	0.011262	0.011329	0.011295	0.011329
0.011396	0.011396	0.011429	0.011396	0.011296	0.011497	0.011463
0.011331	0.011228	0.011329	0.011430	0.011329	0.011463	0.011497
0.011665	0.011368	0.011530	0.011497	0.011430	0.011597	0.011228
0.011397	0.011295	0.011262	0.011262	0.011329	0.011228	0.011329
0.011362	0.011396	0.011295	0.011329	0.011295	0.011631	0.011631
0.011363	0.011397	0.011463	0.011396	0.012036	0.011453	0.011377
0.011447	0.011407	0.011517	0.011407	0.011515	0.011440	0.011405
0.011988	0.011303	0.011437	0.011372	0.011646	0.011502	0.011605
0.012012	0.011437	0.011331	0.011329	0.011329	0.011362	0.011297
0.011195	0.011228	0.011362	0.011329	0.011331	0.011463	0.011431
0.011295	0.011228	0.011262	0.011295	0.011195	0.011262	0.011161

0.011295	0.011329	0.011228	0.011195	0.011295	0.011228	0.011262
0.011262	0.011195	0.011262	0.011295	0.011195	0.011396	0.011430
0.011329	0.011396	0.011329	0.011329	0.011262	0.011665	0.011295
0.011339	0.011262	0.011497	0.011501	0.011363	0.011362	0.011362
0.011362	0.011295	0.011262	0.011295	0.011396	0.011396	0.011329
0.011329	0.011463	0.011631	0.011295	0.011564	0.011564	0.011429
0.011262	0.011329	0.011362	0.011766	0.011329	0.011363	0.011329
0.011362	0.011295	0.011295	0.011530	0.011530	0.011497	0.011295
0.011396	0.011463	0.011329	0.011396	0.011362	0.011228	0.011564
0.011329	0.011295	0.011295	0.011463	0.011295	0.011396	0.011631
0.011329	0.011706	0.011698	0.011467	0.011173	0.011400	0.011215
0.011225	0.011683	0.011236	0.011417	0.011207	0.011315	0.011326
0.011468	0.011126	0.011283	0.011126	0.011168	0.011328	0.011344
0.011399	0.011257	0.011377	0.011307	0.011330	0.011463	0.011530
0.011530	0.011396	0.011500	0.011501	0.011636	0.011661	0.011329
0.011564	0.011430	0.011295	0.011396	0.011195	0.011429	0.011228
0.011295	0.011295	0.011262	0.011228	0.011262	0.011228	0.011329
0.011305	0.011305	0.011300	0.011334	0.011471	0.011436	0.011436
0.011435	0.011363	0.011171	0.011168	0.011297	0.011467	0.011467
0.011262	0.011331	0.011565	0.011533	0.011464	0.011498	0.011429
0.011362	0.011430	0.011429	0.011329	0.011900	0.011430	0.011564
0.011597	0.011666	0.011299	0.011372	0.011262	0.011463	0.011430
0.011464	0.011497	0.011497	0.011497	0.011650	0.011631	0.011268
0.011162	0.011164	0.011265	0.011230	0.011334	0.011135	0.011502
0.011300	0.011411	0.011156	0.011285	0.011296	0.011368	0.011262
0.011228	0.011228	0.011295	0.011262	0.011463	0.011429	0.011362
0.011463	0.011530	0.011262	0.011262	0.011295	0.011295	0.011297
0.011364	0.011295	0.011295	0.011330	0.011463	0.011530	0.011530
0.011396	0.011500	0.011501	0.011636	0.011661	0.011329	0.011497
0.011430	0.011430	0.011263	0.011443	0.011373	0.011401	0.011270
0.011439	0.011653	0.011441	0.011332	0.011228	0.011430	0.011295
0.011396	0.011329	0.011228	0.011330	0.011430	0.011432	0.011329
0.011228	0.011161	0.011295	0.011228	0.011161	0.011264	0.011430
0.011329	0.011262	0.011228	0.011329	0.011295	0.011228	0.011430
0.011295	0.011329	0.011329	0.011262	0.011329	0.011564	0.011228
0.011262	0.011665	0.011262	0.011295	0.011262	0.011631	0.011189
0.011678	0.011188	0.011211	0.011422	0.011508	0.011455	0.011102
0.011165	0.011429	0.011530	0.011637	0.011498	0.011635	0.011766
0.011666	0.011703	0.011665	0.011914	0.011631	0.012506	0.011667
0.012406	0.012173	0.012307	0.012579	0.011631	0.011329	0.011631
0.012070	0.012005	0.011597	0.011497	0.011565	0.011565	0.011534
0.011429	0.011433	0.011497	0.011464	0.011834	0.011900	0.011901
0.011801	0.011901	0.012004	0.011770	0.012070	0.012076	0.012137
0.012138	0.012172	0.012118	0.012138	0.012139	0.011362	0.011665
0.011733	0.012237	0.011738	0.011968	0.012440	0.011502	0.011396
0.011362	0.011362	0.011833	0.011842	0.011975	0.011698	0.011363
S203	0.011732	0.011732	0.012068	0.011833	0.011833	0.011900
0.011706	0.011638	0.011833	0.011329	0.011396	0.011363	0.011329
0.011362	0.011530	0.011329	0.011362	0.011430	0.011396	0.011362
0.011329	0.011362	0.011463	0.011329	0.011295	0.011362	0.011672
0.011597	0.011362	0.011262	0.011497	0.011362	0.011396	0.011362
0.011396	0.011430	0.011396	0.011396	0.011430	0.011396	0.011564
0.011329	0.011396	0.011430	0.011362	0.011463	0.011497	0.011430
0.011396	0.011564	0.011463	0.011732	0.011396	0.011597	0.011396
0.011362	0.011463	0.011530	0.011597	0.011766	0.011463	0.011564
0.011362	0.011430	0.011396	0.011564	0.011867	0.011396	0.011463
0.011362	0.011463	0.011430	0.011497	0.011430	0.011430	0.011430
0.011329	0.011362	0.011329	0.011430	0.011396	0.011430	0.011497
0.011430	0.011396	0.011396	0.011362	0.011497	0.011262	0.011396
0.011564	0.011497	0.011732	0.011564	0.011329	0.011564	0.011396

0.011579	0.011807	0.011626	0.011334	0.011321	0.011428	0.011533
0.012158	0.011569	0.011349	0.011631	0.011631	0.011429	0.011530
0.011463	0.011430	0.011497	0.011396	0.011497	0.011362	0.011429
0.011396	0.011497	0.011698	0.011564	0.011463	0.011430	0.011295
0.011329	0.011329	0.011329	0.011362	0.011564	0.011530	0.011463
0.011564	0.011631	0.011362	0.011362	0.011396	0.011396	0.011398
0.011466	0.011329	0.011396	0.011240	0.011349	0.011306	0.011396
0.011399	0.011335	0.011392	0.011371	0.011298	0.011334	0.011364
0.011636	0.011405	0.011370	0.011329	0.011337	0.011464	0.011430
0.011497	0.011431	0.011430	0.011530	0.011463	0.011766	0.011631
0.011665	0.011530	0.011396	0.011497	0.011295	0.011530	0.011329
0.011396	0.011396	0.011362	0.011329	0.011362	0.011329	0.011430
0.011329	0.011429	0.011430	0.011329	0.011431	0.011530	0.011533
0.011463	0.011430	0.011329	0.011263	0.011396	0.011329	0.011262
0.011366	0.011463	0.011262	0.011463	0.011295	0.011396	0.011362
0.000000	0.011362	0.011262	0.011362	0.011396	0.011262	0.011329
0.011362	0.011362	0.011430	0.011362	0.011396	0.011430	0.011665
0.011766	0.011698	0.011766	0.011532	0.011766	0.011396	0.011362
0.011398	0.011586	0.011289	0.011300	0.011773	0.011265	0.011375
0.011739	0.011672	0.011564	0.011766	0.011470	0.011631	0.011530
0.011530	0.011698	0.011558	0.011431	0.011480	0.011430	0.011500
0.011598	0.011499	0.011568	0.011636	0.011631	0.011699	0.011738
0.011534	0.011637	0.011636	0.011568	0.011834	0.011295	0.011498
0.011329	0.011396	0.011295	0.011295	0.011295	0.011362	0.011430
0.011295	0.011362	0.011262	0.011430	0.011295	0.011362	0.011429
0.011362	0.011799	0.011698	0.011732	0.011262	0.011362	0.011396
0.011396	0.011362	0.011430	0.011463	0.011329	0.011329	0.011329
0.011866	0.011497	0.011497	0.011295	0.011396	0.011497	0.011329
0.011329	0.011396	0.011362	0.011463	0.011329	0.011463	0.011530
0.011530	0.011362	0.011362	0.011295	0.011295	0.011463	0.011396
0.011295	0.011396	0.011262	0.011295	0.011430	0.011564	0.011329
0.011296	0.011430	0.011363	0.011262	0.011597	0.011262	0.011430
0.011437	0.011329	0.011631	0.011463	0.011497	0.011362	0.011463
0.011329	0.011597	0.011262	0.011530	0.011329	0.011329	0.011262
0.011429	0.011699	0.011396	0.011463	0.011295	0.011698	0.011329
0.011329	0.011329	0.011362	0.011396	0.011362	0.011463	0.011530
0.011530	0.011497	0.011396	0.011497	0.011396	0.011329	0.011295
0.011329	0.011329	0.011396	0.011329	0.011228	0.011463	0.011329
0.011362	0.011362	0.011497	0.011530	0.011430	0.011295	0.011363
0.011430	0.011362	0.011463	0.011329	0.011463	0.011564	0.011866
0.011362	0.011766	0.011371	0.017899	0.011322	0.011672	0.011723
0.011683	0.011729	0.011427	0.011408	0.011774	0.011751	0.011425
0.011582	0.011732	0.011255	0.011312	0.011294	0.011455	0.011404
0.011576	0.011544	0.011468	0.011126	0.011497	0.011530	0.011534
0.011335	0.011497	0.011497	0.011463	0.011430	0.011430	0.011396
0.011430	0.011362	0.011396	0.011430	0.011396	0.011329	0.011362
0.011362	0.011430	0.011329	0.011430	0.011463	0.011497	0.011396
0.011430	0.011396	0.011732	0.011732	0.011463	0.011497	0.011530
0.011295	0.011295	0.011329	0.011362	0.011295	0.011530	0.011530
0.011564	0.011564	0.011497	0.011597	0.011766	0.011599	0.011362
0.011430	0.011497	0.011463	0.011362	0.011430	0.011396	0.011430
0.011497	0.011497	0.011530	0.011430	0.011330	0.011597	0.011497
0.011433	0.011329	0.011430	0.011530	0.011430	0.011564	0.011597
0.011766	0.011470	0.011631	0.011530	0.011530	0.011698	0.011329
0.011498	0.011396	0.011362	0.011362	0.011430	0.011329	0.011430
0.011463	0.011497	0.011396	0.011430	0.011396	0.011732	0.011732
0.011463	0.011497	0.011564	0.011497	0.012137	0.011561	0.011482
0.011553	0.011511	0.011623	0.011511	0.011550	0.011543	0.011509
0.012091	0.011406	0.011540	0.011475	0.011749	0.011605	0.011639
0.012115	0.011588	0.011432	0.011430	0.011429	0.011463	0.011398

0.011295	0.011262	0.011463	0.011430	0.011432	0.011564	0.011532
0.011396	0.011262	0.011295	0.011396	0.011228	0.011362	0.011262
0.011396	0.011430	0.011329	0.011295	0.011396	0.011329	0.011362
0.011362	0.011295	0.011362	0.011396	0.011295	0.011430	0.011530
0.011430	0.011497	0.011430	0.011430	0.011362	0.011766	0.011396
0.011443	0.011362	0.011598	0.011603	0.011396	0.011463	0.011463
0.011463	0.011329	0.011362	0.011329	0.011497	0.011497	0.011430
0.011430	0.011564	0.011732	0.011463	0.011665	0.011665	0.011530
0.011362	0.011430	0.011463	0.011867	0.011430	0.011396	0.011430
0.011463	0.011396	0.011396	0.011699	0.011564	0.011665	0.011396
0.011497	0.011497	0.011430	0.011497	0.011463	0.011329	0.011665
0.011429	0.011396	0.011396	0.011564	0.011396	0.011497	0.011732
0.011430	0.011774	0.011799	0.011629	0.011342	0.011502	0.011318
0.011445	0.011839	0.011341	0.011552	0.011304	0.011518	0.011326
0.011570	0.011258	0.011394	0.011258	0.011276	0.011462	0.011385
0.011437	0.011296	0.011483	0.011412	0.011431	0.011564	0.011631
0.011631	0.011497	0.011567	0.011569	0.011738	0.011732	0.011430
0.011665	0.011530	0.011396	0.011497	0.011295	0.011530	0.011329
0.011396	0.011396	0.011362	0.011329	0.011362	0.011329	0.011430
0.011410	0.011410	0.011403	0.011437	0.011573	0.011470	0.011538
0.011537	0.011464	0.011282	0.011276	0.011399	0.011501	0.011569
0.011363	0.011433	0.011666	0.011635	0.011565	0.011599	0.011530
0.011463	0.011530	0.011530	0.011429	0.012001	0.011598	0.011665
0.011698	0.011773	0.011401	0.011476	0.011362	0.011564	0.011530
0.011565	0.011597	0.011598	0.011598	0.011768	0.011732	0.011373
0.011263	0.011268	0.011367	0.011332	0.011436	0.011284	0.011604
0.011403	0.011543	0.011251	0.011435	0.011396	0.011471	0.011295
0.011329	0.011329	0.011329	0.011362	0.011564	0.011530	0.011463
0.011564	0.011631	0.011362	0.011362	0.011396	0.011396	0.011398
0.011466	0.011329	0.011396	0.011431	0.011564	0.011631	0.011631
0.011497	0.011567	0.011569	0.011738	0.011732	0.011430	0.011597
0.011530	0.011530	0.011365	0.011513	0.011477	0.011503	0.011376
0.011473	0.011653	0.011526	0.011434	0.011262	0.011463	0.011329
0.011429	0.011430	0.011329	0.011431	0.011530	0.011533	0.011430
0.011329	0.011263	0.011396	0.011329	0.011262	0.011366	0.011463
0.011430	0.011362	0.011396	0.011430	0.011396	0.011329	0.011530
0.011396	0.011430	0.011430	0.011362	0.011430	0.011665	0.011329
0.011362	0.011766	0.011362	0.011329	0.011362	0.011732	0.011360
0.011791	0.011358	0.011361	0.011584	0.011671	0.011563	0.011311
0.011323	0.011530	0.011631	0.011739	0.011599	0.011737	0.011867
0.011767	0.011804	0.011765	0.011949	0.011732	0.012539	0.011768
0.012507	0.012274	0.012375	0.012680	0.011732	0.011430	0.011732
0.012171	0.012106	0.011698	0.011530	0.011665	0.011665	0.011568
0.011530	0.011535	0.011597	0.011531	0.011935	0.012001	0.012002
0.011902	0.012002	0.012038	0.011872	0.012171	0.012178	0.012238
0.012239	0.012273	0.012221	0.012239	0.012241	0.011463	0.011766
0.011834	0.012338	0.011840	0.012069	0.012541	0.011604	0.011497
0.011463	0.011463	0.011934	0.012006	0.012081	0.011799	0.011465
S204 0.011631	0.011631	0.011967	0.011732	0.011732	0.011799	
0.011604	0.011536	0.011732	0.011228	0.011295	0.011262	0.011228
0.011262	0.011429	0.011228	0.011262	0.011329	0.011295	0.011262
0.011228	0.011262	0.011362	0.011228	0.011262	0.011262	0.011570
0.011497	0.011262	0.011161	0.011396	0.011329	0.011295	0.011329
0.011295	0.011396	0.011295	0.011295	0.011329	0.011295	0.011463
0.011228	0.011295	0.011329	0.011262	0.011362	0.011396	0.011329
0.011295	0.011463	0.011363	0.011631	0.011295	0.011564	0.011295
0.011262	0.011362	0.011429	0.011564	0.011665	0.011362	0.011463
0.011329	0.011396	0.011295	0.011530	0.011765	0.011295	0.011362
0.011262	0.011362	0.011329	0.011396	0.011329	0.011329	0.011329
0.011228	0.011262	0.011228	0.011329	0.011295	0.011329	0.011396

0.011329	0.011295	0.011362	0.011262	0.011396	0.011228	0.011295
0.011463	0.011396	0.011631	0.011463	0.011295	0.011530	0.011295
0.011431	0.011658	0.011475	0.011138	0.011209	0.011318	0.011375
0.012048	0.011365	0.011184	0.011530	0.011530	0.011329	0.011430
0.011362	0.011329	0.011396	0.011295	0.011396	0.011329	0.011262
0.011228	0.011396	0.011597	0.011463	0.011362	0.011329	0.011262
0.011228	0.011228	0.011295	0.011262	0.011463	0.011429	0.011362
0.011463	0.011530	0.011262	0.011262	0.011295	0.011295	0.011297
0.011364	0.011295	0.011295	0.011168	0.011241	0.011200	0.011296
0.011297	0.011232	0.011282	0.011267	0.011196	0.011231	0.011263
0.011528	0.011301	0.011336	0.011228	0.011233	0.011363	0.011329
0.011396	0.011330	0.011329	0.011362	0.011362	0.011732	0.011530
0.011564	0.011430	0.011295	0.011396	0.011195	0.011429	0.011228
0.011295	0.011295	0.011262	0.011228	0.011262	0.011228	0.011329
0.011295	0.011396	0.011329	0.011228	0.011330	0.011430	0.011432
0.011430	0.011329	0.011228	0.011161	0.011295	0.011228	0.011161
0.011264	0.011430	0.011228	0.011430	0.011195	0.011295	0.011262
0.011362	0.000000	0.011295	0.011262	0.011295	0.011161	0.011295
0.011329	0.011262	0.011329	0.011262	0.011228	0.011329	0.011564
0.011665	0.011597	0.011665	0.011431	0.011665	0.011295	0.011262
0.011297	0.011586	0.011426	0.011197	0.011671	0.011163	0.011296
0.011637	0.011570	0.011463	0.011665	0.011368	0.011530	0.011497
0.011429	0.011597	0.011451	0.011225	0.011354	0.011329	0.011399
0.011497	0.011330	0.011534	0.011602	0.011463	0.011598	0.011704
0.011365	0.011603	0.011534	0.011466	0.011733	0.011195	0.011397
0.011362	0.011295	0.011195	0.011195	0.011262	0.011262	0.011329
0.011262	0.011329	0.011228	0.011329	0.011262	0.011262	0.011329
0.011262	0.011698	0.011597	0.011631	0.011161	0.011262	0.011295
0.011295	0.011262	0.011329	0.011362	0.011228	0.011228	0.011228
0.011765	0.011396	0.011396	0.011195	0.011295	0.011463	0.011228
0.011228	0.011295	0.011262	0.011430	0.011228	0.011362	0.011497
0.011429	0.011262	0.011262	0.011195	0.011195	0.011362	0.011295
0.011195	0.011295	0.011161	0.011195	0.011329	0.011463	0.011228
0.011195	0.011329	0.011262	0.011161	0.011497	0.011161	0.011329
0.011403	0.011228	0.011530	0.011362	0.011396	0.011262	0.011362
0.011228	0.011564	0.011161	0.011430	0.011228	0.011228	0.011161
0.011396	0.011598	0.011295	0.011362	0.011195	0.011597	0.011228
0.011228	0.011228	0.011262	0.011362	0.011262	0.011362	0.011429
0.011429	0.011396	0.011295	0.011396	0.011362	0.011228	0.011195
0.011228	0.011228	0.011295	0.011228	0.011195	0.011362	0.011228
0.011262	0.011262	0.011396	0.011429	0.011329	0.011262	0.011262
0.011329	0.011262	0.011362	0.011228	0.011362	0.011463	0.011765
0.011262	0.011665	0.011267	0.017785	0.011191	0.011305	0.011618
0.011579	0.011623	0.011270	0.011176	0.011609	0.011647	0.011242
0.011440	0.011426	0.011138	0.011228	0.011255	0.011258	0.011189
0.011436	0.011433	0.011431	0.011325	0.011396	0.011430	0.011432
0.011301	0.011396	0.011396	0.011362	0.011329	0.011329	0.011295
0.011329	0.000000	0.011295	0.011329	0.011295	0.011228	0.011262
0.011262	0.011329	0.011228	0.011329	0.011362	0.011396	0.011295
0.011329	0.011295	0.011631	0.011631	0.011362	0.011396	0.011430
0.011195	0.011195	0.011228	0.011329	0.011195	0.011429	0.011429
0.011463	0.011463	0.011396	0.011497	0.011665	0.011498	0.011329
0.011329	0.011396	0.011429	0.011262	0.011329	0.011295	0.011329
0.011396	0.011396	0.011429	0.011396	0.011296	0.011497	0.011396
0.011331	0.011228	0.011329	0.011430	0.011329	0.011463	0.011497
0.011665	0.011368	0.011530	0.011497	0.011429	0.011597	0.011228
0.011397	0.011295	0.011262	0.011262	0.011329	0.011228	0.011329
0.011362	0.011396	0.011295	0.011329	0.011295	0.011631	0.011631
0.011362	0.011396	0.011463	0.011396	0.012036	0.011453	0.011377
0.011447	0.011407	0.011517	0.011407	0.011515	0.011440	0.011405

0.011987	0.011303	0.011437	0.011372	0.011646	0.011502	0.011605
0.012012	0.011475	0.011331	0.011329	0.011329	0.011362	0.011297
0.011195	0.011228	0.011362	0.011329	0.011331	0.011463	0.011431
0.011295	0.011228	0.011262	0.011295	0.011195	0.011262	0.011161
0.011295	0.011329	0.011228	0.011195	0.011295	0.011228	0.011262
0.011262	0.011195	0.011262	0.011295	0.011195	0.011396	0.011429
0.011329	0.011396	0.011329	0.011329	0.011262	0.011665	0.011295
0.011339	0.011262	0.011497	0.011501	0.011362	0.011362	0.011362
0.011362	0.011295	0.011262	0.011295	0.011396	0.011396	0.011329
0.011329	0.011463	0.011631	0.011362	0.011564	0.011564	0.011429
0.011262	0.011329	0.011362	0.011765	0.011329	0.011362	0.011329
0.011362	0.011295	0.011295	0.011598	0.011530	0.011564	0.011295
0.011396	0.011463	0.011329	0.011396	0.011362	0.011228	0.011564
0.011329	0.011295	0.011295	0.011463	0.011295	0.011396	0.011631
0.011329	0.011672	0.011698	0.011467	0.011229	0.011400	0.011215
0.011115	0.011683	0.011236	0.011373	0.011255	0.011366	0.011326
0.011417	0.011126	0.011283	0.011126	0.011168	0.011328	0.011344
0.011362	0.011257	0.011377	0.011307	0.011330	0.011463	0.011530
0.011530	0.011396	0.011500	0.011501	0.011636	0.011625	0.011329
0.011564	0.011430	0.011295	0.011396	0.011195	0.011429	0.011228
0.011295	0.011295	0.011262	0.011228	0.011262	0.011228	0.011329
0.011305	0.011305	0.011300	0.011334	0.011471	0.011436	0.011436
0.011435	0.011363	0.011134	0.011132	0.011297	0.011467	0.011467
0.011262	0.011331	0.011565	0.011533	0.011464	0.011498	0.011429
0.011362	0.011429	0.011429	0.011329	0.011900	0.011497	0.011564
0.011597	0.011666	0.011299	0.011372	0.011262	0.011396	0.011430
0.011464	0.011497	0.011497	0.011497	0.011650	0.011631	0.011268
0.011162	0.011164	0.011265	0.011230	0.011334	0.011172	0.011502
0.011300	0.011455	0.011156	0.011285	0.011296	0.011368	0.011262
0.011228	0.011228	0.011295	0.011262	0.011463	0.011429	0.011362
0.011463	0.011530	0.011262	0.011262	0.011295	0.011295	0.011297
0.011364	0.011295	0.011295	0.011330	0.011463	0.011530	0.011530
0.011396	0.011500	0.011501	0.011636	0.011625	0.011329	0.011497
0.011430	0.011430	0.011263	0.011443	0.011373	0.011401	0.011270
0.011439	0.011607	0.011399	0.011332	0.011228	0.011430	0.011295
0.011396	0.011329	0.011228	0.011330	0.011430	0.011432	0.011329
0.011228	0.011161	0.011295	0.011228	0.011161	0.011264	0.011430
0.011329	0.000000	0.011295	0.011329	0.011295	0.011228	0.011430
0.011295	0.011329	0.011329	0.011262	0.011329	0.011564	0.011228
0.011262	0.011665	0.011262	0.011295	0.011262	0.011631	0.011189
0.011678	0.011188	0.011211	0.011463	0.011508	0.011455	0.011144
0.011165	0.011429	0.011530	0.011637	0.011498	0.011635	0.011766
0.011666	0.011703	0.011665	0.011914	0.011631	0.012506	0.011666
0.012405	0.012173	0.012307	0.012578	0.011631	0.011329	0.011631
0.012070	0.012004	0.011597	0.011497	0.011564	0.011564	0.011534
0.011429	0.011433	0.011497	0.011464	0.011833	0.011900	0.011901
0.011800	0.011901	0.012004	0.011770	0.012070	0.012076	0.012137
0.012138	0.012171	0.012050	0.012138	0.012139	0.011362	0.011665
0.011733	0.012236	0.011738	0.011967	0.012440	0.011502	0.011396
0.011362	0.011362	0.011833	0.011842	0.011975	0.011698	0.011363
S205	0.011665	0.011665	0.012001	0.011765	0.011765	0.011833
0.011638	0.011570	0.011765	0.011262	0.011329	0.011296	0.011262
0.011295	0.011463	0.011262	0.011295	0.011362	0.011329	0.011295
0.011262	0.011295	0.011396	0.011262	0.011228	0.011295	0.011604
0.011530	0.011295	0.011195	0.011429	0.011295	0.011329	0.011295
0.011329	0.011362	0.011329	0.011329	0.011362	0.011329	0.011497
0.011262	0.011329	0.011362	0.011295	0.011396	0.011430	0.011362
0.011329	0.011497	0.011396	0.011665	0.011329	0.011530	0.011329
0.011295	0.011396	0.011463	0.011530	0.011698	0.011396	0.011497
0.011295	0.011362	0.011329	0.011497	0.011799	0.011329	0.011396

0.011295	0.011396	0.011362	0.011430	0.011362	0.011362	0.011362
0.011262	0.011295	0.011262	0.011362	0.011329	0.011363	0.011429
0.011362	0.011329	0.011329	0.011295	0.011429	0.011195	0.011329
0.011497	0.011429	0.011665	0.011497	0.011262	0.011497	0.011329
0.011505	0.011733	0.011551	0.011255	0.011247	0.011355	0.011454
0.012085	0.011518	0.011266	0.011564	0.011564	0.011362	0.011463
0.011396	0.011362	0.011429	0.011329	0.011430	0.011295	0.011362
0.011329	0.011429	0.011631	0.011497	0.011396	0.011363	0.011228
0.011262	0.011262	0.011262	0.011295	0.011497	0.011463	0.011396
0.011497	0.011564	0.011295	0.011295	0.011329	0.011329	0.011330
0.011398	0.011262	0.011329	0.011168	0.011277	0.011236	0.011329
0.011331	0.011266	0.011318	0.011302	0.011230	0.011266	0.011297
0.011564	0.011336	0.011301	0.011262	0.011268	0.011397	0.011363
0.011430	0.011364	0.011363	0.011463	0.011396	0.011698	0.011564
0.011597	0.011463	0.011329	0.011430	0.011228	0.011463	0.011262
0.011329	0.011329	0.011295	0.011262	0.011295	0.011262	0.011362
0.011262	0.011362	0.011362	0.011262	0.011363	0.011463	0.011466
0.011396	0.011362	0.011262	0.011195	0.011329	0.011262	0.011195
0.011298	0.011396	0.011195	0.011396	0.011228	0.011329	0.011295
0.011262	0.011295	0.000000	0.011295	0.011329	0.011195	0.011262
0.011295	0.011295	0.011362	0.011295	0.011329	0.011362	0.011597
0.011698	0.011631	0.011698	0.011465	0.011698	0.011329	0.011295
0.011330	0.011586	0.011289	0.011232	0.011705	0.011197	0.011336
0.011671	0.011604	0.011497	0.011698	0.011402	0.011564	0.011463
0.011463	0.011631	0.011487	0.011349	0.011396	0.011363	0.011433
0.011530	0.011431	0.011568	0.011568	0.011564	0.011631	0.011670
0.011466	0.011569	0.011568	0.011500	0.011767	0.011228	0.011431
0.011262	0.011329	0.011228	0.011228	0.011228	0.011295	0.011362
0.011228	0.011295	0.011195	0.011362	0.011228	0.011295	0.011362
0.011295	0.011732	0.011631	0.011665	0.011195	0.011295	0.011329
0.011329	0.011295	0.011362	0.011396	0.011262	0.011262	0.011262
0.011799	0.011430	0.011430	0.011228	0.011329	0.011430	0.011262
0.011262	0.011329	0.011295	0.011396	0.011262	0.011396	0.011463
0.011463	0.011295	0.011295	0.011228	0.011228	0.011396	0.011329
0.011228	0.011329	0.011195	0.011228	0.011362	0.011497	0.011262
0.011228	0.011362	0.011296	0.011195	0.011530	0.011195	0.011362
0.011368	0.011262	0.011564	0.011396	0.011430	0.011295	0.011396
0.011262	0.011530	0.011195	0.011463	0.011262	0.011262	0.011195
0.011362	0.011631	0.011329	0.011396	0.011228	0.011631	0.011262
0.011262	0.011262	0.011295	0.011329	0.011295	0.011396	0.011463
0.011463	0.011430	0.011329	0.011430	0.011329	0.011262	0.011228
0.011262	0.011262	0.011329	0.011262	0.011161	0.011396	0.011262
0.011295	0.011295	0.011430	0.011463	0.011362	0.011228	0.011295
0.011362	0.011295	0.011396	0.011262	0.011396	0.011497	0.011799
0.011295	0.011698	0.011302	0.017785	0.011191	0.011488	0.011653
0.011614	0.011658	0.011322	0.011292	0.011719	0.011682	0.011364
0.011487	0.011610	0.011216	0.011270	0.011216	0.011356	0.011318
0.011529	0.011470	0.011431	0.011193	0.011430	0.011463	0.011466
0.011266	0.011430	0.011430	0.011396	0.011362	0.011363	0.011329
0.011362	0.011295	0.011329	0.011362	0.011329	0.011262	0.011295
0.011295	0.011362	0.011262	0.011363	0.011396	0.011430	0.011329
0.011362	0.011329	0.011665	0.011665	0.011396	0.011430	0.011463
0.011228	0.011228	0.011262	0.011295	0.011228	0.011463	0.011463
0.011497	0.011497	0.011430	0.011530	0.011698	0.011532	0.011295
0.011362	0.011430	0.011396	0.011295	0.011362	0.011329	0.011362
0.011430	0.011430	0.011463	0.011363	0.011262	0.011530	0.011429
0.011365	0.011262	0.011362	0.011463	0.011363	0.011497	0.011530
0.011698	0.011402	0.011564	0.011463	0.011463	0.011631	0.011262
0.011431	0.011329	0.011295	0.011295	0.011362	0.011262	0.011363
0.011396	0.011430	0.011329	0.011362	0.011329	0.011665	0.011665

0.011396	0.011430	0.011497	0.011429	0.012070	0.011489	0.011412
0.011482	0.011442	0.011552	0.011441	0.011550	0.011474	0.011440
0.012022	0.011337	0.011471	0.011406	0.011680	0.011537	0.011571
0.012047	0.011512	0.011365	0.011362	0.011362	0.011396	0.011331
0.011228	0.011195	0.011396	0.011362	0.011364	0.011497	0.011464
0.011329	0.011195	0.011228	0.011329	0.011161	0.011295	0.011195
0.011329	0.011362	0.011262	0.011228	0.011329	0.011262	0.011295
0.011295	0.011228	0.011295	0.011329	0.011228	0.011362	0.011463
0.011362	0.011430	0.011362	0.011362	0.011295	0.011698	0.011329
0.011373	0.011295	0.011531	0.011535	0.011329	0.011396	0.011396
0.011396	0.011262	0.011295	0.011262	0.011430	0.011430	0.011362
0.011362	0.011497	0.011665	0.011396	0.011598	0.011598	0.011463
0.011295	0.011362	0.011396	0.011799	0.011363	0.011329	0.011362
0.011396	0.011329	0.011329	0.011631	0.011497	0.011598	0.011329
0.011430	0.011430	0.011362	0.011430	0.011396	0.011262	0.011597
0.011362	0.011329	0.011329	0.011497	0.011329	0.011430	0.011665
0.011362	0.011706	0.011732	0.011548	0.011229	0.011434	0.011267
0.011280	0.011761	0.011271	0.011462	0.011304	0.011366	0.011220
0.011468	0.011126	0.011283	0.011126	0.011222	0.011328	0.011385
0.011399	0.011257	0.011342	0.011342	0.011364	0.011497	0.011564
0.011564	0.011430	0.011534	0.011535	0.011670	0.011696	0.011362
0.011597	0.011463	0.011329	0.011430	0.011228	0.011463	0.011262
0.011329	0.011329	0.011295	0.011262	0.011295	0.011262	0.011362
0.011340	0.011340	0.011334	0.011368	0.011505	0.011402	0.011470
0.011469	0.011396	0.011208	0.011204	0.011331	0.011433	0.011501
0.011296	0.011365	0.011599	0.011567	0.011497	0.011531	0.011463
0.011396	0.011463	0.011463	0.011362	0.011934	0.011530	0.011598
0.011631	0.011701	0.011333	0.011407	0.011295	0.011497	0.011463
0.011498	0.011530	0.011530	0.011530	0.011650	0.011665	0.011303
0.011196	0.011199	0.011299	0.011264	0.011368	0.011210	0.011536
0.011335	0.011455	0.011203	0.011360	0.011329	0.011402	0.011228
0.011262	0.011262	0.011262	0.011295	0.011497	0.011463	0.011396
0.011497	0.011564	0.011295	0.011295	0.011329	0.011329	0.011330
0.011398	0.011262	0.011329	0.011364	0.011497	0.011564	0.011564
0.011430	0.011534	0.011535	0.011670	0.011696	0.011362	0.011530
0.011463	0.011463	0.011297	0.011478	0.011408	0.011435	0.011305
0.011405	0.011653	0.011441	0.011366	0.011195	0.011396	0.011262
0.011362	0.011362	0.011262	0.011363	0.011463	0.011466	0.011362
0.011262	0.011195	0.011329	0.011262	0.011195	0.011298	0.011396
0.011362	0.011295	0.011329	0.011362	0.011329	0.011262	0.011463
0.011329	0.011362	0.011362	0.011295	0.011362	0.011598	0.011262
0.011295	0.011698	0.011295	0.011262	0.011295	0.011665	0.011275
0.011679	0.011273	0.011311	0.011503	0.011589	0.011491	0.011227
0.011218	0.011463	0.011564	0.011671	0.011531	0.011669	0.011800
0.011699	0.011737	0.011698	0.011880	0.011665	0.012472	0.011700
0.012439	0.012207	0.012307	0.012613	0.011665	0.011363	0.011665
0.012103	0.012038	0.011631	0.011463	0.011598	0.011598	0.011500
0.011463	0.011467	0.011530	0.011464	0.011867	0.011934	0.011935
0.011834	0.011934	0.012038	0.011804	0.012104	0.012110	0.012171
0.012172	0.012205	0.012152	0.012172	0.012173	0.011396	0.011699
0.011767	0.012270	0.011772	0.012001	0.012474	0.011536	0.011430
0.011396	0.011396	0.011867	0.011924	0.012010	0.011732	0.011397
S206	0.011631	0.011631	0.011967	0.011732	0.011732	0.011799
0.011638	0.011570	0.011732	0.011228	0.011295	0.011262	0.011228
0.011262	0.011430	0.011228	0.011262	0.011329	0.011295	0.011262
0.011228	0.011262	0.011362	0.011228	0.011262	0.011262	0.011604
0.011497	0.011262	0.011161	0.011396	0.011329	0.011295	0.011329
0.011295	0.011396	0.011295	0.011295	0.011329	0.011295	0.011463
0.011228	0.011295	0.011329	0.011262	0.011362	0.011396	0.011329
0.011295	0.011463	0.011363	0.011631	0.011295	0.011497	0.011295

0.011262	0.011363	0.011430	0.011497	0.011665	0.011362	0.011463
0.011329	0.011396	0.011295	0.011530	0.011766	0.011295	0.011362
0.011262	0.011362	0.011329	0.011396	0.011329	0.011329	0.011329
0.011228	0.011262	0.011228	0.011329	0.011295	0.011329	0.011396
0.011329	0.011295	0.011362	0.011262	0.011396	0.011228	0.011295
0.011463	0.011396	0.011631	0.011463	0.011295	0.011530	0.011295
0.011468	0.011695	0.011475	0.011177	0.011172	0.011318	0.011375
0.012048	0.011467	0.011184	0.011530	0.011530	0.011329	0.011430
0.011362	0.011329	0.011396	0.011295	0.011396	0.011329	0.011329
0.011295	0.011396	0.011598	0.011463	0.011362	0.011329	0.011262
0.011228	0.011228	0.011295	0.011262	0.011463	0.011430	0.011362
0.011463	0.011530	0.011262	0.011262	0.011295	0.011295	0.011297
0.011364	0.011295	0.011295	0.011168	0.011205	0.011201	0.011296
0.011297	0.011232	0.011245	0.011267	0.011196	0.011232	0.011263
0.011528	0.011302	0.011336	0.011228	0.011233	0.011363	0.011329
0.011397	0.011330	0.011329	0.011429	0.011362	0.011732	0.011531
0.011564	0.011362	0.011295	0.011396	0.011195	0.011429	0.011228
0.011295	0.011295	0.011262	0.011228	0.011262	0.011228	0.011329
0.011295	0.011396	0.011329	0.011228	0.011330	0.011430	0.011432
0.011430	0.011329	0.011228	0.011161	0.011295	0.011228	0.011161
0.011264	0.011430	0.011228	0.011430	0.011127	0.011295	0.011195
0.011362	0.011262	0.011295	0.000000	0.011094	0.011161	0.011295
0.011329	0.011262	0.011329	0.011262	0.011295	0.011329	0.011564
0.011665	0.011597	0.011665	0.011431	0.011665	0.011295	0.011262
0.011297	0.011586	0.011380	0.011198	0.011671	0.011163	0.011257
0.011672	0.011570	0.011463	0.011665	0.011368	0.011530	0.011497
0.011430	0.011597	0.011451	0.011266	0.011312	0.011329	0.011399
0.011497	0.011398	0.011534	0.011602	0.011530	0.011598	0.011704
0.011433	0.011604	0.011534	0.011466	0.011733	0.011195	0.011397
0.011362	0.011295	0.011195	0.011195	0.011262	0.011262	0.011329
0.011262	0.011329	0.011228	0.011329	0.011262	0.011262	0.011329
0.011262	0.011698	0.011598	0.011631	0.011161	0.011262	0.011295
0.011295	0.011195	0.011329	0.011362	0.011228	0.011228	0.011228
0.011765	0.011396	0.011396	0.011195	0.011295	0.011463	0.011228
0.011228	0.011295	0.011262	0.011430	0.011228	0.011362	0.011497
0.011430	0.011262	0.011262	0.011195	0.011195	0.011363	0.011295
0.011195	0.011295	0.011161	0.011195	0.011329	0.011463	0.011228
0.011195	0.011329	0.011262	0.011161	0.011497	0.011161	0.011329
0.011403	0.011228	0.011530	0.011363	0.011396	0.011262	0.011362
0.011228	0.011497	0.011161	0.011430	0.011228	0.011228	0.011161
0.011396	0.011598	0.011295	0.011362	0.011195	0.011598	0.011228
0.011228	0.011228	0.011262	0.011362	0.011262	0.011362	0.011430
0.011430	0.011329	0.011295	0.011396	0.011362	0.011228	0.011195
0.011228	0.011228	0.011295	0.011228	0.011195	0.011362	0.011228
0.011262	0.011262	0.011396	0.011430	0.011329	0.011262	0.011262
0.011329	0.011262	0.011362	0.011228	0.011362	0.011463	0.011766
0.011262	0.011665	0.011267	0.017786	0.011191	0.011427	0.011618
0.011579	0.011623	0.011270	0.011234	0.011719	0.011647	0.011364
0.011487	0.011487	0.011138	0.011270	0.011216	0.011258	0.011232
0.011483	0.011395	0.011431	0.011193	0.011396	0.011430	0.011432
0.011301	0.011396	0.011396	0.011362	0.011329	0.011329	0.011295
0.011329	0.011262	0.011228	0.011329	0.011295	0.011228	0.011262
0.011262	0.011329	0.011228	0.011329	0.011362	0.011396	0.011295
0.011329	0.011295	0.011631	0.011631	0.011363	0.011397	0.011430
0.011195	0.011195	0.011228	0.011329	0.011195	0.011430	0.011430
0.011463	0.011463	0.011396	0.011497	0.011665	0.011498	0.011329
0.011329	0.011396	0.011430	0.011262	0.011329	0.011295	0.011329
[truncated: 679,711 more chars]
